# Gene expression profile and genomic alterations in colonic tumours induced by 1,2-dimethylhydrazine (DMH) in rats

**DOI:** 10.1186/1471-2407-10-194

**Published:** 2010-05-11

**Authors:** Angelo Pietro Femia, Cristina Luceri, Simona Toti, Augusto Giannini, Piero Dolara, Giovanna Caderni

**Affiliations:** 1 Department of Pharmacology, University of Florence, 6 Viale Pieraccini, 50139 Florence, Italy; 2 Department of Pathology, General Hospital of Prato-Azienda Sanitaria Locale 4, Prato, Italy

## Abstract

**Background:**

Azoxymethane (AOM) or 1,2-dimethylhydrazine (DMH)-induced colon carcinogenesis in rats shares many phenotypical similarities with human sporadic colon cancer and is a reliable model for identifying chemopreventive agents. Genetic mutations relevant to human colon cancer have been described in this model, but comprehensive gene expression and genomic analysis have not been reported so far. Therefore, we applied genome-wide technologies to study variations in gene expression and genomic alterations in DMH-induced colon cancer in F344 rats.

**Methods:**

For gene expression analysis, 9 tumours (TUM) and their paired normal mucosa (NM) were hybridized on 4 × 44K Whole rat arrays (Agilent) and selected genes were validated by semi-quantitative RT-PCR. Functional analysis on microarray data was performed by GenMAPP/MappFinder analysis. Array-comparative genomic hybridization (a-CGH) was performed on 10 paired TUM-NM samples hybridized on Rat genome arrays 2 × 105K (Agilent) and the results were analyzed by CGH Analytics (Agilent).

**Results:**

Microarray gene expression analysis showed that *Defcr4*, *Igfbp5*, *Mmp7, Nos2, S100A8 *and *S100A9 *were among the most up-regulated genes in tumours (Fold Change (FC) compared with NM: 183, 48, 39, 38, 36 and 32, respectively), while *Slc26a3*, *Mptx*, *Retlna *and *Muc2 *were strongly down-regulated (FC: -500; -376, -167, -79, respectively). Functional analysis showed that pathways controlling cell cycle, protein synthesis, matrix metalloproteinases, TNFα/NFkB, and inflammatory responses were up-regulated in tumours, while Krebs cycle, the electron transport chain, and fatty acid beta oxidation were down-regulated. a-CGH analysis showed that four TUM out of ten had one or two chromosomal aberrations. Importantly, one sample showed a deletion on chromosome 18 including *Apc*.

**Conclusion:**

The results showed complex gene expression alterations in adenocarcinomas encompassing many altered pathways. While a-CGH analysis showed a low degree of genomic imbalance, it is interesting to note that one of the alterations concerned *Apc*, a key gene in colorectal carcinogenesis. The fact that many of the molecular alterations described in this study are documented in human colon tumours confirms the relevance of DMH-induced cancers as a powerful tool for the study of colon carcinogenesis and chemoprevention.

## Background

Colorectal cancer is one of the most common neoplastic diseases in the Western World [1], developing through a multistage process which involves the accumulation of genetic and epigenetic alterations. Experimental models mimicking this disease in rodents, such as 1,2-dimethylhydrazine (DMH) or azoxymethane (AOM)-induced carcinogenesis, provide a tool for the understanding of the molecular alterations arising in human colon cancer. Inbred strains such as F344 rats, which are frequently used in these experiments, are relatively uniform and the tumors developing after induction show phenotypic and genotypic features similar to those observed in human sporadic colon cancers, notably activation of Wnt signaling pathway and mutations in *Kras *and *Apc *genes [2]. Importantly, AOM/DMH-induced tumours develop almost exclusively in the colon, at variance with other experimental models in which carcinogenesis develops mainly in the small intestine, a site rarely involved in human cancer. Since AOM/DMH tumours are affected by the same dietary variations known to affect human colon carcinogenesis, this model is among the most used and reliable for identification of potentially chemopreventive agents [3].

The advent of genome-wide technologies, such as gene expression microarray and array-based comparative genomic hybridization (a-CGH), has provided the possibility to achieve a comprehensive view of the alterations involved in cancer. Although several papers have been published on human colon tumours [4] and some also in AOM-treated mice [5,6], no studies applying genome-wide technology to DMH-induced cancers in rats have been reported. Given these considerations, we thought it of interest to study the gene expression profile of DMH-induced colon tumours in rats with the 44K Agilent rat arrays, representing the whole rat genome. Moreover, we were also interested in studying genomic alterations in this experimental model. In fact, although genomic instability in AOM-induced colon cancer in rats was previously reported [7], the technique in that study (Random Amplified Polymorphic DNA (RAPD analysis)) did not give information on the affected loci. On the other hand, using low resolution comparative genomic hybridization (CGH) [8], it has also been reported that AOM-induced tumours in mice have a low level of genomic alterations, making it difficult to draw any conclusions. Given these considerations and the possibility of achieving a comprehensive view of genomic alterations involved in tumors with high-resolution genome-wide technologies, we analyzed DMH-tumours with a-CGH using high resolution arrays (2 × 105K Agilent rat genome array).

## Methods

### Tumour induction and harvesting

Briefly, 4- to 5-week old male F344 rats (Nossan, Correzzana, Milan, Italy) were fed a high-fat diet based on the AIN-76 diet as previously described [9]. Rats were housed according to the European Union Regulations on the Care and Use of Laboratory Animals [10]; the experimental protocol was approved by the Commission for Animal Experimentation of the Italian Ministry of Health. At 6-7 weeks of age rats (n = 14) were treated twice, one week apart, with subcutaneous injections of 1,2-dimethylhydrazine (150 mg/kg × 2). Thirty-two weeks after the first DMH injection, rats were sacrificed by CO_2 _asphyxiation and colonic tumors harvested as described [11]. At sacrifice, all the rats had colon tumours. One-half of each tumour was cut and stored in RNAlater (Qiagen) while the second half was fixed in buffered formalin for histopathological and immunohistochemical analysis as described [11]. Apparent normal mucosa from each rat was also scraped from the colon and stored in RNAlater (Qiagen) before DNA or RNA extraction.

### Histological analysis

Histological sections of each tumour were stained with hematoxylin and eosin to confirm the presence and type of tumours by histopathological examination, on the basis of the histotype, grading and pattern of growth as described [11].

### Microarray hybridization

#### RNA isolation, labeling, hybridization and image analysis

Total RNA was extracted from 9 paired samples of normal mucosa (NM) and adenocarcinomas (TUM) using the RNeasy Midi kit (Qiagen, Milan, Italy). For each hybridization, 18 μg of total RNA from each sample (NM or TUM) was used. Each TUM (labeled with Cy5) (CyDye Mono-Reactive Dye Pack, Amersham, Cologno Monzese, Milan, Italy) was individually hybridized on Agilent Whole Rat Genome 4 × 44K microarrays (Agilent Technologies, Palo Alto, CA, USA) using the RNA (labeled with Cy3) from the corresponding NM as reference. As for labeling, we used the indirect labeling method described previously [12]. The hybridization steps were carried out according to the Agilent protocol and images were scanned using a Genepix 4000B microarray scanner (Axon Instruments, Foster City, CA, USA). Image analysis and initial quality control were performed using Agilent Feature Extraction Software v9.5.

### Microarray data analysis

The text files were imported into R 2.5.1 using the Bioconductor "limma" package 2.8.1 for statistical analysis as reported [13]. Values for control spots and spots that did not meet the quality criteria were flagged. Quality criteria included a minimal spot size, a median/mean ratio of at least 0.9 for each spot, non-saturated intensity for both channels, a signal well above background and a minimal signal intensity for at least one channel. Due to the high number of features that did not meet the quality control criteria, one out of the nine hybridizations was excluded from subsequent analysis. The background-corrected intensities of all microarrays were normalized using the LOESS algorithm as reported [13]. The tumour samples were compared to the corresponding normal mucosa using a paired moderated t-test with the Benjamini-Hochberg correction of the FDR (false discovery rate) for the multiple tests [14].

### Functional analysis

Biological pathway analysis was performed using the GenMAPP/MAPPFinder software tandem [15]. For GenMAPP, the "Rn_Std_20070817" gene database and the "Rn_20080619" pathway set were used. MAPPFinder analyses for pathway enrichment were performed for up- and down-regulated genes, separately. MAPPFinder selection criteria were set at Benjamin-Hochberg *p *< 0.05. Only statistically significant pathways showing a permuted *p*-value < 0.05 and a positive (enrichment) z-score >2 were selected.

### Semi-quantitative RT-PCR

One microgram of total RNA for each sample (tumour and normal mucosa) was reverse transcribed using 100 units of SuperScript™ II Reverse Transcriptase (Life Technologies, Milan, Italy) and 1× random hexamers (Roche Diagnostics, Monza, Italy). Seven out of the 9 paired TUM-NM samples were processed for RT-PCR analysis. The following genes were tested: *Defcr4*, *S100A9*, *Igfbp5*, *Slc30a2 *and *Lrg5 *(up-regulated genes); *Mptx*, *Slc26a3*, *Retnla*, *Hpgd *and *Muc2 *(down-regulated genes). The list of the primers used is provided in the Additional file 1: List of the primers used for semi-quantitative RT-PCR. Each gene was co-amplified with β-actin gene (*Actb*) as internal control as described [16]. PCRs were carried out using cDNA aliquots in a 25 μl total volume containing 1× PCR buffer, 2 mM MgCl_2_, 0.5 mM dNTPs, and 1.25 units of Taq polymerase (Sigma-Aldrich, Italy). Primers for each gene were also added (concentration range: 0.16 - 0.4 μM), together with the primers for β-actin (concentration range: 0.015-0.04 μM). The PCR conditions were: 95°C for 7 min and then 35 cycles at 95°C for 30 sec, 60°C for 30 sec and 72°C for 55 sec and a final extension at 72°C for 5 min. The PCR products were separated on 1.6% agarose gel, visualized by ethidium bromide staining. Gel images were captured by a digital photocamera (UviDoc) and the intensity of the bands was analyzed with Quantity-One software (Bio-Rad, Segrate, Milan, Italy). A t-test for paired samples was used to compare normal mucosa and tumor expression as previously described [16].

### Array-comparative genomic hybridization

A different set of ten samples of paired TUM-NM harvested from the same 14 rats was used to explore the presence of genomic aberrations in DMH-induced adenocarcinomas. To this purpose, genomic DNA was extracted by the DNeasy Tissue kit (Qiagen) and each tumour was hybridized on Agilent Rat Genome CGH Microarray 2 × 105K, following the Agilent protocol, using the corresponding normal mucosa as reference. The Rat CGH Agilent arrays contain 97,973 coding and noncoding sequences with an average probe spatial resolution of 19.1 Kb allowing high resolution analysis of the rat genome. Slides were scanned using the Genepix 4000B microarray scanner (Axon Instruments, Foster City, CA, USA). Image analysis and initial quality control were performed using Agilent Feature Extraction Software v9.5. Feature Extraction files were then loaded into CGH Analytics 3.5.14 software (Agilent Technologies, Palo Alto, CA, USA) and analyzed for aberration calls by selecting as follows: algorithm ADM-2, centralization on, fuzzy zero on, threshold at 6.0, aberration filter set at 5 for the minimum number of probes in region and at 0.24 for the minimum absolute average log ratio for region. The Derivative Log Ratio Spread (DLRS) for the ten hybridizations was in the range 0.11-0.24. The Rat genome sequence used was the Rn4 version.

### Immunohistochemical analysis

Tumour sections from 6 adenocarcinomas were processed for immunohistochemistry as described [11], using as primary antibody a rabbit polyclonal antibody against NFkB/p65 (Thermo Fisher Scientific, Fremont, CA) diluted 1:100 in PBS containing 1% bovine serum albumin and incubated for 1 h at room temperature (RT). The slides were processed with Biotinylated Goat Anti-Polyvalent as the secondary antibody (LAB Vision Corporation, CA, USA), and then weakly counterstained with Harris's hematoxylin [11]. Negative controls in which the primary antibody was omitted were performed in each experiment.

## Results

**Figure 1:**
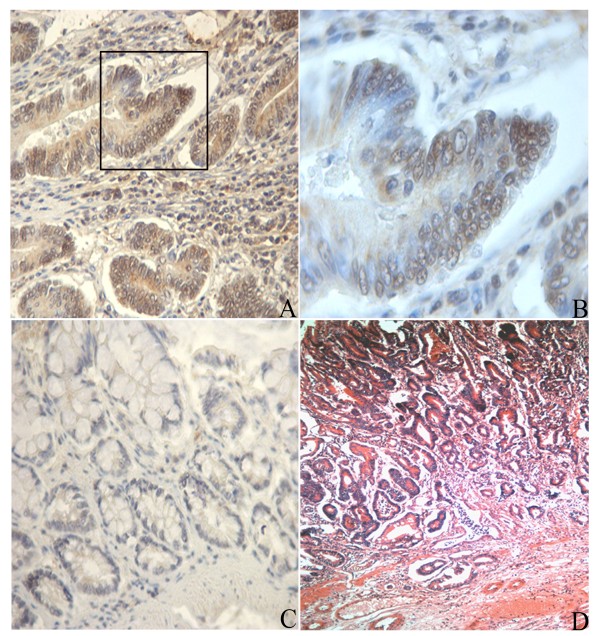
**Immunohistochemistry for NFkB/p65 and histology in a DMH-induced cancer**. Panel A: adenocarcinoma sample challenged with an antibody against NFkB/p65. Panel B: magnification of the inset in panel A; note that positive cells in the tumour show immunoreactivity in both the cytoplasm and the nucleus. Panel C: paired normal mucosa of the tumor challenged with the same antibody. Panel D: H&E stained section of the same tumour. Original magnification in panels A and C: ×400, panel B: ×1000, panel D: ×100.

The analyses described in this paper were carried out in colon cancers harvested from 14 rats induced with DMH as described in the Methods. The mean number of total tumours (both adenomas and cancers)/rat was 2.5 ± 0.34 (SD); adenomas/rat were 1.14 ± 1.09 while cancers/rat were 1.36 ± 0.49; all rats developed tumours. All the cancers analyzed (n = 19) were adenocarcinomas (Fig. 1D), except for one that was a mucinous adenocarcinoma (sample #14). Two samples were graded as moderately differentiated (samples #2 and #14), while the remaining 17 samples were well differentiated cancers.

### Gene expression profile by microarray analysis

Nine colon adenocarcinomas were hybridized on Agilent microarrays and each compared with its respective normal mucosa (NM). Eight out of nine of these samples passed the quality control step and were then analyzed as described in detail in the Methods section. Of the about 41,000 genes and transcripts present in the 4 × 44K Agilent Rat Whole Genome Expression slides, 27,329 probes passed the quality control step. The complete list of these 27,329 gene probes is provided as Additional file 2: Complete microarray gene expression data from the comparison between normal mucosa and adenocarcinomas. This list also comprises the 15,251 significantly up- or down-regulated gene probes as determined by statistical analysis (see the column with heading adjPVal).

Hierarchical clustering analysis of the eight array expression data (n = 8) shows a homogenous expression profile among these samples (Additional file 3: Hierarchical clustering analysis of gene expression data).

**Figure 2:**
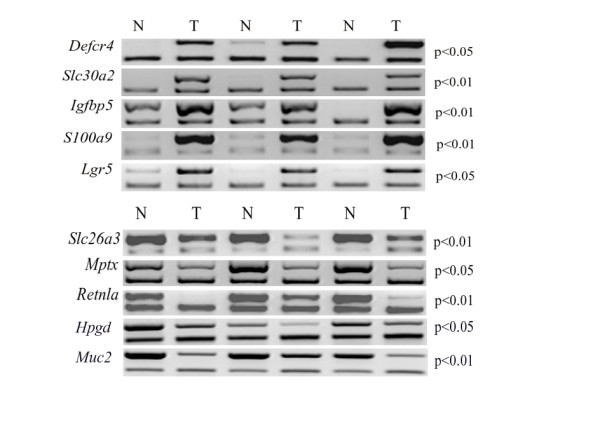
**Semi-quantitative RT-PCR analysis of ten selected genes**. For each up-regulated (first five rows) or down-regulated (last five rows) genes, three representative gel images of paired normal mucosa (N) and tumour (T) were provided. For each RT-PCR experiment, expression of β-actin was used as an internal control. P values refer to the t-test for paired samples (n = 7) to compare tumours with normal mucosa.

Setting a filter to a fold change (FC) equal to ± 10, we found that 266 (1%) and 566 (2%) genes were significantly up- or down-regulated in tumours vs NM, respectively (see Additional file 2). Moreover, using a FC cut-off equal to ± 2, 3724 (13.6%) and 3750 (13.7%) genes were significantly up- and down-regulated in tumors, respectively (see Additional file 2). Table 1 shows the first 25 up-regulated genes in tumours; among these, we found *Defcr4 *(defensin-related cryptdin 4), *Slc30a2 *(a solute carrier specific for zinc transport), *Lum *(lumican, a member of a small leucine-rich proteoglycan family), *Mmp12 *(matrix metallopeptidase 12), *Igfbp5 *(insulin growth factor binding protein 5), *Mmp7 *(matrix metallopeptidase 7), *Nos2 *(nitric oxide synthase 2, inducible), *S100A8 *(S100 calcium binding protein A8 (calgranulin A)) and *S100A9 *(S100 calcium binding protein A9 (calgranulin B)). Among the most down-regulated genes (Table 1), we found *Slc26a3 *(solute carrier family 26, member 3, also known as down-regulated in adenoma (*Dra*)), *Mptx *(mucosal pentraxin), *Retnla *(resistin like-α), *Muc2 *(mucin 2). For a selected group of genes, the results obtained with microarray analysis were confirmed by semi-quantitative RT-PCR. These were *Defcr4*, *S100A9*, *Igfbp5*, *Slc30a2 *and *Lgr5 *(leucine-rich repeat containing G protein coupled receptor 5) which were up-regulated and *Mptx*, *Slc26a3*, *Retnla, Muc2 *and *Hpgd *(hydroxyprostaglandin dehydrogenase 15) which were down-regulated in tumours (Fig. 2).

**Table 1:** List of the first 25 genes up- and down-regulated in tumours.

Gene name	Gene Description [Accession number]	FC
**Up-regulated**		
*Defcr4*	Defensin related cryptdin 4 [NM_001013053]	183.2
*Slc30a2*	Solute carrier family 30 (zinc transporter), member 2 [NM_012890]	71.7
*Lum*	Lumican [NM_031050]	70.0
*Mmp12*	Matrix metallopeptidase 12 [NM_053963]	65.1
*Iggc*	Immunoglobulin gamma2a constant region [DQ402472]	58.8
*Hoxd13_predicted*	Homeo box D13 (predicted) [XM_221511]	50.5
*Igfbp5*	Insulin-like growth factor binding protein 5 [BC087030]	48.4
*CB548350*	Tenascin C [CB548350]	47.0
*Msr2_predicted*	Macrophage scavenger receptor 2 (predicted) [XM_227485]	45.7
*Mx2*	Myxovirus (influenza virus) resistance 2 [NM_134350]	43.9
*Aldh1a3*	Aldehyde dehydrogenase family 1, subfamily A3 [NM_153300]	42.8
*Cxcl2*	Chemokine (C-X-C motif) ligand 2 [NM_053647]	39.9
*Mmp7*	Matrix metallopeptidase 7 [NM_012864]	39.4
*Nos2*	Nitric oxide synthase 2, inducible [NM_012611]	38.1
*S*100*a*8	S100 calcium binding protein A8 (calgranulin A) [NM_053822]	36.2
*Igfbp7*	Insulin-like growth factor binding protein 7 [XM_214014]	35.8
*Col12a1*	PREDICTED: procollagen, type XII, alpha 1 [XM_243912]	35.7
*Plod2*	Procollagen lysine, 2-oxoglutarate 5-dioxygenase 2 [NM_175869]	33.4
*S*100*a*9	S100 calcium binding protein A9 (calgranulin B) [NM_053587]	31.9
*Ctgf*	Connective tissue growth factor [NM_022266]	31.5
*Fn1*	Fibronectin 1 [NM_019143]	31.4
*T_predicted*	T, brachyury homolog (mouse) (predicted) [XM_217890]	30.0
*Sparc*	Secreted acidic cysteine rich glycoprotein [NM_012656]	29.6
*Cyp26b1*	Cytochrome P450, family 26, subfamily b, polypeptide 1 [NM_181087]	29.4
*Nkd1_predicted*	Naked cuticle 1 homolog (Drosophila) (predicted) [XM_001066780]	29.1
**Down-regulated**		
*Slc26a3*	Solute carrier family 26, member 3 [NM_053755]	-502.6
*Mptx*	Mucosal pentraxin [NM_001037642]	-376.5
*Retnla*	Resistin like alpha [NM_053333]	-166.9
*Zg16*	Zymogen granule protein 16 [NM_134409]	-105.3
*Clca3_predicted*	Chloride channel calcium activated 3 (predicted) [XM_217689]	-99.1
*Grap*	GRB2-related adaptor protein [NM_001025749]	-95.2
*Fabp1*	Fatty acid binding protein 1, liver [NM_012556]	-94.7
*Ceacam20_predicted*	CEA-related cell adhesion molecule 20 (predicted) [XM_218430]	-92.8
*Dpep1*	Dipeptidase 1 (renal) [NM_053591]	-90.9
*Pbp2*	Phosphatidylethanlomine binding protein 2 [XM_575702]	-90.4
*B4 galt2_predicted*	UDP-Gal:betaGlcNAc beta 1,4-galactosyltransferase, polypeptide 2 (predicted) [XM_242992]	-90.0
*Apoa1*	Apolipoprotein A-I [NM_012738]	-80.6
*Olr1611_predicted*	Olfactory receptor 1611 (predicted) [XM_223954]	-80.5
*Dmbt1*	PREDICTED: deleted in malignant brain tumours 1 (Dmbt1), [XM_577842]	-80.0
*Muc2*	Mucin 2 [U07615]	-78.7
*Trpm5_predicted*	Transient receptor potential cation channel, subfamily M, member 5 (predicted) [XM_344979]	-73.1
*Chia*	Rattus norvegicus chitinase, acidic [NM_207586]	-72.7
*Kb15*	Type II keratin Kb15 [XM_345877]	-72.6
*Gpr156*	G protein-coupled receptor 156 [NM_153295]	-71.8
*Pdha2*	Pyruvate dehydrogenase E1 alpha 2 [NM_053994]	-71.3
*Hspa1l_mapped*	Heat shock 70 kD protein 1-like (mapped) [NM_212546]	-71.0
*Pcdhb13*	PREDICTED: Protocadherin beta 13 [XM_001055698]	-71.0
*Pcsk4*	Proprotein convertase subtilisin/kexin type 4 [NM_133559]	-70.4
*Masp1*	Mannan-binding lectin serine peptidase 1 [NM_022257]	-70.4
*C1ql1_predicted*	Complement component 1, q subcomponent-like 1 (predicted) [XM_343971]	-70.3

To understand whether specific biological pathways or functional groups of genes were differentially affected in tumours, we analyzed our microarray data set using the Gen MaPP/MappFinder software tool. The results of this analysis showed that 17 pathways were significantly up-regulated in tumours, while 10 were down-regulated (Table 2). The list of genes present in these significantly up- or down-regulated pathways is shown in Additional files 4 and 5 (Additional file 4: List of the genes belonging to the significantly up-regulated pathways; Additional file 5: List of the genes belonging to the significantly down-regulated pathways). Among the up-regulated pathways we found those related to cell cycle, to RNA and protein metabolism, to extracellular matrix remodeling.

**Table 2:** Pathways or functional groups of genes differentially expressed in tumours by GenMapp/MappFinder analysis*.

MAPP Name	Number Changed	Number Measured	Number On MAPP	Z Score
***Up-regulated pathways or functional group of genes ***				
Rn_DNA_replication_Reactome	17	22	40	3.289
Rn_Matrix_Metalloproteinases	12	13	28	3.621
Rn_TNF-alpha-NF-kB_NetPath_9	69	117	159	3.648
Rn_G1_to_S_cell_cycle_Reactome	29	40	69	3.838
Rn_mRNA_processing_Reactome	44	66	125	3.988
Rn_Translation_Factors	24	29	40	4.383
Rn_Ribosomal_Proteins	65	74	81	7.979
Rn_Cell_cycle_KEGG	32	49	80	3.227
Rn_RNA_transcription_Reactome	15	19	41	3.202
Rn_TGF_Beta_Signaling_Pathway	27	39	45	3.372
Rn_Inflammatory_Response_Pathway	16	22	40	2.855
Rn_Proteasome_Degradation	27	42	53	2.848
Rn_Alpha6-Beta4-Integrin_NetPath_1	29	47	64	2.654
Rn_B_Cell_Receptor_NetPath_12	61	112	146	2.573
Rn_Signaling_of_Hepatocyte_Growth_Factor_Receptor_Biocarta	18	27	33	2.527
Rn_Nucleotide_Metabolism	10	14	17	2.175
Rn_Complement_Activation_Classical	7	9	14	2.127
***Down-regulated pathways or functional group of genes***				
Rn_Nuclear_Receptors	16	26	37	3.564
Rn_Unsaturated_Fatty_Acid_Beta_Oxidation_BiGCaT	6	6	6	3.766
Rn_Electron_Transport_Chain	26	44	58	4.298
Rn_Nuclear_receptors_in_lipid_metabolism_and_toxicity	13	19	33	3.700
Rn_Fatty_Acid_Beta_Oxidation_2_BiGCaT	5	6	6	2.873
Rn_Krebs-TCA_Cycle	12	23	26	2.362
Rn_Irinotecan_pathway_PharmGKB	5	6	12	2.873
Rn_Peptide_GPCRs	15	31	62	2.283
Rn_Calcium_regulation_in_cardiac_cells	41	102	150	2.360
Rn_Glucocorticoid_Mineralocorticoid_Metabolism	5	7	9	2.414

* Significantly enriched pathways (z-score >2) are listed. The different columns show the number of genes differentially expressed compared with normal mucosa, the number of genes analyzed and the number of genes listed in each map (http://www.genmapp.org; University of California at San Francisco, San Francisco, CA, USA)

Moreover, since the results of this analysis also pointed out an up-regulation of the inflammatory processes (inflammatory response pathway, TNFα-NFkB, complement activation), we further investigated the involvement of the NFkB pathway, determining in six adenocarcinomas the expression of RelA (p65) with immunohistochemistry. The results (Fig.1, panels A-C) showed that p65, a key member of the NFkB pathway, was highly expressed in the tumours compared to normal mucosa.

Among the pathways and grouping of functional genes down-regulated in tumors (Table 2) we found those related to cellular metabolism (fatty acid beta-oxidation, Krebs-tricarboxylic acid cycle and electron transport chain) and nuclear receptors.

### Genomic alterations by a-CGH

An additional set of ten colon adenocarcinomas harvested from the same rats were analyzed by a-CGH, comparing each tumour sample with its respective normal mucosa (NM). All the hybridized arrays passed the quality control step and were then analyzed by the CGH Analytics 3.5 software (Agilent Technologies) for detection of chromosomal aberrations. In the 10 tumors analyzed, a total of 6 alterations were observed in four tumours (Table 3). Four of these 6 aberrations were amplifications (length of the amplified regions ranged from 67 to 2135 kb) and two were deletions (672 kb and 1362 kb, respectively). Two tumours (samples #3 and #5) showed two aberrations each (two amplifications in tumour #3 and one amplification plus a deletion in tumour #5), while the remaining two samples had only one aberration each.

**Table 3:** Summary of the chromosomal aberrations found in adenocarcinomas from DMH-induced rats.

Tumour	Aberration	Chromosome	Aberration length (kb)	Genes in the aberrant region
# 3	Amplification	1q22	109.8	*Pcsk*
	Amplification	7q11	2135.3	*Sirt6, Tle2, Aes, Gna11, Gna15, Ncln, NF1-C2, Tbxa2r, Pip5k1c, Apba3, Matk, Atcay_predicted, Dapk3, Zbtb7a, Map2k2, Creb3l3, Thop1, Sgta, Slc39a3, Gng7, Gadd45b, Tmprss9_predicted, RGD1308556, Sf3a2, Mknk2, Csnk1 g2, Scamp4, RGD1359682, Tcfe2a, Reep6, Pcsk4, Dazap1, Cirbp, Abca7, Grin3b, Prtn3_predicted, Prg-2, Ptbp1, Palm, Fstl3, Rnf126, Hcn2, Bsg*,
# 5	Deletion	7q13	671.9	*Nudt4, Ac1-114*
	Amplification	2q23	66.9	*LOC360689*
# 6	Amplification	15q22	189.4	Unknown
# 9	Deletion	18 p11-12	1362.1	*Apc, Brd8, Gfra3, Egr1, Etf1, Catna1, Sil1*

**Figure 3:**
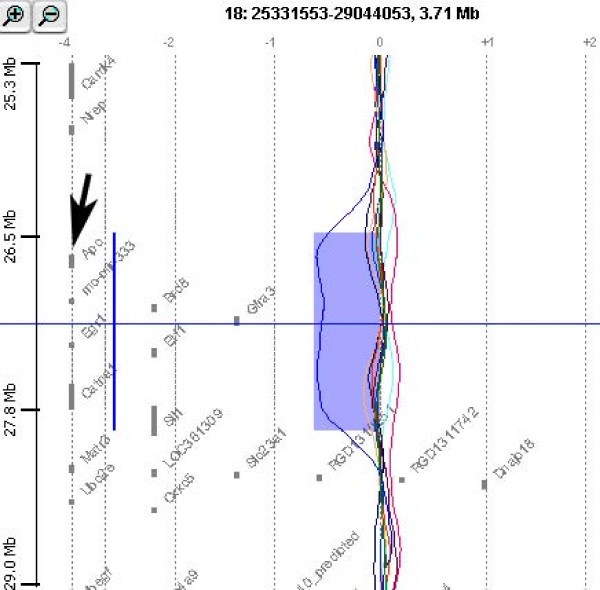
**CGH analytics chromosome view of a region of chromosome 18 of all tumor samples analyzed**. Colored curves represent Log_2 _ratio values for all nucleotide probes plotted as a function of their chromosomal position for each tumor sample. Thick straight vertical blue line and blue shaded area point out a deletion present in sample #9. The black arrow indicates the *Apc *gene position.

The genomic aberrations were located in a region of chromosome 1, amplified in one tumor containing *Pcsk6*, coding for a protease belonging to the pro-protein convertase family. The same tumour carrying this amplification also carried a long amplification on chromosome 7 with more than forty genes mapping in this region (Table 3). Contrary to the long amplification of chromosome 7, the deletion on the same chromosome carried by a different tumour was short, with only two genes (*Nudt4 *and *Ac1-114) *mapping in this region. Notably, in tumour sample #9, we observed an interstitial deletion of 1.36 Mb on chromosome 18 encompassing the *Apc *(*Adenomatous polyposis coli*) gene as well as other genes such as *Catna1 *(coding for β-catenin) (Table 3 and Fig. 3).

## Discussion

This is the first report describing variations in gene expression and genomic alterations in DMH-induced rat colon adenocarcinomas using microarray technologies. We found that many transcripts were differentially expressed in colon tumors, 7400 of them being differentially expressed in tumours with a FC ≥ 2 [3724 (13.6%) and 3750 (13.7%) up-regulated or down-regulated in tumours].

The most up-regulated gene was *Defcr4*, also known as enteric defensin α5, belonging to the family of the α-defensin antimicrobial peptides secreted by Paneth cells [17]. Other markers of Paneth cells such as defensin alpha 6 (*Defa6; *FC = 18.7), matrix metallopeptidase 7 (*Mmp7*), lysozyme (*Lyz; *FC = 13.1), secretory phospholipaseA2 (*Pla2 g2a; *FC = 20), were also up-regulated in our samples, suggesting that these cells are a cellular component of colon tumours, in agreement with previous reports [17-19]. Although it has been suggested that the presence of Paneth cells in colon cancer is a fortuitous consequence of the Wnt pathway activation [17,20], it is interesting to note that *Mmp7 *up-regulation, as observed in our samples, has been correlated with metastasis and apoptosis resistance in cancer [21]. Besides *Mmp7*, other metalloproteinases (MMPs) involved in the degradation of extracellular matrix in the late phases of carcinogenesis were up-regulated in our samples (*Mmp3*, *9*, *10*, *12 *and *14; *FC = 4.3, 18.1, 13.1, 65.1, 3.2, respectively) (see also Additional file 2) [18], a result reinforced by the significant up-regulation of the MMP pathway pointed out by GenMapp analysis. Linked to MMPs in the process of invasion, we also found up-regulation of genes involved in the urokinase-type plasminogen activator system such as urokinase-plasminogen activator u-PA (*Plau *in rat; FC = 9.6), u-PA receptor (*Plaur; *FC = 2) and plasminogen (*Plg; *FC = 13.6) (see Additional file 2) [22]. Interestingly, it has also been suggested that u-PA might catalyze the activation of the mesenchymal-derived cytokine hepatocyte growth factor/Scatter Factor (HGF/SF), thus facilitating invasiveness [22,23], an observation in line with the activation of HGF signaling in our tumours, as pointed out by the GenMapp analysis.

*Slc30a2*, the second most up-regulated gene in our set of colon adenocarcinomas, codes for the protein Znt2 which has been reported to promote zinc efflux into intracellular vesicles when Zn concentration rises in the cell [24]. Accordingly, a previous study showed that Zn concentration was higher in rat colon tumours than in normal mucosa [25].

The *Lum *gene, coding for the protein Lumican, is the third most expressed gene in our set of tumours (Table 1). Lumican plays an important role in collagen fibrillogenesis and its over-expression has been reported in human colorectal cancer [26]. Another dramatically up-regulated gene in our samples is *Igfbp5 *(Insulin-like growth factor-binding protein 5) (Table 1), a component of the IGF axis [27], implicated in colon carcinogenesis with contradictory results [28,29]. Indeed, while *Igfbp5 *is up regulated in adenomas from Apc^Min/+ ^mice, it moves in the opposite direction in human colorectal cancers and cancer cell lines [28]. It has also been shown that IGFBP5 can be cleaved by MMP7 forming IGF-II which in turn can act as a growth factor for colonic myofibroblasts [29], suggesting an important role for IGFBP5 and MMP-7 in the tumoural epithelial-mesenchymal transition. Recent evidence also indicates that Igfbp5 may act as a tumour suppressor by inhibiting angiogenesis [30]. These results suggest the need to further investigate the role of *Igfbp5 *in colon cancers.

Among the most up-regulated genes we also found *Nos2 *(Table 1) coding for the pro-inflammatory enzyme inducible nitric oxide synthase (i-NOS). Many reports document high i-NOS activity in colorectal cancers [31,32]; recently, we also reported that DMH-induced colorectal tumours have high i-NOS expression [33]. Interestingly, *S100A8 *and *S100A9*, whose products form the heterocomplex calprotectin, are strongly up-regulated in our study (Table 1), in agreement with reports on inflammation-associated cancer and in human colorectal carcinomas [34,35]. Accordingly, we also observed an up-regulation of genes involved in prostaglandin synthesis, such as cyclooxygenase 1 and 2 (*Ptgs1 *and *Ptgs2; *FC = 5.4 for both genes, see Additional file 2) and prostaglandin E synthase (*Ptges; FC = 2.2) *associated with strong down-regulation of the gene for the enzyme degrading prostaglandins (15-hydroxyprostaglandin dehydrogenase, *Hpgd*; FC = -29), as demonstrated in both the array and RT-PCR experiments and in agreement with previous reports showing a down-regulation of this gene in colon carcinogenesis [36]. Moreover, GenMapp analysis pointed out that the TNFα-NF-kB pathway was up-regulated in our samples, as also demonstrated by the over-expression of p65 (RelA) observed with both immunohistochemistry (Fig.1, panels A-C) and array technique (FC of RelA: 2.2, see additional file 2). All these data confirm that DMH-induced tumours show activation of inflammatory process pathways, as documented in rats and human colon cancer [32,33,37].

Aberrant activation of the Wnt signaling has been demonstrated in both experimental and human carcinogenesis [38]. Accordingly, many Wnt-target genes were up-regulated in our samples (*Ccnd2 *(FC = 5.9), *Lef1 *(FC = 21.5), *Mmp7*, *Axin2 *(FC = 3.9), *CD44 *(FC = 3.4), *Bmp4 *(FC = 5.1), *Dkk3 *(FC = 14.3), *Sox9 *(FC = 2.9), *Fn1 *(FC = 31.4), *Mmp9 *(FC = 18.1), *Stra6 *(FC = 21.3), *Ptgs2 *(FC = 5.4), *Postn *(FC = 11.8), *Sfrp2 *(FC = 6.3)) [39] (see also Additional file 2). Notably, *Lgr5*, a marker of stem cells in colon epithelium [18] was also up-regulated in the arrays (FC = 10) as well as in RT-PCR experiments, demonstrating that cells presenting this stem marker are over-represented not only in *Min *mice intestinal tumours and human colon cancers [40,41], but also in DMH-induced colonic tumors.

As expected, we also found an up-regulation of pathways related to nucleotide metabolism, DNA replication, cytoplasmic ribosomal protein, translation factors, proteasome degradation, mRNA processing and cell cycle (Table 2).

On the contrary, the up-regulation of the TGF-β pathway in our samples was somewhat unexpected since previous findings in colon cancers showed inactivation of TGF-β signaling [42-44]. In our samples the genes coding for the ligand *Tgfb1 *(FC = 5.7), the receptors *Tgfrb1 *(FC = 2.6) and *Tgfrb2 *(FC = 5.5) as well as *Smad2 *(FC = 2.9) and *Smad4 *(FC = 2.4) were up-regulated, although not *Smad3 *(FC = -2.3) which was down-regulated. It is interesting to note that although TGF-β is a potent inhibitor of normal colonic epithelial cells, it can also promote the survival, invasion and metastasis of colorectal cancer cells, thereby acting as an oncogene, a paradoxical role which could explain our results [45,46].

*Slc26a3*, coding for a Cl(-)/HCO(3)(-) exchanger, is the most down-regulated gene in this report. Although genetic studies linked mutations in the homologous human gene (*SLC26A3*) to congenital chloride-losing diarrhea, it is interesting to note that *Slc26a3 *is also known with the alias "down-regulated in adenoma" (*Dra*), since it was first identified as a gene strongly down-regulated in colon adenomas and adenocarcinomas [47,48]. Its deficiency has been associated with an increase in cellular proliferation [48], a feature that could partly contribute to tumour development also in our samples. *Mptx*, the gene coding for mucosal rat pentraxin, is also strongly down-regulated in the present study. *Mptx *was first described as the most down-regulated gene in the colonic mucosa of rats fed dietary heme and, since it has been suggested to play a role in the clearance of dying cells, its down-regulation could lead to a reduced apoptosis ability, another characteristic of tumour cells [49]. *Retlna *(resistin-like molecule α), which is also down-regulated in our samples (Table 1 and Fig. 2), belongs to a family of resistin-like molecules implicated in metabolism, energy balance and intestinal inflammation [50].

We also observed a strong down-regulation of the *Muc2 *gene, coding for Muc2, the most abundant mucin secreted by normal colonocytes, in agreement with experimental and clinical reports documenting defective mucin production in colorectal cancer [51,52].

Significantly down-regulated pathways were processes such as Krebs-TCA-cycle, electron transport chain, and fatty acid beta-oxidation (Table 2), in agreement with many previous observations showing a metabolic reprogramming of the cancer cell favoring glycolysis even in the presence of oxygen [53].

It has also to be noted that our rats were fed a high-fat diet containing 23% corn oil as a source of fat, a diet currently used in our laboratory since it closely mimics the high-fat diet typical of western countries with populations having a high incidence of colon cancer. Thus one could wonder whether the same results would be observed also in animals fed a low-fat diet. However, since in our experiments we compared tumours with their paired normal mucosa, it is not possible to infer the influence of diet on gene expression profile in tumours. In fact, to carefully address this issue, one would have to perform an additional experiment comparing the tumour profile from low-fat diet rats with that from high-fat diet rats.

In the present study we also evaluated genomic alterations with a-CGH analysis in a different set of tumours harvested from the same rats. This is, to our knowledge, the first attempt to highlight genomic aberrations in DMH-induced colonic tumours in the rat by means of high resolution a-CGH.

Our results show that genomic aberrations are relatively infrequent, with 40% of tumours showing only one or two aberrations (i.e. amplification or deletion), while the remaining 60% show a stable genome. The detected alterations spanned from 66 to 2135 kb and in no case did we observe gains or losses of whole chromosomes. The relatively low level of genomic alterations in this study is in agreement with previous reports on tumours from *Min *mice and AOM-treated A/J mice [8,54]. In a previous study from our laboratory, we reported frequent genomic alterations in AOM-induced colon tumours using RAPD analysis [7]; this discrepancy might be due to the fact that high-resolution a-CGH, at variance with RAPD, does not detect small alterations (such as single base mutations), which could be frequent in this model.

Notably, one of the aberrations found in the present study was a deletion on chromosome 18, spanning a 1362 kb region containing the *Apc *gene, a key gene in colon cancer [38]. Previously, we demonstrated that DMH-induced colon cancers, beside mutations in *Ctnnb1 *(coding for β-catenin) and *Kras*, carry single-nucleotide mutations in *Apc *with a frequency of about 30% [2]. Therefore, the occurrence of an interstitial deletion encompassing this gene could be a further mechanism of *Apc *inactivation in colon carcinogenesis. Accordingly, allelic loss of one *APC *gene copy without detectable *APC *mutation has been reported in human colorectal tumours [55]. It is also interesting to note that among the genes mapping in the region deleted in this sample, we found *Catna1 *(coding for β-catenin). Unlike β-catenin, the contribution of α-catenin to colorectal carcinogenesis is controversial [56]. In fact some reports suggest that α-catenin is essential for tumour formation [56], while others suggest that α-catenin plays a tumour-suppressive role in the late stages of colorectal carcinogenesis [57,58]. Notably all the genes deleted on chromosome 18 (including *Apc *and *Catna1)*, are synthenic with their human homologues genes (i.e. they are also close to each other in the human genome, all of them mapping on the short arm of chromosome 5).

## Conclusions

In conclusion, we have used genome-wide technologies to study the gene expression profile and genomic alterations in DMH-induced colon cancers, for which no information was previously available. Our results show complex gene expression alterations in adenocarcinomas encompassing many altered pathways such as those linked to inflammation, matrix metalloproteases, cell proliferation and metabolism. While a-CGH analysis showed a low degree of genomic imbalance in these tumours, it is interesting to note that one of the alterations found regards *Apc*, a key gene in colorectal carcinogenesis. The fact that many of the alterations described in this study are documented in human colon tumours confirms the relevance of DMH-induced cancers as a powerful tool for the study of colon carcinogenesis and chemoprevention. Moreover, the results of this study encompassing the characterization of genomic alterations in colon adenocarcinomas may serve as the basis for investigations of the derangements occurring in the early phases of colon carcinogenesis, which are easily analyzed using this model.

## Competing interests

The authors declare that they have no competing interests.

## Authors' contributions

GC and APF conceived and designed the work. APF carried out the carcinogenesis experiment, DNA and RNA extraction, RT-PCR validation experiments, a-CGH analysis by CGH Analytics software and drafted the manuscript. CL did the microarray (gene expression) and a-CGH hybridizations, functional analysis by GenMapp/Mapp Finder software and a-CGH analysis by CGH Analytics software. ST carried out the statistical analysis of microarray (gene expression) data. PD critically revised the manuscript and gave final approval of the version to be published. GC carried out the carcinogenesis experiment, drafted the manuscript and gave the main contribution to the interpretation of data. All authors read and approved the final manuscript.

## Pre-publication history

The pre-publication history for this paper can be accessed here:

http://www.biomedcentral.com/1471-2407/10/194/prepub

## Supplementary Material

Additional file 1 **List of the primers used in the RT-PCR experiments**. The file provides the sequences of the primers used for reverse-transcriptase-PCR experiments and the GenBank accession number for each gene. Click here for file

Additional file 2 **Complete microarray gene expression data from the comparison between normal colon mucosa and adenocarcinomas**. The file provides the complete list of the 27,329 probes passing the quality control step as described in the methods. For each probe, the Agilent probe name, gene name, gene identifier, EntrezGene ID http://www.ncbi.nlm.nih.gov/gene, Log_10 _fold change (FC), FC and adjusted P value is reported. The fold changes are means of at least 4 out of 8 colon cancers. AdjPval < 0.05 identifies genes differentially expressed in cancers compared to the corresponding NM. Click here for file

Additional file 3 **Hierarchical cluster analysis of gene expression data**. Hierarchical cluster analysis of gene expression data from the 8 DMH-induced tumours (samples # 11-18) compared to the corresponding normal colon mucosa. The analysis was performed on all genes which passed the quality control step present in 100% of the experiments. Click here for file

Additional file 4 **List of the genes belonging to the significantly up-regulated pathways**. List of the genes (EntrezGene ID: http://www.ncbi.nlm.nih.gov/gene) belonging to the significantly up-regulated pathways shown in Table 2. Click here for file

Additional file 5 **List of the genes belonging to the significantly down-regulated pathways**. List of the genes (EntrezGene ID: http://www.ncbi.nlm.nih.gov/gene) belonging to the significantly down-regulated pathways shown in Table 2. Click here for file
