# Supplementary material for: Gene expression profile and genomic alterations in colonic tumours induced by 1,2-dimethylhydrazine (DMH) in rats
Source: BMC Cancer. 2010 May 11;10:194. doi: 10.1186/1471-2407-10-194 (PMC2877689; doi:10.1186/1471-2407-10-194)
Supplement: Additional file 1 — List of the primers used in the RT-PCR experiments. The file provides the sequences of the primers used for reverse-transcriptase-PCR experiments and the GenBank accession number for each gene. [file 1471-2407-10-194-S1.DOC]

Additional file 1: List of the primers used for semi-quantitative RT-PCR.

| **Gene** | **Primer Fw (5’-3’)** | **Primer Rv (5’-3’)** | **GenBank Accession Number** |
| --- | --- | --- | --- |
| *Defcr4* | CACACTGGACTCCTGCTAACC | GCAGAGTAGTCATCAACATCAGC | NM_001013053 |
| *S100A9* | GCACGAGCTCCTTAGCTTTG | GACTTGGTTGGGCAGATGTT | NM_053587 |
| *Igfbp5* | GAGACTGAGGAGGCCATCTG | GGAGGTCAGAGCCTGTGAAG | BC087030 |
| *Slc30a2* | TTGGAAGTATGGCCAAGACC | CCTGGACTGTGATTGGGTTT | NM_001083122 |
| *Lgr5* | CTGGAGAACCTTTGGGACTG | TGGGACAAATGCAACTGAAG | NM_001106784 |
| *Mptx* | CAACACAAAAACTCAGGACAATG | TTCGTAAGTAAGAGCTCGGAAGTT | NM_001037642 |
| *Retnla* | CCTTCTCATCTGCGTCTTCC | TTCAAGAAGCGGGGTTAATG | NM_053333.1 |
| *Slc26a3* | ATATTGTGGGAACCGATGATG | CATACTAGAACAATCTCGTTCCAAAG | NM_053755 |
| *Muc2* | CCATCTCCACCACCATTACC | CAGATGAAGTCAGTGGGGAAG | U07615.1 |
| *Hpgd* | AAGGGGGAAGCTCCTTTACA | GAGAGGCACTTTTGAAATTGGT | NM_024390.2 |
| *Actb* | ACCACAGCTGAGAGGGAAAT | AGAGGTCTTTACGGATGTCAAC | NM_031144 |
