# Supplementary material for: Gene expression profile and genomic alterations in colonic tumours induced by 1,2-dimethylhydrazine (DMH) in rats
Source: BMC Cancer. 2010 May 11;10:194. doi: 10.1186/1471-2407-10-194 (PMC2877689; doi:10.1186/1471-2407-10-194)
Supplement: Additional file 2 — Complete microarray gene expression data from the comparison between normal colon mucosa and adenocarcinomas. The file provides the complete list of the 27,329 probes passing the quality control step as described in the methods. For each probe, the Agilent probe name, gene name, gene identifier, EntrezGene ID http://www.ncbi.nlm.nih.gov/gene, Log10 fold change (FC), FC and adjusted P value is reported. The fold changes are means of at least 4 out of 8 colon cancers. AdjPval < 0.05 identifies genes differentially expressed in cancers compared to the corresponding NM. [file 1471-2407-10-194-S2.PDF]

Additional file 2

Title: Complete microarray gene expression data from the comparison between normal colon mucosa and adenocarcinomas.

Probe Name	Gene name	Accession Number	Gene ID	Log $_{10}$ FC	FC	adjPVal
A_42_P509923	Defcr4	NM_001013053	290856	2.26	183.17	0.000046
A_42_P663722	Slc30a2	NM_012890	25362	1.86	71.69	1.86E-05
A_44_P458627	Lum	NM_031050	81682	1.85	69.98	5.42E-06
A_44_P555271	Mmp12	NM_053963	117033	1.81	65.13	2.68E-06
A_44_P194278	ENSRNOT00000006998	ENSRNOT00000006998		1.77	58.78	6.55E-05
A_44_P297590	Hoxd13_predicted	XM_221511		1.70	50.50	1.46E-05
A_44_P264240	Igfbp5	BC087030	25285	1.69	48.42	2.15E-06
A_43_P19661	CB548350	CB548350		1.67	47.04	0.000088
A_43_P19999	Msr2_predicted	XM_227485		1.66	45.66	9.54E-06
A_44_P214846	Mx2	NM_134350	286918	1.64	43.90	6.04E-05
A_44_P273783	Aldh1a3	NM_153300	266603	1.63	42.78	5.13E-06
A_44_P515197	Cxcl2	NM_053647	114105	1.60	39.87	6.97E-05
A_43_P11590	Mmp7	NM_012864	25335	1.60	39.36	2.75E-06
A_44_P545686	Nos2	NM_012611	24599	1.58	38.13	2.39E-06
A_44_P1055780	S100a8	NM_053822	116547	1.56	36.15	8.92E-05
A_44_P375102	XM_214014	XM_214014		1.55	35.83	2.17E-06
A_44_P473152	Col12a1	XM_243912	25683	1.55	35.69	2.45E-06
A_44_P536978	XM_345742	XM_345742		1.53	33.61	0.005884
A_44_P521151	Plod2	NM_175869	300901	1.52	33.35	1.69E-05
A_44_P353618	S100a9	NM_053587	94195	1.50	31.89	9.27E-05
A_42_P484738	Ctgf	NM_022266	64032	1.50	31.54	2.78E-06
A_44_P681660	Fn1	NM_019143	25661	1.50	31.42	9.68E-06
A_44_P438295	T_predicted	XM_217890		1.48	29.97	1.96E-05
A_44_P451357	Sparc	NM_012656	24791	1.47	29.60	2.84E-06
A_42_P693964	Cyp26b1	NM_181087	312495	1.47	29.42	2.67E-06
A_44_P840118	TC547435	TC547435		1.46	29.09	1.56E-06
A_44_P562483	Nkd1_predicted	XM_001066780		1.46	29.06	5.29E-06
A_44_P208699	Col15a1	XM_216399	298069	1.46	29.01	2.47E-06
A_43_P17235	RGD1560062_predicted	XM_228209		1.46	28.89	4.57E-05
A_44_P271658	Pap	NM_053289	24618	1.46	28.85	0.000502
A_44_P276538	Gp49b	NM_001013894	292594	1.45	27.97	2.84E-06
A_44_P285534	Igfbp5	NM_012817	25285	1.45	27.91	2.46E-06
A_43_P12462	Msn	NM_030863	81521	1.44	27.72	1.63E-06
A_44_P237664	AW141875	AW141875	25358	1.44	27.44	4.61E-06
A_44_P319721	LOC499991	XM_001067033		1.44	27.36	3.71E-06
A_44_P198771	Chl1	XM_001077843		1.43	27.22	1.87E-05
A_42_P647599	Dcn	NM_024129	29139	1.43	26.92	0.000245
A_44_P500056	Gpx2	NM_183403	29326	1.43	26.92	0.000027
A_44_P749637	Chl1	XM_001077843		1.43	26.89	0.000048
A_42_P585695	Lox	NM_017061	24914	1.42	26.02	2.34E-05
A_44_P291626	CB585728	CB585728	306628	1.41	25.97	1.55E-05
A_44_P458021	Tnfrsf11b	NM_012870	25341	1.41	25.87	0.002919
A_44_P437721	CO566833	CO566833		1.41	25.85	4.21E-05
A_42_P521210	Thbs2	XM_214778	292406	1.41	25.62	2.84E-05
A_44_P941963	AW914058	AW914058		1.40	25.39	5.03E-06
A_44_P392849	Col5a2	XM_343564	85250	1.40	25.14	1.61E-05
A_44_P468549	Hmcn1_predicted	XM_222716	289094	1.39	24.64	7.14E-06
A_42_P728794	Srpx2_predicted	XM_228444		1.39	24.59	7.5E-06
A_44_P405232	Tmem119_predicted	XM_222278		1.39	24.55	8.63E-06
A_44_P452222	Itgal	NM_001033998	308995	1.39	24.47	1.62E-06
A_44_P695547	U39609	U39609		1.39	24.32	0.000135
A_44_P835675	CF110937	CF110937		1.38	23.99	5.39E-06
A_44_P210568	Fn1	NM_019143	25661	1.38	23.82	1.61E-05
A_44_P199096	Rarres1	NM_001014790	310486	1.37	23.62	2.03E-06
A_44_P150185	Ltbp2	NM_021586	59106	1.37	23.21	3.55E-06
A_44_P391982	Mmd	NM_001007673	303439	1.36	23.09	2.29E-06
A_42_P473398	Cxcl1	NM_030845	81503	1.36	22.92	0.000495
A_43_P11577	Anxa3	NM_012823	25291	1.36	22.76	2.88E-05
A_44_P435618	Ednrb	NM_017333	50672	1.35	22.62	5.03E-06
A_42_P556829	Wif1	NM_053738	114557	1.35	22.58	6.94E-05
A_44_P427346	Sulf1	NM_134378	171396	1.35	22.52	0.000112
A_43_P11683	F3	NM_013057	25584	1.35	22.36	5.79E-05

A_44_P304190	LOC310877	NM_001014044	310877	1.35	22.19	2.55E-06
A_44_P487269	RGD1359539	NM_001013945	299354	1.35	22.18	0.000108
A_43_P14249	CF110190	CF110190		1.34	21.97	6.47E-06
A_44_P503116	Sfrp2	XM_227314	310552	1.34	21.91	0.000144
A_42_P510386	Serping1	NM_199093	295703	1.33	21.60	2.39E-05
A_44_P240702	Lef1	NM_130429	161452	1.33	21.54	0.000071
A_44_P1052046	RGD1562717_predicted	XM_344015	363767	1.33	21.50	9.44E-06
A_44_P360772	Tagln	NM_031549	25123	1.33	21.37	3.62E-06
A_44_P506609	Stra6	NM_001029924	363071	1.33	21.32	0.000816
A_44_P806889	A_44_P806889	A_44_P806889		1.33	21.18	1.23E-05
A_43_P15638	BE126741	BE126741	116640	1.33	21.14	0.000189
A_44_P226906	St6gal1	NM_147205	25197	1.33	21.13	1.55E-05
A_44_P208566	Plxdc2_predicted	XM_341567		1.33	21.13	0.000416
A_43_P11881	Bcat1	NM_017253	29592	1.32	20.98	5.52E-05
A_44_P746420	Arl11	NM_001013433	364396	1.32	20.80	0.000278
A_42_P693316	Cfi	NM_024157	79126	1.32	20.70	1.87E-05
A_43_P16442	RGD1560062_predicted	XM_228209		1.32	20.70	9.11E-05
A_44_P144857	Enpep	NM_022251	64017	1.30	20.01	0.000661
A_44_P247081	Pla2g2a	NM_031598	29692	1.30	19.98	1.08E-05
A_44_P209788	Enpep	NM_022251	64017	1.30	19.84	0.001254
A_44_P1040642	Mafb	NM_019316	54264	1.29	19.71	0.000175
A_44_P247281	AI043862	AI043862	306748	1.29	19.62	9.78E-06
A_44_P392846	Col5a2	XM_343564	85250	1.29	19.53	0.000149
A_44_P332422	C1s	NM_138900	192262	1.29	19.49	0.000021
A_44_P230320	Il1b	NM_031512	24494	1.28	19.27	0.000185
A_44_P791638	TC519964	TC519964		1.28	19.25	2.55E-06
A_43_P10201	BG665180	BG665180		1.28	19.22	3.28E-05
A_44_P297777	Flna_predicted	XM_238167	293860	1.28	19.16	1.64E-06
A_44_P1048687	AW920967	AW920967		1.27	18.81	5.11E-06
A_44_P404836	Cp	NM_012532	24268	1.27	18.74	8.92E-05
A_44_P161470	Defa6	NM_001033076	613223	1.27	18.71	0.010567
A_44_P714397	M12981	M12981		1.27	18.41	0.000151
A_42_P598365	Colec12	NM_001025721	361289	1.26	18.34	4.77E-05
A_44_P267053	Myh10	U73303	79433	1.26	18.20	6.13E-06
A_44_P501112	Mmp9	NM_031055	81687	1.26	18.13	0.000105
A_44_P349553	Col6a1_predicted	XM_215375	294337	1.25	17.92	7.33E-06
A_44_P238421	Col1a1	XM_213440		1.25	17.82	2.67E-05
A_44_P146518	Col3a1	NM_032085	84032	1.25	17.81	2.52E-05
A_44_P864923	AW916836	AW916836		1.25	17.76	4.66E-05
A_44_P1007347	Tgm2	NM_019386	56083	1.25	17.61	3.69E-05
A_44_P1029131	RGD1311155	NM_001014166	361749	1.24	17.28	0.000952
A_43_P11684	Alpl	NM_013059	25586	1.23	17.07	0.000281
A_44_P432432	Plat	NM_013151	25692	1.23	17.06	3.61E-05
A_43_P15023	Thbd	NM_031771	83580	1.23	16.90	1.46E-05
A_44_P620927	Clecsf9	NM_001005897	450223	1.23	16.80	0.000276
A_44_P320999	XM_216800	XM_216800		1.22	16.78	3.93E-05
A_44_P409539	Plod2	NM_175869	300901	1.22	16.69	8.47E-05
A_42_P839593	AW142999	AW142999		1.22	16.64	1.31E-05
A_44_P734459	DV718231	DV718231		1.22	16.54	1.99E-06
A_44_P102557	Igkv28	L17078	116471	1.22	16.48	0.000112
A_44_P513385	Dab2	NM_024159	79128	1.22	16.47	0.000021
A_44_P210385	Nid1	M15797	25494	1.21	16.39	1.87E-05
A_42_P802424	Scgb1a1	NM_013051	25575	1.21	16.34	0.000193
A_44_P489608	Gfra4	NM_023967	66023	1.21	16.31	5.69E-05
A_44_P475448	X60291	X60291		1.21	16.30	0.000124
A_42_P685287	Fxyd5	NM_021909	60338	1.21	16.22	2.03E-06
A_44_P282443	AW920715	AW920715		1.21	16.21	4.78E-05
A_44_P270366	Cxcl5	NM_022214	60665	1.21	16.11	2.45E-06
A_42_P468712	Tacstd2	NM_001009540	494343	1.21	16.10	1.93E-05
A_44_P472874	Trpa1	NM_207608	312896	1.21	16.07	4.57E-05
A_43_P12783	Col1a2	NM_053356	84352	1.21	16.04	2.73E-05
A_43_P11672	Il1rl1	NM_013037	25556	1.20	16.00	0.00058
A_44_P278445	Il11	NM_133519	171040	1.20	15.97	0.000182

A_44_P245542	Glp2r	NM_021848	60432	1.20	15.96	8.27E-06
A_44_P715377	Cbfa2t1_predicted	XM_001053893		1.20	15.85	5.41E-06
A_44_P438060	Gna15	NM_053542	89788	1.20	15.80	6.62E-06
A_42_P508085	MGC105601	NM_001009620	287129	1.19	15.66	4.21E-05
A_44_P541276	RGD1307618_predicted	XM_224757		1.19	15.59	6.45E-05
A_44_P426345	Hsd11b1	NM_017080	25116	1.19	15.47	2.44E-05
A_44_P434724	Lox	NM_017061	24914	1.19	15.43	0.000121
A_42_P588944	Mgp	NM_012862	25333	1.19	15.36	0.000132
A_44_P217744	Ms4a4a_predicted	XM_342027	361734	1.19	15.36	6.54E-05
A_44_P520235	Tie1	XM_233462	89806	1.19	15.35	0.000185
A_44_P536476	Perc64	XM_001053103	554172	1.18	15.16	8.92E-05
A_44_P965553	CR754507	CR754507		1.18	15.16	5.62E-05
A_44_P471818	LOC689616	XM_001071384	689616	1.18	15.15	9.68E-06
A_44_P828142	TC544527	TC544527		1.18	15.08	4.87E-05
A_44_P128071	Olr311_predicted	NM_001000555	365385	1.18	15.02	7.5E-06
A_44_P247286	Cxcl14	NM_001013137	306748	1.17	14.94	2.27E-06
A_44_P232897	Procr	NM_001025733	362248	1.17	14.91	1.99E-06
A_44_P363116	Gm1960	NM_138522	171551	1.17	14.84	3.79E-06
A_44_P325766	Icos	NM_022610	64545	1.17	14.80	6.12E-06
A_44_P241616	Ogfrl1	NM_001025708	316290	1.17	14.79	1.61E-05
A_44_P370044	Lamc1	XM_001071300		1.17	14.71	7.99E-06
A_44_P123093	AW914230	AW914230	81521	1.17	14.65	9.89E-05
A_44_P322828	Dkk3	NM_138519	171548	1.16	14.33	4.01E-05
A_44_P447373	Ass	NM_013157	25698	1.16	14.31	4.94E-06
A_44_P169623	CF110660	CF110660		1.15	14.28	0.001254
A_44_P464145	Gpr155_predicted	XM_230989		1.15	14.17	0.000201
A_43_P12791	C1qr1	NM_053383	84398	1.15	13.99	0.000173
A_44_P253208	Adamts9_predicted	XM_232202		1.14	13.94	2.41E-06
A_43_P16767	Igfbp4	NM_001004274	360622	1.14	13.86	3.37E-06
A_44_P415193	Apobec3	NM_001033703	315137	1.14	13.77	1.51E-05
A_44_P457362	Col16a1	XM_345584	366474	1.14	13.74	0.000286
A_44_P731356	TC527039	TC527039		1.14	13.73	5.69E-05
A_44_P285681	Plg	XM_574314		1.13	13.61	0.000113
A_44_P1004886	MGC72614	NM_199105	310540	1.13	13.59	0.000116
A_44_P350521	Col1a1	XM_213440		1.13	13.49	7.33E-05
A_44_P311582	Garp_predicted	XM_218947	293135	1.13	13.48	0.000237
A_44_P444469	RGD1311080_predicted	XM_231864		1.13	13.46	3.94E-05
A_44_P112890	Il1rn	NM_022194	60582	1.13	13.44	3.89E-06
A_44_P380839	Rab31	NM_145094	246324	1.13	13.35	1.02E-06
A_44_P440178	RGD1560177_predicted	XM_574941		1.13	13.34	3.94E-05
A_44_P269536	Hmcn1_predicted	XM_222716	289094	1.12	13.28	0.001704
A_44_P315562	Cald1	NM_013146	25687	1.12	13.27	0.000213
A_44_P1029805	Krt2-7	XM_217035		1.12	13.24	1.57E-06
A_44_P404861	Mmp10	NM_133514	117061	1.12	13.10	0.000272
A_43_P10020	Lyz	NM_012771	25211	1.12	13.10	0.000135
A_44_P600376	M14434	M14434		1.12	13.05	0.000125
A_44_P516885	Eno2	NM_139325	24334	1.11	13.03	0.000165
A_44_P866659	RGD1566394_predicted	XM_001056204		1.11	12.98	7.24E-05
A_44_P803553	AI102989	AI102989		1.11	12.95	6.34E-05
A_42_P518855	Bcl2a1	NM_133416	170929	1.11	12.93	7.24E-06
A_44_P283850	XM_230982	XM_230982		1.11	12.92	5.59E-05
A_44_P199447	Elk3_predicted	XM_343198		1.11	12.86	5.51E-05
A_43_P10016	TC552854	TC552854		1.11	12.84	6.66E-05
A_44_P501541	Plxdc2_predicted	XM_341567		1.10	12.68	1.24E-05
A_44_P239300	XM_221198	XM_221198		1.10	12.63	1.94E-05
A_44_P1042783	MGC109519	NM_001024345	500450	1.10	12.61	2.09E-05
A_44_P857628	Olfml2a_predicted	XM_001060614		1.10	12.61	0.000108
A_43_P14894	Nppb	NM_031545	25105	1.10	12.57	0.000928
A_44_P366294	LOC690139	XM_001073426	690139	1.10	12.54	1.82E-05
A_43_P14604	AW143134	AW143134		1.10	12.54	0.003012
A_44_P1021644	Col4a1	XM_214400	290905	1.09	12.43	7.36E-05
A_44_P283464	AF217587	AF217587		1.09	12.40	7.76E-05
A_44_P355013	U02094	U02094		1.09	12.36	2.24E-05

A_42_P508984	Cxcl4	NM_001007729	360918	1.09	12.33	0.000165
A_44_P392845	Col5a2	XM_343564	85250	1.09	12.30	9.91E-06
A_44_P574648	LOC305633	XM_223745	305633	1.09	12.26	1.32E-06
A_44_P316761	XM_345754	XM_345754		1.09	12.23	0.004072
A_44_P463425	AW917764	AW917764	25285	1.09	12.23	7.24E-06
A_44_P1023977	Sh3kbp1	NM_053360	84357	1.09	12.17	7.43E-06
A_44_P1026838	CB547651	CB547651	25150	1.08	12.13	5.52E-06
A_44_P1014018	Rkhd1_predicted	XM_234921	299613	1.08	12.11	0.000932
A_44_P999845	Sh3kbp1	NM_053360	84357	1.08	12.10	2.67E-06
A_44_P1040532	DV715514	DV715514		1.08	12.09	0.000626
A_44_P411954	Cd2	NM_012830	497761	1.08	12.06	0.000226
A_44_P317912	Aldh1a2	NM_053896	116676	1.08	12.04	0.00117
A_44_P913796	LOC500046	XM_001059531		1.07	11.85	5.61E-05
A_44_P525235	Postn_predicted	XM_342245		1.07	11.84	0.000024
A_42_P495895	Dbn1	NM_031024	81653	1.07	11.81	2.09E-05
A_44_P199806	Pcdh7	NM_001004087	360942	1.07	11.78	0.000201
A_44_P667693	RGD1311080_predicted	XM_231864		1.07	11.76	5.59E-05
A_44_P941068	AW915656	AW915656	290350	1.07	11.71	1.51E-05
A_42_P541872	B3galt3	NM_001013158	310508	1.07	11.65	0.000116
A_44_P500949	Gpx3	NM_022525	64317	1.06	11.57	3.34E-05
A_42_P637189	Apln	NM_031612	58812	1.06	11.54	0.000622
A_44_P652447	ENSRNOT00000008294	ENSRNOT00000008294		1.06	11.53	3.69E-06
A_44_P928825	RGD1564316_predicted	XM_573919	498638	1.06	11.53	0.000123
A_44_P553267	XM_228540	XM_228540		1.06	11.47	0.000074
A_44_P311126	Clu	NM_053021	24854	1.06	11.43	7.06E-05
A_44_P541384	Cmtm3_predicted	XM_226200		1.06	11.42	0.000195
A_43_P15947	Nid1	XM_213954	25494	1.06	11.41	0.000118
A_44_P1038296	Cd68	NM_001031638	287435	1.06	11.39	2.41E-06
A_44_P236761	RGD1309019_predicted	XM_234839	314596	1.06	11.37	8.64E-05
A_42_P529711	BF554994	BF554994		1.05	11.34	8.59E-05
A_44_P739541	TC566878	TC566878		1.05	11.33	1.48E-05
A_44_P1044658	S43941	S43941		1.05	11.33	0.000121
A_44_P531129	M12822	M12822		1.05	11.31	0.000324
A_44_P172601	Icos	NM_022610	64545	1.05	11.28	0.000058
A_44_P536676	B3galt3	NM_001013158	310508	1.05	11.27	0.000129
A_43_P12257	Esm1	NM_022604	64536	1.05	11.23	0.00269
A_44_P664495	Prickle1	XM_001057488		1.05	11.22	1.68E-06
A_44_P414544	M84148	M84148	502832	1.05	11.15	8.74E-05
A_44_P260663	LOC689069	XM_001069394	689069	1.05	11.12	9.57E-05
A_44_P251023	Slc39a10_predicted	XM_343567		1.05	11.09	7.53E-06
A_44_P518127	MGC116096	NM_001024869	295062	1.04	11.06	1.32E-06
A_44_P1018957	Hsd11b1	NM_017080	25116	1.04	11.04	3.95E-06
A_44_P396267	ENSRNOT00000044631	ENSRNOT00000044631		1.04	11.03	7.81E-06
A_43_P21114	Cdh11	XM_341639	84407	1.04	10.98	0.000112
A_43_P15819	Prss22_predicted	XM_220222		1.04	10.96	0.000149
A_44_P256301	Grem1	NM_019282	50566	1.04	10.94	0.001679
A_44_P961911	TC544685	TC544685		1.04	10.92	0.000108
A_43_P20012	CB545387	CB545387		1.04	10.87	3.91E-05
A_44_P199187	MGC108823	NM_001012353	307414	1.03	10.83	0.001238
A_44_P135592	Mcoln3	NM_001012059	308022	1.03	10.77	0.000244
A_44_P212189	AW917894	AW917894		1.03	10.70	5.69E-05
A_44_P1025918	Vwf	XM_342759	116669	1.03	10.67	1.95E-05
A_44_P551075	Nfam1_predicted	XM_343297	362966	1.02	10.55	4.13E-05
A_44_P247956	LOC498113	XM_573321		1.02	10.54	0.000342
A_42_P714311	Ccl3	NM_013025	25542	1.02	10.52	0.000566
A_44_P438675	Pappa_predicted	XM_233037		1.02	10.52	0.000173
A_44_P116369	Smoc1	NM_001002835	314280	1.02	10.49	4.79E-05
A_44_P610377	TC545318	TC545318		1.02	10.46	0.000109
A_44_P372254	Slpi	NM_053372	84386	1.02	10.45	2.89E-05
A_42_P616287	C1r	XM_242644	312705	1.02	10.43	3.49E-05
A_42_P673011	LOC299339	XM_216791	299339	1.02	10.40	0.000102
A_44_P459144	Inhba	NM_017128	29200	1.02	10.39	0.000593
A_44_P245835	RGD1310110_predicted	XM_341307		1.01	10.35	0.000227

A_44_P187554	RGD1560062_predicted	XM_228209		1.01	10.33	0.000256
A_43_P18993	Lamb1_predicted	XM_216679		1.01	10.31	6.44E-06
A_42_P773945	B4galt1_mapped	XM_342820		1.01	10.29	2.06E-05
A_44_P990376	Gpnmb	NM_133298	113955	1.01	10.29	0.000666
A_42_P688802	Tcf8	XM_001056207		1.01	10.28	0.000279
A_44_P962936	Cdca4	NM_001037214	500727	1.01	10.26	0.000341
A_44_P245616	Nid1	XM_213954	25494	1.01	10.26	2.67E-06
A_42_P565621	Slamf9_predicted	XM_213932		1.01	10.23	4.8E-06
A_43_P15993	Itgam	NM_012711	25021	1.01	10.21	8.32E-05
A_44_P405374	LOC290071	NM_001039204		1.01	10.21	0.000014
A_44_P236144	Ptprc	XM_001062978		1.01	10.17	2.63E-05
A_44_P369612	LOC305633	XM_223745	305633	1.01	10.17	7.54E-06
A_44_P508162	Adamts5	NM_198761	304135	1.00	10.11	0.000288
A_44_P506817	BF287204	BF287204	24390	1.00	10.08	3.77E-06
A_44_P606490	RGD1563347_predicted	XM_001062448		1.00	10.01	3.86E-05
A_44_P492530	Lgr5	XM_235149		1.00	9.98	0.000261
A_44_P298331	LOC360619	XM_340894		1.00	9.97	0.000129
A_44_P504794	Gja7	XM_001081521		1.00	9.97	0.000861
A_44_P539274	Irak1bp1_predicted	XM_217210		1.00	9.96	0.000268
A_44_P289922	Cd48	NM_139103	245962	1.00	9.96	3.76E-05
A_42_P695401	Ccl2	NM_031530	24770	1.00	9.90	0.001201
A_44_P504041	RGD1565999_predicted	XM_216325		1.00	9.90	0.001625
A_43_P11522	Cd4	NM_012705	24932	0.99	9.85	4.16E-06
A_43_P19669	Itga4_mapped	XM_230033		0.99	9.84	5.72E-05
A_44_P468331	Rps6ka2	XM_341758	117269	0.99	9.83	1.02E-05
A_44_P1008867	Neto2_predicted	XM_226349		0.99	9.83	0.000301
A_43_P23190	LOC315883	NM_001014094	315883	0.99	9.83	0.000353
A_44_P382318	Pftk1_predicted	XM_342632		0.99	9.81	2.65E-05
A_44_P131290	RGD1559882_predicted	XM_237535	301709	0.99	9.72	3.83E-05
A_44_P384404	Ms4a11_predicted	XM_342028	361735	0.99	9.70	8.64E-05
A_44_P532548	LOC360627	NM_001014120	360627	0.99	9.70	0.000321
A_44_P325508	Anxa3	NM_012823	25291	0.99	9.68	8.54E-05
A_44_P218896	Cyr61	NM_031327	83476	0.98	9.66	2.85E-06
A_44_P247139	MGC112830	NM_001025718	361178	0.98	9.65	0.000407
A_44_P1024315	Slit3	NM_031321	83467	0.98	9.61	0.002771
A_44_P260036	Plau	NM_013085	25619	0.98	9.58	1.25E-05
A_44_P423566	RGD1310827	NM_001034010	362364	0.98	9.56	0.00031
A_44_P914607	Plxnd1_predicted	XM_232283		0.98	9.54	1.81E-05
A_44_P699964	TC540005	TC540005		0.98	9.54	0.000278
A_43_P16967	Snx10	NM_001013085	297096	0.98	9.52	0.000104
A_44_P671652	Lrrc8c	NM_001037179	289443	0.98	9.48	2.13E-05
A_44_P283561	Mgst1	NM_134349	171341	0.98	9.47	2.48E-06
A_44_P456958	Prox1_predicted	XM_223067		0.98	9.46	7.35E-06
A_44_P481160	Wnt5a	NM_022631	64566	0.98	9.46	0.000259
A_44_P916210	TC560958	TC560958		0.98	9.45	9.31E-05
A_42_P457003	Pdgfra	XM_214030		0.97	9.42	1.15E-05
A_44_P1022458	Tubb6	NM_001025675	307351	0.97	9.38	9.23E-07
A_44_P751273	BE116572	BE116572		0.97	9.38	0.000144
A_44_P548083	U46958	U46958		0.97	9.37	7.62E-05
A_44_P919600	TC551459	TC551459		0.97	9.36	0.000294
A_44_P359188	Krt1-5	NM_001008759	287698	0.97	9.36	0.001505
A_44_P894727	BF553172	BF553172		0.97	9.34	4.21E-05
A_44_P377751	ENSRNOT00000036332	ENSRNOT00000036332		0.97	9.33	4.91E-05
A_44_P487764	C1qa	NM_001008515	298566	0.97	9.32	4.68E-06
A_44_P283726	Ifit3	NM_001007694	309526	0.97	9.32	0.000219
A_44_P271511	Adam2	NM_020077	56806	0.97	9.30	0.000408
A_43_P22717	BE127091	BE127091		0.97	9.30	0.000164
A_44_P508683	XM_226886	XM_226886		0.97	9.30	5.74E-05
A_43_P21950	Samhd1_predicted	XM_230789	311580	0.97	9.28	1.59E-05
A_42_P763720	RGD1563994_predicted	XM_001064713		0.96	9.19	1.86E-05
A_44_P342271	Cd34_predicted	XM_223083		0.96	9.18	0.000145
A_44_P341731	BE108374	BE108374		0.96	9.17	1.13E-05
A_43_P12159	Cxcr4	NM_022205	60628	0.96	9.16	0.000057

A_44_P130166	LOC683788	XM_001064232		0.96	9.16	0.000218
A_44_P681836	CO572285	CO572285		0.96	9.16	0.000303
A_44_P805841	LOC498113	XM_573321		0.96	9.09	0.00053
A_44_P375616	Slc16a11_predicted	XM_213334		0.96	9.08	9.77E-05
A_43_P19178	Rasa3	XM_225020		0.96	9.07	1.57E-05
A_44_P370065	Nptx2	S82649		0.96	9.05	5.39E-06
A_42_P547157	CB547174	CB547174		0.96	9.03	9.96E-05
A_44_P996061	Fbn1	NM_031825	83727	0.95	9.01	8.65E-06
A_42_P647571	Cdh11	XM_341639	84407	0.95	9.00	5.39E-06
A_42_P735417	Fcgr2b	NM_175756	289211	0.95	8.99	0.000063
A_44_P993328	Ms4a11_predicted	XM_342028	361735	0.95	8.98	1.31E-05
A_44_P105225	Loxl2_predicted	XM_214225		0.95	8.97	2.82E-05
A_44_P1028196	LOC362934	NM_001017467	362934	0.95	8.95	7.62E-05
A_44_P414087	AA818205	AA818205	117582	0.95	8.93	0.004744
A_44_P362749	Emr1	NM_001007557	316137	0.95	8.92	6.76E-05
A_43_P18751	LOC294762	XM_215491	294762	0.95	8.92	1.51E-06
A_44_P463899	RGD1563400_predicted	XM_341045	360777	0.95	8.91	0.000214
A_44_P652293	Ly49i8	NM_001009486	494194	0.95	8.90	0.000123
A_43_P12395	Tas2r105	NM_023999	78985	0.95	8.90	0.000211
A_44_P423651	Prss12	NM_053504	85266	0.95	8.88	0.007274
A_44_P699523	TC535812	TC535812		0.95	8.87	0.000126
A_44_P956203	RGD1560293_predicted	XM_001059303		0.95	8.84	0.000685
A_44_P474559	Eng	NM_001010968	497010	0.95	8.83	0.000342
A_44_P175240	Cyp7b1	XM_342218		0.95	8.82	0.000708
A_44_P608078	TC539937	TC539937		0.94	8.80	0.000114
A_43_P15980	Angpt2	XM_344544	89805	0.94	8.80	0.002368
A_44_P582557	Gpr153	NM_001034855	619550	0.94	8.79	0.00058
A_44_P116539	AF217590	AF217590		0.94	8.76	0.001494
A_44_P197290	Col5a3	NM_021760	60379	0.94	8.76	9.23E-05
A_42_P546968	Pdgfrb	NM_031525	24629	0.94	8.76	1.87E-05
A_44_P515317	Igfbp4	NM_001004274	360622	0.94	8.69	8.61E-06
A_44_P436280	Pltp_predicted	XM_215939	296371	0.94	8.67	3.48E-05
A_44_P330392	Myh10	NM_031520	79433	0.94	8.67	8.57E-05
A_44_P170874	Ankrd22_predicted	XM_220065		0.94	8.65	0.000001
A_44_P462107	Dst_predicted	XM_237042		0.94	8.65	0.000285
A_44_P561084	Cdh13	NM_138889	192248	0.94	8.64	0.000164
A_44_P305223	Pecam	NM_031591	29583	0.93	8.56	0.000121
A_44_P309107	LOC686899	XM_001076227		0.93	8.55	0.00023
A_44_P320655	F2r	NM_012950	25439	0.93	8.55	0.000476
A_44_P236259	Prkch	NM_031085	81749	0.93	8.50	4.21E-05
A_44_P1071663	Gnb4	NM_001013910	294962	0.93	8.50	0.000243
A_44_P868808	LOC683788	XM_001064232		0.93	8.50	4.89E-06
A_44_P398033	Prnp	NM_012631	24686	0.93	8.50	6.55E-05
A_42_P784614	Htra1	NM_031721	65164	0.93	8.50	4.34E-05
A_42_P836598	Klhl8_predicted	XM_213995		0.93	8.50	2.28E-05
A_44_P487761	C1qa	NM_001008515	298566	0.93	8.48	4.14E-05
A_44_P525969	BF542741	BF542741		0.93	8.46	0.000433
A_42_P758222	Arg1	NM_017134	29221	0.93	8.46	2.62E-05
A_44_P422630	Serping1	NM_199093	295703	0.93	8.44	0.000071
A_42_P591344	Itgb2	XM_001069791	309684	0.92	8.39	0.000107
A_44_P404591	Hod	NM_133621	171160	0.92	8.38	3.09E-05
A_44_P933121	TC551872	TC551872		0.92	8.38	1.63E-06
A_44_P579684	TC527065	TC527065		0.92	8.38	0.000224
A_44_P314312	Nol9	XM_233702	313744	0.92	8.36	0.000278
A_44_P216654	Nap1l1	NM_053561	89825	0.92	8.34	4.38E-05
A_43_P11226	Map4k4_predicted	XM_217381		0.92	8.33	8.53E-07
A_44_P200292	XM_345665	XM_345665		0.92	8.32	2.65E-05
A_42_P499158	Vcam1	NM_012889	25361	0.92	8.32	0.000646
A_42_P689410	Sema3f_predicted	XM_236623	315996	0.92	8.30	7.14E-05
A_44_P146816	Runx2	XM_346016	367218	0.92	8.28	7.47E-05
A_44_P698351	RGD1564553_predicted	XM_577290		0.92	8.26	0.000379
A_44_P992535	Gpr116	NM_139110	245977	0.92	8.24	0.000472
A_44_P550785	Gbp5_predicted	XM_342352		0.92	8.23	6.28E-05

A_44_P117335	RGD1564641_predicted	XM_221446		0.92	8.22	0.00068
A_44_P994198	Glis2_predicted	XM_220169		0.91	8.21	0.000988
A_44_P246554	CB545053	CB545053		0.91	8.19	0.000293
A_44_P402655	Itgal	AY256462	308995	0.91	8.17	3.74E-05
A_44_P103102	Rps6ka6_predicted	XM_228473	317203	0.91	8.13	0.001344
A_44_P465188	Fads1	NM_053445	84575	0.91	8.12	0.000105
A_42_P822726	Myo1g	XM_573653	289785	0.91	8.12	5.97E-06
A_43_P12356	Tmeff1	NM_023020	63845	0.91	8.09	0.000336
A_42_P693948	TC554792	TC554792		0.91	8.09	7.21E-06
A_44_P914382	RGD1559432_predicted	XM_001074860		0.91	8.08	5.95E-05
A_43_P14622	Cybrd1	NM_001011954	295669	0.91	8.06	2.61E-05
A_44_P522318	Gpr109a	NM_181476	353250	0.91	8.04	0.002964
A_44_P715240	RGD1562847_predicted	XM_213732	288568	0.91	8.04	1.33E-05
A_44_P236523	Hoxd10_predicted	XM_221510		0.90	8.03	0.000324
A_44_P288029	Colec12	NM_001025721	361289	0.90	8.00	0.000437
A_42_P525876	Erg	NM_133397	170909	0.90	7.96	7.66E-05
A_44_P332606	C2	NM_172222	24231	0.90	7.95	0.000109
A_44_P622091	XM_579788	XM_579788		0.90	7.93	5.31E-05
A_44_P1023538	C3	NM_016994	24232	0.90	7.92	0.000312
A_44_P607347	TC540229	TC540229		0.90	7.90	0.000267
A_44_P392234	Fgf7	NM_022182	29348	0.90	7.87	0.002026
A_43_P12613	Apln	NM_031612	58812	0.90	7.86	0.000618
A_43_P14911	Il1b	NM_031512	24494	0.90	7.85	0.000386
A_44_P807940	CV116643	CV116643		0.89	7.85	0.000161
A_44_P421534	Aif1	NM_017196	29427	0.89	7.85	1.14E-05
A_44_P1058055	Slc39a10_predicted	XM_343567		0.89	7.85	4.77E-05
A_42_P504852	Ptprb_predicted	XM_235156		0.89	7.83	1.72E-05
A_43_P10498	XM_237039	XM_237039		0.89	7.82	9.23E-07
A_44_P510961	Aebp1_predicted	XM_223583		0.89	7.81	0.001384
A_44_P412998	LOC362934	NM_001017467	362934	0.89	7.80	0.00026
A_44_P684304	RGD1565160_predicted	XM_001065851		0.89	7.80	2.14E-05
A_42_P532103	Anxa2	NM_019905	56611	0.89	7.74	3.92E-05
A_44_P541397	Procr	NM_001025733	362248	0.89	7.73	1.53E-06
A_44_P179745	Fbn1	NM_031825	83727	0.89	7.69	1.83E-05
A_44_P149510	Numbl	NM_001033888	292732	0.88	7.64	0.001185
A_44_P996080	Anxa6	NM_024156	79125	0.88	7.64	8.61E-06
A_44_P666098	BF286307	BF286307		0.88	7.60	0.000301
A_44_P526661	Ranbp5_predicted	XM_224534	306182	0.88	7.60	5.07E-05
A_44_P494510	LOC688913	XM_001068808	688913	0.88	7.59	4.86E-05
A_43_P19561	Lypd1	NM_001007727	360838	0.88	7.58	0.000605
A_43_P16511	LOC679150	XM_001055448		0.88	7.57	9.29E-06
A_42_P572521	C5r1	NM_053619	113959	0.88	7.54	1.72E-05
A_44_P539175	arhgef6	NM_001005565	363509	0.88	7.54	8.46E-05
A_44_P250281	Hip1	XM_347168	192154	0.88	7.54	7.36E-05
A_44_P311541	Ccr6	NM_001013145	308163	0.88	7.52	0.000124
A_43_P12205	Eltd1	NM_022294	64124	0.87	7.48	0.000567
A_44_P475159	Thy1	NM_012673	24832	0.87	7.46	0.00015
A_44_P761764	Rin3_predicted	XM_001066908		0.87	7.45	6.54E-05
A_44_P352195	A_44_P352195	A_44_P352195		0.87	7.44	4.79E-05
A_44_P928419	Mdn1	BC086440	362498	0.87	7.43	0.000145
A_44_P171440	Apoe	NM_138828	25728	0.87	7.40	0.000215
A_42_P693821	Fosl1	NM_012953	25445	0.87	7.40	1.52E-05
A_43_P13307	Gpm6b	NM_138846	192179	0.87	7.39	0.007567
A_44_P849592	BF545957	BF545957		0.87	7.39	0.000182
A_43_P15247	Tac1	NM_012666	24806	0.87	7.37	0.000195
A_44_P477650	Centa2	NM_020101	56826	0.87	7.37	5.26E-05
A_44_P342047	Rgs10	XM_341936		0.87	7.37	8.63E-06
A_44_P281481	Rarres2	NM_001013427	297073	0.86	7.32	0.003281
A_44_P438117	Rgs1	NM_019336	54289	0.86	7.30	2.62E-05
A_44_P175364	Mafb	NM_019316	54264	0.86	7.30	8.92E-05
A_44_P127337	RGD1561067_predicted	XM_573923	498642	0.86	7.30	3.35E-06
A_44_P301936	Anxa3	NM_012823	25291	0.86	7.29	0.000188
A_44_P833626	Pls3	XM_001057425		0.86	7.28	1.13E-05

A_44_P210173	Tnfrsf12a	NM_181086	302965	0.86	7.28	5.03E-06
A_44_P446944	AI136427	AI136427		0.86	7.26	7.74E-05
A_44_P151708	S82585	S82585		0.86	7.24	0.00146
A_42_P509365	Vim	NM_031140	81818	0.86	7.20	7.18E-05
A_43_P11489	S100a4	NM_012618	24615	0.86	7.19	8.35E-05
A_44_P267024	S81289	S81289		0.86	7.19	0.001065
A_44_P399249	Tgm1	NM_031659	60335	0.86	7.17	0.000271
A_44_P1010416	P2ry13	NM_001002853	310444	0.85	7.15	7.52E-05
A_44_P107766	RGD1309540	NM_001013918	295930	0.85	7.15	0.00017
A_44_P508554	Ncf2_predicted	XM_344156		0.85	7.14	0.000383
A_44_P540992	Ereg	NM_021689	59325	0.85	7.13	1.86E-05
A_43_P13100	Slc28a3	NM_080908	140944	0.85	7.13	0.000259
A_44_P271650	Rab31	NM_145094	246324	0.85	7.12	4.42E-05
A_44_P288287	RGD1307724_predicted	XM_232716	297865	0.85	7.10	9.31E-05
A_44_P992516	Hmha1_predicted	XM_001073188		0.85	7.10	0.00021
A_44_P470096	AI501238	AI501238		0.85	7.10	7.82E-05
A_44_P309466	Ephb6	XM_231650		0.85	7.10	0.000453
A_44_P140358	Samhd1_predicted	XM_230789	311580	0.85	7.05	7.74E-05
A_44_P452240	Atp1b2	NM_012507	24214	0.85	7.04	0.001991
A_42_P758810	Bdkrb2	M59967	25245	0.85	7.03	8.67E-05
A_44_P620369	AW920064	AW920064		0.85	7.03	0.001178
A_44_P547771	Cited1	NM_172055	64466	0.85	7.00	0.000491
A_44_P443751	Pdpn	NM_019358	54320	0.85	7.00	0.000288
A_44_P690802	LOC498236	XM_573459		0.84	6.99	0.00012
A_44_P1051894	XM_213775	XM_213775		0.84	6.98	3.58E-05
A_44_P187111	M62828	M62828		0.84	6.97	0.001261
A_44_P272212	XM_213024	XM_213024		0.84	6.95	0.00026
A_42_P751742	LOC683930	XM_001069923		0.84	6.95	2.82E-05
A_44_P309913	CB547989	CB547989	691153	0.84	6.94	0.000938
A_44_P484889	Ifitm3	XM_341957	361673	0.84	6.94	4.72E-05
A_44_P180127	Igfbp3	NM_012588	24484	0.84	6.94	6.34E-06
A_44_P488956	Mrvldc1	BC088295	309375	0.84	6.93	2.39E-06
A_44_P233867	Spon1	NM_172067	64456	0.84	6.92	0.000191
A_44_P604787	AW917268	AW917268		0.84	6.91	0.00008
A_42_P529550	Gng8	NM_139185	245986	0.84	6.89	0.000942
A_44_P531245	S57440	S57440		0.84	6.88	0.000465
A_44_P549494	Cd74	NM_013069	25599	0.84	6.87	0.000032
A_44_P428420	Ctsz	NM_183330	252929	0.84	6.87	2.09E-05
A_44_P1049827	Nsg1	NM_024128	25247	0.84	6.87	0.004842
A_44_P298049	Ncf1	NM_053734	114553	0.84	6.86	1.74E-05
A_44_P114207	Hk3	NM_022179	25060	0.84	6.85	0.000626
A_44_P640644	TC519882	TC519882		0.84	6.84	0.000993
A_44_P144602	LOC301289	XM_236985		0.83	6.84	4.61E-06
A_44_P274573	Plxnd1_predicted	XM_232283		0.83	6.84	0.000514
A_43_P11804	Ptn	NM_017066	24924	0.83	6.83	4.34E-05
A_44_P421322	Nox1	NM_053683	114243	0.83	6.80	3.79E-06
A_44_P992908	Hspb1	NM_031970	24471	0.83	6.80	9.95E-05
A_44_P439597	BG673065	BG673065		0.83	6.79	0.002729
A_44_P449623	CLIC6	NM_176078	304081	0.83	6.78	0.000385
A_42_P693096	AI454457	AI454457		0.83	6.77	9.26E-06
A_44_P283609	RGD1307973_predicted	XM_222150	304469	0.83	6.77	0.000698
A_44_P168405	Fcgr3	NM_053843	116591	0.83	6.76	0.000196
A_44_P540126	Pros1	NM_031086	81750	0.83	6.75	8.57E-05
A_44_P178811	Elk3_predicted	XM_343198		0.83	6.74	3.03E-05
A_44_P441339	Phyhd1	NM_001013081	296621	0.83	6.74	0.000698
A_44_P367688	RT1-Da	NM_001008847	294269	0.83	6.73	9.18E-05
A_44_P144980	Creb3l2	NM_001012188	362339	0.83	6.72	0.000161
A_44_P1000543	CA339586	CA339586		0.83	6.72	0.000539
A_43_P12467	Pde1a	NM_030871	81529	0.83	6.71	0.001681
A_42_P602570	Bex1	NM_001037365	501625	0.83	6.70	0.00037
A_44_P879764	B4galt1_mapped	XM_342820		0.82	6.67	3.31E-05
A_43_P21885	Antxr1	XM_342718		0.82	6.67	0.000217
A_44_P883396	LOC500046	XM_001061323	500046	0.82	6.66	0.000961

A_44_P478515	LOC300225	XM_001063650		0.82	6.65	2.83E-06
A_42_P743682	Lgals2	NM_133599	171134	0.82	6.63	0.010934
A_44_P592613	RGD1560766_predicted	XM_001062621		0.82	6.60	0.000163
A_44_P238312	Dlgh4	NM_019621	29495	0.82	6.60	0.000215
A_43_P23023	RGD1309543_predicted	XM_342085	361790	0.82	6.58	6.45E-06
A_42_P467630	Slc22a1	NM_012697	24904	0.82	6.57	8.98E-05
A_44_P991532	RT1-Da	NM_001008847	294269	0.82	6.55	1.27E-05
A_44_P592145	BC098778	BC098778		0.82	6.55	0.000187
A_43_P16491	Col16a1	XM_345584	366474	0.82	6.54	8.92E-05
A_44_P273468	Slc25a4	NM_053515	85333	0.81	6.53	0.000039
A_43_P19763	Tlr2	NM_198769	310553	0.81	6.51	1.99E-06
A_44_P297100	LOC499022	XM_574316	266718	0.81	6.50	4.86E-05
A_44_P742116	AI454044	AI454044	689039	0.81	6.48	2.73E-05
A_42_P660129	Casp12	NM_130422	156117	0.81	6.46	9.24E-06
A_44_P650885	LOC680308	XM_001056213		0.81	6.45	0.000218
A_44_P602702	CO555173	CO555173		0.81	6.43	8.84E-05
A_43_P15515	Ccr5	NM_053960	117029	0.81	6.42	0.000128
A_44_P745610	TC555784	TC555784		0.81	6.41	1.95E-05
A_42_P807697	Tyrobp	NM_212525	361537	0.81	6.40	2.33E-05
A_44_P919314	Sema3c_predicted	XM_001064163		0.80	6.38	0.004722
A_44_P579594	Sart2_predicted	XM_345110		0.80	6.38	0.000114
A_44_P932042	BF544728	BF544728		0.80	6.37	0.001345
A_43_P18738	Mrc1_predicted	XM_001072782		0.80	6.37	0.000379
A_42_P572258	AW920282	AW920282	296269	0.80	6.36	0.000405
A_44_P136857	LOC301509	XM_237286	301509	0.80	6.36	4.42E-05
A_44_P355152	Kpna3	BC111706	361055	0.80	6.33	0.000966
A_44_P458591	CB583358	CB583358	316325	0.80	6.32	8.92E-05
A_44_P546117	LOC361346	NM_001017462	361346	0.80	6.31	0.000384
A_43_P16277	S63458	S63458		0.80	6.30	0.006743
A_44_P503115	Sfrp2	XM_227314	310552	0.80	6.30	0.00198
A_44_P602851	AW918217	AW918217		0.80	6.30	0.000433
A_44_P340587	CB548303	CB548303		0.80	6.30	4.46E-05
A_42_P555725	RGD1563977_predicted	XM_220117	309557	0.80	6.30	0.000357
A_44_P450075	Ifit1_predicted	XM_220058		0.80	6.30	0.004143
A_44_P445238	Slc4a7	NM_058211	117955	0.80	6.29	4.16E-05
A_44_P464045	Arl11	NM_001013433	364396	0.80	6.29	0.001754
A_42_P652975	Arhgap9	XM_576241		0.80	6.28	2.95E-05
A_43_P12404	Evl	NM_024147	79115	0.80	6.28	9.63E-05
A_44_P852945	TC553952	TC553952		0.80	6.28	0.000175
A_44_P396286	RGD1563434_predicted	XM_228786	317391	0.80	6.26	4.57E-05
A_44_P452176	Chi3l1	NM_053560	89824	0.80	6.26	0.00391
A_44_P209149	Myo5a	NM_022178	25017	0.80	6.25	1.09E-05
A_43_P23209	Dync2li1	NM_001013940	298767	0.80	6.24	0.000214
A_44_P497262	Tcf7_predicted	XM_343891	363595	0.79	6.22	3.39E-05
A_44_P252483	Cmkor1	NM_053352	84348	0.79	6.22	0.000224
A_44_P851230	BF558478	BF558478		0.79	6.22	7.74E-05
A_44_P132960	S100a3	NM_053681	114216	0.79	6.20	0.000533
A_43_P11604	Timp3	NM_012886	25358	0.79	6.20	0.000351
A_44_P240781	Cav	NM_031556	25404	0.79	6.18	9.86E-05
A_44_P458065	Cd81	NM_013087	25621	0.79	6.17	0.000335
A_44_P653221	CA511571	CA511571	314616	0.79	6.16	0.000486
A_44_P294838	Il1a	NM_017019	24493	0.79	6.16	7.38E-05
A_44_P402157	AI409745	AI409745	290401	0.79	6.15	0.000364
A_44_P351209	Phlda1	NM_017180	29380	0.79	6.15	2.84E-06
A_44_P436265	Nkd1_predicted	XM_344728		0.79	6.14	0.00027
A_44_P309170	Alpk3_predicted	XM_344916	365298	0.79	6.13	3.55E-05
A_44_P635138	Mrc1_predicted	XM_001072782		0.79	6.13	0.000415
A_44_P558821	Ptrf_predicted	XM_001081467		0.79	6.13	0.000048
A_42_P686820	Sgce	NM_001002023	432360	0.79	6.13	0.000553
A_44_P425963	LOC679150	XM_001055448		0.79	6.11	2.69E-05
A_44_P716397	BF564017	BF564017		0.79	6.10	0.000256
A_44_P458074	Id3	NM_013058	25585	0.78	6.09	2.91E-05
A_42_P651202	Sat2_predicted	XM_340825		0.78	6.09	4.71E-05

A_44_P377886	LOC679629	XM_001053796	679629	0.78	6.06	0.000567
A_44_P674069	CO562195	CO562195	307343	0.78	6.06	8.05E-05
A_42_P689357	Pctk2	XM_235049		0.78	6.06	0.000368
A_44_P358203	Mark1	NM_053947	117016	0.78	6.05	0.000215
A_44_P819648	Cmtm3_predicted	XM_226200		0.78	6.05	0.000274
A_44_P796146	TC531316	TC531316		0.78	6.04	0.005497
A_42_P777761	40060	NM_001011893	287606	0.78	6.02	0.000191
A_44_P164824	Ttyh3_predicted	XM_221962		0.78	6.02	6.03E-05
A_44_P1035656	Cd24	NM_012752	25145	0.78	6.00	0.000283
A_44_P791682	Usp6nl_predicted	XM_001070749		0.78	6.00	0.002567
A_44_P533794	Tnfrsf1b	NM_130426	156767	0.78	5.99	2.78E-06
A_44_P237994	Abcg1	NM_053502	85264	0.78	5.97	2.92E-05
A_44_P961758	LOC683640	XM_001064629		0.77	5.93	0.008173
A_44_P749094	CPG2	NM_001029909	499010	0.77	5.92	3.34E-05
A_42_P585750	Cd300le_predicted	XM_340932		0.77	5.92	4.89E-06
A_44_P248090	Thbs2	XM_214778	292406	0.77	5.91	0.002723
A_44_P246538	Tgfb2	NM_031131	81809	0.77	5.91	0.0006
A_44_P133196	Adamts12_predicted	XM_226837		0.77	5.90	0.027074
A_44_P914346	LOC682083	XM_001061995		0.77	5.90	0.001317
A_44_P325791	Ywhag	NM_019376	56010	0.77	5.88	7.72E-06
A_44_P161841	Alox12_predicted	XM_213369		0.77	5.86	0.000238
A_43_P21482	Centd3_predicted	XM_001066167		0.77	5.85	0.000432
A_44_P477768	Ccnd2	NM_022267	64033	0.77	5.85	0.001899
A_44_P285741	Cav	NM_031556	25404	0.77	5.84	3.92E-05
A_44_P288291	Phlda3	NM_001012206	363989	0.77	5.84	3.85E-05
A_44_P118914	Z93359	Z93359		0.76	5.81	0.007133
A_44_P171220	Rps6kl1_predicted	XM_234419		0.76	5.81	0.000248
A_43_P12881	Cysltr1	NM_053641	114099	0.76	5.81	5.49E-05
A_44_P762190	TC558399	TC558399		0.76	5.81	4.06E-05
A_43_P17941	Zfpm2_predicted	XM_235253	314930	0.76	5.80	0.001565
A_44_P501162	L17080	L17080		0.76	5.78	0.000639
A_44_P229820	Itga5_mapped	XM_235707		0.76	5.78	2.84E-05
A_44_P594136	TC566486	TC566486		0.76	5.76	0.005273
A_44_P220256	Prtfdc1_predicted	XM_214518		0.76	5.74	0.000092
A_42_P489681	BC085903	BC085903		0.76	5.71	0.001153
A_44_P620208	BF549419	BF549419		0.76	5.70	0.000414
A_44_P763036	TC562474	TC562474		0.76	5.70	0.000202
A_44_P372587	Cx3cr1	NM_133534	171056	0.76	5.70	4.8E-06
A_42_P522899	Tgfb1	NM_021578	59086	0.75	5.69	1.52E-05
A_43_P17697	Ms4a6b	NM_001006975	293749	0.75	5.68	5.83E-06
A_42_P675853	Rhoj	NM_001008320	299145	0.75	5.68	0.00312
A_42_P520349	Mgl1	NM_022393	64195	0.75	5.68	0.000651
A_44_P669616	RGD1564008_predicted	XM_576044	500666	0.75	5.67	0.000014
A_42_P524370	Tcf4	NM_053369	84382	0.75	5.66	3.56E-06
A_44_P487021	Rasl11b	NM_001002830	305302	0.75	5.66	0.000251
A_44_P685064	TC558085	TC558085		0.75	5.65	0.00365
A_44_P149161	Psat1	NM_198738	293820	0.75	5.65	6.94E-05
A_44_P445070	Lgals1	NM_019904	56646	0.75	5.64	0.00036
A_44_P367465	Fzd9	NM_153305	266608	0.75	5.62	0.000553
A_44_P672002	TC537922	TC537922		0.75	5.62	1.57E-06
A_44_P515627	Igj	XM_341195	360922	0.75	5.61	0.003408
A_44_P808277	Srf_predicted	XM_001066019		0.75	5.60	2.47E-06
A_44_P332429	C1s	NM_138900	192262	0.75	5.60	0.00027
A_44_P109684	AF220555	AF220555		0.75	5.59	0.000346
A_44_P471318	Slc44a2_predicted	XM_343355	363024	0.75	5.56	0.000505
A_44_P592983	TC541993	TC541993		0.74	5.56	0.000577
A_44_P147328	RGD1560248_predicted	XM_001066238		0.74	5.56	5.54E-06
A_44_P367525	Hgf	NM_017017	24446	0.74	5.54	0.001029
A_44_P578146	Eif2c1_predicted	XM_233544	313594	0.74	5.54	0.000302
A_44_P503022	BF554877	BF554877		0.74	5.54	0.001318
A_42_P542744	Dchs1_predicted	XM_219128		0.74	5.53	8.53E-05
A_44_P352951	RGD1309472	NM_001013969	303333	0.74	5.53	0.000585
A_44_P515548	RGD1562847_predicted	XM_213732	288568	0.74	5.52	0.000233

A_44_P239155	Gpr68_predicted	XM_234483		0.74	5.52	0.001643
A_42_P832728	Cst7_predicted	XM_230714		0.74	5.52	0.000301
A_43_P21836	Lama2_predicted	XM_219866	309368	0.74	5.52	0.003722
A_44_P257582	Csf1r	NM_001029901	307403	0.74	5.51	5.65E-05
A_44_P1039616	LOC498022	XM_213514	303666	0.74	5.51	0.000294
A_43_P12508	Ptger2	NM_031088	81752	0.74	5.51	0.000771
A_42_P515405	Ier3	NM_212505	294235	0.74	5.48	1.04E-05
A_43_P12533	Tgfbr2	NM_031132	81810	0.74	5.48	3.39E-05
A_43_P15589	Ppat	XM_579602	117544	0.74	5.47	0.000055
A_44_P1038773	Gpx7_predicted	XM_216473		0.74	5.47	0.000642
A_42_P497225	Mfge8	NM_012811	25277	0.74	5.47	9.24E-05
A_44_P397836	Npc1l1	NM_001002025	432367	0.74	5.47	0.001182
A_44_P775722	ENSRNOT00000043097	ENSRNOT00000043097		0.74	5.47	0.002808
A_44_P685305	TC525394	TC525394		0.74	5.46	0.002929
A_44_P176051	Icam2	NM_001007725	360647	0.74	5.46	0.000408
A_44_P299571	Thedc1	NM_022705	64669	0.74	5.45	0.046894
A_44_P343267	CA510534	CA510534		0.74	5.45	0.000496
A_44_P696774	AW141857	AW141857		0.74	5.44	0.002566
A_44_P158837	NP516919	NP516919		0.74	5.44	3.57E-05
A_42_P815346	AA998118	AA998118	24584	0.74	5.44	0.000132
A_43_P14754	AW920712	AW920712		0.74	5.44	0.00039
A_44_P340236	Clec4a1	NM_001005890	362430	0.73	5.43	0.000556
A_44_P538658	Slc7a7	NM_031341	83509	0.73	5.43	0.000697
A_43_P11537	Rbp1	NM_012733	25056	0.73	5.43	0.000103
A_43_P19794	LOC687536	XM_001079002		0.73	5.42	8.54E-05
A_44_P333010	Mmrn2_predicted	XM_224646		0.73	5.42	0.002716
A_44_P429453	Lama5	XM_215963	140433	0.73	5.41	7.38E-06
A_44_P854356	RGD1564108_predicted	XM_238042	291936	0.73	5.40	0.003364
A_44_P443220	Frzb	XM_215757	295691	0.73	5.40	0.001649
A_44_P181548	AW915616	AW915616	116723	0.73	5.39	0.000109
A_44_P993370	Plekhc1	NM_001011915	289992	0.73	5.39	0.000457
A_44_P417031	Tm6sf1_predicted	XM_341878		0.73	5.39	0.000629
A_44_P146570	CF109691	CF109691		0.73	5.38	0.000206
A_44_P573545	BF290411	BF290411	294797	0.73	5.38	0.000762
A_44_P322518	Cct6a	NM_001033684	288620	0.73	5.37	3.84E-05
A_43_P18356	Ift57_predicted	XM_221496		0.73	5.37	8.35E-05
A_44_P137448	Ptgs1	NM_017043	24693	0.73	5.36	0.000291
A_42_P627998	Igfbp3	NM_012588	24484	0.73	5.36	6.94E-05
A_44_P278593	Ptgs2	NM_017232	29527	0.73	5.35	0.002267
A_42_P465601	Myh10	NM_031520	79433	0.73	5.35	0.002272
A_44_P335679	Bmp5_predicted	XM_236415		0.73	5.35	0.000326
A_44_P339297	DV715460	DV715460		0.73	5.35	0.00491
A_44_P508566	Olfml2b_predicted	XM_222868		0.73	5.34	0.000683
A_44_P457526	Ptrf_predicted	XM_220982		0.73	5.34	3.12E-05
A_44_P915843	TC559204	TC559204		0.73	5.34	2.86E-05
A_44_P669066	TC534310	TC534310		0.73	5.32	0.002388
A_44_P255817	RGD1564330_predicted	XM_343179	362848	0.73	5.31	4.89E-05
A_44_P398167	Prdx1	NM_057114	117254	0.72	5.30	0.000023
A_44_P561048	Rcsd1_predicted	XM_341147		0.72	5.30	0.000172
A_44_P409694	Serpine2	XM_343604		0.72	5.29	0.00881
A_44_P344444	Tspan2	NM_022589	64521	0.72	5.29	0.001438
A_44_P456728	Cugbp2	NM_017197	29428	0.72	5.29	1.02E-05
A_42_P686427	AW142560	AW142560		0.72	5.28	0.000109
A_44_P532297	RGD1309085_predicted	XM_232684	297821	0.72	5.28	0.001557
A_44_P366723	Igf1	NM_178866	24482	0.72	5.28	9.19E-05
A_44_P412290	C1qg	NM_001008524	362634	0.72	5.27	5.87E-05
A_44_P376900	LOC681783	XM_001058535		0.72	5.27	0.000938
A_44_P147572	XM_234581	XM_234581		0.72	5.27	0.038165
A_44_P184484	Orc1l	NM_177931	313479	0.72	5.26	0.003209
A_43_P12955	Fcgr3	XM_001077008		0.72	5.26	0.000109
A_44_P326742	Dixdc1	NM_001037654	363062	0.72	5.26	0.00054
A_44_P128126	Mycl1_mapped	XM_233490	298506	0.72	5.26	0.001103
A_44_P270468	Slc15a3	NM_139341	246239	0.72	5.26	0.00011

A_44_P289080	Ptpro	NM_017336	50677	0.72	5.26	0.000301
A_44_P105448	Col4a5_predicted	XM_343778	363457	0.72	5.25	0.000171
A_43_P22317	Tiam1	XM_221672	304109	0.72	5.25	7.54E-05
A_44_P337465	RGD1560037_predicted	XM_001066396		0.72	5.25	0.00065
A_44_P302923	CB606228	CB606228		0.72	5.24	4.15E-05
A_44_P164027	XM_345738	XM_345738		0.72	5.24	0.011707
A_44_P552452	RT1-Bb	NM_001004084	309622	0.72	5.23	2.65E-05
A_44_P365436	Myl9_predicted	XM_215905		0.72	5.22	7.07E-05
A_42_P843692	Cadps	NM_013219	26989	0.72	5.20	0.008824
A_44_P290552	Mchr1	NM_031758	83567	0.72	5.19	0.000484
A_44_P100301	Selpl_predicted	NM_001013230	363930	0.71	5.18	7.5E-06
A_44_P209817	Cav	BC078744	25404	0.71	5.16	0.006511
A_44_P696994	Ifit2	NM_001024753	294091	0.71	5.16	0.00385
A_44_P1038028	Tnfrsf12a	NM_181086	302965	0.71	5.14	0.000197
A_44_P773166	RGD1565845_predicted	XM_001063426		0.71	5.14	0.008495
A_44_P538357	Bmp4	NM_012827	25296	0.71	5.14	6.48E-05
A_44_P366902	LOC360932	XR_009136	360932	0.71	5.13	0.000338
A_44_P438815	LOC501065	XM_576480		0.71	5.13	3.09E-05
A_44_P528988	AA964627	AA964627		0.71	5.12	4.64E-05
A_42_P629062	Cytor4	XM_001080961		0.71	5.12	1.53E-05
A_44_P822447	RGD1565540_predicted	XM_573975		0.71	5.12	1.57E-06
A_44_P880441	AW921293	AW921293		0.71	5.11	0.00213
A_44_P550581	Dmpk_predicted	XM_218411		0.71	5.10	0.00013
A_44_P187246	RGD1562525_predicted	XM_344096		0.71	5.10	0.003588
A_42_P669544	Cpm_predicted	XM_235168		0.71	5.10	0.000637
A_44_P953483	Kcnq5	CF111745		0.71	5.09	2.62E-05
A_44_P1034926	BG671620	BG671620	64160	0.71	5.08	5.39E-06
A_44_P170527	Fzd1	NM_021266	58868	0.71	5.07	0.000548
A_43_P22312	Gnat2_predicted	XM_345271		0.70	5.07	0.00029
A_44_P247219	Cited4	NM_053699	114491	0.70	5.07	0.000074
A_44_P805851	AF217588	AF217588		0.70	5.06	0.006682
A_44_P351211	Phlda1	NM_017180	29380	0.70	5.05	1.87E-06
A_44_P501960	RGD1306248	XM_221437	288091	0.70	5.05	0.000363
A_44_P807696	TC539857	TC539857		0.70	5.05	0.000301
A_44_P538616	Z93363	Z93363		0.70	5.05	0.002844
A_43_P21634	C4a	NM_031504	24233	0.70	5.04	0.000181
A_44_P192568	Gm2a	NM_172335	282838	0.70	5.04	3.41E-05
A_44_P1053404	LOC498279	XM_001053627		0.70	5.01	2.82E-06
A_44_P183039	Utp14a	NM_001014113	317579	0.70	5.01	0.001372
A_44_P390700	Fhl2	NM_031677	63839	0.70	5.00	1.69E-05
A_44_P758339	CF110144	CF110144		0.70	4.98	0.00715
A_44_P105304	Dapp1_predicted	XM_342348		0.70	4.98	1.64E-05
A_42_P566264	Mfng	NM_199110	315119	0.70	4.97	0.001332
A_44_P276352	Id3	NM_013058	25585	0.69	4.95	7.96E-05
A_44_P500803	RT1-Bb	NM_001004084	309622	0.69	4.94	6.77E-05
A_44_P415230	Trim58_predicted	XM_220473		0.69	4.94	0.000989
A_42_P816402	Wdr31	NM_001011976	298096	0.69	4.93	0.001111
A_43_P21945	Ppl_predicted	XM_220174		0.69	4.93	0.000185
A_44_P121517	Cd180_predicted	XM_226731		0.69	4.92	0.001873
A_43_P11614	Anxa1	NM_012904	25380	0.69	4.91	0.000481
A_44_P255109	Snn	NM_001034083	29140	0.69	4.90	0.000196
A_44_P139939	Cp	NM_012532	24268	0.69	4.90	0.002857
A_44_P347283	A_44_P347283	A_44_P347283		0.69	4.90	2.59E-05
A_44_P135183	Sell	NM_019177	29259	0.69	4.90	0.011339
A_44_P254540	AW915585	AW915585	315121	0.69	4.90	2.67E-06
A_44_P424481	Rcc2_predicted	XM_216557	298594	0.69	4.88	4.57E-06
A_44_P145562	Dock2	XM_001068649	360509	0.69	4.88	0.000331
A_44_P271485	Slu7	NM_173836	303057	0.69	4.87	5.07E-05
A_44_P196079	Col5a1	NM_134452	85490	0.69	4.87	0.000284
A_44_P198759	LOC685808	XM_001062634	64025	0.69	4.87	0.000436
A_44_P267610	RGD1564451_predicted	XM_233982		0.69	4.86	0.001162
A_43_P21714	LOC684555	XM_001070959		0.69	4.86	0.001623
A_44_P1004323	Col18a1	XM_241632	85251	0.69	4.85	0.000108

A_42_P664472	Rasa3	XM_225020		0.69	4.85	2.69E-05
A_44_P415021	LOC302759	XR_008022	302759	0.68	4.84	0.000679
A_44_P520159	P2ry2	NM_017255	29597	0.68	4.84	0.000039
A_44_P115349	BF521863	BF521863		0.68	4.81	0.000468
A_44_P190838	RGD1564291_predicted	XM_001070073		0.68	4.79	0.000945
A_44_P380274	Rrm2b_predicted	XM_235367	299976	0.68	4.79	0.007464
A_44_P489468	Myh11	XM_573030	24582	0.68	4.78	9.44E-05
A_44_P299213	Ifi27l	NM_130743		0.68	4.78	0.00292
A_42_P707230	Rcn2	NM_017132	29218	0.68	4.78	3.79E-06
A_42_P570919	Akr1a1	NM_031000	78959	0.68	4.77	0.000115
A_44_P247900	Kcna3	NM_019270	29731	0.68	4.76	0.000475
A_44_P656312	TC542702	TC542702		0.68	4.76	9.55E-05
A_44_P116870	RGD1308384_predicted	NM_001009691	310698	0.68	4.76	4.87E-05
A_44_P630174	AI234967	AI234967		0.68	4.75	0.000129
A_43_P12252	Entpd1	NM_022587	64519	0.68	4.75	0.000077
A_44_P128916	Centb1_predicted	XM_213365		0.68	4.74	5.57E-05
A_44_P552514	Ccr7	NM_199489	287673	0.68	4.74	0.000424
A_44_P496255	Slco3a1	NM_177481	140915	0.67	4.73	0.000173
A_44_P497212	Cdc42ep1_predicted	XM_001076184		0.67	4.72	3.35E-06
A_42_P619403	XM_341720	XM_341720		0.67	4.72	0.000526
A_44_P382683	Cfl2_predicted	XM_345674		0.67	4.72	0.000465
A_44_P253876	CB606211	CB606211		0.67	4.72	0.000438
A_44_P902442	A_44_P902442	A_44_P902442		0.67	4.71	0.000204
A_44_P506659	Celsr1	XM_001070474	300128	0.67	4.69	0.000129
A_42_P579376	Nbl1	NM_031609	50594	0.67	4.69	0.002425
A_44_P149138	Crem	NM_013086		0.67	4.68	5.79E-05
A_42_P762829	Cebpd	NM_013154	25695	0.67	4.68	1.93E-05
A_44_P353729	Axl	NM_001013147	308444	0.67	4.68	0.000101
A_44_P256110	Prkcm	XM_234108	85421	0.67	4.68	0.000133
A_43_P11513	Tnf	NM_012675	24835	0.67	4.67	0.000191
A_42_P736812	Prkcdbp	NM_134449	85332	0.67	4.65	0.000263
A_44_P455038	Man1c1_predicted	XM_342943		0.67	4.64	0.000133
A_44_P744113	Rab6ip1_predicted	XM_219270		0.67	4.64	5.97E-06
A_43_P10133	TC518177	TC518177		0.67	4.64	0.000338
A_44_P504192	Chst2_predicted	XM_345970	367145	0.67	4.63	0.00153
A_44_P480267	Asns	NM_013079	25612	0.67	4.63	5.59E-05
A_43_P19341	XM_342827	XM_342827		0.67	4.63	3.3E-06
A_43_P14503	TC521549	TC521549		0.66	4.61	0.000221
A_44_P361276	LOC680899	XM_001059384	680899	0.66	4.61	0.001194
A_44_P858611	CO556754	CO556754		0.66	4.61	0.000187
A_44_P609721	TC543345	TC543345		0.66	4.60	0.003517
A_44_P637792	Cbfb	NM_001013191	361391	0.66	4.60	0.003796
A_44_P555760	LOC257646	XM_001080473		0.66	4.59	0.000328
A_44_P245907	Efna4_predicted	XM_227416		0.66	4.59	0.000104
A_44_P262297	Myh10	NM_031520	79433	0.66	4.59	0.001369
A_44_P377107	Celsr1	XM_001070474	300128	0.66	4.58	3.61E-05
A_44_P548126	Socs3	NM_053565	89829	0.66	4.58	0.000688
A_44_P284220	LOC689710	XM_001071742	689710	0.66	4.58	2.83E-05
A_44_P391902	XM_345933	XM_345933		0.66	4.57	2.34E-05
A_44_P315397	Ccbe1_predicted	XM_341624	361341	0.66	4.56	0.010186
A_42_P633958	Col4a2_predicted	XM_225043	306628	0.66	4.56	0.000799
A_44_P506043	Gimap4	NM_173153	286938	0.66	4.56	0.000105
A_44_P334991	Fzd6	XM_343230	282581	0.66	4.56	0.001066
A_44_P198716	Pls3	XM_343776	81748	0.66	4.56	1.58E-05
A_44_P1053321	Gbp2	NM_133624	171164	0.66	4.55	1.05E-05
A_44_P271872	XM_218529	XM_218529		0.66	4.55	3.34E-05
A_42_P801679	Ctse	NM_012938	25424	0.66	4.55	0.000212
A_44_P372823	XM_342309	XM_342309		0.66	4.55	0.000223
A_44_P472170	Pole3	NM_001007652	298098	0.66	4.55	0.00043
A_44_P320617	Tpm1	NM_019131	24851	0.66	4.54	0.00042
A_44_P793461	TC560122	TC560122		0.66	4.54	0.011857
A_44_P135339	Hspca	NM_175761	299331	0.66	4.54	6.63E-05
A_44_P534601	Tek	XM_342863		0.66	4.54	0.000488

A_44_P473153	Col12a1	XM_243912	25683	0.66	4.54	5.24E-05
A_43_P22080	Lcp1	NM_001012044	306071	0.66	4.54	0.002458
A_44_P890330	RGD1562622_predicted	XM_578556		0.66	4.53	0.000217
A_43_P21856	Dok1	NM_001025416	312477	0.66	4.53	1.29E-05
A_44_P271941	Zcwcc1_predicted	XM_214072		0.66	4.52	0.004894
A_44_P166581	Ifitm7_predicted	XM_221637		0.65	4.52	9.72E-06
A_44_P157408	Sepw1	NM_013027	25545	0.65	4.51	0.000029
A_44_P762133	RGD1560587_predicted	XM_244186	316539	0.65	4.51	0.005532
A_44_P1011386	RGD1308723_predicted	XM_217694		0.65	4.51	0.000383
A_44_P869351	RGD1308877_predicted	XM_001081240		0.65	4.51	0.002576
A_44_P481501	CF110073	CF110073		0.65	4.51	5.28E-05
A_44_P353718	M15402	M15402		0.65	4.50	0.001925
A_44_P609788	Ank2	XM_001076082		0.65	4.49	0.000278
A_44_P646486	RGD1562552_predicted	XM_001062602		0.65	4.49	7.82E-05
A_43_P11152	Tm4sf1_predicted	XM_215576		0.65	4.48	6.68E-05
A_44_P190155	RGD1311154_predicted	XM_217075	300317	0.65	4.48	0.002602
A_44_P170823	Rab6ip1_predicted	XM_219270		0.65	4.48	2.52E-05
A_44_P320752	Rasl11a	NM_001002829	304268	0.65	4.47	0.000138
A_42_P559337	AW918097	AW918097		0.65	4.47	0.000997
A_44_P884663	LOC684352	XM_001070530		0.65	4.47	6.26E-05
A_44_P170919	LOC679975	XM_001054002		0.65	4.47	0.000467
A_44_P370122	Fads2	NM_031344	83512	0.65	4.46	0.000777
A_44_P403335	XM_233599	XM_233599		0.65	4.46	0.001719
A_44_P210547	Klrb1b	NM_173292	25192	0.65	4.46	0.000634
A_44_P382226	Sox4_predicted	XM_344594	364712	0.65	4.46	7.68E-06
A_44_P350098	Alg9_predicted	XM_345938		0.65	4.45	0.005037
A_44_P121658	Cdh5_predicted	XM_226213		0.65	4.45	0.00064
A_44_P473711	Dock2	XM_001068649	360509	0.65	4.45	6.73E-05
A_44_P1017415	LOC362703	XM_001059356	362703	0.65	4.44	0.001943
A_44_P239320	AI598903	AI598903	289521	0.65	4.44	0.001582
A_44_P152233	XM_346337	XM_346337		0.65	4.44	8.27E-06
A_43_P15902	Vwf	XM_342759	116669	0.65	4.44	0.00037
A_44_P130513	RT1-Db1	NM_001008884	294270	0.65	4.44	7.57E-05
A_42_P647109	Mmrn2_predicted	XM_001059491		0.65	4.44	2.34E-05
A_44_P170093	AA957719	AA957719	304291	0.65	4.43	0.000475
A_42_P656468	Rps8	NM_031706	65136	0.65	4.43	3.15E-05
A_43_P17926	Fli1	NM_001017381	315532	0.65	4.43	0.000304
A_42_P683634	Sparcl1	NM_012946	25434	0.65	4.43	0.000933
A_44_P114308	Hspca	NM_175761	299331	0.65	4.43	8.46E-05
A_44_P194152	Scd2	NM_031841	83792	0.65	4.42	0.000243
A_44_P178460	Itk_predicted	XM_343880		0.65	4.42	0.000384
A_44_P976470	TC558439	TC558439		0.65	4.42	0.000401
A_44_P884555	BE113337	BE113337		0.65	4.42	0.001194
A_42_P662897	Tgfb1i1	XM_341934	84574	0.64	4.41	0.000391
A_44_P282054	Mmp19_predicted	XM_222317		0.64	4.41	0.010001
A_44_P386792	RGD1560022_predicted	XM_222971	305012	0.64	4.41	0.03499
A_44_P931341	TC558560	TC558560		0.64	4.39	0.000259
A_44_P323833	RGD1305094_predicted	XM_222452		0.64	4.39	9.44E-05
A_44_P144649	Serpinb8_predicted	XM_222490		0.64	4.39	0.001074
A_44_P311515	Col9a1	XM_223124	305104	0.64	4.38	0.000431
A_43_P20611	LOC691153	XM_001077023		0.64	4.38	0.000505
A_42_P762150	RGD1562979_predicted	XM_223600		0.64	4.38	0.00082
A_44_P198396	Scd2	NM_031841	83792	0.64	4.38	0.000634
A_43_P15775	Ddx21a	NM_001037201	317399	0.64	4.38	5.62E-05
A_44_P389132	Rgs18	XM_222692		0.64	4.37	0.001008
A_44_P1036291	Sfrp2	XM_227314	310552	0.64	4.37	0.000976
A_44_P387523	Rps10	NM_031109	81773	0.64	4.37	3.02E-05
A_43_P10690	LOC684013	XM_001068514		0.64	4.37	0.000854
A_44_P105412	RGD1307980_predicted	XM_226578	292087	0.64	4.36	0.00072
A_44_P1042473	Lgals3bp	NM_139096	245955	0.64	4.36	4.78E-05
A_43_P23277	RGD1306783	NM_001025668	304928	0.64	4.36	0.000926
A_43_P22631	TC564725	TC564725		0.64	4.36	0.001325
A_44_P527379	XM_344242	XM_344242		0.64	4.36	0.00011

A_44_P472791	Nup107	NM_053830	116555	0.64	4.35	0.004053
A_44_P146200	Rdx	NM_001005889	315655	0.64	4.35	0.001971
A_44_P621522	ENSRNOT00000047221	ENSRNOT00000047221		0.64	4.35	0.000556
A_44_P959647	A_44_P959647	A_44_P959647		0.64	4.35	3.23E-05
A_44_P391949	RGD1311710	NM_001014099	316275	0.64	4.34	0.001429
A_44_P1032296	Capg	NM_001013086	297339	0.64	4.34	1.62E-06
A_44_P182671	LOC679692	XM_001053537		0.64	4.33	1.14E-05
A_44_P342075	Ahnak	XM_001078032		0.64	4.32	0.000293
A_44_P302697	Cdgap_predicted	XM_221438		0.64	4.32	0.000401
A_44_P263635	Prps1	NM_017243	29562	0.64	4.32	0.003722
A_44_P913600	A_44_P913600	A_44_P913600		0.64	4.32	4.75E-05
A_44_P834393	LOC687789	XM_001080152		0.63	4.31	0.00073
A_44_P515888	Col6a2	XM_342115	361821	0.63	4.31	0.000063
A_44_P542390	Ptprd	NM_019140	25529	0.63	4.30	6.54E-05
A_44_P130036	CB606441	CB606441		0.63	4.30	0.005307
A_44_P543258	Slc7a1	NM_013111	25648	0.63	4.29	8.43E-05
A_44_P847838	BG663025	BG663025		0.63	4.29	9.16E-05
A_44_P337088	Eif2b3	NM_133609	171145	0.63	4.29	0.000126
A_44_P792446	Scube1	XM_235529	315174	0.63	4.29	0.000111
A_44_P183784	Akap12	NM_057103	83425	0.63	4.29	0.000191
A_43_P22134	RGD1565091_predicted	XM_221671	288275	0.63	4.28	0.000506
A_44_P426788	Tmepai_predicted	XM_230899		0.63	4.28	2.84E-05
A_44_P206215	RGD1562313_predicted	XM_575186		0.63	4.28	0.000421
A_44_P318318	Mmp3	NM_133523	171045	0.63	4.27	0.006355
A_44_P405066	Scn1b	NM_017288	29686	0.63	4.27	0.01194
A_44_P426107	Akr1b8	NM_173136	286921	0.63	4.27	0.000179
A_44_P539359	Mmp28_predicted	XM_220785		0.63	4.26	0.000543
A_44_P322497	Serpinh1	NM_017173	29345	0.63	4.26	0.002583
A_44_P1011898	Gclm	NM_017305	29739	0.63	4.26	0.009058
A_44_P604227	DN935199	DN935199		0.63	4.26	0.013065
A_44_P868087	ENSRNOT00000033209	ENSRNOT00000033209		0.63	4.25	0.000348
A_44_P115116	Mpeg1	NM_022617	64552	0.63	4.25	0.000614
A_44_P455213	Wnt3_mapped	XM_221016		0.63	4.25	0.002971
A_44_P477819	Nrg1	NM_031588	112400	0.63	4.25	0.000156
A_44_P543643	Schip1	XM_215570	295105	0.63	4.24	0.000973
A_44_P281284	RGD1310185_predicted	XM_233749	313790	0.63	4.24	0.001648
A_44_P299870	Hspca	NM_175761	299331	0.63	4.24	1.48E-05
A_44_P637217	CF109032	CF109032	311575	0.63	4.23	0.001564
A_44_P393551	Gja1	NM_012567	24392	0.63	4.23	0.002119
A_43_P16820	Sox4_predicted	XM_344594	364712	0.63	4.23	4.8E-06
A_44_P130516	RT1-Db1	NM_001008884	294270	0.63	4.22	0.000187
A_44_P267799	Ets2_mapped	XM_239510		0.63	4.22	0.000109
A_44_P535400	AW916612	AW916612		0.63	4.22	0.002782
A_44_P822749	TC521972	TC521972		0.62	4.22	0.00087
A_44_P556707	Abi2	NM_173143	286928	0.62	4.21	0.000444
A_42_P744999	Rab12	XM_001053530		0.62	4.21	4.11E-05
A_44_P281035	Uchl1	NM_017237	29545	0.62	4.21	0.000328
A_44_P731768	TC559609	TC559609		0.62	4.21	0.000379
A_44_P204704	Cpe	NM_013128	25669	0.62	4.21	0.000199
A_44_P993577	RGD1560538_predicted	XM_214633		0.62	4.20	0.006196
A_42_P581567	Gart	XM_573258	288259	0.62	4.20	0.001416
A_44_P401131	Noxo1_predicted	XM_220221		0.62	4.20	1.46E-05
A_44_P549867	AW144312	AW144312	685076	0.62	4.19	0.001434
A_44_P410469	XM_236901	XM_236901		0.62	4.19	0.030341
A_44_P600368	CF110082	CF110082		0.62	4.19	0.023697
A_44_P297818	XM_234745	XM_234745		0.62	4.19	0.005823
A_44_P412098	Thop1	NM_172075	64517	0.62	4.18	3.59E-05
A_44_P513465	Ddx31_predicted	XM_242296		0.62	4.18	0.000459
A_44_P288231	Ncam1	NM_031521	24586	0.62	4.18	0.001556
A_44_P1011253	Nars	NM_001025635	291556	0.62	4.18	0.000335
A_44_P349460	RGD1560514_predicted	XM_574041	498757	0.62	4.17	0.004605
A_42_P478138	AW913982	AW913982		0.62	4.17	0.000343
A_43_P20050	Prnd	XM_230542		0.62	4.16	0.002367

A_44_P480208	Rps3	NM_001009239	140654	0.62	4.16	3.06E-05
A_44_P378799	Igfbp6	NM_013104	25641	0.62	4.16	0.000759
A_44_P311364	Clca2	NM_001013202	362052	0.62	4.15	0.021666
A_44_P253196	Arid5b_predicted	XM_228114		0.62	4.15	7.32E-05
A_43_P18268	LOC687188	XM_001077450		0.62	4.15	0.00065
A_43_P21655	LOC294762	XM_215491	294762	0.62	4.15	0.001234
A_44_P930585	TC523012	TC523012		0.62	4.15	0.004666
A_44_P325479	Ccnb1	NM_171991	25203	0.62	4.14	0.010527
A_44_P560973	Lamc2	XM_213902	192362	0.62	4.14	0.000238
A_44_P504094	AY539911	AY539911		0.62	4.14	0.000237
A_43_P17442	Thbs1	NM_001013062	445442	0.62	4.14	8.23E-06
A_44_P283546	Rps12	NM_031709	65139	0.62	4.14	1.36E-05
A_44_P185243	LOC689788	XM_001072014	689788	0.62	4.14	6.35E-05
A_43_P19735	Cdh5_predicted	XM_226213		0.62	4.14	0.004198
A_44_P574914	AI501053	AI501053		0.62	4.14	1.69E-05
A_44_P699869	CO558803	CO558803		0.62	4.14	0.000385
A_44_P185174	RGD1561134_predicted	XM_225841		0.62	4.14	0.000233
A_44_P838429	TC557499	TC557499		0.62	4.14	0.000254
A_44_P840348	TC565815	TC565815		0.62	4.13	0.000142
A_44_P761639	TC553163	TC553163		0.62	4.13	0.000172
A_44_P868495	TC554267	TC554267		0.61	4.11	0.001641
A_43_P20216	CB545562	CB545562	304860	0.61	4.11	0.006227
A_43_P19712	Gpr65_predicted	XM_234367		0.61	4.11	0.000791
A_44_P870101	TC541449	TC541449		0.61	4.11	0.002675
A_44_P638146	TC522745	TC522745		0.61	4.11	1.26E-05
A_44_P808341	Zfp580_predicted	XM_001072400		0.61	4.11	0.000819
A_43_P14890	Ptpns1	NM_013016	25528	0.61	4.10	0.000339
A_44_P281482	Chchd6_predicted	XM_216212		0.61	4.10	0.000118
A_44_P680445	AI229310	AI229310		0.61	4.10	0.000717
A_44_P444339	Larp1_predicted	XM_220446	303158	0.61	4.10	0.000187
A_44_P183048	Ctps_predicted	XM_233467	313560	0.61	4.10	2.62E-05
A_44_P280933	Vars2	XM_001076616		0.61	4.09	5.59E-05
A_44_P427006	Emp2	NM_001007721	360468	0.61	4.09	0.000115
A_44_P170764	Dhx37_predicted	XM_213772		0.61	4.09	0.000396
A_42_P687443	Ramp3	NM_020100	56820	0.61	4.08	0.000484
A_43_P15246	Pthlh	NM_012636	24695	0.61	4.08	0.008651
A_44_P651631	AY623037	AY623037		0.61	4.08	0.000266
A_44_P768625	Ifit1_predicted	XM_001079971		0.61	4.08	0.006833
A_44_P451627	LOC301509	XM_237286	301509	0.61	4.07	0.007416
A_44_P293423	Hoxb13_predicted	XM_220905		0.61	4.07	0.085246
A_44_P470824	Trip13	NM_001011930	292206	0.61	4.07	0.001121
A_44_P537480	Cox6b2	NM_001039085	654441	0.61	4.07	0.001149
A_44_P510690	Psip1	BC093606	313323	0.61	4.06	0.021731
A_42_P805300	Gpx1	NM_030826	24404	0.61	4.06	6.13E-05
A_44_P127958	Myo9a	NM_134335	171296	0.61	4.05	0.001716
A_44_P125145	CF109884	CF109884		0.61	4.05	0.009001
A_44_P139882	RT1-Db1	NM_001008884	294270	0.61	4.04	0.000115
A_44_P283839	ENSRNOT00000045079	ENSRNOT00000045079		0.61	4.04	7.75E-05
A_44_P272210	Ccne2_predicted	XM_342804		0.61	4.03	0.001412
A_42_P783301	Itga5_mapped	XM_235707		0.61	4.03	1.04E-05
A_44_P365358	Tusc3	NM_001004212	290783	0.61	4.03	0.0001
A_44_P534349	XM_224738	XM_224738		0.61	4.03	0.00342
A_44_P362981	Fabp5	NM_145878	140868	0.61	4.03	0.000218
A_44_P1057102	Itgb1bp1_predicted	XM_216677		0.61	4.03	0.005058
A_43_P10901	Itm2c	NM_001009674	301575	0.60	4.02	3.74E-05
A_43_P12806	Ran	NM_053439	84509	0.60	4.02	2.85E-05
A_44_P380038	RGD1308267_predicted	XM_341654	361375	0.60	4.02	0.000938
A_44_P503669	RGD1559988_predicted	XM_222107		0.60	4.01	0.001639
A_44_P471088	Sp5_predicted	XM_231003		0.60	4.01	0.000861
A_44_P510826	XM_218092	XM_218092		0.60	4.01	0.006546
A_43_P16629	RGD1565002_predicted	XM_216735	299135	0.60	4.00	0.002899
A_44_P299123	Nphp1_predicted	XM_215847		0.60	4.00	0.001315
A_44_P1008073	LOC294446	XM_001061084		0.60	3.99	0.000105

A_44_P338137	Cct6a	NM_001033684	288620	0.60	3.99	6.08E-05
A_44_P1004324	Col18a1	XM_241632	85251	0.60	3.99	0.000179
A_44_P433519	Rhbdl7_predicted	XM_341058	360793	0.60	3.99	1.85E-05
A_44_P491909	Eif4a1	NM_199372	287436	0.60	3.99	3.71E-05
A_44_P463759	Prickle1	XM_235609	315259	0.60	3.98	0.001188
A_44_P147560	Cfl2_predicted	XM_345674		0.60	3.98	9.91E-06
A_44_P518523	Ankrd39_predicted	XM_346038	367251	0.60	3.98	0.000361
A_44_P107535	Ltbp4	XM_238093	292734	0.60	3.98	0.00069
A_44_P501952	XM_340977	XM_340977		0.60	3.98	7.06E-06
A_44_P509500	Akap2	NM_001011974	298024	0.60	3.97	0.00354
A_44_P421844	Loxl4_predicted	XM_239059		0.60	3.97	0.002907
A_44_P753593	AW917592	AW917592		0.60	3.97	0.00532
A_44_P334709	Rps14	NM_022672	29284	0.60	3.96	0.000166
A_44_P264441	Rps2	NM_031838	83789	0.60	3.96	1.56E-05
A_43_P16390	Lamc2	XM_001071267		0.60	3.96	0.000249
A_44_P435955	RGD1561143_predicted	XM_222031	304382	0.60	3.96	0.001055
A_44_P227559	ENSRNOT00000017404	ENSRNOT00000017404		0.60	3.96	0.000302
A_42_P768978	Nrp1	NM_145098	246331	0.60	3.96	0.000989
A_44_P165902	Prkcn	XM_001054495	313834	0.60	3.95	0.001992
A_44_P243367	Chsy1_predicted	XM_218759		0.60	3.95	0.000107
A_44_P124027	RGD1309394_predicted	XM_226458		0.60	3.94	0.000951
A_44_P499142	Rad1_predicted	XM_215497		0.60	3.94	0.008328
A_44_P748601	Dd25	XM_001074497		0.59	3.92	0.000661
A_44_P375004	Acsl4	NM_053623	113976	0.59	3.92	8.73E-05
A_44_P1057427	Zfhx1b	NM_001033701	311071	0.59	3.91	0.000446
A_44_P139744	Id3	NM_013058	25585	0.59	3.91	0.000082
A_44_P388897	Atp2b4	NM_001005871	29600	0.59	3.90	0.000187
A_44_P469531	LOC24906	NM_031537	24906	0.59	3.90	0.000663
A_44_P262371	Axin2	NM_024355	29134	0.59	3.89	0.000521
A_44_P216903	BE113113	BE113113		0.59	3.89	0.016888
A_44_P666038	CF110295	CF110295		0.59	3.89	0.008888
A_42_P762508	Nptx2	NM_001034199	288475	0.59	3.89	0.000321
A_44_P104821	Fstl1	NM_024369	79210	0.59	3.88	9.64E-05
A_42_P817417	PVR	NM_017076	25066	0.59	3.88	4.61E-06
A_44_P974862	LOC362792	XM_576106	362792	0.59	3.87	0.000405
A_44_P325744	Nap1l1	NM_053561	89825	0.59	3.87	0.000102
A_42_P506685	Fxyd2	NM_145717	29639	0.59	3.86	7.25E-05
A_44_P589989	CA339579	CA339579		0.59	3.86	0.004089
A_44_P173706	Gja7	XM_001081521		0.59	3.86	0.000173
A_44_P700241	RGD1564808_predicted	XM_001068586		0.59	3.86	0.0018
A_44_P776915	TC522458	TC522458		0.59	3.86	0.008335
A_44_P964256	TC564799	TC564799		0.59	3.86	0.000153
A_44_P226263	LOC687595	XM_001078994		0.59	3.86	3.23E-05
A_44_P335656	RGD1565311_predicted	XM_343188	362858	0.59	3.85	0.000539
A_44_P157314	BF558825	BF558825		0.59	3.85	0.083098
A_44_P929894	TC537137	TC537137		0.59	3.85	2.96E-05
A_44_P556528	Emilin1_predicted	XM_238447		0.59	3.85	0.00053
A_44_P450440	Kirrel3_predicted	XM_235986		0.59	3.85	0.002064
A_44_P1038885	Slc16a13	NM_001005530	287451	0.59	3.85	0.000122
A_44_P627982	A_44_P627982	A_44_P627982		0.58	3.85	0.003714
A_44_P168856	RGD1565078_predicted	XM_236237		0.58	3.84	0.000109
A_42_P465664	RGD1308876_predicted	XM_342915		0.58	3.84	7.77E-06
A_44_P303883	AI602844	AI602844	303398	0.58	3.84	0.000539
A_44_P868459	Map4k2_predicted	XM_001071701		0.58	3.84	0.001687
A_44_P263105	Limd2	NM_001025715	360646	0.58	3.84	0.000108
A_42_P463880	RGD1308734	NM_001013927	297694	0.58	3.84	0.000068
A_44_P429455	Lama5	XM_215963	140433	0.58	3.84	0.000113
A_44_P253048	RGD1308384_predicted	NM_001009691	310698	0.58	3.84	4.34E-05
A_44_P458851	Col1a2	NM_053356	84352	0.58	3.84	0.000198
A_42_P552949	Rpl10	NM_031100	81764	0.58	3.83	0.000017
A_44_P380132	P2ry10_predicted	XM_228500	317219	0.58	3.83	0.00326
A_44_P306307	Hbb	NM_033234	24440	0.58	3.83	0.00369
A_44_P351921	RGD1564027_predicted	XM_235733	300289	0.58	3.83	2.19E-05

A_44_P506377	Rps2	NM_031838	83789	0.58	3.83	1.86E-05
A_44_P1029956	Cnn3	NM_019359	54321	0.58	3.83	2.84E-05
A_44_P441434	Cav2	NM_131914	363425	0.58	3.82	0.016326
A_44_P252756	Flt3	XM_221874	140635	0.58	3.82	0.000319
A_44_P157222	Tgif	NM_001015020	316742	0.58	3.82	9.82E-06
A_44_P442802	Rassf5	NM_019365	54355	0.58	3.82	0.000296
A_43_P11899	Uhmk1	NM_017293	246332	0.58	3.82	0.001706
A_44_P375900	LOC367311	XM_001065966		0.58	3.81	0.000224
A_44_P184987	RGD1306622_predicted	XM_215195	293736	0.58	3.81	0.003396
A_44_P510951	Ms4a7_predicted	XM_215200		0.58	3.81	0.000187
A_44_P148962	Hps3_predicted	XM_001055848		0.58	3.81	0.000125
A_43_P22969	XM_225491	XM_225491		0.58	3.81	0.013712
A_44_P379214	Arrb2	NM_012911	25388	0.58	3.80	7.13E-06
A_44_P213353	Wdr35_predicted	XM_233960	298876	0.58	3.80	0.001384
A_44_P463378	Rpl27	NM_022514	64306	0.58	3.80	0.000106
A_43_P10312	Dpy19l1_predicted	XM_235970	315496	0.58	3.79	4.62E-05
A_42_P524610	Pphln1_predicted	XM_345865		0.58	3.79	0.009536
A_44_P184659	RGD1306939	XM_225319		0.58	3.79	0.000171
A_44_P506447	Fgd5_predicted	XM_342725		0.58	3.79	0.001365
A_44_P745617	TC558054	TC558054		0.58	3.79	0.000399
A_44_P667315	RGD1565131_predicted	XM_573353	498143	0.58	3.78	1.95E-05
A_44_P348418	AW917057	AW917057	290963	0.58	3.78	0.000237
A_42_P620975	Apeg1	U57097	25381	0.58	3.78	0.000505
A_44_P1039128	Cxcl10	NM_139089	245920	0.58	3.78	0.000917
A_44_P822396	Fbxl11_predicted	XM_341983		0.58	3.78	0.000799
A_44_P461165	Samsn1	NM_130821	170637	0.58	3.78	0.002228
A_43_P12208	Basp1	NM_022300	64160	0.58	3.78	1.32E-05
A_44_P704874	AA963844	AA963844		0.58	3.78	0.00067
A_42_P641962	Pcsk6	NM_012999	25507	0.58	3.77	0.000119
A_42_P601014	LOC501065	XM_576480		0.58	3.77	6.84E-06
A_42_P837865	Nedd1_predicted	XM_216878		0.58	3.77	0.007074
A_44_P305004	Tcfcp2_predicted	XM_001063604		0.58	3.76	0.003594
A_44_P517392	AI407429	AI407429		0.58	3.76	0.000735
A_44_P458855	Ptprn	NM_053881	116660	0.58	3.76	0.00808
A_44_P869850	TC559977	TC559977		0.58	3.76	0.000779
A_44_P791729	TC520825	TC520825		0.57	3.76	0.011401
A_44_P1028314	Rbm28_predicted	XM_231550		0.57	3.75	0.000844
A_44_P330869	RGD1305899_predicted	XM_001058977		0.57	3.75	0.000341
A_44_P777088	Trip13	XM_574293		0.57	3.75	0.00376
A_44_P837580	BG668811	BG668811	65028	0.57	3.75	0.000137
A_44_P271625	Pgk1	NM_053291	24644	0.57	3.74	0.000156
A_44_P409605	Habp2	NM_001001505	292126	0.57	3.74	0.001746
A_44_P545792	Smad9	NM_138872	85435	0.57	3.74	0.000271
A_44_P806758	A_44_P806758	A_44_P806758		0.57	3.74	0.002862
A_42_P641107	Rpa2	NM_021582	59102	0.57	3.73	5.49E-05
A_43_P16199	Fgfr2	XM_341940		0.57	3.73	0.002404
A_44_P473622	RGD1565985_predicted	XM_576070	500689	0.57	3.73	5.23E-05
A_44_P132291	Ipo9_predicted	XM_222661		0.57	3.73	0.000037
A_44_P439122	Egr3	NM_017086	25148	0.57	3.72	0.000991
A_44_P549055	AI044121	AI044121		0.57	3.72	0.000063
A_44_P1022002	Ccl7	NM_001007612	287561	0.57	3.72	0.000321
A_44_P965184	MGC94183	NM_001007660	300359	0.57	3.72	0.019624
A_44_P450786	AW917984	AW917984		0.57	3.72	0.000433
A_44_P183694	AB072614	AB072614		0.57	3.72	3.88E-05
A_44_P1003090	LOC289809	NM_199083	289809	0.57	3.71	0.010961
A_44_P479881	Dusp4	NM_022199	60587	0.57	3.71	0.000221
A_44_P714784	BF289878	BF289878		0.57	3.71	0.002035
A_44_P262192	Cct4	NM_182814	29374	0.57	3.71	0.00077
A_44_P497011	Tcrb	BC091428	24820	0.57	3.71	0.000205
A_44_P222030	Tbx3	NM_181638	353305	0.57	3.71	0.000126
A_44_P338864	Nap1l1	NM_053561	89825	0.57	3.70	2.61E-05
A_44_P191722	RGD1305133	XM_214173		0.57	3.70	0.000733
A_44_P212985	Ltbp3	XM_341997		0.57	3.70	0.000726

A_42_P683837	Serpinb6b	NM_001012214	364705	0.57	3.70	0.000321
A_44_P1042504	LOC295496	XM_001076299		0.57	3.70	0.00198
A_44_P378845	CF109250	CF109250	313874	0.57	3.70	0.003911
A_42_P624195	Gsta3	NM_031509	24421	0.57	3.70	0.073137
A_44_P224972	RGD1306582	NM_001025019	362103	0.57	3.69	0.000221
A_44_P171629	RGD1565408_predicted	XM_341174		0.57	3.69	0.001189
A_42_P750683	Cyr61	NM_031327	83476	0.57	3.69	5.85E-05
A_44_P288481	Heatr1_predicted	XM_341546		0.57	3.69	4.81E-05
A_44_P1057055	Ccl12_predicted	XM_213425		0.57	3.69	0.000458
A_44_P814711	TC559727	TC559727		0.57	3.69	0.005042
A_44_P472483	Rgs19	NM_021661	59293	0.57	3.68	0.000011
A_44_P134213	Tpm1	NM_001034069	24851	0.57	3.68	4.06E-05
A_43_P14162	A2m	NM_012488	24153	0.57	3.68	0.000166
A_43_P19907	Loxl1	NM_001012125	315714	0.57	3.68	6.24E-05
A_44_P403877	AABR03024169	AABR03024169		0.57	3.68	4.16E-05
A_44_P478570	Gcn5l2_predicted	XM_239340		0.57	3.68	0.000088
A_42_P513025	Znf291	XM_343394	117521	0.57	3.67	0.006072
A_42_P601971	Sox30_predicted	XM_220328	57031	0.56	3.67	0.000291
A_44_P348748	CF110996	CF110996		0.56	3.67	0.000661
A_44_P556921	AA997829	AA997829	24242	0.56	3.67	0.000911
A_44_P524359	Anxa1	NM_012904	25380	0.56	3.66	0.00312
A_42_P791677	Areg	NM_017123	29183	0.56	3.66	9.58E-06
A_44_P270854	AW142500	AW142500	305227	0.56	3.66	2.47E-05
A_44_P210921	XM_345084	XM_345084		0.56	3.65	0.000258
A_44_P687814	TC565523	TC565523		0.56	3.65	0.000236
A_44_P891704	TC532126	TC532126		0.56	3.65	0.001366
A_44_P267013	U75411	U75411		0.56	3.65	0.006422
A_44_P1049055	Hspa4l_predicted	XM_215549		0.56	3.65	0.012588
A_44_P181464	Fundc1	NM_001025027	363442	0.56	3.64	0.015298
A_44_P278613	Ptpre	XM_341950	114767	0.56	3.64	0.001832
A_44_P690171	Fbln1_predicted	XM_243637	315191	0.56	3.64	0.003177
A_43_P13601	CB547725	CB547725		0.56	3.64	0.000199
A_44_P513059	Sfrp1	XM_224987	84402	0.56	3.64	0.001119
A_44_P258949	Pde4b	NM_017031	24626	0.56	3.64	0.00017
A_44_P248615	LOC685055	XM_001062084	685055	0.56	3.64	0.000276
A_44_P265307	XM_345749	XM_345749		0.56	3.63	0.052702
A_44_P435764	LOC301509	XM_237286	301509	0.56	3.63	0.000565
A_44_P241498	LOC681287	XM_001058588		0.56	3.63	7.5E-06
A_44_P593668	TC525504	TC525504		0.56	3.63	0.00063
A_44_P211679	BM299612	BM299612		0.56	3.63	8.04E-05
A_44_P344475	Rhoq	NM_053522	85428	0.56	3.62	0.000104
A_44_P289685	AA997980	AA997980		0.56	3.62	0.009236
A_44_P534791	Mettl2_predicted	XM_343972		0.56	3.62	0.004459
A_42_P625157	Zfp503_predicted	XM_223786		0.56	3.62	2.81E-05
A_44_P154436	Tnfaip8_predicted	XM_225940		0.56	3.62	0.002652
A_42_P786011	Ggta1	NM_145674	246766	0.56	3.62	0.0151
A_44_P637322	LOC684079	XM_001068844		0.56	3.62	1.62E-05
A_43_P11812	Bgn	NM_017087	25181	0.56	3.61	0.008521
A_43_P12172	Lgmn	NM_022226	63865	0.56	3.61	0.000363
A_43_P18793	Gfpt2	NM_001002819	360518	0.56	3.61	0.002865
A_42_P473314	Hla-dma	NM_198741	294274	0.56	3.61	0.00002
A_44_P288681	XM_342942	XM_342942		0.56	3.61	1.53E-05
A_42_P475681	Nol8_predicted	XM_341503		0.56	3.61	0.001204
A_42_P453055	Bcam	NM_031752	78958	0.56	3.61	7.28E-05
A_44_P187293	XM_344175	XM_344175		0.56	3.61	1.04E-05
A_44_P114184	Ssg1	XM_573284		0.56	3.61	0.014949
A_42_P799494	Htatip2_predicted	XM_001080298		0.56	3.60	4.07E-05
A_44_P452546	RGD1311822_predicted	XM_227686		0.56	3.60	0.008328
A_44_P229769	Rps2	NM_031838	83789	0.56	3.60	4.96E-06
A_44_P441166	Atm_mapped	XM_236275		0.56	3.60	0.00049
A_44_P342334	LOC683813	XM_001067603		0.56	3.59	7.36E-05
A_44_P1071095	Sox4_predicted	XM_344594	364712	0.56	3.59	0.000044
A_44_P170541	Ptpre	XM_341950	114767	0.55	3.58	0.003082

A_44_P489545	LOC498339	XM_573568		0.55	3.58	0.001317
A_42_P694171	Ppil3	NM_175707	301432	0.55	3.58	0.000104
A_44_P311078	RGD1565619_predicted	XM_225760	307302	0.55	3.57	0.00223
A_42_P470757	Rnf34	NM_001004075	282845	0.55	3.57	3.39E-05
A_44_P653272	TC522219	TC522219		0.55	3.57	0.000599
A_44_P744063	AW919683	AW919683		0.55	3.57	0.014959
A_44_P507571	Ccne1	XM_001077331		0.55	3.57	0.000101
A_44_P331706	Dnajb5_predicted	XM_233767		0.55	3.56	7.74E-05
A_44_P194167	RT1-Ba	X14879	309621	0.55	3.55	0.000626
A_44_P525307	LOC679107	XM_001055210		0.55	3.55	0.001728
A_44_P160877	Cfh	NM_130409	155012	0.55	3.55	0.001682
A_44_P201276	RGD1566307_predicted	XM_218261	308350	0.55	3.55	0.000427
A_43_P10742	CB547617	CB547617	25125	0.55	3.55	0.001053
A_44_P386359	Ninj1	NM_012867	25338	0.55	3.55	2.89E-05
A_44_P964460	TC530765	TC530765		0.55	3.54	0.000436
A_44_P497121	RGD1308967_predicted	XM_232748		0.55	3.54	0.002068
A_44_P109600	Ctsl	NM_013156	25697	0.55	3.54	0.006093
A_43_P10130	CB606181	CB606181	362973	0.55	3.53	0.001211
A_44_P530896	AA858639	AA858639	24267	0.55	3.53	0.001939
A_42_P814235	P2ry6	NM_057124	117264	0.55	3.52	8.81E-05
A_44_P300183	LOC300191	XM_217033	300191	0.55	3.52	3.87E-05
A_44_P140275	Alpk1_predicted	XM_227715	310879	0.55	3.52	0.000244
A_44_P447368	Gnb2l1	NM_130734	83427	0.55	3.52	9.95E-05
A_44_P530211	RGD1304694	XM_343304	362974	0.55	3.52	7.65E-06
A_44_P1014553	Rpl24	NM_022515	64307	0.55	3.52	1.15E-05
A_42_P634675	Centa2	NM_020101	56826	0.55	3.52	0.0001
A_44_P611921	TC563013	TC563013		0.55	3.51	0.006083
A_42_P463754	Itga1	NM_030994	25118	0.55	3.51	0.003525
A_44_P427560	Pom121	NM_053622	113975	0.55	3.51	0.004598
A_44_P457260	RGD1563945_predicted	XM_234528	299305	0.54	3.51	0.005288
A_42_P527724	Fcnb	NM_053634	114091	0.54	3.51	0.007799
A_44_P550145	Scd1	NM_139192	246074	0.54	3.50	0.003262
A_44_P210198	Polg	NM_053528	85472	0.54	3.50	1.55E-05
A_44_P409835	Plvap	NM_020086	56765	0.54	3.50	0.000145
A_44_P395885	Cdkn2a	NM_031550	25163	0.54	3.50	0.003022
A_44_P714868	TC521225	TC521225		0.54	3.50	7.07E-05
A_43_P16588	Sh2d3c_predicted	XM_342414		0.54	3.50	0.0012
A_44_P150594	Tp53	NM_030989	24842	0.54	3.50	1.87E-05
A_44_P683082	A_44_P683082	A_44_P683082		0.54	3.50	0.001097
A_44_P463822	Homer3	NM_053310	29548	0.54	3.50	2.68E-05
A_44_P290022	Lrg1	NM_001009717	367455	0.54	3.50	0.000697
A_44_P901138	TC528328	TC528328		0.54	3.49	0.004114
A_44_P590853	ENSRNOT00000051084	ENSRNOT00000051084		0.54	3.49	0.006792
A_44_P121977	LOC367289	XM_346058	367289	0.54	3.49	0.000995
A_44_P622195	Zfp260	NM_017364	53982	0.54	3.49	0.021599
A_42_P781919	C1qb	NM_019262	29687	0.54	3.49	0.000315
A_44_P205941	Wbscr16_predicted	XM_341066		0.54	3.48	0.000446
A_44_P364620	AB096137	AB096137		0.54	3.48	0.002274
A_44_P1013006	Steap1_predicted	XM_216315		0.54	3.48	0.003254
A_44_P371777	Vof16	NM_147207	259227	0.54	3.47	0.013774
A_44_P121167	LOC294446	XM_001061084		0.54	3.47	4.14E-05
A_44_P558179	A_44_P558179	A_44_P558179		0.54	3.47	0.011394
A_44_P518085	Adamdec1_predicted	XM_214222		0.54	3.47	0.000951
A_44_P1025861	Shprh_predicted	XM_001069964		0.54	3.47	0.00993
A_44_P167965	Gpr56	NM_152242	260326	0.54	3.46	3.78E-05
A_44_P497067	Susd1_predicted	XM_232941		0.54	3.46	0.016766
A_44_P145030	Ddx58_predicted	XM_216380		0.54	3.46	0.011383
A_44_P1050480	Ecm1	NM_053882	116662	0.54	3.46	0.000191
A_44_P462326	Bxdc2	NM_001029915	294799	0.54	3.46	0.000723
A_44_P267041	Lat	NM_030853	81511	0.54	3.46	0.001594
A_44_P426936	RGD1307704_predicted	XM_234396		0.54	3.46	0.000207
A_44_P635239	Nrep	AY724475	338475	0.54	3.46	6.1E-06
A_44_P414311	St6gal1	NM_147205	25197	0.54	3.46	0.000102

A_44_P173330	RGD1307787	NM_001024999	307742	0.54	3.46	5.66E-05
A_43_P14690	Pctk2	XM_001055332		0.54	3.46	4.39E-05
A_42_P615675	Ube2q2_predicted	XM_001072896		0.54	3.46	4.86E-05
A_44_P265702	Ppfibp1_predicted	XM_232536		0.54	3.45	0.00014
A_44_P370175	Rpo1-2	NM_031773	83582	0.54	3.45	0.000412
A_44_P458833	Zbtb25	NM_199496	314245	0.54	3.45	0.001588
A_44_P410016	Ifit2	NM_001024753	294091	0.54	3.45	0.002298
A_44_P831621	CD373310	CD373310		0.54	3.45	0.008776
A_44_P1037456	Col22a1_predicted	XM_001072793		0.54	3.44	0.002874
A_44_P206138	RGD1562119_predicted	XM_341433	361147	0.54	3.44	0.009492
A_44_P480329	RGD1305685	XM_341084		0.54	3.44	7.54E-05
A_44_P391296	Ccl7	NM_001007612	287561	0.54	3.44	0.000799
A_44_P474101	Slc7a1	NM_013111	25648	0.54	3.44	0.000017
A_44_P197786	AA944397	AA944397		0.54	3.44	0.060624
A_44_P1008094	Exosc7	XM_236745	316098	0.54	3.43	0.003542
A_44_P221965	Kremen1	NM_053649	114107	0.54	3.43	0.00117
A_43_P11472	Hmox1	NM_012580	24451	0.54	3.43	0.005502
A_44_P342051	Ecm1	NM_053882	116662	0.53	3.43	0.000623
A_44_P122141	Klhl22_predicted	XM_221268		0.53	3.42	0.0003
A_44_P979645	TC548300	TC548300		0.53	3.42	0.000821
A_44_P405603	XM_342816	XM_342816		0.53	3.42	0.002003
A_44_P417190	Rpp40	NM_001013055	291071	0.53	3.41	0.004765
A_44_P342671	XM_234728	XM_234728		0.53	3.41	0.002801
A_44_P508466	Jundp2	NM_053894	116674	0.53	3.40	0.000137
A_44_P837716	TC519685	TC519685		0.53	3.40	0.016639
A_44_P423281	Etv4_predicted	XM_001081496		0.53	3.40	0.030076
A_44_P222946	LOC360568	XM_340844	360568	0.53	3.40	0.000312
A_44_P480573	Pscdbp	NM_001012086	311047	0.53	3.40	0.003751
A_44_P1011373	RGD1565022_predicted	XM_216007	296580	0.53	3.40	0.003158
A_44_P959510	M15402	M15402		0.53	3.40	0.001715
A_44_P561191	TC520972	TC520972		0.53	3.40	0.001109
A_44_P172982	Zyx	XM_216124	114636	0.53	3.40	1.93E-05
A_44_P520402	LOC679990	XM_574644	679990	0.53	3.40	0.004122
A_43_P11114	TC543047	TC543047		0.53	3.39	7.24E-05
A_43_P20388	RGD1307658_predicted	XM_232819	313069	0.53	3.39	0.00131
A_42_P565385	Rps23	NM_078617	124323	0.53	3.39	8.23E-06
A_43_P23403	Dsc3_predicted	XM_226122		0.53	3.39	0.065573
A_42_P580618	Ip63	NM_021741	60347	0.53	3.38	0.011576
A_44_P271741	Dpysl2	XM_001067745		0.53	3.38	6.81E-05
A_44_P490107	A_44_P490107	A_44_P490107		0.53	3.38	6.01E-05
A_44_P452325	Ltv1	NM_001014157	361452	0.53	3.37	0.001257
A_44_P161790	Tmem103_predicted	XM_343489		0.53	3.37	0.000314
A_44_P552830	A3galt2	NM_138524	171553	0.53	3.37	0.000111
A_43_P11621	Cd44	NM_012924	25406	0.53	3.37	3.39E-05
A_44_P388783	Clec4d	NM_001003707	362432	0.53	3.37	0.096211
A_44_P342451	Rpp38	NM_001033063	291317	0.53	3.37	3.59E-05
A_44_P170939	Ppm1l_predicted	XM_227247		0.53	3.37	0.001139
A_44_P287194	Hspcb	NM_001004082	301252	0.53	3.36	4.62E-05
A_44_P299887	Olfml2a_predicted	XM_231192		0.53	3.36	0.003177
A_44_P867949	LOC681818	XR_007674	685500	0.53	3.36	2.84E-05
A_44_P387934	BG372678	BG372678	307343	0.53	3.36	2.89E-05
A_44_P655718	LOC681949	XM_001059070		0.53	3.36	0.000744
A_44_P591351	DY471341	DY471341		0.53	3.36	0.000342
A_43_P10584	TC519665	TC519665		0.53	3.36	0.025174
A_44_P277935	Arid1a_predicted	XM_216340		0.53	3.36	0.002068
A_44_P187167	Tubb2b	NM_001013886	291081	0.53	3.36	5.24E-05
A_42_P594999	LOC683676	XM_001064798		0.53	3.36	0.015311
A_44_P340180	Anapc10_predicted	XM_341668		0.53	3.35	0.000809
A_44_P212551	Ccl4	NM_053858	116637	0.53	3.35	0.033532
A_42_P490804	Nol5	NM_021754	60373	0.53	3.35	5.55E-05
A_44_P381842	Ptprm	XM_001053209		0.53	3.35	0.002811
A_43_P18149	RGD1305052_predicted	XM_221142	303698	0.53	3.35	0.000135
A_44_P355073	LOC684425	XM_001072835		0.53	3.35	0.000437

A_44_P124195	Nuak1	XM_234998		0.52	3.35	0.000619
A_44_P544974	Mki67ip	BC086585	246042	0.52	3.35	0.001633
A_44_P177845	Fbxo30	NM_001007690	308283	0.52	3.34	0.000154
A_44_P214479	Entpd1	NM_022587	64519	0.52	3.34	0.001428
A_44_P342273	Gprc5b_predicted	XM_215095		0.52	3.34	0.020418
A_44_P652890	MGC124653	NM_001033903	501157	0.52	3.34	0.007877
A_44_P317287	Hsf2	NM_031694	64441	0.52	3.34	0.002933
A_44_P148383	RGD1564952_predicted	XR_007587	304761	0.52	3.34	0.00227
A_44_P536125	Hrmt1l3	NM_053557	89820	0.52	3.34	0.000129
A_44_P262556	RGD1311584	NM_001025115	305269	0.52	3.34	4.34E-05
A_44_P526397	Paip1_predicted	XM_001068994		0.52	3.34	0.005371
A_44_P406636	CB557036	CB557036		0.52	3.34	0.000476
A_44_P133581	RGD1560736_predicted	XM_001064905		0.52	3.34	0.002673
A_44_P507194	Gpc1	NM_030828	58920	0.52	3.33	9.76E-05
A_44_P188606	Nnt_mapped	NM_001013157	310378	0.52	3.33	0.020307
A_44_P386515	Wdr77	NM_001008771	310769	0.52	3.33	1.32E-05
A_44_P775932	ENSRNOT00000036737	ENSRNOT00000036737		0.52	3.33	0.000112
A_44_P452550	XM_226639	XM_226639		0.52	3.33	0.007592
A_42_P473467	Cd276	NM_182824	315716	0.52	3.32	1.77E-05
A_44_P368241	Pola1	XM_242396	85241	0.52	3.32	0.003491
A_44_P494662	RGD1560851_predicted	XM_342749	362428	0.52	3.32	0.00312
A_44_P700084	Eif4e	NM_053974	117045	0.52	3.32	0.003279
A_44_P100729	XM_226408	XM_226408		0.52	3.32	0.000288
A_44_P324087	Ttll12_predicted	XM_235528	300105	0.52	3.32	0.000375
A_44_P119658	LOC365977	XM_345312		0.52	3.32	0.001795
A_44_P398663	LOC302833	XR_007436	302833	0.52	3.32	0.003011
A_44_P265399	Als2cr4_predicted	XM_237179		0.52	3.32	0.000187
A_44_P947418	TC548508	TC548508		0.52	3.31	4.05E-05
A_44_P372656	Grwd1	NM_001012067	308592	0.52	3.31	0.000332
A_44_P1057707	LOC289809	NM_199083	289809	0.52	3.31	0.005419
A_43_P18797	Ipo7_predicted	XM_219265		0.52	3.31	0.001833
A_44_P462661	Il1rn	NM_022194	60582	0.52	3.30	0.000432
A_44_P295223	RGD1310037_predicted	XM_345273	365903	0.52	3.30	9.88E-05
A_44_P321391	Gimap6	NM_001011968	297076	0.52	3.30	0.000873
A_44_P302265	LOC685936	XM_001065833		0.52	3.30	0.01112
A_43_P12589	Epha3	NM_031564	29210	0.52	3.30	0.002487
A_44_P283941	Arhgdib	NM_001009600	362456	0.52	3.30	4.52E-05
A_44_P334494	CK367091	CK367091		0.52	3.30	0.000499
A_44_P439473	Klf7_predicted	XM_343581		0.52	3.30	4.63E-05
A_44_P304064	P2ry14	NM_133577	171108	0.52	3.30	0.003924
A_44_P192988	Csnk1e	NM_031617	58822	0.52	3.30	0.000135
A_44_P1033758	Wdr36_predicted	XM_001060069		0.52	3.30	0.01923
A_44_P290582	Noc4l	NM_001014129	360828	0.52	3.30	4.8E-06
A_44_P160888	Ppp1r14b	NM_172045	259225	0.52	3.29	4.68E-06
A_42_P574719	Slc25a4	NM_053515	85333	0.52	3.29	0.000201
A_44_P306439	Fcgr3a	NM_207603	304966	0.52	3.29	0.003027
A_44_P294737	Reln	NM_080394	24718	0.52	3.29	0.002883
A_42_P608768	RGD1561608_predicted	XM_345202	365770	0.52	3.29	0.000243
A_44_P212826	Ero1l	NM_138528	171562	0.52	3.29	8.84E-05
A_43_P15776	Ptch1	NM_053566	89830	0.52	3.29	3.23E-05
A_43_P12708	Src	NM_031977	83805	0.52	3.29	0.000164
A_42_P700817	Apoa5	NM_080576	140638	0.52	3.29	0.001613
A_43_P12905	Bik	NM_053704	114496	0.52	3.29	2.09E-05
A_44_P508514	XM_222160	XM_222160		0.52	3.28	0.000072
A_44_P729898	Zcwcc1_predicted	XM_214072		0.52	3.28	0.000153
A_44_P168737	RGD1562123_predicted	XM_342840	362522	0.52	3.28	0.00045
A_43_P11792	Sod2	NM_017051	24787	0.52	3.28	9.01E-05
A_44_P494591	Sall1_predicted	XM_226329		0.52	3.28	0.000752
A_44_P219628	Ggt1	NM_053840	116568	0.52	3.28	0.001209
A_44_P869539	TC540306	TC540306		0.52	3.28	0.00062
A_43_P15248	LOC498549	XM_573823	498549	0.52	3.27	0.001829
A_44_P776321	DN932270	DN932270		0.51	3.27	0.0017
A_42_P786010	Ggta1	NM_145674	246766	0.51	3.27	0.012528

A_44_P981712	TC563075	TC563075		0.51	3.27	0.008118
A_44_P850918	AW142569	AW142569		0.51	3.27	0.000272
A_44_P149080	Fyn	NM_012755	25150	0.51	3.27	0.003993
A_44_P180538	Ing3	NM_001034107	312154	0.51	3.27	0.003556
A_44_P225105	Dach2_predicted	XM_001055336		0.51	3.27	0.015665
A_44_P353632	Paics	NM_080910	140946	0.51	3.26	5.79E-05
A_44_P283444	Slc19a1	NM_017299	29723	0.51	3.26	0.000106
A_44_P807777	E2f1	XM_001065036		0.51	3.26	0.012025
A_44_P374817	Atic	NM_031014	81643	0.51	3.26	7.68E-06
A_44_P262798	Col9a3_predicted	XM_342599		0.51	3.26	0.002609
A_44_P946478	TC541995	TC541995		0.51	3.26	0.002461
A_44_P463702	Dusp7	XM_001070874		0.51	3.26	2.08E-05
A_42_P653257	Ptpn7	NM_145683	246781	0.51	3.26	0.000598
A_43_P14927	Mmp14	NM_031056	81707	0.51	3.26	0.00036
A_44_P306486	AY387074	AY387074		0.51	3.26	0.000913
A_43_P12740	Eef1a1	NM_175838	171361	0.51	3.25	0.007135
A_43_P21063	RGD1560587_predicted	XM_244186	316539	0.51	3.25	0.001273
A_44_P361755	Dock11	XM_233283	313438	0.51	3.25	0.000905
A_42_P728472	Rps4x	NM_001007600	29426	0.51	3.25	0.000533
A_44_P935923	LOC680782	XM_001058844	680782	0.51	3.25	0.013103
A_44_P456792	Lamc1	XM_341133	117036	0.51	3.25	3.23E-05
A_44_P426608	Wdhd1_predicted	XM_223933		0.51	3.25	0.011801
A_44_P425848	AW915270	AW915270	362886	0.51	3.25	0.003261
A_44_P1056473	XM_216408	XM_216408		0.51	3.24	0.000895
A_44_P1060286	S100a4	NM_012618	24615	0.51	3.24	0.012959
A_44_P899333	TC555395	TC555395		0.51	3.24	0.000061
A_44_P958650	RGD1307688	NM_001014188	362394	0.51	3.24	0.008436
A_44_P180240	RGD1309602_predicted	XM_222559		0.51	3.24	0.004457
A_44_P1010653	LOC368001	XM_347165		0.51	3.23	0.013608
A_44_P173430	Morc4_predicted	XM_236536	315914	0.51	3.23	0.00412
A_44_P368493	BF396371	BF396371		0.51	3.23	0.004058
A_44_P168637	RGD1308848_predicted	XM_226262		0.51	3.23	0.001121
A_44_P116130	Hspcb	NM_001004082	301252	0.51	3.22	9.64E-05
A_44_P308964	X55180	X55180		0.51	3.22	0.001364
A_44_P160813	Shmt1	XM_213324		0.51	3.22	0.001337
A_44_P807734	BP504238	BP504238		0.51	3.22	0.001104
A_44_P1045724	RGD1308584_predicted	XM_213895		0.51	3.22	0.001553
A_44_P452811	Itga11_predicted	XM_236320		0.51	3.22	0.002739
A_44_P304265	Ehd2	NM_001024897	361512	0.51	3.22	0.002377
A_44_P854181	TC542382	TC542382		0.51	3.22	0.006959
A_44_P558636	Ppp1r9b	NM_053474	84686	0.51	3.21	7.77E-05
A_44_P235848	Bccip_predicted	XM_001055516		0.51	3.21	0.00132
A_44_P249805	LOC681811	XM_001058950		0.51	3.21	0.00029
A_44_P211182	RGD1306658	NM_001014216	363227	0.51	3.21	9.16E-05
A_44_P466796	XM_345984	XM_345984		0.51	3.21	0.017777
A_42_P563648	RGD1566025_predicted	XM_224282		0.51	3.21	2.37E-05
A_44_P222969	RGD1309285_predicted	XM_221028	303599	0.51	3.21	0.001712
A_44_P986963	RGD1564499_predicted	XM_001067952		0.51	3.21	0.017941
A_44_P100054	GalNAc4S6ST	NM_173310	286974	0.51	3.20	0.014645
A_44_P930234	Baz1a_predicted	XM_001079067		0.51	3.20	0.000623
A_43_P15932	Pcdh3	NM_173099	25133	0.51	3.20	0.004231
A_44_P189469	Lama5	XM_215963	140433	0.51	3.20	0.000164
A_44_P268915	BF289003	BF289003	297694	0.51	3.20	0.001077
A_44_P1016884	BF566249	BF566249		0.51	3.20	0.004538
A_44_P209911	Nolc1	NM_022869	64896	0.50	3.20	1.61E-05
A_44_P499309	XM_341708	XM_341708		0.50	3.20	0.000281
A_43_P18841	LOC301509	XM_237286	301509	0.50	3.20	0.002377
A_44_P357853	AW144351	AW144351		0.50	3.20	0.005831
A_44_P206417	A_44_P206417	A_44_P206417		0.50	3.19	2.09E-05
A_44_P390261	DY472268	DY472268		0.50	3.19	0.040824
A_44_P332834	RGD1566336_predicted	XM_217737	308064	0.50	3.19	0.000184
A_44_P183517	CB547153	CB547153		0.50	3.19	4.82E-05
A_44_P318435	Cct5	NM_001004078	294864	0.50	3.18	0.000243

A_44_P558163	Lipt1_predicted	XM_237067		0.50	3.18	0.01277
A_44_P304800	Col4a5_predicted	XM_343778	363457	0.50	3.18	0.001361
A_44_P837142	LOC500086	NM_001025045	500086	0.50	3.18	0.001455
A_44_P824642	TC562235	TC562235		0.50	3.18	0.001401
A_44_P335755	Twsg1_predicted	XM_343637		0.50	3.18	0.000109
A_43_P17490	Rrm2_mapped	NM_001025740	362720	0.50	3.17	0.000278
A_44_P1048611	RGD1308261_predicted	XM_216898	299818	0.50	3.17	0.002794
A_44_P140520	Tuba6	NM_001011995	300218	0.50	3.17	5.64E-05
A_44_P178479	Wdr50_predicted	XM_220851	303456	0.50	3.17	0.007259
A_42_P517399	Emp3	NM_030847	81505	0.50	3.17	0.000795
A_43_P22712	Dmwd	XM_001075375		0.50	3.17	4.46E-06
A_44_P464169	LOC311352	XM_001076480		0.50	3.17	0.000278
A_44_P636957	ENSRNOT00000044253	ENSRNOT00000044253		0.50	3.17	0.012445
A_44_P412277	Rps20	NM_001007603	122772	0.50	3.17	3.13E-05
A_44_P449774	Slc20a2	NM_017223	29502	0.50	3.17	0.000171
A_44_P1040685	Ier5l	NM_001025041	499772	0.50	3.17	6.68E-05
A_44_P328664	XM_233179	XM_233179		0.50	3.17	0.000363
A_44_P619079	AW918367	AW918367		0.50	3.17	0.001204
A_44_P680622	Clspn_predicted	XM_001058264		0.50	3.16	0.005336
A_44_P505851	Set_predicted	NM_001012504	307947	0.50	3.16	4.57E-05
A_44_P564080	A_44_P564080	A_44_P564080		0.50	3.16	0.010724
A_44_P243887	Col6a3_predicted	XM_346073		0.50	3.16	0.001485
A_44_P525151	RGD1309370_predicted	XM_219563		0.50	3.16	0.00027
A_44_P898364	ENSRNOT00000033729	ENSRNOT00000033729		0.50	3.16	0.000406
A_44_P468182	Dnm1	NM_080689	140694	0.50	3.16	0.001853
A_44_P525157	Zfp278	XM_223592		0.50	3.16	0.000898
A_44_P822283	TC535478	TC535478		0.50	3.16	0.008409
A_44_P220032	Ddx18	NM_001006996	308490	0.50	3.16	5.95E-05
A_44_P538045	AI599065	AI599065	282635	0.50	3.16	0.025204
A_44_P1057585	Htatip2_predicted	XM_214927		0.50	3.15	0.00008
A_44_P520058	Uhrf1_mapped	NM_001008882	316129	0.50	3.15	6.99E-05
A_44_P370030	Ptpre	XM_341950	114767	0.50	3.15	0.000995
A_43_P14909	Slc29a1	NM_031684	63997	0.50	3.15	0.000196
A_43_P20320	RGD1309708	NM_001014131	360867	0.50	3.15	0.000176
A_44_P593020	TC559950	TC559950		0.50	3.15	0.003306
A_44_P1026252	Gyltl1b	NM_199107	311202	0.50	3.15	0.032528
A_43_P22657	Satb1	NM_001012129	316164	0.50	3.15	0.00197
A_44_P400591	A_44_P400591	A_44_P400591		0.50	3.15	0.000127
A_44_P428651	Itgav_predicted	XM_230950		0.50	3.14	0.001691
A_44_P998638	Pprc1_predicted	XM_215259		0.50	3.14	0.000161
A_44_P344816	Smox_predicted	XM_218704	308652	0.50	3.14	0.00013
A_44_P354255	XM_214168	XM_214168		0.50	3.14	0.000217
A_44_P143650	AW142444	AW142444		0.50	3.14	0.000898
A_44_P363757	Dus4l_predicted	XM_345654	366593	0.50	3.13	0.018181
A_44_P879970	Sumf1_predicted	XM_342731		0.50	3.13	0.025861
A_44_P473220	RGD1564055_predicted	XM_344177		0.50	3.13	6.49E-05
A_44_P531324	Arhgap9	XM_576241		0.50	3.13	0.001338
A_43_P13011	Nlgn2	NM_053992	117096	0.50	3.13	0.00011
A_44_P262720	Mcm10_predicted	XM_225570		0.50	3.13	0.003677
A_44_P345159	Wipi1_predicted	XM_221063	303630	0.50	3.13	0.000201
A_44_P802638	BG671148	BG671148		0.50	3.13	0.001529
A_44_P844752	BF409128	BF409128		0.49	3.12	0.010917
A_44_P453543	RGD1308127	NM_001014248	365493	0.49	3.12	0.000292
A_44_P281733	Cias1_predicted	XM_220513	287362	0.49	3.12	0.000423
A_43_P11546	Cd24	NM_012752	25145	0.49	3.12	0.002553
A_44_P515912	LOC680308	XM_001056213		0.49	3.12	0.000201
A_42_P628981	Prss23	NM_001007691	308807	0.49	3.12	0.003375
A_44_P401170	Tmem50b	NM_001025014	360698	0.49	3.12	0.007027
A_44_P492879	LOC312299	XM_231705	312299	0.49	3.12	0.002711
A_44_P152395	Rnd1	NM_001013222	362993	0.49	3.12	0.000637
A_44_P474900	RGD1311827	NM_001013875	289753	0.49	3.12	0.00064
A_44_P478591	LOC685611	XM_001064525	685611	0.49	3.12	2.84E-05
A_44_P100618	XM_227387	XM_227387		0.49	3.12	0.00902

A_44_P959202	Vrk2_predicted	XM_001064313		0.49	3.11	0.001089
A_44_P236393	RGD1305605_predicted	XM_214059		0.49	3.11	0.000182
A_44_P773951	RGD1561319_predicted	XM_575592		0.49	3.11	0.000212
A_44_P374894	U06102	U06102		0.49	3.11	0.001475
A_43_P18612	RGD1564108_predicted	XM_238042	291936	0.49	3.11	0.002583
A_44_P817539	Gpiap1	NM_001012185	362173	0.49	3.11	0.00791
A_44_P238019	AW143671	AW143671	362401	0.49	3.11	0.001021
A_44_P553665	Rps9	NM_031108	81772	0.49	3.11	5.49E-05
A_44_P775118	Tmem123	NM_001014205	363013	0.49	3.11	0.000133
A_44_P117119	Gloxd1	NM_001014068	313521	0.49	3.11	0.000563
A_44_P388782	Cdk4	NM_053593	94201	0.49	3.11	5.87E-05
A_44_P921080	CO401386	CO401386		0.49	3.11	0.013523
A_44_P436061	RGD1311900_predicted	XM_223533	305449	0.49	3.10	8.53E-05
A_43_P23147	Rnf43_predicted	XM_220829	303412	0.49	3.10	0.002907
A_44_P398742	Aph1b	XM_217185		0.49	3.10	0.000591
A_44_P229411	Osm_mapped	NM_001006961	289747	0.49	3.10	0.003184
A_44_P468879	Btk	NM_001007798	367901	0.49	3.10	0.000813
A_44_P499129	RGD1311300	NM_001030043		0.49	3.10	0.003559
A_44_P1023770	LOC684802	XM_001073816		0.49	3.10	0.003829
A_44_P276220	Gtf2f2	NM_031042	81674	0.49	3.09	7.93E-05
A_44_P150432	D38557	D38557		0.49	3.09	0.00198
A_44_P869077	CN541273	CN541273		0.49	3.09	0.016446
A_44_P995132	Pcna	NM_022381	25737	0.49	3.09	0.000165
A_44_P852634	LOC501135	XR_007953	501135	0.49	3.09	0.000111
A_44_P285726	Rcn3_predicted	NM_001008694	494125	0.49	3.09	0.000171
A_44_P549198	Ift88_predicted	XM_224165		0.49	3.09	0.003072
A_44_P326727	Snd1	NM_022694	64635	0.49	3.09	8.56E-05
A_44_P899127	LOC500300	NM_001024334	500300	0.49	3.09	0.016533
A_42_P600817	Trpv4	NM_023970	66026	0.49	3.09	0.020584
A_44_P478313	RGD1311080_predicted	XM_231864		0.49	3.09	0.005711
A_42_P508185	Gimap5	NM_145680	246774	0.49	3.08	0.000544
A_43_P12794	Ngfrap1	NM_053401	117089	0.49	3.08	2.19E-05
A_44_P591548	Pols_predicted	XM_225072		0.49	3.08	0.000619
A_44_P901817	TC550176	TC550176		0.49	3.08	0.000191
A_42_P472133	Slc2a6_predicted	XM_238321		0.49	3.08	0.003377
A_44_P393822	RGD1311487	NM_001025119	310674	0.49	3.08	0.00093
A_44_P793372	TC561351	TC561351		0.49	3.08	0.00084
A_44_P128848	A_44_P128848	A_44_P128848		0.49	3.08	0.000704
A_44_P871910	Zfp216_predicted	XM_001079764		0.49	3.08	0.014015
A_42_P699162	Ruvbl1	NM_147177	65137	0.49	3.08	1.52E-05
A_44_P767512	TC562237	TC562237		0.49	3.08	0.023561
A_42_P768732	AW918387	AW918387	313210	0.49	3.08	2.62E-05
A_44_P365421	Rpl15	NM_139114	245981	0.49	3.07	4.79E-05
A_42_P734094	Notch4	NM_001002827	406162	0.49	3.07	4.29E-05
A_44_P541623	RGD1310875_predicted	XM_217013		0.49	3.07	0.019551
A_44_P196824	MGC94010	NM_001007732	361241	0.49	3.07	0.002019
A_44_P346782	Ncdn	NM_053543	89791	0.49	3.07	6.47E-05
A_44_P1052504	Rhot2	NM_181823	287156	0.49	3.07	0.007996
A_44_P161652	A_44_P161652	A_44_P161652		0.49	3.07	0.000343
A_44_P227716	RGD1564291_predicted	XM_227060	310341	0.49	3.07	0.002503
A_44_P453870	BM388719	BM388719	297755	0.49	3.07	0.000178
A_43_P20311	Hs6st1_predicted	XM_237060		0.49	3.07	0.000135
A_44_P729926	AA957540	AA957540		0.49	3.07	7.68E-06
A_44_P491286	AA850885	AA850885		0.49	3.07	0.002005
A_44_P865620	AW914057	AW914057		0.49	3.07	0.002687
A_44_P496788	Fip1l1	NM_001008295	289582	0.49	3.06	0.006168
A_44_P147040	Adam10	XM_217197	29650	0.49	3.06	0.001167
A_44_P290767	Gtf2e2_predicted	XM_224929		0.49	3.06	0.01183
A_44_P501843	Shmt2	NM_001008322	299857	0.49	3.06	0.000071
A_44_P249033	Hmga1	NM_139327	117062	0.49	3.06	0.000112
A_44_P279768	Arid3a_predicted	XM_234884		0.49	3.06	3.26E-05
A_44_P267423	RGD1310754_predicted	XM_001079537		0.49	3.06	0.041221
A_44_P209459	Pafah1b2	NM_022387	64189	0.49	3.06	0.000184

A_42_P768024	Spn	XM_344964	24796	0.49	3.06	0.000302
A_44_P521353	Cct7_predicted	XM_216180		0.49	3.06	0.000287
A_44_P267554	Vsig1	NM_001037784	315920	0.49	3.06	0.009471
A_44_P149087	Fyn	NM_012755	25150	0.49	3.05	8.61E-05
A_44_P231677	XM_344884	XM_344884		0.48	3.05	0.000459
A_42_P751983	Uble1b	XM_218502	308508	0.48	3.05	8.81E-05
A_44_P382372	RGD1560892_predicted	XR_007808	302730	0.48	3.05	0.001335
A_44_P1024341	BF550565	BF550565		0.48	3.05	5.24E-05
A_44_P174278	Bhlhb5_predicted	XM_345190		0.48	3.05	0.001193
A_44_P352268	Plk2	NM_031821	83722	0.48	3.05	0.001402
A_44_P191778	Cyp1b1	NM_012940	25426	0.48	3.05	0.033866
A_43_P19242	Glis2_predicted	XM_220169		0.48	3.05	0.000431
A_44_P992578	Rrm2_mapped	NM_001025740	362720	0.48	3.05	0.000523
A_44_P112586	RGD1560909_predicted	XM_343560		0.48	3.04	0.011981
A_44_P685554	TC561034	TC561034		0.48	3.04	0.000198
A_44_P261874	Hspca	NM_175761	299331	0.48	3.04	3.64E-05
A_44_P480135	Kcnmb1	NM_019273	29747	0.48	3.04	0.02275
A_44_P732819	BQ210430	BQ210430		0.48	3.04	0.036781
A_44_P248619	Plxnb1_predicted	XM_236640		0.48	3.04	2.03E-05
A_44_P363568	XM_344680	XM_344680		0.48	3.04	4.14E-05
A_42_P621642	Lsp1	XM_341964	361680	0.48	3.04	0.000407
A_44_P234212	Olfml3_predicted	XM_227535		0.48	3.04	0.001295
A_42_P672545	Serinc2	NM_001031656	313057	0.48	3.03	8.32E-05
A_44_P875945	BI296619	BI296619		0.48	3.03	0.003574
A_44_P443271	Rbpsuhl_predicted	XM_342576		0.48	3.03	0.002041
A_43_P17525	RGD1305020_predicted	XM_230785		0.48	3.03	0.001793
A_44_P478233	Pak6_predicted	XM_230519		0.48	3.03	0.000121
A_44_P387078	Rbm28_predicted	XM_231550		0.48	3.03	0.000197
A_44_P331142	XM_220700	XM_220700		0.48	3.03	8.92E-05
A_42_P702258	Padi4	NM_017227	29512	0.48	3.03	0.000187
A_44_P659230	A_44_P659230	A_44_P659230		0.48	3.03	0.000307
A_44_P104069	Nola2_predicted	XM_213293		0.48	3.03	5.67E-05
A_44_P179956	Igtp	XM_220451	303163	0.48	3.03	0.001906
A_42_P469969	Igsf10	NM_198768	310448	0.48	3.02	0.009369
A_43_P22238	RGD1306802	NM_001024772	306387	0.48	3.02	0.000159
A_44_P414934	RGD1562003_predicted	XM_341638	361358	0.48	3.02	0.01646
A_44_P386977	XM_342401	XM_342401		0.48	3.02	0.004658
A_44_P309013	Mapt	NM_017212	29477	0.48	3.01	0.009893
A_44_P914868	CO567020	CO567020		0.48	3.01	0.015859
A_44_P714935	Zfp216_predicted	XM_001079764		0.48	3.01	0.007862
A_44_P523924	Ciapin1	NM_001007689	307649	0.48	3.01	8.74E-05
A_44_P269752	Rnf125_predicted	XM_001054760		0.48	3.01	0.001575
A_44_P889591	TC543946	TC543946		0.48	3.01	0.032577
A_44_P494465	Kctd12_predicted	XM_344450	364458	0.48	3.01	0.000135
A_44_P668233	TC553641	TC553641		0.48	3.01	6.26E-05
A_44_P437956	Stc1	NM_031123	81801	0.48	3.00	0.000821
A_44_P119499	LOC690450	XM_001074480	690450	0.48	3.00	0.001405
A_44_P1020422	Nes	NM_012987	25491	0.48	3.00	0.00981
A_44_P745269	Ak3l1	NM_017135	29223	0.48	3.00	0.028564
A_44_P185629	BE117514	BE117514		0.48	3.00	0.008452
A_44_P344096	Ehd3	NM_138890	192249	0.48	3.00	0.062282
A_44_P530187	Plekhq1_predicted	XM_236354	315764	0.48	3.00	0.001008
A_44_P468468	Lbp	NM_017208	29469	0.48	3.00	0.096783
A_44_P269230	Eif3s7	NM_001004283	362952	0.48	3.00	7.74E-05
A_44_P255973	BF556545	BF556545		0.48	3.00	4.81E-05
A_44_P492533	A_44_P492533	A_44_P492533		0.48	2.99	8.73E-05
A_44_P121374	Hsph1	NM_001011901	288444	0.48	2.99	0.002753
A_43_P19312	Gmcl1	NM_001033931	312516	0.48	2.99	0.017233
A_43_P15904	Fcgr1	XM_215643		0.48	2.99	0.001582
A_44_P331464	Gstm5	NM_172038	64352	0.48	2.99	0.00153
A_42_P537051	RGD1566042_predicted	XM_214392		0.48	2.99	0.022529
A_44_P241190	Sdccag10	NM_001013199	361887	0.48	2.99	0.000135
A_44_P1057403	Ptrf_predicted	XM_001081467		0.48	2.99	0.006432

A_44_P457833	Trim32	NM_001012103	313264	0.48	2.99	0.000505
A_44_P543957	Wdr18	NM_001039027	314617	0.47	2.99	0.000191
A_44_P546123	Mcm5_predicted	XM_226316		0.47	2.98	0.000166
A_42_P766909	Ppp1r14a	NM_130403	114004	0.47	2.98	0.00024
A_44_P105069	RGD1559997_predicted	XM_344794		0.47	2.98	0.001256
A_43_P12559	Camkk2	NM_031338	83506	0.47	2.98	0.002388
A_44_P463954	Hlx1_predicted	XM_344184		0.47	2.98	0.003699
A_44_P246202	LOC500703	XR_006625	500703	0.47	2.98	0.000161
A_44_P508239	Shmt1	XM_213324		0.47	2.98	0.000878
A_44_P241241	Them4	NM_001025017	361992	0.47	2.98	0.008653
A_42_P689631	Ephb6	XM_231650		0.47	2.98	0.002913
A_44_P1048043	Pabpc4	XM_001054606		0.47	2.98	0.000111
A_44_P508992	LOC299524	XR_008192	299524	0.47	2.98	9.62E-05
A_44_P473329	Trim59_predicted	XM_001062051		0.47	2.97	0.014047
A_44_P255945	A_44_P255945	A_44_P255945		0.47	2.97	0.001116
A_44_P1036068	RGD1311747_predicted	XM_214341	290706	0.47	2.97	0.000174
A_44_P189696	LOC683179	XM_001064803		0.47	2.97	0.007171
A_44_P309034	Dlc1	XM_341444	58834	0.47	2.97	0.001574
A_44_P279331	Rnf149	XM_343561	363222	0.47	2.97	0.001619
A_43_P18504	Amotl2	XM_343457	65157	0.47	2.96	0.000153
A_44_P147558	Cfl2_predicted	XM_345674		0.47	2.96	0.040988
A_44_P539111	Leprel2_predicted	XM_216278		0.47	2.96	0.000041
A_44_P932360	TC566177	TC566177		0.47	2.96	0.004221
A_43_P13828	AW914006	AW914006	84382	0.47	2.96	0.000124
A_42_P796827	Ddx59	NM_001005535	289402	0.47	2.96	4.34E-05
A_44_P292510	Slc2a3	NM_017102	25551	0.47	2.96	0.010162
A_44_P384531	Rap1gds1_predicted	XM_227749		0.47	2.96	0.000127
A_44_P1017335	Cse1l_predicted	XM_342581		0.47	2.96	0.00016
A_44_P297974	Fmnl1_predicted	XM_213487		0.47	2.96	0.000426
A_44_P156805	Pogz_predicted	XM_227475		0.47	2.96	0.016258
A_42_P682216	RGD1359339	NM_001007679	305070	0.47	2.95	0.000154
A_44_P227121	RGD1306755_predicted	XM_341301	361025	0.47	2.95	0.00071
A_44_P121556	RGD1305222_predicted	XM_214332	290686	0.47	2.95	4.21E-05
A_42_P548796	Tsn	NM_021762	60381	0.47	2.95	0.000351
A_42_P595960	Ppia	NM_017101	25518	0.47	2.95	0.000321
A_42_P746851	AW916157	AW916157		0.47	2.95	4.46E-05
A_42_P512460	MGC125271	NM_001037348	288480	0.47	2.95	3.88E-05
A_43_P18235	RGD1560871_predicted	XM_001072622		0.47	2.95	0.000129
A_42_P550914	Ltb	NM_212507	361795	0.47	2.95	6.94E-05
A_44_P231534	Rps5	XM_341788		0.47	2.95	0.000343
A_44_P1029107	Twistnb_predicted	XM_343050		0.47	2.95	0.000113
A_44_P143061	RGD1559787_predicted	XM_576514		0.47	2.95	0.001162
A_44_P1034439	Cxcl12	NM_022177	24772	0.47	2.94	0.009551
A_44_P855525	TC566261	TC566261		0.47	2.94	0.002719
A_44_P769703	Nrep	NM_178096	338475	0.47	2.94	0.000375
A_44_P174992	Ifi47	NM_172019	246208	0.47	2.94	0.001029
A_44_P697615	AW917911	AW917911		0.47	2.94	0.0038
A_44_P541297	Slc27a3_predicted	XM_215605		0.47	2.94	0.000325
A_43_P12029	Rgs5	NM_019341	54294	0.47	2.94	0.001772
A_42_P743580	Rgs4	NM_017214	29480	0.47	2.94	0.00077
A_44_P440885	Dcdc2_predicted	XM_225316		0.47	2.94	0.000134
A_43_P10050	Rps6	NM_017160	29304	0.47	2.94	8.62E-06
A_44_P631376	TC538612	TC538612		0.47	2.94	0.00911
A_44_P137623	Ppp1r9b	NM_053474	84686	0.47	2.94	0.000106
A_44_P452245	Serpine1	NM_012620	24617	0.47	2.94	0.002747
A_44_P499357	Casp8ap2_predicted	XM_232860		0.47	2.94	0.018133
A_44_P1029697	Chl1	XM_001077843		0.47	2.93	0.003488
A_43_P19558	XM_341729	XM_341729		0.47	2.93	0.000716
A_42_P671125	Trim28	XM_344861	116698	0.47	2.93	7.82E-05
A_44_P554454	Txnrd1	NM_031614	58819	0.47	2.93	7.62E-05
A_42_P541032	Ftl1	NM_022500	29292	0.47	2.93	0.002267
A_44_P525412	Slc6a14	NM_001037544	298340	0.47	2.93	0.007899
A_44_P203993	RGD1562123_predicted	XM_342840	362522	0.47	2.93	0.001915

A_44_P267706	RGD1309798_predicted	XM_235463	300060	0.47	2.93	0.000637
A_44_P158798	Cspg2	XM_215451	114122	0.47	2.93	0.000447
A_44_P443030	Sept6_predicted	XM_223227		0.47	2.93	8.27E-05
A_44_P225268	Smarcd1_predicted	XM_343332		0.47	2.93	0.016746
A_44_P221245	Sox9	XM_001081628		0.47	2.93	0.000218
A_44_P147661	Rab23_predicted	XM_346033		0.47	2.93	0.010926
A_44_P505694	Cyp20a1	NM_199401	316435	0.47	2.93	0.000193
A_42_P708181	Kntc1_predicted	XM_222157		0.47	2.93	0.001508
A_44_P457246	RGD1560732_predicted	XM_574766	499443	0.47	2.93	0.000401
A_44_P527470	A_44_P527470	A_44_P527470		0.47	2.93	0.046966
A_44_P931922	TC528241	TC528241		0.47	2.93	0.002529
A_44_P534635	XM_345665	XM_345665		0.47	2.92	0.000607
A_44_P671320	LOC361230	XM_001061410		0.47	2.92	0.028684
A_44_P1019710	Slc39a6	NM_001024745	291733	0.47	2.92	8.81E-05
A_44_P975868	TC539434	TC539434		0.47	2.92	0.000224
A_44_P414066	Ldhb	NM_012595	24534	0.47	2.92	0.00029
A_44_P1043051	RGD1311316	NM_001012182	362007	0.47	2.92	0.000294
A_44_P839551	TC527740	TC527740		0.47	2.92	5.79E-05
A_44_P297659	LOC687202	XM_001077533		0.47	2.92	0.007196
A_44_P480183	L07406	L07406		0.46	2.92	0.011624
A_44_P415269	Rhbdl6_predicted	XM_221133		0.46	2.91	5.07E-05
A_44_P175349	Crispld2	NM_138518	171547	0.46	2.91	0.003712
A_42_P544108	Arbp	NM_022402	64205	0.46	2.91	0.000478
A_44_P429321	RGD1307315	NM_001008377	362793	0.46	2.91	0.000117
A_44_P973562	Tspan5	NM_001004090	362048	0.46	2.91	0.000301
A_42_P793008	Rps13	NM_130432	161477	0.46	2.91	5.89E-05
A_44_P464812	RGD1560587_predicted	XM_244186	316539	0.46	2.91	0.000385
A_44_P163408	Slc12a2	NM_031798	83629	0.46	2.91	3.98E-05
A_44_P383825	Calu	NM_022535	64366	0.46	2.91	4.77E-05
A_44_P808472	TC524556	TC524556		0.46	2.91	0.001157
A_44_P223045	BQ200849	BQ200849	310201	0.46	2.91	0.010927
A_44_P649871	AW144155	AW144155		0.46	2.91	5.72E-05
A_44_P286107	Hbxip_predicted	XM_215674		0.46	2.91	0.010742
A_44_P342101	Arl3	NM_022700	64664	0.46	2.91	0.000136
A_44_P925410	BF386740	BF386740		0.46	2.90	0.000413
A_44_P153979	Tcf8	NM_013164	25705	0.46	2.90	0.006049
A_44_P541958	AI236164	AI236164	25478	0.46	2.90	0.000626
A_44_P525600	Timm8a	NM_053370	84383	0.46	2.90	5.51E-05
A_44_P210520	Smad2	NM_019191	29357	0.46	2.90	0.001019
A_43_P10332	XM_345550	XM_345550		0.46	2.90	0.000376
A_44_P212178	Rab12	XM_001053530		0.46	2.90	0.003624
A_44_P348533	AW918669	AW918669		0.46	2.90	0.000282
A_44_P585654	CK469296	CK469296		0.46	2.90	0.000377
A_44_P715517	RGD1309228	NM_001017451	298851	0.46	2.90	0.000238
A_44_P231867	RGD1309592	NM_001012346	290963	0.46	2.90	0.001153
A_43_P16666	RGD1309228	NM_001017451	298851	0.46	2.90	8.84E-05
A_44_P512642	Impact	NM_001012235	497198	0.46	2.90	0.024819
A_44_P452860	A_44_P452860	A_44_P452860		0.46	2.90	0.000187
A_44_P466189	Angptl2	NM_133569	171100	0.46	2.89	0.005399
A_42_P813567	Scd2	NM_031841	83792	0.46	2.89	0.000115
A_44_P546352	Tmtc3_predicted	XM_235093	314785	0.46	2.89	0.002304
A_44_P101361	Hmga2	NM_032070	84017	0.46	2.89	0.000338
A_44_P393791	Hps3_predicted	XM_227003		0.46	2.89	0.001764
A_44_P824032	Nup160_predicted	XM_230286		0.46	2.89	0.009608
A_44_P897570	AW144439	AW144439		0.46	2.89	0.027477
A_43_P10919	Exosc8_predicted	XM_215566		0.46	2.89	0.013613
A_43_P16608	XM_228063	XM_228063		0.46	2.89	0.000106
A_44_P466118	Slc11a1	NM_001031658	316519	0.46	2.89	0.002028
A_44_P548203	Rbm3	XM_343773	114488	0.46	2.89	5.74E-05
A_44_P454202	Acvr1	NM_024486	79558	0.46	2.89	0.005919
A_42_P495397	Rpl21	NM_053330	79449	0.46	2.88	0.000129
A_44_P886861	Ccdc32	NM_001024245	296081	0.46	2.88	0.003894
A_44_P355947	Rpl3	NM_198753	300079	0.46	2.88	4.28E-05

A_44_P250446	Zfp580_predicted	XM_218196	308336	0.46	2.88	0.002622
A_44_P487930	BE112513	BE112513		0.46	2.88	0.012115
A_43_P23280	Psip1	NM_175765	313323	0.46	2.88	0.000222
A_43_P15587	Map3k8	NM_053847	116596	0.46	2.88	0.000653
A_44_P403247	XM_347141	XM_347141		0.46	2.88	0.001002
A_43_P10894	Nars	NM_001025635	291556	0.46	2.88	0.000581
A_44_P457548	Parp9_predicted	XM_221404		0.46	2.88	0.015778
A_44_P895315	BF523017	BF523017		0.46	2.88	0.002676
A_44_P849046	AW915417	AW915417	365042	0.46	2.87	0.000181
A_44_P471166	Ddx21a	NM_001037201	317399	0.46	2.87	1.95E-05
A_43_P11845	Stmn1	NM_017166	29332	0.46	2.87	0.000238
A_44_P137759	RGD1308055_predicted	XM_218484	308496	0.46	2.87	0.007755
A_44_P989647	A_44_P989647	A_44_P989647		0.46	2.87	0.00021
A_44_P1004868	RGD1310681_predicted	XM_216357	297903	0.46	2.87	0.020418
A_44_P210202	Polg	NM_053528	85472	0.46	2.87	0.000466
A_44_P145416	Fkbp9	NM_001007646	297123	0.46	2.87	0.003842
A_44_P118929	Tp53	NM_030989	24842	0.46	2.87	7.32E-05
A_43_P16471	Taf6l_predicted	XM_219557		0.46	2.87	0.001426
A_44_P438013	Lag3	NM_212513	297596	0.46	2.87	0.003645
A_44_P229776	Nedd4a	XM_001053672		0.46	2.87	0.000583
A_44_P388777	Cdk4	NM_053593	94201	0.46	2.87	4.76E-05
A_44_P945664	LOC683722	XM_001064124		0.46	2.87	0.005343
A_44_P1044517	RGD1305179_predicted	XM_214648	291921	0.46	2.86	0.040508
A_44_P314447	RGD1305622	NM_001013854	287173	0.46	2.86	0.025789
A_42_P808456	Disp1_predicted	XM_213964		0.46	2.86	0.003917
A_44_P420640	LOC683983	XM_001068349		0.46	2.86	0.001066
A_44_P417489	RGD1307179_predicted	XM_216918	299900	0.46	2.86	0.000512
A_42_P805200	Arid2_predicted	XM_001059099		0.46	2.86	0.000952
A_44_P408108	Nbeal2_predicted	XM_236649	316014	0.46	2.86	8.49E-05
A_44_P425580	Cxcr4	NM_022205	60628	0.46	2.86	0.000406
A_44_P650550	BF289404	BF289404		0.46	2.86	0.008343
A_44_P221437	Dtx4	XM_238163		0.46	2.86	8.73E-05
A_44_P701356	Rbm27_predicted	XM_341605		0.46	2.86	0.008694
A_44_P538670	Slc33a1	NM_022252	64018	0.46	2.86	0.003514
A_44_P992697	Dusp19_predicted	XM_230039		0.46	2.85	0.001386
A_44_P227236	Btn2a2_predicted	XM_225366	306957	0.46	2.85	0.000868
A_44_P385117	Rdx	NM_001005889	315655	0.46	2.85	0.001428
A_44_P487249	Mif	NM_031051	81683	0.46	2.85	0.000112
A_42_P747877	Eif2b1	NM_172029	64514	0.46	2.85	0.000414
A_44_P172814	Tubb5	NM_173102	29214	0.46	2.85	6.53E-05
A_44_P536749	Metapl1_predicted	XM_230988		0.46	2.85	0.002543
A_43_P14271	Rps17	NM_017152	29286	0.45	2.85	0.000031
A_44_P123622	ENSRNOT00000027484	ENSRNOT00000027484		0.45	2.85	0.002388
A_44_P192964	Rcor2	NM_001013994	305811	0.45	2.85	0.001416
A_44_P534557	RGD1561019_predicted	XM_228821	317405	0.45	2.85	0.007399
A_43_P11840	Xdh	NM_017154	497811	0.45	2.85	0.000957
A_44_P508750	LOC684862	XM_001072231		0.45	2.85	0.000464
A_44_P196651	XM_222621	XM_222621		0.45	2.85	0.005572
A_44_P429298	LOC500590	NM_001025773	500590	0.45	2.85	0.000189
A_42_P774439	BG663067	BG663067	305227	0.45	2.84	0.000145
A_42_P700288	LOC690374	XM_221124		0.45	2.84	0.001469
A_44_P190212	LOC367289	XM_346058	367289	0.45	2.84	0.000419
A_44_P175912	RGD1309838_predicted	XM_234727	314539	0.45	2.84	0.000251
A_44_P764517	TC555471	TC555471		0.45	2.84	0.001388
A_44_P142318	Anapc7_predicted	XM_222171		0.45	2.84	0.00155
A_44_P577090	TC558454	TC558454		0.45	2.84	0.002012
A_44_P318846	Prmt5_predicted	XM_344405		0.45	2.84	1.74E-05
A_44_P986010	CF108537	CF108537		0.45	2.84	0.028664
A_44_P259926	Cct5	NM_001004078	294864	0.45	2.84	6.85E-05
A_44_P760131	LOC498264	XR_005936	498264	0.45	2.84	0.002026
A_44_P210039	Smg7_predicted	XM_341132	360855	0.45	2.84	0.040184
A_44_P251430	U48828	U48828		0.45	2.84	0.030819
A_44_P525364	Slc7a6_predicted	XM_226422		0.45	2.84	9.37E-05

A_44_P503570	Parg	NM_031339	83507	0.45	2.83	8.36E-05
A_44_P608365	Zfp265	NM_031616	58821	0.45	2.83	0.00127
A_44_P624482	TC546118	TC546118		0.45	2.83	0.000539
A_44_P305689	Lap3	NM_001011910	289668	0.45	2.83	0.021419
A_44_P915530	Twistnb_predicted	XM_001076417		0.45	2.83	0.004231
A_44_P130837	ENSRNOT00000016986	ENSRNOT00000016986		0.45	2.83	0.020931
A_44_P510222	AW918161	AW918161	502091	0.45	2.83	2.44E-05
A_44_P299909	RGD1305640_predicted	NM_001017932	307494	0.45	2.83	0.000154
A_44_P864185	AW142955	AW142955		0.45	2.83	0.002747
A_44_P250620	RGD1311955_predicted	XM_001065975		0.45	2.83	3.88E-05
A_44_P523244	RGD1305976_predicted	XM_343253	362923	0.45	2.83	0.004141
A_44_P136707	BG664788	BG664788		0.45	2.83	1.71E-05
A_44_P1070980	BF289236	BF289236	313564	0.45	2.83	0.017496
A_44_P265326	Pus3_predicted	XM_235995		0.45	2.83	0.001333
A_44_P883445	A_44_P883445	A_44_P883445		0.45	2.83	0.007237
A_44_P231923	LOC691918	XM_001080091	691918	0.45	2.83	0.000898
A_44_P487676	Rpl32	NM_013226	28298	0.45	2.83	0.00006
A_42_P772136	LOC686611	XM_001074804		0.45	2.83	0.000314
A_44_P538675	Pdcl	NM_022247	64013	0.45	2.82	0.003147
A_43_P10599	BF550231	BF550231	299613	0.45	2.82	6.48E-05
A_44_P343859	Csf1	NM_023981	78965	0.45	2.82	9.59E-05
A_44_P228942	Runx1	NM_017325	50662	0.45	2.82	0.00017
A_44_P111368	MGC125015	NM_001037356	361232	0.45	2.82	0.00011
A_44_P248464	Tspan6	XM_217563	302313	0.45	2.82	0.001041
A_44_P1018129	TC524716	TC524716		0.45	2.82	0.000782
A_44_P438362	LOC685076	XM_001062178		0.45	2.82	0.006415
A_44_P138230	XM_236238	XM_236238		0.45	2.82	0.002129
A_43_P21223	Ftsj3	NM_001012014	303608	0.45	2.82	4.47E-05
A_44_P311771	Bub1b	XM_342494	171576	0.45	2.82	0.000398
A_44_P1039184	Zfp216_predicted	XM_215251		0.45	2.82	0.000107
A_42_P679371	Coro1a	NM_130411	155151	0.45	2.82	0.000639
A_44_P330971	XM_216438	XM_216438		0.45	2.81	7.52E-05
A_44_P370568	LOC686806	XM_001074571		0.45	2.81	3.31E-05
A_43_P17061	Rnf3_predicted	XM_223729		0.45	2.81	0.001216
A_43_P18698	40057	NM_001012460	293507	0.45	2.81	0.002223
A_44_P147702	RGD1311017_predicted	XM_340853	360574	0.45	2.81	6.55E-05
A_44_P607062	Iars_predicted	XM_225196	306804	0.45	2.81	0.000106
A_44_P428455	Cygb	NM_130744	170520	0.45	2.81	0.005895
A_44_P381994	XM_221817	XM_221817		0.45	2.81	0.008127
A_42_P599210	Rexo2	NM_001008326	300689	0.45	2.81	0.000155
A_44_P890506	A_44_P890506	A_44_P890506		0.45	2.81	0.024669
A_44_P128777	RGD1561526_predicted	XM_233885	313906	0.45	2.81	0.003311
A_44_P941132	Chordc1_predicted	XM_235878		0.45	2.81	8.35E-05
A_44_P610017	TC526232	TC526232		0.45	2.81	0.002309
A_42_P622837	RGD1306819	NM_001013860	287593	0.45	2.80	0.000204
A_44_P391824	Rbbp4_predicted	XM_001061087		0.45	2.80	0.000679
A_44_P311247	MGC72932	NM_212515	300092	0.45	2.80	2.38E-05
A_44_P248235	Dok2_predicted	XM_224344		0.45	2.80	0.003069
A_44_P402584	Gtf2i	NM_001001512	353256	0.45	2.80	0.000309
A_44_P401536	Lnk	NM_031621	58838	0.45	2.80	0.018522
A_44_P208880	Dnaja3	NM_001038595	360481	0.45	2.80	0.0012
A_42_P566250	LOC685781	XM_001065242		0.45	2.80	0.000374
A_44_P344679	Abcc4	NM_133411	170924	0.45	2.80	0.000249
A_44_P243604	RGD1566320_predicted	XM_238286		0.45	2.80	0.002429
A_42_P631128	Rps17	NM_017152	29286	0.45	2.79	0.000021
A_44_P318999	A_44_P318999	A_44_P318999		0.45	2.79	0.000484
A_44_P845274	TC547153	TC547153		0.45	2.79	8.53E-05
A_44_P482658	Slc11a2	AF029757	25715	0.45	2.79	0.001587
A_44_P539373	RGD1560033_predicted	XM_220919		0.45	2.79	0.000271
A_44_P252630	RGD1306344	XM_230878		0.45	2.79	0.001156
A_42_P771346	Acvrl1	NM_022441	25237	0.45	2.79	0.010741
A_44_P304825	LOC302786	XR_008128	302786	0.45	2.79	0.000208
A_43_P12315	P2ry12	NM_022800	64803	0.45	2.79	0.003252

A_44_P436008	Lamc2	XM_213902	192362	0.44	2.79	0.000319
A_44_P360478	Norp	NM_022621		0.44	2.79	0.000128
A_44_P154367	Cdc16	NM_001024744	290875	0.44	2.79	0.000924
A_43_P20310	Wiz_predicted	XM_234841		0.44	2.78	0.000432
A_44_P100913	LOC691493	XM_001078534	691493	0.44	2.78	0.006546
A_44_P363252	Set_predicted	NM_001012504	307947	0.44	2.78	0.000143
A_42_P559737	Pgsg	NM_020074	56782	0.44	2.78	0.00027
A_44_P525392	Nup205_predicted	XM_342657		0.44	2.78	0.000058
A_44_P855088	TC562194	TC562194		0.44	2.78	0.000184
A_44_P438822	Spats2_predicted	XM_217030	300221	0.44	2.78	0.013289
A_44_P172701	Cspg5	NM_133652	50568	0.44	2.78	0.012589
A_44_P327250	Kctd13	NM_198736	293497	0.44	2.78	0.001231
A_44_P472994	Anxa5	NM_013132	25673	0.44	2.78	0.000527
A_44_P238978	Ris2_predicted	XM_226545		0.44	2.78	0.000471
A_44_P192325	Klhl13	XM_233297	313445	0.44	2.78	0.007251
A_42_P745356	Sox9	XM_001081628		0.44	2.78	0.000145
A_44_P507274	Dars	NM_053799	116483	0.44	2.78	0.005052
A_44_P129900	Cnot6l_predicted	XM_001062857		0.44	2.78	0.016753
A_44_P407771	Ifi44	XM_227821		0.44	2.78	0.00673
A_44_P416862	Rpl28	NM_022697	64638	0.44	2.77	3.04E-05
A_44_P285247	RGD1559986_predicted	XM_347039	362294	0.44	2.77	0.003067
A_44_P323496	Inpp5d	NM_019311	54259	0.44	2.77	0.00042
A_44_P588315	Xlkd1_predicted	XM_219001		0.44	2.77	0.099402
A_44_P373516	LOC498015	XM_573213	498015	0.44	2.77	0.000181
A_44_P346426	Wdr12	NM_199410	363237	0.44	2.77	0.000123
A_44_P537875	Habp4_predicted	XM_341479		0.44	2.77	0.006086
A_43_P19449	Kirrel1	NM_207606	310695	0.44	2.77	0.004377
A_44_P858398	LOC498145	NM_001017485	498145	0.44	2.77	0.02165
A_42_P679251	Ascl2	NM_031503	24209	0.44	2.77	0.002791
A_44_P142445	RGD1310992	XM_226713		0.44	2.77	0.003314
A_44_P323914	Sms	NM_001033899	363469	0.44	2.77	0.000187
A_44_P231143	Ppox_predicted	XM_213944		0.44	2.77	0.001897
A_44_P373236	Slfn8	NM_001013970	303378	0.44	2.77	0.00245
A_44_P226478	Mrlcb	X05566	50685	0.44	2.77	0.000249
A_43_P12657	Slc29a2	NM_031738	65194	0.44	2.77	0.000388
A_44_P794536	TC528250	TC528250		0.44	2.76	0.000738
A_44_P426307	S68987	S68987		0.44	2.76	6.26E-05
A_44_P238803	Kpna3	NM_001014792	361055	0.44	2.76	0.001761
A_44_P155932	Wdr6	BC084708	301007	0.44	2.76	0.008659
A_44_P172625	Dd25	NM_199403	360863	0.44	2.76	0.001864
A_44_P994364	Jag2	U70050	29147	0.44	2.76	4.81E-05
A_44_P930826	TC524089	TC524089		0.44	2.76	0.002025
A_42_P548791	Tsn	NM_021762	60381	0.44	2.76	3.14E-05
A_44_P363647	LOC312299	XM_231705	312299	0.44	2.76	0.004514
A_44_P332780	Ptprj	NM_017269	29645	0.44	2.76	2.14E-05
A_42_P644800	AW916183	AW916183		0.44	2.76	0.000795
A_44_P715425	TC558008	TC558008		0.44	2.75	0.009784
A_44_P256629	Cnbp1	NM_022598	64530	0.44	2.75	0.00042
A_43_P15565	Mox2r	NM_023953	64357	0.44	2.75	0.006398
A_44_P337900	RGD1306323_predicted	XM_232798	313085	0.44	2.75	0.02708
A_44_P368381	RGD1563347_predicted	XM_576510	501095	0.44	2.75	0.002388
A_44_P529972	Datf1_predicted	XM_342600		0.44	2.75	0.016868
A_44_P461849	RGD1311099_predicted	XM_226323		0.44	2.75	0.00682
A_44_P325534	Fut11	NM_173308	286971	0.44	2.75	0.000408
A_44_P176606	Itpr1	NM_001007235	25262	0.44	2.75	0.000546
A_44_P199161	RGD1561137_predicted	XM_345346		0.44	2.75	4.53E-05
A_44_P229766	U30813	U30813		0.44	2.75	0.00615
A_43_P10735	Fbxo21_predicted	XM_341091		0.44	2.74	0.000167
A_44_P1055795	Rg9mtd1	NM_001008337	304012	0.44	2.74	0.002194
A_42_P588041	Eif5a	NM_001033681	287444	0.44	2.74	3.14E-05
A_42_P677285	Rpl41	NM_139083	124440	0.44	2.74	0.000449
A_44_P166418	RGD1310271_predicted	XM_233727	313778	0.44	2.74	0.000058
A_44_P501483	LOC310385	XR_009527	310385	0.44	2.74	0.000111

A_44_P226941	Pln	NM_022707	64672	0.44	2.74	0.053655
A_44_P169124	BF558918	BF558918		0.44	2.74	0.011378
A_44_P485640	RGD1565459_predicted	XM_001081626		0.44	2.74	3.87E-05
A_44_P508901	LOC367902	NM_001014273	367902	0.44	2.74	0.049245
A_44_P1012748	Plscr3	NM_001012139	360549	0.44	2.74	0.003239
A_42_P799208	DV726678	DV726678		0.44	2.74	0.000317
A_44_P542762	XM_216997	XM_216997		0.44	2.74	2.76E-05
A_42_P821581	XM_236746	XM_236746		0.44	2.74	0.00264
A_44_P349539	Elmo2	XM_342579	362271	0.44	2.74	0.000448
A_42_P704967	Arid5a	NM_001034934	316327	0.44	2.73	0.000801
A_43_P21762	Slc25a30	NM_001013187	361074	0.44	2.73	9.98E-05
A_44_P438143	Nrg1	NM_031588	112400	0.44	2.73	0.002034
A_44_P480527	Wdr3_predicted	XM_227527		0.44	2.73	0.000205
A_42_P792649	RGD1311745	NM_001008329	301079	0.44	2.73	0.021216
A_42_P636651	Ppt1	NM_022502	29411	0.44	2.73	4.87E-05
A_42_P753820	Hmgn1	NM_001013184	360704	0.44	2.73	9.64E-05
A_43_P19578	RGD1560656_predicted	XM_574664		0.44	2.73	0.00027
A_44_P910533	LOC363333	XM_001061702		0.44	2.73	0.000172
A_44_P520535	Pkp4_predicted	XM_215733		0.44	2.73	0.000182
A_44_P336813	RGD1563123_predicted	XM_340943		0.44	2.73	0.005143
A_44_P778966	LOC500118	NM_001025771	500118	0.44	2.73	0.00735
A_44_P1020134	LOC499339	NM_001025036	499339	0.44	2.73	3.21E-05
A_44_P137721	Ttyh3_predicted	XM_221962		0.44	2.72	0.000125
A_44_P516038	RGD1359144	NM_001007705	314399	0.44	2.72	0.008975
A_44_P241230	A_44_P241230	A_44_P241230		0.44	2.72	4.78E-05
A_43_P12451	Slc16a3	NM_030834	80878	0.43	2.72	0.000272
A_44_P307208	XM_217089	XM_217089		0.43	2.72	0.001998
A_44_P761267	TC521072	TC521072		0.43	2.72	0.001183
A_43_P10466	LOC311078	XM_001059730	311078	0.43	2.72	0.000795
A_43_P12216	Pafah1b2	NM_022387	64189	0.43	2.72	0.000235
A_44_P686876	RGD1563715_predicted	XM_001080744		0.43	2.72	0.010062
A_44_P133344	RGD1304869_predicted	NM_001014153	361402	0.43	2.72	0.000129
A_44_P744443	RGD1564150_predicted	XM_573627		0.43	2.72	0.008566
A_42_P760177	LOC362587	XM_342906	362587	0.43	2.72	0.000107
A_44_P354431	RGD1309926_predicted	XM_001063612		0.43	2.72	0.000508
A_44_P595102	TC562246	TC562246		0.43	2.72	0.076682
A_44_P282514	AA859471	AA859471	64191	0.43	2.72	0.003112
A_44_P525827	Cct3	NM_199091	295230	0.43	2.72	0.003006
A_44_P414258	Ptprc	XM_001062978		0.43	2.71	0.000374
A_44_P438620	Herc3_predicted	XM_342701		0.43	2.71	0.002188
A_44_P290559	Pabpc4	XM_216517	298510	0.43	2.71	2.44E-05
A_44_P1035071	Nmt2	NM_207590	291318	0.43	2.71	0.000823
A_44_P560009	L42293	L42293		0.43	2.71	0.000968
A_44_P412427	RGD1311475_predicted	XM_001072042		0.43	2.71	0.000135
A_42_P651334	Rps19	NM_001037346	29287	0.43	2.71	0.000125
A_44_P776823	Syncrip	XM_001065902		0.43	2.71	0.023721
A_44_P532500	XM_217354	XM_217354		0.43	2.71	9.16E-05
A_44_P248598	LOC314859	NM_001017459	314859	0.43	2.71	0.000135
A_44_P1041539	Arhgap22_predicted	XM_224637		0.43	2.71	0.010363
A_44_P189476	Masp1	NM_022257	64023	0.43	2.71	0.029314
A_42_P543226	RGD1563365_predicted	XM_001079678		0.43	2.71	2.85E-05
A_44_P651845	ENSRNOT00000029007	ENSRNOT00000029007		0.43	2.70	0.000789
A_44_P868694	TC538548	TC538548		0.43	2.70	0.012221
A_44_P765452	TC550831	TC550831		0.43	2.70	0.009317
A_44_P299721	Rrm1_mapped	NM_001013236	365320	0.43	2.70	0.000186
A_44_P325772	NP516920	NP516920		0.43	2.70	0.000209
A_44_P176003	RGD1305117	NM_001013182	360492	0.43	2.70	0.003697
A_44_P166501	Rangap1	NM_001012199	362965	0.43	2.70	0.00012
A_44_P537303	Rpl4	NM_022510	64302	0.43	2.70	5.42E-05
A_44_P637047	A_44_P637047	A_44_P637047		0.43	2.70	0.000032
A_42_P573352	RGD1307618_predicted	XM_001070654		0.43	2.70	0.00012
A_43_P23158	LOC498922	XM_574210		0.43	2.70	0.000146
A_44_P330612	XM_346450	XM_346450		0.43	2.70	7.57E-05

A_44_P168396	CK478552	CK478552		0.43	2.70	0.000397
A_43_P15727	Lrp4	NM_031322	83469	0.43	2.70	0.001665
A_44_P761837	TC523524	TC523524		0.43	2.70	0.001294
A_44_P142594	XM_230719	XM_230719		0.43	2.70	7.82E-05
A_44_P519363	AW143857	AW143857		0.43	2.70	0.011913
A_44_P665677	BF566488	BF566488		0.43	2.69	0.045591
A_44_P638605	TC541235	TC541235		0.43	2.69	0.005531
A_44_P306238	BF287427	BF287427	499655	0.43	2.69	0.002842
A_44_P149552	Tead1	XM_001069320	361630	0.43	2.69	0.000303
A_44_P157078	RGD1305089_predicted	XM_234171		0.43	2.69	0.041669
A_44_P714927	TC539131	TC539131		0.43	2.69	0.000952
A_44_P396218	Mtfr1_predicted	XM_230569		0.43	2.69	0.002825
A_44_P531460	RGD1359191	NM_001007706	314462	0.43	2.69	8.74E-05
A_44_P280759	Npm1	NM_012992	25498	0.43	2.69	3.08E-05
A_44_P760780	Mcm3_predicted	XM_236988	316273	0.43	2.69	7.23E-05
A_43_P23014	Lpxn	NM_001009649	293783	0.43	2.69	0.000778
A_44_P916110	DV728510	DV728510		0.43	2.69	0.007259
A_44_P807296	TC521398	TC521398		0.43	2.69	0.010717
A_44_P288858	RGD1563888_predicted	XM_340963		0.43	2.69	0.000262
A_44_P976153	TC523614	TC523614		0.43	2.68	0.003352
A_44_P382435	LOC362587	XM_342906	362587	0.43	2.68	0.000261
A_44_P441773	Cdc37	NM_053743	114562	0.43	2.68	0.000221
A_44_P911683	AW144275	AW144275		0.43	2.68	0.001377
A_44_P246846	Wdsub1	NM_001014179	362137	0.43	2.68	0.005901
A_44_P312217	Rnf138	NM_053588	94196	0.43	2.68	0.00572
A_44_P173554	Sema7a_predicted	XM_243863	315711	0.43	2.68	0.000199
A_44_P1000888	Plac9_predicted	XM_341391		0.43	2.68	0.000971
A_44_P956272	BG673253	BG673253	26295	0.43	2.68	0.005999
A_44_P281297	LOC685210	XM_001062826	685210	0.43	2.68	0.001176
A_43_P13419	Ppp1r14b	NM_172045	259225	0.43	2.68	8.21E-06
A_44_P250267	Coil	NM_017360	50998	0.43	2.68	0.001383
A_44_P945404	TC555723	TC555723		0.43	2.68	8.61E-05
A_44_P269125	Taf9	NM_184048	373541	0.43	2.68	0.000188
A_44_P103225	Cd3e_predicted	XM_236196		0.43	2.67	0.002809
A_44_P435059	RGD1564407_predicted	XM_223440	305373	0.43	2.67	9.91E-05
A_44_P615982	A_44_P615982	A_44_P615982		0.43	2.67	0.01096
A_44_P211202	Pdcd1_predicted	XM_237422		0.43	2.67	0.005147
A_44_P262333	LOC257646	XM_001064688	257646	0.43	2.67	0.000278
A_44_P477785	Eef2	NM_017245	29565	0.43	2.67	0.000579
A_44_P302657	Sez6	XM_239260		0.43	2.67	0.011162
A_44_P456968	St5_predicted	XM_219273		0.43	2.67	0.006211
A_44_P883226	A_44_P883226	A_44_P883226		0.43	2.67	0.006053
A_44_P494632	Flnc_predicted	XM_342653	362332	0.43	2.67	0.032784
A_44_P112217	Lonrf1_predicted	XM_224907	306505	0.43	2.67	0.007972
A_43_P20631	RGD1307973_predicted	XM_222150	304469	0.43	2.67	0.001559
A_44_P522482	LOC297756	NM_001013928	297756	0.43	2.67	0.000178
A_44_P116685	XM_222149	XM_222149		0.43	2.67	0.001477
A_44_P151834	Tagap_predicted	XM_217791		0.43	2.67	0.031829
A_42_P456939	Slc35b4_predicted	XM_216122		0.43	2.67	0.002062
A_44_P606260	ENSRNOT00000039958	ENSRNOT00000039958		0.43	2.67	0.040253
A_44_P306185	Rpl19	NM_031103	81767	0.43	2.67	0.000175
A_44_P248162	RGD1310726_predicted	XM_223341	305288	0.43	2.67	0.001294
A_44_P946005	TC524212	TC524212		0.43	2.67	2.03E-05
A_44_P487313	RGD1559677_predicted	XM_344088		0.43	2.66	0.025711
A_43_P13378	Bmf	NM_139258	246142	0.43	2.66	0.000236
A_44_P466719	Six4_predicted	XM_234292		0.43	2.66	0.003366
A_44_P294675	Slfn3	NM_053687	114247	0.43	2.66	0.025096
A_42_P498066	Tmsb4x	NM_031136	81814	0.43	2.66	9.57E-05
A_44_P220455	Csf3r_predicted	XM_233514		0.43	2.66	0.023374
A_44_P335770	Stxbp4_predicted	XM_220860		0.43	2.66	0.011786
A_43_P16048	Sox5	XM_342784	140587	0.42	2.66	0.005904
A_44_P497033	Was_predicted	XM_228784		0.42	2.66	0.005093
A_44_P791005	ENSRNOT00000040102	ENSRNOT00000040102		0.42	2.66	0.031362

A_42_P604900	Cbx7	NM_199117	362962	0.42	2.66	0.018272
A_44_P518380	Pomgnt1	NM_001007747	362567	0.42	2.66	0.000154
A_44_P326523	RGD1310674	XM_001058775	303790	0.42	2.66	0.00131
A_44_P776231	Tp53i11_predicted	XM_230296		0.42	2.66	6.47E-05
A_44_P667768	A_44_P667768	A_44_P667768		0.42	2.66	0.000413
A_44_P1020708	Rpl26	XM_213346		0.42	2.66	0.00021
A_42_P766292	M6pr	NM_001007700	312689	0.42	2.66	0.000338
A_44_P1045652	LOC680266	XM_001057185	680266	0.42	2.65	0.000679
A_43_P17443	Rin2_predicted	XM_230647		0.42	2.65	8.54E-05
A_44_P231935	RGD1563603_predicted	XM_575211		0.42	2.65	0.00257
A_44_P605812	A_44_P605812	A_44_P605812		0.42	2.65	0.000199
A_44_P246049	Dna2l_predicted	XM_241671	309762	0.42	2.65	0.023583
A_43_P21658	Gpc4	NM_001014108	317322	0.42	2.65	0.000304
A_43_P17960	MGC72584	NM_001009535	494322	0.42	2.65	0.002442
A_44_P483297	Eftud2	XM_213492	287739	0.42	2.65	0.000259
A_44_P428158	AW143195	AW143195		0.42	2.65	0.018986
A_44_P416751	Rpa1	XM_213389		0.42	2.65	0.003277
A_44_P215253	LOC498131	XM_001062707		0.42	2.65	0.00278
A_44_P659391	XM_580163	XM_580163		0.42	2.65	0.001447
A_44_P931367	TC526331	TC526331		0.42	2.65	0.000977
A_44_P782990	RGD1305976_predicted	XM_343253	362923	0.42	2.65	0.002378
A_44_P555253	Dnaja1	NM_022934	65028	0.42	2.65	0.000145
A_44_P823916	CF975312	CF975312		0.42	2.65	0.005094
A_44_P121397	Phf10	NM_001024747	292404	0.42	2.65	0.000699
A_44_P188880	AW915747	AW915747		0.42	2.65	0.006034
A_44_P680369	BF545930	BF545930		0.42	2.65	0.000968
A_44_P961724	TC526460	TC526460		0.42	2.64	0.002048
A_44_P399992	Prkar1a	NM_013181	25725	0.42	2.64	1.82E-05
A_44_P387182	LOC366468	XM_345581		0.42	2.64	0.001788
A_44_P591495	RGD1560170_predicted	XM_001056908		0.42	2.64	0.000451
A_44_P714743	TC538340	TC538340		0.42	2.64	6.83E-05
A_44_P1024125	Hsf2	NM_031694	64441	0.42	2.64	0.020643
A_44_P276338	Mcm6	XM_344135		0.42	2.64	0.000775
A_44_P376905	Spcs2_predicted	XM_214994	293142	0.42	2.64	0.000528
A_42_P569063	LOC361237	NM_001014145	361237	0.42	2.64	0.000365
A_43_P23262	Pscdbp	NM_001012086	311047	0.42	2.64	0.000326
A_44_P554679	Mtss1_predicted	XM_001064860		0.42	2.64	0.016016
A_44_P173320	RGD1561919_predicted	XM_226269		0.42	2.64	7.52E-05
A_44_P459492	Lmo2	NM_001037358	362176	0.42	2.63	0.000809
A_44_P267365	Ddx20	XM_227558	84473	0.42	2.63	0.0067
A_42_P772356	Dhx15_predicted	XM_214053	289693	0.42	2.63	0.000333
A_44_P515275	Cdkn1a	NM_080782	114851	0.42	2.63	0.000161
A_44_P273821	Lyn	NM_030857	81515	0.42	2.63	7.92E-05
A_44_P229604	LOC691499	XM_001078547	691499	0.42	2.63	0.045483
A_43_P18947	Gmps	NM_001024754	295088	0.42	2.63	3.23E-05
A_44_P329357	Hpn	NM_017112	29135	0.42	2.63	0.001187
A_44_P480954	RGD1304598_predicted	XM_220745		0.42	2.63	0.002305
A_44_P433579	RGD1305287	NM_001009633	289468	0.42	2.63	0.029959
A_44_P459105	AF020778	AF020778		0.42	2.63	0.015298
A_44_P154773	LOC498050	XR_008646	498050	0.42	2.63	0.000567
A_44_P433259	Sipa1	NM_001004089	361710	0.42	2.63	0.000118
A_44_P509588	Zfp692_predicted	XM_001075322		0.42	2.63	0.032596
A_42_P826171	LOC684993	XM_001061833		0.42	2.63	0.000388
A_44_P401809	Rps20	NM_001007603	122772	0.42	2.63	4.33E-05
A_44_P950367	A_44_P950367	A_44_P950367		0.42	2.63	0.042249
A_44_P1029294	XM_342367	XM_342367		0.42	2.62	0.001881
A_44_P356236	Bclaf1	XM_214967		0.42	2.62	0.03415
A_44_P217541	Tmem49	NM_138839	192129	0.42	2.62	2.14E-05
A_44_P604435	AI228698	AI228698		0.42	2.62	0.009964
A_42_P589201	Slc15a4	NM_144758	246280	0.42	2.62	0.000267
A_44_P1040889	Cpsf6_predicted	XM_216893		0.42	2.62	0.010961
A_44_P260181	Prss23	NM_001007691	308807	0.42	2.62	0.023758
A_43_P19632	Spred1	XM_230454		0.42	2.62	0.000539

A_43_P18497	Mfap3	NM_001007609	287299	0.42	2.62	0.002368
A_44_P948401	TC543833	TC543833		0.42	2.62	0.024614
A_44_P228313	Mrpl35_predicted	XM_216169		0.42	2.62	0.000412
A_42_P511187	Hgfac	NM_053320	58947	0.42	2.62	0.002218
A_44_P492888	Enc1	NM_001003401	294674	0.42	2.62	0.000953
A_44_P216292	BF523623	BF523623		0.42	2.62	2.73E-05
A_44_P638230	TC523607	TC523607		0.42	2.62	0.02284
A_44_P323106	Abca1	NM_178095	313210	0.42	2.62	7.75E-05
A_44_P384628	XM_226259	XM_226259		0.42	2.62	0.000101
A_44_P407707	Nufip1	NM_001007758	364430	0.42	2.62	0.001058
A_44_P606843	LOC310946	XM_227795		0.42	2.61	0.001825
A_44_P227457	Epha2_predicted	XM_345596		0.42	2.61	0.000109
A_44_P700187	TC557501	TC557501		0.42	2.61	0.000104
A_42_P816645	Gnb4	AF022085	294962	0.42	2.61	0.012158
A_44_P251673	Rbm14	XM_001072105		0.42	2.61	0.001235
A_44_P463709	Il1r1	NM_013123	25663	0.42	2.61	0.005522
A_44_P942846	RGD1306346_predicted	XM_001073045		0.42	2.61	0.017478
A_44_P1000232	Sh3bgrl3_predicted	XM_216547		0.42	2.61	7.71E-05
A_44_P428507	Slc35b2	NM_199111	316241	0.42	2.61	0.000123
A_44_P853167	TC520251	TC520251		0.42	2.61	0.002705
A_42_P724600	Tars	NM_001006976	294810	0.42	2.61	0.001582
A_44_P196347	XM_214948	XM_214948		0.42	2.61	0.032898
A_44_P152020	LOC681545	XR_009539	680895	0.42	2.61	0.000559
A_42_P658005	R3hdm1	XM_573442	304763	0.42	2.61	0.001743
A_44_P937098	TC557723	TC557723		0.42	2.61	0.006877
A_44_P396040	Tmem2_predicted	XM_219898		0.42	2.61	0.000137
A_44_P163131	Zfp307	NM_001012053	306977	0.42	2.61	0.031937
A_44_P309403	RGD1562171_predicted	XM_225692		0.42	2.61	3.02E-05
A_44_P100886	RGD1311723_predicted	XM_343351	363018	0.42	2.61	0.015602
A_44_P386164	LOC294446	XM_001061084		0.42	2.61	5.67E-05
A_44_P182555	Mapre1	NM_138509	114764	0.42	2.61	0.000326
A_44_P294711	Slc11a2	NM_013173	25715	0.42	2.60	0.000859
A_44_P553017	XM_214203	XM_214203		0.42	2.60	0.000136
A_44_P1024773	RGD1561749_predicted	XM_216934	299933	0.42	2.60	0.004768
A_44_P120002	Gtf2i	NM_001001512	353256	0.42	2.60	2.77E-05
A_44_P165755	XM_218031	XM_218031		0.42	2.60	0.00724
A_44_P304579	XM_224191	XM_224191		0.42	2.60	0.000132
A_44_P295051	Tmem9_predicted	XM_213883		0.42	2.60	0.000954
A_44_P143974	Scoc	NM_001013235	364981	0.42	2.60	0.000317
A_44_P287250	Oat	NM_022521	64313	0.42	2.60	0.000525
A_44_P868944	TC521816	TC521816		0.41	2.60	0.007206
A_44_P208464	Rad17	NM_001024778	310034	0.41	2.60	0.005195
A_44_P543473	LOC686781	XM_001075705		0.41	2.60	0.000307
A_44_P176063	XM_221479	XM_221479		0.41	2.60	0.000368
A_42_P719131	TC542981	TC542981		0.41	2.60	0.007488
A_44_P234636	Psmd2	NM_001031639	287984	0.41	2.60	0.000277
A_43_P10490	Zc3h7b_predicted	XM_243630	315158	0.41	2.60	9.88E-05
A_44_P309688	RGD1562399_predicted	XM_217412		0.41	2.60	8.35E-05
A_43_P21509	Rnasen	XM_226843		0.41	2.60	0.000995
A_44_P253504	RGD1306500_predicted	XM_001081783		0.41	2.59	0.015044
A_42_P462987	LOC683383	XM_001063050		0.41	2.59	0.000577
A_43_P12832	Nol3	NM_053516	85383	0.41	2.59	0.147323
A_44_P519834	Ptpn1	NM_012637	24697	0.41	2.59	2.24E-05
A_44_P212695	RGD1565183_predicted	XM_212890		0.41	2.59	7.39E-05
A_42_P623913	Sox18	NM_001024781	311723	0.41	2.59	0.003458
A_44_P459391	XM_214276	XM_214276		0.41	2.59	8.49E-05
A_43_P21158	Emilin2_predicted	XM_237520	316736	0.41	2.59	0.014282
A_43_P13146	Atp2c1	NM_131907	170699	0.41	2.59	0.001282
A_44_P1021853	BF289184	BF289184	313582	0.41	2.59	0.000222
A_44_P452405	RGD1305211_predicted	XM_344924		0.41	2.59	0.002201
A_44_P250579	XM_214188	XM_214188		0.41	2.59	0.001121
A_44_P418824	Fen1	NM_053430	84490	0.41	2.59	0.000679
A_43_P11048	AI102821	AI102821		0.41	2.59	0.000687

A_44_P163903	Rp2h_predicted	XM_346266		0.41	2.59	0.000564
A_44_P898168	RGD1560190_predicted	XM_573512	364049	0.41	2.59	0.030999
A_44_P1030298	RGD1307435_predicted	XM_213757	288606	0.41	2.59	0.001676
A_42_P623688	Gatm	NM_031031	81660	0.41	2.59	0.000126
A_44_P445354	Kcnh8	NM_145095	246325	0.41	2.59	0.006836
A_42_P767698	Ebna1bp2	NM_001008721	114021	0.41	2.58	0.000191
A_42_P749449	Nckap1l_predicted	XM_235709		0.41	2.58	0.003655
A_44_P260598	Tmem123	NM_001014205	363013	0.41	2.58	0.000104
A_44_P151566	Olr517_predicted	NM_001000317	295771	0.41	2.58	0.009903
A_43_P12015	Flt1	NM_019306	54251	0.41	2.58	0.00409
A_44_P641262	LOC499709	NM_001024306	499709	0.41	2.58	6.66E-05
A_43_P13102	Ada	NM_130399	24165	0.41	2.58	0.000933
A_44_P476180	LOC287551	XR_009402	287551	0.41	2.58	0.000131
A_44_P142790	RGD1310357_predicted	XM_236104	315579	0.41	2.58	0.000626
A_43_P12070	Ly6c	NM_020103	56778	0.41	2.58	0.008461
A_44_P245391	Eif2s1	NM_019356	54318	0.41	2.58	0.000299
A_43_P17016	Unc93b1	XM_341972		0.41	2.58	0.000277
A_43_P18723	RGD1305984	NM_001034093	365668	0.41	2.58	0.001279
A_44_P362380	XM_575502	XM_575502		0.41	2.58	0.021753
A_44_P997174	Atrx	XM_217570		0.41	2.58	0.029516
A_44_P283372	Hnrpa1	BC062235	29578	0.41	2.57	9.62E-05
A_44_P248291	Prmt6_predicted	XM_227607		0.41	2.57	0.000226
A_44_P640828	Tgfbr1	NM_012775	29591	0.41	2.57	0.000294
A_43_P18996	Plagl2_predicted	XM_230745		0.41	2.57	0.008322
A_44_P246169	RGD1560519_predicted	XM_233829	313865	0.41	2.57	0.011588
A_42_P634993	Ppil1	NM_001034188	309651	0.41	2.57	0.000197
A_43_P11193	Sdc2	NM_013082	25615	0.41	2.57	0.040005
A_44_P1031534	Rpl39	NM_012875	25347	0.41	2.57	2.22E-05
A_44_P429450	RGD1561440_predicted	XM_344122		0.41	2.57	0.00926
A_44_P476733	Nqo1	NM_017000	24314	0.41	2.57	0.000145
A_44_P950925	A_44_P950925	A_44_P950925		0.41	2.57	0.001428
A_43_P21980	Gnat2_predicted	XM_345271		0.41	2.57	0.005811
A_44_P960517	TC521962	TC521962		0.41	2.57	0.00014
A_44_P518160	Sass6_predicted	XM_227619		0.41	2.57	0.010725
A_44_P1018865	RGD1560198_predicted	XM_217622		0.41	2.57	0.000118
A_44_P482845	Wdfy2_predicted	XM_224296		0.41	2.57	0.00509
A_42_P714075	Sec11l1	NM_031723	65166	0.41	2.57	0.00071
A_44_P260134	Srcrb4d_predicted	XM_222076	304401	0.41	2.57	0.00103
A_44_P506069	Atp2c1	NM_131907	170699	0.41	2.57	0.0005
A_44_P161868	BF542413	BF542413	360692	0.41	2.57	0.013663
A_44_P742962	AI008629	AI008629		0.41	2.57	0.012339
A_44_P959251	LOC682161	XM_001060186		0.41	2.57	0.001971
A_44_P166372	LOC680736	XR_005855	680736	0.41	2.57	0.048216
A_44_P807437	Acrbp	NM_001025049	500316	0.41	2.57	0.000618
A_44_P107495	RGD1563977_predicted	XM_220117	309557	0.41	2.56	0.000128
A_44_P160943	Zfyve27	NM_199104	309376	0.41	2.56	0.000589
A_44_P309979	BF551342	BF551342		0.41	2.56	0.045118
A_44_P102857	Tnfrsf10b_predicted	XM_344431		0.41	2.56	0.000154
A_44_P265374	RGD1308469_predicted	XM_228615	317275	0.41	2.56	7.76E-05
A_42_P467928	Rnf138	NM_053588	94196	0.41	2.56	0.000419
A_44_P399146	Nras	NM_080766	24605	0.41	2.56	0.000466
A_44_P223435	Kif21a_predicted	XM_001056804		0.41	2.56	0.032413
A_44_P557680	Cybasc3	NM_001014164	361729	0.41	2.56	0.000735
A_42_P499472	Cfdp1	NM_199378	292027	0.41	2.56	6.08E-05
A_44_P438718	Stil_predicted	XM_233429	313506	0.41	2.56	0.000847
A_44_P245775	RGD1305014	NM_001014025	309029	0.41	2.56	0.037489
A_44_P360946	RGD1562134_predicted	XM_223094		0.41	2.56	5.74E-05
A_44_P626817	RGD1560656_predicted	XM_574664		0.41	2.56	0.001069
A_44_P392611	BP483835	BP483835		0.41	2.56	0.007439
A_42_P583690	Eif2b4	NM_053950	117019	0.41	2.56	0.001333
A_44_P536315	Rab12	XM_343639		0.41	2.56	0.000304
A_44_P356855	Dcakd	NM_001007724	360639	0.41	2.55	6.56E-05
A_44_P931186	RGD1561597_predicted	XM_575215	499874	0.41	2.55	0.054362

A_44_P290703	XM_226680	XM_226680		0.41	2.55	0.001554
A_44_P499401	RGD1563037_predicted	XM_219837	309357	0.41	2.55	0.000214
A_44_P1053951	Ier5l	XM_001079588		0.41	2.55	0.028324
A_44_P286158	RGD1564574_predicted	XM_226020		0.41	2.55	1.74E-05
A_44_P725710	Zbtb2_predicted	XM_001065469		0.41	2.55	0.058874
A_44_P991682	Pdk3_mapped	XM_216091		0.41	2.55	0.000708
A_44_P175299	M33313	M33313		0.41	2.55	0.00068
A_44_P557731	RGD1359361	NM_001013996	306000	0.41	2.55	0.001256
A_44_P944957	Cggbp1_predicted	XM_001063614		0.41	2.55	0.001973
A_44_P121244	XM_234676	XM_234676		0.41	2.55	0.09064
A_44_P130894	XM_212948	XM_212948		0.41	2.55	0.000191
A_44_P548985	Tgfbi	XM_573983	116487	0.41	2.55	0.000743
A_44_P347253	Zc3h12a_predicted	XM_233517		0.41	2.55	0.000338
A_44_P187056	Itga6	XM_215984	114517	0.41	2.55	7.62E-05
A_42_P542672	AW918541	AW918541		0.41	2.55	0.027103
A_44_P534211	Adss_predicted	XM_222946		0.41	2.55	0.000716
A_44_P419882	RGD1560617_predicted	XM_238518		0.41	2.55	0.005051
A_44_P456716	Ttn	U89530	84015	0.41	2.55	0.008477
A_43_P11514	Tpm4	NM_012678	24852	0.41	2.55	0.000422
A_44_P194821	RGD1562949_predicted	XM_236578	315969	0.41	2.54	0.008109
A_44_P668582	RGD1562105_predicted	XM_001070699		0.41	2.54	0.001323
A_44_P391814	Tmem69	NM_001035001	619582	0.41	2.54	0.020438
A_44_P896243	AW143242	AW143242		0.41	2.54	0.001314
A_44_P104928	Gucy2g	NM_139042	245708	0.41	2.54	0.031856
A_44_P372261	Slpi	NM_053372	84386	0.40	2.54	0.003414
A_44_P536400	XM_342024	XM_342024		0.40	2.54	0.000241
A_44_P112981	Mybbp1a	NM_031668	60571	0.40	2.54	5.62E-05
A_44_P681642	CF106938	CF106938	302697	0.40	2.54	0.000746
A_44_P345561	Ipo9_predicted	XM_222661		0.40	2.54	0.000148
A_44_P263669	Syncrip	XM_343446		0.40	2.54	8.92E-05
A_42_P756876	Padi1	NM_019332	54282	0.40	2.54	0.002802
A_44_P552849	Ctsh	NM_012939	25425	0.40	2.54	0.000432
A_44_P132815	Mbnl1	XM_001062557		0.40	2.54	0.000182
A_44_P996729	Mmp2	U65656	81686	0.40	2.53	0.000648
A_44_P790281	AW915160	AW915160		0.40	2.53	3.88E-05
A_44_P178108	RGD1566097_predicted	XM_225479	307056	0.40	2.53	0.000757
A_43_P21963	LOC307347	XM_225856		0.40	2.53	0.005375
A_44_P471070	LOC686132	XM_001067579		0.40	2.53	0.00131
A_44_P536588	XM_344248	XM_344248		0.40	2.53	9.01E-05
A_44_P499056	Paqr3	NM_001012033	305203	0.40	2.53	0.018805
A_42_P625263	Epb4.1l4a_predicted	XM_226060		0.40	2.53	0.004846
A_44_P461187	St3gal4	NM_203337	363040	0.40	2.53	0.000484
A_44_P309489	Ddx21a	NM_001037201	317399	0.40	2.53	0.000146
A_44_P348685	Atxn10	NM_133313	170821	0.40	2.53	6.76E-05
A_44_P328079	Recc1	XM_214035	89809	0.40	2.53	0.003088
A_44_P191309	Lpin1	XM_576006		0.40	2.53	0.09304
A_44_P409977	Wee1	NM_001012742	308937	0.40	2.53	0.00521
A_44_P430803	Yif1b	NM_198734	292768	0.40	2.53	0.000805
A_44_P287286	XM_216910	XM_216910		0.40	2.53	0.000107
A_44_P184524	Rab28	NM_053978	117049	0.40	2.53	0.007361
A_44_P395771	Tie1	XM_233462	89806	0.40	2.52	0.000799
A_44_P344119	Cd80	NM_012926	25408	0.40	2.52	0.005207
A_44_P594080	TC566600	TC566600		0.40	2.52	0.009753
A_44_P189413	Slc22a19	XM_342011	286961	0.40	2.52	0.021335
A_44_P180865	AI179448	AI179448		0.40	2.52	6.21E-05
A_44_P467198	E2f7_predicted	XM_235118		0.40	2.52	0.002086
A_44_P290786	RGD1559637_predicted	XM_001076757		0.40	2.52	0.005979
A_42_P801831	Fbln1_predicted	XM_243637	315191	0.40	2.52	0.010246
A_44_P363090	Il6st	NM_001008725	25205	0.40	2.52	0.000205
A_42_P724142	Mphosph10_predicted	XM_238166		0.40	2.52	0.000157
A_43_P16964	Ppfibp1_predicted	XM_232536		0.40	2.52	0.000092
A_44_P729134	A_44_P729134	A_44_P729134		0.40	2.52	0.000305
A_44_P971189	LOC679028	XM_001053835		0.40	2.52	0.000288

A_44_P529366	Gspt1	NM_001003978	24420	0.40	2.52	0.002069
A_44_P776411	Recc1	XM_001078203		0.40	2.52	0.006199
A_44_P128932	RGD1309077_predicted	XM_340845		0.40	2.52	0.000107
A_44_P158880	Pfkfb3	NM_057135	117276	0.40	2.52	0.003535
A_44_P134070	Txnrd1	NM_031614	58819	0.40	2.52	0.000348
A_44_P215302	Ube2t_predicted	XM_341124		0.40	2.52	0.05955
A_44_P840829	Hnrpr	NM_175603	319110	0.40	2.52	0.003469
A_44_P351696	A_44_P351696	A_44_P351696		0.40	2.52	0.000182
A_44_P225204	Reep6	NM_001013218	362835	0.40	2.52	0.009842
A_44_P279262	XM_236342	XM_236342		0.40	2.52	0.002406
A_44_P397495	Hif1a	NM_024359	29560	0.40	2.52	0.001467
A_44_P131187	XM_343417	XM_343417		0.40	2.51	0.000712
A_44_P382727	RGD1566215_predicted	XM_217496		0.40	2.51	0.003732
A_44_P248083	XM_221978	XM_221978		0.40	2.51	0.000218
A_44_P1007438	Gdpd1_predicted	XM_220818		0.40	2.51	0.002319
A_44_P504374	RGD1311526_predicted	XM_213927	289159	0.40	2.51	4.52E-05
A_44_P576289	BC088330	BC088330	311622	0.40	2.51	0.006239
A_44_P185525	LOC360713	XM_001066275		0.40	2.51	0.001971
A_44_P475997	LOC315910	XM_236532		0.40	2.51	0.010619
A_43_P12778	Gtf2a2	NM_053345	83828	0.40	2.51	0.000326
A_44_P307172	Eif4a1	NM_199372	287436	0.40	2.51	0.00013
A_44_P283819	Dapk1_predicted	XM_225138		0.40	2.51	0.000197
A_42_P591374	Ddx10_predicted	XM_236263		0.40	2.51	0.000162
A_44_P508348	Ahnak	XM_574618	191572	0.40	2.51	0.000679
A_44_P557154	Nid67	NM_173126	286910	0.40	2.51	0.001199
A_42_P785861	Nipsnap3a	NM_001009422	313211	0.40	2.51	7.75E-05
A_44_P123315	Gpx1	NM_030826	24404	0.40	2.50	0.001194
A_42_P501972	Ppp2r1b	NM_001025418	315648	0.40	2.50	0.000814
A_44_P922150	L22655	L22655	502792	0.40	2.50	0.056668
A_44_P822160	BF562779	BF562779		0.40	2.50	0.076174
A_43_P13189	Ppp1r14c	NM_133425	171010	0.40	2.50	0.000292
A_43_P13274	Lbr	NM_134453	89789	0.40	2.50	0.000926
A_42_P766275	Set	NM_194353	290432	0.40	2.50	0.00017
A_44_P191672	Ka17	NM_212545	287702	0.40	2.50	0.009771
A_44_P131261	Usp22_predicted	XM_220534	303201	0.40	2.50	0.000183
A_44_P445874	XM_343411	XM_343411		0.40	2.50	0.00024
A_44_P559348	Eif3s9	NM_001031640	288516	0.40	2.50	0.000065
A_44_P624335	TC563074	TC563074		0.40	2.50	0.000157
A_44_P330675	Ubtd1	NM_001013153	309373	0.40	2.50	0.000122
A_44_P243238	Got2	NM_013177	25721	0.40	2.50	0.000782
A_44_P457169	Dcp2_predicted	XM_225963		0.40	2.49	0.032462
A_44_P272417	XM_220317	XM_220317		0.40	2.49	0.003832
A_44_P807146	TC520148	TC520148		0.40	2.49	0.053653
A_44_P459359	LOC361781	XM_342077	361781	0.40	2.49	0.008483
A_44_P302383	XM_212720	XM_212720		0.40	2.49	3.15E-05
A_44_P255965	A_44_P255965	A_44_P255965		0.40	2.49	0.000453
A_44_P618177	CF110664	CF110664		0.40	2.49	0.000935
A_44_P311693	A_44_P311693	A_44_P311693		0.40	2.49	5.21E-05
A_44_P473515	Praf1_predicted	XM_233004		0.40	2.49	0.000845
A_44_P342092	Rps11	NM_031110	81774	0.40	2.49	0.000243
A_44_P248562	XM_235784	XM_235784		0.40	2.49	0.000329
A_42_P695017	Nufip1	NM_001007758	364430	0.40	2.49	0.000363
A_42_P775268	MGC94720	NM_001006974	293738	0.40	2.49	0.000065
A_44_P485694	Hnrpa1	NM_017248	29578	0.40	2.49	0.000109
A_44_P128537	RGD1311681	NM_001025029	364674	0.40	2.49	0.005531
A_44_P473648	XM_213132	XM_213132		0.40	2.49	0.000407
A_43_P21166	Ptprm	XM_343640	29616	0.40	2.49	0.006605
A_44_P1004790	Slc30a2	BC061997	25362	0.40	2.49	0.005288
A_42_P810736	Timeless	NM_031340	83508	0.40	2.49	0.02759
A_44_P652332	LOC362557	NM_001025023	362557	0.40	2.49	0.000279
A_44_P391680	Tnpo2_predicted	XM_222478		0.40	2.49	0.000685
A_42_P841762	Cacna2d1	NM_012919	25399	0.40	2.48	0.002578
A_44_P321918	Gramd1a	NM_001014160	361550	0.40	2.48	0.000564

A_44_P265333	RGD1304623_predicted	XM_236385		0.40	2.48	8.05E-05
A_44_P178117	RGD1565767_predicted	XM_230013		0.40	2.48	0.000103
A_44_P405847	RGD1564168_predicted	XM_221056		0.40	2.48	0.004332
A_44_P288401	RGD1559887_predicted	XM_226929		0.39	2.48	0.059403
A_44_P370213	Ncl	NM_012749	25135	0.39	2.48	4.13E-05
A_44_P703840	TC548631	TC548631		0.39	2.48	0.005224
A_44_P442371	Hspbp1	NM_139261	246146	0.39	2.48	0.000104
A_43_P12800	Gnpat	NM_053410	84470	0.39	2.48	0.000315
A_44_P945616	Wnt5b	XM_001057561		0.39	2.48	0.000984
A_44_P203401	RatNP-3b	XM_001074118		0.39	2.48	0.064349
A_44_P988451	RGD1566119_predicted	XM_576399		0.39	2.48	7.52E-05
A_44_P1026631	LOC498549	XM_573823	498549	0.39	2.48	0.000481
A_44_P397460	LOC679114	XM_001054754		0.39	2.47	0.009793
A_44_P407200	Slc16a2	NM_147216	259248	0.39	2.47	0.007229
A_42_P703664	Srxn1	XM_215887		0.39	2.47	0.006897
A_44_P237220	Ttc13	XM_214720	292095	0.39	2.47	0.00185
A_44_P428824	A_44_P428824	A_44_P428824		0.39	2.47	0.003574
A_44_P622172	Epn2	NM_001033914	60443	0.39	2.47	0.000152
A_44_P1020615	Ifi44	XM_227821		0.39	2.47	0.016731
A_43_P20651	CB545955	CB545955	315705	0.39	2.47	0.004621
A_44_P450737	Srpx	NM_022524	64316	0.39	2.47	0.010511
A_44_P574994	LOC499331	NM_001024293	499331	0.39	2.47	0.021238
A_43_P11838	Csrp1	NM_017148	29276	0.39	2.47	0.000172
A_44_P353355	RGD1562047_predicted	XM_573992	498709	0.39	2.47	0.012568
A_43_P16005	Prkcn	XM_001054495	313834	0.39	2.47	0.003074
A_44_P520748	Cad_mapped	XM_343027		0.39	2.47	0.000297
A_44_P306964	Rbms1	NM_001012184	362138	0.39	2.47	0.000287
A_44_P239212	RGD1359634	NM_001007708	315126	0.39	2.47	0.000623
A_44_P546537	Nucb2	NM_021663	59295	0.39	2.47	0.000278
A_44_P284200	Usp18	NM_001014058	312688	0.39	2.47	0.008073
A_44_P257467	RGD1311260	NM_001013967	303211	0.39	2.47	0.006324
A_44_P371952	AW143923	AW143923		0.39	2.47	0.001086
A_44_P1015355	XM_214547	XM_214547		0.39	2.47	0.003094
A_44_P1020608	Ifi44	XM_227821		0.39	2.47	0.015229
A_44_P102343	Gtpbp4	NM_053689	114300	0.39	2.46	0.000318
A_44_P289627	Gria2	NM_017261	29627	0.39	2.46	0.05547
A_44_P257893	Gbp4_predicted	XM_227762	310917	0.39	2.46	0.019955
A_44_P419746	LOC363326	XM_343664	363326	0.39	2.46	0.000913
A_44_P178137	Dll4_predicted	XM_230472		0.39	2.46	0.001171
A_44_P309800	Loh11cr2a	NM_198755	301097	0.39	2.46	0.004398
A_44_P608425	TC544355	TC544355		0.39	2.46	8.94E-05
A_44_P792124	TC522828	TC522828		0.39	2.46	0.005785
A_44_P335539	RGD1566061_predicted	XM_346327		0.39	2.46	0.002895
A_44_P974956	A_44_P974956	A_44_P974956		0.39	2.46	0.000107
A_44_P496685	Znf324_predicted	XM_001055374		0.39	2.46	0.016268
A_44_P151744	Hip1r	XM_001072438	81917	0.39	2.46	0.000337
A_43_P23316	Rad18_predicted	XM_342734		0.39	2.46	0.000865
A_44_P853916	TC541303	TC541303		0.39	2.46	0.000731
A_44_P1011664	Ctnnal1_predicted	XM_216386		0.39	2.46	0.044396
A_44_P279460	BF406820	BF406820		0.39	2.46	0.000227
A_44_P435822	LOC317165	XM_228401		0.39	2.46	7.25E-05
A_44_P345013	BC098807	BC098807	363024	0.39	2.46	0.000584
A_44_P1010816	RGD1310784_predicted	XM_214527		0.39	2.46	0.005325
A_44_P596646	RGD1563612_predicted	XM_575380	500026	0.39	2.46	0.012747
A_44_P807917	TC540831	TC540831		0.39	2.46	0.015344
A_44_P426407	Hist1h2bn_predicted	XM_214483		0.39	2.46	0.045633
A_44_P239095	RGD1564407_predicted	XM_223440	305373	0.39	2.46	5.49E-05
A_44_P335636	Traf3_predicted	XM_343113		0.39	2.46	0.002423
A_44_P724217	RGD1564263_predicted	XM_575214	499873	0.39	2.46	0.064355
A_44_P132754	MGC72957	NM_212510	290641	0.39	2.46	6.04E-05
A_44_P123677	Syngr1	NM_019166	29205	0.39	2.46	0.005454
A_44_P898880	TC539546	TC539546		0.39	2.46	0.022212
A_43_P21845	RGD1310039	NM_001014165	361747	0.39	2.45	0.014295

A_44_P207881	Asf1a_predicted	XM_001059946		0.39	2.45	0.068276
A_44_P365653	LOC361596	NM_001014161	361596	0.39	2.45	0.00025
A_44_P518311	Rbpsuh_predicted	XM_232595		0.39	2.45	0.009893
A_42_P632498	LOC361420	NM_001014155	361420	0.39	2.45	0.000117
A_44_P377245	Miz1	NM_053337	83422	0.39	2.45	0.004076
A_44_P139894	Fgfr2	XM_341940		0.39	2.45	0.035615
A_44_P100500	Numa1	XM_218972	308870	0.39	2.45	0.000785
A_44_P975830	Wdr41_predicted	XM_001067353		0.39	2.45	0.016365
A_44_P430630	Zfp216_predicted	XM_215251		0.39	2.45	0.00131
A_44_P524846	Sars1	NM_001007606	266975	0.39	2.45	0.001304
A_42_P468216	Ifi30	NM_001030026	290644	0.39	2.45	3.04E-05
A_43_P18721	LOC679712	XM_001054154		0.39	2.45	0.000589
A_44_P217909	Mtmr1_predicted	XM_228644	317296	0.39	2.45	0.002068
A_44_P1015178	RGD1562502_predicted	XM_343808		0.39	2.45	7.87E-05
A_44_P442161	Ppp2r4_predicted	XM_342405		0.39	2.45	0.000679
A_44_P218147	Il1rl1l	NM_001013432	315461	0.39	2.45	0.000347
A_42_P722870	AW141147	AW141147		0.39	2.45	3.13E-05
A_43_P21973	Mbnl1	XM_001062557		0.39	2.45	0.000405
A_44_P377887	Krt1-4	NM_001008758	303528	0.39	2.45	0.007257
A_44_P139925	Fkbp4	XM_342763	260321	0.39	2.45	0.001209
A_44_P1053947	Slc5a6	NM_130746	170551	0.39	2.45	0.000464
A_44_P960215	TC537126	TC537126		0.39	2.44	0.000309
A_44_P121823	RGD1306596	NM_001029923	362595	0.39	2.44	0.001196
A_43_P20129	Pcsk9	NM_199253	298296	0.39	2.44	0.001311
A_44_P959890	AABR03000060	AABR03000060		0.39	2.44	0.001046
A_42_P798049	Scamp3	XM_342279	65169	0.39	2.44	0.001787
A_44_P324009	Brms1l_predicted	XM_216712		0.39	2.44	0.012519
A_44_P555389	Tmem49	NM_138839	192129	0.39	2.44	0.004401
A_44_P491308	Cd44	NM_012924	25406	0.39	2.44	0.002158
A_44_P326147	LOC307495	XR_005580	307495	0.39	2.44	0.000651
A_44_P263110	Sox9	XM_343981	140586	0.39	2.44	0.000144
A_44_P823250	TC522919	TC522919		0.39	2.44	0.048812
A_42_P516941	Ube4b_predicted	XM_233679	298652	0.39	2.44	0.07515
A_44_P464371	Dpy19l1_predicted	XM_235970	315496	0.39	2.44	0.000148
A_44_P379830	Inpp5f_predicted	XM_219372		0.39	2.44	0.003843
A_44_P925373	AA997357	AA997357		0.39	2.44	0.006511
A_44_P257931	XM_341575	XM_341575		0.39	2.44	0.027323
A_44_P495611	Wdfy3_predicted	XM_001060403		0.39	2.44	0.070052
A_44_P223446	Mcm2_predicted	XM_232168		0.39	2.44	0.000168
A_44_P301695	Prkar1a	NM_013181	25725	0.39	2.44	0.000528
A_44_P869086	Npat_predicted	XM_001072137		0.39	2.44	0.022932
A_44_P652585	RGD1562975_predicted	XM_575936	500569	0.39	2.44	0.00012
A_43_P17671	LOC362304	NM_001014186	362304	0.39	2.44	0.00133
A_42_P814410	Gnl1	NM_212500	309593	0.39	2.43	0.000474
A_44_P853326	AI059146	AI059146		0.39	2.43	0.000735
A_43_P15621	Rpl26	XM_213346		0.39	2.43	0.00017
A_43_P12416	Ak1	NM_024349	24183	0.39	2.43	0.062024
A_44_P232642	Usp14	NM_001008301	291796	0.39	2.43	0.000555
A_42_P704824	Cirh1a	NM_001009640	291987	0.39	2.43	0.000186
A_44_P1051678	Rasal2_predicted	XM_222780		0.39	2.43	0.007947
A_44_P576844	TC556918	TC556918		0.39	2.43	0.008668
A_42_P703023	Slc38a3	NM_145776	252919	0.39	2.43	0.047988
A_44_P545727	Fln29	XM_341086	114635	0.39	2.43	0.025558
A_42_P646213	RGD1311103_predicted	XM_217304		0.39	2.43	0.388877
A_44_P149720	LOC295635	NM_001013917	295635	0.39	2.43	0.020686
A_44_P680263	XM_579763	XM_579763		0.39	2.43	0.00559
A_44_P322314	AW918276	AW918276		0.39	2.43	0.000276
A_42_P598304	Klrb1a_mapped	NM_001010964	25192	0.38	2.43	0.011397
A_44_P1024965	Whsc2	NM_001008339	305455	0.38	2.42	0.033067
A_44_P269241	RGD1306613_predicted	XM_225401		0.38	2.42	0.010814
A_44_P1013314	G1p2_predicted	XM_216605		0.38	2.42	0.033113
A_44_P233702	Clcf1	NM_207615	365395	0.38	2.42	0.003232
A_44_P1002336	Mdfic_predicted	XM_342643		0.38	2.42	0.000194

A_44_P513587	A_44_P513587	A_44_P513587		0.38	2.42	0.001596
A_44_P866982	AA899481	AA899481		0.38	2.42	0.006045
A_43_P10806	Baz1b	XM_001077467		0.38	2.42	5.22E-05
A_44_P900924	TC543926	TC543926		0.38	2.42	0.000587
A_42_P785428	Cdk2	NM_199501	362817	0.38	2.42	0.000596
A_44_P147047	Adam10	XM_217197	29650	0.38	2.42	0.000512
A_44_P754479	RGD1564361_predicted	XM_573283	498081	0.38	2.42	0.005832
A_44_P959960	TC518391	TC518391		0.38	2.42	0.042561
A_44_P182336	Tanc1	NM_001002854	311055	0.38	2.42	0.000332
A_44_P787936	Stat3	NM_012747	25125	0.38	2.42	0.008781
A_44_P360907	A_44_P360907	A_44_P360907		0.38	2.42	0.002466
A_44_P782486	RGD1563612_predicted	XM_001055704		0.38	2.42	0.064983
A_44_P398558	RGD1305915	XM_231295		0.38	2.42	0.000236
A_44_P229415	A_44_P229415	A_44_P229415		0.38	2.42	0.01395
A_44_P1003715	RGD1566133_predicted	XM_001054815		0.38	2.42	0.000723
A_44_P536168	Nudc	NM_017271	29648	0.38	2.42	0.000106
A_42_P535743	Galnt11	NM_199393	311952	0.38	2.42	0.000749
A_44_P391057	Angptl4	NM_199115	362850	0.38	2.42	0.036872
A_43_P10226	Lrrc49_predicted	XM_236333	300763	0.38	2.42	0.002521
A_42_P662770	MGC125214	XM_001055205	304332	0.38	2.42	0.002314
A_44_P505992	Prpsap2	NM_057131	117272	0.38	2.41	0.000539
A_44_P1001735	Nudcd2	NM_001009621	287199	0.38	2.41	0.000465
A_44_P424320	RGD1306816_predicted	XM_215844		0.38	2.41	7.75E-05
A_42_P465780	RGD1561636_predicted	XM_578241		0.38	2.41	0.000074
A_43_P17786	Setmar	XM_001071895	500281	0.38	2.41	0.011081
A_44_P469103	RGD1564623_predicted	XM_573140		0.38	2.41	0.000208
A_44_P308687	Nsmaf	NM_181389	353233	0.38	2.41	0.013129
A_44_P667548	A_44_P667548	A_44_P667548		0.38	2.41	0.000037
A_44_P180259	Dpt_predicted	XM_213925		0.38	2.41	0.001426
A_44_P511519	Mcm4	XM_001068436		0.38	2.41	0.000351
A_44_P208741	Gnl2	NM_001025736	362593	0.38	2.41	8.79E-05
A_44_P1029403	Bst2	NM_198134	378947	0.38	2.41	0.00126
A_44_P852338	RGD1563145_predicted	XM_575281		0.38	2.41	5.18E-05
A_44_P518192	RGD1563548_predicted	XM_344618	364755	0.38	2.41	0.063185
A_44_P140135	RGD1561639_predicted	XM_219345	308991	0.38	2.41	0.001134
A_43_P17458	RGD1549725	NM_001014105	317191	0.38	2.41	0.014295
A_44_P868092	XM_217339	XM_217339		0.38	2.41	0.002465
A_44_P540542	LOC679712	XM_001054154		0.38	2.41	0.000568
A_44_P567789	A_44_P567789	A_44_P567789		0.38	2.40	0.057488
A_44_P160568	RGD1564451_predicted	XM_233982		0.38	2.40	0.000762
A_44_P257075	Ahcyl1_predicted	XM_001068488		0.38	2.40	0.002525
A_42_P562617	Irf5_predicted	XM_216105		0.38	2.40	0.000244
A_44_P623426	LOC683109	XM_001064493		0.38	2.40	0.004437
A_44_P1006261	LOC682382	XM_001061281		0.38	2.40	0.000522
A_43_P15195	Rpo1-1	NM_001008330	301246	0.38	2.40	7.46E-05
A_44_P534113	Plaa	NM_053866	116645	0.38	2.40	0.000187
A_44_P202988	Odc1	NM_012615	24609	0.38	2.40	0.000954
A_43_P13216	Cdc25b	NM_133572	171103	0.38	2.40	0.000476
A_44_P235815	Clns1a	NM_031719	65160	0.38	2.40	0.002845
A_44_P180308	Rcor2	NM_001013994	305811	0.38	2.40	0.006227
A_44_P455080	Pwp1_predicted	XM_343186	362856	0.38	2.40	0.000397
A_43_P12437	Hhex	NM_024385	79237	0.38	2.40	7.19E-05
A_44_P447853	XM_345149	XM_345149		0.38	2.40	0.001183
A_43_P20897	Cpne7_predicted	XM_341711		0.38	2.40	0.025165
A_44_P485416	RGD1564859_predicted	XM_347061	362536	0.38	2.40	0.009717
A_43_P12768	Bhlhb2	NM_053328	79431	0.38	2.40	0.000144
A_44_P192382	Utp14a	NM_001014113	317579	0.38	2.40	0.000251
A_44_P514939	Bzrp	NM_012515	24230	0.38	2.40	0.00049
A_44_P372471	NM_147139	NM_147139	257645	0.38	2.40	0.005736
A_44_P529691	LOC498249	XM_573473		0.38	2.40	0.027115
A_44_P1040710	LOC690208	XM_001073671	690208	0.38	2.40	0.002725
A_44_P327492	AW917621	AW917621	25291	0.38	2.40	0.001259
A_44_P206439	XM_222435	XM_222435		0.38	2.39	0.000244

A_44_P490159	RGD1307173	NM_001025283	315549	0.38	2.39	0.001425
A_44_P245101	Spg21	NM_001006987	300791	0.38	2.39	0.001328
A_44_P214797	AW917567	AW917567		0.38	2.39	0.008902
A_44_P518385	Yars	NM_001025696	313047	0.38	2.39	0.000236
A_44_P607412	Nfrkb_predicted	XM_001056399		0.38	2.39	0.072528
A_43_P20544	Ephb3_predicted	XM_221311		0.38	2.39	0.014395
A_42_P738549	Napsa	NM_031670	60575	0.38	2.39	0.008293
A_44_P335155	E2f5	XM_574892	116651	0.38	2.39	0.000646
A_43_P13337	Stip1	NM_138911	192277	0.38	2.39	0.00042
A_44_P561113	TC538424	TC538424		0.38	2.39	0.011172
A_44_P523917	Ogt	NM_017107	26295	0.38	2.39	0.015501
A_43_P21134	RGD1305976_predicted	XM_343253	362923	0.38	2.39	0.000626
A_44_P314122	XM_212821	XM_212821		0.38	2.39	0.00042
A_44_P973796	DY319575	DY319575		0.38	2.39	0.000216
A_44_P898704	LOC691098	XM_001072433		0.38	2.39	2.77E-05
A_44_P245192	RGD1565969_predicted	XM_001065252		0.38	2.39	0.001216
A_43_P10545	Lyk5	NM_182820	303605	0.38	2.39	0.000402
A_44_P251086	RGD1562186_predicted	XM_221409	303909	0.38	2.39	0.016803
A_44_P420215	CB606269	CB606269		0.38	2.39	0.000699
A_44_P960013	Pex19	XM_001057414		0.38	2.39	0.002135
A_44_P286024	LOC289471	XM_214002	289471	0.38	2.39	0.016548
A_43_P17734	Aasdhppt_predicted	XM_217078		0.38	2.39	0.012114
A_44_P613696	A_44_P613696	A_44_P613696		0.38	2.38	0.000392
A_44_P540848	Actn1	NM_031005	81634	0.38	2.38	4.52E-05
A_43_P15623	Ddx1	NM_053414	84474	0.38	2.38	0.004806
A_44_P717695	Fem1b_predicted	XM_001074110		0.38	2.38	0.004884
A_42_P472590	Mthfd1l_predicted	XM_341750		0.38	2.38	0.000267
A_44_P311647	Dpysl2	XM_573810		0.38	2.38	0.000715
A_44_P610320	Rab2b	BC092636	305853	0.38	2.38	0.001265
A_44_P400494	Krt1-18	NM_053976	294853	0.38	2.38	0.001974
A_44_P549724	RGD1564943_predicted	XM_001060653		0.38	2.38	0.00342
A_44_P214415	Yeats4_predicted	XM_216890	299810	0.38	2.38	0.001376
A_43_P21303	RGD1564005_predicted	XM_340854	360575	0.38	2.38	0.000278
A_44_P428467	Homez	NM_152849	260325	0.38	2.38	0.015989
A_44_P827706	TC529707	TC529707		0.38	2.38	0.00889
A_44_P421405	Sh3kbp1	NM_053360	84357	0.38	2.38	0.034167
A_44_P304011	Hspa5bp1	NM_178021	338474	0.38	2.38	0.001413
A_44_P468746	Tubb6	NM_001025675	307351	0.38	2.38	0.001272
A_44_P198539	Cblb	NM_133601	171136	0.38	2.38	0.002339
A_44_P718045	Ankfy1_predicted	XM_239269		0.38	2.38	0.024761
A_44_P855435	TC531086	TC531086		0.38	2.38	0.001253
A_44_P373698	Uble1b	XM_218502	308508	0.38	2.38	0.000453
A_44_P1025682	Lrrc46	NM_001004201	287653	0.38	2.38	0.001572
A_42_P734395	Scml4_predicted	XM_228308		0.38	2.38	8.75E-05
A_42_P550172	Hip1r	XM_001072438	81917	0.38	2.38	0.000211
A_44_P1058158	LOC246120	NM_139255	246120	0.38	2.38	0.000317
A_44_P606189	A_44_P606189	A_44_P606189		0.38	2.37	0.001474
A_44_P489862	Tnfaip8l2	NM_001014039	310663	0.38	2.37	0.001354
A_44_P976667	TC525603	TC525603		0.38	2.37	0.077586
A_44_P123732	RGD1563307_predicted	XM_218493		0.38	2.37	0.000197
A_44_P820148	BQ190042	BQ190042		0.37	2.37	9.37E-05
A_43_P20792	LOC690308	XM_001074043		0.37	2.37	0.001588
A_44_P479917	Mtr	NM_030864	81522	0.37	2.37	0.006991
A_42_P840087	Dcun1d5	NM_001009696	315405	0.37	2.37	0.001209
A_42_P827359	Mybl2_predicted	XM_215922		0.37	2.37	0.001359
A_44_P450149	RGD1561928_predicted	XM_224924		0.37	2.37	0.000167
A_44_P126389	Slc25a26_predicted	XM_342726		0.37	2.37	0.001435
A_44_P616357	CB795156	CB795156	291157	0.37	2.37	0.027592
A_44_P852005	RGD1562451_predicted	XM_213689		0.37	2.37	0.000691
A_42_P455863	LOC497813	NM_001024280	497813	0.37	2.37	0.000183
A_43_P19455	Pard6g_predicted	XM_225733		0.37	2.37	0.000381
A_44_P459362	Xpo4_predicted	XM_214191		0.37	2.37	0.00424
A_44_P541633	RGD1305160	NM_001025124	360462	0.37	2.37	0.004075

A_42_P594008	Eif2b3	NM_133609	171145	0.37	2.37	0.00027
A_44_P554741	AI598485	AI598485		0.37	2.37	0.00138
A_44_P102876	RGD1560629_predicted	XM_574885		0.37	2.37	0.006433
A_43_P22402	Fbxo5_predicted	XM_214756		0.37	2.36	0.002857
A_44_P534748	Solh_predicted	XM_239171		0.37	2.36	0.008844
A_44_P1013755	Snx12_predicted	XM_343799		0.37	2.36	0.001494
A_44_P1030215	MGC94600	NM_001006989	301013	0.37	2.36	0.003167
A_44_P135696	A_44_P135696	A_44_P135696		0.37	2.36	0.000793
A_44_P699312	RGD1562539_predicted	XM_573400	360815	0.37	2.36	0.000024
A_44_P874279	TC521786	TC521786		0.37	2.36	0.011217
A_44_P871255	TC549709	TC549709		0.37	2.36	0.003452
A_44_P444485	RGD1565584_predicted	XM_214991		0.37	2.36	0.003307
A_44_P228715	Smad4	NM_019275	50554	0.37	2.36	0.010513
A_42_P505280	Uble1a	NM_001012063	308384	0.37	2.36	6.83E-05
A_44_P571937	BF288545	BF288545		0.37	2.36	0.009716
A_44_P280659	RGD1305110_predicted	XM_223682		0.37	2.36	0.001424
A_44_P130780	Rpl13	NM_031101	81765	0.37	2.36	0.000278
A_44_P199460	A_44_P199460	A_44_P199460		0.37	2.36	0.012083
A_44_P918476	TC546685	TC546685		0.37	2.36	0.003146
A_42_P735353	Scye1	NM_053757	114632	0.37	2.36	0.000107
A_44_P976212	TC557173	TC557173		0.37	2.36	0.010832
A_43_P17429	Rg9mtd3	NM_001013090	298081	0.37	2.36	0.054452
A_44_P100443	Ung	NM_001013124	304577	0.37	2.36	0.000361
A_44_P432139	Rpl5	NM_031099	81763	0.37	2.36	0.000391
A_42_P753270	Zfp444_predicted	XM_214805	292569	0.37	2.35	0.000474
A_44_P805574	LOC689069	XM_001069394	689069	0.37	2.35	0.065693
A_44_P186493	LOC681012	XM_001059906	681012	0.37	2.35	0.000358
A_43_P21003	RGD1306841	NM_001033892	298688	0.37	2.35	0.001682
A_44_P121997	Unc5cl	XM_236916		0.37	2.35	0.00019
A_44_P304521	RGD1311045_predicted	XM_341225	360947	0.37	2.35	0.000481
A_44_P461863	A_44_P461863	A_44_P461863		0.37	2.35	4.94E-05
A_44_P524321	Racgap1_predicted	XM_001062413		0.37	2.35	0.002141
A_44_P494984	BI295563	BI295563	296136	0.37	2.35	0.00872
A_44_P1029397	Bst2	NM_198134	378947	0.37	2.35	0.001883
A_44_P109638	Ubc	NM_017314	50522	0.37	2.35	0.005709
A_44_P114720	Adprhl2_predicted	XM_342918		0.37	2.35	0.000228
A_44_P883204	A_44_P883204	A_44_P883204		0.37	2.35	0.000374
A_43_P19657	Tmem68_predicted	XM_232647		0.37	2.35	0.001259
A_44_P119666	LOC363675	NM_001014229	363675	0.37	2.35	0.002387
A_42_P775698	Hrmt1l2	NM_024363	60421	0.37	2.35	0.000302
A_44_P698581	A_44_P698581	A_44_P698581		0.37	2.35	0.000154
A_44_P905125	Zfx_predicted	XM_001065337		0.37	2.35	0.003619
A_44_P212927	XM_222593	XM_222593		0.37	2.35	0.000219
A_44_P100518	RGD1565838_predicted	XM_342008		0.37	2.35	0.000429
A_44_P166495	Rangap1	NM_001012199	362965	0.37	2.35	0.000274
A_44_P243501	RGD1308517	NM_001037181	290722	0.37	2.35	0.01115
A_43_P17377	Uchl5	NM_001012149	360853	0.37	2.35	0.000346
A_43_P21178	Trip12	NM_001031659	316575	0.37	2.35	0.009905
A_44_P667458	A_44_P667458	A_44_P667458		0.37	2.35	0.000212
A_42_P514585	Tpt1	NM_053867	116646	0.37	2.35	0.002465
A_43_P11820	Kcnj8	NM_017099	25472	0.37	2.34	0.142771
A_44_P212785	Sgpp1	XM_343081	81536	0.37	2.34	0.004397
A_42_P566079	MGC109519	NM_001024345	500450	0.37	2.34	0.000801
A_44_P112446	RGD1566078_predicted	XM_343837		0.37	2.34	0.000119
A_44_P183075	Bcap29	NM_001006980	298943	0.37	2.34	0.004114
A_44_P302250	LOC691531	XM_344537		0.37	2.34	5.12E-05
A_44_P250414	XM_218779	XM_218779		0.37	2.34	0.000106
A_44_P431150	Pole_mapped	XM_222255		0.37	2.34	0.014396
A_44_P518576	RGD1310778_predicted	XM_221670	288272	0.37	2.34	0.014182
A_44_P487898	LOC304138	NM_001013980	304138	0.37	2.34	0.003456
A_44_P480361	RGD1311868	NM_001033061	289150	0.37	2.34	0.024357
A_44_P1033739	LOC498549	XM_573823	498549	0.37	2.34	0.001113
A_44_P196757	Sacs_predicted	XM_224256	305940	0.37	2.34	0.000926

A_43_P21977	RGD1566394_predicted	XM_574584	499285	0.37	2.34	0.031152
A_44_P560195	A_44_P560195	A_44_P560195		0.37	2.34	7.16E-05
A_44_P218014	Ruvbl1	NM_147177	65137	0.37	2.34	0.000176
A_44_P274526	RGD1311378_predicted	XM_230889		0.37	2.34	0.004123
A_43_P19193	Lama1_predicted	XM_237536		0.37	2.34	0.032117
A_44_P415034	LOC298615	XR_006039	298615	0.37	2.34	0.000364
A_43_P10692	TC540999	TC540999		0.37	2.34	0.000166
A_44_P593128	TC542857	TC542857		0.37	2.34	0.045111
A_44_P171128	Alg2	XM_232987	313231	0.37	2.34	0.003491
A_42_P594043	Ift88_predicted	XM_224165		0.37	2.34	0.004994
A_44_P386176	Sec61a2_predicted	XM_341558	361273	0.37	2.34	0.003848
A_44_P244755	BQ193187	BQ193187		0.37	2.34	0.012442
A_43_P18013	Il20ra_predicted	XM_218776		0.37	2.34	0.002758
A_44_P838339	TC555485	TC555485		0.37	2.34	8.37E-05
A_44_P534374	Extl2	XM_227614	310803	0.37	2.34	0.058645
A_44_P558142	RGD1305671_predicted	XM_235656	300231	0.37	2.34	0.000538
A_44_P299247	Aqp1	NM_012778	25240	0.37	2.33	0.000515
A_44_P117282	Arhgef15_predicted	XM_220593		0.37	2.33	0.001518
A_44_P456191	AW144796	AW144796	85252	0.37	2.33	0.001822
A_44_P512974	Acn9	XM_342641		0.37	2.33	0.003087
A_44_P122201	Taf2	XM_343241	170844	0.37	2.33	0.002873
A_44_P1054993	Gli1	XM_001056128		0.37	2.33	0.077586
A_44_P313516	Bdkrb2	NM_173100	25245	0.37	2.33	0.01631
A_44_P1029770	Nol5a	NM_001025732	362214	0.37	2.33	0.000855
A_44_P557210	Myo1b	NM_053986	117057	0.37	2.33	0.008093
A_44_P981159	TC545808	TC545808		0.37	2.33	0.00037
A_44_P506313	Iars_predicted	XM_225196	306804	0.37	2.33	0.000179
A_42_P528844	Acsl5	NM_053607	94340	0.37	2.33	0.000879
A_44_P463980	RGD1565332_predicted	XM_223483		0.37	2.33	0.003171
A_42_P816020	Trpv2	NM_017207	29465	0.37	2.33	0.001921
A_44_P544023	Tuba6	NM_001011995	300218	0.37	2.33	9.85E-05
A_44_P405655	LOC499339	NM_001025036	499339	0.37	2.33	0.000121
A_42_P820847	Clec14a	NM_001014077	314148	0.37	2.33	0.001308
A_44_P518101	XM_226847	XM_226847		0.37	2.33	0.000048
A_44_P1030919	Nab2	XM_235224	314910	0.37	2.33	0.0089
A_44_P555594	Cse1l_predicted	XM_342581		0.37	2.33	0.003767
A_42_P501057	Gls2	NM_138904	192268	0.37	2.33	0.036643
A_44_P135213	Ptprc	XM_001062978		0.37	2.33	0.000361
A_44_P166955	BF523428	BF523428		0.37	2.33	0.021272
A_42_P643272	Sip1	NM_053389	84404	0.37	2.33	0.001624
A_44_P375480	Ccdc21	XM_342940		0.37	2.33	0.001764
A_44_P508189	Olr300_predicted	NM_001000237	293599	0.37	2.33	0.114006
A_44_P911129	AA956727	AA956727	361814	0.37	2.33	0.01668
A_44_P361569	BF559362	BF559362		0.37	2.33	0.001066
A_44_P180255	Akt1s1_predicted	XM_238103		0.37	2.32	0.000678
A_44_P745027	RGD1563036_predicted	XR_009438	366179	0.37	2.32	0.000589
A_44_P168713	Crip2	NM_022501	338401	0.37	2.32	0.013613
A_44_P374011	LOC498225	NM_001017489	498225	0.37	2.32	0.002062
A_44_P513232	BC099181	BC099181		0.37	2.32	0.000647
A_42_P744464	RGD1565449_predicted	XM_340740	360464	0.37	2.32	0.023482
A_44_P435782	C2ta	NM_053529	85483	0.37	2.32	0.002092
A_44_P310960	LOC294446	XM_001061084		0.37	2.32	0.000286
A_44_P437848	RGD1564253_predicted	XM_228876	317423	0.37	2.32	0.008201
A_42_P494276	Armc9_predicted	XM_217465		0.37	2.32	0.000952
A_44_P450518	RGD1563195_predicted	XM_213367		0.37	2.32	0.000624
A_42_P552233	XM_213765	XM_213765		0.37	2.32	0.000136
A_42_P597308	RGD1306762_predicted	XM_214202	290279	0.37	2.32	0.034777
A_44_P201499	XM_344596	XM_344596		0.37	2.32	0.009401
A_44_P759995	LOC683447	XM_001065985		0.37	2.32	0.000386
A_44_P189375	Fancd2	NM_001001719	312641	0.37	2.32	0.001325
A_44_P459554	RGD1309682	NM_001014055	312200	0.37	2.32	0.010424
A_43_P13047	40058	NM_057148	117515	0.37	2.32	0.001319
A_44_P292437	Mina	NM_153309	266670	0.37	2.32	0.00013

A_44_P649915	AW913985	AW913985	29484	0.37	2.32	0.00286
A_44_P221434	Dtx4	XM_238163		0.37	2.32	0.000668
A_43_P18178	LOC502872	XM_001054553		0.37	2.32	0.000184
A_44_P164309	Itga5_mapped	XM_235707		0.36	2.32	0.000274
A_44_P398691	Atp13a2_predicted	XM_342962	362645	0.36	2.32	0.000336
A_44_P142724	Mcm3_predicted	XM_236988	316273	0.36	2.31	0.000294
A_44_P128381	Serpinb3	NM_001008887	304688	0.36	2.31	0.018558
A_44_P366153	RGD1564645_predicted	XM_217220	300874	0.36	2.31	0.000242
A_44_P730729	TC542216	TC542216		0.36	2.31	0.033414
A_44_P883575	A_44_P883575	A_44_P883575		0.36	2.31	0.00027
A_44_P504215	Srebf2_predicted	NM_001033694	300095	0.36	2.31	0.003428
A_44_P561749	TC523617	TC523617		0.36	2.31	0.000896
A_44_P607477	TC557674	TC557674		0.36	2.31	0.092276
A_44_P151075	BF550737	BF550737		0.36	2.31	0.000231
A_44_P379911	XM_345191	XM_345191		0.36	2.31	0.000361
A_44_P1031421	Akap13	XM_214969		0.36	2.31	0.000383
A_44_P893828	TC548475	TC548475		0.36	2.31	0.058672
A_44_P550191	Slc2a8	NM_053494	85256	0.36	2.31	0.010481
A_44_P414713	Mkrn3_predicted	XM_218735		0.36	2.31	0.003778
A_44_P401161	RGD1565395_predicted	XM_220874		0.36	2.31	0.00046
A_44_P997825	Myd116	NM_133546	171071	0.36	2.31	0.00082
A_44_P987603	RGD1305117	NM_001013182	360492	0.36	2.31	0.002857
A_44_P1040460	Rps24	NM_031112	81776	0.36	2.31	0.000102
A_42_P481816	Esam	NM_001004245	300519	0.36	2.31	0.001492
A_44_P274700	RGD1304687_predicted	XM_001055538		0.36	2.31	0.009539
A_44_P882551	RGD1560871_predicted	XM_001072622		0.36	2.31	0.00048
A_44_P387213	XM_213072	XM_213072		0.36	2.30	0.00011
A_42_P708060	Gpc1	NM_030828	58920	0.36	2.30	0.07546
A_44_P220008	LOC304396	NM_001013981	304396	0.36	2.30	0.000399
A_44_P550988	RGD1562291_predicted	XM_237502	301676	0.36	2.30	0.052852
A_44_P215794	Cct2	NM_001005905	299809	0.36	2.30	0.002856
A_44_P173511	RGD1562287_predicted	XM_001074199		0.36	2.30	0.000249
A_42_P488076	AW142579	AW142579		0.36	2.30	0.002372
A_44_P423810	Actb	NM_031144	81822	0.36	2.30	0.001429
A_44_P548784	Trmu_predicted	XM_343306	362976	0.36	2.30	0.001716
A_44_P487112	Wee1	NM_001012742	308937	0.36	2.30	0.002603
A_44_P420957	AW915587	AW915587		0.36	2.30	0.001
A_44_P337776	XM_225974	XM_225974		0.36	2.30	2.45E-05
A_44_P276640	Tbc1d12_predicted	XM_220062		0.36	2.30	0.009964
A_44_P1044997	LOC690297	XM_001074000	690297	0.36	2.30	0.000244
A_43_P19646	XM_341833	XM_341833		0.36	2.30	0.000457
A_43_P21130	Sh3bp2	XM_223534	305450	0.36	2.30	0.002815
A_44_P407583	Kif14_predicted	XM_341126		0.36	2.30	0.003093
A_44_P419676	Zc3hc1_predicted	XM_216113	296957	0.36	2.30	0.000902
A_44_P456172	Mterfd3	NM_001014265	366856	0.36	2.30	0.001124
A_44_P1034486	NTF2	NM_001007629	291981	0.36	2.30	0.000311
A_42_P643622	TC542063	TC542063		0.36	2.30	0.001311
A_43_P18587	RGD1307983_predicted	XM_216387	298032	0.36	2.30	0.003963
A_44_P421727	Card11_predicted	XM_001073551		0.36	2.30	0.02191
A_44_P182463	Map1b	XM_215469		0.36	2.30	0.00332
A_44_P568715	BC088315	BC088315	300772	0.36	2.30	0.005473
A_42_P606938	Rpl18	NM_031102	81766	0.36	2.29	0.000053
A_44_P117194	RGD1311640_predicted	XM_235095	314787	0.36	2.29	0.051525
A_44_P838725	TC524202	TC524202		0.36	2.29	0.000036
A_44_P542995	Rnf25	NM_001012004	301515	0.36	2.29	0.001732
A_42_P671364	Banf1	NM_053631	114087	0.36	2.29	0.000281
A_43_P13812	Snag1_predicted	XM_226769		0.36	2.29	0.00011
A_44_P478340	Msc_predicted	XM_232587	312897	0.36	2.29	0.00042
A_44_P636724	A_44_P636724	A_44_P636724		0.36	2.29	0.001199
A_44_P266920	ST7	NM_001004102	296911	0.36	2.29	0.001969
A_44_P498815	LOC313641	XM_233606		0.36	2.29	0.000293
A_44_P827421	RGD1560468_predicted	XM_001071098		0.36	2.29	0.004867
A_44_P669107	TC522932	TC522932		0.36	2.29	0.042257

A_44_P340316	RGD1311578	NM_001008318	298748	0.36	2.29	0.003159
A_43_P10571	RGD1311848_predicted	XM_217285	301008	0.36	2.29	0.003634
A_44_P1008356	RGD1561181_predicted	XM_575909		0.36	2.29	0.000705
A_44_P337879	RGD1565685_predicted	XM_228714	317344	0.36	2.29	0.088573
A_44_P149321	Pdlim3	NM_053650	114108	0.36	2.29	0.012259
A_44_P316883	RGD1304728_predicted	XM_340837	360560	0.36	2.29	0.009492
A_44_P438492	Rbm13	NM_001014002	306526	0.36	2.29	0.000128
A_44_P974489	Dusp22_predicted	XM_341523		0.36	2.29	0.000751
A_44_P293357	Ccdc51	NM_001014098	316008	0.36	2.29	0.007739
A_44_P730320	Ect2_predicted	XM_001057016		0.36	2.29	0.005645
A_44_P405555	XM_228025	XM_228025		0.36	2.29	0.000338
A_44_P316319	Shkbp1_predicted	XM_214873	292735	0.36	2.29	0.000178
A_44_P759911	ENSRNOT00000015702	ENSRNOT00000015702		0.36	2.29	0.000402
A_42_P653911	Hspbap1	NM_134419	171460	0.36	2.29	0.000654
A_44_P822414	RGD1311316	NM_001012182	362007	0.36	2.29	0.00755
A_43_P11495	Prps2	NM_012634	24689	0.36	2.29	0.000244
A_43_P11908	Sema3a	NM_017310	29751	0.36	2.29	0.015368
A_44_P1022889	CF106932	CF106932	313087	0.36	2.28	0.003631
A_44_P867809	ENSRNOT00000049172	ENSRNOT00000049172		0.36	2.28	0.000296
A_44_P420503	Slpil2	NM_001008872	408229	0.36	2.28	0.005833
A_44_P121682	RGD1561993_predicted	XM_230870		0.36	2.28	0.000576
A_44_P419107	LOC310612	AY325142	310612	0.36	2.28	0.000277
A_44_P300788	Kcna3	NM_019270	29731	0.36	2.28	0.033429
A_44_P382129	LOC499014	NM_001039343	499014	0.36	2.28	0.00779
A_44_P405319	Hrasls5	NM_001039007	293711	0.36	2.28	0.000621
A_44_P447158	Pdlim3	BC081703	114108	0.36	2.28	0.0005
A_44_P554160	Ppp1r7	NM_001009825	301618	0.36	2.28	0.000421
A_44_P316533	XM_342429	XM_342429		0.36	2.28	0.008993
A_44_P230712	Rasa1	NM_013135	25676	0.36	2.28	0.003049
A_44_P883477	RGD1562596_predicted	XM_575668	362438	0.36	2.28	0.000716
A_43_P12900	Gtpbp4	NM_053689	114300	0.36	2.28	2.16E-05
A_44_P361202	LOC680575	XM_001057847	680575	0.36	2.28	0.000566
A_44_P459613	Cbfa2t1_predicted	XM_342808		0.36	2.28	0.000314
A_44_P414890	RGD1565969_predicted	XM_341545	361261	0.36	2.28	0.000479
A_44_P110788	Itgad	NM_031691	64350	0.36	2.28	0.003233
A_43_P12736	Pld2	NM_033299	25097	0.36	2.28	0.001764
A_44_P309673	A_44_P309673	A_44_P309673		0.36	2.28	0.000736
A_44_P177425	Creb1	NM_134443	81646	0.36	2.28	0.00275
A_44_P443542	Mars2_predicted	XM_237156	316403	0.36	2.28	0.000828
A_44_P139956	MGC72973	NM_198776	361619	0.36	2.28	0.049441
A_44_P374618	Cdkn1c	NM_182735	246060	0.36	2.28	0.016425
A_44_P117107	Rpsa	NM_017138	29236	0.36	2.28	0.000406
A_44_P113421	XM_214515	XM_214515		0.36	2.28	8.35E-05
A_44_P482538	Mcm4	XM_001068436		0.36	2.28	0.000247
A_44_P177405	Txndc13	XM_001081259		0.36	2.27	0.002576
A_44_P391784	RGD1563524_predicted	XM_229115		0.36	2.27	0.000142
A_44_P309179	RGD1310439_predicted	XM_223074	305073	0.36	2.27	0.010278
A_42_P622623	Tbc1d10a	NM_001015022	360968	0.36	2.27	0.002867
A_44_P413102	AA957447	AA957447		0.36	2.27	0.021349
A_44_P272120	Snrpb	XM_001059527	171365	0.36	2.27	0.000297
A_43_P10625	TC523715	TC523715		0.36	2.27	0.000675
A_44_P608475	TC526603	TC526603		0.36	2.27	0.00274
A_44_P310949	Eif3s7	NM_001004283	362952	0.36	2.27	0.000573
A_44_P421820	Tnfrsf26_predicted	XM_341968		0.36	2.27	0.004195
A_44_P653813	LOC678810	XM_001053660		0.36	2.27	0.000767
A_44_P638191	RGD1563344_predicted	XM_574086	498803	0.36	2.27	0.01847
A_44_P744264	A_44_P744264	A_44_P744264		0.36	2.27	0.000167
A_44_P280410	Adprh	NM_183325	25371	0.36	2.27	0.000277
A_43_P13119	Runx3	NM_130425	156726	0.36	2.27	0.004113
A_44_P276932	LOC293842	XR_006790	293842	0.36	2.27	0.00157
A_44_P484393	Ptma	NM_021740	29222	0.36	2.27	0.000397
A_44_P233988	RGD1306873	NM_001037191	304285	0.36	2.27	0.000139
A_44_P822839	TC521914	TC521914		0.36	2.27	0.015152

A_44_P117207	RGD1561871_predicted	XM_235190		0.36	2.27	0.002161
A_44_P364968	LOC683504	XM_001066236		0.36	2.27	0.012806
A_44_P111337	AW915033	AW915033	362788	0.36	2.27	0.000166
A_44_P451434	LOC685210	XM_001062826	685210	0.36	2.27	0.000435
A_44_P453297	Actr10	NM_001009602	299121	0.36	2.26	0.020961
A_44_P178240	Rrs1_predicted	XM_232622		0.36	2.26	0.000148
A_44_P139989	Hist1h2bh	XM_001054570		0.35	2.26	0.010916
A_44_P499092	LOC685332	XM_001063377	685332	0.35	2.26	0.001446
A_44_P496643	A_44_P496643	A_44_P496643		0.35	2.26	0.007194
A_44_P398210	Csf2rb1	NM_133555	171081	0.35	2.26	0.028247
A_44_P319104	Zdhhc9	NM_001039016	302808	0.35	2.26	0.003285
A_43_P16878	Vwa1	NM_001013938	298683	0.35	2.26	0.000935
A_44_P157789	Rnps1	NM_001011890	287113	0.35	2.26	0.000691
A_42_P613305	Ramp2	NM_031646	58966	0.35	2.26	0.003441
A_44_P824731	TC563517	TC563517		0.35	2.26	0.00243
A_44_P295458	Xkr8	NM_001012099	313033	0.35	2.26	0.008879
A_44_P198535	Akap12	NM_057103	83425	0.35	2.26	0.003299
A_44_P255509	Rpl21	NM_053330	79449	0.35	2.26	0.000109
A_42_P732126	Nek9_predicted	XM_216755		0.35	2.26	0.000928
A_44_P329909	AW143475	AW143475	171357	0.35	2.26	0.002865
A_44_P198684	Tlr5	XM_223016		0.35	2.26	0.007921
A_44_P290519	Dut	NM_053592	497778	0.35	2.26	0.000423
A_44_P863709	AW143870	AW143870		0.35	2.26	0.00037
A_43_P17699	Fbxl5_predicted	XM_223508		0.35	2.26	0.000516
A_44_P379864	ENSRNOT00000041829	ENSRNOT00000041829		0.35	2.26	0.0017
A_44_P379244	Scarb1	NM_031541	25073	0.35	2.26	0.00163
A_42_P479920	Srp14_predicted	XM_215815		0.35	2.26	0.000518
A_44_P216373	Prkcn	XM_001054495	313834	0.35	2.26	0.002094
A_44_P987655	RGD1311752_predicted	XM_001065350		0.35	2.26	0.001365
A_44_P606441	TC556498	TC556498		0.35	2.26	0.005733
A_44_P579385	Ahnak	XM_574618	191572	0.35	2.26	0.001053
A_44_P173365	Tnpo3	XM_216112		0.35	2.26	0.001575
A_44_P557228	Rela	NM_199267	309165	0.35	2.26	0.000125
A_44_P140161	RGD1309710_predicted	XM_215167		0.35	2.26	3.48E-05
A_44_P838920	TC524544	TC524544		0.35	2.26	0.005584
A_44_P1019523	LOC691098	XM_001072433		0.35	2.25	0.000293
A_44_P471182	Pfc_mapped	XM_216784		0.35	2.25	0.004056
A_44_P912777	RGD1310553	NM_001008517	301374	0.35	2.25	0.046248
A_44_P852957	TC519676	TC519676		0.35	2.25	0.00796
A_44_P885072	TC559412	TC559412		0.35	2.25	0.010442
A_44_P393273	Slc6a9	NM_053818	116509	0.35	2.25	0.000474
A_44_P431109	Irs1	NM_012969	25467	0.35	2.25	0.00588
A_44_P336148	AA858641	AA858641		0.35	2.25	0.07711
A_44_P116429	Pcdha4	NM_053933	116741	0.35	2.25	0.00102
A_44_P457468	XM_236837	XM_236837		0.35	2.25	8.09E-05
A_42_P764359	Serbp1	NM_145086	246303	0.35	2.25	0.00097
A_44_P180452	Mrps2_predicted	XM_342398		0.35	2.25	0.001953
A_44_P824467	TC543245	TC543245		0.35	2.25	0.001315
A_44_P356271	XM_344137	XM_344137		0.35	2.25	0.004396
A_44_P297985	Ube2o_predicted	XM_221132	303689	0.35	2.25	0.001461
A_44_P459210	Gprasp1	NM_134386	171407	0.35	2.25	0.051476
A_44_P1041493	TC517105	TC517105		0.35	2.25	0.056431
A_42_P664520	Fen1	NM_053430	84490	0.35	2.25	0.000813
A_44_P488489	Fcho2_predicted	XM_219503	309129	0.35	2.25	0.055369
A_44_P158302	LOC500251	NM_001025047	500251	0.35	2.25	0.009665
A_44_P312154	XM_221470	XM_221470		0.35	2.25	0.002173
A_43_P19157	Chst2_predicted	XM_345970	367145	0.35	2.25	0.007503
A_44_P215347	Enam_predicted	XM_223338		0.35	2.25	0.02417
A_44_P470976	Rhobtb2	NM_001013133	306004	0.35	2.25	0.007291
A_44_P561388	TC521878	TC521878		0.35	2.25	0.000993
A_44_P164188	RGD1310490_predicted	XM_221401	303903	0.35	2.25	0.02133
A_44_P469011	Tspan31	NM_001008378	362890	0.35	2.25	0.000618
A_44_P930997	TC539887	TC539887		0.35	2.24	0.003931

A_44_P301420	Fundc1	NM_001025027	363442	0.35	2.24	0.010892
A_43_P20079	RGD1304935_predicted	XM_233830	313866	0.35	2.24	0.02942
A_44_P336423	LOC689959	XM_001065356		0.35	2.24	0.01129
A_44_P344730	Arhgap29	NM_001009405	310833	0.35	2.24	0.000409
A_43_P10744	RGD1309784	NM_001014212	363099	0.35	2.24	0.001377
A_44_P681227	AW143725	AW143725	89825	0.35	2.24	0.00018
A_44_P156548	A_44_P156548	A_44_P156548		0.35	2.24	8.81E-05
A_44_P257719	Blm_predicted	XM_218837		0.35	2.24	0.029986
A_44_P246287	XM_235612	XM_235612		0.35	2.24	0.000286
A_44_P429476	Zfp295_predicted	XM_221622		0.35	2.24	0.007355
A_44_P744519	Npm1	NM_012992	25498	0.35	2.24	0.000569
A_44_P652131	RGD1564893_predicted	XM_575099		0.35	2.24	0.001639
A_43_P11220	Fnbp1	XM_580014		0.35	2.24	7.38E-05
A_44_P471141	Galnt2_predicted	XM_238057		0.35	2.24	0.000146
A_42_P592981	Mfap2_predicted	XM_233602		0.35	2.24	0.007692
A_44_P657280	RGD1306153	XM_001075665	361410	0.35	2.24	0.02227
A_44_P931207	BE101695	BE101695		0.35	2.24	0.003004
A_42_P559038	Plekhg2_predicted	XM_214862	292750	0.35	2.24	0.000388
A_44_P575385	RGD1310571	NM_001014147	361301	0.35	2.24	0.000319
A_43_P19545	Meis3_predicted	XM_341796		0.35	2.24	0.005414
A_44_P105292	XM_227635	XM_227635		0.35	2.24	0.079625
A_44_P134405	Slc12a2	NM_031798	83629	0.35	2.24	0.000962
A_42_P762814	Tctex1	NM_031318	83462	0.35	2.24	6.83E-05
A_44_P466474	LOC291205	XR_007380	291205	0.35	2.24	0.000292
A_44_P401105	Foxp4_predicted	XM_343526		0.35	2.24	0.000191
A_44_P483152	RGD1310474_predicted	XM_234236	314169	0.35	2.24	0.037908
A_44_P1048048	Pabpc4	XM_216517	298510	0.35	2.24	0.000691
A_44_P415412	Farslb	NM_001004252	301544	0.35	2.24	0.001812
A_42_P834588	Rpl13a	NM_173340	317646	0.35	2.24	0.000242
A_44_P471053	LOC295496	XM_215716		0.35	2.24	0.000126
A_44_P141797	BF549837	BF549837	114510	0.35	2.24	0.001623
A_44_P532180	A_44_P532180	A_44_P532180		0.35	2.24	0.002745
A_44_P160545	AW917479	AW917479		0.35	2.24	0.00475
A_44_P415132	XM_235791	XM_235791		0.35	2.23	0.000847
A_44_P197052	Pla2g2d	NM_001013428	298579	0.35	2.23	0.010669
A_43_P23197	MGC95208	NM_001005552	304176	0.35	2.23	0.002039
A_43_P12513	Rpl37	NM_031106	81770	0.35	2.23	0.000181
A_44_P542594	BF558816	BF558816		0.35	2.23	0.001792
A_44_P159569	Wdr45l_predicted	NM_001039587	360682	0.35	2.23	0.003264
A_44_P521946	AA850244	AA850244		0.35	2.23	0.054973
A_43_P17605	Tnfsf5ip1_predicted	XM_214550		0.35	2.23	0.001902
A_44_P299086	AI112947	AI112947	311372	0.35	2.23	0.026555
A_44_P427814	XM_216805	XM_216805		0.35	2.23	0.005504
A_44_P622236	TC554773	TC554773		0.35	2.23	0.001476
A_44_P412843	RGD1561463_predicted	XR_009002	366822	0.35	2.23	0.002203
A_44_P365506	Serpinb5	NM_057108	116589	0.35	2.23	0.000775
A_44_P453874	Rpl7	XM_216318	297755	0.35	2.23	0.000225
A_42_P647710	Farsla	NM_001024237	288917	0.35	2.23	0.000182
A_44_P424235	LOC680717	XM_001058556		0.35	2.23	0.000278
A_44_P705875	CX569335	CX569335		0.35	2.23	0.077207
A_44_P189796	RGD1305984	NM_001034093	365668	0.35	2.23	0.001577
A_44_P312726	Tmod3	NM_001011997	300838	0.35	2.23	0.000891
A_44_P526933	Ctnnb1	NM_053357	84353	0.35	2.23	0.004166
A_44_P120669	AI511097	AI511097		0.35	2.23	0.013733
A_44_P943514	LOC685491	XM_001061612	313048	0.35	2.23	0.000248
A_44_P284899	Appbp2	XM_001081113		0.35	2.23	0.027738
A_43_P12079	Ptges	NM_021583	59103	0.35	2.23	0.020041
A_44_P835769	CX570717	CX570717		0.35	2.22	0.014269
A_42_P659546	Actn1	NM_031005	81634	0.35	2.22	0.000178
A_44_P653870	Pdrp	NM_024384	79227	0.35	2.22	8.14E-05
A_44_P262392	Eif4ebp1	NM_053857	116636	0.35	2.22	0.000464
A_44_P212803	AF050659	AF050659		0.35	2.22	0.025695
A_44_P528072	CB546969	CB546969	502531	0.35	2.22	0.000626

A_43_P19521	Gpaa1	NM_001004240	300046	0.35	2.22	0.000458
A_44_P194928	Pigx	XM_213602		0.35	2.22	0.00079
A_44_P278866	LOC681260	XM_001060577	681260	0.35	2.22	0.000344
A_44_P421915	LOC685088	XM_001062249	685088	0.35	2.22	0.019993
A_44_P267809	LOC690585	XM_001075123		0.35	2.22	0.000201
A_44_P379513	Slc25a15	XM_224969		0.35	2.22	0.000381
A_44_P100207	Cnp1	NM_012809	25275	0.35	2.22	0.000186
A_42_P549916	Ston2_predicted	XM_234454	314349	0.35	2.22	0.002525
A_44_P351576	Gpatc1_predicted	XM_214906		0.35	2.22	0.042278
A_44_P304349	RGD1560341_predicted	XM_574154		0.35	2.22	0.004005
A_44_P485098	XM_342013	XM_342013		0.35	2.22	0.000697
A_42_P661992	LOC686634	XM_001075058		0.35	2.22	0.003543
A_44_P379588	Il1r1	NM_013123	25663	0.35	2.22	0.004547
A_44_P346744	LOC680097	XM_001055696	680097	0.35	2.22	0.008469
A_44_P652923	RGD1304653_predicted	XM_343551		0.35	2.22	0.024429
A_44_P725617	BF548107	BF548107	154968	0.35	2.22	0.037206
A_44_P993382	Ssrp1	NM_031121	81785	0.35	2.22	0.001076
A_44_P171634	Oprs1	NM_030996	29336	0.35	2.22	0.000693
A_44_P1059795	Eif2s2	NM_199380	296302	0.35	2.22	0.000211
A_42_P586761	Nope_predicted	XM_343412		0.35	2.22	0.00326
A_44_P597431	Rcn1_predicted	XM_342481		0.35	2.21	0.014949
A_44_P273697	Itgb1	NM_017022	24511	0.35	2.21	0.000548
A_44_P337691	Mfsd1_predicted	XM_342258	361957	0.35	2.21	0.000899
A_42_P774045	Rpl8	NM_001034916	26962	0.35	2.21	0.000426
A_44_P424367	Hspa8	NM_024351	24468	0.35	2.21	0.000229
A_43_P11984	Itsn1	XM_573259	29491	0.35	2.21	0.000431
A_44_P246241	RGD1306259_predicted	XM_235115	314814	0.35	2.21	0.020472
A_42_P515454	Csnk1e	NM_031617	58822	0.35	2.21	0.000171
A_44_P189496	Fcgr1	AF416291	295279	0.34	2.21	0.023561
A_44_P491590	Creb1	NM_134443	81646	0.34	2.21	0.004823
A_44_P981153	CO562477	CO562477		0.34	2.21	0.039087
A_44_P503446	Ephb1	XM_217250		0.34	2.21	0.109082
A_44_P330726	Sfrs14_predicted	XM_341413		0.34	2.21	0.005189
A_44_P977733	TC530532	TC530532		0.34	2.21	0.074786
A_43_P15739	Rapgef1	XM_001079347		0.34	2.21	0.002389
A_44_P370537	Polr3f_predicted	XM_230642		0.34	2.21	0.007239
A_44_P531770	Top2a	NM_022183	360243	0.34	2.21	0.002064
A_44_P166406	RGD1311709_predicted	XM_216567		0.34	2.21	0.000462
A_44_P238927	RGD1561740_predicted	XM_342486	362186	0.34	2.21	0.007641
A_44_P715283	CV080648	CV080648		0.34	2.21	0.03095
A_43_P20765	M16829	M16829		0.34	2.21	0.012447
A_44_P511485	A_44_P511485	A_44_P511485		0.34	2.21	0.000413
A_44_P853835	TC523484	TC523484		0.34	2.21	0.000358
A_44_P629936	TC533777	TC533777		0.34	2.21	0.062563
A_44_P377706	A_44_P377706	A_44_P377706		0.34	2.21	0.060154
A_43_P17706	LOC684666	XM_001071515		0.34	2.21	0.007739
A_44_P540660	C3orf6h	NM_182736	288022	0.34	2.21	0.000507
A_44_P277876	Tpst1	NM_001011903	288617	0.34	2.21	0.001912
A_44_P500013	Anpep	NM_031012	81641	0.34	2.21	0.010086
A_44_P279229	LOC678886	XM_001053649		0.34	2.21	0.000348
A_44_P318932	XM_231081	XM_231081		0.34	2.21	5.79E-05
A_44_P524953	Rpl6	NM_053971	117042	0.34	2.21	0.000434
A_44_P396320	RGD1565734_predicted	XM_228546		0.34	2.21	0.038176
A_44_P142807	XM_235128	XM_235128		0.34	2.21	0.000413
A_43_P11462	Ets1	NM_012555	24356	0.34	2.21	0.003463
A_44_P455330	CB548031	CB548031		0.34	2.20	0.000136
A_44_P289951	AW914233	AW914233	81814	0.34	2.20	0.000649
A_44_P215788	Cct2	NM_001005905	299809	0.34	2.20	0.000063
A_43_P23025	RGD1562173_predicted	XM_576402	500989	0.34	2.20	0.000573
A_44_P232205	Bin2	NM_001012223	366988	0.34	2.20	0.000359
A_44_P153753	Ttl	NM_138536	171572	0.34	2.20	0.000301
A_44_P1055552	Rpl23	NM_001007599	29282	0.34	2.20	8.25E-05
A_44_P879176	BI395726	BI395726		0.34	2.20	0.022629

A_44_P479890	Prrx1	NM_153821	266813	0.34	2.20	0.041923
A_44_P949804	BU758404	BU758404		0.34	2.20	0.024928
A_44_P389243	XM_344423	XM_344423		0.34	2.20	0.0002
A_44_P547045	Herc3_predicted	XM_342701		0.34	2.20	0.0009
A_44_P130968	Txndc13	XM_215853	296182	0.34	2.20	0.005689
A_44_P192426	Dhx57	XM_345625	366532	0.34	2.20	0.00199
A_44_P403457	RGD1311824_predicted	XM_237209	301466	0.34	2.20	0.046163
A_44_P758141	Csnk2a2_predicted	XM_226237		0.34	2.20	0.000692
A_43_P19462	B3gnt6_predicted	XM_219693		0.34	2.20	0.004493
A_44_P360496	Sec61a1	NM_199256	80843	0.34	2.20	0.000495
A_42_P486763	Ptma	NM_021740	29222	0.34	2.20	0.000966
A_44_P245814	Uhrf2_predicted	XM_219801		0.34	2.20	0.002801
A_44_P850592	CB312819	CB312819		0.34	2.20	0.006691
A_44_P436431	Klhdc2	NM_001034133	299113	0.34	2.20	0.000849
A_44_P625503	RGD1560391_predicted	NM_001039036	499883	0.34	2.20	0.002865
A_44_P184909	RGD1559752_predicted	XM_222520		0.34	2.20	0.000519
A_43_P12994	Nup155	NM_053952	117021	0.34	2.20	0.003738
A_44_P534196	RGD1310950_predicted	XM_213983	289400	0.34	2.19	0.00806
A_43_P16331	Zfp74	XM_344876	365224	0.34	2.19	0.001213
A_44_P347778	Bspry	NM_022261	64027	0.34	2.19	0.007345
A_44_P417453	RGD1307067_predicted	XM_343168	362840	0.34	2.19	0.000151
A_44_P528721	AI230806	AI230806		0.34	2.19	0.009297
A_43_P12007	Slc3a2	NM_019283	50567	0.34	2.19	0.000348
A_44_P105991	LOC687057	XM_001076906		0.34	2.19	0.01212
A_42_P837288	Renbp	NM_031095	81759	0.34	2.19	0.004993
A_44_P388755	Kif15	NM_181635	353302	0.34	2.19	0.005879
A_44_P203937	RGD1563986_predicted	XM_231707		0.34	2.19	0.05238
A_44_P459409	Rwdd4a	NM_001034994	502084	0.34	2.19	0.001501
A_44_P130254	Np4	NM_173299	286958	0.34	2.19	0.045242
A_44_P110606	Dus2l_predicted	XM_214665		0.34	2.19	0.00064
A_44_P1011780	Pla2g12a_predicted	XM_342340		0.34	2.19	0.000919
A_44_P192246	Aven_predicted	XM_230438		0.34	2.19	0.000974
A_44_P243279	Parvb_predicted	XM_343303	362973	0.34	2.19	0.001955
A_44_P473524	Snx30_predicted	XM_232945		0.34	2.19	0.001998
A_44_P456738	Txnrd1	NM_031614	58819	0.34	2.19	0.001193
A_44_P485388	Chd7_predicted	XM_232671		0.34	2.19	0.004294
A_44_P1025013	LOC680129	XM_001054653		0.34	2.19	0.00012
A_44_P466507	Cstf3_predicted	XM_342477		0.34	2.19	0.066361
A_44_P637734	Ddx11_predicted	XM_237570	316767	0.34	2.19	0.003488
A_43_P18345	LOC684534	XM_001070862		0.34	2.19	0.002158
A_44_P846589	Hspd1	NM_022229	63868	0.34	2.19	0.00039
A_44_P958991	LOC499224	XR_008920	499224	0.34	2.19	0.003619
A_44_P925990	Ifnar1_predicted	XM_213649		0.34	2.19	0.000526
A_43_P11459	Ednra	NM_012550	24326	0.34	2.19	0.01894
A_44_P231357	Top1mt	NM_001002798	300029	0.34	2.19	0.001517
A_44_P250041	Psmb7	NM_053532	85492	0.34	2.19	0.000449
A_42_P748981	Emg1_predicted	XM_232345		0.34	2.19	0.00011
A_44_P415177	LOC684113	XM_001068985		0.34	2.19	0.000296
A_42_P475759	Gars	XM_216152	297113	0.34	2.19	0.004818
A_44_P452632	XM_226538	XM_226538		0.34	2.19	0.003254
A_44_P126412	LOC313067	XM_232818		0.34	2.19	0.009411
A_44_P947255	TC563989	TC563989		0.34	2.19	0.005075
A_44_P426808	Tspyl	NM_001013033	29544	0.34	2.19	0.00222
A_43_P14233	Pttg1	NM_022391	64193	0.34	2.19	0.000766
A_43_P20159	Atrx	XM_217570		0.34	2.19	0.001015
A_44_P292115	AW532136	AW532136		0.34	2.19	0.015575
A_44_P253108	Spbc25	NM_001009654	295661	0.34	2.19	0.000888
A_44_P254238	Bcl2l1	NM_001033670	24888	0.34	2.19	0.001567
A_44_P180299	Klc2_predicted	XM_219696		0.34	2.19	0.000131
A_44_P637146	LOC501546	XM_576948		0.34	2.19	0.002223
A_43_P20381	LOC683676	XM_001064746		0.34	2.19	0.000987
A_44_P276297	Cops8	NM_001013227	363283	0.34	2.18	0.000204
A_44_P439475	Klf7_predicted	XM_343581		0.34	2.18	0.000278

A_44_P434023	Lsm16_predicted	XM_001072111		0.34	2.18	0.000267
A_44_P377515	LOC498685	XM_573969		0.34	2.18	0.000836
A_42_P694161	RGD1311463	NM_001014045	311279	0.34	2.18	0.000606
A_44_P364005	AW920322	AW920322		0.34	2.18	0.037306
A_44_P265831	Rnf32	NM_001012095	311936	0.34	2.18	0.021911
A_42_P500802	Nxt1_predicted	XM_215858		0.34	2.18	0.000877
A_43_P15969	LOC361377	NM_001014152	361377	0.34	2.18	0.105415
A_44_P433681	Actl6a	NM_001039033	361925	0.34	2.18	0.000493
A_44_P349769	XM_227850	XM_227850		0.34	2.18	0.001336
A_44_P171871	RGD1559931_predicted	XM_224620	306264	0.34	2.18	0.091852
A_43_P11938	Zfp260	NM_017364	53982	0.34	2.18	0.001293
A_44_P713734	LOC364806	XR_008758	364806	0.34	2.18	0.000197
A_44_P378090	RGD1305593_predicted	XM_001065112		0.34	2.18	0.000144
A_42_P606971	Slc7a8	NM_053442	84551	0.34	2.18	0.00533
A_44_P589674	AI406628	AI406628		0.34	2.18	0.004027
A_44_P852219	ENSRNOT00000044873	ENSRNOT00000044873		0.34	2.18	0.000194
A_44_P419645	Rbmxrt_predicted	XM_226369		0.34	2.18	0.00127
A_44_P466951	Zmpste24_predicted	XM_233483		0.34	2.18	0.008053
A_43_P13714	CB547640	CB547640	690243	0.34	2.18	0.000911
A_44_P1012870	RGD1309095_predicted	XM_217391		0.34	2.18	0.002422
A_44_P295606	RGD1310683_predicted	XM_237211	316452	0.34	2.18	0.036462
A_42_P475656	Pip5k2b	NM_053550	89812	0.34	2.18	0.000187
A_44_P231753	Nkx1-2_predicted	XM_219437		0.34	2.18	0.080099
A_44_P995874	XM_215270	XM_215270		0.34	2.18	0.000389
A_44_P667977	LOC367705	XR_007894	367705	0.34	2.18	0.000271
A_42_P654061	Acsl4	NM_053623	113976	0.34	2.18	0.008647
A_44_P290823	XM_345350	XM_345350		0.34	2.18	2.87E-05
A_42_P642026	RGD1304706	NM_001038494	292792	0.34	2.18	0.000453
A_44_P107097	Dcps	NM_153302	266605	0.34	2.18	0.001231
A_44_P883254	LOC680639	XM_001057131		0.34	2.18	0.00014
A_44_P283758	RGD1560876_predicted	XM_344414	364395	0.34	2.18	0.055883
A_44_P212470	RGD1310681_predicted	XM_216357	297903	0.34	2.18	0.002122
A_44_P452024	Bzw1	NM_198789	363232	0.34	2.17	0.000297
A_43_P14014	Rpl27	NM_022514	64306	0.34	2.17	4.96E-05
A_43_P13181	Clcc1	NM_133414	170927	0.34	2.17	0.013872
A_44_P420177	AI170861	AI170861		0.34	2.17	0.003045
A_44_P952129	CA509994	CA509994		0.34	2.17	0.000142
A_44_P370082	Timm22	XM_340856	79463	0.34	2.17	0.000281
A_44_P185470	RGD1561963_predicted	XM_001060744		0.34	2.17	0.001493
A_44_P794371	TC547212	TC547212		0.34	2.17	0.019937
A_43_P17246	Ipo4_predicted	XM_214199		0.34	2.17	0.000415
A_44_P550396	Zfp569	XM_001067115	499123	0.34	2.17	0.036781
A_42_P755831	Tomm20	NM_152935	266601	0.34	2.17	8.09E-05
A_44_P821374	Dpp3	NM_053748	114591	0.34	2.17	0.001295
A_42_P617230	Pus1	NM_001025563	304567	0.34	2.17	0.003724
A_43_P17824	Nphp1_predicted	XM_215847		0.34	2.17	0.000378
A_42_P669820	Efemp2	NM_001005907	293677	0.34	2.17	0.026275
A_44_P634770	Cxxc5	NM_001007628	291670	0.34	2.17	0.000228
A_44_P771792	MGC109145	XM_575279	499934	0.34	2.17	0.000853
A_44_P715524	LOC690085	AY724520	690085	0.34	2.17	0.0292
A_44_P187524	Aars	XM_214690	292023	0.34	2.17	0.001643
A_44_P637334	TC535848	TC535848		0.34	2.16	0.060002
A_44_P1027438	Gpiap1	NM_001012185	362173	0.34	2.16	0.00247
A_44_P253146	Sema6d_predicted	XM_230583		0.34	2.16	0.003032
A_44_P252678	Phb2	NM_001013035	114766	0.34	2.16	0.001136
A_44_P913360	ENSRNOT00000028121	ENSRNOT00000028121		0.34	2.16	0.00018
A_44_P511049	Prosc_predicted	XM_224947		0.34	2.16	0.006833
A_44_P715491	DV722189	DV722189		0.34	2.16	0.134853
A_44_P163795	LOC680014	XM_001055446	680014	0.34	2.16	0.00579
A_42_P743825	Psme3	NM_001011894	287716	0.34	2.16	0.000399
A_44_P224259	Pxn	NM_001012147	360820	0.34	2.16	0.002096
A_44_P299780	Hnrpc	NM_001025633	290046	0.34	2.16	0.000475
A_44_P1050510	LOC686809	XM_001075804		0.34	2.16	0.001859

A_42_P721011	Psma5	NM_017282	29672	0.33	2.16	0.000293
A_44_P530253	Adam23_predicted	NM_001029899	301460	0.33	2.16	0.025118
A_44_P247568	AA849222	AA849222		0.33	2.16	0.000433
A_44_P590784	LOC499933	NM_001025768	499933	0.33	2.16	0.008438
A_44_P821748	RGD1565616_predicted	XM_575237		0.33	2.16	0.012796
A_44_P1013753	Snx12_predicted	XM_343799		0.33	2.16	0.001898
A_44_P320448	LOC688133	XM_001081155		0.33	2.16	0.017541
A_44_P554271	Impact	NM_001012235	497198	0.33	2.16	0.008435
A_44_P232431	AI060205	AI060205		0.33	2.16	0.005142
A_42_P644717	Osbp_predicted	XM_344995		0.33	2.16	0.013591
A_44_P527480	Ccna2	NM_053702	114494	0.33	2.16	0.000741
A_44_P119153	A_44_P119153	A_44_P119153		0.33	2.16	0.004484
A_44_P606833	Mtch2_predicted	XM_215769		0.33	2.16	0.065471
A_44_P552706	Dync1h1	NM_019226	29489	0.33	2.16	0.001121
A_44_P401030	Clec14a	NM_001014077	314148	0.33	2.15	0.000505
A_44_P1043261	Rpo1-1	NM_001008330	301246	0.33	2.15	0.000181
A_43_P12236	Oat	NM_022521	64313	0.33	2.15	0.002568
A_43_P22685	Lars	NM_001009637	291624	0.33	2.15	0.001777
A_44_P520697	LOC682230	XM_001060561		0.33	2.15	0.00096
A_43_P10888	Sort1	XM_342317	83576	0.33	2.15	0.004561
A_43_P15456	Duox2	NM_024141	79107	0.33	2.15	0.012274
A_44_P775388	A_44_P775388	A_44_P775388		0.33	2.15	0.001314
A_44_P261883	Bzw2	NM_134402	171439	0.33	2.15	0.002064
A_44_P424265	LOC296637	XM_001079886		0.33	2.15	0.000345
A_44_P149036	Rpsa	NM_017138	29236	0.33	2.15	0.000694
A_44_P290936	RGD1559566_predicted	XM_234521		0.33	2.15	0.000338
A_42_P776359	Mtvr2	NM_001015013	309170	0.33	2.15	4.58E-05
A_42_P741217	LOC689765	XM_001071932	689765	0.33	2.15	0.001019
A_44_P246947	Slc25a4	NM_053515	85333	0.33	2.15	0.017858
A_44_P591026	LOC366928	XR_008236	366928	0.33	2.15	0.000503
A_44_P372907	Me2_predicted	XM_225729		0.33	2.15	0.021568
A_43_P17340	Paqr7	NM_001034081	313615	0.33	2.15	0.002114
A_44_P321686	LOC681096	XM_001060299		0.33	2.15	0.025012
A_43_P17687	Tacc3	NM_001004424	360962	0.33	2.15	0.000401
A_44_P121051	Rpl17	NM_201415	291434	0.33	2.15	0.000613
A_44_P140148	Cd248_predicted	XM_238151		0.33	2.15	0.051962
A_44_P264130	RGD1308816_predicted	XM_214651		0.33	2.15	0.009236
A_42_P817668	Rnf149	XM_343561	363222	0.33	2.15	0.000887
A_44_P806901	TC535264	TC535264		0.33	2.15	0.007573
A_44_P405733	A_44_P405733	A_44_P405733		0.33	2.15	0.083654
A_44_P1058112	Yars2	NM_001009627	287924	0.33	2.15	0.031688
A_44_P563159	RGD1562135_predicted	XM_575333	499979	0.33	2.15	0.004995
A_44_P557994	LOC298125	XR_008242	298125	0.33	2.15	0.000133
A_44_P351301	Pa2g4	NM_001004206	288778	0.33	2.15	0.001764
A_44_P522492	Clcc1	NM_133414	170927	0.33	2.15	0.008423
A_44_P180755	Tigd5_predicted	XM_235436		0.33	2.15	0.07271
A_44_P491653	Zfp384	NM_133429	171018	0.33	2.14	0.030615
A_44_P605733	Rpl5	NM_031099	81763	0.33	2.14	0.001166
A_42_P602472	RGD1310861	NM_001008290	288667	0.33	2.14	0.002192
A_44_P320620	Tpm1	NM_019131	24851	0.33	2.14	0.000179
A_44_P426638	Osmr	NM_001005384	310132	0.33	2.14	0.026555
A_44_P480126	Itga6	XM_215984	114517	0.33	2.14	0.000166
A_44_P575845	ENSRNOT00000048644	ENSRNOT00000048644		0.33	2.14	0.002923
A_44_P941895	Prosc_predicted	XM_224947		0.33	2.14	0.000416
A_43_P17622	Lass5_predicted	XM_345870		0.33	2.14	0.002262
A_44_P729514	RGD1560049_predicted	XM_001081500		0.33	2.14	0.001749
A_42_P659466	Rraga	NM_053973	117044	0.33	2.14	0.000935
A_44_P384645	Arv1_predicted	XM_214713		0.33	2.14	0.000226
A_44_P429202	RGD1563952_predicted	XM_343782	363463	0.33	2.14	0.022379
A_44_P1035706	RGD1562012_predicted	XM_579762	497962	0.33	2.14	0.002094
A_44_P549017	Aff2_predicted	XM_001054673		0.33	2.14	0.000805
A_44_P244655	LOC691145	XM_001076990	691145	0.33	2.14	0.019401
A_44_P135168	Ywhae	NM_031603	29753	0.33	2.14	6.76E-05

A_44_P326090	Ahcyl1_predicted	XM_342312		0.33	2.14	0.003489
A_44_P985925	BX883042	BX883042		0.33	2.14	0.00066
A_44_P1058389	Npm1	NM_012992	25498	0.33	2.14	0.000327
A_44_P417879	Pdrp	NM_024384	79227	0.33	2.14	0.000596
A_44_P311209	Extl3	NM_020097	56819	0.33	2.14	0.000592
A_44_P255125	Itpkb	NM_019312	54260	0.33	2.14	0.000348
A_44_P388170	AI014087	AI014087	27139	0.33	2.14	0.024362
A_44_P1006377	ENSRNOT00000011219	ENSRNOT00000011219		0.33	2.14	0.000662
A_44_P389670	LOC360568	XM_340844	360568	0.33	2.14	0.000984
A_43_P15888	Amotl2	XM_343457	65157	0.33	2.14	0.000297
A_44_P791276	A_44_P791276	A_44_P791276		0.33	2.14	0.002866
A_44_P453564	RGD1309995_predicted	XM_235003	314690	0.33	2.14	0.041775
A_44_P288925	Ppm1l_predicted	XM_227247		0.33	2.14	0.0005
A_42_P573610	Eif2c1_predicted	XM_233544	313594	0.33	2.13	0.000402
A_44_P1035807	Sephs1	XM_214509		0.33	2.13	0.000259
A_44_P356047	Hnrpab	NM_031330	83498	0.33	2.13	0.006638
A_43_P12682	Rest	NM_031788	83618	0.33	2.13	0.000565
A_44_P713299	BF285467	BF285467	680259	0.33	2.13	0.00052
A_44_P620665	RGD1305976_predicted	XM_343253	362923	0.33	2.13	0.003251
A_44_P109848	RGD1566016_predicted	XM_341116	360840	0.33	2.13	0.001757
A_44_P459516	Cep152_predicted	XM_230555	311391	0.33	2.13	0.015012
A_43_P11255	RGD1559930_predicted	XM_215843	296115	0.33	2.13	0.004766
A_44_P982756	DV727100	DV727100		0.33	2.13	0.061494
A_44_P187767	LOC302898	NM_001008876	302898	0.33	2.13	0.000403
A_44_P335617	Tardbp	NM_001011979	298648	0.33	2.13	0.000503
A_44_P410341	Ipp_predicted	XM_233415		0.33	2.13	0.001959
A_44_P362992	Tcf12	NM_013176	25720	0.33	2.13	0.011487
A_44_P520091	Harpb64	XM_216665	298917	0.33	2.13	0.003162
A_44_P578164	TC545684	TC545684		0.33	2.13	0.005024
A_44_P852573	A_44_P852573	A_44_P852573		0.33	2.13	0.000662
A_44_P550611	RGD1564385_predicted	XM_341876		0.33	2.13	0.005258
A_44_P962896	TC564051	TC564051		0.33	2.13	0.000749
A_44_P199301	A_44_P199301	A_44_P199301		0.33	2.13	0.044838
A_44_P184726	Rela	NM_199267	309165	0.33	2.13	0.001116
A_44_P267501	RGD1311358	NM_001017448	294519	0.33	2.13	0.006527
A_44_P340329	Pum2	XM_001068003		0.33	2.13	0.00158
A_44_P365155	Hprt	NM_012583	24465	0.33	2.13	0.01008
A_44_P700164	Mrps14_predicted	XM_213906		0.33	2.13	0.028478
A_44_P620966	ENSRNOT00000037975	ENSRNOT00000037975		0.33	2.13	0.003167
A_44_P140400	Rpl10a	NM_031065	81729	0.33	2.13	0.000184
A_44_P120231	Snag1_predicted	XM_226769		0.33	2.13	0.006338
A_44_P182847	Trim27_predicted	XM_214485		0.33	2.13	9.64E-05
A_44_P433378	Gpr55_predicted	XM_576605		0.33	2.13	0.003609
A_44_P239271	RGD1560788_predicted	XM_343952	363662	0.33	2.13	0.020084
A_44_P277010	Imp3_predicted	XM_236276		0.33	2.13	0.000191
A_44_P107871	RGD1565579_predicted	XM_346330		0.33	2.13	0.000172
A_44_P886495	Spred1	XM_230454		0.33	2.13	0.000729
A_44_P252435	Nhn1	NM_201416	292067	0.33	2.12	0.000785
A_44_P141091	AW915665	AW915665		0.33	2.12	0.001987
A_44_P929750	CA511666	CA511666		0.33	2.12	0.010261
A_42_P803673	LOC360228	NM_001003706	360228	0.33	2.12	0.135661
A_44_P1018382	XM_228822	XM_228822		0.33	2.12	0.056862
A_44_P549998	Dhps	NM_001004207	288923	0.33	2.12	0.001011
A_44_P490296	Gga3_predicted	XM_340935		0.33	2.12	0.001273
A_44_P136730	LOC682543	XM_001061959		0.33	2.12	0.007279
A_44_P550662	Ms4a6a_predicted	XM_215145	293750	0.33	2.12	0.008902
A_44_P360325	Cct3	NM_199091	295230	0.33	2.12	8.86E-05
A_44_P718305	Atf1	XM_001064437		0.33	2.12	0.000844
A_44_P1034030	Aff4_predicted	XM_220420		0.33	2.12	0.020455
A_44_P123949	LOC682319	XM_001061003		0.33	2.12	0.003824
A_43_P21383	CB544480	CB544480		0.33	2.12	0.000349
A_44_P433783	Sfpi1	NM_001005892	366126	0.33	2.12	0.003521
A_44_P389875	BC093376	BC093376		0.33	2.12	0.024584

A_44_P1014311	Atg16l1_predicted	XM_343619		0.33	2.12	0.002482
A_44_P304259	U78132	U78132		0.33	2.12	0.001132
A_44_P409975	Wee1	NM_001012742	308937	0.33	2.12	0.001201
A_44_P157840	AI101637	AI101637	313678	0.33	2.12	0.078255
A_44_P513038	Smarca4	XM_343358	171379	0.33	2.12	0.000242
A_44_P445369	LOC360824	XM_001080476		0.33	2.12	0.004766
A_42_P680545	RGD1305235	NM_001017447	292267	0.33	2.12	0.031239
A_42_P455078	Zfp207	NM_001039020	303763	0.33	2.12	0.004564
A_44_P1044172	Ift74	NM_001007001	313365	0.33	2.12	0.001405
A_44_P553751	Avpi1	NM_134373	171386	0.33	2.12	0.001058
A_44_P149476	Lrch4_predicted	XM_341047	360779	0.33	2.12	0.002587
A_44_P365584	Il1rl1	U04317	25556	0.33	2.12	0.005046
A_44_P333550	LOC305076	NM_001013986	305076	0.33	2.12	0.005557
A_44_P641058	Rbpsuh_predicted	XM_232595		0.33	2.12	0.000129
A_44_P489869	Igsf2_predicted	XM_227554	310727	0.33	2.12	0.001237
A_44_P252906	RGD1305145	NM_001034921	293128	0.33	2.12	0.000795
A_44_P754987	Chordc1_predicted	XM_001075005		0.33	2.12	0.003894
A_44_P400729	RGD1306064_predicted	XM_224581	306229	0.33	2.12	0.038045
A_44_P316239	Lyn	NM_030857	81515	0.33	2.12	0.001224
A_44_P621378	LOC687647	XM_001079535		0.33	2.11	0.000354
A_44_P471819	LOC689616	XM_001071384	689616	0.33	2.11	0.023353
A_44_P219322	BC091431	BC091431		0.33	2.11	0.000322
A_44_P171285	Bop1	NM_001024250	300050	0.33	2.11	0.000426
A_44_P959572	Eef1g	XM_574616		0.33	2.11	0.001595
A_44_P457499	RGD1560464_predicted	XM_343883		0.33	2.11	0.001518
A_44_P608632	BF544933	BF544933		0.33	2.11	0.003336
A_44_P601020	A_44_P601020	A_44_P601020		0.33	2.11	0.002996
A_44_P487819	Rpl21	NM_053330	79449	0.33	2.11	0.00013
A_44_P135549	Ap1m1	XM_240364		0.33	2.11	0.00012
A_44_P774946	AW143545	AW143545		0.32	2.11	0.000236
A_44_P717610	TC537784	TC537784		0.32	2.11	0.001505
A_44_P538691	Rpo1-4	NM_031772	83581	0.32	2.11	0.000343
A_44_P753384	TC538043	TC538043		0.32	2.11	0.002711
A_44_P137406	Pebp1	NM_017236	29542	0.32	2.11	0.001295
A_43_P20329	Pum1_predicted	XM_342928		0.32	2.11	0.00046
A_44_P643807	LOC304037	XM_001055321		0.32	2.11	0.023232
A_44_P572344	Tnks2_predicted	XM_001080123		0.32	2.11	0.000537
A_44_P536708	XM_214409	XM_214409		0.32	2.11	0.000158
A_44_P524844	Nfix	XM_213849		0.32	2.11	0.002593
A_44_P245752	Pigc	NM_001012207	364032	0.32	2.11	0.000616
A_44_P976687	LOC681870	XM_001058771		0.32	2.11	0.001268
A_42_P664837	Kdelc2	NM_001025123	315664	0.32	2.11	0.000828
A_44_P359684	Nfkbiz_predicted	XM_221537		0.32	2.11	0.000708
A_43_P13445	LOC499474	NM_001029925	499474	0.32	2.11	0.000614
A_42_P569708	Gpsm3	NM_001003974	406163	0.32	2.11	0.007844
A_44_P219921	Hist1h2bm_predicted	XM_341530		0.32	2.11	0.020938
A_44_P318616	Rpl7a_predicted	XM_001061939		0.32	2.11	0.000376
A_44_P913245	A_44_P913245	A_44_P913245		0.32	2.11	0.002939
A_44_P684912	TC542127	TC542127		0.32	2.11	0.030152
A_44_P1014902	LOC691610	XM_001079020	691610	0.32	2.11	0.0079
A_44_P265066	Calml3	NM_001012054	307100	0.32	2.11	0.00152
A_44_P422696	Scarb1	NM_031541	25073	0.32	2.11	0.00054
A_44_P456564	Nob1p	NM_199086	291996	0.32	2.10	0.000187
A_44_P255341	Ipo9_predicted	XM_222661		0.32	2.10	9.76E-05
A_44_P457349	Slc5a9_predicted	XM_345567		0.32	2.10	0.05643
A_43_P23029	Fars2	NM_001013139	306879	0.32	2.10	0.001199
A_42_P735218	LOC688885	XM_001068700	688885	0.32	2.10	0.00153
A_42_P797295	Sulf2	XM_230861		0.32	2.10	0.021668
A_44_P205476	AA818700	AA818700	288353	0.32	2.10	0.005823
A_43_P14871	Ltbp1	NM_021587	59107	0.32	2.10	0.012949
A_44_P443138	RGD1561628_predicted	XM_001054273		0.32	2.10	0.002099
A_44_P343517	AW142420	AW142420		0.32	2.10	0.065636
A_42_P571955	Phf5a	NM_138888	192246	0.32	2.10	0.000408

A_44_P476780	Stk39	NM_019362	54348	0.32	2.10	0.026919
A_44_P332971	XM_223966	XM_223966		0.32	2.10	0.001872
A_44_P763137	TC562291	TC562291		0.32	2.10	0.05203
A_44_P236306	Fbxl10	XM_222177	304495	0.32	2.10	0.000243
A_44_P149782	XM_230768	XM_230768		0.32	2.10	0.000539
A_44_P1027904	Pop5_predicted	XM_213794		0.32	2.10	0.001722
A_44_P378383	Rnf26_predicted	XM_236181	300659	0.32	2.10	0.000275
A_44_P494130	Nfkb1	XM_342346	81736	0.32	2.10	0.000165
A_44_P339947	LOC360912	XM_341186	360912	0.32	2.10	0.047017
A_44_P389664	RGD1563668_predicted	XM_573095		0.32	2.10	0.000916
A_43_P11044	G0s2	NM_001009632	289388	0.32	2.10	0.048431
A_44_P806236	A_44_P806236	A_44_P806236		0.32	2.10	0.000199
A_43_P16960	CB547898	CB547898	361598	0.32	2.10	0.002951
A_44_P1028952	Kctd1	XM_214617	291772	0.32	2.10	0.013591
A_44_P487073	LOC306079	XM_224414		0.32	2.10	0.000241
A_43_P10642	Poldip3_predicted	XM_001077668		0.32	2.10	0.00036
A_44_P393531	Adipoq	NM_144744	246253	0.32	2.10	0.03195
A_44_P454580	RGD1310835_predicted	XM_213702		0.32	2.10	0.00435
A_42_P765298	Cuta	NM_212494	294288	0.32	2.10	0.000319
A_42_P607039	Anapc1_predicted	XM_230589		0.32	2.10	0.000236
A_44_P438641	LOC300303	XM_213108	300303	0.32	2.10	0.000151
A_44_P125865	Nup98	XM_574504	81738	0.32	2.10	0.000938
A_44_P428997	RGD1311970_predicted	XM_226971		0.32	2.10	0.000691
A_44_P1026560	Syncrip	XM_001065902		0.32	2.10	0.009884
A_43_P16632	Eif2s2	NM_199380	296302	0.32	2.10	0.000086
A_44_P1002582	Pcnt1	NM_001025401	287830	0.32	2.10	0.002007
A_44_P443412	RGD1309929	NM_001029917	298787	0.32	2.10	0.002425
A_44_P348257	Ttc5	NM_001013131	305837	0.32	2.10	0.095957
A_44_P269666	LOC689414	XM_001070712	689414	0.32	2.10	0.019338
A_44_P424379	Usp39_predicted	XM_216173		0.32	2.10	0.007592
A_43_P17401	Ppme1	XM_341891	361613	0.32	2.10	0.000597
A_44_P1001528	Tcirg1	BC061859	293650	0.32	2.10	0.003539
A_44_P716307	TC526697	TC526697		0.32	2.10	0.002757
A_44_P423880	Rfc2	NM_053786	116468	0.32	2.10	0.000722
A_43_P10996	Atp1b2	NM_012507	24214	0.32	2.10	0.002021
A_44_P821513	A_44_P821513	A_44_P821513		0.32	2.10	0.005402
A_44_P245795	Rps6ka4_predicted	XM_342004		0.32	2.10	0.000368
A_43_P11888	Psme1	NM_017264	29630	0.32	2.10	0.001058
A_44_P559225	Fem1b_predicted	XM_001074110		0.32	2.10	0.0017
A_44_P297619	Cndp2	NM_001010920	291394	0.32	2.10	0.000417
A_44_P744488	A_44_P744488	A_44_P744488		0.32	2.09	0.009105
A_44_P501992	Ero1l	BC071175	171562	0.32	2.09	0.002818
A_43_P20729	Bat2	NM_212462	294250	0.32	2.09	0.000223
A_44_P119127	Bub3	XM_341943		0.32	2.09	0.002388
A_42_P541034	Ftl1	NM_022500	29292	0.32	2.09	0.004312
A_44_P792811	TC542264	TC542264		0.32	2.09	0.013708
A_44_P636337	RGD1565566_predicted	XM_574336	499057	0.32	2.09	0.000248
A_44_P199368	Jmjd2a_predicted	XM_233441		0.32	2.09	0.00665
A_44_P145142	Rkhd1_predicted	XM_234921	299613	0.32	2.09	0.002017
A_43_P19501	Dnajc18	NM_001013887	291677	0.32	2.09	0.003639
A_44_P996369	LOC686098	XM_001066536	686098	0.32	2.09	0.016814
A_44_P126311	ENSRNOT00000012428	ENSRNOT00000012428		0.32	2.09	0.000238
A_44_P250798	RGD1566136_predicted	XM_213106		0.32	2.09	9.18E-05
A_44_P653701	TC540900	TC540900		0.32	2.09	0.005879
A_44_P438894	Klhl11_predicted	XM_213471		0.32	2.09	0.002734
A_43_P12162	Ppp2r2b	NM_022209	60660	0.32	2.09	0.008487
A_44_P398148	Sh3bp5	NM_054011	117186	0.32	2.09	0.036923
A_42_P749550	RGD1562622_predicted	XM_578556		0.32	2.09	0.000415
A_44_P152205	RGD1561791_predicted	XM_575674	500323	0.32	2.09	0.064823
A_44_P853254	TC555318	TC555318		0.32	2.09	0.000962
A_44_P365703	Nipsnap1	XM_341249	360971	0.32	2.09	0.004086
A_44_P376502	Prdx4	NM_053512	85274	0.32	2.09	0.00265
A_44_P1047467	RGD1305614_predicted	XM_221343	303824	0.32	2.09	0.002952

A_43_P13173	Lfng	NM_133393	170905	0.32	2.09	0.000262
A_44_P428863	Fbxl14_predicted	XM_232330	312675	0.32	2.09	0.000602
A_42_P843366	Adnp	NM_022681	64622	0.32	2.09	0.001479
A_44_P489688	B3galt7_predicted	XM_218342		0.32	2.09	0.008142
A_42_P666663	RGD1307018	XM_342417		0.32	2.09	0.000434
A_44_P286236	RGD1310827	NM_001034010	362364	0.32	2.09	0.015882
A_44_P508724	ENSRNOT00000037018	ENSRNOT00000037018		0.32	2.09	0.000795
A_44_P485158	RGD1305984	NM_001034093	365668	0.32	2.09	0.000782
A_44_P552926	Mpzl1	NM_001007728	360871	0.32	2.09	0.001997
A_42_P521902	Gtf3c5_predicted	XM_342399		0.32	2.09	0.008381
A_44_P374860	Flot2	NM_031830	83764	0.32	2.09	0.000368
A_44_P865831	BF523591	BF523591	293128	0.32	2.09	0.030642
A_42_P544648	Rps16	XM_341815	140655	0.32	2.09	0.003224
A_44_P920548	CO561291	CO561291		0.32	2.09	0.000539
A_43_P17478	Dtymk_predicted	XM_217478		0.32	2.09	0.001155
A_44_P367499	Dnajc2	NM_053776	116456	0.32	2.09	0.000467
A_44_P275059	Scyl2_predicted	XM_235050	314717	0.32	2.09	0.136709
A_43_P15982	Tspy	NM_022923	25223	0.32	2.09	0.044735
A_44_P257863	ENSRNOT00000037938	ENSRNOT00000037938		0.32	2.09	0.001635
A_42_P580844	Cdc2a	NM_019296	54237	0.32	2.09	0.005233
A_44_P222799	Nin_predicted	XM_216723		0.32	2.08	0.006095
A_44_P105878	Cul2_predicted	XM_341542		0.32	2.08	0.006462
A_44_P313077	DV725436	DV725436		0.32	2.08	0.013076
A_44_P991273	Rad23b	NM_001025275	298012	0.32	2.08	0.000777
A_44_P182221	Gstp1	NM_012577	29438	0.32	2.08	0.000169
A_44_P304560	RGD1565093_predicted	XM_342164		0.32	2.08	0.000095
A_42_P548520	Pyp_mapped	XM_215416	294504	0.32	2.08	0.005663
A_44_P316959	AI112954	AI112954	287644	0.32	2.08	0.015069
A_43_P21455	Lama2_predicted	XM_219866	309368	0.32	2.08	0.105647
A_44_P497282	Rab11fip4_predicted	XM_220737		0.32	2.08	0.010758
A_43_P20917	Rtf1_predicted	XM_345421		0.32	2.08	0.013018
A_44_P976979	TC561570	TC561570		0.32	2.08	0.01523
A_44_P556531	Emilin1_predicted	XM_238447		0.32	2.08	0.020084
A_42_P674821	TC523661	TC523661		0.32	2.08	0.051142
A_44_P231628	Snapc2	NM_001013121	304204	0.32	2.08	0.000234
A_44_P372710	Map4k2_predicted	XM_219531		0.32	2.08	0.000762
A_44_P370500	Moap1	NM_001013101	299261	0.32	2.08	0.029535
A_44_P464042	RGD1304646_predicted	XM_224449	306103	0.32	2.08	0.004377
A_44_P109680	U78140	U78140	316096	0.32	2.08	0.009851
A_44_P546267	A_44_P546267	A_44_P546267		0.32	2.08	0.000998
A_44_P108063	Rhbdf1	NM_001030034	303008	0.32	2.08	0.000488
A_44_P448461	Aqp4	NM_012825	25293	0.32	2.08	0.074775
A_44_P421764	Nav1_predicted	XM_001063421		0.32	2.08	0.000432
A_44_P295361	Tprkb	NM_001013926	297411	0.32	2.08	0.019187
A_44_P300637	Zfp131	XM_227104	310375	0.32	2.08	0.047049
A_44_P667171	Zfp84_predicted	XM_218463		0.32	2.08	0.002146
A_44_P443584	Ccdc43	XM_340915	360637	0.32	2.08	0.000297
A_44_P540669	Arnt2	NM_012781	25243	0.32	2.08	0.007824
A_44_P330576	LOC288750	NM_198727	288750	0.32	2.08	0.010836
A_44_P398003	Klrc3	NM_001029908	500338	0.32	2.08	0.012545
A_44_P699787	LOC686286	XM_001073291		0.32	2.08	0.00141
A_43_P17043	MGC94780	NM_001007007	361056	0.32	2.08	0.002058
A_44_P1011870	LOC686616	XM_001074875		0.32	2.08	0.000316
A_44_P363814	RGD1563141_predicted	XM_001076724		0.32	2.08	0.001926
A_44_P117234	RGD1306698_predicted	XM_235527	315173	0.32	2.08	0.001188
A_44_P434158	Alg3	NM_001011897	287983	0.32	2.08	0.001315
A_44_P652863	TC534641	TC534641		0.32	2.08	0.00013
A_44_P456492	Dscr1	NM_153724	266766	0.32	2.08	0.027518
A_44_P546296	H6pd_predicted	XM_233688		0.32	2.08	0.000449
A_44_P1024529	Sfrs2	NM_001009720	494445	0.32	2.07	0.001688
A_44_P208981	AA925539	AA925539	308445	0.32	2.07	0.021367
A_44_P351780	Pols_predicted	XM_225072		0.32	2.07	0.000685
A_44_P900802	TC543724	TC543724		0.32	2.07	0.010651

A_42_P622878	Entpd4_predicted	XM_341346		0.32	2.07	0.003732
A_43_P15921	Ccnf	XM_340763		0.32	2.07	0.004755
A_44_P635178	RGD1307018	XM_001078459		0.32	2.07	0.000348
A_44_P498347	RGD1560606_predicted	XM_235023	314713	0.32	2.07	0.011285
A_43_P16460	AI598357	AI598357	287734	0.32	2.07	0.048843
A_42_P673973	RGD1311873	NM_001013982	304496	0.32	2.07	0.001399
A_44_P239281	Cdc6_predicted	XM_340896		0.32	2.07	0.002897
A_44_P448431	Cd8a	NM_031538	24930	0.32	2.07	0.003749
A_44_P623094	Prpsap1	NM_022545	64390	0.32	2.07	0.00097
A_44_P1044830	Lactb_predicted	XM_217181		0.32	2.07	0.011371
A_44_P158420	Impdh2	NM_199099	301005	0.32	2.07	0.001019
A_44_P117148	Baz1a_predicted	XM_234156	314126	0.32	2.07	0.001115
A_44_P499545	Tmem106c	NM_001008358	315286	0.32	2.07	0.00685
A_43_P13444	Kctd11_predicted	XM_343923		0.32	2.07	0.006241
A_44_P932768	TC534706	TC534706		0.32	2.07	0.000902
A_42_P579174	Cd3d	NM_013169	25710	0.32	2.07	0.025538
A_44_P779609	Zdhhc6	NM_001037652	361771	0.32	2.07	0.000993
A_44_P686269	TC530637	TC530637		0.32	2.07	0.03431
A_44_P1034346	RGD1307752	NM_001013922	296315	0.32	2.07	0.000683
A_44_P944991	TC519738	TC519738		0.32	2.07	0.00702
A_44_P149588	Papolg_predicted	XM_223690		0.32	2.07	0.012464
A_42_P665848	Tm4sf12	NM_001015026	362326	0.32	2.07	0.024698
A_44_P543631	A_44_P543631	A_44_P543631		0.32	2.07	0.000135
A_44_P852666	A_44_P852666	A_44_P852666		0.32	2.07	0.000783
A_44_P466376	RGD1307201_predicted	XM_341327		0.32	2.07	0.004893
A_44_P480533	A_44_P480533	A_44_P480533		0.32	2.07	0.000902
A_44_P1032161	Chek2	NM_053677	114212	0.32	2.07	0.000601
A_44_P620300	BG671963	BG671963		0.32	2.07	0.008916
A_44_P231724	XM_219278	XM_219278		0.32	2.07	0.000183
A_44_P996521	LOC681300	XM_001061130		0.32	2.07	0.000756
A_44_P824241	TC544869	TC544869		0.32	2.07	0.018133
A_44_P356428	RGD1309886	NM_001033896	310626	0.32	2.07	0.004097
A_42_P755445	RGD1304726	NM_001024993	303518	0.32	2.07	0.005286
A_44_P112606	Cops7b_predicted	XM_343614		0.32	2.07	0.002293
A_44_P322500	XM_215270	XM_215270		0.32	2.07	0.005703
A_44_P163689	XM_341356	XM_341356		0.32	2.07	0.076721
A_44_P346913	RGD1563402_predicted	XM_219543		0.32	2.07	0.001103
A_44_P200065	X82202	X82202		0.32	2.07	0.000984
A_44_P297880	Card10_predicted	XM_243622	315120	0.32	2.07	0.006162
A_44_P148370	Plagl2_predicted	XM_230745		0.32	2.07	0.022584
A_43_P14616	DV727304	DV727304		0.32	2.07	0.018487
A_44_P807993	Rnasen	XM_001058381		0.32	2.07	0.014525
A_44_P997826	Rnpep	NM_031097	81761	0.32	2.07	0.000266
A_42_P468632	Rps28	XM_001079210		0.32	2.07	0.001667
A_43_P19634	Chst1	NM_001011955	295934	0.31	2.07	0.00329
A_44_P272404	LOC686616	XM_001074875		0.31	2.06	0.000351
A_44_P856900	RGD1560399_predicted	XM_573998		0.31	2.06	0.001442
A_44_P527376	Ate1_predicted	XM_001077921		0.31	2.06	0.003492
A_44_P962266	TC554566	TC554566		0.31	2.06	0.035008
A_44_P535742	Hspd1	NM_022229	63868	0.31	2.06	0.000263
A_44_P103015	Nanos3_predicted	XM_222459		0.31	2.06	0.007229
A_44_P423786	Eef1g	XM_574616		0.31	2.06	0.000937
A_43_P15738	Pea15	NM_001013231	364052	0.31	2.06	0.000321
A_44_P702668	Tal1_predicted	XM_233430		0.31	2.06	0.004705
A_44_P470884	Prc1_predicted	XM_218820		0.31	2.06	0.003891
A_44_P212887	Mapkapk3	NM_001012127	315994	0.31	2.06	0.005093
A_43_P10723	Bub1b	XM_342494	171576	0.31	2.06	0.003547
A_44_P765162	TC529350	TC529350		0.31	2.06	0.090592
A_44_P114393	Ptar1	XM_239074		0.31	2.06	0.011552
A_44_P346832	RGD1566317_predicted	XM_213790	288689	0.31	2.06	0.049422
A_43_P19573	Sass6_predicted	XM_227619		0.31	2.06	0.003623
A_44_P391672	Siglec1_predicted	XM_230608		0.31	2.06	0.020406
A_44_P149199	C1qbp	NM_019259	29681	0.31	2.06	0.000675

A_44_P292327	G6pdx	NM_017006	24377	0.31	2.06	0.001641
A_44_P405203	Hebp2_predicted	XM_218664		0.31	2.06	0.011005
A_44_P389527	Ppp1r8_predicted	XM_232739		0.31	2.06	0.000591
A_44_P416641	Ywhaz	NM_013011	25578	0.31	2.06	0.002081
A_44_P130732	LOC682999	XM_001064066		0.31	2.06	0.001124
A_44_P179282	Fkbp4	XM_342763	260321	0.31	2.06	0.001174
A_44_P1049275	CB605666	CB605666		0.31	2.06	0.002967
A_44_P212102	AI044349	AI044349		0.31	2.06	0.034212
A_43_P21641	Hcls1	NM_001011898	288077	0.31	2.06	0.009398
A_44_P135170	Ywhae	NM_031603	29753	0.31	2.06	0.00832
A_42_P632544	RGD1564058_predicted	XM_213835		0.31	2.06	0.000926
A_44_P247408	Aprin_predicted	XM_221833		0.31	2.06	0.003217
A_43_P12161	Gtf2a1	NM_022208	83830	0.31	2.05	0.015062
A_42_P773604	Tesk1	NM_031578	29460	0.31	2.05	0.039129
A_44_P187414	XM_341465	XM_341465		0.31	2.05	0.037092
A_44_P367438	Stk38	NM_001015025	361813	0.31	2.05	0.003047
A_44_P248714	RGD1305628_predicted	XM_213639	288204	0.31	2.05	0.042297
A_44_P370625	XM_213016	XM_213016		0.31	2.05	0.001897
A_43_P19502	Lrrc28_predicted	XM_341870		0.31	2.05	0.001867
A_44_P351943	Nfx1	XM_001059410		0.31	2.05	0.001031
A_44_P465281	AW142556	AW142556	58820	0.31	2.05	0.004421
A_44_P776350	TC553984	TC553984		0.31	2.05	0.099759
A_44_P506017	Hist1h2bn_predicted	XM_214483		0.31	2.05	0.022278
A_44_P231351	Olr1163_predicted	NM_001000869	405166	0.31	2.05	0.000909
A_44_P866704	Ap1gbp1	XM_001081085		0.31	2.05	0.013922
A_44_P442169	Glrx2	NM_001013034	114022	0.31	2.05	0.003082
A_44_P916687	TC528669	TC528669		0.31	2.05	0.009424
A_43_P22310	RGD1304624_predicted	XM_234159		0.31	2.05	0.008699
A_44_P698564	A_44_P698564	A_44_P698564		0.31	2.05	0.001193
A_42_P731541	Psmb5	XM_341314		0.31	2.05	0.00024
A_44_P529718	RGD1310509_predicted	XM_341914		0.31	2.05	0.013392
A_44_P789773	AW917283	AW917283		0.31	2.05	0.049487
A_44_P1028858	Grn	NM_017113	29143	0.31	2.05	0.001942
A_44_P126613	LOC681650	XM_001060078		0.31	2.05	0.000653
A_44_P135896	XM_216910	XM_216910		0.31	2.05	0.000272
A_44_P507943	AW140408	AW140408	24511	0.31	2.05	0.000222
A_44_P312309	Eif3s10	XM_238649		0.31	2.05	0.000287
A_44_P531727	LOC367195	XM_001078509		0.31	2.05	0.001139
A_44_P652658	Brd3_predicted	XM_001075609		0.31	2.05	0.016766
A_42_P640277	Enc1	NM_001003401	294674	0.31	2.05	0.003658
A_42_P453935	Naca_predicted	XM_213821		0.31	2.05	0.001086
A_43_P15345	Plod1	NM_053827	116552	0.31	2.05	0.001678
A_44_P1020428	Nes	NM_012987	25491	0.31	2.05	0.135661
A_42_P491376	RGD1566016_predicted	XM_341116	360840	0.31	2.05	0.00054
A_44_P461154	Timp1	NM_053819	116510	0.31	2.05	0.000505
A_43_P17203	BF523141	BF523141	361554	0.31	2.05	0.001034
A_44_P423803	Actb	NM_031144	81822	0.31	2.05	0.015194
A_44_P422182	LOC686736	XM_001078389		0.31	2.05	0.000377
A_44_P424295	Pabpc2_predicted	XM_225992		0.31	2.05	0.00902
A_44_P445536	Narg1_predicted	XM_241375		0.31	2.05	0.003004
A_44_P283835	Exosc2_predicted	XM_345336		0.31	2.05	0.004767
A_44_P1018749	BF290252	BF290252	116967	0.31	2.05	0.024502
A_44_P553297	RGD1561055_predicted	XM_577041		0.31	2.05	0.004503
A_44_P426707	RGD1560511_predicted	XM_001053199		0.31	2.05	0.013882
A_43_P23203	Abhd3_predicted	XM_214618		0.31	2.05	0.039538
A_44_P856056	RGD1311563	XM_343499		0.31	2.04	0.017639
A_44_P267106	RGD1564883_predicted	XM_231785	312363	0.31	2.04	0.000302
A_44_P732301	TC566188	TC566188		0.31	2.04	0.000268
A_43_P13554	LOC680280	XM_001056437	680280	0.31	2.04	0.000116
A_44_P538406	Fkbp1a	NM_013102	25639	0.31	2.04	0.000131
A_44_P288108	Abcf1	XM_001056151		0.31	2.04	0.001014
A_44_P441947	LOC687711	XM_001079870		0.31	2.04	0.000172
A_44_P361283	Fam51a1	NM_001007756	363462	0.31	2.04	0.011734

A_44_P480630	Chd6_predicted	XM_230814		0.31	2.04	0.005057
A_44_P469584	Cd14	NM_021744	60350	0.31	2.04	0.002267
A_44_P135413	A_44_P135413	A_44_P135413		0.31	2.04	0.038233
A_44_P322118	Rps7	NM_031570	29258	0.31	2.04	0.000647
A_44_P791176	RGD1564842_predicted	XM_576307	500906	0.31	2.04	0.12068
A_44_P730677	RGD1311517	NM_001014072	313775	0.31	2.04	0.001093
A_44_P503490	Bub1b	XM_342494	171576	0.31	2.04	0.018724
A_44_P493270	Rgc32	XM_001072931		0.31	2.04	0.017839
A_44_P309202	Slc25a22	NM_001014027	309111	0.31	2.04	0.015799
A_44_P294687	Cdk5	NM_080885	140908	0.31	2.04	0.018142
A_44_P260542	XM_346356	XM_346356		0.31	2.04	0.002939
A_44_P1007965	RGD1305961_predicted	XM_216666		0.31	2.04	0.003672
A_44_P301633	Zfp191	NM_182955	360204	0.31	2.04	0.008194
A_44_P497977	AA998448	AA998448	64387	0.31	2.04	0.016845
A_44_P785427	Aer61	NM_001009502	494219	0.31	2.04	0.000415
A_44_P123788	LOC293589	XM_001056818		0.31	2.04	0.000081
A_44_P401284	Rnf19_predicted	XM_343228	362900	0.31	2.04	0.003303
A_42_P528083	Trmt5_predicted	XM_343080		0.31	2.04	0.002961
A_44_P313918	RGD1308513	NM_001014231	364050	0.31	2.04	0.003898
A_44_P513994	Rpl10	NM_031100	81764	0.31	2.04	0.000297
A_44_P376236	AI171162	AI171162	685433	0.31	2.04	0.067436
A_43_P15341	Tdg	NM_053729	114521	0.31	2.04	0.000614
A_44_P138224	Vezt	NM_001006984	299738	0.31	2.04	0.043538
A_44_P651866	ENSRNOT00000033065	ENSRNOT00000033065		0.31	2.04	0.00164
A_44_P636043	LOC287522	XM_220702		0.31	2.04	0.011898
A_43_P15614	Pfkp	L25387		0.31	2.04	0.003542
A_44_P915286	TC523542	TC523542		0.31	2.04	0.032521
A_42_P618436	RGD1305138_predicted	XM_235689	315329	0.31	2.04	0.000971
A_43_P11860	Mecr	NM_017209	29470	0.31	2.04	0.075713
A_44_P383310	Gata3	NM_133293	85471	0.31	2.04	0.001963
A_43_P11985	Ccl20	NM_019233	29538	0.31	2.04	0.075792
A_44_P349356	Tnpo1	XM_219500	309126	0.31	2.04	0.019376
A_43_P10476	Cops5	NM_001025695	312916	0.31	2.04	0.001522
A_44_P426672	Bcas2_predicted	XM_215664		0.31	2.04	0.005322
A_44_P340375	RGD1564209_predicted	XM_345912	367046	0.31	2.04	0.000386
A_44_P393442	Mtap6	NM_017204	29457	0.31	2.04	0.004411
A_44_P433964	RGD1311648	NM_001014075	313949	0.31	2.04	0.005741
A_44_P521163	LOC680726	XM_001061274	680726	0.31	2.04	0.001921
A_44_P411392	DV726507	DV726507		0.31	2.04	0.086724
A_44_P483177	Tbrg1	NM_001009344	300521	0.31	2.04	0.002581
A_43_P12413	Cyba	NM_024160	79129	0.31	2.04	0.005645
A_44_P932384	Plxnc1_predicted	XM_343200		0.31	2.03	0.024871
A_44_P110091	RGD1562699_predicted	XM_238022		0.31	2.03	0.002921
A_44_P577274	TC524552	TC524552		0.31	2.03	0.00908
A_44_P403621	Sgkl	XM_001060531		0.31	2.03	0.011403
A_44_P760650	RGD1562823_predicted	XM_576310	500907	0.31	2.03	0.000336
A_42_P801379	Fgfrl1	NM_199114	360903	0.31	2.03	0.009594
A_44_P475197	Fgfr1	NM_024146	79114	0.31	2.03	0.006081
A_44_P273796	Krim1	NM_182823	314587	0.31	2.03	0.019286
A_43_P17457	Cd163_predicted	XM_232342		0.31	2.03	0.1341
A_44_P496706	Ikbke_predicted	XM_344139		0.31	2.03	0.009794
A_42_P563376	Pcyox1	NM_145085	246302	0.31	2.03	0.005553
A_44_P102661	Spata7	NM_138862	192225	0.31	2.03	0.016747
A_44_P373076	Nol10	NM_001014076	313981	0.31	2.03	0.001159
A_44_P913155	AW915016	AW915016	689765	0.31	2.03	0.002719
A_44_P1018464	Prss25	XM_001054494		0.31	2.03	0.000322
A_44_P365807	Snx7	NM_001012083	310815	0.31	2.03	0.007312
A_42_P641234	Snrpa	NM_001008303	292729	0.31	2.03	0.002204
A_44_P290915	Sema3c_predicted	XM_231381	296787	0.31	2.03	0.006703
A_44_P640131	TC531581	TC531581		0.31	2.03	0.008982
A_44_P327958	Olr1521_predicted	NM_001000039	287613	0.31	2.03	0.004312
A_44_P264460	Xrcc5	NM_177419	363247	0.31	2.03	0.003489
A_44_P262791	Ddx27	XM_342582	362274	0.31	2.03	0.00048

A_44_P313625	LOC361750	AY321330		0.31	2.03	0.0005
A_44_P640421	TC568193	TC568193		0.31	2.03	0.00988
A_43_P17545	XM_234303	XM_234303		0.31	2.03	0.004572
A_44_P370255	Pgam5	NM_001025272	288731	0.31	2.03	0.000855
A_44_P1040584	Med6_predicted	XM_216759		0.31	2.03	0.004865
A_42_P843394	Pcolce	NM_019237	29569	0.31	2.03	0.017663
A_43_P22858	LOC681234	XM_001058421		0.31	2.03	0.060126
A_44_P226553	AW917515	AW917515		0.31	2.03	0.174134
A_44_P213407	RGD1562602_predicted	XM_236296	315713	0.31	2.03	0.003492
A_44_P944227	A_44_P944227	A_44_P944227		0.31	2.03	0.000221
A_44_P252775	LOC680615	XM_001058007		0.31	2.03	0.024267
A_44_P1003333	Chst12	NM_001037775	304322	0.31	2.03	0.118501
A_44_P729229	LOC366863	XR_009322	366863	0.31	2.03	0.006396
A_43_P19944	Upk3a_predicted	XM_235546	315190	0.31	2.03	0.012554
A_44_P160529	AW915849	AW915849	294292	0.31	2.03	0.00048
A_44_P404843	Cxcr3	NM_053415	84475	0.31	2.03	0.003122
A_42_P489223	RGD1560519_predicted	XM_233829	313865	0.31	2.03	0.001753
A_44_P470528	Hrpap20	NM_198783	362495	0.31	2.03	0.009855
A_44_P506392	Abce1	XM_341669		0.31	2.03	0.001705
A_44_P396239	Zfp64	NM_001012093	311661	0.31	2.03	0.003728
A_44_P1032051	LOC687565	XM_001079167		0.31	2.03	0.002801
A_43_P17929	LOC689844	XM_001072241	689844	0.31	2.02	0.004381
A_44_P622201	BC098778	BC098778		0.31	2.02	0.011918
A_44_P190226	Med31_predicted	XM_213393	287475	0.31	2.02	0.002088
A_44_P725696	AI070470	AI070470		0.31	2.02	0.005828
A_44_P249902	Vps29_predicted	XM_213780		0.31	2.02	0.001605
A_44_P817696	Echdc1	NM_001007734	361465	0.31	2.02	0.050239
A_44_P1014445	RGD1562173_predicted	XM_576402	500989	0.31	2.02	0.000711
A_44_P944757	Hnrpa1	NM_017248	29578	0.31	2.02	0.000214
A_44_P485227	RGD1309747_predicted	XM_225143	306665	0.31	2.02	0.002173
A_44_P274924	LOC301131	XM_236787	301131	0.31	2.02	0.000349
A_44_P930446	LOC685088	XM_001062249	685088	0.31	2.02	0.072301
A_42_P705866	RGD1304861	NM_001013971	303419	0.31	2.02	0.002832
A_44_P242756	Pcna	NM_022381	25737	0.31	2.02	0.00165
A_44_P636641	LOC681460	XM_001056922		0.31	2.02	0.003206
A_44_P1035190	Bub1_predicted	XM_215849		0.31	2.02	0.012071
A_42_P833613	Pi4k2a	NM_053735	114554	0.31	2.02	0.001046
A_44_P308724	Maged2	NM_080479	113947	0.31	2.02	0.002702
A_44_P245886	Spata5_predicted	XM_342235		0.31	2.02	0.005213
A_44_P101052	Thap7	XM_213578		0.31	2.02	0.000959
A_44_P621304	LOC502174	XR_008116	502174	0.31	2.02	0.001961
A_42_P720077	Fmo5	NM_144739	246248	0.31	2.02	0.013467
A_42_P702242	Sema4d_predicted	XM_225215	306790	0.31	2.02	0.000889
A_44_P121015	Tdg	NM_053729	114521	0.31	2.02	0.000859
A_44_P854406	A_44_P854406	A_44_P854406		0.31	2.02	0.001139
A_42_P631453	Matn2_predicted	XM_216941	299996	0.31	2.02	0.030655
A_44_P506464	XM_237259	XM_237259		0.31	2.02	0.000304
A_44_P238246	Ilf3	NM_053412	84472	0.31	2.02	0.000986
A_42_P606126	Mmp13	XM_001072242		0.30	2.02	0.10537
A_42_P739721	LOC296637	XM_216035	296637	0.30	2.02	0.00105
A_44_P464027	XM_226645	XM_226645		0.30	2.02	0.000724
A_44_P668278	TC554028	TC554028		0.30	2.02	0.016983
A_43_P13533	TC556180	TC556180		0.30	2.02	0.000278
A_44_P415010	Mcm2_predicted	XM_232168		0.30	2.02	0.003963
A_43_P20568	RGD1311526_predicted	XM_001070243		0.30	2.02	0.000785
A_44_P282004	LOC679383	XM_001056099		0.30	2.02	0.028947
A_44_P353505	Atpbd1c	NM_201991	360810	0.30	2.01	0.005557
A_44_P344599	Tead1	XM_001069278	361630	0.30	2.01	0.000304
A_43_P16505	RGD1311316	NM_001012182	362007	0.30	2.01	0.015607
A_44_P607583	RGD1359191	NM_001007706	314462	0.30	2.01	0.003455
A_44_P282826	BF558098	BF558098		0.30	2.01	0.001912
A_44_P879104	Ash1l_predicted	XM_001074246		0.30	2.01	0.133766
A_44_P550497	Bid	NM_022684	64625	0.30	2.01	0.00071

A_44_P173355	XM_227945	XM_227945		0.30	2.01	0.000398
A_44_P448972	AI412152	AI412152	360928	0.30	2.01	0.002662
A_44_P240832	ENSRNOT00000006311	ENSRNOT00000006311		0.30	2.01	0.001413
A_44_P775665	ENSRNOT00000050671	ENSRNOT00000050671		0.30	2.01	0.069477
A_44_P213208	Otud4	XM_226388	307774	0.30	2.01	0.000658
A_44_P694872	Fubp1	NM_001037653	654496	0.30	2.01	0.004099
A_42_P552640	Mmp16	NM_080776	65205	0.30	2.01	0.006377
A_44_P1036430	Usp14	NM_001008301	291796	0.30	2.01	0.000859
A_44_P489782	XM_212905	XM_212905		0.30	2.01	0.000216
A_44_P555028	LOC291840	NM_001003705	291840	0.30	2.01	0.000602
A_44_P1030648	LOC687694	XM_001079792		0.30	2.01	0.009496
A_44_P345161	Qtrtd1_predicted	XM_221768	288364	0.30	2.01	0.00675
A_44_P527185	Gtf3c1	NM_133541	171063	0.30	2.01	0.001741
A_44_P945846	CO555049	CO555049	362728	0.30	2.01	0.001741
A_44_P311963	RGD1560468_predicted	XM_575939	500572	0.30	2.01	0.002465
A_44_P325522	Lmnb1	NM_053905	116685	0.30	2.01	0.001974
A_42_P757258	Mphosph1_predicted	XM_220055		0.30	2.01	0.077106
A_44_P477555	Ns5atp9	NM_201418	300795	0.30	2.01	0.002992
A_44_P507813	RGD1304825_predicted	XM_001074564		0.30	2.01	0.000431
A_44_P102481	Sox9	XM_001081628		0.30	2.01	0.000546
A_44_P358797	RGD1566352_predicted	XR_007663	291028	0.30	2.01	0.000179
A_44_P172343	DV727576	DV727576	293967	0.30	2.01	0.035138
A_44_P181738	BI395523	BI395523		0.30	2.01	0.016257
A_43_P13136	Fgf3	NM_130817	170633	0.30	2.01	0.035761
A_44_P370817	Kb24	NM_001008812	315320	0.30	2.01	0.096796
A_43_P20107	Rfx3	NM_001012172	361746	0.30	2.01	0.002156
A_42_P574731	Emd	NM_012948	25437	0.30	2.01	0.000475
A_44_P222551	Hist2h2aa_predicted	XM_345255		0.30	2.01	0.039813
A_43_P17149	Polr3h_predicted	XM_216998	300088	0.30	2.01	0.000417
A_44_P425450	AI535429	AI535429		0.30	2.01	0.000354
A_43_P13177	Ilk	NM_133409	170922	0.30	2.01	0.000547
A_44_P299776	XM_214160	XM_214160		0.30	2.01	0.000637
A_43_P12961	Eif4ebp1	NM_053857	116636	0.30	2.01	0.00274
A_44_P1012333	BC090353	BC090353		0.30	2.01	0.020683
A_44_P543733	ENSRNOT00000036613	ENSRNOT00000036613		0.30	2.01	0.005375
A_44_P466595	LOC297481	XM_216226		0.30	2.01	0.180533
A_44_P1014086	RGD1309995_predicted	XM_235003	314690	0.30	2.00	0.005421
A_44_P417267	Thap11_predicted	XM_226417		0.30	2.00	0.002353
A_44_P196291	Lamp2	NM_017068	24944	0.30	2.00	0.008755
A_44_P119575	LOC501026	XM_576437	501026	0.30	2.00	0.001046
A_44_P328323	RGD1564808_predicted	XM_214034	289584	0.30	2.00	0.000844
A_44_P450128	Cherp_predicted	XM_214307		0.30	2.00	0.003444
A_44_P384184	AY331040	AY331040		0.30	2.00	0.026032
A_44_P557571	RGD1311012_predicted	XM_344792		0.30	2.00	0.041148
A_44_P242721	Tob2	NM_001007146	315159	0.30	2.00	0.009648
A_43_P23455	Dhx33_predicted	XM_213370		0.30	2.00	0.016055
A_43_P14566	Aatf	NM_053720	114512	0.30	2.00	0.000295
A_43_P14978	AW918202	AW918202		0.30	2.00	0.005012
A_44_P547209	Datf1_predicted	XM_001060118		0.30	2.00	0.03539
A_44_P172950	Hist1h2an_predicted	XM_225386		0.30	2.00	0.025285
A_44_P260725	RGD1306595	NM_001025626	287554	0.30	2.00	0.000312
A_44_P557474	Pitpnb	NM_053742	114561	0.30	2.00	0.002472
A_44_P176501	RGD1306106	NM_001009702	360895	0.30	1.999	0.002073
A_44_P329090	Tm4sf12	NM_001015026	362326	0.30	1.998	0.010254
A_44_P440842	Setdb1_predicted	XM_001060316		0.30	1.998	0.004947
A_42_P546775	RGD1305264_predicted	XM_216644		0.30	1.998	0.00013
A_44_P536806	Psmd7_predicted	XM_226439		0.30	1.998	0.002073
A_44_P335603	RGD1311424_predicted	XM_343001		0.30	1.997	0.000584
A_42_P674018	LOC679140	XM_001054866		0.30	1.997	0.001309
A_44_P838798	RGD1559552_predicted	XM_573188	497991	0.30	1.997	0.00756
A_43_P17256	LOC681542	XM_001057364		0.30	1.997	0.001065
A_44_P127156	M55017	M55017		0.30	1.996	0.000428
A_44_P224991	XM_226023	XM_226023		0.30	1.996	0.000184

A_43_P10914	XM_228865	XM_228865		0.30	1.996	0.000903
A_44_P493956	Pdia4	NM_053849	116598	0.30	1.996	0.002593
A_44_P1001963	AI556258	AI556258		0.30	1.996	0.003547
A_44_P997079	RGD1308881_predicted	XM_220981	303542	0.30	1.996	0.016198
A_43_P15423	Mgat3	NM_019239	29582	0.30	1.995	0.00397
A_42_P783292	H2afy	NM_017182	29384	0.30	1.995	0.004106
A_44_P277068	RGD1562399_predicted	XM_217412		0.30	1.995	0.000163
A_44_P475371	Ccnc	XM_001058860		0.30	1.995	0.043179
A_44_P363484	Atp13a1_predicted	XM_214310		0.30	1.995	0.0009
A_44_P196424	RGD708545	NM_020080	56769	0.30	1.995	0.009166
A_44_P234564	LOC363544	NM_001014225	363544	0.30	1.994	0.000998
A_44_P394636	Inpp5d	NM_019311	54259	0.30	1.994	0.000463
A_42_P714281	Ccdc58_predicted	XM_213612		0.30	1.994	0.000421
A_44_P213062	Dcp1a_predicted	XM_341395	361109	0.30	1.994	0.021677
A_44_P825566	Tubb4	XM_001059513		0.30	1.994	0.003192
A_44_P829047	CO402576	CO402576		0.30	1.994	0.00213
A_44_P944832	BG671311	BG671311	291434	0.30	1.994	0.000812
A_44_P290966	Pcdh19_predicted	XM_228429	317183	0.30	1.993	0.018145
A_44_P839339	LOC298977	XM_216701	298977	0.30	1.993	0.001229
A_43_P12801	Ilf3	NM_053412	84472	0.30	1.993	0.000333
A_43_P18264	Zfhx2	XM_001054926		0.30	1.993	0.002435
A_44_P778323	TC527564	TC527564		0.30	1.992	0.005141
A_44_P128427	RGD1309501_predicted	XM_223660	305552	0.30	1.992	0.040273
A_44_P250371	A_44_P250371	A_44_P250371		0.30	1.992	0.002529
A_44_P561626	Ube2q2_predicted	XM_001072896		0.30	1.991	0.002041
A_42_P668972	Acp1	NM_021262	24161	0.30	1.990	0.000476
A_44_P222765	XM_342901	XM_342901		0.30	1.990	0.000201
A_44_P400922	Rad52_predicted	XM_216230		0.30	1.990	0.056197
A_44_P348973	Lypla3	NM_001004277	361401	0.30	1.990	0.00201
A_44_P168531	XM_227173	XM_227173		0.30	1.989	0.003162
A_42_P701855	Slc4a11_predicted	XM_230605		0.30	1.989	0.007399
A_44_P374068	Pdlim7	NM_173125	286908	0.30	1.989	0.002612
A_43_P14872	Sdc1	NM_013026	25216	0.30	1.989	0.000993
A_44_P925669	AW917673	AW917673	690795	0.30	1.988	0.009791
A_44_P320312	Rbm13	NM_001014002	306526	0.30	1.988	0.000191
A_44_P539724	Clpb	NM_022947	65041	0.30	1.988	0.008865
A_44_P477879	Cit	NM_001029911	83620	0.30	1.988	0.001623
A_44_P210440	Akap13	XM_214969		0.30	1.988	0.003814
A_42_P792971	Ccl25	NM_001037203	360750	0.30	1.988	0.005323
A_44_P871871	TC542475	TC542475		0.30	1.988	0.001511
A_44_P132470	Pfn1	NM_022511	64303	0.30	1.987	0.001092
A_44_P311821	Afg3l1_predicted	XM_341714		0.30	1.987	0.000422
A_44_P548299	Thumpd1	NM_001009688	309041	0.30	1.986	0.001116
A_44_P761087	Ets2_mapped	XM_001053903		0.30	1.986	0.001185
A_44_P194925	Pigx	XM_213602		0.30	1.986	0.000651
A_44_P955940	Dhx36_predicted	XM_001058645		0.30	1.985	0.003621
A_44_P707055	TC517924	TC517924		0.30	1.985	0.032799
A_44_P321346	ENSRNOT00000023913	ENSRNOT00000023913		0.30	1.985	0.000389
A_43_P15681	LOC687849	XM_001080339		0.30	1.985	0.000201
A_44_P526056	Hnrpk	NM_057141	117282	0.30	1.985	0.001628
A_43_P20282	Kpna4	NM_001014793	361959	0.30	1.984	0.00488
A_42_P627101	AW920530	AW920530		0.30	1.984	0.001831
A_42_P832417	Vsnl1	NM_012686	24877	0.30	1.984	0.002816
A_43_P11789	Ppp2cb	NM_017040	24673	0.30	1.983	0.001481
A_43_P10326	CF110545	CF110545		0.30	1.983	0.000389
A_44_P105506	A_44_P105506	A_44_P105506		0.30	1.982	0.003492
A_44_P407138	Nme1	NM_138548	191575	0.30	1.982	0.000558
A_44_P511202	Qrsl1	NM_001014034	309911	0.30	1.982	0.01957
A_44_P199553	Lrrc58_predicted	XM_221433	303919	0.30	1.982	0.000368
A_44_P171328	RGD1564820_predicted	XM_217404		0.30	1.982	0.006602
A_44_P251031	Trim16_predicted	XM_001078237		0.30	1.982	0.003037
A_42_P678430	Card9	NM_022303	64171	0.30	1.981	0.002679
A_44_P390615	AW915082	AW915082	29578	0.30	1.981	0.012589

A_43_P13409	Zdhhc2	NM_145096	246326	0.30	1.981	0.014712
A_44_P575533	Rpl31	NM_022506	64298	0.30	1.981	0.000202
A_43_P18045	RGD1559841_predicted	XM_226428	307816	0.30	1.981	0.000772
A_44_P283807	Adam9_predicted	NM_001014772	290834	0.30	1.980	0.005219
A_44_P426734	Polr2d_predicted	XM_344659		0.30	1.980	0.001605
A_44_P734807	TC553353	TC553353		0.30	1.980	0.098989
A_44_P466608	Usp5_predicted	XM_238380		0.30	1.980	0.001969
A_44_P760732	LOC689741	XM_001071836		0.30	1.980	0.02643
A_44_P435596	Zfp36	NM_133290	79426	0.30	1.980	0.067094
A_43_P23467	XM_225436	XM_225436		0.30	1.980	0.006649
A_42_P842946	Nol5a	NM_001025732	362214	0.30	1.980	0.000535
A_44_P375593	RGD1305001_predicted	XM_222526	304714	0.30	1.980	0.001201
A_44_P1011129	Csnk2a2_predicted	XM_226237		0.30	1.980	0.004098
A_44_P457368	RGD1561890_predicted	XM_233989		0.30	1.980	0.004379
A_44_P684073	TC538910	TC538910		0.30	1.980	0.038726
A_44_P1008463	RGD1311849_predicted	XM_001053590		0.30	1.980	0.004232
A_44_P100991	RGD1310352	XM_220404		0.30	1.980	0.001813
A_44_P790750	ENSRNOT00000019908	ENSRNOT00000019908		0.30	1.979	0.000478
A_44_P346963	XM_344373	XM_344373		0.30	1.979	0.010587
A_43_P13221	Ppp6c	NM_133589	171121	0.30	1.979	0.005762
A_44_P329944	AA945177	AA945177	64459	0.30	1.978	0.01464
A_44_P1011335	ENSRNOT00000013636	ENSRNOT00000013636		0.30	1.978	0.001015
A_44_P196922	Cdyl2_predicted	XM_226510		0.30	1.978	0.002522
A_43_P11734	Gnai1	NM_013145	25686	0.30	1.978	0.251266
A_44_P224614	Tcf21	NM_001032397	252856	0.30	1.978	0.003047
A_44_P542785	CO387330	CO387330	289623	0.30	1.978	0.042333
A_44_P138269	RGD1305776	NM_001025703	315160	0.30	1.978	0.001605
A_42_P789727	Rpl36	NM_022504	58927	0.30	1.978	0.000278
A_44_P100584	Il7r_predicted	XM_226824		0.30	1.977	0.080495
A_42_P719478	RGD1560606_predicted	XM_235023	314713	0.30	1.977	0.004982
A_44_P382312	RGD1306056_predicted	XM_230961	296474	0.30	1.977	0.002075
A_43_P10391	Ppp3r1	NM_017309	29748	0.30	1.976	0.000847
A_44_P700958	CO554673	CO554673		0.30	1.976	0.000534
A_44_P365611	Gpr31_predicted	XM_217867		0.30	1.976	0.014839
A_44_P610089	TC558537	TC558537		0.30	1.976	0.00306
A_44_P792845	TC541525	TC541525		0.30	1.974	0.017696
A_44_P540720	Plekhg5	NM_201272	310999	0.30	1.974	0.000455
A_44_P513853	Nxn_predicted	XM_340857		0.30	1.974	0.00122
A_44_P387344	RGD1561464_predicted	XM_220750	303348	0.30	1.973	0.003618
A_44_P288536	LOC688712	XM_001068022		0.30	1.972	0.001041
A_44_P225891	Nf1	NM_012609	24592	0.29	1.972	0.003659
A_43_P18735	Cpsf3	NM_001030030	298916	0.29	1.972	0.000757
A_44_P1009757	Il1rap	NM_012968	25466	0.29	1.972	0.001945
A_44_P790393	LOC681708	XM_001058055		0.29	1.972	0.000456
A_44_P440600	Gng11	NM_022396	64199	0.29	1.972	0.180903
A_44_P443008	Mrpl46	NM_001013068	293054	0.29	1.972	0.002757
A_44_P408504	Orc2l	NM_001012003	301430	0.29	1.971	0.00207
A_44_P410000	Stx18	NM_001012151	360953	0.29	1.971	0.01164
A_42_P642757	Pard3	NM_031235	81918	0.29	1.971	0.000833
A_44_P737376	CV076506	CV076506		0.29	1.971	0.001073
A_43_P15205	RGD1559787_predicted	XM_576514		0.29	1.971	0.000368
A_44_P550454	ENSRNOT00000008889	ENSRNOT00000008889		0.29	1.970	0.001412
A_42_P494979	AW919130	AW919130		0.29	1.970	0.044284
A_44_P384712	RGD1306507_predicted	XM_232785	313063	0.29	1.970	0.01695
A_44_P536769	RGD1565118_predicted	XM_225889	307395	0.29	1.970	0.060168
A_44_P452318	Zfp105	NM_001012128	316096	0.29	1.970	0.011568
A_44_P160550	Capn2	NM_017116	29154	0.29	1.970	0.003738
A_44_P506577	A_44_P506577	A_44_P506577		0.29	1.970	0.045348
A_44_P1044046	Ndufaf1_predicted	XM_215814		0.29	1.970	0.013064
A_44_P289697	Mettl6	NM_001007623	290564	0.29	1.969	0.001493
A_42_P689755	Pak2	NM_053306	29432	0.29	1.969	0.007921
A_44_P996006	Slc35b4_predicted	XM_216122		0.29	1.969	0.003917
A_44_P446817	RGD1561797_predicted	XM_579997	499655	0.29	1.969	0.005922

A_44_P182020	Psmb8	NM_080767	24968	0.29	1.969	0.002593
A_44_P920111	CF107866	CF107866	313019	0.29	1.969	0.011171
A_44_P508341	Gpx4	NM_017165	29328	0.29	1.969	0.014055
A_44_P1043081	LOC689890	XM_001072402		0.29	1.968	0.001162
A_44_P187476	Ckap5	XM_230282	311191	0.29	1.968	0.015182
A_44_P652351	A_44_P652351	A_44_P652351		0.29	1.968	0.001664
A_44_P321110	Eif3s8	XM_215080	293484	0.29	1.968	0.000844
A_44_P468141	Plaur	NM_134352	50692	0.29	1.967	0.006831
A_43_P18277	Npepps	XM_340889		0.29	1.967	0.033683
A_44_P513800	Trfp	NM_001013178	316209	0.29	1.967	0.006053
A_44_P391926	Ptp4a3_predicted	XM_343259	362930	0.29	1.966	0.006973
A_43_P11361	TC553641	TC553641		0.29	1.966	0.001065
A_43_P14318	Apex1	NM_024148	79116	0.29	1.966	0.001759
A_44_P398368	A_44_P398368	A_44_P398368		0.29	1.966	0.000531
A_44_P217967	LOC307783	XR_008978	307783	0.29	1.966	0.002288
A_44_P472996	Anxa5	NM_013132	25673	0.29	1.965	0.002156
A_43_P19830	Ythdf2_predicted	XM_232772		0.29	1.965	0.008093
A_44_P1053706	Nip7	NM_138847	192180	0.29	1.965	0.007457
A_44_P107653	LOC290396	XM_224392	290396	0.29	1.965	0.004205
A_44_P555343	RGD1305254_predicted	XM_218855	308797	0.29	1.964	0.026399
A_44_P304277	ENSRNOT00000024996	ENSRNOT00000024996		0.29	1.964	0.032371
A_43_P10284	TC535452	TC535452		0.29	1.964	0.006816
A_44_P283048	AW140450	AW140450	305851	0.29	1.964	0.004204
A_44_P354665	Zfp672	NM_001007669	303165	0.29	1.963	0.013182
A_43_P18107	RGD1307034_predicted	XM_221867		0.29	1.963	0.001508
A_44_P443572	RGD1561360_predicted	XM_220630	303270	0.29	1.962	0.000611
A_44_P210791	Ash2l_predicted	XM_214369		0.29	1.962	0.000751
A_44_P439573	DV728042	DV728042		0.29	1.962	0.002378
A_44_P198820	Gnl3	NM_175580	290556	0.29	1.962	0.003326
A_44_P334715	Klc3	NM_138520	171549	0.29	1.962	0.007846
A_44_P1034668	RGD1308066_predicted	XM_213289	287285	0.29	1.962	0.011071
A_44_P927439	Adam9_predicted	NM_001014772	290834	0.29	1.961	0.022103
A_44_P161579	LOC679550	XM_001053441		0.29	1.961	0.001773
A_44_P137996	Tcof1_predicted	XM_214552		0.29	1.961	0.000181
A_44_P960947	TC557241	TC557241		0.29	1.961	0.015054
A_43_P20941	Drg1	NM_001009685	305470	0.29	1.961	0.000831
A_42_P553201	Acp5	NM_019144	25732	0.29	1.960	0.007894
A_44_P386349	LOC362065	NM_199408	362065	0.29	1.960	0.000368
A_44_P335629	A_44_P335629	A_44_P335629		0.29	1.959	0.002181
A_43_P17012	RGD1307010	NM_001014227	363644	0.29	1.959	0.00091
A_43_P22903	Zfp192_predicted	XM_225349	306974	0.29	1.959	0.009538
A_44_P489168	BF562116	BF562116		0.29	1.959	0.013963
A_44_P673662	GalNAc4S6ST	NM_173310	286974	0.29	1.958	0.00369
A_44_P1043535	Raly	NM_001011958	296301	0.29	1.958	0.00016
A_42_P625220	Eif3s12_predicted	XM_214886		0.29	1.958	0.000514
A_44_P668620	Ccdc43	XM_001081522		0.29	1.958	0.000951
A_44_P224900	Tspan14_predicted	XM_224699	306324	0.29	1.957	0.000138
A_44_P297420	RGD1559927_predicted	XM_219327		0.29	1.957	0.028296
A_44_P197086	Rpusd4	NM_001025284	315550	0.29	1.957	0.003547
A_44_P683965	TC552897	TC552897		0.29	1.957	0.001882
A_43_P21225	Dixdc1	NM_001037654	363062	0.29	1.957	0.001573
A_43_P10614	TC536291	TC536291		0.29	1.957	0.001153
A_44_P866539	AW913986	AW913986		0.29	1.956	0.000958
A_44_P233932	Notch1	XM_342392		0.29	1.956	0.000539
A_43_P11687	Parp1	NM_013063	25591	0.29	1.955	0.004087
A_44_P228341	MGC116373	NM_001025701	314949	0.29	1.954	0.004075
A_44_P409339	Ctnnb1	NM_053357	84353	0.29	1.954	0.003283
A_44_P1058445	Hprt	NM_012583	24465	0.29	1.953	0.001633
A_44_P168778	LOC313391	XM_233220		0.29	1.953	0.008312
A_44_P686912	TC556029	TC556029		0.29	1.953	0.007695
A_42_P622746	RGD1563689_predicted	XM_573801	498530	0.29	1.953	0.001382
A_44_P438598	XM_342104	XM_342104		0.29	1.953	0.000386
A_44_P258912	Aldoa	NM_012495	24189	0.29	1.953	0.001242

A_44_P384683	Utx_predicted	XM_228424	317178	0.29	1.953	0.029154
A_44_P1037110	Dnd1	NM_001025414	307492	0.29	1.953	0.009173
A_42_P555125	Atp1b3	NM_012913	25390	0.29	1.952	0.000933
A_44_P1046118	RGD1563597_predicted	XM_573301		0.29	1.952	0.001786
A_44_P199274	Ddx3x	XM_228701		0.29	1.952	0.001415
A_44_P526881	RGD1304782_predicted	XM_001067861		0.29	1.952	0.004514
A_44_P319382	LOC691543	XM_001078752		0.29	1.952	0.053561
A_44_P760360	RGD1566198_predicted	XM_578123		0.29	1.952	0.001288
A_44_P291552	RGD1310174_predicted	XM_216525		0.29	1.951	0.001665
A_44_P1052817	BF289572	BF289572		0.29	1.951	0.053112
A_44_P268215	XM_219341	XM_219341		0.29	1.951	0.000194
A_44_P154721	Zfp263_predicted	XM_220217		0.29	1.951	0.000397
A_42_P527852	Xrcc6	NM_139080	25019	0.29	1.950	0.001974
A_44_P473437	RGD1564425_predicted	XM_215842		0.29	1.950	0.001454
A_44_P667478	LOC364947	XR_008153	364947	0.29	1.950	0.001687
A_44_P148161	Rrm1_mapped	NM_001013236	365320	0.29	1.950	0.000968
A_43_P11591	Nfya	NM_012865	29508	0.29	1.950	0.001259
A_44_P1014818	Pms2_predicted	XM_213712		0.29	1.950	0.013916
A_42_P800949	Hipk3	NM_031787	83617	0.29	1.949	0.01054
A_44_P185355	Bcl11b_predicted	XM_234514		0.29	1.949	0.000313
A_44_P125681	Dnmt1	NM_053354	84350	0.29	1.949	0.002111
A_43_P18690	RGD1307094_predicted	XM_223406		0.29	1.949	0.000952
A_44_P171337	Fzd7_predicted	XM_237191	301440	0.29	1.949	0.000964
A_44_P313825	Prkr	NM_019335	54287	0.29	1.948	0.030258
A_44_P428487	Gusb	NM_017015	24434	0.29	1.948	0.00755
A_44_P352104	Slc25a17_predicted	XM_216993	300083	0.29	1.948	0.020838
A_44_P491924	Nedd8	NM_138878	25490	0.29	1.948	0.002171
A_44_P378129	XM_237039	XM_237039		0.29	1.948	0.001405
A_44_P140225	Eif4g1	XM_001060756		0.29	1.947	0.002664
A_44_P279007	Prpf40a_predicted	XM_215739		0.29	1.947	0.000722
A_44_P543268	LOC685766	XM_001081538		0.29	1.947	0.012702
A_44_P300965	Prkrir_predicted	XM_218949	308845	0.29	1.947	0.000925
A_43_P21090	Endogl1_predicted	XM_236696		0.29	1.947	0.002787
A_44_P610991	TC530432	TC530432		0.29	1.946	0.017494
A_44_P240274	Emcn	NM_001004228	295490	0.29	1.946	0.001081
A_44_P500935	Sbk1	NM_147135	113907	0.29	1.946	0.009038
A_43_P17008	Bpgm	NM_199382	296973	0.29	1.945	0.0009
A_44_P513246	Fbxo21_predicted	XM_341091		0.29	1.945	0.002486
A_44_P804257	Trim37_predicted	XM_001081112		0.29	1.945	0.021005
A_44_P793931	TC547446	TC547446		0.29	1.945	0.000503
A_44_P226301	BI276075	BI276075		0.29	1.944	0.013968
A_44_P1011193	RGD1560755_predicted	XM_001062341		0.29	1.944	0.003846
A_44_P386303	RGD1311444_predicted	XM_001078026		0.29	1.944	0.112967
A_44_P556586	H3f3b	BC086580	117056	0.29	1.944	0.001904
A_44_P615724	Cdk5rap2	XM_575844	286919	0.29	1.944	0.003458
A_42_P536564	Etf1	NM_001008344	307503	0.29	1.944	0.003032
A_44_P414829	XM_214330	XM_214330		0.29	1.944	0.001902
A_44_P142418	Polm	NM_001011912	289757	0.29	1.943	0.005944
A_44_P557962	XM_232602	XM_232602		0.29	1.943	0.000799
A_44_P1041610	Atp6v0b_predicted	XM_216510		0.29	1.943	0.001065
A_44_P233592	Lcmt1	NM_199405	361643	0.29	1.943	0.014182
A_44_P328872	RGD1565297_predicted	XM_573294		0.29	1.943	0.001257
A_44_P157045	RGD1560716_predicted	XM_233424		0.29	1.943	0.000683
A_43_P15268	Rps15	NM_017151	29285	0.29	1.942	0.00768
A_44_P185171	Pak6_predicted	XM_230519		0.29	1.942	0.0003
A_43_P18608	TC559871	TC559871		0.29	1.942	0.008073
A_43_P17523	RGD1306477	NM_001024891	316557	0.29	1.942	0.007371
A_44_P386331	Fkbp1a	NM_013102	25639	0.29	1.941	0.001183
A_43_P12557	Polr2f	NM_031335	83503	0.29	1.941	0.000351
A_43_P14247	Rps3a	NM_017153	29288	0.29	1.941	0.000222
A_44_P535953	Ercc8_predicted	XM_226789		0.29	1.941	0.001957
A_44_P271855	XM_219411	XM_219411		0.29	1.941	0.002502
A_44_P550808	Zfp482_predicted	XM_345345		0.29	1.941	0.036129

A_44_P227065	Kif22	NM_001009645	293502	0.29	1.941	0.005585
A_42_P827124	LOC680737	XM_001058626	680737	0.29	1.940	0.001099
A_44_P496776	Hmx1_predicted	XM_341238		0.29	1.940	0.003401
A_44_P183220	Cdc27	NM_001024793	360643	0.29	1.940	0.008677
A_44_P475587	Elavl1_predicted	XM_344063		0.29	1.940	0.001576
A_44_P1002452	Nt5dc2	NM_001009271	290558	0.29	1.939	0.000535
A_44_P135958	Rpl37a_predicted	XM_343587		0.29	1.939	0.007566
A_44_P341298	RGD1560358_predicted	XM_001058883		0.29	1.939	0.140198
A_44_P111959	Slc15a4	BC087709	246280	0.29	1.939	0.001876
A_44_P262229	Ppid	NM_001004279	361967	0.29	1.939	0.000663
A_44_P576228	Orc2l	XM_576571		0.29	1.939	0.002118
A_44_P283688	RGD1560936_predicted	XM_219309		0.29	1.939	0.000154
A_44_P680341	Rin2_predicted	XM_001054910		0.29	1.939	0.000324
A_44_P272324	Mbd3_predicted	XM_343162		0.29	1.939	0.000654
A_43_P10468	Myl9_predicted	XM_001067182		0.29	1.939	0.009143
A_44_P458987	LOC364556	XM_001053123		0.29	1.939	0.00085
A_44_P269596	Psmd13_predicted	XM_344976		0.29	1.939	0.00072
A_44_P269566	RGD1311800	NM_001013988	305234	0.29	1.939	0.011054
A_44_P303716	Trim33_predicted	XM_001064349		0.29	1.938	0.005978
A_43_P13203	Expi	NM_133537	171059	0.29	1.938	0.059273
A_44_P445195	Bat1a	NM_133300	114612	0.29	1.938	0.000294
A_43_P10039	LOC500420	XM_575783	500420	0.29	1.938	0.021185
A_44_P1014786	Rsbn1_predicted	XM_227540	310749	0.29	1.938	0.109879
A_42_P819842	RGD1305587_predicted	XM_215413	294499	0.29	1.938	0.126882
A_44_P531848	Rplp2	NM_001030021	140662	0.29	1.938	0.000102
A_44_P618385	Efhd2	NM_001031648	298609	0.29	1.937	0.000866
A_44_P557138	RGD1309263_predicted	XM_001065172		0.29	1.937	0.00832
A_43_P11457	Ace	NM_012544	24310	0.29	1.937	0.009209
A_44_P175786	XM_228865	XM_228865		0.29	1.937	0.000685
A_44_P299542	Nlgn2	NM_053992	117096	0.29	1.937	0.000505
A_44_P297383	Arpc5	NM_001025717	360854	0.29	1.937	0.003787
A_44_P840891	RGD1562791_predicted	XM_574731	499415	0.29	1.936	0.001775
A_44_P1040736	Laptm4a	NM_199384	298875	0.29	1.936	0.000919
A_43_P16954	LOC686323	XM_001069537		0.29	1.936	0.002976
A_44_P261450	Nfkbia	XM_343065		0.29	1.936	0.00025
A_44_P375322	Trpc4ap	XM_342553		0.29	1.936	0.014741
A_44_P132184	BF290460	BF290460		0.29	1.936	0.004586
A_44_P363291	Kcnk10	NM_023096	65272	0.29	1.936	0.012003
A_44_P551311	CB557492	CB557492		0.29	1.935	0.00182
A_44_P698793	LOC502984	XR_006110	502984	0.29	1.935	0.001845
A_44_P318967	XM_342490	XM_342490		0.29	1.934	0.019573
A_44_P288467	LOC685425	XM_001063750	685425	0.29	1.934	0.012208
A_44_P196146	Agtrl1	NM_031349	83518	0.29	1.934	0.008238
A_44_P293161	A_44_P293161	A_44_P293161		0.29	1.934	0.001053
A_44_P1058966	Apbb1ip	XM_225631	307171	0.29	1.934	0.007605
A_44_P370033	Ptpre	XM_341950	114767	0.29	1.934	0.008746
A_42_P582859	Rps18	NM_213557	294282	0.29	1.934	0.000605
A_44_P393835	XM_227733	XM_227733		0.29	1.934	0.000578
A_44_P386602	Brca2	NM_031542	360254	0.29	1.933	0.00237
A_43_P12314	Nucks	NM_022799	64709	0.29	1.933	0.001153
A_43_P20998	RGD1305547_predicted	XM_340870		0.29	1.933	0.003156
A_43_P19205	Smarcc1_predicted	XM_236644		0.29	1.933	0.002593
A_43_P16762	Nudt5	NM_001007733	361274	0.29	1.933	0.000705
A_44_P517033	LOC312502	XM_001073801		0.29	1.932	0.047435
A_44_P825266	TC560298	TC560298		0.29	1.932	0.004723
A_44_P550918	XM_232633	XM_232633		0.29	1.932	0.005664
A_44_P926632	AI045167	AI045167		0.29	1.932	0.053268
A_44_P690194	BG664107	BG664107		0.29	1.931	0.003353
A_44_P700864	DV729193	DV729193		0.29	1.931	0.003261
A_44_P545635	RGD1560766_predicted	XM_001062621		0.29	1.931	0.02332
A_44_P976187	LOC499677	NM_001024303	499677	0.29	1.931	0.005034
A_44_P174682	Nfyc	NM_012866	25337	0.29	1.930	0.141833
A_44_P429994	BQ209906	BQ209906	301008	0.29	1.930	0.018493

A_44_P878617	TC529378	TC529378		0.29	1.930	0.025196
A_44_P1006977	Lars	NM_001009637	291624	0.29	1.930	0.002961
A_44_P499560	Aff3_predicted	XM_343559	363220	0.29	1.929	0.007353
A_44_P1015001	Nedd4a	XM_343427		0.29	1.929	0.000506
A_43_P11682	Ube2i	NM_013050	25573	0.29	1.929	0.000655
A_44_P550203	Ugcgl1	NM_133596	171129	0.29	1.928	0.011071
A_43_P10348	MGC114417	NM_001024877	300149	0.29	1.928	0.000792
A_44_P580017	TC526108	TC526108		0.29	1.928	0.004325
A_44_P424051	Snrpd2_predicted	XM_214847	292688	0.29	1.928	0.000115
A_42_P454929	Cdc42ep5_predicted	XM_341784		0.29	1.928	0.000113
A_44_P821851	ENSRNOT00000007811	ENSRNOT00000007811		0.29	1.928	0.000273
A_44_P102946	Cul2_predicted	XM_341542		0.28	1.927	0.006861
A_43_P17779	RGD1564042_predicted	XM_215493		0.28	1.927	0.001199
A_44_P365947	LOC606294	NM_001031627	606294	0.28	1.927	0.038334
A_44_P508462	Anp32b	NM_131911	170724	0.28	1.927	0.00093
A_42_P552807	Hcfc1_predicted	XM_343843	363519	0.28	1.927	0.000312
A_43_P16773	LOC301117	XM_217313	301117	0.28	1.927	0.000799
A_44_P142665	DV728658	DV728658		0.28	1.927	0.092942
A_44_P485122	Cep55	NM_001025646	294074	0.28	1.927	0.003899
A_44_P991949	RGD1559547_predicted	XM_001072704		0.28	1.927	0.090057
A_44_P546419	Havcr2	XM_001069084		0.28	1.927	0.005732
A_44_P374803	Grip1	NM_032069	84016	0.28	1.927	0.008763
A_44_P142531	A_44_P142531	A_44_P142531		0.28	1.927	0.014959
A_44_P436424	Plekhm2_predicted	XM_233611	313668	0.28	1.926	0.000951
A_44_P366303	Matn2_predicted	XM_216941	299996	0.28	1.925	0.058914
A_43_P12753	Pgam1	NM_053290	24642	0.28	1.925	0.000414
A_44_P241371	Cstf1	NM_001013161	311670	0.28	1.925	0.055537
A_44_P777328	TC540923	TC540923		0.28	1.925	0.076347
A_44_P1045612	Fxyd3	NM_172317	116831	0.28	1.925	0.00279
A_43_P16529	Gadd45b	NM_001008321	299626	0.28	1.925	0.001588
A_44_P281415	Gpr97_predicted	XM_226243	291854	0.28	1.924	0.053965
A_44_P166749	Tcfe3_predicted	XM_228760	317376	0.28	1.924	0.000237
A_44_P440923	Rpl18	NM_031102	81766	0.28	1.924	0.000241
A_44_P452114	Gls	AY083459	24398	0.28	1.924	0.027731
A_44_P280963	Kifc1	NM_001005878	294286	0.28	1.923	0.00274
A_44_P163018	Nubp1	NM_001009619	287042	0.28	1.923	0.011919
A_44_P733009	TC559843	TC559843		0.28	1.923	0.002638
A_44_P215488	A_44_P215488	A_44_P215488		0.28	1.922	0.009991
A_44_P268893	CF110587	CF110587		0.28	1.922	0.008389
A_44_P134865	RGD1309198_predicted	XM_232775	313056	0.28	1.922	0.000378
A_44_P344969	LOC683224	XM_001064982		0.28	1.922	0.000148
A_44_P411173	DV727337	DV727337		0.28	1.922	0.076465
A_44_P284344	CB546810	CB546810		0.28	1.922	0.029107
A_44_P687308	TC544614	TC544614		0.28	1.921	0.027731
A_44_P135843	Rps10	NM_031109	81773	0.28	1.921	0.000149
A_42_P537715	Wdr61	NM_001025743	363064	0.28	1.921	0.002373
A_44_P478114	Cdca2	XM_224308		0.28	1.921	0.014407
A_44_P284029	ENSRNOT00000028933	ENSRNOT00000028933		0.28	1.920	0.002256
A_44_P779731	TC541278	TC541278		0.28	1.920	0.006199
A_44_P605260	Pigs	NM_001006602	303277	0.28	1.919	0.00381
A_44_P540738	Matr3	NM_019149	29150	0.28	1.919	0.003739
A_44_P1028065	Gtf2e2_predicted	XM_224929		0.28	1.918	0.016784
A_44_P288826	RGD1308134_predicted	XM_213357	287452	0.28	1.918	0.000222
A_44_P316726	A_44_P316726	A_44_P316726		0.28	1.918	0.000201
A_44_P102401	Rab15	NM_198749	299156	0.28	1.918	0.001065
A_44_P898864	Cep27_predicted	XM_001076273		0.28	1.918	0.080477
A_43_P14887	Dlx5	NM_012943	25431	0.28	1.918	0.038941
A_44_P382696	Syncrip	XM_001065902		0.28	1.917	0.004473
A_43_P17974	Cog4_predicted	XM_341688		0.28	1.917	0.013583
A_44_P196456	Gnb2	NM_031037	81667	0.28	1.917	0.008381
A_44_P945950	DV719240	DV719240		0.28	1.917	0.004896
A_44_P154675	LOC299725	XR_005762	299725	0.28	1.917	0.072477
A_44_P309473	XM_228778	XM_228778		0.28	1.917	0.004041

A_44_P393202	RGD1560812_predicted	XM_576015	500640	0.28	1.917	0.132615
A_44_P215711	LOC362587	XM_342906	362587	0.28	1.916	0.000256
A_43_P11739	Ctsl	NM_013156	25697	0.28	1.916	0.004603
A_44_P175078	Col27a1	NM_198747	298101	0.28	1.916	0.033319
A_44_P171196	RGD1563157_predicted	XM_233992		0.28	1.916	0.0004
A_44_P614154	LOC310946	XM_001078514		0.28	1.916	0.079125
A_44_P400377	Phtf1	XM_342306	252962	0.28	1.915	0.003456
A_44_P914137	A_44_P914137	A_44_P914137		0.28	1.915	0.000281
A_44_P374387	AW143043	AW143043		0.28	1.915	0.006398
A_44_P254896	Ddb1	NM_171995		0.28	1.915	0.005075
A_42_P783318	Trp53i13_predicted	XM_213407		0.28	1.915	0.002718
A_44_P510087	DY471385	DY471385		0.28	1.915	0.126481
A_44_P201379	Ldb1_predicted	XM_219948		0.28	1.915	0.000341
A_42_P723437	Thyn1	NM_001007661	300470	0.28	1.914	0.001865
A_42_P658562	RGD1304711_predicted	XM_214334	290679	0.28	1.914	0.001866
A_44_P656617	TC545944	TC545944		0.28	1.914	0.14639
A_44_P292503	Crebl1	NM_001002809	406169	0.28	1.914	0.002796
A_44_P590086	BG667093	BG667093		0.28	1.914	0.000501
A_44_P536576	Ipo7_predicted	XM_219265		0.28	1.914	0.004799
A_44_P326183	RGD1308706_predicted	XM_214649	291925	0.28	1.914	0.006396
A_44_P180645	Usp24_predicted	XM_233260	313427	0.28	1.914	0.004332
A_44_P352344	Plrg1	NM_021757	60376	0.28	1.914	0.007273
A_44_P405259	Nav2	XM_341864	171563	0.28	1.913	0.015035
A_44_P667364	ENSRNOT00000039319	ENSRNOT00000039319		0.28	1.912	0.001107
A_44_P407889	RGD1565460_predicted	XM_228087		0.28	1.912	0.002743
A_44_P471691	Vcl_predicted	XM_223781		0.28	1.912	0.0043
A_42_P730505	Rce1	XM_219685	309153	0.28	1.912	0.000408
A_42_P797420	Cox4nb	NM_001012165	361425	0.28	1.912	0.00054
A_44_P451565	Clasp2	NM_053722		0.28	1.911	0.002141
A_44_P538531	Pitpnb	NM_053742	114561	0.28	1.911	0.004203
A_44_P443101	Cln5_predicted	XM_224477	306128	0.28	1.911	0.061186
A_44_P458241	LOC681942	XM_001059166		0.28	1.911	0.000238
A_43_P16153	LOC690209	XM_345900		0.28	1.911	0.006122
A_44_P1020640	Dus2l_predicted	XM_214665		0.28	1.911	0.003032
A_44_P804740	Cdca4	NM_001037214	500727	0.28	1.910	0.000248
A_44_P395709	Rac1	NM_134366	363875	0.28	1.910	0.006634
A_44_P163865	Exosc6_predicted	XM_226471	307850	0.28	1.910	0.000417
A_44_P365379	Gadd45g	XM_237999		0.28	1.910	0.000225
A_44_P592008	TC522565	TC522565		0.28	1.910	0.006278
A_44_P416178	CO387879	CO387879		0.28	1.910	0.00461
A_42_P768776	Zfp330_predicted	XM_341666		0.28	1.910	0.012339
A_44_P871648	Dcakd	NM_001007724	360639	0.28	1.910	0.001659
A_44_P1071405	Narg1_predicted	XM_241375		0.28	1.909	0.006691
A_44_P545735	Znf291	XM_343394	117521	0.28	1.909	0.005128
A_44_P501752	RGD1559909_predicted	XM_347063		0.28	1.909	9.64E-05
A_44_P881475	AA874941	AA874941	298199	0.28	1.909	0.001382
A_44_P169307	Rad50	NM_022246	64012	0.28	1.909	0.003378
A_44_P767417	Ripx	NM_001025127	360921	0.28	1.909	0.018478
A_44_P255149	Klrc3	NM_001029908	500338	0.28	1.909	0.029294
A_44_P763162	TC556147	TC556147		0.28	1.908	0.005597
A_44_P112371	RGD1563950_predicted	XM_233308	298316	0.28	1.908	0.000887
A_44_P292686	Hps1	NM_040669	114638	0.28	1.908	0.001699
A_43_P19059	RGD1560656_predicted	XM_574664		0.28	1.907	0.002235
A_44_P483259	XM_346049	XM_346049		0.28	1.907	0.000532
A_44_P232113	A_44_P232113	A_44_P232113		0.28	1.907	0.000278
A_44_P497553	Gcnt1	NM_022276	64043	0.28	1.907	0.003027
A_44_P358988	XM_574299	XM_574299		0.28	1.906	0.092123
A_44_P258124	RGD1565310_predicted	XM_001057346		0.28	1.906	0.13261
A_44_P385330	BF545883	BF545883		0.28	1.906	0.062311
A_44_P564669	TC544957	TC544957		0.28	1.906	0.133585
A_43_P17703	Dph5	NM_001017449	295394	0.28	1.905	0.00038
A_44_P869973	TC551940	TC551940		0.28	1.905	0.00036
A_44_P278810	XM_214963	XM_214963		0.28	1.905	0.003054

A_43_P18913	RGD1561571_predicted	XM_235649	315297	0.28	1.905	0.015209
A_44_P1070826	Gng10	NM_053660	114119	0.28	1.905	0.001763
A_44_P667648	LOC682841	XM_001063350		0.28	1.904	0.003525
A_42_P781659	Reps1_predicted	XM_214954		0.28	1.904	0.00141
A_44_P454587	Cops6_predicted	XM_222002		0.28	1.904	0.000792
A_44_P175482	Plxna2_predicted	XM_223080		0.28	1.904	0.084305
A_44_P236857	Zfpn1a2_predicted	XM_237228		0.28	1.904	0.172116
A_44_P543711	XM_574071	XM_574071		0.28	1.904	0.000534
A_44_P1038611	XM_230750	XM_230750		0.28	1.903	0.000591
A_44_P793551	TC528633	TC528633		0.28	1.903	0.023334
A_44_P178671	BI289714	BI289714		0.28	1.903	0.00197
A_44_P396303	LOC682072	XM_001056160		0.28	1.903	0.000402
A_44_P346959	Isgf3g	NM_001012041	305896	0.28	1.902	0.03515
A_44_P217590	Prkwnk1	NM_053794	116477	0.28	1.902	0.001713
A_44_P396409	RGD1563395_predicted	XM_216794		0.28	1.902	0.001105
A_44_P197896	Kifap3_predicted	XM_213920		0.28	1.902	0.005468
A_44_P331128	Senp3	XM_220607	303245	0.28	1.901	0.004195
A_43_P21776	Rfc5_predicted	XM_222214		0.28	1.901	0.000238
A_44_P945829	TC523525	TC523525		0.28	1.901	0.004277
A_44_P285803	Rs21c6	NM_138892	192252	0.28	1.901	0.002538
A_44_P898681	RGD1565410_predicted	XM_578686	503162	0.28	1.901	0.015787
A_44_P793124	RGD1560834_predicted	XM_001067122		0.28	1.901	0.011183
A_44_P497065	Stoml2	NM_001031646	298203	0.28	1.900	0.001221
A_44_P995544	Wdr5	NM_001039034	362093	0.28	1.900	0.022083
A_44_P262645	Gdf1_predicted	XM_224733		0.28	1.900	0.019739
A_44_P196702	Gpr125_predicted	XM_223485		0.28	1.900	0.006773
A_44_P853060	AABR03024699	AABR03024699		0.28	1.900	0.007528
A_44_P299389	Eno1	NM_012554	24333	0.28	1.899	0.002252
A_44_P965692	TC537677	TC537677		0.28	1.899	0.034241
A_44_P134739	Unr	NM_054006	117180	0.28	1.899	0.000851
A_44_P605101	BF557668	BF557668		0.28	1.899	0.002158
A_44_P309584	RGD1306228_predicted	XM_343073	362748	0.28	1.899	0.037218
A_44_P211774	DY316829	DY316829		0.28	1.899	0.122981
A_44_P250194	G3bp	XM_340802	171092	0.28	1.899	0.011628
A_44_P715835	TC542511	TC542511		0.28	1.899	0.003933
A_44_P1009552	Gtf2ird1	NM_001001504	246770	0.28	1.898	0.001095
A_44_P944550	A_44_P944550	A_44_P944550		0.28	1.898	0.000764
A_44_P318861	RGD1564142_predicted	XM_001060720		0.28	1.897	0.011731
A_44_P768805	TC551538	TC551538		0.28	1.897	0.029688
A_44_P398631	LOC366396	XR_008699	366396	0.28	1.897	0.000124
A_44_P494396	XM_212746	XM_212746		0.28	1.897	0.00104
A_44_P419659	LOC361420	BC062077	361420	0.28	1.896	0.003371
A_42_P827480	RGD1561211_predicted	XM_577781	502317	0.28	1.896	0.003578
A_44_P764112	Dpf2_predicted	XM_001074896		0.28	1.896	0.000365
A_44_P138800	RGD1304626_predicted	XM_001061993		0.28	1.896	0.037411
A_43_P11430	Adsl_predicted	XM_235496	315150	0.28	1.896	0.004196
A_44_P245979	RGD1560542_predicted	XM_575187		0.28	1.896	0.027006
A_43_P15289	Enpep	NM_022251	64017	0.28	1.895	0.009037
A_44_P361487	LOC287167	NM_001013853	287167	0.28	1.895	0.025909
A_43_P13069	Chek1	NM_080400	140583	0.28	1.895	0.005353
A_44_P430989	Fnbp1	NM_138914	192348	0.28	1.895	0.000388
A_44_P166513	Atf1	XM_235657	315305	0.28	1.894	0.001724
A_43_P11484	Mme	NM_012608	24590	0.28	1.894	0.005572
A_44_P1036339	LOC689842	XR_006738	689842	0.28	1.894	0.001958
A_44_P724503	BE349658	BE349658		0.28	1.893	0.007806
A_42_P673212	Cad_mapped	XM_343027		0.28	1.893	0.000993
A_44_P513764	Nudcd1_predicted	XM_343235	362906	0.28	1.892	0.004049
A_44_P288548	RGD1306534_predicted	XM_230873	311647	0.28	1.892	0.010115
A_44_P182658	RGD1560834_predicted	XM_222679	289052	0.28	1.892	0.014715
A_43_P16707	Ccl6	NM_001004202	287910	0.28	1.892	0.161294
A_44_P337865	A_44_P337865	A_44_P337865		0.28	1.892	0.002882
A_44_P483678	BF558220	BF558220	363294	0.28	1.891	0.003292
A_44_P117175	XM_343362	XM_343362		0.28	1.891	0.010452

A_44_P827926	Rkhd2_predicted	XM_001062342		0.28	1.891	0.00348
A_44_P329980	BE115640	BE115640		0.28	1.891	0.003065
A_44_P1047136	LOC293589	XM_001056886		0.28	1.891	0.11266
A_42_P561580	Rps27a	NM_031113	81777	0.28	1.891	0.001124
A_42_P651962	Hspd1	NM_022229	63868	0.28	1.891	0.018644
A_44_P729396	TC551894	TC551894		0.28	1.890	0.006546
A_44_P433431	Hist1h2ao_predicted	XM_344599		0.28	1.890	0.0404
A_42_P716570	Tia1	XM_575591		0.28	1.890	0.004194
A_44_P492581	Klhdc3	NM_001012203	363192	0.28	1.890	0.000707
A_43_P19267	Riok2	NM_001009687	308201	0.28	1.890	0.00081
A_44_P714133	A_44_P714133	A_44_P714133		0.28	1.890	0.00054
A_44_P421826	RGD1563315_predicted	XM_223544		0.28	1.890	0.002323
A_44_P387331	RGD1564148_predicted	XM_220587		0.28	1.889	0.012971
A_44_P884020	TC520003	TC520003		0.28	1.889	0.000659
A_44_P468304	Terc	NR_001567		0.28	1.889	0.006018
A_44_P1008398	Stxbp6_predicted	XM_343057	362734	0.28	1.889	0.034074
A_44_P310386	Psmd7_predicted	XM_226439		0.28	1.889	0.002326
A_44_P222640	RGD1563817_predicted	XM_344732		0.28	1.889	0.000346
A_44_P210722	RGD1305526_predicted	XM_215438		0.28	1.888	0.008973
A_44_P744421	RGD1561419_predicted	XM_573507		0.28	1.888	0.025749
A_44_P163816	RGD1307128	NM_001012354	311346	0.28	1.888	0.142062
A_44_P231961	LOC689421	XM_001071003		0.28	1.887	0.000982
A_44_P526060	Hnrpk	NM_057141	117282	0.28	1.887	0.000341
A_43_P14135	Prdx2	NM_017169	29338	0.28	1.887	0.001455
A_44_P714581	Strn4_predicted	XM_218432		0.28	1.887	0.001935
A_44_P546185	Prickle2_predicted	XM_232196		0.28	1.887	0.038268
A_44_P442733	Mafg	NM_022386	64188	0.28	1.886	0.001068
A_44_P279224	RGD1308923_predicted	XM_342994		0.28	1.886	0.00036
A_44_P553262	LOC688570	XM_001067468	688570	0.28	1.886	0.002002
A_44_P349148	Dhodh	NM_001008553	65156	0.28	1.886	0.002136
A_44_P159292	Chd4	XM_232354	117535	0.28	1.886	0.002356
A_44_P436808	Cilp_predicted	XM_236348		0.28	1.885	0.033317
A_44_P516101	RGD1309552	NM_001025285	362957	0.28	1.885	0.001653
A_44_P1060432	XM_214279	XM_214279		0.28	1.885	0.004145
A_44_P306626	LOC502125	XM_577573		0.28	1.885	0.040903
A_44_P443493	Neil1	NM_001025754	367090	0.28	1.884	0.00262
A_44_P201752	Sirt6	NM_001031649	299638	0.28	1.884	0.000226
A_44_P777689	Tmem2_predicted	XM_001079816		0.28	1.884	0.000368
A_44_P970328	LOC685834	XM_001065442	685834	0.28	1.884	0.001702
A_44_P728951	Rps2	NM_031838	83789	0.28	1.884	0.000227
A_44_P447766	RGD1564138_predicted	XM_001076168		0.28	1.884	0.000302
A_44_P768254	TC527917	TC527917		0.28	1.884	0.164099
A_44_P547231	Ches1_predicted	XM_234377		0.27	1.884	0.096615
A_44_P238891	Ssna1_predicted	XM_231052		0.27	1.884	0.001201
A_44_P247849	LOC311254	XM_230327		0.27	1.883	0.04789
A_44_P419141	Nup153	XM_001056084		0.27	1.883	0.00163
A_43_P21231	Suv39h1_predicted	XM_217594		0.27	1.883	0.005075
A_44_P368320	LOC500591	XM_001076183		0.27	1.883	0.015652
A_44_P294467	Paxip1_predicted	XM_231271		0.27	1.883	0.003748
A_43_P13018	Cand1	NM_054004	117152	0.27	1.883	0.009776
A_44_P435664	Rnf166	NM_001002279	365022	0.27	1.882	0.000863
A_44_P249606	MGC112830	NM_001025718	361178	0.27	1.882	0.001244
A_44_P539284	Zbtb38	NM_001012471	315936	0.27	1.882	0.000669
A_44_P222878	Eif3s6ip	NM_001034134	300069	0.27	1.882	0.000274
A_42_P582144	Tor1aip1	NM_145092	246314	0.27	1.882	0.005081
A_44_P527962	AA899782	AA899782		0.27	1.882	0.01343
A_44_P1052798	Ate1_predicted	XM_215086		0.27	1.882	0.001521
A_44_P1070991	U28975	U28975		0.27	1.881	0.011588
A_44_P492321	RGD1307772	XM_214666	291974	0.27	1.881	0.002146
A_44_P258417	Mad2l1bp	NM_001009699	316237	0.27	1.881	0.001917
A_44_P473565	Usp24_predicted	XM_233260	313427	0.27	1.881	0.002467
A_44_P436588	Plekhm1	NM_001009677	303584	0.27	1.881	0.002158
A_44_P371823	XM_242940	XM_242940		0.27	1.881	0.005418

A_42_P557485	Gnai2	NM_031035	81664	0.27	1.880	0.000262
A_44_P314505	Arl6_predicted	XM_344009		0.27	1.880	0.000409
A_44_P652274	A_44_P652274	A_44_P652274		0.27	1.880	0.022472
A_44_P622334	DN934079	DN934079		0.27	1.880	0.010583
A_44_P511248	BC087069	BC087069	366057	0.27	1.880	0.016803
A_44_P375832	BE115850	BE115850	310855	0.27	1.880	0.000928
A_44_P945383	TC521659	TC521659		0.27	1.880	0.001775
A_44_P401165	Stmn1	NM_017166	29332	0.27	1.880	0.00706
A_44_P344346	Arpc1b	NM_019289	54227	0.27	1.879	0.003187
A_44_P338381	RGD1559440_predicted	XM_231528	312166	0.27	1.879	0.063027
A_44_P520190	Exoc2	NM_134414	171455	0.27	1.879	0.002833
A_44_P510881	LOC690000	XM_001073955	690000	0.27	1.879	0.000961
A_44_P339659	Hdac5	XM_001081495		0.27	1.879	0.00681
A_44_P670412	TC546050	TC546050		0.27	1.879	0.119182
A_44_P361522	XM_346101	XM_346101		0.27	1.878	0.000779
A_44_P315649	Etv1_predicted	XM_001074388		0.27	1.878	0.014972
A_42_P771848	Eif4b	NM_001008324	300253	0.27	1.878	0.001221
A_44_P438409	Ccnj_predicted	XM_220004		0.27	1.878	0.009016
A_44_P464436	Scube1	XM_235529	315174	0.27	1.878	0.252168
A_44_P182182	Spata6	NM_134392	171413	0.27	1.877	0.042369
A_43_P21199	Luzp5_predicted	XM_343124	362798	0.27	1.877	0.003077
A_44_P317539	Psma7	NM_001008217	29674	0.27	1.877	0.002753
A_44_P505809	Mrps18b	NM_212534	294230	0.27	1.877	0.004458
A_44_P382525	Trmt12	XM_235337		0.27	1.877	0.01631
A_44_P339318	BG671893	BG671893		0.27	1.877	0.015029
A_44_P215698	A_44_P215698	A_44_P215698		0.27	1.877	0.0003
A_44_P834734	AB026259	AB026259		0.27	1.877	0.005042
A_44_P294055	Ltv1	NM_001014157	361452	0.27	1.876	0.001001
A_44_P539098	Shq1_predicted	XM_232222	297483	0.27	1.876	0.003767
A_44_P789383	BF555793	BF555793		0.27	1.876	0.002739
A_44_P363597	Raly	NM_001011958	296301	0.27	1.876	0.001721
A_44_P792481	LOC682340	XM_001059298		0.27	1.876	0.000876
A_44_P225259	Rbx1	NM_001034135	300084	0.27	1.876	0.000935
A_42_P519770	Rpl29	NM_017150	29283	0.27	1.876	0.00016
A_44_P1030201	RGD1564946_predicted	XM_234416	299199	0.27	1.875	0.00437
A_44_P463110	BF285698	BF285698	306147	0.27	1.875	0.222014
A_44_P994686	Tnnt2	NM_012676	24837	0.27	1.875	0.030771
A_42_P466272	Psen2	NM_031087	81751	0.27	1.874	0.00077
A_44_P506363	Trim44	NM_001013203	362172	0.27	1.874	0.001031
A_44_P424268	Zbtb26_predicted	XM_231250		0.27	1.874	0.006852
A_44_P175495	Cxcl11	NM_182952	305236	0.27	1.874	0.043677
A_43_P23108	Mcm7	NM_001004203	288532	0.27	1.874	0.002187
A_44_P138126	Cova1_predicted	XM_229131	302817	0.27	1.873	0.017663
A_44_P361301	Tln1	XM_001053379	313494	0.27	1.873	0.00155
A_44_P271643	Nln	NM_053970	117041	0.27	1.873	0.001181
A_44_P274242	Siglec10_predicted	XM_214918		0.27	1.873	0.00564
A_44_P434541	Ada	NM_130399	24165	0.27	1.873	0.003634
A_44_P456339	AA925212	AA925212	84401	0.27	1.873	0.001434
A_44_P441107	Tfcp2l4_predicted	XM_233561		0.27	1.873	0.092633
A_44_P304940	RGD1564744_predicted	XM_234961	314652	0.27	1.873	0.000698
A_42_P524707	Prim2	NM_001024762	301323	0.27	1.872	0.003564
A_44_P253335	Gpr157	NM_001012107	313725	0.27	1.872	0.025996
A_44_P279222	RGD1308923_predicted	XM_342994		0.27	1.872	0.000448
A_44_P426181	Hyal1	NM_207616	367166	0.27	1.871	0.04786
A_44_P1071344	Atxn2l_predicted	XM_341928	361649	0.27	1.871	0.001641
A_44_P389356	RGD1565619_predicted	XM_225760	307302	0.27	1.871	0.00822
A_44_P448068	RGD1304910_predicted	XM_231724		0.27	1.871	0.005299
A_44_P567387	TC525601	TC525601		0.27	1.870	0.057581
A_44_P423600	LOC245925	NM_139093	245925	0.27	1.870	0.018499
A_44_P1029492	Snapc1_predicted	XM_234299		0.27	1.870	0.049347
A_42_P777045	Rhog	NM_001037195	308875	0.27	1.870	0.000166
A_43_P16242	Pou2f2	XM_341802	117058	0.27	1.870	0.015277
A_42_P733209	Dgat2	NM_001012345	252900	0.27	1.869	0.005454

A_43_P17723	Ankrd49_predicted	XM_001069095		0.27	1.869	0.01596
A_44_P356222	ENSRNOT00000051889	ENSRNOT00000051889		0.27	1.869	0.02781
A_44_P666461	Pex11b	XM_001063078		0.27	1.869	0.005859
A_44_P128693	Bicd1_predicted	XM_342789		0.27	1.869	0.011168
A_44_P487492	XM_344494	XM_344494		0.27	1.869	0.000189
A_44_P541937	Mid2_predicted	XM_343825	363502	0.27	1.868	0.014276
A_44_P550250	Rcbtb2	NM_199084	290363	0.27	1.868	0.135661
A_43_P13144	Cdkn2c	NM_131902	54238	0.27	1.868	0.007963
A_44_P359509	Usp47_predicted	XM_218997		0.27	1.868	0.003451
A_43_P15352	Nploc4	NM_080577	140639	0.27	1.868	0.010105
A_44_P292915	Tbc1d1_predicted	XM_341215	360937	0.27	1.868	0.000848
A_44_P187503	RGD1563994_predicted	XM_001064713		0.27	1.868	0.00338
A_44_P446556	LOC362587	XM_342906	362587	0.27	1.867	0.000963
A_44_P624151	TC543922	TC543922		0.27	1.867	0.020311
A_44_P380182	RGD1564040_predicted	XM_233236		0.27	1.867	0.000453
A_43_P15490	Clstn2	NM_134377	171394	0.27	1.867	0.040728
A_44_P323678	Depdc1b_predicted	XM_226798		0.27	1.867	0.038384
A_42_P525098	Lsg1	NM_001013421	288029	0.27	1.867	0.01399
A_44_P526303	AI058424	AI058424	361549	0.27	1.867	0.008717
A_44_P405533	LOC361399	NM_001033068	361399	0.27	1.866	0.001216
A_44_P594455	Eif4g2_predicted	XM_341907		0.27	1.866	0.022424
A_44_P960146	Gga3_predicted	XM_340935		0.27	1.866	0.000521
A_44_P370507	XM_225950	XM_225950		0.27	1.866	0.000273
A_44_P289658	Nup160_predicted	XM_230286		0.27	1.866	0.003735
A_43_P19580	RGD1307009	NM_001013884	290997	0.27	1.866	0.004799
A_43_P12964	Plaa	NM_053866	116645	0.27	1.866	0.000348
A_43_P12067	Leprot	NM_020099	24536	0.27	1.866	0.002743
A_42_P755326	Itgb6	NM_001004263	311061	0.27	1.866	0.004954
A_44_P354844	Thap11_predicted	XM_001075955		0.27	1.866	0.030161
A_44_P522195	AI071307	AI071307	294674	0.27	1.865	0.007115
A_44_P105741	Mak3_predicted	XM_213624		0.27	1.865	0.000912
A_43_P21454	Smc2l1_predicted	XM_342837		0.27	1.865	0.005198
A_44_P915165	XM_580077	XM_580077		0.27	1.865	0.031428
A_44_P973782	BC091575	BC091575		0.27	1.865	0.011839
A_44_P683182	RGD1564980_predicted	XM_575373		0.27	1.865	0.000261
A_44_P220372	A_44_P220372	A_44_P220372		0.27	1.865	0.037697
A_42_P659682	AW143252	AW143252		0.27	1.864	0.047624
A_44_P463912	LOC288654	XM_001079100		0.27	1.864	0.002343
A_44_P1057690	Dnase1l3	NM_053907	116687	0.27	1.864	0.00361
A_44_P254229	Rbbp7	NM_031816	83712	0.27	1.864	0.001072
A_44_P350762	NTF2	NM_001007629	291981	0.27	1.864	0.005215
A_44_P1006988	LOC685082	XM_001062211	685082	0.27	1.863	0.010861
A_44_P294965	RT1-DOb	NM_001008846	365542	0.27	1.863	0.000772
A_44_P253424	LOC363188	XM_343528		0.27	1.863	0.124781
A_44_P918056	RGD1560863_predicted	XM_574612	499310	0.27	1.863	0.0121
A_44_P410112	XM_214416	XM_214416		0.27	1.863	0.000266
A_44_P478919	Snrpd1_predicted	XM_214621		0.27	1.863	0.002354
A_44_P339987	RGD1564983_predicted	XM_219578		0.27	1.863	0.000854
A_44_P279296	RGD1562949_predicted	XM_236578	315969	0.27	1.863	0.006005
A_44_P278829	ENSRNOT00000040084	ENSRNOT00000040084		0.27	1.863	0.000635
A_44_P841539	TC543580	TC543580		0.27	1.863	0.069754
A_44_P319057	Dock11	XM_233283	313438	0.27	1.862	0.005263
A_42_P574960	U08214	U08214		0.27	1.862	0.004482
A_42_P745602	Vipr2	NM_017238	29555	0.27	1.862	0.008644
A_44_P120405	AW526982	AW526982	310553	0.27	1.862	0.00926
A_42_P458530	Ripk3	NM_139342	246240	0.27	1.861	0.000332
A_44_P170652	Soat1	NM_031118	81782	0.27	1.861	0.00016
A_44_P292819	LOC683674	XM_001064018		0.27	1.861	0.000418
A_44_P276276	ENSRNOT00000013630	ENSRNOT00000013630		0.27	1.861	0.114995
A_43_P13117	Lcp2	NM_130421	155918	0.27	1.861	0.015958
A_44_P328864	RGD1565297_predicted	XM_573294		0.27	1.860	0.002567
A_44_P699345	TC537554	TC537554		0.27	1.860	0.142989
A_44_P323280	Spata6	NM_134392	171413	0.27	1.860	0.069763

A_42_P549786	B3gat2	NM_022609	64544	0.27	1.860	0.01141
A_44_P298210	Casp8	NM_022277	64044	0.27	1.860	0.002155
A_44_P576197	CO571318	CO571318		0.27	1.860	0.031931
A_44_P990043	Pdcd11_predicted	XM_219966		0.27	1.860	0.000692
A_42_P820657	Il1r2	NM_053953	117022	0.27	1.860	0.01822
A_43_P19441	RGD1310953	NM_001008366	361416	0.27	1.859	0.006126
A_44_P235679	BG665133	BG665133	80850	0.27	1.859	0.005064
A_44_P310771	DY471696	DY471696		0.27	1.859	0.002958
A_44_P652611	A_44_P652611	A_44_P652611		0.27	1.859	0.002675
A_44_P576954	Ddx58_predicted	XM_001067411		0.27	1.858	0.013057
A_42_P557315	RGD1308371_predicted	XM_214995		0.27	1.858	0.023068
A_44_P456933	LOC688032	XR_009425	691340	0.27	1.858	0.010113
A_44_P123033	Klhl5	XM_223418		0.27	1.858	0.002826
A_42_P690274	Atox1	NM_053359	84355	0.27	1.858	0.000765
A_44_P227604	Eme1_predicted	XM_220879		0.27	1.858	0.045421
A_42_P713089	Pkp4_predicted	XM_215733		0.27	1.858	0.000251
A_44_P222660	XM_226568	XM_226568		0.27	1.858	0.000477
A_44_P387912	LOC686883	XM_001076161		0.27	1.857	0.010257
A_44_P757980	Eif4ebp2	NM_001033069	361845	0.27	1.857	0.007271
A_44_P591437	Polr3d	NM_001031653	306012	0.27	1.857	0.000799
A_44_P217273	Unr	NM_054006	117180	0.27	1.857	0.001805
A_43_P11686	Kdr	NM_013062	25589	0.27	1.856	0.052375
A_44_P288393	XM_224348	XM_224348		0.27	1.856	0.000381
A_43_P12000	Sema4f	NM_019272	29745	0.27	1.856	0.047905
A_44_P106598	RGD1309228	NM_001017451	298851	0.27	1.856	0.005376
A_44_P274491	Large_predicted	XM_341645		0.27	1.856	0.131591
A_44_P548334	XM_223646	XM_223646		0.27	1.856	0.000235
A_44_P162170	AW920040	AW920040	362766	0.27	1.856	0.028562
A_42_P796041	Zfp637	XM_342745	362425	0.27	1.855	0.004144
A_44_P112428	LOC363333	XM_343671	363333	0.27	1.855	0.001096
A_44_P667607	RGD1562326_predicted	XM_001080919		0.27	1.855	0.050701
A_44_P231227	Mosc2	NM_134410	171451	0.27	1.855	0.004979
A_44_P494310	Bri3	NM_001009604	304284	0.27	1.855	0.00035
A_44_P593525	Atp11c_predicted	XR_007360	317599	0.27	1.855	0.142017
A_44_P270812	Kua_predicted	XM_342588	362278	0.27	1.855	0.17297
A_44_P462076	Acy1	NM_001005383	300981	0.27	1.855	0.00262
A_44_P218023	XM_215586	XM_215586		0.27	1.854	0.000542
A_44_P621111	ENSRNOT00000050351	ENSRNOT00000050351		0.27	1.854	0.286898
A_44_P278670	LOC687717	XM_001079893		0.27	1.854	0.000974
A_44_P351136	Pafah2	NM_177932	313611	0.27	1.854	0.001338
A_44_P134143	Hspb8	NM_053612	113906	0.27	1.854	0.015981
A_44_P717706	Thtpa	NM_001007682	305889	0.27	1.854	0.003861
A_44_P461944	Morf4l2	NM_001007714	317413	0.27	1.854	0.001872
A_44_P340451	XM_343863	XM_343863		0.27	1.853	0.002364
A_44_P523016	LOC499913	NM_001024314	499913	0.27	1.853	0.008071
A_44_P461666	RGD1562774_predicted	XR_009271	365437	0.27	1.853	0.001126
A_44_P581181	RGD1309394_predicted	XM_001077341		0.27	1.853	0.003631
A_44_P891064	AW917113	AW917113		0.27	1.853	0.147952
A_44_P155463	LOC684304	XM_001069799		0.27	1.853	0.020806
A_43_P20339	Hmha1_predicted	XM_234898		0.27	1.852	0.002472
A_44_P118075	RGD1562702_predicted	XM_575227		0.27	1.852	0.001005
A_44_P1037275	Vkorc1l1	NM_203338	399684	0.27	1.852	0.051954
A_44_P515852	Tpx2_predicted	XM_230735		0.27	1.852	0.005419
A_44_P1015220	Ncbp1	NM_001014785	298075	0.27	1.852	0.002472
A_44_P468372	Gnb1	NM_030987	24400	0.27	1.852	0.009806
A_44_P168986	RGD1562415_predicted	XM_221359		0.27	1.851	0.001153
A_44_P206399	LOC500592	XM_575965	500592	0.27	1.851	0.019956
A_44_P616925	DV715356	DV715356	500040	0.27	1.851	0.000441
A_44_P1019324	Eif4a1	NM_199372	287436	0.27	1.851	0.000679
A_44_P341910	Retnlg	NM_181625	288135	0.27	1.851	0.106317
A_42_P833106	Rad54l2_predicted	XM_343471	363135	0.27	1.850	0.000357
A_43_P10653	TC556257	TC556257		0.27	1.850	0.001932
A_43_P17109	Tomm40	NM_212520	308416	0.27	1.849	0.0043

A_44_P340362	XM_217084	XM_217084		0.27	1.849	0.00508
A_44_P234682	AW143273	AW143273		0.27	1.849	0.000879
A_43_P14210	Ppif	NM_172243	282819	0.27	1.849	0.001606
A_44_P515855	XM_226343	XM_226343		0.27	1.848	0.007832
A_44_P407877	A_44_P407877	A_44_P407877		0.27	1.848	0.017019
A_44_P210952	XM_343767	XM_343767		0.27	1.848	0.001634
A_43_P12837	Ddx52	NM_053525	85432	0.27	1.848	0.000456
A_44_P1037526	Eif3s10	XM_238649		0.27	1.848	0.001094
A_44_P653513	LOC691170	XM_001077082	691170	0.27	1.848	0.006569
A_44_P1032771	Fxyd4	NM_022388	64190	0.27	1.848	0.264995
A_44_P265682	LOC498549	XM_573823	498549	0.27	1.848	0.004547
A_44_P156433	Vcp	NM_053864	116643	0.27	1.848	0.000897
A_44_P238897	Svil_predicted	XM_341540		0.27	1.848	0.041772
A_44_P436915	AA875381	AA875381		0.27	1.848	0.016865
A_44_P439665	Fkbp4	XM_342763	260321	0.27	1.847	0.008471
A_44_P1011147	LOC310958	XM_001078082		0.27	1.847	0.012188
A_44_P786613	AW920454	AW920454		0.27	1.847	0.000414
A_44_P376624	RGD1565589_predicted	XM_573529		0.27	1.847	0.093275
A_44_P287977	Olr1522_predicted	NM_001000040	287614	0.27	1.847	0.046622
A_44_P145239	XM_220318	XM_220318		0.27	1.847	0.000952
A_42_P641323	Dnpep	NM_001024879	301529	0.27	1.847	0.004457
A_44_P468650	RGD1566135_predicted	XM_344425		0.27	1.847	0.000342
A_44_P818671	AW918229	AW918229		0.27	1.847	0.007957
A_44_P1023611	RGD1562258_predicted	XM_575576		0.27	1.846	0.001318
A_44_P143922	Hes6	NM_001013179	316626	0.27	1.846	0.003596
A_44_P493980	Nptxr	NM_030841	81005	0.27	1.846	0.009771
A_44_P286215	RT1-Ke4	NM_001008885	294281	0.27	1.846	0.006396
A_43_P18764	Nudt14_predicted	XM_216799		0.27	1.845	0.001966
A_44_P726147	Zfyve20_predicted	XM_001076679		0.27	1.845	0.024367
A_44_P185337	RGD1560187_predicted	XM_342957	362641	0.27	1.844	0.002759
A_44_P269630	Taf9	NM_001012463	373541	0.27	1.844	0.014098
A_44_P133650	Trim37_predicted	XM_340872		0.27	1.844	0.001124
A_43_P19181	Xpo7	XM_341353		0.27	1.844	0.000427
A_44_P278701	Pklr	NM_012624	24651	0.27	1.844	0.012166
A_44_P408187	Tfg	NM_001012144	360709	0.27	1.844	0.002073
A_44_P132533	Rfc4_predicted	XM_213598		0.27	1.844	0.01772
A_44_P229416	Sfxn4_predicted	XM_342072		0.27	1.844	0.002005
A_44_P487355	Zfp281	NM_001012030	305083	0.27	1.843	0.003655
A_44_P116510	Kalrn	NM_032062	84009	0.27	1.843	0.101155
A_44_P916510	Kctd5_predicted	XM_220224		0.27	1.843	0.001782
A_44_P131968	RGD1560286_predicted	XM_575942	500575	0.27	1.843	0.009549
A_44_P867760	BC087069	BC087069	366057	0.27	1.842	0.008706
A_44_P1011018	2610020o08rik	NM_001024872	298342	0.27	1.842	0.005258
A_44_P1037041	Map3k6_predicted	XM_232732		0.27	1.842	0.000626
A_44_P538870	RGD1308276	NM_001008306	294019	0.27	1.841	0.040294
A_44_P380937	CV110226	CV110226		0.27	1.841	0.100223
A_44_P501204	Nup54	NM_017361	53372	0.27	1.841	0.004231
A_44_P576019	TC552644	TC552644		0.26	1.841	0.193754
A_43_P17481	Rtf1_predicted	XM_345421		0.26	1.841	0.001767
A_44_P512996	Aldoc	NM_012497	24191	0.26	1.840	0.021886
A_44_P354018	RGD1559708_predicted	XM_224779		0.26	1.840	0.001089
A_44_P271678	Dag1	XM_343483	114489	0.26	1.840	0.002264
A_43_P18153	Glt8d1	NM_001007683	306253	0.26	1.840	0.000801
A_44_P149734	Hat1	NM_001009657	296501	0.26	1.840	0.009258
A_44_P426648	Phc3_predicted	XM_226976		0.26	1.840	0.070356
A_44_P231689	LOC292958	XR_009528	292958	0.26	1.839	0.002526
A_44_P564260	Fem1c_predicted	XM_228396		0.26	1.839	0.000389
A_44_P762420	TC558238	TC558238		0.26	1.839	0.001564
A_44_P151779	XM_346706	XM_346706		0.26	1.839	0.009287
A_44_P387308	Adamtsl4	NM_001034012	310670	0.26	1.839	0.009239
A_44_P241541	Tcp11l2	NM_001017458	314683	0.26	1.838	0.007011
A_44_P487910	Lrrc33	NM_001024995	303875	0.26	1.838	0.003082
A_44_P318604	Vps33a	NM_022961	65081	0.26	1.838	0.012904

A_44_P371552	Pcbp2	NM_001013223	363005	0.26	1.838	0.000817
A_44_P293944	Ghitm	NM_001005908	290596	0.26	1.838	0.012604
A_44_P545745	Cdh18_predicted	XM_226899		0.26	1.837	0.01651
A_44_P326259	RGD1308257	XM_342758		0.26	1.837	0.003518
A_43_P15232	Nfkb1	XM_342346	81736	0.26	1.837	0.001259
A_44_P836677	Fau	NM_001012739	29752	0.26	1.837	0.00036
A_44_P818956	LOC681623	XM_001057666		0.26	1.837	0.001684
A_42_P767403	Zbtb4_predicted	XM_220612	287441	0.26	1.836	0.001355
A_43_P15730	Sfpq	NM_001025271	252855	0.26	1.836	0.011997
A_44_P534678	Usp28_predicted	XM_236240		0.26	1.836	0.038378
A_44_P160868	Grpel1	NM_024487	79563	0.26	1.836	0.005978
A_44_P334139	LOC687346	XM_001075972		0.26	1.836	0.003391
A_43_P10161	Kifap3_predicted	XM_213920		0.26	1.836	0.000373
A_44_P487532	Tmem56_predicted	XM_345284	365924	0.26	1.835	0.116097
A_43_P10835	AW920769	AW920769	291914	0.26	1.835	0.005453
A_44_P447895	Spg20	XM_215564		0.26	1.835	0.039554
A_44_P177704	AF370889	AF370889	24851	0.26	1.835	0.001905
A_44_P177914	AW915489	AW915489	289323	0.26	1.834	0.010249
A_44_P281750	Poldip2_predicted	XM_237790		0.26	1.834	0.00138
A_44_P442889	Npy1r	NM_001013032	29358	0.26	1.834	0.022984
A_42_P462771	Cd9	XM_001063955		0.26	1.834	0.031297
A_44_P180138	Hist1h2bl	NM_022647	64647	0.26	1.833	0.038011
A_44_P445572	Sort1	XM_342317	83576	0.26	1.833	0.001582
A_44_P184896	LOC683722	XM_001064064		0.26	1.833	0.010762
A_44_P807091	Cd2bp2_predicted	XM_215082		0.26	1.833	0.016166
A_44_P942315	AW143698	AW143698		0.26	1.833	0.042786
A_44_P182412	Ccne1	XM_574426		0.26	1.833	0.016139
A_44_P959529	LOC500199	XM_001063161		0.26	1.833	0.001301
A_44_P946496	TC543869	TC543869		0.26	1.832	0.001903
A_44_P927153	Gnb1	NM_030987	24400	0.26	1.832	0.008375
A_42_P813898	Dgcr8_predicted	XM_221273		0.26	1.832	0.012775
A_44_P945096	RGD1309459	BC085931	360477	0.26	1.832	0.006432
A_44_P473472	RGD1307434_predicted	XM_342153		0.26	1.831	0.001554
A_44_P820605	AW143900	AW143900		0.26	1.831	0.000844
A_44_P288756	RGD1565117_predicted	XM_235217		0.26	1.831	0.00021
A_44_P670133	TC561992	TC561992		0.26	1.831	0.012114
A_43_P13622	Pddc1_predicted	XM_219483		0.26	1.830	0.001095
A_44_P293904	Syk	NM_012758	25155	0.26	1.830	0.002946
A_44_P408679	Ssx2ip	NM_175597	308023	0.26	1.830	0.010212
A_44_P512656	BQ200408	BQ200408	25307	0.26	1.830	0.006728
A_44_P635769	Mks1	NM_001034917	287612	0.26	1.829	0.044551
A_43_P19519	Vcl_predicted	XM_001057629		0.26	1.829	0.001111
A_44_P328283	RGD1566323_predicted	XM_219706	309256	0.26	1.829	0.005117
A_44_P227228	Rexo4	NM_001033884	311826	0.26	1.829	0.000263
A_43_P11685	Id2	NM_013060	25587	0.26	1.828	0.002398
A_43_P13967	Gsta2	NM_017013	24422	0.26	1.828	0.203672
A_43_P10222	Tmem18	NM_001007748	362722	0.26	1.828	0.004875
A_44_P356579	Fxna	NM_184050	373544	0.26	1.827	0.014472
A_44_P328421	Rab33b_predicted	XM_345208		0.26	1.827	0.035492
A_44_P914438	Cdkn1a	NM_080782	114851	0.26	1.827	0.00046
A_44_P525649	A_44_P525649	A_44_P525649		0.26	1.827	0.001037
A_44_P323661	LOC685671	XR_006259	685671	0.26	1.826	0.005545
A_43_P20550	RGD1305833	NM_001009676	302937	0.26	1.826	0.008092
A_44_P161509	Suv39h2_predicted	XM_344633		0.26	1.826	0.006902
A_43_P12835	Rhoq	NM_053522	85428	0.26	1.826	0.000359
A_42_P765356	Sephs1	XM_214509		0.26	1.826	0.014122
A_44_P501305	RGD1305631_predicted	XM_221972		0.26	1.825	0.001068
A_44_P475418	Nfia	NM_012988	25492	0.26	1.825	0.003203
A_44_P508602	Crlz1	NM_001012036	305258	0.26	1.825	0.003615
A_43_P10931	Gpr68_predicted	XM_001065526		0.26	1.825	0.029702
A_44_P974665	A_44_P974665	A_44_P974665		0.26	1.825	0.00039
A_44_P126092	Ruvbl2	NM_001025405	292907	0.26	1.825	0.000995
A_44_P482605	Gipc1	NM_053341	83823	0.26	1.825	0.000367

A_44_P443019	RGD1561198_predicted	XM_001073796		0.26	1.825	0.001232
A_44_P316342	Det1	NM_001037194	308775	0.26	1.824	0.004807
A_44_P769800	TC542803	TC542803		0.26	1.824	0.000429
A_44_P156609	Zipro1	NM_001012021	304342	0.26	1.824	0.004337
A_44_P370240	Ncor2_predicted	XM_341072		0.26	1.824	0.002621
A_44_P380243	A_44_P380243	A_44_P380243		0.26	1.824	0.001634
A_44_P492772	RGD1308396_predicted	XM_214175		0.26	1.823	0.000283
A_44_P257640	Bmpr2	XM_217409	140590	0.26	1.823	0.003597
A_44_P284110	Atad2_predicted	XM_235326	314993	0.26	1.823	0.005998
A_44_P575890	TC557416	TC557416		0.26	1.823	0.012235
A_44_P695179	Klc2_predicted	XM_219696		0.26	1.823	0.001605
A_44_P419482	LOC305913	XM_224241	305913	0.26	1.823	0.0105
A_44_P808578	TC525845	TC525845		0.26	1.822	0.075757
A_44_P222166	Cd86	NM_020081	56822	0.26	1.822	0.007145
A_44_P455271	BF556006	BF556006		0.26	1.822	0.001566
A_42_P710191	XM_225559	XM_225559		0.26	1.822	0.009642
A_44_P945920	TC541335	TC541335		0.26	1.822	0.049818
A_44_P281761	Eno1	NM_012554	24333	0.26	1.821	0.001085
A_44_P806560	ENSRNOT00000038391	ENSRNOT00000038391		0.26	1.821	0.000868
A_42_P483419	Oaz1	NM_139081	25502	0.26	1.820	0.001453
A_44_P612277	BF396985	BF396985		0.26	1.820	0.005419
A_44_P807901	Smc6l1_predicted	XM_001068591		0.26	1.820	0.020931
A_44_P347241	RGD1564397_predicted	XM_228681	302489	0.26	1.820	0.04274
A_42_P722409	Farslb	NM_001004252	301544	0.26	1.820	0.002169
A_44_P994027	Psmd8	XM_214888	292766	0.26	1.820	0.002712
A_44_P100565	A_44_P100565	A_44_P100565		0.26	1.820	0.000608
A_44_P487162	Rab6a	XM_001062702	84379	0.26	1.820	0.006375
A_42_P623909	Bcl2l1	NM_031535	24888	0.26	1.819	0.004541
A_44_P317388	Mcam	NM_023983	78967	0.26	1.819	0.083066
A_43_P12130	Glp2r	NM_021848	60432	0.26	1.819	0.002982
A_44_P945669	LOC499933	NM_001025768	499933	0.26	1.819	0.019291
A_44_P492561	Cdc42ep1_predicted	XM_235488		0.26	1.819	0.000408
A_42_P611223	Cgrrf1	NM_053899	116679	0.26	1.819	0.002882
A_44_P1007561	Vars2l	NM_213563	309596	0.26	1.819	0.011304
A_42_P772821	Bcap31	NM_001004224	293852	0.26	1.818	0.002013
A_44_P947801	LOC500893	NM_001029926	500893	0.26	1.818	0.111114
A_44_P499287	Orc6l	NM_001033690	291927	0.26	1.818	0.006088
A_42_P462299	Dnase2	NM_138539	171575	0.26	1.818	0.000869
A_44_P399678	LOC682967	XM_001063895		0.26	1.818	0.008283
A_44_P201582	RGD1306153	XM_001075665	361410	0.26	1.818	0.000903
A_44_P382484	Folr4_predicted	XM_345898		0.26	1.818	0.00757
A_44_P106615	CF106932	CF106932	313087	0.26	1.818	0.032305
A_44_P112237	ENSRNOT00000052249	ENSRNOT00000052249		0.26	1.817	0.001248
A_44_P459202	Ptpn23	NM_057204	117552	0.26	1.817	0.001654
A_43_P18106	Ahi1	NM_001002277	308923	0.26	1.816	0.002161
A_44_P480337	RGD1306359_predicted	XM_218199		0.26	1.816	0.001902
A_44_P267103	RGD1564883_predicted	XM_231785	312363	0.26	1.816	0.00192
A_44_P187830	RGD1304621_predicted	XM_221669	304104	0.26	1.816	0.001223
A_44_P243689	RGD1563359_predicted	XM_232983		0.26	1.816	0.004185
A_44_P391915	ENSRNOT00000002159	ENSRNOT00000002159		0.26	1.815	0.001116
A_44_P714054	LOC366367	XR_007494	366367	0.26	1.815	0.0014
A_42_P699937	Eif3s6ip	NM_001034134	300069	0.26	1.815	0.000194
A_43_P21653	LOC499991	XM_001067033		0.26	1.814	0.313026
A_44_P320955	Sfxn1	NM_001012213	364678	0.26	1.814	0.000576
A_44_P102671	Asam	NM_173154	286939	0.26	1.814	0.008355
A_43_P15884	Kif1b	AF155823	117548	0.26	1.814	0.024885
A_44_P203731	RGD1559647_predicted	XR_008080	310988	0.26	1.814	0.030149
A_43_P20902	Donson	NM_001008287	288257	0.26	1.813	0.002425
A_44_P327535	RGD1563508_predicted	XM_001078729		0.26	1.813	0.049039
A_44_P463973	Sephs2	XM_219347		0.26	1.813	0.009666
A_42_P829301	Slc1a5	NM_175758	292657	0.26	1.813	0.003047
A_44_P140591	Lrp12_predicted	XM_235261	314941	0.26	1.813	0.001954
A_44_P534624	RGD1562689_predicted	XM_342946	362628	0.26	1.812	0.141969

A_43_P16024	Ptpru	XM_342930	116680	0.26	1.812	0.007271
A_44_P463504	Mcam	NM_023983	78967	0.26	1.812	0.06008
A_44_P342208	A_44_P342208	A_44_P342208		0.26	1.812	0.000379
A_44_P203646	RGD1565176_predicted	XM_223044		0.26	1.812	0.014273
A_44_P446129	XM_216384	XM_216384		0.26	1.812	0.000707
A_44_P372674	Prkrir_predicted	XM_218949	308845	0.26	1.811	0.001571
A_43_P11257	Hprt	NM_012583	24465	0.26	1.811	0.000494
A_43_P15643	RGD1307982	NM_001033927	296488	0.26	1.811	0.001009
A_44_P1050728	LOC312030	XM_231361	312030	0.26	1.811	0.005202
A_44_P233907	LOC680430	XM_001057149		0.26	1.811	0.024743
A_44_P494250	Hist1h4m_predicted	XM_225382		0.26	1.811	0.02132
A_44_P217604	Msh2	NM_031058	81709	0.26	1.811	0.004003
A_44_P160321	Mrlcb	X05566	50685	0.26	1.810	0.001221
A_44_P137508	Map1lc3b	NM_022867	64862	0.26	1.810	0.002094
A_44_P319087	Rngtt_predicted	XM_232865		0.26	1.810	0.037559
A_44_P117389	M33313	M33313		0.26	1.810	0.001831
A_44_P683392	RGD1565365_predicted	XM_578751		0.26	1.810	0.000398
A_44_P123891	RGD1563262_predicted	XM_342269		0.26	1.810	0.00246
A_44_P314498	Arl6_predicted	XM_344009		0.26	1.810	0.018272
A_44_P525094	Nipa2_predicted	XM_218718		0.26	1.809	0.01575
A_44_P368862	CB545010	CB545010		0.26	1.809	0.006253
A_44_P229731	RGD1307897	BC091339	314172	0.26	1.809	0.005115
A_44_P1060362	RGD735112	NM_199391	304055	0.26	1.809	0.001322
A_44_P377179	Gbx1	XM_001063850	246149	0.26	1.809	0.056059
A_42_P773816	Cd1d1	NM_017079	25109	0.26	1.809	0.013925
A_44_P193221	XM_215028	XM_215028		0.26	1.809	0.000364
A_43_P20474	Ptprb_predicted	XM_235156		0.26	1.809	0.007399
A_43_P20479	Irf2bp1_predicted	XM_218405		0.26	1.808	0.001459
A_44_P686826	TC519897	TC519897		0.26	1.808	0.002031
A_44_P337699	RGD1309007_predicted	XM_215595	295160	0.26	1.808	0.081281
A_44_P341285	Rtcd1	NM_001004227	295395	0.26	1.808	0.005698
A_44_P468803	Ythdf1	NM_001024756	296467	0.26	1.808	0.002553
A_44_P259453	AA997347	AA997347	292764	0.26	1.808	0.027023
A_44_P345194	AA818425	AA818425		0.26	1.808	0.079922
A_44_P284859	Htatsf1_predicted	XM_229199		0.26	1.808	0.006467
A_44_P671422	TC517984	TC517984		0.26	1.808	0.0303
A_44_P525665	Atg16l1_predicted	XM_343619		0.26	1.807	0.005683
A_44_P137262	Nmt1	NM_148891	259274	0.26	1.807	0.001236
A_44_P340632	RGD1307234_predicted	XM_244261	316732	0.26	1.807	0.002716
A_44_P792495	TC524051	TC524051		0.26	1.807	0.010525
A_44_P532145	XM_341476	XM_341476		0.26	1.807	0.014465
A_43_P10136	RGD1304846	NM_001014121	360650	0.26	1.807	0.0023
A_44_P293302	Cep57	XM_235823		0.26	1.807	0.014736
A_42_P536326	Bicd2	NM_198765	306809	0.26	1.807	0.000339
A_43_P22222	RGD1308087	NM_001030036	305861	0.26	1.806	0.018293
A_44_P792409	TC557492	TC557492		0.26	1.806	0.014633
A_44_P1026688	Sypl	NM_001014263	366595	0.26	1.806	0.005739
A_44_P445938	P4ha2_predicted	XM_340798		0.26	1.806	0.095461
A_44_P778450	TC545912	TC545912		0.26	1.806	0.236055
A_44_P957773	AA963699	AA963699		0.26	1.806	0.029565
A_44_P396294	XM_228842	XM_228842		0.26	1.806	0.001592
A_44_P463309	Plk1	NM_017100	25515	0.26	1.805	0.003645
A_43_P20016	Rkhd2_predicted	XM_225735		0.26	1.805	0.002506
A_44_P149293	Trim23	XM_342183	81002	0.26	1.805	0.021666
A_44_P513490	RGD1311742	NM_001025636	291676	0.26	1.805	0.002368
A_44_P485369	Dock11	XM_233283	313438	0.26	1.805	0.021658
A_44_P130582	Prelp	NM_053385	84400	0.26	1.805	0.048921
A_44_P787598	AW915403	AW915403	56822	0.26	1.805	0.041909
A_44_P1012567	Fads3	NM_173137	286922	0.26	1.805	0.005695
A_44_P418320	BM986276	BM986276	29497	0.26	1.804	0.000565
A_44_P227361	Bms1l	XM_342746	362426	0.26	1.804	0.000607
A_44_P342858	Ephb2_predicted	XM_233574	313633	0.26	1.804	0.009521
A_44_P137397	Pebp1	NM_017236	29542	0.26	1.804	0.001308

A_44_P647968	BI395759	BI395759		0.26	1.804	0.000381
A_43_P10511	XM_342289	XM_342289		0.26	1.804	0.001008
A_44_P789583	AW921101	AW921101	501569	0.26	1.803	0.094019
A_44_P518500	Racgap1_predicted	XM_235650		0.26	1.803	0.002611
A_44_P388630	AW917689	AW917689		0.26	1.803	0.00219
A_44_P730398	Taf1_predicted	XM_001061884		0.26	1.803	0.012274
A_44_P351438	RGD1560575_predicted	XR_009430	497965	0.26	1.803	0.010347
A_44_P142194	AY539899	AY539899		0.26	1.802	0.011742
A_44_P297134	Pdcd2	XM_341763	58934	0.26	1.802	0.018454
A_44_P521237	RGD1307882_predicted	XM_236501	315903	0.26	1.802	0.000984
A_44_P466873	Dscr2_predicted	XM_213645		0.26	1.802	0.000857
A_42_P822048	S70011	S70011		0.26	1.802	0.000413
A_44_P123887	Smc4l1	XM_001066172		0.26	1.802	0.005175
A_44_P755445	A_44_P755445	A_44_P755445		0.26	1.802	0.091443
A_42_P552143	Cfl1	NM_017147	29271	0.26	1.802	0.003384
A_44_P212835	RGD1562827_predicted	XM_577577	502129	0.26	1.802	0.049215
A_44_P127014	Nek9_predicted	XM_216755		0.26	1.802	0.00192
A_44_P527154	RGD1564767_predicted	NM_001024282	498753	0.26	1.802	0.050681
A_44_P151905	LOC305181	XR_006068	305181	0.26	1.801	0.003312
A_44_P114729	RGD1308048_predicted	XM_216543	298557	0.26	1.801	0.00705
A_44_P250673	RGD1307018	XM_342417		0.26	1.801	0.012764
A_42_P648826	Ankrd52_predicted	XM_343139	362811	0.26	1.801	0.001046
A_44_P930283	DV716667	DV716667		0.26	1.800	0.01243
A_44_P306355	Plod1	NM_053827	116552	0.26	1.800	0.001364
A_44_P729258	A_44_P729258	A_44_P729258		0.26	1.800	0.066383
A_44_P885330	TC525733	TC525733		0.26	1.800	0.188366
A_44_P728960	ENSRNOT00000000702	ENSRNOT00000000702		0.26	1.800	0.000481
A_42_P843592	Nudcd1_predicted	XM_001063815		0.26	1.800	0.087301
A_44_P808337	DV718357	DV718357		0.26	1.799	0.01442
A_44_P1030714	Glul	NM_017073	24957	0.26	1.799	0.008879
A_44_P533723	AW253367	AW253367	288710	0.25	1.799	0.012544
A_44_P371629	AW142568	AW142568		0.25	1.798	0.001899
A_44_P269078	Tcp1	NM_012670	24818	0.25	1.798	0.002337
A_43_P21138	RGD1306873	NM_001037191	304285	0.25	1.798	0.002929
A_44_P534585	XM_346249	XM_346249		0.25	1.798	0.163634
A_44_P1031524	Rpl34_predicted	XM_342342		0.25	1.798	0.001579
A_44_P251897	AI010087	AI010087	287379	0.25	1.798	0.111394
A_43_P17033	Pum2	XM_216661		0.25	1.798	0.015548
A_44_P515494	Ddr1	NM_013137	25678	0.25	1.798	0.002502
A_44_P501602	ENSRNOT00000052065	ENSRNOT00000052065		0.25	1.797	0.001139
A_44_P523170	A_44_P523170	A_44_P523170		0.25	1.797	0.001485
A_44_P213363	Pik3cd_predicted	XM_345606		0.25	1.797	0.007097
A_44_P325782	Hsd3b7	NM_139329	246211	0.25	1.796	0.003138
A_44_P1070510	Col4a1	XM_214400	290905	0.25	1.796	0.00695
A_43_P19366	RGD1562321_predicted	XM_230665		0.25	1.796	0.001101
A_44_P531553	AW917981	AW917981		0.25	1.796	0.014428
A_44_P295385	XM_341294	XM_341294		0.25	1.796	0.001479
A_43_P11556	Tgfbr1	NM_012775	29591	0.25	1.795	0.002158
A_44_P373272	LOC680409	XM_001058939	680409	0.25	1.795	0.006918
A_44_P510279	BF542361	BF542361		0.25	1.795	0.00348
A_43_P18884	Zbtb1	NM_001004444	314246	0.25	1.795	0.000975
A_42_P834176	Dapk3	NM_022546	64391	0.25	1.794	0.004393
A_43_P15356	Mgea5	NM_131904	154968	0.25	1.794	0.023339
A_44_P436078	Cutc_predicted	XM_342052		0.25	1.794	0.138304
A_44_P1049242	Ard1_predicted	XM_343842	363518	0.25	1.794	0.001171
A_44_P508733	LOC691420	XM_001078179	691420	0.25	1.794	0.004265
A_44_P501082	Rap1b	NM_134346	171337	0.25	1.794	0.018516
A_44_P845610	TC544906	TC544906		0.25	1.794	0.011487
A_44_P823729	TC525308	TC525308		0.25	1.794	0.030853
A_44_P521687	Dnaja2	NM_032079	84026	0.25	1.794	0.001013
A_44_P391599	Tbc1d7_predicted	XM_341510		0.25	1.793	0.00233
A_44_P791545	CO400785	CO400785		0.25	1.793	0.086509
A_44_P946665	TC527810	TC527810		0.25	1.793	0.079125

A_44_P745166	RGD1311283_predicted	XM_001065858		0.25	1.793	0.080495
A_43_P11906	Kcnab2	NM_017304	29738	0.25	1.793	0.001871
A_44_P180854	A_44_P180854	A_44_P180854		0.25	1.793	0.001991
A_44_P367345	AA957788	AA957788	315548	0.25	1.793	0.038972
A_44_P286086	RGD1564291_predicted	XM_001070073		0.25	1.792	0.00097
A_43_P15275	Ptprj	NM_017269	29645	0.25	1.792	0.003966
A_44_P404567	Ptbp1	NM_022516	29497	0.25	1.792	0.002382
A_43_P15923	Nav2	XM_341864	171563	0.25	1.792	0.004332
A_44_P265911	Cnr2	NM_020543	57302	0.25	1.792	0.095551
A_43_P12409	Anxa4	NM_024155	79124	0.25	1.792	0.006458
A_44_P170849	Anapc4	XM_223496		0.25	1.791	0.008214
A_44_P309398	LOC691947	XM_001080279	311371	0.25	1.791	0.003017
A_44_P553037	Fyb_predicted	XM_226812	310131	0.25	1.791	0.009078
A_44_P771409	BG667707	BG667707		0.25	1.791	0.005062
A_44_P220630	Cbx1_predicted	XM_340885	360609	0.25	1.791	0.001231
A_44_P260522	RGD1561252_predicted	XR_008133	366382	0.25	1.791	0.001832
A_43_P15645	Hnrpm	NM_053876	116655	0.25	1.791	0.000514
A_44_P998042	RGD1309621	XM_229579	316982	0.25	1.790	0.005111
A_44_P869273	TC522992	TC522992		0.25	1.790	0.15047
A_44_P396382	LOC313707	XR_007065	313707	0.25	1.790	0.001409
A_42_P544321	AI136185	AI136185		0.25	1.790	0.000381
A_43_P10593	Stat1	NM_032612	25124	0.25	1.790	0.02217
A_44_P208624	RGD1308612	XM_001059141		0.25	1.790	0.005427
A_44_P1037771	Znf593_predicted	XM_216542		0.25	1.790	0.000214
A_44_P358756	Frem2_predicted	XM_227142	310418	0.25	1.789	0.041776
A_42_P751001	Hmgn2	NM_001025624	114637	0.25	1.789	0.003047
A_44_P1024165	AW915150	AW915150		0.25	1.789	0.076444
A_44_P541914	Dapk3	NM_022546	64391	0.25	1.789	0.006186
A_44_P349818	XM_237243	XM_237243		0.25	1.789	0.000447
A_44_P383928	Phyh2	NM_053493	85255	0.25	1.789	0.12899
A_44_P525681	RGD1566186_predicted	XM_221698		0.25	1.789	0.013179
A_44_P1046787	RGD1559717_predicted	XM_216317	297757	0.25	1.789	0.072035
A_44_P342773	Mdh1b_predicted	XM_237203		0.25	1.789	0.004899
A_44_P177801	Hnrpf	NM_022397	64200	0.25	1.788	0.005308
A_44_P432109	Txnl2	NM_032614	58815	0.25	1.788	0.012374
A_44_P519251	Cd14	NM_021744	60350	0.25	1.788	0.00673
A_44_P197285	AA956971	AA956971		0.25	1.788	0.010235
A_44_P836678	LOC681191	XM_001058530		0.25	1.788	0.003323
A_44_P701025	TC526771	TC526771		0.25	1.788	0.031815
A_44_P307964	LOC366277	NM_001014259	366277	0.25	1.788	0.001551
A_44_P288015	Mrps10	NM_001008859	363187	0.25	1.787	0.009776
A_44_P508864	XM_228869	XM_228869		0.25	1.787	0.002187
A_42_P547305	AW920735	AW920735		0.25	1.787	0.001565
A_44_P105213	A_44_P105213	A_44_P105213		0.25	1.787	0.00037
A_43_P17427	Chic2_predicted	XM_214031		0.25	1.787	0.001198
A_42_P728389	Snx27	NM_152847	260323	0.25	1.786	0.033395
A_44_P884444	RGD1563952_predicted	XM_343782	363463	0.25	1.786	0.005137
A_44_P548543	RGD1306721	XM_342602	362288	0.25	1.786	0.012678
A_43_P12622	Pgcp	NM_031640	58952	0.25	1.786	0.046996
A_44_P543017	Nhlrc1	NM_199236	364682	0.25	1.785	0.070791
A_44_P492629	XM_340867	XM_340867		0.25	1.785	0.001017
A_44_P201693	RGD1564536_predicted	XR_007706	367300	0.25	1.785	0.002677
A_44_P170070	Rnf111_predicted	XM_236380		0.25	1.785	0.063848
A_44_P820421	BF522212	BF522212	316241	0.25	1.785	0.000361
A_44_P250892	RGD1309198_predicted	XM_232775	313056	0.25	1.785	0.006782
A_44_P368723	Cstf3_predicted	XM_001072972		0.25	1.785	0.024492
A_44_P431198	Pycr2	NM_001012208	364064	0.25	1.784	0.00075
A_43_P15574	Dpp7	NM_031973	83799	0.25	1.784	0.010036
A_44_P298013	LOC497867	XM_001076872		0.25	1.784	0.000766
A_44_P236566	Neurl2_predicted	XM_230848		0.25	1.784	0.000738
A_44_P960243	Zbed4_predicted	XM_235553	315211	0.25	1.784	0.002987
A_44_P243881	ENSRNOT00000029821	ENSRNOT00000029821		0.25	1.784	0.005095
A_44_P333433	Larp1_predicted	XM_220446	303158	0.25	1.784	0.00475

A_44_P187595	Reck_predicted	XM_233371		0.25	1.784	0.007815
A_44_P548025	Hnrpm	NM_053876	116655	0.25	1.783	0.01021
A_44_P105476	LOC302804	XR_008523	302804	0.25	1.783	0.019955
A_44_P511608	XM_226441	XM_226441		0.25	1.783	0.001227
A_44_P592238	TC540467	TC540467		0.25	1.783	0.001102
A_43_P12727	Stat1	NM_032612	25124	0.25	1.783	0.005098
A_44_P158957	RGD1563095_predicted	XM_221915	288485	0.25	1.783	0.012869
A_44_P102822	Mrvldc1	XM_219885		0.25	1.782	0.048427
A_44_P423613	Ehd4	NM_139324	192204	0.25	1.782	0.00218
A_44_P142652	Pcgf1	NM_001007000	312480	0.25	1.782	0.00421
A_43_P12896	Yme1l1	NM_053682	114217	0.25	1.782	0.036851
A_44_P513251	LOC684325	XM_001069884		0.25	1.782	0.003252
A_42_P555426	TC523746	TC523746		0.25	1.782	0.012796
A_43_P18844	Mtap_predicted	XM_001055425		0.25	1.781	0.00312
A_44_P366118	RGD1565010_predicted	XM_234947	314660	0.25	1.781	0.000754
A_44_P351585	Gpatc1_predicted	XM_214906		0.25	1.781	0.06167
A_44_P379751	XM_222068	XM_222068		0.25	1.781	0.010007
A_44_P496216	Hnrpu	NM_057139	117280	0.25	1.781	0.000786
A_44_P144966	Abcf2_predicted	XM_001057136		0.25	1.780	0.001734
A_44_P325268	LOC498353	NM_001017499	498353	0.25	1.780	0.005877
A_44_P162882	AI144951	AI144951		0.25	1.780	0.113935
A_44_P993359	LOC686796	XM_001075767		0.25	1.780	0.000725
A_44_P606035	A_44_P606035	A_44_P606035		0.25	1.780	0.001964
A_44_P277026	Apol3	NM_001013175	315108	0.25	1.780	0.010407
A_44_P533596	DV724511	DV724511		0.25	1.780	0.197577
A_44_P590876	RGD1562105_predicted	XM_575596		0.25	1.780	0.173367
A_42_P648200	MGC125015	NM_001037356	361232	0.25	1.780	0.002679
A_43_P22977	Strn3	NM_001029897	114520	0.25	1.780	0.003597
A_43_P16995	Scye1	NM_053757	114632	0.25	1.779	0.000698
A_44_P302102	XM_341179	XM_341179		0.25	1.779	0.022904
A_44_P227354	Bms1l	XM_342746	362426	0.25	1.779	0.002494
A_44_P494894	Ttc1	NM_001005529	287208	0.25	1.779	0.012259
A_44_P1056810	Psmd1	NM_031978	83806	0.25	1.779	0.000581
A_44_P339757	AF050661	AF050661		0.25	1.779	0.082206
A_44_P373178	Pphln1_predicted	XM_345865		0.25	1.779	0.024263
A_44_P100740	Nudt3	NM_001024243	294292	0.25	1.778	0.000795
A_44_P151459	Gp1bb	NM_053930	116728	0.25	1.778	0.214678
A_44_P166228	Rpusd2_predicted	XM_230464	311326	0.25	1.778	0.00103
A_44_P571699	AI227836	AI227836		0.25	1.778	0.002019
A_44_P1016350	Smu1	NM_057195	117541	0.25	1.778	0.001259
A_43_P18346	Surf6_predicted	NM_001015014	303076	0.25	1.778	0.012644
A_44_P711740	AW914791	AW914791	359728	0.25	1.778	0.079383
A_44_P433957	Zmym4_predicted	XM_233529		0.25	1.777	0.008107
A_44_P530320	CB547657	CB547657		0.25	1.777	0.107292
A_44_P1031202	Park7	NM_057143	117287	0.25	1.777	0.00107
A_44_P310152	Rps21	NM_031111	81775	0.25	1.777	0.001458
A_44_P447814	LOC680477	XM_001057628		0.25	1.777	0.009627
A_44_P253641	Glmn	XM_213992		0.25	1.776	0.050443
A_44_P412301	Rpl14	NM_022949	65043	0.25	1.776	0.00475
A_44_P1054213	Hspa5	NM_013083	25617	0.25	1.776	0.004361
A_44_P1015203	RGD1562732_predicted	XM_574740	499422	0.25	1.776	0.007562
A_44_P316440	RGD1311881	NM_001035251	310091	0.25	1.776	0.002163
A_44_P958535	AW917755	AW917755		0.25	1.775	0.058206
A_44_P215087	Exoc4	XM_342655	116654	0.25	1.775	0.016253
A_44_P1035353	TC519348	TC519348		0.25	1.775	0.035107
A_44_P429483	RGD1308154_predicted	XM_344027	363799	0.25	1.775	0.000421
A_43_P19969	LOC683313	XM_001065178		0.25	1.775	0.119291
A_44_P199420	RGD1309188_predicted	XM_235924		0.25	1.775	0.001444
A_44_P473687	Zcrb1	NM_001034940	362990	0.25	1.775	0.025574
A_44_P1007110	RGD1565432_predicted	XM_574455	499157	0.25	1.775	0.009008
A_44_P984629	Dd5	XM_576252	117060	0.25	1.775	0.000882
A_43_P17644	RGD1310879_predicted	XM_223282		0.25	1.775	0.000599
A_44_P760998	RGD1562135_predicted	XM_575333	499979	0.25	1.774	0.001171

A_44_P372461	Preb	BC078936	58842	0.25	1.774	0.002059
A_44_P221295	Acy1l2_predicted	XM_232864		0.25	1.774	0.003843
A_44_P262949	XM_216729	XM_216729		0.25	1.774	0.01332
A_44_P180012	Dgat2	NM_001012345	252900	0.25	1.774	0.01306
A_44_P106014	Cetn2	XM_215222	84593	0.25	1.774	0.015173
A_42_P831092	Baz1b	XM_347166	368002	0.25	1.774	0.000502
A_44_P485246	XM_231092	XM_231092		0.25	1.773	0.009601
A_42_P550344	Tle3	NM_053400	84424	0.25	1.773	0.006315
A_44_P534781	RGD1564550_predicted	XM_220862		0.25	1.773	0.007678
A_44_P142817	Fem1b_predicted	XM_236321		0.25	1.773	0.002267
A_44_P945544	TC556606	TC556606		0.25	1.773	0.046964
A_44_P357111	RGD1565498_predicted	XM_576234		0.25	1.773	0.014195
A_44_P817849	LOC680726	XM_001061391	680726	0.25	1.772	0.017548
A_44_P199487	RGD1304653_predicted	XM_343551		0.25	1.772	0.001897
A_44_P820225	BF289455	BF289455	499437	0.25	1.772	0.008704
A_44_P286506	Tada2l	NM_001012141	360581	0.25	1.772	0.005355
A_43_P12819	Pola2	NM_053480	85242	0.25	1.772	0.001883
A_44_P124091	RGD1563301_predicted	XM_232669		0.25	1.772	0.000374
A_44_P337351	Cxcl12	NM_001033883	24772	0.25	1.771	0.150711
A_43_P10170	RGD1565043_predicted	XM_001074859		0.25	1.771	0.031055
A_44_P681064	CO405369	CO405369		0.25	1.770	0.002252
A_44_P550228	Lamb2	NM_012974	25473	0.25	1.770	0.024974
A_42_P678870	Cyp2f2	NM_019303	54246	0.25	1.770	0.065111
A_44_P294817	Blmh	NM_001034163	287552	0.25	1.770	0.00326
A_44_P170907	XM_346935	XM_346935		0.25	1.770	0.230612
A_44_P508055	Bcl2l11	NM_171988	64547	0.25	1.770	0.00662
A_42_P753130	XM_219231	XM_219231		0.25	1.769	0.012734
A_44_P790767	A_44_P790767	A_44_P790767		0.25	1.769	0.001184
A_44_P217451	Adora3	NM_012896	25370	0.25	1.769	0.052711
A_44_P146821	Hivep2	NM_024137	29721	0.25	1.769	0.003883
A_44_P158675	Wdr46	NM_212491	309628	0.25	1.769	0.001317
A_44_P332817	Cdc5l	NM_053527	85434	0.25	1.769	0.00687
A_44_P284129	Axud1_predicted	XM_343504		0.25	1.769	0.000801
A_44_P1002693	Hddc2_predicted	XM_341741		0.25	1.769	0.001427
A_44_P165588	Gpr77	NM_001003710	445269	0.25	1.769	0.073867
A_44_P531752	Bcat2	NM_022400	64203	0.25	1.768	0.025155
A_44_P776383	TC552845	TC552845		0.25	1.768	0.002169
A_44_P948069	A_44_P948069	A_44_P948069		0.25	1.768	0.016767
A_44_P461085	Gtf2h1_predicted	XM_341863		0.25	1.768	0.003289
A_44_P688137	TC564875	TC564875		0.25	1.768	0.002581
A_44_P397875	Khsrp	NM_133602	171137	0.25	1.768	0.000589
A_44_P435996	Slc41a1_predicted	XM_344140		0.25	1.767	0.006029
A_42_P797206	XM_342555	XM_342555		0.25	1.767	0.001945
A_44_P244040	LOC296462	NM_001013924	296462	0.25	1.767	0.007139
A_44_P961883	TC525644	TC525644		0.25	1.767	0.004896
A_44_P667382	A_44_P667382	A_44_P667382		0.25	1.767	0.003543
A_44_P365925	Cdh26_predicted	XM_230942		0.25	1.767	0.007002
A_44_P185195	Fbxw9	XM_213838	288921	0.25	1.767	0.003221
A_42_P811559	C1qbp	NM_019259	29681	0.25	1.767	0.00198
A_43_P12333	RT1-M3	NM_022921	24747	0.25	1.767	0.000985
A_44_P539947	Syncrip	XM_001065902		0.25	1.766	0.019669
A_44_P400421	Csnk2b	NM_031021	81650	0.25	1.766	0.004457
A_42_P632078	Gmps	NM_001024754	295088	0.25	1.766	0.001341
A_44_P307308	LOC301725	XM_237568		0.25	1.766	0.003235
A_44_P397526	Psmc1	NM_057123	117263	0.25	1.766	0.000605
A_44_P142553	Hoxd4_predicted	XM_221521		0.25	1.766	0.181854
A_44_P181511	Phf17_predicted	XM_227074		0.25	1.766	0.023444
A_44_P391372	LOC292471	XR_005720	292471	0.25	1.766	0.001049
A_44_P509099	Cnot8	NM_001008382	363603	0.25	1.765	0.025548
A_43_P13277	Psmc3ip	NM_134458	140938	0.25	1.765	0.097369
A_43_P16457	Creld2	NM_001037208	362978	0.25	1.765	0.012857
A_44_P334195	Qars	NM_001007624	290868	0.25	1.765	0.006513
A_44_P426823	Phc1_predicted	XM_232336		0.25	1.765	0.01643

A_44_P417658	Tfpt	NM_138870	85423	0.25	1.765	0.001853
A_44_P347187	RGD1564836_predicted	XM_228930		0.25	1.764	0.002909
A_42_P765499	Armc1_predicted	XM_215537		0.25	1.764	0.021264
A_44_P354062	Tram1l1_predicted	XM_227674		0.25	1.764	0.002489
A_44_P464393	Ccdc59_predicted	XM_235154		0.25	1.764	0.008147
A_42_P632305	Fkbp3_predicted	XM_216717		0.25	1.764	0.021751
A_44_P995038	RGD1561181_predicted	XM_575909		0.25	1.763	0.012992
A_43_P14410	TC522491	TC522491		0.25	1.763	0.004326
A_44_P503662	Ftsj2_predicted	XM_221949		0.25	1.763	0.00163
A_44_P445911	Maff_predicted	XM_345857	366960	0.25	1.763	0.003686
A_42_P767737	LOC680782	XM_001058844	680782	0.25	1.763	0.002817
A_44_P382831	Epm2aip1_predicted	XM_236659	316021	0.25	1.763	0.003919
A_44_P426558	Hspd1	NM_022229	63868	0.25	1.763	0.00284
A_44_P274986	BU671460	BU671460	1E+08	0.25	1.762	0.000707
A_44_P601053	CO563830	CO563830		0.25	1.762	0.001204
A_44_P290375	Ptgir_predicted	XM_218457		0.25	1.762	0.141288
A_44_P452731	Suv39h1_predicted	XM_001063363		0.25	1.762	0.003425
A_44_P477620	Pla2g4a	NM_133551	24653	0.25	1.762	0.012291
A_44_P729217	ENSRNOT00000038255	ENSRNOT00000038255		0.25	1.762	0.037024
A_44_P564121	DV726863	DV726863	316810	0.25	1.762	0.042496
A_44_P463659	Cmtm6	NM_001007802	316035	0.25	1.762	0.011825
A_44_P808724	TC525179	TC525179		0.25	1.762	0.015702
A_44_P306863	XM_344447	XM_344447		0.25	1.762	0.000963
A_44_P700588	TC542097	TC542097		0.25	1.762	0.001143
A_44_P511304	Lck_mapped	XM_232763	313050	0.25	1.762	0.002068
A_44_P198947	RGD1306245_predicted	XM_218481		0.25	1.761	0.051977
A_44_P160669	Ddost_predicted	NM_001012104	313648	0.25	1.761	0.007196
A_42_P654166	RGD1304593_predicted	XM_342288	361988	0.25	1.761	0.000775
A_44_P525324	RGD1309107	XM_230459	296060	0.25	1.761	0.021883
A_44_P333165	RGD1304869_predicted	NM_001014153	361402	0.25	1.761	0.004145
A_44_P496631	Timm9	XM_001072892	171139	0.25	1.761	0.002651
A_44_P720428	A_44_P720428	A_44_P720428		0.25	1.761	0.007179
A_44_P233736	Tspan5	NM_001004090	362048	0.25	1.761	0.004236
A_44_P1003072	Dusp11	NM_001025650	297412	0.25	1.760	0.001736
A_44_P231817	RGD1562409_predicted	XM_575149		0.25	1.760	0.000815
A_44_P223808	Pex19	XM_225711		0.25	1.760	0.001153
A_44_P384483	RGD1564325_predicted	XM_224616		0.25	1.760	0.001261
A_44_P996611	XM_345470	XM_345470		0.25	1.760	0.000567
A_44_P221742	Psmd5_predicted	XM_216041		0.25	1.760	0.010893
A_44_P226858	Mrfap1	NM_001009264	282585	0.25	1.760	0.005506
A_44_P196663	Trove2_predicted	XM_222686		0.25	1.759	0.008479
A_44_P516045	Znf124_predicted	XM_234843		0.25	1.759	0.001096
A_44_P158783	Aoc3	NM_031582	29473	0.25	1.759	0.061494
A_44_P555561	LOC691729	XM_001073038		0.25	1.758	0.007134
A_44_P158216	Pik3r2	NM_022185	29741	0.25	1.758	0.008893
A_44_P118793	AY325158	AY325158		0.25	1.758	0.001991
A_44_P275664	LOC688133	XM_001081155		0.24	1.758	0.030812
A_44_P900349	TC559247	TC559247		0.24	1.757	0.004799
A_44_P639519	TC528331	TC528331		0.24	1.757	0.013379
A_44_P809374	Clcf1	NM_207615	365395	0.24	1.757	0.010251
A_44_P1023480	RGD1309020	XM_214453		0.24	1.757	0.224776
A_43_P20922	RGD1308221_predicted	XM_222477	304645	0.24	1.757	0.004303
A_44_P492594	RGD1308696	NM_001008278	287278	0.24	1.757	0.001809
A_44_P471104	Vps18_predicted	XM_230471		0.24	1.756	0.016485
A_43_P10488	R3hcc1_predicted	XM_341347	361064	0.24	1.756	0.000584
A_44_P321931	Ddx10_predicted	XM_236263		0.24	1.756	0.008021
A_44_P475705	XM_345051	XM_345051		0.24	1.756	0.000863
A_43_P14881	Ssrp1	NM_031121	81785	0.24	1.756	0.001228
A_44_P789108	BG380705	BG380705		0.24	1.756	0.044288
A_44_P135224	Plk3	XM_342888	58936	0.24	1.756	0.008236
A_44_P290231	Psap	NM_013013	25524	0.24	1.756	0.035056
A_44_P356014	Cog6	NM_001004262	310411	0.24	1.756	0.104097
A_44_P789180	RGD1560155_predicted	XM_225523		0.24	1.755	0.195038

A_44_P792746	TC558767	TC558767		0.24	1.755	0.007702
A_44_P676347	TC559389	TC559389		0.24	1.755	0.008872
A_44_P139916	Hdac2	XM_342149	84577	0.24	1.754	0.002613
A_44_P730150	TC556399	TC556399		0.24	1.754	0.004145
A_43_P10754	H13_predicted	XM_230734		0.24	1.754	0.001209
A_44_P407722	RGD1565495_predicted	XR_008573	306336	0.24	1.754	0.003567
A_44_P150860	XM_346663	XM_346663		0.24	1.754	0.000829
A_44_P256052	BF556510	BF556510		0.24	1.754	0.037534
A_43_P12339	Chd8	XM_573762	65027	0.24	1.754	0.013123
A_44_P288327	XM_223346	XM_223346		0.24	1.753	0.002206
A_44_P838573	TC557849	TC557849		0.24	1.753	0.227469
A_44_P518489	RGD1311559_predicted	XM_235398	315055	0.24	1.753	0.00179
A_42_P561149	CB547219	CB547219		0.24	1.753	0.004821
A_44_P219128	AW527533	AW527533		0.24	1.753	0.021067
A_44_P252666	Nup62	NM_023098	65274	0.24	1.753	0.000539
A_42_P505972	Ube2s_predicted	XM_214806		0.24	1.753	0.003796
A_44_P146578	AW917539	AW917539		0.24	1.753	0.002507
A_44_P1021476	Zfp637	XM_342745	362425	0.24	1.753	0.004796
A_44_P250319	Npepps	XM_340889		0.24	1.753	0.00374
A_44_P348614	Sp110	NM_001034137	301570	0.24	1.752	0.031522
A_44_P480817	Mmp19_predicted	XM_222317		0.24	1.752	0.014787
A_44_P440466	Garnl1	AF041106	56785	0.24	1.752	0.010109
A_44_P192364	RGD1563579_predicted	XM_346339		0.24	1.751	0.000415
A_43_P10939	XM_217175	XM_217175		0.24	1.751	0.019536
A_43_P19215	Deadc1_predicted	XM_341731	361453	0.24	1.751	0.002267
A_43_P21669	Smug1	NM_177934	315344	0.24	1.751	0.001757
A_43_P21150	Tbc1d15	XM_345825	366896	0.24	1.751	0.008256
A_44_P466757	RGD1564007_predicted	XM_217153	300761	0.24	1.751	0.000425
A_44_P621838	ENSRNOT00000035664	ENSRNOT00000035664		0.24	1.750	0.009229
A_42_P660936	Srp19_predicted	XM_214599		0.24	1.750	0.01272
A_44_P120425	AI230862	AI230862	308430	0.24	1.750	0.020708
A_44_P245864	RGD1309487	NM_001013909	294783	0.24	1.750	0.008547
A_43_P11285	XM_345604	XM_345604		0.24	1.750	0.004075
A_44_P534089	Ccnb1	NM_171991	25203	0.24	1.749	0.004829
A_44_P191753	Wars	NM_001013170	314442	0.24	1.749	0.00223
A_44_P389688	Wdr68_predicted	XM_221032		0.24	1.749	0.003165
A_44_P252417	Abcc1	NM_022281	24565	0.24	1.749	0.000683
A_44_P325337	Prmt4	NM_001030041	363026	0.24	1.749	0.002553
A_44_P405790	LOC683377	XM_001065691		0.24	1.749	0.001764
A_44_P351159	Nfs1	NM_053462	84594	0.24	1.749	0.005094
A_44_P823844	TC542378	TC542378		0.24	1.749	0.09672
A_44_P373103	Ston2_predicted	XM_234454	314349	0.24	1.748	0.006213
A_44_P345081	RGD1307309_predicted	XM_235513	315164	0.24	1.748	0.016519
A_44_P849099	Osbpl11_predicted	XM_001065111		0.24	1.748	0.020931
A_44_P126021	Igf1	M17335	24482	0.24	1.747	0.145824
A_44_P480749	LOC368084	XM_347254	368084	0.24	1.747	0.000831
A_43_P22481	Pigo_predicted	XM_233141	313341	0.24	1.747	0.001381
A_44_P461451	Limk2	NM_024135	29524	0.24	1.747	0.013764
A_42_P577458	Psmb2	NM_017284	29675	0.24	1.747	0.002197
A_44_P404931	Znrd1	NM_213567	361784	0.24	1.746	0.001903
A_44_P898303	A_44_P898303	A_44_P898303		0.24	1.746	0.002809
A_42_P674683	Mrpl17	NM_133539	171061	0.24	1.746	0.000414
A_44_P396327	XM_238572	XM_238572		0.24	1.746	0.0009
A_44_P442976	Adck4	NM_001012065	308453	0.24	1.746	0.002522
A_43_P19511	Dhx9_predicted	XM_239780		0.24	1.746	0.003324
A_44_P386664	LOC306962	XM_225372	306962	0.24	1.746	0.055342
A_44_P446994	BF550209	BF550209	305240	0.24	1.746	0.006833
A_44_P180268	Lrrc8d	NM_001008338	305131	0.24	1.746	0.003535
A_44_P295187	Gmip_predicted	XM_224742		0.24	1.746	0.006958
A_43_P10318	Amfr_predicted	XM_001062954		0.24	1.746	0.007732
A_44_P625309	Dhx36_predicted	XM_001058645		0.24	1.745	0.007562
A_44_P236378	LOC289672	XR_007696	289672	0.24	1.745	0.003367
A_44_P591857	TC539072	TC539072		0.24	1.745	0.002175

A_44_P503996	Rsbn1l_predicted	XM_231305	311987	0.24	1.745	0.071335
A_44_P330755	Unc5d_predicted	XM_240446		0.24	1.745	0.042851
A_42_P722299	RGD1566242_predicted	XM_215704	295457	0.24	1.745	0.000594
A_44_P140223	Eif4g1	XM_001060756		0.24	1.745	0.00869
A_44_P956958	AW143890	AW143890		0.24	1.745	0.01726
A_42_P596503	Camlg	NM_053334	81715	0.24	1.744	0.058465
A_43_P17650	RGD1305984	NM_001034093	365668	0.24	1.744	0.001039
A_44_P117216	RGD1310852	NM_001025007	314992	0.24	1.744	0.001137
A_44_P189026	AW913920	AW913920	361255	0.24	1.744	0.019422
A_44_P942198	BC086438	BC086438		0.24	1.744	0.05203
A_44_P389142	Mesdc1	NM_001013149	308795	0.24	1.744	0.008271
A_44_P135290	Rest	NM_031788	83618	0.24	1.744	0.002454
A_44_P457521	RGD1306451_predicted	XM_237515	316731	0.24	1.744	0.016425
A_44_P651922	Rpl29	NM_017150	29283	0.24	1.744	0.000458
A_42_P800163	Rpl23a	XM_001060002		0.24	1.744	0.000418
A_44_P412724	Usp45_predicted	XM_232828		0.24	1.743	0.01269
A_44_P516622	Taf1_predicted	XM_228551	317256	0.24	1.743	0.01783
A_44_P415854	LOC681599	XM_001057569		0.24	1.743	0.027394
A_43_P14985	Rpl19	NM_031103	81767	0.24	1.743	0.002064
A_44_P235985	Fasn	NM_017332	50671	0.24	1.743	0.001595
A_44_P487562	Net1	NM_001039023	307098	0.24	1.743	0.004096
A_44_P649046	AW918392	AW918392	499430	0.24	1.743	0.001216
A_44_P387246	Sin3a_predicted	XM_343395		0.24	1.743	0.002179
A_44_P393978	Nfe2l3_predicted	XM_231763	312331	0.24	1.743	0.042367
A_44_P409770	AF217591	AF217591		0.24	1.743	0.216687
A_44_P119465	XM_229173	XM_229173		0.24	1.742	0.122099
A_44_P432826	Rap1gds1_predicted	XM_227749		0.24	1.742	0.013467
A_44_P404982	ENSRNOT00000046977	ENSRNOT00000046977		0.24	1.742	0.028191
A_44_P436218	LOC682957	XM_001063848		0.24	1.742	0.017537
A_43_P21275	Plxnb1_predicted	XM_236640		0.24	1.742	0.013703
A_42_P596024	Spnb3	NM_019167	29211	0.24	1.742	0.055537
A_42_P619248	RGD1560511_predicted	XM_001053199		0.24	1.742	0.004569
A_44_P540279	AW144704	AW144704	64550	0.24	1.742	0.005958
A_44_P400355	Ccnh	NM_052981	84389	0.24	1.742	0.038885
A_44_P156785	RGD1311847	NM_001013879	290615	0.24	1.741	0.018272
A_44_P119486	RGD1304935_predicted	XM_233830	313866	0.24	1.741	0.037891
A_44_P154651	XM_213823	XM_213823		0.24	1.741	0.000663
A_44_P320356	AW921307	AW921307	361403	0.24	1.741	0.002738
A_44_P232230	RGD1309906	NM_001009246	287406	0.24	1.741	0.002165
A_44_P475866	RGD1306817_predicted	XM_225691	291406	0.24	1.741	0.004542
A_44_P396579	AW142584	AW142584	686120	0.24	1.740	0.002208
A_44_P174781	Kbtbd7	NM_001012045	306073	0.24	1.740	0.003735
A_44_P299999	Ltbr	NM_001008315	297604	0.24	1.740	0.002009
A_44_P1060513	Thex1	NM_001014143	361159	0.24	1.740	0.003321
A_43_P21070	Il17r_predicted	XM_232247		0.24	1.740	0.006379
A_44_P978472	LOC619440	NM_001034109	619440	0.24	1.740	0.001588
A_44_P382449	LOC686943	XR_009082	680142	0.24	1.740	0.003856
A_44_P239122	Ppm1g	NM_147209	259229	0.24	1.740	0.001653
A_44_P550945	Suhw3_predicted	XM_001069185		0.24	1.739	0.00604
A_44_P286911	Mapk14	NM_031020	81649	0.24	1.739	0.027738
A_43_P19065	Pcnxl3	XM_219515	309167	0.24	1.739	0.004545
A_44_P110450	RGD1311861_predicted	XM_001073139		0.24	1.739	0.015212
A_44_P396419	Cwf19l2_predicted	XM_343131	362804	0.24	1.739	0.005686
A_44_P497692	BF523562	BF523562	688018	0.24	1.739	0.001859
A_44_P119577	RGD1565403_predicted	XM_576453	501041	0.24	1.739	0.002414
A_44_P158079	BI395434	BI395434		0.24	1.739	0.015016
A_44_P804315	BI395655	BI395655		0.24	1.738	0.003542
A_44_P445521	Rnasen	XM_226843		0.24	1.738	0.004005
A_43_P18626	Atm_mapped	XM_236275		0.24	1.738	0.001931
A_42_P752901	RGD1562409_predicted	XM_575149		0.24	1.738	0.000783
A_44_P884037	TC518992	TC518992		0.24	1.738	0.005589
A_44_P575080	RGD1561832_predicted	XM_001080370		0.24	1.738	0.053822
A_44_P554299	BE110908	BE110908	497900	0.24	1.737	0.005826

A_43_P16311	LOC313641	XM_233606		0.24	1.737	0.005556
A_44_P711158	Cul4b_predicted	XM_001058651		0.24	1.737	0.009861
A_42_P647500	Kif3a	XM_001073152		0.24	1.737	0.013741
A_44_P278975	Slc39a8	NM_001011952	295455	0.24	1.737	0.001502
A_44_P421757	Fbxo46	NM_001025642	292686	0.24	1.737	0.008808
A_44_P1034823	Snx4_predicted	XM_340997	360725	0.24	1.736	0.00138
A_44_P621820	LOC501532	XR_007736	501532	0.24	1.735	0.002691
A_43_P15415	P34	NM_134398	171435	0.24	1.735	0.006246
A_44_P496013	BF556147	BF556147		0.24	1.735	0.010122
A_44_P520859	Pkmyt1_predicted	XM_213222		0.24	1.735	0.009206
A_44_P468312	Taf2	XM_343241	170844	0.24	1.735	0.008731
A_44_P450107	RGD1560076_predicted	XM_226796		0.24	1.735	0.004499
A_44_P839289	BE119030	BE119030		0.24	1.735	0.009975
A_43_P21000	Lrch3_predicted	XM_221380		0.24	1.735	0.002357
A_44_P178561	BF555800	BF555800		0.24	1.735	0.00753
A_44_P102477	RGD1307739	XM_342424		0.24	1.734	0.005823
A_44_P454076	LOC683968	XM_001068260		0.24	1.734	0.011943
A_44_P492635	Psmd3	NM_001008281	287670	0.24	1.734	0.003
A_44_P168184	Ran	AF507943	751812	0.24	1.734	0.002865
A_44_P351898	Mad2l1_predicted	XM_216161		0.24	1.734	0.017407
A_44_P541582	RGD1561243_predicted	XM_001064241		0.24	1.734	0.002111
A_44_P391688	RGD1566073_predicted	XM_344752		0.24	1.734	0.000799
A_44_P408165	LOC303332	NM_001024992	303332	0.24	1.734	0.004285
A_44_P445444	MGC95092	NM_001009706	361663	0.24	1.733	0.017228
A_44_P477427	AI104456	AI104456	309368	0.24	1.733	0.078018
A_44_P462132	Map2k4	NM_001030023	287398	0.24	1.733	0.016973
A_44_P543619	RGD1560183_predicted	XM_224552	290501	0.24	1.733	0.017946
A_44_P837669	Abce1	XM_341669		0.24	1.733	0.00954
A_44_P500598	Eif2s3x	XM_216704	299027	0.24	1.733	0.008703
A_43_P14866	RGD1564993_predicted	XM_216025		0.24	1.732	0.000678
A_44_P450361	A_44_P450361	A_44_P450361		0.24	1.732	0.006544
A_44_P192117	XM_215481	XM_215481		0.24	1.732	0.000986
A_44_P178775	AA956095	AA956095		0.24	1.732	0.080434
A_44_P431154	RGD1564862_predicted	XM_341801		0.24	1.731	0.002237
A_43_P17943	Usp31_predicted	XM_219292		0.24	1.731	0.002573
A_43_P16986	Xpo6	XM_574559	293476	0.24	1.731	0.001318
A_44_P837519	TC524251	TC524251		0.24	1.731	0.113628
A_44_P667238	Smndc1	NM_001025400	287768	0.24	1.731	0.004781
A_44_P558231	Slc7a4_predicted	XM_221263		0.24	1.731	0.112261
A_44_P547699	AA900593	AA900593		0.24	1.730	0.001164
A_44_P204569	AW914905	AW914905	116477	0.24	1.730	0.003456
A_44_P860405	A_44_P860405	A_44_P860405		0.24	1.730	0.005957
A_42_P517236	LOC304037	XM_221604		0.24	1.730	0.003469
A_43_P14745	RGD1359616	NM_001007663	300782	0.24	1.730	0.010666
A_44_P459422	Cttnbp2nl_predicted	XM_227556		0.24	1.730	0.000516
A_44_P290992	LOC293903	XM_001057123		0.24	1.730	0.024112
A_43_P12868	Smpd3	NM_053605	94338	0.24	1.730	0.011186
A_44_P1019326	Eif4a1	NM_199372	287436	0.24	1.730	0.002522
A_42_P830641	RGD1306209	XM_231752	312303	0.24	1.729	0.002576
A_43_P22707	A_43_P22707	A_43_P22707		0.24	1.729	0.001094
A_44_P348825	Arf2	NM_024150	79119	0.24	1.729	0.015805
A_43_P18492	Nfkb2	NM_001008349	309452	0.24	1.729	0.000305
A_44_P852537	RGD1566396_predicted	XM_001067433		0.24	1.729	0.001588
A_44_P524326	Racgap1_predicted	XM_235650		0.24	1.729	0.002662
A_44_P281086	Siva_predicted	XM_343117		0.24	1.729	0.000636
A_44_P1008026	AI230394	AI230394		0.24	1.729	0.000912
A_44_P1020324	Amd1	NM_031011	81640	0.24	1.728	0.001853
A_44_P777164	TC523760	TC523760		0.24	1.728	0.199621
A_44_P732935	TC558960	TC558960		0.24	1.728	0.047839
A_44_P372839	Cenpe_predicted	XM_342345	362044	0.24	1.728	0.006717
A_44_P448645	Brp16	NM_001007707	315094	0.24	1.728	0.000889
A_43_P22795	Taf1a	NM_001037204	360893	0.24	1.728	0.042867
A_44_P499528	Tmem7_predicted	XM_236656		0.24	1.728	0.029986

A_44_P161400	Kctd6_predicted	XM_223921		0.24	1.728	0.027146
A_44_P100289	ENSRNOT00000049514	ENSRNOT00000049514		0.24	1.728	0.02003
A_44_P379947	Rsbn1_predicted	XM_227540	310749	0.24	1.728	0.051183
A_44_P789035	RGD1563612_predicted	XM_575380	500026	0.24	1.728	0.025165
A_44_P211028	ENSRNOT00000044933	ENSRNOT00000044933		0.24	1.727	0.00269
A_44_P1017820	Leo1	NM_001005548	300837	0.24	1.727	0.22415
A_44_P156718	LOC682368	XM_001061225		0.24	1.727	0.003909
A_44_P531685	Rpl35	NM_212511	296709	0.24	1.727	0.002956
A_42_P772697	RGD1561833_predicted	XM_342921		0.24	1.727	0.134642
A_44_P379680	ENSRNOT00000046279	ENSRNOT00000046279		0.24	1.727	0.0186
A_44_P1033023	Parp1	NM_013063	25591	0.24	1.727	0.000962
A_44_P503699	RGD1563497_predicted	XM_344891	365243	0.24	1.726	0.010632
A_44_P645135	RGD1565486_predicted	XM_573045	497865	0.24	1.726	0.009744
A_44_P354053	Gdap2	NM_001013201	362004	0.24	1.726	0.011997
A_44_P445306	Shc1	NM_053517	85385	0.24	1.726	0.002112
A_44_P944540	A_44_P944540	A_44_P944540		0.24	1.726	0.000992
A_44_P945759	Tsga14	NM_001025770	500069	0.24	1.726	0.003809
A_44_P131133	A_44_P131133	A_44_P131133		0.24	1.726	0.022646
A_44_P122812	Gkap1	NM_001012160	361202	0.24	1.726	0.028661
A_44_P838583	LOC684669	XM_001071521		0.24	1.725	0.032915
A_43_P20507	Armc6_predicted	XM_224735		0.24	1.725	0.004932
A_44_P255771	LOC688393	XM_001066740	688393	0.24	1.725	0.01079
A_44_P557012	BE119977	BE119977	360993	0.24	1.725	0.033739
A_44_P1019604	Ube2c_predicted	XM_215924		0.24	1.725	0.009714
A_44_P231409	RGD1308955	XM_215122		0.24	1.725	0.003491
A_44_P161674	RGD1561150_predicted	XM_233177		0.24	1.725	0.002073
A_44_P532352	Irak1_predicted	XM_343844	363520	0.24	1.725	0.003751
A_42_P453959	RGD1564403_predicted	XM_231157		0.24	1.725	0.051298
A_44_P138105	LOC679161	XM_001054250		0.24	1.724	0.011537
A_44_P233742	Tspan5	NM_001004090	362048	0.24	1.724	0.058983
A_44_P246311	MGC112682	NM_001017473	497900	0.24	1.724	0.000889
A_44_P1010631	RGD1308665	NM_001034936	360505	0.24	1.724	0.00181
A_43_P10569	Eif3s10	XM_238649		0.24	1.724	0.001302
A_44_P987040	Insig2	NM_178091	288985	0.24	1.724	0.220423
A_44_P853300	CK228733	CK228733		0.24	1.724	0.080244
A_44_P308673	Commd5	NM_139108	245974	0.24	1.724	0.012635
A_44_P206258	Usp10	NM_001034146	307905	0.24	1.723	0.009692
A_44_P405071	Cdk6	XM_342638	114483	0.24	1.723	0.005995
A_44_P408313	AW144303	AW144303		0.24	1.723	0.013574
A_44_P419239	Pdap1	NM_022595	64527	0.24	1.723	0.001426
A_43_P12617	Grb14	NM_031623	58844	0.24	1.723	0.001831
A_42_P803590	LOC289378	XM_001066750		0.24	1.723	0.007487
A_44_P281468	RGD1563815_predicted	XM_345092	365549	0.24	1.722	0.118451
A_44_P394228	RGD1304601	NM_001024990	302972	0.24	1.722	0.004375
A_43_P16724	RGD1307688	NM_001014188	362394	0.24	1.722	0.001021
A_44_P1007247	Elmo2	XM_342579	362271	0.24	1.722	0.015638
A_44_P208827	Pigb_predicted	XM_236397		0.24	1.722	0.075697
A_44_P475051	Tpm1	NM_019131	24851	0.24	1.722	0.003874
A_44_P104630	Lat2	NM_173840	317676	0.24	1.722	0.003076
A_42_P743495	Rwdd1	NM_147146	259218	0.24	1.721	0.018357
A_43_P19423	LOC360619	XM_340894		0.24	1.721	0.242898
A_44_P480780	Ephb2_predicted	XM_233574	313633	0.24	1.721	0.006171
A_44_P516003	Smap1l	XM_216529	298500	0.24	1.721	0.000952
A_44_P805961	ENSRNOT00000051622	ENSRNOT00000051622		0.24	1.721	0.027022
A_44_P262625	XM_344424	XM_344424		0.24	1.721	0.00047
A_44_P253012	Spry2	NM_001012046	306141	0.24	1.721	0.002252
A_44_P851664	AA801123	AA801123		0.24	1.720	0.000379
A_42_P726743	Adck1_predicted	XM_001062410		0.24	1.720	0.016485
A_42_P700674	Actg1	XM_001076100	287876	0.24	1.720	0.015495
A_42_P688442	Usf1	NM_031777	83586	0.24	1.720	0.001979
A_44_P108588	Fst	NM_012561	24373	0.24	1.720	0.018357
A_44_P668678	TC539221	TC539221		0.24	1.720	0.025203
A_44_P748419	TC527868	TC527868		0.24	1.720	0.024997

A_43_P16458	Atp6v1c1	NM_001011992	299971	0.24	1.720	0.005168
A_44_P168619	Zfp289	NM_001033707	362162	0.24	1.720	0.005909
A_44_P474234	BF558116	BF558116		0.24	1.720	0.001722
A_44_P260502	LOC296875	XR_008542	296875	0.24	1.719	0.016419
A_44_P768338	TC562129	TC562129		0.24	1.719	0.001718
A_44_P215539	Spire1_predicted	XM_225864		0.24	1.719	0.004297
A_44_P431452	A_44_P431452	A_44_P431452		0.24	1.719	0.000719
A_44_P958487	AI136348	AI136348		0.24	1.718	0.014282
A_44_P560871	TC553785	TC553785		0.24	1.718	0.003739
A_44_P389462	Zmym3	XM_001061563		0.24	1.718	0.00473
A_44_P607687	TC558368	TC558368		0.24	1.718	0.078114
A_44_P466305	A_44_P466305	A_44_P466305		0.24	1.718	0.07276
A_44_P170524	Tpcn1	NM_139332	246215	0.23	1.718	0.004014
A_44_P527432	Nsd1_predicted	XM_001066409		0.23	1.718	0.001565
A_44_P489729	RGD1561780_predicted	XM_223019		0.23	1.718	0.001902
A_42_P785926	Mrps23_predicted	XM_340874		0.23	1.717	0.007943
A_43_P13030	Serpinb5	NM_057108	116589	0.23	1.717	0.001006
A_44_P403401	LOC684195	XM_001069352		0.23	1.717	0.00286
A_44_P1060194	Fbl	NM_001025643	292747	0.23	1.717	0.010836
A_42_P576454	Nrm	NM_212508	361791	0.23	1.717	0.005816
A_44_P504326	AW918202	AW918202		0.23	1.717	0.022775
A_44_P234129	Dlg7_predicted	XM_223937	289997	0.23	1.716	0.001951
A_44_P220112	XM_219547	XM_219547		0.23	1.716	0.001125
A_44_P366776	Ddx17	XM_235480		0.23	1.716	0.030942
A_44_P318891	XM_227366	XM_227366		0.23	1.716	0.014064
A_44_P642884	TC568517	TC568517		0.23	1.716	0.054454
A_42_P609522	Oprs1	NM_030996	29336	0.23	1.716	0.002591
A_43_P23097	Stom	NM_001011965	296655	0.23	1.716	0.032769
A_42_P577677	Tnni1	NM_017184	29388	0.23	1.716	0.083664
A_44_P470845	Prpf31_predicted	XM_218173		0.23	1.716	0.001656
A_44_P621230	A_44_P621230	A_44_P621230		0.23	1.715	0.00145
A_44_P477268	Rnf187_predicted	XM_340805	360533	0.23	1.715	0.005484
A_44_P333474	LOC360627	NM_001014120	360627	0.23	1.715	0.038101
A_44_P852000	RGD1565144_predicted	XM_573295		0.23	1.715	0.053168
A_44_P191467	Atp6v0e1	NM_053578	94170	0.23	1.715	0.01651
A_44_P1047331	RGD1308877_predicted	XM_230548		0.23	1.715	0.00213
A_44_P522994	Malt1_predicted	XM_225927	307366	0.23	1.714	0.00782
A_43_P20133	Skil	XM_001057072	114208	0.23	1.714	0.008746
A_43_P13327	Ubb	NM_138895	192255	0.23	1.714	0.00146
A_44_P1048613	RGD1308261_predicted	XM_001081031		0.23	1.714	0.000381
A_43_P20044	XM_343104	XM_343104		0.23	1.714	0.001987
A_44_P885046	TC524385	TC524385		0.23	1.714	0.059323
A_43_P12016	Inpp5d	NM_019311	54259	0.23	1.714	0.008359
A_44_P496483	MGC108785	NM_001012351	304277	0.23	1.713	0.000665
A_44_P440971	Lamc3_predicted	XM_231139		0.23	1.713	0.004594
A_44_P259138	BI273834	BI273834		0.23	1.713	0.097578
A_42_P666951	Tnfrsf5	NM_134360	171369	0.23	1.713	0.008298
A_44_P208415	RGD1561589_predicted	XM_223330	305284	0.23	1.713	0.001469
A_42_P841620	LOC683463	XM_001066042		0.23	1.713	0.048366
A_44_P117072	Mrpl50_predicted	XM_342835		0.23	1.713	0.006874
A_44_P315022	Rad23b	NM_001025275	298012	0.23	1.712	0.002043
A_44_P100959	LOC312030	XM_231361	312030	0.23	1.712	0.011552
A_44_P838355	TC539813	TC539813		0.23	1.712	0.006395
A_44_P171203	Nubpl_predicted	XM_216689	299008	0.23	1.712	0.00331
A_44_P1036329	BF547960	BF547960		0.23	1.712	0.029494
A_44_P558390	RGD1311021_predicted	XM_218824		0.23	1.712	0.004305
A_42_P559414	Wnt4	NM_053402	84426	0.23	1.712	0.018097
A_44_P394279	RGD1560070_predicted	XM_001081777		0.23	1.712	0.099909
A_44_P304009	RGD1560913_predicted	XM_001076464		0.23	1.712	0.049441
A_44_P667917	RGD1565399_predicted	XR_007482	367250	0.23	1.711	0.00072
A_44_P144943	RGD1306947	XM_213850		0.23	1.711	0.003187
A_44_P185102	MGC124992	XM_001077253	499697	0.23	1.711	0.010747
A_44_P342202	Cask	NM_022184	29647	0.23	1.711	0.010508

A_44_P117078	LOC685162	XM_001062622		0.23	1.711	0.000814
A_42_P753262	Zfp444_predicted	XM_001057809		0.23	1.711	0.001094
A_43_P20452	Tjap1_predicted	XM_236932		0.23	1.711	0.002731
A_44_P758203	RGD1305356	NM_001008373	361888	0.23	1.711	0.003054
A_44_P250654	Habp4_predicted	XM_341479		0.23	1.711	0.001388
A_44_P665161	AW143873	AW143873		0.23	1.711	0.032576
A_44_P833647	BC091319	BC091319		0.23	1.711	0.012788
A_44_P414509	U08214	U08214		0.23	1.711	0.004805
A_44_P484950	BC099786	BC099786	114630	0.23	1.711	0.012479
A_44_P349525	LOC680996	XM_001059842		0.23	1.710	0.002008
A_44_P1029654	RGD1560300_predicted	XM_001062922		0.23	1.710	0.003293
A_44_P402995	A_44_P402995	A_44_P402995		0.23	1.710	0.000601
A_44_P329170	Ubap2_predicted	XM_232901		0.23	1.710	0.001479
A_44_P197258	AI101900	AI101900	363213	0.23	1.709	0.007388
A_44_P715081	TC522327	TC522327		0.23	1.709	0.003636
A_44_P450416	Angel1_predicted	XM_343091		0.23	1.709	0.002263
A_44_P895402	AW916198	AW916198		0.23	1.709	0.003755
A_44_P174949	AW917562	AW917562		0.23	1.708	0.004334
A_44_P483013	XM_213066	XM_213066		0.23	1.708	0.001422
A_44_P760064	ENSRNOT00000028053	ENSRNOT00000028053		0.23	1.708	0.035931
A_44_P266519	LOC361128	BC098704	361128	0.23	1.708	0.001008
A_44_P701318	TC525304	TC525304		0.23	1.708	0.01096
A_44_P201389	Psmc6	XM_214147	289990	0.23	1.707	0.00127
A_44_P506741	Azi1_predicted	XM_340945		0.23	1.707	0.002637
A_44_P316348	RGD1309586_predicted	XM_344187		0.23	1.707	0.140775
A_44_P1045425	Ptk2	NM_013081	25614	0.23	1.707	0.018325
A_44_P295327	RGD1305754_predicted	XM_230830		0.23	1.707	0.0268
A_44_P253940	Hrpap20	NM_198783	362495	0.23	1.707	0.014005
A_44_P177058	Id2	NM_013060	25587	0.23	1.707	0.027366
A_42_P802538	RGD1306917_predicted	XM_342099		0.23	1.707	0.000311
A_44_P316303	Leng8	XM_001058218		0.23	1.706	0.025485
A_44_P510714	Arid1b	NM_172157	282546	0.23	1.706	0.022337
A_43_P11905	Kcnab1	NM_017303	29737	0.23	1.706	0.327108
A_44_P326451	RGD1565890_predicted	XM_343855	363536	0.23	1.705	0.051091
A_44_P484948	ENSRNOT00000002167	ENSRNOT00000002167		0.23	1.705	0.010597
A_44_P418315	AW920993	AW920993		0.23	1.705	0.054407
A_44_P666838	AW920987	AW920987		0.23	1.705	0.028719
A_44_P1045354	Slc35b1	NM_199081	287642	0.23	1.705	0.001276
A_43_P12822	Kif3c	NM_053486	85248	0.23	1.705	0.023944
A_44_P638290	LOC500110	NM_001024327	500110	0.23	1.705	0.009458
A_44_P218229	XM_347113	XM_347113		0.23	1.704	0.001261
A_44_P1055052	Nol1_predicted	XM_235295	314969	0.23	1.704	0.000414
A_44_P738831	BE105152	BE105152		0.23	1.704	0.118141
A_44_P118833	D10770	D10770		0.23	1.704	0.011435
A_44_P1010528	RGD1561600_predicted	XM_214476	291105	0.23	1.704	0.003458
A_44_P991114	Aprin_predicted	XM_221833		0.23	1.704	0.001397
A_44_P505977	AF050660	AF050660		0.23	1.704	0.031368
A_44_P791798	AW141463	AW141463		0.23	1.704	0.002223
A_44_P339898	RGD1310857	NM_001039022	304542	0.23	1.704	0.010177
A_44_P364672	Ppgb	NM_001011959	296370	0.23	1.703	0.029753
A_44_P1057272	Ehmt2	NM_212463	361798	0.23	1.703	0.003944
A_44_P853102	TC554571	TC554571		0.23	1.703	0.006844
A_44_P100557	Zfp219	NM_001007681	305848	0.23	1.703	0.004162
A_44_P538551	Ceacam1	NM_031755	81613	0.23	1.703	0.157976
A_42_P727530	Mnat1	NM_153472	266713	0.23	1.703	0.003873
A_44_P303533	AW144359	AW144359		0.23	1.703	0.021753
A_44_P156705	LOC689075	XM_001069421		0.23	1.703	0.00181
A_44_P473935	H33234	H33234	170587	0.23	1.703	0.032079
A_44_P383889	Ubb	NM_138895	192255	0.23	1.703	0.036465
A_42_P517554	Polg2_predicted	XM_221047		0.23	1.703	0.031636
A_44_P996233	Prim1	NM_001008768	246327	0.23	1.703	0.001543
A_44_P391726	Rpia_predicted	XM_342707		0.23	1.702	0.004305
A_44_P487841	Srprb	NM_001013252	300965	0.23	1.702	0.007551

A_43_P13244	Rap1b	NM_134346	171337	0.23	1.702	0.004035
A_44_P161208	LOC311710	XM_230955	114588	0.23	1.702	0.015638
A_43_P10309	Fxr1h	NM_001012179	361927	0.23	1.702	0.002602
A_43_P15245	Npr1	NM_012613	24603	0.23	1.702	0.007533
A_44_P233231	AY148303	AY148303		0.23	1.702	0.004906
A_44_P134634	RGD1562272_predicted	XM_220843	303433	0.23	1.701	0.003049
A_42_P655425	Ppwd1_predicted	XM_215474		0.23	1.701	0.010672
A_44_P929706	Fbxl11_predicted	XM_341983		0.23	1.701	0.002243
A_44_P443228	Fech_predicted	XM_341622		0.23	1.701	0.040557
A_44_P100609	Frg1_predicted	XM_001064153		0.23	1.701	0.005163
A_44_P100873	Pigh_predicted	XM_343083		0.23	1.701	0.1652
A_44_P590197	AW915353	AW915353	361999	0.23	1.701	0.002554
A_44_P791908	RGD1560859_predicted	XR_008965	500243	0.23	1.701	0.019713
A_44_P313245	BQ200469	BQ200469		0.23	1.701	0.002757
A_44_P354480	Usp36_predicted	XM_221143		0.23	1.700	0.004312
A_44_P483032	Ftsj1_predicted	XM_343771	363450	0.23	1.700	0.00342
A_44_P410670	Stk11_predicted	XM_234900		0.23	1.700	0.001897
A_44_P975403	LOC684112	XM_001068984		0.23	1.700	0.096323
A_44_P492808	RGD1562046_predicted	XM_001070395		0.23	1.700	0.001391
A_44_P844244	TC525735	TC525735		0.23	1.699	0.208471
A_44_P851281	CB614165	CB614165		0.23	1.699	0.030974
A_42_P782490	RGD1560268_predicted	XM_226464	307829	0.23	1.699	0.003082
A_44_P147529	RGD1564921_predicted	XM_345576	366461	0.23	1.699	0.015368
A_44_P561563	TC540547	TC540547		0.23	1.699	0.008923
A_44_P1046566	RGD1564623_predicted	XM_573140		0.23	1.699	0.000811
A_44_P880417	BF551339	BF551339		0.23	1.699	0.001282
A_43_P16492	LOC679532	XM_001053380		0.23	1.699	0.006449
A_44_P417245	A_44_P417245	A_44_P417245		0.23	1.698	0.089945
A_44_P433905	Cldn2_predicted	XM_236535		0.23	1.698	0.009346
A_44_P520943	Exosc5_predicted	XM_218343		0.23	1.698	0.000408
A_44_P456072	NIPBL	XM_238213		0.23	1.698	0.107585
A_44_P135455	RGD1307760	NM_001039611	361645	0.23	1.698	0.042213
A_44_P277264	Skil	XM_001057072	114208	0.23	1.697	0.008872
A_44_P560788	CO566111	CO566111		0.23	1.697	0.001002
A_44_P666786	Akt1s1_predicted	XM_238103		0.23	1.697	0.001165
A_43_P17065	Helz_predicted	XM_237808		0.23	1.697	0.00616
A_44_P422335	RGD1560788_predicted	XM_343952	363662	0.23	1.696	0.02923
A_44_P208949	Srp68_predicted	XM_343986		0.23	1.696	0.004741
A_44_P191962	Echdc1	NM_001007734	361465	0.23	1.696	0.028296
A_44_P403088	Dennd2c_predicted	XM_001067655		0.23	1.696	0.197144
A_44_P179325	RGD1564084_predicted	NM_001025287	363076	0.23	1.696	0.011479
A_44_P806713	A_44_P806713	A_44_P806713		0.23	1.696	0.091813
A_44_P356808	Runx2	XM_346016	367218	0.23	1.696	0.012496
A_44_P433894	RGD1560821_predicted	XM_346332	367865	0.23	1.695	0.000607
A_44_P698367	A_44_P698367	A_44_P698367		0.23	1.695	0.005055
A_44_P510188	AW143279	AW143279		0.23	1.695	0.011037
A_43_P10481	TC542779	TC542779		0.23	1.695	0.001878
A_44_P261818	BC087666	BC087666		0.23	1.695	0.08837
A_44_P285694	Bfsp1	XM_342529		0.23	1.695	0.101539
A_42_P841993	Hirip3	NM_001025725	361650	0.23	1.694	0.007375
A_44_P838483	TC522858	TC522858		0.23	1.694	0.008053
A_44_P453834	AI548404	AI548404	680097	0.23	1.694	0.077653
A_44_P414903	Pcgf4_predicted	XM_225606		0.23	1.694	0.025056
A_44_P450445	RGD1307947	NM_001014083	314788	0.23	1.694	0.0185
A_44_P870200	TC544322	TC544322		0.23	1.694	0.013645
A_44_P791184	XM_576381	XM_576381		0.23	1.694	0.001691
A_44_P536786	Sec23b_predicted	XM_342531		0.23	1.693	0.005705
A_43_P21451	LOC362015	XM_342313		0.23	1.693	0.004217
A_44_P828941	TC543761	TC543761		0.23	1.693	0.177309
A_44_P239306	LOC287938	NM_001013863	287938	0.23	1.693	0.000993
A_44_P897023	Snx24	NM_001008364	361328	0.23	1.693	0.010489
A_44_P367653	Mrpl16	NM_001009647	293754	0.23	1.692	0.001014
A_44_P513148	Uhmk1	NM_017293	246332	0.23	1.692	0.002672

A_44_P946443	TC559951	TC559951		0.23	1.692	0.1312
A_44_P930673	Wdr48_predicted	XM_343503	363164	0.23	1.692	0.018161
A_44_P332734	Psma3l	BN000326		0.23	1.692	0.000834
A_44_P759588	CF110322	CF110322		0.23	1.691	0.004741
A_42_P746214	Spr	XM_342714		0.23	1.691	0.003311
A_44_P512828	Pom210	NM_053322	58958	0.23	1.691	0.019539
A_44_P513413	XM_227251	XM_227251		0.23	1.691	0.048202
A_42_P820650	AW918031	AW918031		0.23	1.691	0.047472
A_44_P491621	Adrbk1	NM_012776	25238	0.23	1.691	0.001531
A_44_P152329	Chordc1_predicted	XM_235878		0.23	1.691	0.013021
A_43_P16050	Wnt11	XM_238122	140584	0.23	1.691	0.039053
A_44_P516306	RGD1308535_predicted	XM_001073257		0.23	1.690	0.000635
A_44_P517317	LOC683077	XM_001058171		0.23	1.690	0.027428
A_44_P762275	LOC687516	XM_001078904		0.23	1.690	0.005645
A_44_P494445	RGD1311455_predicted	XM_341304		0.23	1.690	0.023482
A_44_P334352	Abcf1	XM_001056151		0.23	1.690	0.002029
A_44_P405022	Kb1	NM_001008802	300250	0.23	1.690	0.00257
A_44_P970247	Nicn1	NM_001034999	619581	0.23	1.690	0.089543
A_44_P1005213	Cdca1	NM_001012028	304951	0.23	1.689	0.02401
A_44_P110196	A_44_P110196	A_44_P110196		0.23	1.689	0.002373
A_44_P281387	Znf397_predicted	XM_344656	364827	0.23	1.689	0.042134
A_44_P877059	RGD1305754_predicted	XM_230830		0.23	1.689	0.005044
A_44_P1031390	Trappc4	NM_001003708	367073	0.23	1.689	0.002625
A_43_P20684	LOC366431	NM_001014260	366431	0.23	1.689	0.022427
A_44_P1019826	LOC689116	XM_001064621		0.23	1.689	0.004933
A_44_P1055584	Dhx40	NM_001005873	287595	0.23	1.689	0.030655
A_44_P1006717	RGD1311580_predicted	XM_238021		0.23	1.689	0.006879
A_44_P210461	Ldha	NM_017025	24533	0.23	1.688	0.034735
A_44_P133697	RGD1562236_predicted	XM_001065586		0.23	1.688	0.023477
A_44_P500694	Cldnd1	NM_001006955	288182	0.23	1.688	0.009472
A_44_P398522	Aqr_predicted	XM_345418		0.23	1.688	0.002547
A_44_P219807	Mapk7	XM_340813		0.23	1.688	0.000527
A_44_P419528	RGD1305302	NM_001014000	306455	0.23	1.687	0.009477
A_44_P356070	Muc4	XM_221384	303887	0.23	1.687	0.026376
A_44_P492914	BF550034	BF550034	288779	0.23	1.687	0.003661
A_44_P112641	RGD1304570_predicted	XM_221214		0.23	1.687	0.003522
A_44_P503999	Asf1a_predicted	XM_215389	294408	0.23	1.687	0.045523
A_44_P180717	Pstpip1_predicted	XM_217152		0.23	1.687	0.009716
A_44_P821587	Dlc1	XM_341444	58834	0.23	1.687	0.078631
A_44_P253122	RGD1306717_predicted	XM_001072521		0.23	1.686	0.002389
A_44_P405821	Pde6d_predicted	XM_343613		0.23	1.686	0.00245
A_44_P168631	LOC502176	NM_001025057	502176	0.23	1.686	0.001488
A_42_P830713	RGD1564964_predicted	XM_236438	315843	0.23	1.686	0.001592
A_44_P288300	XM_219699	XM_219699		0.23	1.686	0.001142
A_44_P151933	RGD1562784_predicted	XM_219560	309197	0.23	1.685	0.010464
A_44_P477322	Rtn4	NM_031831	83765	0.23	1.685	0.003047
A_42_P621736	Rplp1	NM_001007604	140661	0.23	1.685	0.002662
A_44_P532027	XM_238155	XM_238155		0.23	1.685	0.002702
A_44_P1017481	Timp2	NM_021989	29543	0.23	1.685	0.018965
A_44_P521699	Mpzl1	NM_001007728	360871	0.23	1.685	0.018008
A_44_P884959	TC522800	TC522800		0.23	1.685	0.11805
A_44_P165855	Tpt1	NM_053867	116646	0.23	1.685	0.016301
A_44_P311998	Ppan	NM_001011980	298699	0.23	1.684	0.003983
A_44_P847098	BC083922	BC083922	680266	0.23	1.684	0.006901
A_44_P254144	RGD1311920_predicted	XM_223651	305544	0.23	1.684	0.029809
A_44_P501268	Npuk68	NM_138920	192359	0.23	1.684	0.009836
A_44_P154023	RT1-M2	NM_001001717		0.23	1.684	0.207914
A_44_P363494	RGD1563847_predicted	XR_007323	310383	0.23	1.684	0.036643
A_44_P347662	Prkab2	NM_022627	64562	0.23	1.683	0.003264
A_44_P438750	Rdh12_predicted	XM_234334		0.23	1.683	0.029267
A_44_P463878	Slc38a2	NM_181090	29642	0.23	1.683	0.030094
A_43_P17772	Commd7	NM_001030029	296285	0.23	1.683	0.004542
A_44_P335400	Arl5b	NM_001015031	364788	0.23	1.683	0.019153

A_44_P311856	Smarcad1_predicted	XM_231860		0.23	1.683	0.023274
A_44_P1060108	Tmsb10	NM_021261	50665	0.23	1.683	0.004482
A_44_P424514	Yipf2	NM_001014208	363027	0.23	1.683	0.027809
A_44_P356166	Pigm	NM_024144	79112	0.23	1.683	0.045348
A_44_P344577	Nvl_predicted	XM_213963		0.23	1.683	0.084815
A_44_P368082	XM_342365	XM_342365		0.23	1.682	0.056674
A_44_P914723	RGD1561062_predicted	XM_573179		0.23	1.682	0.039085
A_44_P115896	XM_220645	XM_220645		0.23	1.682	0.002139
A_44_P138081	RGD1560170_predicted	XM_228713	317343	0.23	1.682	0.003751
A_44_P121832	XM_216546	XM_216546		0.23	1.682	0.00483
A_42_P481176	Scamp4	NM_031725	65170	0.23	1.682	0.000805
A_42_P797638	Wtip_predicted	XM_341839	316101	0.23	1.682	0.002083
A_44_P162892	U68562	U68562		0.23	1.681	0.00593
A_44_P1026398	Sub1	NM_001009618	192269	0.23	1.681	0.001718
A_44_P269522	RGD1309892	NM_001013871	289034	0.23	1.681	0.029178
A_44_P407691	RGD1305356	NM_001008373	361888	0.23	1.681	0.001605
A_44_P1048388	LOC307798	NM_001037193	307798	0.23	1.681	0.001732
A_44_P625026	RGD1311490_predicted	XM_001071145		0.23	1.681	0.002561
A_42_P506267	Zmpste24_predicted	XM_233483		0.23	1.681	0.044565
A_44_P492617	Itm2c	NM_001009674	301575	0.23	1.681	0.013622
A_44_P347464	RGD1559917_predicted	XM_212687		0.23	1.681	0.00082
A_44_P793731	TC542831	TC542831		0.23	1.681	0.003722
A_43_P12246	Prpsap1	NM_022545	64390	0.23	1.681	0.007173
A_44_P206284	RGD1566215_predicted	XM_217496		0.23	1.681	0.006802
A_44_P929683	Arpp19	CF110174	60336	0.23	1.680	0.006469
A_44_P559106	Cct8_predicted	XM_213673		0.23	1.680	0.006651
A_44_P430937	Ptpro	NM_017336	50677	0.23	1.680	0.004401
A_44_P536275	Adamts4	XM_001053685		0.23	1.680	0.178958
A_44_P538275	L26525	L26525		0.23	1.680	0.000892
A_44_P161342	RGD1309034	NM_001013987	305120	0.23	1.680	0.015044
A_44_P315977	Slc23a2	NM_017316	50622	0.23	1.680	0.022491
A_44_P457226	Sf3b3_predicted	XM_214697		0.23	1.680	0.004307
A_44_P349602	LOC686480	XM_001073751		0.23	1.680	0.010777
A_44_P161467	LOC361990	NM_001014175	361990	0.23	1.679	0.005909
A_44_P636897	ENSRNOT00000048493	ENSRNOT00000048493		0.23	1.679	0.220521
A_44_P481525	Eif5	NM_020075	56783	0.23	1.679	0.011156
A_44_P1050474	Rps5	XM_218293		0.23	1.679	0.015569
A_44_P477735	RGD1564943_predicted	XM_001060653		0.23	1.679	0.00832
A_44_P110542	Dbnl	NM_031352	83527	0.23	1.679	0.006537
A_44_P348983	Npepps	XM_340889		0.23	1.679	0.018837
A_44_P544351	Tcn2	NM_022534	64365	0.22	1.679	0.00423
A_44_P543350	Kpnb1	NM_017063	24917	0.22	1.679	0.004549
A_44_P146616	H2a	NM_021840	64646	0.22	1.679	0.032494
A_44_P555833	XM_343288	XM_343288		0.22	1.678	0.008938
A_44_P100318	Bckdk	NM_019244	29603	0.22	1.678	0.004213
A_44_P510548	Olr1286_predicted	NM_001000799	405084	0.22	1.678	0.033991
A_43_P10163	Wars	NM_001013170	314442	0.22	1.678	0.001136
A_44_P869506	TC535876	TC535876		0.22	1.677	0.000694
A_43_P16655	Ubqln2_predicted	XM_228806		0.22	1.677	0.023131
A_44_P300618	Usp24_predicted	XM_233260	313427	0.22	1.677	0.002857
A_44_P234613	Mrpl45_predicted	XM_213446		0.22	1.677	0.001234
A_44_P333293	RGD1564942_predicted	XM_216496	298409	0.22	1.677	0.060639
A_44_P517686	Tomm70a	NM_212519	304017	0.22	1.677	0.022841
A_44_P517896	Hsd17b12	NM_032066	84013	0.22	1.677	0.003869
A_44_P140051	Ap1s1_predicted	XM_341052		0.22	1.677	0.019817
A_43_P21117	Rbm19_predicted	XM_222200		0.22	1.677	0.004275
A_42_P646075	Ppid	NM_001004279	361967	0.22	1.676	0.003056
A_44_P297609	Znf532_predicted	XM_225923		0.22	1.676	0.004741
A_44_P509769	AA955473	AA955473	294337	0.22	1.676	0.033025
A_44_P956005	Slc25a30	AY724532	361074	0.22	1.676	0.004785
A_42_P585079	Ube2e3_predicted	XM_215754		0.22	1.676	0.014966
A_44_P367486	Lig1	NM_001024268	81513	0.22	1.676	0.008697
A_44_P556579	RGD1565596_predicted	XM_575600	500252	0.22	1.676	0.003137

A_42_P756652	RGD620382	NM_133525	171047	0.22	1.676	0.001747
A_44_P465608	AA957183	AA957183	83620	0.22	1.676	0.003402
A_44_P166777	LOC499235	XM_574528	499235	0.22	1.676	0.001724
A_44_P231918	RGD1563192_predicted	XM_225959	307446	0.22	1.675	0.080068
A_42_P514419	RGD1307679	NM_001009599	311166	0.22	1.675	0.010533
A_44_P445359	Pex7	NM_001034147	308718	0.22	1.675	0.006799
A_44_P991940	Socs6_predicted	XM_225667		0.22	1.675	0.13106
A_43_P21118	Klhl24	NM_181473	303803	0.22	1.675	0.016744
A_44_P760225	A_44_P760225	A_44_P760225		0.22	1.675	0.001101
A_44_P220104	Drap1_predicted	XM_215177		0.22	1.674	0.001261
A_44_P1024542	Stau1	NM_053436	84496	0.22	1.674	0.014074
A_44_P562972	TC542639	TC542639		0.22	1.674	0.072501
A_44_P896969	RGD1308696	NM_001008278	287278	0.22	1.674	0.005047
A_44_P407243	Zmynd19	NM_198770	311791	0.22	1.674	0.008678
A_43_P16378	Olr1283	NM_001000518	315572	0.22	1.674	0.077052
A_42_P737455	AI071866	AI071866		0.22	1.674	0.001271
A_44_P482294	Dck	NM_024158	79127	0.22	1.674	0.011687
A_42_P521609	Morc3_predicted	XM_221635		0.22	1.674	0.002865
A_44_P836866	A_44_P836866	A_44_P836866		0.22	1.674	0.004641
A_44_P177827	Gls	NM_012569	24398	0.22	1.674	0.012591
A_44_P930130	DV725357	DV725357	362455	0.22	1.674	0.00531
A_42_P772256	Gtf2ird1	NM_001001504	246770	0.22	1.674	0.002332
A_44_P248663	MGC124653	NM_001033903	501157	0.22	1.674	0.00314
A_42_P457895	Supv3l1	NM_001012462	294385	0.22	1.673	0.017259
A_44_P395764	Pcsk2	NM_012746	25121	0.22	1.673	0.021147
A_44_P267309	Cdk105	NM_134415	171456	0.22	1.673	0.001347
A_44_P250412	Tmbim1	NM_001007713	316516	0.22	1.673	0.040508
A_44_P542910	Golga7	NM_001007731	361171	0.22	1.673	0.009492
A_44_P498831	XM_216751	XM_216751		0.22	1.672	0.062443
A_44_P944758	Hnrpa1	NM_017248	29578	0.22	1.672	0.00457
A_44_P215056	Itpr1	NM_001007235	25262	0.22	1.672	0.003482
A_43_P18725	RGD1560168_predicted	XM_346069		0.22	1.672	0.009555
A_44_P163685	Xrcc4	NM_001006999	309995	0.22	1.672	0.031476
A_44_P342487	RGD1560813_predicted	XM_574181	498894	0.22	1.671	0.001657
A_44_P646692	LOC681254	XM_001060960	681254	0.22	1.671	0.025729
A_44_P990713	Rnu3ip2_predicted	XM_343469		0.22	1.671	0.003783
A_44_P267621	XM_233699	XM_233699		0.22	1.671	0.119103
A_44_P346822	RGD1310213_predicted	XM_222288	304592	0.22	1.671	0.000735
A_44_P528926	DV725021	DV725021		0.22	1.670	0.041055
A_44_P1011464	AA799294	AA799294	309722	0.22	1.670	0.027515
A_44_P454372	Chd4	XM_232354	117535	0.22	1.670	0.00747
A_44_P191878	Myo1c	NM_023092	65261	0.22	1.670	0.000777
A_44_P229299	LOC304361	XM_222048	304361	0.22	1.670	0.002686
A_44_P324072	LOC684063	XM_001068728		0.22	1.670	0.005578
A_44_P208411	RGD1561589_predicted	XM_223330	305284	0.22	1.670	0.001878
A_44_P517012	Rac2	NM_001008384	366957	0.22	1.669	0.055263
A_44_P226908	Ywhah	NM_013052	25576	0.22	1.669	0.003836
A_43_P10689	Dmn	XM_001055657	308709	0.22	1.669	0.033195
A_44_P722152	A_44_P722152	A_44_P722152		0.22	1.669	0.037411
A_43_P19444	Adam8_predicted	XM_219470		0.22	1.668	0.005578
A_44_P382363	RGD1308119	NM_001008333	302032	0.22	1.668	0.030899
A_42_P608325	Brd1_predicted	XM_235552		0.22	1.668	0.005422
A_44_P587550	AW915632	AW915632		0.22	1.668	0.015921
A_44_P468653	LOC310182	XR_006143	310182	0.22	1.668	0.006278
A_44_P142829	Mlh1	NM_031053	81685	0.22	1.668	0.01181
A_43_P16039	Runx2	XM_346016	367218	0.22	1.668	0.053143
A_44_P402668	Fbxo6b	NM_138917	192351	0.22	1.667	0.011549
A_44_P258277	Dhrs7b	NM_001008507	287380	0.22	1.667	0.009664
A_43_P21582	Tmtc2_predicted	XM_235136	299762	0.22	1.667	0.005049
A_43_P18208	Pitrm1_predicted	XM_225517		0.22	1.667	0.007135
A_44_P257628	Aip1	NM_173117	286897	0.22	1.667	0.001805
A_44_P491824	Camk2g	NM_133605	171140	0.22	1.667	0.001428
A_44_P262289	Hmbs	NM_013168	25709	0.22	1.667	0.005135

A_44_P790553	A_44_P790553	A_44_P790553		0.22	1.666	0.004722
A_44_P1030766	Pcf11_predicted	XM_341883	361605	0.22	1.666	0.240041
A_43_P18907	Lrig1_predicted	XM_232237	312574	0.22	1.666	0.009779
A_43_P16672	Txndc1	NM_001024800	362751	0.22	1.666	0.022721
A_44_P344863	Ddx50	XM_001058536		0.22	1.666	0.002057
A_44_P511178	Pus7_predicted	XM_216060	296751	0.22	1.666	0.004002
A_44_P191540	Fpgt	NM_199494	310935	0.22	1.666	0.104233
A_44_P102652	Ikbkap	NM_080899	140934	0.22	1.666	0.003881
A_44_P659008	TC543546	TC543546		0.22	1.666	0.124933
A_44_P987888	AW914294	AW914294		0.22	1.665	0.028573
A_44_P881890	BF289610	BF289610		0.22	1.665	0.005492
A_44_P914528	Ccdc21	XM_342940		0.22	1.665	0.020037
A_44_P513487	Gca_predicted	XM_229977		0.22	1.665	0.01786
A_43_P22210	Zfp644_predicted	XM_223150	305127	0.22	1.665	0.003012
A_44_P776069	RGD1311559_predicted	XM_235398	315055	0.22	1.665	0.003359
A_44_P140416	RGD1310710_predicted	XM_232333	312677	0.22	1.665	0.014753
A_44_P496438	Cyb5r4	NM_133427	171015	0.22	1.664	0.003
A_43_P17615	RGD1306924_predicted	XM_001076537		0.22	1.664	0.000683
A_44_P168661	RGD1309731_predicted	XM_226503	307845	0.22	1.664	0.01055
A_44_P544552	Usp3	NM_001025424	363084	0.22	1.664	0.033998
A_44_P542887	Josd3	NM_001014207	363017	0.22	1.664	0.004976
A_44_P759589	CF110322	CF110322		0.22	1.664	0.01755
A_44_P525295	RGD1307355	XM_231122		0.22	1.664	0.129048
A_44_P527795	Sesn3_predicted	XM_235825		0.22	1.664	0.003239
A_44_P205721	Ppie	XM_216524		0.22	1.664	0.001477
A_44_P289424	Mrps14_predicted	XM_213906		0.22	1.663	0.002961
A_43_P21758	Usp9x_predicted	XM_343766	363445	0.22	1.663	0.007044
A_42_P481696	Mthfd1	NM_022508	64300	0.22	1.663	0.001935
A_44_P512728	Pxn	NM_001012147	360820	0.22	1.663	0.001693
A_44_P568560	CD371170	CD371170		0.22	1.663	0.003564
A_44_P367624	LOC317588	AY321321	317588	0.22	1.662	0.00423
A_44_P885959	TC561803	TC561803		0.22	1.662	0.013392
A_44_P383555	Asb16	NM_001017988	303566	0.22	1.662	0.022301
A_44_P471426	Traf3ip1	NM_001012204	363286	0.22	1.662	0.001518
A_42_P572366	Myst2	NM_181081	303470	0.22	1.662	0.018311
A_44_P640734	TC556525	TC556525		0.22	1.662	0.040472
A_44_P855350	TC530277	TC530277		0.22	1.662	0.003827
A_44_P461579	Ptpn4	XM_341109	246116	0.22	1.662	0.009369
A_44_P185138	ENSRNOT00000036884	ENSRNOT00000036884		0.22	1.661	0.000346
A_44_P375597	RGD1311710	NM_001014099	316275	0.22	1.661	0.01087
A_44_P452677	Ing4	XM_216265		0.22	1.661	0.002308
A_44_P388720	Fstl3	NM_053629	114031	0.22	1.661	0.062772
A_42_P707872	Stk39	NM_019362	54348	0.22	1.661	0.126628
A_44_P1027866	Nup93	NM_001011925	291874	0.22	1.661	0.0021
A_43_P18388	Ddx51_predicted	XM_222252		0.22	1.661	0.000499
A_43_P16436	RGD1566282_predicted	XM_236533	315911	0.22	1.661	0.054943
A_44_P480830	LOC690895	XM_345907		0.22	1.661	0.084395
A_44_P210692	RGD1564930_predicted	XM_573657		0.22	1.661	0.005375
A_44_P473459	Tm4sf12	NM_001015026	362326	0.22	1.661	0.130098
A_44_P302237	Slc39a1_predicted	XM_342286	361986	0.22	1.660	0.003114
A_44_P927847	AW920761	AW920761		0.22	1.660	0.048418
A_44_P517650	Adam17	NM_020306	57027	0.22	1.660	0.005504
A_44_P825753	TC559674	TC559674		0.22	1.660	0.033389
A_44_P152290	RGD1306498_predicted	XM_216345	297879	0.22	1.660	0.002223
A_42_P752336	Nef3	NM_017029	24588	0.22	1.660	0.199132
A_43_P12202	Snx16	NM_022289	64088	0.22	1.659	0.013888
A_44_P875993	A_44_P875993	A_44_P875993		0.22	1.659	0.03483
A_44_P836806	ENSRNOT00000001255	ENSRNOT00000001255		0.22	1.659	0.002177
A_44_P212682	LOC502374	AY325138	502374	0.22	1.659	0.000745
A_42_P697274	AW920707	AW920707		0.22	1.659	0.274292
A_44_P326124	LOC679385	XM_001056105		0.22	1.659	0.009991
A_44_P433217	Ppp1r11	NM_212542	294207	0.22	1.659	0.003392
A_44_P361014	Peli3_predicted	XM_219692	309157	0.22	1.658	0.013024

A_44_P203242	Utrn	NM_013070	25600	0.22	1.658	0.002899
A_43_P17139	Ifi35	NM_001009625	287719	0.22	1.658	0.00421
A_44_P223305	CB547154	CB547154	310807	0.22	1.658	0.017767
A_44_P156988	Mbtps2	NM_001035007	302705	0.22	1.658	0.023604
A_44_P556895	Ddit4	NM_080906	140942	0.22	1.657	0.005202
A_44_P478110	Rab2b	NM_001037645	305853	0.22	1.657	0.056824
A_44_P187706	LOC315676	XM_001075770	315676	0.22	1.657	0.127761
A_44_P336664	BF558804	BF558804		0.22	1.657	0.006901
A_44_P125733	Madcam1	NM_019317	54266	0.22	1.657	0.10416
A_44_P1033388	Agl_predicted	XM_342331		0.22	1.656	0.171376
A_44_P698714	LOC500199	XM_001063161		0.22	1.656	0.012533
A_44_P356492	LOC362154	NM_001010963	362154	0.22	1.656	0.005281
A_44_P916289	TC545184	TC545184		0.22	1.656	0.001309
A_44_P539019	Qser1_predicted	XM_230348	311266	0.22	1.656	0.011264
A_42_P670631	RGD1305645_predicted	XM_343562	363225	0.22	1.656	0.01587
A_44_P152421	XM_340800	XM_340800		0.22	1.655	0.035944
A_43_P20736	Dph2	NM_001015007	298452	0.22	1.655	0.000659
A_42_P485589	Map2k5	NM_017246	29568	0.22	1.655	0.001323
A_44_P1020448	RGD1309896_predicted	XM_341852	361569	0.22	1.655	0.008107
A_43_P12584	Nfyb	NM_031553	25336	0.22	1.655	0.004178
A_44_P880354	Prkacb_predicted	XM_215070		0.22	1.655	0.01353
A_42_P683988	Exosc9	NM_001025406	294975	0.22	1.655	0.004275
A_44_P1035546	Ddx56	NM_001004211	289780	0.22	1.655	0.001778
A_44_P547133	Leprot	NM_020099	24536	0.22	1.655	0.047619
A_44_P105377	Cenpb_predicted	XM_342521		0.22	1.655	0.012094
A_44_P550590	RGD1310133_predicted	XM_218477		0.22	1.654	0.004934
A_43_P15253	Icam1	NM_012967	25464	0.22	1.654	0.001046
A_44_P928834	A_44_P928834	A_44_P928834		0.22	1.654	0.005797
A_44_P334145	LOC687346	XM_001075972		0.22	1.654	0.004434
A_44_P107285	Psmb6	NM_057099	29666	0.22	1.654	0.001074
A_43_P18282	RGD1311345	XM_341483	361201	0.22	1.653	0.000689
A_44_P776131	AABR03074271	AABR03074271		0.22	1.653	0.048426
A_44_P1019537	Cbx5_predicted	XM_217062		0.22	1.653	0.002267
A_44_P290591	Pvrl2	NM_001012064	308417	0.22	1.653	0.006168
A_43_P22187	Nol6_predicted	XM_232898		0.22	1.652	0.003245
A_43_P18066	Nup98	XM_574504	81738	0.22	1.652	0.008766
A_44_P710780	AW143784	AW143784		0.22	1.652	0.007739
A_44_P450562	RGD1310043_predicted	XM_221115	287827	0.22	1.652	0.004246
A_44_P514539	Eif4b	NM_001008324	300253	0.22	1.652	0.010814
A_43_P15527	Hmgb1	NM_012963	25459	0.22	1.651	0.003679
A_44_P269709	LOC689377	XM_001070580		0.22	1.651	0.009668
A_44_P398355	A_44_P398355	A_44_P398355		0.22	1.651	0.062834
A_44_P135304	Cdk105	NM_134415	171456	0.22	1.651	0.000645
A_44_P957346	AI230598	AI230598		0.22	1.651	0.032026
A_44_P914285	LOC681996	XM_001062172		0.22	1.651	0.015288
A_44_P525250	RGD1560208_predicted	XM_227370		0.22	1.651	0.013483
A_44_P729179	LOC500598	NM_001024349	500598	0.22	1.651	0.078631
A_44_P204078	RGD1561926_predicted	XM_576077		0.22	1.651	0.002867
A_44_P454177	Rab40c	NM_182675	359728	0.22	1.651	0.023022
A_44_P1040609	Ascc3l1	BC099211	296126	0.22	1.651	0.001602
A_44_P297092	RGD1563764_predicted	XM_575385		0.22	1.650	0.001554
A_44_P236453	LOC688540	XM_001064901	688540	0.22	1.650	0.135169
A_44_P135678	RGD1305020_predicted	XM_230785		0.22	1.650	0.001034
A_44_P212294	DY472414	DY472414		0.22	1.650	0.030606
A_43_P19410	LOC361335	XM_001057816		0.22	1.650	0.002311
A_43_P12625	Gprk6	NM_031657	59076	0.22	1.650	0.001058
A_44_P248193	Rel_predicted	XM_223688		0.22	1.650	0.000605
A_44_P761215	BC091405	BC091405		0.22	1.650	0.034327
A_44_P257221	Atp6v1b2	NM_057213	117596	0.22	1.650	0.003525
A_44_P236876	LOC678715	XM_001053109		0.22	1.650	0.021319
A_44_P884698	TC556147	TC556147		0.22	1.650	0.013781
A_44_P150036	Atf7_predicted	XM_235694		0.22	1.650	0.024454
A_44_P555921	ENSRNOT00000003511	ENSRNOT00000003511		0.22	1.650	0.001823

A_44_P515607	Rkhd3_predicted	XM_218846	308790	0.22	1.650	0.001116
A_44_P494754	Clspn_predicted	XM_233524		0.22	1.650	0.005049
A_44_P123926	LOC310968	XM_227820	310968	0.22	1.649	0.184685
A_44_P405270	XM_218755	XM_218755		0.22	1.649	0.055082
A_44_P229122	Spag5	XM_340848		0.22	1.649	0.041437
A_44_P448029	LOC680111	XM_001055763	680111	0.22	1.649	0.149295
A_44_P1022237	Rab26	NM_133580	171111	0.22	1.649	0.154025
A_42_P613948	MGC93975	NM_001004221	292878	0.22	1.648	0.001969
A_43_P12219	Qdpr	NM_022390	64192	0.22	1.648	0.002203
A_44_P295400	Akna_predicted	XM_342848		0.22	1.648	0.00721
A_44_P475804	A_44_P475804	A_44_P475804		0.22	1.648	0.010227
A_43_P22042	Pla2g7	NM_001009353	301265	0.22	1.648	0.019058
A_44_P396522	H2a	NM_021840	64646	0.22	1.648	0.055896
A_42_P752134	Kpna2	NM_053483	85245	0.22	1.648	0.003162
A_44_P582605	A_44_P582605	A_44_P582605		0.22	1.648	0.013768
A_44_P203620	RGD1566016_predicted	XM_341116	360840	0.22	1.647	0.003917
A_43_P11873	Pxmp3	NM_017234	29534	0.22	1.647	0.037654
A_42_P637279	Foxa2	NM_012743	25099	0.22	1.647	0.002188
A_44_P222481	Mrps27_predicted	XM_342180		0.22	1.647	0.010428
A_44_P445694	RGD1306697_predicted	XM_231599	312225	0.22	1.647	0.003868
A_44_P173575	Rassf1	NM_001007754	363140	0.22	1.647	0.008461
A_43_P20266	RGD1562456_predicted	XM_226817		0.22	1.647	0.019801
A_44_P304873	Toe1_predicted	XM_216504		0.22	1.647	0.004462
A_42_P695042	Csf1	NM_023981	78965	0.22	1.647	0.004775
A_44_P311136	Vav1	NM_012759	25156	0.22	1.647	0.015996
A_44_P123974	LOC361335	XM_001057816		0.22	1.647	0.000566
A_44_P201796	Slc25a32_predicted	XM_235359	315023	0.22	1.647	0.004714
A_43_P22661	ENSRNOT00000018311	ENSRNOT00000018311		0.22	1.647	0.058687
A_44_P1048234	RGD1565180_predicted	XM_343497		0.22	1.647	0.009411
A_44_P484011	AW142545	AW142545	309681	0.22	1.646	0.003063
A_42_P553444	Pcsk5	XM_342032	116548	0.22	1.646	0.01509
A_44_P342166	Plcg1	NM_013187	25738	0.22	1.646	0.004153
A_44_P267529	Terf1	NM_001012464	297758	0.22	1.646	0.026192
A_44_P821710	A_44_P821710	A_44_P821710		0.22	1.646	0.002553
A_44_P419265	Zfp96	NM_153475		0.22	1.646	0.011176
A_44_P155879	AW144203	AW144203		0.22	1.646	0.008379
A_43_P19909	RGD1563715_predicted	XM_573146		0.22	1.646	0.001225
A_43_P19560	Brf1_predicted	XM_216801		0.22	1.646	0.002966
A_44_P1030098	LOC287961	XM_213587		0.22	1.646	0.004929
A_44_P440280	Atp6v0c	NM_130823	170667	0.22	1.646	0.00505
A_44_P450556	Meox1_predicted	XM_343970		0.22	1.646	0.11205
A_44_P555213	Cenpf	XM_223060	257649	0.22	1.646	0.018121
A_43_P10981	CB605839	CB605839	294230	0.22	1.645	0.00461
A_44_P605893	LOC364713	XR_008631	364713	0.22	1.645	0.028844
A_43_P16583	Snx2_predicted	XM_214539		0.22	1.645	0.003024
A_44_P455656	Bmpr1a	NM_030849	81507	0.22	1.645	0.012005
A_44_P138363	LOC363849	XM_344057	363849	0.22	1.645	0.001976
A_44_P314133	XM_225858	XM_225858		0.22	1.645	0.002757
A_44_P285530	Eif2s2	NM_199380	296302	0.22	1.645	0.002785
A_43_P20957	RGD1310093_predicted	XM_220978		0.22	1.645	0.024544
A_42_P477073	Ctbp1	NM_019201	29382	0.22	1.645	0.003352
A_44_P806641	XM_236024	XM_236024		0.22	1.645	0.00269
A_43_P18517	Baz2a_predicted	XM_222315		0.22	1.644	0.012849
A_44_P225046	RGD1309887_predicted	XM_228042	294307	0.22	1.644	0.014851
A_44_P304469	RGD1309326	NM_001014018	308568	0.22	1.644	0.000937
A_44_P762216	RGD1563166_predicted	XM_575612		0.22	1.644	0.004703
A_44_P304542	Gas2l1_predicted	XM_341251		0.22	1.644	0.004219
A_43_P17631	BC093376	BC093376		0.22	1.644	0.012788
A_44_P478066	Incenp_predicted	XM_215184		0.22	1.644	0.01473
A_44_P392370	Tbn_predicted	XM_001061330		0.22	1.644	0.031584
A_43_P19100	RGD1310674	XM_001058775	303790	0.22	1.644	0.002594
A_42_P696084	Cspg4	NM_031022	81651	0.22	1.643	0.028551
A_44_P312166	XM_344042	XM_344042		0.22	1.643	0.093694

A_43_P22180	RGD1563977_predicted	XM_001053351		0.22	1.643	0.177716
A_44_P521963	AW915317	AW915317	94197	0.22	1.643	0.009069
A_43_P16700	Cdc26	NM_001013240	366381	0.22	1.643	0.010861
A_44_P494696	A_44_P494696	A_44_P494696		0.22	1.643	0.005669
A_43_P17979	XM_227427	XM_227427		0.22	1.643	0.002429
A_44_P966553	TC523446	TC523446		0.22	1.643	0.030213
A_44_P375158	LOC681172	XM_001060612		0.22	1.642	0.000515
A_44_P507881	AI502676	AI502676	252855	0.22	1.642	0.008487
A_44_P318756	Ttc23	NM_001025681	308708	0.22	1.642	0.034021
A_44_P434462	Pemt	NM_013003	25511	0.22	1.642	0.009521
A_42_P545721	Snrpe_predicted	XM_001065908		0.22	1.642	0.006598
A_44_P258168	Kif26a_predicted	XM_234565	314473	0.22	1.642	0.007357
A_44_P400135	Hs1bp1	NM_181627	291202	0.22	1.642	0.001596
A_43_P17107	CB547916	CB547916	302502	0.22	1.641	0.181415
A_44_P860225	TC560545	TC560545		0.22	1.641	0.025165
A_44_P1009426	XM_345633	XM_345633		0.22	1.641	0.007594
A_44_P436597	RGD1306284	NM_001008283	287918	0.22	1.641	0.08446
A_43_P23404	Sufu	NM_001024899	361769	0.22	1.641	0.000438
A_43_P16449	Pelo	NM_001007634	294754	0.22	1.641	0.001518
A_44_P259574	Pgam1	NM_053290	24642	0.22	1.641	0.021278
A_44_P235028	AI101484	AI101484		0.22	1.641	0.102317
A_44_P483212	RGD1307697_predicted	XM_216922	299909	0.22	1.641	0.009676
A_44_P276811	Matn4_predicted	XM_215932		0.22	1.641	0.090498
A_44_P325299	AW914920	AW914920	360916	0.22	1.641	0.199638
A_42_P797965	RGD1306959_predicted	XM_341902		0.21	1.640	0.009521
A_44_P500923	Hnrph1	NM_080896	140931	0.21	1.640	0.008445
A_44_P138347	LOC619573	NM_001034958	619573	0.21	1.640	0.008699
A_44_P992502	Narg1_predicted	XM_241375		0.21	1.640	0.002707
A_44_P605123	DV716579	DV716579		0.21	1.640	0.14639
A_44_P615549	Nfatc3_predicted	XM_341680		0.21	1.640	0.00257
A_44_P981373	Rap2b	CO567301		0.21	1.640	0.011799
A_43_P12637	Uba52	NM_031687	64156	0.21	1.640	0.000993
A_43_P21085	Klhl2_predicted	XM_214331	290692	0.21	1.640	0.005275
A_44_P243081	LOC498560	XM_341367		0.21	1.640	0.004221
A_44_P313283	Capn2	NM_017116	29154	0.21	1.639	0.004377
A_44_P363348	LOC680493	XM_001057427		0.21	1.639	0.002131
A_44_P199489	Wdr75	XM_234768		0.21	1.639	0.005958
A_44_P147726	RGD1305052_predicted	XM_221142	303698	0.21	1.639	0.004103
A_42_P517712	RGD1565864_predicted	XM_234568	299317	0.21	1.639	0.007983
A_44_P1014862	Ubxd6_predicted	XM_214360		0.21	1.639	0.022119
A_44_P886515	TC561906	TC561906		0.21	1.639	0.002691
A_44_P988046	BG668924	BG668924		0.21	1.639	0.024263
A_44_P394270	XM_346909	XM_346909		0.21	1.639	0.082958
A_44_P504036	Dusp16_predicted	XM_232473		0.21	1.638	0.006279
A_44_P109927	Ahsa2_predicted	XM_223680		0.21	1.638	0.007241
A_44_P505729	Mutyh	NM_133316	170841	0.21	1.638	0.01303
A_42_P598602	Cd3g	XM_217136		0.21	1.638	0.11221
A_44_P690321	AI598485	AI598485		0.21	1.638	0.003034
A_44_P523261	RGD1559743_predicted	XM_236683	316048	0.21	1.638	0.001413
A_44_P1060444	RGD1305158_predicted	XM_216368		0.21	1.638	0.131467
A_43_P11630	Fgf10	NM_012951	25443	0.21	1.637	0.088133
A_43_P14869	Adss_predicted	XM_222946		0.21	1.637	0.00284
A_44_P412503	Nisch	XM_240330	306255	0.21	1.637	0.001673
A_44_P258203	RGD1561984_predicted	XM_236218		0.21	1.637	0.001582
A_44_P510918	Rnf121_predicted	XM_238909		0.21	1.637	0.11675
A_44_P406805	Rfc3	NM_001009629	288414	0.21	1.636	0.008266
A_43_P20207	Klhl21_predicted	XM_233701		0.21	1.636	0.003344
A_44_P343060	Pik3r3	NM_022213	60664	0.21	1.636	0.079414
A_44_P291060	Thrap5_predicted	XM_216837		0.21	1.636	0.011612
A_44_P499076	A_44_P499076	A_44_P499076		0.21	1.636	0.014264
A_44_P365901	RGD1311739	NM_001025691	311428	0.21	1.636	0.011572
A_44_P107801	LOC680616	XM_001058014	680616	0.21	1.636	0.101545
A_44_P1042372	Kifc1	NM_001005878	294286	0.21	1.636	0.012011

A_44_P480157	Kif4	XM_343797	84393	0.21	1.636	0.010047
A_44_P137732	XM_344104	XM_344104		0.21	1.635	0.000859
A_44_P674506	AI599250	AI599250		0.21	1.635	0.002212
A_44_P250203	Rbm12	NM_001037657	652928	0.21	1.635	0.007438
A_42_P700829	Dnttip2_predicted	XM_215688		0.21	1.635	0.000892
A_44_P930764	Bfar	NM_001013125	304709	0.21	1.635	0.032371
A_44_P185367	Eif3s4	NM_001013095	298700	0.21	1.635	0.006394
A_44_P137039	AA964058	AA964058	81770	0.21	1.634	0.006886
A_44_P1044499	RGD1306067	XM_345440	366203	0.21	1.634	0.006807
A_44_P264910	RGD1305903_predicted	XM_219346	308992	0.21	1.634	0.005804
A_43_P13059	Ube1c	NM_057205	117553	0.21	1.634	0.184575
A_42_P600947	LOC301130	XM_217309	301130	0.21	1.634	0.012493
A_44_P1004951	Usp6nl_predicted	XM_214508		0.21	1.634	0.035636
A_44_P440556	Muc13	NM_139041	207126	0.21	1.634	0.031399
A_42_P639337	LOC367808	NM_001014272	367808	0.21	1.633	0.006204
A_44_P351636	Zcchc4_predicted	XM_341224		0.21	1.633	0.071767
A_43_P12466	Ogg1	NM_030870	81528	0.21	1.633	0.002903
A_43_P10054	Osbpl9_predicted	XM_216477		0.21	1.633	0.004722
A_44_P354385	Aste1	XM_001072964		0.21	1.633	0.045442
A_44_P913962	ENSRNOT00000048665	ENSRNOT00000048665		0.21	1.633	0.00221
A_44_P167883	RGD1303127	NM_001004244	300206	0.21	1.632	0.001496
A_44_P444001	Rhoh	NM_001013430	305341	0.21	1.632	0.00426
A_44_P321515	XM_345668	XM_345668		0.21	1.632	0.227133
A_44_P271769	Acp2	NM_016988	24162	0.21	1.632	0.005877
A_43_P16591	LOC678860	XM_001053611		0.21	1.632	0.043326
A_44_P820573	RGD1311331_predicted	XM_001071788		0.21	1.632	0.007921
A_44_P297175	Cd36	NM_031561	29184	0.21	1.631	0.020579
A_44_P311969	LOC362681	XM_342996		0.21	1.631	0.003679
A_44_P823025	TC557159	TC557159		0.21	1.631	0.011847
A_44_P792507	CO406134	CO406134		0.21	1.631	0.08387
A_44_P223651	Cggbp1_predicted	XM_213679		0.21	1.631	0.01156
A_43_P11786	Pim1	NM_017034	24649	0.21	1.630	0.064518
A_44_P683778	BQ780539	BQ780539		0.21	1.630	0.005328
A_44_P1047364	RGD1566265_predicted	XM_343810	363487	0.21	1.630	0.206442
A_44_P557857	Ccdc32	NM_001024245	296081	0.21	1.630	0.11588
A_44_P431572	Rasgrp3_predicted	XM_233873		0.21	1.630	0.123555
A_44_P347374	A_44_P347374	A_44_P347374		0.21	1.630	0.021681
A_44_P1036408	Ywhaq	NM_013053	25577	0.21	1.630	0.003792
A_44_P654973	Rimbp2	XM_001073406		0.21	1.630	0.200325
A_44_P331765	LOC500855	XM_576251	500855	0.21	1.630	0.005332
A_44_P281193	XM_341927	XM_341927		0.21	1.630	0.004412
A_43_P14221	Tkt	NM_022592	64524	0.21	1.630	0.009173
A_44_P496934	Srfbp1	NM_001005536	291469	0.21	1.629	0.012783
A_44_P109174	BG665185	BG665185		0.21	1.629	0.279604
A_44_P466489	MGC93920	NM_001007642	295663	0.21	1.629	0.041358
A_44_P432889	LOC682571	XM_001062077		0.21	1.629	0.001236
A_44_P196984	Fcmd_predicted	XM_342838		0.21	1.629	0.049287
A_44_P357879	Tnpo1	XM_219500	309126	0.21	1.629	0.004941
A_43_P17577	Vps11_predicted	XM_236189		0.21	1.629	0.026361
A_44_P239171	Rpl35a	NM_021264	57809	0.21	1.628	0.001202
A_44_P479520	Eif4g2_predicted	XM_341907		0.21	1.628	0.005903
A_44_P377803	Exph5_predicted	XM_236272	315663	0.21	1.628	0.021611
A_44_P101625	Camk2a	NM_012920	25400	0.21	1.628	0.095175
A_44_P281457	LOC679899	XM_001054883		0.21	1.628	0.00244
A_43_P18701	Galc	NM_001005888	314360	0.21	1.628	0.004331
A_44_P153949	H2afy	NM_017182	29384	0.21	1.628	0.00104
A_44_P481330	AI145784	AI145784	361814	0.21	1.628	0.004654
A_44_P384379	Nup98	XM_574504	81738	0.21	1.628	0.017397
A_44_P1059056	Appbp1	NM_032072	84019	0.21	1.628	0.003837
A_43_P20097	LOC362587	XM_342906	362587	0.21	1.627	0.004109
A_44_P746394	TC555572	TC555572		0.21	1.627	0.01861
A_44_P326810	XM_226441	XM_226441		0.21	1.627	0.003486
A_44_P224997	RGD1563127_predicted	XM_347035	362194	0.21	1.627	0.005143

A_43_P21605	Tarsl2	NM_001014020	308701	0.21	1.627	0.007937
A_43_P12756	Pkm2	NM_053297	25630	0.21	1.627	0.02271
A_44_P1024729	RGD1311429_predicted	XM_001081571		0.21	1.627	0.035249
A_43_P21088	RGD1306214_predicted	XM_229993	311093	0.21	1.627	0.005003
A_44_P622466	CO569886	CO569886		0.21	1.627	0.139524
A_44_P303651	Prkwnk1	NM_053794	116477	0.21	1.626	0.004652
A_44_P314492	Tiam1	XM_221672	304109	0.21	1.626	0.0029
A_44_P911610	AW530749	AW530749		0.21	1.626	0.03816
A_44_P487579	XM_226089	XM_226089		0.21	1.626	0.011358
A_44_P346856	Ptov1	NM_001008304	292888	0.21	1.626	0.000619
A_44_P416761	Lmna	NM_001002016	60374	0.21	1.626	0.013592
A_44_P215654	XM_343809	XM_343809		0.21	1.626	0.010591
A_44_P212850	Fgf2	NM_019305	54250	0.21	1.626	0.016681
A_44_P537367	CB547440	CB547440		0.21	1.626	0.007953
A_44_P684075	RGD1563764_predicted	XM_575385		0.21	1.626	0.006829
A_44_P822789	TC555365	TC555365		0.21	1.626	0.010587
A_44_P808496	RGD1562590_predicted	XM_001058587		0.21	1.626	0.003181
A_44_P323649	XM_223835	XM_223835		0.21	1.626	0.010032
A_43_P18835	LOC606294	NM_001031627	606294	0.21	1.625	0.039267
A_44_P391302	Cmklr1	NM_022218	60669	0.21	1.625	0.005335
A_43_P11990	Gjb5	NM_019241	29586	0.21	1.625	0.074764
A_44_P369056	CF978977	CF978977	288108	0.21	1.625	0.001625
A_44_P266879	Rdbp	NM_212548	294258	0.21	1.625	0.002319
A_44_P501142	Srebf1	XM_213329	78968	0.21	1.625	0.00589
A_44_P359150	G3bp	XM_340802	171092	0.21	1.625	0.008327
A_44_P356711	Ttc7b_predicted	XM_343094		0.21	1.625	0.005915
A_44_P309704	Ctns_predicted	XM_001080248		0.21	1.625	0.006875
A_42_P645110	RGD1306053	NM_001029919	312694	0.21	1.624	0.023509
A_44_P411919	Psmd1	NM_031978	83806	0.21	1.624	0.001813
A_44_P745432	TC537925	TC537925		0.21	1.624	0.00303
A_44_P412625	RGD1560391_predicted	NM_001039036	499883	0.21	1.624	0.015209
A_44_P452078	Srr	NM_198757	303306	0.21	1.624	0.045932
A_43_P14621	Dbi	NM_031853	25045	0.21	1.624	0.017945
A_44_P210907	Txnl4b	NM_001013891	292008	0.21	1.624	0.016811
A_44_P203514	Eif4e	NM_053974	117045	0.21	1.623	0.007065
A_44_P325285	Ecgf1	NM_001012122	315219	0.21	1.623	0.000813
A_44_P106221	Calca	NM_017338	24241	0.21	1.623	0.04923
A_44_P361357	Sfpq	XM_001058863		0.21	1.623	0.012333
A_44_P982920	A_44_P982920	A_44_P982920		0.21	1.623	0.019475
A_44_P1014388	LOC499392	XM_574708		0.21	1.622	0.010584
A_44_P685298	LOC679306	XM_001056215		0.21	1.622	0.079383
A_44_P851620	RGD1306107_predicted	XM_227412		0.21	1.622	0.002543
A_44_P503673	Ulk1_mapped	XM_341100		0.21	1.622	0.020147
A_42_P460122	Higd2a_predicted	XM_214433		0.21	1.622	0.00926
A_44_P556989	Ngfr	NM_012610	24596	0.21	1.622	0.026551
A_43_P21324	Mesdc2	NM_001008345	308796	0.21	1.622	0.022394
A_44_P316916	RGD1560437_predicted	XM_213595		0.21	1.622	0.020171
A_44_P236776	RGD1561474_predicted	XM_243478		0.21	1.622	0.063405
A_43_P13386	Prpf19	NM_139333	246216	0.21	1.622	0.008842
A_44_P473767	Rpl21	NM_053330	79449	0.21	1.622	0.002788
A_44_P543611	XM_344411	XM_344411		0.21	1.622	0.005864
A_44_P256328	CB544976	CB544976	192357	0.21	1.621	0.00724
A_44_P1054967	Sirt7_predicted	XM_221204		0.21	1.621	0.075105
A_44_P236539	XM_230521	XM_230521		0.21	1.621	0.045293
A_44_P544121	Kctd6_predicted	XM_223921		0.21	1.621	0.008741
A_44_P503964	Trmt1	NM_001013870	288914	0.21	1.621	0.010246
A_44_P257832	Farp1_predicted	XM_224535		0.21	1.620	0.005809
A_44_P578250	DV714571	DV714571		0.21	1.620	0.257957
A_43_P16611	Usp9x_predicted	XM_001056701		0.21	1.620	0.016074
A_44_P408011	Fusip1	NM_001025738	362630	0.21	1.620	0.004521
A_43_P16323	RGD1359435	XM_001074993		0.21	1.619	0.063406
A_44_P398729	Stno_predicted	XM_234899		0.21	1.619	0.007565
A_44_P423353	AW143318	AW143318		0.21	1.619	0.009411

A_44_P316507	Tmed9	NM_001009703	361207	0.21	1.619	0.004764
A_44_P321945	Cyb5r1	NM_001013126	304805	0.21	1.619	0.004499
A_44_P852695	LOC367738	XR_007382	367738	0.21	1.618	0.007784
A_44_P148440	RGD1565940_predicted	XM_573456		0.21	1.618	0.003654
A_44_P492268	XM_212982	XM_212982		0.21	1.618	0.005812
A_44_P164120	Gtf3c3_predicted	XM_237623		0.21	1.618	0.004176
A_44_P503809	LOC690163	XM_001073523	690163	0.21	1.618	0.020831
A_44_P520475	Anp32e	NM_001013200	361999	0.21	1.618	0.005059
A_44_P377722	Peflin	NM_001007651	297900	0.21	1.618	0.008617
A_44_P443686	AA956612	AA956612		0.21	1.618	0.025958
A_44_P301574	Pcnx	XM_234385	314288	0.21	1.618	0.002077
A_44_P930354	RGD1308918_predicted	XM_219441		0.21	1.618	0.063011
A_44_P946401	TC543078	TC543078		0.21	1.617	0.22793
A_43_P22409	RGD1565672_predicted	XM_001064634		0.21	1.617	0.118587
A_44_P165989	LOC361596	NM_001014161	361596	0.21	1.617	0.004791
A_44_P246268	Ngp_predicted	XM_236646		0.21	1.617	0.023674
A_44_P548455	RGD1563300_predicted	XM_225320		0.21	1.616	0.002871
A_44_P455160	Tcfap4_predicted	XM_340756		0.21	1.616	0.004518
A_44_P1056383	RGD1311269_predicted	XM_217397	301419	0.21	1.616	0.036928
A_44_P250368	Tk1	XM_001081763		0.21	1.616	0.021367
A_44_P696248	LOC362012	XM_342311	362012	0.21	1.616	0.001633
A_44_P410033	Wdr41_predicted	XM_342175		0.21	1.616	0.021779
A_44_P527883	Maml1_predicted	XM_220373		0.21	1.616	0.006117
A_44_P271184	DY315710	DY315710		0.21	1.616	0.004816
A_44_P130849	Ercc6_predicted	XM_224627		0.21	1.616	0.006598
A_43_P20777	Nsun2_predicted	XM_341474		0.21	1.616	0.002372
A_44_P426904	Eif2c4_predicted	XM_233545		0.21	1.616	0.033228
A_44_P477905	Psmb4	NM_031629	58854	0.21	1.616	0.001608
A_44_P884680	RGD1309403_predicted	XM_001057453		0.21	1.616	0.137852
A_44_P384506	RGD1311793_predicted	XM_224908		0.21	1.616	0.003668
A_42_P459149	Phkg2	NM_080584	140671	0.21	1.616	0.010642
A_44_P867817	LOC688583	XM_001067503		0.21	1.616	0.001259
A_44_P222786	RGD1563296_predicted	XM_001076141		0.21	1.615	0.043585
A_43_P15924	Pola1	XM_242396	85241	0.21	1.615	0.018744
A_44_P151982	LOC686418	XM_001074019		0.21	1.615	0.005894
A_44_P230813	XM_220753	XM_220753		0.21	1.615	0.001053
A_44_P991975	Eef1d	NM_001013104	300033	0.21	1.615	0.001999
A_44_P497339	Asf1a_predicted	XM_215389	294408	0.21	1.615	0.004168
A_44_P155572	Kctd10	NM_001009973	494521	0.21	1.615	0.022722
A_44_P562884	TC560826	TC560826		0.21	1.615	0.028817
A_44_P880146	BG666928	BG666928	64625	0.21	1.615	0.001627
A_44_P523052	Agpat3_predicted	XM_215367		0.21	1.615	0.018954
A_44_P186902	Pgpep1	NM_201988	290648	0.21	1.615	0.007959
A_44_P384090	Des	NM_022531	64362	0.21	1.615	0.05724
A_44_P363505	Lsm1_predicted	XM_344536		0.21	1.615	0.007466
A_44_P335059	Ddx46	NM_139098	245957	0.21	1.615	0.015413
A_44_P185320	Lrrc41	NM_001009710	362566	0.21	1.615	0.007112
A_44_P105697	Scly	NM_001007755	363285	0.21	1.615	0.015812
A_44_P269865	LOC367808	NM_001014272	367808	0.21	1.614	0.007372
A_43_P15432	Pscd1	NM_053910	116691	0.21	1.614	0.025413
A_44_P250678	Golga1_predicted	XM_231193		0.21	1.614	0.118089
A_44_P270169	AA900215	AA900215		0.21	1.614	0.012246
A_44_P130909	XM_216037	XM_216037		0.21	1.614	0.003475
A_44_P203106	Ifrd1	NM_019242	29596	0.21	1.613	0.003943
A_44_P134414	AA858962	AA858962	25703	0.21	1.613	0.1749
A_42_P681694	Maea	NM_001008319	298982	0.21	1.613	0.004627
A_43_P12227	Crip2	NM_022501	338401	0.21	1.613	0.047632
A_44_P114023	LOC501706	XM_577111		0.21	1.613	0.004604
A_43_P12540	Dgkz	NM_031143	81821	0.21	1.613	0.003897
A_44_P178252	Ccl21b	NM_001008513	298006	0.21	1.613	0.1741
A_44_P867677	ENSRNOT00000035789	ENSRNOT00000035789		0.21	1.613	0.000993
A_44_P532832	BF389503	BF389503	315438	0.21	1.613	0.020764
A_44_P206302	Slco5a1_predicted	XM_232599		0.21	1.613	0.092385

A_44_P156439	Trim28	XM_344861	116698	0.21	1.613	0.003955
A_44_P257526	Abcc4	NM_133411	170924	0.21	1.613	0.009153
A_44_P345169	Prkdc_predicted	XM_341020		0.21	1.612	0.006229
A_44_P306568	Tmco1	NM_001009631	289196	0.21	1.612	0.062418
A_44_P187166	Tubb2b	NM_001013886	291081	0.21	1.612	0.003827
A_43_P16640	RGD1311547	XM_343298	362967	0.21	1.612	0.002209
A_44_P154383	A_44_P154383	A_44_P154383		0.21	1.612	0.050828
A_42_P698188	Neo1	XM_343402	81735	0.21	1.611	0.004919
A_44_P322362	Map3k1	NM_053887	116667	0.21	1.611	0.004979
A_44_P466832	RGD1565950_predicted	XM_213548	287899	0.21	1.611	0.037413
A_44_P721908	AW534369	AW534369		0.21	1.611	0.108665
A_44_P960576	TC539128	TC539128		0.21	1.611	0.00535
A_42_P753803	Mta1	NM_022588	64520	0.21	1.611	0.009494
A_44_P1008284	TC554752	TC554752		0.21	1.610	0.026314
A_44_P131325	LOC498121	XM_573332		0.21	1.610	0.050841
A_44_P1040567	Nme2	NM_031833	83782	0.21	1.610	0.009369
A_43_P19497	Eps15	NM_001009424	313474	0.21	1.610	0.027465
A_44_P109648	Ctla4	NM_031674	63835	0.21	1.610	0.011457
A_44_P215775	LOC683708	XM_001067144		0.21	1.610	0.008121
A_44_P736091	LOC680426	XM_001057119	680426	0.21	1.610	0.013391
A_44_P442546	Prm1_mapped	NM_001002850	24685	0.21	1.610	0.015721
A_44_P326204	RGD1561676_predicted	XM_001079196		0.21	1.610	0.002036
A_44_P489956	Xrn2_predicted	XM_342535		0.21	1.610	0.042338
A_44_P1028211	Isg20l2	NM_001007741	361977	0.21	1.610	0.011011
A_44_P146893	Kif2	XM_345150	84391	0.21	1.610	0.016491
A_44_P378362	LOC680419	XM_001057090		0.21	1.609	0.007065
A_42_P484663	Calcoco1	NM_139190	246047	0.21	1.609	0.068683
A_44_P274316	Hif1an_predicted	XM_219961	309434	0.21	1.609	0.043206
A_44_P901239	TC562387	TC562387		0.21	1.609	0.06256
A_43_P19636	Paip1_predicted	XM_345160		0.21	1.608	0.007047
A_44_P915588	TC542077	TC542077		0.21	1.608	0.043925
A_43_P13648	Ipo11_predicted	XM_226752		0.21	1.608	0.010363
A_42_P780943	Acbd6	NM_001011906	289125	0.21	1.608	0.010416
A_44_P400507	Calu	NM_022535	64366	0.21	1.608	0.023909
A_44_P253268	LOC304035	XM_221603		0.21	1.608	0.00237
A_42_P652897	Tnfrsf6	NM_139194	246097	0.21	1.608	0.112431
A_42_P507857	Psmd9	NM_130430	161475	0.21	1.608	0.001377
A_44_P397736	Ube2s_predicted	XM_001071810		0.21	1.608	0.002653
A_43_P18475	Zfp499_predicted	XM_238764		0.21	1.608	0.00163
A_44_P255449	Col4a3bp_predicted	XM_345143		0.21	1.608	0.021238
A_44_P606085	RGD1560934_predicted	XM_575328		0.21	1.608	0.031688
A_44_P745749	A_44_P745749	A_44_P745749		0.21	1.608	0.152827
A_44_P419508	A_44_P419508	A_44_P419508		0.21	1.608	0.024743
A_44_P299500	Gmpr2	NM_001013036	192357	0.21	1.608	0.005392
A_44_P180168	Isrip	NM_175604	319113	0.21	1.608	0.004222
A_44_P234735	CB547952	CB547952	688018	0.21	1.607	0.002195
A_44_P1033359	A_44_P1033359	A_44_P1033359		0.21	1.607	0.00201
A_44_P293315	Bbs4_predicted	XM_217154		0.21	1.607	0.136445
A_44_P995374	LOC682888	XM_001063567		0.21	1.607	0.014693
A_44_P547107	BF286916	BF286916		0.21	1.607	0.09334
A_44_P186813	Rars_predicted	XM_213276		0.21	1.607	0.017394
A_44_P134917	AW142508	AW142508		0.21	1.607	0.022281
A_44_P120807	DY471696	DY471696		0.21	1.607	0.007961
A_44_P199077	Phgdhl1	NM_001034937	361094	0.21	1.607	0.012714
A_44_P595964	LOC686590	XM_001072973		0.21	1.606	0.004976
A_44_P130785	RGD1306193	NM_001024792	360636	0.21	1.606	0.003461
A_42_P490576	Cntrob_predicted	XM_220600	303240	0.21	1.606	0.018044
A_43_P21557	Ctnnbl1	NM_001024870	296320	0.21	1.606	0.002051
A_44_P532473	Traip_predicted	XM_345981		0.21	1.606	0.020254
A_43_P16043	Itga2	XM_345156	170921	0.21	1.606	0.003079
A_44_P549610	Bckdhb	XM_343439		0.21	1.606	0.004992
A_42_P626023	Cenpj_predicted	XM_224232		0.21	1.606	0.019536
A_42_P803153	Rpl27a_predicted	XM_215041		0.21	1.606	0.003796

A_44_P340079	A_44_P340079	A_44_P340079		0.21	1.605	0.010777
A_44_P971443	AW918781	AW918781		0.21	1.605	0.008449
A_44_P352113	RGD1306433_predicted	XM_343331		0.21	1.604	0.002721
A_44_P451638	Ppp1r9a	NM_053473	84685	0.21	1.604	0.010237
A_44_P133044	A_44_P133044	A_44_P133044		0.21	1.604	0.011631
A_43_P11560	Aqp5	NM_012779	25241	0.21	1.604	0.023155
A_44_P499377	RGD1309148_predicted	XM_216433	298147	0.21	1.604	0.006348
A_44_P870574	TC526052	TC526052		0.21	1.604	0.005306
A_44_P348012	RGD1565584_predicted	XM_214991		0.21	1.604	0.012374
A_44_P128202	M87788	M87788		0.21	1.604	0.172698
A_44_P141288	Rab34	NM_001012140	360571	0.21	1.604	0.133497
A_44_P841373	TC544524	TC544524		0.21	1.604	0.008476
A_44_P288556	Rbm34	NM_001014015	307956	0.21	1.604	0.001687
A_44_P823437	CO397607	CO397607		0.21	1.604	0.077471
A_43_P10724	Apip_predicted	XM_215785		0.21	1.604	0.006023
A_44_P393985	XM_233107	XM_233107		0.21	1.604	0.012591
A_44_P302728	AA875420	AA875420		0.21	1.604	0.003865
A_42_P462239	Acad9	XM_574921	294973	0.21	1.603	0.019156
A_42_P816767	Nt5c3_predicted	XM_231803		0.21	1.603	0.026212
A_44_P495675	AA850372	AA850372	305851	0.21	1.603	0.005157
A_43_P11050	BF555763	BF555763	307098	0.21	1.603	0.003725
A_43_P10874	MGC109519	NM_001024345	500450	0.20	1.603	0.066941
A_44_P147373	RGD1563180_predicted	XM_226249		0.20	1.603	0.000889
A_44_P1059334	Mtdh	NM_133398	170910	0.20	1.603	0.003597
A_44_P525817	Brd4	XM_343175	362844	0.20	1.603	0.002349
A_44_P344560	RGD1310358_predicted	XM_218532		0.20	1.603	0.005745
A_42_P820829	RGD1305890	NM_001014090	315594	0.20	1.603	0.03307
A_44_P541230	RGD1305475_predicted	XM_223828	305714	0.20	1.603	0.068388
A_44_P482588	Sost	NM_030584	80722	0.20	1.603	0.005573
A_42_P460152	Lrp6_predicted	XM_232466		0.20	1.603	0.018144
A_44_P791370	TC541070	TC541070		0.20	1.602	0.002656
A_44_P959443	Cbfa2t2_predicted	XM_215882	296293	0.20	1.602	0.017514
A_44_P187213	Ipo13	NM_053778	116458	0.20	1.602	0.005544
A_44_P864560	Lcp2	NM_130421	155918	0.20	1.602	0.078612
A_44_P515563	Gltscr1_predicted	XM_214817		0.20	1.602	0.041614
A_44_P242895	Hyou1	NM_138867	192235	0.20	1.602	0.004937
A_42_P752416	Ptk9l_predicted	XM_228196		0.20	1.602	0.009091
A_44_P241258	Sdfr2_predicted	XM_227622	310810	0.20	1.602	0.022258
A_44_P763294	TC528496	TC528496		0.20	1.602	0.271506
A_43_P20505	Bbx_predicted	XM_221497		0.20	1.602	0.015252
A_44_P115518	AW918130	AW918130		0.20	1.602	0.121124
A_44_P992592	Timm13	NM_145781	252928	0.20	1.602	0.001471
A_44_P1006334	Psma6	NM_017283	29673	0.20	1.602	0.00312
A_44_P149599	RGD1311558_predicted	XM_214734		0.20	1.602	0.013755
A_44_P759962	LOC365232	XM_001070056		0.20	1.601	0.006747
A_44_P325560	Apom	NM_019373	55939	0.20	1.601	0.014608
A_42_P772878	Bag3	NM_001011936	293524	0.20	1.601	0.02408
A_44_P1022634	RGD1309308_predicted	XM_232850	313115	0.20	1.601	0.068104
A_44_P291862	Srpk1	BC090554		0.20	1.601	0.012271
A_44_P821956	LOC366611	XR_009195	366611	0.20	1.601	0.025985
A_42_P582467	Podxl	NM_138848	192181	0.20	1.601	0.127557
A_44_P576764	RGD1564719_predicted	XM_001079729		0.20	1.601	0.004311
A_44_P760291	ENSRNOT00000040155	ENSRNOT00000040155		0.20	1.601	0.012207
A_44_P314735	AI137547	AI137547		0.20	1.601	0.002274
A_44_P417179	Zfp367	NM_001012051	306695	0.20	1.600	0.006544
A_44_P575340	Hprt	NM_012583	24465	0.20	1.600	0.099602
A_44_P535713	Magoh_predicted	XM_216485	298385	0.20	1.600	0.016931
A_44_P194256	Gstt2	NM_012796	29487	0.20	1.600	0.004692
A_44_P790861	A_44_P790861	A_44_P790861		0.20	1.600	0.007083
A_44_P991045	RGD1359460	NM_001006959	289562	0.20	1.599	0.076848
A_44_P1030669	Bin1	NM_053959	117028	0.20	1.599	0.001565
A_44_P560819	Chd6_predicted	XM_230814		0.20	1.599	0.067512
A_44_P533172	RGD1565589_predicted	XM_001062709		0.20	1.599	0.069677

A_44_P788404	BG670091	BG670091		0.20	1.599	0.226993
A_44_P217841	RGD1565245_predicted	XM_344527		0.20	1.599	0.071258
A_44_P396546	Hspa8	NM_024351	24468	0.20	1.599	0.015852
A_42_P732189	Rpl15	NM_139114	245981	0.20	1.599	0.001604
A_44_P482749	XM_223037	XM_223037		0.20	1.598	0.006152
A_43_P18491	Map2k7	NM_001025425	363855	0.20	1.598	0.002502
A_43_P14285	Statip1	NM_001034145	307545	0.20	1.598	0.006612
A_44_P303818	DV726713	DV726713		0.20	1.598	0.016519
A_44_P328489	RGD1307374_predicted	XM_214609	291736	0.20	1.598	0.011245
A_44_P342279	Adamts3_predicted	XM_223279		0.20	1.597	0.054549
A_44_P274785	RGD1310922_predicted	XM_213260	287170	0.20	1.597	0.027761
A_44_P1009723	Fau	NM_001012739	29752	0.20	1.597	0.008381
A_43_P16419	XM_236784	XM_236784		0.20	1.597	0.009059
A_44_P870123	TC554509	TC554509		0.20	1.597	0.005076
A_44_P358818	XM_229953	XM_229953		0.20	1.597	0.019489
A_44_P1044934	Anapc5_predicted	XM_213783		0.20	1.597	0.001055
A_43_P17473	Bcl9_predicted	XM_241475		0.20	1.597	0.002267
A_44_P361741	40063	NM_022616	64551	0.20	1.597	0.008509
A_44_P808057	Lrrc8	NM_001024782	311846	0.20	1.596	0.002629
A_44_P528706	AW141558	AW141558		0.20	1.596	0.004954
A_44_P215191	Aprt_predicted	NM_001013061	292072	0.20	1.596	0.00902
A_44_P821972	A_44_P821972	A_44_P821972		0.20	1.596	0.001764
A_44_P761386	TC521288	TC521288		0.20	1.596	0.006892
A_44_P347070	RGD1310251	NM_001024240	291675	0.20	1.596	0.054686
A_44_P138838	Kb9	NM_001008805	407757	0.20	1.595	0.020033
A_44_P466023	Cenpc1	NM_001004098	305270	0.20	1.595	0.046332
A_44_P517884	Hist2h4_predicted	XM_227462		0.20	1.595	0.032641
A_44_P229440	RGD1308297	XM_224510		0.20	1.595	0.010828
A_44_P344562	XM_212869	XM_212869		0.20	1.595	0.002052
A_43_P18111	Exdl2_predicted	XM_343086		0.20	1.594	0.020129
A_44_P259125	Nkiras1_predicted	XM_223837		0.20	1.594	0.025415
A_44_P440953	A_44_P440953	A_44_P440953		0.20	1.594	0.208916
A_44_P342416	XM_344565	XM_344565		0.20	1.594	0.00381
A_42_P815235	RGD1306157_predicted	XM_214662	291947	0.20	1.594	0.002021
A_44_P699224	LOC691534	XM_001078710	691534	0.20	1.594	0.001978
A_42_P559343	PNAS-4	NM_001013873	289277	0.20	1.594	0.011703
A_44_P506635	Ifrd2_predicted	XM_217254		0.20	1.594	0.001722
A_44_P746275	DY309001	DY309001		0.20	1.594	0.040532
A_44_P745100	RGD1566242_predicted	XM_001077768		0.20	1.594	0.012423
A_44_P976775	TC543536	TC543536		0.20	1.594	0.029126
A_44_P621013	Olfml1	NM_001013192	361621	0.20	1.593	0.07395
A_44_P407736	RGD1304890	XM_001057949		0.20	1.593	0.021512
A_44_P278562	Bclaf1	XM_001073455		0.20	1.593	0.11653
A_44_P778660	TC546204	TC546204		0.20	1.593	0.173278
A_44_P183007	A_44_P183007	A_44_P183007		0.20	1.593	0.010387
A_44_P454523	ENSRNOT00000044525	ENSRNOT00000044525		0.20	1.593	0.106017
A_44_P919954	BP465716	BP465716	680700	0.20	1.593	0.011921
A_44_P168132	Dd5	XM_576252	117060	0.20	1.592	0.011111
A_44_P201583	LOC690183	XM_001073579		0.20	1.592	0.008978
A_44_P839332	Plscr4	XM_576448		0.20	1.592	0.138762
A_43_P17909	RGD1559547_predicted	XM_214011		0.20	1.592	0.003688
A_44_P567329	AA900868	AA900868		0.20	1.592	0.037671
A_42_P805088	RGD1560813_predicted	XM_574181	498894	0.20	1.592	0.004347
A_44_P239009	RGD1559871_predicted	XM_575487	500135	0.20	1.592	0.008381
A_44_P259719	Ppp1ca	NM_031527	24668	0.20	1.592	0.001394
A_43_P15562	Metap2	NM_022539	64370	0.20	1.592	0.020948
A_44_P513738	Hmg20a_predicted	XM_236267		0.20	1.591	0.011884
A_44_P974399	RGD1559938_predicted	XM_573498		0.20	1.591	0.00712
A_44_P553939	Fyttd1	XM_001081958		0.20	1.591	0.047632
A_43_P11306	RGD1311474	NM_001010945	288591	0.20	1.591	0.003239
A_44_P869382	TC541426	TC541426		0.20	1.591	0.057122
A_43_P19988	RGD1559610_predicted	XM_233498		0.20	1.591	0.005746
A_44_P334891	Rcl1	NM_001013152	309301	0.20	1.590	0.018001

A_44_P238722	Bmp2k	XM_573559	498333	0.20	1.590	0.018628
A_44_P485614	ENSRNOT00000029749	ENSRNOT00000029749		0.20	1.590	0.004447
A_44_P537024	RGD1560410_predicted	XM_343272		0.20	1.590	0.039025
A_44_P730928	TC522506	TC522506		0.20	1.590	0.02227
A_44_P698554	Hist2h3c2_predicted	XM_001062251		0.20	1.590	0.090571
A_44_P323971	Ptch1_predicted	XM_345570		0.20	1.590	0.015062
A_44_P340884	Polr2a_mapped	XM_343922	363633	0.20	1.590	0.009343
A_44_P495101	CB545196	CB545196	29408	0.20	1.590	0.021238
A_44_P121390	Phf10	NM_001024747	292404	0.20	1.590	0.004027
A_44_P491998	RGD1564396_predicted	XM_221917	288486	0.20	1.590	0.013731
A_44_P1047615	Lsm14a_predicted	XM_341841	361554	0.20	1.589	0.00245
A_44_P182485	Sorbs3	NM_001005762	282843	0.20	1.589	0.011377
A_44_P559080	CF108752	CF108752		0.20	1.589	0.412067
A_44_P1025102	Plscr1	NM_057194	117540	0.20	1.589	0.005442
A_44_P389564	A_44_P389564	A_44_P389564		0.20	1.589	0.082652
A_42_P612834	Tmem93_predicted	XM_213394		0.20	1.589	0.057538
A_44_P432733	RGD1306064_predicted	XM_224581	306229	0.20	1.589	0.067152
A_44_P748551	TC536024	TC536024		0.20	1.589	0.025911
A_44_P121177	Tpm3	NM_057208	117557	0.20	1.589	0.022082
A_44_P319297	XM_343876	XM_343876		0.20	1.589	0.00445
A_44_P424552	Plscr4	NM_001012000	300900	0.20	1.589	0.096329
A_44_P1026139	XM_214487	XM_214487		0.20	1.589	0.088387
A_44_P363847	XM_220163	XM_220163		0.20	1.589	0.005627
A_44_P375380	LOC679713	XM_001054156	679713	0.20	1.589	0.006612
A_44_P527238	Slc25a25	NM_145677	246771	0.20	1.589	0.041868
A_44_P260384	RGD1307915_predicted	XM_240915		0.20	1.588	0.037833
A_44_P391806	Tmem32_predicted	XM_229191		0.20	1.588	0.037064
A_44_P810190	LOC686980	XM_001076594		0.20	1.588	0.010298
A_44_P344664	RGD1309922_predicted	XM_224329	306007	0.20	1.588	0.00245
A_44_P281889	Ptms	NM_031975	83801	0.20	1.588	0.005373
A_44_P667745	LOC684899	XM_001072379		0.20	1.588	0.091694
A_44_P422006	XM_344715	XM_344715		0.20	1.588	0.004503
A_44_P899318	TC517749	TC517749		0.20	1.588	0.016414
A_44_P455108	LOC678808	XM_001053247		0.20	1.587	0.003616
A_44_P293363	LOC367196	XM_346003	367196	0.20	1.587	0.070052
A_42_P579799	RGD1560155_predicted	XM_225523		0.20	1.587	0.097369
A_42_P528248	Wdfy3_predicted	XM_223196	305164	0.20	1.587	0.034814
A_44_P396165	RGD1306215_predicted	XM_216002		0.20	1.587	0.008141
A_44_P147771	AA955624	AA955624		0.20	1.587	0.014282
A_44_P170884	LOC289943	XR_008381	289943	0.20	1.587	0.076004
A_44_P229552	Evx2_predicted	XM_221512		0.20	1.587	0.088181
A_44_P384833	Mum1_predicted	XM_343166		0.20	1.586	0.06041
A_44_P457702	RGD1308513	NM_001014231	364050	0.20	1.586	0.015782
A_44_P540910	Casp6	NM_031775	83584	0.20	1.586	0.009706
A_44_P1044092	LOC690229	XM_001076401		0.20	1.586	0.010634
A_44_P384919	XM_340811	XM_340811		0.20	1.586	0.055118
A_44_P437327	Sesn3_predicted	XM_235825		0.20	1.585	0.008651
A_44_P254466	BF285222	BF285222		0.20	1.585	0.167581
A_44_P478604	LOC680266	XM_001057185	680266	0.20	1.585	0.00521
A_44_P560432	LOC366365	XR_008762	366365	0.20	1.585	0.014809
A_44_P533531	AI406723	AI406723	313668	0.20	1.585	0.034941
A_44_P589372	AW142520	AW142520		0.20	1.585	0.015678
A_44_P384438	RGD1563446_predicted	XM_342166		0.20	1.585	0.020464
A_44_P286837	RGD1566309_predicted	XM_576906		0.20	1.585	0.01964
A_44_P472271	Rgl1_predicted	XM_222717		0.20	1.585	0.005185
A_44_P814386	TC543302	TC543302		0.20	1.585	0.022673
A_44_P696677	AW142892	AW142892		0.20	1.585	0.035553
A_44_P302313	Fnbp4	XM_230291	311183	0.20	1.584	0.005809
A_43_P17047	LOC688429	XM_001065920		0.20	1.584	0.076008
A_44_P532436	RGD1564821_predicted	XM_343194	362865	0.20	1.584	0.08383
A_44_P366434	MGC94207	NM_001007751	362946	0.20	1.584	0.003929
A_44_P793591	TC538123	TC538123		0.20	1.584	0.005667
A_44_P448223	RGD1563958_predicted	XM_236007		0.20	1.584	0.004109

A_44_P105229	Nnt_mapped	NM_001013157	310378	0.20	1.584	0.050832
A_44_P541165	RGD1306614	NM_001034911	293454	0.20	1.584	0.015994
A_44_P1039809	Rasip1_predicted	XM_214916		0.20	1.584	0.016255
A_44_P269930	RGD1310174_predicted	XM_216525		0.20	1.584	0.003056
A_44_P930467	TC557832	TC557832		0.20	1.584	0.097452
A_44_P478917	Snrpd1_predicted	XM_214621		0.20	1.584	0.010073
A_44_P195881	AW144129	AW144129		0.20	1.584	0.013002
A_44_P714847	TC521502	TC521502		0.20	1.584	0.051955
A_42_P815485	RGD1306583	NM_001013908	294709	0.20	1.583	0.012992
A_44_P156317	Dad1	NM_138910	192275	0.20	1.583	0.003491
A_44_P525229	Anxa8	NM_001031654	306283	0.20	1.583	0.030852
A_44_P896107	LOC498404	NM_001017502	498404	0.20	1.583	0.008695
A_44_P424396	LOC312777	NM_001037353	312777	0.20	1.583	0.005482
A_44_P1010500	Ppgb	NM_001011959	296370	0.20	1.583	0.015829
A_44_P182386	Sec61g	XM_346040		0.20	1.583	0.001079
A_43_P15801	Lancl1	NM_053723	114515	0.20	1.582	0.019345
A_44_P495542	Plagl2_predicted	XM_230745		0.20	1.582	0.009107
A_44_P125761	Olr7_predicted	NM_001000768	405043	0.20	1.582	0.22061
A_44_P424164	RGD1565956_predicted	XM_217582		0.20	1.582	0.002685
A_44_P278602	Ap3m1	NM_133593	171126	0.20	1.582	0.015867
A_44_P514058	AI013816	AI013816	690285	0.20	1.582	0.027214
A_44_P483334	BU759069	BU759069		0.20	1.582	0.002547
A_44_P546338	Loxl1	NM_001012125	315714	0.20	1.582	0.005402
A_43_P11215	Pdcd5_predicted	XM_214911		0.20	1.582	0.001675
A_44_P154785	XM_340981	XM_340981		0.20	1.582	0.002465
A_43_P17880	Nek6	NM_182953	360161	0.20	1.582	0.005011
A_44_P766713	BF398791	BF398791		0.20	1.582	0.007209
A_44_P542933	Zfp36l1	NM_017172	29344	0.20	1.582	0.00296
A_42_P835252	Timm9	XM_001072892	171139	0.20	1.582	0.002007
A_42_P835787	Zfp358_predicted	XM_341026		0.20	1.582	0.003959
A_44_P512799	Grik5	NM_017262	24407	0.20	1.581	0.009637
A_43_P14686	Ubap2_predicted	XM_232901		0.20	1.581	0.009509
A_44_P236930	CB606450	CB606450	360658	0.20	1.581	0.004799
A_44_P467269	Ccnf	XM_340763		0.20	1.581	0.009411
A_43_P15859	Ocm	NM_012995	25503	0.20	1.581	0.216964
A_44_P244662	LOC691145	XM_001076990	691145	0.20	1.581	0.029302
A_44_P342020	Shc1	NM_053517	85385	0.20	1.581	0.012464
A_44_P278439	Ube2n	NM_053928	116725	0.20	1.580	0.00558
A_43_P18603	Nek4	XM_224610	306252	0.20	1.580	0.02825
A_44_P341133	RGD1564930_predicted	XM_001054328		0.20	1.580	0.005187
A_44_P212424	RGD1560212_predicted	XM_574178		0.20	1.580	0.002069
A_43_P14199	TC562565	TC562565		0.20	1.580	0.019747
A_44_P383411	Psma3	NM_017280	29670	0.20	1.580	0.005427
A_44_P852603	ENSRNOT00000033714	ENSRNOT00000033714		0.20	1.580	0.010644
A_44_P225239	RGD1561808_predicted	XM_345962		0.20	1.580	0.002711
A_44_P769974	A_44_P769974	A_44_P769974		0.20	1.579	0.004638
A_44_P484779	Utrn	NM_013070	25600	0.20	1.579	0.013095
A_44_P313652	RT1-Aw2	M10094	24737	0.20	1.579	0.002719
A_44_P1000389	Eprs	NM_001024238	289352	0.20	1.579	0.003449
A_44_P871726	RGD1359435	XM_001074993		0.20	1.579	0.11383
A_44_P853775	LOC691221	XM_001077260	691221	0.20	1.579	0.016859
A_44_P638867	TC559866	TC559866		0.20	1.579	0.026046
A_44_P1006090	Spg20	XM_215564		0.20	1.579	0.00604
A_44_P506064	Aldh1l2_predicted	XM_235005	299699	0.20	1.579	0.017751
A_43_P21345	Tbc1d14	NM_001012152	360956	0.20	1.579	0.067378
A_44_P255397	Cars_predicted	XM_215134		0.20	1.579	0.00497
A_44_P248947	Pole4_predicted	XM_342710		0.20	1.579	0.00589
A_44_P410676	Stk11_predicted	XM_234900		0.20	1.578	0.012114
A_44_P498642	Zfp455	NM_173314	286979	0.20	1.578	0.021426
A_44_P522336	Nup88	NM_053616	113929	0.20	1.578	0.005538
A_44_P653193	LOC687424	XM_001076559		0.20	1.578	0.036365
A_42_P828695	RGD1307506_predicted	XM_215486		0.20	1.578	0.272928
A_44_P433334	Tra1_predicted	NM_001012197	362862	0.20	1.578	0.011935

A_44_P449734	Thoc1	XM_229013		0.20	1.578	0.010621
A_44_P752074	LOC309957	NM_001014035	309957	0.20	1.578	0.014959
A_44_P414775	Cno	XM_344255	364183	0.20	1.578	0.001412
A_44_P516073	RGD1310552_predicted	XM_217194		0.20	1.578	0.116666
A_44_P745658	TC538748	TC538748		0.20	1.578	0.022484
A_44_P119442	XM_216492	XM_216492		0.20	1.577	0.021349
A_44_P181192	AI170446	AI170446		0.20	1.577	0.012208
A_44_P525461	RGD1566108_predicted	XM_217627	302810	0.20	1.577	0.045826
A_43_P20053	Fut8	NM_001002289	432392	0.20	1.577	0.008902
A_44_P842474	TC533942	TC533942		0.20	1.577	0.142888
A_44_P480353	XM_341846	XM_341846		0.20	1.577	0.015873
A_44_P203746	XM_344416	XM_344416		0.20	1.577	0.001658
A_44_P255985	RGD1310128_predicted	XM_214417		0.20	1.577	0.006807
A_44_P557921	XM_342106	XM_342106		0.20	1.577	0.014504
A_44_P272353	LOC367117	XM_345956		0.20	1.576	0.009752
A_44_P530697	RGD1565575_predicted	XM_342400	362096	0.20	1.576	0.005831
A_44_P1056489	St13	NM_031122	81800	0.20	1.576	0.005267
A_44_P459322	XM_219600	XM_219600		0.20	1.576	0.043057
A_44_P793558	TC562671	TC562671		0.20	1.576	0.038554
A_44_P728719	LOC498176	NM_001017487	498176	0.20	1.576	0.006413
A_44_P137815	Pwwp2_predicted	XM_341955		0.20	1.576	0.010493
A_44_P214040	B2m	NM_012512	24223	0.20	1.575	0.160755
A_44_P578687	TC531204	TC531204		0.20	1.575	0.005084
A_44_P913689	A_44_P913689	A_44_P913689		0.20	1.575	0.002339
A_44_P445687	Anks1_predicted	XM_228027		0.20	1.575	0.016779
A_44_P451916	Tap1	NM_032055	24811	0.20	1.575	0.049449
A_44_P114869	Ftl1	NM_022500	29292	0.20	1.575	0.017247
A_44_P697057	Arhgap1_predicted	XM_001078147		0.20	1.575	0.007176
A_44_P324034	RGD1308472_predicted	XM_235942	315484	0.20	1.575	0.081251
A_44_P107605	A_44_P107605	A_44_P107605		0.20	1.575	0.001986
A_44_P162583	Rac1	CF110052	363875	0.20	1.574	0.009198
A_44_P495383	LOC682182	XM_001060304		0.20	1.574	0.019037
A_43_P10264	Sf3b2_predicted	XM_215182		0.20	1.574	0.009292
A_43_P22864	XM_216497	XM_216497		0.20	1.574	0.007488
A_44_P1071100	LOC499120	XM_574414	499120	0.20	1.574	0.033539
A_42_P715019	RGD1311849_predicted	XM_233144	313346	0.20	1.574	0.010036
A_42_P654627	Prpf8	XM_213385	287530	0.20	1.574	0.007388
A_43_P21628	Ddx23_predicted	XM_217050		0.20	1.574	0.006186
A_44_P527680	Otud6b_predicted	XM_232795		0.20	1.573	0.065764
A_43_P14552	XM_343422	XM_343422		0.20	1.573	0.002583
A_44_P321544	Slc41a2_predicted	XM_343191		0.20	1.573	0.007386
A_44_P234270	RGD1309730_predicted	XM_215788		0.20	1.573	0.156424
A_44_P344724	XM_240449	XM_240449		0.20	1.573	0.238125
A_43_P18752	Rbm4_predicted	XM_215201	170900	0.20	1.573	0.008879
A_44_P224326	Rnf38	NM_134467	171501	0.20	1.573	0.004203
A_44_P668137	TC551673	TC551673		0.20	1.573	0.003615
A_44_P410080	RGD1563620_predicted	XM_227252		0.20	1.572	0.010694
A_44_P264880	A_44_P264880	A_44_P264880		0.20	1.572	0.006802
A_44_P209604	Mtmr3	NM_001012038	305482	0.20	1.572	0.023336
A_44_P943876	LOC499885	NM_001025043	499885	0.20	1.572	0.010745
A_44_P278770	Sart3_predicted	XM_222279		0.20	1.572	0.017228
A_44_P127802	RGD1309570	NM_001039455	361828	0.20	1.572	0.005989
A_44_P1003117	Mcm3ap_predicted	XM_215376		0.20	1.571	0.00336
A_44_P205902	Rpl11	NM_001025739	362631	0.20	1.571	0.004556
A_44_P989639	RGD1565655_predicted	XM_574388	499097	0.20	1.571	0.125707
A_44_P572015	AW141996	AW141996		0.20	1.571	0.001764
A_44_P501566	Fem1c_predicted	XM_228396		0.20	1.571	0.002569
A_44_P1045805	Taf11	NM_001008350	309638	0.20	1.571	0.006005
A_44_P114491	RGD1559750_predicted	XM_227632		0.20	1.571	0.003525
A_44_P281352	Dnajb4	NM_001013076	295549	0.20	1.571	0.03751
A_44_P368365	RGD1562139_predicted	XM_235395		0.20	1.570	0.003201
A_44_P356125	Sap2	XM_342209	266808	0.20	1.570	0.042661
A_44_P128902	LOC497867	XM_573047	497867	0.20	1.570	0.004017

A_42_P824217	Dapp1_predicted	XM_342348		0.20	1.570	0.018339
A_44_P1051765	Thap3_predicted	XM_342984		0.20	1.569	0.008398
A_42_P823777	RGD1561004_predicted	XM_001057408		0.20	1.569	0.029017
A_44_P614304	Fem1c_predicted	XM_228396		0.20	1.569	0.007635
A_43_P15259	Tnfrsf1a	NM_013091	25625	0.20	1.569	0.008731
A_44_P148720	Vac14	NM_177930	307842	0.20	1.569	0.001768
A_44_P744765	ENSRNOT00000037986	ENSRNOT00000037986		0.20	1.569	0.00597
A_44_P503860	RGD1560755_predicted	XM_001062341		0.20	1.569	0.008171
A_44_P536442	Phb	NM_031851	25344	0.20	1.569	0.003784
A_42_P467956	RGD1562244_predicted	XM_230024	295678	0.20	1.569	0.01638
A_42_P666771	Ptprg	NM_134356	171357	0.20	1.569	0.016802
A_44_P466281	Zbtb37_predicted	XM_222803		0.20	1.568	0.067174
A_44_P1021165	Trim41_predicted	XM_220357	303088	0.20	1.568	0.001752
A_44_P207549	AW915718	AW915718	25460	0.20	1.568	0.021426
A_44_P742369	AI136377	AI136377		0.20	1.568	0.012699
A_42_P806899	Jmjd3_predicted	XM_343919		0.20	1.568	0.003969
A_43_P22751	Mtf2	XM_341180	360905	0.20	1.568	0.006991
A_42_P667782	RGD1307883	NM_001009673	301463	0.20	1.568	0.001557
A_44_P109791	Pja2	NM_138896	192256	0.20	1.568	0.010934
A_44_P137862	Rai16_predicted	XM_224339	306015	0.20	1.568	0.021857
A_42_P751368	RGD1304704	XM_217550		0.20	1.568	0.002383
A_44_P426966	RGD1310727_predicted	XM_001071459		0.20	1.568	0.011292
A_44_P241324	Rbm22	NM_001025676	307410	0.20	1.568	0.008142
A_44_P541223	RGD1305475_predicted	XM_223828	305714	0.20	1.568	0.00844
A_44_P485592	Mrps9	XM_217388	301371	0.20	1.567	0.025544
A_44_P505180	Kif5b	NM_057202	117550	0.20	1.567	0.013076
A_44_P255570	LOC680806	XM_001058968	680806	0.20	1.567	0.008474
A_44_P402873	Perq1_predicted	XM_001069187		0.20	1.567	0.002372
A_44_P161343	Tnrc6_predicted	XM_219297		0.20	1.567	0.008755
A_44_P204956	LOC366515	NM_001014262	366515	0.20	1.567	0.002593
A_44_P193210	LOC678860	XM_001053666		0.20	1.567	0.049624
A_44_P234011	Pxn	NM_001012147	360820	0.20	1.567	0.010928
A_44_P991787	Ankrd46	NM_001013948	299982	0.20	1.567	0.025969
A_42_P648800	Fzd7_predicted	XM_237191	301440	0.20	1.567	0.006792
A_43_P15713	Hnrpl	XM_001068144	80846	0.19	1.567	0.002802
A_44_P104250	Mrps14_predicted	XM_213906		0.19	1.566	0.031364
A_44_P1059463	Fubp1	NM_001037653	654496	0.19	1.566	0.007179
A_44_P560036	A_44_P560036	A_44_P560036		0.19	1.566	0.001501
A_43_P19603	Zc3h7b_predicted	XM_243630	315158	0.19	1.566	0.001935
A_44_P200463	Waspip	NM_057192	117538	0.19	1.566	0.007454
A_44_P270700	BG663460	BG663460		0.19	1.565	0.083545
A_44_P836576	Ptk2	NM_013081	25614	0.19	1.565	0.016301
A_44_P154355	Rfx5_predicted	XM_227426		0.19	1.565	0.003567
A_44_P448259	RGD1562153_predicted	XM_576285		0.19	1.565	0.003434
A_44_P484826	Gdi2	NM_017276	29662	0.19	1.565	0.070282
A_44_P503082	Tcfe2a	NM_133524	171046	0.19	1.565	0.0037
A_44_P324906	AW142380	AW142380	367153	0.19	1.565	0.072147
A_44_P295151	Flnb_predicted	XM_224561		0.19	1.565	0.027906
A_44_P184955	RGD1307366_predicted	XM_001066026		0.19	1.565	0.018923
A_44_P338680	Cltb	NM_053835	116561	0.19	1.564	0.001019
A_44_P138932	AI112752	AI112752		0.19	1.564	0.033262
A_44_P467057	RGD1307688	NM_001014188	362394	0.19	1.564	0.001764
A_44_P328136	Mx1	NM_173096	24575	0.19	1.564	0.171571
A_44_P103028	XM_230930	XM_230930		0.19	1.564	0.001413
A_44_P102774	Polr3e_predicted	XM_341919		0.19	1.564	0.005943
A_44_P304402	Tpp2	NM_031137	81815	0.19	1.564	0.004457
A_44_P171183	MGC112775	XM_575887		0.19	1.564	0.011772
A_44_P159365	RGD1310453_predicted	XM_001060264		0.19	1.564	0.024254
A_44_P825195	TC551373	TC551373		0.19	1.564	0.004768
A_44_P532071	LOC679113	XM_001054752		0.19	1.564	0.071875
A_44_P489839	Cnot7_predicted	XM_001058225		0.19	1.564	0.013176
A_44_P903850	AY724476	AY724476	365865	0.19	1.564	0.01059
A_44_P210736	XM_224238	XM_224238		0.19	1.563	0.01622

A_44_P539738	Unc13b	NM_022862	64830	0.19	1.563	0.019723
A_44_P955967	AI176314	AI176314		0.19	1.563	0.029971
A_44_P464089	Prcc_predicted	XM_227476	310687	0.19	1.563	0.132709
A_44_P105591	RGD1306886_predicted	XM_234946		0.19	1.563	0.043916
A_44_P1046471	MGC94954	NM_001004243	300189	0.19	1.563	0.051262
A_44_P1028549	Casp4	NM_053736	114555	0.19	1.563	0.026632
A_42_P828934	Sf3b4	NM_001011951	295270	0.19	1.563	0.028203
A_44_P241079	RGD1565762_predicted	XM_232701		0.19	1.563	0.001718
A_42_P538334	RGD1307583_predicted	XM_341397	361111	0.19	1.563	0.005169
A_42_P624717	LOC682469	XM_001061636		0.19	1.563	0.100263
A_44_P922030	TC552083	TC552083		0.19	1.563	0.001525
A_43_P15013	TC559957	TC559957		0.19	1.562	0.030299
A_44_P386374	Mapk1	NM_053842	116590	0.19	1.562	0.020957
A_43_P11075	RGD1563952_predicted	XM_343782	363463	0.19	1.562	0.018783
A_43_P17799	Mtx2	NM_001008286	288150	0.19	1.562	0.016044
A_44_P245305	Dld	NM_199385	298942	0.19	1.562	0.044551
A_44_P231497	Ddx1	NM_053414	84474	0.19	1.562	0.014995
A_44_P330931	Prpf4	XM_233022		0.19	1.562	0.00461
A_44_P556146	Pms2_predicted	XM_213712		0.19	1.562	0.004039
A_44_P103296	TC524308	TC524308		0.19	1.562	0.034846
A_42_P655728	XM_234925	XM_234925		0.19	1.562	0.00269
A_44_P589702	Clcn6_predicted	XM_001074014		0.19	1.562	0.017384
A_44_P197064	Tmem63c_predicted	XM_234431		0.19	1.562	0.060984
A_44_P859569	BE097232	BE097232		0.19	1.562	0.042786
A_44_P369701	Ei24	NM_001025660	300514	0.19	1.561	0.027323
A_43_P11350	Nt5dc2	NM_001009271	290558	0.19	1.561	0.008355
A_44_P339739	BX883043	BX883043		0.19	1.561	0.005541
A_44_P110445	Sap30bp_predicted	XM_340938		0.19	1.561	0.065827
A_42_P473302	DV728597	DV728597		0.19	1.561	0.00572
A_44_P180215	LOC498171	XM_573387		0.19	1.561	0.010366
A_44_P206170	Phf2_predicted	XM_225206		0.19	1.561	0.010454
A_42_P685418	Rbm8_predicted	XM_215637	295284	0.19	1.560	0.00322
A_42_P514798	Zcchc11_predicted	XM_233345		0.19	1.560	0.20787
A_44_P370617	Atf7ip_predicted	XM_232488		0.19	1.560	0.002809
A_44_P149248	LOC691222	XM_001077261	691222	0.19	1.560	0.013195
A_42_P531684	LOC363198	NM_001014215	363198	0.19	1.560	0.034783
A_44_P463858	Bhlhb3	NM_133303	117095	0.19	1.560	0.373483
A_43_P13007	Rps15a	NM_053982	117053	0.19	1.560	0.015963
A_42_P769766	RGD1310899_predicted	XM_216747		0.19	1.559	0.002479
A_44_P944375	ENSRNOT00000041388	ENSRNOT00000041388		0.19	1.559	0.028593
A_44_P548730	Bcl9l_predicted	XM_217124		0.19	1.559	0.003332
A_43_P11246	Eif4a1	NM_199372	287436	0.19	1.559	0.005428
A_44_P902140	TC562851	TC562851		0.19	1.559	0.018696
A_44_P363471	Chmp7_predicted	XM_344430		0.19	1.559	0.003098
A_44_P480786	TC552207	TC552207		0.19	1.559	0.002842
A_44_P208091	Zfp622	NM_001009652	294846	0.19	1.558	0.012857
A_44_P114788	Adamts8_predicted	XM_235977		0.19	1.558	0.091868
A_44_P238939	XM_345411	XM_345411		0.19	1.558	0.002069
A_44_P294753	RGD621098	NM_139040	207123	0.19	1.558	0.018455
A_44_P399044	LOC685491	XM_001061719	313048	0.19	1.558	0.007991
A_44_P503579	Rpl17	NM_201415	291434	0.19	1.558	0.00429
A_44_P347023	RGD1565742_predicted	XM_227709		0.19	1.558	0.08116
A_44_P775708	RGD1560584_predicted	XM_575506		0.19	1.558	0.00475
A_44_P166310	Nxt2_predicted	XM_235755		0.19	1.558	0.053653
A_44_P869988	TC543503	TC543503		0.19	1.558	0.149826
A_44_P222679	MGC124888	NM_001033891	297387	0.19	1.558	0.007293
A_44_P264379	Phax	NM_173133	286917	0.19	1.558	0.015368
A_44_P225353	RGD1311458	NM_001009678	303948	0.19	1.557	0.00462
A_44_P621925	TC551904	TC551904		0.19	1.557	0.001389
A_43_P21650	Adpgk	XM_236306	315722	0.19	1.557	0.019083
A_44_P200507	Khdrbs1	NM_130405	117268	0.19	1.557	0.016033
A_44_P1005881	Arhgdia	NM_001007005	360678	0.19	1.557	0.002694
A_44_P400195	Atp5c1	NM_053825	116550	0.19	1.557	0.061466

A_44_P123102	LOC294154	NM_001039607	294154	0.19	1.557	0.017328
A_43_P19515	Josd3	NM_001014207	363017	0.19	1.557	0.007794
A_44_P194172	Ptpn4	XM_341109	246116	0.19	1.557	0.041801
A_43_P10219	RGD1308014_predicted	XM_223075	289382	0.19	1.556	0.012333
A_43_P13440	CB545469	CB545469	83841	0.19	1.556	0.029126
A_44_P336951	LOC503175	XM_578699	503175	0.19	1.556	0.004696
A_43_P16512	RGD1311021_predicted	XM_218824		0.19	1.556	0.021261
A_44_P131031	XM_212807	XM_212807		0.19	1.556	0.044458
A_44_P821788	ENSRNOT00000037340	ENSRNOT00000037340		0.19	1.556	0.02516
A_43_P18033	LOC306766	NM_001014007	306766	0.19	1.555	0.009346
A_44_P318722	XM_229566	XM_229566		0.19	1.555	0.016654
A_44_P451869	Sumo2	NM_133594	171127	0.19	1.555	0.001041
A_44_P466907	BQ203443	BQ203443	303702	0.19	1.555	0.084109
A_44_P250467	Cd22_predicted	XM_218523		0.19	1.555	0.017233
A_42_P806126	Ixl_predicted	XM_214868		0.19	1.555	0.005142
A_43_P16145	Otof	XM_001062291	84573	0.19	1.555	0.028919
A_44_P511491	Etv4_predicted	XM_340910		0.19	1.555	0.062346
A_43_P10353	Eif4g1	XM_001060756		0.19	1.555	0.004741
A_44_P471279	RGD1310427_predicted	XM_342959		0.19	1.554	0.009835
A_43_P13670	Tmem101	XM_220940	303564	0.19	1.554	0.008061
A_42_P630663	RGD1308126_predicted	XM_213563		0.19	1.554	0.019496
A_43_P17681	Ptdsr	NM_001012143	360665	0.19	1.554	0.008793
A_43_P14669	S100a13_predicted	XM_215607	295213	0.19	1.554	0.015884
A_44_P232508	AW917502	AW917502		0.19	1.554	0.009751
A_43_P21256	Rev3l	XM_228273		0.19	1.554	0.070156
A_44_P459249	Srp72_predicted	XM_214017		0.19	1.553	0.005732
A_44_P884119	LOC312863	NM_001014061	312863	0.19	1.553	0.026459
A_44_P318029	LOC689959	XM_001065356		0.19	1.553	0.003664
A_44_P443349	RGD1306195	NM_001014062	313163	0.19	1.553	0.00572
A_42_P508722	AA964747	AA964747		0.19	1.553	0.075817
A_44_P1040187	RGD1307789	NM_001010947	288912	0.19	1.553	0.001181
A_44_P287089	Gmfb	NM_031032	81661	0.19	1.553	0.002128
A_44_P667865	Ptk2	NM_013081	25614	0.19	1.553	0.028951
A_44_P959355	LOC361963	XR_008469	361963	0.19	1.553	0.016662
A_42_P489817	AW915389	AW915389		0.19	1.552	0.030943
A_44_P149662	RGD1311086	NM_001024979	295228	0.19	1.552	0.005229
A_44_P279912	Crebbp	NM_133381	54244	0.19	1.552	0.09789
A_44_P286138	RGD1307927_predicted	XM_231184	311913	0.19	1.552	0.027489
A_44_P199661	RGD1563853_predicted	XM_341464	361181	0.19	1.552	0.047301
A_44_P508400	Exoc7	NM_022691	64632	0.19	1.552	0.009509
A_43_P10591	Tcea1	NM_001025735	362479	0.19	1.552	0.004606
A_44_P749192	TC527489	TC527489		0.19	1.552	0.097762
A_44_P384937	Mterfd2	NM_001037209	363289	0.19	1.551	0.004486
A_44_P463313	Plk1	NM_017100	25515	0.19	1.551	0.054487
A_43_P13089	Txnl1	NM_080887	140922	0.19	1.551	0.014304
A_43_P19045	Banp_predicted	XM_214701		0.19	1.551	0.015109
A_44_P416596	Pdp2	NM_145091	246311	0.19	1.551	0.039982
A_44_P143939	Spred1	XM_230454		0.19	1.551	0.021883
A_44_P175345	Polb	NM_017141	29240	0.19	1.551	0.024107
A_43_P10428	Snrp1c_predicted	XM_001078182		0.19	1.551	0.003727
A_42_P649762	Sh3gl1	NM_031239	81922	0.19	1.551	0.001065
A_44_P266946	Rpl9	NM_001007598	29257	0.19	1.551	0.001335
A_44_P652021	LOC364851	XR_008458	364851	0.19	1.550	0.017472
A_44_P173039	Nsun5_predicted	XM_213749	288595	0.19	1.550	0.004204
A_44_P304919	LOC501285	XM_576700	501285	0.19	1.550	0.002111
A_44_P590505	ENSRNOT00000033038	ENSRNOT00000033038		0.19	1.550	0.187845
A_44_P249092	Ggcx	NM_031756	81716	0.19	1.550	0.019058
A_42_P609456	Thap1	NM_001008340	306547	0.19	1.550	0.021005
A_44_P319310	XM_237365	XM_237365		0.19	1.550	0.021091
A_44_P445737	Ube2j1_predicted	XM_216362		0.19	1.550	0.003415
A_44_P996952	RGD1561028	NM_001039454	315283	0.19	1.549	0.030487
A_44_P351563	ENSRNOT00000014877	ENSRNOT00000014877		0.19	1.549	0.011746
A_44_P821792	A_44_P821792	A_44_P821792		0.19	1.549	0.00739

A_44_P1058502	Ppfia1_predicted	XM_238162		0.19	1.549	0.007961
A_44_P140778	Strn	NM_019148	29149	0.19	1.549	0.024303
A_43_P18082	RGD1565845_predicted	XM_342266		0.19	1.548	0.11764
A_44_P900229	TC541330	TC541330		0.19	1.548	0.051965
A_44_P107160	Gps1	NM_053969	117039	0.19	1.548	0.011285
A_44_P346669	Siahbp1	XM_343268	84401	0.19	1.548	0.007902
A_44_P853268	TC551918	TC551918		0.19	1.548	0.068493
A_44_P186336	Rab15	M83679	299156	0.19	1.548	0.054555
A_44_P557541	Strn	NM_019148	29149	0.19	1.548	0.014783
A_44_P468828	RGD1307930_predicted	XM_232121	312490	0.19	1.547	0.147818
A_44_P257804	XM_229392	XM_229392		0.19	1.547	0.003809
A_44_P712117	CO382995	CO382995		0.19	1.547	0.009952
A_44_P349614	LOC679693	XM_001054608	679693	0.19	1.547	0.01227
A_44_P533786	Aurkb	NM_053749	114592	0.19	1.547	0.018404
A_44_P460797	Itgae	AF020046	83577	0.19	1.547	0.005336
A_44_P164889	Csnk1a1	NM_053615	113927	0.19	1.547	0.005379
A_44_P489793	XM_223834	XM_223834		0.19	1.547	0.001262
A_44_P595745	TC530682	TC530682		0.19	1.547	0.013398
A_44_P243385	Zfp143	NM_001012169	361627	0.19	1.546	0.067378
A_44_P419531	LOC687694	XM_001079792		0.19	1.546	0.012071
A_44_P274251	RGD1304620_predicted	XM_222799		0.19	1.546	0.028963
A_44_P1026425	Eftud2	XM_001081526		0.19	1.546	0.010288
A_44_P859235	TC556838	TC556838		0.19	1.546	0.073003
A_44_P160688	H35674	H35674	290558	0.19	1.545	0.082391
A_44_P653818	TC523183	TC523183		0.19	1.545	0.146902
A_43_P17656	Hnrpul1_predicted	XM_341807		0.19	1.545	0.009458
A_44_P1003794	TC537551	TC537551		0.19	1.545	0.066816
A_44_P958404	BF567886	BF567886		0.19	1.545	0.153447
A_44_P813059	TC562758	TC562758		0.19	1.545	0.007196
A_44_P121857	LOC691052	XM_001076666		0.19	1.545	0.00736
A_44_P440207	Cd244	NM_022259	64025	0.19	1.544	0.036887
A_44_P189299	Ccnd1	NM_171992	58919	0.19	1.544	0.001732
A_44_P323430	Nme7	NM_138532	171566	0.19	1.544	0.110619
A_44_P791077	Cdk5rap2	XM_575844	286919	0.19	1.544	0.024265
A_44_P352172	Tubd1_predicted	XM_213412		0.19	1.544	0.011156
A_44_P397441	RGD1560994_predicted	XM_575765	500402	0.19	1.544	0.003947
A_44_P382349	XM_228774	XM_228774		0.19	1.544	0.008426
A_44_P448360	Lmln_predicted	XM_344025		0.19	1.544	0.035164
A_44_P259365	Pum2	XM_216661		0.19	1.544	0.004783
A_44_P246128	LOC501281	XM_576696	501281	0.19	1.544	0.003218
A_44_P767184	BQ211775	BQ211775		0.19	1.543	0.093282
A_44_P358928	RGD1562216_predicted	XM_575678		0.19	1.543	0.0393
A_43_P18664	RGD1311095_predicted	XM_217277	301004	0.19	1.543	0.042163
A_42_P497429	Myadm	NM_183332	369016	0.19	1.543	0.009772
A_44_P866691	AI072569	AI072569	114765	0.19	1.543	0.022281
A_44_P210686	TC517135	TC517135		0.19	1.543	0.004891
A_44_P187640	LOC368190	XM_347344		0.19	1.543	0.014611
A_43_P13393	Gpsm1	NM_144745	246254	0.19	1.543	0.006301
A_44_P302087	RGD1305958	XM_219705	309255	0.19	1.543	0.108783
A_44_P502842	Psmd11_predicted	XM_220754		0.19	1.543	0.016778
A_44_P447974	A_44_P447974	A_44_P447974		0.19	1.543	0.103779
A_44_P204207	Smyd4_predicted	XM_220708		0.19	1.542	0.014388
A_44_P377278	Adcy3	NM_130779	64508	0.19	1.542	0.005711
A_44_P532270	Herc6	XM_342700	362376	0.19	1.542	0.173278
A_44_P445476	RGD1564130_predicted	XM_347206	368042	0.19	1.541	0.039295
A_44_P905278	RGD1566204_predicted	XM_573164		0.19	1.541	0.007116
A_44_P193928	Ssbp1	NM_183328	54304	0.19	1.541	0.001853
A_44_P363672	RGD1560601_predicted	XM_241817	317432	0.19	1.541	0.005459
A_44_P900361	TC559490	TC559490		0.19	1.541	0.103779
A_43_P14483	G10	NM_053556	89819	0.19	1.541	0.006199
A_44_P536995	Rnf111_predicted	XM_236380		0.19	1.541	0.030481
A_44_P522879	RGD1564706_predicted	XM_341373		0.19	1.541	0.00275
A_44_P530131	Asb2	NM_001011984	299266	0.19	1.541	0.021146

A_44_P185441	Tnfrsf21_predicted	XM_236992		0.19	1.540	0.008699
A_44_P958935	A_44_P958935	A_44_P958935		0.19	1.540	0.002811
A_44_P281154	RGD1566122_predicted	XM_218345		0.19	1.540	0.027852
A_44_P268168	LOC686310	XM_001069404		0.19	1.540	0.007996
A_44_P558204	RGD1561189_predicted	XM_340907	360632	0.19	1.540	0.006687
A_44_P857470	TC551209	TC551209		0.19	1.540	0.068238
A_44_P318553	Met	NM_031517	24553	0.19	1.540	0.02702
A_44_P330670	XM_344264	XM_344264		0.19	1.540	0.020344
A_44_P725183	AI548201	AI548201		0.19	1.540	0.004379
A_44_P477571	Sdccag8	NM_177929	305002	0.19	1.540	0.032462
A_43_P12350	Ap2s1	NM_022952	65046	0.19	1.540	0.040525
A_42_P607026	Lysmd2_predicted	XM_217189		0.19	1.540	0.210889
A_44_P496848	Arrdc2	XM_224720		0.19	1.540	0.007056
A_44_P299722	Rrm1_mapped	NM_001013236	365320	0.19	1.539	0.012989
A_44_P1058222	Arpc5l	NM_001037767	296710	0.19	1.539	0.006946
A_44_P867698	A_44_P867698	A_44_P867698		0.19	1.539	0.015459
A_44_P239650	BF558456	BF558456		0.19	1.539	0.174174
A_44_P140164	XM_219771	XM_219771		0.19	1.539	0.007298
A_44_P805242	AW917651	AW917651	353252	0.19	1.539	0.01449
A_44_P457442	Exosc4_predicted	XM_216949	300045	0.19	1.539	0.004378
A_44_P928592	A_44_P928592	A_44_P928592		0.19	1.539	0.012591
A_44_P177907	Rccd1_predicted	XM_218819	308760	0.19	1.539	0.046873
A_44_P314353	RGD1306649	NM_001037765	288772	0.19	1.538	0.004331
A_43_P19885	Fbxl7_predicted	XM_342204		0.19	1.538	0.036937
A_44_P180080	Map1lc3b	NM_022867	64862	0.19	1.538	0.022729
A_44_P352698	AABR03065982	AABR03065982		0.19	1.538	0.006493
A_42_P627572	Mdc1	XM_227971	309595	0.19	1.537	0.01281
A_42_P717643	Nck1_predicted	XM_217246		0.19	1.537	0.049055
A_44_P229490	Rpl31	NM_022506	64298	0.19	1.537	0.002845
A_44_P142914	RGD1307394_predicted	XM_001081752		0.19	1.537	0.011609
A_44_P159197	Rcn1_predicted	XM_342481		0.19	1.537	0.153242
A_44_P246237	RGD1560011_predicted	XM_001073577		0.19	1.537	0.00938
A_43_P13566	Pogk_predicted	XM_222846		0.19	1.537	0.03711
A_42_P468662	Polr2d_predicted	XM_344659		0.19	1.536	0.033087
A_44_P318364	Olr1083	NM_001000710	404959	0.19	1.536	0.035636
A_44_P755355	A_44_P755355	A_44_P755355		0.19	1.536	0.020968
A_43_P10252	Eif3s6	NM_001011990	299872	0.19	1.536	0.007448
A_44_P123631	LOC291411	XM_225706		0.19	1.536	0.015617
A_44_P777113	LOC684233	XM_001068152		0.19	1.536	0.036715
A_44_P228599	Sec22l1	NM_001025686	310710	0.19	1.536	0.049792
A_43_P10746	Tuba4	NM_001007004	316531	0.19	1.536	0.007456
A_44_P370855	Bmpr2	XM_217409	140590	0.19	1.536	0.012918
A_44_P760160	A_44_P760160	A_44_P760160		0.19	1.536	0.009236
A_43_P17663	RGD1564914_predicted	XM_575987		0.19	1.536	0.014956
A_44_P195280	XM_342979	XM_342979		0.19	1.536	0.012693
A_44_P549501	AW915249	AW915249		0.19	1.536	0.062643
A_44_P126061	Oasl2	NM_001009682	304549	0.19	1.536	0.073839
A_44_P194398	RGD1310774_predicted	XM_223381		0.19	1.535	0.029239
A_44_P248358	Tk2_predicted	XM_226211		0.19	1.535	0.057766
A_43_P20392	RGD1564964_predicted	XM_236438	315843	0.19	1.535	0.006425
A_44_P370637	RGD1562265_predicted	XM_233076		0.19	1.535	0.002377
A_43_P18358	Alg1_predicted	XM_340750		0.19	1.535	0.018108
A_44_P253242	RGD1559904_predicted	XM_232783		0.19	1.535	0.016736
A_43_P18842	Arrdc3	NM_001007797	309945	0.19	1.535	0.048018
A_44_P701086	TC526880	TC526880		0.19	1.535	0.08191
A_44_P883350	ENSRNOT00000034377	ENSRNOT00000034377		0.19	1.535	0.002803
A_44_P992478	Dhrs1	NM_001007621	290234	0.19	1.535	0.020195
A_44_P229316	Lrrc43	XM_213813	288751	0.19	1.535	0.177417
A_44_P233963	Mras	NM_012981	25482	0.19	1.534	0.026487
A_42_P807866	Ril	NM_017062	24915	0.19	1.534	0.154461
A_44_P207773	AA997141	AA997141	58845	0.19	1.534	0.13387
A_42_P549271	Mrpl43_predicted	XM_219938		0.19	1.534	0.003021
A_44_P497144	LOC314393	XM_001065684		0.19	1.534	0.029878

A_44_P142265	Mmp16	NM_080776	65205	0.19	1.534	0.00819
A_43_P17583	CB544821	CB544821	362015	0.19	1.534	0.005814
A_44_P260201	Zfp668_predicted	XM_219363		0.19	1.534	0.002376
A_44_P605492	LOC498028	NM_001017481	498028	0.19	1.534	0.185428
A_44_P883826	TC538354	TC538354		0.19	1.534	0.012808
A_44_P575816	LOC501384	XM_576797		0.19	1.534	0.359418
A_44_P191924	Frap1	NM_019906	56718	0.19	1.534	0.030409
A_44_P340159	XM_230285	XM_230285		0.19	1.534	0.015958
A_44_P220555	Rbm15b_predicted	XM_236613	315988	0.19	1.534	0.005393
A_44_P703479	Tgfbr1	NM_012775	29591	0.19	1.534	0.031326
A_44_P884231	CR754959	CR754959	246303	0.19	1.533	0.04764
A_44_P426102	40071	NM_133297	113922	0.19	1.533	0.007921
A_44_P824014	TC560203	TC560203		0.19	1.533	0.07835
A_43_P10076	Stx6	NM_031665	60562	0.19	1.533	0.003861
A_44_P511228	XM_232629	XM_232629		0.19	1.533	0.010899
A_44_P1033595	RGD1309871_predicted	XM_219541		0.19	1.533	0.013504
A_43_P19970	RGD1308139_predicted	XM_340840		0.19	1.533	0.004084
A_44_P251840	Aebp2_predicted	XM_216295		0.19	1.532	0.005837
A_43_P16804	Pole3	NM_001007652	298098	0.19	1.532	0.007234
A_44_P494771	Ubiad1_predicted	XM_233672		0.19	1.532	0.005967
A_43_P23155	BF553320	BF553320	307126	0.19	1.532	0.027251
A_42_P727711	LOC689755	XM_001071892		0.19	1.532	0.010881
A_44_P994783	RGD1311451_predicted	XM_213217	287063	0.19	1.532	0.049799
A_43_P18091	Brp16	NM_001007707	315094	0.19	1.532	0.01771
A_44_P437641	RGD1359108	NM_001007702	313155	0.19	1.532	0.035262
A_44_P1053754	Ube2q2_predicted	XM_001072896		0.19	1.532	0.001108
A_44_P318843	Prmt5_predicted	XM_344405		0.19	1.532	0.030942
A_44_P653343	TC556687	TC556687		0.19	1.532	0.014396
A_44_P156553	Mrps26_predicted	NM_001013206	362216	0.19	1.532	0.002671
A_42_P742797	Brf1_predicted	XM_216801		0.19	1.532	0.022076
A_44_P206825	CA339683	CA339683	364738	0.19	1.531	0.014362
A_43_P16917	Ehd1	XM_001074569	293692	0.18	1.531	0.002339
A_44_P886690	TC549128	TC549128		0.18	1.530	0.005266
A_44_P1009846	XM_216518	XM_216518		0.18	1.530	0.105098
A_42_P462992	LOC683383	XM_001063050		0.18	1.530	0.013314
A_44_P215595	Smarcb1	NM_001025728	361825	0.18	1.530	0.011071
A_43_P12773	Ctbp2	NM_053335	81717	0.18	1.530	0.060573
A_44_P440752	Fbxl11_predicted	XM_341983		0.18	1.530	0.003459
A_44_P287301	Bcl6_predicted	XM_221333		0.18	1.530	0.01395
A_44_P445405	Lrrk1_predicted	XM_218760	308703	0.18	1.530	0.0021
A_42_P762618	Ccs	NM_053425	84485	0.18	1.530	0.006448
A_44_P375151	TC535896	TC535896		0.18	1.530	0.003175
A_44_P1004737	CA503871	CA503871		0.18	1.530	0.008238
A_44_P114459	LOC684106	XM_001068955		0.18	1.530	0.005663
A_44_P398587	Usp9x_predicted	XM_343766	363445	0.18	1.530	0.104218
A_44_P147774	AA900222	AA900222		0.18	1.530	0.016258
A_42_P793807	LOC245960	NM_139101	245960	0.18	1.529	0.014611
A_44_P412816	Stag2_predicted	XM_233108	313304	0.18	1.529	0.056152
A_44_P732364	LOC678739	XM_001053211	678739	0.18	1.529	0.015283
A_44_P344425	Dock9	XM_224538		0.18	1.529	0.018858
A_44_P181836	AW915351	AW915351		0.18	1.529	0.033723
A_42_P653673	Snrpd1_predicted	XM_214621		0.18	1.529	0.030956
A_42_P457773	RGD1304696	NM_001024998	307180	0.18	1.528	0.025558
A_44_P837503	TC534506	TC534506		0.18	1.528	0.093525
A_44_P374658	Arf3	NM_080904	140940	0.18	1.528	0.020686
A_44_P867900	ENSRNOT00000034844	ENSRNOT00000034844		0.18	1.528	0.022729
A_44_P940889	BG667380	BG667380	79215	0.18	1.528	0.007586
A_44_P109993	Abhd8_predicted	XM_224713		0.18	1.528	0.005551
A_42_P609263	Lrrc42	NM_001025653	298309	0.18	1.528	0.022472
A_44_P148280	Crcp	NM_053670	114205	0.18	1.527	0.012604
A_44_P557141	CO568196	CO568196		0.18	1.527	0.007556
A_42_P723060	Dnalc4	NM_001009666	300078	0.18	1.527	0.00415
A_44_P380960	DV716962	DV716962		0.18	1.527	0.120545

A_44_P286409	Ttk_predicted	XM_236477		0.18	1.527	0.027164
A_44_P541456	Arpc4_predicted	XM_238365		0.18	1.527	0.011037
A_44_P810605	TC544086	TC544086		0.18	1.527	0.295322
A_44_P368213	XM_232281	XM_232281		0.18	1.527	0.002691
A_44_P236355	Rps6kc1	XM_213966		0.18	1.526	0.005092
A_44_P213133	Endog	NM_001034938	362100	0.18	1.526	0.014047
A_44_P668035	TC530939	TC530939		0.18	1.526	0.130851
A_43_P10207	CB547605	CB547605		0.18	1.526	0.026919
A_44_P1016061	DV729072	DV729072		0.18	1.526	0.181415
A_44_P107634	RGD1305288	NM_001013995	305882	0.18	1.526	0.013224
A_44_P448108	RGD1562201_predicted	XM_346328		0.18	1.526	0.024289
A_44_P471411	Rai1_predicted	XM_220520	303188	0.18	1.525	0.077494
A_44_P670272	TC527451	TC527451		0.18	1.525	0.352294
A_43_P10641	Mgst2_predicted	XM_215562		0.18	1.525	0.008239
A_44_P510949	Ms4a7_predicted	XM_215200		0.18	1.525	0.022491
A_44_P358194	Fmo3	NM_053433	84493	0.18	1.525	0.064444
A_44_P515410	Nfix	XM_213849		0.18	1.524	0.007206
A_44_P411386	DV726507	DV726507		0.18	1.524	0.193545
A_44_P940344	RGD1562326_predicted	XM_001080919		0.18	1.524	0.077142
A_44_P140805	LOC361100	XM_341386	361100	0.18	1.524	0.012092
A_44_P436473	LOC691849	XM_001079851	691849	0.18	1.524	0.037833
A_44_P266705	RGD1309341	NM_001014141	361137	0.18	1.524	0.01841
A_44_P326133	XM_231258	XM_231258		0.18	1.524	0.208432
A_44_P789739	Slc19a2	NM_001030024	289175	0.18	1.524	0.143272
A_44_P445953	RGD1562841_predicted	XM_343874		0.18	1.524	0.010548
A_44_P243556	XM_225514	XM_225514		0.18	1.524	0.003047
A_42_P766027	LOC690372	XM_218195	308335	0.18	1.524	0.011081
A_44_P235978	Fasn	NM_017332	50671	0.18	1.524	0.002723
A_43_P10938	CF109238	CF109238	313027	0.18	1.523	0.024479
A_44_P545873	Znf324_predicted	XM_344862	365192	0.18	1.523	0.004572
A_44_P243801	RGD1564969_predicted	XM_345827		0.18	1.523	0.001946
A_44_P142541	AA956967	AA956967		0.18	1.523	0.162597
A_43_P23256	BF563521	BF563521	313131	0.18	1.523	0.041669
A_42_P561754	Pex11a	NM_053487	85249	0.18	1.523	0.249557
A_44_P346848	RGD1310320	NM_001037776	304735	0.18	1.523	0.006703
A_44_P337579	RGD1308302	NM_001008346	308911	0.18	1.523	0.053632
A_44_P481398	Unc13a	NM_022861	64829	0.18	1.523	0.051924
A_44_P1048004	XM_216505	XM_216505		0.18	1.523	0.044396
A_44_P905893	TC556853	TC556853		0.18	1.523	0.07812
A_44_P430965	Pvrl1	XM_236210	192183	0.18	1.522	0.031688
A_44_P194439	Dnajc9_predicted	XM_344286		0.18	1.522	0.042118
A_44_P1015189	AW915667	AW915667	25238	0.18	1.522	0.003391
A_44_P452466	Cab39l	NM_001011917	290291	0.18	1.522	0.004142
A_44_P297179	XM_345618	XM_345618		0.18	1.522	0.028605
A_44_P548065	Bcl2l1	NM_031535	24888	0.18	1.522	0.017537
A_44_P762670	A_44_P762670	A_44_P762670		0.18	1.522	0.042447
A_44_P366648	BF557173	BF557173		0.18	1.522	0.063102
A_44_P193407	RGD1307812	NM_001014170	361946	0.18	1.522	0.032931
A_44_P549604	LOC686736	XM_001078389		0.18	1.522	0.018212
A_42_P640700	RGD1310725	NM_001039008	297077	0.18	1.521	0.072385
A_44_P398014	Hdac3	NM_053448	84578	0.18	1.521	0.014156
A_44_P994599	Rnut1	NM_001004270	316108	0.18	1.521	0.011618
A_44_P283636	Cic_predicted	XM_218337		0.18	1.521	0.005998
A_44_P1056788	LOC680945	XM_001059596		0.18	1.521	0.007051
A_44_P389283	Ash1l_predicted	XM_227409		0.18	1.520	0.107246
A_44_P314206	Hdac2	XM_342149	84577	0.18	1.520	0.01209
A_43_P21532	Pcdhb17	XM_001065253		0.18	1.520	0.106742
A_44_P346674	Hdac7a	XM_345868	84582	0.18	1.520	0.012445
A_44_P1003378	XM_216221	XM_216221		0.18	1.520	0.061223
A_44_P100980	Rev1l_predicted	XM_237071		0.18	1.520	0.008364
A_43_P22126	XM_221892	XM_221892		0.18	1.520	0.004158
A_44_P570959	DV727605	DV727605		0.18	1.520	0.019972
A_42_P601802	Tslpr	NM_134465	171499	0.18	1.520	0.003475

A_43_P15265	Ppp3cb	NM_017042	24675	0.18	1.520	0.017625
A_44_P513840	RGD1307767_predicted	XM_220584		0.18	1.519	0.12922
A_44_P260285	XM_344449	XM_344449		0.18	1.519	0.009669
A_44_P477212	BI278569	BI278569		0.18	1.519	0.014879
A_43_P18795	Agpat6	XM_214379		0.18	1.519	0.003525
A_44_P1039760	Tcte1l	NM_001013228	363448	0.18	1.519	0.007641
A_44_P274550	Ube2d1_predicted	XM_342125		0.18	1.519	0.030766
A_44_P463972	Eif4g2	XM_001072139	678831	0.18	1.519	0.014633
A_44_P149441	LOC314140	NM_001009694	314140	0.18	1.519	0.00791
A_44_P100996	Rpl29	NM_017150	29283	0.18	1.518	0.003953
A_44_P203769	MGC94542	NM_001006964	290631	0.18	1.518	0.014631
A_44_P667125	ENSRNOT00000036114	ENSRNOT00000036114		0.18	1.518	0.00502
A_44_P148865	Homer1	NM_031707	29546	0.18	1.518	0.061305
A_44_P238733	Tmed5	NM_001007619	289883	0.18	1.518	0.144065
A_44_P317773	Ptpn11	NM_013088	25622	0.18	1.518	0.01248
A_44_P499022	E2f8	XM_218601	308607	0.18	1.518	0.023137
A_44_P304408	RGD1559475_predicted	XM_574266	498977	0.18	1.518	0.00905
A_43_P18144	Fundc1	NM_001025027	363442	0.18	1.518	0.010426
A_44_P393915	RGD1559526_predicted	XM_344718		0.18	1.518	0.037695
A_44_P824102	TC526114	TC526114		0.18	1.518	0.038521
A_42_P794901	Ywhah	NM_013052	25576	0.18	1.517	0.004737
A_42_P841860	Atp5g2	NM_133556	171082	0.18	1.517	0.002696
A_44_P135402	A_44_P135402	A_44_P135402		0.18	1.517	0.105098
A_44_P411505	Ccnd1	NM_171992	58919	0.18	1.517	0.006793
A_43_P12417	Hspa8	NM_024351	24468	0.18	1.517	0.024112
A_44_P126131	Tbc1d1_predicted	XM_341215	360937	0.18	1.517	0.010619
A_44_P465573	AI170070	AI170070	312863	0.18	1.517	0.026868
A_44_P524472	RGD1311752_predicted	XM_215477		0.18	1.517	0.219063
A_44_P928907	RGD1565049_predicted	XM_574241	498952	0.18	1.517	0.02935
A_43_P17141	Smyd2	XM_213972	289372	0.18	1.517	0.010917
A_44_P332168	Nsfl1c	NM_031981	83809	0.18	1.517	0.005986
A_44_P579345	TC556628	TC556628		0.18	1.517	0.009411
A_44_P279202	Phc2	XM_232751	313038	0.18	1.516	0.008653
A_44_P300079	Prpf38a_predicted	XM_216475		0.18	1.516	0.013502
A_42_P671348	Gup1_predicted	XM_217299		0.18	1.516	0.006975
A_44_P550378	Ppp4c	NM_134359	171366	0.18	1.516	0.00501
A_43_P18668	Btrc	NM_001007148	361765	0.18	1.516	0.118143
A_44_P357579	Ddx3x	XM_001060162		0.18	1.516	0.010321
A_44_P762130	TC541630	TC541630		0.18	1.516	0.033986
A_44_P999768	AA944506	AA944506	288051	0.18	1.516	0.006763
A_44_P975522	CF108541	CF108541		0.18	1.516	0.008018
A_44_P454480	Kcnj15	NM_133321	170847	0.18	1.515	0.140783
A_42_P643574	Aaas_predicted	XM_217063		0.18	1.515	0.003596
A_43_P15782	Gtf2ird1	AY115565	246770	0.18	1.515	0.007042
A_44_P249531	Ppp2r2d	NM_144746	246255	0.18	1.515	0.007765
A_44_P392334	Ppp4r1	NM_080907	140943	0.18	1.515	0.037773
A_44_P853901	TC541648	TC541648		0.18	1.514	0.106317
A_44_P293187	RGD1560997_predicted	XM_346305		0.18	1.514	0.002821
A_44_P116726	Lins2_predicted	XM_218748	308704	0.18	1.514	0.01634
A_44_P760475	ENSRNOT00000006954	ENSRNOT00000006954		0.18	1.513	0.114209
A_43_P12148	Top2a	NM_022183	360243	0.18	1.513	0.031696
A_44_P372181	Dbt	XM_001073131		0.18	1.513	0.131773
A_44_P255954	RGD1566063_predicted	XM_573223	498024	0.18	1.513	0.010898
A_44_P484806	Pcmt1	NM_013073	25604	0.18	1.513	0.005484
A_43_P22823	Nsd1_predicted	XM_001066409		0.18	1.513	0.019135
A_44_P1001301	Eef1g	XM_574616		0.18	1.513	0.0023
A_44_P762095	AF452731	AF452731		0.18	1.513	0.077524
A_43_P12802	Ufd1l	NM_053418	84478	0.18	1.513	0.020999
A_44_P331097	Arhgap8_predicted	NM_001012121	315189	0.18	1.513	0.020348
A_44_P439562	AI234128	AI234128	361315	0.18	1.513	0.02554
A_44_P450104	LOC290396	XM_224392	290396	0.18	1.512	0.039165
A_44_P314231	A_44_P314231	A_44_P314231		0.18	1.512	0.006168
A_44_P187249	XM_341085	XM_341085		0.18	1.512	0.003645

A_44_P218179	Gns	NM_001011989	299825	0.18	1.512	0.014282
A_44_P1033009	Ppp1r7	NM_001009825	301618	0.18	1.512	0.008367
A_44_P1035802	XM_214528	XM_214528		0.18	1.512	0.009437
A_44_P109303	Sec63_predicted	XM_228305		0.18	1.512	0.044735
A_42_P618256	Tmem5	NM_001024759	299841	0.18	1.512	0.004607
A_44_P192244	Aven_predicted	XM_230438		0.18	1.512	0.008772
A_44_P480455	Efs_predicted	XM_237944		0.18	1.512	0.135516
A_44_P410159	Fkbp7_predicted	XM_215758		0.18	1.512	0.122733
A_44_P105566	A_44_P105566	A_44_P105566		0.18	1.511	0.015239
A_44_P171109	RGD1564278_predicted	XM_001053101		0.18	1.511	0.002289
A_44_P538816	RGD1560931_predicted	XM_344211		0.18	1.511	0.032584
A_44_P516017	Efhd2	NM_001031648	298609	0.18	1.511	0.005732
A_44_P468365	Gnb1	NM_030987	24400	0.18	1.511	0.016485
A_44_P354444	Bcs1l	NM_001007666	301514	0.18	1.511	0.084493
A_44_P175875	Pigv	NM_001010966	366478	0.18	1.511	0.041539
A_44_P482519	Itgb7	XM_343336	25713	0.18	1.511	0.025096
A_44_P548726	RGD1308123	NM_001009664	299647	0.18	1.511	0.011479
A_42_P524098	XM_217340	XM_217340		0.18	1.511	0.017658
A_44_P1057176	Geft	NM_199395	314904	0.18	1.510	0.196268
A_44_P192360	RGD1307791_predicted	NM_001039914	297968	0.18	1.510	0.003752
A_44_P137247	Trim35	NM_001025142	498538	0.18	1.510	0.099908
A_44_P313173	BI286340	BI286340		0.18	1.510	0.12901
A_44_P251908	Xpnpep1	NM_131913	170751	0.18	1.510	0.005028
A_44_P1005383	RGD1304846	NM_001014121	360650	0.18	1.509	0.007325
A_44_P825523	Smurf2_predicted	XM_001081579		0.18	1.509	0.004558
A_43_P18706	Trim32	NM_001012103	313264	0.18	1.509	0.009965
A_44_P206270	XM_231272	XM_231272		0.18	1.509	0.009143
A_44_P714750	RGD1562438_predicted	XM_001077874		0.18	1.509	0.106222
A_43_P15577	Rab11b	NM_032617	79434	0.18	1.509	0.015139
A_44_P234420	XM_343828	XM_343828		0.18	1.509	0.029511
A_44_P311827	Slc26a8_predicted	XM_228038		0.18	1.509	0.169908
A_44_P1044410	Sf3b2_predicted	XM_215182		0.18	1.508	0.006622
A_44_P926567	AW914146	AW914146	24627	0.18	1.508	0.013553
A_44_P476107	XM_217278	XM_217278		0.18	1.508	0.006045
A_43_P21165	Kpna6	NM_001015029	362607	0.18	1.508	0.051525
A_44_P543753	LOC499896	XM_575242		0.18	1.508	0.040593
A_44_P403005	Trub1	NM_001012173	361775	0.18	1.508	0.003281
A_44_P489512	LOC681314	XM_001061020		0.18	1.508	0.008084
A_44_P144393	Ihpk2	XM_576471		0.18	1.508	0.05978
A_44_P867959	ENSRNOT00000037445	ENSRNOT00000037445		0.18	1.508	0.102082
A_44_P448222	RGD1562252_predicted	XM_236223		0.18	1.508	0.08786
A_44_P219481	Rabep1	NM_019124	54190	0.18	1.507	0.008679
A_42_P700879	Pqbp1	NM_001013957	302557	0.18	1.507	0.008988
A_44_P129298	Slc45a3_predicted	XM_222629	304785	0.18	1.507	0.263402
A_44_P437941	Mrpl17	NM_133539	171061	0.18	1.507	0.017908
A_44_P254254	AI013204	AI013204	84353	0.18	1.507	0.011544
A_44_P445916	Ddx23_predicted	XM_217050		0.18	1.507	0.003529
A_44_P323599	LOC687726	XM_001079932		0.18	1.507	0.00354
A_44_P790500	LOC687079	XM_001077003		0.18	1.507	0.100388
A_44_P368427	Ksr1_predicted	XM_340852		0.18	1.507	0.083744
A_44_P172092	Aamp_predicted	XM_217441		0.18	1.507	0.002098
A_44_P326422	Ibtk_predicted	XM_236481	315858	0.18	1.507	0.0845
A_44_P524690	Polr2g	NM_053948	117017	0.18	1.506	0.020573
A_44_P354911	Svil_predicted	XM_341540		0.18	1.506	0.01614
A_44_P415354	Gata6	NM_019185	29300	0.18	1.506	0.032601
A_43_P10327	RGD1359310	NM_001007659	300240	0.18	1.506	0.003215
A_44_P986952	AW916350	AW916350	292060	0.18	1.506	0.031002
A_42_P761277	RGD1305689	NM_001008297	290529	0.18	1.506	0.06757
A_44_P466255	LOC690517	XM_001074661		0.18	1.506	0.021987
A_44_P274815	Wsb1	NM_001025664	303336	0.18	1.506	0.150711
A_44_P269453	Eif2b2	NM_032058	84005	0.18	1.506	0.00198
A_43_P14040	TC544588	TC544588		0.18	1.506	0.025082
A_44_P721633	TC543282	TC543282		0.18	1.506	0.025118

A_43_P23210	Fmip	NM_001012153	360972	0.18	1.505	0.006975
A_44_P549600	AW141288	AW141288	117056	0.18	1.505	0.011554
A_44_P236806	Gga1	NM_001011994	300066	0.18	1.505	0.007206
A_44_P302676	RGD1566002_predicted	XM_221003		0.18	1.505	0.009224
A_44_P109650	Hnrpab	AF108653	83498	0.18	1.505	0.048065
A_44_P243948	CA503874	CA503874	302935	0.18	1.504	0.095981
A_44_P330170	Dnm1l	NM_053655	114114	0.18	1.504	0.015289
A_43_P21780	CB547076	CB547076	309887	0.18	1.504	0.019092
A_44_P725683	AW915625	AW915625		0.18	1.504	0.151377
A_44_P242969	Gpam	NM_017274	29653	0.18	1.504	0.011891
A_44_P142428	LOC679437	XM_001056340		0.18	1.504	0.002758
A_44_P516958	RGD1562407_predicted	XM_001064372		0.18	1.504	0.043485
A_44_P521264	Pura_predicted	XM_001063244		0.18	1.504	0.115663
A_44_P667509	ENSRNOT00000031239	ENSRNOT00000031239		0.18	1.504	0.002608
A_42_P735544	Vapa	NM_031631	58857	0.18	1.504	0.012479
A_44_P555618	RGD1308722_predicted	XM_342116		0.18	1.504	0.309444
A_44_P514899	AI011063	AI011063		0.18	1.503	0.023022
A_44_P221599	Rbm4_predicted	XM_215201	170900	0.18	1.503	0.003275
A_44_P466041	Thtpa	NM_001007682	305889	0.18	1.503	0.004099
A_44_P473366	Usp33	XM_001068666	310960	0.18	1.503	0.099656
A_44_P524830	RGD1310191	XM_341102		0.18	1.503	0.036642
A_44_P527536	Mrrf	NM_001008354	311903	0.18	1.503	0.003206
A_44_P486829	Mtf2	XM_341180	360905	0.18	1.503	0.028951
A_44_P837569	Atp8b2	XM_001074928		0.18	1.503	0.013502
A_44_P378315	Srp54	NM_053871	116650	0.18	1.503	0.006841
A_44_P390314	AW916077	AW916077	64630	0.18	1.503	0.015918
A_44_P550828	Spry4_predicted	XM_225969		0.18	1.503	0.039656
A_44_P989737	A_44_P989737	A_44_P989737		0.18	1.503	0.002323
A_44_P144741	Blnk	NM_001025767	499356	0.18	1.503	0.026019
A_44_P883950	BE108909	BE108909		0.18	1.503	0.035487
A_44_P295563	Col7a1_predicted	XM_238554		0.18	1.503	0.037377
A_43_P17816	Cdv1	NM_199120	373066	0.18	1.502	0.003717
A_43_P14365	Zmynd11	NM_203369	291259	0.18	1.502	0.01313
A_44_P545921	XM_223153	XM_223153		0.18	1.502	0.002258
A_44_P147030	Sp4	NM_012761	25162	0.18	1.502	0.147903
A_44_P362579	Cdc25a	NM_133571	171102	0.18	1.502	0.162524
A_44_P241741	CB606017	CB606017		0.18	1.502	0.022506
A_44_P621785	XM_236024	XM_236024		0.18	1.502	0.007816
A_43_P20877	Terf1	NM_001012464	297758	0.18	1.502	0.036939
A_44_P976610	TC559618	TC559618		0.18	1.502	0.007211
A_44_P526729	Klf10	NM_031135	81813	0.18	1.502	0.023314
A_44_P573304	AW916728	AW916728		0.18	1.502	0.009543
A_43_P10962	Gpiap1	NM_001012185	362173	0.18	1.502	0.009582
A_42_P577136	Fcer2a	NM_001033924	171075	0.18	1.502	0.014753
A_44_P544340	Obfc2b	NM_001034939	362813	0.18	1.502	0.024243
A_44_P129489	Lrpap1	XM_341239	116565	0.18	1.502	0.068548
A_44_P852414	ENSRNOT00000007669	ENSRNOT00000007669		0.18	1.502	0.010849
A_42_P617931	XM_342362	XM_342362		0.18	1.502	0.028564
A_44_P203696	XM_341263	XM_341263		0.18	1.501	0.214147
A_44_P511571	RGD1562438_predicted	XM_001077874		0.18	1.501	0.128035
A_44_P150267	Akap13	XM_001063231		0.18	1.501	0.040938
A_44_P170778	Irgq_predicted	XM_218444	292708	0.18	1.501	0.006277
A_44_P283715	Ehbp1_predicted	XM_223664	305556	0.18	1.501	0.080115
A_44_P1022711	RGD1309720	NM_001029916	295971	0.18	1.501	0.2368
A_44_P356538	Zfp330_predicted	XM_341666		0.18	1.501	0.011429
A_44_P459279	RGD1305036_predicted	XM_218937	308837	0.18	1.501	0.047049
A_43_P21386	CB546740	CB546740		0.18	1.501	0.022918
A_44_P466700	Lpin1	NM_001012111	313977	0.18	1.501	0.032754
A_44_P1017125	Sav1_predicted	XM_216725		0.18	1.501	0.045869
A_44_P1027075	Tmem38a_predicted	XM_224706		0.18	1.500	0.041553
A_42_P838326	Mrps17_predicted	XM_213762		0.18	1.500	0.014753
A_44_P562251	Luzp5_predicted	XM_001061369		0.18	1.500	0.011734
A_44_P226029	Rbmxrt_predicted	XM_226369		0.18	1.500	0.040593

A_44_P548303	Thumpd1	NM_001009688	309041	0.18	1.500	0.021873
A_44_P575991	TC552266	TC552266		0.18	1.500	0.038879
A_44_P144659	RGD1308428_predicted	XR_009297	308509	0.18	1.500	0.006812
A_44_P405843	Tns4	NM_001024881	303517	0.18	1.500	0.015194
A_44_P275029	Mvp	NM_022715	64681	0.18	1.500	0.029286
A_43_P12624	Lxn	NM_031655	59073	0.18	1.499	0.013529
A_43_P23116	LOC365592	NM_001014249	365592	0.18	1.499	0.03464
A_44_P959127	A_44_P959127	A_44_P959127		0.18	1.499	0.008863
A_44_P946706	TC544067	TC544067		0.18	1.499	0.023651
A_44_P538317	MGC73003	NM_198777	361728	0.18	1.499	0.004012
A_44_P925674	AW917673	AW917673	690795	0.18	1.499	0.003645
A_44_P107552	XM_222887	XM_222887		0.18	1.499	0.050611
A_44_P1009692	Hmgn2	NM_001025624	114637	0.18	1.499	0.002512
A_44_P556079	RGD1564259_predicted	XM_218081	292477	0.18	1.499	0.264886
A_43_P23298	RGD1311730_predicted	XM_001079737		0.18	1.499	0.068532
A_44_P776641	TC538208	TC538208		0.18	1.499	0.302206
A_44_P485747	RGD1310783	NM_001013856	287249	0.18	1.499	0.016868
A_44_P1017890	Pcgf1	NM_001007000	312480	0.18	1.499	0.014213
A_44_P1033459	Uxs1	NM_139336	246232	0.18	1.499	0.041229
A_42_P710166	Ppfia1_predicted	XM_238162		0.18	1.498	0.114978
A_44_P583166	BF402083	BF402083		0.18	1.498	0.007178
A_44_P338002	XM_234588	XM_234588		0.18	1.498	0.004313
A_44_P215602	LOC294396	XR_007611	294396	0.18	1.498	0.015402
A_43_P11377	Pin1_predicted	XM_216609		0.18	1.498	0.005358
A_44_P711071	Smg7_predicted	XM_341132	360855	0.18	1.498	0.003282
A_43_P18207	LOC688968	XM_001069006	688968	0.18	1.498	0.002928
A_44_P222511	RGD1562936_predicted	XR_007989	294835	0.18	1.497	0.01613
A_44_P471099	Rbm27_predicted	XM_341605		0.18	1.497	0.016896
A_44_P105174	Tmem109	NM_001007736	361732	0.18	1.497	0.005709
A_44_P913386	RGD1561062_predicted	XM_573179		0.18	1.497	0.013082
A_44_P916981	Stag1_predicted	XM_001067112		0.18	1.497	0.054461
A_44_P806211	Cln8	NM_001007686	306619	0.18	1.497	0.006856
A_44_P241168	Ttc18_predicted	XM_341281		0.18	1.497	0.086967
A_44_P999676	MGC94192	NM_001004272	360550	0.18	1.497	0.011239
A_42_P561644	Timm10	NM_172074	64464	0.18	1.497	0.026508
A_42_P767077	Gja4	NM_021654	25655	0.18	1.496	0.072259
A_44_P405381	C1qtnf3_predicted	XM_226833	294806	0.18	1.496	0.063594
A_44_P421944	RGD1562724_predicted	XM_225322		0.18	1.496	0.009522
A_44_P422237	RGD1307682	NM_001024760	300675	0.17	1.496	0.063956
A_44_P457053	XM_224246	XM_224246		0.17	1.496	0.01154
A_43_P12816	Synj1	XM_573256	85238	0.17	1.496	0.023257
A_42_P586064	Pacs1	NM_134406	171444	0.17	1.496	0.003245
A_44_P991376	Orc3l	NM_001025282	313138	0.17	1.496	0.077618
A_43_P12707	Ptms	NM_031975	83801	0.17	1.496	0.006998
A_44_P328767	MGC94326	NM_001007709	315141	0.17	1.495	0.031146
A_44_P319236	Syncrip	XM_343446		0.17	1.495	0.004752
A_43_P19119	Snf1lk2_predicted	XM_001071064		0.17	1.495	0.011747
A_44_P218305	Igsf11	NM_001013120	303926	0.17	1.495	0.028586
A_42_P527887	Atg4b	NM_001025711	316640	0.17	1.495	0.002119
A_44_P714409	Sfrs3_predicted	XM_342107		0.17	1.495	0.0148
A_44_P336503	AI012337	AI012337	300092	0.17	1.495	0.008337
A_44_P645108	DY471256	DY471256		0.17	1.495	0.044756
A_44_P973613	BF558764	BF558764	303831	0.17	1.494	0.043918
A_42_P504250	RGD1309624_predicted	XM_216706		0.17	1.494	0.018923
A_44_P161033	Hdh	XM_573634	29424	0.17	1.494	0.031976
A_44_P1034533	CK470667	CK470667		0.17	1.494	0.004899
A_44_P801562	TC540734	TC540734		0.17	1.494	0.006653
A_44_P522136	AI229412	AI229412		0.17	1.494	0.017823
A_44_P197104	RGD1310270_predicted	XM_235153		0.17	1.494	0.100588
A_44_P775787	LOC500420	XM_575783	500420	0.17	1.494	0.017059
A_43_P11509	Syp	NM_012664	24804	0.17	1.494	0.017751
A_44_P380332	Cyfip2_predicted	XM_220333		0.17	1.494	0.105718
A_44_P998998	Nsfl1c	NM_031981	83809	0.17	1.494	0.006793

A_44_P508845	RGD1560795_predicted	XM_216190		0.17	1.493	0.013839
A_44_P107972	LOC685179	XM_001055795	685179	0.17	1.493	0.007252
A_44_P835425	Otud4	XM_226388	307774	0.17	1.493	0.002781
A_43_P10843	Ahcyl1_predicted	XM_342312		0.17	1.493	0.023202
A_44_P428872	XM_341137	XM_341137		0.17	1.493	0.088188
A_44_P462010	Zbtb17	NM_001012105	313666	0.17	1.493	0.012146
A_44_P613180	A_44_P613180	A_44_P613180		0.17	1.493	0.035056
A_44_P594610	Cdca8	NM_001025050	500545	0.17	1.493	0.156361
A_43_P17839	Prkra	NM_001024780	311130	0.17	1.493	0.018551
A_44_P324066	LOC684063	XM_001068728		0.17	1.493	0.005033
A_44_P175530	Plek	NM_001025750	364206	0.17	1.493	0.571694
A_44_P148581	AW143740	AW143740		0.17	1.493	0.04389
A_44_P1060345	Cpox	NM_001037095	304024	0.17	1.493	0.171684
A_44_P250879	Dmrta2_predicted	XM_233363		0.17	1.493	0.043918
A_42_P484456	RGD1305975_predicted	XM_216181		0.17	1.492	0.018454
A_43_P12465	Nrp2	NM_030869	81527	0.17	1.492	0.011162
A_44_P215326	Irf6_predicted	XM_344194		0.17	1.492	0.04667
A_44_P505885	Vdac1	NM_031353	83529	0.17	1.492	0.012863
A_44_P867828	A_44_P867828	A_44_P867828		0.17	1.492	0.008293
A_43_P21374	Mkks	NM_001008353	311456	0.17	1.492	0.048647
A_44_P227023	Clasp1	XM_001053715		0.17	1.492	0.005242
A_44_P201858	LOC681499	XM_001057117		0.17	1.492	0.010124
A_44_P483313	Mrpl38	NM_001009369	303685	0.17	1.492	0.005939
A_44_P231859	Chd1l_predicted	XM_227510		0.17	1.492	0.044343
A_43_P20794	Osbpl5	XM_001066269	361686	0.17	1.492	0.008119
A_43_P10034	Ube2n	NM_053928	116725	0.17	1.492	0.008649
A_44_P461489	RGD1559440_predicted	XM_231528	312166	0.17	1.492	0.157167
A_44_P159040	Vegfb	NM_053549	89811	0.17	1.492	0.022948
A_44_P836900	LOC364524	XR_008330	364524	0.17	1.492	0.003107
A_44_P297525	LOC681050	XM_001060090	681050	0.17	1.492	0.007309
A_44_P1017575	Ttc1	NM_001005529	287208	0.17	1.491	0.035822
A_44_P558014	LOC362758	XM_001059623		0.17	1.491	0.090461
A_44_P487482	XM_345185	XM_345185		0.17	1.491	0.012815
A_44_P308858	Imp4	NM_001009700	316317	0.17	1.491	0.013303
A_42_P589190	Aacs	NM_023104	65984	0.17	1.491	0.03139
A_43_P11552	Ptpra	NM_012763	25167	0.17	1.491	0.021284
A_44_P387584	Gtf2e1	XM_221426	303918	0.17	1.491	0.021037
A_43_P15848	Kcnf1	XM_216678		0.17	1.491	0.013416
A_44_P202096	Pold4	NM_001013195	361698	0.17	1.491	0.014804
A_44_P431546	A_44_P431546	A_44_P431546		0.17	1.491	0.03823
A_44_P793692	TC546142	TC546142		0.17	1.491	0.075829
A_44_P1032103	RGD1308279_predicted	XM_225732	307236	0.17	1.490	0.095689
A_44_P1012279	RGD1307397	NM_001030027	290842	0.17	1.490	0.031187
A_44_P475181	Sla	NM_178097	338477	0.17	1.490	0.099905
A_44_P561933	TC554220	TC554220		0.17	1.490	0.031688
A_44_P211997	BF282863	BF282863	362838	0.17	1.490	0.041567
A_44_P995094	Eftud1_predicted	XM_218845		0.17	1.490	0.035824
A_44_P522580	Slc34a2	NM_053380	84395	0.17	1.490	0.231155
A_44_P405148	Gorasp2	NM_001007720	113961	0.17	1.490	0.011959
A_44_P307313	Snf8	NM_001007804	287645	0.17	1.490	0.008624
A_44_P860387	LOC691918	XM_001080091	691918	0.17	1.490	0.023627
A_44_P106541	Alg5	NM_001025407	295051	0.17	1.490	0.114204
A_44_P681544	AW921259	AW921259	304546	0.17	1.490	0.007824
A_44_P229710	Gpatc3_predicted	XM_342933	362615	0.17	1.489	0.008406
A_43_P15962	Ccna2	NM_053702	114494	0.17	1.489	0.038172
A_42_P670901	TC555668	TC555668		0.17	1.489	0.082924
A_44_P525878	Cul5	NM_022683	64624	0.17	1.489	0.050803
A_44_P419878	XM_345983	XM_345983		0.17	1.489	0.019096
A_42_P518958	Synj2bp	NM_022599	64531	0.17	1.489	0.013456
A_44_P376979	Isrip	NM_175604	319113	0.17	1.489	0.006379
A_44_P554465	Npepps	XM_340889		0.17	1.489	0.011712
A_44_P108291	Rbms2	NM_001025403	288771	0.17	1.489	0.006098
A_43_P16530	Pop7_predicted	XM_213733		0.17	1.489	0.005336

A_42_P576953	Pspla1	NM_138882	85311	0.17	1.489	0.039484
A_43_P16599	RGD1565196_predicted	XM_576003		0.17	1.489	0.069763
A_44_P874053	TC533908	TC533908		0.17	1.489	0.115131
A_44_P763009	TC526916	TC526916		0.17	1.488	0.07343
A_44_P224489	Cacybp	NM_001004208	289144	0.17	1.488	0.027956
A_44_P321329	Polr2c	NM_001012473	361365	0.17	1.488	0.010532
A_43_P15750	Cfd	XM_343169		0.17	1.488	0.065463
A_44_P529672	LOC292662	XR_009582	292662	0.17	1.488	0.008369
A_44_P523112	RGD1306148_predicted	XM_232937	313196	0.17	1.488	0.012183
A_44_P1027405	Raph1_predicted	XM_343579		0.17	1.488	0.02653
A_44_P1007215	Upp1	NM_001030025	289801	0.17	1.488	0.030572
A_43_P16737	Rabl4_predicted	XM_216964	300062	0.17	1.488	0.013941
A_44_P387680	CB547362	CB547362		0.17	1.488	0.015329
A_44_P528302	Gabpa_predicted	XM_001065754		0.17	1.488	0.067217
A_44_P165037	LOC687202	XM_001077533		0.17	1.488	0.011855
A_44_P206550	Dullard	XM_001080620	287447	0.17	1.488	0.028219
A_44_P478298	Centg3_predicted	XM_342612		0.17	1.487	0.009009
A_44_P733726	TC563593	TC563593		0.17	1.487	0.075467
A_44_P346931	Pnpt1	XM_341266	360992	0.17	1.487	0.098315
A_44_P377473	A_44_P377473	A_44_P377473		0.17	1.487	0.007831
A_44_P992662	Ftcd	NM_053567	89833	0.17	1.487	0.143414
A_44_P388853	Dnmt1	NM_053354	84350	0.17	1.487	0.01181
A_44_P461544	Ccnb2	NM_001009470	363088	0.17	1.487	0.071752
A_43_P13424	Yy1	NM_173290	24919	0.17	1.487	0.020246
A_44_P386689	Chrm2	NM_031016	81645	0.17	1.486	0.305164
A_44_P405886	AI010270	AI010270	116777	0.17	1.486	0.049656
A_44_P852083	ENSRNOT00000037020	ENSRNOT00000037020		0.17	1.486	0.204571
A_44_P237578	RGD1306894_predicted	XM_344763	364996	0.17	1.486	0.014489
A_44_P416968	RGD1306565_predicted	XM_344798	365057	0.17	1.486	0.070356
A_42_P772965	Aldh7a1	XM_214535	291450	0.17	1.486	0.042316
A_44_P837196	LOC681425	XM_001056732		0.17	1.486	0.008084
A_44_P620895	LOC500124	NM_001024329	500124	0.17	1.486	0.194892
A_44_P440392	RGD1310975	NM_001013998	306231	0.17	1.486	0.024698
A_44_P332957	LOC686098	XM_001066536	686098	0.17	1.485	0.041546
A_44_P833822	AW915845	AW915845	315994	0.17	1.485	0.044969
A_44_P493946	Acox3	NM_053339	83522	0.17	1.485	0.003306
A_44_P245530	Ifngr	NM_053783	116465	0.17	1.485	0.098038
A_44_P827555	Pak6_predicted	XM_230519		0.17	1.485	0.051142
A_44_P445792	Eps15	NM_001009424	313474	0.17	1.485	0.024183
A_43_P19890	BF522086	BF522086		0.17	1.485	0.005079
A_44_P244704	AW920988	AW920988		0.17	1.485	0.03731
A_44_P164192	RGD1310490_predicted	XM_221401	303903	0.17	1.485	0.138474
A_44_P253310	XM_343003	XM_343003		0.17	1.485	0.010187
A_44_P168613	Wdr33_predicted	XM_226076		0.17	1.485	0.004399
A_44_P790825	LOC365643	XR_006490	365643	0.17	1.485	0.013833
A_44_P745710	CF106934	CF106934	296708	0.17	1.485	0.176066
A_44_P499759	Mto1_predicted	XM_001060155		0.17	1.485	0.004272
A_42_P715712	BI395758	BI395758		0.17	1.484	0.026334
A_44_P301621	Pp3111	NM_172018	246185	0.17	1.484	0.012949
A_44_P312318	CB545036	CB545036	301345	0.17	1.484	0.003461
A_44_P263440	Hbp1	NM_013221	27080	0.17	1.484	0.141784
A_44_P238789	RGD1561198_predicted	XM_001073796		0.17	1.484	0.004894
A_44_P404538	Cnot10	NM_001007003	316034	0.17	1.484	0.010581
A_44_P1025883	Scamp3	XM_342279	65169	0.17	1.484	0.009489
A_44_P349002	E2f1	XM_230765	399489	0.17	1.484	0.00907
A_43_P23049	Cryz	NM_001012183	362061	0.17	1.484	0.023014
A_44_P1031443	Ciz1_predicted	XM_216034		0.17	1.483	0.004738
A_43_P19148	Sos1	XM_233820	313845	0.17	1.483	0.075829
A_44_P768244	CB556786	CB556786		0.17	1.483	0.083643
A_43_P18902	LOC500034	XM_001057511		0.17	1.483	0.116374
A_44_P501335	A_44_P501335	A_44_P501335		0.17	1.483	0.007641
A_44_P983049	CD373272	CD373272		0.17	1.483	0.151442
A_44_P105791	BF548544	BF548544		0.17	1.482	0.004726

A_44_P392782	Slc35e1_predicted	XM_224707		0.17	1.482	0.068804
A_43_P16070	Axl	NM_001013147	308444	0.17	1.482	0.283626
A_43_P18343	Gga2	XM_215045	293455	0.17	1.482	0.004932
A_44_P461657	LOC293697	XM_001071880		0.17	1.482	0.002581
A_43_P16160	Cenpf	XM_223060	257649	0.17	1.482	0.061822
A_44_P537843	AA858648	AA858648		0.17	1.482	0.015305
A_43_P20533	Rbm14	XM_001072105		0.17	1.482	0.01346
A_44_P488637	Timm17a	NM_019351	54311	0.17	1.482	0.012464
A_44_P679871	AI059193	AI059193		0.17	1.482	0.076456
A_44_P141899	Ppp1r2	NM_138823	192361	0.17	1.482	0.052458
A_44_P1033385	Agl_predicted	XM_001073642		0.17	1.482	0.066099
A_44_P566937	AA957027	AA957027		0.17	1.482	0.072604
A_44_P276782	Trp53bp1_predicted	XM_215812		0.17	1.481	0.039704
A_44_P143567	Fdps	NM_031840	83791	0.17	1.481	0.00414
A_44_P1026233	RGD1306304_predicted	XM_231050		0.17	1.481	0.004722
A_43_P15444	Smo	NM_012807	25273	0.17	1.481	0.0517
A_44_P508695	LOC678931	XM_001053876		0.17	1.481	0.050857
A_44_P372101	AW919076	AW919076	29556	0.17	1.481	0.018487
A_44_P212800	L07402	L07402		0.17	1.481	0.142072
A_44_P490277	RGD1563134_predicted	XM_220690		0.17	1.481	0.007329
A_44_P205551	Rab8b	NM_153317	266688	0.17	1.481	0.008421
A_44_P714446	LOC683960	XM_001068230		0.17	1.481	0.007599
A_44_P450032	RGD1559442_predicted	XM_001077749		0.17	1.481	0.091479
A_44_P123370	Ppm1f	NM_175755	287931	0.17	1.480	0.077701
A_44_P399459	Col4a3bp_predicted	XM_345143		0.17	1.480	0.035609
A_43_P18176	Ube1dc1	NM_001009669	300968	0.17	1.480	0.004846
A_44_P527726	RGD1308469_predicted	XM_228615	317275	0.17	1.480	0.006726
A_44_P366192	Sec61g	XM_346040		0.17	1.480	0.004304
A_44_P550971	RGD1566225_predicted	XM_229139		0.17	1.480	0.300778
A_44_P243321	RGD1310191	XM_341102		0.17	1.480	0.050105
A_44_P487549	RGD1565022_predicted	XM_216007	296580	0.17	1.480	0.006105
A_44_P792034	Mcart1	NM_001024785	313241	0.17	1.480	0.106317
A_44_P1039678	Rda279	XM_340970	245975	0.17	1.480	0.013733
A_44_P208421	Oraov1_predicted	XM_219536		0.17	1.479	0.01501
A_44_P533934	Gdf7	XM_345646	252833	0.17	1.479	0.199527
A_44_P1027274	A_44_P1027274	A_44_P1027274		0.17	1.479	0.032292
A_44_P437038	XM_342816	XM_342816		0.17	1.479	0.01418
A_44_P444759	RGD1310191	XM_341102		0.17	1.479	0.043423
A_44_P869099	LOC680222	XM_001056172	680222	0.17	1.479	0.011809
A_44_P265058	Gmnn_predicted	XM_214477		0.17	1.479	0.067661
A_44_P336608	BI395586	BI395586	300783	0.17	1.479	0.031189
A_44_P1013822	LOC302640	NM_001013960	302640	0.17	1.479	0.002405
A_44_P525557	RGD1359682	NM_001006983	299617	0.17	1.479	0.02644
A_43_P12354	Vps33a	NM_022961	65081	0.17	1.479	0.02284
A_44_P253858	Sumo1	NM_001009672	301442	0.17	1.478	0.054286
A_42_P821898	RGD1561825_predicted	XM_342870	362549	0.17	1.478	0.052972
A_44_P264659	Dpysl3	NM_012934	25418	0.17	1.478	0.051299
A_44_P508307	Rgs11	XM_573061	54291	0.17	1.478	0.074487
A_44_P354806	Vps45	NM_172072	64516	0.17	1.478	0.044361
A_44_P244197	RGD1561116_predicted	XM_343061	362737	0.17	1.478	0.033115
A_44_P311562	XM_341840	XM_341840		0.17	1.478	0.021668
A_43_P15494	Cebpa	NM_012524	24252	0.17	1.478	0.004894
A_43_P16847	Spsb1_predicted	XM_233686		0.17	1.478	0.052972
A_44_P272301	1200013a08rik	NM_001007002	313770	0.17	1.478	0.07653
A_44_P199219	Gadd45gip1	XM_213842	288916	0.17	1.478	0.004224
A_44_P625132	TC550942	TC550942		0.17	1.478	0.016619
A_44_P400801	Zfp184_predicted	XM_225379		0.17	1.477	0.04909
A_44_P1017035	Slc26a4	NM_019214	29440	0.17	1.477	0.094695
A_44_P945248	TC517707	TC517707		0.17	1.477	0.194693
A_44_P840489	TC526915	TC526915		0.17	1.477	0.063326
A_44_P1055681	Prss15	NM_133404	170916	0.17	1.477	0.006959
A_44_P450140	XM_224783	XM_224783		0.17	1.476	0.006557
A_44_P360926	Nalp5_predicted	XM_218237		0.17	1.476	0.002568

A_44_P393064	Clasp1	XM_001053715		0.17	1.476	0.005379
A_42_P788760	Wdr21_predicted	XM_343088		0.17	1.476	0.003658
A_44_P746299	TC541394	TC541394		0.17	1.476	0.061017
A_44_P548319	Nap1l4	NM_001012170	361684	0.17	1.476	0.003743
A_43_P20861	RGD1305779_predicted	XM_234036	313969	0.17	1.476	0.024036
A_44_P260046	LOC680312	XM_001056594		0.17	1.476	0.10634
A_44_P377635	XM_228716	XM_228716		0.17	1.476	0.006313
A_43_P20503	RGD1304878	XM_234506		0.17	1.476	0.053153
A_44_P1008811	BF549878	BF549878		0.17	1.476	0.012027
A_44_P166447	RGD1561264_predicted	XM_233740		0.17	1.476	0.007357
A_42_P741027	Jun	NM_021835	24516	0.17	1.476	0.013974
A_44_P311609	Bccip_predicted	XM_341947		0.17	1.476	0.030356
A_43_P11236	Laptm5	NM_053538	89783	0.17	1.476	0.288521
A_44_P154631	RGD1310263_predicted	XM_234110		0.17	1.476	0.086684
A_44_P980775	TC534269	TC534269		0.17	1.476	0.199354
A_44_P356866	RGD1311078_predicted	XM_001081737		0.17	1.476	0.009347
A_44_P149962	XM_345752	XM_345752		0.17	1.476	0.207375
A_44_P360804	Mpg	NM_012601	24561	0.17	1.476	0.065418
A_44_P503434	E2f6	XM_233986	313978	0.17	1.476	0.028126
A_44_P821829	ENSRNOT00000045141	ENSRNOT00000045141		0.17	1.476	0.476176
A_44_P255495	Spry1_predicted	XM_227050		0.17	1.476	0.005117
A_44_P488475	LOC686590	XM_001072973		0.17	1.475	0.025389
A_44_P454386	Eln	NM_012722	25043	0.17	1.475	0.032029
A_44_P180987	Lrpprc	NM_001008519	313867	0.17	1.475	0.021876
A_44_P337740	Fars2	NM_001013139	306879	0.17	1.475	0.021987
A_42_P707970	Mgat1	NM_030861	81519	0.17	1.475	0.007192
A_44_P752503	AI102173	AI102173		0.17	1.475	0.054273
A_44_P993660	BG666613	BG666613		0.17	1.475	0.038237
A_44_P328340	RGD1561605_predicted	XM_223519	289717	0.17	1.475	0.043538
A_44_P107056	Hmgn2	NM_001025624	114637	0.17	1.475	0.010084
A_44_P949715	TC549858	TC549858		0.17	1.475	0.005574
A_44_P318692	XM_341743	XM_341743		0.17	1.475	0.030819
A_44_P470935	Ewsr1	XM_001068285		0.17	1.474	0.019858
A_43_P11785	Pde4d	NM_017032	24627	0.17	1.474	0.01052
A_44_P630109	A_44_P630109	A_44_P630109		0.17	1.474	0.107753
A_44_P334225	AW141519	AW141519		0.17	1.474	0.040614
A_44_P440987	Bicc1_predicted	XM_342126		0.17	1.474	0.019176
A_42_P721270	Nfix	XM_213849		0.17	1.474	0.01058
A_44_P175461	RGD1565602_predicted	XM_239761		0.17	1.474	0.077069
A_44_P899121	TC554162	TC554162		0.17	1.474	0.058091
A_44_P112061	RGD1308331_predicted	XM_341829	361543	0.17	1.474	0.008879
A_44_P179719	Spop	XM_213437	287643	0.17	1.473	0.015208
A_43_P18203	Sf3a3	NM_001025698	313583	0.17	1.473	0.04119
A_44_P328930	BF555161	BF555161		0.17	1.473	0.043264
A_44_P1058703	RGD1307526	BC092633	315792	0.17	1.473	0.043212
A_42_P530171	Lsm4_predicted	XM_214318		0.17	1.473	0.009954
A_42_P624382	Cdipt	NM_138899	192260	0.17	1.473	0.004601
A_43_P16488	Chrac1_predicted	XM_235400	315058	0.17	1.473	0.019257
A_44_P496668	Zcwpw1_predicted	XM_222057	304368	0.17	1.473	0.049451
A_44_P152445	Eif4e2_predicted	XM_343616		0.17	1.473	0.004655
A_44_P387374	RGD1309443_predicted	XM_221315	287990	0.17	1.473	0.025985
A_44_P178631	Aytl2_predicted	XM_341747	361467	0.17	1.473	0.064006
A_44_P222726	Slc31a2	NM_001033693	298091	0.17	1.473	0.005837
A_44_P544019	Tcf20_mapped	XM_345861	366964	0.17	1.472	0.010804
A_44_P204246	RGD1306346_predicted	XM_221375		0.17	1.472	0.008312
A_44_P898805	A_44_P898805	A_44_P898805		0.17	1.472	0.034843
A_44_P995681	Melk_predicted	XM_342828		0.17	1.472	0.05683
A_44_P336714	RGD1565969_predicted	XM_001065252		0.17	1.472	0.024095
A_44_P1042294	RGD1565549_predicted	XM_240329	306254	0.17	1.472	0.020084
A_44_P993300	RGD1307103_predicted	XM_345486		0.17	1.472	0.008335
A_44_P377373	Olfml1	NM_001013192	361621	0.17	1.472	0.287437
A_44_P397269	LOC361309	NM_001014148	361309	0.17	1.472	0.105089
A_44_P223549	Pdgfb	XM_343293		0.17	1.472	0.244468

A_44_P200647	Hmgb2	XM_573903		0.17	1.472	0.058513
A_44_P421650	Lamp1	NM_012857	25328	0.17	1.472	0.070883
A_44_P553357	RGD1307722_predicted	XM_001075648		0.17	1.472	0.076434
A_44_P868335	ENSRNOT00000011219	ENSRNOT00000011219		0.17	1.471	0.109045
A_44_P447109	AW143170	AW143170		0.17	1.471	0.006617
A_44_P508917	LOC290341	XR_008528	290341	0.17	1.471	0.031409
A_44_P184505	Hbegf	NM_012945	25433	0.17	1.471	0.014364
A_44_P455198	Fbxl17_predicted	XM_237458		0.17	1.471	0.05557
A_43_P11991	Ifrd1	NM_019242	29596	0.17	1.471	0.019344
A_43_P12303	Rpl30	NM_022699	64640	0.17	1.471	0.012733
A_44_P856007	Iqgap1_predicted	XM_001066654		0.17	1.471	0.013181
A_44_P161637	Taf1_predicted	XM_228551	317256	0.17	1.471	0.011241
A_44_P929051	RGD1565435_predicted	XR_008396	502743	0.17	1.471	0.005423
A_44_P140551	Ncln	NM_001014082	314648	0.17	1.471	0.013015
A_44_P413845	BI281756	BI281756	117287	0.17	1.471	0.213627
A_42_P556457	Gmcl1	NM_001033931	312516	0.17	1.470	0.061495
A_44_P954507	TC523052	TC523052		0.17	1.470	0.079437
A_43_P15339	Pitx1	NM_053624	113983	0.17	1.470	0.138929
A_44_P1011989	Mrps12_predicted	XM_214890		0.17	1.470	0.008208
A_44_P556538	Phf3_predicted	XM_001053271		0.17	1.470	0.319454
A_44_P957446	AW143719	AW143719		0.17	1.470	0.099232
A_44_P361124	XM_227645	XM_227645		0.17	1.470	0.008461
A_43_P17812	Wdr47	XM_001071369		0.17	1.470	0.192997
A_43_P20986	Gfod1_predicted	XM_225228	306842	0.17	1.470	0.161758
A_44_P699398	RGD1559144_predicted	XM_001078410		0.17	1.470	0.066369
A_44_P118355	AW921472	AW921472		0.17	1.470	0.027883
A_44_P883659	A_44_P883659	A_44_P883659		0.17	1.470	0.008401
A_43_P10314	LOC499076	XM_574359	499076	0.17	1.470	0.006016
A_42_P675659	Commd10	NM_001004276	361323	0.17	1.469	0.026734
A_44_P113096	X68396	X68396		0.17	1.469	0.023765
A_44_P744512	LOC680511	XM_001057500		0.17	1.469	0.098928
A_44_P481086	Zw10	NM_001024801	363059	0.17	1.469	0.023173
A_44_P468573	LOC289517	XR_008796	289517	0.17	1.469	0.017165
A_43_P17071	Gapvd1_predicted	XM_231161	311880	0.17	1.469	0.003709
A_44_P286695	Rfng	NM_021849	60433	0.17	1.469	0.025414
A_44_P901617	TC560500	TC560500		0.17	1.469	0.005459
A_44_P370319	XM_219295	XM_219295		0.17	1.469	0.041462
A_44_P462823	Nub1	NM_001013925	296731	0.17	1.468	0.081713
A_44_P473443	ENSRNOT00000019331	ENSRNOT00000019331		0.17	1.468	0.003979
A_43_P21781	RGD1309437	NM_001013866	288176	0.17	1.468	0.014627
A_43_P11201	MGC112682	NM_001017473	497900	0.17	1.468	0.00918
A_44_P439276	Rbm24_predicted	XM_214446	291031	0.17	1.468	0.037171
A_44_P393829	Ttf2_predicted	XM_215670		0.17	1.468	0.017815
A_44_P513577	Cdc2l6_predicted	XM_228203		0.17	1.468	0.015922
A_44_P480939	Zfp287_predicted	XM_220549		0.17	1.468	0.027253
A_44_P140532	XM_345678	XM_345678		0.17	1.468	0.007062
A_44_P1024113	Cd2bp2_predicted	XM_215082		0.17	1.468	0.0056
A_44_P244076	CB548506	CB548506		0.17	1.468	0.047573
A_42_P471578	Tsc2	NM_012680	24855	0.17	1.468	0.016978
A_42_P625181	Hspa4	NM_153629	266759	0.17	1.468	0.013608
A_44_P487394	Cln3	NM_001006971	293485	0.17	1.467	0.033866
A_44_P320687	Llgl1	NM_152844	54265	0.17	1.467	0.02
A_44_P525519	Atad3a	NM_001034922	298682	0.17	1.467	0.019475
A_44_P903977	TC532772	TC532772		0.17	1.467	0.128981
A_44_P316504	Tmed9	NM_001009703	361207	0.17	1.467	0.010436
A_44_P106248	M95791	M95791		0.17	1.467	0.007692
A_44_P274756	Cdcp1_predicted	XM_236747		0.17	1.467	0.035974
A_44_P290905	LOC690662	XM_001075132		0.17	1.467	0.003253
A_43_P16751	LOC500199	XM_001063161		0.17	1.466	0.01041
A_44_P356513	RGD1306601_predicted	XM_230527		0.17	1.466	0.033869
A_44_P215544	RGD1565920_predicted	XM_344713		0.17	1.466	0.004145
A_44_P526975	Hcr	NM_001002822	406196	0.17	1.466	0.008127
A_44_P394011	Hrpap20	NM_198783	362495	0.17	1.466	0.017435

A_44_P652690	TC535527	TC535527		0.17	1.466	0.003049
A_44_P225691	Znf142_predicted	XM_237291		0.17	1.466	0.022104
A_44_P177747	Ap1gbp1	XM_573165		0.17	1.465	0.070476
A_43_P12189	Tsnax	NM_022262	64028	0.17	1.465	0.01089
A_44_P455028	Lyk4	NM_183056	360230	0.17	1.465	0.014282
A_44_P302225	Mina	NM_153309	266670	0.17	1.465	0.21199
A_44_P318883	RGD1560648_predicted	XM_344518		0.17	1.465	0.017254
A_44_P993791	Pcdh1_predicted	XM_225997	307481	0.17	1.464	0.038972
A_42_P620591	Arpc3_predicted	XM_213782		0.17	1.464	0.00508
A_44_P1008050	Ncoa4_predicted	NM_001034008	619385	0.17	1.464	0.041456
A_44_P1051316	TC518081	TC518081		0.17	1.464	0.01136
A_44_P593949	TC531218	TC531218		0.17	1.464	0.150783
A_44_P212878	Nfyb	NM_031553	25336	0.17	1.464	0.017935
A_44_P534249	Ociad1	NM_001013874	289590	0.17	1.464	0.048647
A_43_P10479	RGD1559787_predicted	XM_576514		0.17	1.464	0.013324
A_42_P504507	AW144699	AW144699	362704	0.17	1.463	0.006708
A_44_P545895	LOC289134	XR_008864	289134	0.17	1.463	0.024557
A_44_P381360	AW915808	AW915808		0.17	1.463	0.013755
A_44_P996917	RGD1311703	NM_001013898	293160	0.17	1.463	0.02354
A_44_P767445	A_44_P767445	A_44_P767445		0.17	1.463	0.149129
A_43_P18631	RGD1560268_predicted	XM_226464	307829	0.17	1.463	0.036781
A_44_P459484	A_44_P459484	A_44_P459484		0.17	1.463	0.127912
A_44_P192058	RGD1308918_predicted	XM_219441		0.17	1.463	0.105098
A_44_P265423	RGD1307222_predicted	XM_220693	303300	0.17	1.463	0.014949
A_42_P458880	Elk4_predicted	XM_222630		0.17	1.463	0.00869
A_44_P802937	Gnb1	NM_030987	24400	0.17	1.463	0.017678
A_44_P428724	Stfa3_predicted	XM_213617		0.17	1.463	0.101117
A_44_P300110	Zfyve26_predicted	XM_234335		0.17	1.462	0.040528
A_44_P424570	Poldip3_predicted	XM_235521	315170	0.16	1.462	0.020471
A_44_P414299	Pdzk1ip1	NM_130401	81916	0.16	1.462	0.003649
A_44_P481381	Add2	NM_012491	24171	0.16	1.462	0.137799
A_44_P1045157	Mtfr1_predicted	XM_001055144		0.16	1.462	0.050802
A_44_P419922	Haghl	NM_001013114	302995	0.16	1.462	0.045495
A_44_P675031	CO383678	CO383678		0.16	1.462	0.036487
A_44_P776335	DY309796	DY309796		0.16	1.462	0.008136
A_44_P793008	TC526524	TC526524		0.16	1.462	0.018773
A_44_P499242	XM_226008	XM_226008		0.16	1.462	0.004103
A_43_P10249	RGD1307648	NM_001013905	294004	0.16	1.462	0.008
A_43_P19628	RGD1560084_predicted	XM_223280	289515	0.16	1.462	0.130591
A_44_P423992	ENSRNOT00000020651	ENSRNOT00000020651		0.16	1.461	0.072261
A_42_P669004	BG663343	BG663343	296136	0.16	1.461	0.017663
A_42_P808945	Mustn1	NM_181368	290553	0.16	1.461	0.050977
A_42_P746089	LOC680891	XM_001059359		0.16	1.461	0.019517
A_44_P473449	XM_227913	XM_227913		0.16	1.461	0.008938
A_44_P165039	LOC687202	XM_001077533		0.16	1.461	0.004737
A_44_P837740	Asb6	NM_001011963	296627	0.16	1.461	0.004316
A_44_P992306	Gnpda2_predicted	XM_214029		0.16	1.461	0.088867
A_44_P897537	Tcea1	NM_001025735	362479	0.16	1.461	0.024759
A_44_P359618	Zfp265	NM_031616	58821	0.16	1.460	0.026252
A_44_P274021	Myh11	XM_001053321		0.16	1.460	0.079032
A_43_P20976	RGD1563910_predicted	XM_342608	362295	0.16	1.460	0.012522
A_42_P724535	Pomgnt1	NM_001007747	362567	0.16	1.460	0.009067
A_44_P291065	ENSRNOT00000011337	ENSRNOT00000011337		0.16	1.460	0.112575
A_44_P133060	Nipa1_predicted	XM_218719		0.16	1.460	0.04667
A_44_P432095	AA859319	AA859319		0.16	1.460	0.106972
A_44_P543505	RGD1305713	NM_001024749	293059	0.16	1.460	0.004569
A_44_P1036591	Tmem39b	NM_001014192	362608	0.16	1.460	0.024025
A_44_P105727	RGD1565360_predicted	XM_221630	288240	0.16	1.459	0.022858
A_42_P544402	Lztr2	NM_053571	89868	0.16	1.459	0.026217
A_44_P463262	BG665360	BG665360		0.16	1.459	0.194996
A_43_P12243	Prdx3	NM_022540	64371	0.16	1.459	0.025562
A_44_P975167	Hist1h2an_predicted	XM_225386		0.16	1.459	0.099284
A_44_P427003	RGD1306472_predicted	XM_235542		0.16	1.459	0.222034

A_44_P301812	Rap1a	NM_001005765	295347	0.16	1.459	0.022379
A_44_P343797	Ubxd2	NM_001012025	304766	0.16	1.459	0.008348
A_42_P585155	Fxc1	NM_053371	84384	0.16	1.459	0.004258
A_44_P160349	Nsun4_predicted	XM_238407		0.16	1.459	0.050142
A_44_P391596	Tbc1d7_predicted	XM_341510		0.16	1.459	0.013607
A_44_P990937	Smarca4	XM_343358	171379	0.16	1.459	0.003844
A_44_P379797	XM_222771	XM_222771		0.16	1.458	0.002388
A_42_P794052	LOC691221	XM_001077260	691221	0.16	1.458	0.0773
A_44_P942245	Aytl2_predicted	XM_341747	361467	0.16	1.458	0.100483
A_44_P108697	AW918624	AW918624		0.16	1.458	0.024658
A_42_P616384	Trappc6b_predicted	XM_216708		0.16	1.458	0.038738
A_44_P377579	XM_230447	XM_230447		0.16	1.458	0.124078
A_44_P834642	M89646	M89646	81776	0.16	1.458	0.016151
A_44_P387324	LOC363251	NM_001014217	363251	0.16	1.458	0.019219
A_44_P503428	Umpk_predicted	XM_222855		0.16	1.458	0.03095
A_44_P355350	BI303634	BI303634	362884	0.16	1.458	0.053865
A_44_P146703	Pccb	NM_017030	24624	0.16	1.458	0.063792
A_44_P802347	BG672648	BG672648	302363	0.16	1.458	0.039561
A_44_P531741	Masp1	AY149996	64023	0.16	1.458	0.123332
A_42_P577431	LOC680611	XM_001057993		0.16	1.458	0.009929
A_44_P316422	A_44_P316422	A_44_P316422		0.16	1.458	0.193713
A_44_P354335	RGD1305692	NM_001033070	362819	0.16	1.458	0.00369
A_42_P704383	Bre	NM_199270	362704	0.16	1.458	0.011799
A_44_P208049	Sh2bpsm1	NM_134456	89817	0.16	1.457	0.017659
A_44_P321675	Map3k3_predicted	XM_221034		0.16	1.457	0.020476
A_44_P1031737	Pecr	NM_133299	113956	0.16	1.457	0.117034
A_44_P249704	Bhlhb9	NM_207611	317407	0.16	1.457	0.048279
A_44_P131796	M16433	M16433		0.16	1.457	0.051719
A_44_P176766	AI228110	AI228110	50555	0.16	1.457	0.15102
A_44_P408050	Ckap4_predicted	XM_343189		0.16	1.457	0.033237
A_44_P1045759	RGD1309104_predicted	XM_213896		0.16	1.457	0.056742
A_44_P466111	Nfib	XM_342854		0.16	1.457	0.024112
A_43_P20534	Fbxo22	NM_001037770	300724	0.16	1.457	0.017674
A_44_P402956	Prkacb_predicted	XM_215070		0.16	1.457	0.020217
A_44_P412647	RGD1311055_predicted	XM_226534		0.16	1.457	0.007629
A_44_P957913	BF289621	BF289621	313609	0.16	1.457	0.078172
A_43_P16816	LOC686883	XM_001076161		0.16	1.457	0.012032
A_44_P762292	Serpinf2	NM_001011892	287527	0.16	1.457	0.031503
A_44_P370335	Lyar	NM_001011911	289707	0.16	1.457	0.074222
A_44_P804200	Atg16l1_predicted	XM_001067113		0.16	1.456	0.004293
A_44_P472583	AW914016	AW914016	362013	0.16	1.456	0.026328
A_43_P13056	Il10ra	NM_057193	117539	0.16	1.456	0.112135
A_44_P426924	LOC689296	XM_001070314		0.16	1.456	0.153848
A_44_P555473	Rent1_predicted	XM_224732	684527	0.16	1.456	0.010566
A_44_P1024714	Tbl1x_predicted	XM_217623		0.16	1.456	0.004265
A_44_P550626	Fbxo28_predicted	XM_223125		0.16	1.456	0.009182
A_44_P579360	TC556218	TC556218		0.16	1.456	0.004451
A_42_P712162	AW143907	AW143907		0.16	1.455	0.027382
A_44_P519698	AW914793	AW914793		0.16	1.455	0.031585
A_44_P241862	Top1	NM_022615	64550	0.16	1.455	0.020965
A_44_P897008	AW141286	AW141286		0.16	1.455	0.017108
A_44_P260099	Ppap2c	NM_139252	246115	0.16	1.455	0.09798
A_44_P727641	AI045193	AI045193		0.16	1.455	0.057616
A_44_P475795	Rnf44	NM_001024795	361212	0.16	1.455	0.117827
A_44_P929396	ENSRNOT00000034494	ENSRNOT00000034494		0.16	1.455	0.014182
A_44_P336429	Hdgf	NM_053707	114499	0.16	1.454	0.02863
A_43_P17836	CB545323	CB545323	297911	0.16	1.454	0.081406
A_44_P163189	Stxbp5	NM_178345	81022	0.16	1.454	0.065464
A_44_P806154	A_44_P806154	A_44_P806154		0.16	1.454	0.170903
A_44_P729572	AI230668	AI230668	361400	0.16	1.454	0.024952
A_44_P776088	TC536242	TC536242		0.16	1.454	0.015068
A_43_P13564	Mapkbp1_predicted	XM_342498		0.16	1.454	0.026109
A_44_P523629	CB547165	CB547165	298017	0.16	1.454	0.043222

A_44_P438667	Mcart1	NM_001024785	313241	0.16	1.454	0.086329
A_43_P12873	Csnk1a1	NM_053615	113927	0.16	1.454	0.019517
A_44_P346938	RGD1309313	NM_001014030	309454	0.16	1.453	0.010098
A_44_P778557	TC528927	TC528927		0.16	1.453	0.064742
A_44_P192134	Mapk8	XM_341399	116554	0.16	1.453	0.027621
A_44_P308932	Csk	NM_001030039	315707	0.16	1.453	0.025929
A_44_P768830	TC544943	TC544943		0.16	1.453	0.016105
A_44_P271946	RGD1305166_predicted	XM_220007	309486	0.16	1.452	0.00752
A_42_P556888	Chrd	XM_221307	117275	0.16	1.452	0.010587
A_42_P622574	Daxx	NM_080891	140926	0.16	1.452	0.00434
A_44_P241599	RGD1559144_predicted	XM_236726	301076	0.16	1.452	0.038277
A_44_P227083	Klhl5	XM_223418		0.16	1.452	0.041953
A_43_P12811	Srm	NM_053464	84596	0.16	1.452	0.043005
A_44_P775982	Armc9_predicted	XM_217465		0.16	1.452	0.028608
A_43_P20296	RGD1309207	NM_001014196	362739	0.16	1.452	0.078671
A_44_P361075	LOC294903	XR_007618	294903	0.16	1.451	0.058835
A_44_P321176	hr	NM_024364	60563	0.16	1.451	0.031666
A_44_P489500	Acvr2a	NM_031571	29263	0.16	1.451	0.229027
A_44_P1019923	Casp2	NM_022522	64314	0.16	1.451	0.014447
A_44_P792035	Mcart1	NM_001024785	313241	0.16	1.451	0.014263
A_44_P269940	Ythdf2_predicted	XM_232772		0.16	1.451	0.00604
A_44_P1027964	RGD1305311_predicted	XM_237386	316616	0.16	1.451	0.102718
A_44_P333109	LOC680203	XM_001056106		0.16	1.451	0.181735
A_43_P14264	Vegfc	NM_053653	114111	0.16	1.451	0.025541
A_44_P140378	RGD1559986_predicted	XM_347039	362294	0.16	1.451	0.008906
A_44_P312546	Aldh2	NM_032416	29539	0.16	1.451	0.022673
A_44_P480653	Pon2	NM_001013082	296851	0.16	1.450	0.100803
A_43_P17821	Zfml_predicted	XM_242556		0.16	1.450	0.040569
A_44_P959944	TC518223	TC518223		0.16	1.450	0.009866
A_44_P330904	XM_343775	XM_343775		0.16	1.450	0.083066
A_44_P309650	RGD1305327	NM_001012747	362955	0.16	1.450	0.013755
A_44_P836925	RGD1559908_predicted	XM_001066920		0.16	1.450	0.023199
A_43_P10825	TC521024	TC521024		0.16	1.450	0.065397
A_44_P469357	RGD1565840_predicted	XM_215794	295975	0.16	1.450	0.008509
A_44_P520175	Masp1	AJ487622	64023	0.16	1.450	0.481099
A_44_P119527	Crsp6	XM_217086		0.16	1.449	0.031937
A_44_P509473	Csnk1d	NM_139060	64462	0.16	1.449	0.012048
A_44_P118082	Gprk2l	NM_022928	59077	0.16	1.449	0.005573
A_44_P635423	AW141130	AW141130		0.16	1.449	0.330905
A_44_P277126	Senp5_predicted	XM_221369	303874	0.16	1.448	0.015652
A_44_P621723	ENSRNOT00000029566	ENSRNOT00000029566		0.16	1.448	0.03014
A_44_P852862	TC535593	TC535593		0.16	1.448	0.088927
A_44_P218097	XM_342889	XM_342889		0.16	1.448	0.023677
A_44_P266984	R1b	NM_001017480	497985	0.16	1.448	0.009348
A_44_P812604	TC533089	TC533089		0.16	1.448	0.022429
A_44_P826338	TC563754	TC563754		0.16	1.448	0.181149
A_44_P107219	Gltscr2	NM_207591	292624	0.16	1.448	0.021696
A_44_P500527	AI227763	AI227763		0.16	1.447	0.03261
A_44_P178448	Srebf1	XM_213329	78968	0.16	1.447	0.023516
A_43_P19306	RGD1309501_predicted	XM_223660	305552	0.16	1.447	0.154573
A_44_P340256	Dgat2l4_predicted	XM_228583		0.16	1.447	0.065662
A_44_P185311	XM_343845	XM_343845		0.16	1.447	0.142663
A_44_P163396	Hrsp12	NM_031714	65151	0.16	1.447	0.036532
A_44_P746333	TC541438	TC541438		0.16	1.447	0.088864
A_43_P11259	Mrpl19	NM_001029898	297372	0.16	1.447	0.037284
A_44_P766913	TC523894	TC523894		0.16	1.447	0.043506
A_44_P168280	RGD1561179_predicted	XM_574742		0.16	1.447	0.09825
A_44_P286888	Vapa	NM_031631	58857	0.16	1.447	0.122398
A_43_P19947	Dhx36_predicted	XM_227203		0.16	1.447	0.007755
A_44_P884308	TC555681	TC555681		0.16	1.447	0.28166
A_44_P513114	Trim3	NM_031786	83616	0.16	1.447	0.060991
A_44_P816687	A_44_P816687	A_44_P816687		0.16	1.447	0.017015
A_43_P17330	Nup35	NM_001004229	295692	0.16	1.446	0.014908

A_44_P532096	LOC290577	XR_006087	290577	0.16	1.446	0.059198
A_44_P929119	RGD1560353_predicted	XM_575626		0.16	1.446	0.009827
A_43_P11363	LOC363188	XM_001061775		0.16	1.446	0.040457
A_44_P144237	Erp29	NM_053961	117030	0.16	1.446	0.016432
A_44_P520509	Surf4	NM_001033868	619346	0.16	1.446	0.026054
A_44_P346572	Ssr1	NM_001008891	361233	0.16	1.446	0.043574
A_44_P237153	Birc5	NM_022274	64041	0.16	1.446	0.036784
A_44_P137911	LOC679811	XM_001054550	679811	0.16	1.446	0.021583
A_43_P17939	TC525627	TC525627		0.16	1.446	0.062066
A_44_P463302	LOC689025	XM_001069213	689025	0.16	1.446	0.004737
A_42_P604490	Faim	NM_080895	140930	0.16	1.446	0.031534
A_44_P421625	Cspg6	NM_031583	29486	0.16	1.446	0.039171
A_44_P126406	XM_228958	XM_228958		0.16	1.446	0.00532
A_42_P529681	TC539193	TC539193		0.16	1.446	0.019906
A_44_P216511	AW918690	AW918690	502782	0.16	1.445	0.307947
A_44_P464231	A_44_P464231	A_44_P464231		0.16	1.445	0.121762
A_44_P513886	XM_573956	XM_573956		0.16	1.445	0.028247
A_44_P999869	Ptpn11	NM_013088	25622	0.16	1.445	0.005275
A_44_P761444	TC555919	TC555919		0.16	1.445	0.415051
A_44_P260475	Hdac11_predicted	XM_238362		0.16	1.445	0.207393
A_44_P359156	XM_237323	XM_237323		0.16	1.444	0.020293
A_44_P204109	RGD1560796_predicted	XM_236387		0.16	1.444	0.150139
A_44_P335734	Pigq	NM_001007607	287159	0.16	1.444	0.009569
A_44_P1041495	AW143783	AW143783		0.16	1.444	0.262715
A_44_P503261	Snai2	NM_013035	25554	0.16	1.444	0.208734
A_42_P645510	RGD1310427_predicted	XM_342959		0.16	1.444	0.019884
A_42_P487432	RGD1304977_predicted	XM_238325	296634	0.16	1.444	0.041966
A_44_P511333	RGD1560227_predicted	XM_229677		0.16	1.443	0.062671
A_44_P148695	RGD1307915_predicted	XM_001057982		0.16	1.443	0.009648
A_43_P21931	LOC315508	NM_001034000	315508	0.16	1.443	0.010671
A_42_P554336	Secp43	NM_023027	65241	0.16	1.443	0.008777
A_44_P996589	Nbn	NM_138873	85482	0.16	1.443	0.028525
A_44_P752342	BF563060	BF563060		0.16	1.443	0.012496
A_44_P501827	Cpsf6_predicted	XM_216893		0.16	1.443	0.058231
A_44_P823633	TC524738	TC524738		0.16	1.443	0.215836
A_44_P412831	Lsm7_predicted	XM_343157		0.16	1.443	0.005799
A_44_P444034	LOC680258	XM_001056356	680258	0.16	1.443	0.026543
A_44_P935273	Mthfs	NM_001009349	300886	0.16	1.443	0.028258
A_44_P1042901	Pum2	XM_216661		0.16	1.443	0.011778
A_44_P313761	Mgat1	NM_030861	81519	0.16	1.443	0.008053
A_44_P102061	Zc3h7b_predicted	XM_243630	315158	0.16	1.443	0.007056
A_43_P12664	Ggcx	NM_031756	81716	0.16	1.443	0.022377
A_44_P290706	XM_226680	XM_226680		0.16	1.443	0.030548
A_44_P194836	Zdhhc3	NM_001039014	301081	0.16	1.442	0.031669
A_44_P557840	A_44_P557840	A_44_P557840		0.16	1.442	0.458232
A_44_P450486	Ttll1	NM_001012200	362969	0.16	1.442	0.042786
A_43_P12631	Capn10	NM_031673	63834	0.16	1.442	0.020205
A_42_P769290	AW532904	AW532904	116561	0.16	1.442	0.011925
A_44_P830170	TC524570	TC524570		0.16	1.442	0.06498
A_44_P393282	40065	NM_176856	83788	0.16	1.442	0.022022
A_44_P521399	Rasl10b_predicted	XM_001081057		0.16	1.442	0.099592
A_42_P570241	Pbef1	NM_177928	297508	0.16	1.442	0.085713
A_44_P430345	Prmt4	NM_001030041	363026	0.16	1.441	0.010122
A_44_P933149	TC524731	TC524731		0.16	1.441	0.298099
A_44_P427695	Tor3a	NM_001009683	304884	0.16	1.441	0.052058
A_44_P322108	AW915738	AW915738		0.16	1.441	0.02281
A_44_P311642	Supt16h_predicted	XM_223981		0.16	1.441	0.032044
A_44_P219919	Psme4	XM_001055468	498433	0.16	1.441	0.031343
A_44_P465329	Mal	NM_012798	25263	0.16	1.441	0.344759
A_44_P300171	Gcat	NM_001024277	366959	0.16	1.441	0.00606
A_44_P741709	BF551036	BF551036		0.16	1.441	0.030636
A_44_P454847	Ptpmt1	XM_342460		0.16	1.441	0.021284
A_44_P173478	Cebpz_predicted	XM_343002		0.16	1.441	0.009866

A_44_P248542	XM_234091	XM_234091		0.16	1.441	0.023138
A_43_P12839	Sybl1	NM_053531	85491	0.16	1.441	0.030312
A_44_P126078	RGD1310257_predicted	XM_218322	308381	0.16	1.441	0.03415
A_44_P935107	ENSRNOT00000009270	ENSRNOT00000009270		0.16	1.441	0.040727
A_44_P377428	Nhlrc2_predicted	XM_217640		0.16	1.440	0.100492
A_44_P447539	Vamp2	NM_012663	24803	0.16	1.440	0.016182
A_44_P135883	Rbm7_predicted	XM_236212		0.16	1.440	0.077942
A_44_P424135	Ms4a3_predicted	XM_219599	293753	0.16	1.440	0.035586
A_44_P755663	A_44_P755663	A_44_P755663		0.16	1.440	0.055704
A_44_P619541	Dnajc8	NM_001013168	313035	0.16	1.440	0.012296
A_44_P118666	Hs3st1	NM_053391	84406	0.16	1.440	0.012607
A_44_P226963	LOC685867	XM_001065562		0.16	1.440	0.042496
A_44_P472381	Josd2_predicted	XM_214929		0.16	1.440	0.002587
A_44_P747226	TC542677	TC542677		0.16	1.440	0.062071
A_44_P807990	TC541703	TC541703		0.16	1.440	0.029591
A_44_P323634	LOC685245	XM_001062991	685245	0.16	1.440	0.013511
A_44_P958966	LOC360902	XR_005722	360902	0.16	1.440	0.044756
A_44_P139742	Npc2	NM_173118	286898	0.16	1.440	0.063326
A_44_P384425	Ranbp6_predicted	XM_219796		0.16	1.440	0.007002
A_44_P344492	Stat4	NM_001012226	367264	0.16	1.440	0.063558
A_44_P215913	Ush1g_predicted	XM_221106		0.16	1.440	0.119368
A_44_P1018394	Apoa1bp_predicted	XM_215635		0.16	1.439	0.020134
A_44_P792230	TC522584	TC522584		0.16	1.439	0.01643
A_44_P149098	Fez2	NM_053600	94269	0.16	1.439	0.017933
A_44_P423921	Dll1	NM_032063	84010	0.16	1.439	0.011014
A_44_P715184	Kif3b_predicted	XM_001061444		0.16	1.439	0.150713
A_42_P732259	RGD1304835	NM_001024976	294430	0.16	1.439	0.274072
A_44_P236815	Xylb	NM_001033704	316067	0.16	1.439	0.011576
A_44_P158777	Prkacb_predicted	X53261	293508	0.16	1.439	0.127543
A_44_P217774	Lysmd3	NM_001009698	315923	0.16	1.439	0.130303
A_44_P1014383	LOC315970	NM_001014097	315970	0.16	1.439	0.008983
A_44_P1029472	RGD1305592	NM_001013901	293500	0.16	1.439	0.008888
A_44_P975304	TC537306	TC537306		0.16	1.439	0.036985
A_44_P489995	Mrps33_predicted	XM_216135		0.16	1.439	0.022931
A_43_P17527	Trnt1	NM_001024261	312616	0.16	1.439	0.025334
A_44_P364560	AW916971	AW916971		0.16	1.438	0.031281
A_43_P20065	Dbt	XM_342328		0.16	1.438	0.040441
A_44_P1034541	Pim3	NM_022602	64534	0.16	1.438	0.235785
A_43_P13046	Sec22l2	NM_057147	117513	0.16	1.438	0.015787
A_44_P482807	RGD1305314	NM_001030038	309478	0.16	1.438	0.075625
A_44_P413540	CV080220	CV080220		0.16	1.438	0.013287
A_44_P206552	Slfn5_predicted	XM_220775	303377	0.16	1.438	0.012585
A_44_P995316	Sgpp1	XM_343081	81536	0.16	1.438	0.060244
A_44_P185459	Zfp39_predicted	XM_220504		0.16	1.438	0.025964
A_44_P111435	AW143657	AW143657	313914	0.16	1.438	0.034574
A_43_P12712	Impa1	NM_032057	83523	0.16	1.437	0.104366
A_44_P498712	Ke2	NM_212506	309629	0.16	1.437	0.024285
A_43_P15715	Psmd2	NM_001031639	287984	0.16	1.437	0.023961
A_44_P336699	Ripx	NM_001025127	360921	0.16	1.437	0.017017
A_44_P605826	ENSRNOT00000029547	ENSRNOT00000029547		0.16	1.437	0.142462
A_44_P695752	BG379104	BG379104		0.16	1.437	0.023538
A_43_P18101	Elf3	NM_001024768	304815	0.16	1.437	0.243578
A_44_P247871	Polk	XM_342178	171525	0.16	1.437	0.026846
A_42_P609658	Psmc2	NM_033236	25581	0.16	1.437	0.01136
A_42_P542960	RGD1562829_predicted	XM_001072746		0.16	1.437	0.09453
A_43_P15719	Tra1_predicted	NM_001012197	362862	0.16	1.437	0.012433
A_44_P210410	Tubb2b	NM_001013886	291081	0.16	1.437	0.017191
A_44_P149148	Ppp3r1	NM_017309	29748	0.16	1.437	0.025847
A_44_P808324	TC524779	TC524779		0.16	1.437	0.089943
A_44_P316273	A_44_P316273	A_44_P316273		0.16	1.437	0.0148
A_43_P18538	Asb6	NM_001011963	296627	0.16	1.436	0.007539
A_44_P337365	Fcer2a	NM_133550	171075	0.16	1.436	0.045031
A_44_P288809	Wdr24	XM_001061380		0.16	1.436	0.008293

A_44_P173960	S68944	S68944		0.16	1.436	0.242055
A_44_P473098	LOC679983	XM_001055174	679983	0.16	1.436	0.083873
A_44_P482796	LOC681361	XM_001061408	681361	0.16	1.436	0.014217
A_44_P176042	LOC688900	XM_001068751	688900	0.16	1.436	0.048351
A_44_P173144	XM_219574	XM_219574		0.16	1.436	0.01608
A_44_P221588	Ppp3ca	AY724517	24674	0.16	1.435	0.003377
A_44_P933459	RGD1308874	NM_001034003	366227	0.16	1.435	0.008314
A_43_P16889	RGD1307929	XM_220636	303280	0.16	1.435	0.109671
A_44_P540855	Actn1	NM_031005	81634	0.16	1.435	0.007899
A_43_P15548	Gabra6	NM_021841	29708	0.16	1.435	0.138087
A_43_P19245	BF524882	BF524882	308669	0.16	1.435	0.028877
A_44_P205908	Psg4	NM_145780	252924	0.16	1.435	0.027832
A_44_P323285	Slc8a1	NM_019268	29715	0.16	1.434	0.252118
A_43_P16171	AF473843	AF473843		0.16	1.434	0.03443
A_42_P815827	Ube2a	NM_001013933	298317	0.16	1.434	0.130432
A_44_P223978	Cdkn1c	NM_001033757	246060	0.16	1.434	0.248259
A_43_P12242	Ppap2a	NM_022538	64369	0.16	1.434	0.069763
A_44_P790508	ENSRNOT00000026119	ENSRNOT00000026119		0.16	1.434	0.008477
A_42_P467037	Prkr	NM_019335	54287	0.16	1.434	0.115898
A_44_P158012	Tpr	XM_222745		0.16	1.434	0.007943
A_44_P236636	LOC367796	XR_007347	367796	0.16	1.434	0.004769
A_44_P959608	ENSRNOT00000036540	ENSRNOT00000036540		0.16	1.434	0.004645
A_44_P728469	Pter	NM_022224	63852	0.16	1.434	0.009346
A_44_P591698	TC538440	TC538440		0.16	1.434	0.03021
A_44_P186860	Nfkbib	NM_030867	81525	0.16	1.434	0.017524
A_44_P493606	Camk2n1	NM_173337	287005	0.16	1.434	0.005698
A_44_P187080	Atp2a2	NM_017290	29693	0.16	1.433	0.005235
A_43_P22189	XM_233843	XM_233843		0.16	1.433	0.032892
A_44_P835235	AW142956	AW142956	261737	0.16	1.433	0.103042
A_44_P107423	Nup153	XM_341508		0.16	1.433	0.023962
A_42_P759427	Strn4_predicted	XM_218432		0.16	1.433	0.018151
A_44_P508379	Cd37	NM_017124	29185	0.16	1.433	0.106482
A_44_P413543	CV080220	CV080220		0.16	1.433	0.08111
A_44_P651575	AW915359	AW915359		0.16	1.433	0.016437
A_44_P161535	LOC364882	XR_007897	364882	0.16	1.432	0.018448
A_44_P247411	Aprin_predicted	XM_221833		0.16	1.432	0.012847
A_44_P224744	LOC683414	XM_001065821		0.16	1.432	0.044791
A_44_P586876	LOC681708	XM_001058055		0.16	1.432	0.004725
A_44_P960333	TC555275	TC555275		0.16	1.432	0.038877
A_44_P1008163	Gas8	NM_001039030	361438	0.16	1.432	0.139143
A_43_P15312	Gna12	NM_031034	81663	0.16	1.432	0.018898
A_44_P303393	Dnajc9_predicted	XM_344286		0.16	1.432	0.015546
A_43_P13362	Rbm16	NM_139094	245926	0.16	1.431	0.012159
A_44_P267349	XM_215650	XM_215650		0.16	1.431	0.007288
A_44_P235919	Ccng1	NM_012923	25405	0.16	1.431	0.100019
A_44_P506317	LOC499768	XM_575106	499768	0.16	1.431	0.009513
A_44_P283784	Nmd3_predicted	XM_001067083		0.16	1.431	0.073331
A_44_P125432	Ubtf	XM_340913		0.16	1.431	0.025712
A_44_P452936	Foxk2_predicted	XM_221212		0.16	1.431	0.039744
A_44_P483888	LOC681973	XM_001059147		0.16	1.431	0.132298
A_44_P994095	RGD1306614	NM_001034911	293454	0.16	1.431	0.005336
A_44_P124259	LOC681258	XM_001060967		0.16	1.431	0.021378
A_42_P756350	Cdkl3	NM_021772	60396	0.16	1.431	0.207974
A_43_P11265	Nono	NM_001012356	317259	0.16	1.430	0.019779
A_42_P714166	MGC108827	NM_001017491	498232	0.16	1.430	0.102257
A_44_P821396	RGD1564625_predicted	XM_220013	309475	0.16	1.430	0.07618
A_44_P972824	Dnm1l	NM_053655	114114	0.16	1.430	0.045826
A_44_P419291	Chn2	NM_032084	84031	0.16	1.430	0.196032
A_44_P552794	Hist1h4b	NM_022686	64627	0.16	1.430	0.104699
A_42_P640923	Cflar	NM_057138	117279	0.16	1.430	0.024954
A_44_P284180	XM_237391	XM_237391		0.16	1.430	0.049054
A_44_P420946	Armc8_predicted	XM_236599	315949	0.16	1.430	0.117891
A_44_P758613	AA946149	AA946149		0.16	1.430	0.068961

A_44_P1004150	Vrk1	NM_001012194	362779	0.16	1.430	0.041614
A_42_P708952	Zbtb8os_predicted	XM_001060735		0.16	1.430	0.052037
A_44_P222566	XM_225097	XM_225097		0.16	1.429	0.047572
A_44_P446801	BE112918	BE112918		0.16	1.429	0.128795
A_44_P245916	Cept1	NM_001007699	310773	0.16	1.429	0.145404
A_43_P21031	Pex5_predicted	XM_232343	312703	0.16	1.429	0.024502
A_44_P535706	Magoh_predicted	XM_216485	298385	0.16	1.429	0.074418
A_44_P493665	AW143071	AW143071	25573	0.16	1.429	0.017143
A_44_P803793	BF548601	BF548601		0.16	1.429	0.309219
A_44_P515247	Tpd52l2	NM_198744	296480	0.16	1.429	0.011682
A_44_P525645	RGD1305362_predicted	XM_220442	303155	0.15	1.429	0.032132
A_42_P626033	Txnl2	NM_032614	58815	0.15	1.429	0.03688
A_44_P196805	Golga7	NM_001007731	361171	0.15	1.429	0.0078
A_43_P19785	AW920545	AW920545		0.15	1.429	0.00962
A_44_P125564	Bok	NM_017312	29884	0.15	1.429	0.031092
A_44_P478278	Terf2_predicted	XM_341683		0.15	1.428	0.010802
A_44_P463557	Rpn1	NM_013067	25596	0.15	1.428	0.036615
A_44_P220647	Zbtb11_predicted	XM_221562		0.15	1.428	0.109202
A_44_P699377	LOC681004	XM_001057305	681004	0.15	1.428	0.057774
A_44_P1059422	Actr2	NM_001009268	289820	0.15	1.428	0.06128
A_44_P340241	XM_237260	XM_237260		0.15	1.428	0.022379
A_44_P631280	BM387035	BM387035		0.15	1.428	0.029989
A_44_P489062	Ahcyl1_predicted	XM_001068488		0.15	1.428	0.028777
A_43_P22269	LOC362845	XM_343177	362845	0.15	1.428	0.014093
A_43_P20587	Gypc	NM_001013233	364837	0.15	1.428	0.016609
A_44_P224568	Zfp162	NM_058210	117855	0.15	1.427	0.013085
A_44_P438849	Mrpl22_predicted	XM_213307		0.15	1.427	0.031409
A_43_P20775	Brf2	NM_001024773	306542	0.15	1.427	0.016525
A_44_P448083	Mkrn2	NM_001008314	297525	0.15	1.427	0.006088
A_43_P16445	RGD1566014_predicted	XM_577058		0.15	1.427	0.011786
A_44_P929214	LOC493574	NM_001008386	493574	0.15	1.427	0.118725
A_44_P144316	Olr1470	NM_001000723	404975	0.15	1.427	0.226485
A_44_P536665	RGD1304748	NM_001025634	290500	0.15	1.427	0.083896
A_44_P390454	Gdi2	NM_017276	29662	0.15	1.427	0.019408
A_44_P855945	A_44_P855945	A_44_P855945		0.15	1.427	0.025664
A_44_P257982	A_44_P257982	A_44_P257982		0.15	1.427	0.121685
A_44_P336098	Ap3m2	NM_133305	140667	0.15	1.427	0.227269
A_42_P525454	Tenc1_predicted	XM_235710		0.15	1.427	0.086648
A_44_P443629	BI275292	BI275292	89805	0.15	1.427	0.035714
A_44_P849269	AW918162	AW918162	64351	0.15	1.427	0.010124
A_44_P236512	LOC295643	XR_008064	295643	0.15	1.427	0.047141
A_44_P305272	Scara3_predicted	XM_344421		0.15	1.427	0.43391
A_44_P536613	Ch25h	NM_001025415	309527	0.15	1.427	0.137748
A_44_P225644	AI072161	AI072161		0.15	1.427	0.033418
A_44_P189650	XM_341080	XM_341080		0.15	1.427	0.03299
A_44_P130811	Arsk	XM_345131		0.15	1.426	0.06747
A_43_P18579	Fbxw2_predicted	XM_231162		0.15	1.426	0.034655
A_44_P277820	AF267750	AF267750	25599	0.15	1.426	0.166044
A_42_P815727	Usp48	NM_198785	362636	0.15	1.426	0.014954
A_44_P492279	XM_342482	XM_342482		0.15	1.426	0.110653
A_42_P765031	ORF19	XM_346083	367328	0.15	1.426	0.020686
A_44_P426591	RGD1561178_predicted	XM_001075088		0.15	1.426	0.05978
A_44_P883146	Thtpa	NM_001007682	305889	0.15	1.426	0.018796
A_44_P553170	LOC681315	XM_001063869		0.15	1.426	0.041797
A_44_P409860	RGD1565073_predicted	XM_578415		0.15	1.426	0.058311
A_43_P20961	Stk36_predicted	XM_217435	301516	0.15	1.426	0.02495
A_44_P526390	Paip1_predicted	XM_001068994		0.15	1.426	0.116744
A_43_P10399	Kctd9_predicted	XM_344426		0.15	1.426	0.049509
A_44_P442858	Egfl4	XM_341803	114029	0.15	1.426	0.049334
A_44_P475829	Hoxd1_predicted	XM_221520		0.15	1.425	0.095078
A_44_P233618	Blvra	NM_053850	116599	0.15	1.425	0.005477
A_43_P18971	Exosc3_predicted	XM_233001		0.15	1.425	0.006067
A_43_P20547	Prcp_predicted	XM_214993		0.15	1.425	0.029535

A_43_P21464	LOC313391	XM_233220		0.15	1.425	0.006946
A_44_P732751	TC517702	TC517702		0.15	1.425	0.035988
A_44_P454277	Ikbkg	NM_199103	309295	0.15	1.425	0.027452
A_44_P121839	XM_345657	XM_345657		0.15	1.425	0.022472
A_44_P318828	RGD1307475	NM_001014233	364298	0.15	1.425	0.085033
A_44_P269964	RGD1311562_predicted	XM_001080518		0.15	1.425	0.073684
A_44_P440878	Agpat2_predicted	XM_231089		0.15	1.424	0.060053
A_44_P265163	RGD1562046_predicted	XM_578248	502749	0.15	1.424	0.016259
A_44_P269808	Slc9a8	NM_001025281	311651	0.15	1.424	0.045988
A_44_P316975	Usp8_predicted	XM_215821		0.15	1.424	0.041053
A_44_P650755	BG673332	BG673332	29670	0.15	1.424	0.048342
A_44_P539680	Garnl1	XM_001079197		0.15	1.424	0.121147
A_44_P606068	ENSRNOT00000043460	ENSRNOT00000043460		0.15	1.424	0.190962
A_43_P15524	Ccnd3	NM_012766	25193	0.15	1.424	0.006413
A_44_P172029	Phgdhl1	NM_001034937	361094	0.15	1.424	0.011071
A_44_P652919	RGD1304653_predicted	XM_343551		0.15	1.424	0.014226
A_44_P323550	MGC109149	NM_001009705	361520	0.15	1.423	0.081257
A_44_P285001	AI030751	AI030751	500054	0.15	1.423	0.059863
A_44_P215817	Arih2_predicted	NM_001012275	316005	0.15	1.423	0.038941
A_43_P13870	CB547748	CB547748		0.15	1.423	0.311108
A_44_P344906	Dnajb5_predicted	XM_233767		0.15	1.423	0.071638
A_44_P330332	Prkaca	XM_341661		0.15	1.423	0.013638
A_44_P760403	ENSRNOT00000014194	ENSRNOT00000014194		0.15	1.423	0.06194
A_44_P575227	Xrn2_predicted	XM_342535		0.15	1.423	0.050894
A_44_P168941	Gtlf3b_predicted	XM_343907	363614	0.15	1.423	0.047524
A_44_P1058980	Crlz1	NM_001012036	305258	0.15	1.423	0.01861
A_44_P438529	ENSRNOT00000025591	ENSRNOT00000025591		0.15	1.423	0.018921
A_42_P717621	RGD1304607_predicted	XM_342020	361727	0.15	1.423	0.021172
A_43_P17471	Chac1_predicted	XM_342497	362196	0.15	1.423	0.019632
A_42_P592082	Gtf2h4	NM_212501	294236	0.15	1.422	0.007733
A_44_P411677	AW143389	AW143389		0.15	1.422	0.0227
A_44_P206115	RGD1559963_predicted	XM_227018		0.15	1.422	0.025509
A_44_P229923	Snx6_predicted	XM_343062		0.15	1.422	0.063722
A_42_P736967	Zdhhc18	NM_001039339	362613	0.15	1.422	0.014427
A_44_P115048	Terf2ip	NM_001013143	307861	0.15	1.422	0.053393
A_44_P427067	LOC691075	XM_001076752		0.15	1.421	0.011852
A_43_P18935	Phtf1	XM_342306	252962	0.15	1.421	0.047206
A_44_P104985	Serpinb2	NM_021696	60325	0.15	1.421	0.270561
A_44_P475564	Glycam1	NM_012794	25258	0.15	1.421	0.179681
A_44_P331281	LOC691318	XM_001077680	691318	0.15	1.421	0.027146
A_44_P760545	LOC499896	XM_575242		0.15	1.421	0.039923
A_44_P501051	ENSRNOT00000027501	ENSRNOT00000027501		0.15	1.421	0.03261
A_44_P158107	Argbp2	NM_053770	114901	0.15	1.421	0.054217
A_44_P558048	RGD1309387	NM_001033897	313777	0.15	1.421	0.014254
A_44_P474755	Ppgb	NM_001011959	296370	0.15	1.420	0.025051
A_44_P312605	BG153336	BG153336	361825	0.15	1.420	0.072882
A_42_P682589	Nr4a1	NM_024388	79240	0.15	1.420	0.102214
A_44_P514379	Acaca	X53003	60581	0.15	1.420	0.019223
A_44_P1030501	RGD1310931_predicted	XM_214300		0.15	1.420	0.0073
A_44_P417175	RGD1305834_predicted	XM_227717	310880	0.15	1.420	0.019387
A_44_P1046263	RGD1561243_predicted	XM_343383	363055	0.15	1.420	0.014363
A_44_P762376	DV727084	DV727084		0.15	1.420	0.015539
A_43_P20604	Ctdp1_predicted	XM_225717		0.15	1.420	0.051826
A_44_P290394	Slc27a1	NM_053580	94172	0.15	1.420	0.018357
A_43_P14436	TC541905	TC541905		0.15	1.420	0.108057
A_44_P496972	XM_226326	XM_226326		0.15	1.420	0.096935
A_44_P242614	Znrf1_predicted	XM_342692		0.15	1.420	0.023481
A_44_P567275	A_44_P567275	A_44_P567275		0.15	1.420	0.058962
A_44_P699410	TC519876	TC519876		0.15	1.420	0.01509
A_44_P822364	TC553976	TC553976		0.15	1.420	0.019065
A_44_P256345	Smarcad1_predicted	XM_231860		0.15	1.420	0.023663
A_44_P470043	Brd1_predicted	XM_235552		0.15	1.420	0.333328
A_42_P652202	Rabggta	NM_031654	58983	0.15	1.419	0.017089

A_44_P557894	LOC291930	XR_008926	291930	0.15	1.419	0.017112
A_44_P685613	RGD1310645_predicted	XM_001061052		0.15	1.419	0.0128
A_44_P266340	AW918765	AW918765		0.15	1.419	0.01089
A_44_P1026706	RGD1560433_predicted	XM_001061644		0.15	1.419	0.01139
A_44_P372529	Crem	NM_017334	25620	0.15	1.419	0.033364
A_44_P278116	LOC686310	XM_001069404		0.15	1.419	0.017055
A_44_P308181	Gapvd1_predicted	XM_231161	311880	0.15	1.419	0.035042
A_44_P466183	Rap2b	NM_133410	170923	0.15	1.419	0.035056
A_44_P1041791	Abcf1	XM_001056151		0.15	1.419	0.05794
A_44_P190702	AI230360	AI230360		0.15	1.419	0.017254
A_44_P463034	CA508371	CA508371	361207	0.15	1.418	0.030573
A_43_P18507	RGD1308031	NM_001009667	300442	0.15	1.418	0.047496
A_43_P19014	Zfp482_predicted	XM_345345		0.15	1.418	0.05952
A_44_P405704	XM_234486	XM_234486		0.15	1.418	0.00987
A_44_P525203	RGD1306787_predicted	XM_224289	305968	0.15	1.418	0.082727
A_44_P340490	RGD1310429_predicted	XM_221232	303764	0.15	1.418	0.018681
A_44_P323591	Sh2bp1	XM_238127	293184	0.15	1.418	0.040751
A_44_P436442	Cpsf2_predicted	XM_216766		0.15	1.418	0.009648
A_43_P17260	Rsrc1	NM_001014172	361956	0.15	1.418	0.007045
A_44_P331567	Stim2_predicted	XM_223454		0.15	1.417	0.189126
A_43_P20541	Eed_predicted	XM_214996		0.15	1.417	0.028664
A_44_P267265	A_44_P267265	A_44_P267265		0.15	1.417	0.010747
A_42_P711589	Sfxn3	NM_022948	65042	0.15	1.417	0.018455
A_44_P852451	ENSRNOT00000021284	ENSRNOT00000021284		0.15	1.417	0.16627
A_44_P180280	RGD1565642_predicted	XM_341932	361652	0.15	1.417	0.027167
A_43_P18126	Slc30a7	XM_227612	310801	0.15	1.417	0.014816
A_43_P22881	BF400622	BF400622		0.15	1.417	0.096308
A_44_P472011	Pigw	NM_194461	378774	0.15	1.417	0.101712
A_43_P16455	RGD1310211_predicted	XM_224181		0.15	1.417	0.007515
A_43_P22883	Cdadc1	XM_573797		0.15	1.417	0.199854
A_44_P190115	Snx19_predicted	XM_235940		0.15	1.417	0.030713
A_44_P182913	XM_226340	XM_226340		0.15	1.416	0.007544
A_43_P10283	Epm2aip1_predicted	XM_001076356		0.15	1.416	0.092703
A_44_P1010867	MGC124888	NM_001033891	297387	0.15	1.416	0.014633
A_44_P535913	RGD1309077_predicted	XM_001080751		0.15	1.416	0.005332
A_44_P203613	Hnrpul1_predicted	XM_341807		0.15	1.416	0.025251
A_44_P252507	Zfp111	NM_133323	170849	0.15	1.416	0.069105
A_44_P125659	Grina	NM_153308	266668	0.15	1.416	0.118624
A_44_P218247	Atpaf2_predicted	XM_220522		0.15	1.416	0.033275
A_44_P402435	AJ005161	AJ005161		0.15	1.416	0.014677
A_44_P910739	DY472814	DY472814		0.15	1.416	0.300652
A_44_P824580	TC562224	TC562224		0.15	1.416	0.313567
A_44_P403501	LOC684436	XM_001065639		0.15	1.416	0.098977
A_44_P255837	A_44_P255837	A_44_P255837		0.15	1.416	0.005428
A_44_P396198	Cugbp1	NM_001025421	362160	0.15	1.415	0.019131
A_44_P459533	Cbfb	NM_001013191	361391	0.15	1.415	0.005831
A_43_P17540	AI548708	AI548708		0.15	1.415	0.030859
A_44_P1040854	Sec11l3	NM_153628	266758	0.15	1.415	0.036109
A_44_P194872	Top3a_predicted	XM_001077625		0.15	1.415	0.014839
A_44_P683068	Pde4d	NM_017032	24627	0.15	1.415	0.051685
A_42_P519910	Eif3s5_predicted	XM_215037		0.15	1.415	0.010045
A_44_P808732	LOC687346	XM_001075972		0.15	1.415	0.006614
A_44_P870562	TC562406	TC562406		0.15	1.415	0.155692
A_43_P15022	AW916092	AW916092	294048	0.15	1.415	0.042661
A_44_P588779	Arhgap5	XM_001075475		0.15	1.415	0.174675
A_43_P12531	Tceb2	NM_031129	81807	0.15	1.415	0.006478
A_44_P398300	MGC94288	NM_001005538	293343	0.15	1.415	0.072217
A_44_P528552	Tyki_predicted	XM_234017		0.15	1.415	0.143513
A_44_P776282	Ppp1r2	NM_138823	192361	0.15	1.414	0.063502
A_44_P923195	A_44_P923195	A_44_P923195		0.15	1.414	0.014753
A_44_P1060382	Hyal2	NM_172040	64468	0.15	1.414	0.032833
A_44_P157009	Rnf20_predicted	XM_232995		0.15	1.414	0.044788
A_44_P501818	RGD1310980_predicted	XM_343381		0.15	1.414	0.192019

A_44_P122723	BG667918	BG667918		0.15	1.414	0.137014
A_44_P370761	Nfrkb_predicted	XM_236009		0.15	1.414	0.014632
A_44_P512090	AW918093	AW918093		0.15	1.413	0.035695
A_42_P554044	AW144325	AW144325		0.15	1.413	0.076227
A_44_P256973	RGD1310316	NM_001014029	309420	0.15	1.413	0.081789
A_44_P501577	A_44_P501577	A_44_P501577		0.15	1.413	0.246267
A_44_P344636	RGD1310016_predicted	XM_219778	309306	0.15	1.413	0.019747
A_42_P841714	RGD1309707_predicted	XM_001071299		0.15	1.413	0.028212
A_43_P19812	RGD1305420	NM_001025652	298072	0.15	1.413	0.026432
A_43_P13581	CB547833	CB547833		0.15	1.413	0.111409
A_44_P283544	Rps12	NM_031709	65139	0.15	1.413	0.012251
A_44_P164007	RGD1561653_predicted	XM_343060	362736	0.15	1.413	0.03093
A_44_P462029	Itgb8_predicted	XM_343126		0.15	1.413	0.056831
A_44_P455059	LOC366473	NM_001014261	366473	0.15	1.413	0.045826
A_44_P683487	RGD1562161_predicted	XM_576964	501559	0.15	1.412	0.164071
A_44_P265013	RGD1305653_predicted	XM_215648	295293	0.15	1.412	0.113642
A_43_P22304	Casp8ap2_predicted	XM_232860		0.15	1.412	0.063832
A_44_P557493	Pggt1b	NM_031082	81746	0.15	1.412	0.168316
A_44_P400158	Stk6	NM_153296	261730	0.15	1.412	0.089663
A_44_P486568	Arpc1a	NM_031146	81824	0.15	1.412	0.062503
A_44_P118468	AA799762	AA799762	296470	0.15	1.412	0.115083
A_44_P163611	RGD1561783_predicted	XM_577816	502348	0.15	1.412	0.067418
A_44_P971288	Otud4	XM_226388	307774	0.15	1.411	0.048861
A_44_P713567	LOC687994	XM_001080810		0.15	1.411	0.026572
A_44_P708147	RGD1561440_predicted	XM_001061954		0.15	1.411	0.004419
A_44_P151914	XM_212875	XM_212875		0.15	1.411	0.01187
A_44_P1007105	Ppap2b	NM_138905	192270	0.15	1.411	0.118677
A_44_P503874	RGD1561239_predicted	XM_221883		0.15	1.411	0.50697
A_44_P206002	RGD1310712_predicted	XM_001064849		0.15	1.411	0.046839
A_44_P248516	RGD1310440_predicted	XM_342912	362594	0.15	1.411	0.006162
A_43_P21146	Atp13a1_predicted	XM_214310		0.15	1.411	0.008355
A_44_P431488	RGD1562962_predicted	XM_235716		0.15	1.411	0.008472
A_44_P809228	TC528216	TC528216		0.15	1.411	0.16106
A_44_P123078	AW913992	AW913992	54265	0.15	1.411	0.020802
A_44_P683816	Sh3px3_predicted	XM_001073948		0.15	1.411	0.040882
A_44_P819834	Fkrp	NM_001025678	308390	0.15	1.411	0.040625
A_44_P987960	CO398009	CO398009		0.15	1.411	0.04527
A_44_P300038	LOC681300	XM_001061130		0.15	1.411	0.007427
A_44_P393966	RGD1308432_predicted	XM_342642	362324	0.15	1.410	0.186259
A_44_P119356	XM_341673	XM_341673		0.15	1.410	0.013607
A_44_P839778	TC545838	TC545838		0.15	1.410	0.043154
A_44_P478430	Centb5_predicted	XM_233719		0.15	1.410	0.014959
A_44_P356215	Kb18	NM_001008828	300247	0.15	1.410	0.017373
A_44_P592899	RGD1562251_predicted	XM_001074636		0.15	1.410	0.126498
A_44_P396266	RGD1304751	XM_231428	312083	0.15	1.410	0.135661
A_44_P246452	CB545779	CB545779		0.15	1.409	0.071875
A_44_P607859	TC558285	TC558285		0.15	1.409	0.274275
A_43_P11241	Chd4	XM_232354	117535	0.15	1.409	0.042207
A_44_P1047710	A_44_P1047710	A_44_P1047710		0.15	1.409	0.071432
A_44_P203064	LOC682390	XM_001061306		0.15	1.409	0.033729
A_44_P105441	XM_233295	XM_233295		0.15	1.409	0.060022
A_44_P882450	D86711	D86711	311165	0.15	1.409	0.067755
A_44_P126597	Paqr4	NM_001017377	302967	0.15	1.409	0.010989
A_44_P728403	Neo1	XM_001074913		0.15	1.409	0.028743
A_44_P760039	RGD1564621_predicted	XM_573105	497918	0.15	1.409	0.01221
A_44_P477903	Apbb3	NM_053957	117026	0.15	1.409	0.035695
A_44_P1048999	Rhpn1_predicted	XM_216954	300030	0.15	1.409	0.039162
A_44_P562001	TC525017	TC525017		0.15	1.409	0.321318
A_44_P431400	RGD1307344_predicted	XR_007289	288900	0.15	1.409	0.226257
A_43_P14802	Ncoa6	XM_001064189		0.15	1.408	0.017228
A_42_P698409	Enpp3	NM_019370	54410	0.15	1.408	0.102332
A_44_P395572	Nr1d2	NM_147210	259241	0.15	1.408	0.125166
A_44_P839920	RGD1566108_predicted	XM_001059711		0.15	1.408	0.074203

A_44_P395044	Synj1	XM_573256	85238	0.15	1.408	0.034843
A_44_P368170	Actr5_predicted	XM_342563		0.15	1.408	0.039983
A_44_P183241	Bcl6_predicted	XM_221333		0.15	1.408	0.078625
A_44_P404071	LOC690354	XM_001074195	690354	0.15	1.408	0.03483
A_44_P165692	RGD1308874	NM_001034003	366227	0.15	1.408	0.05181
A_44_P1054403	Ndufa2_predicted	XM_214570		0.15	1.407	0.018155
A_44_P219092	CF109537	CF109537		0.15	1.407	0.144091
A_44_P470635	Ptbp1	BC061858	29497	0.15	1.407	0.02653
A_44_P1046976	Rent1_predicted	XM_224732	684527	0.15	1.407	0.006888
A_44_P186914	Ggtla1	NM_019235	29566	0.15	1.407	0.150481
A_44_P238693	Nav2	XM_341864	171563	0.15	1.407	0.102563
A_44_P151751	Efha1	XM_341328		0.15	1.407	0.037035
A_44_P419827	Mtmr2_predicted	XM_235822		0.15	1.407	0.008396
A_44_P197807	RGD1304748	NM_001025634	290500	0.15	1.407	0.022036
A_44_P352043	Bcl11b_predicted	XM_234514		0.15	1.407	0.012291
A_44_P948037	Slc39a13	NM_001039196	295928	0.15	1.406	0.053828
A_43_P22646	Utx_predicted	XM_001055391		0.15	1.406	0.022507
A_44_P544400	RGD1307392_predicted	XM_216750	299209	0.15	1.406	0.086509
A_43_P13111	Scap2	NM_130413	155183	0.15	1.406	0.023381
A_44_P1000383	XM_213969	XM_213969		0.15	1.406	0.041692
A_44_P212567	Dlgap4	NM_173145	286930	0.15	1.406	0.058807
A_44_P475130	LOC314964	XM_001067475		0.15	1.406	0.079203
A_44_P681754	BF549535	BF549535		0.15	1.406	0.135661
A_44_P552751	M95791	M95791		0.15	1.406	0.030943
A_44_P140615	LOC681599	XM_001057569		0.15	1.406	0.029659
A_44_P105497	XM_346218	XM_346218		0.15	1.405	0.01868
A_44_P249925	MGC114417	XM_001055014		0.15	1.405	0.028951
A_43_P21414	LOC683259	XM_001065140		0.15	1.405	0.077069
A_43_P22846	XM_219684	XM_219684		0.15	1.405	0.02643
A_43_P15316	Tfam	NM_031326	83474	0.15	1.405	0.064349
A_43_P15181	RGD1563824_predicted	XM_574313	499020	0.15	1.405	0.045629
A_44_P441147	XM_222307	XM_222307		0.15	1.405	0.011262
A_43_P17186	RGD1304774	NM_001025128	360969	0.15	1.405	0.02602
A_44_P349250	Paf1	NM_001024898	361531	0.15	1.405	0.014875
A_42_P565437	Sf3a2	NM_001011986	299620	0.15	1.404	0.005578
A_44_P401515	Exoc6	NM_019277	50556	0.15	1.404	0.094803
A_44_P107132	Mapk8ip	NM_053777	116457	0.15	1.404	0.324975
A_44_P561254	RGD1309922_predicted	XM_224329	306007	0.15	1.404	0.007378
A_44_P293750	Rnf14	XM_001066493		0.15	1.404	0.110299
A_44_P116346	Soat2	NM_153728	266770	0.15	1.404	0.133077
A_44_P463891	RGD1564816_predicted	XM_573341		0.15	1.404	0.007528
A_43_P18241	XM_224859	XM_224859		0.15	1.404	0.032191
A_44_P515965	A_44_P515965	A_44_P515965		0.15	1.404	0.021691
A_42_P513939	RGD1565685_predicted	XM_228714	317344	0.15	1.404	0.04696
A_42_P618414	Mto1_predicted	XM_217208		0.15	1.404	0.025891
A_44_P623844	TC526485	TC526485		0.15	1.404	0.013721
A_42_P493785	Cdk2ap1_predicted	XM_341076	360804	0.15	1.404	0.026425
A_44_P956310	AI171999	AI171999	85428	0.15	1.404	0.351376
A_44_P866732	AA848648	AA848648	64824	0.15	1.404	0.021911
A_42_P762248	RGD1307934_predicted	XM_001055620		0.15	1.404	0.070341
A_44_P218284	RGD1560049_predicted	XM_573199	498003	0.15	1.404	0.070839
A_44_P811924	DV718192	DV718192		0.15	1.404	0.008463
A_43_P18366	Tor1b	NM_001039197	311854	0.15	1.403	0.029562
A_44_P299505	LOC192182	AF147718		0.15	1.403	0.027194
A_44_P337125	Slco4a1	NM_133608	171144	0.15	1.403	0.075197
A_43_P19526	CB546113	CB546113	652928	0.15	1.403	0.007577
A_44_P188983	LOC686914	XM_001076312		0.15	1.403	0.044362
A_44_P1012711	Solt_predicted	XM_215471		0.15	1.403	0.063226
A_44_P373413	Ppp1r7	NM_001009825	301618	0.15	1.403	0.053798
A_44_P607014	TC534441	TC534441		0.15	1.403	0.251735
A_44_P307106	RGD1310951_predicted	XM_001056694		0.15	1.403	0.044819
A_44_P536545	LOC679255	XM_001055461		0.15	1.403	0.008423
A_44_P307971	L04739	L04739		0.15	1.403	0.18416

A_43_P16723	LOC301119	XM_238569		0.15	1.403	0.011978
A_44_P325241	AI137378	AI137378		0.15	1.403	0.038871
A_43_P21228	Actl6b_predicted	XM_213739		0.15	1.402	0.073053
A_44_P541561	TC524937	TC524937		0.15	1.402	0.108215
A_43_P15001	Vezf1_predicted	XM_213421	287615	0.15	1.402	0.03399
A_44_P972929	Zfp91	XM_001079228		0.15	1.402	0.033683
A_44_P683219	LOC502786	XR_006715	502786	0.15	1.402	0.015134
A_43_P23039	Socs7_predicted	XM_213443	287659	0.15	1.402	0.015764
A_44_P271720	Cd151	NM_022523	64315	0.15	1.402	0.02397
A_44_P211111	RGD1565416_predicted	XM_236367	315776	0.15	1.402	0.079623
A_44_P105434	Sec63_predicted	XM_228305		0.15	1.402	0.016865
A_44_P243528	XM_341482	XM_341482		0.15	1.401	0.011346
A_44_P433068	Fam3c	NM_198771	312159	0.15	1.401	0.070228
A_44_P328302	Glmn	XM_213992		0.15	1.401	0.191667
A_44_P340401	RGD1565056_predicted	XM_235376		0.15	1.401	0.007797
A_44_P262977	RGD1308729_predicted	XM_234835	314584	0.15	1.401	0.086459
A_42_P759043	RGD1311783_predicted	XM_215266	294012	0.15	1.401	0.009676
A_43_P10854	Hbs1l	NM_001011934	293408	0.15	1.401	0.041847
A_44_P412672	LOC690777	XM_001072800	690777	0.15	1.401	0.037207
A_44_P960266	RGD1307493_predicted	XM_001080742		0.15	1.401	0.037813
A_42_P693011	Chrnb1	NM_012528	24261	0.15	1.401	0.041338
A_43_P13245	Pabpc1	NM_134353	171350	0.15	1.401	0.021814
A_44_P208275	Ugcg	NM_031795	83626	0.15	1.401	0.061765
A_44_P852185	A_44_P852185	A_44_P852185		0.15	1.401	0.160292
A_44_P267199	RGD1311713_predicted	XM_218461	308478	0.15	1.401	0.041834
A_44_P342538	RGD1565474_predicted	XM_342675	362353	0.15	1.401	0.042492
A_44_P932802	Map3k7_predicted	XM_232855		0.15	1.401	0.008319
A_42_P585829	Serpini2	XM_342263	171149	0.15	1.400	0.025495
A_43_P22513	Cyb561d1_predicted	XM_342315		0.15	1.400	0.040173
A_44_P231243	Insig2	NM_178091	288985	0.15	1.400	0.208095
A_44_P175391	Tmed10	NM_053467	84599	0.15	1.400	0.031005
A_44_P499117	Ebpl_predicted	XM_341334	361054	0.15	1.400	0.135169
A_44_P186249	LOC684513	XM_001070765		0.15	1.400	0.007046
A_44_P147806	CB545854	CB545854	313707	0.15	1.400	0.013076
A_44_P349844	Tlk2_predicted	XM_221023	303592	0.15	1.400	0.151549
A_42_P603488	RGD1305831_predicted	XM_213700		0.15	1.400	0.015076
A_44_P671194	TC568188	TC568188		0.15	1.400	0.099227
A_42_P810408	Tarsl1	NM_001014040	310672	0.15	1.400	0.008685
A_44_P438053	Lrrc46	NM_001004201	287653	0.15	1.399	0.04225
A_43_P17439	Gtf3c2	NM_001025120	313914	0.15	1.399	0.035071
A_44_P316415	RGD1305500_predicted	XM_217663		0.15	1.399	0.024984
A_44_P325935	XM_218589	XM_218589		0.15	1.399	0.045577
A_44_P161192	Smad5	NM_021692	59328	0.15	1.399	0.025438
A_43_P12266	Bard1	NM_022622	64557	0.15	1.399	0.141391
A_44_P714246	LOC682205	XM_001060401		0.15	1.399	0.011919
A_44_P188596	Exoc5	NM_022204	60627	0.15	1.399	0.071768
A_44_P360795	Picalm	NM_053554	89816	0.15	1.399	0.023053
A_42_P759822	XM_237254	XM_237254		0.15	1.398	0.033235
A_44_P539349	Tm4sf5_predicted	XM_220569		0.15	1.398	0.057053
A_44_P157604	RGD1565556_predicted	XM_001076955		0.15	1.398	0.243666
A_44_P495260	CB547615	CB547615		0.15	1.398	0.200834
A_44_P421417	Spon2	NM_138533	171569	0.15	1.398	0.049512
A_43_P17172	Prpf8	XM_213385	287530	0.15	1.398	0.018876
A_44_P130559	Psmb1	NM_053590	94198	0.15	1.398	0.046492
A_44_P263072	Eef1b2_predicted	XM_343580		0.15	1.398	0.011656
A_44_P637897	LOC681972	XM_001059145		0.15	1.398	0.004934
A_44_P328292	XM_341158	XM_341158		0.15	1.398	0.056844
A_44_P466100	Pcdhgc3	NM_053943	116782	0.15	1.398	0.040991
A_44_P1010553	MGC116266	NM_001024867	290775	0.15	1.397	0.091005
A_44_P483174	Brd4	XM_343175	362844	0.15	1.397	0.036143
A_44_P902199	TC518110	TC518110		0.15	1.397	0.030629
A_44_P163645	Wdr1	NM_001014135	360950	0.15	1.397	0.035025
A_43_P14214	Psma6	NM_017283	29673	0.15	1.397	0.021755

A_44_P445090	Gaa	NM_199118	367562	0.15	1.397	0.045345
A_44_P201084	LOC500109	XM_001053923		0.15	1.397	0.085638
A_44_P455693	Syt11	NM_031667	60568	0.15	1.397	0.072604
A_43_P17230	RGD1304762	NM_001034918	291705	0.15	1.396	0.053926
A_44_P602122	LOC287992	AY383659	287992	0.15	1.396	0.084812
A_42_P710738	Phgdh	NM_031620	58835	0.14	1.396	0.166793
A_44_P260487	M6pr	NM_001007700	312689	0.14	1.396	0.052157
A_42_P571960	Phf5a	NM_138888	192246	0.14	1.396	0.022668
A_44_P583020	AW921320	AW921320		0.14	1.396	0.112037
A_43_P11448	Cp	NM_012532	24268	0.14	1.396	0.056591
A_44_P854689	TC544249	TC544249		0.14	1.396	0.0634
A_42_P632107	RGD1307047	XM_227583	310781	0.14	1.395	0.01971
A_44_P265299	Alkbh_predicted	XM_343092		0.14	1.395	0.035646
A_42_P672401	Dhx30	NM_001013249	367172	0.14	1.395	0.012188
A_44_P852513	A_44_P852513	A_44_P852513		0.14	1.395	0.031719
A_44_P447839	Zfp503_predicted	XM_223786		0.14	1.395	0.037551
A_42_P491366	Tmem19	NM_199098	299800	0.14	1.395	0.018551
A_44_P385350	BQ191577	BQ191577		0.14	1.395	0.037466
A_44_P339798	LOC680522	XM_001057550		0.14	1.395	0.090018
A_44_P492651	Tbcd_predicted	XM_340955	360683	0.14	1.395	0.01252
A_44_P121511	RGD1563478_predicted	XM_344312		0.14	1.395	0.036695
A_44_P130547	LOC691194	XM_001077174		0.14	1.395	0.116307
A_44_P858481	A_44_P858481	A_44_P858481		0.14	1.395	0.008694
A_44_P956990	AW144454	AW144454		0.14	1.395	0.47086
A_44_P208849	Dnalc4	NM_001009666	300078	0.14	1.395	0.010937
A_44_P991158	RGD1565385_predicted	XM_342453	362153	0.14	1.395	0.036737
A_44_P154414	XM_225604	XM_225604		0.14	1.394	0.037064
A_44_P323933	Smc2l1_predicted	XM_342837		0.14	1.394	0.042127
A_44_P621328	A_44_P621328	A_44_P621328		0.14	1.394	0.016358
A_44_P802495	RGD1562438_predicted	XM_001077874		0.14	1.394	0.060388
A_44_P258336	Tmem39a	NM_001013865	288092	0.14	1.394	0.0092
A_42_P525962	RGD1561792_predicted	XM_216648	298861	0.14	1.394	0.02051
A_44_P822920	DV717319	DV717319		0.14	1.394	0.063724
A_44_P778608	TC527987	TC527987		0.14	1.394	0.036902
A_43_P17565	XM_221138	XM_221138		0.14	1.394	0.015368
A_44_P366005	RGD1560954_predicted	XM_228468		0.14	1.394	0.06549
A_44_P558540	Tnfaip6	XM_001065494		0.14	1.393	0.035166
A_44_P414838	RGD1561841_predicted	XM_227107		0.14	1.393	0.009614
A_44_P450214	RGD1562244_predicted	XM_230024	295678	0.14	1.393	0.045373
A_44_P902385	TC541522	TC541522		0.14	1.393	0.104806
A_43_P17493	RGD1305457	NM_001009430	314730	0.14	1.393	0.212189
A_44_P658529	RGD1559931_predicted	XM_224620	306264	0.14	1.393	0.051393
A_44_P609775	TC548033	TC548033		0.14	1.393	0.011681
A_44_P238608	Casp9	NM_031632	58918	0.14	1.393	0.043594
A_43_P18108	LOC686495	XM_001074408		0.14	1.393	0.251968
A_44_P246343	Taf15_predicted	XM_237792		0.14	1.392	0.018349
A_44_P619150	AW144111	AW144111	24917	0.14	1.392	0.025287
A_44_P378118	BF284341	BF284341		0.14	1.392	0.008918
A_44_P104396	AW143532	AW143532		0.14	1.392	0.235353
A_44_P363208	XM_575518	XM_575518		0.14	1.392	0.443315
A_44_P382020	Wbscr21	XM_341104		0.14	1.392	0.019607
A_43_P15806	Ptprg	NM_134356	171357	0.14	1.392	0.043144
A_44_P1024788	LOC498606	NM_001025143	498606	0.14	1.392	0.029664
A_44_P237159	CB544419	CB544419		0.14	1.392	0.032024
A_44_P119504	Lrrc47_predicted	XM_001076460		0.14	1.392	0.021057
A_43_P18294	CA511295	CA511295	312678	0.14	1.391	0.080655
A_44_P424347	RGD1559496_predicted	XM_226564		0.14	1.391	0.015044
A_44_P299641	Olr1572_predicted	XM_221921		0.14	1.391	0.191311
A_42_P601961	Susd3_predicted	XM_225203		0.14	1.391	0.013876
A_44_P527495	XM_227480	XM_227480		0.14	1.391	0.028155
A_44_P524922	NP072211	NP072211		0.14	1.391	0.033412
A_44_P201358	XM_219534	XM_219534		0.14	1.391	0.007581
A_44_P142306	A_44_P142306	A_44_P142306		0.14	1.391	0.0359

A_44_P794762	LOC682812	XM_001059812		0.14	1.391	0.022918
A_44_P363913	ENSRNOT00000048551	ENSRNOT00000048551		0.14	1.391	0.021987
A_42_P832028	Sec13l1	NM_001006978	297522	0.14	1.391	0.047811
A_44_P527611	Tradd	XM_341671	246756	0.14	1.391	0.040932
A_44_P307633	Cep1_predicted	XM_231168		0.14	1.391	0.026252
A_44_P928879	Fbn2	NM_031826	689008	0.14	1.391	0.177442
A_44_P232265	RGD1559865_predicted	XM_346093		0.14	1.390	0.014373
A_44_P223358	BF555594	BF555594		0.14	1.390	0.087544
A_44_P459745	A_44_P459745	A_44_P459745		0.14	1.390	0.065089
A_42_P540908	Asna1	XM_001066031		0.14	1.390	0.032179
A_44_P1051799	XM_215147	XM_215147		0.14	1.390	0.020876
A_44_P210235	Hnrpr	NM_175603	319110	0.14	1.390	0.017247
A_44_P473928	Spry1_predicted	XM_227050		0.14	1.390	0.051531
A_44_P517180	Gns	XM_576231		0.14	1.390	0.030518
A_44_P1013872	Mon1b_predicted	XM_001076362		0.14	1.390	0.020665
A_44_P188857	Pcm1	XM_344524	81740	0.14	1.390	0.031497
A_44_P575559	A_44_P575559	A_44_P575559		0.14	1.390	0.084114
A_44_P384772	LOC313535	XM_233435	313535	0.14	1.390	0.075375
A_44_P1017079	Rnaseh1	NM_001013097	298933	0.14	1.390	0.022729
A_44_P426717	RGD1309839_predicted	XM_225627		0.14	1.390	0.12869
A_44_P413024	Nrbp	NM_001034997	619579	0.14	1.389	0.095308
A_42_P836863	Oxsr1_predicted	XM_236687		0.14	1.389	0.053343
A_44_P651053	Supt16h_predicted	XM_223981		0.14	1.389	0.034908
A_44_P496454	Tceb2	NM_031129	81807	0.14	1.389	0.014783
A_44_P639552	TC528134	TC528134		0.14	1.389	0.06927
A_44_P262863	Itgb1bp2_predicted	XM_228553		0.14	1.389	0.124783
A_44_P742138	AW918487	AW918487	309196	0.14	1.389	0.032093
A_44_P147138	Vangl2_predicted	XM_222896		0.14	1.389	0.047576
A_43_P11192	RGD1310571	NM_001014147	361301	0.14	1.389	0.023379
A_44_P182642	Rbbp5_predicted	XM_222669		0.14	1.389	0.014245
A_44_P117850	AABR03055518	AABR03055518		0.14	1.389	0.136011
A_42_P664238	Vps72_predicted	XM_227428		0.14	1.389	0.030023
A_43_P12863	Dut	NM_053592	497778	0.14	1.388	0.049445
A_44_P515334	Dgat1	NM_053437	84497	0.14	1.388	0.067657
A_44_P291172	A_44_P291172	A_44_P291172		0.14	1.388	0.044491
A_44_P1039606	RGD1309383	NM_001024257	307805	0.14	1.388	0.053219
A_44_P449555	Yif1	NM_172017	171441	0.14	1.388	0.07373
A_44_P282276	M17068	M17068		0.14	1.388	0.047561
A_44_P790928	A_44_P790928	A_44_P790928		0.14	1.388	0.009965
A_44_P557575	RGD1563264_predicted	XM_217716		0.14	1.388	0.020938
A_44_P441129	RGD1559724_predicted	XM_234128		0.14	1.388	0.030306
A_44_P189813	Glt25d1_predicted	XM_214295		0.14	1.387	0.024419
A_44_P1013157	RGD1309634_predicted	XM_223536	305452	0.14	1.387	0.104699
A_42_P546857	RGD1565675_predicted	XM_214404	290925	0.14	1.387	0.008879
A_42_P565275	Aspm_predicted	XM_213891		0.14	1.387	0.119879
A_43_P12430	Mtpn	NM_024374	79215	0.14	1.387	0.030591
A_42_P561835	Chd1l_predicted	XM_227510		0.14	1.387	0.073746
A_43_P11380	LOC315508	NM_001034000	315508	0.14	1.387	0.013571
A_42_P585359	LOC293103	NM_001013897	293103	0.14	1.387	0.090094
A_44_P111197	AI044643	AI044643	685203	0.14	1.387	0.135903
A_44_P201315	LOC682080	XM_001058878		0.14	1.387	0.071274
A_42_P779260	Rbm19_predicted	XM_222200		0.14	1.387	0.066453
A_44_P574705	AA875646	AA875646		0.14	1.387	0.063003
A_44_P309266	Pbk_predicted	XM_224300		0.14	1.386	0.099051
A_44_P1000812	RGD1564681_predicted	XM_573648		0.14	1.386	0.013318
A_43_P13355	Bambi	NM_139082	83837	0.14	1.386	0.07058
A_44_P134685	Tpm3	NM_173111	117557	0.14	1.386	0.009526
A_44_P409582	Vcpip1	NM_176857	286761	0.14	1.386	0.019659
A_44_P185925	Phactr2	NM_214458	308291	0.14	1.386	0.251769
A_44_P500315	Lman1	NM_053886	116666	0.14	1.386	0.123107
A_43_P15267	Akt2	NM_017093	25233	0.14	1.386	0.025572
A_44_P182592	Lrrc8e	NM_001034139	304203	0.14	1.385	0.042288
A_44_P928735	LOC498266	NM_001017494	498266	0.14	1.385	0.090461

A_44_P103170	Eya3_predicted	XM_232735		0.14	1.385	0.044853
A_44_P158434	BF288087	BF288087	309217	0.14	1.385	0.199039
A_44_P1040999	Arih1	NM_001013108	300756	0.14	1.385	0.023068
A_44_P546170	XM_216109	XM_216109		0.14	1.385	0.025691
A_44_P976680	LOC681870	XM_001058771		0.14	1.385	0.065699
A_44_P245427	Trim39	NM_213562	309591	0.14	1.385	0.212094
A_44_P590374	LOC685955	XM_001065927		0.14	1.385	0.014747
A_42_P812263	Sat_mapped	NM_001007667	302642	0.14	1.385	0.140373
A_42_P619996	BF523561	BF523561		0.14	1.385	0.144526
A_44_P687542	LOC688018	XR_009418	688018	0.14	1.385	0.019451
A_44_P1004958	Thoc1	XM_229013		0.14	1.385	0.04935
A_44_P414974	XM_342637	XM_342637		0.14	1.384	0.02504
A_44_P1015388	Ankrd24_predicted	XM_216841		0.14	1.384	0.104185
A_42_P636350	Cyp11a1	NM_017286	29680	0.14	1.384	0.316622
A_44_P222353	RGD1309546_predicted	NM_001040128	292268	0.14	1.384	0.131454
A_43_P12157	Lif	NM_022196	60584	0.14	1.384	0.030043
A_44_P1008738	DV719651	DV719651	79559	0.14	1.384	0.026673
A_44_P436859	Mre11a	NM_022279	64046	0.14	1.384	0.105429
A_44_P1012502	Ddx24	NM_199119	373065	0.14	1.384	0.009942
A_42_P521006	LOC293589	XM_001056886		0.14	1.384	0.023219
A_44_P386799	Nudt9	NM_001006991	305149	0.14	1.384	0.06193
A_44_P119226	Ankrd50_predicted	XM_215553	294988	0.14	1.384	0.061283
A_44_P432452	Akr1b4	NM_012498	24192	0.14	1.384	0.043
A_43_P18281	LOC681004	XM_001057305	681004	0.14	1.384	0.067206
A_44_P262025	Gtf3a	XM_001069721		0.14	1.384	0.021987
A_42_P665460	AW144706	AW144706		0.14	1.383	0.007733
A_44_P740817	BF522747	BF522747		0.14	1.383	0.172647
A_42_P814107	Pold3	NM_001024750	293144	0.14	1.383	0.011158
A_44_P262401	Gcsh	BC088114	171133	0.14	1.383	0.040457
A_44_P440870	Sptlc1_predicted	XM_341495		0.14	1.383	0.067985
A_43_P10233	Vav2_predicted	XM_216030		0.14	1.383	0.062097
A_44_P761172	TC553104	TC553104		0.14	1.383	0.130655
A_42_P804387	Lypd3	NM_021759	60378	0.14	1.383	0.148178
A_44_P356658	Pdzd4_predicted	XM_219716	293856	0.14	1.383	0.032298
A_44_P404219	RGD1566282_predicted	XM_236533	315911	0.14	1.383	0.09539
A_44_P396123	S100a16_predicted	XM_342291		0.14	1.383	0.089337
A_42_P736889	XM_228841	XM_228841		0.14	1.383	0.084011
A_44_P238392	Mrpl49	XM_219525		0.14	1.383	0.090694
A_44_P406957	AW914303	AW914303	303601	0.14	1.383	0.084393
A_44_P153617	AA900122	AA900122	24533	0.14	1.383	0.045902
A_42_P548067	Blvrb_predicted	XM_214823		0.14	1.383	0.031766
A_44_P994550	Rarsl_predicted	XM_216367		0.14	1.383	0.011393
A_44_P425465	BF289943	BF289943	362237	0.14	1.382	0.100113
A_44_P313215	AI010816	AI010816		0.14	1.382	0.080072
A_44_P475840	Med19_predicted	XM_230275		0.14	1.382	0.017072
A_44_P376091	Rgnef_predicted	XM_342179		0.14	1.382	0.061882
A_44_P111453	Armc1_predicted	XM_215537		0.14	1.382	0.249943
A_42_P544965	Rpl22	NM_031104	81768	0.14	1.382	0.01029
A_42_P489167	Htra4_predicted	XM_224963		0.14	1.382	0.048297
A_44_P473741	Ube2z	NM_001037643	303478	0.14	1.382	0.031838
A_44_P515107	Stch	NM_019271	29734	0.14	1.382	0.127784
A_44_P278827	XM_218912	XM_218912		0.14	1.381	0.025431
A_42_P648006	AW915161	AW915161		0.14	1.381	0.084222
A_44_P807329	TC538614	TC538614		0.14	1.381	0.029247
A_44_P357783	BQ201465	BQ201465		0.14	1.381	0.20909
A_44_P452668	Sumf1_predicted	XM_342731		0.14	1.381	0.030168
A_43_P10568	CB544298	CB544298		0.14	1.381	0.083112
A_44_P820780	AW917383	AW917383		0.14	1.381	0.023356
A_44_P131177	A_44_P131177	A_44_P131177		0.14	1.381	0.234863
A_44_P296924	Rdh10	NM_181478	353252	0.14	1.381	0.101141
A_43_P19089	Med4	NM_001024256	306030	0.14	1.381	0.009914
A_44_P727480	AW142602	AW142602		0.14	1.381	0.03411
A_44_P419625	LOC690701	XM_001075285		0.14	1.381	0.019272

A_44_P957310	Snx6_predicted	XM_343062		0.14	1.381	0.095241
A_44_P806917	XM_580045	XM_580045		0.14	1.381	0.036288
A_43_P10683	Ppil2	NM_001017383	360746	0.14	1.380	0.14466
A_44_P229376	Sdad1	NM_001006958	289504	0.14	1.380	0.032413
A_44_P751748	TC528618	TC528618		0.14	1.380	0.511835
A_44_P501005	Olr323_predicted	NM_001000245	293761	0.14	1.380	0.171504
A_44_P281882	RGD1560047_predicted	XM_215401	294429	0.14	1.380	0.089124
A_44_P408151	Jmjd4_predicted	XM_220561		0.14	1.380	0.085112
A_44_P359190	Gabpa_predicted	XM_344002		0.14	1.380	0.022924
A_44_P110594	AI012859	AI012859	359726	0.14	1.380	0.039387
A_44_P1028765	RGD1310066	XM_343221	362894	0.14	1.380	0.030377
A_44_P177742	Sart1	NM_031596	29678	0.14	1.380	0.013858
A_44_P264293	Maoa	XM_343764	29253	0.14	1.380	0.038328
A_44_P469113	Slfn2_predicted	XM_220779		0.14	1.380	0.126628
A_44_P358664	Stim2_predicted	XM_223454		0.14	1.380	0.150514
A_43_P12133	Epn2	NM_021852	60443	0.14	1.380	0.040595
A_44_P212934	LOC361571	NM_001033706	361571	0.14	1.380	0.026229
A_44_P605271	AW143544	AW143544	353252	0.14	1.380	0.029594
A_44_P776556	Chac1_predicted	XM_001080834		0.14	1.380	0.032955
A_44_P362541	Scoc	NM_001013235	364981	0.14	1.380	0.047018
A_44_P550337	Apxl	XM_228847		0.14	1.379	0.031503
A_44_P338068	LOC500909	NM_001024355	500909	0.14	1.379	0.072334
A_44_P179422	Unr	NM_054006	117180	0.14	1.379	0.034497
A_44_P377355	ENSRNOT00000019073	ENSRNOT00000019073		0.14	1.379	0.018771
A_44_P373629	Ccnc	D14013		0.14	1.379	0.12051
A_44_P550702	Polr3d	NM_001031653	306012	0.14	1.379	0.031875
A_44_P959751	A_44_P959751	A_44_P959751		0.14	1.379	0.03262
A_44_P1017913	Dars2	NM_001034143	304919	0.14	1.379	0.28014
A_44_P131248	XM_237105	XM_237105		0.14	1.378	0.031158
A_42_P737542	RGD1564603_predicted	XM_575208	499866	0.14	1.378	0.018551
A_44_P505282	Coq7	NM_012785	25249	0.14	1.378	0.110689
A_44_P521545	Trnt1	NM_001024261	312616	0.14	1.378	0.021931
A_43_P10934	Fbxo38_predicted	XM_225885		0.14	1.378	0.082704
A_42_P552341	Sorl1_predicted	XM_217115	300652	0.14	1.378	0.010187
A_42_P778115	Phlda2_predicted	XM_215132	293637	0.14	1.378	0.052537
A_44_P144665	XM_218548	XM_218548		0.14	1.378	0.180743
A_44_P575923	TC567857	TC567857		0.14	1.378	0.011186
A_44_P159004	XM_222829	XM_222829		0.14	1.378	0.021449
A_44_P230219	BF556147	BF556147		0.14	1.378	0.02747
A_43_P22613	Stk4_predicted	XM_230833		0.14	1.378	0.012869
A_44_P537673	Zfp297b	NM_001012094	311872	0.14	1.378	0.103693
A_44_P147693	XM_220544	XM_220544		0.14	1.378	0.010063
A_44_P700547	CV079334	CV079334		0.14	1.378	0.088189
A_44_P956049	RGD1307395	XM_001055505		0.14	1.378	0.478093
A_43_P18069	RGD1309414_predicted	XM_341279	361004	0.14	1.378	0.016981
A_44_P410232	Dmtf1	NM_053693	114485	0.14	1.378	0.10883
A_42_P600777	Xab2	NM_139109	245976	0.14	1.377	0.017989
A_44_P116711	A_44_P116711	A_44_P116711		0.14	1.377	0.137716
A_44_P400223	Zmynd11	NM_203367	291259	0.14	1.377	0.040866
A_44_P898566	LOC366355	XR_008382	366355	0.14	1.377	0.071975
A_44_P324891	CN544457	CN544457	362592	0.14	1.377	0.016497
A_42_P510241	Brms1	NM_001009605	293668	0.14	1.377	0.012773
A_44_P1007333	Gle1l	NM_001025731	362098	0.14	1.377	0.011156
A_44_P332444	Pik3c3	NM_022958	65052	0.14	1.377	0.228223
A_43_P12764	Degs1	NM_053323	58970	0.14	1.376	0.085534
A_44_P1019148	Ckap1_predicted	NM_001040180	292777	0.14	1.376	0.008495
A_42_P826853	Tnfrsf17_predicted	XM_213211		0.14	1.376	0.026212
A_44_P137742	RGD1311005_predicted	XM_341099		0.14	1.376	0.026192
A_44_P482894	RGD1564469_predicted	XM_227546		0.14	1.376	0.097814
A_42_P749820	Cdh13	NM_138889	192248	0.14	1.376	0.161285
A_43_P14228	Cotl1_predicted	XM_341700		0.14	1.376	0.090329
A_44_P1046845	RGD1309016	NM_001009391	305177	0.14	1.376	0.00903
A_42_P570300	Depdc5_predicted	XM_223584		0.14	1.376	0.014406

A_44_P255642	RGD1306721	XM_342602	362288	0.14	1.376	0.014753
A_44_P373389	Atxn2_predicted	XM_001079639		0.14	1.376	0.075187
A_44_P552764	Tpp1	NM_031357	83534	0.14	1.376	0.017852
A_44_P311468	Snx27	NM_152847	260323	0.14	1.376	0.041132
A_44_P1055903	RGD1563278_predicted	XM_221487	303963	0.14	1.376	0.049472
A_42_P575140	Yy1	XM_001069615		0.14	1.375	0.041776
A_43_P12651	Limk1	NM_031727	65172	0.14	1.375	0.056548
A_44_P159810	AI237189	AI237189		0.14	1.375	0.031827
A_42_P598733	Gs3	XM_001060252		0.14	1.375	0.013018
A_44_P302599	RGD1563141_predicted	XM_235483	315135	0.14	1.375	0.05627
A_44_P234624	Smurf2_predicted	XM_221048		0.14	1.375	0.068576
A_43_P14646	Kars	NM_001006967	292028	0.14	1.375	0.028532
A_43_P18712	RGD1309482	NM_001014246	365458	0.14	1.375	0.021428
A_44_P220217	RGD1562808_predicted	XM_227719		0.14	1.375	0.007196
A_44_P1071355	Usp10	NM_001034146	307905	0.14	1.375	0.014362
A_43_P10191	Rnf7_predicted	XM_217235		0.14	1.375	0.076137
A_44_P696494	BI395606	BI395606	288702	0.14	1.375	0.031024
A_44_P1036268	Atxn2l_predicted	XM_341928	361649	0.14	1.375	0.05393
A_44_P496541	Hnrpl	XM_001068191	80846	0.14	1.374	0.026823
A_43_P13167	Crebbp	NM_133381	54244	0.14	1.374	0.076216
A_44_P550738	Rsrc1	NM_001014172	361956	0.14	1.374	0.026117
A_44_P335278	ENSRNOT00000030280	ENSRNOT00000030280		0.14	1.374	0.030582
A_44_P457218	Ddx28_predicted	XM_344762		0.14	1.374	0.122553
A_42_P703751	RGD1309441_predicted	XM_213467		0.14	1.374	0.011303
A_44_P947936	TC565055	TC565055		0.14	1.374	0.076682
A_42_P533219	Slc30a6_predicted	XM_216643		0.14	1.374	0.075823
A_43_P18267	RGD1309016	NM_001009391	305177	0.14	1.374	0.035467
A_44_P295478	Slc38a6	NM_001013099	299139	0.14	1.374	0.057155
A_44_P154327	A_44_P154327	A_44_P154327		0.14	1.374	0.066446
A_44_P207721	Adss_predicted	XM_222946		0.14	1.374	0.033796
A_44_P475919	Adamts14_predicted	XM_228290		0.14	1.373	0.221917
A_43_P18086	RGD1310311	NM_001014197	362746	0.14	1.373	0.01807
A_42_P678414	RGD1563106_predicted	XM_220595		0.14	1.373	0.063326
A_44_P295522	Mll_mapped	XM_236194		0.14	1.373	0.029164
A_44_P883557	ENSRNOT00000034207	ENSRNOT00000034207		0.14	1.373	0.030861
A_44_P251081	RGD1562969_predicted	XM_221081	287803	0.14	1.373	0.064496
A_44_P371091	BF283568	BF283568		0.14	1.373	0.112853
A_43_P14255	AW915423	AW915423		0.14	1.372	0.254355
A_44_P748640	RGD1565449_predicted	XM_001075629		0.14	1.372	0.011609
A_44_P215898	XM_212703	XM_212703		0.14	1.372	0.014567
A_44_P206292	XM_216266	XM_216266		0.14	1.372	0.146953
A_44_P329784	RGD1565584_predicted	XM_001062098		0.14	1.372	0.180042
A_44_P217946	Rassf2	NM_001037096	311437	0.14	1.372	0.193664
A_44_P231066	LOC314432	NM_001014080	314432	0.14	1.372	0.114175
A_44_P148773	AI169077	AI169077	83509	0.14	1.372	0.1173
A_44_P466407	RGD1309985_predicted	XM_342231	361931	0.14	1.372	0.087496
A_44_P217785	RGD1306062_predicted	XM_226606	294595	0.14	1.372	0.124366
A_44_P219780	Surf2	NM_001033866	619345	0.14	1.372	0.07776
A_42_P538232	AI030852	AI030852		0.14	1.372	0.324911
A_44_P776032	A_44_P776032	A_44_P776032		0.14	1.371	0.063565
A_44_P543930	Zfp410_predicted	XM_234409		0.14	1.371	0.076431
A_44_P511101	RGD1560916_predicted	XM_341573	361288	0.14	1.371	0.358145
A_42_P635258	RGD1306064_predicted	XM_224581	306229	0.14	1.371	0.102589
A_44_P170351	Vamp5	NM_053555	89818	0.14	1.371	0.036162
A_43_P17392	Arhgap21_predicted	XM_225628	307178	0.14	1.371	0.022401
A_44_P439995	Mkl1_predicted	XM_235497	315151	0.14	1.371	0.024745
A_44_P425076	Pik4cb	NM_031083	81747	0.14	1.371	0.012251
A_44_P965236	TC562998	TC562998		0.14	1.371	0.135921
A_44_P367680	Odf2	NM_017213	29479	0.14	1.371	0.039994
A_43_P15937	Sah	NM_033231	24763	0.14	1.371	0.115893
A_42_P799947	Sfrs14_predicted	XM_001071083		0.14	1.371	0.082924
A_42_P618538	Lta4h	NM_001030031	299732	0.14	1.370	0.053345
A_44_P578054	TC562057	TC562057		0.14	1.370	0.135101

A_44_P683666	Cpsf6_predicted	XM_001080973		0.14	1.370	0.045138
A_44_P215210	Ptprt_predicted	XM_342568		0.14	1.370	0.064263
A_44_P762543	TC525657	TC525657		0.14	1.370	0.2683
A_44_P546459	Psmd12	NM_001005875	287772	0.14	1.370	0.043345
A_44_P1013894	AW918228	AW918228	60336	0.14	1.370	0.027524
A_44_P1013048	RGD1310597	NM_001025647	294667	0.14	1.370	0.080265
A_44_P377520	LOC365983	XM_345317		0.14	1.370	0.012645
A_44_P187144	Hnrph1	NM_080896	140931	0.14	1.370	0.0327
A_43_P15512	Gpr175	NM_053534	85494	0.14	1.370	0.0108
A_42_P770458	Exoc2	NM_134414	171455	0.14	1.370	0.023208
A_44_P821841	A_44_P821841	A_44_P821841		0.14	1.370	0.107366
A_44_P819390	AW143969	AW143969		0.14	1.370	0.048143
A_44_P194591	RGD1563354_predicted	XM_230799	311592	0.14	1.370	0.19899
A_43_P19036	Thrap3	NM_001009693	313591	0.14	1.370	0.016662
A_44_P132411	RGD1310230	NM_001039015	301563	0.14	1.370	0.060333
A_43_P23093	LOC687696	XM_001080504		0.14	1.369	0.031041
A_44_P713490	LOC365354	XR_009200	365354	0.14	1.369	0.065094
A_44_P218186	Tbc1d2b	XM_236472		0.14	1.369	0.042436
A_44_P414789	RGD1565370_predicted	XM_212922		0.14	1.369	0.021911
A_44_P704181	RGD1560335_predicted	XM_001060543		0.14	1.369	0.387113
A_44_P884221	TC520871	TC520871		0.14	1.369	0.050894
A_44_P434844	Rqcd1	NM_001009357	301513	0.14	1.369	0.026105
A_44_P243262	RGD1562378_predicted	XM_575704		0.14	1.369	0.103315
A_44_P212703	Kcnq1	NM_032073	84020	0.14	1.369	0.05515
A_43_P19051	RGD1560686_predicted	XM_001073292		0.14	1.368	0.117851
A_42_P696769	AW144236	AW144236		0.14	1.368	0.013415
A_42_P813095	Leng4_predicted	XM_001056203		0.14	1.368	0.209572
A_42_P818546	LOC291964	XM_001072430		0.14	1.368	0.053856
A_44_P159743	LOC679572	XM_001053332	679572	0.14	1.368	0.04514
A_44_P522874	Zbed3	NM_001025729	361881	0.14	1.368	0.017692
A_44_P808350	DV727526	DV727526		0.14	1.368	0.060049
A_44_P541053	Fbln5	NM_019153	29158	0.14	1.368	0.021614
A_44_P412121	Zfp335	XM_342578	259270	0.14	1.368	0.056895
A_44_P958992	LOC499224	XR_008920	499224	0.14	1.368	0.166081
A_44_P396397	Smarce1_predicted	XM_234076		0.14	1.368	0.172267
A_43_P14359	RGD1308009	XM_001056829		0.14	1.367	0.037427
A_44_P212708	Psmd4	NM_031331	83499	0.14	1.367	0.014017
A_44_P351118	Hmmr	NM_012964	25460	0.14	1.367	0.119899
A_44_P226960	LOC685867	XM_001065562		0.14	1.367	0.115956
A_44_P432314	RGD1305486	NM_001014073	313873	0.14	1.367	0.018451
A_43_P10706	Tbpl1_predicted	XM_001056405		0.14	1.367	0.008423
A_44_P138231	XM_236238	XM_236238		0.14	1.367	0.0691
A_44_P368703	BF283115	BF283115	362751	0.14	1.367	0.06796
A_44_P682439	Prcp_predicted	XM_214993		0.14	1.367	0.046908
A_44_P253023	RGD1565516_predicted	XM_227009		0.14	1.367	0.02111
A_43_P18437	Chpt1	NM_001007750	362866	0.14	1.366	0.124471
A_44_P240819	RGD1311563	AY310157	363160	0.14	1.366	0.050317
A_44_P511088	Abl1_mapped	XM_231137	311860	0.14	1.366	0.00768
A_44_P220277	Ext2_predicted	XM_230303		0.14	1.366	0.084109
A_44_P231283	Nupl1	NM_139091	245922	0.14	1.366	0.040205
A_43_P19806	XM_341175	XM_341175		0.14	1.366	0.037863
A_44_P283366	Myst2	NM_181081	303470	0.14	1.366	0.104761
A_44_P110252	RGD1308616_predicted	XM_342892	362573	0.14	1.366	0.1075
A_44_P484644	RGD708449	NM_173324	286990	0.14	1.366	0.024443
A_44_P544297	Wbp11	NM_001009661	297695	0.14	1.366	0.015651
A_44_P159745	LOC679572	XM_001053332	679572	0.14	1.366	0.0262
A_42_P562625	Xpnpep1	NM_131913	170751	0.14	1.365	0.017255
A_44_P395928	Ap4m1	NM_001037977	304344	0.14	1.365	0.012879
A_44_P191941	Pex6	NM_057125	117265	0.14	1.365	0.023407
A_44_P1033194	LOC246187	XM_574233	246187	0.14	1.365	0.022557
A_43_P15761	Gria1	NM_031608	50592	0.14	1.365	0.024857
A_42_P647009	Mrip	NM_053814	116504	0.14	1.365	0.023482
A_44_P457129	LOC310958	XM_227809	310958	0.14	1.365	0.054555

A_44_P282853	Smarcd2	NM_031983	83833	0.14	1.365	0.02827
A_44_P332712	NP659940	NP659940		0.14	1.365	0.083689
A_44_P545988	RGD1306353_predicted	XM_224235		0.14	1.365	0.042512
A_43_P17643	RGD1566094_predicted	XM_576181	500793	0.14	1.365	0.034761
A_44_P314301	Zfp46_predicted	XM_233568		0.14	1.365	0.04142
A_44_P1039871	RGD1311251	NM_001024265	315665	0.13	1.364	0.053596
A_44_P667896	RGD1560011_predicted	XM_001073577		0.13	1.364	0.11371
A_43_P13264	Lphn2	NM_134408	171447	0.13	1.364	0.174162
A_42_P496786	Trap1	NM_001039001	287069	0.13	1.364	0.050797
A_44_P211090	RGD1311136	XM_216835	299608	0.13	1.364	0.032592
A_44_P365580	Sqle	NM_017136	29230	0.13	1.364	0.093738
A_44_P237915	Eif3s3	NM_198751	299899	0.13	1.364	0.007042
A_44_P313971	LOC689625	XM_001071416	689625	0.13	1.364	0.066383
A_44_P262490	XM_217974	XM_217974		0.13	1.364	0.062691
A_42_P688027	Crhr1	NM_030999	58959	0.13	1.364	0.066913
A_43_P12571	Jak2	NM_031514	24514	0.13	1.364	0.016205
A_44_P314169	Fuk_predicted	XM_226508		0.13	1.364	0.037877
A_44_P321352	RGD1305725_predicted	XM_342561	362255	0.13	1.364	0.144012
A_44_P530196	RGD1566252_predicted	XM_345841		0.13	1.364	0.069454
A_44_P976576	LOC679811	XM_001054550	679811	0.13	1.364	0.305199
A_44_P243794	ENSRNOT00000031905	ENSRNOT00000031905		0.13	1.364	0.092787
A_43_P18275	LOC363266	BC099202	363266	0.13	1.364	0.023382
A_44_P592479	TC553470	TC553470		0.13	1.364	0.040017
A_43_P16644	Umps	NM_001025402	288051	0.13	1.364	0.030361
A_44_P605605	A_44_P605605	A_44_P605605		0.13	1.363	0.042207
A_44_P310851	LOC682071	XM_001058229		0.13	1.363	0.044002
A_43_P17296	Sf4	NM_001011920	290666	0.13	1.363	0.016359
A_43_P14620	Sod1	NM_017050	24786	0.13	1.363	0.019164
A_43_P12970	Rere	NM_053885	116665	0.13	1.363	0.058008
A_44_P981679	RGD1560783_predicted	XM_001078208		0.13	1.363	0.137615
A_44_P530153	LOC314600	XM_001081510		0.13	1.363	0.181059
A_43_P18454	RGD1564491_predicted	XM_221305	303812	0.13	1.363	0.091168
A_44_P438613	Amid_predicted	XM_342137		0.13	1.363	0.043412
A_44_P821528	A_44_P821528	A_44_P821528		0.13	1.362	0.068532
A_42_P550702	Znhit2_predicted	XM_219526		0.13	1.362	0.009132
A_44_P187374	Rab5c_predicted	XM_001081435		0.13	1.362	0.012423
A_44_P202608	AW141911	AW141911	501282	0.13	1.362	0.032512
A_44_P233612	Ogfr	NM_053340	83525	0.13	1.362	0.020147
A_44_P318255	Commd3	NM_198732	291339	0.13	1.362	0.158968
A_42_P629721	RGD1304906_predicted	XM_214703		0.13	1.362	0.024915
A_43_P11663	Rds	NM_013021	25534	0.13	1.362	0.146729
A_44_P107461	Faf1	NM_130406	140657	0.13	1.361	0.121872
A_44_P116555	Cd6	NM_175577	25752	0.13	1.361	0.096323
A_44_P411796	RGD1309482	NM_001014246	365458	0.13	1.361	0.095609
A_44_P489474	Ubc2e	NM_001037292	641452	0.13	1.361	0.031799
A_44_P274755	Jrk_predicted	XM_235416		0.13	1.361	0.022199
A_44_P777199	Mrrf	NM_001008354	311903	0.13	1.361	0.027665
A_44_P270510	Aff4_predicted	XM_220420		0.13	1.361	0.127153
A_44_P1040796	Atp6v1g1_predicted	XM_216411		0.13	1.361	0.034458
A_44_P998312	Zdhhc6	NM_001037652	361771	0.13	1.361	0.038867
A_44_P218241	Tgfbrap1_predicted	XM_237113		0.13	1.360	0.00803
A_44_P289312	BF557240	BF557240		0.13	1.360	0.09204
A_44_P525534	RGD1562381_predicted	XM_234319		0.13	1.360	0.01241
A_43_P11144	Pdrg1	NM_001014762	296278	0.13	1.360	0.010797
A_43_P15890	Nfic	XM_345789		0.13	1.360	0.037086
A_44_P192018	A_44_P192018	A_44_P192018		0.13	1.360	0.123248
A_44_P732488	TC567669	TC567669		0.13	1.360	0.12869
A_44_P178172	RGD1559531_predicted	XM_226370		0.13	1.360	0.045902
A_44_P1054883	AY011335	AY011335		0.13	1.360	0.04003
A_44_P378749	RGD1564605_predicted	XM_347220	368057	0.13	1.360	0.13919
A_44_P329688	RGD1307915_predicted	XM_001057982		0.13	1.360	0.080781
A_43_P22020	RGD1565575_predicted	XM_342400	362096	0.13	1.360	0.076757
A_44_P356262	Pld3	NM_001012167	361527	0.13	1.359	0.083463

A_44_P113247	AA848526	AA848526		0.13	1.359	0.042444
A_44_P520829	Pik3r4_predicted	XM_343466		0.13	1.359	0.070521
A_44_P415199	Tubgcp6_predicted	XM_343310		0.13	1.359	0.042337
A_44_P137802	Smpd1	NM_001006997	308909	0.13	1.359	0.061885
A_44_P869091	LOC680222	XM_001056172	680222	0.13	1.359	0.022395
A_44_P464221	RGD1566373_predicted	XM_345098		0.13	1.359	0.011862
A_44_P438394	XM_215147	XM_215147		0.13	1.359	0.033683
A_44_P671470	A_44_P671470	A_44_P671470		0.13	1.359	0.126964
A_44_P512820	Rps29	NM_012876	25348	0.13	1.359	0.104757
A_44_P170244	LOC691538	XM_001078734	686263	0.13	1.359	0.272724
A_44_P557106	AA817887	AA817887	64303	0.13	1.359	0.046966
A_43_P15304	Grb2	NM_030846	81504	0.13	1.359	0.020307
A_44_P995051	Ssx2ip	NM_175597	308023	0.13	1.359	0.406669
A_44_P245825	XM_345039	XM_345039		0.13	1.358	0.02781
A_42_P581845	Smn1	NM_022509	64301	0.13	1.358	0.032809
A_44_P215634	RGD1564253_predicted	XM_228876	317423	0.13	1.358	0.069108
A_44_P168335	XM_341734	XM_341734		0.13	1.358	0.01822
A_42_P746212	Spr	XM_001069367		0.13	1.358	0.035087
A_44_P159586	BF565379	BF565379	309957	0.13	1.358	0.084013
A_44_P499088	LOC685332	XM_001063377	685332	0.13	1.358	0.051965
A_44_P209548	AW915893	AW915893		0.13	1.358	0.029474
A_42_P472341	AW918453	AW918453		0.13	1.358	0.042128
A_44_P684463	TC540481	TC540481		0.13	1.358	0.187111
A_44_P1031056	Plekha3	NM_001013077	295674	0.13	1.358	0.019821
A_44_P109617	Ybx1	NM_031563	500538	0.13	1.358	0.09172
A_44_P209821	LOC687307	XM_001072658		0.13	1.357	0.079928
A_44_P135464	Dcun1d4_predicted	XM_341206		0.13	1.357	0.161285
A_42_P658264	Nfkb2	NM_001008349	309452	0.13	1.357	0.007966
A_44_P123898	XM_341452	XM_341452		0.13	1.357	0.040281
A_43_P11540	Adra2a	NM_012739	25083	0.13	1.357	0.278253
A_44_P668591	TC521506	TC521506		0.13	1.357	0.161607
A_42_P733295	Armc5	NM_001009455	361653	0.13	1.357	0.021883
A_44_P344999	Mphosph6_predicted	XM_001080959		0.13	1.357	0.047275
A_44_P696483	LOC300472	XM_217099	300472	0.13	1.357	0.035445
A_44_P333296	LOC680027	XM_001055214	680027	0.13	1.357	0.058892
A_44_P115378	Ssbp3	NM_053358	84354	0.13	1.357	0.065706
A_44_P989302	Kif2	XM_345150	84391	0.13	1.357	0.212244
A_43_P21719	CB547029	CB547029	300679	0.13	1.357	0.010663
A_44_P137981	XM_225973	XM_225973		0.13	1.357	0.145751
A_44_P690201	DY318971	DY318971	316737	0.13	1.356	0.189016
A_44_P267541	XM_228492	XM_228492		0.13	1.356	0.026362
A_44_P381483	LOC289233	XM_213930	289233	0.13	1.356	0.034476
A_44_P327830	Bysl	NM_182674	359727	0.13	1.356	0.017277
A_44_P245472	Eif1a	NM_001008773	317163	0.13	1.356	0.124482
A_44_P496899	LOC362068	XM_001056150		0.13	1.356	0.152618
A_44_P257126	AI011321	AI011321	245976	0.13	1.356	0.301796
A_44_P1046937	RGD1305492_predicted	XM_001066754		0.13	1.356	0.101706
A_44_P173211	Mrpl47	NM_001037183	294963	0.13	1.356	0.181958
A_44_P139869	Lr8	NM_134390	171411	0.13	1.356	0.089964
A_44_P384677	RGD1563384_predicted	XM_228392		0.13	1.356	0.14136
A_44_P332310	Zfp180	NM_144757	246279	0.13	1.356	0.111368
A_44_P203195	Madd	NM_053585	94193	0.13	1.356	0.056305
A_44_P422271	Cerk_predicted	XM_217019	300129	0.13	1.356	0.081187
A_44_P701459	TC562803	TC562803		0.13	1.356	0.341302
A_43_P16769	RGD1305274_predicted	XM_216484		0.13	1.356	0.019281
A_42_P725471	Ptpn21	NM_133545	171070	0.13	1.356	0.06716
A_44_P833796	AW918437	AW918437	360573	0.13	1.356	0.100426
A_44_P807649	TC556681	TC556681		0.13	1.355	0.03367
A_44_P323879	RGD1308233_predicted	XM_228117		0.13	1.355	0.050705
A_42_P547945	Mtch1	XM_215358	294313	0.13	1.355	0.050155
A_44_P476005	Scmh1_predicted	XM_342900		0.13	1.355	0.029144
A_43_P22468	Abca4_predicted	XM_241525		0.13	1.355	0.157276
A_44_P180334	RGD1562510_predicted	XM_223924		0.13	1.355	0.025929

A_44_P412916	XM_237097	XM_237097		0.13	1.355	0.060984
A_44_P130416	Olr614_predicted	NM_001000337	295839	0.13	1.355	0.130818
A_44_P158805	Pcdhb22	XM_001056235		0.13	1.355	0.042841
A_43_P17852	RGD1560552_predicted	XM_001075510		0.13	1.355	0.239863
A_42_P790241	Mrpl32_predicted	XM_214491		0.13	1.355	0.041553
A_44_P1010226	Mrpl1_predicted	XM_214008		0.13	1.354	0.008918
A_44_P494147	Nelf	NM_057190	117536	0.13	1.354	0.009236
A_44_P290688	XM_220073	XM_220073		0.13	1.354	0.055058
A_44_P668313	RGD1560011_predicted	XM_001073577		0.13	1.354	0.081445
A_43_P22203	Zfp406_predicted	XM_343255	362925	0.13	1.354	0.151881
A_44_P379976	Lrrc8	NM_001024782	311846	0.13	1.354	0.031326
A_44_P516074	RGD1565496_predicted	XM_217180		0.13	1.354	0.092291
A_44_P536645	TC541896	TC541896		0.13	1.354	0.035746
A_44_P852241	LOC365777	XR_008696	365777	0.13	1.354	0.107679
A_43_P11313	Rbm17	NM_001013058	291295	0.13	1.354	0.048173
A_44_P308359	AI180270	AI180270	498256	0.13	1.354	0.016033
A_44_P194936	AW520758	AW520758		0.13	1.354	0.319955
A_44_P124265	RGD1559808_predicted	XM_236845		0.13	1.354	0.013601
A_44_P142454	RGD1563050_predicted	XM_226772		0.13	1.354	0.109369
A_44_P142163	Skil	XM_001057072	114208	0.13	1.354	0.067651
A_44_P508945	Heyl_predicted	XM_233495		0.13	1.354	0.13422
A_42_P456277	XM_216243	XM_216243		0.13	1.353	0.063393
A_44_P418163	Tpd52l2	NM_198744	296480	0.13	1.353	0.020165
A_43_P11155	TC535241	TC535241		0.13	1.353	0.046873
A_44_P370365	XM_223850	XM_223850		0.13	1.353	0.070006
A_44_P691466	TC526026	TC526026		0.13	1.353	0.20414
A_44_P281393	XM_344684	XM_344684		0.13	1.353	0.045307
A_44_P356715	XM_234741	XM_234741		0.13	1.353	0.234716
A_44_P961262	Epc2_predicted	XM_001055791		0.13	1.353	0.050007
A_43_P21584	XM_230983	XM_230983		0.13	1.353	0.029331
A_44_P149412	Ddx39	NM_053563	89827	0.13	1.353	0.019977
A_44_P264600	Fbxo16	NM_001013132	305970	0.13	1.353	0.058446
A_44_P360685	Mug1	M22360	497794	0.13	1.353	0.41882
A_44_P126366	RGD1309792	NM_001017456	312199	0.13	1.353	0.021793
A_44_P751206	AI236146	AI236146	298648	0.13	1.353	0.131324
A_44_P236350	A_44_P236350	A_44_P236350		0.13	1.353	0.037092
A_44_P274823	Mrpl27_predicted	XM_213439		0.13	1.353	0.02781
A_44_P143797	Chd7_predicted	XM_232671		0.13	1.352	0.118475
A_44_P100790	RGD1310012_predicted	XM_233376	313490	0.13	1.352	0.051938
A_43_P19599	Bbx_predicted	XM_221497		0.13	1.352	0.090336
A_44_P522293	Ufc1	NM_001003709	445268	0.13	1.352	0.01125
A_44_P1020967	LOC680537	XM_001058413	680537	0.13	1.352	0.062643
A_42_P726413	RGD1306746_predicted	XM_232140		0.13	1.352	0.236146
A_44_P332543	Tomm22	NM_212514	300075	0.13	1.352	0.101712
A_44_P283812	LOC310721	NM_001014041	310721	0.13	1.352	0.085652
A_42_P587720	Slc38a5	NM_138854	192208	0.13	1.352	0.098671
A_44_P932059	LOC679890	XM_001055037		0.13	1.352	0.085666
A_44_P159922	RGD1309748_predicted	XM_220139		0.13	1.352	0.058432
A_44_P549826	AA996902	AA996902		0.13	1.352	0.243561
A_44_P198550	Ric8b	NM_175598	314681	0.13	1.351	0.156511
A_44_P807238	TC555196	TC555196		0.13	1.351	0.029392
A_44_P508066	Nnat	NM_053601	94270	0.13	1.351	0.518301
A_43_P13367	Rnh1	NM_139105	245964	0.13	1.351	0.030458
A_44_P959399	Sar1a	NM_001007739	361842	0.13	1.351	0.021558
A_44_P231849	Whsc1l1_predicted	XM_214371		0.13	1.351	0.090336
A_44_P344928	RGD1563375_predicted	XM_345546		0.13	1.351	0.045909
A_44_P1036513	RGD1566117_predicted	XM_227828	310982	0.13	1.351	0.036937
A_44_P445415	XM_218914	XM_218914		0.13	1.351	0.039991
A_44_P135666	RGD1562199_predicted	XM_230571	311404	0.13	1.351	0.038328
A_42_P671442	RGD1308082	NM_001009636	290995	0.13	1.350	0.02948
A_44_P229469	Bpy2ip1_predicted	XM_214321		0.13	1.350	0.238021
A_44_P533943	Rtn3	NM_080909	140945	0.13	1.350	0.188241
A_44_P282433	Akt1	NM_033230	24185	0.13	1.350	0.024379

A_44_P499372	Brwd3_predicted	XM_228518	317213	0.13	1.350	0.076763
A_44_P607813	Mll_mapped	XM_001067729		0.13	1.350	0.014295
A_44_P203117	Fnbp4	XM_230291	311183	0.13	1.350	0.036181
A_44_P340193	Hsdl1	NM_001024896	361418	0.13	1.350	0.01523
A_44_P369760	Igbp1	NM_031624	58845	0.13	1.350	0.021499
A_44_P217821	Bap1_predicted	XM_224614		0.13	1.350	0.056486
A_44_P127318	MGC114417	XM_001055014		0.13	1.350	0.035492
A_44_P166930	Aldh7a1	XM_214535	291450	0.13	1.350	0.104908
A_44_P219372	CA507680	CA507680	289388	0.13	1.350	0.067231
A_44_P213392	Psmc4	NM_057122	117262	0.13	1.350	0.031552
A_42_P781735	Dynll1	NM_053319	58945	0.13	1.350	0.014893
A_44_P698627	LOC683172	XM_001064776		0.13	1.350	0.02649
A_44_P166598	Crkl	NM_001008284	287942	0.13	1.350	0.028111
A_44_P506644	Cpsf1_predicted	XM_345849	366952	0.13	1.350	0.021751
A_42_P455785	Copb1	NM_080781	114023	0.13	1.350	0.041797
A_44_P252441	St3gal2	NM_031695	64442	0.13	1.350	0.070883
A_44_P354284	RGD1309802_predicted	XM_233480		0.13	1.349	0.088201
A_44_P776260	TC535167	TC535167		0.13	1.349	0.07616
A_44_P550981	Dmap1	NM_001015006	298447	0.13	1.349	0.031614
A_44_P159145	LOC362012	XM_342311	362012	0.13	1.349	0.091694
A_43_P18183	RGD1359108	NM_001007702	313155	0.13	1.349	0.05142
A_42_P768766	Hdac3	NM_053448	84578	0.13	1.349	0.022841
A_44_P272327	RGD1308796_predicted	XM_234837	299566	0.13	1.349	0.149729
A_44_P693682	TC556755	TC556755		0.13	1.349	0.098913
A_44_P898658	ENSRNOT00000048545	ENSRNOT00000048545		0.13	1.349	0.141753
A_44_P274397	Lass2	NM_001033700	310667	0.13	1.349	0.026468
A_44_P492053	Gtf2h1_predicted	XM_341863		0.13	1.349	0.030566
A_42_P770055	AI535554	AI535554		0.13	1.349	0.071107
A_44_P326717	LOC360932	XM_341209	360932	0.13	1.348	0.173856
A_44_P360409	Mapk14	NM_031020	81649	0.13	1.348	0.016519
A_44_P200638	RGD1565681_predicted	XM_577104	501702	0.13	1.348	0.156287
A_44_P206520	Lman2l_predicted	XM_217385		0.13	1.348	0.05309
A_42_P659432	BF558775	BF558775	367846	0.13	1.348	0.018923
A_44_P877632	ENSRNOT00000016111	ENSRNOT00000016111		0.13	1.348	0.008498
A_44_P198669	LOC500684	XM_001060736		0.13	1.348	0.030673
A_44_P193704	Btbd1	NM_001011932	293060	0.13	1.348	0.168393
A_44_P409645	Bmsc-UbP	NM_001004247	300744	0.13	1.348	0.03095
A_44_P698873	A_44_P698873	A_44_P698873		0.13	1.348	0.053613
A_44_P204173	A_44_P204173	A_44_P204173		0.13	1.348	0.03462
A_43_P17311	RGD1310828_predicted	XM_223542	305458	0.13	1.348	0.083098
A_44_P398753	Xpot_predicted	XM_235185		0.13	1.347	0.070077
A_44_P1051607	Ube2q_predicted	XM_215612		0.13	1.347	0.177661
A_43_P12922	Ubqln1	NM_053747	114590	0.13	1.347	0.021724
A_44_P555378	RGD1308324_predicted	XM_223308		0.13	1.347	0.08844
A_44_P326917	Gabrp	NM_031029	81658	0.13	1.347	0.104637
A_44_P485661	AA925321	AA925321		0.13	1.347	0.016662
A_44_P929695	Cdc2l6_predicted	XM_228203		0.13	1.347	0.105761
A_44_P492723	Arhgap8	NM_001004242	300115	0.13	1.347	0.03567
A_44_P954901	AI009258	AI009258		0.13	1.346	0.097769
A_44_P327602	AW143111	AW143111	295586	0.13	1.346	0.094773
A_44_P592184	TC522913	TC522913		0.13	1.346	0.082659
A_44_P546686	AI410446	AI410446	295500	0.13	1.346	0.285283
A_44_P837128	RGD1560157_predicted	XM_575412		0.13	1.346	0.09735
A_42_P609809	Ergic3_predicted	XM_215908		0.13	1.346	0.058146
A_44_P415003	Rpusd3_predicted	XM_342737		0.13	1.346	0.01096
A_44_P504221	Ccnt1_predicted	XM_235633		0.13	1.346	0.092311
A_44_P359169	RGD1561541_predicted	XM_340871		0.13	1.346	0.158697
A_44_P224789	Sccpdh	NM_001013985	305021	0.13	1.346	0.196731
A_44_P1038121	Adam8_predicted	XM_219470		0.13	1.346	0.104357
A_44_P746697	Pkp2	XM_001065241		0.13	1.346	0.258268
A_44_P426128	Olr1748_predicted	NM_001001428	405199	0.13	1.346	0.17185
A_44_P284094	RGD1311456_predicted	XM_343420	363089	0.13	1.346	0.070168
A_43_P16703	Tcte1l	NM_001013228	363448	0.13	1.346	0.172222

A_44_P150805	AA963280	AA963280		0.13	1.346	0.051376
A_44_P375148	ENSRNOT00000047113	ENSRNOT00000047113		0.13	1.346	0.117357
A_44_P156254	Znf382	NM_144749	246264	0.13	1.346	0.047835
A_44_P991833	Lsm3_predicted	XM_216220		0.13	1.345	0.080324
A_44_P370645	Sec61b_predicted	XM_216400		0.13	1.345	0.032539
A_44_P884427	TC554663	TC554663		0.13	1.345	0.248138
A_44_P668354	Gpsm1	BC086535	246254	0.13	1.345	0.014611
A_44_P608624	CB718612	CB718612		0.13	1.345	0.121685
A_43_P17304	Srrm2_predicted	XM_220207	302969	0.13	1.345	0.014608
A_44_P407932	XM_228868	XM_228868		0.13	1.345	0.043418
A_44_P867386	ENSRNOT00000051722	ENSRNOT00000051722		0.13	1.345	0.060495
A_43_P19180	Rufy1	XM_340794	360521	0.13	1.345	0.026216
A_44_P272186	RGD1564541_predicted	XM_233309		0.13	1.345	0.014559
A_44_P149608	LOC683538	XM_001066436		0.13	1.345	0.089093
A_44_P305073	Atp6v1a1_predicted	XM_001060277		0.13	1.344	0.046195
A_43_P12193	Fbxl20	NM_022272	64039	0.13	1.344	0.134557
A_44_P512295	LOC503000	XM_001075213		0.13	1.344	0.028294
A_44_P604267	Chd8	XM_573762	65027	0.13	1.344	0.021996
A_44_P401074	Wdsof1_predicted	XM_001061920		0.13	1.344	0.067883
A_44_P606799	LOC679950	XM_001055051		0.13	1.344	0.206167
A_44_P347348	RGD1310430_predicted	XM_343234	362905	0.13	1.344	0.15009
A_44_P356876	RGD1304790	NM_001039002	288031	0.13	1.344	0.058917
A_44_P279041	Stard7_predicted	XM_238280		0.13	1.344	0.116091
A_44_P640481	TC567393	TC567393		0.13	1.343	0.044393
A_44_P195468	Yt521	NM_133423	170956	0.13	1.343	0.067002
A_44_P457386	XM_343090	XM_343090		0.13	1.343	0.281774
A_44_P868315	TC517731	TC517731		0.13	1.343	0.017593
A_44_P323404	Cntf	NM_013166	25707	0.13	1.343	0.209049
A_44_P226999	Oas1i	NM_001009680	304507	0.13	1.343	0.276255
A_44_P259617	CB545107	CB545107	681024	0.13	1.343	0.056438
A_44_P348607	Gtf3c2	NM_001025120	313914	0.13	1.343	0.108089
A_43_P17239	RGD1562920_predicted	XM_214790	292486	0.13	1.343	0.076247
A_44_P151920	RGD1310799_predicted	XM_219439		0.13	1.343	0.064888
A_44_P1050015	Vcpip1	NM_176857	286761	0.13	1.343	0.182411
A_44_P348582	AA945624	AA945624	291084	0.13	1.343	0.088044
A_44_P1008329	Fgfr1op2	NM_201421	362463	0.13	1.343	0.01596
A_44_P116176	Armet_predicted	XM_236614		0.13	1.343	0.105018
A_44_P941990	BF548061	BF548061		0.13	1.342	0.050142
A_44_P321220	XM_227168	XM_227168		0.13	1.342	0.028741
A_44_P452622	LOC291967	NM_001039099	291967	0.13	1.342	0.093275
A_44_P224884	RGD1565258_predicted	XM_344408	364385	0.13	1.342	0.025239
A_44_P547801	Gstt2	NM_012796	29487	0.13	1.342	0.103779
A_44_P142201	Ide	NM_013159	25700	0.13	1.342	0.025635
A_44_P973186	AW142467	AW142467	85385	0.13	1.342	0.069609
A_44_P412641	LOC291964	XM_214682	291964	0.13	1.342	0.011982
A_44_P558652	RGD1565983_predicted	XM_001061562		0.13	1.342	0.009494
A_43_P15389	Frk	NM_024368	79209	0.13	1.342	0.107268
A_44_P118908	NP516896	NP516896		0.13	1.342	0.048902
A_44_P110110	Cst3	NM_012837	25307	0.13	1.342	0.043329
A_44_P837036	A_44_P837036	A_44_P837036		0.13	1.342	0.010712
A_42_P833212	Wbscr1	NM_001006957	288599	0.13	1.342	0.018188
A_44_P1039994	Irf7	NM_001033691	293624	0.13	1.341	0.495505
A_44_P489120	Tram1	NM_001007701	312903	0.13	1.341	0.056014
A_42_P662710	Dedd	NM_031800	83631	0.13	1.341	0.025115
A_44_P551054	Rassf3_predicted	XM_343213		0.13	1.341	0.059051
A_44_P316839	Aarsl_predicted	XM_236942		0.13	1.341	0.019223
A_44_P445756	Ocrl_mapped	XM_229106		0.13	1.341	0.201288
A_44_P131463	AA955255	AA955255		0.13	1.341	0.376841
A_44_P498522	Kctd9_predicted	XM_344426		0.13	1.341	0.062004
A_44_P1048296	Osbpl1a	NM_172023	259221	0.13	1.341	0.052122
A_43_P16537	LOC288165	XM_213636		0.13	1.341	0.018151
A_44_P810179	LOC307798	NM_001037193	307798	0.13	1.341	0.02332
A_44_P390746	AW143482	AW143482		0.13	1.341	0.382998

A_44_P432937	AW914950	AW914950	296655	0.13	1.340	0.144092
A_44_P335898	CB548034	CB548034	408223	0.13	1.340	0.037206
A_43_P12304	Sgta	NM_022703	64667	0.13	1.340	0.017039
A_44_P593331	TC544665	TC544665		0.13	1.340	0.287676
A_44_P222487	Mrps27_predicted	XM_342180		0.13	1.340	0.034655
A_44_P250460	XM_218424	XM_218424		0.13	1.340	0.025019
A_44_P320518	RGD1562702_predicted	XM_575227		0.13	1.340	0.023142
A_44_P652670	Eif2s3x	XM_001056649		0.13	1.340	0.059208
A_44_P184874	RGD1563636_predicted	XM_218063		0.13	1.340	0.041553
A_42_P609446	Nf1	NM_012609	24592	0.13	1.340	0.028817
A_44_P108772	Ube4b_predicted	XM_233679	298652	0.13	1.340	0.147353
A_44_P203838	RGD1564088_predicted	XM_225531		0.13	1.339	0.025388
A_44_P767212	AW917822	AW917822		0.13	1.339	0.500716
A_44_P511324	Rps15a	NM_053982	117053	0.13	1.339	0.01688
A_44_P213237	LOC691155	XM_001077030	691155	0.13	1.339	0.053534
A_42_P712718	RGD1303272	NM_001004280	362134	0.13	1.339	0.134126
A_44_P369144	AW916097	AW916097	305956	0.13	1.339	0.016306
A_44_P543548	Sfrs10	NM_057119	117259	0.13	1.339	0.099523
A_44_P346661	Tpm3	NM_173111	117557	0.13	1.339	0.069784
A_44_P493005	Foxq1	NM_022858	64826	0.13	1.339	0.239151
A_44_P550697	Ankrd32_predicted	XM_001059377		0.13	1.339	0.108194
A_44_P347420	Usp43_predicted	XM_220582		0.13	1.339	0.03117
A_44_P455774	AI009276	AI009276		0.13	1.339	0.223691
A_43_P11388	CB545173	CB545173		0.13	1.339	0.060068
A_44_P176494	Cdc73	NM_001024769	304832	0.13	1.339	0.041106
A_44_P634618	AW143298	AW143298		0.13	1.339	0.056152
A_44_P307013	LOC682690	XM_001062637		0.13	1.339	0.042245
A_44_P1038818	BG662888	BG662888		0.13	1.338	0.029384
A_44_P184497	Cyb5b	NM_030586	80773	0.13	1.338	0.157216
A_44_P1034910	Rtn2	NM_201562	308410	0.13	1.338	0.035056
A_44_P518161	LOC291138	XR_009575	291138	0.13	1.338	0.078738
A_44_P729193	LOC500700	XM_001063975		0.13	1.338	0.054017
A_44_P534626	XM_342978	XM_342978		0.13	1.338	0.081257
A_44_P1035926	RGD1560340_predicted	XM_222534	304719	0.13	1.338	0.135215
A_44_P460973	AW914768	AW914768	25622	0.13	1.338	0.020939
A_44_P543744	Vps39	XM_575216	362199	0.13	1.338	0.109369
A_44_P347219	A_44_P347219	A_44_P347219		0.13	1.337	0.047576
A_44_P231811	XM_224540	XM_224540		0.13	1.337	0.030023
A_44_P356605	Las1l_predicted	XM_216095	296865	0.13	1.337	0.024876
A_44_P481317	Cdk7	XM_215467	171150	0.13	1.337	0.324284
A_42_P630566	RGD1561203_predicted	XM_573313	498109	0.13	1.337	0.022351
A_44_P152373	A_44_P152373	A_44_P152373		0.13	1.337	0.027118
A_44_P286008	LOC684849	XM_001072181		0.13	1.337	0.105718
A_43_P13342	Gstp2	NM_138974	29438	0.13	1.337	0.183102
A_44_P331083	RGD1306591	XM_343289	362959	0.13	1.337	0.031372
A_44_P245968	XM_342444	XM_342444		0.13	1.337	0.14014
A_44_P981062	A_44_P981062	A_44_P981062		0.13	1.337	0.011618
A_44_P504120	Crocc_predicted	XM_233603		0.13	1.337	0.013366
A_44_P276596	Ctf2_predicted	XM_219355	293515	0.13	1.337	0.134356
A_43_P21753	Sox30_predicted	XM_220328	57031	0.13	1.337	0.111012
A_44_P991515	RGD1559939_predicted	XM_575360		0.13	1.337	0.157548
A_44_P231893	RGD1309709_predicted	XM_342420	362118	0.13	1.337	0.292272
A_43_P19332	Nfkbiz_predicted	XM_221537		0.13	1.337	0.277766
A_42_P510494	RGD1306192_predicted	XM_341722	361443	0.13	1.336	0.012005
A_42_P511015	Nup133_predicted	XM_001053507		0.13	1.336	0.051266
A_44_P869444	TC540890	TC540890		0.13	1.336	0.42034
A_43_P21176	RGD1565253_predicted	XM_343404	363073	0.13	1.336	0.032117
A_44_P401511	Exoc6	NM_019277	50556	0.13	1.336	0.017217
A_44_P232164	Mrpl42_predicted	XM_216882		0.13	1.336	0.046512
A_44_P418694	Rabggta	NM_031654	58983	0.13	1.336	0.016423
A_44_P597013	A_44_P597013	A_44_P597013		0.13	1.336	0.388393
A_44_P332683	Wdr7	NM_023975	66031	0.13	1.336	0.150597
A_44_P140314	Cdc25c_predicted	XM_226071		0.13	1.336	0.083161

A_44_P845503	LOC683455	XM_001066011		0.13	1.336	0.314412
A_44_P374341	Strn3	NM_001029897	114520	0.13	1.336	0.045835
A_44_P458319	DV718436	DV718436		0.13	1.336	0.128476
A_44_P390102	LOC679668	XM_001053335		0.13	1.336	0.014677
A_44_P248541	Sypl	NM_001014263	366595	0.13	1.335	0.078329
A_43_P17160	Ppil2	NM_001017383	360746	0.13	1.335	0.034931
A_44_P382291	Cbfa2t2_predicted	XM_215882	296293	0.13	1.335	0.100113
A_44_P201537	RGD1560612_predicted	XM_342467	362166	0.13	1.335	0.117257
A_44_P141359	AA874812	AA874812		0.13	1.335	0.190078
A_43_P19424	Aebp2_predicted	XM_216295		0.13	1.335	0.019344
A_44_P253422	LOC300472	XM_217099	300472	0.13	1.335	0.122876
A_44_P326218	Lemd2	NM_001039032	361807	0.13	1.335	0.081744
A_44_P860400	RGD1566064_predicted	XM_341141	360865	0.13	1.335	0.018904
A_44_P619184	AW914780	AW914780		0.13	1.335	0.035023
A_44_P812352	TC567149	TC567149		0.13	1.335	0.039035
A_44_P263801	BM390456	BM390456	117056	0.13	1.335	0.021459
A_44_P450455	RGD1305255_predicted	XM_235191	299824	0.13	1.335	0.230873
A_44_P662747	CR462562	CR462562	500432	0.13	1.335	0.0691
A_44_P683415	LOC683419	XM_001064635		0.13	1.335	0.032371
A_44_P361554	RGD1308154_predicted	XM_344027	363799	0.13	1.335	0.112616
A_43_P18256	XM_213245	XM_213245		0.13	1.335	0.15788
A_42_P813325	Atrx	XM_217570		0.13	1.334	0.034005
A_44_P445972	Thrap1_predicted	XM_220813		0.13	1.334	0.11851
A_43_P11429	RGD1308470	NM_001014198	362778	0.13	1.334	0.072087
A_43_P16755	Alg5	NM_001025407	295051	0.13	1.334	0.052427
A_44_P806646	RGD1561651_predicted	XM_343738	363412	0.13	1.334	0.033743
A_44_P131897	Pkcbpb15	NM_021764	60383	0.13	1.334	0.055162
A_44_P245974	Fchsd1_predicted	XM_225999		0.13	1.334	0.062304
A_42_P654862	RGD1306107_predicted	XM_227412		0.13	1.334	0.039397
A_44_P439252	AA800206	AA800206	291245	0.13	1.334	0.027023
A_44_P296523	BE098764	BE098764	300078	0.13	1.334	0.145411
A_44_P445166	Inppl1	NM_022944	65038	0.12	1.333	0.102659
A_44_P322451	AA686461	AA686461	64640	0.12	1.333	0.070409
A_44_P283878	Nat5_predicted	XM_342534		0.12	1.333	0.036754
A_44_P922379	LOC691026	XM_001076577	691026	0.12	1.333	0.042412
A_44_P591001	A_44_P591001	A_44_P591001		0.12	1.333	0.019275
A_44_P417916	Myd88	NM_198130	301059	0.12	1.333	0.055526
A_44_P135198	Tbkbp1	NM_172021	266764	0.12	1.333	0.199679
A_44_P252091	Rheb	NM_013216	26954	0.12	1.333	0.042242
A_44_P291175	Kntc2_predicted	XM_217489	301701	0.12	1.333	0.09536
A_44_P545462	LOC257650	NM_147142	257650	0.12	1.333	0.370583
A_44_P776937	RGD1560191_predicted	XM_001070702		0.12	1.333	0.110493
A_44_P242525	AA899063	AA899063	295229	0.12	1.333	0.134449
A_42_P739344	BF559919	BF559919		0.12	1.333	0.042561
A_44_P504061	A_44_P504061	A_44_P504061		0.12	1.333	0.032335
A_44_P363874	LOC690898	XM_001074803	690898	0.12	1.333	0.091098
A_44_P390045	BQ780513	BQ780513	293023	0.12	1.333	0.097193
A_43_P13066	Echs1	NM_078623	140547	0.12	1.333	0.06471
A_44_P220301	Gpr114_predicted	XM_240979		0.12	1.333	0.10956
A_44_P880618	LOC501039	XM_001065705		0.12	1.333	0.216456
A_44_P483163	RGD1565082_predicted	XM_234465	314381	0.12	1.333	0.166938
A_44_P618757	DY574819	DY574819		0.12	1.333	0.245917
A_42_P695036	Alkbh4_predicted	XM_222071		0.12	1.333	0.031719
A_44_P1056875	Phb2	NM_001013035	114766	0.12	1.332	0.037386
A_43_P20646	Eva1_predicted	XM_236197		0.12	1.332	0.125955
A_44_P163242	Tacstd1	NM_138541	171577	0.12	1.332	0.232759
A_44_P286104	RGD1308535_predicted	XM_227419		0.12	1.332	0.019669
A_44_P367205	RGD1565940_predicted	XM_573456		0.12	1.332	0.118544
A_44_P360782	AF037200	AF037200		0.12	1.332	0.028564
A_43_P11330	Pdia6	XM_576132		0.12	1.332	0.082184
A_44_P221796	AW140985	AW140985		0.12	1.332	0.051232
A_44_P1002141	TSEN34	NM_001006968	292534	0.12	1.332	0.049987
A_44_P434386	Ash1l_predicted	XM_227409		0.12	1.332	0.085193

A_44_P133092	LOC289482	NM_001024742	289482	0.12	1.332	0.036162
A_42_P729743	Fxyd6	NM_022005	63847	0.12	1.332	0.106928
A_43_P11971	Cd47	NM_019195	29364	0.12	1.332	0.060323
A_44_P760124	RGD1562613_predicted	XM_573460		0.12	1.332	0.035587
A_44_P424734	AW918981	AW918981		0.12	1.332	0.078398
A_44_P419858	XM_345835	XM_345835		0.12	1.331	0.021121
A_42_P546266	Ercc1_predicted	XM_214833		0.12	1.331	0.017328
A_44_P486607	Snx5_predicted	XM_215872		0.12	1.331	0.159459
A_43_P10538	LOC679129	XM_001054818		0.12	1.331	0.081675
A_44_P654924	TC560208	TC560208		0.12	1.331	0.171959
A_42_P582801	DERP6	NM_001001718	287446	0.12	1.331	0.022114
A_43_P17088	Agpat5_predicted	XM_224993	306582	0.12	1.331	0.149858
A_43_P22046	LOC690810	XM_001075711		0.12	1.331	0.011588
A_42_P497323	Ppp1r10	NM_022951	65045	0.12	1.331	0.050535
A_44_P888790	TC546033	TC546033		0.12	1.331	0.043485
A_44_P995783	AI411753	AI411753		0.12	1.330	0.037685
A_44_P154211	ENSRNOT00000005197	ENSRNOT00000005197		0.12	1.330	0.034483
A_44_P512617	RGD1564804_predicted	XM_233456		0.12	1.330	0.068998
A_44_P807849	Rap1a	NM_001005765	295347	0.12	1.330	0.118593
A_44_P593422	TC528213	TC528213		0.12	1.330	0.022491
A_44_P947165	TC533154	TC533154		0.12	1.330	0.34222
A_42_P667600	Mmp23	NM_053606	94339	0.12	1.330	0.23251
A_44_P1024065	Hdac5	XM_001081495		0.12	1.330	0.093107
A_44_P276829	Tsga2	NM_001012176	361818	0.12	1.330	0.117592
A_44_P944011	RGD1562316_predicted	XM_574685		0.12	1.330	0.204358
A_44_P914899	TC522206	TC522206		0.12	1.330	0.090723
A_44_P861558	Ncor2_predicted	XM_341072		0.12	1.330	0.029889
A_44_P522270	Prpf38a_predicted	XM_001060413		0.12	1.330	0.072477
A_44_P241277	Traf2_predicted	XM_231032		0.12	1.330	0.050423
A_44_P391008	Yes1	NM_033298	24884	0.12	1.330	0.084056
A_44_P699615	TC521357	TC521357		0.12	1.330	0.271305
A_44_P760606	LOC366774	XR_007927	366774	0.12	1.330	0.130631
A_44_P170396	Timm44	NM_017267	29635	0.12	1.330	0.08686
A_44_P1000115	RGD735065	NM_199379	294311	0.12	1.329	0.093795
A_44_P636597	LOC498391	XR_007986	498391	0.12	1.329	0.034021
A_44_P483000	RGD1305633_predicted	XM_228076	309673	0.12	1.329	0.072474
A_44_P637134	RGD1561575_predicted	XR_007584	367720	0.12	1.329	0.122093
A_44_P633224	BG381596	BG381596		0.12	1.329	0.019972
A_44_P106571	BP503923	BP503923		0.12	1.329	0.177152
A_44_P501049	AY310141	AY310141		0.12	1.329	0.186065
A_44_P438922	Klhl6_predicted	XM_221290		0.12	1.329	0.159837
A_44_P386965	RGD1564620_predicted	XM_345302	365962	0.12	1.329	0.247488
A_44_P415212	A_44_P415212	A_44_P415212		0.12	1.329	0.051425
A_44_P188613	Nnt_mapped	NM_001013157	310378	0.12	1.329	0.211228
A_44_P416695	Ka15	NM_001004022	287700	0.12	1.329	0.027538
A_44_P420036	AW917512	AW917512		0.12	1.329	0.035362
A_44_P301805	Ryk	NM_080402	140585	0.12	1.329	0.093284
A_44_P126572	Znf251_predicted	XM_345851	366954	0.12	1.329	0.042845
A_44_P1006068	RGD1307749_predicted	XM_238473		0.12	1.329	0.052561
A_43_P10081	XM_227000	XM_227000		0.12	1.329	0.067395
A_43_P13421	Aip	NM_172327	282827	0.12	1.328	0.015739
A_44_P160763	Pank2_predicted	XM_215826	296167	0.12	1.328	0.020435
A_44_P727508	BF281819	BF281819		0.12	1.328	0.19901
A_44_P1011224	Atp8b2	XM_001062605	361984	0.12	1.328	0.035461
A_44_P149190	Ftl1	K01930	29292	0.12	1.328	0.200509
A_44_P930917	Arhgef5	XM_342676	140898	0.12	1.328	0.102353
A_44_P362723	Psmd7_predicted	XM_226439		0.12	1.328	0.072526
A_44_P321032	RGD1564052_predicted	XM_344095		0.12	1.328	0.023877
A_44_P258497	Hrmt1l1	NM_001025144	499420	0.12	1.328	0.040583
A_44_P379991	RGD1564400_predicted	XM_212955	295660	0.12	1.328	0.053644
A_42_P678974	LOC690300	XM_001074020		0.12	1.328	0.139575
A_43_P23290	Sh3pxd2a_predicted	XM_219971		0.12	1.328	0.067531
A_44_P464419	XM_343249	XM_343249		0.12	1.328	0.277055

A_44_P222865	XM_235305	XM_235305		0.12	1.328	0.069012
A_44_P166037	LOC679295	XM_001055442		0.12	1.327	0.070207
A_42_P539352	Adrm1	NM_031708	65138	0.12	1.327	0.033683
A_44_P135829	Srrm1_predicted	XM_001069368		0.12	1.327	0.038891
A_42_P473389	Bmyc_mapped	NM_001013163	311807	0.12	1.327	0.030161
A_44_P899141	Ibtk_predicted	XM_001062352		0.12	1.327	0.145904
A_44_P521532	DV726884	DV726884	500364	0.12	1.327	0.30164
A_44_P538839	Rasgrp2_predicted	XM_342003		0.12	1.327	0.107243
A_44_P257646	LOC681263	XM_001060987		0.12	1.327	0.11663
A_44_P480240	Rnf4	NM_019182	29274	0.12	1.327	0.039787
A_42_P698051	Slc39a3	NM_001008356	314637	0.12	1.326	0.059175
A_44_P808972	TC560106	TC560106		0.12	1.326	0.166618
A_42_P677296	Phf22	NM_001007640	295448	0.12	1.326	0.052972
A_44_P606742	Osbp_predicted	XM_001075837		0.12	1.326	0.077207
A_44_P1035720	LOC683353	XM_001064234		0.12	1.326	0.180533
A_44_P222021	Tubb2c	NM_199094	296554	0.12	1.326	0.024583
A_42_P673095	Trappc3	NM_001008376	362599	0.12	1.326	0.032385
A_43_P10235	Armc8_predicted	XM_236599	315949	0.12	1.326	0.047684
A_44_P468123	Csnk1g2	NM_023102	65278	0.12	1.326	0.035752
A_44_P344397	Bles03	NM_001024233	266609	0.12	1.325	0.088418
A_44_P614514	AABR03073784	AABR03073784		0.12	1.325	0.017521
A_44_P998384	Slc34a3	NM_139338	246234	0.12	1.325	0.273951
A_42_P658220	LOC690516	XM_001074660		0.12	1.325	0.058201
A_44_P300919	Lcmt2	NM_001011956	296098	0.12	1.325	0.046354
A_44_P761160	TC537691	TC537691		0.12	1.325	0.041895
A_44_P508711	XM_215628	XM_215628		0.12	1.325	0.070265
A_42_P696561	AW918243	AW918243		0.12	1.325	0.024723
A_44_P201385	Psmc6	XM_214147	289990	0.12	1.325	0.080699
A_42_P788810	AW918709	AW918709		0.12	1.325	0.061889
A_44_P506374	Nyw1	XM_001069366		0.12	1.325	0.036181
A_44_P142719	RGD1561651_predicted	XM_343738	363412	0.12	1.324	0.05683
A_44_P1004781	LOC680229	XM_001056227	680229	0.12	1.324	0.084131
A_44_P428075	RGD1305302	NM_001014000	306455	0.12	1.324	0.021909
A_43_P20948	Dd5	XM_576252	117060	0.12	1.324	0.017559
A_42_P796502	Hsd17b7	NM_017235	29540	0.12	1.324	0.176402
A_43_P19892	Aph1a	NM_001014255	365872	0.12	1.324	0.043022
A_44_P344762	Fubp3	NM_001039337	362106	0.12	1.324	0.072086
A_44_P159355	RGD1307814_predicted	XM_342878	362559	0.12	1.324	0.092739
A_44_P793224	RGD1564560_predicted	XM_576400		0.12	1.324	0.027372
A_44_P238642	LOC689030	XM_001069243	689030	0.12	1.324	0.103764
A_44_P1041179	Ep400	XM_222251		0.12	1.324	0.046747
A_44_P338962	RGD1563991_predicted	XM_346307		0.12	1.324	0.039309
A_44_P882219	Mll_mapped	XM_001067729		0.12	1.324	0.092792
A_44_P304711	A_44_P304711	A_44_P304711		0.12	1.323	0.068998
A_44_P292479	Dncic2	NM_053880	116659	0.12	1.323	0.132478
A_42_P649672	Sv2a	NM_057210	117559	0.12	1.323	0.126045
A_44_P196723	XM_214110	XM_214110		0.12	1.323	0.013126
A_44_P107949	Dnajc11_predicted	XM_342983		0.12	1.323	0.022912
A_44_P729238	Dd5	XM_576252	117060	0.12	1.323	0.045689
A_44_P175543	Cox15	NM_001033699	309391	0.12	1.323	0.039577
A_44_P314431	RGD1306729_predicted	XM_220200		0.12	1.323	0.166175
A_44_P329015	LOC691543	XM_001078752		0.12	1.323	0.247487
A_44_P461545	XM_213747	XM_213747		0.12	1.323	0.030912
A_44_P347442	Cryzl1	NM_001013044	288256	0.12	1.323	0.045869
A_44_P635869	DY311261	DY311261	309156	0.12	1.323	0.289669
A_44_P496864	Sema4a	NM_001012078	310630	0.12	1.323	0.129825
A_44_P116172	Armet_predicted	XM_236614		0.12	1.323	0.040509
A_44_P174721	RGD1307882_predicted	XM_236501	315903	0.12	1.323	0.023697
A_42_P540950	Ier2	NM_001009541	494344	0.12	1.323	0.036406
A_44_P545945	RGD1310323	NM_001008348	309172	0.12	1.323	0.077663
A_44_P461197	Sod1	NM_017050	24786	0.12	1.322	0.019272
A_44_P452398	RGD1306105_predicted	XM_341190		0.12	1.322	0.191511
A_44_P749897	TC547525	TC547525		0.12	1.322	0.048494

A_44_P160872	Rap2a	NM_053741	114560	0.12	1.322	0.03342
A_44_P370229	RGD1311980	XM_573367	304334	0.12	1.322	0.282151
A_42_P624251	LOC361399	NM_001033068	361399	0.12	1.322	0.126028
A_44_P384112	Ppp3cc	NM_134367	171378	0.12	1.322	0.209456
A_44_P698373	ENSRNOT00000043609	ENSRNOT00000043609		0.12	1.322	0.020084
A_43_P13536	Copz1_predicted	XM_235705		0.12	1.322	0.069982
A_44_P463222	BI273981	BI273981		0.12	1.322	0.08147
A_44_P1036480	LOC503338	XM_001069951		0.12	1.321	0.059685
A_44_P991484	CA510796	CA510796	287398	0.12	1.321	0.12235
A_44_P871882	TC525010	TC525010		0.12	1.321	0.029079
A_42_P750634	XM_341921	XM_341921		0.12	1.321	0.146945
A_44_P975113	RGD1311648	NM_001014075	313949	0.12	1.321	0.016772
A_44_P788707	AW920082	AW920082		0.12	1.321	0.124446
A_44_P882206	AW917544	AW917544		0.12	1.321	0.029343
A_42_P480723	Cd8b	NM_031539	24931	0.12	1.321	0.057806
A_44_P822943	TC535666	TC535666		0.12	1.321	0.056857
A_44_P147980	RGD1561626_predicted	XM_001056443		0.12	1.321	0.038379
A_44_P733362	Rnf11_predicted	XM_237327		0.12	1.321	0.020729
A_44_P353827	Arpp19	NM_031660	60336	0.12	1.321	0.023068
A_44_P423084	Mllt10	NM_001012162	361285	0.12	1.320	0.147818
A_44_P900209	Abhd2_predicted	XM_214979		0.12	1.320	0.085865
A_44_P201028	Nfil3	NM_053727	114519	0.12	1.320	0.034177
A_44_P348922	Cebpb	NM_024125	24253	0.12	1.320	0.094214
A_44_P303044	Supt6h	XM_239258	303281	0.12	1.320	0.029002
A_44_P1056271	Phr1_predicted	XM_214245		0.12	1.320	0.080512
A_42_P686234	Muc4	XM_221384	303887	0.12	1.320	0.238434
A_43_P22070	Zbtb24_predicted	XM_345114		0.12	1.320	0.072604
A_44_P168102	Ppp5c	NM_031729	65179	0.12	1.320	0.062157
A_44_P260055	Gyg1	NM_031043	81675	0.12	1.320	0.058432
A_44_P606112	LOC500084	XM_001066092		0.12	1.320	0.139083
A_42_P702005	LOC678925	XM_001054722		0.12	1.320	0.034624
A_43_P11911	Rab3ip	NM_017313	29885	0.12	1.320	0.081698
A_44_P899549	RGD1566062_predicted	XM_578126	502632	0.12	1.320	0.072294
A_44_P262113	Napa	NM_080585	140673	0.12	1.320	0.116679
A_44_P840778	Dnajb6	NM_001013209	362293	0.12	1.319	0.025095
A_44_P715994	TC525422	TC525422		0.12	1.319	0.125006
A_43_P18787	Ncoa6	XM_342552	116464	0.12	1.319	0.066583
A_44_P532020	RGD1306820_predicted	XM_219414		0.12	1.319	0.065909
A_44_P168348	A_44_P168348	A_44_P168348		0.12	1.319	0.245611
A_42_P714245	Trappc4	NM_001003708	367073	0.12	1.319	0.100858
A_44_P238314	Dlgh4	NM_019621	29495	0.12	1.319	0.392341
A_44_P215131	Mk1	NM_134399	171436	0.12	1.319	0.043717
A_44_P1034950	Adora2a	NM_053294	25369	0.12	1.319	0.626537
A_42_P495428	RGD1307736	NM_001013983	304543	0.12	1.318	0.039574
A_44_P1060064	RGD1310810	NM_001024883	306549	0.12	1.318	0.054874
A_44_P350823	Cherp_predicted	XM_214307		0.12	1.318	0.222287
A_44_P778617	TC535512	TC535512		0.12	1.318	0.067002
A_44_P271368	AW917142	AW917142		0.12	1.318	0.054952
A_44_P625285	TC518879	TC518879		0.12	1.318	0.152256
A_44_P247667	Ube2g1	NM_022690	64631	0.12	1.318	0.055537
A_44_P267762	Dnpep	NM_001024879	301529	0.12	1.318	0.050415
A_44_P267724	Kif21a_predicted	XM_217022		0.12	1.318	0.154998
A_44_P512255	Rgs4	NM_017214	29480	0.12	1.317	0.455751
A_44_P240414	Tex261	NM_001017537	297392	0.12	1.317	0.01639
A_42_P604121	Tfg	NM_001012144	360709	0.12	1.317	0.100113
A_44_P442060	Smurf2_predicted	XM_001081579		0.12	1.317	0.114432
A_44_P110429	RGD1307493_predicted	XM_001080742		0.12	1.317	0.068009
A_44_P1047628	Pars2	NM_001014064	313429	0.12	1.317	0.055184
A_44_P785474	TC525405	TC525405		0.12	1.317	0.096852
A_44_P182816	RGD1310061	NM_001024972	290912	0.12	1.317	0.034625
A_44_P400527	Zfp347	NM_133390	170902	0.12	1.317	0.137707
A_43_P17610	Pomt2	NM_001037097	366697	0.12	1.317	0.202725
A_44_P417083	Atad1	AY325215	309532	0.12	1.317	0.052361

A_44_P314152	RGD1561317_predicted	XM_345433		0.12	1.316	0.015627
A_44_P386693	Gjb4	NM_053984	117055	0.12	1.316	0.146089
A_42_P654739	Rnf6_predicted	XM_221888		0.12	1.316	0.080784
A_44_P902209	Add1	NM_016990	24170	0.12	1.316	0.071103
A_44_P147582	Rab5b_predicted	XM_213824		0.12	1.316	0.043312
A_44_P634358	Adnp	NM_022681	64622	0.12	1.316	0.065841
A_44_P408008	Trappc3	NM_001008376	362599	0.12	1.316	0.065636
A_44_P486760	Ubxd2	NM_001012025	304766	0.12	1.316	0.109368
A_44_P715035	RGD1359108	NM_001007702	313155	0.12	1.316	0.095789
A_43_P13057	Baiap2	NM_057196	117542	0.12	1.316	0.071875
A_42_P701582	Gstt1	NM_053293	25260	0.12	1.316	0.032931
A_44_P345102	Arpc5l	NM_001037767	296710	0.12	1.316	0.0506
A_43_P12245	Rhob	NM_022542	64373	0.12	1.316	0.181743
A_42_P619308	Mrps18a	NM_198756	301249	0.12	1.316	0.024267
A_44_P328256	Nalp12_predicted	XM_218181	292541	0.12	1.315	0.517144
A_44_P123954	XM_226181	XM_226181		0.12	1.315	0.056813
A_44_P410954	Slc43a1_predicted	XM_230050		0.12	1.315	0.077582
A_44_P671204	TC534115	TC534115		0.12	1.315	0.030255
A_44_P867638	A_44_P867638	A_44_P867638		0.12	1.315	0.020001
A_43_P11613	Anp32a	NM_012903	25379	0.12	1.315	0.043322
A_44_P957147	BF554238	BF554238		0.12	1.315	0.056579
A_44_P669179	TC523266	TC523266		0.12	1.315	0.197683
A_44_P947026	TC528047	TC528047		0.12	1.315	0.174151
A_43_P15668	Tlk2_predicted	XM_221023	303592	0.12	1.315	0.038142
A_44_P349652	Ralbp1	NM_032067	84014	0.12	1.315	0.037396
A_44_P262934	Dnajc8	NM_001013168	313035	0.12	1.314	0.113899
A_43_P14292	Mrps23_predicted	XM_340874		0.12	1.314	0.092615
A_44_P548327	LOC681062	XM_001060131	681062	0.12	1.314	0.052154
A_44_P128229	Baiap2	NM_057196	117542	0.12	1.314	0.091123
A_44_P147087	Chd1_predicted	XM_238731		0.12	1.314	0.050994
A_43_P16775	Impact	NM_001012235	497198	0.12	1.314	0.122783
A_44_P651446	AI236521	AI236521	691849	0.12	1.313	0.085534
A_44_P620356	AW917690	AW917690	309338	0.12	1.313	0.035934
A_44_P1056100	Ttk_predicted	XM_236477		0.12	1.313	0.136912
A_44_P435751	Stau1	NM_053436	84496	0.12	1.313	0.037171
A_44_P523853	Pygm	XM_342002		0.12	1.313	0.075067
A_44_P443235	Ell3	NM_001011957	296102	0.12	1.313	0.043004
A_44_P327515	LOC501515	XM_001060307	501515	0.12	1.313	0.139146
A_44_P956951	RGD1310685_predicted	XM_001069036		0.12	1.313	0.146504
A_43_P18920	Ercc8_predicted	XM_226789		0.12	1.313	0.024614
A_44_P432259	AF486617	AF486617		0.12	1.312	0.043073
A_43_P14699	LOC686259	XM_001073157		0.12	1.312	0.051359
A_44_P398492	Mib1_predicted	XM_226175		0.12	1.312	0.040654
A_44_P1002996	LOC293494	NM_001013900	293494	0.12	1.312	0.032135
A_44_P816824	A_44_P816824	A_44_P816824		0.12	1.312	0.191198
A_44_P408033	A_44_P408033	A_44_P408033		0.12	1.312	0.044593
A_44_P260297	LOC679469	XM_001055601		0.12	1.312	0.156735
A_44_P238971	Ranbp10_predicted	XM_341676	361396	0.12	1.312	0.039273
A_44_P303762	Nkiras2_predicted	XM_213462		0.12	1.312	0.082924
A_44_P468380	Kcnip2	NM_020094	56817	0.12	1.312	0.217676
A_44_P263957	Oxr1	XM_576258	117520	0.12	1.312	0.101541
A_44_P175681	Gramd3	NM_001014011	307288	0.12	1.312	0.096309
A_43_P11497	Ptpn1	NM_012637	24697	0.12	1.312	0.037035
A_44_P687385	TC561908	TC561908		0.12	1.312	0.095882
A_44_P421363	Hmbs	NM_013168	25709	0.12	1.311	0.047343
A_44_P386579	LOC307582	XM_226159		0.12	1.311	0.218943
A_44_P963992	TC563722	TC563722		0.12	1.311	0.180205
A_44_P424316	RGD1306286_predicted	XM_001056478		0.12	1.311	0.027155
A_44_P236656	Gla_mapped	XM_343817		0.12	1.311	0.3187
A_42_P592059	Znhit1_predicted	XM_001076915		0.12	1.311	0.087786
A_44_P872777	Eif4g3_predicted	XM_216563		0.12	1.311	0.170969
A_43_P18756	Atp11a_predicted	XM_225014		0.12	1.310	0.090583
A_44_P188280	Adrb2	NM_012492	24176	0.12	1.310	0.081544

A_43_P17872	CB545618	CB545618	361384	0.12	1.310	0.222712
A_44_P255294	Hbs1l	NM_001011934	293408	0.12	1.310	0.028909
A_44_P874141	AI137494	AI137494	302980	0.12	1.310	0.049958
A_44_P810851	TC562476	TC562476		0.12	1.310	0.1598
A_44_P762950	TC544794	TC544794		0.12	1.310	0.122107
A_44_P1016829	Trib1	XM_343250		0.12	1.310	0.032503
A_44_P313975	XM_215252	XM_215252		0.12	1.310	0.184889
A_44_P295118	RGD1563315_predicted	XM_223544		0.12	1.310	0.214701
A_44_P422063	Gopc_predicted	XM_228173		0.12	1.310	0.022987
A_44_P770724	Dmxl1_predicted	XM_001054434		0.12	1.310	0.158807
A_44_P563985	TC533697	TC533697		0.12	1.310	0.441918
A_44_P108421	Fndc3a_predicted	XM_224350		0.12	1.310	0.153321
A_43_P20945	CB546262	CB546262		0.12	1.310	0.055323
A_42_P496725	Ssb	NM_031119	81783	0.12	1.310	0.050172
A_44_P414615	Daxx	NM_080891	140926	0.12	1.309	0.093943
A_44_P468412	Nup62	NM_023098	65274	0.12	1.309	0.033369
A_44_P243862	RGD1306941_predicted	XM_237162		0.12	1.309	0.065271
A_44_P165513	Cip98	NM_181088	313255	0.12	1.309	0.244883
A_43_P14683	Usp7	NM_001024790	360471	0.12	1.309	0.056971
A_44_P382747	Srebf2_predicted	NM_001033694	300095	0.12	1.309	0.181743
A_44_P222147	Hibadh	NM_022243	63938	0.12	1.309	0.10565
A_43_P16856	Reck_predicted	XM_233371		0.12	1.309	0.095073
A_44_P487594	XM_242065	XM_242065		0.12	1.309	0.03007
A_44_P356622	XM_232929	XM_232929		0.12	1.309	0.219047
A_44_P405768	RGD1565653_predicted	XM_345990		0.12	1.309	0.025799
A_43_P12834	Elf1	NM_053520	85424	0.12	1.309	0.038045
A_44_P323729	XM_345245	XM_345245		0.12	1.309	0.204895
A_44_P335219	Il21r	NM_001012469	308977	0.12	1.308	0.215137
A_44_P290570	Zfand2a	NM_001008363	360772	0.12	1.308	0.098173
A_44_P470149	LOC680426	XM_001057119	680426	0.12	1.308	0.057059
A_44_P165139	LOC501619	NM_001024367	501619	0.12	1.308	0.160063
A_44_P775809	A_44_P775809	A_44_P775809		0.12	1.308	0.054942
A_43_P13170	Tmlhe	NM_133387	170898	0.12	1.308	0.177185
A_44_P323372	Argbp2	AF396458	114901	0.12	1.308	0.074082
A_44_P105785	BG381460	BG381460		0.12	1.308	0.032972
A_44_P548559	LOC690315	XM_001070872		0.12	1.308	0.093656
A_42_P478706	LOC361990	NM_001014175	361990	0.12	1.307	0.047632
A_44_P185411	RGD1309461	XM_235402	315059	0.12	1.307	0.049459
A_44_P222603	Ss18	XM_341580	361295	0.12	1.307	0.065341
A_44_P743412	AI229438	AI229438		0.12	1.307	0.30832
A_44_P135610	Larp5_predicted	XM_225526		0.12	1.307	0.083321
A_44_P114638	Tsen2	NM_001014057	312649	0.12	1.307	0.057367
A_44_P499461	ENSRNOT00000005854	ENSRNOT00000005854		0.12	1.307	0.036279
A_44_P159244	RGD1561676_predicted	XM_001079196		0.12	1.307	0.03722
A_44_P433739	Wdr34	NM_001005542	296618	0.12	1.307	0.059629
A_43_P13290	Mawbp	NM_138530	171564	0.12	1.306	0.319985
A_42_P606890	Abi2	NM_173143	286928	0.12	1.306	0.14975
A_44_P488594	Tspan6	XM_217563	302313	0.12	1.306	0.142787
A_44_P137013	Fntb	NM_172034	64511	0.12	1.306	0.050843
A_44_P464196	Cd97	NM_001012164	361383	0.12	1.306	0.188446
A_44_P314081	Sptlc1_predicted	XM_341495		0.12	1.306	0.196933
A_44_P775257	A_44_P775257	A_44_P775257		0.12	1.306	0.104952
A_44_P934022	LOC500110	NM_001024327	500110	0.12	1.306	0.035352
A_44_P157167	Eif4b	NM_001008324	300253	0.12	1.306	0.023706
A_44_P999395	Lmbr1l	NM_001013950	300215	0.12	1.306	0.026353
A_44_P257528	LOC287010	AF053093		0.12	1.306	0.016376
A_44_P399203	Efna3	XM_001072657		0.12	1.306	0.174319
A_44_P810585	TC544925	TC544925		0.12	1.306	0.262376
A_44_P240461	AA800571	AA800571		0.12	1.305	0.033134
A_43_P13709	Fgd3_predicted	XM_341505		0.12	1.305	0.048991
A_42_P533792	Srpk2_predicted	XM_238336		0.12	1.305	0.038877
A_44_P491782	Stk19	NM_001013197	361800	0.12	1.305	0.018853
A_44_P109862	Ivns1abp_predicted	XM_213898		0.12	1.305	0.122384

A_44_P776728	TC521707	TC521707		0.12	1.305	0.262033
A_44_P203740	RGD1305077_predicted	XM_224451	306104	0.12	1.305	0.13968
A_44_P237739	Lsr	NM_032616	64355	0.12	1.305	0.07683
A_44_P1016988	RGD1310448_predicted	XM_230493	311347	0.12	1.305	0.097407
A_43_P20707	RGD1563290_predicted	XM_225891	307396	0.12	1.305	0.373263
A_44_P1030801	Gcs1	NM_031749	78947	0.12	1.305	0.066164
A_44_P388072	RGD1311909_predicted	XM_232287	312654	0.12	1.305	0.083859
A_44_P206675	AW921244	AW921244		0.12	1.304	0.118116
A_44_P299026	AI234700	AI234700	362294	0.12	1.304	0.259876
A_44_P463843	Adh1	NM_019286	24172	0.12	1.304	0.311042
A_44_P160331	AW142140	AW142140	360571	0.12	1.304	0.174162
A_44_P363204	Csnk2a1	NM_053824	116549	0.12	1.304	0.156943
A_44_P714565	LOC690367	XM_001072732		0.12	1.304	0.034369
A_42_P589570	RGD1549725	NM_001014105	317191	0.12	1.304	0.100113
A_42_P771373	Npy	NM_012614	24604	0.12	1.304	0.375736
A_44_P718340	TC546813	TC546813		0.12	1.304	0.027209
A_44_P282074	Prkdc_predicted	XM_341020		0.12	1.304	0.065769
A_44_P961464	TC525849	TC525849		0.12	1.304	0.105098
A_43_P11066	LOC685232	XM_001058095	360716	0.12	1.304	0.070945
A_44_P466500	Pcdhgc3	NM_053943	116782	0.12	1.304	0.080705
A_44_P245314	Cacnb3	NM_012828	25297	0.12	1.304	0.093695
A_44_P139851	Impad1	XM_575759	312952	0.12	1.304	0.07311
A_42_P815566	Crsp3	XM_220093	309565	0.12	1.303	0.108673
A_44_P852055	LOC498265	NM_001017493	498265	0.12	1.303	0.043934
A_44_P925577	AW915558	AW915558	304547	0.12	1.303	0.014362
A_44_P211061	Etv1_predicted	XM_343055		0.12	1.303	0.120697
A_42_P734384	Sptlc1_predicted	XM_341495		0.12	1.303	0.045906
A_44_P309140	Supt5h	XM_218382		0.12	1.303	0.040017
A_44_P259979	Jak1	XM_342872	84598	0.11	1.303	0.034603
A_44_P304930	Dazap1	NM_001025742	362836	0.11	1.303	0.059261
A_42_P629363	Pfas_predicted	XM_213373		0.11	1.303	0.09422
A_42_P540438	Acyp1_predicted	XM_216757		0.11	1.303	0.399417
A_44_P654083	TC542149	TC542149		0.11	1.303	0.11039
A_44_P419344	LOC685906	XM_001065714	685906	0.11	1.303	0.102512
A_44_P124717	Rbm34	NM_001014015	307956	0.11	1.303	0.030979
A_44_P183979	AW141315	AW141315	287703	0.11	1.303	0.034334
A_43_P10662	RGD1307896_predicted	XM_229475		0.11	1.303	0.069208
A_42_P751756	RGD1308917_predicted	XM_216728		0.11	1.302	0.139962
A_44_P944402	LOC679098	XM_001054697		0.11	1.302	0.153613
A_44_P479777	AI011806	AI011806	296315	0.11	1.302	0.054129
A_44_P559239	AW921223	AW921223	688429	0.11	1.302	0.199728
A_44_P356984	AA900736	AA900736		0.11	1.302	0.131709
A_44_P500708	C1qg	NM_001008524	362634	0.11	1.302	0.334377
A_44_P296774	BF420163	BF420163	81613	0.11	1.302	0.063393
A_44_P263237	CB546826	CB546826		0.11	1.302	0.023131
A_43_P18915	Stk11ip_predicted	XM_237307		0.11	1.302	0.029107
A_44_P294359	RGD1564792_predicted	XM_001061968		0.11	1.301	0.18999
A_44_P925784	Wdr37_predicted	XM_001060920		0.11	1.301	0.093943
A_44_P224911	MGC116096	NM_001024869	295062	0.11	1.301	0.023275
A_42_P492195	Fibp	NM_172334	282837	0.11	1.301	0.170623
A_44_P1053105	Gpsn2	NM_138549	191576	0.11	1.301	0.198423
A_44_P403755	Dusp12	NM_022248	64014	0.11	1.301	0.029681
A_44_P267985	Zfp238	NM_022678	64619	0.11	1.301	0.076781
A_44_P139718	Pdk1	NM_053826	116551	0.11	1.301	0.05978
A_44_P328581	LOC500251	NM_001025047	500251	0.11	1.301	0.122612
A_44_P219811	Ube2v2	NM_183052	287927	0.11	1.300	0.254185
A_43_P12543	Psmc5	NM_031149	81827	0.11	1.300	0.065892
A_44_P1059623	LOC312030	XM_231361	312030	0.11	1.300	0.122553
A_44_P389400	Trp53rk_predicted	XM_342580		0.11	1.300	0.043841
A_44_P230640	Pafah1b1	NM_031763	83572	0.11	1.300	0.070684
A_42_P606251	Mrpl13	NM_001006985	299938	0.11	1.300	0.026654
A_44_P666329	AW143599	AW143599		0.11	1.300	0.072831
A_43_P15901	Jub	NM_053503	85265	0.11	1.300	0.383356

A_44_P516818	RGD1562348_predicted	XM_214012		0.11	1.300	0.192079
A_44_P299214	X80508	X80508		0.11	1.300	0.093284
A_44_P384544	Riok1	XM_214454	291061	0.11	1.300	0.030185
A_42_P713629	RGD1305215	NM_001014013	307643	0.11	1.299	0.055933
A_44_P838277	TC539970	TC539970		0.11	1.299	0.252167
A_44_P225176	RGD1566139_predicted	XM_229709		0.11	1.299	0.069688
A_44_P965649	Tbl1xr1_predicted	XM_001056572		0.11	1.299	0.185349
A_43_P17954	RGD1562231_predicted	XM_217615		0.11	1.299	0.08557
A_44_P256261	Rp1h	XM_001062983		0.11	1.299	0.10131
A_44_P915877	LOC683460	XM_001065403		0.11	1.299	0.271642
A_44_P541992	Cables1_predicted	XM_226165		0.11	1.299	0.342555
A_44_P1004959	Thoc1	AY387056	291797	0.11	1.299	0.086425
A_42_P624773	Cpz	NM_031766	83575	0.11	1.299	0.269597
A_44_P267294	RGD1562018_predicted	XM_341306	361030	0.11	1.299	0.130878
A_44_P1019454	Mrpl41	NM_001013426	296551	0.11	1.299	0.042982
A_43_P21341	RGD1308557_predicted	XM_232664		0.11	1.299	0.038284
A_43_P16778	Ube2f	NM_001008381	363284	0.11	1.299	0.048843
A_44_P898412	ENSRNOT00000050366	ENSRNOT00000050366		0.11	1.298	0.045397
A_42_P632328	Kctd3	XM_223057		0.11	1.298	0.099402
A_44_P296057	Cnn1	NM_031747	65204	0.11	1.298	0.214025
A_44_P870183	TC525332	TC525332		0.11	1.298	0.410605
A_42_P755052	Zfp91	XM_001079228		0.11	1.298	0.01684
A_43_P20332	Thoc2_predicted	XM_233081	313308	0.11	1.298	0.196762
A_44_P856786	A_44_P856786	A_44_P856786		0.11	1.298	0.109671
A_44_P257444	Atad1	NM_001035002	309532	0.11	1.298	0.035249
A_44_P913781	A_44_P913781	A_44_P913781		0.11	1.298	0.095698
A_44_P925867	Napg	XM_001063511		0.11	1.297	0.303428
A_44_P372555	Jmjd1a	NM_175764	312440	0.11	1.297	0.196182
A_44_P135730	RGD1561594_predicted	XM_228802	317394	0.11	1.297	0.159269
A_44_P116606	Ank2	XM_342337	362036	0.11	1.297	0.215295
A_44_P390669	AW921499	AW921499		0.11	1.297	0.46789
A_44_P358632	Rpl21	NM_053330	79449	0.11	1.297	0.069579
A_44_P555498	ENSRNOT00000047215	ENSRNOT00000047215		0.11	1.297	0.018455
A_42_P479312	Rbm5	XM_217263	300996	0.11	1.297	0.094756
A_42_P625546	Ctnnbl1	NM_001024870	296320	0.11	1.297	0.022395
A_44_P268610	RGD1559896_predicted	XM_574254		0.11	1.296	0.028578
A_44_P539322	Sema4c_predicted	XM_237095	301346	0.11	1.296	0.299263
A_43_P12332	Nolc1	NM_022869	64896	0.11	1.296	0.145824
A_44_P412695	Mlf2_predicted	XM_232350		0.11	1.296	0.03596
A_43_P16871	Fyttd1	XM_001081958		0.11	1.296	0.042684
A_44_P550640	Rras2	NM_001013434	365355	0.11	1.296	0.124653
A_44_P469517	BF551572	BF551572		0.11	1.296	0.231648
A_44_P208444	Cwf19l1_predicted	XM_345035		0.11	1.296	0.068674
A_44_P377201	LOC684125	XM_001069045		0.11	1.296	0.197577
A_44_P1060312	Uchl5	NM_001012149	360853	0.11	1.296	0.070284
A_44_P483325	BU759069	BU759069		0.11	1.296	0.020536
A_44_P343303	Ppt2	NM_019367	54398	0.11	1.296	0.044266
A_44_P756687	Pet112l_predicted	XM_001066526		0.11	1.296	0.174259
A_44_P885147	Def8	NM_001024774	307973	0.11	1.296	0.120047
A_44_P270760	AW916078	AW916078	360703	0.11	1.296	0.138329
A_44_P501783	Ppp2r5e_predicted	XM_216739		0.11	1.296	0.309549
A_44_P259727	Rims4	NM_170666	266976	0.11	1.296	0.093027
A_44_P269912	XM_235176	XM_235176		0.11	1.296	0.023355
A_44_P961009	TC556658	TC556658		0.11	1.295	0.099523
A_42_P542583	Hn1	NM_001005876	287828	0.11	1.295	0.115543
A_44_P231955	Rem1	NM_001025753	366232	0.11	1.295	0.057404
A_44_P290424	Siah1a	NM_080905	140941	0.11	1.295	0.041827
A_44_P194294	MGC105830	NM_001008370	1E+08	0.11	1.295	0.144747
A_42_P601920	Cpt2	NM_012930	25413	0.11	1.295	0.189502
A_44_P127910	AW917546	AW917546	497991	0.11	1.295	0.061027
A_43_P22124	Dnajc7	NM_213625	303536	0.11	1.295	0.11088
A_44_P652617	Chd8	XM_573762	65027	0.11	1.295	0.041936
A_44_P259931	Arsb	NM_033443	25227	0.11	1.295	0.188037

A_42_P519868	Gsr	NM_053906	116686	0.11	1.295	0.049049
A_44_P288997	AW916759	AW916759		0.11	1.295	0.089902
A_44_P883305	ENSRNOT00000036927	ENSRNOT00000036927		0.11	1.295	0.125006
A_44_P234309	Smarca5_predicted	XM_226380		0.11	1.294	0.061515
A_44_P194543	Asb13_predicted	XM_341551	361268	0.11	1.294	0.053136
A_43_P12596	Ghrh	NM_031577	29446	0.11	1.294	0.142388
A_43_P18689	CB548152	CB548152	361959	0.11	1.294	0.063034
A_44_P527300	Tchp_predicted	XM_222232	304547	0.11	1.294	0.026639
A_44_P670167	TC544613	TC544613		0.11	1.294	0.17761
A_43_P10008	RGD1562920_predicted	XM_214790	292486	0.11	1.294	0.054321
A_44_P482595	F11r	NM_053796	116479	0.11	1.294	0.065307
A_44_P354078	Hist1h1a_predicted	XM_225330		0.11	1.294	0.192324
A_44_P718743	TC564717	TC564717		0.11	1.294	0.356594
A_44_P1016309	RGD1560168_predicted	XM_346069		0.11	1.293	0.031105
A_43_P18226	Myo1f_predicted	XM_234942		0.11	1.293	0.711913
A_44_P607372	TC557516	TC557516		0.11	1.293	0.043281
A_44_P1070712	ENSRNOT00000040510	ENSRNOT00000040510		0.11	1.293	0.492689
A_42_P778437	Mrpl37	NM_001004235	56281	0.11	1.293	0.021731
A_44_P104976	Ap2b1	NM_080583	140670	0.11	1.293	0.071767
A_44_P1051685	Cxxc1	XM_238016		0.11	1.293	0.022131
A_44_P398574	Prdm1_predicted	XM_228320		0.11	1.293	0.226189
A_44_P300215	Stk11ip_predicted	XM_237307		0.11	1.293	0.078313
A_43_P17424	Chaf1b	NM_001024741	288242	0.11	1.293	0.122005
A_44_P502287	B4galt6	NM_031740	65196	0.11	1.293	0.515302
A_44_P389255	A_44_P389255	A_44_P389255		0.11	1.292	0.184621
A_43_P19880	LOC308954	NM_001024777	308954	0.11	1.292	0.102827
A_44_P356322	XM_219482	XM_219482		0.11	1.292	0.082406
A_43_P11527	Adm	NM_012715	25026	0.11	1.292	0.067026
A_44_P683157	LOC499900	NM_001024313	499900	0.11	1.292	0.098829
A_44_P419168	LOC363675	NM_001014229	363675	0.11	1.292	0.05321
A_43_P15308	Mtap1a	NM_030995	25152	0.11	1.292	0.221427
A_44_P558079	RGD1359127	NM_001007657	299612	0.11	1.292	0.023314
A_44_P494183	Adcy7	XM_226333	84420	0.11	1.292	0.216493
A_44_P158768	Supt5h	XM_218382		0.11	1.292	0.021707
A_44_P482837	Phr1_predicted	XM_214245		0.11	1.292	0.268347
A_44_P726255	AI500919	AI500919		0.11	1.292	0.158161
A_44_P220388	RGD1561086_predicted	XM_231467		0.11	1.291	0.038754
A_44_P352152	LOC683932	XM_001068107		0.11	1.291	0.192589
A_44_P640180	TC560973	TC560973		0.11	1.291	0.242564
A_44_P435739	Hes1	NM_024360	29577	0.11	1.291	0.115416
A_43_P22672	Exo1_predicted	XM_222932		0.11	1.291	0.074708
A_44_P763188	TC562653	TC562653		0.11	1.291	0.099051
A_43_P12297	Snap23	NM_022689	64630	0.11	1.291	0.052221
A_44_P451163	AI009405	AI009405	24484	0.11	1.291	0.193848
A_44_P158965	Mrpl18_predicted	XM_214751		0.11	1.291	0.033134
A_44_P170283	AW917743	AW917743		0.11	1.291	0.151781
A_44_P152335	RGD1564023_predicted	XM_345796		0.11	1.291	0.028654
A_44_P344391	Bles03	NM_001024233	266609	0.11	1.290	0.040472
A_44_P822041	LOC685108	XM_001062327		0.11	1.290	0.042069
A_44_P353978	XM_345064	XM_345064		0.11	1.290	0.034765
A_43_P10414	Ubxd8	NM_001017445	291000	0.11	1.290	0.096783
A_44_P160381	LOC498351	XM_001066807		0.11	1.290	0.120175
A_43_P14875	Prep	NM_031324	83471	0.11	1.290	0.042249
A_42_P743301	Psma1	NM_017278	29668	0.11	1.290	0.123613
A_43_P21480	LOC308320	XM_218215		0.11	1.290	0.239751
A_44_P292141	Pycard	CK842857	282817	0.11	1.290	0.066953
A_44_P1014163	Gsn	NM_001004080	296654	0.11	1.290	0.106363
A_44_P454861	LOC295698	XR_008540	295698	0.11	1.290	0.133595
A_44_P257774	RGD1309256	XM_214087	289783	0.11	1.290	0.047899
A_43_P17007	RGD1561069_predicted	XM_574248		0.11	1.290	0.031912
A_44_P883709	LOC501529	XR_009264	501529	0.11	1.290	0.12935
A_44_P881540	AW142697	AW142697		0.11	1.289	0.065195
A_44_P185027	Sall2_predicted	XM_223992		0.11	1.289	0.161059

A_44_P379420	Gdi1	NM_017088	25183	0.11	1.289	0.146945
A_44_P264785	Slc38a1	NM_138832	170567	0.11	1.289	0.108651
A_44_P175822	LOC682946	XM_001063810		0.11	1.289	0.056895
A_42_P517743	Glmn	XM_213992		0.11	1.289	0.204215
A_42_P740386	Zfp513	NM_001012110	313913	0.11	1.289	0.169204
A_44_P545363	Fh1	NM_017005	24368	0.11	1.289	0.124058
A_42_P836880	Ldlr	NM_175762	300438	0.11	1.289	0.160104
A_44_P209075	RGD1310738_predicted	XM_223565		0.11	1.289	0.028532
A_44_P103278	RGD1310686	NM_001008360	360480	0.11	1.289	0.04819
A_44_P293083	LOC679898	XM_001054881	679898	0.11	1.289	0.315039
A_43_P14654	TC525058	TC525058		0.11	1.289	0.061626
A_44_P995373	LOC682888	XM_001063567		0.11	1.289	0.156196
A_42_P506345	Bat3	NM_053609	94342	0.11	1.289	0.021349
A_44_P697832	AW920624	AW920624		0.11	1.289	0.176274
A_44_P1009948	RGD1304587	XM_220532	303180	0.11	1.289	0.031326
A_44_P170710	Id2	NM_013060	25587	0.11	1.289	0.074816
A_44_P254201	BF398403	BF398403	25478	0.11	1.289	0.212455
A_44_P100507	Numa1	XM_218972	308870	0.11	1.288	0.038153
A_44_P346557	Olr93_predicted	NM_001000141	293234	0.11	1.288	0.142017
A_44_P501503	Slc25a24_predicted	XM_227597	310791	0.11	1.288	0.137028
A_44_P265026	RGD1565234_predicted	XM_001055604		0.11	1.288	0.035974
A_44_P344340	Zfoc1	U78135	498177	0.11	1.288	0.039171
A_44_P143472	RGD1562596_predicted	XM_001065923		0.11	1.288	0.105954
A_44_P286245	Crbn	NM_001015003	297498	0.11	1.288	0.066834
A_44_P453567	RGD1309995_predicted	XM_235003	314690	0.11	1.288	0.451299
A_44_P562496	TC526252	TC526252		0.11	1.288	0.085062
A_44_P488918	RGD1565591_predicted	XM_001077382		0.11	1.288	0.15705
A_44_P203411	Inpp4b	NM_053917	116699	0.11	1.288	0.243741
A_44_P262656	LOC680521	XM_001057547		0.11	1.288	0.514306
A_42_P787743	RGD1306184	NM_001014159	361545	0.11	1.287	0.016533
A_44_P220868	Rpn2	NM_031698	64701	0.11	1.287	0.091757
A_44_P521800	AA875099	AA875099	25497	0.11	1.287	0.096106
A_42_P577129	Tcf1	NM_012669	24817	0.11	1.287	0.069717
A_44_P713838	RGD1559623_predicted	XM_575065	499731	0.11	1.287	0.233091
A_43_P18307	LOC308846	XM_218951		0.11	1.287	0.147862
A_44_P351795	Nup188	XM_345335	366016	0.11	1.287	0.194928
A_43_P17778	Ng35	NR_002143		0.11	1.287	0.147903
A_44_P547976	Aprin_predicted	XM_221833		0.11	1.287	0.127912
A_44_P235256	AW916378	AW916378		0.11	1.287	0.030548
A_43_P22670	RGD1564946_predicted	XM_234416	299199	0.11	1.287	0.024544
A_44_P415957	Mid1	NM_022927	54252	0.11	1.287	0.268409
A_44_P977479	TC543760	TC543760		0.11	1.287	0.138152
A_44_P236532	Ctnnd1_predicted	XM_242062		0.11	1.287	0.054461
A_44_P166046	RGD1311026	NM_001039031	361730	0.11	1.287	0.068269
A_44_P1009892	Pja2	NM_138896	192256	0.11	1.287	0.306389
A_44_P204134	XM_343280	XM_343280		0.11	1.287	0.138244
A_44_P127740	Maf1	NM_001014085	315093	0.11	1.287	0.026823
A_44_P196904	Asf1b_predicted	XM_222451		0.11	1.286	0.07032
A_43_P11099	AI045798	AI045798		0.11	1.286	0.188366
A_44_P196856	RGD1309199	NM_001007799	362149	0.11	1.286	0.108813
A_44_P173617	XM_220179	XM_220179		0.11	1.286	0.225842
A_44_P622978	TC557877	TC557877		0.11	1.286	0.120457
A_44_P353854	RGD1309762_predicted	XM_222205	304503	0.11	1.286	0.097142
A_44_P126161	Cdc37l1	NM_001011941	293886	0.11	1.286	0.424869
A_44_P428682	Aoc2	XM_001060422		0.11	1.286	0.070052
A_44_P975099	Impact	NM_001012235	497198	0.11	1.286	0.058834
A_44_P449958	RGD1310724	NM_001014127	360803	0.11	1.286	0.088934
A_44_P442835	U78126	U78126	292610	0.11	1.286	0.028624
A_44_P899704	A_44_P899704	A_44_P899704		0.11	1.286	0.107638
A_42_P528007	Srp54	NM_053871	116650	0.11	1.286	0.156536
A_42_P723173	Id1	NM_012797	25261	0.11	1.286	0.284013
A_43_P15307	Mdk	NM_030859	81517	0.11	1.285	0.259961
A_44_P419162	Smad1	NM_013130	25671	0.11	1.285	0.148646

A_44_P525187	Rnase9	NM_001008561	364301	0.11	1.285	0.47734
A_44_P501623	Pcmtd2_predicted	XM_230971		0.11	1.285	0.364525
A_44_P114589	RGD1306674_predicted	XM_226449	307834	0.11	1.285	0.030943
A_44_P290961	A_44_P290961	A_44_P290961		0.11	1.285	0.085193
A_44_P433425	Pdhb	NM_001007620	289950	0.11	1.285	0.111528
A_43_P22553	RGD1560020_predicted	XM_001061499		0.11	1.285	0.091215
A_42_P811423	Vcl_predicted	XM_001057629		0.11	1.285	0.083911
A_44_P278243	BQ190284	BQ190284	312444	0.11	1.285	0.139981
A_44_P501850	LOC314964	XM_001065978	314964	0.11	1.285	0.113439
A_44_P625286	TC518879	TC518879		0.11	1.285	0.14667
A_44_P531870	Ephx2	NM_022936	65030	0.11	1.285	0.314396
A_43_P19998	XM_233168	XM_233168		0.11	1.285	0.11432
A_44_P855196	TC526532	TC526532		0.11	1.285	0.116922
A_43_P19738	RGD1560908_predicted	XM_223589		0.11	1.285	0.117175
A_44_P125994	Uck1_predicted	XM_231143		0.11	1.284	0.048794
A_44_P961679	TC543281	TC543281		0.11	1.284	0.090956
A_43_P21172	RGD1564081_predicted	XM_001073215		0.11	1.284	0.070623
A_44_P553158	RGD1307433	NM_001014014	307652	0.11	1.284	0.049956
A_44_P623770	TC543488	TC543488		0.11	1.284	0.324404
A_44_P1026431	Xrn2_predicted	XM_342535		0.11	1.284	0.161176
A_43_P15689	Ythdf2_predicted	XM_001063404		0.11	1.284	0.065359
A_44_P823666	Zhx2	XM_001067896		0.11	1.284	0.081409
A_44_P429440	Tmem11_predicted	XM_220530	303196	0.11	1.284	0.035823
A_44_P311620	LOC686579	XM_001078152		0.11	1.284	0.101328
A_44_P403189	Svs6_predicted	XM_342575		0.11	1.284	0.177063
A_44_P523045	XM_344781	XM_344781		0.11	1.284	0.085328
A_44_P821599	LOC679116	XM_001054758		0.11	1.284	0.10562
A_44_P398792	Tpt1	NM_053867	116646	0.11	1.284	0.05438
A_43_P16691	Arid1a_predicted	XM_216340		0.11	1.284	0.066059
A_42_P842057	Hip2_predicted	XM_214043		0.11	1.284	0.060951
A_44_P118027	LOC681578	XM_001057755		0.11	1.284	0.29424
A_44_P128147	Tf	NM_001013110	24825	0.11	1.283	0.562489
A_44_P791255	ENSRNOT00000023364	ENSRNOT00000023364		0.11	1.283	0.125437
A_44_P537385	RGD1311730_predicted	XM_001079737		0.11	1.283	0.064638
A_44_P609518	TC551367	TC551367		0.11	1.283	0.163573
A_42_P815698	LOC497967	NM_001037360	497967	0.11	1.283	0.072501
A_44_P831282	Hrmt1l1	NM_001025144	499420	0.11	1.283	0.098228
A_44_P1002173	Cuedc2_predicted	XM_215274		0.11	1.283	0.052157
A_42_P757370	Prkce	NM_017171	29340	0.11	1.283	0.300544
A_44_P436149	A_44_P436149	A_44_P436149		0.11	1.283	0.076271
A_44_P441039	Hint2_predicted	XM_233377		0.11	1.282	0.042771
A_44_P891514	TC561929	TC561929		0.11	1.282	0.116504
A_44_P783809	BE118459	BE118459	689128	0.11	1.282	0.469511
A_44_P1046847	RGD1309016	NM_001009391	305177	0.11	1.282	0.133803
A_44_P222829	XM_235811	XM_235811		0.11	1.282	0.150617
A_42_P692306	Tbl3	NM_001008277	287120	0.11	1.282	0.053434
A_44_P577111	LOC360912	BC086609	360912	0.11	1.282	0.073646
A_44_P672457	TC546388	TC546388		0.11	1.282	0.112135
A_42_P738726	Dpy19l3_predicted	XM_218514	308519	0.11	1.282	0.118776
A_44_P516789	BE120498	BE120498	499157	0.11	1.282	0.059881
A_44_P365384	Pax3	XM_343601	114502	0.11	1.281	0.199854
A_42_P468805	Cdk5rap1	NM_145721	252827	0.11	1.281	0.204679
A_44_P928928	RGD1565157_predicted	XR_008813	361973	0.11	1.281	0.126953
A_44_P107701	LOC683246	XM_001065089		0.11	1.281	0.171569
A_43_P12692	Git1	NM_031814	83709	0.11	1.281	0.051515
A_44_P471757	Tcerg1_predicted	XM_225983		0.11	1.281	0.105557
A_44_P192312	Cnot4	NM_001037782	312227	0.11	1.281	0.107292
A_44_P977375	TC558648	TC558648		0.11	1.281	0.098833
A_44_P206154	RGD1307244_predicted	XM_345231		0.11	1.281	0.26101
A_44_P456985	XM_223446	XM_223446		0.11	1.280	0.144644
A_42_P702933	Fbf1_predicted	XM_213526		0.11	1.280	0.095759
A_44_P1049020	LOC679221	XM_001055029		0.11	1.280	0.041588
A_44_P435016	Nudt2	NM_207596	297998	0.11	1.280	0.116055

A_44_P100117	Ring1	NM_212549	309626	0.11	1.280	0.046986
A_44_P139182	AI059948	AI059948	83620	0.11	1.280	0.057294
A_44_P1025944	Slc35a4	NM_147140	257647	0.11	1.280	0.102886
A_44_P523000	LOC691960	XM_001080361	691960	0.11	1.280	0.223748
A_44_P157118	Foxred1_predicted	XM_235988		0.11	1.280	0.048843
A_42_P800669	BF289687	BF289687		0.11	1.280	0.024263
A_44_P344990	Slc35e2_predicted	XM_233711		0.11	1.280	0.373815
A_42_P664259	Abca2	NM_024396	79248	0.11	1.280	0.089368
A_44_P304374	Zfp422_predicted	NM_001012745	360389	0.11	1.279	0.059454
A_44_P636436	Tmem109	NM_001007736	361732	0.11	1.279	0.069441
A_44_P288333	RGD1565560_predicted	XM_223504		0.11	1.279	0.06932
A_44_P269950	RGD1564242_predicted	XM_234056	314033	0.11	1.279	0.216752
A_44_P555418	RGD1563099_predicted	XM_217678		0.11	1.279	0.035268
A_44_P900140	TC523749	TC523749		0.11	1.279	0.082244
A_44_P106883	AI547833	AI547833	289278	0.11	1.279	0.092426
A_44_P333057	Mtrr	NM_001039003	290947	0.11	1.279	0.099461
A_44_P117245	XM_213105	XM_213105		0.11	1.279	0.115699
A_43_P22270	LOC680726	XM_001061274	680726	0.11	1.279	0.17865
A_44_P466827	RGD1309220	NM_001034835	316328	0.11	1.279	0.082629
A_43_P20858	Nhlrc2_predicted	XM_217640		0.11	1.279	0.100894
A_44_P684779	LOC691396	XM_001078065	691396	0.11	1.279	0.052477
A_44_P803194	BE119385	BE119385	252959	0.11	1.279	0.043485
A_44_P989601	A_44_P989601	A_44_P989601		0.11	1.279	0.083643
A_44_P356355	RGD1308326_predicted	XM_217671	308009	0.11	1.278	0.385059
A_44_P516867	RGD1306193	NM_001024792	360636	0.11	1.278	0.043299
A_44_P1047375	Rnf10	NM_001011904	288710	0.11	1.278	0.039161
A_44_P836950	ENSRNOT00000022805	ENSRNOT00000022805		0.11	1.278	0.03609
A_44_P1041677	RGD1560888_predicted	XM_001061556		0.11	1.278	0.189092
A_44_P976124	TC557724	TC557724		0.11	1.278	0.176172
A_44_P883933	TC518924	TC518924		0.11	1.278	0.175853
A_44_P273989	Ppp2ca	NM_017039	24672	0.11	1.278	0.178103
A_44_P1017841	RGD1311835	NM_001014202	362912	0.11	1.278	0.137398
A_44_P129187	Il17r_predicted	XM_232247		0.11	1.278	0.08591
A_44_P340276	LOC678934	XM_001054058		0.11	1.278	0.247627
A_44_P544063	Irgm	NM_001012007	303090	0.11	1.278	0.164929
A_44_P437532	RGD1308695_predicted	XM_213894		0.11	1.278	0.314041
A_43_P14290	RGD1564361_predicted	XM_001066912		0.11	1.278	0.065086
A_44_P308925	Abat	NM_031003	81632	0.11	1.278	0.111587
A_43_P10127	MGC94335	NM_001004251	301418	0.11	1.278	0.061017
A_43_P15263	Grm1	NM_017011	24414	0.11	1.278	0.283969
A_43_P12481	Gabbr1	AB016161		0.11	1.278	0.318902
A_44_P173516	XM_234405	XM_234405		0.11	1.277	0.102353
A_44_P302433	LOC317546	XM_001055457		0.11	1.277	0.257575
A_42_P815372	Snrpb2_predicted	XM_342528		0.11	1.277	0.078839
A_43_P20192	Nsd1_predicted	XM_225168		0.11	1.277	0.035823
A_42_P747311	Ptpn23	NM_057204	117552	0.11	1.277	0.037483
A_43_P15824	Acvr2b	NM_031554	25366	0.11	1.277	0.063226
A_44_P837344	Esco2_predicted	XM_224405		0.11	1.277	0.120274
A_42_P692054	Nfia	NM_012988	25492	0.11	1.277	0.094367
A_44_P1054453	Uap1l1_predicted	XM_216004	296560	0.11	1.277	0.082461
A_43_P10230	Slc9a6_predicted	XM_217630	302863	0.11	1.277	0.221239
A_44_P552986	Ttc9c	NM_001007693	309196	0.11	1.277	0.078666
A_44_P222779	RGD1305162_predicted	XM_342937		0.11	1.277	0.058765
A_44_P699895	TC556630	TC556630		0.11	1.277	0.155797
A_44_P194905	Ddx42_predicted	XM_221037		0.11	1.276	0.148003
A_44_P257898	Rreb1_predicted	XM_225250		0.11	1.276	0.077423
A_44_P1071281	AI072660	AI072660		0.11	1.276	0.329594
A_43_P21527	XM_225165	XM_225165		0.11	1.276	0.141683
A_42_P582004	RGD1304793_predicted	XM_232731	313021	0.11	1.276	0.026006
A_44_P229887	Acbd4	NM_001012013	303577	0.11	1.276	0.066446
A_44_P370798	XM_343271	XM_343271		0.11	1.276	0.10428
A_42_P713975	LOC499779	XM_575112	499779	0.11	1.276	0.072089
A_44_P180676	RGD1566064_predicted	XM_341141	360865	0.11	1.276	0.075141

A_44_P142843	LOC685834	XM_001065442	685834	0.11	1.276	0.071335
A_44_P200431	AA965274	AA965274	306168	0.11	1.276	0.067737
A_44_P434564	BF396498	BF396498	306251	0.11	1.276	0.10063
A_43_P19376	AW918874	AW918874	311427	0.11	1.275	0.350344
A_44_P253468	Polr2a_mapped	XM_343922	363633	0.11	1.275	0.060624
A_44_P443343	Pdk3_mapped	XM_216091		0.11	1.275	0.212555
A_44_P249976	RGD1305246	NM_001014028	309399	0.11	1.275	0.37912
A_44_P483369	AA945701	AA945701		0.11	1.275	0.247992
A_43_P16038	Prkcm	XM_234108	85421	0.11	1.275	0.191495
A_44_P962013	TC544019	TC544019		0.11	1.275	0.302038
A_43_P12711	Rps6kb1	NM_031985	83840	0.11	1.275	0.052972
A_44_P606810	TC520801	TC520801		0.11	1.275	0.043895
A_44_P168586	LOC687383	XM_001078263		0.11	1.275	0.335024
A_44_P560952	Scamp1	XM_001059581		0.11	1.275	0.425578
A_44_P145549	Rnf4	NM_019182	29274	0.11	1.275	0.103744
A_42_P620921	RGD1562747_predicted	XM_214774		0.11	1.275	0.147323
A_44_P849067	Rnmt	NM_001008299	291534	0.11	1.274	0.101737
A_44_P780805	RGD1310199	NM_001017446	291737	0.11	1.274	0.229559
A_44_P777373	RGD1560519_predicted	XM_001060686		0.11	1.274	0.113392
A_44_P375553	Lrp1	XM_243524	299858	0.11	1.274	0.162529
A_44_P959692	LOC683646	XM_001066878		0.11	1.274	0.04667
A_44_P715389	Qrsl1	NM_001014034	309911	0.11	1.274	0.236146
A_44_P1018398	Kdelc1	XM_237102		0.11	1.274	0.613199
A_43_P10890	Tbca	NM_001013245	366995	0.11	1.274	0.063707
A_42_P837214	Mgmt	NM_012861	25332	0.11	1.274	0.293968
A_43_P16155	Braf	XM_231692	114486	0.11	1.274	0.124764
A_44_P987226	Plxna2_predicted	XM_001070296		0.11	1.274	0.069537
A_44_P391220	LOC499120	XM_574414	499120	0.11	1.274	0.043691
A_44_P341870	Prkag2	NM_184051	373545	0.11	1.274	0.476106
A_44_P140100	RGD1308992_predicted	XM_341142	360866	0.11	1.274	0.286799
A_44_P773287	AI454908	AI454908		0.11	1.274	0.144117
A_44_P243847	RGD1308299_predicted	XM_346013	367214	0.10	1.273	0.241209
A_44_P622886	TC538008	TC538008		0.10	1.273	0.05322
A_44_P405775	Asb8_predicted	XM_235618		0.10	1.273	0.081301
A_44_P285507	Fmr1	NM_052804	24948	0.10	1.273	0.259257
A_44_P394215	RGD1561157_predicted	XM_346883	360487	0.10	1.273	0.03474
A_44_P401955	LOC500855	XM_576251	500855	0.10	1.273	0.04753
A_44_P806040	Tlk2_predicted	XM_221023	303592	0.10	1.273	0.235768
A_44_P899961	Ckap5	XM_001068589		0.10	1.273	0.047838
A_43_P19378	Papd4	NM_001008372	361878	0.10	1.273	0.067793
A_42_P607092	Glipr1	NM_001011987	299783	0.10	1.273	0.13095
A_44_P463783	Zfp94	NM_001037212	499095	0.10	1.273	0.174164
A_44_P668226	Arcn1	NM_001007662	300674	0.10	1.273	0.08377
A_44_P366274	BE120098	BE120098	306322	0.10	1.273	0.237111
A_44_P539410	BF389611	BF389611		0.10	1.273	0.068493
A_44_P330594	Prkd2	NM_001013895	292658	0.10	1.272	0.040698
A_44_P183185	Fxr2h_predicted	XM_213335	287433	0.10	1.272	0.05995
A_44_P476024	LOC362652	NM_001014194	362652	0.10	1.272	0.219155
A_44_P118724	Arc	NM_019361	54323	0.10	1.272	0.088336
A_44_P575662	Afaf	NM_001039345	500502	0.10	1.272	0.136314
A_43_P20823	Stac2_predicted	XM_343962		0.10	1.272	0.272656
A_42_P537881	Itpa_mapped	XM_230604		0.10	1.272	0.076497
A_42_P516640	Rabep1	NM_019124	54190	0.10	1.272	0.071824
A_44_P354415	Trem1_predicted	XM_217336		0.10	1.272	0.305454
A_44_P680749	RGD1565033_predicted	XM_573212		0.10	1.272	0.107222
A_43_P21848	RGD1359201	XM_001077093		0.10	1.272	0.088312
A_44_P254413	Hnrpu	NM_057139	117280	0.10	1.272	0.075375
A_43_P15563	LOC679840	XM_001054684		0.10	1.271	0.168707
A_42_P783205	Dclre1c	NM_147145	259171	0.10	1.271	0.072814
A_44_P606573	TC535418	TC535418		0.10	1.271	0.097665
A_44_P267993	CB546126	CB546126		0.10	1.271	0.15538
A_44_P208896	LOC316457	XM_237217	316457	0.10	1.271	0.047049
A_44_P452217	Z93354	Z93354		0.10	1.271	0.23833

A_44_P995906	Polh_predicted	XM_236934		0.10	1.271	0.030317
A_44_P1051281	Tpr	XM_222745		0.10	1.271	0.086524
A_44_P503395	Olr63	NM_001000542	365323	0.10	1.271	0.415863
A_44_P347007	Atp6v1a1_predicted	XM_001060277		0.10	1.271	0.094126
A_44_P466727	Adck1_predicted	XM_345709		0.10	1.271	0.076881
A_44_P364678	BF550568	BF550568	300968	0.10	1.270	0.108316
A_44_P226162	AA997614	AA997614	25427	0.10	1.270	0.129289
A_44_P290357	Eml2	NM_138921	192360	0.10	1.270	0.026252
A_44_P622113	BF557076	BF557076		0.10	1.270	0.120866
A_44_P265583	Atpaf1_predicted	XM_233402		0.10	1.270	0.140623
A_42_P813893	RGD1560991_predicted	XM_577689	502227	0.10	1.269	0.086437
A_44_P133924	BE101505	BE101505	362831	0.10	1.269	0.087082
A_42_P453894	RGD1306682_predicted	XM_340892		0.10	1.269	0.157939
A_44_P604860	BC098774	BC098774		0.10	1.269	0.115841
A_44_P418912	DV718192	DV718192		0.10	1.269	0.070211
A_44_P295376	RGD1563079_predicted	XM_001061018		0.10	1.269	0.197369
A_44_P972656	AI548160	AI548160		0.10	1.269	0.291559
A_44_P211015	XM_229147	XM_229147		0.10	1.269	0.185574
A_44_P934649	TC532273	TC532273		0.10	1.269	0.130992
A_43_P16067	Kif11	XM_001060913	171304	0.10	1.269	0.150374
A_44_P650446	AI060246	AI060246		0.10	1.268	0.318437
A_44_P656856	CK596682	CK596682		0.10	1.268	0.461708
A_44_P457139	A_44_P457139	A_44_P457139		0.10	1.268	0.213967
A_43_P14883	Psma4	NM_017281	29671	0.10	1.268	0.045526
A_44_P344487	Eif2c2	NM_021597	59117	0.10	1.268	0.073003
A_44_P120635	RGD1561940_predicted	XR_007829	364686	0.10	1.268	0.315886
A_44_P806046	A_44_P806046	A_44_P806046		0.10	1.268	0.120666
A_44_P409526	Gpsm1	NM_144745	246254	0.10	1.268	0.090807
A_43_P12125	Junb	NM_021836	24517	0.10	1.268	0.123322
A_44_P375091	RGD1307904_predicted	XM_341920	361641	0.10	1.268	0.09422
A_44_P488185	Ascc3_predicted	XM_228345		0.10	1.268	0.285842
A_44_P436063	RGD1560623_predicted	XR_008555	305492	0.10	1.268	0.206259
A_44_P207595	AA818261	AA818261		0.10	1.268	0.185009
A_44_P101375	Vps16	NM_001005541	296159	0.10	1.267	0.133082
A_44_P168867	Laptm4b	NM_001013174	315047	0.10	1.267	0.107667
A_44_P760327	MGC94282	NM_001007649	297627	0.10	1.267	0.128901
A_44_P409803	Cd5	NM_019295	54236	0.10	1.267	0.090533
A_43_P12403	Fgr	NM_024145	79113	0.10	1.267	0.153655
A_44_P787241	Pfas_predicted	XM_213373		0.10	1.267	0.101736
A_43_P19727	Tnfrsf19l_predicted	XM_341893		0.10	1.267	0.349603
A_44_P776708	TC521828	TC521828		0.10	1.267	0.417584
A_43_P22777	Cyfip1_predicted	XM_218717		0.10	1.267	0.072774
A_44_P341189	Foxk2_predicted	XM_221212		0.10	1.267	0.103903
A_43_P10725	Rab18	NM_001012468	307039	0.10	1.267	0.13724
A_44_P667890	RGD1560011_predicted	XM_001073577		0.10	1.267	0.112619
A_44_P454420	RT1-S3	XM_215303		0.10	1.267	0.139499
A_44_P833199	AW143635	AW143635		0.10	1.267	0.567371
A_44_P559055	AA925039	AA925039	81750	0.10	1.266	0.185152
A_44_P473234	Zfp688_predicted	XM_215090	293511	0.10	1.266	0.142771
A_43_P16718	Cdc23	XM_214588		0.10	1.266	0.06472
A_44_P508434	Neu1	NM_031522	24591	0.10	1.266	0.199679
A_44_P551275	Eif5b	XM_218162		0.10	1.266	0.064007
A_44_P148044	Hnrpll_predicted	XM_233805	313842	0.10	1.266	0.092955
A_44_P449799	Pabpn1	XM_214172	116697	0.10	1.266	0.040751
A_44_P284250	CA509859	CA509859	296612	0.10	1.266	0.454021
A_44_P474037	Igf1r	NM_052807	25718	0.10	1.266	0.075309
A_44_P123156	AA894004	AA894004	297339	0.10	1.266	0.208683
A_44_P991565	Bad	NM_022698	64639	0.10	1.265	0.076216
A_44_P229528	Hist1h2bm_predicted	XM_341530		0.10	1.265	0.385479
A_44_P640999	CO561668	CO561668	308624	0.10	1.265	0.121236
A_44_P108982	Sucla2_predicted	XM_341354		0.10	1.265	0.133158
A_44_P310471	Vapa	NM_031631	58857	0.10	1.265	0.06906
A_42_P618372	Nradd	NM_139259	246143	0.10	1.265	0.313316

A_44_P852498	RGD1562996_predicted	XM_575911	500549	0.10	1.265	0.181736
A_44_P269119	Dync1li1	NM_145772	252902	0.10	1.265	0.241911
A_42_P672793	Pcnxl3	XM_219515	309167	0.10	1.265	0.067305
A_42_P726132	RGD1305647_predicted	XM_230497	311350	0.10	1.265	0.104233
A_44_P356787	RGD1562434_predicted	XM_236722	316087	0.10	1.265	0.240563
A_44_P267218	Hbxap_predicted	XM_218939	308839	0.10	1.265	0.041229
A_42_P810287	Tacc1	NM_001004107	306562	0.10	1.265	0.140104
A_44_P546058	A_44_P546058	A_44_P546058		0.10	1.265	0.451384
A_44_P977515	TC563154	TC563154		0.10	1.265	0.135903
A_44_P111347	AW915245	AW915245	360471	0.10	1.265	0.109033
A_43_P15542	Parva	NM_020656	57341	0.10	1.265	0.077902
A_44_P807294	TC521402	TC521402		0.10	1.264	0.253518
A_43_P15311	Adcy2	NM_031007	81636	0.10	1.264	0.100891
A_44_P210892	Nanp	NM_001009409	311530	0.10	1.264	0.113843
A_44_P251295	Usp1	NM_001015015	313387	0.10	1.264	0.294376
A_44_P479376	BG372885	BG372885	292688	0.10	1.264	0.047696
A_42_P841017	Elavl2	NM_173309	286973	0.10	1.264	0.333413
A_44_P617630	AW914552	AW914552		0.10	1.264	0.179075
A_44_P368032	RGD1305574	XM_341388	361102	0.10	1.264	0.027809
A_43_P17680	RGD1559552_predicted	XM_573188	497991	0.10	1.264	0.193664
A_44_P807109	DV726956	DV726956		0.10	1.264	0.189965
A_44_P928669	RGD1562309_predicted	XM_577124		0.10	1.264	0.23895
A_44_P702279	BQ204552	BQ204552		0.10	1.264	0.515501
A_44_P184553	Aptx	NM_148889	259271	0.10	1.264	0.165398
A_44_P895621	AA956382	AA956382	24157	0.10	1.263	0.260549
A_44_P436577	Ssh2_predicted	XM_220743		0.10	1.263	0.072774
A_42_P728063	AA875623	AA875623		0.10	1.263	0.238298
A_44_P546159	RGD1310651_predicted	XM_342610	362297	0.10	1.263	0.074366
A_44_P252922	Sec23ip	XM_001080274	309010	0.10	1.263	0.193637
A_44_P426029	AA849721	AA849721	287155	0.10	1.263	0.180108
A_44_P478104	Rab2b	NM_001037645	305853	0.10	1.263	0.153839
A_44_P489486	Ryr2	U95147	84025	0.10	1.263	0.299209
A_44_P381917	Sphk1	NM_133386	170897	0.10	1.263	0.317393
A_44_P553190	Gan_predicted	XM_226517		0.10	1.263	0.306221
A_44_P847392	TC532514	TC532514		0.10	1.262	0.211966
A_42_P499859	Pard6a	NM_001003654	307799	0.10	1.262	0.081759
A_44_P270669	AW918519	AW918519		0.10	1.262	0.192354
A_42_P793687	RGD1559904_predicted	XM_001053130		0.10	1.262	0.120145
A_44_P513472	RGD1564243_predicted	XM_225362		0.10	1.262	0.073469
A_44_P281795	Zfp592_predicted	XM_214975		0.10	1.262	0.10634
A_44_P121227	Casp9	NM_031632	58918	0.10	1.262	0.108258
A_44_P196933	RGD1307801	NM_001014031	309656	0.10	1.262	0.159925
A_43_P17696	Hspa14	NM_001004257	307133	0.10	1.262	0.117111
A_44_P532057	LOC501979	XM_577408		0.10	1.262	0.175098
A_44_P551909	Ep400	XM_222251		0.10	1.262	0.05752
A_44_P406874	AI169370	AI169370	64158	0.10	1.262	0.164254
A_44_P446409	Atf2	NM_031018	81647	0.10	1.262	0.053336
A_42_P755992	Psarl	NM_001035249	287979	0.10	1.262	0.080077
A_44_P101023	RGD1564778_predicted	XM_239335		0.10	1.262	0.050233
A_44_P530424	RGD1309459	BC085931	360477	0.10	1.262	0.064028
A_44_P1013385	Eef1e1_predicted	XM_214451		0.10	1.262	0.083129
A_44_P211992	BE097621	BE097621	362838	0.10	1.262	0.053343
A_44_P272253	LOC288978	XM_213864	288978	0.10	1.262	0.234519
A_44_P178129	Tcerg1_predicted	XM_225983		0.10	1.262	0.187542
A_44_P391196	Csh1l1	NM_033233	24282	0.10	1.262	0.358798
A_44_P283772	RGD1561342_predicted	XM_573876		0.10	1.261	0.101546
A_44_P354302	Trim54	NM_001013217	362708	0.10	1.261	0.053632
A_44_P517964	RGD1564549_predicted	XM_214831		0.10	1.261	0.043865
A_44_P108003	RGD1564315_predicted	XM_343227	362899	0.10	1.261	0.379958
A_44_P255415	LOC682196	XM_001060366		0.10	1.261	0.049439
A_44_P154112	Prkaa2	NM_023991	78975	0.10	1.261	0.687566
A_44_P548468	Kif5b	NM_057202	117550	0.10	1.261	0.104952
A_44_P886161	TC545568	TC545568		0.10	1.261	0.274072

A_44_P805040	Ppfibp2	XM_219201	308918	0.10	1.261	0.358385
A_44_P931036	TC559652	TC559652		0.10	1.261	0.050455
A_44_P652243	ENSRNOT00000031802	ENSRNOT00000031802		0.10	1.261	0.153288
A_44_P415082	Mrps15	NM_001007653	298517	0.10	1.261	0.088782
A_44_P806297	LOC365756	XR_008047	365756	0.10	1.261	0.187222
A_44_P222855	ENSRNOT00000048196	ENSRNOT00000048196		0.10	1.261	0.044454
A_43_P11492	Prl	NM_012629	24683	0.10	1.261	0.057188
A_44_P345009	Bag5	NM_001008526	366734	0.10	1.260	0.047049
A_43_P15231	Coq3	NM_019187	29309	0.10	1.260	0.224347
A_44_P213371	A_44_P213371	A_44_P213371		0.10	1.260	0.048856
A_44_P170821	Tmem63a_predicted	XM_223000	289318	0.10	1.260	0.204827
A_42_P604425	Hebp1_predicted	XM_342775		0.10	1.260	0.107505
A_43_P23187	RGD1560851_predicted	XM_342749	362428	0.10	1.260	0.25631
A_44_P745488	Pcnx	XM_001055794		0.10	1.260	0.122882
A_44_P330692	Birc1b	XM_226742	191568	0.10	1.260	0.492662
A_44_P142368	RGD1309666_predicted	XM_344915	365297	0.10	1.260	0.113926
A_44_P386949	RGD1311283_predicted	XM_225063		0.10	1.260	0.098038
A_44_P506169	Tial1_mapped	NM_001013193	361655	0.10	1.260	0.131893
A_44_P319187	Mobkl2b_predicted	XM_343161		0.10	1.260	0.096776
A_44_P154218	XM_213991	XM_213991		0.10	1.259	0.09064
A_42_P545928	Klhl12	NM_153730	266772	0.10	1.259	0.271794
A_44_P311892	Topors_predicted	XM_342819		0.10	1.259	0.216132
A_43_P15427	Sct	NM_022670	24769	0.10	1.259	0.191051
A_44_P258173	Dos_predicted	XM_234901		0.10	1.259	0.07628
A_44_P623236	RGD1562875_predicted	XM_221920	288489	0.10	1.259	0.060158
A_44_P503868	LOC310891	XR_009039	310891	0.10	1.259	0.123537
A_44_P420225	RGD1563982_predicted	XM_001073901		0.10	1.259	0.403315
A_44_P111941	Ptpre	XM_341950	114767	0.10	1.259	0.149539
A_43_P16639	LOC680782	XM_001058844	680782	0.10	1.259	0.085177
A_44_P591224	TC542461	TC542461		0.10	1.258	0.06143
A_44_P518041	H3f3b	BC086580	117056	0.10	1.258	0.094911
A_44_P992854	Rhoa	NM_057132	117273	0.10	1.258	0.052461
A_42_P640478	Gnaq	NM_031036	81666	0.10	1.258	0.121029
A_44_P526852	AI600124	AI600124	500065	0.10	1.258	0.183603
A_44_P994768	Ssr3	NM_031120	81784	0.10	1.258	0.130591
A_44_P482444	ENSRNOT00000051206	ENSRNOT00000051206		0.10	1.258	0.399062
A_43_P10125	AW142713	AW142713	360764	0.10	1.258	0.03625
A_44_P342966	Rwdd3	XM_342332	65026	0.10	1.258	0.281773
A_44_P412418	LOC685393	XM_001063618		0.10	1.258	0.085193
A_44_P791413	TC517666	TC517666		0.10	1.258	0.063946
A_42_P637618	RGD1305823	XM_340737		0.10	1.258	0.045721
A_43_P11465	Fst	NM_012561	24373	0.10	1.257	0.220043
A_44_P538334	Impa2	NM_172224	282636	0.10	1.257	0.200623
A_44_P379367	RT1-N1	NM_012646	24748	0.10	1.257	0.265961
A_44_P270096	LOC287522	XM_220702		0.10	1.257	0.093795
A_44_P462768	BF563517	BF563517	690830	0.10	1.257	0.118891
A_43_P17626	RGD1563441_predicted	XM_573122	497935	0.10	1.257	0.095692
A_44_P527134	Oxr1	XM_576258	117520	0.10	1.257	0.139065
A_44_P499318	LOC294154	NM_001039607	294154	0.10	1.257	0.116293
A_44_P403139	Lnp_predicted	XM_342450		0.10	1.257	0.333266
A_44_P157855	AI178000	AI178000	360823	0.10	1.257	0.055537
A_42_P837441	TC556984	TC556984		0.10	1.257	0.160714
A_43_P13557	CA511336	CA511336		0.10	1.257	0.035379
A_42_P495691	Bcl7c_predicted	XM_215076		0.10	1.257	0.071204
A_44_P353556	Olr404_predicted	NM_001000382	296667	0.10	1.257	0.215928
A_44_P257995	RGD1562218	NM_001034919	292100	0.10	1.257	0.146109
A_44_P318466	Trim25_mapped	NM_001009536	494338	0.10	1.257	0.125046
A_44_P485067	Rgma_predicted	XM_218791		0.10	1.257	0.355423
A_44_P588037	BG666601	BG666601		0.10	1.256	0.454275
A_44_P544835	RGD1566310_predicted	XM_573451		0.10	1.256	0.240277
A_44_P199202	RGD1306286_predicted	XM_001056478		0.10	1.256	0.116755
A_43_P21855	Wipi2	NM_001007615	288498	0.10	1.256	0.083382
A_44_P192548	Gtpbp2	XM_343535	363195	0.10	1.256	0.085018

A_44_P346365	RGD1560191_predicted	XM_001070702		0.10	1.256	0.123248
A_44_P1046705	RGD1559901_predicted	XM_574478		0.10	1.256	0.038608
A_44_P680735	Ap1s1_predicted	XM_341052		0.10	1.256	0.070082
A_44_P292558	Skiv2l	NM_213559	294260	0.10	1.256	0.065464
A_44_P520382	LOC474154	NM_001007014	474154	0.10	1.256	0.100351
A_43_P16552	Git2	NM_001005553	304546	0.10	1.256	0.101164
A_44_P1034613	LOC619574	NM_001034959	619574	0.10	1.256	0.041497
A_44_P210883	Btbd3_predicted	XM_230616		0.10	1.256	0.16101
A_43_P15788	Kifc3	XM_240978		0.10	1.256	0.082532
A_44_P461351	Cdw92	NM_001033852	85254	0.10	1.256	0.131574
A_43_P15061	BF555924	BF555924		0.10	1.256	0.212924
A_44_P911528	DQ480745	DQ480745	303602	0.10	1.256	0.061626
A_43_P13225	Gcsh	NM_133598	171133	0.10	1.256	0.083964
A_44_P198965	Zfp592_predicted	XM_214975		0.10	1.256	0.056668
A_44_P372215	Tmem110	NM_198774	361110	0.10	1.255	0.12253
A_42_P808294	Ccndbp1	NM_001013204	362201	0.10	1.255	0.060987
A_44_P108217	LOC686567	XM_001074770		0.10	1.255	0.240245
A_44_P420869	BM391518	BM391518		0.10	1.255	0.193554
A_44_P351452	Bmp1	XM_573814	83470	0.10	1.255	0.098303
A_44_P531511	Rars_predicted	XM_213276		0.10	1.255	0.303086
A_44_P482928	Abt1	XM_225368		0.10	1.255	0.130905
A_44_P318941	RGD1564062_predicted	XM_225467		0.10	1.255	0.084274
A_44_P218490	Fndc3b_predicted	XM_226988	294925	0.10	1.255	0.23812
A_44_P128042	Sh3md2	NM_198764	306417	0.10	1.255	0.064574
A_43_P11134	Arpc5	NM_001025717	360854	0.10	1.255	0.067378
A_44_P431354	RGD1309592	NM_001012346	290963	0.10	1.255	0.11761
A_42_P629394	Smc1l1	NM_031683	63996	0.10	1.255	0.069104
A_44_P1055467	Ap3s1_predicted	XM_217560		0.10	1.255	0.208913
A_44_P1024602	Sytl1	NM_001025651	297872	0.10	1.255	0.184844
A_44_P114606	XM_228088	XM_228088		0.10	1.254	0.035379
A_44_P499113	Mettl3	NM_001024794	361035	0.10	1.254	0.050341
A_44_P249164	Gabbr1	NM_031028	81657	0.10	1.254	0.100449
A_44_P1034087	Coq3	NM_019187	29309	0.10	1.254	0.411756
A_44_P309613	XM_234852	XM_234852		0.10	1.254	0.157711
A_44_P440719	Ly9_predicted	XM_222892	289227	0.10	1.254	0.3343
A_44_P227194	TC560062	TC560062		0.10	1.254	0.540257
A_44_P405877	RGD1563342_predicted	XM_222254	304572	0.10	1.254	0.237455
A_44_P404064	Tiparp_predicted	XM_227217		0.10	1.254	0.189528
A_44_P156468	Pik3ca	XM_001059350		0.10	1.254	0.192707
A_44_P465868	Dhrs4	NM_153315	266686	0.10	1.254	0.056486
A_44_P545838	Arhgap4	NM_144740	246249	0.10	1.254	0.04927
A_44_P377712	XM_345562	XM_345562		0.10	1.253	0.127617
A_42_P603585	Atg12	NM_001038495	361321	0.10	1.253	0.235715
A_44_P513360	Cdkn3_predicted	XM_214152		0.10	1.253	0.14646
A_44_P163783	A_44_P163783	A_44_P163783		0.10	1.253	0.31285
A_44_P215393	RGD1306327	NM_001025129	361018	0.10	1.253	0.391163
A_44_P1018246	Xpa_predicted	XM_216403		0.10	1.253	0.316445
A_44_P992468	RGD1563971_predicted	XM_344066		0.10	1.253	0.058835
A_44_P358216	Pank4	NM_133531	171053	0.10	1.253	0.191967
A_44_P362714	Bach1_predicted	XM_221712		0.10	1.253	0.040632
A_43_P13113	Slc36a1	NM_130415	155205	0.10	1.253	0.079777
A_44_P267595	Eml4_predicted	XM_233839		0.10	1.253	0.173817
A_44_P254855	Usp33	XM_001080019		0.10	1.253	0.297799
A_44_P112149	Tnks2_predicted	XM_220047		0.10	1.253	0.213828
A_44_P464176	Cdan1_predicted	XM_230494		0.10	1.253	0.055767
A_44_P397763	Nolc1	NM_022869	64896	0.10	1.252	0.237507
A_44_P124592	Cyb561_predicted	XM_221030		0.10	1.252	0.302735
A_44_P303742	AW143088	AW143088	64823	0.10	1.252	0.125781
A_44_P651672	ENSRNOT00000044547	ENSRNOT00000044547		0.10	1.252	0.208715
A_44_P151422	Znf14	NM_153731	266773	0.10	1.252	0.307612
A_44_P427200	Opa1	NM_133585	171116	0.10	1.252	0.119622
A_43_P14109	AW143330	AW143330		0.10	1.252	0.067002
A_44_P988511	Csrp2bp_predicted	XM_001054440		0.10	1.252	0.372136

A_44_P110348	RGD1562236_predicted	XM_235349	315019	0.10	1.251	0.148895
A_44_P414757	Sec3l1	NM_001024770	305287	0.10	1.251	0.2841
A_44_P353553	Glo1	NM_207594	294320	0.10	1.251	0.138769
A_44_P792621	Tmem63a_predicted	XM_223000	289318	0.10	1.251	0.233341
A_44_P356615	RGD1307723_predicted	XM_232902		0.10	1.251	0.400607
A_43_P12328	Ireb2	NM_022863	64831	0.10	1.251	0.153389
A_44_P152557	AI170603	AI170603	288920	0.10	1.251	0.077683
A_44_P355842	Ly6g5c	NM_198739	294245	0.10	1.251	0.359547
A_44_P680974	CO387403	CO387403		0.10	1.251	0.47388
A_44_P607259	LOC501665	NM_001024368	501665	0.10	1.251	0.073784
A_44_P329925	Vps37b_predicted	XM_213769		0.10	1.251	0.114645
A_43_P18691	RGD1311162_predicted	XM_222770		0.10	1.250	0.156722
A_44_P384083	Nyw1	XM_001069191		0.10	1.250	0.127617
A_44_P386936	Gpr89_predicted	XM_342301	362003	0.10	1.250	0.042099
A_44_P450460	RGD1311362	NM_001025024	362914	0.10	1.250	0.068113
A_43_P11610	Alad	NM_012899	25374	0.10	1.250	0.119332
A_43_P15012	TC556885	TC556885		0.10	1.250	0.120187
A_44_P286573	AA955833	AA955833		0.10	1.250	0.187111
A_42_P801027	TC562846	TC562846		0.10	1.250	0.092872
A_44_P1042535	AI236795	AI236795	301252	0.10	1.250	0.488073
A_44_P519097	AW917872	AW917872	24675	0.10	1.250	0.1355
A_44_P281328	Upf3a	NM_001012159	361176	0.10	1.250	0.072294
A_44_P487425	RGD1566097_predicted	XM_001067026		0.10	1.250	0.130992
A_44_P542672	MGC124992	XM_001073185		0.10	1.250	0.159383
A_44_P781549	TC567801	TC567801		0.10	1.250	0.372136
A_44_P658508	DV723824	DV723824		0.10	1.250	0.210706
A_43_P19290	BF551243	BF551243		0.10	1.250	0.094126
A_44_P318323	LOC286989	NM_173323	286989	0.10	1.250	0.590963
A_42_P552004	Tnfrsf4	NM_013049	25572	0.10	1.250	0.363031
A_44_P1020482	Vgcnl1	NM_153630	266760	0.10	1.250	0.251102
A_44_P931625	DV725773	DV725773		0.10	1.249	0.133803
A_44_P848951	AW915522	AW915522		0.10	1.249	0.129455
A_44_P549028	Ank2	XM_001076082		0.10	1.249	0.271506
A_44_P494779	XM_234397	XM_234397		0.10	1.249	0.149522
A_42_P606963	RGD1308637	NM_001024885	308820	0.10	1.249	0.152963
A_44_P836445	Aph1a	NM_001014255	365872	0.10	1.249	0.07874
A_43_P12006	Gja9	NM_019281	50564	0.10	1.249	0.194698
A_44_P638264	TC522472	TC522472		0.10	1.249	0.214851
A_44_P638995	TC560244	TC560244		0.10	1.249	0.184338
A_44_P335165	RGD1564560_predicted	XM_001067324		0.10	1.249	0.052228
A_44_P429379	RGD1564268_predicted	XM_235399		0.10	1.249	0.064144
A_44_P779207	TC550311	TC550311		0.10	1.249	0.3101
A_44_P500431	Lman1	NM_053886	116666	0.10	1.249	0.22552
A_44_P379699	Rapgef1	XM_216018	63881	0.10	1.249	0.037089
A_44_P412972	Dgcr14	NM_001012472	360741	0.10	1.249	0.04953
A_44_P856083	A_44_P856083	A_44_P856083		0.10	1.249	0.236096
A_44_P129608	RGD1305793	XM_001067947		0.10	1.249	0.107937
A_44_P555019	Cdc42	NM_171994	64465	0.10	1.248	0.088186
A_44_P403065	LOC364560	XM_001053364		0.10	1.248	0.086879
A_43_P18674	Tmem39a	NM_001013865	288092	0.10	1.248	0.054452
A_44_P1037328	Pias1_predicted	XM_217188		0.10	1.248	0.038625
A_44_P380397	RGD1306356	XM_221195	303740	0.10	1.248	0.138329
A_44_P283686	LOC691036	XM_001076611	691036	0.10	1.248	0.110692
A_43_P16811	RGD1305486	NM_001014073	313873	0.10	1.248	0.068316
A_44_P165929	A_44_P165929	A_44_P165929		0.10	1.248	0.128451
A_44_P330633	A_44_P330633	A_44_P330633		0.10	1.248	0.094457
A_44_P807660	Rcor1_predicted	XM_001071532		0.10	1.248	0.166537
A_44_P250900	XM_233984	XM_233984		0.10	1.248	0.148613
A_44_P107662	Ythdf3_predicted	XM_342217		0.10	1.248	0.098356
A_44_P1016799	LOC362683	XM_001053592	362683	0.10	1.248	0.157548
A_44_P1019726	Leng9_predicted	XM_214812	292596	0.10	1.247	0.053965
A_44_P776000	LOC684978	XM_001061754		0.10	1.247	0.042879
A_44_P696669	Ehd1	XM_001074569	293692	0.10	1.247	0.046354

A_43_P17108	Srrm1_predicted	XM_233556		0.10	1.247	0.104487
A_44_P944294	LOC499617	XM_574942		0.10	1.247	0.075524
A_44_P579705	TC537938	TC537938		0.10	1.247	0.06481
A_44_P222475	RGD1308127	NM_001014248	365493	0.10	1.247	0.139843
A_44_P454872	RGD1308009	XM_001056829		0.10	1.247	0.15132
A_44_P420710	LOC500295	XM_575644		0.10	1.247	0.166939
A_43_P17496	Rbm18_predicted	XM_231176		0.10	1.247	0.089475
A_43_P23479	RGD1309571	NM_001013989	305240	0.10	1.247	0.099905
A_43_P11359	AW921109	AW921109		0.10	1.246	0.177844
A_44_P102189	RGD1309656_predicted	XM_223500	305386	0.10	1.246	0.315193
A_44_P1037493	Tssc4	NM_001013194	361682	0.10	1.246	0.473821
A_44_P144788	XM_226839	XM_226839		0.10	1.246	0.244214
A_44_P995959	Htf9c	NM_001011895	287953	0.10	1.246	0.091591
A_43_P17694	Cnot3_predicted	XM_218187		0.10	1.246	0.045552
A_44_P349675	Wasf2	NM_001013167	313024	0.10	1.246	0.10928
A_42_P509876	RGD1563482_predicted	XM_573395		0.10	1.246	0.193195
A_44_P1051965	D123	NM_053877	116656	0.10	1.246	0.041038
A_43_P20951	Anapc1_predicted	XM_230589		0.10	1.246	0.045652
A_44_P401097	Zfp105	NM_001012128	316096	0.10	1.246	0.033866
A_44_P698996	LOC685605	XM_001064507		0.10	1.246	0.184822
A_44_P131358	AW523568	AW523568		0.10	1.246	0.182397
A_43_P19709	RGD1565055_predicted	XM_343266	362938	0.10	1.245	0.064535
A_44_P871223	TC562367	TC562367		0.10	1.245	0.054263
A_44_P996204	Ncoa1_predicted	XM_233944		0.10	1.245	0.433314
A_44_P461304	Dnajb11	NM_001015021	360734	0.10	1.245	0.107251
A_44_P677737	CB812382	CB812382		0.10	1.245	0.08552
A_42_P768799	Rabac1	NM_031774	83583	0.10	1.245	0.091377
A_44_P441135	LOC690422	XM_001072932	690422	0.09	1.244	0.070326
A_42_P573118	RGD1309594	NM_001008351	309681	0.09	1.244	0.041093
A_44_P791968	TC521842	TC521842		0.09	1.244	0.122437
A_44_P353520	Hes2	NM_019236	29567	0.09	1.244	0.264886
A_44_P715962	TC543168	TC543168		0.09	1.244	0.204272
A_44_P432965	Fdps	NM_031840	83791	0.09	1.244	0.139598
A_44_P252997	LOC305913	XM_224241	305913	0.09	1.244	0.174005
A_44_P226928	Pdzk3	NM_022940	65034	0.09	1.244	0.153466
A_42_P486964	Rph3al	NM_133591	171123	0.09	1.244	0.352119
A_44_P394164	RGD1561191_predicted	XM_238505	299979	0.09	1.244	0.199818
A_44_P369617	AA945152	AA945152		0.09	1.244	0.658596
A_44_P242305	Ahctf1_predicted	XM_341161	682398	0.09	1.244	0.080292
A_44_P482629	Pcsk2	NM_012746	25121	0.09	1.244	0.258629
A_44_P621913	BM392119	BM392119		0.09	1.244	0.145773
A_44_P546217	RGD1559904_predicted	XM_232783		0.09	1.244	0.134375
A_43_P12642	Olr226	NM_031710	65140	0.09	1.244	0.130251
A_44_P494326	Gcn1l1_predicted	XM_001080241		0.09	1.244	0.084106
A_44_P138057	Rpl13	NM_031101	81765	0.09	1.244	0.112431
A_44_P304752	RGD1564764_predicted	XM_226367		0.09	1.244	0.17647
A_42_P499546	Hrasls5	NM_001039007	293711	0.09	1.244	0.235927
A_44_P848759	AA900063	AA900063		0.09	1.243	0.271895
A_44_P478360	RGD1563568_predicted	XM_229143	317589	0.09	1.243	0.09212
A_42_P555884	RGD1561481_predicted	XM_577055		0.09	1.243	0.26888
A_44_P349233	XM_222188	XM_222188		0.09	1.243	0.123124
A_44_P1020467	RGD1309385_predicted	XM_340760	360488	0.09	1.243	0.048928
A_44_P380329	A_44_P380329	A_44_P380329		0.09	1.243	0.113246
A_44_P661802	Eif2a	XM_578017		0.09	1.243	0.075074
A_44_P887061	BC107438	BC107438		0.09	1.243	0.137468
A_44_P267444	LOC296117	NM_001013919	296117	0.09	1.243	0.213339
A_43_P13114	Anxa7	NM_130416	155423	0.09	1.243	0.109369
A_43_P19315	Zcchc14_predicted	XM_344780	365018	0.09	1.243	0.069075
A_44_P180482	Tp53i11_predicted	XM_230296		0.09	1.243	0.122437
A_42_P574153	Ndel1	NM_133320	170845	0.09	1.243	0.086091
A_44_P216143	Luc7l	NM_001024269	360503	0.09	1.243	0.081089
A_44_P334788	Jup	NM_031047	81679	0.09	1.242	0.07486
A_44_P1027005	Calm3	NM_012518	24244	0.09	1.242	0.112345

A_43_P19684	Ptch1	NM_053566	89830	0.09	1.242	0.39403
A_44_P312877	BF420507	BF420507	298914	0.09	1.242	0.41804
A_42_P514733	RGD1562232_predicted	XM_001080976		0.09	1.242	0.039561
A_44_P581903	AA900845	AA900845		0.09	1.242	0.145171
A_44_P274036	Kif5b	NM_057202	117550	0.09	1.242	0.149295
A_44_P118137	Ddhd1	NM_001033066	305816	0.09	1.242	0.105814
A_43_P20802	LOC680006	XM_001055286		0.09	1.242	0.068316
A_44_P363824	Brd1_predicted	XM_235552		0.09	1.242	0.114111
A_42_P492130	LOC363309	XM_343649	363309	0.09	1.242	0.067356
A_44_P156211	Itpkc	NM_178094	308451	0.09	1.242	0.115052
A_43_P15942	Cpa3	XM_342219	54242	0.09	1.242	0.529908
A_44_P288498	Kbtbd4_predicted	XM_230307		0.09	1.242	0.051205
A_44_P874636	AA850319	AA850319	299811	0.09	1.242	0.270922
A_44_P330908	LOC312863	NM_001014061	312863	0.09	1.241	0.166416
A_44_P1034522	Impad1	AY321340		0.09	1.241	0.133186
A_44_P361809	Mmp2	NM_031054	81686	0.09	1.241	0.184487
A_44_P316374	LOC289673	XR_007662	289673	0.09	1.241	0.134707
A_44_P1045515	Sipa1l1	NM_139330	246212	0.09	1.241	0.131165
A_44_P168800	Pdik1l_predicted	XM_233567		0.09	1.241	0.146877
A_44_P353900	XM_222900	XM_222900		0.09	1.241	0.338522
A_42_P839033	RGD1311298_predicted	XM_213532	287840	0.09	1.241	0.096577
A_44_P543283	Plk3	XM_342888	58936	0.09	1.241	0.092939
A_44_P1025952	XM_214875	XM_214875		0.09	1.241	0.13664
A_44_P447222	LOC687014	XM_001076731		0.09	1.241	0.099879
A_44_P219470	Snx17	NM_001011981	298836	0.09	1.241	0.045385
A_44_P533992	Sfrs15	XM_001073074		0.09	1.241	0.052103
A_43_P19339	Msl2l1_predicted	XM_236567	315959	0.09	1.241	0.110493
A_43_P12212	Cap1	NM_022383	64185	0.09	1.240	0.15705
A_44_P173675	Chmp6_predicted	XM_237816		0.09	1.240	0.169941
A_44_P300100	LOC680424	XM_001057106		0.09	1.240	0.049487
A_44_P251384	Hist1h2ba	NM_022643	24829	0.09	1.240	0.307766
A_44_P229430	Aggf1	XM_226709	310005	0.09	1.240	0.144422
A_44_P236628	Casc1_predicted	XM_216310		0.09	1.240	0.213922
A_44_P1041142	Phax	NM_173133	286917	0.09	1.240	0.153572
A_44_P506039	Tsc1	NM_021854	60445	0.09	1.240	0.363436
A_44_P1019639	Gnai3	NM_013106	25643	0.09	1.240	0.119406
A_43_P21101	RGD1562559_predicted	XM_227201	310460	0.09	1.240	0.261573
A_44_P271989	Zfr	XM_345169	365703	0.09	1.240	0.050951
A_44_P399933	LOC689988	XM_001072817		0.09	1.240	0.225612
A_44_P318103	LOC291411	XM_225706		0.09	1.240	0.111789
A_44_P489811	LOC306096	XM_001073748		0.09	1.240	0.104751
A_44_P282164	Pdia2_predicted	XM_213263		0.09	1.240	0.2584
A_42_P567527	Traf4_predicted	XM_220640		0.09	1.239	0.064566
A_44_P190200	Chd3	XM_220602	303241	0.09	1.239	0.179157
A_44_P123994	Dstn	NM_001033666	502674	0.09	1.239	0.267761
A_44_P309441	Ncoa5_predicted	XM_215931		0.09	1.239	0.082677
A_43_P10139	CB547604	CB547604		0.09	1.239	0.218095
A_43_P19826	BE126320	BE126320		0.09	1.239	0.122615
A_44_P801044	LOC501194	NM_001025775	501194	0.09	1.239	0.156383
A_44_P575300	XM_213991	XM_213991		0.09	1.239	0.099985
A_44_P928964	RGD1566050_predicted	XM_574765	499442	0.09	1.239	0.378612
A_44_P346318	RGD1309054_predicted	XM_214707	292082	0.09	1.239	0.165106
A_44_P109628	Mss4	NM_001007678	304807	0.09	1.239	0.220314
A_44_P478319	RGD1565451_predicted	XM_233279	298322	0.09	1.239	0.123033
A_44_P407533	XM_221864	XM_221864		0.09	1.239	0.127183
A_44_P638891	TC542987	TC542987		0.09	1.239	0.103985
A_44_P1038238	C12orf10	NM_001005545	300258	0.09	1.238	0.040217
A_44_P201603	Hnrpa2b1_predicted	XM_342684		0.09	1.238	0.159067
A_44_P945781	LOC682723	XM_001063051		0.09	1.238	0.39137
A_44_P492030	Tnfrsf11a	XM_001056466	304695	0.09	1.238	0.050863
A_44_P1017662	Acsl1	NM_012820	25288	0.09	1.238	0.108015
A_42_P514244	Dyrk1a	NM_012791	25255	0.09	1.238	0.14614
A_43_P11770	G6pdx	NM_017006	24377	0.09	1.238	0.141093

A_44_P313988	Peo1_predicted	XM_219939		0.09	1.238	0.041224
A_44_P725429	AA946126	AA946126	361888	0.09	1.238	0.457078
A_43_P13791	AA818985	AA818985		0.09	1.238	0.18148
A_44_P149954	RGD1562289_predicted	XM_576917		0.09	1.238	0.075881
A_44_P124772	Ascc3_predicted	XM_228345		0.09	1.238	0.104185
A_44_P671605	Glis2_predicted	XM_220169		0.09	1.238	0.289646
A_44_P541438	RGD1308699	NM_001014032	309790	0.09	1.238	0.378007
A_44_P671234	TC532920	TC532920		0.09	1.237	0.221918
A_44_P461932	XM_228533	XM_228533		0.09	1.237	0.068148
A_44_P809536	AW144200	AW144200	306009	0.09	1.237	0.282697
A_44_P137279	Dpyd	NM_031027	81656	0.09	1.237	0.627537
A_44_P553105	Spin	NM_001024796	361217	0.09	1.237	0.11761
A_44_P279329	Abcc10_predicted	XM_236930		0.09	1.237	0.184889
A_43_P16915	RGD1308290_predicted	XM_341121		0.09	1.237	0.163479
A_44_P456689	Fgfr4	XM_344570		0.09	1.237	0.058835
A_44_P161846	Hist3h2ba_predicted	XM_220506		0.09	1.236	0.200834
A_44_P546574	Ankrd32_predicted	XM_001059377		0.09	1.236	0.204444
A_44_P344519	LOC679577	XM_001053564	304302	0.09	1.236	0.106299
A_44_P475218	Pip5k2c	NM_080480	140607	0.09	1.236	0.097369
A_44_P538725	Ppil4_predicted	XM_341727		0.09	1.236	0.154776
A_44_P204648	AB040488	AB040488		0.09	1.236	0.355585
A_44_P430581	RGD1309025_predicted	XM_215089		0.09	1.236	0.212555
A_44_P963309	TC557496	TC557496		0.09	1.236	0.231732
A_44_P605838	ENSRNOT00000028232	ENSRNOT00000028232		0.09	1.236	0.203761
A_44_P588645	RGD1305633_predicted	XM_001071108		0.09	1.236	0.093795
A_42_P592306	AI237079	AI237079		0.09	1.236	0.113867
A_43_P10604	Ube2a	NM_001013933	298317	0.09	1.236	0.049173
A_44_P220305	XM_230540	XM_230540		0.09	1.236	0.145256
A_44_P419600	RGD1309263_predicted	XM_214574		0.09	1.235	0.278445
A_44_P977865	TC561006	TC561006		0.09	1.235	0.138159
A_44_P464428	Zfp647_predicted	XM_343279	362948	0.09	1.235	0.347175
A_44_P409950	Asb7_predicted	XM_344905		0.09	1.235	0.191097
A_44_P267128	Tmpo	NM_012887	25359	0.09	1.235	0.211459
A_44_P380392	RGD1306356	XM_221195	303740	0.09	1.235	0.070282
A_44_P419891	RGD1311433_predicted	XM_213210	287054	0.09	1.235	0.082055
A_44_P259329	Tlk1_predicted	XM_242032		0.09	1.235	0.130501
A_44_P387017	XM_345386	XM_345386		0.09	1.235	0.182248
A_44_P215620	LOC312502	XM_001073801		0.09	1.235	0.132039
A_43_P10887	Map1b	XM_215469		0.09	1.235	0.417211
A_44_P301248	RGD1309450_predicted	XM_001053322		0.09	1.235	0.41081
A_44_P999653	LOC690308	XM_001074043		0.09	1.235	0.060013
A_42_P455886	RGD1308430_predicted	XM_001054512		0.09	1.235	0.07711
A_44_P518176	RGD1307673_predicted	XM_214448	291034	0.09	1.235	0.41177
A_42_P714804	Hdgfrp2	NM_133548	171073	0.09	1.235	0.147956
A_43_P15436	Ep300	XM_576312	170915	0.09	1.234	0.207375
A_43_P16810	CB546156	CB546156	406230	0.09	1.234	0.084928
A_43_P22177	Klhl18_predicted	XM_236647	316012	0.09	1.234	0.277247
A_44_P1050473	Rps5	XM_218293		0.09	1.234	0.107757
A_44_P475444	Cfh	AF436847	155012	0.09	1.234	0.409346
A_44_P558888	Crry	NM_001005330	54243	0.09	1.234	0.246479
A_44_P175549	LOC361016	NM_001014137	361016	0.09	1.234	0.243783
A_44_P140233	A_44_P140233	A_44_P140233		0.09	1.234	0.087001
A_44_P1014263	RGD1311457_predicted	XM_230510	311359	0.09	1.234	0.095582
A_44_P1022851	RGD1311164_predicted	XM_238378		0.09	1.234	0.277958
A_44_P213082	Aga	NM_001031641	290923	0.09	1.234	0.425898
A_44_P138301	Eppb9_predicted	XM_213314		0.09	1.234	0.096126
A_44_P621980	Calb1	NM_031984	83839	0.09	1.234	0.22326
A_44_P540969	Lkap	NM_133421	170946	0.09	1.234	0.186404
A_44_P331074	A_44_P331074	A_44_P331074		0.09	1.234	0.140423
A_43_P19783	RGD1359158	NM_001007737	361740	0.09	1.233	0.11571
A_44_P621366	ENSRNOT00000023228	ENSRNOT00000023228		0.09	1.233	0.07776
A_44_P330717	LOC294844	XM_001062661		0.09	1.233	0.134943
A_44_P487778	RGD1309138_predicted	XM_345669		0.09	1.233	0.054893

A_44_P129666	AI717054	AI717054		0.09	1.233	0.307001
A_44_P443479	Mns1	NM_001007752	363093	0.09	1.233	0.220948
A_44_P309224	XM_223612	XM_223612		0.09	1.233	0.217447
A_44_P1038946	Unr	NM_054006	117180	0.09	1.233	0.093694
A_44_P420131	Stx4a	NM_031125	81803	0.09	1.233	0.164523
A_44_P1037886	Sap18	NM_001033685	290284	0.09	1.233	0.127994
A_44_P497193	Senp8	NM_001012355	315723	0.09	1.233	0.146624
A_44_P393929	RGD1305121_predicted	NM_001039713	291905	0.09	1.233	0.053632
A_44_P1006624	LOC362683	XM_001053592	362683	0.09	1.233	0.282324
A_44_P115932	Zfp36l2	NM_001036626	298765	0.09	1.233	0.080017
A_44_P743637	Lrp12_predicted	XM_235261	314941	0.09	1.233	0.122437
A_44_P381881	Stat3	NM_012747	25125	0.09	1.233	0.214694
A_44_P898516	RGD1560638_predicted	XM_001069238		0.09	1.232	0.157573
A_44_P321115	Commd8_predicted	XM_214033		0.09	1.232	0.13199
A_44_P757324	DV714070	DV714070		0.09	1.232	0.127784
A_44_P972745	BF563201	BF563201		0.09	1.232	0.473939
A_44_P778851	TC563964	TC563964		0.09	1.232	0.294302
A_44_P774368	Abl1_mapped	XM_231137	311860	0.09	1.232	0.058099
A_44_P513602	ENSRNOT00000034802	ENSRNOT00000034802		0.09	1.232	0.127535
A_43_P17784	Cdk10	NM_001025722	361434	0.09	1.232	0.082571
A_44_P128435	RGD1307934_predicted	XM_219870	293953	0.09	1.232	0.361066
A_44_P270330	CA338808	CA338808		0.09	1.232	0.43732
A_44_P433349	Cdk5rap2	XM_575844	286919	0.09	1.231	0.242204
A_42_P729693	Synj2bp	NM_022599	64531	0.09	1.231	0.165419
A_44_P896219	AI071511	AI071511		0.09	1.231	0.27467
A_44_P258008	Fgd2_predicted	XM_228047		0.09	1.231	0.524369
A_44_P690844	BF404441	BF404441		0.09	1.231	0.212414
A_44_P137978	RGD1308872_predicted	XM_214620	291787	0.09	1.231	0.069492
A_44_P132598	Smagp	NM_182817	300236	0.09	1.231	0.195038
A_44_P181721	LOC680066	XM_001055542	680066	0.09	1.231	0.373912
A_42_P621628	LOC310665	NM_001025118	310665	0.09	1.230	0.247188
A_44_P487627	Zfp217_predicted	XM_231017		0.09	1.230	0.154977
A_44_P932190	RGD1561296_predicted	XM_575607	362399	0.09	1.230	0.153761
A_44_P205796	Cetn3	XM_342168	170895	0.09	1.230	0.197844
A_44_P288785	A_44_P288785	A_44_P288785		0.09	1.230	0.08765
A_44_P489961	XM_230747	XM_230747		0.09	1.230	0.077074
A_44_P276778	Trp53bp1_predicted	XM_215812		0.09	1.230	0.056915
A_44_P251804	Ppp2cb	NM_017040	24673	0.09	1.230	0.131454
A_44_P161525	RGD1310199	NM_001017446	291737	0.09	1.230	0.175724
A_44_P276784	Catsper2	NM_001012220	366174	0.09	1.230	0.120267
A_44_P300127	Plekhj1	XM_234909		0.09	1.230	0.133737
A_42_P711741	Capzb	NM_001005903	298584	0.09	1.230	0.113867
A_43_P11166	RGD1307279	NM_001008343	307210	0.09	1.230	0.22789
A_42_P708819	Thpo	NM_031133	81811	0.09	1.230	0.132003
A_44_P272266	RGD1562076_predicted	XM_577060		0.09	1.230	0.125822
A_44_P127238	RGD1562228_predicted	XM_574502	499214	0.09	1.230	0.108614
A_44_P406825	Phc2	XM_232751	313038	0.09	1.229	0.056497
A_44_P356436	XM_345249	XM_345249		0.09	1.229	0.098138
A_44_P345371	BQ199734	BQ199734		0.09	1.229	0.08446
A_44_P306884	A_44_P306884	A_44_P306884		0.09	1.229	0.133312
A_44_P363156	Hnrpd	NM_024404	79256	0.09	1.229	0.148866
A_44_P960156	Usp32_predicted	XM_001081109		0.09	1.229	0.294047
A_44_P480519	Prpf3_predicted	XM_342295		0.09	1.229	0.201175
A_44_P225129	XM_219766	XM_219766		0.09	1.229	0.126983
A_44_P153060	AI103782	AI103782	25508	0.09	1.229	0.207974
A_44_P425884	Egln1	NM_178334	308913	0.09	1.229	0.086958
A_44_P455091	RGD1308076	NM_001025277	300687	0.09	1.229	0.105423
A_43_P20612	RGD1305986_predicted	XM_341092	360819	0.09	1.229	0.091466
A_44_P297119	Ppp2r1a	NM_057140	117281	0.09	1.229	0.118891
A_44_P520869	RGD1305506_predicted	XM_213249	287178	0.09	1.228	0.195127
A_44_P809389	TC529771	TC529771		0.09	1.228	0.408397
A_44_P215479	RGD1310671_predicted	XM_225150	306734	0.09	1.228	0.310595
A_44_P340143	Commd9	NM_001033692	295956	0.09	1.228	0.071062

A_42_P634910	Rab4b	NM_017355	50866	0.09	1.228	0.072501
A_44_P1018684	LOC498351	XM_001066751		0.09	1.228	0.121225
A_44_P234378	XM_228586	XM_228586		0.09	1.228	0.101174
A_42_P728278	Mrpl2	NM_001034136	301240	0.09	1.228	0.127617
A_43_P21550	Tbc1d2b	XM_236472		0.09	1.228	0.074882
A_43_P19039	RGD1560583_predicted	XM_342487		0.09	1.228	0.158565
A_44_P161602	Rab43	NM_001024331	500249	0.09	1.228	0.081142
A_44_P325439	Fbxw4_predicted	XM_219944		0.09	1.228	0.150177
A_42_P538868	Dvl1	NM_031820	83721	0.09	1.228	0.108258
A_44_P127624	AW915714	AW915714		0.09	1.227	0.058617
A_44_P976320	TC520958	TC520958		0.09	1.227	0.107505
A_44_P381848	Dnm1l	NM_053655	114114	0.09	1.227	0.071379
A_44_P446375	Atad2_predicted	XM_235326	314993	0.09	1.227	0.254211
A_44_P335594	Tek	XM_342863		0.09	1.227	0.171536
A_44_P241018	Ireb2	NM_022863	64831	0.09	1.227	0.205198
A_44_P258343	RGD1564487_predicted	XM_001067436		0.09	1.227	0.23089
A_44_P372602	LOC684609	XM_001071223		0.09	1.227	0.172233
A_44_P1070720	RGD1307394_predicted	XM_001081752		0.09	1.227	0.044903
A_44_P411635	Cnot4	NM_001037782	312227	0.09	1.227	0.062916
A_44_P501665	RGD1304842_predicted	XM_232266		0.09	1.227	0.095284
A_44_P137930	RGD1304822_predicted	XM_215698		0.09	1.227	0.213558
A_44_P173568	Pop1_predicted	XM_235391	315045	0.09	1.227	0.069608
A_44_P466688	LOC683416	XM_001065840		0.09	1.227	0.294087
A_44_P393049	BI287851	BI287851	361821	0.09	1.227	0.426603
A_44_P496884	XM_341491	XM_341491		0.09	1.227	0.099402
A_42_P548889	MGC108974	NM_001017513	499108	0.09	1.227	0.141477
A_44_P515821	LOC362156	NM_001039338	362156	0.09	1.227	0.324507
A_44_P517682	Tomm70a	NM_212519	304017	0.09	1.227	0.045495
A_44_P278985	Ippk	NM_001008556	306808	0.09	1.227	0.212903
A_44_P272006	XM_237965	XM_237965		0.09	1.227	0.265313
A_44_P271842	Zfp53_predicted	XM_218041		0.09	1.227	0.224421
A_44_P668555	TC538212	TC538212		0.09	1.227	0.375639
A_44_P303155	Agtpbp1_predicted	XM_001061502		0.09	1.226	0.177716
A_44_P391360	Zhx1	NM_133620	171159	0.09	1.226	0.370119
A_44_P436409	Wdtc1_predicted	XM_232730		0.09	1.226	0.19192
A_44_P558908	AW920092	AW920092		0.09	1.226	0.464131
A_44_P363039	Adi1	NM_199097	298934	0.09	1.226	0.1937
A_44_P466271	Sphk2	NM_001012066	308589	0.09	1.226	0.050896
A_44_P590671	LOC680938	XM_001059551		0.09	1.226	0.14566
A_44_P541548	Agtrap	NM_001007654	298646	0.09	1.226	0.099754
A_43_P14576	TC520249	TC520249		0.09	1.226	0.22426
A_43_P19143	Map2k4	NM_001030023	287398	0.09	1.226	0.116336
A_44_P110419	Chchd2	NM_001015019	316643	0.09	1.226	0.121134
A_44_P489244	Casc3	NM_147144	259170	0.09	1.226	0.092929
A_44_P507427	AW142895	AW142895		0.09	1.226	0.465911
A_44_P461343	Rara	NM_031528	24705	0.09	1.225	0.17921
A_44_P282318	AA819333	AA819333	140665	0.09	1.225	0.133312
A_44_P761751	TC556789	TC556789		0.09	1.225	0.23292
A_43_P14115	Lap3	NM_001011910	289668	0.09	1.225	0.115762
A_42_P545405	AW917672	AW917672		0.09	1.225	0.134389
A_44_P324108	Usp49_predicted	XM_236919	316211	0.09	1.225	0.184844
A_42_P642507	LOC288526	XM_237868	288526	0.09	1.225	0.099704
A_44_P236418	Gfm2	XM_226707	294672	0.09	1.225	0.25529
A_44_P791927	TC555896	TC555896		0.09	1.225	0.240563
A_44_P429781	Mak10	NM_133324	64472	0.09	1.225	0.184658
A_44_P506224	A_44_P506224	A_44_P506224		0.09	1.225	0.217337
A_43_P16150	Znf291	XM_343394	117521	0.09	1.225	0.095175
A_43_P19140	CB548253	CB548253	688018	0.09	1.225	0.116336
A_44_P205923	RGD1564085_predicted	XM_218062	308265	0.09	1.225	0.111904
A_42_P781594	LOC360760	XM_001068449		0.09	1.225	0.238039
A_44_P1004536	Bdh1	NM_053995	117099	0.09	1.225	0.430859
A_42_P477449	Fbxl17_predicted	XM_001071661		0.09	1.225	0.272656
A_43_P22641	Mesp1_predicted	XM_218826		0.09	1.225	0.208913

A_43_P20800	Amigo	NM_206881	295365	0.09	1.224	0.422484
A_44_P557872	LOC296165	XM_001059497		0.09	1.224	0.094845
A_43_P19656	RGD1562836_predicted	XM_220372	303100	0.09	1.224	0.196152
A_44_P745115	TC523252	TC523252		0.09	1.224	0.116856
A_44_P1003229	XM_216763	XM_216763		0.09	1.224	0.151781
A_44_P824046	TC558562	TC558562		0.09	1.224	0.341541
A_44_P494521	Sec24b_predicted	XM_215706		0.09	1.224	0.373263
A_44_P510857	RGD1311663_predicted	XM_218208	292548	0.09	1.224	0.203396
A_44_P317520	Atp6ap2	XM_217592	302526	0.09	1.224	0.182998
A_44_P407668	LOC683012	XM_001064095		0.09	1.224	0.13972
A_44_P755961	LOC684112	XM_001068984		0.09	1.224	0.069102
A_44_P207634	LOC361309	NM_001014148	361309	0.09	1.224	0.176979
A_44_P576902	TC557101	TC557101		0.09	1.224	0.290873
A_44_P478479	XM_236187	XM_236187		0.09	1.224	0.12228
A_44_P161574	Mocs3_predicted	XM_230874		0.09	1.224	0.259999
A_44_P253345	RGD1563583_predicted	XR_007629	299208	0.09	1.224	0.288148
A_44_P1050929	LOC300284	NM_001013951	300284	0.09	1.224	0.282525
A_44_P295669	Rbm27_predicted	XM_341605		0.09	1.223	0.108871
A_44_P407132	Twist2	NM_021691	59327	0.09	1.223	0.10836
A_44_P511214	LOC682205	XM_001060401		0.09	1.223	0.244955
A_42_P508358	RGD1304977_predicted	XM_001077983		0.09	1.223	0.118434
A_42_P769180	Hint1_predicted	XM_001074494		0.09	1.223	0.079288
A_44_P337606	Zfp91	XM_001079228		0.09	1.223	0.165059
A_43_P18581	LOC367994	XM_347159		0.09	1.223	0.207974
A_44_P355480	LOC682679	XM_001062593		0.09	1.223	0.161228
A_44_P930730	TC523659	TC523659		0.09	1.223	0.286821
A_44_P438399	Osbp2_predicted	XM_223556		0.09	1.223	0.247627
A_44_P205887	Rab14	NM_053589	94197	0.09	1.223	0.237382
A_44_P538831	Mcart1	NM_001024785	313241	0.09	1.223	0.208413
A_44_P484902	Pitpna	NM_017231	29525	0.09	1.223	0.313938
A_44_P326078	LOC361153	XM_341438	361153	0.09	1.223	0.143513
A_42_P523688	RGD1304580	NM_001009283	292781	0.09	1.223	0.508837
A_44_P461794	RGD1311501_predicted	XM_216020		0.09	1.223	0.190376
A_44_P394150	LOC300491	XR_007645	300491	0.09	1.222	0.229762
A_44_P192530	RGD1565081_predicted	XM_001076974		0.09	1.222	0.267174
A_44_P774203	Birc6_predicted	XM_001065439		0.09	1.222	0.437567
A_44_P656317	TC559396	TC559396		0.09	1.222	0.443683
A_44_P349702	XM_238465	XM_238465		0.09	1.222	0.125575
A_44_P543917	Fbxo42_predicted	XM_342963		0.09	1.222	0.226929
A_44_P434859	BC090353	BC090353		0.09	1.222	0.325132
A_44_P172941	Pxk	NM_182821	306203	0.09	1.222	0.201419
A_44_P321716	AA955932	AA955932		0.09	1.222	0.235806
A_44_P224696	Zhx2	XM_235318	314988	0.09	1.222	0.120819
A_43_P19210	LOC361420	NM_001014155	361420	0.09	1.222	0.306276
A_42_P607215	Znf498_predicted	XM_344076		0.09	1.222	0.070389
A_44_P288463	LOC685425	XM_001063750	685425	0.09	1.222	0.11898
A_44_P1000203	Mrpl44	NM_001031650	301552	0.09	1.222	0.087594
A_42_P791342	RGD1562788_predicted	XM_237474	301665	0.09	1.222	0.206795
A_44_P777003	TC540329	TC540329		0.09	1.222	0.308365
A_44_P999889	LOC313618	NM_001025699	313618	0.09	1.222	0.116702
A_42_P585328	Xrcc1	NM_053435	84495	0.09	1.222	0.239476
A_44_P450173	Man1a2_predicted	XM_227543		0.09	1.222	0.087786
A_44_P248133	Tagln2	NM_001013127	304983	0.09	1.221	0.167819
A_44_P348853	Zfp99	NM_145724	252860	0.09	1.221	0.14327
A_44_P228351	Cd47	NM_019195	29364	0.09	1.221	0.125311
A_44_P431629	Bnip2_predicted	XM_217191		0.09	1.221	0.222878
A_43_P23126	Tcfcp2l2	NM_001037354	313994	0.09	1.221	0.152763
A_43_P18031	Tm9sf1	NM_001012155	361043	0.09	1.221	0.170803
A_44_P339202	LOC499076	XM_574359	499076	0.09	1.221	0.122861
A_43_P16636	Dhdds	NM_001011978	298541	0.09	1.221	0.197967
A_44_P700230	TC541304	TC541304		0.09	1.221	0.542881
A_44_P763861	TC532911	TC532911		0.09	1.221	0.236624
A_44_P309160	LOC304860	NM_001013984	304860	0.09	1.221	0.211738

A_43_P10933	LOC500855	XM_576251	500855	0.09	1.221	0.107181
A_44_P490253	RGD1562489_predicted	XM_220370		0.09	1.221	0.174506
A_44_P592077	TC522757	TC522757		0.09	1.221	0.338465
A_43_P13270	Cdk105	NM_134415	171456	0.09	1.221	0.118688
A_43_P11333	Hyou1	NM_138867	192235	0.09	1.221	0.251223
A_44_P159400	XM_234370	XM_234370		0.09	1.220	0.119518
A_44_P524981	Galnt1	NM_024373	79214	0.09	1.220	0.25069
A_44_P255997	CB544471	CB544471		0.09	1.220	0.040751
A_44_P929423	LOC687154	XM_001077270		0.09	1.220	0.315157
A_44_P700638	TC556985	TC556985		0.09	1.220	0.594981
A_44_P529939	Kif3b_predicted	XM_215883		0.09	1.220	0.257434
A_44_P977206	TC526713	TC526713		0.09	1.220	0.118557
A_44_P154245	LOC360932	XM_341209	360932	0.09	1.220	0.29192
A_44_P506693	RGD1359242	NM_001014219	363255	0.09	1.220	0.059323
A_44_P101211	Mcl1	NM_021846	60430	0.09	1.220	0.130992
A_43_P11836	Drd3	NM_017140	29238	0.09	1.220	0.366977
A_44_P168482	Mrpl52_predicted	XM_341312		0.09	1.220	0.058146
A_44_P994692	Zap70_mapped	NM_001012002	301348	0.09	1.220	0.639725
A_44_P539475	CB545813	CB545813		0.09	1.220	0.177333
A_44_P422080	Alms1_predicted	XM_216189		0.09	1.220	0.065841
A_44_P327417	BF556301	BF556301		0.09	1.220	0.324063
A_44_P207252	RGD1304825_predicted	XM_216186		0.09	1.220	0.206383
A_44_P1036481	RGD1566064_predicted	XM_341141	360865	0.09	1.220	0.132934
A_44_P103114	RGD1304595_predicted	XM_233020		0.09	1.220	0.149476
A_44_P1031638	RGD1309863	NM_001037189	303346	0.09	1.220	0.086945
A_44_P140351	RGD1311267	NM_001039024	311429	0.09	1.220	0.18458
A_43_P17743	Pir	NM_001009474	363465	0.09	1.220	0.224421
A_44_P597380	BF564712	BF564712		0.09	1.220	0.423575
A_44_P372759	RGD1305755	NM_001039336	305923	0.09	1.219	0.115077
A_44_P515577	Samd4b	XM_218384		0.09	1.219	0.095605
A_44_P518479	RGD1308555	NM_001013954	300866	0.09	1.219	0.21762
A_42_P540933	Csk	NM_001030039	315707	0.09	1.219	0.095313
A_43_P12569	Idh1	NM_031510	24479	0.09	1.219	0.205424
A_44_P683655	Arf3	NM_080904	140940	0.09	1.219	0.088352
A_44_P374620	Cdkn1c	NM_182735	246060	0.09	1.219	0.175492
A_44_P417264	RGD1564796_predicted	XM_345455	366229	0.09	1.219	0.313026
A_44_P338999	DV727635	DV727635	302913	0.09	1.219	0.1211
A_43_P17683	XM_243623	XM_243623		0.09	1.219	0.057362
A_44_P293014	A_44_P293014	A_44_P293014		0.09	1.219	0.300078
A_44_P141910	Spg7	NM_181388	353231	0.09	1.219	0.107505
A_44_P722210	TC562469	TC562469		0.09	1.219	0.348584
A_43_P11989	Gjb3	NM_019240	29585	0.09	1.219	0.07206
A_44_P269697	RGD1561543_predicted	XM_227463		0.09	1.219	0.346203
A_44_P513500	XM_230155	XM_230155		0.09	1.219	0.314711
A_43_P12401	Mepe	NM_024142	79110	0.09	1.219	0.299147
A_44_P821613	A_44_P821613	A_44_P821613		0.09	1.219	0.181743
A_44_P930113	TC539191	TC539191		0.09	1.219	0.138561
A_43_P10797	BG666794	BG666794	295701	0.09	1.219	0.210938
A_44_P1017536	Ddx24	NM_199119	373065	0.09	1.219	0.106566
A_44_P838368	TC539690	TC539690		0.09	1.218	0.145212
A_43_P17082	Dnajb1_predicted	XM_341663		0.09	1.218	0.290484
A_44_P317259	Eif4g3_predicted	XM_216563		0.09	1.218	0.147353
A_44_P421927	RGD1306230	NM_001037655	365902	0.09	1.218	0.223309
A_44_P961210	TC524576	TC524576		0.09	1.218	0.114645
A_44_P288578	Ptk9l_predicted	XM_228196		0.09	1.218	0.323845
A_44_P453337	LOC502603	NM_001035255	502603	0.09	1.218	0.241821
A_44_P424635	Zfpn1a3_predicted	XM_220921		0.09	1.218	0.33787
A_43_P15667	MGC105830	NM_001008370	1E+08	0.09	1.218	0.180703
A_44_P567198	AI136316	AI136316		0.09	1.218	0.106299
A_44_P1031813	RGD1309308_predicted	XM_232850	313115	0.09	1.218	0.288173
A_44_P618535	Plekhf2_predicted	XM_342803		0.09	1.218	0.154058
A_44_P239345	BE349699	BE349699	298436	0.09	1.218	0.334549
A_44_P685605	TC544700	TC544700		0.09	1.218	0.382457

A_43_P17667	Txndc11_predicted	XM_220122	302899	0.09	1.218	0.083859
A_43_P12887	Flt4	NM_053652	114110	0.09	1.218	0.371665
A_44_P1018913	Znrf1_predicted	XM_342692		0.09	1.218	0.322784
A_44_P227429	RGD1564957_predicted	XM_233240	313413	0.09	1.217	0.277038
A_44_P452486	NIPBL	XM_238213		0.09	1.217	0.191587
A_44_P361001	RGD1305824_predicted	XM_341978		0.09	1.217	0.182222
A_44_P498846	RGD1562348_predicted	XM_214012		0.09	1.217	0.148425
A_44_P233916	Mynn	NM_001012178	361924	0.09	1.217	0.326075
A_44_P276501	Ppp4r1	NM_080907	140943	0.09	1.217	0.173511
A_44_P433803	RGD1311037	NM_001014258	366196	0.09	1.217	0.176484
A_44_P400893	RGD1307778	NM_001014054	312135	0.09	1.217	0.237417
A_44_P819884	AW142995	AW142995		0.09	1.217	0.19091
A_44_P318661	Mthfr_predicted	XM_342975	362657	0.09	1.217	0.341411
A_44_P252921	Sec23ip	XM_001080274	309010	0.09	1.217	0.161618
A_44_P438520	XM_345331	XM_345331		0.09	1.217	0.201195
A_44_P465880	RGD735194	NM_199389	303183	0.09	1.217	0.319422
A_44_P140332	RGD1359600	NM_001007688	307343	0.09	1.217	0.248321
A_44_P590984	ENSRNOT00000037810	ENSRNOT00000037810		0.09	1.217	0.198242
A_44_P648613	BF543295	BF543295		0.09	1.217	0.312455
A_44_P495689	Stk16	NM_173142	286927	0.09	1.217	0.131976
A_44_P501973	AA925062	AA925062		0.09	1.217	0.316827
A_44_P342245	RGD1565602_predicted	XM_239761		0.09	1.217	0.204827
A_44_P140250	Hfe2	NM_001012080	310681	0.09	1.217	0.304215
A_44_P214218	RGD1565793_predicted	XM_576382	500972	0.09	1.217	0.239944
A_44_P313708	ENSRNOT00000001004	ENSRNOT00000001004		0.09	1.216	0.110493
A_44_P929020	ENSRNOT00000029014	ENSRNOT00000029014		0.08	1.216	0.19449
A_44_P527491	RGD1309765_predicted	XM_224848	290746	0.08	1.216	0.164812
A_44_P440686	Map3k10	XM_218368	308463	0.08	1.216	0.258826
A_42_P518818	Dbt	XM_342328		0.08	1.216	0.070466
A_44_P274433	RGD1562289_predicted	XM_576917		0.08	1.216	0.189714
A_43_P20674	Btbd15	NM_001034942	363035	0.08	1.216	0.180743
A_44_P515257	Cdc91l1	NM_181637	353304	0.08	1.216	0.332007
A_44_P303890	Tbx2_predicted	XM_220810		0.08	1.215	0.129769
A_44_P168564	RGD1305386	NM_001039609	310839	0.08	1.215	0.082571
A_43_P16415	Ndufv1	NM_001006972	293655	0.08	1.215	0.133152
A_44_P361091	LOC499624	XM_574948	499624	0.08	1.215	0.196977
A_44_P325376	LOC499391	XM_574706		0.08	1.215	0.113498
A_44_P898105	A_44_P898105	A_44_P898105		0.08	1.215	0.111963
A_44_P533137	Lancl2_predicted	XM_216158		0.08	1.215	0.333043
A_44_P283224	Slc45a1	NM_144747	246258	0.08	1.215	0.278258
A_44_P208491	Card6_predicted	XM_226804		0.08	1.215	0.118573
A_44_P356596	RGD1304592_predicted	XM_342783	362461	0.08	1.215	0.141592
A_44_P413979	Rab10	NM_017359	50993	0.08	1.215	0.128071
A_44_P323843	Tomm34_predicted	XM_230832		0.08	1.215	0.117515
A_44_P591761	TC555779	TC555779		0.08	1.215	0.172503
A_44_P1046554	Ubqln1	NM_053747	114590	0.08	1.215	0.164501
A_44_P440558	Wfs1	NM_031823	83725	0.08	1.215	0.079863
A_44_P285915	RGD1563824_predicted	XM_574313	499020	0.08	1.215	0.197576
A_44_P1043576	RGD1559895_predicted	XM_223974	305845	0.08	1.215	0.057983
A_44_P379280	XM_216889	XM_216889		0.08	1.215	0.17857
A_43_P16545	RGD1559786	NM_001034132	298384	0.08	1.215	0.119478
A_43_P22034	RGD1560158_predicted	XM_342191	361894	0.08	1.215	0.226445
A_43_P14983	TC551503	TC551503		0.08	1.215	0.387471
A_44_P695391	AW917152	AW917152	361160	0.08	1.215	0.302924
A_42_P762924	Herc1_predicted	XM_236362	315771	0.08	1.214	0.183162
A_44_P402479	Senp2	NM_023989	78973	0.08	1.214	0.200293
A_44_P121160	Tpi1	NM_022922	24849	0.08	1.214	0.421812
A_44_P928527	LOC365045	XR_008697	365045	0.08	1.214	0.142988
A_44_P1053063	Phpt1_predicted	XM_216013		0.08	1.214	0.170702
A_44_P552977	Ttc9c	NM_001007693	309196	0.08	1.214	0.058086
A_44_P109383	DN931087	DN931087		0.08	1.214	0.376802
A_43_P14694	DV724759	DV724759	25744	0.08	1.214	0.131165
A_44_P438508	Mrpl36_predicted	XM_344560		0.08	1.214	0.06511

A_43_P11566	Dspp	NM_012790	25254	0.08	1.214	0.117226
A_44_P522701	Adcyap1	NM_016989	24166	0.08	1.214	0.09262
A_44_P320737	LOC314323	NM_199109	314323	0.08	1.214	0.239944
A_44_P577496	Ophn1_predicted	XM_001068634		0.08	1.214	0.367322
A_44_P180041	Nme6	XM_343488	58964	0.08	1.214	0.154737
A_44_P119713	AW535026	AW535026	307545	0.08	1.214	0.293985
A_44_P217462	U78138	U78138		0.08	1.213	0.129329
A_44_P165295	RGD1310571	NM_001014147	361301	0.08	1.213	0.074254
A_43_P11307	Impdh1_predicted	XM_342650		0.08	1.213	0.163905
A_44_P110966	Gpr176	XM_342493	117257	0.08	1.213	0.41289
A_44_P382632	Tmprss7_predicted	XM_221464		0.08	1.213	0.491574
A_44_P388650	Camk4	NM_012727	25050	0.08	1.213	0.408825
A_44_P352123	LOC363198	NM_001014215	363198	0.08	1.213	0.350194
A_44_P404161	BQ202728	BQ202728	288257	0.08	1.213	0.33131
A_44_P745191	Prkacb_predicted	XM_215070		0.08	1.213	0.289935
A_44_P246435	CA506658	CA506658	301575	0.08	1.213	0.321523
A_44_P584408	TC541474	TC541474		0.08	1.213	0.365958
A_44_P236588	Trpm2	NM_001011559	294329	0.08	1.213	0.33047
A_44_P607177	LOC294513	NM_001013907	294513	0.08	1.213	0.160818
A_44_P445289	Dnah10	XM_001071882	117252	0.08	1.213	0.309183
A_44_P466211	Tceb3	NM_017103	25562	0.08	1.213	0.333245
A_44_P362498	AW143788	AW143788		0.08	1.212	0.368215
A_44_P351714	RGD1560032_predicted	XM_224186		0.08	1.212	0.122868
A_44_P370722	Mlh3_predicted	XM_234420		0.08	1.212	0.419415
A_44_P671449	TC517114	TC517114		0.08	1.212	0.062298
A_44_P324042	Srpr	NM_001034150	315548	0.08	1.212	0.177949
A_43_P18684	Ric8a	XM_215118	293614	0.08	1.212	0.104069
A_44_P213470	Zfp13_predicted	XM_220197		0.08	1.212	0.245863
A_43_P18174	Mbnl3_predicted	XM_228685		0.08	1.212	0.271223
A_44_P240552	AW919083	AW919083		0.08	1.212	0.526242
A_44_P393422	Leprotl1	NM_001013188	361160	0.08	1.212	0.184658
A_43_P11100	RGD1559786	NM_001034132	298384	0.08	1.212	0.085892
A_44_P745881	TC556244	TC556244		0.08	1.212	0.15995
A_44_P301067	Tmem23	NM_181386	353229	0.08	1.212	0.337795
A_44_P198112	March5_predicted	XM_215286		0.08	1.211	0.226578
A_42_P763522	LOC304138	NM_001013980	304138	0.08	1.211	0.42457
A_44_P289277	AW915944	AW915944		0.08	1.211	0.250338
A_42_P503768	Gemin6	NM_001009466	362688	0.08	1.211	0.117599
A_44_P260170	Bcl2l12_predicted	XM_341850		0.08	1.211	0.068543
A_44_P766609	A_44_P766609	A_44_P766609		0.08	1.211	0.513468
A_42_P716283	Zmym1_predicted	XM_233535		0.08	1.211	0.354182
A_43_P12075	Cnr2	NM_020543	57302	0.08	1.211	0.337753
A_44_P358082	BI289649	BI289649	310749	0.08	1.211	0.084056
A_43_P12973	Frag1	NM_053895	116675	0.08	1.211	0.148895
A_44_P471901	Rod1	NM_031346	83515	0.08	1.211	0.133803
A_44_P490206	Eif1b_predicted	XM_217294		0.08	1.211	0.235648
A_44_P543813	Ascc3_predicted	XM_228345		0.08	1.211	0.186199
A_44_P382006	RGD1560774_predicted	XM_222162	304479	0.08	1.211	0.124078
A_43_P11151	CA506387	CA506387	307810	0.08	1.210	0.102257
A_42_P586256	Prim1	NM_001008768	246327	0.08	1.210	0.125473
A_44_P500880	App	NM_019288	54226	0.08	1.210	0.244122
A_44_P931264	BF567278	BF567278		0.08	1.210	0.461139
A_44_P366056	Mmachc_predicted	XM_233418		0.08	1.210	0.179348
A_44_P323664	RGD1559682_predicted	XM_341363		0.08	1.210	0.24011
A_44_P496677	XM_222179	XM_222179		0.08	1.210	0.184409
A_44_P454180	Rab40c	NM_182675	359728	0.08	1.210	0.151255
A_42_P592157	Pbx2	NM_001002828	406164	0.08	1.210	0.136912
A_44_P153865	Olr379_predicted	NM_001000261	293812	0.08	1.210	0.163994
A_44_P417479	Ppm1h	NM_001013173		0.08	1.209	0.353499
A_44_P417051	Dcun1d3	NM_001024886	309035	0.08	1.209	0.08793
A_44_P419966	RGD1306926_predicted	XM_221197	303742	0.08	1.209	0.161648
A_44_P138041	RGD1306091_predicted	XM_226572	307950	0.08	1.209	0.408304
A_44_P333329	Rexo1	XM_576183	314630	0.08	1.209	0.144091

A_44_P279256	XM_345795	XM_345795		0.08	1.209	0.35765
A_44_P368101	Rbm17	NM_001013058	291295	0.08	1.209	0.111154
A_44_P213963	RGD1307890	NM_001037192	304851	0.08	1.209	0.190654
A_44_P529524	Pou2f1	XM_341148	171068	0.08	1.209	0.12676
A_44_P381837	Exoc3	NM_001024964	252881	0.08	1.209	0.241327
A_43_P16427	RGD1561001_predicted	XM_001081049		0.08	1.209	0.15329
A_44_P810032	TC526279	TC526279		0.08	1.209	0.091172
A_44_P192559	RGD1310553	NM_001008517	301374	0.08	1.209	0.28166
A_44_P262677	Hist2h3c2_predicted	XM_001062251		0.08	1.209	0.489409
A_44_P220197	Zfp364_predicted	XM_342300		0.08	1.208	0.14378
A_44_P991662	Kif1b	NM_057200	117548	0.08	1.208	0.103522
A_44_P419976	Tbx1_predicted	XM_341009		0.08	1.208	0.300334
A_44_P621119	LOC685485	XM_001063981		0.08	1.208	0.486665
A_44_P950330	AW525193	AW525193		0.08	1.208	0.289761
A_44_P157645	Iqgap1_predicted	XM_001066654		0.08	1.208	0.119627
A_44_P797917	A_44_P797917	A_44_P797917		0.08	1.208	0.687658
A_43_P15216	Dolpp1_predicted	XM_001079565		0.08	1.208	0.292011
A_44_P229102	Coro7	XM_220167	192276	0.08	1.208	0.090033
A_43_P13006	Arfrp1	NM_053980	117051	0.08	1.208	0.108885
A_44_P402070	AI029470	AI029470	54133	0.08	1.207	0.392831
A_44_P901113	TC562125	TC562125		0.08	1.207	0.313026
A_44_P773535	Gprc5b_predicted	XM_215095		0.08	1.207	0.589707
A_44_P208004	Tle4	NM_019141	25565	0.08	1.207	0.463823
A_44_P638620	DV717439	DV717439		0.08	1.207	0.353778
A_44_P492587	Eif5b	XM_218162		0.08	1.207	0.315628
A_44_P500820	BG666773	BG666773		0.08	1.207	0.227537
A_44_P294706	Tlr9	NM_198131	338457	0.08	1.207	0.252731
A_44_P490591	RGD1565847_predicted	XM_345906		0.08	1.207	0.290041
A_42_P520472	Kcng2	XM_001058122		0.08	1.207	0.143582
A_43_P18302	Phr1_predicted	XM_214245		0.08	1.207	0.232575
A_44_P110109	Cst3	NM_012837	25307	0.08	1.207	0.248327
A_44_P1048487	Zfyve9_predicted	XM_233341		0.08	1.207	0.515582
A_44_P413646	M23889	M23889		0.08	1.207	0.099473
A_44_P193915	Drb1	NM_153306	266631	0.08	1.207	0.183139
A_42_P552187	Mtdh	NM_133398	170910	0.08	1.206	0.103819
A_44_P141404	Arfgef1_predicted	XM_232614	312915	0.08	1.206	0.223916
A_42_P842367	Lrpprc	NM_001008519	313867	0.08	1.206	0.089232
A_43_P20943	Epc1_predicted	XM_225457		0.08	1.206	0.165787
A_44_P410455	Wdr48_predicted	XM_343503	363164	0.08	1.206	0.294431
A_44_P1004447	Arid2_predicted	XM_345867	366980	0.08	1.206	0.240581
A_43_P15804	Pou2f1	XM_001075635		0.08	1.206	0.2228
A_44_P149114	Atp6v1b2	NM_057213	117596	0.08	1.206	0.174653
A_44_P380018	XM_345404	XM_345404		0.08	1.206	0.353459
A_44_P243826	Adck5	XM_343273	362943	0.08	1.206	0.131263
A_44_P457436	Thrap6_predicted	XM_216917	299905	0.08	1.206	0.259101
A_44_P495648	Sec24b_predicted	XM_215706		0.08	1.206	0.073404
A_44_P790897	LOC679683	XM_001054118		0.08	1.206	0.118927
A_44_P548812	RGD1305553_predicted	XM_220236	302986	0.08	1.206	0.154999
A_44_P222924	XM_347115	XM_347115		0.08	1.206	0.479538
A_42_P606382	RGD1560544_predicted	XM_215181		0.08	1.206	0.177884
A_44_P142492	Ubqln4_predicted	XM_227405		0.08	1.205	0.146734
A_44_P214443	BQ194536	BQ194536	301226	0.08	1.205	0.225704
A_44_P645309	Pip5k1a	NM_001012743	309419	0.08	1.205	0.388959
A_43_P10248	Ublcp1	NM_001014117	360514	0.08	1.205	0.189867
A_42_P473425	Unc13d	NM_138844	192177	0.08	1.205	0.24308
A_44_P545958	ENSRNOT00000052276	ENSRNOT00000052276		0.08	1.205	0.120368
A_44_P229481	LOC681021	XM_001059976	681021	0.08	1.205	0.352392
A_43_P20717	Lars2_predicted	XM_343511		0.08	1.205	0.20643
A_42_P665106	Psmc4	NM_057122	117262	0.08	1.205	0.187222
A_44_P838436	LOC679007	XM_001054254		0.08	1.205	0.377245
A_43_P15517	Jund	NM_138875	24518	0.08	1.205	0.239118
A_44_P947558	TC531849	TC531849		0.08	1.204	0.477505
A_44_P103832	Hmgb1	NM_012963	25459	0.08	1.204	0.241289

A_44_P203416	Inpp4b	NM_053917	116699	0.08	1.204	0.300778
A_44_P669819	TC543467	TC543467		0.08	1.204	0.43952
A_44_P286467	RGD1309266_predicted	XM_237056	316335	0.08	1.204	0.145384
A_44_P622866	TC540414	TC540414		0.08	1.204	0.319359
A_44_P744309	RGD1560340_predicted	XM_222534	304719	0.08	1.204	0.156511
A_44_P898694	ENSRNOT00000046166	ENSRNOT00000046166		0.08	1.204	0.101258
A_44_P624391	TC545311	TC545311		0.08	1.204	0.285143
A_44_P161220	Dbccr1	NM_080482	140610	0.08	1.204	0.257644
A_44_P340121	RGD1565122_predicted	XM_229983	295654	0.08	1.204	0.201973
A_44_P223899	AW143174	AW143174		0.08	1.204	0.37374
A_44_P247644	Tmed7	XM_001063185		0.08	1.204	0.341317
A_44_P349336	Dlg5_predicted	XM_223768		0.08	1.204	0.139378
A_44_P999910	XM_223687	XM_223687		0.08	1.204	0.146089
A_43_P12215	Mafg	NM_022386	64188	0.08	1.204	0.104034
A_42_P626008	Traf4af1	NM_001004264	311325	0.08	1.204	0.336833
A_44_P652213	A_44_P652213	A_44_P652213		0.08	1.204	0.162546
A_44_P462306	BU671504	BU671504		0.08	1.204	0.116679
A_44_P232030	LOC297826	XM_001066983		0.08	1.204	0.281668
A_44_P1007841	XM_344042	XM_344042		0.08	1.204	0.215333
A_44_P717619	A_44_P717619	A_44_P717619		0.08	1.203	0.144776
A_44_P158378	AI639455	AI639455	315970	0.08	1.203	0.438395
A_43_P18523	Sfrs8	NM_001034924	304431	0.08	1.203	0.088782
A_44_P232851	Tomm20	BU946530	266601	0.08	1.203	0.350883
A_44_P713677	LOC501961	XR_008547	501961	0.08	1.203	0.193637
A_44_P524680	Trpc4	NM_080396	84494	0.08	1.203	0.274139
A_44_P1025802	Metap1_predicted	XM_215717		0.08	1.203	0.193195
A_44_P175837	XM_229646	XM_229646		0.08	1.203	0.095576
A_44_P398462	RGD1564781_predicted	XR_008189	291065	0.08	1.203	0.360711
A_44_P494788	XM_343347	XM_343347		0.08	1.203	0.113118
A_44_P100919	ENSRNOT00000007048	ENSRNOT00000007048		0.08	1.203	0.313294
A_44_P173199	Actr8_predicted	XM_341393		0.08	1.203	0.13845
A_44_P150841	March5_predicted	XM_215286		0.08	1.203	0.197418
A_44_P519963	Ppm1a	NM_017038	24666	0.08	1.203	0.195811
A_44_P151638	Cyp11b1	NM_012537	500892	0.08	1.203	0.267556
A_42_P726573	Npr2	NM_053838	116564	0.08	1.203	0.17857
A_44_P182042	AI176309	AI176309		0.08	1.203	0.073533
A_44_P264195	AW915009	AW915009		0.08	1.203	0.314872
A_43_P14764	Nrxn1	NM_021767	60391	0.08	1.203	0.476826
A_44_P217875	Dsp	XM_225259	306871	0.08	1.202	0.13068
A_43_P17233	Sars2_predicted	XM_214884		0.08	1.202	0.142914
A_44_P171031	RGD1311910_predicted	XM_001057895		0.08	1.202	0.161285
A_44_P123908	RGD1311435	XM_342338	362037	0.08	1.202	0.099251
A_43_P18148	Arhgef17_predicted	XM_218963		0.08	1.202	0.162291
A_44_P403978	Supt4h2_predicted	XM_213415		0.08	1.202	0.162504
A_44_P1034375	Ptgfrn	NM_019243	29602	0.08	1.202	0.208913
A_44_P110972	Gpr176	XM_342493	117257	0.08	1.202	0.309615
A_44_P498921	Ppig	NM_031793	83624	0.08	1.202	0.281201
A_44_P471233	RGD1560293_predicted	XM_229107	317578	0.08	1.202	0.363569
A_44_P471386	Dazap2	NM_001013107	300235	0.08	1.202	0.198549
A_44_P499433	Tssc1	NM_001012192	362721	0.08	1.202	0.107724
A_42_P639047	RGD1309350_predicted	XM_215112	293613	0.08	1.202	0.287832
A_43_P19411	Ppm1d_predicted	XM_213418		0.08	1.202	0.298327
A_44_P798989	TC560812	TC560812		0.08	1.201	0.310893
A_44_P229533	RGD1561337_predicted	XM_574397	499105	0.08	1.201	0.145559
A_44_P592915	BF558512	BF558512		0.08	1.201	0.13493
A_44_P1042163	RGD1310414	NM_001013883	290811	0.08	1.201	0.074708
A_44_P838165	TC539274	TC539274		0.08	1.201	0.138678
A_44_P200146	Oxr1	XM_576258	117520	0.08	1.201	0.177725
A_44_P478074	LOC289740	XM_214069		0.08	1.201	0.203793
A_44_P882995	RGD1561950_predicted	XM_573110	497923	0.08	1.201	0.512879
A_44_P104282	BI278232	BI278232	361722	0.08	1.201	0.229978
A_44_P651802	ENSRNOT00000028014	ENSRNOT00000028014		0.08	1.201	0.105599
A_44_P297217	Sirt2	NM_001008368	361532	0.08	1.201	0.178087

A_44_P153217	BF555980	BF555980		0.08	1.201	0.184822
A_44_P472500	Glud1	NM_012570	24399	0.08	1.201	0.223207
A_43_P12538	Pax8	NM_031141	81819	0.08	1.201	0.425485
A_44_P533560	RGD1565754_predicted	XM_574642	499335	0.08	1.201	0.092843
A_44_P314370	XM_235981	XM_235981		0.08	1.201	0.261269
A_44_P342397	LOC682744	XM_001062895		0.08	1.201	0.277357
A_43_P10966	Map2k1ip1	NM_001008375	362045	0.08	1.200	0.098793
A_43_P16965	RGD1310877_predicted	XM_223775	305667	0.08	1.200	0.301992
A_43_P22566	XM_222619	XM_222619		0.08	1.200	0.145786
A_44_P699332	LOC301128	XM_001057990		0.08	1.200	0.109009
A_44_P182479	Kcnk4	NM_053804	116489	0.08	1.200	0.641798
A_44_P998707	Dctn4	NM_053404	84428	0.08	1.200	0.06672
A_44_P111118	AW920965	AW920965	29144	0.08	1.200	0.130565
A_44_P942609	DV719627	DV719627		0.08	1.200	0.62805
A_44_P746137	LOC682864	XM_575744		0.08	1.200	0.143323
A_42_P793772	Klhl9_predicted	XM_233157		0.08	1.200	0.267579
A_44_P433641	RGD1304610_predicted	XM_223939		0.08	1.200	0.374084
A_42_P753429	Slc5a1	NM_013033	25552	0.08	1.200	0.150704
A_44_P276698	RGD1311265	NM_001014173	361976	0.08	1.200	0.152691
A_44_P102962	LOC291758	XM_001054025		0.08	1.199	0.379741
A_44_P370465	XM_214407	XM_214407		0.08	1.199	0.129142
A_43_P20647	Gatad2b	NM_001024888	310614	0.08	1.199	0.117354
A_44_P274376	Rfxank	NM_001013136	306353	0.08	1.199	0.218569
A_44_P524566	Actc1	NM_019183	29275	0.08	1.199	0.253996
A_44_P286259	Fbxl4_predicted	XM_232833		0.08	1.199	0.699597
A_42_P788740	Folr2_predicted	XM_215013		0.08	1.199	0.347688
A_44_P342550	Dysf_predicted	XM_232123		0.08	1.199	0.284272
A_44_P792368	TC523218	TC523218		0.08	1.199	0.165606
A_42_P686567	TC555302	TC555302		0.08	1.199	0.116867
A_44_P252220	Mocs2	NM_001007633	294753	0.08	1.199	0.391694
A_44_P745579	TC555636	TC555636		0.08	1.199	0.139672
A_44_P209926	RGD1561653_predicted	XM_343060	362736	0.08	1.199	0.333521
A_44_P138144	LOC302022	XM_001073811		0.08	1.198	0.274176
A_44_P142352	Dhx9_predicted	XM_239780		0.08	1.198	0.101305
A_43_P12679	Atp6ap1	NM_031785	83615	0.08	1.198	0.124781
A_44_P513687	XM_233941	XM_233941		0.08	1.198	0.144332
A_44_P271248	Cog8_predicted	XM_214673		0.08	1.198	0.266809
A_44_P416960	Vil1_predicted	XM_237288		0.08	1.198	0.418844
A_44_P823241	TC540819	TC540819		0.08	1.198	0.58629
A_44_P487753	Zmym4_predicted	XM_233529		0.08	1.198	0.103596
A_43_P19277	RGD1308747_predicted	XM_340836	360559	0.08	1.198	0.381885
A_43_P12143	Cyln2	NM_021997	29264	0.08	1.198	0.206795
A_44_P365835	RGD1305178	NM_001025003	311855	0.08	1.198	0.170986
A_44_P571899	BG667685	BG667685		0.08	1.198	0.252118
A_43_P13338	Ppp1r3b	NM_138912	192280	0.08	1.198	0.539533
A_44_P684932	TC541895	TC541895		0.08	1.197	0.130448
A_44_P252855	A_44_P252855	A_44_P252855		0.08	1.197	0.235527
A_44_P220083	Fbxl19_predicted	XM_219356		0.08	1.197	0.152656
A_44_P168103	Skp1a	NM_001007608	287280	0.08	1.197	0.122783
A_43_P12348	Dlgap1	NM_022946	65040	0.08	1.197	0.281318
A_42_P537443	Jund	NM_138875	24518	0.08	1.197	0.212094
A_44_P229618	Tmem1_predicted	XM_228065	309678	0.08	1.197	0.201346
A_44_P534508	Zfp212	XM_231749	297066	0.08	1.197	0.094752
A_44_P118734	Olr95_predicted	NM_001001024	405909	0.08	1.197	0.489469
A_44_P403482	RGD1311493_predicted	XM_213493	287734	0.08	1.197	0.310735
A_44_P524190	AW143108	AW143108	297968	0.08	1.197	0.179839
A_44_P163618	Mixl1_predicted	XM_222997		0.08	1.197	0.611999
A_44_P998072	TC517732	TC517732		0.08	1.197	0.210176
A_44_P1043145	Catna1	NM_001007145	307505	0.08	1.197	0.217298
A_44_P199008	RGD1305514_predicted	XM_342029		0.08	1.197	0.229858
A_44_P504274	RGD1307976_predicted	XM_217474		0.08	1.196	0.116681
A_44_P284642	RGD1311732	NM_001024866	289859	0.08	1.196	0.14476
A_44_P174365	AW142013	AW142013	498266	0.08	1.196	0.068548

A_44_P1044736	Wbp4	NM_053766	114765	0.08	1.196	0.302735
A_44_P156589	Ngef_predicted	XM_346854	246217	0.08	1.196	0.143054
A_44_P131125	LOC366551	XR_007527	366551	0.08	1.196	0.305517
A_44_P124225	MGC116373	NM_001025701	314949	0.08	1.196	0.094439
A_44_P1028017	Cript	NM_019907	56725	0.08	1.196	0.214744
A_44_P679254	AA894210	AA894210		0.08	1.196	0.185242
A_44_P149540	RGD1561704_predicted	XM_223051		0.08	1.196	0.152441
A_44_P915168	Zdhhc18	NM_001039339	362613	0.08	1.196	0.223081
A_44_P368116	RGD1562474_predicted	XM_215736	295602	0.08	1.196	0.312606
A_44_P363308	Ptprk	NM_001029902	360302	0.08	1.196	0.378938
A_44_P281155	Nek7_predicted	XM_341127		0.08	1.196	0.207736
A_44_P140684	Col8a1_predicted	XM_221536		0.08	1.196	0.137716
A_44_P168970	Zfp294	XM_001055727	288308	0.08	1.196	0.104812
A_44_P419822	RGD1310994	NM_001012470	314329	0.08	1.196	0.090157
A_44_P219402	Rps6ka5_predicted	XM_001064992		0.08	1.196	0.502381
A_43_P21799	RGD1310093_predicted	XM_220978		0.08	1.195	0.51278
A_43_P20228	Ankrd49_predicted	XM_235833	315434	0.08	1.195	0.116239
A_44_P388349	AW915388	AW915388		0.08	1.195	0.481485
A_42_P642575	Agmat	XM_216570		0.08	1.195	0.514538
A_44_P356503	RGD1562232_predicted	XM_575221	499878	0.08	1.195	0.13792
A_43_P16547	Rras_predicted	XM_341851		0.08	1.195	0.198878
A_44_P234087	LOC498358	XM_001057251		0.08	1.195	0.202536
A_44_P731165	TC543872	TC543872		0.08	1.195	0.306722
A_44_P277043	XM_343338	XM_343338		0.08	1.195	0.196897
A_44_P836742	RGD1559450_predicted	XM_573124	497937	0.08	1.195	0.171651
A_44_P170566	St8sia4	XM_346078		0.08	1.195	0.249327
A_44_P325525	Kcng3	NM_133426	171011	0.08	1.195	0.421244
A_44_P151804	Stat6_predicted	XM_343223		0.08	1.195	0.098805
A_43_P10813	RGD1310925_predicted	XM_342717		0.08	1.195	0.235398
A_43_P17534	CB546701	CB546701		0.08	1.195	0.213582
A_44_P500250	RGD1565253_predicted	XM_001073932		0.08	1.195	0.14639
A_44_P930417	DV720316	DV720316		0.08	1.195	0.135024
A_44_P405360	RGD1306437	NM_001009635	290303	0.08	1.195	0.261638
A_44_P403475	Slc43a2_predicted	XM_213381		0.08	1.195	0.454091
A_44_P366177	XM_343333	XM_343333		0.08	1.195	0.098354
A_44_P747006	DV728556	DV728556		0.08	1.194	0.4843
A_44_P520324	XM_213911	XM_213911		0.08	1.194	0.179719
A_44_P230733	Ube2e2	XM_341288		0.08	1.194	0.159801
A_42_P829251	Crebbp	NM_133381	54244	0.08	1.194	0.108877
A_43_P23200	Ptges2_predicted	XM_231144		0.08	1.194	0.190962
A_44_P387207	RGD1560665_predicted	XM_576033	500655	0.08	1.194	0.185848
A_44_P114851	Tmem8_predicted	XM_220264		0.08	1.194	0.289191
A_44_P499256	RGD1561740_predicted	XM_342486	362186	0.08	1.194	0.309011
A_44_P287442	AI071097	AI071097	170922	0.08	1.194	0.285842
A_44_P1007362	Ccdc5	NM_138864	192228	0.08	1.194	0.275546
A_44_P100023	Wrb	NM_199373	288233	0.08	1.194	0.099188
A_44_P109893	Zfp629	XM_219354		0.08	1.194	0.152137
A_44_P328311	LOC499261	XR_009310	499261	0.08	1.194	0.393384
A_43_P18895	Sorcs2_predicted	XM_223522		0.08	1.194	0.151226
A_44_P579054	TC560492	TC560492		0.08	1.194	0.16592
A_44_P884297	TC536728	TC536728		0.08	1.194	0.121147
A_44_P494112	Grb2	D49848	81504	0.08	1.194	0.122594
A_44_P257135	Slc29a3	NM_181639	353307	0.08	1.194	0.177595
A_43_P12206	Xylt2	NM_022296	64134	0.08	1.193	0.141592
A_44_P215530	Tnks1bp1_predicted	XM_215763	295707	0.08	1.193	0.108333
A_44_P553001	XM_215265	XM_215265		0.08	1.193	0.352776
A_44_P448307	Arpc2_predicted	XM_217432		0.08	1.193	0.239916
A_44_P378484	Gnas	L10326	24896	0.08	1.193	0.360252
A_42_P638128	Actg2	NM_012893	25365	0.08	1.193	0.232792
A_44_P262608	Bdp1_predicted	XM_226738		0.08	1.193	0.1625
A_44_P259119	RGD1306148_predicted	XM_232937	313196	0.08	1.193	0.498872
A_44_P581843	CO401680	CO401680		0.08	1.193	0.438379
A_44_P299659	Alkbh2_predicted	XM_222273	304578	0.08	1.193	0.308181

A_44_P130641	Txn2	NM_053331	79462	0.08	1.193	0.224971
A_44_P402675	Rxrb	NM_206849	361801	0.08	1.193	0.09044
A_43_P11978	Men1	NM_019208	29417	0.08	1.192	0.218166
A_44_P130263	Prok1	NM_138851	192205	0.08	1.192	0.176415
A_44_P511204	Ndufb11_predicted	XM_216785		0.08	1.192	0.248654
A_43_P13685	RGD1309696_predicted	XM_234572	314478	0.08	1.192	0.505391
A_44_P554698	Psme2	NM_017257	29614	0.08	1.192	0.192019
A_44_P619439	BF566748	BF566748		0.08	1.192	0.459522
A_44_P158467	BG666679	BG666679	360600	0.08	1.192	0.416737
A_44_P243619	XM_342569	XM_342569		0.08	1.192	0.13276
A_44_P313431	Thoc2_predicted	XM_233081	313308	0.08	1.192	0.122882
A_44_P485502	Slc24a4_predicted	XM_234470		0.08	1.192	0.241022
A_44_P348023	AW918397	AW918397	301007	0.08	1.192	0.25207
A_44_P210431	ENSRNOT00000007441	ENSRNOT00000007441		0.08	1.192	0.159148
A_44_P197124	Mtss1_predicted	XM_343248	362918	0.08	1.192	0.148505
A_43_P11260	TC551913	TC551913		0.08	1.191	0.281774
A_42_P595591	Amd1	NM_031011	81640	0.08	1.191	0.214732
A_44_P419123	Angpt2	XM_344544	89805	0.08	1.191	0.710264
A_44_P1059985	Spbc24_predicted	XM_343359	363028	0.08	1.191	0.337969
A_43_P12972	Sdc3	NM_053893	116673	0.08	1.191	0.217298
A_44_P283399	Cask	NM_022184	29647	0.08	1.191	0.326396
A_44_P304759	RGD1563459_predicted	XR_007322	292088	0.08	1.191	0.136148
A_44_P483390	BI290095	BI290095	24763	0.08	1.191	0.262975
A_44_P997815	Cxxc1	XM_238016		0.08	1.191	0.17567
A_44_P108102	XM_345483	XM_345483		0.08	1.191	0.100223
A_43_P12274	Lst1	NM_022634	64569	0.08	1.191	0.471806
A_44_P990522	Cit	AF039218	83620	0.08	1.191	0.286963
A_44_P546228	RGD1310893_predicted	XM_342832		0.08	1.191	0.142771
A_44_P1038397	DV727624	DV727624	497975	0.08	1.191	0.540553
A_44_P1044270	RGD1308492	NM_001033060	287463	0.08	1.191	0.108072
A_43_P13161	Prdm4	NM_133312	170820	0.08	1.191	0.111343
A_44_P301449	Pdcd6ip	XM_001076624		0.08	1.191	0.153466
A_44_P304842	Tle1_predicted	XM_342851	362533	0.08	1.191	0.266403
A_44_P248651	RGD1564452_predicted	XM_213214		0.08	1.191	0.12051
A_44_P229656	RGD1306576_predicted	XM_216375		0.08	1.190	0.144381
A_43_P12820	Pik3cb	NM_053481	85243	0.08	1.190	0.186235
A_43_P12150	Pik3r2	NM_022185	29741	0.08	1.190	0.373352
A_44_P294650	Brca1	NM_012514	497672	0.08	1.190	0.274485
A_44_P1023688	Ap2b1	NM_080583	140670	0.08	1.190	0.088525
A_44_P745253	TC517939	TC517939		0.08	1.190	0.162448
A_44_P884867	TC540725	TC540725		0.08	1.190	0.35958
A_44_P1054404	Ndufa2_predicted	XM_214570		0.08	1.190	0.155579
A_44_P549011	Apba2	NM_031780	83610	0.08	1.190	0.314281
A_44_P651794	LOC688708	XM_001068010		0.08	1.190	0.223261
A_43_P14511	TC517935	TC517935		0.08	1.190	0.266596
A_44_P131694	LOC361377	NM_001014152	361377	0.08	1.190	0.231102
A_44_P323993	LOC684551	XM_001070942		0.08	1.190	0.353251
A_43_P18218	Itga3_predicted	XM_340884		0.08	1.190	0.190408
A_44_P686919	TC557051	TC557051		0.08	1.190	0.357552
A_44_P881194	LOC679937	XM_001055011	679937	0.08	1.190	0.273428
A_44_P346901	Slc10a4	NM_001008555	305309	0.08	1.190	0.311737
A_44_P491929	Pprf18	NM_138523	171552	0.08	1.190	0.129329
A_44_P1009178	Mapkap1	NM_001011964	296648	0.08	1.189	0.13931
A_44_P656290	TC543567	TC543567		0.08	1.189	0.495313
A_44_P109817	Perq1_predicted	XM_222024		0.08	1.189	0.184163
A_44_P324482	LOC498295	NM_001025762	498295	0.08	1.189	0.233701
A_44_P510401	RGD1310147_predicted	XM_340793		0.08	1.189	0.131818
A_42_P633664	Niban	NM_022242	63912	0.08	1.189	0.260898
A_42_P638181	Tpo1	NM_133395	170907	0.08	1.189	0.189463
A_44_P154131	Man2c1	NM_139256	246136	0.08	1.189	0.22315
A_44_P164529	CB544995	CB544995	619436	0.08	1.189	0.145481
A_44_P367952	Atp8a1_predicted	XM_223390	289615	0.08	1.189	0.26073
A_44_P293667	Vps33b	NM_022286	64060	0.08	1.189	0.28414

A_44_P533572	AI639343	AI639343		0.08	1.189	0.218986
A_43_P15163	Stag2_predicted	XM_001059059		0.08	1.189	0.21523
A_44_P116443	Matr3	NM_019149	29150	0.07	1.188	0.247124
A_44_P549304	Il18bp	NM_053374	84388	0.07	1.188	0.267282
A_43_P10955	TC523349	TC523349		0.07	1.188	0.739144
A_44_P494828	LOC681572	XM_001057466		0.07	1.188	0.300579
A_43_P19234	LOC310946	XM_227795		0.07	1.188	0.26073
A_44_P562717	TC544937	TC544937		0.07	1.188	0.623825
A_44_P342586	LOC308650	NM_001014019	308650	0.07	1.188	0.109913
A_44_P773350	CO386862	CO386862		0.07	1.188	0.144742
A_44_P196699	Tbc1d10b_predicted	XM_344965		0.07	1.188	0.244035
A_44_P176342	BG663185	BG663185		0.07	1.188	0.394478
A_44_P561715	TC539851	TC539851		0.07	1.188	0.517345
A_44_P791351	CO400483	CO400483		0.07	1.188	0.299809
A_44_P1004037	Acly	NM_016987	24159	0.07	1.188	0.250058
A_44_P278509	RGD1303066	NM_212498	361789	0.07	1.188	0.319239
A_43_P11441	Atp4b	NM_012510	24217	0.07	1.188	0.261177
A_44_P484485	Fastk	NM_001011967	296741	0.07	1.187	0.175772
A_44_P193181	Gapdh	NM_017008	24383	0.07	1.187	0.224362
A_44_P1015495	Rsafd1_predicted	XM_222117		0.07	1.187	0.123925
A_44_P303867	RGD1306116_predicted	XM_342068	361774	0.07	1.187	0.440172
A_44_P223741	AW142694	AW142694	310324	0.07	1.187	0.457857
A_43_P13035	Sfrs10	NM_057119	117259	0.07	1.187	0.229762
A_44_P699301	TC535784	TC535784		0.07	1.187	0.134563
A_44_P105163	RGD1566072_predicted	XM_219424	309081	0.07	1.187	0.092355
A_44_P882921	A_44_P882921	A_44_P882921		0.07	1.187	0.153517
A_44_P142606	Papd5_predicted	XM_226334		0.07	1.187	0.273989
A_44_P458395	Fbxl5_predicted	XM_223508		0.07	1.187	0.318983
A_44_P363742	Slc4a1ap_predicted	XM_233883		0.07	1.186	0.187544
A_44_P339308	AI574843	AI574843		0.07	1.186	0.336566
A_44_P304057	Ppp2r2a	NM_053999	117104	0.07	1.186	0.314474
A_44_P438808	Zfp406_predicted	XM_343255	362925	0.07	1.186	0.308799
A_44_P286623	AA819657	AA819657		0.07	1.186	0.238912
A_43_P14611	Mccc1	NM_001009653	294972	0.07	1.186	0.306573
A_44_P340852	Rap1a	NM_001005765	295347	0.07	1.186	0.294285
A_44_P602391	BE101765	BE101765	408245	0.07	1.186	0.166977
A_44_P225407	AA799487	AA799487	294321	0.07	1.186	0.241387
A_44_P541082	A_44_P541082	A_44_P541082		0.07	1.186	0.117655
A_43_P10907	Acn9	XM_342641		0.07	1.186	0.237193
A_44_P120239	BG378709	BG378709	314961	0.07	1.186	0.309549
A_44_P576056	Ankfy1_predicted	XM_001080190		0.07	1.185	0.365749
A_44_P746024	TC522535	TC522535		0.07	1.185	0.194012
A_44_P1070538	Kit	AF296694		0.07	1.185	0.347569
A_44_P213803	AA925483	AA925483		0.07	1.185	0.28769
A_44_P361424	XM_235039	XM_235039		0.07	1.185	0.288867
A_44_P254687	Nthl1_predicted	XM_213228		0.07	1.185	0.179071
A_44_P319099	A_44_P319099	A_44_P319099		0.07	1.185	0.352417
A_44_P360620	Olr383_predicted	NM_001000262	293816	0.07	1.185	0.198887
A_44_P537103	LOC681314	XM_001061020		0.07	1.185	0.135661
A_44_P148407	Pdia6	NM_001004442	286906	0.07	1.185	0.394043
A_44_P495480	Fos	NM_022197	314322	0.07	1.185	0.507734
A_44_P346408	Egfr	NM_031507	24329	0.07	1.185	0.407459
A_44_P419379	Snrp70_predicted	XM_341857		0.07	1.185	0.241555
A_44_P861993	CO389165	CO389165		0.07	1.185	0.285103
A_44_P466852	RGD1560902_predicted	XM_343924		0.07	1.185	0.340934
A_44_P810151	TC536356	TC536356		0.07	1.185	0.187498
A_44_P447708	RGD1564022_predicted	XM_221987		0.07	1.185	0.286898
A_42_P603980	Wfdc2	NM_173109	286888	0.07	1.184	0.428847
A_44_P420552	Zcchc7_predicted	XM_216407		0.07	1.184	0.293714
A_44_P109887	Ccni_predicted	XM_214007		0.07	1.184	0.494272
A_44_P407750	RGD1559872_predicted	XM_225035	306622	0.07	1.184	0.46563
A_42_P651681	Rfrp	NM_023952	60570	0.07	1.184	0.35237
A_44_P150353	Mast2_predicted	XM_233782		0.07	1.184	0.360912

A_44_P185324	Ibrdc3_predicted	XM_233761		0.07	1.184	0.142392
A_44_P429364	Ptdss1	NM_001012113	314553	0.07	1.184	0.380384
A_44_P225159	RGD1562801_predicted	XR_009577	500627	0.07	1.184	0.259346
A_44_P157144	Recql4_predicted	XM_216973	300057	0.07	1.184	0.293234
A_44_P397851	Lyst	NM_053518	85419	0.07	1.184	0.17647
A_44_P226697	P4ha3	NM_198775	361612	0.07	1.184	0.290993
A_44_P974892	TC540109	TC540109		0.07	1.184	0.385896
A_44_P1050595	Ela1	NM_012552	24331	0.07	1.184	0.329398
A_44_P576215	TC520453	TC520453		0.07	1.184	0.13964
A_44_P652746	LOC687633	XM_001077967		0.07	1.184	0.115353
A_44_P808019	TC523509	TC523509		0.07	1.183	0.43575
A_44_P227089	XM_341986	XM_341986		0.07	1.183	0.210586
A_42_P579788	XM_230480	XM_230480		0.07	1.183	0.119275
A_44_P534695	Armc8_predicted	XM_236599	315949	0.07	1.183	0.31575
A_44_P651030	AW917535	AW917535		0.07	1.183	0.641644
A_44_P331276	Fhit	NM_021774	60398	0.07	1.183	0.706585
A_44_P623489	CF106785	CF106785		0.07	1.183	0.278572
A_44_P488467	Plekhf2_predicted	XM_342803		0.07	1.183	0.259716
A_44_P161831	Stub1	NM_001025625	287155	0.07	1.183	0.148231
A_44_P608187	Sema3g	XM_001062426		0.07	1.183	0.307215
A_44_P599767	BQ211218	BQ211218	315695	0.07	1.182	0.157939
A_44_P467942	Copg	NM_001031822	297428	0.07	1.182	0.290537
A_44_P1045680	LOC288010	NM_001013864	288010	0.07	1.182	0.245495
A_44_P837271	ENSRNOT00000044563	ENSRNOT00000044563		0.07	1.182	0.43391
A_44_P515679	Rab11fip2_predicted	XM_217660		0.07	1.182	0.157064
A_44_P424633	RGD1307981_predicted	XM_340880		0.07	1.182	0.305212
A_42_P606504	Stk35_predicted	XM_230598		0.07	1.182	0.106317
A_42_P736349	Ttc5	NM_001013131	305837	0.07	1.182	0.302775
A_43_P10089	Slc45a3_predicted	XM_001059048		0.07	1.182	0.769123
A_44_P318778	Trim34_predicted	XM_219045	293294	0.07	1.182	0.591992
A_44_P447497	LOC681759	XM_001057257		0.07	1.182	0.620454
A_43_P22149	Socs4_predicted	XM_223934		0.07	1.182	0.11906
A_44_P561667	TC556754	TC556754		0.07	1.182	0.221427
A_43_P10534	39874	NM_001034108	362849	0.07	1.182	0.161146
A_42_P652357	Cttn	NM_021868	60465	0.07	1.182	0.105373
A_44_P913530	A_44_P913530	A_44_P913530		0.07	1.182	0.164471
A_44_P996774	Eif4a2	NM_001008335	303831	0.07	1.182	0.218866
A_44_P487157	Rab6a	XM_001062702	84379	0.07	1.182	0.351975
A_44_P1000875	Ntel1	XM_001071735		0.07	1.182	0.258287
A_44_P128583	LOC501281	XM_576696	501281	0.07	1.182	0.265689
A_44_P412437	RGD1562225_predicted	XM_344999		0.07	1.182	0.21374
A_44_P387152	XM_229267	XM_229267		0.07	1.182	0.200291
A_44_P484870	RGD1359684	BC091200	290071	0.07	1.182	0.412487
A_44_P330168	Dnm1l	NM_053655	114114	0.07	1.182	0.127542
A_44_P441252	Slc5a10_predicted	XM_220540		0.07	1.182	0.40862
A_43_P10227	AW917579	AW917579	79249	0.07	1.182	0.145784
A_44_P369847	Keap1	NM_057152	117519	0.07	1.181	0.201298
A_44_P157229	Usp18	NM_001014058	312688	0.07	1.181	0.570519
A_43_P11601	Sod3	NM_012880	25352	0.07	1.181	0.430879
A_44_P201729	RGD1311815	NM_001034149	313668	0.07	1.181	0.097171
A_44_P883154	Matr3	NM_019149	29150	0.07	1.181	0.222172
A_44_P358336	Pla2g6	NM_001005560	360426	0.07	1.181	0.29274
A_43_P22190	Mnab_predicted	XM_231249		0.07	1.181	0.209456
A_44_P352538	Sectm1_predicted	NM_001013043	287885	0.07	1.181	0.179861
A_44_P990434	RGD1560964_predicted	XM_215220		0.07	1.181	0.157066
A_44_P142562	Tmx2	NM_001007643	295701	0.07	1.181	0.242166
A_44_P230720	Trib1	XM_001065461		0.07	1.181	0.269918
A_44_P298304	Secp43	NM_023027	65241	0.07	1.181	0.175165
A_44_P761228	DV714301	DV714301		0.07	1.181	0.155731
A_44_P558607	RGD1305464	NM_001025011		0.07	1.181	0.145559
A_44_P745407	TC537397	TC537397		0.07	1.181	0.563904
A_42_P674598	Rpn1	NM_013067	25596	0.07	1.181	0.176082
A_44_P1005540	Brd7_predicted	XM_341653		0.07	1.181	0.288362

A_44_P332928	RGD1306820_predicted	XM_219414		0.07	1.181	0.352239
A_44_P161389	Ndst2_predicted	XM_223793		0.07	1.181	0.193331
A_44_P282145	XM_220256	XM_220256		0.07	1.181	0.180743
A_44_P867316	LOC688452	XM_001060463	688452	0.07	1.181	0.170069
A_44_P468661	LOC688966	XM_001067949	688966	0.07	1.180	0.301803
A_44_P166426	XM_229703	XM_229703		0.07	1.180	0.078359
A_43_P16992	Auh_predicted	XM_341497		0.07	1.180	0.357165
A_43_P16489	Commd9	NM_001033692	295956	0.07	1.180	0.125343
A_44_P286528	Narf	NM_001039207	360681	0.07	1.180	0.462964
A_44_P506358	Trim44	NM_001013203	362172	0.07	1.180	0.170069
A_44_P522915	A_44_P522915	A_44_P522915		0.07	1.180	0.368808
A_44_P1043415	LOC290577	XR_006087	290577	0.07	1.180	0.312844
A_43_P18324	Arl10	NM_207165	306767	0.07	1.179	0.186953
A_44_P684922	Aff4_predicted	XM_220420		0.07	1.179	0.225059
A_44_P360510	Bhmt	NM_030850	81508	0.07	1.179	0.263262
A_43_P19130	LOC684663	XM_001071585		0.07	1.179	0.240668
A_43_P13142	Cgi94	NM_130825	170669	0.07	1.179	0.190375
A_43_P15840	Flt3	XM_221874	140635	0.07	1.179	0.115973
A_44_P326350	LOC366515	NM_001014262	366515	0.07	1.179	0.223919
A_44_P794598	Hnrpa3	NM_198132	362152	0.07	1.179	0.23498
A_44_P608966	TC563456	TC563456		0.07	1.179	0.22789
A_44_P237886	Vdac1	NM_031353	83529	0.07	1.179	0.226321
A_43_P16521	Tfg	NM_001012144	360709	0.07	1.179	0.2098
A_44_P405726	Midn_predicted	XM_234902	314623	0.07	1.179	0.283202
A_44_P372457	Hoxc10	XM_235698	315338	0.07	1.179	0.345617
A_44_P1044441	Phtf1	XM_342306	252962	0.07	1.179	0.235715
A_44_P975743	Midn_predicted	XM_001076784		0.07	1.179	0.14189
A_44_P116703	Erf_predicted	XM_218336	292721	0.07	1.179	0.170809
A_44_P367958	RGD1311490_predicted	XM_341980		0.07	1.179	0.290355
A_44_P119122	MGC124824	XM_573556		0.07	1.179	0.201121
A_44_P263060	Nubp2	NM_001011891	287125	0.07	1.178	0.28812
A_43_P21461	Zfp319_predicted	XM_226240	291849	0.07	1.178	0.225666
A_42_P692064	AW915467	AW915467		0.07	1.178	0.463474
A_43_P10477	BF555477	BF555477		0.07	1.178	0.269138
A_44_P163669	XM_217642	XM_217642		0.07	1.178	0.23477
A_44_P885009	Mtf2	XM_001056894		0.07	1.178	0.181301
A_44_P384462	RGD1565713_predicted	XM_224201		0.07	1.178	0.18638
A_44_P380575	Slc7a5	NM_017353	50719	0.07	1.178	0.282924
A_44_P243231	Nr2f6	NM_139113	245980	0.07	1.178	0.133608
A_44_P637827	Als2cr13_predicted	XM_343576		0.07	1.178	0.53543
A_43_P23165	Ophn1_predicted	XM_231453		0.07	1.178	0.278601
A_44_P605194	Ccdc49_predicted	XM_001081381		0.07	1.178	0.20513
A_44_P670239	Dusp18	NM_001013128	305477	0.07	1.178	0.149524
A_44_P538946	LOC365842	NM_001014254	365842	0.07	1.178	0.259966
A_44_P112570	XM_220134	XM_220134		0.07	1.178	0.231941
A_44_P264988	Trio	XM_226888		0.07	1.178	0.145823
A_44_P870253	TC544101	TC544101		0.07	1.178	0.319995
A_44_P613019	TC558588	TC558588		0.07	1.178	0.549621
A_44_P1034991	Rnf14	XM_001066493		0.07	1.177	0.2546
A_44_P145152	XM_234844	XM_234844		0.07	1.177	0.20206
A_43_P19640	Tnks1bp1_predicted	XM_215763	295707	0.07	1.177	0.223207
A_44_P536985	Rnf126	NM_001033702	314613	0.07	1.177	0.118306
A_44_P248411	XM_345108	XM_345108		0.07	1.177	0.506138
A_44_P148807	Scyl1	NM_001011938	293684	0.07	1.177	0.117424
A_44_P736182	TC542145	TC542145		0.07	1.177	0.518947
A_43_P17387	RGD1304924_predicted	XM_220231	302982	0.07	1.177	0.128213
A_44_P582118	AW914982	AW914982		0.07	1.177	0.242774
A_44_P126249	LOC684096	XM_001068917		0.07	1.177	0.246902
A_44_P315138	Prkrir_predicted	XM_218949	308845	0.07	1.177	0.131064
A_44_P334218	Lss	NM_031049	81681	0.07	1.177	0.119899
A_43_P13913	LOC684322	XM_001069861		0.07	1.177	0.122707
A_42_P669954	BF559364	BF559364		0.07	1.177	0.566665
A_44_P487468	Olfm4_predicted	XM_224413		0.07	1.177	0.859322

A_44_P1004470	Tm2d3_predicted	XM_214962		0.07	1.177	0.140379
A_44_P772369	RGD1308795_predicted	XM_236392	315804	0.07	1.177	0.376586
A_42_P594613	Dusp6	NM_053883	116663	0.07	1.177	0.561599
A_44_P808457	TC525716	TC525716		0.07	1.177	0.282316
A_44_P106037	Hnrpab	NM_031330	83498	0.07	1.177	0.194795
A_44_P388040	Pkd1	AF277452	24650	0.07	1.177	0.441127
A_43_P19508	RGD1565449_predicted	XM_340740	360464	0.07	1.176	0.164993
A_44_P655694	TC567670	TC567670		0.07	1.176	0.422864
A_44_P536076	LOC288750	NM_198727	288750	0.07	1.176	0.477952
A_43_P11997	Klrc2	NM_019261	29684	0.07	1.176	0.3442
A_44_P589393	AW144183	AW144183	301062	0.07	1.176	0.329398
A_43_P21050	Plxna3_mapped	XM_219723		0.07	1.176	0.175872
A_44_P248728	Polq_predicted	XM_221423		0.07	1.176	0.326007
A_44_P541074	RragB	NM_053972	117043	0.07	1.176	0.399421
A_44_P100870	RGD1308251_predicted	XM_342993		0.07	1.176	0.287444
A_44_P525110	XM_344922	XM_344922		0.07	1.176	0.128736
A_44_P853410	TC556101	TC556101		0.07	1.176	0.408171
A_44_P711618	AW527267	AW527267	362804	0.07	1.176	0.214744
A_44_P499479	RGD1311723_predicted	XM_001069542		0.07	1.176	0.293035
A_44_P315723	AA900371	AA900371		0.07	1.175	0.308709
A_44_P523304	LOC367289	XM_346058	367289	0.07	1.175	0.336778
A_44_P469188	Appbp2	XM_001081113		0.07	1.175	0.236146
A_44_P270056	RGD1309459	NM_001007800	360477	0.07	1.175	0.262788
A_43_P23457	RGD1311331_predicted	XM_233975	313950	0.07	1.175	0.143676
A_43_P20205	Ldb2_predicted	XM_214054		0.07	1.175	0.204741
A_44_P191013	AI231289	AI231289	315119	0.07	1.175	0.401755
A_44_P159296	Atp6v1h	NM_001013929	297797	0.07	1.175	0.291954
A_44_P344773	AABR03024136	AABR03024136		0.07	1.175	0.427371
A_44_P398230	Heca_predicted	XM_218660		0.07	1.175	0.401315
A_44_P457599	Foxj1	NM_053832	116557	0.07	1.175	0.293973
A_44_P180147	Sap2	XM_342209	266808	0.07	1.175	0.209373
A_44_P518226	XM_225838	XM_225838		0.07	1.175	0.195463
A_44_P328270	RGD1561854_predicted	XM_344144		0.07	1.175	0.177228
A_44_P252090	Rheb	NM_013216	26954	0.07	1.175	0.317025
A_44_P1022421	Hnrpd	NM_024404	79256	0.07	1.175	0.33352
A_44_P319425	AI710695	AI710695	64183	0.07	1.175	0.432636
A_44_P431516	Samd6	NM_001015028	362515	0.07	1.174	0.386355
A_44_P550423	Sqstm1	AF053095	113894	0.07	1.174	0.281866
A_44_P506124	XM_222590	XM_222590		0.07	1.174	0.09855
A_44_P652984	TC537996	TC537996		0.07	1.174	0.200339
A_42_P466997	XM_223622	XM_223622		0.07	1.174	0.103888
A_44_P321761	BF550393	BF550393		0.07	1.174	0.225132
A_44_P729822	TC555357	TC555357		0.07	1.174	0.242549
A_42_P755087	RGD1304686	NM_001008305	293673	0.07	1.174	0.236096
A_44_P493284	St13	NM_031122	81800	0.07	1.174	0.326099
A_44_P981926	TC560837	TC560837		0.07	1.174	0.510071
A_44_P206480	RGD1311223_predicted	XM_345971	367146	0.07	1.174	0.184091
A_44_P227483	RGD1566408_predicted	XM_575928		0.07	1.174	0.215684
A_44_P424723	Snf1lk	NM_021693	59329	0.07	1.173	0.396442
A_44_P105578	XM_233741	XM_233741		0.07	1.173	0.134183
A_44_P792633	RGD1311267	NM_001039024	311429	0.07	1.173	0.489109
A_42_P762905	Mrpl23	NM_022529	64360	0.07	1.173	0.215688
A_43_P13540	CB546013	CB546013		0.07	1.173	0.430088
A_44_P1003372	Smpdl3a	NM_001005539	294422	0.07	1.173	0.3963
A_43_P13220	Blcap	NM_133582	171113	0.07	1.173	0.181059
A_44_P534396	Wdr37_predicted	XM_225512		0.07	1.173	0.412747
A_44_P513428	Polr3c	NM_001012081	310685	0.07	1.173	0.188915
A_43_P18676	RGD1311624	XM_219358		0.07	1.173	0.132566
A_44_P324410	Tmem87a_predicted	XM_345422	366170	0.07	1.173	0.394676
A_44_P852623	A_44_P852623	A_44_P852623		0.07	1.173	0.265515
A_43_P21140	Cstf2t_predicted	XM_219809		0.07	1.173	0.337848
A_44_P489826	XM_226802	XM_226802		0.07	1.173	0.367718
A_44_P923554	A_44_P923554	A_44_P923554		0.07	1.173	0.334322

A_44_P165777	Bzrap1	XM_213427	287609	0.07	1.173	0.628584
A_44_P553374	Commd4_predicted	XM_343396		0.07	1.172	0.206039
A_44_P165336	BF550302	BF550302		0.07	1.172	0.13387
A_44_P239176	RGD1308959	NM_001008380	363077	0.07	1.172	0.233318
A_44_P678738	BF398611	BF398611		0.07	1.172	0.61524
A_42_P837576	Cbx1_predicted	XM_340885	360609	0.07	1.172	0.265893
A_44_P175167	RGD1305481	XM_215260		0.07	1.172	0.241351
A_43_P20319	RGD1305854_predicted	XM_231388	312046	0.07	1.172	0.250831
A_44_P400177	Atp1a3	NM_012506	24213	0.07	1.172	0.643918
A_44_P286853	Akt3	NM_031575	29414	0.07	1.172	0.370782
A_44_P546476	RGD1566176_predicted	XM_001060129		0.07	1.172	0.151036
A_44_P147477	RGD1564806_predicted	XM_228538	317237	0.07	1.172	0.415936
A_42_P675802	Olfm3	NM_145777	252920	0.07	1.172	0.400464
A_44_P809141	TC562260	TC562260		0.07	1.172	0.259368
A_44_P407894	RGD1306209	XM_231752	312303	0.07	1.172	0.178005
A_44_P940774	BM384514	BM384514		0.07	1.171	0.28365
A_44_P853909	TC557569	TC557569		0.07	1.171	0.440773
A_44_P532212	Chchd5_predicted	XM_215828		0.07	1.171	0.240875
A_44_P235165	Ankrd13	NM_001012148	360823	0.07	1.171	0.283384
A_43_P21124	Spast_predicted	XM_343018		0.07	1.171	0.188037
A_44_P313786	Cdtw1	NM_134395	171432	0.07	1.171	0.269144
A_44_P546432	LOC687643	XM_001079501		0.07	1.171	0.360252
A_42_P615837	Ckmt2	X59736		0.07	1.171	0.385534
A_44_P916433	TC562121	TC562121		0.07	1.171	0.4193
A_44_P271018	BG668512	BG668512	29287	0.07	1.171	0.118675
A_44_P365598	Rnf146	NM_001012060	308051	0.07	1.171	0.274416
A_44_P834809	AW144192	AW144192		0.07	1.171	0.437848
A_44_P370892	Tmem104_predicted	XM_221104	303670	0.07	1.171	0.232844
A_44_P581083	TC532172	TC532172		0.07	1.171	0.305159
A_44_P166244	Hspa12b_predicted	XM_230610		0.07	1.171	0.741248
A_44_P838021	BG662519	BG662519	114506	0.07	1.170	0.291825
A_44_P1028233	Anapc2	XM_215994	296558	0.07	1.170	0.32432
A_44_P566326	TC545093	TC545093		0.07	1.170	0.276363
A_43_P15212	Pole3	NM_001007652	298098	0.07	1.170	0.241746
A_44_P285759	Celsr2	XM_001070611		0.07	1.170	0.124672
A_44_P370146	Myo9b	NM_012984	25486	0.07	1.170	0.179348
A_44_P492255	Nr6a1	XM_342427	362125	0.07	1.170	0.333847
A_44_P550885	Hipk2_predicted	XM_342662		0.07	1.170	0.278928
A_43_P14144	Umpk_predicted	XM_222855		0.07	1.170	0.162394
A_43_P23129	BF551144	BF551144	360302	0.07	1.170	0.370038
A_44_P100466	XM_218590	XM_218590		0.07	1.170	0.262453
A_43_P18805	RGD1310066	XM_343221	362894	0.07	1.170	0.185424
A_44_P132523	Ndufs8_predicted	XM_215197		0.07	1.170	0.226123
A_44_P655406	TC565478	TC565478		0.07	1.170	0.742522
A_44_P542535	DV718172	DV718172	691155	0.07	1.169	0.180692
A_42_P479422	Traf7_predicted	XM_001055375		0.07	1.169	0.20977
A_44_P417317	Ddx47	NM_001015005	297685	0.07	1.169	0.487661
A_44_P962800	TC531775	TC531775		0.07	1.169	0.292547
A_44_P415880	Cd320	NM_001014201	362851	0.07	1.169	0.219327
A_44_P145970	CB583358	CB583358	316325	0.07	1.169	0.315795
A_44_P813903	TC552055	TC552055		0.07	1.169	0.187328
A_42_P730411	Elf2	NM_001012181	361944	0.07	1.169	0.101555
A_44_P432626	BF388440	BF388440	361814	0.07	1.169	0.304109
A_43_P17428	Oact5	NM_001012189	362434	0.07	1.169	0.279767
A_44_P302405	Tia1	NM_001012096	312510	0.07	1.169	0.177643
A_44_P482476	Fcgr3	M64368	116591	0.07	1.169	0.533083
A_44_P489852	A_44_P489852	A_44_P489852		0.07	1.169	0.178387
A_44_P301654	Dnajc14	NM_053690	114481	0.07	1.169	0.197293
A_44_P354154	RGD1305243_predicted	XM_341690	361409	0.07	1.169	0.36439
A_44_P1054708	MGC72612	NM_001009538	494340	0.07	1.169	0.46711
A_44_P297389	A_44_P297389	A_44_P297389		0.07	1.168	0.103882
A_44_P527709	Gtf2f1	NM_001007711	316123	0.07	1.168	0.194547
A_44_P232223	RGD1561893_predicted	XM_237153	301415	0.07	1.168	0.6998

A_42_P623013	Recql5_predicted	XM_237812		0.07	1.168	0.23592
A_44_P788523	AW143863	AW143863		0.07	1.168	0.466117
A_44_P929454	TC551929	TC551929		0.07	1.168	0.646451
A_43_P16896	AW507178	AW507178	294718	0.07	1.168	0.275347
A_44_P931596	XM_579857	XM_579857		0.07	1.168	0.273618
A_44_P833772	LOC685179	XM_001055795	685179	0.07	1.168	0.201578
A_44_P487081	Npuk68	NM_001033951	192359	0.07	1.168	0.42927
A_44_P189326	Pdia3	NM_017319	29468	0.07	1.168	0.381594
A_43_P10867	CB547678	CB547678		0.07	1.168	0.364305
A_44_P280898	Dbp	NM_012543	24309	0.07	1.168	0.181614
A_44_P498259	Slc9a6_predicted	XM_217630	302863	0.07	1.168	0.55572
A_42_P777285	AI013497	AI013497		0.07	1.168	0.217352
A_44_P433632	Pcgf6	NM_001013154	309457	0.07	1.168	0.402617
A_44_P464085	Prcc_predicted	XM_227476	310687	0.07	1.167	0.188971
A_44_P276866	Golt1b_predicted	XM_342782	362460	0.07	1.167	0.350394
A_44_P804042	AW143970	AW143970		0.07	1.167	0.284192
A_44_P838735	TC524373	TC524373		0.07	1.167	0.397615
A_44_P775373	LOC363766	XR_007878	363766	0.07	1.167	0.289744
A_42_P634560	Dscam	NM_133587	171119	0.07	1.167	0.265474
A_43_P14027	Mipep	NM_031052	81684	0.07	1.167	0.260675
A_44_P1007933	LOC683917	XM_001068997		0.07	1.167	0.315789
A_44_P222385	LOC678741	XM_001053214	678741	0.07	1.167	0.350429
A_42_P551239	Brd9_predicted	XM_217740		0.07	1.167	0.356417
A_44_P522987	Dmxl1_predicted	XM_001054434		0.07	1.167	0.286807
A_44_P506568	LOC689496	XM_001070990		0.07	1.167	0.239951
A_44_P491604	Txndc9	NM_172032	280671	0.07	1.167	0.284114
A_44_P398592	Loh12cr1_predicted	XM_342773		0.07	1.167	0.167659
A_44_P505472	AW918239	AW918239		0.07	1.167	0.686338
A_44_P465895	Atp6v1f	NM_053884	116664	0.07	1.167	0.179179
A_44_P187238	MGC112727	NM_001037787	360762	0.07	1.166	0.176398
A_44_P1028435	LOC687055	XM_001076884		0.07	1.166	0.344065
A_44_P548524	RGD1564093_predicted	XM_213841		0.07	1.166	0.152426
A_43_P21308	Dhx57	XM_345625	366532	0.07	1.166	0.375877
A_44_P304467	Znf507_predicted	XM_218515		0.07	1.166	0.165816
A_44_P213254	Mrps25	NM_001025408	297459	0.07	1.166	0.259769
A_43_P10011	RGD1307395	XM_001055560		0.07	1.166	0.395184
A_44_P746102	TC540505	TC540505		0.07	1.166	0.403266
A_44_P209461	Rab28	NM_053978	117049	0.07	1.166	0.164099
A_44_P868828	TC555639	TC555639		0.07	1.166	0.138142
A_44_P255792	Papola_predicted	XM_234508		0.07	1.166	0.195291
A_44_P720354	AI010615	AI010615	306527	0.07	1.166	0.427298
A_44_P309105	A_44_P309105	A_44_P309105		0.07	1.166	0.374573
A_42_P510530	LOC689637	XM_001073061		0.07	1.166	0.172436
A_44_P1036769	Matn1	NM_001006979	297894	0.07	1.166	0.54671
A_43_P14636	Vps35_mapped	XM_214646		0.07	1.165	0.222175
A_44_P227281	Btf3	NM_001008309	294680	0.07	1.165	0.277389
A_44_P529638	RGD1563971_predicted	XM_344066		0.07	1.165	0.16062
A_44_P222011	Mrlcb	NM_017343	50685	0.07	1.165	0.395214
A_44_P607611	Bag4	NM_001025130	361167	0.07	1.165	0.450282
A_44_P208887	Rpusd1_predicted	XM_213253		0.07	1.165	0.241273
A_43_P16927	Pofut2_predicted	XM_228073		0.07	1.165	0.336519
A_44_P194828	XM_235410	XM_235410		0.07	1.165	0.269957
A_44_P154769	LOC688133	XM_001081155		0.07	1.165	0.226922
A_44_P618632	AW916925	AW916925		0.07	1.165	0.468864
A_44_P534753	Zmynd15_predicted	XM_213338		0.07	1.165	0.215011
A_44_P1011305	Mylk_predicted	XM_213611		0.07	1.165	0.273534
A_43_P15200	RGD1562348_predicted	XM_214012		0.07	1.165	0.206381
A_43_P18040	Slc19a2	NM_001030024	289175	0.07	1.164	0.194342
A_44_P527075	Hey1	XM_342216	155437	0.07	1.164	0.243031
A_43_P22360	Hccs_predicted	XM_228867	317444	0.07	1.164	0.30164
A_44_P490188	XM_343247	XM_343247		0.07	1.164	0.319591
A_44_P795015	TC560463	TC560463		0.07	1.164	0.301246
A_44_P299293	Scap1	NM_173311	286975	0.07	1.164	0.580358

A_44_P161038	Sgta	NM_022703	64667	0.07	1.164	0.191044
A_43_P14618	TC555070	TC555070		0.07	1.164	0.308698
A_44_P859124	A_44_P859124	A_44_P859124		0.07	1.164	0.378612
A_44_P486411	AI012802	AI012802	24439	0.07	1.164	0.201872
A_44_P433542	Hnrpa3	NM_198132	362152	0.07	1.164	0.200896
A_44_P927671	DV718923	DV718923	307820	0.07	1.164	0.277427
A_44_P1006595	Zfp598_predicted	XM_213230		0.07	1.164	0.20218
A_44_P518250	A_44_P518250	A_44_P518250		0.07	1.164	0.266283
A_44_P419064	Cfb	NM_212466	294257	0.07	1.164	0.319995
A_44_P195921	Psmd10	NM_053925	116722	0.07	1.164	0.172319
A_44_P698199	A_44_P698199	A_44_P698199		0.07	1.163	0.24011
A_44_P378886	AI231159	AI231159	292082	0.07	1.163	0.290939
A_44_P365443	Lphn2	NM_134408	171447	0.07	1.163	0.558432
A_43_P17077	RGD1306300_predicted	XM_341945		0.07	1.163	0.188254
A_44_P1013959	Lcmt1	NM_199405	361643	0.07	1.163	0.197871
A_44_P543537	LOC691484	XM_001078485		0.07	1.163	0.277646
A_44_P622216	Snx19_predicted	XM_001055595		0.07	1.163	0.485519
A_44_P201766	Raver1h	NM_001013939	298705	0.07	1.163	0.268372
A_44_P137618	Prdx5	NM_053610	113898	0.07	1.163	0.374795
A_44_P259908	Glud1	NM_012570	24399	0.07	1.163	0.398503
A_43_P10703	Zfp91	XM_001079228		0.07	1.163	0.320943
A_42_P554295	RGD1561393_predicted	XM_342232	361932	0.07	1.163	0.238278
A_44_P400324	Maf	NM_019318	54267	0.07	1.163	0.357324
A_44_P482413	Olr725_predicted	NM_001000514	311180	0.07	1.163	0.35401
A_44_P283518	Slc27a1	NM_053580	94172	0.07	1.163	0.17744
A_44_P145005	XM_342766	XM_342766		0.07	1.163	0.382197
A_44_P297428	RGD1562646_predicted	XM_223468	305392	0.07	1.163	0.36407
A_44_P540637	Hnrpf	NM_022397	64200	0.07	1.162	0.265638
A_44_P958005	AA963087	AA963087		0.07	1.162	0.231289
A_44_P303858	RGD1306116_predicted	XM_342068	361774	0.07	1.162	0.205508
A_44_P853006	LOC686289	XM_001073320		0.07	1.162	0.225776
A_44_P680056	Ap1g1	XM_341686	171494	0.07	1.162	0.183553
A_44_P175807	Rnf12	NM_001024892	317241	0.07	1.162	0.220801
A_44_P264275	AI555150	AI555150		0.07	1.162	0.306045
A_44_P507448	RGD1359509	NM_001009671	301416	0.07	1.162	0.406801
A_43_P22800	LOC685958	XM_001065934		0.07	1.162	0.278445
A_44_P311985	RGD1559921_predicted	XM_345694		0.07	1.162	0.294288
A_44_P263740	Tmem86a_predicted	XM_218592	308602	0.07	1.162	0.148646
A_44_P184685	Sertad1	NM_001007735	361526	0.07	1.162	0.319359
A_44_P959299	LOC682955	XM_001063844		0.07	1.162	0.159801
A_44_P225364	Rpl31	NM_022506	64298	0.07	1.162	0.258551
A_44_P325609	Olr214_predicted	NM_001000197	293361	0.07	1.162	0.311073
A_43_P23101	Bucs1_predicted	XM_341917		0.07	1.162	0.345617
A_44_P633002	AI104487	AI104487	64551	0.07	1.162	0.378136
A_42_P575104	Sostdc1	NM_153737	266803	0.07	1.162	0.470733
A_43_P14884	Akap8	XM_343176	116633	0.07	1.162	0.166479
A_44_P473048	L20994	L20994		0.07	1.162	0.412487
A_44_P388973	Got1	NM_012571	24401	0.07	1.162	0.377843
A_44_P505932	Cttn	NM_021868	60465	0.07	1.162	0.2354
A_44_P1023498	Flot1	NM_022701	64665	0.07	1.162	0.240446
A_43_P17382	Lrrc16_predicted	XM_001070748		0.07	1.161	0.315436
A_44_P1024476	TC520546	TC520546		0.07	1.161	0.285976
A_44_P300232	Appbp2	XM_220805	303396	0.06	1.161	0.396406
A_42_P666843	Pgls_predicted	XM_214296		0.06	1.161	0.142848
A_43_P19299	RGD1309983_predicted	XM_235571	315203	0.06	1.161	0.401224
A_44_P511359	Mum1_predicted	XM_343166		0.06	1.161	0.174506
A_44_P394978	AI104924	AI104924	29556	0.06	1.161	0.545358
A_44_P911233	AW143854	AW143854		0.06	1.161	0.556628
A_42_P673309	Styxl1	NM_001037788	360792	0.06	1.161	0.209023
A_44_P744931	A_44_P744931	A_44_P744931		0.06	1.161	0.217298
A_44_P103692	RGD1309059_predicted	XM_234484		0.06	1.161	0.270969
A_44_P367761	Ccng1	NM_012923	25405	0.06	1.161	0.470321
A_44_P368605	CB546892	CB546892	680434	0.06	1.161	0.521246

A_43_P10405	RGD1560924_predicted	XM_575725		0.06	1.161	0.362938
A_44_P487300	A_44_P487300	A_44_P487300		0.06	1.161	0.148653
A_44_P295494	LOC366780	XM_345764		0.06	1.161	0.315666
A_44_P779359	TC551102	TC551102		0.06	1.161	0.261061
A_44_P1017897	MGC109149	NM_001009705	361520	0.06	1.161	0.419483
A_44_P264628	Fgfr1	U95164	79114	0.06	1.161	0.295398
A_44_P558018	LOC681725	XR_007639	686096	0.06	1.161	0.264104
A_44_P411875	BE098875	BE098875	301514	0.06	1.161	0.355915
A_43_P15897	Rarb	XM_223843	24706	0.06	1.160	0.273818
A_44_P857191	TC565036	TC565036		0.06	1.160	0.233918
A_44_P439687	BQ200332	BQ200332	292729	0.06	1.160	0.257951
A_44_P456642	Dctn2	NM_001004239	299850	0.06	1.160	0.281114
A_43_P22757	Slc16a6	NM_198760	303772	0.06	1.160	0.368059
A_44_P155576	AA859465	AA859465	362039	0.06	1.160	0.36727
A_44_P300506	Calr	NM_022399	64202	0.06	1.160	0.358786
A_44_P1008950	LOC290651	NM_001013880	290651	0.06	1.160	0.245024
A_42_P538483	Rnf40	NM_153471	266712	0.06	1.160	0.182181
A_42_P538374	RGD1310316	NM_001014029	309420	0.06	1.160	0.355011
A_44_P527563	LOC687307	XM_001072658		0.06	1.160	0.627091
A_44_P685128	TC542190	TC542190		0.06	1.160	0.180902
A_44_P337615	Tbrg4	NM_001012154	360977	0.06	1.160	0.193985
A_44_P451392	Fbxo33_predicted	XM_234205		0.06	1.160	0.509286
A_43_P12152	Robo1	NM_022188	58946	0.06	1.160	0.403692
A_44_P169313	BE105548	BE105548	114764	0.06	1.160	0.323729
A_44_P111921	Dmtf1	AF352169	114485	0.06	1.160	0.311073
A_42_P722646	RGD1565411_predicted	XM_343330	363000	0.06	1.159	0.21534
A_44_P700178	TC541059	TC541059		0.06	1.159	0.311108
A_42_P478500	Slc22a17	NM_177421	305886	0.06	1.159	0.273951
A_44_P253001	XM_344384	XM_344384		0.06	1.159	0.435166
A_44_P202659	Ndufs4	NM_001025146	499529	0.06	1.159	0.640571
A_44_P421978	RGD1562997_predicted	XM_001073245		0.06	1.159	0.184227
A_44_P575173	LOC680286	XM_001056463		0.06	1.159	0.599615
A_44_P528843	AI008393	AI008393	363332	0.06	1.159	0.44361
A_44_P421295	Cyp11b3	NM_181824	353498	0.06	1.159	0.665812
A_43_P17977	LOC681927	XM_001056512		0.06	1.159	0.238453
A_44_P305923	CA507578	CA507578	29541	0.06	1.159	0.370249
A_44_P740128	AA955406	AA955406		0.06	1.159	0.393213
A_44_P158333	BF402538	BF402538	314642	0.06	1.159	0.241386
A_44_P109977	Trim13	NM_001012210	364398	0.06	1.159	0.281755
A_44_P808557	TC525896	TC525896		0.06	1.159	0.262013
A_44_P558249	BF558337	BF558337		0.06	1.159	0.16474
A_44_P289267	BF551324	BF551324	362591	0.06	1.158	0.488017
A_44_P457066	XM_224528	XM_224528		0.06	1.158	0.315302
A_44_P806792	TC562629	TC562629		0.06	1.158	0.561905
A_43_P16181	AJ003232	AJ003232	294270	0.06	1.158	0.572454
A_44_P807259	TC554613	TC554613		0.06	1.158	0.181539
A_44_P102265	BQ204131	BQ204131		0.06	1.158	0.384056
A_42_P831504	AI103276	AI103276	499691	0.06	1.158	0.229559
A_44_P487411	LOC688405	XM_001069579		0.06	1.158	0.198242
A_44_P940501	AW143282	AW143282		0.06	1.158	0.506091
A_42_P794613	Mvd	NM_031062	81726	0.06	1.158	0.343136
A_44_P485325	XM_230930	XM_230930		0.06	1.158	0.206705
A_44_P189461	C8b_mapped	XM_233250	313421	0.06	1.158	0.740469
A_42_P467994	Slc12a4	NM_019229	29501	0.06	1.158	0.451234
A_44_P191055	Pogz_predicted	XM_227475		0.06	1.158	0.220795
A_44_P309008	Kif11	XM_001060913	171304	0.06	1.158	0.460442
A_44_P189065	Ssr2_predicted	XM_215619		0.06	1.158	0.276698
A_44_P1031285	LOC368070	XM_347237		0.06	1.158	0.340479
A_43_P17598	Dpp8_predicted	XM_236345		0.06	1.157	0.394329
A_43_P10424	RGD1560049_predicted	XM_573199	498003	0.06	1.157	0.623825
A_44_P436528	Tenc1_predicted	XM_235710		0.06	1.157	0.193347
A_44_P713545	A_44_P713545	A_44_P713545		0.06	1.157	0.250748
A_44_P110035	Trim33_predicted	XM_345266	365894	0.06	1.157	0.356406

A_44_P269675	LOC685894	XM_001065667		0.06	1.157	0.211353
A_44_P669900	TC541871	TC541871		0.06	1.157	0.309287
A_44_P862144	A_44_P862144	A_44_P862144		0.06	1.157	0.224381
A_43_P12524	Ssr3	NM_031120	81784	0.06	1.157	0.334915
A_44_P199764	Nek7_predicted	XM_001066105		0.06	1.157	0.206563
A_44_P1002041	Recql	NM_001012098	312824	0.06	1.157	0.234482
A_44_P948572	TC526296	TC526296		0.06	1.157	0.180596
A_44_P203371	Hdlbp	NM_172039	64474	0.06	1.157	0.325452
A_44_P557905	LOC682185	XM_001060320		0.06	1.157	0.187111
A_44_P728540	ENSRNOT00000048160	ENSRNOT00000048160		0.06	1.157	0.143144
A_44_P128929	Zfp692_predicted	XM_220455	303164	0.06	1.157	0.302206
A_44_P557983	RGD1561582_predicted	XM_228464	302337	0.06	1.157	0.207871
A_44_P520392	RGD1311481_predicted	XM_219527	309178	0.06	1.157	0.411417
A_44_P214836	Hand2	NM_022696	64637	0.06	1.157	0.500207
A_43_P11701	Sdc2	NM_013082	25615	0.06	1.157	0.553162
A_44_P365694	Vps37c_predicted	XM_217901		0.06	1.157	0.16281
A_44_P395483	CK843871	CK843871		0.06	1.156	0.515081
A_44_P868650	TC538314	TC538314		0.06	1.156	0.24245
A_44_P368044	XM_214290	XM_214290		0.06	1.156	0.151891
A_44_P492291	Mbd1	NM_001011924	291439	0.06	1.156	0.247045
A_44_P316136	Hdh	XM_573634	29424	0.06	1.156	0.24186
A_44_P622593	Gspt1	NM_001003978	24420	0.06	1.156	0.418813
A_42_P516535	Noxa1_predicted	XM_231042		0.06	1.156	0.293079
A_44_P349408	LOC499653	NM_001024804		0.06	1.156	0.268372
A_44_P622837	TC522970	TC522970		0.06	1.156	0.4022
A_43_P12379	Apaf1	NM_023979	78963	0.06	1.156	0.214178
A_44_P535029	AW434178	AW434178	300025	0.06	1.156	0.496042
A_43_P18014	CB605626	CB605626	309887	0.06	1.156	0.43834
A_43_P17716	CB545120	CB545120	301618	0.06	1.156	0.289219
A_44_P314187	A_44_P314187	A_44_P314187		0.06	1.156	0.207483
A_44_P206526	RGD1565950_predicted	XM_213548	287899	0.06	1.156	0.558038
A_42_P586681	Upf2_predicted	XM_341556		0.06	1.156	0.206268
A_44_P745879	TC539458	TC539458		0.06	1.156	0.334465
A_44_P1000525	Smc5l1_predicted	XM_215254		0.06	1.155	0.237507
A_44_P782553	LOC368190	XM_347344		0.06	1.155	0.422864
A_44_P100813	BM389227	BM389227	501282	0.06	1.155	0.305199
A_42_P509457	Arl6ip4	NM_001025630	288656	0.06	1.155	0.199728
A_44_P1006208	AW915541	AW915541	289088	0.06	1.155	0.342169
A_44_P577705	RGD1559826_predicted	XM_573506		0.06	1.155	0.361999
A_44_P154877	Zswim5_predicted	XM_233421		0.06	1.155	0.438379
A_44_P728586	ENSRNOT00000013367	ENSRNOT00000013367		0.06	1.155	0.470243
A_42_P835247	Egln2	NM_001004083	308457	0.06	1.155	0.323845
A_44_P792646	TC525174	TC525174		0.06	1.155	0.56239
A_43_P18939	Hook2	XM_239718	304669	0.06	1.155	0.351363
A_42_P524302	Jtb	NM_019213	29439	0.06	1.155	0.169172
A_44_P975369	Itga3_predicted	XM_001081309		0.06	1.155	0.226519
A_44_P510515	Slc6a6	NM_017206	29464	0.06	1.155	0.315319
A_42_P577560	Lcn7	NM_053582	94174	0.06	1.155	0.352928
A_44_P1000449	Pde7a	XM_215540	81744	0.06	1.155	0.587483
A_43_P15280	Tef	NM_019194	29362	0.06	1.155	0.28238
A_44_P825084	TC566987	TC566987		0.06	1.155	0.373582
A_44_P606549	Srpr	NM_001034150	315548	0.06	1.155	0.247189
A_42_P822588	Alox15	NM_031010	81639	0.06	1.155	0.562769
A_43_P21821	CB547114	CB547114		0.06	1.155	0.276255
A_44_P1014934	TC518380	TC518380		0.06	1.155	0.228753
A_44_P519336	AW141777	AW141777	54312	0.06	1.155	0.323316
A_44_P365672	Atg16l2_predicted	XM_218966		0.06	1.155	0.273243
A_44_P1031034	Rsn	NM_031745	65201	0.06	1.155	0.492753
A_44_P504298	Cdc42ep4_predicted	XM_221077		0.06	1.155	0.191198
A_44_P323533	Sbno1	XM_222152		0.06	1.154	0.186532
A_44_P350709	BF285026	BF285026	300519	0.06	1.154	0.329761
A_44_P242252	Ube2d3	NM_031237	81920	0.06	1.154	0.178087
A_44_P208930	Mks1	NM_001034917	287612	0.06	1.154	0.144852

A_44_P342117	Wbp2	NM_138975	192645	0.06	1.154	0.304109
A_44_P509872	Stard7_predicted	XM_238280		0.06	1.154	0.376843
A_42_P636232	Ppic	NM_001004215	291463	0.06	1.154	0.297127
A_43_P18798	Fbxw8_predicted	XM_222223		0.06	1.154	0.17911
A_44_P352061	XM_234862	XM_234862		0.06	1.154	0.271893
A_43_P18360	Prkx	NM_001033963	501563	0.06	1.154	0.20566
A_44_P119405	Cops7a_predicted	XM_232351		0.06	1.154	0.177325
A_42_P679004	Zfp295_predicted	XM_001056449		0.06	1.154	0.257475
A_44_P379645	AF030090	AF030090		0.06	1.154	0.264104
A_44_P266776	H2afx	NM_199388	300668	0.06	1.154	0.361853
A_44_P459845	Hoxb3_predicted	XM_220893		0.06	1.154	0.617796
A_44_P173048	Sfrs9	NM_001009255	288701	0.06	1.153	0.474301
A_44_P1033541	Eif4b	NM_001008324	300253	0.06	1.153	0.268027
A_44_P836767	A_44_P836767	A_44_P836767		0.06	1.153	0.34458
A_44_P152682	BQ209756	BQ209756	316085	0.06	1.153	0.354787
A_44_P1050395	BF391513	BF391513	317335	0.06	1.153	0.4972
A_44_P203967	Recql	NM_001012098	312824	0.06	1.153	0.138037
A_44_P805210	Ssh3	NM_001012217	365396	0.06	1.153	0.246784
A_44_P515321	ENSRNOT00000019364	ENSRNOT00000019364		0.06	1.153	0.152137
A_44_P975731	TC539236	TC539236		0.06	1.153	0.236624
A_44_P720473	TC538691	TC538691		0.06	1.153	0.372213
A_44_P121063	Aqp7	NM_019157	29171	0.06	1.153	0.424996
A_44_P501493	Crtc2	NM_001033895	310615	0.06	1.153	0.141969
A_44_P444257	Cdkn2d	NM_001009719	494444	0.06	1.153	0.488876
A_42_P589674	Taf6	XM_001066806		0.06	1.153	0.281283
A_43_P20164	LOC316457	XM_237217	316457	0.06	1.153	0.179405
A_44_P664687	AW916443	AW916443		0.06	1.153	0.48884
A_44_P592627	RGD1563203_predicted	XM_001057159		0.06	1.153	0.237155
A_43_P22577	RGD1563091_predicted	XM_575365	500011	0.06	1.153	0.683621
A_44_P134688	Tpm3	NM_173111	117557	0.06	1.153	0.229542
A_44_P1006655	Rps27	NM_053597	94266	0.06	1.153	0.222387
A_44_P542133	AI013214	AI013214		0.06	1.152	0.364431
A_44_P375207	LOC678818	XM_001053295		0.06	1.152	0.429202
A_44_P302012	RGD1566355_predicted	XM_235722	315362	0.06	1.152	0.335257
A_42_P690223	Chd7_predicted	XM_232671		0.06	1.152	0.2368
A_42_P701169	LOC690263	XM_001076608		0.06	1.152	0.271913
A_44_P778542	TC528843	TC528843		0.06	1.152	0.460847
A_44_P373474	Pik3c2a_predicted	XM_341911		0.06	1.152	0.490078
A_44_P155603	BG667930	BG667930		0.06	1.152	0.254673
A_43_P16393	Adam4	NM_020305	57022	0.06	1.152	0.358734
A_43_P20037	Ect2_predicted	XM_342220		0.06	1.152	0.378984
A_44_P670611	TC529518	TC529518		0.06	1.152	0.400055
A_44_P349199	Gria4	NM_017263	29629	0.06	1.152	0.173367
A_43_P11442	B2m	NM_012512	24223	0.06	1.152	0.59416
A_44_P126154	Cdc37l1	NM_001011941	293886	0.06	1.152	0.314888
A_42_P636608	Zfp307	NM_001012053	306977	0.06	1.152	0.286963
A_44_P477675	Olr1364_predicted	NM_001000858	405150	0.06	1.152	0.231032
A_44_P436164	Ckap2_predicted	XM_224970	306575	0.06	1.152	0.552343
A_44_P826605	TC549865	TC549865		0.06	1.152	0.616723
A_44_P260496	XM_228943	XM_228943		0.06	1.152	0.429785
A_44_P506561	Lzic	NM_001013241	366507	0.06	1.152	0.464569
A_44_P552118	Cnih_predicted	XM_214153		0.06	1.152	0.278218
A_44_P119674	XM_340952	XM_340952		0.06	1.151	0.39687
A_44_P621592	LOC366328	XR_007613	366328	0.06	1.151	0.285976
A_44_P227286	XM_234810	XM_234810		0.06	1.151	0.641925
A_43_P10346	CB547689	CB547689		0.06	1.151	0.1652
A_44_P187860	CB548514	CB548514		0.06	1.151	0.366633
A_44_P501797	Mgea6_predicted	XM_001079741		0.06	1.151	0.375585
A_44_P522682	Arf6	NM_024152	79121	0.06	1.151	0.395049
A_44_P344306	Cops2	NM_153297	261736	0.06	1.151	0.339223
A_44_P351627	XM_219208	XM_219208		0.06	1.151	0.315789
A_44_P118315	LOC686393	XM_001073919		0.06	1.151	0.413115
A_44_P368406	Ctdsp1	XM_343588	363249	0.06	1.151	0.206563

A_42_P512417	Dpm2	NM_019252	29640	0.06	1.151	0.295037
A_43_P11819	Ctsc	NM_017097	25423	0.06	1.150	0.49959
A_44_P220777	Swap70_predicted	XM_219262		0.06	1.150	0.322227
A_43_P14812	RGD1560566_predicted	XM_219337	308986	0.06	1.150	0.226409
A_44_P159974	Myst3	XM_225008	306571	0.06	1.150	0.367129
A_44_P398969	Gtpbp2	XM_343535	363195	0.06	1.150	0.35564
A_44_P990309	Zfp655	NM_001008362	360764	0.06	1.150	0.341142
A_44_P1042598	BM391736	BM391736	114637	0.06	1.150	0.23942
A_44_P389408	XM_226570	XM_226570		0.06	1.150	0.4286
A_44_P356561	XM_228077	XM_228077		0.06	1.150	0.264104
A_44_P669034	RGD1566329_predicted	XM_573180		0.06	1.150	0.463377
A_44_P363522	B4galt7	NM_001031661	364675	0.06	1.150	0.228436
A_44_P196532	Mk1	NM_134399	171436	0.06	1.150	0.35836
A_44_P518434	Mrpl4_predicted	XM_343354		0.06	1.150	0.469795
A_44_P345476	Myh9	NM_013194	25745	0.06	1.149	0.222843
A_44_P384304	A_44_P384304	A_44_P384304		0.06	1.149	0.201672
A_44_P760892	Eif4g2	XM_001077834		0.06	1.149	0.335578
A_42_P792817	Me1	M30596	24552	0.06	1.149	0.590268
A_43_P17521	Cog1_predicted	XM_239373		0.06	1.149	0.250506
A_44_P622658	TC556508	TC556508		0.06	1.149	0.286694
A_44_P181679	Brd2	NM_212495	294276	0.06	1.149	0.274692
A_44_P334940	LOC498331	XM_213997	498331	0.06	1.149	0.570976
A_43_P10908	CA512649	CA512649		0.06	1.149	0.321294
A_42_P620042	Gsk3b	NM_032080	84027	0.06	1.149	0.260227
A_44_P519917	F2rl3	NM_053808	116498	0.06	1.149	0.533425
A_43_P12882	Cds2	NM_053643	114101	0.06	1.149	0.34635
A_44_P434216	Rdh11	NM_001012193	362757	0.06	1.149	0.295707
A_44_P402645	Ihpk1	NM_053316	50560	0.06	1.149	0.355823
A_44_P358384	Dlat	NM_031025	81654	0.06	1.148	0.372857
A_44_P274173	Gcnt3	NM_173312	286976	0.06	1.148	0.465683
A_44_P172899	Mc2r	XM_574167		0.06	1.148	0.489137
A_43_P21196	LOC681234	XM_001058421		0.06	1.148	0.324385
A_44_P173345	Stx16_predicted	XM_342595		0.06	1.148	0.212085
A_44_P1060234	Pdcl3	NM_001025709	316348	0.06	1.148	0.242663
A_44_P1011038	RGD1561069_predicted	XM_001077235		0.06	1.148	0.39727
A_44_P949940	TC550297	TC550297		0.06	1.148	0.318518
A_44_P201511	39879	NM_001012087	311059	0.06	1.148	0.268936
A_43_P10634	Kdelc2	NM_001025123	315664	0.06	1.148	0.3252
A_44_P106923	AA900254	AA900254		0.06	1.148	0.337282
A_43_P21507	Herc1_predicted	XM_236362	315771	0.06	1.148	0.256532
A_44_P1053587	Mta3_predicted	XM_216633		0.06	1.148	0.375639
A_44_P144064	Stk16	NM_173142	286927	0.06	1.148	0.280232
A_43_P16533	Rnaset2_predicted	XM_214769		0.06	1.147	0.265638
A_44_P524471	RGD1311752_predicted	XM_215477		0.06	1.147	0.335671
A_44_P699091	Sdccag3	NM_001013135	306322	0.06	1.147	0.301416
A_44_P557752	LOC679753	XM_001054331		0.06	1.147	0.193789
A_44_P501439	Marveld2_predicted	XM_345145		0.06	1.147	0.366122
A_44_P536553	A_44_P536553	A_44_P536553		0.06	1.147	0.289798
A_44_P295256	Papd1_predicted	XM_225468		0.06	1.147	0.312048
A_44_P505025	Acsl1	NM_012820	25288	0.06	1.147	0.372242
A_44_P331033	RGD1311077_predicted	XM_345897		0.06	1.147	0.285283
A_44_P244138	Ptpn9	NM_001013040	266611	0.06	1.147	0.469231
A_44_P181860	Dr1	NM_001011914	289881	0.06	1.147	0.195307
A_44_P449522	Zfp131	XM_227104	310375	0.06	1.147	0.225827
A_43_P20312	RGD1311847	NM_001013879	290615	0.06	1.146	0.282406
A_44_P100636	LOC686853	XM_001076022		0.06	1.146	0.322003
A_44_P643604	BQ211845	BQ211845		0.06	1.146	0.26901
A_44_P409210	BF413896	BF413896	314329	0.06	1.146	0.460773
A_43_P20501	Zfp217_predicted	XM_231017		0.06	1.146	0.37344
A_44_P637586	BC086562	BC086562	365755	0.06	1.146	0.417095
A_43_P19211	Rbbp6	XM_219296	308968	0.06	1.146	0.2785
A_44_P591118	LOC680452	XM_001055969		0.06	1.146	0.266546
A_44_P166233	XM_225724	XM_225724		0.06	1.146	0.297319

A_44_P434468	LOC691381	XM_001077980		0.06	1.146	0.284482
A_44_P403444	Als2	XM_343574		0.06	1.145	0.37496
A_44_P457099	LOC680466	XM_001056392		0.06	1.145	0.32131
A_44_P416633	Agrn	NM_175754	25592	0.06	1.145	0.378355
A_44_P397356	BF389793	BF389793	25061	0.06	1.145	0.52319
A_44_P413112	Usf2	NM_031139	81817	0.06	1.145	0.264212
A_43_P15635	Pmpca	M57728	296588	0.06	1.145	0.261826
A_44_P475778	RGD1562346_predicted	XM_227459		0.06	1.145	0.53321
A_44_P360219	NM_019206	NM_019206		0.06	1.145	0.542548
A_44_P116981	Rfx1_predicted	XM_222456		0.06	1.145	0.316442
A_42_P618064	AW144502	AW144502		0.06	1.145	0.337449
A_43_P18979	Rabl3_predicted	XM_340992		0.06	1.145	0.372362
A_43_P15701	Rab13	NM_031092	81756	0.06	1.145	0.195734
A_44_P220466	RGD1310845_predicted	XM_233942	313928	0.06	1.145	0.43206
A_42_P745623	RGD1563123_predicted	XM_340943		0.06	1.145	0.277958
A_44_P529811	Myo10_predicted	XM_226874		0.06	1.145	0.229762
A_43_P20354	LOC361646	XM_341925	361646	0.06	1.145	0.210354
A_43_P13332	Ppap2b	NM_138905	192270	0.06	1.145	0.580077
A_44_P108052	XM_213224	XM_213224		0.06	1.145	0.268027
A_44_P410331	XM_219839	XM_219839		0.06	1.144	0.27941
A_44_P531575	Sap1	NM_153740	266809	0.06	1.144	0.36152
A_44_P704947	A_44_P704947	A_44_P704947		0.06	1.144	0.393407
A_44_P350612	CA513258	CA513258		0.06	1.144	0.493621
A_44_P185351	RGD1565752_predicted	XM_234351		0.06	1.144	0.33728
A_44_P513058	AF053360	AF053360	117557	0.06	1.144	0.372037
A_44_P747084	TC526125	TC526125		0.06	1.144	0.480629
A_44_P479131	BF414888	BF414888	54238	0.06	1.144	0.320568
A_44_P312532	BM383877	BM383877	362293	0.06	1.144	0.552524
A_43_P13088	Sc4mol	NM_080886	140910	0.06	1.144	0.209286
A_44_P991773	RGD1305179_predicted	XM_214648	291921	0.06	1.144	0.466849
A_43_P23152	Rhoh	NM_001013430	305341	0.06	1.144	0.36727
A_42_P755414	AW918668	AW918668		0.06	1.144	0.510801
A_44_P959263	A_44_P959263	A_44_P959263		0.06	1.144	0.387938
A_44_P479359	Mvk	NM_031063	81727	0.06	1.144	0.212893
A_44_P446713	Pabpn1	XM_214172	116697	0.06	1.144	0.210622
A_44_P136187	BQ193796	BQ193796	499072	0.06	1.143	0.550839
A_44_P799470	BF563211	BF563211		0.06	1.143	0.664763
A_44_P442279	BM389034	BM389034	287069	0.06	1.143	0.255087
A_44_P583973	BF289888	BF289888		0.06	1.143	0.481357
A_44_P884577	TC539893	TC539893		0.06	1.143	0.561687
A_44_P1033661	Prpf3_predicted	XM_342295		0.06	1.143	0.186296
A_44_P367977	RGD1563350_predicted	XM_219896		0.06	1.143	0.562919
A_44_P655753	TC551624	TC551624		0.06	1.143	0.602781
A_44_P254973	Mds024	NM_198051	378794	0.06	1.143	0.344086
A_44_P398176	Ivd	NM_012592	24513	0.06	1.143	0.408581
A_44_P696866	CF111640	CF111640	315689	0.06	1.143	0.231732
A_44_P168112	Skp1a	NM_001007608	287280	0.06	1.143	0.370769
A_44_P100451	Fvt1_predicted	XM_341106		0.06	1.143	0.481372
A_43_P22686	LOC680897	XM_001059380	680897	0.06	1.143	0.445357
A_44_P288418	March1_predicted	XM_341421	361135	0.06	1.143	0.720516
A_44_P738805	MGC116121	NM_001024905	498830	0.06	1.143	0.421799
A_44_P390826	BF282632	BF282632	293627	0.06	1.143	0.32891
A_42_P704583	LOC690349	XM_001076808		0.06	1.143	0.352943
A_44_P806862	Zfand3	NM_001012175	361816	0.06	1.143	0.260549
A_44_P237683	AW142447	AW142447	679934	0.06	1.142	0.263323
A_44_P347771	CB579735	CB579735		0.06	1.142	0.408621
A_44_P119993	Gphn	NM_022865	64845	0.06	1.142	0.306379
A_44_P170971	ENSRNOT00000039294	ENSRNOT00000039294		0.06	1.142	0.315117
A_43_P11320	XM_343337	XM_343337		0.06	1.142	0.267157
A_44_P344858	Pi16_predicted	XM_215351	294312	0.06	1.142	0.409525
A_44_P793787	TC528875	TC528875		0.06	1.142	0.487661
A_44_P527363	LOC679894	XM_001054874		0.06	1.142	0.405095
A_44_P238270	Klrk1	NM_133512	24934	0.06	1.142	0.397905

A_42_P583618	RGD1309393_predicted	XM_214874		0.06	1.142	0.189437
A_44_P931196	RGD1560924_predicted	XM_575725		0.06	1.142	0.237095
A_44_P291092	MGC116373	NM_001025701	314949	0.06	1.142	0.251266
A_44_P518764	LOC363060	NM_001014209	363060	0.06	1.142	0.650231
A_44_P1060170	Vps33a	NM_022961	65081	0.06	1.142	0.302276
A_44_P975956	TC540196	TC540196		0.06	1.142	0.614775
A_42_P487567	Dsp	XM_001058477		0.06	1.142	0.372976
A_42_P468038	Rps25	NM_001005528	122799	0.06	1.142	0.264084
A_44_P608519	RGD1564664_predicted	XM_575179		0.06	1.142	0.362298
A_44_P654629	A_44_P654629	A_44_P654629		0.06	1.142	0.316622
A_44_P178162	LOC363266	BC099202	363266	0.06	1.142	0.355585
A_44_P808310	TC541487	TC541487		0.06	1.142	0.363339
A_44_P515360	Scamp1	XM_342174		0.06	1.142	0.309998
A_42_P593993	Limk2	NM_024135	29524	0.06	1.141	0.270344
A_44_P994732	RGD1310609_predicted	XM_215064	293488	0.06	1.141	0.186518
A_44_P306374	Hsbp1	NM_173119	286899	0.06	1.141	0.273345
A_44_P177969	RGD1311827	NM_001013875	289753	0.06	1.141	0.272813
A_44_P1019654	Ercc5_mapped	XM_217387		0.06	1.141	0.312026
A_44_P255384	RGD1562394_predicted	XM_344225		0.06	1.141	0.239273
A_43_P16523	RGD1305240_predicted	XM_341861		0.06	1.141	0.22579
A_42_P714875	Clptm1_predicted	XM_214859		0.06	1.141	0.382556
A_44_P977339	DV725919	DV725919		0.06	1.141	0.288655
A_43_P14382	RGD1566118_predicted	XM_347003	361797	0.06	1.141	0.225347
A_44_P221287	Cc2d1b	XM_233342	313478	0.06	1.141	0.266934
A_44_P205301	Grinl1a	NM_183402	192147	0.06	1.140	0.185092
A_43_P17188	Cdkal1_predicted	XM_341524		0.06	1.140	0.287758
A_44_P714986	CK478004	CK478004		0.06	1.140	0.222925
A_44_P961602	Rasl12_predicted	XM_001075445		0.06	1.140	0.730929
A_44_P529627	LOC314432	NM_001014080	314432	0.06	1.140	0.255087
A_44_P827752	TC561481	TC561481		0.06	1.140	0.406465
A_44_P107671	RGD1307703_predicted	XM_344495		0.06	1.140	0.391065
A_44_P340839	LOC680014	XM_001055446	680014	0.06	1.140	0.39091
A_44_P229633	Etnk1_predicted	XM_232515		0.06	1.140	0.480453
A_44_P107199	Sqstm1	NM_181550	113894	0.06	1.140	0.381907
A_44_P836591	AF473847	AF473847	24392	0.06	1.140	0.593567
A_44_P501292	XM_344800	XM_344800		0.06	1.140	0.17704
A_43_P21350	Ankfy1_predicted	XM_239269		0.06	1.140	0.23203
A_44_P116597	Cx3cl1	NM_134455	89808	0.06	1.139	0.406933
A_44_P116809	LOC361014	NM_001014136	361014	0.06	1.139	0.421745
A_44_P228236	AI233993	AI233993		0.06	1.139	0.567864
A_44_P975347	TC537485	TC537485		0.06	1.139	0.352349
A_43_P20970	LOC689577	XM_001071243	689577	0.06	1.139	0.296781
A_44_P132822	Crry	NM_001005265	54243	0.06	1.139	0.473799
A_44_P196939	RGD1559781_predicted	XM_228124		0.06	1.139	0.328124
A_43_P14080	TC552179	TC552179		0.06	1.139	0.468837
A_44_P776812	A_44_P776812	A_44_P776812		0.06	1.139	0.242774
A_42_P506826	Whsc2	NM_001008339	305455	0.06	1.139	0.338883
A_43_P10033	Apobec2_predicted	XM_217334		0.06	1.139	0.570854
A_42_P544887	Stx6	NM_031665	60562	0.06	1.139	0.327653
A_44_P1033703	Adipor2	NM_001037979	312670	0.06	1.139	0.464478
A_44_P468665	LOC684626	XM_001071305		0.06	1.139	0.186568
A_44_P182792	Fkbp8	NM_001037180	290652	0.06	1.139	0.352985
A_44_P852993	Oxsr1_predicted	XM_001078098		0.06	1.139	0.37912
A_44_P184527	Atp5g2	NM_133556	171082	0.06	1.139	0.266119
A_44_P555632	Dguok_predicted	XM_216194		0.06	1.139	0.387282
A_43_P19994	Rlf_predicted	XM_233485	313566	0.06	1.139	0.352683
A_44_P377314	XM_222110	XM_222110		0.06	1.139	0.294673
A_44_P468983	LOC314655	NM_001034930	314655	0.06	1.139	0.26503
A_44_P994614	Ndufb6_predicted	XM_216378		0.06	1.138	0.378055
A_44_P156629	Suv420h2_predicted	XM_218221		0.06	1.138	0.322617
A_44_P110334	RGD1560796_predicted	XM_236387		0.06	1.138	0.366314
A_42_P784252	Cda08	NM_133557	171083	0.06	1.138	0.415022
A_44_P267765	Ybx2_predicted	XM_220618	303250	0.06	1.138	0.642041

A_44_P546180	Cdc40_predicted	XM_342154		0.06	1.138	0.39573
A_44_P551952	Mrpl55_predicted	XM_213321		0.06	1.138	0.305129
A_44_P452926	LOC288331	XR_008267	288331	0.06	1.138	0.422816
A_44_P368769	AI044961	AI044961	83791	0.06	1.138	0.340888
A_44_P176422	BQ782323	BQ782323		0.06	1.138	0.489212
A_44_P194088	Zcchc10	XM_346887	360524	0.06	1.138	0.29744
A_42_P512252	Fgfr1op2	NM_201421	362463	0.06	1.138	0.226541
A_44_P385988	Ahcyl1_predicted	XM_001068488		0.06	1.138	0.28297
A_44_P459729	XM_345785	XM_345785		0.06	1.138	0.473824
A_44_P239222	Senp1_predicted	XM_217046	300193	0.06	1.138	0.276034
A_42_P692798	Tspan2	NM_022589	64521	0.06	1.138	0.454047
A_42_P581060	Glrx5_predicted	XM_343103		0.06	1.138	0.210622
A_44_P849334	DV719405	DV719405		0.06	1.138	0.339486
A_44_P181518	LOC503000	XM_001075213		0.06	1.137	0.329398
A_44_P119419	RGD1307986_predicted	XM_001060925		0.06	1.137	0.337212
A_42_P621826	Tg	NM_030988	24826	0.06	1.137	0.26287
A_44_P154148	XM_344087	XM_344087		0.06	1.137	0.392773
A_44_P322860	Camkk1	NM_031662	60341	0.06	1.137	0.459681
A_43_P21537	XM_214361	XM_214361		0.06	1.137	0.203792
A_43_P18644	TC543339	TC543339		0.06	1.137	0.409466
A_44_P263312	BQ208479	BQ208479		0.06	1.137	0.267158
A_44_P404320	BI395734	BI395734		0.06	1.137	0.515845
A_44_P203596	RGD1560935_predicted	XM_222258		0.06	1.137	0.257594
A_44_P159341	LOC303734	XM_221188	303734	0.06	1.137	0.316286
A_44_P511168	LOC678914	XM_001053665		0.06	1.137	0.469203
A_42_P791872	LOC682454	XM_001061587		0.06	1.137	0.286799
A_43_P12875	Bbs2	NM_053618	113948	0.06	1.137	0.287555
A_44_P532446	A_44_P532446	A_44_P532446		0.06	1.136	0.336888
A_43_P11439	Atp1b2	NM_012507	24214	0.06	1.136	0.781168
A_44_P1048140	AI072036	AI072036	306328	0.06	1.136	0.558832
A_44_P260580	Btbd5_predicted	XM_234235		0.06	1.136	0.196477
A_44_P488022	LOC501039	XM_001065705		0.06	1.136	0.461978
A_44_P899616	RGD1560913_predicted	XM_574627		0.06	1.136	0.375282
A_42_P739322	Nde1	NM_053347	83836	0.06	1.136	0.343181
A_44_P536210	Olr110_predicted	NM_001000743	405007	0.06	1.136	0.440313
A_44_P461763	RGD1308111_predicted	XM_227454		0.06	1.136	0.305129
A_44_P383920	Phyh2	NM_053493	85255	0.06	1.136	0.519321
A_44_P409270	AW913948	AW913948	25579	0.06	1.136	0.206328
A_44_P1046697	Ctdsp1	XM_343588	363249	0.06	1.136	0.380428
A_43_P20539	Scrn2	NM_001012142	360612	0.06	1.136	0.390392
A_44_P102001	Thumpd2	NM_001012108	313851	0.06	1.136	0.491841
A_44_P449969	Chfr	NM_001009258	288734	0.06	1.136	0.392642
A_44_P451652	AW143240	AW143240		0.06	1.136	0.397989
A_44_P282855	Smarcd2	NM_031983	83833	0.06	1.136	0.299649
A_44_P604422	AW142685	AW142685		0.06	1.136	0.472003
A_44_P330943	XM_346119	XM_346119		0.06	1.136	0.477489
A_44_P142335	Dyrk1b_predicted	XM_218378		0.06	1.136	0.326092
A_44_P287368	AI171778	AI171778	360488	0.06	1.136	0.480524
A_44_P410192	Hars2_predicted	XM_342532		0.06	1.135	0.234719
A_42_P482829	Vps11_predicted	XM_236189		0.06	1.135	0.369283
A_44_P191903	Dmtf1	NM_053693	114485	0.05	1.135	0.469207
A_44_P557037	BI278241	BI278241	499057	0.05	1.135	0.451205
A_44_P632322	BI395739	BI395739		0.05	1.135	0.452687
A_43_P14565	RGD1559740_predicted	XM_213960		0.05	1.135	0.313316
A_44_P157123	XM_343421	XM_343421		0.05	1.135	0.302893
A_44_P497124	ENSRNOT00000016333	ENSRNOT00000016333		0.05	1.135	0.3318
A_44_P122372	Pldn	NM_001025714	317630	0.05	1.135	0.369496
A_44_P415901	Mrpl15_predicted	XM_216323		0.05	1.135	0.515249
A_44_P373209	Clk4	NM_001013041	287269	0.05	1.135	0.449283
A_44_P307837	Gfer	NM_013222	27100	0.05	1.135	0.23825
A_42_P483286	Tceal8	NM_001014275	367909	0.05	1.134	0.298415
A_44_P166216	Ndfip1	NM_001013059	291609	0.05	1.134	0.488185
A_44_P717288	TC567384	TC567384		0.05	1.134	0.446782

A_43_P11531	Sstr1	NM_012719	25033	0.05	1.134	0.739277
A_44_P1025310	Psmb3	NM_017285	29676	0.05	1.134	0.313847
A_43_P21431	Mtmr3	NM_001012038	305482	0.05	1.134	0.25115
A_44_P360126	BE096754	BE096754		0.05	1.134	0.544171
A_44_P351982	Dnase1l1	NM_001014223	363522	0.05	1.134	0.281249
A_44_P173138	LOC309169	XM_001071262		0.05	1.134	0.426327
A_44_P317196	BF556130	BF556130	500464	0.05	1.134	0.446774
A_44_P454264	Olr1064_predicted	NM_001001076	405966	0.05	1.134	0.498632
A_44_P808264	BP504249	BP504249		0.05	1.134	0.616723
A_44_P331164	RGD1308813	NM_001013974	303606	0.05	1.134	0.359857
A_44_P246146	LOC302855	NM_001025663	302855	0.05	1.134	0.22579
A_44_P305256	BF284519	BF284519		0.05	1.134	0.362322
A_44_P342770	Cramp1l_predicted	XM_213246	287127	0.05	1.134	0.45022
A_42_P475623	Klf5	NM_053394	84410	0.05	1.134	0.320398
A_44_P501365	RGD1309712	NM_001013991	305623	0.05	1.134	0.239555
A_44_P466464	RGD1305847_predicted	XM_231058	296532	0.05	1.134	0.343048
A_44_P344880	Uxt	NM_001006982	299313	0.05	1.134	0.306661
A_44_P389544	Txndc1	NM_001024800	362751	0.05	1.133	0.416772
A_44_P442867	Becn1	NM_053739	114558	0.05	1.133	0.363692
A_42_P612220	Ddx20	XM_227558	84473	0.05	1.133	0.228802
A_44_P431493	XM_345503	XM_345503		0.05	1.133	0.335996
A_44_P538779	Kdelr1	NM_001017385	361577	0.05	1.133	0.421812
A_44_P1053055	Ccbl1	NM_001013164	311844	0.05	1.133	0.440555
A_42_P578953	Ptrh1_predicted	XM_342416		0.05	1.133	0.318745
A_44_P105004	Csnk1g3	NM_022855	64823	0.05	1.133	0.425287
A_43_P16641	Snapap	NM_001025648	295217	0.05	1.133	0.291321
A_44_P268395	AA943735	AA943735	116509	0.05	1.133	0.538097
A_44_P293817	Alms1_predicted	XM_216189		0.05	1.133	0.41139
A_44_P493688	RGD1309216	NM_001008371	361726	0.05	1.133	0.3252
A_44_P861814	TC520927	TC520927		0.05	1.133	0.66253
A_42_P472304	Phf15_predicted	XM_220398		0.05	1.132	0.22789
A_44_P108086	Rras2	NM_001013434	365355	0.05	1.132	0.443466
A_42_P585119	RGD735029	NM_199493	307480	0.05	1.132	0.198794
A_44_P527635	RGD1309682	NM_001014055	312200	0.05	1.132	0.398209
A_44_P428970	LOC678917	XM_001053801		0.05	1.132	0.333126
A_43_P17341	LOC303057	XM_220315	303057	0.05	1.132	0.326426
A_44_P852017	RGD1560731_predicted	XM_573364		0.05	1.132	0.522421
A_43_P19769	RGD1307943_predicted	XM_343049		0.05	1.132	0.456372
A_44_P249391	Traf7_predicted	XM_340764	360491	0.05	1.132	0.32684
A_42_P562435	Gtl3	NM_001037978	307642	0.05	1.132	0.475978
A_44_P459301	LOC309016	XM_219377	309016	0.05	1.132	0.535261
A_44_P667524	RGD1565059_predicted	XM_574953	499630	0.05	1.132	0.630506
A_44_P414879	Ndor1_predicted	XM_231049		0.05	1.132	0.338792
A_44_P231994	Slc37a3_predicted	XM_231626		0.05	1.132	0.623266
A_44_P910901	AI172165	AI172165		0.05	1.132	0.404756
A_44_P231657	Zfp628_predicted	XM_341779		0.05	1.132	0.291954
A_43_P12786	Fabp4	NM_053365	79451	0.05	1.132	0.691946
A_44_P529833	Ttc14_predicted	XM_227030		0.05	1.132	0.458361
A_44_P165938	Sumf2	NM_001025125	360800	0.05	1.132	0.351742
A_44_P248602	RGD1565455_predicted	XM_236400		0.05	1.132	0.249429
A_44_P859016	AA800007	AA800007	245981	0.05	1.131	0.290908
A_42_P641168	Mrs2l	NM_024001	79032	0.05	1.131	0.625852
A_43_P15106	E2f8	XM_218601	308607	0.05	1.131	0.553613
A_43_P22242	Ssh3	NM_001012217	365396	0.05	1.131	0.295649
A_44_P367676	Odf2	NM_017213	29479	0.05	1.131	0.365453
A_44_P689411	AI044786	AI044786		0.05	1.131	0.501851
A_43_P15664	LOC682507	XM_001061794		0.05	1.131	0.427951
A_42_P761159	Mrps18c_predicted	XM_214003		0.05	1.131	0.317025
A_44_P168265	Prkaa1	NM_019142	65248	0.05	1.131	0.545983
A_44_P141992	Slc6a12	NM_017335	50676	0.05	1.131	0.474342
A_44_P149267	Slc38a6	NM_001013099	299139	0.05	1.131	0.338605
A_44_P976747	TC559203	TC559203		0.05	1.130	0.543362
A_44_P198910	XM_213721	XM_213721		0.05	1.130	0.312461

A_44_P139694	Tnfaip1	NM_182950	287543	0.05	1.130	0.273984
A_44_P668704	TC539092	TC539092		0.05	1.130	0.276257
A_44_P279285	RGD1307365	XM_001054857	300880	0.05	1.130	0.521647
A_44_P460124	AA900322	AA900322		0.05	1.130	0.522019
A_44_P412805	RGD1304567	NM_001009711	362671	0.05	1.130	0.384473
A_42_P513911	RGD1559763_predicted	XM_217612	302670	0.05	1.130	0.302716
A_44_P669746	Cova1_predicted	XM_229131	302817	0.05	1.130	0.33037
A_44_P414938	XM_230560	XM_230560		0.05	1.130	0.381527
A_44_P682186	AW917472	AW917472		0.05	1.130	0.577182
A_44_P384798	B3galt6_predicted	XM_233721		0.05	1.130	0.253813
A_44_P575571	ENSRNOT00000008848	ENSRNOT00000008848		0.05	1.130	0.318468
A_44_P624710	TC547881	TC547881		0.05	1.130	0.392773
A_44_P244995	Zfp513	NM_001012110	313913	0.05	1.130	0.315493
A_44_P482710	Spnb4	XM_218364	308458	0.05	1.130	0.416891
A_44_P213336	RGD1561395_predicted	XM_233230		0.05	1.130	0.317277
A_44_P326221	Lemd2	NM_001039032	361807	0.05	1.129	0.319007
A_44_P348868	Dync1h1	NM_019226	29489	0.05	1.129	0.268509
A_43_P18861	XM_215551	XM_215551		0.05	1.129	0.470431
A_44_P1032285	Cdc27	NM_001024793	360643	0.05	1.129	0.407784
A_42_P502839	LOC503000	XM_001075213		0.05	1.129	0.354471
A_44_P206466	Senp6_predicted	XM_217209		0.05	1.129	0.327399
A_43_P15570	Ykt6	NM_031692	64351	0.05	1.129	0.315908
A_43_P12796	Dctn4	NM_053404	84428	0.05	1.129	0.371631
A_44_P337882	RGD1561004_predicted	XM_001057408		0.05	1.129	0.256378
A_44_P914323	DV718684	DV718684		0.05	1.129	0.533335
A_44_P967459	AI113186	AI113186	363140	0.05	1.129	0.567371
A_44_P427648	LOC689688	XM_219529		0.05	1.129	0.430452
A_44_P192275	Nfatc3_predicted	XM_341680		0.05	1.129	0.322132
A_44_P236025	Pofut1	NM_001002278	311551	0.05	1.129	0.2987
A_42_P716940	Top3b_predicted	XM_213564		0.05	1.129	0.306573
A_43_P23361	Isg20l1_predicted	XM_341874		0.05	1.129	0.420458
A_43_P19436	RGD1304885_predicted	XM_215597	295171	0.05	1.129	0.302753
A_44_P175447	XM_218396	XM_218396		0.05	1.129	0.474435
A_44_P440381	AY321326	AY321326		0.05	1.128	0.352943
A_44_P545867	RGD1309359_predicted	XM_222229		0.05	1.128	0.383072
A_44_P685985	TC527977	TC527977		0.05	1.128	0.578253
A_44_P540690	Slc5a3	NM_053715	114507	0.05	1.128	0.651652
A_44_P302043	LOC304484	NM_001025665	304484	0.05	1.128	0.54246
A_44_P139075	Txnl1	NM_080887	140922	0.05	1.128	0.557349
A_44_P776683	TC555263	TC555263		0.05	1.128	0.609955
A_43_P18455	RGD1559552_predicted	XM_573188	497991	0.05	1.128	0.47721
A_44_P427939	AI071957	AI071957		0.05	1.128	0.45809
A_43_P12739	Pawr	NM_033485	64513	0.05	1.128	0.394015
A_44_P426948	RGD1563255_predicted	XM_576176		0.05	1.128	0.33868
A_44_P438269	Azin1	NM_022585	58961	0.05	1.128	0.542531
A_44_P377670	Cpne3_predicted	XM_232809		0.05	1.128	0.504419
A_44_P751844	TC524186	TC524186		0.05	1.128	0.654325
A_43_P16059	Vegfb	NM_053549	89811	0.05	1.128	0.270561
A_44_P180557	A_44_P180557	A_44_P180557		0.05	1.128	0.290675
A_44_P457505	RGD1561755_predicted	XM_346068		0.05	1.128	0.442051
A_44_P318770	A_44_P318770	A_44_P318770		0.05	1.128	0.767185
A_44_P321021	RGD1561029_predicted	XM_001067264		0.05	1.128	0.462726
A_43_P15744	Ctxn	XM_001065365		0.05	1.127	0.427883
A_44_P394291	A_44_P394291	A_44_P394291		0.05	1.127	0.667635
A_44_P651992	A_44_P651992	A_44_P651992		0.05	1.127	0.337795
A_44_P274733	RGD1564315_predicted	XM_343227	362899	0.05	1.127	0.599337
A_42_P524577	Rtn1	NM_053865	116644	0.05	1.127	0.668196
A_44_P717432	TC534199	TC534199		0.05	1.127	0.326232
A_44_P1051396	Wbp5_predicted	XM_215278	294067	0.05	1.127	0.488134
A_44_P746068	TC523053	TC523053		0.05	1.127	0.417068
A_44_P477830	LOC286985	NM_173319	286985	0.05	1.127	0.562489
A_44_P291456	CB547787	CB547787		0.05	1.127	0.420577
A_44_P225109	ENSRNOT00000013942	ENSRNOT00000013942		0.05	1.126	0.493703

A_44_P454429	Fgfr2	XM_001077699		0.05	1.126	0.514447
A_43_P19662	CB547048	CB547048	309855	0.05	1.126	0.314546
A_42_P824178	Slc25a1	NM_017307	29743	0.05	1.126	0.316755
A_44_P899288	TC538431	TC538431		0.05	1.126	0.309058
A_44_P1054436	Tfip11	NM_001008291	288718	0.05	1.126	0.321036
A_44_P900324	Tbl1x_predicted	XM_217623		0.05	1.126	0.433005
A_44_P943171	AW917210	AW917210		0.05	1.126	0.548193
A_44_P558064	A_44_P558064	A_44_P558064		0.05	1.126	0.620869
A_44_P377731	Peflin	NM_001007651	297900	0.05	1.126	0.581718
A_43_P23304	Panx1	NM_199397	315435	0.05	1.126	0.720908
A_42_P524904	Prkcbp1	XM_001071994		0.05	1.126	0.461978
A_44_P103357	RGD1306995_predicted	XM_001069247		0.05	1.126	0.58212
A_44_P420793	RGD1560796_predicted	XM_001053952		0.05	1.126	0.329327
A_42_P470417	RGD1308106_predicted	XM_342009	361719	0.05	1.126	0.308467
A_44_P359104	Morf4l1	NM_001011999	300891	0.05	1.126	0.350526
A_44_P1023173	Cnot10	NM_001007003	316034	0.05	1.126	0.266344
A_44_P1041279	Usp48	NM_198785	362636	0.05	1.126	0.450282
A_44_P1053365	LOC360807	NM_001014128	360807	0.05	1.125	0.385059
A_44_P230723	Trib1	XM_001065461		0.05	1.125	0.396861
A_44_P606874	TC538416	TC538416		0.05	1.125	0.496265
A_44_P497331	AA900088	AA900088		0.05	1.125	0.573927
A_44_P760935	TC536029	TC536029		0.05	1.125	0.644099
A_44_P403058	XM_341409	XM_341409		0.05	1.125	0.32105
A_44_P310792	Cog8_predicted	XM_214673		0.05	1.125	0.411024
A_44_P147751	BQ781097	BQ781097		0.05	1.125	0.603005
A_43_P22205	LOC680470	XM_001057332	680470	0.05	1.125	0.378015
A_44_P423528	Ndufa8	XM_216044		0.05	1.125	0.451277
A_44_P324180	Fyttd1	XM_001081958		0.05	1.125	0.442429
A_42_P568034	Rbks_predicted	XM_343024		0.05	1.124	0.478238
A_44_P450758	Qtrt1	NM_022250	64016	0.05	1.124	0.277205
A_44_P867129	RGD1305773_predicted	XM_213209	287044	0.05	1.124	0.355754
A_43_P10818	AW917957	AW917957		0.05	1.124	0.398317
A_44_P101856	AA997627	AA997627		0.05	1.124	0.267413
A_44_P487874	TC546269	TC546269		0.05	1.124	0.37756
A_44_P789324	CO561445	CO561445		0.05	1.124	0.301522
A_44_P629540	A_44_P629540	A_44_P629540		0.05	1.124	0.304158
A_44_P450278	RGD1564320_predicted	XM_345097		0.05	1.124	0.445489
A_44_P1014527	Alms1_predicted	XM_216189		0.05	1.124	0.381492
A_44_P454993	XM_346127	XM_346127		0.05	1.124	0.315436
A_42_P574411	Thoc3_predicted	XM_237957		0.05	1.124	0.399491
A_44_P492104	RGD1310168_predicted	XM_219540	309186	0.05	1.124	0.347387
A_44_P426524	Creg_predicted	XM_213921		0.05	1.124	0.340568
A_44_P186407	XM_239254	XM_239254		0.05	1.124	0.307791
A_44_P182061	RGD1306925	NM_001024965	501550	0.05	1.124	0.487661
A_43_P22868	Sgol2_predicted	XM_237176	316425	0.05	1.123	0.444936
A_42_P668072	Smad1	NM_013130	25671	0.05	1.123	0.39352
A_43_P14032	RGD1561800_predicted	XM_001054222		0.05	1.123	0.559714
A_44_P358306	Olr193_predicted	NM_001000186	293336	0.05	1.123	0.629096
A_44_P554862	LOC498351	XM_001066807		0.05	1.123	0.371921
A_44_P377611	Tmem60_predicted	XM_216066		0.05	1.123	0.372136
A_44_P121039	Canx	NM_172008	29144	0.05	1.123	0.347402
A_44_P920164	RGD1565561_predicted	XM_001061818		0.05	1.123	0.495472
A_44_P175703	RGD1559726_predicted	XM_225656		0.05	1.123	0.37344
A_44_P898943	TC552287	TC552287		0.05	1.123	0.33961
A_44_P996423	RGD1307672	NM_001008308	294390	0.05	1.123	0.444334
A_44_P150089	Eral1	NM_001013229	363646	0.05	1.123	0.231648
A_44_P499702	Cables2_predicted	XM_230952	311703	0.05	1.123	0.558742
A_44_P468544	Cyb5r1	NM_001013126	304805	0.05	1.123	0.476456
A_44_P267669	RGD1310937_predicted	XM_217149		0.05	1.123	0.434583
A_43_P20713	Klhl20_predicted	XM_222806		0.05	1.123	0.400881
A_44_P198935	Vasp_predicted	XM_341799		0.05	1.123	0.416887
A_44_P368386	A_44_P368386	A_44_P368386		0.05	1.123	0.262648
A_44_P534305	Adamts6_predicted	XM_342184		0.05	1.122	0.684563

A_44_P568584	A_44_P568584	A_44_P568584		0.05	1.122	0.491693
A_44_P172974	Rxra	NM_012805	25271	0.05	1.122	0.362653
A_44_P699830	TC556589	TC556589		0.05	1.122	0.583187
A_44_P530265	LOC287522	XM_220702		0.05	1.122	0.468821
A_43_P19546	RGD1307729_predicted	XM_224295	305955	0.05	1.122	0.250676
A_44_P989677	RGD1304579	NM_001014023	308906	0.05	1.122	0.42935
A_44_P465986	Rab2l	NM_212547	294283	0.05	1.122	0.319356
A_44_P124335	RGD1559936_predicted	XM_221082	303659	0.05	1.122	0.664908
A_44_P305801	MGC125215	NM_001033894	305434	0.05	1.122	0.707832
A_44_P157652	Aes	NM_019220	29466	0.05	1.122	0.511807
A_42_P775256	Ctf1	NM_017129	29201	0.05	1.122	0.557087
A_43_P18601	Rbbp6	XM_219296	308968	0.05	1.122	0.31503
A_44_P124751	MGC116171	NM_001024904	498678	0.05	1.122	0.367334
A_42_P560525	LOC680747	XM_001058684		0.05	1.122	0.427895
A_44_P808218	RGD1311937_predicted	XM_001069629		0.05	1.121	0.415
A_43_P21690	Usp25_predicted	XM_221722		0.05	1.121	0.540168
A_43_P10313	BX883052	BX883052		0.05	1.121	0.660218
A_44_P873794	RGD1565941_predicted	XM_227600	295378	0.05	1.121	0.756729
A_43_P16894	LOC595134	NM_001030054	595134	0.05	1.121	0.432976
A_44_P547957	Dhx8	XM_213460		0.05	1.121	0.326729
A_42_P743310	Atp5f1	NM_134365	171375	0.05	1.121	0.414341
A_44_P213517	RGD1565360_predicted	XM_221630	288240	0.05	1.121	0.325452
A_44_P536873	A_44_P536873	A_44_P536873		0.05	1.121	0.421543
A_44_P515349	Pik3r1	NM_013005	25513	0.05	1.121	0.4529
A_43_P21640	Adprtl1	XM_341326	361046	0.05	1.121	0.301482
A_44_P252345	LOC683519	XM_001066036		0.05	1.121	0.455746
A_44_P312371	Slc30a4	NM_172066	64469	0.05	1.121	0.459993
A_44_P452705	RGD1311757_predicted	XM_233379	313493	0.05	1.121	0.436022
A_44_P394401	Disp2_predicted	XM_230531		0.05	1.121	0.46357
A_42_P723967	Scfd1	NM_019364	54350	0.05	1.121	0.473771
A_44_P194633	Stag2_predicted	XM_233108	313304	0.05	1.121	0.394149
A_44_P191864	Hras	XM_001061671	293621	0.05	1.121	0.306225
A_44_P264833	LOC288654	XM_213773	288654	0.05	1.121	0.500489
A_44_P145023	RGD1305828_predicted	XM_216331	297824	0.05	1.121	0.69616
A_43_P10798	CA507280	CA507280		0.05	1.121	0.533579
A_44_P518518	RGD1564912_predicted	XM_236965		0.05	1.120	0.529258
A_44_P431447	Nfat5_predicted	XM_226436		0.05	1.120	0.322784
A_44_P335175	RGD1311892_predicted	XM_213984	289399	0.05	1.120	0.503159
A_44_P256143	Arsk	XM_345131		0.05	1.120	0.611461
A_44_P915891	TC543595	TC543595		0.05	1.120	0.496505
A_44_P358712	XM_344326	XM_344326		0.05	1.120	0.261329
A_43_P11393	CB547753	CB547753		0.05	1.120	0.500556
A_42_P607061	Diablo	NM_001008292	288753	0.05	1.120	0.353931
A_44_P687109	TC559682	TC559682		0.05	1.120	0.557235
A_44_P303509	RGD1561961_predicted	XM_213926	289181	0.05	1.120	0.577101
A_43_P12011	Cltc	NM_019299	54241	0.05	1.120	0.397341
A_44_P137273	Fbxl7_predicted	XM_342204		0.05	1.120	0.527732
A_44_P216687	AW141280	AW141280		0.05	1.120	0.259961
A_43_P21615	Hic1_predicted	XM_220706		0.05	1.120	0.416867
A_44_P430890	Gpt2_predicted	NM_001012057	307759	0.05	1.120	0.45657
A_44_P334157	AI176460	AI176460		0.05	1.120	0.462902
A_44_P515670	A_44_P515670	A_44_P515670		0.05	1.120	0.669798
A_44_P898857	RGD1307696_predicted	XM_230827		0.05	1.120	0.440418
A_44_P622684	Zswim6	XM_226779		0.05	1.120	0.500772
A_44_P729342	U08214	U08214		0.05	1.120	0.427341
A_43_P10574	Snx11	NM_001012012	303493	0.05	1.120	0.369899
A_44_P513771	RGD1305176_predicted	XM_236612	315987	0.05	1.120	0.489493
A_44_P853384	TC556092	TC556092		0.05	1.119	0.389311
A_44_P149406	Cbr4	NM_182672	359725	0.05	1.119	0.399569
A_43_P16321	Rps5	XM_218293		0.05	1.119	0.369753
A_44_P488312	AI230784	AI230784		0.05	1.119	0.388335
A_44_P430012	AW142402	AW142402		0.05	1.119	0.544711
A_44_P719742	RGD1563508_predicted	XM_001078729		0.05	1.119	0.457562

A_44_P1004142	RGD1562795_predicted	XM_342603		0.05	1.119	0.329067
A_44_P825644	TC541437	TC541437		0.05	1.119	0.662315
A_44_P591170	A_44_P591170	A_44_P591170		0.05	1.119	0.389442
A_44_P487798	Stat2	NM_001011905	288774	0.05	1.119	0.59525
A_44_P1007449	RGD1310159_predicted	XM_222190	304500	0.05	1.119	0.304813
A_44_P455174	RGD1307381	NM_001014116	360498	0.05	1.119	0.261255
A_44_P294087	RGD1305793	XM_001067947		0.05	1.119	0.393384
A_44_P386281	AW434274	AW434274	289924	0.05	1.119	0.647
A_43_P12535	Klf10	NM_031135	81813	0.05	1.119	0.43391
A_44_P1037410	Ophn1_predicted	XM_001068634		0.05	1.119	0.45494
A_44_P182721	RGD1562776_predicted	XM_223623		0.05	1.119	0.3723
A_42_P607939	Chchd1_predicted	XM_341280		0.05	1.119	0.368326
A_44_P686406	CV077424	CV077424	498188	0.05	1.118	0.323845
A_44_P217250	Eno3	NM_012949	25438	0.05	1.118	0.779132
A_44_P203493	Tceb1	NM_022593	64525	0.05	1.118	0.3038
A_43_P10049	Tm2d2	NM_001017444	290833	0.05	1.118	0.412861
A_44_P215720	XM_232746	XM_232746		0.05	1.118	0.296611
A_44_P842364	TC565879	TC565879		0.05	1.118	0.74726
A_44_P580464	TC530262	TC530262		0.05	1.118	0.398909
A_44_P834980	Tollip_predicted	XM_001063419		0.05	1.118	0.347452
A_44_P361548	Prdm15_predicted	XM_239504		0.05	1.118	0.331658
A_44_P483288	Txnl5_predicted	XM_213382		0.05	1.118	0.357108
A_44_P835214	Klhl24	NM_181473	303803	0.05	1.118	0.395623
A_44_P838611	TC540840	TC540840		0.05	1.118	0.56638
A_44_P885356	TC559889	TC559889		0.05	1.118	0.454408
A_43_P10403	LOC499913	NM_001024314	499913	0.05	1.118	0.30121
A_43_P21143	Cul3_predicted	XM_217454		0.05	1.118	0.377249
A_44_P715909	BG668700	BG668700		0.05	1.118	0.470381
A_44_P837237	A_44_P837237	A_44_P837237		0.05	1.117	0.338792
A_44_P531142	BE109637	BE109637	498034	0.05	1.117	0.515845
A_44_P302310	LOC681331	XR_006043	681331	0.05	1.117	0.27454
A_44_P361286	XM_228592	XM_228592		0.05	1.117	0.373995
A_44_P407362	Art5	NM_001013039	259167	0.05	1.117	0.532154
A_44_P449567	Rtn4rl1	NM_181377	303311	0.05	1.117	0.525088
A_44_P445465	RGD1306371_predicted	XM_234083	314061	0.05	1.117	0.377454
A_43_P12209	Stat5b	NM_022380	25126	0.05	1.117	0.364196
A_44_P567088	Mrrf	NM_001008354	311903	0.05	1.117	0.296316
A_43_P12574	Neu1	NM_031522	24591	0.05	1.117	0.48324
A_44_P343913	BF286260	BF286260		0.05	1.117	0.54899
A_43_P14682	H2afz	NM_022674	58940	0.05	1.117	0.534073
A_44_P147600	Npat_predicted	XM_236292		0.05	1.117	0.41843
A_44_P973249	Itgav_predicted	XM_001068715		0.05	1.116	0.430273
A_44_P485094	XM_219046	XM_219046		0.05	1.116	0.466727
A_44_P116316	Ap1b1	NM_017277	29663	0.05	1.116	0.427722
A_44_P302536	LOC368070	XM_347237		0.05	1.116	0.412068
A_44_P1052467	Gosr2	NM_031685	64154	0.05	1.116	0.359862
A_44_P499011	RGD1565712_predicted	XM_341827	361541	0.05	1.116	0.298528
A_44_P281959	RGD1310304_predicted	XM_218006		0.05	1.116	0.575494
A_44_P429267	XM_242940	XM_242940		0.05	1.116	0.358648
A_44_P140514	RGD1563607_predicted	XM_216657		0.05	1.116	0.456688
A_44_P491771	Dncli2	NM_031026	81655	0.05	1.116	0.435641
A_44_P147317	Ap2b1	NM_080583	140670	0.05	1.116	0.443035
A_44_P531063	Map2k1	NM_031643	170851	0.05	1.116	0.366122
A_44_P620591	AA892259	AA892259	292060	0.05	1.115	0.315055
A_44_P484979	Rdh7	NM_133543	360420	0.05	1.115	0.744461
A_44_P222371	Kptn_predicted	XM_217806		0.05	1.115	0.412115
A_44_P459741	Hexa	NM_001004443	300757	0.05	1.115	0.518016
A_44_P918128	TC559787	TC559787		0.05	1.115	0.327389
A_44_P410296	RGD1308165_predicted	XM_342834		0.05	1.115	0.371456
A_44_P317158	RGD1561474_predicted	XM_243478		0.05	1.115	0.327538
A_44_P275761	Otub1_predicted	XM_215178		0.05	1.115	0.313787
A_44_P377593	A_44_P377593	A_44_P377593		0.05	1.115	0.329897
A_44_P526866	Klf5	NM_053394	84410	0.05	1.115	0.342997

A_44_P389053	Synj2bp	NM_022599	64531	0.05	1.115	0.337278
A_44_P527516	RGD1307816_predicted	XM_215702	295446	0.05	1.115	0.511801
A_44_P214891	Dynll2	NM_080697	140734	0.05	1.115	0.401963
A_43_P10107	TC523775	TC523775		0.05	1.115	0.565525
A_44_P909863	AI511173	AI511173		0.05	1.115	0.461592
A_44_P182257	Stx17	NM_145723	252853	0.05	1.115	0.531068
A_42_P759184	LOC361100	XM_341386	361100	0.05	1.115	0.412847
A_44_P142168	Adcyap1	NM_016989	24166	0.05	1.114	0.562424
A_43_P13273	Col5a1	NM_134452	85490	0.05	1.114	0.479259
A_44_P226726	Olr288_predicted	NM_001000231	293590	0.05	1.114	0.560122
A_44_P161374	XM_219537	XM_219537		0.05	1.114	0.589262
A_44_P521711	Map2k6	NM_053703	114495	0.05	1.114	0.828629
A_44_P713970	LOC683328	XR_008246	689530	0.05	1.114	0.540645
A_44_P403316	RGD1308616_predicted	XM_342892	362573	0.05	1.114	0.417095
A_44_P901229	RGD1310257_predicted	XM_001073289		0.05	1.114	0.377806
A_44_P310037	AA901297	AA901297	83579	0.05	1.114	0.540353
A_44_P286471	Sec24a_predicted	XM_213299		0.05	1.114	0.505473
A_44_P1014745	RGD1309729	XM_214418		0.05	1.114	0.341151
A_43_P19276	Cep68_predicted	XM_214106	289822	0.05	1.114	0.261214
A_44_P121477	Mrpl21_predicted	XM_219576		0.05	1.114	0.369499
A_44_P1003694	LOC288913	NM_198728	288913	0.05	1.114	0.427101
A_44_P494768	XM_233953	XM_233953		0.05	1.114	0.326737
A_44_P274650	Zfp691_predicted	XM_233452		0.05	1.114	0.373912
A_44_P506708	XM_346090	XM_346090		0.05	1.114	0.281013
A_43_P17068	RGD1307812	NM_001014170	361946	0.05	1.113	0.482294
A_44_P365999	LOC684046	XM_001068674		0.05	1.113	0.359568
A_44_P496853	LOC290721	XR_007426	290721	0.05	1.113	0.483288
A_43_P20641	CB545948	CB545948	289175	0.05	1.113	0.364606
A_44_P1052463	Gosr2	NM_031685	64154	0.05	1.113	0.358611
A_44_P161709	RGD1563613_predicted	XM_345662		0.05	1.113	0.329332
A_44_P146655	Mef2d	NM_030860	81518	0.05	1.113	0.364305
A_44_P751482	TC526049	TC526049		0.05	1.113	0.392824
A_44_P468694	XM_225042	XM_225042		0.05	1.113	0.482144
A_44_P527858	XM_235589	XM_235589		0.05	1.113	0.694072
A_44_P127294	BM386689	BM386689	297077	0.05	1.113	0.374731
A_44_P418365	BG380154	BG380154	290811	0.05	1.112	0.373631
A_43_P20142	Atp6v0a2_predicted	NM_053775	116455	0.05	1.112	0.645676
A_44_P298026	RGD1563063_predicted	XM_001074682		0.05	1.112	0.700753
A_44_P115798	BI277793	BI277793	25577	0.05	1.112	0.651639
A_44_P252861	RGD1562826_predicted	XM_341096	360822	0.05	1.112	0.401728
A_44_P697681	CO403545	CO403545		0.05	1.112	0.549612
A_44_P553347	Rbm25_predicted	XM_345703		0.05	1.112	0.441281
A_44_P404960	Olr19_predicted	NM_001000117	293081	0.05	1.112	0.324384
A_42_P486517	Cox7a2l_predicted	XM_216635		0.05	1.112	0.431953
A_44_P214922	Vldlr	NM_013155	25696	0.05	1.112	0.48303
A_44_P991335	LOC679870	XM_001055380		0.05	1.112	0.528814
A_44_P1057074	XM_221050	XM_221050		0.05	1.112	0.453178
A_44_P1053316	TC524726	TC524726		0.05	1.112	0.452401
A_44_P1059966	Sdf2_predicted	XM_213377		0.05	1.112	0.350362
A_43_P16021	Lama2_predicted	XM_219866	309368	0.05	1.112	0.378218
A_44_P497355	AW142773	AW142773		0.05	1.112	0.610695
A_44_P166572	LOC688197	XM_001081469		0.05	1.112	0.424518
A_43_P18029	XM_236360	XM_236360		0.05	1.112	0.473605
A_44_P299268	Pscd3	NM_053912	116693	0.05	1.112	0.541627
A_44_P556367	Taf5l	XM_226577		0.05	1.111	0.322227
A_44_P802511	RGD1566319_predicted	XM_001065176		0.05	1.111	0.324267
A_44_P351835	RGD1565536_predicted	XM_230530	311323	0.05	1.111	0.616385
A_43_P10269	Itm2a	NM_001025712	317218	0.05	1.111	0.482753
A_44_P339571	Btg3	NM_019290	54230	0.05	1.111	0.415944
A_44_P529986	XM_342117	XM_342117		0.05	1.111	0.598549
A_44_P164512	Stat1	NM_032612	25124	0.05	1.111	0.505207
A_44_P1046054	Brd4	XM_343175	362844	0.05	1.111	0.286963
A_44_P957296	AW143366	AW143366		0.05	1.111	0.554584

A_44_P223607	AW918180	AW918180		0.05	1.111	0.483706
A_44_P883338	ENSRNOT00000048826	ENSRNOT00000048826		0.05	1.111	0.398665
A_44_P935947	Hnrpa2b1_predicted	XM_001055961		0.05	1.111	0.333636
A_43_P13976	Ppib	NM_022536	64367	0.05	1.111	0.388701
A_44_P174057	AI102773	AI102773	311881	0.05	1.111	0.715027
A_44_P464504	Slc25a19	NM_001007674	303676	0.05	1.111	0.261573
A_44_P375031	LOC365166	XR_007972		0.05	1.110	0.545888
A_44_P975418	CK599755	CK599755		0.05	1.110	0.529976
A_43_P11146	Lyplal1_predicted	XM_213970		0.05	1.110	0.308146
A_44_P1021621	BF566013	BF566013		0.05	1.110	0.43481
A_44_P465931	Clic1	NM_001002807	406864	0.05	1.110	0.395184
A_44_P259644	LOC497934	NM_001017474	497934	0.05	1.110	0.377434
A_44_P418753	Rab11fip2_predicted	XM_001073638		0.05	1.110	0.502731
A_42_P780932	Fcna	NM_031348	83517	0.05	1.110	0.602042
A_44_P134584	Arhgdia	NM_001007005	360678	0.05	1.110	0.42164
A_44_P274762	RGD1306568	NM_001013963	302890	0.05	1.110	0.308365
A_44_P376279	CK478298	CK478298		0.05	1.110	0.587966
A_43_P19600	Zfp7_predicted	XM_001075493		0.05	1.109	0.543732
A_44_P402948	TC540893	TC540893		0.05	1.109	0.640788
A_44_P316520	LOC687779	XM_001080118		0.05	1.109	0.287832
A_44_P853449	TC539606	TC539606		0.04	1.109	0.560122
A_44_P101162	CB546932	CB546932	315150	0.04	1.109	0.426638
A_44_P550867	A_44_P550867	A_44_P550867		0.04	1.109	0.518282
A_44_P913975	A_44_P913975	A_44_P913975		0.04	1.109	0.444633
A_44_P638377	TC523393	TC523393		0.04	1.109	0.505641
A_44_P312606	BG153336	BG153336	361825	0.04	1.109	0.485316
A_44_P405689	RGD1565591_predicted	XM_233731	313757	0.04	1.109	0.451594
A_43_P14915	C6	NM_176074	24237	0.04	1.109	0.546682
A_44_P215582	RGD1310660	NM_001009316	296470	0.04	1.109	0.477864
A_44_P713894	LOC689823	XM_001072159	689823	0.04	1.109	0.517857
A_42_P541660	Nfatc4	XM_240184		0.04	1.109	0.53115
A_42_P698815	Zcchc9	NM_001013156	309986	0.04	1.109	0.293436
A_44_P525318	Pcdhb14_predicted	XM_001055753		0.04	1.109	0.513468
A_44_P470430	Fap	NM_138850	192203	0.04	1.108	0.830337
A_44_P1001772	Rnf41	NM_001012195	362814	0.04	1.108	0.357706
A_44_P406491	AA874794	AA874794	117089	0.04	1.108	0.479515
A_44_P448193	RGD1304719	NM_001037769	299341	0.04	1.108	0.544616
A_44_P206595	Zdhhc8	NM_001039021	303796	0.04	1.108	0.397956
A_44_P210809	RGD1308377_predicted	XM_225093		0.04	1.108	0.634221
A_44_P255482	Gatad2a	NM_001013881	290669	0.04	1.108	0.392773
A_44_P404824	Map1lc3a	NM_199500	362245	0.04	1.108	0.400607
A_44_P141075	AI011151	AI011151		0.04	1.108	0.473485
A_44_P189674	XM_222600	XM_222600		0.04	1.108	0.408072
A_44_P135434	XM_222925	XM_222925		0.04	1.108	0.429834
A_43_P20851	Ppfibp2	XM_219201	308918	0.04	1.108	0.474498
A_44_P561643	TC540526	TC540526		0.04	1.108	0.538623
A_44_P267185	LOC686610	XM_001071365		0.04	1.108	0.420872
A_44_P276451	Btbd14b	NM_134413	171454	0.04	1.108	0.544953
A_44_P116758	LOC361309	NM_001014148	361309	0.04	1.108	0.445011
A_44_P779338	TC544476	TC544476		0.04	1.107	0.484652
A_42_P769969	LOC682212	XM_001060462		0.04	1.107	0.475474
A_44_P555890	Mnt_predicted	XM_220698		0.04	1.107	0.352848
A_44_P633631	AW915807	AW915807		0.04	1.107	0.690979
A_42_P601490	Usp31_predicted	XM_219292		0.04	1.107	0.59334
A_44_P116230	Slc12a9	NM_134405	171443	0.04	1.107	0.329327
A_44_P440905	Ercc3	NM_001031644	291703	0.04	1.107	0.489401
A_44_P318607	Nupl1	NM_139091	245922	0.04	1.107	0.511135
A_44_P234077	XM_228776	XM_228776		0.04	1.107	0.374068
A_44_P468756	A_44_P468756	A_44_P468756		0.04	1.106	0.409536
A_42_P686078	AW143067	AW143067		0.04	1.106	0.589137
A_43_P14574	Vamp3	NM_057097	29528	0.04	1.106	0.339919
A_44_P292900	LOC309016	XM_219377	309016	0.04	1.106	0.572679
A_44_P941089	Rnf26_predicted	XM_001066398		0.04	1.106	0.407456

A_44_P342562	Fgd1	NM_001037546	363460	0.04	1.106	0.513246
A_44_P975488	TC554996	TC554996		0.04	1.106	0.544253
A_43_P20723	Phactr3	NM_214459	362284	0.04	1.106	0.364504
A_44_P244710	Usp9x_predicted	XM_001056701		0.04	1.106	0.458954
A_43_P12423	Hif1a	NM_024359	29560	0.04	1.106	0.424992
A_44_P177575	Olr597_predicted	NM_001000331	295827	0.04	1.106	0.39403
A_44_P119177	Parp2_predicted	XM_214157		0.04	1.106	0.508535
A_44_P182975	Rpgr	XM_346270		0.04	1.105	0.636911
A_44_P121908	A_44_P121908	A_44_P121908		0.04	1.105	0.596531
A_44_P216497	LOC686240	XM_001073036		0.04	1.105	0.456729
A_43_P10467	Vkorc1l1	NM_203338	399684	0.04	1.105	0.456349
A_44_P135260	Hapln3	NM_001008559	308773	0.04	1.105	0.569313
A_43_P16717	Cdc2l5	XM_225404	306998	0.04	1.105	0.496963
A_44_P264569	LOC498525	AY325260	498525	0.04	1.105	0.364725
A_44_P283339	Tmed3	NM_001004249	300888	0.04	1.105	0.587126
A_44_P699859	TC539563	TC539563		0.04	1.105	0.540327
A_44_P470961	Ell2	XM_226624	309918	0.04	1.105	0.450962
A_44_P195267	BF391710	BF391710		0.04	1.105	0.505641
A_43_P10213	Etnk1_predicted	XM_001074330		0.04	1.105	0.547007
A_44_P837227	LOC687994	XM_001080810		0.04	1.105	0.51848
A_44_P310366	LOC308846	XM_001065167		0.04	1.105	0.533189
A_42_P753675	Arhgdig_predicted	XM_340775		0.04	1.105	0.555871
A_44_P529477	Prdm2_mapped	U17837		0.04	1.105	0.322167
A_44_P728932	ENSRNOT00000028742	ENSRNOT00000028742		0.04	1.105	0.350169
A_43_P22996	RGD1307679	NM_001009599	311166	0.04	1.105	0.442438
A_44_P506751	Abcf3	NM_001011896	287982	0.04	1.105	0.370559
A_44_P288707	LOC684410	XM_001070275		0.04	1.105	0.539074
A_44_P349680	RGD1563296_predicted	XM_001076141		0.04	1.105	0.549663
A_44_P636923	RGD1562214_predicted	XM_001057512		0.04	1.104	0.311727
A_43_P23215	Smpdl3b	NM_001025737	362619	0.04	1.104	0.552263
A_44_P945157	TC554961	TC554961		0.04	1.104	0.78247
A_44_P459338	Fxna	NM_184050	373544	0.04	1.104	0.65337
A_44_P944221	ENSRNOT00000021215	ENSRNOT00000021215		0.04	1.104	0.470756
A_44_P207215	Nck2_predicted	XM_237115		0.04	1.104	0.32153
A_43_P19072	RGD1305866_predicted	XM_343265	362937	0.04	1.104	0.312194
A_44_P508261	LOC687625	XM_001074320		0.04	1.104	0.496343
A_44_P471270	XM_232762	XM_232762		0.04	1.104	0.389442
A_44_P121935	Hdac10	XM_001053786		0.04	1.104	0.372589
A_44_P853208	LOC498145	NM_001017485	498145	0.04	1.104	0.481447
A_44_P191593	Iag2	NM_053946	116967	0.04	1.104	0.558699
A_44_P451446	AI029012	AI029012	24792	0.04	1.103	0.681455
A_42_P718324	BG667212	BG667212	360990	0.04	1.103	0.473824
A_44_P110120	RGD1563818_predicted	XM_215940	296356	0.04	1.103	0.820368
A_44_P325005	Pak1	NM_017198	29431	0.04	1.103	0.454194
A_44_P485409	A_44_P485409	A_44_P485409		0.04	1.103	0.46509
A_44_P359074	XM_234875	XM_234875		0.04	1.103	0.454562
A_44_P121749	XM_229059	XM_229059		0.04	1.103	0.729333
A_44_P992404	Cpsf5	NM_001039004	291877	0.04	1.103	0.544316
A_44_P541187	Tpcn2_predicted	XM_219555		0.04	1.103	0.435641
A_44_P119682	XM_341014	XM_341014		0.04	1.103	0.299461
A_44_P653988	TC559283	TC559283		0.04	1.103	0.452559
A_44_P999630	Cdc16	NM_001024744	290875	0.04	1.103	0.535624
A_42_P614520	AW917927	AW917927		0.04	1.103	0.45087
A_44_P1054085	LOC681059	XM_001060127	681059	0.04	1.103	0.395419
A_44_P290016	BG666843	BG666843		0.04	1.103	0.559499
A_44_P326108	Mrrf	NM_001008354	311903	0.04	1.102	0.389629
A_43_P11481	Me1	NM_012600	24552	0.04	1.102	0.633153
A_42_P589680	Tarbp2	NM_001034941	363006	0.04	1.102	0.349575
A_44_P322425	AW142643	AW142643		0.04	1.102	0.720771
A_44_P895064	RGD1562692_predicted	XM_001069337		0.04	1.102	0.49396
A_44_P364447	RGD1309930	NM_001014102	316426	0.04	1.102	0.687458
A_42_P627394	Adam10	XM_001054737		0.04	1.102	0.512675
A_44_P163599	XM_344897	XM_344897		0.04	1.102	0.417526

A_44_P522477	Tmem106b	NM_001004267	312132	0.04	1.102	0.667635
A_44_P820184	Yy1	XM_001069615		0.04	1.102	0.567073
A_44_P224566	Zfp2	U78123		0.04	1.102	0.514355
A_44_P168842	LOC300429	XR_009092	300429	0.04	1.102	0.462601
A_44_P341801	Hdlbp	NM_172039	64474	0.04	1.102	0.447037
A_44_P277466	Abca3	XM_220219	302973	0.04	1.102	0.361844
A_44_P249784	AI406369	AI406369	290705	0.04	1.101	0.498735
A_44_P358576	A_44_P358576	A_44_P358576		0.04	1.101	0.389493
A_44_P501923	XM_346066	XM_346066		0.04	1.101	0.646164
A_44_P997787	RGD1562579_predicted	XM_213799	288717	0.04	1.101	0.602247
A_44_P191615	Taok1	NM_173327	286993	0.04	1.101	0.600918
A_42_P466362	Adcy8	NM_017142	29241	0.04	1.101	0.686252
A_44_P546083	A_44_P546083	A_44_P546083		0.04	1.101	0.558742
A_44_P310658	Ppp4r2_predicted	XM_216225		0.04	1.101	0.431692
A_44_P792192	TC557253	TC557253		0.04	1.101	0.566585
A_44_P651694	A_44_P651694	A_44_P651694		0.04	1.101	0.454385
A_44_P248144	RGD1560335_predicted	XM_223731	305626	0.04	1.101	0.510974
A_44_P271062	RGD1566224_predicted	XM_575742		0.04	1.101	0.59546
A_44_P540067	Rbm10	NM_152861	64510	0.04	1.101	0.419418
A_44_P144232	Fgl2	NM_053455	84586	0.04	1.101	0.805339
A_44_P295133	RGD1563029_predicted	XM_219888		0.04	1.100	0.374633
A_44_P190294	BE099508	BE099508	315019	0.04	1.100	0.386893
A_44_P159254	A_44_P159254	A_44_P159254		0.04	1.100	0.517472
A_44_P403410	Rbm9_predicted	XM_343281		0.04	1.100	0.375496
A_44_P256199	CB547883	CB547883		0.04	1.100	0.553346
A_44_P468873	LOC363484	XM_001055438		0.04	1.100	0.83769
A_44_P931132	BC098828	BC098828		0.04	1.100	0.400615
A_44_P291872	BF281523	BF281523		0.04	1.100	0.465647
A_44_P461678	RGD1564887_predicted	XM_574677	499363	0.04	1.100	0.730055
A_44_P457447	RGD1305283_predicted	XM_343501	363162	0.04	1.100	0.560806
A_44_P210240	P4hb	NM_012998	25506	0.04	1.100	0.467263
A_44_P243377	RGD1566190_predicted	XM_223007		0.04	1.100	0.452862
A_44_P544846	Mkks	NM_001008353	311456	0.04	1.100	0.665041
A_44_P1045960	Gng5	NM_024377	79218	0.04	1.100	0.443315
A_42_P737265	RGD1311188_predicted	XM_235439	315088	0.04	1.100	0.559071
A_42_P519018	Mtmr9	NM_001005761	282584	0.04	1.100	0.365196
A_42_P745147	Brca2	NM_031542	360254	0.04	1.100	0.585228
A_44_P383596	AW915928	AW915928		0.04	1.099	0.524795
A_43_P16214	Dnah11	XM_234720	117253	0.04	1.099	0.523548
A_44_P191838	Arhgef5	XM_342676	140898	0.04	1.099	0.577736
A_44_P1003517	Psmd6	NM_198730	289924	0.04	1.099	0.43003
A_44_P541461	Alas2	NM_013197	25748	0.04	1.099	0.724257
A_43_P10587	Ube2m_predicted	XM_341790		0.04	1.099	0.385136
A_44_P293948	Ghitm	NM_001005908	290596	0.04	1.099	0.645662
A_44_P156665	XM_218761	XM_218761		0.04	1.099	0.438185
A_44_P623384	TC519225	TC519225		0.04	1.099	0.603767
A_44_P824706	TC546354	TC546354		0.04	1.099	0.40204
A_43_P17793	Angel2_predicted	XM_223023	305035	0.04	1.099	0.540104
A_44_P256181	LOC685258	XM_001062468	685258	0.04	1.099	0.656036
A_44_P852878	DV714770	DV714770		0.04	1.099	0.523644
A_44_P475661	RGD1308014_predicted	XM_223075	289382	0.04	1.099	0.462625
A_42_P823306	Snx1	NM_053411	84471	0.04	1.099	0.532262
A_44_P220541	LOC685258	XM_001062468	685258	0.04	1.099	0.490338
A_43_P12545	Ube2d3	NM_031237	81920	0.04	1.099	0.437618
A_44_P1017763	Rpp21	NM_001002831	406230	0.04	1.098	0.397128
A_43_P20321	Ccdc22_predicted	XM_228770	317381	0.04	1.098	0.39576
A_44_P555021	Cdc42	NM_171994	64465	0.04	1.098	0.416772
A_43_P16800	XM_214428	XM_214428		0.04	1.098	0.351892
A_44_P919148	Mxd4_predicted	XM_001065075		0.04	1.098	0.652369
A_44_P715197	DV720643	DV720643		0.04	1.098	0.577558
A_43_P19529	Thrap3	NM_001009693	313591	0.04	1.098	0.495584
A_43_P18800	Cc2d1b	XM_233342	313478	0.04	1.098	0.334712
A_44_P475040	AI104878	AI104878	361165	0.04	1.098	0.388968

A_44_P986792	Sorl1_predicted	XM_001065506		0.04	1.098	0.671268
A_44_P945741	TC523222	TC523222		0.04	1.098	0.645208
A_44_P730568	TC558100	TC558100		0.04	1.098	0.468864
A_42_P503597	Mrpl30_predicted	XM_217378		0.04	1.098	0.429202
A_44_P377653	A_44_P377653	A_44_P377653		0.04	1.098	0.382538
A_43_P19401	RGD1311429_predicted	XM_001081571		0.04	1.098	0.55601
A_44_P182934	Rpl35a	NM_021264	57809	0.04	1.098	0.387803
A_44_P179972	Strn3	NM_001029897	114520	0.04	1.098	0.392902
A_44_P377629	LOC680457	XM_001057268	680457	0.04	1.098	0.395214
A_42_P674856	Mesdc1	NM_001013149	308795	0.04	1.098	0.456875
A_42_P471507	Unc50	NM_138919	192356	0.04	1.098	0.602168
A_44_P605618	RGD1561254_predicted	XM_574497	499210	0.04	1.097	0.712933
A_44_P231269	Pdk4	NM_053551	89813	0.04	1.097	0.835371
A_42_P631242	AW915015	AW915015		0.04	1.097	0.727504
A_44_P110044	RGD1565561_predicted	XM_001061818		0.04	1.097	0.535401
A_44_P538695	Ephb2_predicted	XM_233574	313633	0.04	1.097	0.624121
A_43_P18261	Mesdc2	NM_001008345	308796	0.04	1.097	0.408171
A_43_P15497	Msx2	NM_012982	25483	0.04	1.097	0.59334
A_44_P898176	LOC684978	XM_001061754		0.04	1.097	0.32245
A_44_P109700	A_44_P109700	A_44_P109700		0.04	1.097	0.43282
A_44_P156949	Plekhk1_predicted	XM_228115		0.04	1.097	0.481536
A_44_P1054864	Arcn1	NM_001007662	300674	0.04	1.097	0.404709
A_44_P552875	Dtx2	XM_347157		0.04	1.097	0.340644
A_44_P144775	Ccdc25_predicted	XM_341340		0.04	1.097	0.501784
A_44_P511128	Nusap1_predicted	XM_230478		0.04	1.097	0.609915
A_44_P326176	RGD1563628_predicted	XM_222244	304554	0.04	1.096	0.60276
A_44_P260574	A_44_P260574	A_44_P260574		0.04	1.096	0.463113
A_44_P452492	Hspa8	NM_024351	24468	0.04	1.096	0.554584
A_44_P511379	RGD1310503_predicted	XM_236221	315645	0.04	1.096	0.39501
A_44_P991459	RGD1305156	NM_001024876	299957	0.04	1.096	0.562769
A_42_P543694	LOC500901	XM_001073789		0.04	1.096	0.518773
A_44_P316592	Pigt_predicted	XM_215919		0.04	1.096	0.433763
A_44_P428560	Olr883_predicted	NM_001001358	288803	0.04	1.096	0.711386
A_44_P378225	Pycrl	NM_001011993	300035	0.04	1.096	0.502554
A_44_P107541	Siglec5_predicted	XM_218613		0.04	1.096	0.705831
A_44_P424541	LOC299827	XM_216902		0.04	1.096	0.583433
A_44_P534808	LOC682422	XM_001061459		0.04	1.096	0.623578
A_44_P165543	Plcb2	NM_053478	85240	0.04	1.096	0.707134
A_44_P177414	MGC72581	NM_199375	290635	0.04	1.096	0.379484
A_44_P119435	Creb3	NM_001013092	298400	0.04	1.096	0.36466
A_44_P309215	Tnip2	NM_001024771	305451	0.04	1.096	0.452747
A_44_P440815	Rai17_predicted	XM_341389		0.04	1.095	0.546568
A_44_P367551	Sqstm1	NM_175843		0.04	1.095	0.544964
A_44_P446886	BI300764	BI300764	300813	0.04	1.095	0.603662
A_44_P966888	A_44_P966888	A_44_P966888		0.04	1.095	0.534073
A_44_P469040	RGD1560783_predicted	XM_001078208		0.04	1.095	0.580101
A_44_P311334	Pde4d	L27060	24627	0.04	1.095	0.713371
A_44_P530130	RGD1311562_predicted	XM_216730		0.04	1.095	0.528013
A_44_P240248	Nfib	XM_342854		0.04	1.095	0.467177
A_44_P998128	Brd3_predicted	XM_342396		0.04	1.095	0.425115
A_43_P21173	Fbxl14_predicted	XM_232330	312675	0.04	1.095	0.432571
A_42_P665627	Mgat5	NM_023095	65271	0.04	1.095	0.418801
A_44_P375469	XM_233518	XM_233518		0.04	1.095	0.511578
A_44_P131012	LOC362304	NM_001014186	362304	0.04	1.095	0.49321
A_44_P408818	BQ207606	BQ207606	309458	0.04	1.095	0.672931
A_43_P11759	Rps26	NM_013224	27139	0.04	1.095	0.510919
A_44_P821676	LOC499602	NM_001025039	499602	0.04	1.095	0.670147
A_44_P476663	Asb3_predicted	XM_344277		0.04	1.095	0.650509
A_43_P16389	Lamb3	XM_223087	305078	0.04	1.095	0.571497
A_44_P126506	LOC688673	XM_001067842	681879	0.04	1.095	0.553274
A_43_P10430	TC538235	TC538235		0.04	1.095	0.490167
A_44_P822769	TC538973	TC538973		0.04	1.095	0.505391
A_44_P161797	RGD1308012_predicted	XM_343313		0.04	1.095	0.488555

A_44_P265709	Il2rb	NM_013195	25746	0.04	1.095	0.689345
A_44_P760697	LOC363251	NM_001014217	363251	0.04	1.094	0.424019
A_43_P18032	CB544891	CB544891		0.04	1.094	0.494631
A_44_P220394	RGD1560099_predicted	XM_228526	302388	0.04	1.094	0.466399
A_44_P291157	XM_237184	XM_237184		0.04	1.094	0.602511
A_44_P169878	BE107159	BE107159	313323	0.04	1.094	0.813233
A_44_P577924	Fbxo42_predicted	XM_342963		0.04	1.094	0.457167
A_44_P125443	BF289687	BF289687		0.04	1.094	0.430365
A_43_P10757	RGD1560129_predicted	XM_001054191		0.04	1.094	0.498513
A_44_P225029	RGD1311952_predicted	XM_230941	311692	0.04	1.094	0.546791
A_44_P402731	U78143	U78143		0.04	1.094	0.522471
A_44_P344234	Olr172_predicted	NM_001000177	293319	0.04	1.094	0.680691
A_44_P737521	TC548798	TC548798		0.04	1.094	0.518461
A_44_P729183	A_44_P729183	A_44_P729183		0.04	1.094	0.37146
A_44_P1013485	RGD1307254	NM_001024875	298712	0.04	1.094	0.539642
A_44_P518050	Sf3a1_predicted	XM_223566		0.04	1.094	0.523449
A_43_P18817	LOC684302	XM_001069325		0.04	1.094	0.346292
A_44_P257501	Dzf17	U78145	654808	0.04	1.093	0.671487
A_43_P17571	AY325216	AY325216		0.04	1.093	0.645204
A_44_P268325	Ccni_predicted	XM_214007		0.04	1.093	0.544516
A_44_P312890	Atg12	NM_001038495	361321	0.04	1.093	0.547529
A_44_P310714	Sv2b	NM_057207	117556	0.04	1.093	0.623013
A_44_P407653	RGD1566416_predicted	XM_223532	305448	0.04	1.093	0.465911
A_43_P17264	RGD1565549_predicted	XM_240329	306254	0.04	1.093	0.374084
A_42_P797558	Mark2	NM_021699	60328	0.04	1.093	0.408366
A_44_P1010281	RGD1309414_predicted	XM_341279	361004	0.04	1.093	0.501629
A_44_P337294	Dci	NM_017306	29740	0.04	1.093	0.585564
A_42_P700089	Bat4	NM_001034157	415064	0.04	1.093	0.511488
A_43_P20397	XM_230770	XM_230770		0.04	1.093	0.627537
A_44_P359957	BU946565	BU946565		0.04	1.093	0.60212
A_44_P356391	Blk	NM_001025751	364403	0.04	1.093	0.734702
A_44_P837644	Ankfy1_predicted	XM_001080190		0.04	1.093	0.624109
A_44_P520725	Dnajc6_predicted	XM_233231		0.04	1.093	0.584566
A_44_P527556	Taf7_predicted	XM_226031		0.04	1.092	0.558432
A_44_P484579	Capon	NM_138922	192363	0.04	1.092	0.773281
A_44_P112788	Bcl2l2	NM_021850	60434	0.04	1.092	0.537759
A_44_P382611	RGD1304758	NM_001031651	303567	0.04	1.092	0.592309
A_44_P699044	TC541991	TC541991		0.04	1.092	0.572629
A_44_P411162	BF287028	BF287028	689226	0.04	1.092	0.599221
A_44_P994116	Ttbk1_predicted	XM_230495		0.04	1.092	0.532097
A_44_P321669	Map3k3_predicted	XM_221034		0.04	1.092	0.595391
A_44_P180017	Fmr1	NM_052804	24948	0.04	1.092	0.47122
A_44_P651772	RGD1306538	NM_001014024	309000	0.04	1.092	0.462985
A_43_P12590	Clcn7	NM_031568	29233	0.04	1.092	0.413332
A_43_P18610	RGD1563235_predicted	XM_226575		0.04	1.092	0.421543
A_44_P461587	RGD1311142_predicted	XM_341847	361564	0.04	1.092	0.6026
A_44_P433603	XM_219517	XM_219517		0.04	1.092	0.413941
A_44_P456852	Ddx1	NM_053414	84474	0.04	1.092	0.54451
A_44_P186883	Clcnkb	NM_173103	79430	0.04	1.092	0.540221
A_43_P18254	Bcar3_predicted	XM_227657		0.04	1.092	0.562655
A_44_P539412	BF389611	BF389611		0.04	1.092	0.608656
A_44_P684162	TC555852	TC555852		0.04	1.092	0.48303
A_44_P1013472	LOC300173	XM_217027	300173	0.04	1.092	0.483865
A_44_P368263	ENSRNOT00000003392	ENSRNOT00000003392		0.04	1.092	0.446181
A_44_P958051	ENSRNOT00000008375	ENSRNOT00000008375		0.04	1.092	0.617053
A_44_P405679	Rap1ga1	XM_233609	313644	0.04	1.091	0.636302
A_44_P534728	LOC363174	XM_343514	363174	0.04	1.091	0.6396
A_44_P110053	Lrrc16_predicted	XM_225336	306941	0.04	1.091	0.68895
A_43_P20639	Spsb2	NM_001009660	297592	0.04	1.091	0.464549
A_44_P759334	Dr1	NM_001011914	289881	0.04	1.091	0.456729
A_44_P726736	AI136297	AI136297		0.04	1.091	0.543732
A_43_P13106	Khdrbs1	NM_130405	117268	0.04	1.091	0.576705
A_44_P543780	RGD1311198_predicted	XM_226412		0.04	1.091	0.585305

A_44_P389339	XM_225567	XM_225567		0.04	1.091	0.761883
A_43_P13109	Cyp26a1	NM_130408	154985	0.04	1.091	0.688921
A_44_P645099	A_44_P645099	A_44_P645099		0.04	1.091	0.674861
A_44_P300249	XM_344000	XM_344000		0.04	1.091	0.563732
A_42_P726091	BX883048	BX883048		0.04	1.091	0.692027
A_44_P138185	Ankmy2_predicted	XM_234115		0.04	1.091	0.35327
A_44_P430978	Pou4f1	XM_341372	114503	0.04	1.091	0.588049
A_44_P808679	TC541828	TC541828		0.04	1.091	0.554441
A_42_P814765	Tm7sf2	NM_001013071	293688	0.04	1.091	0.66798
A_44_P193990	RAMP4	NM_030835	80881	0.04	1.091	0.497712
A_44_P442873	Becn1	NM_053739	114558	0.04	1.090	0.493692
A_44_P529947	Kif3b_predicted	XM_215883		0.04	1.090	0.495882
A_44_P468193	Mxd3	NM_145773	252915	0.04	1.090	0.515959
A_44_P143247	RGD1565602_predicted	XM_239761		0.04	1.090	0.566604
A_43_P12780	Agps	NM_053350	84114	0.04	1.090	0.613259
A_42_P722761	Tbc1d17_predicted	XM_214940		0.04	1.090	0.447877
A_44_P232016	Slc9a7_predicted	XM_228416		0.04	1.090	0.565116
A_44_P524272	LOC317312	NM_001014107	317312	0.04	1.090	0.534689
A_44_P386099	AI175475	AI175475	300092	0.04	1.090	0.574986
A_44_P380218	RGD1564906_predicted	XM_576039	500661	0.04	1.090	0.784673
A_42_P648597	Htatip	NM_001005872	192218	0.04	1.090	0.493647
A_44_P466614	Lactb2	NM_001024247	297768	0.04	1.090	0.736791
A_44_P188869	AW915311	AW915311	309922	0.04	1.090	0.70471
A_42_P719221	Vip	XM_217838	117064	0.04	1.090	0.699986
A_44_P347938	Grinl1a	NM_183402	192147	0.04	1.090	0.434115
A_44_P747905	TC547118	TC547118		0.04	1.090	0.596518
A_44_P634724	AW143399	AW143399		0.04	1.090	0.614426
A_43_P22299	CB547530	CB547530	302986	0.04	1.090	0.598163
A_44_P221056	Ralbp1	NM_032067	84014	0.04	1.090	0.413558
A_44_P228932	Pde4a	NM_013101	25638	0.04	1.090	0.532144
A_44_P230960	AW144271	AW144271		0.04	1.090	0.543853
A_44_P131915	AI177503	AI177503	117056	0.04	1.089	0.501151
A_43_P21538	XM_219336	XM_219336		0.04	1.089	0.365467
A_43_P14161	Rpl36a	NM_031105	81769	0.04	1.089	0.544171
A_44_P524711	Anubl1	XM_001054583		0.04	1.089	0.679616
A_44_P1038266	RGD1304816_predicted	XM_342846	362528	0.04	1.089	0.506362
A_44_P424069	Zfp84_predicted	XM_218463		0.04	1.089	0.417015
A_42_P755546	LOC498225	NM_001017489	498225	0.04	1.089	0.429533
A_44_P745245	Dullard	XM_001079431		0.04	1.089	0.504412
A_44_P670842	TC530834	TC530834		0.04	1.089	0.509867
A_44_P596814	TC551638	TC551638		0.04	1.089	0.563411
A_44_P213535	Dvl3_predicted	XM_221304		0.04	1.089	0.504414
A_44_P274374	Rfxank	NM_001013136	306353	0.04	1.089	0.54606
A_44_P494047	Olr252_predicted	NM_001000219	293398	0.04	1.089	0.695516
A_44_P413265	AA875527	AA875527		0.04	1.089	0.51032
A_44_P622576	TC556283	TC556283		0.04	1.088	0.384801
A_44_P260948	BI289820	BI289820	500200	0.04	1.088	0.68065
A_44_P275523	AW142611	AW142611	361351	0.04	1.088	0.546785
A_44_P1018067	RGD1309550	XM_216781	299295	0.04	1.088	0.534967
A_44_P463745	Tnfrsf6	NM_139194	246097	0.04	1.088	0.726172
A_44_P269984	LOC684198	XM_001068840		0.04	1.088	0.632855
A_43_P11856	Cox4i1	NM_017202	29445	0.04	1.088	0.64173
A_44_P1002407	AI137378	AI137378		0.04	1.088	0.529618
A_44_P747587	TC528506	TC528506		0.04	1.088	0.676052
A_44_P223009	CA506853	CA506853	308870	0.04	1.088	0.636252
A_44_P283864	LOC311134	XM_001066067		0.04	1.088	0.565014
A_44_P363448	XM_226715	XM_226715		0.04	1.088	0.671881
A_43_P12611	Pten	NM_031606	50557	0.04	1.088	0.628066
A_44_P775747	LOC679137	XM_001055330		0.04	1.088	0.421021
A_44_P840798	Eya3_predicted	XM_001064052		0.04	1.088	0.613199
A_43_P17326	LOC310756	XM_227549		0.04	1.088	0.533446
A_44_P999488	Bnip1	NM_080897	140932	0.04	1.088	0.567864
A_44_P472442	Bnip3	NM_053420	84480	0.04	1.088	0.574363

A_44_P1023146	Gtf2b	NM_031041	81673	0.04	1.088	0.643225
A_43_P19499	RGD1562321_predicted	XM_230665		0.04	1.088	0.448893
A_44_P808635	TC525241	TC525241		0.04	1.088	0.542748
A_43_P13509	Slc39a13	NM_001039196	295928	0.04	1.088	0.417514
A_44_P576312	TC552847	TC552847		0.04	1.088	0.741722
A_44_P469057	RGD1561527_predicted	XM_236973		0.04	1.087	0.505641
A_44_P360761	Actr3	NM_031068	81732	0.04	1.087	0.648034
A_44_P593508	RGD1309765_predicted	XM_001059837		0.04	1.087	0.716594
A_44_P484836	Tjp2	NM_053773	115769	0.04	1.087	0.436533
A_44_P975447	TC554993	TC554993		0.04	1.087	0.482294
A_44_P384223	Arf4	NM_024151	79120	0.04	1.087	0.63454
A_44_P435683	Olr745_predicted	NM_001000578	366123	0.04	1.087	0.693629
A_44_P1022403	MGC94190	NM_001004204	288616	0.04	1.087	0.50341
A_42_P773606	RGD1562091_predicted	XM_214867		0.04	1.087	0.557471
A_44_P547850	Soat	NM_198049	289459	0.04	1.087	0.608018
A_44_P180229	XM_341055	XM_341055		0.04	1.087	0.737553
A_44_P494543	Cep1_predicted	XM_231168		0.04	1.087	0.486469
A_43_P18977	Cenpc1	NM_001004098	305270	0.04	1.086	0.449619
A_44_P959764	A_44_P959764	A_44_P959764		0.04	1.086	0.538216
A_44_P333168	RGD1565149_predicted	XM_226551		0.04	1.086	0.716594
A_44_P561486	TC522678	TC522678		0.04	1.086	0.50241
A_44_P473653	LOC300506	XR_005718		0.04	1.086	0.815053
A_42_P634259	AA956352	AA956352		0.04	1.086	0.629446
A_44_P198333	XM_341948	XM_341948		0.04	1.086	0.461139
A_44_P301799	Ryk	NM_080402	140585	0.04	1.086	0.543583
A_44_P654666	TC545195	TC545195		0.04	1.086	0.469826
A_44_P415149	XM_343384	XM_343384		0.04	1.086	0.427109
A_42_P617594	Unc119	NM_017188	29402	0.04	1.085	0.738061
A_44_P351766	MGC124992	XM_001077253	499697	0.04	1.085	0.48121
A_43_P11752	Maob	NM_013198	25750	0.04	1.085	0.451671
A_44_P170755	Prkar1b	NM_001033679	25521	0.04	1.085	0.599135
A_44_P340448	Usp7	NM_001024790	360471	0.04	1.085	0.46302
A_44_P546823	RGD1305240_predicted	XM_341861		0.04	1.085	0.450318
A_44_P821839	A_44_P821839	A_44_P821839		0.04	1.085	0.460478
A_42_P669639	RGD1309821_predicted	XM_345524		0.04	1.085	0.654896
A_44_P529562	Slc30a1	NM_022853	58976	0.04	1.085	0.562489
A_44_P452868	XM_343854	XM_343854		0.04	1.085	0.433173
A_44_P471853	Epc1_predicted	XM_225457		0.04	1.085	0.590951
A_44_P670104	TC527529	TC527529		0.04	1.085	0.519272
A_44_P407788	LOC307084	XR_008457	307084	0.04	1.084	0.657411
A_44_P313445	Clcn4-2	NM_022198	60586	0.04	1.084	0.522854
A_44_P749588	TC529338	TC529338		0.04	1.084	0.673921
A_44_P189139	Tparl	NM_001024802	364137	0.04	1.084	0.545358
A_43_P16761	RGD1306599_predicted	XM_231116	311840	0.04	1.084	0.554827
A_44_P696396	RGD1560037_predicted	XM_001066396		0.04	1.084	0.824586
A_44_P492397	XM_229208	XM_229208		0.03	1.084	0.645452
A_44_P1047723	Urod	XM_342887		0.03	1.084	0.53705
A_43_P12299	Rab5a	NM_022692	64633	0.03	1.084	0.543561
A_42_P755878	Reep4	NM_001025279	306014	0.03	1.084	0.767632
A_44_P368202	RGD1564712_predicted	XM_238621		0.03	1.084	0.750717
A_44_P407660	Ptar1	XM_001079960		0.03	1.083	0.490654
A_44_P194331	RGD1310536_predicted	XM_344855		0.03	1.083	0.486138
A_44_P799251	TC545895	TC545895		0.03	1.083	0.552036
A_44_P325198	AI535313	AI535313	689994	0.03	1.083	0.484923
A_44_P398545	MGC116202	XM_001067251		0.03	1.083	0.632251
A_44_P302611	LOC688442	XM_001066960	688442	0.03	1.083	0.626034
A_44_P387005	RGD1309314_predicted	XM_001078618		0.03	1.083	0.549835
A_44_P900584	TC541788	TC541788		0.03	1.083	0.479119
A_42_P755071	Pla2g4b_predicted	XM_230486		0.03	1.083	0.375496
A_43_P10410	Txndc12	XM_216482	298370	0.03	1.083	0.457728
A_44_P482054	Tufm_predicted	XM_215069		0.03	1.083	0.571862
A_42_P836063	Kbtbd3_predicted	XM_235782		0.03	1.083	0.483496
A_44_P177931	RGD1311634_predicted	XM_215003	293155	0.03	1.083	0.444756

A_42_P522562	Hccs_predicted	XM_228867	317444	0.03	1.083	0.494064
A_44_P337057	Necap2	NM_199096	298598	0.03	1.083	0.656856
A_44_P795512	TC567621	TC567621		0.03	1.083	0.823816
A_44_P562894	Rnd1	NM_001013222	362993	0.03	1.082	0.826479
A_44_P764653	TC538215	TC538215		0.03	1.082	0.770518
A_44_P498719	Olr208_predicted	NM_001000194	293355	0.03	1.082	0.546554
A_44_P376411	BF548067	BF548067		0.03	1.082	0.554417
A_43_P18737	CB544852	CB544852	266611	0.03	1.082	0.438228
A_44_P492843	Vapb	NM_021847	60431	0.03	1.082	0.640571
A_42_P665381	AW917571	AW917571		0.03	1.082	0.714065
A_44_P127597	Dnajb9	NM_012699	24908	0.03	1.082	0.68431
A_44_P333833	BE104111	BE104111	316767	0.03	1.082	0.653936
A_43_P11508	Vamp2	NM_012663	24803	0.03	1.082	0.41942
A_44_P203421	Synj1	XM_573256	85238	0.03	1.082	0.498934
A_44_P403396	Sh3px3_predicted	XM_236271	315696	0.03	1.082	0.519699
A_44_P756474	BM389513	BM389513	309622	0.03	1.082	0.723716
A_44_P192010	Tubgcp5_predicted	XM_218711		0.03	1.082	0.671029
A_44_P409797	Ppp5c	NM_031729	65179	0.03	1.081	0.573528
A_42_P832915	Plod3	NM_178101	288583	0.03	1.081	0.54159
A_42_P814081	Med8_predicted	XM_342894		0.03	1.081	0.567815
A_42_P761516	Myst3	XM_225008	306571	0.03	1.081	0.522282
A_44_P245563	RGD1306249	NM_001008757	303527	0.03	1.081	0.55172
A_44_P483190	RGD1308795_predicted	XM_236392	315804	0.03	1.081	0.599667
A_44_P561179	RGD1565784_predicted	XM_573057		0.03	1.081	0.490121
A_44_P488713	AI230625	AI230625		0.03	1.081	0.765929
A_44_P373071	Zfp36l2	NM_001036626	298765	0.03	1.081	0.633405
A_44_P559148	CB568740	CB568740		0.03	1.081	0.839082
A_43_P16525	Rbm7_predicted	XM_236212		0.03	1.081	0.553536
A_44_P105889	BF401054	BF401054	288449	0.03	1.081	0.542055
A_44_P736967	A_44_P736967	A_44_P736967		0.03	1.081	0.569246
A_42_P661018	Phlpp	NM_021657	59265	0.03	1.080	0.511559
A_44_P415384	LOC291967	NM_001039099	291967	0.03	1.080	0.762939
A_44_P974681	RGD1562992_predicted	XM_575362		0.03	1.080	0.547431
A_44_P437278	LOC246295	XM_001076921		0.03	1.080	0.641913
A_43_P11046	TC519225	TC519225		0.03	1.080	0.636215
A_44_P418349	AI058866	AI058866	29543	0.03	1.080	0.636748
A_44_P1022699	Osgep	XM_214163	290028	0.03	1.080	0.490433
A_43_P15898	Bmp1	XM_573814	83470	0.03	1.080	0.630204
A_42_P471271	Ptpn6	NM_053908	116689	0.03	1.080	0.469826
A_43_P18310	XM_237312	XM_237312		0.03	1.080	0.656382
A_44_P808007	TC523550	TC523550		0.03	1.080	0.738539
A_44_P352420	Mcrs1	XM_217048	300222	0.03	1.080	0.607117
A_44_P553008	A_44_P553008	A_44_P553008		0.03	1.080	0.739874
A_44_P498990	Usp30_predicted	XM_222274		0.03	1.079	0.448363
A_44_P1036875	RGD1309634_predicted	XM_223536	305452	0.03	1.079	0.429701
A_44_P331119	Spsb3_predicted	XM_220230		0.03	1.079	0.443035
A_42_P695531	Tpbg	NM_031807	83684	0.03	1.079	0.546033
A_44_P220045	RGD1310608	NM_001037646	308577	0.03	1.079	0.619257
A_44_P487439	RGD1560501_predicted	XM_344285		0.03	1.079	0.538653
A_44_P959088	RGD1566146_predicted	XM_577126		0.03	1.079	0.770083
A_44_P1029543	XM_216940	XM_216940		0.03	1.079	0.575839
A_44_P593898	LOC680731	XM_001058609		0.03	1.079	0.635933
A_44_P205479	Pdcd7_predicted	XM_343413		0.03	1.079	0.535791
A_42_P717631	AI230097	AI230097		0.03	1.079	0.765366
A_44_P895891	Ncoa5_predicted	XM_001071075		0.03	1.079	0.599245
A_44_P413236	AW920588	AW920588		0.03	1.079	0.692251
A_44_P358807	Surf6_predicted	NM_001015014	303076	0.03	1.079	0.697506
A_42_P509385	Prkx	NM_001033963	501563	0.03	1.079	0.540745
A_44_P361780	CB606190	CB606190	619580	0.03	1.079	0.526985
A_43_P21205	Scrt1_predicted	XM_345848	366951	0.03	1.079	0.68802
A_44_P573915	AW142606	AW142606	691501	0.03	1.079	0.496374
A_44_P210178	Carhsp1	NM_152790	260416	0.03	1.079	0.584995
A_44_P520299	ENSRNOT00000022565	ENSRNOT00000022565		0.03	1.079	0.473146

A_44_P466321	RGD1307468_predicted	XM_223428	289635	0.03	1.078	0.612208
A_44_P339867	Ssr4	NM_017199	29435	0.03	1.078	0.474916
A_42_P769597	Qpctl_predicted	XM_214869		0.03	1.078	0.540353
A_44_P184977	XM_341948	XM_341948		0.03	1.078	0.653288
A_44_P972303	AW916976	AW916976		0.03	1.078	0.599124
A_44_P653448	TC522621	TC522621		0.03	1.078	0.582295
A_44_P838002	TC555480	TC555480		0.03	1.078	0.580281
A_44_P745201	TC553565	TC553565		0.03	1.078	0.735924
A_44_P669345	Hdac8_predicted	XM_343804	363481	0.03	1.078	0.702289
A_44_P175846	LOC307671	XM_226278		0.03	1.078	0.556963
A_44_P1018181	Mmaa_predicted	XM_214659		0.03	1.078	0.722094
A_44_P455073	LOC684822	XM_001072065		0.03	1.077	0.533317
A_43_P12408	Accn2	NM_024154	79123	0.03	1.077	0.639083
A_44_P512136	Dhcr7	NM_022389	64191	0.03	1.077	0.543459
A_44_P975471	BC089955	BC089955	498192	0.03	1.077	0.664994
A_44_P792456	TC524037	TC524037		0.03	1.077	0.503473
A_44_P609428	TC567245	TC567245		0.03	1.077	0.573123
A_44_P760031	RGD1308952	XM_220262	303002	0.03	1.077	0.593633
A_44_P343694	Hp1bp3	NM_199108	313647	0.03	1.077	0.603175
A_44_P226601	F8	NM_183331	302470	0.03	1.077	0.499085
A_44_P919504	RGD1566072_predicted	XM_219424	309081	0.03	1.077	0.441228
A_44_P870317	DV720434	DV720434		0.03	1.077	0.704538
A_44_P1053383	Rab7	NM_023950	29448	0.03	1.077	0.485518
A_44_P297114	RGD1308915	NM_001014226	363545	0.03	1.077	0.618052
A_44_P279024	Agpat7_predicted	XM_215783		0.03	1.077	0.473172
A_44_P822950	AW916957	AW916957		0.03	1.077	0.533189
A_44_P620454	AI072305	AI072305		0.03	1.077	0.646905
A_44_P555808	Psrc2	XM_235146	314836	0.03	1.077	0.673588
A_44_P255373	Mosc2	NM_134410	171451	0.03	1.076	0.491346
A_44_P741619	DV726028	DV726028		0.03	1.076	0.659721
A_44_P978775	TC527025	TC527025		0.03	1.076	0.795617
A_44_P1015233	RGD1563438_predicted	XM_213333		0.03	1.076	0.507194
A_44_P402760	Atp6v0a1	NM_031604	29757	0.03	1.076	0.569033
A_44_P415155	A_44_P415155	A_44_P415155		0.03	1.076	0.644749
A_44_P516115	RGD1566264_predicted	XM_345884		0.03	1.076	0.556628
A_44_P147622	Dnajc13_predicted	XM_343462		0.03	1.076	0.626537
A_44_P562106	TC559156	TC559156		0.03	1.076	0.572679
A_44_P768945	Dtnbp1	NM_001037664	641528	0.03	1.076	0.757833
A_44_P1052772	XM_236381	XM_236381		0.03	1.076	0.635085
A_44_P458878	Tcf19	NM_213561	406195	0.03	1.076	0.586612
A_44_P116999	XM_344770	XM_344770		0.03	1.076	0.589707
A_44_P944361	ENSRNOT00000002063	ENSRNOT00000002063		0.03	1.075	0.651626
A_42_P480108	mrpl24	NM_001007637	295224	0.03	1.075	0.516683
A_44_P433865	Xpc_predicted	XM_232194		0.03	1.075	0.514895
A_44_P184365	AI576132	AI576132		0.03	1.075	0.642153
A_44_P428027	AI101727	AI101727		0.03	1.075	0.610314
A_44_P916811	TC529382	TC529382		0.03	1.075	0.616041
A_44_P252841	RGD1310193_predicted	XM_221911		0.03	1.075	0.608707
A_43_P15362	Slc9a5	NM_138858	192215	0.03	1.075	0.664632
A_44_P387253	BI302132	BI302132		0.03	1.075	0.629999
A_44_P599208	TC546494	TC546494		0.03	1.075	0.750003
A_43_P10423	RGD1306332_predicted	XM_001060870		0.03	1.075	0.510451
A_44_P273990	Ppp2ca	NM_017039	24672	0.03	1.075	0.634709
A_44_P537079	A_44_P537079	A_44_P537079		0.03	1.075	0.591465
A_44_P243770	XM_343361	XM_343361		0.03	1.075	0.645932
A_44_P829841	Pofut2_predicted	XM_228073		0.03	1.075	0.631673
A_44_P121446	Tmed5	NM_001007619	289883	0.03	1.075	0.647927
A_42_P748135	Spen_predicted	XM_001072623		0.03	1.075	0.587415
A_44_P326122	RGD1308019_predicted	XM_231045		0.03	1.074	0.624209
A_44_P782176	AI043960	AI043960		0.03	1.074	0.669704
A_44_P920467	AI177911	AI177911	292964	0.03	1.074	0.511157
A_44_P227517	A_44_P227517	A_44_P227517		0.03	1.074	0.766642
A_44_P996806	Slc30a5_predicted	XM_226722		0.03	1.074	0.66291

A_43_P10032	Ubn1_predicted	XM_220175		0.03	1.074	0.590159
A_44_P286226	Hnrph3_predicted	XM_342131		0.03	1.074	0.517399
A_44_P124176	RGD1306798_predicted	XM_343089	362763	0.03	1.074	0.564944
A_44_P227616	RGD1310868_predicted	XM_221141		0.03	1.074	0.558163
A_44_P175049	Fxna	NM_184050	373544	0.03	1.074	0.758547
A_44_P234362	Tmem43	NM_001007745	362401	0.03	1.074	0.574335
A_42_P840460	Ccdc52	NM_001008285	288111	0.03	1.074	0.662977
A_44_P108032	Arsa	NM_001034933	315222	0.03	1.074	0.62651
A_44_P513029	RT1-CE1	NM_001008832	309603	0.03	1.074	0.740935
A_44_P318707	RGD1562948_predicted	XM_344812		0.03	1.074	0.643085
A_44_P147254	Cilp2_predicted	XM_224741		0.03	1.074	0.638047
A_44_P337253	LOC498750	NM_001017510	498750	0.03	1.074	0.569452
A_44_P341428	Tfip11	NM_001008291	288718	0.03	1.074	0.54933
A_42_P826202	Zfp597	NM_153732	266774	0.03	1.073	0.648293
A_44_P275249	Crk	NM_019302	54245	0.03	1.073	0.622052
A_43_P20602	XM_215991	XM_215991		0.03	1.073	0.51713
A_44_P124250	RGD1308260_predicted	XM_236774	301111	0.03	1.073	0.694072
A_44_P1032487	Tm9sf4	NM_001025649	296279	0.03	1.073	0.591061
A_44_P510470	Pex11a	NM_053487	85249	0.03	1.073	0.612859
A_44_P612842	XM_341458	XM_341458		0.03	1.073	0.783983
A_44_P424658	RGD1359380	NM_001007676	303922	0.03	1.073	0.770188
A_44_P502315	BF522734	BF522734	302898	0.03	1.073	0.609955
A_44_P191862	Hras	XM_001062236		0.03	1.073	0.635785
A_44_P119721	BQ210650	BQ210650		0.03	1.073	0.692031
A_42_P515931	XM_220256	XM_220256		0.03	1.073	0.481979
A_43_P11954	Cav3	NM_019155	29161	0.03	1.073	0.800308
A_44_P361868	Rtf1_predicted	XM_001080858		0.03	1.073	0.677104
A_44_P660676	TC528190	TC528190		0.03	1.073	0.676835
A_44_P717349	TC550822	TC550822		0.03	1.073	0.704956
A_44_P269760	XM_341617	XM_341617		0.03	1.073	0.730868
A_44_P197156	Tbcc_predicted	XM_236922		0.03	1.073	0.464848
A_44_P164170	Gpr2_predicted	XM_343968		0.03	1.073	0.63586
A_44_P524820	Cabp1	NM_133529	171051	0.03	1.073	0.556181
A_44_P309513	LOC501619	NM_001024367	501619	0.03	1.072	0.676672
A_44_P522461	Olr1229_predicted	NM_001000444	300554	0.03	1.072	0.790816
A_44_P134313	Spred1	XM_230454		0.03	1.072	0.627537
A_43_P17161	Mkrn2	NM_001008314	297525	0.03	1.072	0.646164
A_42_P514575	Kif1c	NM_145877	113886	0.03	1.072	0.571999
A_42_P548118	Aco1	NM_017321	50655	0.03	1.072	0.610359
A_43_P19420	XM_341956	XM_341956		0.03	1.072	0.717174
A_44_P684899	BE104772	BE104772		0.03	1.072	0.464794
A_44_P159137	XM_227423	XM_227423		0.03	1.072	0.633963
A_44_P529403	Tbc1d20	NM_001004281	362237	0.03	1.072	0.653844
A_42_P656217	Hfe	NM_053301	29199	0.03	1.072	0.579714
A_42_P706831	RGD1562079_predicted	XM_579917	499125	0.03	1.072	0.580281
A_44_P1029820	Mfn2	NM_130894	64476	0.03	1.072	0.588017
A_43_P21348	LOC691984	XM_001077762		0.03	1.072	0.548257
A_44_P822852	LOC686393	XM_001073919		0.03	1.072	0.654325
A_44_P292495	Asam	NM_173154	286939	0.03	1.072	0.730151
A_44_P241929	BF549324	BF549324	297176	0.03	1.072	0.713749
A_44_P262407	Ggtl3	NM_130423	156275	0.03	1.072	0.667502
A_44_P137717	RGD1308124_predicted	XM_218663		0.03	1.071	0.679413
A_44_P342750	LOC299828	XM_216903	299828	0.03	1.071	0.68065
A_42_P614160	LOC361309	NM_001014148	361309	0.03	1.071	0.545294
A_44_P195306	Ywhab	NM_019377	56011	0.03	1.071	0.635672
A_44_P135964	LOC687039	XM_001076829		0.03	1.071	0.744461
A_44_P143387	U14522	U14522		0.03	1.071	0.878435
A_44_P206973	Fbxw11_predicted	XM_220281		0.03	1.071	0.629078
A_44_P1005858	CO404131	CO404131		0.03	1.071	0.657036
A_44_P387067	LOC682004	XM_001059323		0.03	1.071	0.733494
A_44_P728524	Zfp54_predicted	XM_001054427		0.03	1.071	0.661587
A_44_P810883	CO401828	CO401828		0.03	1.071	0.707095
A_44_P498078	BG667163	BG667163		0.03	1.071	0.585593

A_42_P621796	LOC56825	NM_020091	56825	0.03	1.071	0.704022
A_43_P11707	Vamp1	NM_013090	25624	0.03	1.071	0.558163
A_44_P882856	ENSRNOT00000042478	ENSRNOT00000042478		0.03	1.071	0.674191
A_44_P448286	RGD1304927_predicted	XM_346029	367235	0.03	1.071	0.825825
A_44_P199028	Dkk1_predicted	XM_219804		0.03	1.071	0.767509
A_44_P578364	TC546170	TC546170		0.03	1.071	0.783882
A_44_P858612	A_44_P858612	A_44_P858612		0.03	1.070	0.865167
A_44_P333495	XM_221299	XM_221299		0.03	1.070	0.530921
A_42_P835665	Ostf1	NM_148892	259275	0.03	1.070	0.60979
A_44_P363095	LOC287522	XM_220702		0.03	1.070	0.494505
A_42_P573643	Shbg	NM_012650	24775	0.03	1.070	0.628063
A_44_P227201	Tubgcp3	XM_225013		0.03	1.070	0.705832
A_44_P266591	AI137104	AI137104	81761	0.03	1.070	0.650463
A_44_P522940	XM_341458	XM_341458		0.03	1.070	0.624209
A_43_P15242	Nr3c1	NM_012576	24413	0.03	1.070	0.736602
A_43_P21699	Pi4k2b	NM_001005883	305419	0.03	1.070	0.687794
A_44_P412987	AA956038	AA956038		0.03	1.070	0.702666
A_44_P789637	BE116623	BE116623		0.03	1.070	0.698681
A_42_P614249	Tnks1bp1_predicted	XM_215763	295707	0.03	1.070	0.588762
A_43_P15572	Vegfa	NM_031836	83785	0.03	1.070	0.618944
A_42_P676860	XM_213328	XM_213328		0.03	1.070	0.608018
A_44_P266333	AI231522	AI231522		0.03	1.070	0.587199
A_44_P929478	TC542376	TC542376		0.03	1.070	0.586612
A_44_P1042876	Hspa1a	NM_031971	24472	0.03	1.070	0.709433
A_42_P512838	Trem2_predicted	XM_217335		0.03	1.069	0.754899
A_44_P213508	Ccdc49_predicted	XM_340890		0.03	1.069	0.629786
A_44_P288377	XM_226733	XM_226733		0.03	1.069	0.605072
A_44_P330414	Slc8a1	U04933	29715	0.03	1.069	0.876273
A_43_P18451	Gpr19	NM_080579	312787	0.03	1.069	0.758547
A_44_P1023049	Nfkbil1	NM_212509	361794	0.03	1.069	0.500207
A_44_P238964	Insm1_predicted	XM_345443	366210	0.03	1.069	0.692467
A_44_P1022762	Zfp653_predicted	XM_001074209		0.03	1.069	0.574814
A_43_P13023	Neu3	NM_054010	117185	0.03	1.069	0.713764
A_44_P608972	TC563522	TC563522		0.03	1.069	0.703013
A_44_P409729	Opa1	NM_133585	171116	0.03	1.068	0.722001
A_44_P107571	LOC293494	NM_001013900	293494	0.03	1.068	0.592111
A_43_P16589	CF110262	CF110262		0.03	1.068	0.641843
A_44_P187320	Lias	NM_001012037	305348	0.03	1.068	0.763604
A_44_P111430	AW143657	AW143657	313914	0.03	1.068	0.765287
A_44_P715937	Zfpn1a5_predicted	XM_001053740		0.03	1.068	0.54939
A_44_P441596	Rapgef2_predicted	XM_227301		0.03	1.068	0.713944
A_44_P375042	RGD1309888	NM_001014243	365215	0.03	1.068	0.627896
A_44_P531876	Map3k2	XM_001059840		0.03	1.068	0.669854
A_42_P842580	RGD1566239_predicted	XM_224728		0.03	1.068	0.6408
A_42_P702691	RGD1311375_predicted	XM_228301		0.03	1.068	0.624512
A_44_P315742	Hspa9a_predicted	XM_214583		0.03	1.068	0.664445
A_44_P468504	A_44_P468504	A_44_P468504		0.03	1.068	0.728644
A_42_P707617	Ywhaq	NM_013053	25577	0.03	1.068	0.700947
A_44_P391035	Giot1	NM_133563	171090	0.03	1.068	0.641798
A_44_P356027	Sc5d	NM_053642	114100	0.03	1.068	0.575986
A_44_P550037	BQ781796	BQ781796	294004	0.03	1.068	0.70796
A_42_P573232	BF566546	BF566546	289623	0.03	1.068	0.674169
A_44_P426541	Arhgap24	NM_001012032	305156	0.03	1.068	0.759104
A_44_P928744	ENSRNOT00000046284	ENSRNOT00000046284		0.03	1.068	0.517948
A_43_P12479	Mapk14	U73142	81649	0.03	1.068	0.554935
A_42_P582378	Sc65	NM_021581	59101	0.03	1.067	0.541498
A_43_P20080	RGD1308329_predicted	XM_220308	303039	0.03	1.067	0.541904
A_44_P653304	CV106990	CV106990		0.03	1.067	0.739446
A_44_P586192	AA819112	AA819112		0.03	1.067	0.573646
A_44_P542314	RGD1310899_predicted	CN541737	299198	0.03	1.067	0.701325
A_44_P867478	Tex2	XM_221043	303611	0.03	1.067	0.647754
A_44_P1005933	Lztfl1	NM_001024266	316102	0.03	1.067	0.749193
A_44_P588759	BF563670	BF563670		0.03	1.067	0.630897

A_43_P12285	Mecp2	NM_022673	29386	0.03	1.067	0.489297
A_44_P1019096	RGD1307907_predicted	XM_342515	362212	0.03	1.067	0.70096
A_43_P16664	RGD1311899	NM_001009630	288704	0.03	1.066	0.591001
A_43_P11464	Fgg	NM_012559	24367	0.03	1.066	0.740442
A_43_P19229	Mocs1_predicted	XM_236911		0.03	1.066	0.609614
A_44_P745288	TC553657	TC553657		0.03	1.066	0.815728
A_44_P108125	RGD1562187_predicted	XM_221597		0.03	1.066	0.62519
A_43_P19313	Nqo2	NM_001004214	291084	0.03	1.066	0.65654
A_44_P422103	Rpl5	NM_031099	81763	0.03	1.066	0.576705
A_44_P386375	Mapk1	NM_053842	116590	0.03	1.066	0.717399
A_44_P675125	TC563034	TC563034		0.03	1.066	0.743482
A_44_P211785	BQ198768	BQ198768	114120	0.03	1.066	0.700753
A_42_P815446	Zfyve20_predicted	XM_232195		0.03	1.066	0.635613
A_44_P883270	ENSRNOT00000051568	ENSRNOT00000051568		0.03	1.066	0.723354
A_44_P777257	TC540533	TC540533		0.03	1.066	0.741182
A_44_P327293	AI408948	AI408948	54231	0.03	1.066	0.674921
A_43_P11035	TC557931	TC557931		0.03	1.066	0.58501
A_44_P521168	BG376030	BG376030	310640	0.03	1.066	0.640571
A_43_P22140	RGD1562411_predicted	XM_340875	360595	0.03	1.066	0.749665
A_44_P413324	BF558477	BF558477		0.03	1.066	0.640779
A_44_P201726	RGD1311324	NM_001014195	362711	0.03	1.065	0.707134
A_44_P183294	AW535890	AW535890	501161	0.03	1.065	0.740829
A_44_P426498	RGD1564617_predicted	XM_218267		0.03	1.065	0.591123
A_44_P492013	Map3k4_predicted	XM_217804		0.03	1.065	0.726732
A_44_P194863	Spag7_predicted	XM_220574		0.03	1.065	0.624352
A_44_P171321	RGD1359509	NM_001009671	301416	0.03	1.065	0.57693
A_43_P19762	RGD1564228_predicted	XM_001081442		0.03	1.065	0.517422
A_44_P176771	BF401685	BF401685	89816	0.03	1.065	0.909844
A_44_P363607	Zswim4_predicted	XM_222460		0.03	1.065	0.550161
A_44_P473186	Thrap2_predicted	XM_341090		0.03	1.065	0.652369
A_44_P707159	CV075361	CV075361		0.03	1.065	0.705979
A_44_P716549	TC545284	TC545284		0.03	1.064	0.77329
A_44_P349452	RGD1565509_predicted	XM_344588		0.03	1.064	0.645695
A_44_P557726	Wnt8b_predicted	XM_219959		0.03	1.064	0.600918
A_44_P223817	Atp5a1	NM_023093	65262	0.03	1.064	0.72809
A_44_P439722	Calm2	NM_017326	50663	0.03	1.064	0.700205
A_44_P118777	Olr1433_predicted	NM_001000525	363606	0.03	1.064	0.686967
A_44_P116187	BG669136	BG669136		0.03	1.064	0.779802
A_44_P423509	Usp33	XM_001080019		0.03	1.064	0.745733
A_44_P131046	RGD1560105_predicted	XR_007638	367761	0.03	1.064	0.552794
A_44_P201352	mrpl11	NM_001006973	293666	0.03	1.064	0.671881
A_43_P21142	Tlk1_predicted	XM_242032		0.03	1.064	0.637067
A_44_P293008	Ing1l_predicted	XM_214344		0.03	1.064	0.64406
A_44_P520461	Plcl3_predicted	XM_227208	310463	0.03	1.064	0.862177
A_44_P490100	RGD1565906_predicted	XM_343020	362702	0.03	1.064	0.572679
A_44_P701038	TC527657	TC527657		0.03	1.064	0.567535
A_44_P515138	BM386498	BM386498	65081	0.03	1.064	0.680469
A_44_P269407	Adam32	XM_001060880	361170	0.03	1.064	0.734633
A_43_P16575	RGD1308048_predicted	XM_216543	298557	0.03	1.064	0.658715
A_44_P622283	Spg20	XM_215564		0.03	1.064	0.786347
A_44_P228891	Faah	NM_024132	29347	0.03	1.064	0.720258
A_44_P376592	AI411616	AI411616	306253	0.03	1.063	0.602727
A_44_P1010482	LOC680493	XM_001057427		0.03	1.063	0.679277
A_44_P251756	Stx12	NM_022939	65033	0.03	1.063	0.702739
A_44_P748589	XM_580071	XM_580071		0.03	1.063	0.558832
A_44_P606174	LOC500348	NM_001024338	500348	0.03	1.063	0.650873
A_44_P104704	Taar7b	NM_175586	294126	0.03	1.063	0.76852
A_44_P158973	Rab35	NM_001013046	288700	0.03	1.063	0.673524
A_42_P594475	LOC682926	XM_001060811		0.03	1.063	0.810039
A_44_P518528	Trim11_predicted	XM_340806		0.03	1.063	0.56492
A_44_P391932	L3mbtl2	NM_001033695	300320	0.03	1.063	0.643253
A_44_P113360	AI102738	AI102738	84353	0.03	1.063	0.715228
A_44_P370052	Ldhc	NM_017266	29634	0.03	1.063	0.725638

A_44_P203711	Depdc5_predicted	XM_223584		0.03	1.063	0.689521
A_42_P806077	AW532188	AW532188		0.03	1.063	0.76803
A_44_P410393	Polrmt_predicted	XM_216836		0.03	1.063	0.627875
A_44_P868573	TC520529	TC520529		0.03	1.063	0.643145
A_43_P15613	Nrtn	NM_053399	84423	0.03	1.063	0.658592
A_44_P558660	Ptk9	NM_001008521	315265	0.03	1.062	0.690846
A_42_P782405	AA859831	AA859831		0.03	1.062	0.616955
A_44_P706878	BM986669	BM986669		0.03	1.062	0.68563
A_44_P239105	Zfyve9_predicted	XM_233341		0.03	1.062	0.721977
A_44_P284949	AI230310	AI230310		0.03	1.062	0.61885
A_42_P801580	Mtch2_predicted	XM_215769		0.03	1.062	0.634099
A_44_P297398	Rps6kc1	XM_001053120		0.03	1.062	0.589731
A_44_P319080	Car8	NM_001009662	297814	0.03	1.062	0.79385
A_44_P546383	LOC678774	XM_001053106		0.03	1.062	0.573113
A_44_P890094	CK602919	CK602919		0.03	1.062	0.722739
A_44_P386734	Trrap_predicted	XM_213706		0.03	1.062	0.717773
A_44_P822557	LOC686203	XM_001075630		0.03	1.061	0.710203
A_44_P236647	RGD1311249	NM_001013931	298201	0.03	1.061	0.584735
A_44_P306096	LOC682826	XM_001063287		0.03	1.061	0.730151
A_42_P672907	Tspan8	NM_133526	171048	0.03	1.061	0.718946
A_44_P253112	Pfdn1_predicted	XM_341596		0.03	1.061	0.556364
A_44_P117254	LOC680844	XM_001059150	680844	0.03	1.061	0.829217
A_44_P272376	Mkl1_predicted	XM_235497	315151	0.03	1.061	0.743969
A_44_P225005	Elac1_predicted	XM_226184		0.03	1.061	0.665608
A_44_P1008120	Nans_predicted	XM_216398		0.03	1.061	0.659076
A_43_P11256	Cyc1_predicted	XM_216944	300047	0.03	1.061	0.670106
A_44_P1043468	Aurkc_predicted	XM_214811		0.03	1.061	0.663621
A_43_P12355	Lphn1	NM_022962	65096	0.03	1.061	0.802768
A_44_P213149	Mastl_predicted	XM_225629		0.03	1.061	0.652369
A_44_P205704	Olr1437_predicted	NM_001000015	287325	0.03	1.061	0.777931
A_44_P290341	U2af1l4	NM_001008775	361542	0.03	1.061	0.539708
A_44_P344197	Olr1410_predicted	NM_001000784	405062	0.03	1.061	0.662719
A_44_P358894	Nub1	NM_001013925	296731	0.03	1.060	0.686229
A_44_P643256	A_44_P643256	A_44_P643256		0.03	1.060	0.770188
A_44_P223638	AI009647	AI009647	361269	0.03	1.060	0.737027
A_44_P210563	H1f0	NM_012578	24437	0.03	1.060	0.703073
A_44_P931865	TC521997	TC521997		0.03	1.060	0.612708
A_44_P540065	Rbm10	NM_152861	64510	0.03	1.060	0.657036
A_44_P410600	BF398009	BF398009	302915	0.03	1.060	0.810247
A_44_P193195	Sbno1	XM_001074548		0.03	1.060	0.710264
A_44_P414460	Rnd3	NM_001007641	295588	0.03	1.060	0.797183
A_44_P269158	Arhgap27	NM_198759	303583	0.03	1.060	0.715845
A_44_P808602	TC525318	TC525318		0.03	1.060	0.675185
A_44_P436351	Taf5l	XM_226577		0.03	1.060	0.750037
A_44_P541431	RGD1310794	NM_001024246	296840	0.03	1.060	0.807897
A_44_P442941	Ralbp1	NM_032067	84014	0.03	1.060	0.577398
A_44_P889502	BF284137	BF284137		0.03	1.060	0.802405
A_43_P13793	A_43_P13793	A_43_P13793		0.03	1.059	0.680585
A_44_P929767	TC536649	TC536649		0.03	1.059	0.757115
A_44_P492606	RGD1306073_predicted	XM_343599	363259	0.03	1.059	0.786425
A_44_P401140	Zfp535	NM_001012008	303226	0.03	1.059	0.768397
A_44_P308197	Ubap1	NM_001012190	362502	0.03	1.059	0.583606
A_43_P12782	Ikbkb	NM_053355	84351	0.03	1.059	0.626459
A_44_P424174	Phyhip	NM_001017376	290356	0.02	1.059	0.677471
A_43_P19050	RGD1307597_predicted	XM_234415		0.02	1.059	0.578688
A_43_P14954	Sos1	XM_233820	313845	0.02	1.059	0.705115
A_44_P1003716	RGD1566133_predicted	XM_001054815		0.02	1.059	0.588998
A_44_P393956	XM_227906	XM_227906		0.02	1.059	0.731911
A_42_P733175	Ibrdc1_predicted	XM_238063		0.02	1.059	0.70493
A_44_P990467	BE113961	BE113961		0.02	1.059	0.732667
A_44_P889931	Pign_predicted	XM_001055659		0.02	1.059	0.715639
A_44_P889400	AI137941	AI137941	299618	0.02	1.059	0.742968
A_44_P392701	AI230921	AI230921		0.02	1.059	0.684587

A_44_P332472	Cbwd1	NM_133535	171057	0.02	1.059	0.704022
A_44_P746410	LOC501546	XM_576948		0.02	1.059	0.666837
A_42_P505241	Qprt	NM_001009646	293504	0.02	1.059	0.778981
A_44_P279002	Prpf40a_predicted	XM_215739		0.02	1.059	0.616998
A_44_P513706	LOC299084	XM_001068677		0.02	1.059	0.773449
A_44_P481749	H3f3b	NM_053985	117056	0.02	1.059	0.631167
A_44_P902361	Peo1_predicted	XM_001063420		0.02	1.058	0.594512
A_42_P537971	AI059678	AI059678		0.02	1.058	0.656676
A_44_P469554	Figf	NM_031761	360457	0.02	1.058	0.835541
A_44_P416477	Uqcrc1	NM_001004250	301011	0.02	1.058	0.703599
A_44_P515923	RGD1561596_predicted	XM_228761	317377	0.02	1.058	0.647
A_42_P558503	Brd2	NM_212495	294276	0.02	1.058	0.55625
A_44_P465148	H1f4	NM_133285	201097	0.02	1.058	0.789239
A_44_P224832	RGD1309216	NM_001008371	361726	0.02	1.058	0.623586
A_43_P21226	Usp8_predicted	XM_215821		0.02	1.058	0.63341
A_44_P838983	TC559316	TC559316		0.02	1.058	0.748649
A_43_P18570	Mll_mapped	XM_236194		0.02	1.058	0.570056
A_44_P176053	XM_221094	XM_221094		0.02	1.058	0.739277
A_44_P264567	LOC682467	XM_001061628		0.02	1.058	0.60135
A_43_P17495	Zfp346_predicted	XM_225169		0.02	1.058	0.716144
A_44_P260198	Aff1_predicted	XM_223161		0.02	1.058	0.725827
A_43_P17992	LOC299827	XM_216902		0.02	1.058	0.698782
A_44_P372317	Olr174_predicted	NM_001001028	405914	0.02	1.058	0.754285
A_42_P511401	LOC686293	XM_001073337		0.02	1.057	0.738497
A_44_P146050	XM_215101	XM_215101		0.02	1.057	0.796122
A_44_P774038	AW143275	AW143275		0.02	1.057	0.7007
A_44_P146465	BM986219	BM986219	64205	0.02	1.057	0.794809
A_44_P531656	Olr53_predicted	NM_001001008	405378	0.02	1.057	0.780913
A_44_P575104	RGD1564267_predicted	XM_574547		0.02	1.057	0.629078
A_44_P159171	Mylip_predicted	XM_225220		0.02	1.057	0.708005
A_44_P699780	DN934467	DN934467		0.02	1.057	0.604579
A_44_P316364	RGD1562778_predicted	XM_344967		0.02	1.057	0.831017
A_44_P470444	Syngap1	NM_181092	192117	0.02	1.057	0.717346
A_44_P310596	RGD1311155	NM_001014166	361749	0.02	1.057	0.658101
A_42_P708541	Mrps5_predicted	XM_215833		0.02	1.057	0.767917
A_44_P490265	RGD1564730_predicted	XM_237400		0.02	1.057	0.828048
A_42_P471768	Eif4e2_predicted	XM_343616		0.02	1.057	0.580659
A_44_P946130	TC525048	TC525048		0.02	1.057	0.81531
A_43_P22480	Pnkp	NM_001004259	308576	0.02	1.057	0.591262
A_44_P244104	BI296434	BI296434	362827	0.02	1.057	0.763825
A_44_P1070896	LOC682316	XM_001060977		0.02	1.057	0.592111
A_44_P128964	RGD1560175_predicted	XM_221454	303946	0.02	1.057	0.67274
A_44_P560311	ENSRNOT00000036604	ENSRNOT00000036604		0.02	1.057	0.626009
A_44_P683316	LOC683729	XM_001067233		0.02	1.056	0.589663
A_44_P469129	RGD1562156_predicted	XM_221091		0.02	1.056	0.86778
A_42_P592850	BF420136	BF420136		0.02	1.056	0.724619
A_44_P146640	Adrbk2	NM_012897	25372	0.02	1.056	0.730088
A_44_P191583	Qscn6	NM_053431	84491	0.02	1.056	0.623586
A_42_P598965	Wdr23	NM_001009686	305895	0.02	1.056	0.680469
A_44_P469088	Prkag3_predicted	XM_237293		0.02	1.056	0.708339
A_44_P158599	Ppp1r14c	NM_133425	171010	0.02	1.056	0.825388
A_44_P386840	Dnajb6	NM_001013209	362293	0.02	1.056	0.694933
A_44_P267473	A_44_P267473	A_44_P267473		0.02	1.056	0.611062
A_44_P394239	RGD1561262_predicted	XM_343911	363618	0.02	1.056	0.675894
A_44_P1020105	Aff4_predicted	XM_220420		0.02	1.056	0.688469
A_43_P16895	RGD1304626_predicted	XM_224672	306306	0.02	1.056	0.599221
A_44_P151105	AW144099	AW144099	308667	0.02	1.056	0.747729
A_43_P20914	Pard6b_predicted	XM_342589		0.02	1.056	0.652639
A_44_P473731	Abr_predicted	XM_220717		0.02	1.056	0.752653
A_44_P1036696	Napg	XM_225881		0.02	1.056	0.617485
A_44_P528852	BE111755	BE111755	361402	0.02	1.056	0.727595
A_42_P745802	BI395664	BI395664		0.02	1.056	0.694444
A_44_P1011538	LOC683353	XM_001064234		0.02	1.056	0.776981

A_44_P337786	Pla2g4b_predicted	XM_230486		0.02	1.056	0.589707
A_44_P534154	RGD1307636_predicted	XM_344085		0.02	1.056	0.827319
A_44_P390086	Rab2	NM_031718	65158	0.02	1.055	0.682369
A_44_P367716	Anpep	AF039891	81641	0.02	1.055	0.875704
A_44_P990065	Arhgap5	XM_216688		0.02	1.055	0.738267
A_42_P611304	AI385356	AI385356		0.02	1.055	0.623006
A_44_P424076	LOC691002	XM_001076463	691002	0.02	1.055	0.654332
A_44_P122988	RGD1309550	XM_216781	299295	0.02	1.055	0.6751
A_44_P454192	Sp1	NM_012655	24790	0.02	1.055	0.629674
A_44_P901456	TC562063	TC562063		0.02	1.055	0.609398
A_44_P231597	Hpcal1	NM_017356	50871	0.02	1.055	0.640346
A_44_P593572	TC543532	TC543532		0.02	1.055	0.69461
A_43_P19916	RGD1560908_predicted	XM_223589		0.02	1.055	0.746702
A_44_P740525	RGD1565362_predicted	XM_001055452		0.02	1.055	0.725346
A_43_P23007	LOC315676	XM_001075770	315676	0.02	1.055	0.79888
A_44_P318662	Tac2	NM_019162	29191	0.02	1.055	0.743268
A_44_P366277	AA818893	AA818893		0.02	1.055	0.615727
A_44_P415448	BM383614	BM383614		0.02	1.055	0.70806
A_44_P529321	Csh1	NM_017363	53950	0.02	1.055	0.767917
A_44_P383667	LOC56764	NM_020089	56764	0.02	1.055	0.838985
A_44_P744884	ENSRNOT00000011102	ENSRNOT00000011102		0.02	1.055	0.745664
A_44_P929443	TC526030	TC526030		0.02	1.055	0.780252
A_43_P17682	XM_342699	XM_342699		0.02	1.055	0.629096
A_44_P407636	LOC683865	XM_001067816		0.02	1.055	0.67559
A_43_P10225	Fgfr2	XM_001079450		0.02	1.055	0.828024
A_44_P578267	TC526001	TC526001		0.02	1.055	0.708124
A_44_P278325	RGD1566014_predicted	XM_001053469		0.02	1.055	0.666059
A_43_P21144	RGD1562692_predicted	XM_341290	361015	0.02	1.055	0.640913
A_44_P166482	Mrpl41	NM_001013426	296551	0.02	1.055	0.655904
A_44_P539266	XM_345820	XM_345820		0.02	1.055	0.75164
A_44_P329475	BQ204283	BQ204283	291339	0.02	1.054	0.732715
A_44_P356679	Atpbd1b_predicted	XM_342932	362614	0.02	1.054	0.714147
A_44_P332432	Duox1	NM_153739	266807	0.02	1.054	0.717269
A_42_P577938	RGD1310450	NM_001014218	363254	0.02	1.054	0.662308
A_44_P445615	Pcdhb5_predicted	XM_001055177		0.02	1.054	0.740935
A_44_P169662	Faf1	NM_130406	140657	0.02	1.054	0.75872
A_44_P577978	Slc39a14_predicted	XM_001070144		0.02	1.054	0.810822
A_44_P731895	Grap	NM_001025749	363616	0.02	1.054	0.778022
A_44_P478002	Lsm14a_predicted	XM_341841	361554	0.02	1.054	0.585777
A_44_P476971	AW920949	AW920949	113976	0.02	1.054	0.653658
A_43_P18495	Slc39a8	NM_001011952	295455	0.02	1.054	0.700574
A_42_P795269	LOC682182	XM_001060304		0.02	1.054	0.794858
A_43_P12626	Msln	NM_031658	60333	0.02	1.054	0.881015
A_44_P493812	AI113190	AI113190	307806	0.02	1.054	0.761785
A_44_P637450	LOC679333	XM_001055867		0.02	1.054	0.637375
A_42_P665879	Atf5	NM_172336	282840	0.02	1.054	0.843739
A_44_P391169	Lmx1b	XM_342419	114501	0.02	1.054	0.691673
A_44_P592461	Msh3	XM_001065837		0.02	1.054	0.785075
A_44_P148347	RGD1306343_predicted	XM_219680		0.02	1.054	0.705192
A_44_P430547	Ntrk1	NM_021589	59109	0.02	1.054	0.739187
A_44_P416050	AA925326	AA925326		0.02	1.053	0.809179
A_44_P130154	LOC683512	XM_001066288		0.02	1.053	0.627082
A_44_P489721	RGD1562967_predicted	XM_218795		0.02	1.053	0.764734
A_44_P991121	Aprin_predicted	XM_221833		0.02	1.053	0.726399
A_44_P792628	RGD1311267	NM_001039024	311429	0.02	1.053	0.713944
A_44_P597723	TC523083	TC523083		0.02	1.053	0.758594
A_44_P1016170	Prpf4b	NM_001011923	291078	0.02	1.053	0.746996
A_44_P265965	Smpd2	NM_031360	83537	0.02	1.053	0.650843
A_44_P160876	Rap2a	NM_053741	114560	0.02	1.053	0.627396
A_44_P638062	TC539655	TC539655		0.02	1.053	0.613977
A_44_P607972	TC542025	TC542025		0.02	1.053	0.795936
A_43_P17933	Vps4b	NM_001025716	360834	0.02	1.053	0.759897
A_42_P543774	Trib3	NM_144755	246273	0.02	1.053	0.723354

A_44_P333232	Plekhf2_predicted	XM_342803		0.02	1.053	0.781469
A_44_P363774	LOC500715	XM_580110	500715	0.02	1.052	0.632913
A_44_P190568	Cd83_predicted	XM_341509		0.02	1.052	0.823392
A_44_P340345	Rpl10l_predicted	XM_234245	299106	0.02	1.052	0.679994
A_42_P519024	Mtmr9	NM_001005761	282584	0.02	1.052	0.770828
A_44_P187586	LOC683508	XM_001066253		0.02	1.052	0.658715
A_44_P638014	Irf6_predicted	XM_001067431		0.02	1.052	0.659358
A_43_P10200	Slc30a5_predicted	XM_226722		0.02	1.052	0.725275
A_44_P668927	TC556692	TC556692		0.02	1.052	0.688202
A_44_P1024739	Wipi2	NM_001007615	288498	0.02	1.052	0.643182
A_43_P18411	Brunol4_predicted	XM_226107		0.02	1.052	0.694713
A_44_P457991	AABR03006190	AABR03006190		0.02	1.052	0.926013
A_44_P522209	Man2a1	XM_001068826	25478	0.02	1.052	0.783641
A_44_P520909	Phospho1_predicted	XM_220877		0.02	1.052	0.834166
A_44_P971386	LOC499110	XM_574405	499110	0.02	1.052	0.578295
A_44_P103082	Tspyl2_predicted	XM_217607	302612	0.02	1.052	0.746374
A_44_P281772	Senp7_predicted	XM_221566		0.02	1.052	0.662977
A_44_P267789	Sp6_predicted	XM_343961		0.02	1.052	0.75407
A_44_P411881	XM_575696	XM_575696		0.02	1.052	0.718322
A_44_P451930	NAPE-PLD	NM_199381	296757	0.02	1.051	0.835052
A_44_P131583	BF549497	BF549497		0.02	1.051	0.686225
A_44_P356812	Kctd5_predicted	XM_220224		0.02	1.051	0.667635
A_44_P870203	Baz2b_predicted	XM_229225		0.02	1.051	0.708636
A_44_P351789	Cd83_predicted	XM_341509		0.02	1.051	0.804857
A_44_P704258	RGD1561842_predicted	XM_575315		0.02	1.051	0.909778
A_43_P20634	LOC361335	XM_001057816		0.02	1.051	0.670215
A_42_P737844	RGD1307414_predicted	XM_341259	360983	0.02	1.051	0.790816
A_44_P132160	AA925907	AA925907	117505	0.02	1.051	0.7896
A_44_P935860	Nrip1_predicted	CO401949		0.02	1.051	0.696179
A_44_P363638	LOC362317	XM_342634		0.02	1.051	0.770295
A_42_P688843	Polr2i_predicted	XM_214895		0.02	1.051	0.68544
A_44_P202183	AA866231	AA866231		0.02	1.051	0.68931
A_44_P340603	BF288191	BF288191	548326	0.02	1.050	0.648837
A_44_P1052143	Lzic	NM_001013241	366507	0.02	1.050	0.735801
A_44_P168285	Cyp51	NM_012941	25427	0.02	1.050	0.723889
A_44_P384754	Btf3	NM_001008309	294680	0.02	1.050	0.695894
A_44_P260375	Cobll1_predicted	XM_229988		0.02	1.050	0.770628
A_44_P482399	Vmac	NM_001001720	363327	0.02	1.050	0.716594
A_44_P379360	Fut7	NM_199491	296564	0.02	1.050	0.70442
A_42_P703403	Clta	NM_031974	83800	0.02	1.050	0.705788
A_43_P11800	Bax	NM_017059	24887	0.02	1.050	0.692298
A_44_P558451	Aqp6	NM_022181	29170	0.02	1.050	0.770188
A_44_P328520	RGD1308153_predicted	XM_226235		0.02	1.050	0.747273
A_44_P999532	Rpl36a	NM_031105	81769	0.02	1.050	0.831498
A_44_P836120	BG670441	BG670441		0.02	1.050	0.801734
A_44_P622060	DV718625	DV718625		0.02	1.050	0.716117
A_44_P546506	BF402375	BF402375		0.02	1.050	0.709655
A_44_P473535	A_44_P473535	A_44_P473535		0.02	1.050	0.662308
A_44_P338095	Hibch	NM_001013112	301384	0.02	1.050	0.794282
A_44_P426387	Snap29	NM_053810	116500	0.02	1.050	0.751696
A_44_P528799	AABR03073563	AABR03073563		0.02	1.050	0.663065
A_44_P1047108	Ddt	NM_024131	29318	0.02	1.049	0.70471
A_44_P309629	Ccpg1_predicted	XM_343429		0.02	1.049	0.765419
A_44_P1037217	RGD1565486_predicted	XM_573045	497865	0.02	1.049	0.699452
A_42_P671477	Kcnk3	NM_033376	29553	0.02	1.049	0.723889
A_44_P356691	RGD1566169_predicted	XM_238429	298605	0.02	1.049	0.673439
A_44_P995870	Mrpl34	NM_001006965	290632	0.02	1.049	0.726962
A_43_P22165	RGD1560269_predicted	XM_001065756		0.02	1.049	0.766436
A_44_P212622	Olr583_predicted	NM_001000928	405248	0.02	1.049	0.657201
A_44_P265434	Mif4gd	NM_001014122	360659	0.02	1.049	0.648252
A_43_P13996	TC537589	TC537589		0.02	1.049	0.800221
A_44_P996393	Mgat2	NM_053604	94273	0.02	1.049	0.799681
A_44_P639536	DN932581	DN932581		0.02	1.049	0.778963

A_42_P585467	Rab25_predicted	XM_227404		0.02	1.049	0.738248
A_44_P567217	AW143105	AW143105		0.02	1.049	0.793292
A_44_P700105	TC523502	TC523502		0.02	1.049	0.846481
A_44_P138790	Cirbp	NM_031147	81825	0.02	1.048	0.684183
A_44_P497253	LOC689240	XM_001070091	689240	0.02	1.048	0.822941
A_43_P16950	RGD1560540_predicted	XM_236428		0.02	1.048	0.690516
A_44_P321452	LOC309891	NM_001014033	309891	0.02	1.048	0.72276
A_44_P290407	LOC685243	XM_001061561		0.02	1.048	0.713764
A_44_P632415	LOC679203	XM_001054856		0.02	1.048	0.641253
A_44_P422014	LOC690571	XM_215885		0.02	1.048	0.790933
A_44_P466293	RGD1310712_predicted	XM_001064849		0.02	1.048	0.783088
A_42_P548492	C1galt1c1	NM_001030033	302499	0.02	1.048	0.785666
A_44_P246799	CB547862	CB547862		0.02	1.048	0.843166
A_44_P996997	Fcgrt	NM_033351	29558	0.02	1.048	0.767599
A_44_P356707	Prpf39_predicted	XM_234238		0.02	1.048	0.77988
A_44_P480234	RGD621352	NM_138865	192229	0.02	1.048	0.683862
A_43_P11558	Apod	NM_012777	25239	0.02	1.048	0.831144
A_44_P561352	RGD1310440_predicted	XM_342912	362594	0.02	1.047	0.807201
A_44_P527610	Tradd	XM_341671	246756	0.02	1.047	0.734997
A_44_P368208	Cul4b_predicted	XM_228689		0.02	1.047	0.809639
A_44_P902244	Rnf14	XM_001066493		0.02	1.047	0.728977
A_44_P154742	LOC687705	XM_001079735		0.02	1.047	0.764734
A_42_P661837	Crnkl1	NM_053797	116481	0.02	1.047	0.702419
A_44_P500867	AW917411	AW917411	312439	0.02	1.047	0.702726
A_44_P476936	Agtr1a	NM_030985	24180	0.02	1.047	0.695066
A_44_P459851	Dscr3_predicted	XM_340974		0.02	1.046	0.817779
A_42_P479096	Tfb2m	NM_001008293	289307	0.02	1.046	0.790303
A_44_P358728	RGD1308773_predicted	XM_224175		0.02	1.046	0.832379
A_44_P1004282	RGD1309307_predicted	XM_341321		0.02	1.046	0.73054
A_43_P14470	AA851065	AA851065	301261	0.02	1.046	0.820601
A_44_P483290	RGD1561440_predicted	XM_344122		0.02	1.046	0.661425
A_44_P129009	AA957332	AA957332		0.02	1.046	0.824884
A_44_P713885	A_44_P713885	A_44_P713885		0.02	1.046	0.828751
A_42_P659321	RGD1304620_predicted	XM_001069323		0.02	1.046	0.76809
A_42_P736315	Rtp4_predicted	XM_001056345		0.02	1.046	0.883147
A_43_P20289	Rabl2a	NM_001013221	362987	0.02	1.046	0.720258
A_44_P234395	RGD1565350_predicted	XM_342831		0.02	1.046	0.671173
A_44_P427523	Ppp2r1a	NM_057140	117281	0.02	1.046	0.755483
A_44_P996268	Rasd2	NM_133568	171099	0.02	1.046	0.835723
A_44_P713084	BQ193562	BQ193562		0.02	1.046	0.735941
A_44_P146902	Map4k3	XM_343006	170920	0.02	1.046	0.846829
A_44_P568726	A_44_P568726	A_44_P568726		0.02	1.046	0.798107
A_44_P698503	A_44_P698503	A_44_P698503		0.02	1.046	0.727403
A_44_P1028692	Smtn	XM_214078	289734	0.02	1.045	0.73349
A_44_P386924	ENSRNOT00000022160	ENSRNOT00000022160		0.02	1.045	0.762432
A_44_P287766	AI603162	AI603162		0.02	1.045	0.713846
A_44_P404954	Olr513_predicted	NM_001000313	295766	0.02	1.045	0.910031
A_44_P558254	AA925618	AA925618		0.02	1.045	0.816181
A_44_P513546	Wwox_predicted	XM_214696		0.02	1.045	0.830337
A_44_P361390	RGD1308350	XM_001072750		0.02	1.045	0.775941
A_44_P444297	AA963764	AA963764	366848	0.02	1.045	0.705899
A_44_P519312	LOC679060	XM_001054517		0.02	1.045	0.69242
A_44_P412577	Gpr21_predicted	XM_231251		0.02	1.045	0.674861
A_43_P17988	Tmem77	NM_001025018	362011	0.02	1.045	0.779486
A_44_P226992	XM_341753	XM_341753		0.02	1.045	0.7884
A_44_P506186	RGD1565738_predicted	XM_001077973		0.02	1.045	0.794292
A_44_P180501	LOC682690	XM_001062637		0.02	1.045	0.815995
A_44_P668402	TC520560	TC520560		0.02	1.044	0.814438
A_44_P777454	TC541596	TC541596		0.02	1.044	0.862177
A_44_P144817	LOC364597	XR_008014	364597	0.02	1.044	0.71513
A_44_P292836	Ankrd13	NM_001012148	360823	0.02	1.044	0.853703
A_44_P798959	RGD1307961_predicted	XM_219988	309467	0.02	1.044	0.720287
A_44_P685508	TC526257	TC526257		0.02	1.044	0.784722

A_44_P1012668	AA900363	AA900363		0.02	1.044	0.745969
A_43_P10271	TC520358	TC520358		0.02	1.044	0.68978
A_44_P368143	XM_230074	XM_230074		0.02	1.044	0.831691
A_44_P241532	A_44_P241532	A_44_P241532		0.02	1.044	0.747969
A_43_P21957	Cd7_predicted	XM_221216		0.02	1.044	0.826822
A_43_P22542	Nsdhl	NM_001009399	309262	0.02	1.044	0.688014
A_44_P495894	AW915384	AW915384	681389	0.02	1.044	0.730489
A_44_P1050144	RGD1307071_predicted	XM_230657	311502	0.02	1.044	0.779132
A_44_P564102	TC518405	TC518405		0.02	1.044	0.727903
A_43_P10986	Msl31	NM_001014111	317464	0.02	1.044	0.759408
A_44_P101612	AA875411	AA875411	65041	0.02	1.044	0.805286
A_44_P352973	AW916530	AW916530	305031	0.02	1.044	0.844627
A_44_P913615	A_44_P913615	A_44_P913615		0.02	1.044	0.673206
A_44_P623197	RGD1310139_predicted	XM_001063274		0.02	1.044	0.870783
A_44_P191022	Phyh	NM_053674	114209	0.02	1.044	0.818014
A_44_P436455	XM_213110	XM_213110		0.02	1.044	0.82438
A_44_P211440	Araf	NM_022532	64363	0.02	1.044	0.77079
A_44_P367110	Freq	NM_024366	65153	0.02	1.044	0.842901
A_44_P305852	Mbd6_predicted	XM_343219	362892	0.02	1.043	0.743224
A_44_P839432	TC561019	TC561019		0.02	1.043	0.832711
A_44_P607722	LOC498176	NM_001017487	498176	0.02	1.043	0.782316
A_44_P466034	XM_213268	XM_213268		0.02	1.043	0.696815
A_44_P107372	RT1-CE2	NM_001008840	414779	0.02	1.043	0.828871
A_44_P194768	Coq10a_predicted	XM_343138		0.02	1.043	0.70493
A_44_P102369	Socs1	NM_145879	252971	0.02	1.043	0.77061
A_43_P13468	Hspa4l_predicted	XM_001063049		0.02	1.043	0.74598
A_44_P368220	XM_232281	XM_232281		0.02	1.043	0.717236
A_44_P354338	Fbxl12	NM_001025700	313782	0.02	1.043	0.714469
A_44_P112043	RGD1309562_predicted	XM_213811		0.02	1.043	0.728058
A_44_P789473	LOC365157	XM_001053999		0.02	1.043	0.763642
A_44_P548581	Fus	NM_001012137	317385	0.02	1.043	0.716421
A_44_P128564	RGD1564499_predicted	XM_574083		0.02	1.043	0.76864
A_42_P797466	RGD1560470_predicted	XM_342384	362083	0.02	1.042	0.707593
A_44_P182996	RGD1564480_predicted	XM_577020	501620	0.02	1.042	0.873497
A_44_P550364	Dynll2	NM_080697	140734	0.02	1.042	0.731143
A_44_P386901	XM_224454	XM_224454		0.02	1.042	0.681935
A_44_P457190	LOC691770	XM_001079587	691770	0.02	1.042	0.747167
A_44_P241510	XM_243038	XM_243038		0.02	1.042	0.835825
A_44_P532237	Fanca_predicted	XM_341713		0.02	1.042	0.743224
A_42_P676304	Ifitm1_predicted	XM_215117		0.02	1.042	0.890085
A_44_P323882	Btbd9	NM_001013073	294318	0.02	1.042	0.84984
A_44_P421157	CK357683	CK357683		0.02	1.042	0.794613
A_42_P602482	LOC362317	XM_342634		0.02	1.042	0.832515
A_44_P466355	Lzts2	NM_001014247	365468	0.02	1.042	0.699525
A_44_P623125	TC524191	TC524191		0.02	1.042	0.792239
A_44_P144202	Serpinf1	NM_177927	287526	0.02	1.042	0.822968
A_44_P548748	Mbd6_predicted	XM_343219	362892	0.02	1.042	0.709848
A_44_P327945	G4	NM_001003975	406868	0.02	1.042	0.678864
A_43_P17406	Vps16	NM_001005541	296159	0.02	1.042	0.685848
A_44_P608318	CA511179	CA511179	498533	0.02	1.042	0.810048
A_44_P149358	U78124	U78124		0.02	1.042	0.839958
A_44_P240156	BI281697	BI281697		0.02	1.042	0.719398
A_44_P203949	XM_228736	XM_228736		0.02	1.042	0.779297
A_44_P540304	AW140804	AW140804		0.02	1.041	0.783397
A_44_P573958	CF110118	CF110118	314046	0.02	1.041	0.794809
A_44_P353609	LOC501233	XM_576654		0.02	1.041	0.790906
A_44_P685517	TC528464	TC528464		0.02	1.041	0.838585
A_44_P174933	BE108949	BE108949	314224	0.02	1.041	0.75007
A_44_P258773	Fadd	NM_152937	266610	0.02	1.041	0.766443
A_44_P312745	AW918759	AW918759	313825	0.02	1.041	0.740912
A_44_P312333	Zfml_predicted	XM_242556		0.02	1.041	0.692452
A_44_P285220	BE107760	BE107760	83792	0.02	1.041	0.829896
A_44_P1005450	Ktn1_predicted	XM_341305	361029	0.02	1.041	0.746374

A_44_P499327	RGD1304876_predicted	XM_342693		0.02	1.041	0.73054
A_44_P541020	B3galt4	NM_133553	171079	0.02	1.041	0.702726
A_43_P11369	RGD1304792_predicted	XM_232102	312474	0.02	1.041	0.824347
A_44_P187408	XM_341465	XM_341465		0.02	1.041	0.785781
A_44_P899938	LOC683022	XM_001064150		0.02	1.041	0.840961
A_43_P21191	Btbd11_predicted	XM_234988		0.02	1.041	0.913253
A_44_P714184	A_44_P714184	A_44_P714184		0.02	1.041	0.823993
A_44_P731083	TC543044	TC543044		0.02	1.041	0.783983
A_43_P20879	Ddef2_predicted	XM_343039		0.02	1.040	0.824586
A_44_P344032	AI556191	AI556191	315133	0.02	1.040	0.733188
A_44_P171961	AI599689	AI599689	297388	0.02	1.040	0.837103
A_44_P215467	Taf13_predicted	XM_227586		0.02	1.040	0.81197
A_44_P638987	DV717005	DV717005		0.02	1.040	0.856702
A_44_P557585	A_44_P557585	A_44_P557585		0.02	1.040	0.746374
A_44_P274605	RGD1562165_predicted	XM_345534		0.02	1.040	0.805082
A_44_P119696	AA851514	AA851514	287709	0.02	1.040	0.835852
A_44_P529473	Bm259	XM_341392	252959	0.02	1.040	0.836961
A_44_P101460	CB544681	CB544681	360942	0.02	1.040	0.78871
A_44_P261266	Usp11	NM_001008861	408217	0.02	1.040	0.736871
A_44_P874471	CF114181	CF114181		0.02	1.040	0.821846
A_44_P515326	Dgat1	NM_053437	84497	0.02	1.040	0.783088
A_43_P13103	Dhfr	NM_130400	24312	0.02	1.040	0.809045
A_44_P503916	LOC680558	XM_001057715	680558	0.02	1.040	0.739815
A_44_P1012591	Osbpl1a	NM_172023	259221	0.02	1.040	0.829894
A_42_P638166	RGD1560368_predicted	XM_227306	310544	0.02	1.040	0.903197
A_42_P712949	Fbxo23	BC097431	306771	0.02	1.040	0.780879
A_43_P21910	Pkn3	XM_216019		0.02	1.039	0.802841
A_44_P372148	BU759022	BU759022	297073	0.02	1.039	0.754168
A_44_P545924	RGD1306730	NM_001039608	309009	0.02	1.039	0.819383
A_44_P147549	Hkr3	XM_001075497	362668	0.02	1.039	0.719431
A_42_P717912	40063	NM_022616	64551	0.02	1.039	0.733529
A_44_P372752	ENSRNOT00000020175	ENSRNOT00000020175		0.02	1.039	0.835371
A_42_P543877	Tlcd1	NM_001013858	287472	0.02	1.039	0.879752
A_44_P456483	Eif2b5	NM_138866	192234	0.02	1.039	0.802771
A_44_P220827	Zfp367	NM_001012051	306695	0.02	1.039	0.828755
A_43_P22679	RGD1566009_predicted	XM_220211	302953	0.02	1.039	0.7287
A_44_P913407	A_44_P913407	A_44_P913407		0.02	1.039	0.819342
A_43_P22746	Cdc73	NM_001024769	304832	0.02	1.039	0.730151
A_43_P13188	Yt521	NM_133423	170956	0.02	1.039	0.820113
A_44_P293453	BF548047	BF548047	691318	0.02	1.039	0.880441
A_44_P662325	LOC500990	NM_001037364	500990	0.02	1.039	0.829051
A_42_P797381	Tap2	NM_032056	24812	0.02	1.039	0.700734
A_44_P885003	TC524256	TC524256		0.02	1.039	0.83769
A_44_P1049638	XM_214457	XM_214457		0.02	1.039	0.793531
A_44_P193736	AI011505	AI011505		0.02	1.039	0.839958
A_44_P279208	Eif4g3_predicted	XM_216563		0.02	1.038	0.791627
A_43_P16166	Cd69	NM_134327	29187	0.02	1.038	0.780012
A_44_P177938	Mical2_predicted	XM_344951	365352	0.02	1.038	0.774024
A_44_P1024703	Cyb561d2	NM_001007753	363137	0.02	1.038	0.756491
A_44_P382765	Apba3	NM_031781	83611	0.02	1.038	0.702601
A_44_P461244	Hyal3	NM_207599	300993	0.02	1.038	0.795369
A_44_P164947	AI177413	AI177413	641434	0.02	1.038	0.750691
A_44_P411362	Rbaf600	NM_001039026	313658	0.02	1.038	0.706415
A_44_P915547	TC558708	TC558708		0.02	1.038	0.85902
A_44_P182698	Mobkl1a_predicted	XM_341194		0.02	1.038	0.872301
A_44_P870486	A_44_P870486	A_44_P870486		0.02	1.038	0.717834
A_43_P18879	Arsa	NM_001034933	315222	0.02	1.038	0.794056
A_44_P145422	BF559782	BF559782		0.02	1.038	0.783504
A_44_P156952	Pou5f1	NM_001009178	294562	0.02	1.038	0.739133
A_44_P325960	LOC685009	XM_001061888	685009	0.02	1.038	0.810861
A_44_P615732	RGD1565267_predicted	XM_575918		0.02	1.037	0.702115
A_44_P409619	Olr1658_predicted	NM_001000105	291161	0.02	1.037	0.924872
A_44_P335752	RGD1306000_predicted	XM_343905		0.02	1.037	0.745969

A_44_P510940	Mki67_predicted	XM_225460	291234	0.02	1.037	0.834166
A_44_P913816	LOC680112	XM_001055766		0.02	1.037	0.838985
A_44_P1006348	Ttc4	NM_001013214	362556	0.02	1.037	0.823931
A_44_P337134	Taar8b	NM_175601	319106	0.02	1.037	0.782037
A_44_P156043	RGD1306324	NM_001039012	298675	0.02	1.037	0.80659
A_44_P249690	Itgb3	NM_153720	29302	0.02	1.037	0.853326
A_44_P490235	A_44_P490235	A_44_P490235		0.02	1.037	0.758143
A_43_P16942	CB546044	CB546044	361454	0.02	1.037	0.82108
A_44_P330765	Ccrk	NM_001025752	364666	0.02	1.037	0.806346
A_44_P532476	LOC685714	XM_001068839		0.02	1.037	0.804249
A_44_P402664	Fbxo6b	NM_138917	192351	0.02	1.037	0.734537
A_44_P187685	Ormdl2_predicted	XM_213832		0.02	1.037	0.756731
A_44_P245761	LOC308846	XM_218951		0.02	1.037	0.795368
A_44_P422305	RGD1559859_predicted	XM_347117	363244	0.02	1.036	0.825388
A_43_P17964	Arcn1	NM_001007662	300674	0.02	1.036	0.806406
A_44_P140003	Nrf1_predicted	XM_231566	312195	0.02	1.036	0.794715
A_42_P491707	BF546374	BF546374		0.02	1.036	0.744397
A_44_P417429	Coq6	NM_001011983	299195	0.02	1.036	0.752713
A_44_P159455	Ibtk_predicted	XM_001062352		0.02	1.036	0.776303
A_44_P494913	XM_220594	XM_220594		0.02	1.036	0.918767
A_44_P554917	RT1-Cl	NM_206848	24737	0.02	1.036	0.823343
A_44_P916583	CB614494	CB614494		0.02	1.036	0.915682
A_44_P473754	Son	XM_221656	304092	0.02	1.036	0.828858
A_44_P285390	Atp6v1d	NM_199386	299159	0.02	1.036	0.792066
A_44_P426033	RGD1307381	NM_001014116	360498	0.02	1.036	0.777329
A_44_P159191	Pura_predicted	XM_226016	307498	0.02	1.036	0.726245
A_44_P492409	Jmjd2c_predicted	XM_216426		0.02	1.036	0.881015
A_43_P11661	Rab3a	NM_013018	25531	0.02	1.035	0.850728
A_44_P1019036	RGD1565004_predicted	XM_576463		0.02	1.035	0.797456
A_44_P309052	RT1-M6-2	NM_001008853	365527	0.02	1.035	0.874157
A_44_P406775	LOC363146	NM_001014214	363146	0.02	1.035	0.865569
A_44_P267117	Gale	NM_080783	114860	0.02	1.035	0.767997
A_44_P915481	TC541274	TC541274		0.02	1.035	0.928187
A_44_P388174	AABR03055433	AABR03055433		0.01	1.035	0.8562
A_44_P414984	LOC502627	XM_001054404		0.01	1.035	0.82766
A_44_P541337	RGD1562407_predicted	XM_001064372		0.01	1.035	0.806487
A_44_P255501	Ube2q_predicted	XM_215612		0.01	1.035	0.802172
A_44_P437966	Apcs	NM_017170	29339	0.01	1.035	0.789587
A_44_P917243	TC533180	TC533180		0.01	1.035	0.839295
A_44_P105680	Zfp496_predicted	XM_220512	287361	0.01	1.035	0.803403
A_44_P551540	H32804	H32804		0.01	1.035	0.751585
A_43_P16642	Cmas	NM_001009419	312826	0.01	1.035	0.823816
A_44_P974063	BF284864	BF284864	312381	0.01	1.035	0.843291
A_44_P454065	Jag1	NM_019147	29146	0.01	1.035	0.828462
A_44_P427028	RGD1306844	XM_343592	363252	0.01	1.035	0.76333
A_44_P610365	TC528192	TC528192		0.01	1.034	0.784273
A_42_P519311	Cklf	NM_139111	245978	0.01	1.034	0.788514
A_44_P1002601	BF549601	BF549601		0.01	1.034	0.796757
A_44_P760798	TC552020	TC552020		0.01	1.034	0.866118
A_44_P744814	Hnrpa3	NM_198132	362152	0.01	1.034	0.822626
A_44_P1017345	Bag1_predicted	XM_216377		0.01	1.034	0.796497
A_44_P182402	Cnksr3	NM_001012061	308113	0.01	1.034	0.900158
A_44_P227421	ENSRNOT00000041594	ENSRNOT00000041594		0.01	1.034	0.784609
A_44_P806840	Apol3	NM_001013175	315108	0.01	1.034	0.824683
A_44_P349236	Gltscr1_predicted	XM_214817		0.01	1.034	0.773204
A_44_P852833	LOC294154	NM_001039607	294154	0.01	1.034	0.810907
A_44_P262078	Gfi1	NM_012566	24388	0.01	1.034	0.943912
A_42_P828898	RGD1305793	XM_219958	309456	0.01	1.034	0.816076
A_44_P372237	Mterfd1	NM_199387	299514	0.01	1.034	0.814605
A_44_P636769	ENSRNOT00000043099	ENSRNOT00000043099		0.01	1.034	0.76983
A_44_P336489	Txndc13	XM_001081259		0.01	1.034	0.826752
A_43_P17202	Mdm4	NM_001012026	304798	0.01	1.034	0.835825
A_44_P166186	Tor1b	NM_001039197	311854	0.01	1.033	0.76974

A_44_P479283	RGD1305534_predicted	XM_001062758		0.01	1.033	0.868363
A_44_P345175	AA925317	AA925317		0.01	1.033	0.802564
A_44_P391939	eplin	XM_217039	300228	0.01	1.033	0.822842
A_44_P532062	XM_341339	XM_341339		0.01	1.033	0.809351
A_44_P529905	RGD1309102_predicted	XM_214614		0.01	1.033	0.834083
A_44_P261179	LOC362845	XM_343177	362845	0.01	1.033	0.803795
A_44_P375623	LOC686820	XM_001075859		0.01	1.033	0.890085
A_44_P196591	Sh3bp4	NM_022693	64634	0.01	1.033	0.775036
A_42_P823894	Map3k1	NM_053887	116667	0.01	1.033	0.776224
A_44_P475406	Hsf1	XM_001061027	79245	0.01	1.033	0.773449
A_44_P917870	TC542653	TC542653		0.01	1.033	0.908823
A_44_P888000	TC548611	TC548611		0.01	1.033	0.909778
A_44_P255986	CB544856	CB544856	287106	0.01	1.033	0.806251
A_43_P11181	LOC686668	XM_001074291		0.01	1.033	0.8211
A_44_P200233	Nrd1	NM_012993	25499	0.01	1.033	0.816181
A_44_P1003496	Stk16	NM_173142	286927	0.01	1.033	0.806251
A_44_P1027244	RGD1305007	NM_001013976	303749	0.01	1.033	0.778705
A_44_P383199	RGD1308706_predicted	XM_214649	291925	0.01	1.033	0.832651
A_44_P1045748	Lpin2_predicted	XM_237521		0.01	1.033	0.797537
A_42_P605386	MGC94113	NM_001007655	298689	0.01	1.033	0.799436
A_43_P17854	Ift80	NM_001013911	295106	0.01	1.033	0.800061
A_43_P16682	Eif2s3x	XM_216704	299027	0.01	1.033	0.790665
A_44_P451120	Lmbrd1	NM_139189	246046	0.01	1.033	0.922235
A_43_P21444	Arfgef2	NM_181083	296380	0.01	1.032	0.839658
A_44_P319208	Dpp8_predicted	XM_236345		0.01	1.032	0.818411
A_43_P21027	RGD1560606_predicted	XM_235023	314713	0.01	1.032	0.778981
A_44_P555567	RGD1310645_predicted	XM_225679	307213	0.01	1.032	0.852444
A_44_P899694	AI060265	AI060265		0.01	1.032	0.787013
A_43_P11371	RGD1563595_predicted	XM_575086	499751	0.01	1.032	0.842243
A_44_P590658	ENSRNOT00000000055	ENSRNOT00000000055		0.01	1.032	0.851489
A_44_P494652	Chchd4	NM_001013431	312559	0.01	1.032	0.807205
A_44_P524576	Nupl1	AF000899	245922	0.01	1.032	0.764849
A_43_P14687	Fez2	BC100060	94269	0.01	1.032	0.810499
A_42_P729340	Oas1	NM_138913	192281	0.01	1.032	0.933126
A_42_P615851	RGD1565642_predicted	XM_341932	361652	0.01	1.032	0.788061
A_44_P414850	Mrps31_predicted	XM_214383		0.01	1.032	0.788497
A_44_P837015	ENSRNOT00000031061	ENSRNOT00000031061		0.01	1.032	0.874724
A_44_P187083	Atp2a2	NM_017290	29693	0.01	1.032	0.825596
A_44_P401939	LOC680726	XM_001061146	680726	0.01	1.032	0.801526
A_42_P793693	RGD1559904_predicted	XM_232783		0.01	1.032	0.779457
A_44_P168569	LOC502603	NM_001035255	502603	0.01	1.032	0.820113
A_44_P291755	AI230320	AI230320		0.01	1.032	0.840965
A_44_P1049289	Ptplb_predicted	XM_213610	288058	0.01	1.032	0.802504
A_44_P290798	RGD1561537_predicted	XM_341480	361197	0.01	1.032	0.78411
A_44_P691559	AW144675	AW144675		0.01	1.032	0.877372
A_42_P631493	Csda	NM_031979	83807	0.01	1.031	0.790751
A_44_P159478	Pdcd6ip	NM_001029910	501083	0.01	1.031	0.81852
A_43_P20832	Ankrd32_predicted	XM_226614		0.01	1.031	0.79374
A_44_P1028012	BX883045	BX883045		0.01	1.031	0.830337
A_44_P136285	Jam3	NM_001004269	315509	0.01	1.031	0.891102
A_43_P18702	RGD1562582_predicted	XM_001068226		0.01	1.031	0.798273
A_44_P423775	Olr1326_predicted	NM_001000474	300626	0.01	1.031	0.908034
A_43_P13040	Rhoa	NM_057132	117273	0.01	1.031	0.856667
A_44_P145269	Nkiras2_predicted	XM_213462		0.01	1.031	0.806346
A_44_P429092	Sap130_predicted	XM_226078	307527	0.01	1.031	0.815803
A_44_P1048663	Ccnl1	NM_053662	114121	0.01	1.031	0.835541
A_44_P104679	Pik3cb	NM_053481	85243	0.01	1.031	0.810038
A_43_P10681	TC554503	TC554503		0.01	1.030	0.832578
A_44_P238661	RGD1564490_predicted	XM_577254		0.01	1.030	0.836375
A_44_P477446	LOC498351	XM_001066807		0.01	1.030	0.7826
A_44_P167189	AA943125	AA943125		0.01	1.030	0.840354
A_44_P538749	XM_222208	XM_222208		0.01	1.030	0.758051
A_43_P22415	RGD1565857_predicted	XM_219735		0.01	1.030	0.836891

A_44_P192247	LOC687752	XR_009338	691279	0.01	1.030	0.836091
A_44_P263130	XM_217395	XM_217395		0.01	1.030	0.915473
A_44_P172712	Olr304_predicted	NM_001001039	405925	0.01	1.030	0.912337
A_44_P455228	Mgat5b_predicted	XM_221136		0.01	1.030	0.900078
A_44_P352220	AA818966	AA818966		0.01	1.030	0.841007
A_44_P339862	Mrpl3_predicted	XM_217239		0.01	1.030	0.84984
A_42_P704370	Arl6ip5	NM_023972	66028	0.01	1.030	0.793292
A_44_P749102	TC544844	TC544844		0.01	1.030	0.838538
A_44_P866091	Slc12a7	XM_001060536		0.01	1.030	0.843116
A_44_P205928	Zfp95_predicted	XM_221910		0.01	1.030	0.821911
A_44_P405397	Btd	NM_001012047	306262	0.01	1.030	0.893726
A_44_P410062	XM_224553	XM_224553		0.01	1.030	0.865745
A_44_P378331	RGD1305031	NM_001025640	292640	0.01	1.030	0.802764
A_44_P206180	A_44_P206180	A_44_P206180		0.01	1.030	0.810953
A_44_P510206	BE119829	BE119829	140586	0.01	1.030	0.804037
A_44_P178105	LOC686132	XM_001067579		0.01	1.030	0.853703
A_44_P253324	Smc6l1_predicted	XM_233970		0.01	1.029	0.846461
A_44_P370678	LOC293917	XR_006663	293917	0.01	1.029	0.830634
A_44_P1035051	Ppp1r15b_predicted	XM_222633		0.01	1.029	0.827201
A_44_P455206	RGD1561118_predicted	XM_220759		0.01	1.029	0.817674
A_44_P159561	XM_221736	XM_221736		0.01	1.029	0.846453
A_44_P731778	TC527926	TC527926		0.01	1.029	0.842034
A_44_P290867	RGD1565291_predicted	XM_225738		0.01	1.029	0.826873
A_44_P330435	AF216218	AF216218		0.01	1.029	0.908561
A_43_P18526	XM_229173	XM_229173		0.01	1.029	0.86301
A_44_P124074	XM_217067	XM_217067		0.01	1.029	0.856234
A_43_P12167	Gpd1	NM_022215	60666	0.01	1.029	0.914611
A_44_P926681	AA800797	AA800797		0.01	1.029	0.838538
A_43_P15100	BF553214	BF553214		0.01	1.029	0.885902
A_43_P16163	Gbx2	XM_346072	114500	0.01	1.029	0.807897
A_44_P271423	Zc3hav1	NM_173045	252832	0.01	1.029	0.883429
A_44_P411808	Dyrk2_predicted	XM_235179		0.01	1.029	0.865745
A_43_P11651	Pemt	NM_013003	25511	0.01	1.029	0.868745
A_44_P332618	Actn4	NM_031675	63836	0.01	1.029	0.841113
A_44_P412170	P4ha1	NM_172062	64475	0.01	1.029	0.931733
A_44_P233749	LOC690435	XM_001074452	683871	0.01	1.029	0.870033
A_44_P508809	Dpm1_predicted	XM_215949		0.01	1.029	0.839082
A_44_P267068	Nudt1	NM_057120	117260	0.01	1.029	0.790141
A_44_P337094	Prkag1	NM_013010	25520	0.01	1.029	0.881349
A_44_P304238	LOC316717	M58436	316717	0.01	1.029	0.915864
A_44_P1046651	Chfr	NM_001009258	288734	0.01	1.029	0.860171
A_44_P328499	RGD1562100_predicted	XR_007403	302297	0.01	1.028	0.877553
A_44_P229079	Rb1	XM_344434	24708	0.01	1.028	0.849756
A_44_P555534	RGD1560691_predicted	XM_001065973		0.01	1.028	0.848923
A_44_P839458	TC526689	TC526689		0.01	1.028	0.851489
A_44_P519817	AI232703	AI232703	85239	0.01	1.028	0.856296
A_44_P853289	RGD1359158	NM_001007737	361740	0.01	1.028	0.832651
A_44_P342721	A_44_P342721	A_44_P342721		0.01	1.028	0.833346
A_44_P946394	TC525395	TC525395		0.01	1.028	0.873981
A_44_P128472	RGD1566115_predicted	NM_001024239	290348	0.01	1.028	0.824492
A_44_P384468	LOC686892	XM_001074939		0.01	1.028	0.832651
A_44_P410050	A_44_P410050	A_44_P410050		0.01	1.028	0.818621
A_44_P699110	TC539917	TC539917		0.01	1.027	0.867639
A_44_P314110	RGD1308367	NM_001008311	295619	0.01	1.027	0.900736
A_44_P429083	Rnd3	NM_001007641	295588	0.01	1.027	0.870452
A_44_P538526	Numb	DQ336705	29419	0.01	1.027	0.819385
A_44_P151433	Pde8b	NM_199268	309962	0.01	1.027	0.964806
A_44_P259561	BM385639	BM385639		0.01	1.027	0.880106
A_43_P20387	Arfgef1_predicted	XM_232614	312915	0.01	1.027	0.882775
A_44_P527770	A_44_P527770	A_44_P527770		0.01	1.027	0.886402
A_42_P531078	Gsta4	XM_217195		0.01	1.027	0.926551
A_42_P611561	RGD1308901_predicted	XM_214898		0.01	1.027	0.812461
A_44_P490615	BE106565	BE106565	304092	0.01	1.027	0.848592

A_44_P263037	LOC362972	XM_343302	362972	0.01	1.027	0.881349
A_43_P20658	Angpt4_predicted	XM_215890		0.01	1.027	0.936217
A_44_P255934	Usp32_predicted	XM_220798		0.01	1.027	0.873815
A_44_P229537	RGD1561337_predicted	XM_574397	499105	0.01	1.027	0.797682
A_44_P386866	LOC688785	XM_001070868	688785	0.01	1.027	0.817562
A_44_P216831	AA848859	AA848859	316557	0.01	1.027	0.870356
A_44_P852790	TC542800	TC542800		0.01	1.027	0.928644
A_44_P371125	Sult1b1	NM_022513	64305	0.01	1.027	0.887194
A_44_P366222	XM_220863	XM_220863		0.01	1.026	0.871468
A_44_P490147	LOC691093	XM_001076820		0.01	1.026	0.796341
A_44_P1003343	LOC362994	NM_001014203	362994	0.01	1.026	0.8845
A_44_P1045997	Wdr22	XM_234345	314273	0.01	1.026	0.854354
A_43_P13702	Clp1	NM_001025136	498008	0.01	1.026	0.834166
A_42_P680097	Churc1_predicted	XM_216740		0.01	1.026	0.906673
A_43_P10359	Ublcp1	NM_001014117	360514	0.01	1.026	0.862172
A_42_P628764	RGD1306936_predicted	XM_216139		0.01	1.026	0.838229
A_44_P838603	DV714996	DV714996	287877	0.01	1.026	0.908845
A_44_P306840	Ddhd1	NM_001033066	305816	0.01	1.026	0.838538
A_44_P503350	Olr1142_predicted	NM_001000877	405175	0.01	1.026	0.852306
A_44_P515593	XM_344148	XM_344148		0.01	1.026	0.843529
A_44_P224793	Sccpdh	NM_001013985	305021	0.01	1.026	0.878768
A_44_P240930	Scamp2	NM_023955	65168	0.01	1.026	0.865617
A_44_P180654	Dnali1	NM_001031647	298524	0.01	1.025	0.907549
A_43_P17460	Limd1_predicted	XM_236734		0.01	1.025	0.846453
A_44_P126134	Syvn1	XM_341999	361712	0.01	1.025	0.806317
A_44_P112119	Centd1_predicted	XM_223437		0.01	1.025	0.905629
A_44_P671340	TC561747	TC561747		0.01	1.025	0.839406
A_44_P388546	AA945052	AA945052	79238	0.01	1.025	0.869759
A_44_P321600	Tmem42_predicted	XM_343510	363171	0.01	1.025	0.842114
A_44_P315626	Ankrd15	XM_001055725	309429	0.01	1.025	0.832526
A_44_P311955	Prkce	NM_017171	29340	0.01	1.025	0.905207
A_44_P196625	Galnt9_predicted	XM_222253		0.01	1.025	0.845581
A_44_P366251	Hic2_predicted	XM_221303		0.01	1.025	0.895789
A_44_P117296	XM_346074	XM_346074		0.01	1.025	0.898118
A_42_P640641	Homer2	NM_053309	29547	0.01	1.025	0.887645
A_44_P557398	LOC499144	U78127	499144	0.01	1.025	0.834757
A_44_P421488	Trpc7	XM_225159	282822	0.01	1.025	0.885444
A_43_P23211	Ssfa2_predicted	XM_230036		0.01	1.025	0.874941
A_43_P10088	Rnaseh2a	NM_001013234	364974	0.01	1.025	0.830337
A_44_P578428	TC541225	TC541225		0.01	1.025	0.912299
A_44_P118805	Hgs	NM_019387	56084	0.01	1.025	0.815216
A_44_P940539	BG378253	BG378253	500434	0.01	1.025	0.899385
A_44_P241358	RGD1311066	XM_215901		0.01	1.025	0.825182
A_44_P278140	AW143234	AW143234		0.01	1.025	0.877892
A_43_P12137	Gnb3	NM_021858	60449	0.01	1.025	0.909496
A_42_P529445	RGD1308179_predicted	XM_214117		0.01	1.025	0.874666
A_44_P234393	RGD1565350_predicted	XM_342831		0.01	1.025	0.814725
A_44_P497174	Gramd1b_predicted	XM_217113	300644	0.01	1.025	0.8748
A_44_P975250	LOC683313	XM_001065178		0.01	1.024	0.944053
A_44_P785849	A_44_P785849	A_44_P785849		0.01	1.024	0.922714
A_44_P1047567	RGD1307395	XM_001055505		0.01	1.024	0.873981
A_44_P435149	AA850494	AA850494	245963	0.01	1.024	0.847343
A_43_P15455	Npffr1	NM_022291	64107	0.01	1.024	0.880027
A_44_P463324	AA799768	AA799768		0.01	1.024	0.875936
A_44_P128729	Akna_predicted	XM_342848		0.01	1.024	0.929449
A_44_P243316	LOC689779	XM_001071967		0.01	1.024	0.864994
A_44_P208021	Olr1452_predicted	NM_001000773	405048	0.01	1.024	0.942643
A_44_P407507	Kns2	XM_343114		0.01	1.024	0.86599
A_44_P165918	RGD1560633_predicted	XM_218078		0.01	1.024	0.844837
A_44_P222821	LOC366772	NM_001014264		0.01	1.023	0.927107
A_44_P142582	Zfp236_predicted	XM_225695	291409	0.01	1.023	0.835742
A_44_P321886	LOC679251	XM_001054244		0.01	1.023	0.840811
A_44_P281693	RGD1306274	XM_235561		0.01	1.023	0.819206

A_44_P171372	Vat1	NM_001033683	287721	0.01	1.023	0.914956
A_43_P11401	Mrgpre	NM_001002288	404660	0.01	1.023	0.899787
A_44_P396794	CB546357	CB546357	500420	0.01	1.023	0.856961
A_44_P509070	XM_343344	XM_343344		0.01	1.023	0.945022
A_44_P384698	XM_216321	XM_216321		0.01	1.023	0.867615
A_44_P409674	RGD1562149_predicted	XM_576071	500690	0.01	1.023	0.839868
A_44_P306810	Trpt1_predicted	XM_219539		0.01	1.023	0.827789
A_44_P261693	AI113125	AI113125	316102	0.01	1.023	0.842194
A_44_P490954	LOC682690	XM_001062637		0.01	1.023	0.911409
A_44_P104361	XM_214284	XM_214284		0.01	1.022	0.855569
A_44_P288828	XM_220490	XM_220490		0.01	1.022	0.874998
A_44_P384134	Zfp637	U78139		0.01	1.022	0.817811
A_44_P382643	AA899254	AA899254		0.01	1.022	0.847781
A_42_P825274	Hspb2	NM_130431	161476	0.01	1.022	0.855788
A_43_P17448	Rft1_predicted	XM_214262	290552	0.01	1.022	0.910204
A_44_P401062	RGD1359593	NM_001014266	366872	0.01	1.022	0.910829
A_44_P146476	BI285662	BI285662		0.01	1.022	0.859782
A_44_P452721	LOC316130	XM_236783	316130	0.01	1.022	0.825825
A_44_P669215	TC523303	TC523303		0.01	1.022	0.856594
A_43_P17229	RGD1307554_predicted	XM_214832		0.01	1.022	0.86722
A_44_P317661	BI274511	BI274511		0.01	1.022	0.917292
A_42_P656742	Vps28_predicted	XM_216962	300052	0.01	1.022	0.893987
A_44_P139611	AW530784	AW530784	494445	0.01	1.022	0.866143
A_43_P10292	siat7D	XM_001080040		0.01	1.022	0.910204
A_43_P18234	LOC679693	XM_001054608	679693	0.01	1.022	0.866343
A_44_P746478	TC523771	TC523771		0.01	1.022	0.899384
A_44_P914096	LOC363418	XM_576872		0.01	1.021	0.856594
A_44_P394657	BE107614	BE107614	25638	0.01	1.021	0.879469
A_44_P911967	Pias4	XM_001075850		0.01	1.021	0.872157
A_44_P529342	Syt15	NM_181632	306285	0.01	1.021	0.929449
A_44_P468816	RGD1304806_predicted	XM_001072414		0.01	1.021	0.925029
A_44_P269652	A_44_P269652	A_44_P269652		0.01	1.021	0.874678
A_44_P548740	Frs2_predicted	XM_235164		0.01	1.021	0.909358
A_44_P389612	LOC363146	NM_001014214	363146	0.01	1.021	0.86637
A_43_P19475	RGD1311123	NM_001014118	360539	0.01	1.021	0.930153
A_44_P251959	BE100164	BE100164	24674	0.01	1.021	0.9013
A_44_P428068	Rp9h_predicted	XM_343363		0.01	1.021	0.858485
A_44_P445903	Dnajc13_predicted	XM_343462		0.01	1.021	0.879752
A_42_P556316	Mtus1	BC072537	306487	0.01	1.021	0.909844
A_44_P224705	Inpp4a	NM_031002	80849	0.01	1.021	0.874686
A_44_P974203	ENSRNOT00000049792	ENSRNOT00000049792		0.01	1.021	0.890812
A_44_P705222	CF109741	CF109741		0.01	1.021	0.941359
A_44_P166087	LOC294072	XM_220029		0.01	1.020	0.925675
A_44_P393521	Col23a1	NM_181636	353303	0.01	1.020	0.908845
A_44_P355628	Ykt6	NM_031692	64351	0.01	1.020	0.899763
A_43_P21520	Lrp8_predicted	XM_342877	362558	0.01	1.020	0.942097
A_44_P764671	TC561998	TC561998		0.01	1.020	0.903889
A_44_P490490	AI044674	AI044674		0.01	1.020	0.867043
A_44_P114919	RGD1559590_predicted	XM_221419		0.01	1.020	0.894713
A_43_P15584	Mbtps1	NM_053569	89842	0.01	1.020	0.845178
A_43_P19328	LOC691506	XM_001077297		0.01	1.020	0.881349
A_44_P825142	TC532596	TC532596		0.01	1.020	0.877156
A_44_P236412	A_44_P236412	A_44_P236412		0.01	1.020	0.895238
A_44_P913444	RGD1564382_predicted	XM_573967		0.01	1.020	0.885113
A_44_P290539	Mycn_mapped	NM_001013096	298894	0.01	1.020	0.879256
A_44_P111816	Adcy4	NM_019285	54223	0.01	1.020	0.919425
A_44_P517303	Gstm5	BQ191682	64352	0.01	1.020	0.895089
A_43_P22230	Alms1_predicted	XM_216189		0.01	1.019	0.864534
A_44_P551842	Abtb2	NM_134403	171440	0.01	1.019	0.908157
A_44_P681989	Tmem32_predicted	XM_001057974		0.01	1.019	0.890035
A_44_P368279	Odz3_predicted	XM_224841	306451	0.01	1.019	0.895889
A_43_P12402	Slc27a5	NM_024143	79111	0.01	1.019	0.932286
A_43_P21295	Cdc7_predicted	XM_341183		0.01	1.019	0.92387

A_44_P729690	BC090008	BC090008	25271	0.01	1.019	0.916744
A_44_P199560	AA899832	AA899832	25282	0.01	1.019	0.875996
A_43_P23278	BF565010	BF565010	308218	0.01	1.019	0.900491
A_43_P11329	Zfp574	NM_001024258	308434	0.01	1.019	0.900542
A_44_P141594	AW915371	AW915371		0.01	1.019	0.869207
A_44_P698981	XM_229267	XM_229267		0.01	1.019	0.9201
A_44_P764252	TC523241	TC523241		0.01	1.019	0.896895
A_44_P356445	Lrig2_predicted	XM_227547		0.01	1.019	0.934445
A_44_P171143	LOC316856	XM_229366		0.01	1.019	0.865577
A_44_P118880	Kcnip4	NM_181365	259243	0.01	1.019	0.915477
A_44_P284097	A_44_P284097	A_44_P284097		0.01	1.019	0.883233
A_43_P15431	Mterf	NM_053499	85261	0.01	1.019	0.860213
A_42_P709692	Pnrc1	NM_173322	286988	0.01	1.018	0.908062
A_44_P1040230	Ubl4a_predicted	XM_215228		0.01	1.018	0.894087
A_42_P808973	Marcksl1	NM_030862	81520	0.01	1.018	0.909377
A_44_P538789	XM_222813	XM_222813		0.01	1.018	0.885416
A_43_P12233	Sult1b1	NM_022513	64305	0.01	1.018	0.908176
A_44_P1000973	RGD1307832_predicted	XM_231046		0.01	1.018	0.867245
A_44_P222954	A_44_P222954	A_44_P222954		0.01	1.018	0.885846
A_44_P252545	Grik2	NM_019309	54257	0.01	1.018	0.89215
A_44_P379791	RGD1309809	NM_001024868	292898	0.01	1.018	0.903223
A_44_P162457	Zfr	XM_345169	365703	0.01	1.018	0.907571
A_44_P438791	Trim29_predicted	XM_236207		0.01	1.018	0.905501
A_44_P1070933	BC070518	BC070518		0.01	1.018	0.876049
A_44_P321527	Ppp1r13b_predicted	XM_234555		0.01	1.018	0.884516
A_44_P713474	A_44_P713474	A_44_P713474		0.01	1.018	0.915951
A_44_P105831	Clic4	NM_031818	83718	0.01	1.018	0.916044
A_44_P520718	AE017191	AE017191		0.01	1.018	0.915269
A_43_P11561	Arnt	NM_012780	25242	0.01	1.018	0.876948
A_44_P437236	AW916017	AW916017		0.01	1.018	0.931463
A_42_P568943	RGD1311874_predicted	XM_217167		0.01	1.018	0.912805
A_44_P264901	Cnot6l_predicted	XM_341191		0.01	1.018	0.9264
A_44_P307029	Terf2ip	NM_001013143	307861	0.01	1.018	0.86265
A_44_P682849	Rundc1_predicted	XM_001081465		0.01	1.018	0.899723
A_44_P960755	TC540294	TC540294		0.01	1.018	0.941035
A_44_P667224	RGD1560565_predicted	XM_001062059		0.01	1.018	0.895238
A_44_P135944	Adcy9_predicted	XM_220178		0.01	1.018	0.915559
A_44_P455065	LOC691318	XM_001077680	691318	0.01	1.018	0.888139
A_44_P786208	TC529002	TC529002		0.01	1.017	0.885193
A_43_P11636	Hspe1	NM_012966	25462	0.01	1.017	0.911422
A_43_P19606	Slc12a7	XM_001060536		0.01	1.017	0.91925
A_44_P133672	A_44_P133672	A_44_P133672		0.01	1.017	0.932176
A_44_P297501	LOC678993	XM_001054150		0.01	1.017	0.91813
A_44_P130722	Nmb_predicted	XM_218815		0.01	1.017	0.885444
A_42_P554373	Tmprss2	NM_130424	156435	0.01	1.017	0.914984
A_42_P459306	RGD1305687_predicted	XM_237786	287453	0.01	1.017	0.909424
A_42_P627330	Mrps35_predicted	XM_216306		0.01	1.017	0.911317
A_43_P16685	Abca3	XM_220219	302973	0.01	1.017	0.893628
A_44_P899877	TC523758	TC523758		0.01	1.017	0.921605
A_44_P428980	XM_224059	XM_224059		0.01	1.017	0.958318
A_44_P372122	Tubg1	NM_145778	252921	0.01	1.017	0.896177
A_44_P909601	BF290439	BF290439		0.01	1.017	0.877265
A_43_P12287	Ppp1r1a	NM_022676	58977	0.01	1.017	0.918855
A_44_P361408	LOC691922	XM_343193	691922	0.01	1.017	0.932965
A_44_P264124	Gps2_predicted	XM_220615		0.01	1.017	0.868341
A_44_P823554	RGD1309666_predicted	XM_001066604		0.01	1.017	0.914678
A_44_P655449	TC565874	TC565874		0.01	1.016	0.91651
A_44_P561538	TC522397	TC522397		0.01	1.016	0.874557
A_43_P14179	Ap2m1	NM_053837	116563	0.01	1.016	0.893442
A_42_P624333	Akr7a2	NM_134407	171445	0.01	1.016	0.892855
A_43_P19945	RGD1308601_predicted	XM_225699		0.01	1.016	0.889674
A_44_P429469	Hemk2_predicted	XM_213675	288309	0.01	1.016	0.91651
A_44_P347105	XM_345436	XM_345436		0.01	1.016	0.88424

A_44_P564310	TC560120	TC560120		0.01	1.016	0.942832
A_44_P880046	DV719732	DV719732	361242	0.01	1.016	0.944903
A_44_P904790	A_44_P904790	A_44_P904790		0.01	1.016	0.890782
A_44_P378990	AI170568	AI170568	29740	0.01	1.015	0.955049
A_44_P769938	CB313746	CB313746		0.01	1.015	0.92578
A_43_P21748	Aim1_predicted	XM_001067657		0.01	1.015	0.940943
A_44_P625125	TC551570	TC551570		0.01	1.015	0.929454
A_44_P522063	AI102612	AI102612	300235	0.01	1.015	0.929454
A_44_P198926	LOC304558	XM_222260	304558	0.01	1.015	0.938557
A_44_P527921	Lig3	NM_001012011	303369	0.01	1.015	0.92234
A_44_P429254	LOC316794	XM_237593		0.01	1.015	0.910204
A_42_P577445	LOC364802	XM_344643	364802	0.01	1.015	0.883621
A_44_P395522	Camk1g	NM_182842	171358	0.01	1.015	0.970805
A_43_P20118	Suclg2	XM_001074551		0.01	1.015	0.94184
A_44_P384353	Tead2_mapped	XM_218630		0.01	1.015	0.943912
A_44_P174445	Lrrn2_predicted	XM_222670	289020	0.01	1.015	0.939472
A_43_P20567	RGD1561144_predicted	XM_226482		0.01	1.015	0.926925
A_44_P402936	Iars2_predicted	XM_001065536		0.01	1.015	0.92492
A_44_P595505	TC563599	TC563599		0.01	1.015	0.924231
A_43_P20299	Cbx3	NM_001008313		0.01	1.014	0.94184
A_44_P578376	TC542956	TC542956		0.01	1.014	0.913473
A_44_P184774	Gosr1	NM_053584	94189	0.01	1.014	0.937386
A_44_P484755	Pctk1	NM_001004132	81741	0.01	1.014	0.928325
A_44_P267255	XM_215186	XM_215186		0.01	1.014	0.905501
A_44_P932586	TC551627	TC551627		0.01	1.014	0.921513
A_44_P134672	Snapc3	NM_001013212	362537	0.01	1.014	0.916396
A_44_P156782	RGD1311847	NM_001013879	290615	0.01	1.014	0.91813
A_44_P469069	Mapk8ip3	XM_220232	302983	0.01	1.014	0.918538
A_44_P496025	Eml4_predicted	XM_233839		0.01	1.014	0.905127
A_44_P116841	Spata13_predicted	XM_224253	305938	0.01	1.014	0.926828
A_44_P1025208	Mrpl18_predicted	XM_214751		0.01	1.014	0.907951
A_43_P18093	XM_235674	XM_235674		0.01	1.014	0.932857
A_44_P224987	RGD1561599_predicted	XM_345363	366065	0.01	1.014	0.931247
A_44_P272294	Asxl2_predicted	XM_233937		0.01	1.014	0.910204
A_44_P607386	TC555733	TC555733		0.01	1.014	0.949257
A_44_P876045	XM_580064	XM_580064		0.01	1.014	0.952291
A_44_P438193	Ap2a2	NM_031008	81637	0.01	1.013	0.933242
A_44_P400520	Nfyc	NM_012866	25337	0.01	1.013	0.901784
A_43_P10101	TC556091	TC556091		0.01	1.013	0.933183
A_44_P346491	Sfrs5	NM_019257	29667	0.01	1.013	0.921788
A_44_P554799	Rab11a	NM_031152	81830	0.01	1.013	0.908176
A_44_P649990	RGD1565549_predicted	XM_001059164		0.01	1.013	0.91925
A_44_P714860	LOC502663	XM_001080969		0.01	1.013	0.944063
A_44_P325368	AW915440	AW915440	679907	0.01	1.013	0.90491
A_44_P210116	Ptk9	NM_001008521	315265	0.01	1.013	0.942892
A_44_P133592	Mst1r_predicted	XM_236628		0.01	1.013	0.911784
A_44_P267465	Hsf4_predicted	XM_214668		0.01	1.013	0.926828
A_43_P21976	RGD1565738_predicted	XM_223405	305342	0.01	1.013	0.90632
A_43_P22483	A_43_P22483	A_43_P22483		0.01	1.013	0.932176
A_44_P293248	A_44_P293248	A_44_P293248		0.01	1.013	0.926946
A_44_P548842	LOC287533	NM_001013859	287533	0.01	1.013	0.935026
A_44_P330479	Fnta	NM_012847	25318	0.01	1.013	0.925638
A_44_P241409	Vgll4	NM_001015004	297523	0.01	1.013	0.926192
A_44_P699909	RGD1564784_predicted	XM_574833	499508	0.01	1.013	0.96926
A_44_P479305	AA924557	AA924557	309111	0.01	1.013	0.937916
A_44_P412092	Thop1	NM_172075	64517	0.01	1.012	0.944035
A_44_P807394	TC555986	TC555986		0.01	1.012	0.938223
A_44_P543718	ENSRNOT00000016167	ENSRNOT00000016167		0.01	1.012	0.951986
A_44_P132039	Yipf2	NM_001014208	363027	0.01	1.012	0.902349
A_44_P296063	BF546967	BF546967		0.01	1.012	0.939357
A_44_P255694	LOC302733	XR_007852	302733	0.01	1.012	0.944035
A_44_P342892	AA819108	AA819108		0.01	1.012	0.955361
A_44_P166142	A_44_P166142	A_44_P166142		0.01	1.012	0.952882

A_44_P929345	LOC501289	XM_576703		0.01	1.012	0.915398
A_44_P991616	Ube1dc1	NM_001009669	300968	0.01	1.012	0.932176
A_44_P623316	LOC499120	XM_574414	499120	0.01	1.012	0.926909
A_42_P668126	Sema6c	NM_017308	29744	0.01	1.012	0.919605
A_44_P250983	LOC680451	XM_001056969	680451	0.01	1.012	0.937916
A_44_P473217	RGD1309708	NM_001014131	360867	0.01	1.012	0.923405
A_44_P623926	TC560843	TC560843		0.01	1.012	0.930995
A_44_P140244	LOC682447	XM_001061206		0.01	1.012	0.975194
A_44_P123571	Acy1	AY580165	300981	0.01	1.012	0.929986
A_44_P622493	TC538904	TC538904		0.01	1.012	0.943813
A_44_P210492	Syt2	NM_012665	24805	0.00	1.012	0.924351
A_44_P520858	TC541935	TC541935		0.00	1.012	0.925638
A_44_P421267	BG153357	BG153357		0.00	1.011	0.95679
A_43_P18482	TC542996	TC542996		0.00	1.011	0.970032
A_44_P390362	Ahsg	NM_012898	25373	0.00	1.011	0.923854
A_44_P697841	BF567145	BF567145	287622	0.00	1.011	0.961422
A_43_P20314	XM_341813	XM_341813		0.00	1.011	0.929428
A_44_P744575	A_44_P744575	A_44_P744575		0.00	1.011	0.935026
A_44_P439658	AI105088	AI105088	287419	0.00	1.011	0.932403
A_44_P638511	Paqr5	NM_001014092	315741	0.00	1.011	0.944903
A_44_P384017	Sncg	NM_031688	64347	0.00	1.011	0.942832
A_42_P501877	Gls	NM_012569	24398	0.00	1.011	0.944053
A_44_P189854	A_44_P189854	A_44_P189854		0.00	1.011	0.934657
A_44_P320669	Zp3	NM_053762	114639	0.00	1.011	0.954499
A_44_P588967	AI008244	AI008244		0.00	1.011	0.946957
A_43_P14099	BG665505	BG665505		0.00	1.011	0.971
A_44_P549445	Cs	NM_130755	170587	0.00	1.011	0.944903
A_44_P427774	AW920764	AW920764		0.00	1.010	0.967118
A_44_P189646	MGC125073	XM_001059346		0.00	1.010	0.932965
A_44_P140491	Slc35d1_predicted	XM_216462		0.00	1.010	0.944179
A_43_P18768	Ace2	NM_001012006	302668	0.00	1.010	0.943912
A_44_P1042125	LOC682709	XM_001060585		0.00	1.010	0.945438
A_42_P633709	RGD1305038_predicted	XM_234920	314627	0.00	1.010	0.94952
A_44_P1010659	LOC368001	XM_001077499		0.00	1.010	0.931239
A_44_P546040	LOC680102	XM_001055031		0.00	1.010	0.961422
A_43_P18312	Ccdc59_predicted	XM_235154		0.00	1.010	0.921363
A_43_P14163	Odc1	NM_012615	24609	0.00	1.010	0.943912
A_44_P610159	TC561947	TC561947		0.00	1.010	0.944035
A_44_P352055	Adamtsl5_predicted	XM_234919		0.00	1.010	0.968662
A_43_P12031	Slc14a1	NM_019346	54301	0.00	1.010	0.975461
A_44_P320894	Vamp1	NM_013090	25624	0.00	1.010	0.935414
A_44_P1005031	Fbxo7	NM_001012222	366854	0.00	1.009	0.940038
A_42_P734749	Lrfn3_predicted	XM_218483		0.00	1.009	0.972504
A_43_P12618	Nr1h3	NM_031627	58852	0.00	1.009	0.937916
A_44_P607659	TC541160	TC541160		0.00	1.009	0.946404
A_44_P421333	Nr2f2	NM_080778	113984	0.00	1.009	0.949583
A_44_P398550	RGD1305883_predicted	XM_226571	307949	0.00	1.009	0.953977
A_44_P914022	LOC679198	XM_001055196		0.00	1.009	0.969695
A_44_P365882	RGD1308697	NM_001014046	311328	0.00	1.009	0.958613
A_43_P12815	Ptp4a2	NM_053475	85237	0.00	1.009	0.943912
A_44_P436181	RGD1561519_predicted	XM_001067576		0.00	1.009	0.955798
A_44_P920120	LOC686098	XM_001066536	686098	0.00	1.008	0.978164
A_44_P361212	TC518066	TC518066		0.00	1.008	0.964345
A_44_P251376	Brwd1_predicted	XM_221627		0.00	1.008	0.962622
A_43_P17958	LOC683259	XM_001065140		0.00	1.008	0.9556
A_44_P386888	RGD1311147_predicted	XM_214200		0.00	1.008	0.968337
A_43_P12035	Timm23	NM_019352	54312	0.00	1.008	0.946937
A_44_P110307	XM_234897	XM_234897		0.00	1.008	0.964166
A_44_P462092	LOC500893	NM_001029926	500893	0.00	1.008	0.951116
A_44_P163652	Spred2	XM_223647		0.00	1.008	0.941984
A_44_P559041	Ighmbp2	BC099790	29532	0.00	1.008	0.969819
A_44_P221650	LOC499624	XM_001057984		0.00	1.008	0.953526
A_43_P21741	RGD1307830_predicted	XM_222746	304863	0.00	1.008	0.939263

A_44_P342712	Os-9	NM_001007265	362891	0.00	1.008	0.962421
A_44_P157179	Tcfeb	NM_001025707	316214	0.00	1.008	0.953581
A_44_P358782	Wdr47	XM_001081957	310785	0.00	1.008	0.944179
A_44_P230030	Snap29	NM_053810	116500	0.00	1.008	0.948171
A_44_P431587	RGD1561116_predicted	XM_343061	362737	0.00	1.008	0.957882
A_44_P407543	RGD1306932	NM_001024996	304346	0.00	1.008	0.945022
A_44_P270016	LOC299943	XR_008447	299943	0.00	1.007	0.960459
A_44_P219788	Surf2	NM_001033866	619345	0.00	1.007	0.958302
A_44_P652500	LOC687014	XM_001076731		0.00	1.007	0.943544
A_44_P177162	RGD1310722_predicted	XM_231617	312248	0.00	1.007	0.953837
A_44_P419472	A_44_P419472	A_44_P419472		0.00	1.007	0.946957
A_42_P630463	Abcb9	NM_022238	63886	0.00	1.007	0.957008
A_44_P929321	A_44_P929321	A_44_P929321		0.00	1.007	0.954103
A_44_P177169	Fgl2	NM_053455	84586	0.00	1.007	0.977865
A_43_P11098	Cdc42bpb	NM_053620	113960	0.00	1.007	0.959255
A_42_P707937	Ap2b1	NM_080583	140670	0.00	1.007	0.943196
A_44_P867229	Btbd3_predicted	XM_001081301		0.00	1.007	0.968662
A_43_P15598	Cd79b	NM_133533	171055	0.00	1.007	0.973427
A_44_P265138	RGD1564626_predicted	XM_226511	292045	0.00	1.007	0.956686
A_44_P233450	AI229612	AI229612		0.00	1.007	0.963227
A_44_P511529	AW143917	AW143917		0.00	1.007	0.976203
A_44_P622645	RGD1559763_predicted	XM_001067753		0.00	1.007	0.970666
A_44_P434088	RGD1309561_predicted	XM_213277		0.00	1.007	0.983431
A_44_P289460	RGD1564664_predicted	XM_575179		0.00	1.006	0.98024
A_44_P417470	RGD1560364_predicted	XM_343418	363087	0.00	1.006	0.979524
A_44_P180786	Plekhb2_predicted	XM_217372		0.00	1.006	0.972237
A_44_P205401	AA858581	AA858581	288669	0.00	1.006	0.976304
A_44_P414527	Efna2	XM_234903	84358	0.00	1.006	0.983801
A_42_P708569	Ube3a_predicted	XM_341867	361585	0.00	1.006	0.967909
A_44_P106581	AW143880	AW143880	362687	0.00	1.006	0.973615
A_44_P1047638	Ndfip2_predicted	XM_341374		0.00	1.006	0.978488
A_43_P15426	Trpc3	NM_021771		0.00	1.005	0.979424
A_43_P17864	St7l	NM_001007639	295344	0.00	1.005	0.971965
A_44_P482700	Ttyh1_predicted	XM_218263		0.00	1.005	0.966265
A_44_P518284	Eif4ebp2	NM_001033069	361845	0.00	1.005	0.964345
A_44_P1018997	Tmco1	NM_001009631	289196	0.00	1.005	0.969305
A_42_P538670	Cybb	NM_023965	66021	0.00	1.005	0.976304
A_43_P10831	Rnpc2	NM_001013207	362251	0.00	1.005	0.978946
A_43_P10788	TC555260	TC555260		0.00	1.005	0.97358
A_44_P728623	TC529954	TC529954		0.00	1.005	0.975522
A_42_P602724	Ubd	NM_053299	29168	0.00	1.005	0.979581
A_44_P473603	A_44_P473603	A_44_P473603		0.00	1.005	0.978946
A_44_P274255	XM_344172	XM_344172		0.00	1.005	0.967071
A_43_P22243	CB544723	CB544723	171107	0.00	1.005	0.9844
A_44_P334556	Tmtc2_predicted	XM_001080732		0.00	1.005	0.96819
A_44_P556850	Arhgef12	NM_001013246	367072	0.00	1.005	0.97397
A_44_P203671	XM_219350	XM_219350		0.00	1.005	0.975522
A_44_P916009	TC560678	TC560678		0.00	1.005	0.983801
A_44_P729533	TC535635	TC535635		0.00	1.005	0.974685
A_44_P509129	Tex2	XM_221043	303611	0.00	1.005	0.980226
A_44_P359121	Dcamkl3_predicted	XM_236661	316023	0.00	1.004	0.987713
A_44_P358921	Eif5	NM_020075	56783	0.00	1.004	0.978986
A_44_P312089	LOC316326	NM_001014100	316326	0.00	1.004	0.970524
A_44_P497007	RGD1309594	NM_001008351	309681	0.00	1.004	0.971009
A_44_P351855	RGD1305440	NM_001033997	296161	0.00	1.004	0.976278
A_44_P529375	Olr1579_predicted	NM_001000078	289239	0.00	1.004	0.986532
A_44_P319169	XM_216604	XM_216604		0.00	1.004	0.973044
A_44_P131036	Sdccag3	NM_001013135	306322	0.00	1.004	0.968912
A_44_P340606	BF288191	BF288191	548326	0.00	1.004	0.984768
A_44_P127749	BF289788	BF289788	360916	0.00	1.004	0.991264
A_44_P898713	A_44_P898713	A_44_P898713		0.00	1.004	0.981119
A_44_P845705	A_44_P845705	A_44_P845705		0.00	1.004	0.9817
A_44_P648619	AI144728	AI144728		0.00	1.004	0.980556

A_44_P355493	AW915744	AW915744	500034	0.00	1.004	0.986967
A_44_P335458	Dhx38_predicted	XM_238048		0.00	1.004	0.978946
A_44_P412856	AABR03062217	AABR03062217		0.00	1.004	0.976375
A_44_P804806	DV726988	DV726988		0.00	1.004	0.980226
A_44_P471305	Rcor1_predicted	XM_234546		0.00	1.004	0.985473
A_43_P21938	Ulk2_predicted	XM_220541	303206	0.00	1.003	0.975919
A_44_P173239	Ap4b1_predicted	XM_227538		0.00	1.003	0.978924
A_44_P396362	LOC362683	XM_001053592	362683	0.00	1.003	0.978804
A_44_P190088	A_44_P190088	A_44_P190088		0.00	1.003	0.983445
A_44_P220696	AA924993	AA924993	313587	0.00	1.003	0.979531
A_44_P140557	Dock6_predicted	XM_345909		0.00	1.003	0.980022
A_43_P20370	Diap1_predicted	XM_226014		0.00	1.003	0.98544
A_44_P747170	TC527024	TC527024		0.00	1.003	0.987831
A_44_P806655	LOC363146	NM_001014214	363146	0.00	1.003	0.980226
A_44_P251425	AA926345	AA926345		0.00	1.003	0.989673
A_43_P16786	Nudt22	NM_199090	293703	0.00	1.003	0.981119
A_44_P140028	Ick	NM_138886	84411	0.00	1.003	0.982397
A_44_P428739	Shc3	XM_001054242		0.00	1.003	0.987852
A_42_P564712	Taok2	NM_022702	64666	0.00	1.003	0.97999
A_44_P660621	TC561185	TC561185		0.00	1.002	0.98506
A_44_P276739	XM_231150	XM_231150		0.00	1.002	0.984854
A_44_P314386	Cohh1_predicted	XM_001058951		0.00	1.002	0.98905
A_44_P429354	LOC300481	XM_001054228		0.00	1.002	0.984164
A_44_P1037131	Actr1a_predicted	XM_238177		0.00	1.002	0.986174
A_44_P281216	Nudt8_predicted	XM_341975	361692	0.00	1.002	0.984743
A_44_P299830	RGD1359508	NM_001009707	361941	0.00	1.002	0.99284
A_44_P952038	A_44_P952038	A_44_P952038		0.00	1.002	0.990118
A_44_P431577	A_44_P431577	A_44_P431577		0.00	1.002	0.985051
A_44_P794522	BM384275	BM384275		0.00	1.002	0.985801
A_44_P204204	A_44_P204204	A_44_P204204		0.00	1.002	0.993218
A_44_P679698	BG669046	BG669046		0.00	1.002	0.986963
A_44_P125226	AW532988	AW532988	114244	0.00	1.002	0.991871
A_42_P723851	Plcl1	NM_053456	84587	0.00	1.002	0.990518
A_44_P1030168	Ppfia2_predicted	XM_343204		0.00	1.002	0.994241
A_44_P929254	A_44_P929254	A_44_P929254		0.00	1.002	0.987232
A_44_P664191	Arhgef2	NM_001012079	310635	0.00	1.002	0.992875
A_44_P395832	Dcbld2	NM_130419	155696	0.00	1.002	0.994279
A_44_P484869	Arhgef5	XM_342676	140898	0.00	1.002	0.990518
A_44_P106775	BG662522	BG662522		0.00	1.001	0.993457
A_44_P596904	AABR03113706	AABR03113706		0.00	1.001	0.991838
A_44_P238843	MGC116266	NM_001024867	290775	0.00	1.001	0.991227
A_43_P14655	MGC94704	NM_001006986	300447	0.00	1.001	0.989049
A_43_P22901	XM_341207	XM_341207		0.00	1.001	0.993218
A_44_P191986	ENSRNOT00000040238	ENSRNOT00000040238		0.00	1.001	0.993218
A_44_P727274	RGD1306894_predicted	XM_344763	364996	0.00	1.001	0.992626
A_44_P822121	TC551985	TC551985		0.00	1.001	0.995107
A_44_P226802	Htr4	NM_012853	25324	0.00	1.001	0.997232
A_44_P1060495	Znf618_predicted	XM_233016	313253	0.00	1.001	0.995547
A_44_P245767	A_44_P245767	A_44_P245767		0.00	1.001	0.995161
A_43_P17336	Stac_predicted	XM_343491	363152	0.00	1.001	0.99556
A_44_P207963	Afap	NM_080900	140935	0.00	1.001	0.995638
A_44_P1021456	LOC689249	XM_001070121	291860	0.00	1.001	0.995701
A_44_P327301	AW141564	AW141564	24375	0.00	1.001	0.996244
A_42_P751720	LOC683720	XM_235687		0.00	1.001	0.998366
A_44_P196793	Spap1_predicted	XM_227483		0.00	1.001	0.996844
A_44_P102708	RGD1310383_predicted	XM_222189		0.00	1.001	0.997131
A_44_P536282	Spnb2	NM_001013130	305614	0.00	1.000	0.996865
A_44_P261243	Map3k11	NM_001013150	309168	0.00	1.000	0.996753
A_44_P915255	TC557961	TC557961		0.00	1.000	0.999116
A_44_P355620	BF549650	BF549650		0.00	1.000	0.999315
A_44_P1003357	RGD1561045_predicted	XM_228032	309643	0.00	1.000	0.999584
A_44_P947069	TC545323	TC545323		0.00	1.000	0.999446
A_44_P255254	Ywhab	NM_019377	56011	0.00	1.000	0.999446

A_44_P513945	AA818874	AA818874		0.00	1.000	0.999758
A_42_P831552	BG665395	BG665395	171433	0.00	1.000	1
A_44_P272383	Spats2_predicted	XM_001061947		0.00	-1.000	0.999116
A_43_P17140	Fliih	NM_001008279	287375	0.00	-1.000	0.999116
A_44_P731728	TC546221	TC546221		0.00	-1.000	0.999307
A_42_P491318	XM_213717	XM_213717		0.00	-1.000	0.999024
A_44_P452291	Hk2	NM_012735	25059	0.00	-1.000	0.998599
A_44_P341251	CK473284	CK473284	690263	0.00	-1.000	0.998874
A_43_P12803	Arid4b	NM_053421	84481	0.00	-1.000	0.998112
A_44_P189043	Gphn	NM_022865	64845	0.00	-1.000	0.998724
A_42_P518316	RGD1562860_predicted	XM_341223	360945	0.00	-1.000	0.998599
A_42_P507877	Arhgap23_predicted	XM_220918	303501	0.00	-1.000	0.998366
A_44_P231399	Samm50	NM_001004241	300111	0.00	-1.001	0.99641
A_44_P370199	A4galt	NM_022240	63888	0.00	-1.001	0.996865
A_44_P490004	Btbd9	NM_001013073	294318	0.00	-1.001	0.996918
A_44_P411139	BQ193604	BQ193604	292804	0.00	-1.001	0.997084
A_42_P676838	Alkbh5_predicted	XM_220525	303193	0.00	-1.001	0.992279
A_44_P201585	XM_342607	XM_342607		0.00	-1.001	0.990879
A_42_P729705	Sugt1	XM_214242	290408	0.00	-1.001	0.992058
A_44_P182810	RGD1564725_predicted	XM_341459		0.00	-1.001	0.995259
A_44_P460456	Rae1	NM_001033708	362281	0.00	-1.001	0.990518
A_44_P1024388	RGD735106	NM_198766	308060	0.00	-1.001	0.994072
A_44_P196685	Eif4g2	XM_001077834		0.00	-1.001	0.992044
A_44_P871622	Syvn1	XM_341999	361712	0.00	-1.001	0.991871
A_44_P776983	TC556781	TC556781		0.00	-1.001	0.994342
A_44_P145235	Zfp672	NM_001007669	303165	0.00	-1.001	0.992305
A_44_P114227	Xlas	NM_001024823	24896	0.00	-1.001	0.993038
A_44_P513322	Txk	NM_001024255	305311	0.00	-1.002	0.992569
A_44_P518559	RGD1560397_predicted	XM_340876	360596	0.00	-1.002	0.989049
A_44_P1028007	Clic1	NM_001002807	406864	0.00	-1.002	0.987986
A_44_P993651	RGD1565363_predicted	XM_001079962		0.00	-1.002	0.986126
A_44_P337969	XM_234037	XM_234037		0.00	-1.002	0.987831
A_44_P483992	LOC678910	XM_001053761		0.00	-1.002	0.990118
A_44_P790674	LOC498289	XM_573517	498289	0.00	-1.002	0.98905
A_44_P403084	Cdc42se1	NM_001039044	499672	0.00	-1.002	0.98569
A_44_P279452	AA957814	AA957814		0.00	-1.002	0.983801
A_44_P115800	AA818085	AA818085	25577	0.00	-1.002	0.988594
A_42_P491119	Lgals4	NM_012975	25474	0.00	-1.002	0.990094
A_44_P402445	Nefh	NM_012607	24587	0.00	-1.002	0.986107
A_44_P902718	DN933938	DN933938		0.00	-1.003	0.986049
A_44_P116329	Olr1431_predicted	NM_001000778	405054	0.00	-1.003	0.987986
A_44_P605545	LOC365156	XR_007561	308200	0.00	-1.003	0.98569
A_44_P194614	LOC690407	XM_001071548		0.00	-1.003	0.98544
A_44_P161622	Pbx3_predicted	XM_231158		0.00	-1.003	0.985473
A_44_P733485	TC562868	TC562868		0.00	-1.003	0.982234
A_44_P396460	Scap_predicted	XM_217279		0.00	-1.003	0.979369
A_44_P194416	Zdhhc24	NM_001039100	293665	0.00	-1.003	0.990391
A_44_P380441	Rgs19	NM_021661	59293	0.00	-1.003	0.984982
A_44_P348026	RGD1560397_predicted	XM_340876	360596	0.00	-1.003	0.979706
A_44_P400857	Fts	NM_001011926	291906	0.00	-1.003	0.983498
A_44_P555236	Atp6v1g2	NM_212490	368044	0.00	-1.003	0.984982
A_44_P198035	AI113177	AI113177	114121	0.00	-1.003	0.981685
A_44_P102574	AB097858	AB097858		0.00	-1.003	0.986963
A_42_P649879	Timm17b_predicted	XM_228758		0.00	-1.003	0.97481
A_44_P282024	LOC366889	NM_001039340	366889	0.00	-1.003	0.990332
A_44_P348111	Mtmr4_predicted	XM_001081167		0.00	-1.003	0.980886
A_44_P325911	Sirt4_predicted	XM_222243		0.00	-1.003	0.985051
A_44_P1030276	Dap3	NM_001011950	295238	0.00	-1.003	0.976375
A_44_P367550	Sqstm1	NM_175843		0.00	-1.004	0.980022
A_44_P1030897	LOC290549	NM_207589	290549	0.00	-1.004	0.979473
A_44_P486304	Ocil	NM_130402	113937	0.00	-1.004	0.982107
A_44_P193644	RGD1561065_predicted	XM_001062296		0.00	-1.004	0.978946
A_44_P282482	Atp2a2	NM_017290	29693	0.00	-1.004	0.979991

A_44_P449108	AI043579	AI043579		0.00	-1.004	0.984164
A_44_P400560	XM_220091	XM_220091		0.00	-1.004	0.979522
A_44_P187789	LOC682838	XM_001063333		0.00	-1.004	0.975241
A_44_P1034209	Lamb3	XM_223087	305078	0.00	-1.004	0.981851
A_44_P897314	AA850930	AA850930		0.00	-1.004	0.983498
A_44_P173622	LOC303067	NM_001013966	303067	0.00	-1.004	0.976848
A_43_P12940	Bak1	NM_053812	116502	0.00	-1.004	0.975364
A_42_P541625	LOC679014	XM_001054221		0.00	-1.004	0.975757
A_44_P649177	RGD1564560_predicted	XM_576400		0.00	-1.004	0.972237
A_44_P319349	Pkp2	XM_213560	287925	0.00	-1.004	0.985051
A_43_P12529	Stxbp2	NM_031126	81804	0.00	-1.004	0.980884
A_44_P316286	RGD1562406_predicted	XM_213736	288559	0.00	-1.004	0.97358
A_44_P552517	Npr3	NM_012868	25339	0.00	-1.004	0.975123
A_44_P403816	AW142588	AW142588		0.00	-1.004	0.977505
A_44_P435703	RGD1303271	NM_001004268	313018	0.00	-1.004	0.976455
A_44_P126868	Fkbp1b	NM_022675	58950	0.00	-1.004	0.972651
A_44_P375185	Slc39a14_predicted	XM_224332		0.00	-1.004	0.978558
A_43_P14528	Lactb2	NM_001024247	297768	0.00	-1.004	0.978549
A_44_P934549	BF522944	BF522944		0.00	-1.004	0.976613
A_44_P350652	BI395724	BI395724	83805	0.00	-1.004	0.983717
A_44_P139763	Bet1	NM_019251	29631	0.00	-1.005	0.986758
A_44_P837289	RGD1565622_predicted	XM_580086		0.00	-1.005	0.970032
A_42_P480346	RGD1565358_predicted	XM_235062		0.00	-1.005	0.964759
A_44_P324140	LOC681140	XM_001060456		0.00	-1.005	0.96869
A_44_P448710	Cdw92	NM_053492	85254	0.00	-1.005	0.975675
A_44_P306900	LOC310487	XM_001065668		0.00	-1.005	0.976203
A_44_P999404	Cd164	NM_031812	83689	0.00	-1.005	0.96804
A_44_P468681	Krtcap2_predicted	XM_215616		0.00	-1.005	0.962672
A_44_P405183	ENSRNOT00000048838	ENSRNOT00000048838		0.00	-1.005	0.972249
A_44_P706821	TC547555	TC547555		0.00	-1.005	0.975522
A_44_P766691	DY472283	DY472283		0.00	-1.005	0.981288
A_44_P180262	RGD1562397_predicted	XM_344178		0.00	-1.006	0.967817
A_44_P113132	AA899907	AA899907	300253	0.00	-1.006	0.973199
A_44_P518221	Disp2_predicted	XM_230531		0.00	-1.006	0.984539
A_44_P741307	AA818541	AA818541		0.00	-1.006	0.975522
A_44_P1041616	Pdcd6_predicted	XM_217732		0.00	-1.006	0.96819
A_44_P308944	BX883044	BX883044		0.00	-1.006	0.959994
A_44_P594969	TC526106	TC526106		0.00	-1.006	0.977015
A_44_P513731	XM_345910	XM_345910		0.00	-1.006	0.974548
A_44_P323558	Ccnt2_predicted	XM_001054848		0.00	-1.006	0.970573
A_44_P234344	LOC685879	XM_001065610	685879	0.00	-1.006	0.975675
A_44_P232043	Gpr63_predicted	XM_232847		0.00	-1.006	0.957906
A_44_P609103	TC540919	TC540919		0.00	-1.006	0.962108
A_44_P447885	A_44_P447885	A_44_P447885		0.00	-1.006	0.975504
A_44_P411515	AI029795	AI029795	64194	0.00	-1.006	0.978011
A_44_P839581	TC527072	TC527072		0.00	-1.006	0.984164
A_42_P813840	Tapbpl_predicted	XM_232355		0.00	-1.006	0.954944
A_43_P19506	XM_219575	XM_219575		0.00	-1.006	0.961916
A_44_P187676	RGD1306493_predicted	XM_234706	314523	0.00	-1.006	0.962622
A_44_P1018447	RGD1307875_predicted	XM_221956	288515	0.00	-1.006	0.964121
A_44_P227551	Fyco1_predicted	XM_236739		0.00	-1.006	0.963278
A_43_P17608	RGD1309450_predicted	XM_001053322		0.00	-1.007	0.965893
A_44_P801516	A_44_P801516	A_44_P801516		0.00	-1.007	0.957443
A_44_P165833	Chka	NM_017127	29194	0.00	-1.007	0.975675
A_44_P132522	Ndufs8_predicted	XM_215197		0.00	-1.007	0.963312
A_44_P471440	Nbr1	NM_001024765	303554	0.00	-1.007	0.968233
A_43_P13183	Syf2	NM_133417	170933	0.00	-1.007	0.95929
A_44_P164287	Nub1	NM_001013925	296731	0.00	-1.007	0.960388
A_44_P149188	Ftl1	NM_022500	29292	0.00	-1.007	0.97454
A_44_P222256	Ddx5	NM_001007613	287765	0.00	-1.007	0.965815
A_44_P393731	Cdc42bpg_predicted	XM_219530	293693	0.00	-1.007	0.962328
A_43_P15498	Chrng	NM_019145	25753	0.00	-1.007	0.967909
A_44_P394336	AI169253	AI169253	24482	0.00	-1.008	0.970862

A_44_P346599	Bet1l	NM_019368	54400	0.00	-1.008	0.944179
A_42_P571260	Maf1	NM_001014085	315093	0.00	-1.008	0.963278
A_43_P19787	LOC679811	XM_001054490	679811	0.00	-1.008	0.958318
A_44_P123255	Api5_predicted	XM_342470	362170	0.00	-1.008	0.944185
A_44_P929197	A_44_P929197	A_44_P929197		0.00	-1.008	0.964461
A_44_P993647	RGD1565363_predicted	XM_001079962		0.00	-1.008	0.956369
A_44_P303872	RGD1310669	NM_001024987	300361	0.00	-1.008	0.947682
A_44_P783197	CF114015	CF114015		0.00	-1.008	0.954079
A_43_P23040	Pqlc2_predicted	XM_342958		0.00	-1.008	0.975005
A_44_P407239	Olr720_predicted	NM_001001067	405955	0.00	-1.008	0.967909
A_44_P151138	AW921292	AW921292		0.00	-1.008	0.976865
A_44_P491126	RGD1563216_predicted	XM_576075		0.00	-1.008	0.944185
A_44_P543784	RGD1561758_predicted	XM_226598		0.00	-1.008	0.937883
A_43_P12448	Cyb5b	NM_030586	80773	0.00	-1.008	0.959436
A_44_P407292	Olr1070_predicted	NM_001000591	366816	0.00	-1.009	0.96585
A_44_P288702	A_44_P288702	A_44_P288702		0.00	-1.009	0.983072
A_44_P554795	Rab11a	NM_031152	81830	0.00	-1.009	0.947687
A_44_P948125	RGD1565793_predicted	XM_576382	500972	0.00	-1.009	0.944823
A_44_P402027	BI281955	BI281955	24852	0.00	-1.009	0.961916
A_44_P429066	Ankrd16	NM_001033698	307102	0.00	-1.009	0.944903
A_44_P361177	XM_230279	XM_230279		0.00	-1.009	0.966265
A_44_P617381	RGD1565135_predicted	XM_001064065		0.00	-1.009	0.943912
A_44_P412749	LOC301124	XM_238570	301124	0.00	-1.009	0.942839
A_44_P409146	Arf1	NM_022518	64310	0.00	-1.009	0.932965
A_44_P137334	Tfb1m	NM_181474	308140	0.00	-1.009	0.950403
A_44_P961459	TC543011	TC543011		0.00	-1.009	0.975894
A_44_P749338	TC562198	TC562198		0.00	-1.009	0.963283
A_44_P268540	Scamp5	NM_031726	65171	0.00	-1.009	0.948484
A_44_P888303	LOC685031	XM_001062012		0.00	-1.010	0.938916
A_44_P693379	TC536464	TC536464		0.00	-1.010	0.932403
A_44_P1034849	Nfib	XM_342854		0.00	-1.010	0.939357
A_43_P15489	Il24	NM_133311	170819	0.00	-1.010	0.961759
A_44_P468837	LOC679682	XM_001053996		0.00	-1.010	0.944622
A_42_P459774	Fbxl19_predicted	XM_219356		0.00	-1.010	0.947682
A_44_P100936	Sox14_predicted	XM_236605		0.00	-1.010	0.939299
A_44_P684770	TC558217	TC558217		0.00	-1.010	0.94723
A_43_P10751	Ngrn	NM_001033900	499191	0.00	-1.010	0.922603
A_42_P492688	Zfp608_predicted	XM_225755		0.00	-1.010	0.935026
A_44_P264558	Dek	NM_001004255	306817	0.00	-1.010	0.955716
A_44_P509088	ENSRNOT00000022063	ENSRNOT00000022063		0.00	-1.010	0.958261
A_44_P1060459	Sirt5	NM_001004256	306840	0.00	-1.010	0.945394
A_42_P813939	RGD1563028_predicted	XM_001079632		0.00	-1.010	0.970032
A_44_P476830	Hspa4	NM_153629	266759	0.00	-1.010	0.959777
A_44_P375339	Ptk6_predicted	XM_345485		0.00	-1.010	0.936068
A_44_P490784	Kb39	NM_001008807	369017	0.00	-1.010	0.973615
A_44_P214914	Accn1	NM_012892	25364	0.00	-1.010	0.979069
A_44_P868060	LOC500974	XM_576384		0.00	-1.010	0.941659
A_44_P438563	Pqlc1	BC079072	361352	0.00	-1.010	0.922452
A_44_P825629	TC536184	TC536184		0.00	-1.010	0.964178
A_44_P389703	A_44_P389703	A_44_P389703		0.00	-1.010	0.970524
A_44_P194138	Egfr	NM_031507	24329	0.00	-1.010	0.943912
A_44_P380300	Nfkbil2_predicted	XM_345850	366953	0.00	-1.010	0.950013
A_44_P140676	XM_340969	XM_340969		0.00	-1.011	0.939263
A_44_P421880	Nkx2-6_predicted	XM_344429	364418	0.00	-1.011	0.941359
A_43_P21423	Pak4_predicted	XM_218388		0.00	-1.011	0.929921
A_44_P405321	RGD1561881_predicted	XM_219750		0.00	-1.011	0.938916
A_44_P149572	Rassf7_predicted	XM_215119		0.00	-1.011	0.924369
A_44_P248705	Usp25_predicted	XM_221722		0.00	-1.011	0.962623
A_44_P302417	RGD1310433_predicted	XM_238368		0.00	-1.011	0.917713
A_44_P703333	TC529719	TC529719		0.00	-1.011	0.955716
A_44_P232100	LOC684165	XM_001069225		0.00	-1.011	0.939357
A_44_P193486	AA997511	AA997511	294673	0.00	-1.011	0.963543
A_44_P169863	Thrap2_predicted	XM_001079998		0.00	-1.011	0.921788

A_44_P184614	Ndufa11	NM_212517	301123	0.00	-1.011	0.919596
A_44_P541317	Hs2st1	XM_214740	292155	0.00	-1.011	0.9605
A_44_P870987	TC530581	TC530581		0.00	-1.011	0.949354
A_44_P282097	C07140	C07140	363498	0.00	-1.011	0.93926
A_43_P15866	Ap1g1	XM_341686	171494	0.00	-1.011	0.918727
A_42_P494612	LOC682092	XM_001057820		0.00	-1.011	0.919328
A_44_P126310	Ik	NM_001005537	291659	0.00	-1.011	0.930444
A_44_P1012606	Npap60	NM_012991	25497	0.00	-1.012	0.944053
A_42_P560053	Pfkl	NM_013190	25741	-0.01	-1.012	0.941595
A_44_P822082	LOC690789	XR_006945	690789	-0.01	-1.012	0.921457
A_44_P107139	Mark3	NM_130749	170577	-0.01	-1.012	0.934372
A_44_P157915	AI030192	AI030192	293455	-0.01	-1.012	0.947306
A_44_P372795	CB771865	CB771865		-0.01	-1.012	0.939263
A_44_P201019	AY387071	AY387071		-0.01	-1.012	0.960681
A_44_P340261	RGD1305807	NM_001025276	298077	-0.01	-1.012	0.927413
A_44_P382142	XM_344342	XM_344342		-0.01	-1.012	0.951241
A_44_P333477	ENSRNOT00000028906	ENSRNOT00000028906		-0.01	-1.012	0.937916
A_44_P502486	Aggf1	XM_226709	310005	-0.01	-1.012	0.907308
A_44_P910191	BF291173	BF291173		-0.01	-1.012	0.921844
A_44_P255350	Slc25a19	NM_001007674	303676	-0.01	-1.012	0.915279
A_44_P549123	CA504564	CA504564	360853	-0.01	-1.013	0.93489
A_43_P10441	RGD1310937_predicted	XM_217149		-0.01	-1.013	0.94723
A_44_P249957	AA799591	AA799591		-0.01	-1.013	0.913217
A_44_P292586	Olr469_predicted	NM_001000299	295739	-0.01	-1.013	0.950898
A_44_P166318	A_44_P166318	A_44_P166318		-0.01	-1.013	0.905127
A_44_P253059	LOC310756	XM_227549		-0.01	-1.013	0.94562
A_44_P119633	A_44_P119633	A_44_P119633		-0.01	-1.013	0.905822
A_44_P149880	RGD1308958_predicted	XM_216388	298020	-0.01	-1.013	0.95745
A_44_P135148	Mfn2	NM_130894	64476	-0.01	-1.013	0.940765
A_44_P522836	Dusp18	NM_001013128	305477	-0.01	-1.013	0.905629
A_43_P14599	Nup37_predicted	XM_216872		-0.01	-1.013	0.954343
A_44_P480605	LOC291543	XM_001066230		-0.01	-1.013	0.911409
A_44_P326515	Chmp6_predicted	XM_237816		-0.01	-1.013	0.908157
A_44_P549962	AA893626	AA893626	363064	-0.01	-1.013	0.945022
A_44_P162738	BQ191086	BQ191086	362040	-0.01	-1.013	0.937883
A_44_P269055	Ly6b	NM_139257	246138	-0.01	-1.013	0.958439
A_44_P445724	XM_216324	XM_216324		-0.01	-1.013	0.926061
A_44_P714531	AW918102	AW918102	499755	-0.01	-1.013	0.915778
A_44_P713910	RGD1562209_predicted	XM_001064478		-0.01	-1.014	0.915297
A_44_P445588	Barx1_predicted	XM_344575		-0.01	-1.014	0.902588
A_44_P324093	RGD1561500_predicted	XM_235711	315327	-0.01	-1.014	0.908098
A_44_P211893	Rexo1	XM_576183	314630	-0.01	-1.014	0.914134
A_44_P929512	Chd8	XM_001075710		-0.01	-1.014	0.896001
A_42_P696578	Scnm1_predicted	XM_227429		-0.01	-1.014	0.893854
A_43_P12343	Stx12	NM_022939	65033	-0.01	-1.014	0.919101
A_44_P144900	Pcdhga5	NM_001037137	116782	-0.01	-1.014	0.945022
A_44_P810365	CV104092	CV104092		-0.01	-1.014	0.955716
A_44_P780510	TC527666	TC527666		-0.01	-1.014	0.953768
A_42_P692068	Tmem111	NM_001008355	312640	-0.01	-1.014	0.907366
A_42_P592144	Fath	NM_031819	83720	-0.01	-1.014	0.90489
A_44_P177465	Gas7	NM_053484	85246	-0.01	-1.014	0.959994
A_42_P521707	RT1-N3	L23128	24750	-0.01	-1.014	0.945022
A_44_P426764	A_44_P426764	A_44_P426764		-0.01	-1.014	0.902993
A_44_P241338	Gtl3	NM_001037978	307642	-0.01	-1.014	0.919551
A_44_P342730	Arih2_predicted	NM_001012275	316005	-0.01	-1.014	0.941035
A_44_P529240	Rhbg	NM_183054	310625	-0.01	-1.014	0.90157
A_44_P357470	Bmpr1a	NM_030849	81507	-0.01	-1.015	0.906586
A_44_P398433	Tnks_predicted	XM_224923		-0.01	-1.015	0.910233
A_44_P409493	Pdgfd	NM_023962	66018	-0.01	-1.015	0.934372
A_44_P454961	RGD1561065_predicted	XM_001062175		-0.01	-1.015	0.91741
A_44_P393778	LOC691130	XM_001076936		-0.01	-1.015	0.944791
A_44_P454376	MGC108747	NM_001009628	25087	-0.01	-1.015	0.930249
A_44_P389642	Nagpa_predicted	XM_340751		-0.01	-1.015	0.929454

A_43_P11871	Padi3	NM_017230	29520	-0.01	-1.015	0.952804
A_44_P388813	Olr11_predicted	NM_001000114	293070	-0.01	-1.015	0.936476
A_43_P21510	Abcb7	NM_212518	302395	-0.01	-1.015	0.911317
A_44_P145841	AA891571	AA891571	291844	-0.01	-1.015	0.936476
A_44_P300270	Lztr1_predicted	XM_341017		-0.01	-1.015	0.881299
A_44_P473020	Hdac5	AF321133	84580	-0.01	-1.015	0.926239
A_44_P187254	Znf579_predicted	XM_218200	308339	-0.01	-1.015	0.952553
A_44_P470932	Ms4a10_predicted	XM_215198		-0.01	-1.016	0.923473
A_44_P306511	Iapp	NM_012586	24476	-0.01	-1.016	0.954212
A_44_P205771	Dncic2	NM_053880	116659	-0.01	-1.016	0.879132
A_44_P699369	RGD1307597_predicted	XM_001056938		-0.01	-1.016	0.900275
A_42_P655382	Dctn3_predicted	XM_342822		-0.01	-1.016	0.888662
A_44_P480327	RGD1305685	XM_341084		-0.01	-1.016	0.936826
A_44_P501898	Chtf18_predicted	XM_213250		-0.01	-1.016	0.900344
A_44_P983152	RGD1560552_predicted	XM_575603		-0.01	-1.016	0.922343
A_44_P417092	RGD1309660_predicted	XM_226652	309953	-0.01	-1.016	0.885235
A_44_P1005770	Itgb4bp	NM_001037352	305506	-0.01	-1.017	0.892495
A_44_P203451	Slc35a4	NM_147140	257647	-0.01	-1.017	0.93767
A_44_P546372	LOC679586	XM_001053597		-0.01	-1.017	0.905177
A_44_P236820	LOC685608	XM_001065355	685608	-0.01	-1.017	0.946583
A_44_P222702	RGD1560852_predicted	XM_575638	500288	-0.01	-1.017	0.933183
A_44_P323073	AW917635	AW917635		-0.01	-1.017	0.963543
A_44_P306602	Irf1	NM_012591	24508	-0.01	-1.017	0.896001
A_44_P347270	Dtnb	NM_001012191	362715	-0.01	-1.017	0.904801
A_44_P466429	RGD1309453_predicted	XM_227479		-0.01	-1.017	0.94952
A_44_P121968	A_44_P121968	A_44_P121968		-0.01	-1.017	0.909106
A_42_P618274	Tm9sf1	NM_001012155	361043	-0.01	-1.017	0.903176
A_44_P225566	Cask	NM_022184	29647	-0.01	-1.017	0.92347
A_44_P431000	Zfp98_predicted	XM_341789		-0.01	-1.017	0.9229
A_44_P342568	XM_228999	XM_228999		-0.01	-1.017	0.896001
A_44_P832019	LOC364393	NM_001014235	364393	-0.01	-1.017	0.913217
A_44_P971665	AW916050	AW916050	315880	-0.01	-1.017	0.9215
A_43_P18039	Map3k7ip2	NM_001012062	308267	-0.01	-1.017	0.921788
A_44_P1008751	RGD1565609_predicted	XM_576214	500824	-0.01	-1.017	0.872213
A_44_P435422	LOC309957	NM_001014035	309957	-0.01	-1.017	0.939357
A_44_P233147	Ccnt2_predicted	XM_001054848		-0.01	-1.017	0.918199
A_43_P17761	LOC680614	XM_001058003		-0.01	-1.017	0.894722
A_44_P747104	TC526840	TC526840		-0.01	-1.017	0.933183
A_44_P548341	A_44_P548341	A_44_P548341		-0.01	-1.017	0.941554
A_44_P639705	TC528804	TC528804		-0.01	-1.018	0.934438
A_42_P632374	Ryr1	XM_341818	114207	-0.01	-1.018	0.956205
A_44_P436798	RGD1564914_predicted	XM_575987		-0.01	-1.018	0.87576
A_44_P304275	Jmjd1c	XM_228122	171120	-0.01	-1.018	0.870957
A_44_P166278	Vps26	NM_001007740	361846	-0.01	-1.018	0.89618
A_44_P404737	BQ208553	BQ208553	315166	-0.01	-1.018	0.891512
A_44_P853991	TC523936	TC523936		-0.01	-1.018	0.900218
A_43_P18374	Plek2_predicted	XM_234329		-0.01	-1.018	0.895925
A_43_P15513	Ltb4r2	NM_053640	114098	-0.01	-1.018	0.932403
A_44_P1017757	Rpp21	NM_001002831	406230	-0.01	-1.018	0.902588
A_44_P1056309	Snx8_predicted	XM_221947		-0.01	-1.018	0.86751
A_44_P464005	A_44_P464005	A_44_P464005		-0.01	-1.018	0.885757
A_44_P403209	Btbd9	NM_001013073	294318	-0.01	-1.018	0.936217
A_44_P261259	AF465254	AF465254		-0.01	-1.018	0.936631
A_44_P606969	TC521200	TC521200		-0.01	-1.018	0.94807
A_43_P20521	Zswim5_predicted	XM_233421		-0.01	-1.018	0.929622
A_44_P248343	XM_228406	XM_228406		-0.01	-1.018	0.896491
A_44_P744940	RGD1565640_predicted	XM_578754		-0.01	-1.018	0.926432
A_44_P304043	Dyt1	NM_153303	266606	-0.01	-1.018	0.873564
A_44_P138276	Olr1102_predicted	XM_235620		-0.01	-1.018	0.927358
A_44_P315661	Hmgcr	NM_013134	25675	-0.01	-1.018	0.908556
A_44_P518492	LOC686766	XM_001076539		-0.01	-1.018	0.90297
A_43_P17238	Rnd2	NM_001010953	303553	-0.01	-1.018	0.909778
A_44_P513568	RGD1559440_predicted	XM_231528	312166	-0.01	-1.018	0.914785

A_44_P481667	Nfe2l1_predicted	XM_340886		-0.01	-1.018	0.900216
A_42_P489764	Gjb2	NM_001004099	394266	-0.01	-1.019	0.949994
A_43_P12434	Gucy2e	NM_024380	79222	-0.01	-1.019	0.883147
A_44_P698626	ENSRNOT00000029739	ENSRNOT00000029739		-0.01	-1.019	0.933183
A_43_P12662	Alcam	NM_031753	79559	-0.01	-1.019	0.882156
A_44_P419898	Cldn9	NM_001011889	287099	-0.01	-1.019	0.943385
A_44_P190541	Rutbc1_predicted	XM_220699		-0.01	-1.019	0.854558
A_44_P1052934	Tmem97	NM_001008334	303330	-0.01	-1.019	0.891194
A_44_P259977	Arntl2	AF327071	170903	-0.01	-1.019	0.926562
A_44_P445556	LOC310579	XR_007493	310579	-0.01	-1.019	0.923854
A_44_P501234	RT1-A3	NM_001008830	24737	-0.01	-1.019	0.909016
A_43_P18072	Als2	XM_343574		-0.01	-1.019	0.86722
A_44_P351947	A_44_P351947	A_44_P351947		-0.01	-1.019	0.865294
A_44_P231719	Tmem9b_predicted	XM_215038		-0.01	-1.019	0.917512
A_44_P384086	Kcnc3	NM_053997	117101	-0.01	-1.019	0.941595
A_43_P11944	Avpr2	NM_019136	25108	-0.01	-1.019	0.922452
A_44_P1017720	RGD1308412	NM_001024980	295283	-0.01	-1.020	0.926551
A_44_P135482	A_44_P135482	A_44_P135482		-0.01	-1.020	0.897041
A_44_P668113	TC522655	TC522655		-0.01	-1.020	0.860445
A_44_P249617	AI008007	AI008007		-0.01	-1.020	0.899038
A_44_P511299	Tmem53_predicted	XM_233431		-0.01	-1.020	0.936826
A_44_P288252	LOC679812	XM_001054556	679812	-0.01	-1.020	0.919444
A_44_P428534	Adipor1	NM_207587	289036	-0.01	-1.020	0.88424
A_43_P16567	LOC684314	XM_001070374		-0.01	-1.020	0.878435
A_44_P322433	AW142643	AW142643		-0.01	-1.020	0.862495
A_44_P686682	TC568477	TC568477		-0.01	-1.020	0.856234
A_44_P165975	Ankrd27_predicted	XM_341843	361555	-0.01	-1.020	0.896001
A_44_P333374	RGD1560736_predicted	XM_001064905		-0.01	-1.020	0.933402
A_44_P852728	LOC363483	NM_001014222	363483	-0.01	-1.020	0.910031
A_43_P22750	Ralgps2	XM_222773		-0.01	-1.020	0.910239
A_44_P247997	Fancc	NM_012557	24361	-0.01	-1.020	0.926946
A_44_P177678	CB547239	CB547239	503192	-0.01	-1.020	0.87369
A_44_P564250	RGD1559880_predicted	BC099078	500584	-0.01	-1.020	0.875704
A_44_P1041034	Polr2j_predicted	XM_213753		-0.01	-1.020	0.908157
A_43_P18186	Pdzrn3_predicted	XM_232226	312607	-0.01	-1.020	0.921147
A_44_P128982	LOC619574	NM_001034959	619574	-0.01	-1.020	0.855393
A_43_P20103	Gtpbp1_predicted	XM_216982	300077	-0.01	-1.020	0.887587
A_44_P297353	XM_218274	XM_218274		-0.01	-1.020	0.909778
A_43_P12552	Tpmt	NM_031329	83497	-0.01	-1.021	0.858767
A_44_P943483	BF543478	BF543478		-0.01	-1.021	0.892992
A_44_P268919	AA818888	AA818888	64156	-0.01	-1.021	0.874557
A_44_P481629	Prkce	NM_017171	29340	-0.01	-1.021	0.881349
A_44_P984158	BI291259	BI291259	691759	-0.01	-1.021	0.867615
A_44_P365531	Hsd17b4	NM_024392	79244	-0.01	-1.021	0.868341
A_44_P420646	RGD1310606	NM_001024984	297728	-0.01	-1.021	0.874163
A_44_P317228	Brd8	NM_001008509	291691	-0.01	-1.021	0.874557
A_44_P480801	Aldoal1	NM_001013943	299052	-0.01	-1.021	0.908601
A_44_P236071	LOC317456	XM_001056948		-0.01	-1.021	0.921448
A_44_P305694	AI412156	AI412156	24233	-0.01	-1.021	0.908176
A_44_P410378	RGD1310769_predicted	XM_216756		-0.01	-1.021	0.888974
A_44_P439073	LOC679306	XM_001056215		-0.01	-1.021	0.868853
A_44_P610417	TC560558	TC560558		-0.01	-1.021	0.901134
A_44_P1019957	Ubc	NM_017314	50522	-0.01	-1.021	0.850757
A_44_P121736	RGD1564338_predicted	XM_346293		-0.01	-1.021	0.865247
A_44_P187827	Spop	XM_213437	287643	-0.01	-1.021	0.875282
A_44_P140645	Wnt6_predicted	XM_237295		-0.01	-1.021	0.91813
A_44_P346843	A_44_P346843	A_44_P346843		-0.01	-1.022	0.885193
A_44_P560458	LOC500522	XM_001068868		-0.01	-1.022	0.899089
A_42_P603096	Kcnj12	NM_053981	117052	-0.01	-1.022	0.889979
A_44_P622435	RGD1310027_predicted	XR_009495	303774	-0.01	-1.022	0.8238
A_44_P551271	Cd320	NM_001014201	362851	-0.01	-1.022	0.883621
A_44_P102570	Ptcra	XM_001065627		-0.01	-1.022	0.869999
A_44_P994512	Sympk	XM_214843	292683	-0.01	-1.022	0.912484

A_44_P318448	Egfl4	XM_341803	114029	-0.01	-1.022	0.9086
A_44_P803484	BG666368	BG666368	288774	-0.01	-1.022	0.851485
A_43_P22445	XM_342057	XM_342057		-0.01	-1.022	0.860297
A_43_P17507	LOC500015	XM_001069386		-0.01	-1.022	0.934372
A_44_P156079	AA900984	AA900984		-0.01	-1.022	0.855569
A_44_P377698	LOC303083	XR_006438	303083	-0.01	-1.022	0.85248
A_44_P667934	RGD1562029_predicted	XM_576575	501150	-0.01	-1.022	0.868745
A_43_P12018	Kcnn1	NM_019313	54261	-0.01	-1.022	0.916948
A_42_P475151	MGC116147	NM_001024864	287061	-0.01	-1.022	0.874564
A_43_P21925	Mll2_predicted	XM_001062568		-0.01	-1.022	0.825707
A_44_P520840	RGD1561176_predicted	XM_343494	363155	-0.01	-1.022	0.866343
A_44_P192543	Fkbp11	NM_001013105	300211	-0.01	-1.022	0.908694
A_44_P538054	AW143922	AW143922		-0.01	-1.022	0.95679
A_44_P226771	Mapk8ip3	XM_220232	302983	-0.01	-1.022	0.857276
A_44_P1043278	XM_213470	XM_213470		-0.01	-1.022	0.967005
A_44_P536780	Kcng2	XM_225718		-0.01	-1.022	0.893593
A_44_P489295	Kif1b	NM_057200	117548	-0.01	-1.023	0.831408
A_44_P356242	Zcchc8_predicted	XM_222159		-0.01	-1.023	0.833792
A_44_P305081	Ube2l3_predicted	XM_344046		-0.01	-1.023	0.864008
A_44_P125744	Olr996_predicted	NM_001000700	404924	-0.01	-1.023	0.912299
A_42_P785632	Tbp	NM_001004198	117526	-0.01	-1.023	0.88055
A_44_P921044	A_44_P921044	A_44_P921044		-0.01	-1.023	0.853206
A_44_P685139	LOC687611	XM_001079386		-0.01	-1.023	0.866643
A_44_P372736	Elovl3_predicted	XM_219951		-0.01	-1.023	0.943284
A_42_P829570	Rdm1_predicted	XM_220931		-0.01	-1.023	0.857997
A_43_P16961	Msl31	NM_001014111	317464	-0.01	-1.023	0.882729
A_44_P346730	Vps54	NM_173147	286932	-0.01	-1.023	0.890085
A_44_P344267	Katna1	NM_001004217	292464	-0.01	-1.023	0.842125
A_44_P368400	RGD1308665	NM_001034936	360505	-0.01	-1.023	0.911014
A_44_P541608	Pls1_predicted	XM_236560		-0.01	-1.023	0.909395
A_44_P804051	AW143981	AW143981		-0.01	-1.023	0.834212
A_42_P824657	RGD1307357_predicted	XM_214678		-0.01	-1.023	0.905195
A_44_P365240	Ptpn2	NM_053990	117063	-0.01	-1.023	0.851176
A_44_P340359	Znf183	NM_001004445	314313	-0.01	-1.023	0.811988
A_43_P19053	Pskh1_predicted	XM_344760		-0.01	-1.023	0.810644
A_44_P1056278	Samm50	NM_001004241	300111	-0.01	-1.023	0.83755
A_42_P462354	Svil_predicted	XM_341540		-0.01	-1.024	0.937123
A_44_P1058421	Phf12	XM_220644	303274	-0.01	-1.024	0.909576
A_44_P262804	LOC687681	XM_228081		-0.01	-1.024	0.82117
A_44_P138257	Tsta3_predicted	XM_216968	300036	-0.01	-1.024	0.860982
A_44_P807031	Tbc1d10b_predicted	XM_344965		-0.01	-1.024	0.821729
A_44_P173370	XM_228217	XM_228217		-0.01	-1.024	0.852344
A_44_P374610	Adra2b	NM_138505	24174	-0.01	-1.024	0.836011
A_44_P396249	LOC679596	XM_001053633		-0.01	-1.024	0.877197
A_44_P759538	AA892246	AA892246	361092	-0.01	-1.024	0.872808
A_43_P10118	AA956227	AA956227		-0.01	-1.024	0.876701
A_42_P631818	Lect1	NM_030854	81512	-0.01	-1.024	0.918992
A_44_P499851	Safb	NM_022394	64196	-0.01	-1.024	0.876861
A_44_P424108	Trim21_predicted	XM_219011		-0.01	-1.024	0.86832
A_43_P20085	Snrp70_predicted	XM_341857		-0.01	-1.024	0.846453
A_43_P13710	TC537630	TC537630		-0.01	-1.024	0.819361
A_44_P320908	AF403546	AF403546	25601	-0.01	-1.024	0.86612
A_44_P538989	RGD1308147	NM_001014010	307008	-0.01	-1.024	0.882391
A_43_P21732	Elk1	XM_001078496		-0.01	-1.024	0.844128
A_44_P695453	RGD1305441	NM_001014245	365407	-0.01	-1.024	0.853206
A_44_P286756	LOC686059	XM_574411	114207	-0.01	-1.024	0.951706
A_44_P308308	RGD1566420_predicted	XM_574226		-0.01	-1.024	0.882742
A_44_P281595	Nmnat1	NM_001037556	298653	-0.01	-1.024	0.860307
A_44_P480307	LOC367858	XM_001056262		-0.01	-1.024	0.926551
A_44_P1020541	TC539589	TC539589		-0.01	-1.025	0.856234
A_43_P13859	LOC684139	XM_001069100		-0.01	-1.025	0.908384
A_44_P577519	TC542441	TC542441		-0.01	-1.025	0.864449
A_44_P199413	Gpr132_predicted	XM_234574		-0.01	-1.025	0.915163

A_42_P701840	Pop4	NM_001009642	292831	-0.01	-1.025	0.910204
A_43_P22174	Wdr1	NM_001014135	360950	-0.01	-1.025	0.887645
A_44_P154596	RGD1563072_predicted	XM_233525		-0.01	-1.025	0.848058
A_44_P711506	BF548241	BF548241	287745	-0.01	-1.025	0.804477
A_44_P845395	AI556631	AI556631		-0.01	-1.025	0.932176
A_44_P389438	LOC679890	XM_001055091		-0.01	-1.025	0.802771
A_43_P19327	Bach1_predicted	XM_221712		-0.01	-1.025	0.863508
A_44_P232144	Prkcsh_predicted	XM_238534		-0.01	-1.025	0.833294
A_44_P359745	Bcan	NM_012916	25393	-0.01	-1.025	0.893824
A_44_P113699	Asah1	NM_053407	84431	-0.01	-1.025	0.857538
A_44_P1011726	Sharpin	NM_031153	81859	-0.01	-1.025	0.808917
A_42_P645352	RGD1309829_predicted	XM_215857	296190	-0.01	-1.026	0.868447
A_44_P462364	LOC502414	NM_001025059	502414	-0.01	-1.026	0.821911
A_44_P234547	Phf21b_predicted	XM_235545	300117	-0.01	-1.026	0.848409
A_43_P18210	RGD1306148_predicted	XM_232937	313196	-0.01	-1.026	0.826001
A_44_P510662	Sdfr1	NM_019380	56064	-0.01	-1.026	0.845355
A_44_P188157	AA925569	AA925569	294912	-0.01	-1.026	0.878428
A_44_P381011	BQ203756	BQ203756		-0.01	-1.026	0.907736
A_44_P119289	Psd4	XM_001053201	311785	-0.01	-1.026	0.843041
A_44_P264208	Ak3	NM_013218	26956	-0.01	-1.026	0.849801
A_44_P438150	Dag1	XM_343483	114489	-0.01	-1.026	0.796122
A_44_P379891	Pspc1	NM_001025672	305910	-0.01	-1.026	0.844317
A_44_P1053605	Hcn1	NM_053375	84390	-0.01	-1.026	0.86722
A_43_P13451	RGD1562438_predicted	XM_223399	305338	-0.01	-1.027	0.876799
A_44_P141377	Dbi	NM_031853	25045	-0.01	-1.027	0.877754
A_44_P792560	TC540863	TC540863		-0.01	-1.027	0.94143
A_44_P1030747	Bspry	BC062051	64027	-0.01	-1.027	0.821422
A_44_P636847	LOC500088	NM_001024326	500088	-0.01	-1.027	0.825982
A_42_P459349	RGD1311939_predicted	XM_233838		-0.01	-1.027	0.861485
A_42_P789482	Daam1_predicted	XM_001080564		-0.01	-1.027	0.831144
A_44_P253439	Cldn6_predicted	XM_220202		-0.01	-1.027	0.922235
A_44_P480285	Kif2c	NM_134472	171529	-0.01	-1.027	0.88386
A_43_P17919	LOC684560	XM_001070979		-0.01	-1.027	0.835632
A_44_P227175	RGD1306797_predicted	XM_227211		-0.01	-1.027	0.824799
A_44_P701112	TC526912	TC526912		-0.01	-1.027	0.899723
A_44_P714084	LOC688990	XM_001069068	688990	-0.01	-1.027	0.814335
A_44_P189542	Mpp2	XM_340912	85275	-0.01	-1.027	0.932965
A_44_P959674	A_44_P959674	A_44_P959674		-0.01	-1.027	0.837034
A_44_P318911	A_44_P318911	A_44_P318911		-0.01	-1.027	0.910829
A_44_P152563	AA900375	AA900375	313706	-0.01	-1.027	0.926946
A_44_P317702	BG378874	BG378874	294067	-0.01	-1.027	0.864547
A_44_P148318	AI233266	AI233266	361538	-0.01	-1.027	0.865638
A_42_P517515	RGD1307481	NM_001008327	300797	-0.01	-1.028	0.826867
A_44_P715151	TC520950	TC520950		-0.01	-1.028	0.852306
A_44_P189837	RGD1562691_predicted	XM_341468		-0.01	-1.028	0.797488
A_43_P19040	Ranbp6_predicted	XM_219796		-0.01	-1.028	0.923264
A_44_P172604	Ugcg	NM_031795	83626	-0.01	-1.028	0.822866
A_44_P929979	Gopc_predicted	XM_228173		-0.01	-1.028	0.907727
A_44_P442951	XM_344059	XM_344059		-0.01	-1.028	0.939357
A_44_P1034637	Uros	NM_001012068	309070	-0.01	-1.028	0.821185
A_42_P650709	RGD1306954	NM_001008288	288269	-0.01	-1.028	0.842317
A_42_P496411	LOC680465	XM_001057975		-0.01	-1.028	0.844789
A_44_P807034	TC519961	TC519961		-0.01	-1.028	0.893028
A_44_P236001	Olr715_predicted	NM_001000623	404815	-0.01	-1.028	0.848058
A_42_P488012	BF558262	BF558262		-0.01	-1.028	0.822842
A_44_P264777	Dnajb9	NM_012699	24908	-0.01	-1.028	0.810581
A_44_P518585	Gtpbp8	NM_001025015	360714	-0.01	-1.028	0.890085
A_43_P10970	Map3k3_predicted	XM_221034		-0.01	-1.028	0.87083
A_44_P545654	Slc1a2	NM_017215	29482	-0.01	-1.028	0.944035
A_44_P522388	Nr2c2	NM_017323	50659	-0.01	-1.028	0.833328
A_44_P554309	AI014085	AI014085	361588	-0.01	-1.028	0.838732
A_44_P426986	Zbtb39_predicted	XM_234781	299510	-0.01	-1.028	0.87834
A_44_P451782	AI169740	AI169740	83928	-0.01	-1.028	0.89336

A_44_P946035	RGD1308929_predicted	XM_341892		-0.01	-1.028	0.857781
A_44_P936248	Slc24a3	XM_001054941		-0.01	-1.028	0.879752
A_44_P104804	Tmem66	NM_001004213	290796	-0.01	-1.028	0.872695
A_44_P522439	rnf141	NM_001001800	308900	-0.01	-1.028	0.861515
A_44_P260850	CB548261	CB548261		-0.01	-1.029	0.865638
A_44_P107184	Rbbp9	NM_019219	29459	-0.01	-1.029	0.826228
A_44_P452133	Fath	NM_031819	83720	-0.01	-1.029	0.860185
A_44_P273678	Tceb3	NM_017103	25562	-0.01	-1.029	0.780267
A_43_P22040	Tbx6_predicted	XM_344963		-0.01	-1.029	0.861085
A_44_P373082	Gpr153	NM_001034855	619550	-0.01	-1.029	0.795936
A_43_P21613	Trps1_predicted	XM_001064835		-0.01	-1.029	0.809312
A_44_P430872	Rab21	NM_001004238	299799	-0.01	-1.029	0.83508
A_44_P628055	A_44_P628055	A_44_P628055		-0.01	-1.029	0.858247
A_44_P297463	RGD1311704_predicted	XM_001054503		-0.01	-1.029	0.798106
A_43_P15314	P2rx3	NM_031075	81739	-0.01	-1.029	0.949115
A_44_P139291	LOC680409	XM_001058939	680409	-0.01	-1.029	0.791369
A_44_P264837	Gtpbp6_predicted	XM_344118	363931	-0.01	-1.029	0.795368
A_44_P1034737	LOC362419	XM_001053478		-0.01	-1.030	0.844352
A_44_P547377	AI043968	AI043968	29161	-0.01	-1.030	0.86088
A_42_P637276	LOC680407	XM_001057742	680407	-0.01	-1.030	0.800221
A_42_P620197	RGD1311660_predicted	XM_213726	288518	-0.01	-1.030	0.83769
A_44_P696826	AW915383	AW915383	29254	-0.01	-1.030	0.872098
A_44_P117380	BF559538	BF559538		-0.01	-1.030	0.85806
A_44_P925168	BI395744	BI395744		-0.01	-1.030	0.902349
A_44_P405628	LOC363332	XM_343670		-0.01	-1.030	0.792189
A_44_P825005	TC532111	TC532111		-0.01	-1.030	0.863675
A_42_P590466	RGD1305833	NM_001009676	302937	-0.01	-1.030	0.779676
A_43_P16626	Metrn	NM_001009962	287151	-0.01	-1.030	0.91535
A_44_P189660	Zfp110	NM_001024775	308362	-0.01	-1.030	0.771973
A_44_P461807	Baz2b_predicted	XM_229225		-0.01	-1.030	0.865394
A_44_P293169	Mical1_predicted	XM_215424		-0.01	-1.031	0.811671
A_44_P836253	BC089066	BC089066	1E+08	-0.01	-1.031	0.795388
A_44_P540693	Slc5a3	NM_053715	114507	-0.01	-1.031	0.833119
A_44_P1046162	Zfand3	NM_001012175	361816	-0.01	-1.031	0.831991
A_44_P462018	A_44_P462018	A_44_P462018		-0.01	-1.031	0.791088
A_44_P219796	Nckap1	XM_230038		-0.01	-1.031	0.793401
A_44_P111989	AB007601	AB007601	295669	-0.01	-1.031	0.837727
A_42_P687727	Sdf4	NM_130412	155173	-0.01	-1.031	0.875553
A_44_P312381	AA899770	AA899770		-0.01	-1.031	0.866617
A_43_P17354	LOC686295	XM_001073342		-0.01	-1.031	0.824884
A_44_P150023	LOC503165	XM_001077733		-0.01	-1.031	0.794292
A_44_P294961	Exoc8	NM_139043	245709	-0.01	-1.031	0.841409
A_44_P171035	Csrp2bp_predicted	XM_342530	362224	-0.01	-1.031	0.855406
A_44_P156247	Smu1	NM_057195	117541	-0.01	-1.031	0.847081
A_44_P496519	Ccl22	NM_057203	117551	-0.01	-1.031	0.849632
A_44_P358028	LOC688261	XM_001081691		-0.01	-1.031	0.788366
A_44_P1055366	AW919175	AW919175		-0.01	-1.031	0.799903
A_44_P134186	Vps4a	NM_145678	246772	-0.01	-1.032	0.814689
A_43_P15754	Hspa9a_predicted	XM_214583		-0.01	-1.032	0.816623
A_44_P159500	RGD1307700	NM_001009714	363219	-0.01	-1.032	0.78791
A_43_P18596	LOC303057	XM_220315	303057	-0.01	-1.032	0.772861
A_44_P476120	XM_576486	XM_576486		-0.01	-1.032	0.774078
A_44_P1004260	Tmem15_predicted	XM_231121		-0.01	-1.032	0.76253
A_43_P22732	BF286666	BF286666		-0.01	-1.032	0.762844
A_43_P18992	RGD1310433_predicted	XM_238368		-0.01	-1.032	0.858094
A_44_P959815	RGD1562081_predicted	XM_580214	501590	-0.01	-1.032	0.775813
A_44_P481892	RGD1309079	XM_001068478		-0.01	-1.032	0.852955
A_44_P271416	Rabggtb	NM_138708	25533	-0.01	-1.032	0.849629
A_43_P17411	Ell2	XM_226624	309918	-0.01	-1.032	0.754601
A_44_P550511	Fbxo11	NM_181631	301674	-0.01	-1.032	0.800349
A_44_P332984	Zfp395_predicted	XM_224298		-0.01	-1.032	0.883429
A_44_P521505	AA866448	AA866448	245955	-0.01	-1.032	0.841741
A_43_P17180	mrpl9	NM_001007696	310653	-0.01	-1.032	0.807897

A_44_P1034793	Nedd4l	XM_574161	291553	-0.01	-1.032	0.887587
A_44_P1036170	Nle1_predicted	XM_220770	303372	-0.01	-1.032	0.804805
A_44_P139876	Myh8	XM_001078064		-0.01	-1.032	0.786809
A_44_P189785	Ap3b1_predicted	XM_226666		-0.01	-1.032	0.815803
A_44_P189917	XM_230775	XM_230775		-0.01	-1.032	0.773686
A_44_P522303	Il11ra1	NM_139116	245983	-0.01	-1.033	0.886814
A_44_P1042436	LOC502479	NM_001037656	502479	-0.01	-1.033	0.94369
A_44_P1027602	Unc45a	NM_001037647	308759	-0.01	-1.033	0.854881
A_44_P944575	ENSRNOT00000030256	ENSRNOT00000030256		-0.01	-1.033	0.835541
A_44_P332903	ENSRNOT00000037731	ENSRNOT00000037731		-0.01	-1.033	0.846453
A_44_P423419	AA945418	AA945418	81924	-0.01	-1.033	0.850713
A_44_P884640	TC536203	TC536203		-0.01	-1.033	0.840254
A_43_P20930	Thumpd2	NM_001012108	313851	-0.01	-1.033	0.853216
A_42_P527788	Susd2_predicted	XM_215372		-0.01	-1.033	0.859088
A_44_P494097	Sfrs15	XM_213658		-0.01	-1.033	0.76892
A_44_P454890	ENSRNOT00000043042	ENSRNOT00000043042		-0.01	-1.033	0.803535
A_44_P252549	Olr186_predicted	NM_001001031	405917	-0.01	-1.033	0.860851
A_44_P682450	BF285009	BF285009		-0.01	-1.033	0.781043
A_44_P435197	AI170772	AI170772	171082	-0.01	-1.033	0.84227
A_44_P142437	A_44_P142437	A_44_P142437		-0.01	-1.033	0.84589
A_44_P900608	TC560346	TC560346		-0.01	-1.033	0.825179
A_43_P20978	Zfp297	NM_001009172	309630	-0.01	-1.034	0.842845
A_43_P21347	Capn7	NM_001030037	306260	-0.01	-1.034	0.782346
A_43_P12530	Suox	NM_031127	81805	-0.01	-1.034	0.885463
A_44_P940897	AW915566	AW915566		-0.01	-1.034	0.827131
A_42_P456155	BG665384	BG665384	690848	-0.01	-1.034	0.74572
A_44_P451578	AI236342	AI236342	294718	-0.01	-1.034	0.780524
A_44_P429133	XM_342562	XM_342562		-0.01	-1.034	0.809822
A_44_P148464	RGD1562434_predicted	XR_009196	316087	-0.01	-1.034	0.869991
A_42_P506830	Mrpl53_predicted	XM_342712		-0.01	-1.034	0.734062
A_44_P932158	TC560901	TC560901		-0.01	-1.034	0.727921
A_44_P700914	TC560438	TC560438		-0.01	-1.034	0.72915
A_44_P340088	LOC290864	XM_214377		-0.01	-1.034	0.825075
A_43_P20636	RGD1562576_predicted	XM_220289		-0.01	-1.035	0.84984
A_44_P139522	BM986262	BM986262	79449	-0.01	-1.035	0.737027
A_43_P19917	Prkcbp1	XM_215942		-0.01	-1.035	0.752616
A_44_P264230	LOC685374	XM_001062165	685374	-0.01	-1.035	0.790975
A_44_P900655	DV729278	DV729278		-0.01	-1.035	0.882742
A_43_P15169	TC551865	TC551865		-0.01	-1.035	0.828599
A_44_P391659	LOC678801	XM_001053228		-0.01	-1.035	0.783413
A_44_P232290	Dirc2	NM_001012017	303902	-0.01	-1.035	0.837996
A_44_P853966	TC541745	TC541745		-0.01	-1.035	0.772875
A_43_P16935	Mus81	NM_001025645	293678	-0.01	-1.035	0.821896
A_43_P21652	CB546971	CB546971		-0.02	-1.035	0.829061
A_42_P625051	LOC691478	XM_001078470	691478	-0.02	-1.035	0.848564
A_43_P19174	MGC72992	NM_001009542	494345	-0.02	-1.035	0.811285
A_42_P579280	BF283858	BF283858		-0.02	-1.036	0.79069
A_44_P590280	Galnt1	BC081794	79214	-0.02	-1.036	0.817184
A_42_P489613	RGD1562657_predicted	XM_341994	361709	-0.02	-1.036	0.802117
A_44_P392797	AA892373	AA892373	83841	-0.02	-1.036	0.869999
A_44_P139659	AA851223	AA851223	25438	-0.02	-1.036	0.868843
A_44_P167788	AI137143	AI137143	288906	-0.02	-1.036	0.826001
A_44_P501669	RGD1560666_predicted	XM_228858	317439	-0.02	-1.036	0.794715
A_44_P1004617	XM_343475	XM_343475		-0.02	-1.036	0.764936
A_44_P512773	AW917238	AW917238	313491	-0.02	-1.036	0.877344
A_44_P415115	XM_345660	XM_345660		-0.02	-1.036	0.820069
A_44_P472461	BC099186	BC099186	500247	-0.02	-1.036	0.874335
A_44_P1057903	Mll5	XM_231287	311968	-0.02	-1.036	0.733529
A_42_P584734	Gdf10	NM_024375	79216	-0.02	-1.036	0.773402
A_44_P191225	AW915456	AW915456	25578	-0.02	-1.036	0.798543
A_44_P501242	Ncoa3	XM_215947	84584	-0.02	-1.037	0.767599
A_44_P478503	A_44_P478503	A_44_P478503		-0.02	-1.037	0.85806
A_44_P395907	Dlat	NM_031025	81654	-0.02	-1.037	0.769507

A_44_P243163	Chx10	XM_345704	171360	-0.02	-1.037	0.832506
A_44_P940929	AA818661	AA818661	294154	-0.02	-1.037	0.808917
A_44_P421772	XM_214961	XM_214961		-0.02	-1.037	0.819385
A_44_P724603	AW920343	AW920343		-0.02	-1.037	0.845503
A_43_P13529	RGD1562438_predicted	XM_223399	305338	-0.02	-1.037	0.793292
A_44_P414282	Abi1	NM_024397	79249	-0.02	-1.037	0.813229
A_44_P182807	RGD1564725_predicted	XM_341459		-0.02	-1.037	0.767599
A_44_P372969	A_44_P372969	A_44_P372969		-0.02	-1.037	0.846461
A_44_P273948	Hmgb1	AY321325	25459	-0.02	-1.037	0.832578
A_44_P471136	P7	NM_001010948	291942	-0.02	-1.037	0.814946
A_43_P20518	Arhgef18_predicted	XM_221775		-0.02	-1.037	0.8157
A_43_P18767	Parp2_predicted	XM_214157		-0.02	-1.038	0.717924
A_44_P747568	TC527896	TC527896		-0.02	-1.038	0.809695
A_44_P1034155	LOC686234	XM_001073018		-0.02	-1.038	0.739098
A_44_P331109	Ubn1_predicted	XM_220175		-0.02	-1.038	0.791369
A_44_P459593	XM_216272	XM_216272		-0.02	-1.038	0.796964
A_44_P393625	XM_214749	XM_214749		-0.02	-1.038	0.775855
A_42_P534782	Sirt3_predicted	XM_215124		-0.02	-1.038	0.790276
A_42_P599080	Clec4f	NM_053753	114598	-0.02	-1.038	0.858094
A_44_P358227	Mapk15	NM_173331	286997	-0.02	-1.038	0.763454
A_43_P20360	Usp1	NM_001015015	313387	-0.02	-1.038	0.773449
A_44_P506673	XM_220196	XM_220196		-0.02	-1.038	0.816076
A_43_P19003	Fpgt	NM_199494	310935	-0.02	-1.038	0.750927
A_44_P527033	Olr230_predicted	NM_001000205	293374	-0.02	-1.038	0.830752
A_44_P152144	Psmb10	NM_001025637	291983	-0.02	-1.038	0.815728
A_44_P994454	Srrm2_predicted	XM_220207	302969	-0.02	-1.038	0.789909
A_44_P149563	RGD1561579_predicted	XM_219364	309003	-0.02	-1.038	0.812388
A_44_P161296	XM_219822	XM_219822		-0.02	-1.038	0.734979
A_44_P245690	A_44_P245690	A_44_P245690		-0.02	-1.038	0.926562
A_44_P428642	Zbp1	XM_342594	171091	-0.02	-1.038	0.860868
A_44_P310168	Tgfa	NM_012671	24827	-0.02	-1.039	0.800992
A_44_P107300	Adcy6	NM_012821	25289	-0.02	-1.039	0.745039
A_44_P430298	Defb24	CB797048	641632	-0.02	-1.039	0.732902
A_44_P278010	BQ210717	BQ210717	362566	-0.02	-1.039	0.933987
A_44_P722223	A_44_P722223	A_44_P722223		-0.02	-1.039	0.718946
A_44_P243760	RGD1560842_predicted	XM_234659		-0.02	-1.039	0.885444
A_44_P139686	Zfp161	NM_172325	282825	-0.02	-1.039	0.721379
A_44_P478176	LOC686192	XM_001073285		-0.02	-1.039	0.794809
A_44_P449422	Frs2_predicted	XM_235164		-0.02	-1.039	0.716898
A_44_P1013376	Adipor2	AY724514	312670	-0.02	-1.039	0.851146
A_44_P634889	AW920687	AW920687		-0.02	-1.039	0.801756
A_44_P974849	ENSRNOT00000037382	ENSRNOT00000037382		-0.02	-1.039	0.776748
A_44_P275491	Mcfd2	NM_139253	246117	-0.02	-1.039	0.773902
A_44_P234399	LOC678928	XM_001053866		-0.02	-1.039	0.783431
A_44_P638104	TC522886	TC522886		-0.02	-1.039	0.849666
A_44_P220410	LOC317279	XR_008875	691684	-0.02	-1.039	0.737327
A_44_P621338	LOC499563	NM_001024299	499563	-0.02	-1.039	0.780252
A_42_P813975	Jmjd1a	NM_175764	312440	-0.02	-1.040	0.799436
A_44_P320858	Ghr	NM_017094	25235	-0.02	-1.040	0.823574
A_43_P16892	LOC684554	XM_001072036		-0.02	-1.040	0.812461
A_44_P206290	LOC297481	XM_216226		-0.02	-1.040	0.846704
A_42_P543374	Dffa	NM_053679	114214	-0.02	-1.040	0.807928
A_44_P838096	Rsbn1_predicted	XM_227540	310749	-0.02	-1.040	0.864008
A_42_P553918	Prx	NM_023976	78960	-0.02	-1.040	0.789239
A_42_P788749	Mta2	XM_342015	361724	-0.02	-1.040	0.793401
A_43_P21739	XM_237289	XM_237289		-0.02	-1.040	0.792162
A_44_P276080	Hsn2	NM_001002823	406234	-0.02	-1.040	0.836712
A_44_P234450	A_44_P234450	A_44_P234450		-0.02	-1.040	0.803378
A_44_P202434	AA819129	AA819129	499422	-0.02	-1.040	0.773796
A_44_P368192	XM_228096	XM_228096		-0.02	-1.040	0.826471
A_44_P145408	LOC686590	XM_001072973		-0.02	-1.040	0.716646
A_44_P347400	Pank3_predicted	XM_340785		-0.02	-1.040	0.757324
A_44_P884616	Zfp406_predicted	XM_001068250		-0.02	-1.040	0.809045

A_44_P283899	XM_344771	XM_344771		-0.02	-1.040	0.771762
A_44_P557703	A_44_P557703	A_44_P557703		-0.02	-1.040	0.80505
A_44_P577917	TC559862	TC559862		-0.02	-1.040	0.847318
A_42_P698310	Calb2	NM_053988	117059	-0.02	-1.040	0.771909
A_44_P990769	TC536478	TC536478		-0.02	-1.041	0.797407
A_44_P992412	Mcts1	XM_217587		-0.02	-1.041	0.774643
A_43_P20215	Zfp297	NM_001009172	309630	-0.02	-1.041	0.720036
A_44_P466209	Tceb3	NM_017103	25562	-0.02	-1.041	0.780879
A_44_P322608	AA892378	AA892378	288584	-0.02	-1.041	0.725182
A_44_P348941	Olr1584_predicted	NM_001000081	289247	-0.02	-1.041	0.82543
A_42_P527101	Scly	NM_001007755	363285	-0.02	-1.041	0.769053
A_44_P377560	Pou4f3_predicted	XM_344675		-0.02	-1.041	0.779048
A_44_P174970	AI172318	AI172318	314964	-0.02	-1.041	0.743113
A_44_P100732	AW918477	AW918477	292064	-0.02	-1.041	0.800349
A_44_P279032	XM_346965	XM_346965		-0.02	-1.041	0.820601
A_44_P522959	Hivep1	XM_225241		-0.02	-1.041	0.721949
A_44_P348642	RGD1310651_predicted	XM_342610	362297	-0.02	-1.041	0.838732
A_44_P282947	BM383785	BM383785	361816	-0.02	-1.041	0.74061
A_44_P116677	Dhrsx_predicted	XM_213723		-0.02	-1.041	0.784722
A_44_P227010	RGD1306181_predicted	XM_218223		-0.02	-1.041	0.790303
A_42_P510432	RGD1311605_predicted	XM_216650	298841	-0.02	-1.041	0.741817
A_44_P260174	Rcsd1_predicted	XM_341147		-0.02	-1.041	0.921815
A_44_P333853	AA924381	AA924381		-0.02	-1.041	0.776266
A_44_P731205	TC540864	TC540864		-0.02	-1.041	0.746518
A_44_P144557	LOC499660	XM_574984	499660	-0.02	-1.041	0.80429
A_44_P577582	TC543219	TC543219		-0.02	-1.042	0.90632
A_44_P351595	Atpbd3_predicted	XM_218640		-0.02	-1.042	0.797682
A_44_P214705	RGD1310313_predicted	XM_001059237		-0.02	-1.042	0.854806
A_44_P416540	AW920889	AW920889		-0.02	-1.042	0.83769
A_44_P1028878	Polb	NM_017141	29240	-0.02	-1.042	0.765473
A_44_P181927	AI171644	AI171644	25278	-0.02	-1.042	0.804177
A_44_P698919	ENSRNOT00000030288	ENSRNOT00000030288		-0.02	-1.042	0.808107
A_44_P990142	MGC94223	NM_001005533	288416	-0.02	-1.042	0.736602
A_44_P485337	Def6_predicted	XM_228031	309642	-0.02	-1.042	0.849568
A_44_P1045141	Zdhhc4	NM_001013123	304291	-0.02	-1.042	0.774905
A_44_P190003	A_44_P190003	A_44_P190003		-0.02	-1.042	0.757944
A_44_P556980	AA998065	AA998065	25415	-0.02	-1.042	0.780012
A_44_P229501	Rhoc_predicted	XM_215659		-0.02	-1.042	0.845706
A_44_P916632	TC526483	TC526483		-0.02	-1.042	0.82766
A_44_P112597	Sept8_predicted	XM_220423		-0.02	-1.043	0.737287
A_44_P147633	XM_235417	XM_235417		-0.02	-1.043	0.811049
A_44_P807338	Cog3	BC091298	361073	-0.02	-1.043	0.763494
A_44_P788597	CF110668	CF110668		-0.02	-1.043	0.744285
A_44_P154513	LOC680858	XM_001059205	680858	-0.02	-1.043	0.91056
A_44_P423490	Palmd	NM_001025688	310811	-0.02	-1.043	0.864534
A_44_P301963	Psmb9	NM_012708	24967	-0.02	-1.043	0.803061
A_42_P732437	AW916336	AW916336		-0.02	-1.043	0.815803
A_42_P492882	Nckipsd_predicted	XM_238555		-0.02	-1.043	0.783504
A_43_P22285	Dnajc4	NM_001013196	361717	-0.02	-1.043	0.699986
A_44_P186801	AA924925	AA924925	192270	-0.02	-1.043	0.770534
A_44_P607704	TC541645	TC541645		-0.02	-1.043	0.750717
A_44_P1038694	Man2a1	XM_001068826	25478	-0.02	-1.043	0.73565
A_44_P198598	Olr348_predicted	NM_001000757	405030	-0.02	-1.043	0.861085
A_44_P361504	RGD1559667_predicted	XM_573106		-0.02	-1.043	0.772875
A_44_P555910	Ccdc44_predicted	XM_001081567		-0.02	-1.044	0.703253
A_42_P807798	Hemk1_predicted	XM_217259		-0.02	-1.044	0.751685
A_44_P401858	Ris1	NM_057212	117582	-0.02	-1.044	0.879132
A_44_P947423	TC548540	TC548540		-0.02	-1.044	0.823574
A_43_P19496	RGD1310680_predicted	XM_219694		-0.02	-1.044	0.860982
A_44_P1053550	Lzts2	NM_001014247	365468	-0.02	-1.044	0.77595
A_44_P977216	TC527123	TC527123		-0.02	-1.044	0.765393
A_44_P198620	Nos3	NM_021838	24600	-0.02	-1.044	0.766563
A_43_P21174	RGD1310081_predicted	XM_001081956	310137	-0.02	-1.044	0.717924

A_42_P657494	RGD1310022	XM_214983	293058	-0.02	-1.044	0.777329
A_43_P17410	AW141874	AW141874	290577	-0.02	-1.044	0.760882
A_44_P1044984	Atg16l2_predicted	XM_218966		-0.02	-1.044	0.733799
A_43_P11203	RGD1559617_predicted	XM_213347		-0.02	-1.044	0.711626
A_44_P290895	Dpep3	NM_001008383	364994	-0.02	-1.044	0.840268
A_44_P846888	DN937505	DN937505		-0.02	-1.044	0.782951
A_42_P729012	Ceecam1	NM_001011962	296616	-0.02	-1.045	0.851782
A_44_P190617	Xkr8	NM_001012099	313033	-0.02	-1.045	0.738303
A_44_P128523	RGD1565806_predicted	XM_225053		-0.02	-1.045	0.720834
A_44_P457008	RGD1560600_predicted	XM_214099		-0.02	-1.045	0.790748
A_44_P271964	A_44_P271964	A_44_P271964		-0.02	-1.045	0.683877
A_44_P885018	Atp8b1_predicted	XM_001064261		-0.02	-1.045	0.713057
A_44_P455632	CB546843	CB546843		-0.02	-1.045	0.832711
A_42_P841516	Eif2ak1	NM_013223		-0.02	-1.045	0.747673
A_44_P1026917	C1galt1c1	NM_001030033	302499	-0.02	-1.045	0.726929
A_44_P203773	Golph4	XM_227268	310526	-0.02	-1.045	0.730055
A_44_P135688	Zfp93_predicted	XM_230880		-0.02	-1.045	0.773927
A_42_P518620	AA955618	AA955618		-0.02	-1.045	0.646614
A_43_P15773	Cdk7	XM_215467	171150	-0.02	-1.045	0.654538
A_44_P981728	BI288870	BI288870	680199	-0.02	-1.046	0.72843
A_42_P738337	Grin2c	NM_012575	24411	-0.02	-1.046	0.767229
A_42_P627241	Ebf1	NM_053820	116543	-0.02	-1.046	0.865745
A_43_P13350	Rin1	NM_139038	207119	-0.02	-1.046	0.800134
A_44_P310787	Rab22a_predicted	XM_345479		-0.02	-1.046	0.784594
A_42_P673119	BF556273	BF556273	287615	-0.02	-1.046	0.731568
A_44_P431053	Dpyd	NM_031027	81656	-0.02	-1.046	0.817624
A_43_P20341	Grlf1_predicted	XM_001053554	306400	-0.02	-1.046	0.747275
A_42_P501233	Adra1d	NM_024483	29413	-0.02	-1.046	0.829346
A_43_P13406	Arfgap1	NM_145090	246310	-0.02	-1.046	0.72672
A_44_P252900	A_44_P252900	A_44_P252900		-0.02	-1.046	0.849629
A_43_P10009	RGD1563127_predicted	XM_347035	362194	-0.02	-1.046	0.666837
A_44_P323892	RGD1559951_predicted	XM_228717		-0.02	-1.047	0.767657
A_44_P158361	Hnrpdl	NM_001033696	305178	-0.02	-1.047	0.671881
A_44_P474165	Nr1h2	NM_031626	58851	-0.02	-1.047	0.66395
A_44_P408222	CA339340	CA339340		-0.02	-1.047	0.756351
A_42_P495923	RGD1563250_predicted	XM_217094		-0.02	-1.047	0.743482
A_43_P17568	RGD1564947_predicted	XM_228781	317368	-0.02	-1.047	0.800134
A_44_P367556	Sqstm1	NM_175843		-0.02	-1.047	0.757559
A_44_P372431	XM_230113	XM_230113		-0.02	-1.047	0.702726
A_44_P306778	LOC691885	XM_001079970	691885	-0.02	-1.047	0.783088
A_44_P540205	BG379007	BG379007	294331	-0.02	-1.047	0.805002
A_44_P837980	TC538352	TC538352		-0.02	-1.047	0.791492
A_44_P562712	TC546005	TC546005		-0.02	-1.048	0.923469
A_44_P148708	DV719178	DV719178		-0.02	-1.048	0.763588
A_44_P180484	LOC295778	XR_006174	295778	-0.02	-1.048	0.765501
A_42_P597242	RGD1303142	NM_201560	296346	-0.02	-1.048	0.740797
A_44_P441328	BQ211264	BQ211264		-0.02	-1.048	0.751277
A_44_P228874	Gclc	NM_012815	25283	-0.02	-1.048	0.836018
A_44_P596864	A_44_P596864	A_44_P596864		-0.02	-1.048	0.719073
A_43_P11831	Ak3l1	NM_017135	29223	-0.02	-1.048	0.874002
A_44_P392143	Npap60	NM_012991	25497	-0.02	-1.048	0.806881
A_44_P430874	Rab21	NM_001004238	299799	-0.02	-1.048	0.768907
A_44_P335369	Mcoln2	NM_001039005	292168	-0.02	-1.048	0.832382
A_44_P636410	LOC691551	XM_001078789	691551	-0.02	-1.048	0.794056
A_44_P1008845	A_44_P1008845	A_44_P1008845		-0.02	-1.048	0.770566
A_44_P198648	Olr1323_predicted	NM_001000472	300624	-0.02	-1.048	0.851489
A_44_P463071	M27315	M27315		-0.02	-1.048	0.795548
A_43_P16469	Pcoln3_predicted	XM_344786		-0.02	-1.048	0.784619
A_43_P16031	Mapk4	XM_225726	54268	-0.02	-1.048	0.785442
A_44_P975601	LOC364393	NM_001014235	364393	-0.02	-1.048	0.783504
A_44_P944998	LOC686324	XM_001073547		-0.02	-1.049	0.650438
A_43_P21032	B4galt5_predicted	XM_342584		-0.02	-1.049	0.750934
A_44_P452171	Avp	NM_016992	24221	-0.02	-1.049	0.694986

A_44_P520557	Arhgap1_predicted	XM_230284		-0.02	-1.049	0.647377
A_44_P492189	Pgrmc2	NM_001008374	361940	-0.02	-1.049	0.645695
A_44_P479681	BF388757	BF388757	289084	-0.02	-1.049	0.805002
A_43_P19891	Rrh_predicted	XM_227718		-0.02	-1.049	0.785914
A_44_P296561	BG668164	BG668164	25676	-0.02	-1.049	0.721053
A_44_P335247	RGD1306343_predicted	XM_219680		-0.02	-1.049	0.765393
A_44_P452570	A_44_P452570	A_44_P452570		-0.02	-1.049	0.810038
A_44_P594411	Nat8	AY212271		-0.02	-1.049	0.76892
A_44_P1014005	Cdc42se1	NM_001039044	499672	-0.02	-1.049	0.707088
A_43_P13441	Sec23a_predicted	XM_347236		-0.02	-1.049	0.812239
A_44_P315480	BF551475	BF551475		-0.02	-1.049	0.765828
A_44_P296698	Lypla2	NM_031342	83510	-0.02	-1.049	0.805722
A_44_P430939	Map1lc3b	U05784	64862	-0.02	-1.049	0.637062
A_44_P435945	Dppa1_predicted	XM_221795		-0.02	-1.050	0.698266
A_44_P1050200	RGD1307059_predicted	XM_233720		-0.02	-1.050	0.763956
A_44_P112335	LOC361399	NM_001033068	361399	-0.02	-1.050	0.757595
A_44_P707011	CO393635	CO393635		-0.02	-1.050	0.842098
A_44_P500653	Nicn1	NM_001034999	619581	-0.02	-1.050	0.755894
A_44_P162911	AI227674	AI227674	287703	-0.02	-1.050	0.667566
A_44_P409866	Hbp1	NM_013221	27080	-0.02	-1.050	0.757944
A_43_P15523	Prp15	NM_012632	24687	-0.02	-1.050	0.784812
A_42_P787181	Zfp422_predicted	NM_001012745	360389	-0.02	-1.050	0.661779
A_44_P762016	DV727678	DV727678		-0.02	-1.050	0.920903
A_44_P258674	RGD1561490_predicted	XM_001071847		-0.02	-1.050	0.727118
A_44_P119624	XM_213295	XM_213295		-0.02	-1.050	0.817598
A_44_P326623	Zyg11bl	XM_242300	311842	-0.02	-1.050	0.695048
A_44_P491085	BC100146	BC100146	290831	-0.02	-1.050	0.759104
A_44_P960566	TC522030	TC522030		-0.02	-1.050	0.861856
A_44_P177448	Pafah1b1	NM_031763	83572	-0.02	-1.050	0.74715
A_44_P1053645	Zfpn1a5_predicted	XM_001053740		-0.02	-1.050	0.68895
A_44_P431039	Rab8a	NM_053998	117103	-0.02	-1.050	0.737529
A_44_P689950	CF110132	CF110132		-0.02	-1.051	0.815995
A_44_P243874	RGD1560850_predicted	XM_001078999		-0.02	-1.051	0.768371
A_44_P1009491	Cct8_predicted	XM_213673		-0.02	-1.051	0.755233
A_42_P527141	Foxm1	NM_031633	58921	-0.02	-1.051	0.773503
A_44_P766518	CO400565	CO400565		-0.02	-1.051	0.768397
A_44_P316804	Rpp25	NM_001012124	315705	-0.02	-1.051	0.874479
A_44_P517939	XM_344790	XM_344790		-0.02	-1.051	0.86722
A_44_P205883	Rab14	NM_053589	94197	-0.02	-1.051	0.687909
A_43_P12658	Kcnh1	NM_031742	65198	-0.02	-1.051	0.886796
A_44_P131230	RGD1561028	NM_001039454	315283	-0.02	-1.051	0.79272
A_44_P424024	Nmbr	NM_012799	25264	-0.02	-1.051	0.880578
A_44_P527253	Hmgcl	NM_024386	79238	-0.02	-1.051	0.674169
A_44_P839149	TC525493	TC525493		-0.02	-1.052	0.617053
A_44_P134676	Snapc3	NM_001013212	362537	-0.02	-1.052	0.756606
A_44_P629790	CF110950	CF110950		-0.02	-1.052	0.854313
A_44_P683323	RGD1559880_predicted	BC099078	500584	-0.02	-1.052	0.656084
A_44_P393953	LOC686799	XM_001075913		-0.02	-1.052	0.609614
A_43_P18160	Tacc2	NM_001004415	309025	-0.02	-1.052	0.712547
A_44_P453278	BF561497	BF561497	312320	-0.02	-1.052	0.714713
A_44_P701794	TC531145	TC531145		-0.02	-1.052	0.651007
A_42_P509967	Slc7a9	NM_053929	116726	-0.02	-1.052	0.729141
A_44_P550412	LOC499884	XM_001078325		-0.02	-1.052	0.716864
A_44_P196056	Pdlim5	NM_053326	64353	-0.02	-1.052	0.780879
A_44_P461842	XM_231999	XM_231999		-0.02	-1.052	0.884605
A_44_P516194	RGD1306364	XM_573215	303635	-0.02	-1.052	0.915192
A_44_P288117	Inhbb	XM_344130	25196	-0.02	-1.053	0.871548
A_44_P382114	ENSRNOT00000018198	ENSRNOT00000018198		-0.02	-1.053	0.671524
A_43_P15662	Tfrc	XM_001072774		-0.02	-1.053	0.784722
A_44_P902822	LOC682920	XM_001063709		-0.02	-1.053	0.637391
A_44_P623856	Dkk4_predicted	XM_001077211		-0.02	-1.053	0.744303
A_44_P843972	A_44_P843972	A_44_P843972		-0.02	-1.053	0.695018
A_44_P382512	Tmod3	NM_001011997	300838	-0.02	-1.053	0.66291

A_43_P21033	Smyd5_predicted	XM_232134		-0.02	-1.053	0.647296
A_44_P449638	Psma2	NM_017279	29669	-0.02	-1.053	0.68931
A_44_P900888	TC525562	TC525562		-0.02	-1.053	0.756115
A_44_P253161	ENSRNOT00000032201	ENSRNOT00000032201		-0.02	-1.053	0.744461
A_44_P329577	Arl2bp	NM_001024906	498910	-0.02	-1.054	0.652134
A_44_P389448	RGD1560334_predicted	XM_228900		-0.02	-1.054	0.705191
A_44_P1020631	Pex11c_predicted	XM_213688		-0.02	-1.054	0.618852
A_44_P259521	AA874885	AA874885	29426	-0.02	-1.054	0.688738
A_44_P309325	Lig4_predicted	XM_225045		-0.02	-1.054	0.778981
A_44_P329098	AI556568	AI556568		-0.02	-1.054	0.661019
A_44_P388572	RGD1309471	NM_001034829	293719	-0.02	-1.054	0.613199
A_44_P629449	BF404510	BF404510		-0.02	-1.054	0.906376
A_44_P250433	RGD1306702	NM_001025413	304325	-0.02	-1.054	0.732308
A_44_P452527	XM_227422	XM_227422		-0.02	-1.054	0.643253
A_44_P427089	XM_341008	XM_341008		-0.02	-1.054	0.691587
A_44_P278675	Zfoc1	U78135	498177	-0.02	-1.054	0.800651
A_44_P107928	XM_233509	XM_233509		-0.02	-1.054	0.780316
A_43_P21680	RGD1307325	NM_001014001	306469	-0.02	-1.054	0.729141
A_43_P16753	Man2a1	XM_001068826	25478	-0.02	-1.054	0.658751
A_44_P117412	BQ209985	BQ209985	498130	-0.02	-1.054	0.732103
A_44_P538771	Sipa1l3	NM_001013066	292771	-0.02	-1.054	0.644055
A_44_P116846	LOC361098	XM_341384	361098	-0.02	-1.055	0.738634
A_44_P1037817	XM_214428	XM_214428		-0.02	-1.055	0.665812
A_44_P715050	TC556580	TC556580		-0.02	-1.055	0.568679
A_44_P914577	BF557161	BF557161		-0.02	-1.055	0.565199
A_44_P391706	Abcb8	NM_001007796	362302	-0.02	-1.055	0.624094
A_44_P544256	Rab6ip2	NM_170788	266806	-0.02	-1.055	0.736265
A_44_P321698	RGD1305422_predicted	XM_221382	303885	-0.02	-1.055	0.622052
A_44_P468406	AABR03000021	AABR03000021		-0.02	-1.055	0.667029
A_44_P285849	Kif5c_predicted	XM_241981		-0.02	-1.055	0.627537
A_44_P456387	BE106074	BE106074	690257	-0.02	-1.055	0.739446
A_44_P515029	Map1b	XM_215469		-0.02	-1.055	0.862782
A_43_P13908	TC519842	TC519842		-0.02	-1.055	0.633389
A_44_P669097	TC523313	TC523313		-0.02	-1.055	0.745716
A_44_P132392	AI011757	AI011757	304966	-0.02	-1.055	0.712561
A_42_P752796	Me2_predicted	XM_225729		-0.02	-1.055	0.740971
A_44_P1033985	RGD1310139_predicted	XM_001063274		-0.02	-1.055	0.710234
A_44_P308530	CO561288	CO561288		-0.02	-1.055	0.71685
A_43_P20453	Mrgprg	NM_203470	309133	-0.02	-1.055	0.769245
A_44_P1015957	LOC498962	XM_574249	498962	-0.02	-1.056	0.610592
A_42_P713737	RGD1306126	XM_213267	287150	-0.02	-1.056	0.730871
A_44_P989357	Smtn	XM_001061663	289734	-0.02	-1.056	0.645031
A_44_P880370	BF405869	BF405869		-0.02	-1.056	0.764994
A_43_P17863	Inpp1	NM_001012131	316376	-0.02	-1.056	0.75054
A_44_P264299	Ckb	NM_012529	24264	-0.02	-1.056	0.765663
A_44_P606749	Mtmr3	NM_001012038	305482	-0.02	-1.056	0.616794
A_44_P490578	AI030261	AI030261		-0.02	-1.056	0.79924
A_44_P173657	Mllt6_predicted	XM_239329	303504	-0.02	-1.056	0.682678
A_44_P508603	BF543038	BF543038		-0.02	-1.056	0.731568
A_44_P381906	Slk	NM_019349	54308	-0.02	-1.056	0.769631
A_44_P462058	RGD1562547_predicted	XM_213087		-0.02	-1.056	0.614956
A_44_P808013	TC523509	TC523509		-0.02	-1.056	0.676586
A_44_P305027	RGD1564447_predicted	XM_346041		-0.02	-1.056	0.705667
A_43_P16037	PRKCQ	XM_341553	85420	-0.02	-1.056	0.811213
A_44_P452883	Sec24a_predicted	XM_213299		-0.02	-1.056	0.671268
A_44_P511184	RGD1560358_predicted	XM_342143		-0.02	-1.056	0.664065
A_44_P452163	Kcnj3	NM_031610	50599	-0.02	-1.057	0.554977
A_43_P18395	XM_219391	XM_219391		-0.02	-1.057	0.574837
A_44_P293442	XM_221276	XM_221276		-0.02	-1.057	0.715818
A_44_P1051990	AW141000	AW141000		-0.02	-1.057	0.702407
A_42_P599116	RGD1561121_predicted	XM_218447	308431	-0.02	-1.057	0.788366
A_42_P666128	Ihh	XM_576589	84399	-0.02	-1.057	0.740507
A_42_P761225	Lypd2_predicted	XM_216960	300017	-0.02	-1.057	0.642369

A_44_P977637	TC529016	TC529016		-0.02	-1.057	0.695137
A_44_P276951	RGD1564762_predicted	XM_232771	313052	-0.02	-1.057	0.629048
A_43_P20466	Sec24a_predicted	XM_213299		-0.02	-1.057	0.596518
A_44_P313904	Dars2	NM_001034143	304919	-0.02	-1.057	0.6284
A_44_P395975	Ndnl2	XM_219708	309259	-0.02	-1.057	0.712533
A_44_P532389	XM_216597	XM_216597		-0.02	-1.057	0.719802
A_42_P624403	Fdx1	NM_017126	29189	-0.02	-1.057	0.691783
A_44_P551995	Usp47_predicted	XM_218997		-0.02	-1.057	0.649118
A_44_P285517	Vps24	NM_172331	282834	-0.02	-1.057	0.674294
A_44_P527780	A_44_P527780	A_44_P527780		-0.02	-1.057	0.538216
A_44_P128711	Sh3bgrl_predicted	XM_217566	302363	-0.02	-1.057	0.646677
A_43_P21901	RGD1308147	NM_001014010	307008	-0.02	-1.057	0.639692
A_44_P184962	Olr240_predicted	XM_219206		-0.02	-1.057	0.812239
A_43_P11423	Api5_predicted	XM_342470	362170	-0.02	-1.057	0.679775
A_42_P570848	Pcsk1	NM_017091	25204	-0.02	-1.058	0.712804
A_44_P853293	Ctdp1_predicted	XM_001058378		-0.02	-1.058	0.643909
A_44_P777507	LOC682834	XM_001063327		-0.02	-1.058	0.660623
A_44_P119538	Edg6_predicted	XM_234930		-0.02	-1.058	0.608985
A_44_P380002	Psd2_predicted	XM_226019		-0.02	-1.058	0.811671
A_44_P406606	BI286685	BI286685	25489	-0.02	-1.058	0.807331
A_44_P453037	BQ194105	BQ194105	450225	-0.02	-1.058	0.803942
A_44_P1004694	XM_216945	XM_216945		-0.02	-1.058	0.640277
A_44_P570446	CR475266	CR475266		-0.02	-1.058	0.692448
A_44_P213304	A_44_P213304	A_44_P213304		-0.02	-1.058	0.671049
A_44_P373731	BF281960	BF281960		-0.02	-1.058	0.755508
A_44_P698940	LOC680133	XM_001055675	680133	-0.02	-1.058	0.727247
A_44_P123158	AA965150	AA965150	297339	-0.02	-1.058	0.730805
A_44_P114406	RGD1359592	NM_001013878	289900	-0.02	-1.058	0.640249
A_44_P227905	Terf2ip	NM_001013143	307861	-0.02	-1.058	0.726438
A_44_P377535	A_44_P377535	A_44_P377535		-0.02	-1.059	0.57569
A_44_P166464	Scyl2_predicted	XM_235050	314717	-0.02	-1.059	0.805871
A_44_P792195	TC556623	TC556623		-0.02	-1.059	0.674191
A_44_P628867	TC545828	TC545828		-0.02	-1.059	0.767185
A_42_P785075	Actr6_predicted	XM_235051		-0.02	-1.059	0.579974
A_44_P765923	TC566971	TC566971		-0.02	-1.059	0.844317
A_44_P590999	Mtac2d1	NM_001025152	500707	-0.02	-1.059	0.769233
A_43_P10710	RGD1561264_predicted	XM_233740		-0.02	-1.059	0.702726
A_44_P116591	Cx3cl1	NM_134455	89808	-0.02	-1.059	0.78733
A_44_P613835	Pura_predicted	XM_001063244		-0.02	-1.059	0.633557
A_42_P511410	Gripap1	NM_053807	116493	-0.02	-1.059	0.667793
A_43_P12277	Vax2	NM_022637	64572	-0.02	-1.059	0.641534
A_43_P16993	RGD1304823	NM_001037196	308976	-0.03	-1.059	0.702574
A_44_P606697	RGD1564490_predicted	XM_577254		-0.03	-1.059	0.713764
A_44_P389444	LOC362464	XM_342787	170903	-0.03	-1.059	0.633865
A_44_P462958	BI274429	BI274429	301382	-0.03	-1.059	0.638331
A_44_P335703	RGD1560717_predicted	XM_343508		-0.03	-1.059	0.785541
A_43_P21457	Phf17_predicted	XM_227074		-0.03	-1.059	0.60705
A_44_P343891	BU759225	BU759225		-0.03	-1.059	0.624913
A_42_P802591	Mpp5_predicted	XM_234328		-0.03	-1.060	0.714469
A_44_P591583	TC554454	TC554454		-0.03	-1.060	0.665326
A_44_P557979	Ubap1	NM_001012190	362502	-0.03	-1.060	0.638289
A_43_P18344	Ripk2	XM_342810	362491	-0.03	-1.060	0.679256
A_44_P101792	Fosl2	NM_012954	25446	-0.03	-1.060	0.767917
A_44_P494608	Gab1_predicted	XM_341667		-0.03	-1.060	0.64947
A_44_P353739	Kcne3	NM_022235	63883	-0.03	-1.060	0.697506
A_44_P656737	TC545356	TC545356		-0.03	-1.060	0.76614
A_44_P425935	BI286096	BI286096	361658	-0.03	-1.060	0.814689
A_44_P305556	Cgm4	NM_012525	24257	-0.03	-1.060	0.706534
A_44_P731013	TC543194	TC543194		-0.03	-1.060	0.665249
A_44_P295803	BF390414	BF390414	246755	-0.03	-1.060	0.723841
A_42_P800988	Ptafr	NM_053321	58949	-0.03	-1.060	0.802126
A_44_P272396	XM_237103	XM_237103		-0.03	-1.060	0.704393
A_44_P503898	Tor2a	NM_001007744	362112	-0.03	-1.060	0.661618

A_44_P792531	Fbxl12	NM_001025700	313782	-0.03	-1.060	0.571049
A_44_P156768	Sacs_predicted	XM_224256	305940	-0.03	-1.060	0.857776
A_44_P735184	DV726720	DV726720		-0.03	-1.061	0.61811
A_43_P16884	Acin1	XM_240178	305884	-0.03	-1.061	0.570816
A_44_P135277	Alcam	NM_031753	79559	-0.03	-1.061	0.589022
A_44_P189506	L21005	L21005		-0.03	-1.061	0.74797
A_42_P499767	Padi2	NM_017226	29511	-0.03	-1.061	0.825825
A_43_P22748	RGD1565367_predicted	XM_231601	312226	-0.03	-1.061	0.810822
A_44_P715347	Epb4.1l5	NM_001012023	304733	-0.03	-1.061	0.856961
A_44_P431564	Tal1_predicted	XM_233430		-0.03	-1.061	0.625818
A_44_P105634	RGD1359378	NM_001007658	300015	-0.03	-1.061	0.609437
A_44_P288058	Kns2	XM_001072327		-0.03	-1.061	0.740442
A_44_P203793	Mllt11	NM_001013912	295264	-0.03	-1.061	0.704341
A_44_P452606	Hps4_predicted	XM_222245		-0.03	-1.061	0.634752
A_44_P383899	Fut8	NM_001002289	432392	-0.03	-1.061	0.710955
A_44_P382056	Pex19	XM_225711		-0.03	-1.061	0.745245
A_44_P224853	RGD1311704_predicted	XM_345033	365462	-0.03	-1.061	0.649994
A_44_P494741	RGD1561626_predicted	XM_576503	501088	-0.03	-1.061	0.579014
A_44_P179904	Sh2d2a	NM_207605	310688	-0.03	-1.061	0.804952
A_44_P351231	Olr46_predicted	NM_001001001	405365	-0.03	-1.062	0.800134
A_44_P766535	AW917640	AW917640		-0.03	-1.062	0.731313
A_43_P20287	Gli3	XM_225411	140588	-0.03	-1.062	0.678256
A_43_P11494	Prph1	NM_012633	24688	-0.03	-1.062	0.790521
A_44_P135401	LOC292543	NM_001025639	292543	-0.03	-1.062	0.723293
A_44_P358110	Tsg101	NM_181628	292925	-0.03	-1.062	0.703409
A_44_P241391	XM_231750	XM_231750		-0.03	-1.062	0.658751
A_44_P152120	RGD1308579	NM_001014151	361351	-0.03	-1.062	0.772344
A_44_P964790	Rsbn1_predicted	XM_227540	310749	-0.03	-1.062	0.782602
A_44_P838331	CK478952	CK478952		-0.03	-1.062	0.839658
A_44_P515398	Sycn	NM_139086	245917	-0.03	-1.063	0.642254
A_44_P238829	Eaf1_predicted	XM_224618		-0.03	-1.063	0.794635
A_44_P380685	AA925170	AA925170		-0.03	-1.063	0.73337
A_44_P262569	Tollip_predicted	XM_341961		-0.03	-1.063	0.629078
A_44_P424703	AI145026	AI145026	116557	-0.03	-1.063	0.764864
A_44_P506199	XM_344992	XM_344992		-0.03	-1.063	0.650025
A_44_P194894	Osbpl2	NM_001013079	296461	-0.03	-1.063	0.617485
A_44_P365918	A_44_P365918	A_44_P365918		-0.03	-1.063	0.797644
A_44_P637541	XM_343572	XM_343572		-0.03	-1.063	0.606407
A_43_P16212	Dnahc5	XM_226891	294854	-0.03	-1.063	0.583433
A_44_P487504	LOC498178	XM_573394	498178	-0.03	-1.063	0.613466
A_44_P139730	Palm	NM_130829	170673	-0.03	-1.063	0.708558
A_44_P798337	BE116860	BE116860		-0.03	-1.063	0.729313
A_44_P991819	Krt1-19	NM_199498	360626	-0.03	-1.063	0.812943
A_42_P744047	Cga	J00757	116700	-0.03	-1.063	0.620496
A_44_P736362	A_44_P736362	A_44_P736362		-0.03	-1.063	0.710713
A_44_P917235	TC566914	TC566914		-0.03	-1.064	0.643089
A_44_P365663	TC525349	TC525349		-0.03	-1.064	0.718492
A_44_P349854	Gna13	NM_001013119	303634	-0.03	-1.064	0.686568
A_44_P760315	ENSRNOT00000042328	ENSRNOT00000042328		-0.03	-1.064	0.725182
A_44_P195511	AI555310	AI555310		-0.03	-1.064	0.691462
A_43_P19500	RGD1309065_predicted	XM_235526	315179	-0.03	-1.064	0.66161
A_43_P10072	Zbtb9	NM_213564	294289	-0.03	-1.064	0.605179
A_44_P1049872	Znf292	NM_001008879	50552	-0.03	-1.064	0.740442
A_44_P196779	RGD1311565	NM_001024971	290626	-0.03	-1.064	0.570816
A_44_P520929	C1qtnf1	NM_001007675	303701	-0.03	-1.064	0.76803
A_44_P269316	Rxrg	NM_031765	83574	-0.03	-1.064	0.659385
A_44_P445344	Ddit3	NM_024134	29467	-0.03	-1.064	0.639692
A_44_P1005725	RGD1563351_predicted	XM_238200	294350	-0.03	-1.064	0.700032
A_44_P513877	LOC678703	XM_001052976		-0.03	-1.064	0.64226
A_44_P190968	AI172411	AI172411	64317	-0.03	-1.064	0.795388
A_44_P261608	AA998009	AA998009	297893	-0.03	-1.064	0.627396
A_44_P276614	Gpr123_predicted	XM_219468		-0.03	-1.064	0.678418
A_43_P14262	RGD1565310_predicted	XM_001057346		-0.03	-1.064	0.672918

A_44_P566495	CO562769	CO562769		-0.03	-1.064	0.678794
A_44_P998800	RGD1304906_predicted	XM_214703		-0.03	-1.064	0.624931
A_44_P456747	Nfib	XM_342854		-0.03	-1.065	0.555766
A_44_P550768	TC543442	TC543442		-0.03	-1.065	0.730759
A_44_P837382	RGD1565175_predicted	XM_576511	501096	-0.03	-1.065	0.796642
A_44_P590906	Klri2	NM_001012648	503650	-0.03	-1.065	0.77061
A_43_P18105	Cd163_predicted	XM_232342		-0.03	-1.065	0.665199
A_44_P666975	AA892333	AA892333	300218	-0.03	-1.065	0.691184
A_44_P501191	Exoc6	NM_019277	50556	-0.03	-1.065	0.595171
A_44_P339110	AI059063	AI059063	246186	-0.03	-1.065	0.66197
A_44_P295626	Tbx4_predicted	XM_220811		-0.03	-1.065	0.698769
A_44_P431358	A_44_P431358	A_44_P431358		-0.03	-1.065	0.647377
A_44_P238180	LOC691318	XM_001077680	691318	-0.03	-1.065	0.590237
A_44_P643268	LOC300173	AY623035	300173	-0.03	-1.065	0.700662
A_44_P763262	TC527918	TC527918		-0.03	-1.065	0.629992
A_44_P871398	TC533462	TC533462		-0.03	-1.066	0.682461
A_43_P21961	Rabgef1_predicted	XM_341067		-0.03	-1.066	0.585544
A_42_P644209	LOC499124	XM_001078842		-0.03	-1.066	0.61057
A_44_P299136	Hace1_predicted	XM_342160		-0.03	-1.066	0.575169
A_44_P475877	ENSRNOT00000034688	ENSRNOT00000034688		-0.03	-1.066	0.802877
A_44_P534772	Ankrd12_predicted	XM_237588		-0.03	-1.066	0.668196
A_44_P262958	LOC683544	XM_001066450		-0.03	-1.066	0.802172
A_44_P167393	BE111720	BE111720	301056	-0.03	-1.066	0.69135
A_44_P403511	AA925007	AA925007		-0.03	-1.066	0.731394
A_43_P22150	LOC684297	XM_001069774		-0.03	-1.066	0.645031
A_44_P307980	Cited2	NM_053698	114490	-0.03	-1.066	0.763545
A_44_P121457	Dctn5	NM_001037778	308961	-0.03	-1.066	0.519707
A_44_P720516	A_44_P720516	A_44_P720516		-0.03	-1.066	0.750864
A_43_P13986	Gnb1	NM_030987	24400	-0.03	-1.066	0.614796
A_44_P602724	BF522056	BF522056	361838	-0.03	-1.066	0.691587
A_44_P192876	BI274340	BI274340	305291	-0.03	-1.066	0.854112
A_44_P388803	Olr769_predicted	NM_001000371	296022	-0.03	-1.066	0.794141
A_44_P327970	Olr856_predicted	NM_001000515	313546	-0.03	-1.066	0.675715
A_44_P1032908	RGD1311072	NM_001033890	296410	-0.03	-1.066	0.528796
A_42_P601850	Apxl	XM_001064752		-0.03	-1.066	0.704796
A_44_P154693	LOC300963	NM_001037772	300963	-0.03	-1.067	0.561381
A_44_P232255	RGD1565100_predicted	XM_346070	367308	-0.03	-1.067	0.713944
A_44_P654271	TC558982	TC558982		-0.03	-1.067	0.506312
A_44_P380527	Trappc1	NM_001039378	287427	-0.03	-1.067	0.578585
A_44_P853766	RGD1559716_predicted	XM_001081576		-0.03	-1.067	0.772875
A_42_P630249	Ctcf	NM_031824	83726	-0.03	-1.067	0.558568
A_44_P155159	BQ204967	BQ204967	297065	-0.03	-1.067	0.740431
A_44_P110098	RGD1311552_predicted	XM_230503		-0.03	-1.067	0.772486
A_44_P518259	Wwp2_predicted	XM_214669		-0.03	-1.067	0.5755
A_44_P142029	Nenf	NM_001002851	289380	-0.03	-1.067	0.698118
A_42_P780097	Lrrc59	NM_001008280	287633	-0.03	-1.067	0.677046
A_44_P471049	XM_342333	XM_342333		-0.03	-1.067	0.621064
A_43_P21969	Aff3_predicted	XM_343559	363220	-0.03	-1.067	0.728238
A_44_P153915	ENSRNOT00000044774	ENSRNOT00000044774		-0.03	-1.067	0.7149
A_44_P227631	A_44_P227631	A_44_P227631		-0.03	-1.067	0.69235
A_44_P356476	ENSRNOT00000041982	ENSRNOT00000041982		-0.03	-1.067	0.662072
A_44_P897247	Rab11a	NM_031152	81830	-0.03	-1.067	0.664492
A_44_P841393	TC527107	TC527107		-0.03	-1.067	0.699785
A_44_P436101	Rgnef_predicted	XM_342179		-0.03	-1.067	0.710303
A_44_P555428	Mccc2	NM_001012177	361884	-0.03	-1.067	0.659215
A_44_P314398	Dnajc13_predicted	XM_343462		-0.03	-1.067	0.697434
A_44_P295680	AI385133	AI385133	690976	-0.03	-1.067	0.686389
A_42_P518016	LOC313618	NM_001025699	313618	-0.03	-1.067	0.550006
A_44_P896040	Cybasc3	NM_001014164	361729	-0.03	-1.067	0.789992
A_44_P268427	BM986635	BM986635	24672	-0.03	-1.067	0.630228
A_44_P222710	LOC679693	XM_001054666	679693	-0.03	-1.067	0.591123
A_44_P1034990	Rnf14	XM_001066493		-0.03	-1.068	0.590752
A_44_P187732	Spag1	NM_001012116	315033	-0.03	-1.068	0.637443

A_44_P714966	TC539303	TC539303		-0.03	-1.068	0.57367
A_44_P534146	LOC360760	XM_346914	360760	-0.03	-1.068	0.661697
A_44_P479286	Rab3gap2	XM_001064454		-0.03	-1.068	0.570555
A_44_P245640	Sftpb	NM_138842	192155	-0.03	-1.068	0.696735
A_44_P853685	TC522766	TC522766		-0.03	-1.068	0.58831
A_44_P831631	A_44_P831631	A_44_P831631		-0.03	-1.068	0.692022
A_43_P13971	Sdcbp	NM_031986	83841	-0.03	-1.068	0.623887
A_44_P522847	Pik3ap1_predicted	XM_220008		-0.03	-1.068	0.773999
A_44_P1052067	Plekha4	NM_199101	308584	-0.03	-1.068	0.608533
A_43_P19445	XM_223116	XM_223116		-0.03	-1.068	0.694329
A_44_P552852	LOC679158	XM_001054955		-0.03	-1.068	0.619526
A_43_P12396	Secisbp2	NM_024002	79049	-0.03	-1.068	0.580403
A_44_P120475	Adprtl1	XM_341326	361046	-0.03	-1.068	0.755759
A_43_P15734	Sf3b1	XM_343570	84486	-0.03	-1.069	0.554426
A_44_P615345	RGD1560307_predicted	XM_573224	360661	-0.03	-1.069	0.75564
A_44_P148531	AI501931	AI501931		-0.03	-1.069	0.899235
A_43_P12184	Gpr85	NM_022254	64020	-0.03	-1.069	0.71376
A_44_P452772	LOC679584	XM_001053589	679584	-0.03	-1.069	0.624164
A_42_P635146	RGD1562501_predicted	XM_215630		-0.03	-1.069	0.578337
A_44_P274658	RGD1563417_predicted	XM_575931		-0.03	-1.069	0.503078
A_43_P18831	Syde1_predicted	XM_343173	362842	-0.03	-1.069	0.744303
A_44_P364929	BI285820	BI285820	293551	-0.03	-1.069	0.586612
A_44_P505055	AI012475	AI012475	298906	-0.03	-1.069	0.642626
A_43_P15913	Cit	NM_001029911	83620	-0.03	-1.069	0.744922
A_43_P10471	XM_341001	XM_341001		-0.03	-1.069	0.592571
A_44_P731292	TC560565	TC560565		-0.03	-1.069	0.651008
A_44_P1028477	Atf6_predicted	XM_222871		-0.03	-1.069	0.6284
A_44_P109876	Slc30a10_predicted	XM_223046		-0.03	-1.069	0.747275
A_44_P235336	Rev3l	XM_228273		-0.03	-1.069	0.735418
A_44_P416738	Fscn3	NM_001004232	296947	-0.03	-1.069	0.562379
A_44_P1046323	RGD1306404_predicted	XM_216063		-0.03	-1.069	0.590313
A_44_P637629	TC538360	TC538360		-0.03	-1.069	0.682381
A_44_P535434	Ptk9	NM_001008521	315265	-0.03	-1.069	0.666764
A_44_P157776	AA858875	AA858875	500450	-0.03	-1.070	0.775846
A_43_P13843	Msl2l1_predicted	XM_001071576		-0.03	-1.070	0.579262
A_44_P441296	LOC291545	XM_001065665		-0.03	-1.070	0.552343
A_44_P277760	BI285572	BI285572	363145	-0.03	-1.070	0.523744
A_44_P426301	U61746	U61746	171339	-0.03	-1.070	0.69146
A_44_P363377	Gapdh	NM_017008	24383	-0.03	-1.070	0.653302
A_43_P19847	Fchsd2_predicted	XM_218965		-0.03	-1.070	0.552582
A_43_P15473	Clock	NM_021856	60447	-0.03	-1.070	0.653288
A_43_P16919	Rnf2	NM_001025667	304850	-0.03	-1.070	0.572762
A_43_P12365	Vti1a	NM_023101	65277	-0.03	-1.070	0.483509
A_44_P498905	RGD1564327_predicted	XM_344634	84381	-0.03	-1.070	0.565569
A_44_P199482	XM_235669	XM_235669		-0.03	-1.071	0.636716
A_44_P1022395	Fbxl3	XM_224478	306129	-0.03	-1.071	0.75202
A_44_P1026598	Dhrsx_predicted	XM_213723		-0.03	-1.071	0.570816
A_43_P17038	Ppp1r3c	NM_001012072	309513	-0.03	-1.071	0.650981
A_44_P374776	Olr1093_predicted	NM_001001388	366844	-0.03	-1.071	0.721553
A_44_P562118	TC557975	TC557975		-0.03	-1.071	0.622031
A_44_P100009	Diap1_predicted	XM_226014		-0.03	-1.071	0.706468
A_44_P647819	AW142419	AW142419		-0.03	-1.071	0.590237
A_44_P888412	TC519772	TC519772		-0.03	-1.071	0.635343
A_44_P283971	Zfp189_predicted	XM_232972		-0.03	-1.071	0.693047
A_44_P520403	A_44_P520403	A_44_P520403		-0.03	-1.071	0.725638
A_44_P857088	TC530512	TC530512		-0.03	-1.071	0.693539
A_44_P889367	CO383501	CO383501		-0.03	-1.071	0.796964
A_44_P502527	Usp9x_predicted	XM_001056701		-0.03	-1.071	0.692933
A_44_P303507	RGD1561961_predicted	XM_213926	289181	-0.03	-1.071	0.506312
A_44_P176078	AA956496	AA956496	296741	-0.03	-1.071	0.67389
A_44_P444897	RGD1560047_predicted	XM_001053839		-0.03	-1.071	0.614132
A_44_P184907	LOC690661	XM_001075125	690661	-0.03	-1.071	0.544695
A_44_P522973	XM_229918	XM_229918		-0.03	-1.071	0.614016

A_44_P121096	Dncl2a	NM_131910	170714	-0.03	-1.071	0.55601
A_44_P575034	Hiat1_predicted	XM_215693		-0.03	-1.071	0.649344
A_44_P535993	XM_343818	XM_343818		-0.03	-1.071	0.630897
A_44_P991399	Paf1	NM_001024898	361531	-0.03	-1.072	0.592111
A_44_P189832	Myst3	XM_225008	306571	-0.03	-1.072	0.544906
A_44_P364776	BI288582	BI288582	85251	-0.03	-1.072	0.713721
A_44_P313767	Tfrc	XM_340999	64678	-0.03	-1.072	0.623285
A_42_P525317	Cacnb2	NM_053851	116600	-0.03	-1.072	0.810901
A_44_P387145	LOC259245	NM_147213	259245	-0.03	-1.072	0.761375
A_44_P420005	LOC360618	XM_340893		-0.03	-1.072	0.574101
A_44_P560207	A_44_P560207	A_44_P560207		-0.03	-1.072	0.544616
A_43_P10988	Nek7_predicted	XM_001066105		-0.03	-1.072	0.559913
A_43_P20131	Smarca3_predicted	XM_215728		-0.03	-1.072	0.594794
A_42_P708593	Per2	NM_031678	63840	-0.03	-1.072	0.84255
A_44_P324116	LOC685327	XM_001063357		-0.03	-1.072	0.752701
A_44_P729856	TC538581	TC538581		-0.03	-1.073	0.622313
A_43_P12879	Idh3a	NM_053638	114096	-0.03	-1.073	0.537415
A_42_P691927	Brp44l	NM_133561	171087	-0.03	-1.073	0.601125
A_43_P16688	Katnb1	NM_001024746	291852	-0.03	-1.073	0.535351
A_44_P903115	TC562299	TC562299		-0.03	-1.073	0.625702
A_44_P298268	Lkap	NM_133421	170946	-0.03	-1.073	0.727554
A_43_P11178	TC538790	TC538790		-0.03	-1.073	0.570359
A_44_P165986	Klk7_predicted	XM_218649		-0.03	-1.073	0.626483
A_44_P946504	TC560137	TC560137		-0.03	-1.073	0.613491
A_44_P215974	BI289684	BI289684		-0.03	-1.073	0.545979
A_42_P620762	Atf4	NM_024403	79255	-0.03	-1.073	0.630643
A_44_P438478	XM_224877	XM_224877		-0.03	-1.073	0.6301
A_44_P372439	Ppp3ca	NM_017041	24674	-0.03	-1.073	0.535371
A_44_P297057	Olr1605_predicted	NM_001000088	289923	-0.03	-1.073	0.752672
A_44_P448113	Cbx3	NM_001008313		-0.03	-1.073	0.60163
A_44_P266801	Dffb	NM_053362	84359	-0.03	-1.073	0.498671
A_44_P344415	Pfkfb2	NM_080477	24640	-0.03	-1.073	0.676585
A_44_P328998	AI070971	AI070971	312474	-0.03	-1.073	0.579512
A_44_P1033819	Gtf2h2_predicted	XM_215466		-0.03	-1.073	0.513601
A_44_P309780	Dnajb1_predicted	XM_341663		-0.03	-1.073	0.65654
A_44_P266803	Rab8a	NM_053998	117103	-0.03	-1.073	0.634592
A_44_P210852	Sh3rf2	NM_001034187	307472	-0.03	-1.073	0.624632
A_42_P512232	BF393048	BF393048	308392	-0.03	-1.073	0.695137
A_44_P280776	Bcl2	NM_016993	24224	-0.03	-1.073	0.684572
A_44_P296956	Gfm	NM_053625	114017	-0.03	-1.073	0.530323
A_44_P172489	Acsl1	NM_012820	25288	-0.03	-1.074	0.552824
A_44_P401949	Chd1_predicted	XM_001056703		-0.03	-1.074	0.615364
A_44_P609981	TC542549	TC542549		-0.03	-1.074	0.765419
A_42_P513580	LOC690214	XM_001073696	690214	-0.03	-1.074	0.622316
A_44_P389297	A_44_P389297	A_44_P389297		-0.03	-1.074	0.578448
A_44_P777899	RGD1308317_predicted	XM_001076411		-0.03	-1.074	0.802764
A_44_P222233	XM_346203	XM_346203		-0.03	-1.074	0.632043
A_43_P14688	TC556501	TC556501		-0.03	-1.074	0.574335
A_44_P367243	Ypel5	NM_001035221	298792	-0.03	-1.074	0.663898
A_44_P717261	TC532756	TC532756		-0.03	-1.074	0.5805
A_42_P788541	Pfdn5_predicted	XM_217061		-0.03	-1.074	0.496433
A_44_P576175	Hdac7a	XM_345868	84582	-0.03	-1.074	0.764994
A_44_P229387	Zfpn1a5_predicted	XM_219325		-0.03	-1.074	0.490595
A_44_P551086	A_44_P551086	A_44_P551086		-0.03	-1.074	0.660315
A_44_P931019	TC524085	TC524085		-0.03	-1.074	0.57442
A_43_P18974	LOC309016	XM_219377	309016	-0.03	-1.074	0.596746
A_44_P513814	A_44_P513814	A_44_P513814		-0.03	-1.074	0.621415
A_44_P370595	XM_345122	XM_345122		-0.03	-1.074	0.622178
A_44_P109278	BM986464	BM986464	24530	-0.03	-1.074	0.523278
A_43_P17325	RGD1305208_predicted	XM_236654	316017	-0.03	-1.074	0.555174
A_44_P384036	Olr602_predicted	NM_001000333	295829	-0.03	-1.074	0.760375
A_44_P225097	LOC362852	XM_001059703		-0.03	-1.074	0.453867
A_43_P20814	Slamf8_predicted	XM_222909		-0.03	-1.074	0.749335

A_44_P839176	Usp25_predicted	XM_001065002		-0.03	-1.074	0.832711
A_44_P530255	A_44_P530255	A_44_P530255		-0.03	-1.074	0.582115
A_44_P534651	LOC687014	XM_001076731		-0.03	-1.074	0.492503
A_44_P295590	RGD1565744_predicted	XM_340768		-0.03	-1.075	0.518455
A_44_P206190	Mnab_predicted	XM_231249		-0.03	-1.075	0.742522
A_44_P701809	TC531182	TC531182		-0.03	-1.075	0.601598
A_44_P135845	RGD1307503_predicted	XM_234543	314456	-0.03	-1.075	0.575127
A_44_P217423	Mapk9	NM_017322	50658	-0.03	-1.075	0.704393
A_44_P493802	Prkar2a	NM_019264	29699	-0.03	-1.075	0.606595
A_44_P320274	LOC678905	XM_001053746		-0.03	-1.075	0.71686
A_44_P794608	AI112975	AI112975		-0.03	-1.075	0.626537
A_44_P140455	RGD1310951_predicted	XM_232943	313202	-0.03	-1.075	0.496374
A_42_P668042	Zwint	NM_147138	257644	-0.03	-1.075	0.537358
A_44_P273888	Olr786_predicted	NM_001000916	405231	-0.03	-1.075	0.699529
A_44_P243416	Zfp509_predicted	XM_223524		-0.03	-1.075	0.555441
A_44_P125042	LOC361100	XM_001067768		-0.03	-1.076	0.576863
A_43_P11580	Abcc2	NM_012833	25303	-0.03	-1.076	0.619893
A_43_P17162	Snx13_predicted	XM_343053		-0.03	-1.076	0.623205
A_44_P521235	Fuk_predicted	XM_226508		-0.03	-1.076	0.454827
A_44_P352031	Mphosph6_predicted	XM_234352		-0.03	-1.076	0.533627
A_44_P527840	RGD1306603_predicted	XM_343481	363144	-0.03	-1.076	0.586612
A_43_P23055	Spata3_predicted	XM_343612		-0.03	-1.076	0.508535
A_44_P683566	TC550497	TC550497		-0.03	-1.076	0.575466
A_44_P321506	LOC691431	XM_001078245	691431	-0.03	-1.076	0.787068
A_42_P698972	Tapbp	NM_033098	25217	-0.03	-1.076	0.55601
A_44_P238204	Mig12	NM_206950	404280	-0.03	-1.076	0.610876
A_44_P288799	XM_346025	XM_346025		-0.03	-1.076	0.607343
A_44_P354469	BF555106	BF555106	360695	-0.03	-1.077	0.658366
A_44_P153269	BM389685	BM389685	304020	-0.03	-1.077	0.655774
A_44_P463460	Ly6g6e	NM_001001972	406866	-0.03	-1.077	0.663903
A_42_P658815	Atp6v1e1	NM_198745	297566	-0.03	-1.077	0.635761
A_44_P248322	XM_214522	XM_214522		-0.03	-1.077	0.578158
A_44_P527798	AABR03055881	AABR03055881		-0.03	-1.077	0.626034
A_44_P313844	Ogt	NM_017107	26295	-0.03	-1.077	0.578158
A_43_P14870	Nmnat3	NM_001013224	363118	-0.03	-1.077	0.545358
A_42_P626104	Fbxw11_predicted	XM_001063835		-0.03	-1.077	0.66611
A_44_P253771	BF282712	BF282712	298605	-0.03	-1.077	0.772875
A_44_P154049	Axin1	NM_024405	79257	-0.03	-1.077	0.561805
A_44_P383077	BG372857	BG372857	313048	-0.03	-1.078	0.688172
A_44_P109834	Rdh13_predicted	XM_341783		-0.03	-1.078	0.569407
A_43_P18924	Sdccag1	XM_216724		-0.03	-1.078	0.580358
A_43_P16916	Zfp282_predicted	XM_216140		-0.03	-1.078	0.76864
A_44_P325841	Dctn1	NM_024130	29167	-0.03	-1.078	0.454126
A_44_P187530	RT1-T24-1	NM_001008858	361787	-0.03	-1.078	0.831894
A_44_P243564	A_44_P243564	A_44_P243564		-0.03	-1.078	0.642085
A_44_P380727	XM_340767	XM_340767		-0.03	-1.078	0.516895
A_44_P651282	AW917248	AW917248		-0.03	-1.078	0.646796
A_44_P576209	TC520507	TC520507		-0.03	-1.078	0.504557
A_44_P380384	Dscr6_predicted	XM_213661		-0.03	-1.078	0.589847
A_44_P901573	DV726589	DV726589		-0.03	-1.079	0.71314
A_44_P806572	A_44_P806572	A_44_P806572		-0.03	-1.079	0.501851
A_44_P546431	RGD1561732_predicted	XM_343869	363554	-0.03	-1.079	0.668687
A_44_P559366	AW914472	AW914472	81515	-0.03	-1.079	0.713853
A_44_P442813	Cxadr	NM_053570	89843	-0.03	-1.079	0.723244
A_44_P989558	LOC500540	NM_001024347	500540	-0.03	-1.079	0.656372
A_44_P531891	Madd	NM_053585	94193	-0.03	-1.079	0.571999
A_44_P118878	Pcdhb7	XM_001055294		-0.03	-1.079	0.720217
A_43_P13344	Mfn1	NM_138976	192647	-0.03	-1.079	0.425902
A_44_P122206	Timm8b	NM_022541	64372	-0.03	-1.079	0.518293
A_44_P494366	RGD1560598_predicted	XM_001071732		-0.03	-1.079	0.672858
A_43_P13962	Tmem50a_predicted	XM_216545	298552	-0.03	-1.079	0.499492
A_42_P598934	LOC367314	NM_001014269	367314	-0.03	-1.080	0.56546
A_43_P20975	Zc3hdc3_predicted	XM_235433	300032	-0.03	-1.080	0.455372

A_43_P13110	Il23a	NM_130410	155140	-0.03	-1.080	0.454047
A_44_P404088	AA891666	AA891666	287645	-0.03	-1.080	0.676997
A_43_P21402	CB546753	CB546753		-0.03	-1.080	0.459681
A_44_P147123	RGD1563512_predicted	XM_001073857		-0.03	-1.080	0.607833
A_44_P194939	BF548548	BF548548		-0.03	-1.080	0.740187
A_44_P1012529	Arfgef2	NM_181083	296380	-0.03	-1.080	0.593891
A_44_P499549	Ppp2r5d	XM_001062510	363193	-0.03	-1.080	0.591262
A_44_P210632	RGD1560986_predicted	XM_218487	292786	-0.03	-1.080	0.545994
A_42_P613321	Mrpl54_predicted	XM_216854		-0.03	-1.080	0.501719
A_44_P686665	TC567391	TC567391		-0.03	-1.080	0.615722
A_44_P500295	BI274118	BI274118	29466	-0.03	-1.080	0.498194
A_43_P10596	TC529813	TC529813		-0.03	-1.081	0.675748
A_44_P130696	Opa3_predicted	XM_218416		-0.03	-1.081	0.449408
A_44_P992289	LOC681796	XM_001058464		-0.03	-1.081	0.486411
A_44_P295565	LOC689427	XM_001070764		-0.03	-1.081	0.574437
A_44_P807879	TC523049	TC523049		-0.03	-1.081	0.582641
A_44_P315714	Asl	NM_021577	59085	-0.03	-1.081	0.442474
A_44_P395821	Gbl	NM_022404	64226	-0.03	-1.081	0.575689
A_44_P529845	RGD1564276_predicted	XM_001070018		-0.03	-1.081	0.555766
A_44_P807536	TC522244	TC522244		-0.03	-1.081	0.475263
A_44_P272346	XM_345930	XM_345930		-0.03	-1.081	0.507068
A_42_P666259	Wbp11	NM_001009661	297695	-0.03	-1.081	0.594882
A_44_P521843	AI171692	AI171692	311844	-0.03	-1.081	0.494291
A_42_P783113	Ndufab1_predicted	XM_215044		-0.03	-1.081	0.625285
A_44_P159326	XM_343667	XM_343667		-0.03	-1.081	0.452774
A_44_P353583	Olr917_predicted	NM_001001354	288795	-0.03	-1.081	0.64249
A_43_P16582	XM_215142	XM_215142		-0.03	-1.082	0.603072
A_44_P286394	LOC300768	XM_217183	300768	-0.03	-1.082	0.696842
A_44_P1038400	DV727624	DV727624	497975	-0.03	-1.082	0.550093
A_44_P424087	Iqgap1_predicted	XM_341877		-0.03	-1.082	0.582641
A_44_P510659	Sdfr1	NM_019380	56064	-0.03	-1.082	0.579783
A_43_P13034	Cntn1	NM_057118	117258	-0.03	-1.082	0.539871
A_44_P480390	Sez6l2_predicted	XM_219339		-0.03	-1.082	0.602484
A_44_P447440	Stau2	NM_134466	171500	-0.03	-1.082	0.685345
A_44_P547825	Insl3	NM_053680	114215	-0.03	-1.082	0.820113
A_44_P139815	Olr282_predicted	NM_001000224	293424	-0.03	-1.082	0.66161
A_44_P758868	AW142652	AW142652	27137	-0.03	-1.082	0.513468
A_44_P173029	LOC304286	XM_239606	304286	-0.03	-1.082	0.509064
A_44_P175279	Acot8	NM_130756	170588	-0.03	-1.082	0.659609
A_44_P329644	BF403892	BF403892	303604	-0.03	-1.082	0.784272
A_44_P560285	ENSRNOT00000031474	ENSRNOT00000031474		-0.03	-1.082	0.456446
A_44_P307265	Frs3	NM_001017382	316213	-0.03	-1.082	0.485607
A_44_P536365	Nbr1	NM_001024765	303554	-0.03	-1.082	0.594481
A_44_P532284	RGD1563086_predicted	XM_346261		-0.03	-1.082	0.49321
A_44_P760235	ENSRNOT00000031148	ENSRNOT00000031148		-0.03	-1.082	0.448212
A_42_P462711	RGD1306660_predicted	XM_214304	290627	-0.03	-1.082	0.500772
A_42_P799390	Cdkn1c	NM_001033757	246060	-0.03	-1.082	0.737553
A_42_P683425	RGD1559748_predicted	XM_230758		-0.03	-1.082	0.822968
A_43_P16535	Mrps34_predicted	XM_213234		-0.03	-1.082	0.604699
A_43_P10241	CB606336	CB606336		-0.03	-1.082	0.412847
A_44_P879120	AW914513	AW914513		-0.03	-1.083	0.468053
A_44_P159584	Pcqap_predicted	XM_341015		-0.03	-1.083	0.469526
A_44_P445831	Slc39a9	NM_001034929	314275	-0.03	-1.083	0.510029
A_44_P130684	Mip1	NM_001008557	308326	-0.03	-1.083	0.670419
A_44_P563128	RGD1563798_predicted	XM_575298		-0.03	-1.083	0.68802
A_44_P166018	LOC365348	XR_009159	365348	-0.03	-1.083	0.439054
A_44_P121251	AB072251	AB072251		-0.03	-1.083	0.696661
A_43_P10254	RGD1307160_predicted	XM_219953	309451	-0.03	-1.083	0.514973
A_44_P1071020	AY387062	AY387062		-0.03	-1.083	0.707095
A_44_P450326	A_44_P450326	A_44_P450326		-0.03	-1.083	0.58626
A_44_P901368	TC528868	TC528868		-0.03	-1.083	0.424996
A_43_P16496	AI136738	AI136738	298066	-0.03	-1.083	0.476296
A_43_P18792	XM_343669	XM_343669		-0.03	-1.083	0.534627

A_44_P222282	XM_344530	XM_344530		-0.03	-1.083	0.684454
A_44_P519581	Tm2d1_predicted	XM_342866		-0.03	-1.084	0.698681
A_42_P722644	RGD1565411_predicted	XM_343330	363000	-0.03	-1.084	0.55594
A_44_P1013113	Mysm1_predicted	XM_216460	298247	-0.03	-1.084	0.551391
A_44_P669601	TC524990	TC524990		-0.03	-1.084	0.623586
A_42_P518462	Hmgn3	NM_001007020	113990	-0.03	-1.084	0.555386
A_42_P812805	Smoc2_predicted	XM_214777		-0.03	-1.084	0.817048
A_44_P767065	CF106917	CF106917		-0.03	-1.084	0.772875
A_44_P288982	Zbtb8_predicted	XM_232766		-0.03	-1.084	0.512879
A_44_P194387	RGD1565169_predicted	XM_001079912		-0.03	-1.084	0.703696
A_44_P375995	BI282744	BI282744	362282	-0.03	-1.084	0.501151
A_44_P1031109	Npdc1	NM_001004231	296562	-0.04	-1.084	0.48303
A_44_P959588	BC099085	BC099085		-0.04	-1.084	0.607181
A_43_P20754	LOC688993	XM_001069104	688993	-0.04	-1.084	0.792149
A_44_P468373	Ltbp3	XM_341997		-0.04	-1.084	0.768117
A_44_P550346	Bcl2l2	NM_021850	60434	-0.04	-1.084	0.582788
A_44_P190917	AI178191	AI178191	361309	-0.04	-1.084	0.749356
A_44_P501277	Pvrl1	XM_236210	192183	-0.04	-1.085	0.421047
A_44_P683430	LOC501180	NM_001024364	501180	-0.04	-1.085	0.650073
A_42_P570394	AW918264	AW918264		-0.04	-1.085	0.576208
A_44_P226470	AW534166	AW534166		-0.04	-1.085	0.653916
A_44_P490180	RGD1312026_predicted	XM_236266		-0.04	-1.085	0.397026
A_44_P620192	AW917285	AW917285		-0.04	-1.085	0.52799
A_44_P477777	Dpde1	XM_214325	290646	-0.04	-1.085	0.599913
A_44_P234442	Kbtbd9_predicted	XM_233955		-0.04	-1.085	0.65851
A_43_P16625	Rpa3_predicted	XM_216097		-0.04	-1.085	0.580077
A_44_P678659	AI555407	AI555407		-0.04	-1.085	0.713086
A_44_P485772	Aanat	NM_012818	25120	-0.04	-1.085	0.575871
A_42_P611911	Car15_predicted	XM_221762		-0.04	-1.085	0.624891
A_42_P505188	Wibg_predicted	XM_345769		-0.04	-1.085	0.416341
A_44_P131499	AA817908	AA817908	364879	-0.04	-1.085	0.491948
A_44_P238495	Spnb1	AJ242018		-0.04	-1.086	0.459691
A_44_P137677	Rhov	NM_138542	171581	-0.04	-1.086	0.572899
A_42_P506402	Slc25a25	NM_145677	246771	-0.04	-1.086	0.450737
A_44_P175864	Trit1_predicted	XM_342905		-0.04	-1.086	0.652134
A_44_P290836	ENSRNOT00000039637	ENSRNOT00000039637		-0.04	-1.086	0.486171
A_44_P444800	Pon2	NM_001013082	296851	-0.04	-1.086	0.543064
A_44_P182891	A_44_P182891	A_44_P182891		-0.04	-1.086	0.742968
A_43_P13569	CB546133	CB546133		-0.04	-1.086	0.45552
A_44_P475363	Arhgap17	NM_022244	63994	-0.04	-1.086	0.565834
A_44_P953481	AI138049	AI138049		-0.04	-1.086	0.565116
A_43_P21247	MGC112844	XM_342152		-0.04	-1.086	0.581246
A_44_P262899	LOC689337	XR_006618	689337	-0.04	-1.086	0.704393
A_44_P189292	Klhl17	NM_145671	246757	-0.04	-1.086	0.452774
A_44_P203682	Ptdss2_predicted	XM_215113		-0.04	-1.086	0.41289
A_44_P845195	LOC685131	XM_001062447		-0.04	-1.086	0.625979
A_44_P295548	XM_235346	XM_235346		-0.04	-1.086	0.45491
A_44_P485376	RGD1305424	NM_001024262	312727	-0.04	-1.086	0.708253
A_44_P978172	TC567950	TC567950		-0.04	-1.086	0.772875
A_44_P251944	Idi1	NM_053539	89784	-0.04	-1.086	0.576863
A_44_P142536	A_44_P142536	A_44_P142536		-0.04	-1.087	0.560519
A_42_P647072	Mapkapk2	NM_178102	289014	-0.04	-1.087	0.661214
A_44_P480636	Ddx19	NM_001005381	292022	-0.04	-1.087	0.441693
A_44_P316898	Higd1b_predicted	XM_213488		-0.04	-1.087	0.689386
A_44_P299933	LOC680089	XM_001055639	680089	-0.04	-1.087	0.557787
A_43_P21280	Centg2_predicted	XM_237381		-0.04	-1.087	0.522187
A_44_P114267	LOC304239	XM_001071596		-0.04	-1.087	0.37344
A_44_P747322	Dyx1c1	NM_001007010	363096	-0.04	-1.087	0.491574
A_44_P291537	MGC116121	NM_001024905	498830	-0.04	-1.087	0.471028
A_44_P337488	Sh3bp1	XM_235500	727679	-0.04	-1.087	0.671268
A_44_P229597	XM_230844	XM_230844		-0.04	-1.087	0.648408
A_44_P363736	Fhl3_predicted	XM_233499		-0.04	-1.087	0.795652
A_44_P234460	RGD1565338_predicted	XM_216715	299105	-0.04	-1.087	0.503019

A_42_P599810	Idh3g	NM_031551	25179	-0.04	-1.087	0.397221
A_44_P267908	BF282153	BF282153	293656	-0.04	-1.087	0.463991
A_42_P456701	Mtfmt	NM_001009697	315763	-0.04	-1.087	0.567864
A_44_P318805	Depdc5_predicted	XM_223584		-0.04	-1.087	0.42624
A_43_P17917	RGD1308916_predicted	XM_235661		-0.04	-1.087	0.619576
A_44_P994835	RGD1305455	XM_001067104		-0.04	-1.088	0.69381
A_43_P22110	Nfatc2ip	NM_001007692	308983	-0.04	-1.088	0.525261
A_43_P21709	Jmjd3_predicted	XM_343919		-0.04	-1.088	0.599956
A_44_P394016	RGD1565054_predicted	XM_234147		-0.04	-1.088	0.58784
A_44_P762631	TC560743	TC560743		-0.04	-1.088	0.64367
A_44_P441937	BF281400	BF281400	690441	-0.04	-1.088	0.609132
A_44_P829762	AW919867	AW919867		-0.04	-1.088	0.722754
A_44_P962199	CK363699	CK363699	499651	-0.04	-1.088	0.464083
A_44_P635400	Rab2b	BC092636	305853	-0.04	-1.088	0.416023
A_44_P358627	Chd2_predicted	XM_218790		-0.04	-1.088	0.490167
A_43_P13653	CB546610	CB546610		-0.04	-1.088	0.510802
A_44_P339994	LOC364223	XR_008213	364223	-0.04	-1.088	0.647346
A_44_P998964	Tnnt3	NM_031532	24838	-0.04	-1.088	0.506387
A_43_P12876	Cdc42bpb	NM_053620	113960	-0.04	-1.088	0.482144
A_44_P390979	Camk1	NM_134468	171503	-0.04	-1.089	0.568904
A_44_P461315	Son	XM_221656	304092	-0.04	-1.089	0.392197
A_44_P763629	CO402416	CO402416		-0.04	-1.089	0.627091
A_43_P14788	RGD1560047_predicted	XM_215401	294429	-0.04	-1.089	0.557416
A_44_P522080	AA894259	AA894259		-0.04	-1.089	0.570359
A_44_P357358	Atp6v0e2	NM_001002253	436582	-0.04	-1.089	0.403022
A_44_P309679	RGD1307773_predicted	XM_213235	287115	-0.04	-1.089	0.46671
A_44_P884080	XM_573260	XM_573260		-0.04	-1.089	0.55079
A_44_P345101	Bai3_predicted	XM_217367		-0.04	-1.089	0.576457
A_44_P180795	RGD1306908	NM_001034138	303037	-0.04	-1.089	0.638012
A_42_P644278	Bloc1s2	NM_001037349	293938	-0.04	-1.089	0.531068
A_44_P102607	Sec31l1	NM_033021	93646	-0.04	-1.089	0.474967
A_44_P988320	BG374488	BG374488		-0.04	-1.090	0.602168
A_44_P852282	ENSRNOT00000030924	ENSRNOT00000030924		-0.04	-1.090	0.543876
A_44_P165044	CF110171	CF110171		-0.04	-1.090	0.580534
A_44_P1003728	LOC296935	XM_001060472		-0.04	-1.090	0.647754
A_44_P468956	Daam1_predicted	XM_234275		-0.04	-1.090	0.613114
A_44_P689394	Cox8h	AI103885	25250	-0.04	-1.090	0.429202
A_44_P109573	Accn2	NM_024154	79123	-0.04	-1.090	0.551012
A_44_P539047	RGD1560953_predicted	XM_214664		-0.04	-1.090	0.41139
A_44_P483197	Arid3b_predicted	XM_345945		-0.04	-1.090	0.444508
A_44_P391871	Rtl1_predicted	XM_243381		-0.04	-1.090	0.455168
A_42_P454990	LOC312678	XM_232246		-0.04	-1.090	0.535791
A_43_P21327	Plxnc1_predicted	XM_343200		-0.04	-1.090	0.833923
A_44_P461271	Olr920_predicted	NM_001001356	288798	-0.04	-1.090	0.713315
A_44_P408232	BF283659	BF283659	497991	-0.04	-1.090	0.612311
A_44_P1011595	Phf7	NM_001012211	364510	-0.04	-1.090	0.602064
A_44_P667876	ENSRNOT00000033438	ENSRNOT00000033438		-0.04	-1.090	0.505368
A_44_P142670	XM_346308	XM_346308		-0.04	-1.090	0.474078
A_44_P272048	XM_225086	XM_225086		-0.04	-1.090	0.479907
A_44_P186185	AW142323	AW142323	24584	-0.04	-1.090	0.496719
A_42_P644929	Nxf1	NM_021579	59087	-0.04	-1.091	0.574814
A_42_P715836	Shank3	NM_021676	59312	-0.04	-1.091	0.548716
A_43_P16750	LOC680409	XM_001058939	680409	-0.04	-1.091	0.609955
A_42_P710309	DV723243	DV723243		-0.04	-1.091	0.423086
A_44_P114034	Per1	NM_001034125	287422	-0.04	-1.091	0.40065
A_44_P210735	Fndc3a_predicted	XM_224350		-0.04	-1.091	0.598345
A_44_P243701	LOC503337	XM_578871		-0.04	-1.091	0.470108
A_43_P12741	Zfp354a	NM_052798	24522	-0.04	-1.091	0.438185
A_44_P289445	Parp16	NM_001014093	315760	-0.04	-1.091	0.704948
A_43_P22638	Pappa_predicted	XM_233037		-0.04	-1.091	0.610169
A_43_P15847	Cyp27a1	NM_178847	301517	-0.04	-1.091	0.684863
A_44_P374902	Zfp384	NM_133429	171018	-0.04	-1.091	0.583751
A_42_P819964	Cox6a2	NM_012812	25278	-0.04	-1.091	0.463048

A_44_P184465	Calm3	NM_012518	24244	-0.04	-1.091	0.457167
A_44_P560038	ENSRNOT00000039062	ENSRNOT00000039062		-0.04	-1.092	0.447806
A_44_P394089	LOC686156	XM_001072678		-0.04	-1.092	0.4752
A_44_P394134	Icam5_predicted	XM_233737	313785	-0.04	-1.092	0.644655
A_44_P125823	LOC500877	AY325143	500877	-0.04	-1.092	0.464344
A_42_P536292	Ngb	NM_033359	85382	-0.04	-1.092	0.722339
A_44_P342792	RGD1311334_predicted	XM_220710	303312	-0.04	-1.092	0.45445
A_43_P16302	U17392	U17392		-0.04	-1.092	0.743314
A_44_P375502	Degs2	NM_001017457	314438	-0.04	-1.092	0.51379
A_44_P508613	Pkp3_predicted	XM_219478		-0.04	-1.092	0.613965
A_43_P18677	TC523242	TC523242		-0.04	-1.092	0.561893
A_44_P436013	LOC686118	XM_001066602		-0.04	-1.092	0.697328
A_44_P297059	Olr741_predicted	NM_001000575	366120	-0.04	-1.092	0.777537
A_44_P175584	RGD1561520_predicted	XM_345159		-0.04	-1.092	0.410425
A_42_P516561	Atn1	NM_017228	29515	-0.04	-1.092	0.417889
A_44_P367741	Inadl	AF313483		-0.04	-1.092	0.395122
A_44_P367407	AA997148	AA997148	300891	-0.04	-1.092	0.605418
A_44_P916717	TC563099	TC563099		-0.04	-1.092	0.508965
A_43_P17475	RGD1563633_predicted	XM_238366	297504	-0.04	-1.092	0.569688
A_44_P122547	Pdcd6ip	XM_001076624		-0.04	-1.093	0.533189
A_43_P10952	AABR03057257	AABR03057257		-0.04	-1.093	0.445238
A_44_P211760	AI072228	AI072228	361226	-0.04	-1.093	0.652754
A_44_P480623	RGD1563839_predicted	XM_345435	366192	-0.04	-1.093	0.513246
A_44_P1042387	Vdac3	NM_031355	83532	-0.04	-1.093	0.5131
A_43_P17055	LOC690853	XM_001075880	690853	-0.04	-1.093	0.547999
A_44_P112267	RGD1560737_predicted	XM_225445		-0.04	-1.093	0.507854
A_44_P238906	XM_345356	XM_345356		-0.04	-1.093	0.472946
A_44_P399038	BF559096	BF559096		-0.04	-1.093	0.635966
A_44_P273595	AW915828	AW915828	303514	-0.04	-1.093	0.430351
A_44_P704262	TC549054	TC549054		-0.04	-1.093	0.589707
A_44_P810132	LOC500319	NM_001040008	500319	-0.04	-1.093	0.454035
A_43_P11186	TC554654	TC554654		-0.04	-1.093	0.609591
A_44_P1049618	XM_579926	XM_579926		-0.04	-1.093	0.437783
A_43_P12767	Pdlim5	NM_053326	64353	-0.04	-1.093	0.527024
A_44_P644196	AW530337	AW530337	287954	-0.04	-1.093	0.558432
A_44_P467237	BF523472	BF523472		-0.04	-1.094	0.391725
A_44_P818552	BE120450	BE120450		-0.04	-1.094	0.647916
A_44_P532459	RGD1307882_predicted	XM_236501	315903	-0.04	-1.094	0.578585
A_44_P250144	Olr1686	NM_001001373	294152	-0.04	-1.094	0.529234
A_44_P206583	Pycr1_predicted	XM_221200		-0.04	-1.094	0.510786
A_44_P408026	XM_342979	XM_342979		-0.04	-1.094	0.419394
A_44_P1026260	Serpinb5	NM_057108	116589	-0.04	-1.094	0.439114
A_44_P380142	A_44_P380142	A_44_P380142		-0.04	-1.094	0.580095
A_44_P213524	RGD1307410	XM_340946	360673	-0.04	-1.094	0.446581
A_44_P912354	AW141160	AW141160	24525	-0.04	-1.094	0.603662
A_42_P488840	Tiprl_predicted	XM_341145	360869	-0.04	-1.094	0.370461
A_44_P471461	Gramd1c_predicted	XM_340988	360717	-0.04	-1.094	0.778981
A_44_P297805	A_44_P297805	A_44_P297805		-0.04	-1.094	0.578158
A_44_P378631	Decr2	NM_171996	64461	-0.04	-1.094	0.552214
A_44_P780874	TC565389	TC565389		-0.04	-1.094	0.557265
A_44_P869017	TC536130	TC536130		-0.04	-1.094	0.525532
A_44_P839975	TC559748	TC559748		-0.04	-1.094	0.576705
A_43_P21322	Hcls1	NM_001011898	288077	-0.04	-1.094	0.694988
A_44_P276968	Mgea6_predicted	XM_216709		-0.04	-1.094	0.57167
A_44_P415349	Gata6	NM_019185	29300	-0.04	-1.094	0.54823
A_44_P145114	Yipf4	NM_001009712	362699	-0.04	-1.094	0.528814
A_44_P319281	RGD1562212_predicted	XM_237091		-0.04	-1.095	0.419433
A_43_P19342	Mast2_predicted	XM_233782		-0.04	-1.095	0.385616
A_44_P548714	LOC365981	XM_345315		-0.04	-1.095	0.517899
A_43_P20408	Pepd_mapped	NM_001009641	292808	-0.04	-1.095	0.451277
A_43_P17985	LOC679252	XM_001054661		-0.04	-1.095	0.561805
A_44_P884171	TC538280	TC538280		-0.04	-1.095	0.461375
A_44_P484525	AA924218	AA924218	64862	-0.04	-1.095	0.589235

A_44_P459750	XM_345836	XM_345836		-0.04	-1.095	0.775855
A_43_P12687	Deaf1	NM_031801	83632	-0.04	-1.095	0.46711
A_44_P928367	RGD1306344	XM_230878		-0.04	-1.095	0.481612
A_44_P456923	Cblc	NM_001034920	292699	-0.04	-1.095	0.5174
A_44_P345136	Spag9_predicted	XM_340879		-0.04	-1.095	0.589657
A_43_P12473	Adar	NM_031006	81635	-0.04	-1.095	0.555079
A_44_P102952	A_44_P102952	A_44_P102952		-0.04	-1.095	0.650025
A_44_P384172	AF053097	AF053097		-0.04	-1.095	0.61175
A_44_P529311	Clcn1	NM_013147	25688	-0.04	-1.095	0.637247
A_44_P361380	ENSRNOT00000038715	ENSRNOT00000038715		-0.04	-1.095	0.404481
A_44_P287241	RGD1561110_predicted	XM_574832	499507	-0.04	-1.095	0.548105
A_44_P525607	Grap2	NM_001034944	366962	-0.04	-1.095	0.811671
A_44_P318259	Crkrs	NM_138916	192350	-0.04	-1.095	0.688493
A_43_P13754	RGD1559469_predicted	XM_216445		-0.04	-1.095	0.642958
A_43_P10601	Srrm2_predicted	XM_220207	302969	-0.04	-1.095	0.525088
A_44_P1027101	Xlas	NM_001024823	24896	-0.04	-1.096	0.415554
A_44_P454591	RGD1310304_predicted	XM_218006		-0.04	-1.096	0.573939
A_44_P422564	AA956758	AA956758		-0.04	-1.096	0.610537
A_42_P548410	Cte1	NM_031315	50559	-0.04	-1.096	0.557899
A_44_P713687	A_44_P713687	A_44_P713687		-0.04	-1.096	0.477928
A_43_P21046	Plxna2_predicted	XM_223080		-0.04	-1.096	0.389442
A_44_P149679	Dclre1b	NM_001025687	310745	-0.04	-1.096	0.41843
A_44_P280789	Olr475_predicted	NM_001000686	404891	-0.04	-1.096	0.6284
A_44_P144796	LOC685718	XM_001064975		-0.04	-1.096	0.440947
A_44_P777576	TC541217	TC541217		-0.04	-1.097	0.476532
A_44_P436367	B3gntl1	NM_001015035	367384	-0.04	-1.097	0.521217
A_44_P295125	ENSRNOT00000032878	ENSRNOT00000032878		-0.04	-1.097	0.462814
A_44_P496026	Eml4_predicted	XM_233839		-0.04	-1.097	0.540869
A_44_P525452	XM_228475	XM_228475		-0.04	-1.097	0.445238
A_44_P1035040	Ccrl2_predicted	XM_236658		-0.04	-1.097	0.590909
A_44_P480702	A_44_P480702	A_44_P480702		-0.04	-1.097	0.481099
A_42_P774021	Copz2_predicted	XM_340887		-0.04	-1.097	0.622052
A_44_P769167	BF391695	BF391695		-0.04	-1.097	0.593036
A_44_P334717	Klc3	NM_138520	171549	-0.04	-1.097	0.608033
A_44_P151399	Arl6ip1	XM_001078034		-0.04	-1.097	0.401487
A_44_P879588	BG057530	BG057530	300079	-0.04	-1.097	0.661346
A_44_P433942	LOC313386	XR_007801	313386	-0.04	-1.097	0.441398
A_44_P106954	Arf1	NM_022518	64310	-0.04	-1.097	0.529446
A_43_P16443	Birc6_predicted	XM_233842	313876	-0.04	-1.097	0.427489
A_44_P191299	Fbxo39	NM_001039018	303287	-0.04	-1.097	0.638012
A_43_P18467	Pbef1	NM_177928	297508	-0.04	-1.097	0.563088
A_44_P288796	Bfar	NM_001013125	304709	-0.04	-1.097	0.382683
A_44_P147426	Nt5dc1_predicted	XM_215405		-0.04	-1.097	0.599956
A_44_P549509	RGD1560367_predicted	XM_236624		-0.04	-1.097	0.408999
A_44_P229697	LOC681446	XM_001056840		-0.04	-1.098	0.53848
A_44_P424505	LOC691556	XM_001078808	691556	-0.04	-1.098	0.455384
A_44_P253087	Sh3glb2	NM_001009692	311848	-0.04	-1.098	0.459681
A_44_P485581	Xpo5_predicted	XM_343534		-0.04	-1.098	0.513468
A_43_P11782	Mbp	NM_001025291	24547	-0.04	-1.098	0.507266
A_44_P346103	AI407543	AI407543	309969	-0.04	-1.098	0.546091
A_44_P108108	Usp16	XM_213676	288306	-0.04	-1.098	0.684585
A_43_P16741	RGD1305031	NM_001025640	292640	-0.04	-1.098	0.544539
A_44_P534269	XM_223521	XM_223521		-0.04	-1.098	0.552828
A_44_P126638	Ttll6_predicted	XM_220904	287646	-0.04	-1.098	0.479538
A_44_P384895	Phf3_predicted	XM_343548		-0.04	-1.098	0.471022
A_44_P219679	Olr367_predicted	NM_001001047	405933	-0.04	-1.098	0.550062
A_42_P799113	Egfl7	NM_139104	245963	-0.04	-1.098	0.81197
A_42_P706230	Sez6	XM_239260		-0.04	-1.098	0.567135
A_44_P363849	LOC682192	XM_001059458		-0.04	-1.098	0.470909
A_44_P324508	BF414003	BF414003	304500	-0.04	-1.098	0.677542
A_44_P222761	RGD1309931_predicted	XM_342856	362538	-0.04	-1.098	0.672386
A_43_P12222	Pmpcb	NM_022395	64198	-0.04	-1.098	0.371011
A_44_P995085	Yipf3	NM_001007801	301245	-0.04	-1.099	0.376776

A_43_P15552	Ccr2	NM_021866	60463	-0.04	-1.099	0.752383
A_44_P103472	BF544619	BF544619	503192	-0.04	-1.099	0.441198
A_44_P183134	Smad6_predicted	XM_345947		-0.04	-1.099	0.632043
A_44_P289256	AI705009	AI705009	293949	-0.04	-1.099	0.458089
A_44_P973964	AA859636	AA859636	298558	-0.04	-1.099	0.392773
A_44_P236961	AA925511	AA925511	293676	-0.04	-1.099	0.47456
A_44_P157187	A_44_P157187	A_44_P157187		-0.04	-1.099	0.444631
A_44_P365879	XM_230467	XM_230467		-0.04	-1.099	0.623211
A_44_P422233	A_44_P422233	A_44_P422233		-0.04	-1.099	0.476296
A_44_P478284	RGD1310008	NM_001014156	361437	-0.04	-1.099	0.718113
A_44_P147677	LOC691956	XM_001079920	691956	-0.04	-1.100	0.543407
A_44_P159221	RGD1563001_predicted	XM_230567		-0.04	-1.100	0.665578
A_44_P545816	Ascl1	NM_022384	64186	-0.04	-1.100	0.378536
A_44_P622305	LOC683663	XM_001066976		-0.04	-1.100	0.388093
A_43_P14847	Mrpl48_predicted	XM_215009		-0.04	-1.100	0.507854
A_44_P118649	Yipf1	NM_199383	298312	-0.04	-1.100	0.557077
A_44_P523072	Oatl1_predicted	XM_217595		-0.04	-1.100	0.333379
A_44_P827872	A_44_P827872	A_44_P827872		-0.04	-1.100	0.519278
A_44_P1051068	RGD1310969_predicted	XM_230473	296084	-0.04	-1.100	0.400141
A_44_P389265	XM_232181	XM_232181		-0.04	-1.100	0.526077
A_44_P1047578	Cldn19	NM_001008514	298487	-0.04	-1.100	0.602647
A_44_P699797	Tk2_predicted	XM_001057449		-0.04	-1.100	0.417261
A_44_P532380	RGD1309944_predicted	XM_233584		-0.04	-1.100	0.354475
A_44_P182568	Fntb	NM_172034	64511	-0.04	-1.100	0.355011
A_44_P257326	Cacna2d3	NM_175595	306243	-0.04	-1.100	0.639725
A_43_P22154	Rbp3	XM_341400	24711	-0.04	-1.100	0.549662
A_44_P175057	Slc18a1	NM_013152	25693	-0.04	-1.100	0.792066
A_44_P254767	Gss	NM_012962	25458	-0.04	-1.100	0.479119
A_44_P232084	LOC301133	XM_236794		-0.04	-1.101	0.684752
A_43_P23313	MGC94604	NM_001006960	289745	-0.04	-1.101	0.612208
A_44_P288794	Bfar	NM_001013125	304709	-0.04	-1.101	0.345473
A_44_P1033692	Entpd2	NM_172030	64467	-0.04	-1.101	0.533369
A_44_P917549	A_44_P917549	A_44_P917549		-0.04	-1.101	0.507063
A_44_P1039165	Phf3_predicted	XM_343548		-0.04	-1.101	0.404063
A_44_P210486	Atp5g1	NM_017311	29754	-0.04	-1.101	0.344065
A_44_P403017	XM_226656	XM_226656		-0.04	-1.101	0.448759
A_42_P489761	Acaca	NM_022193	60581	-0.04	-1.101	0.463383
A_44_P959143	Tmem58_predicted	XM_341125		-0.04	-1.101	0.6522
A_42_P827677	Creld1	NM_001024783	312638	-0.04	-1.101	0.297427
A_44_P168972	ENSRNOT00000040415	ENSRNOT00000040415		-0.04	-1.101	0.523424
A_44_P548398	RGD1308759_predicted	XM_214306		-0.04	-1.101	0.40078
A_44_P391750	RGD1563148_predicted	XM_342769		-0.04	-1.101	0.493481
A_44_P309782	BF287291	BF287291	315134	-0.04	-1.101	0.496844
A_44_P153769	Ica1	NM_030844	81024	-0.04	-1.101	0.466213
A_43_P16236	XM_344336	XM_344336		-0.04	-1.102	0.649344
A_44_P277153	BI298443	BI298443		-0.04	-1.102	0.620804
A_44_P167259	BC087666	BC087666		-0.04	-1.102	0.678269
A_44_P251442	BQ196498	BQ196498	500797	-0.04	-1.102	0.490066
A_44_P388310	Pcyt1a	NM_078622	140544	-0.04	-1.102	0.527312
A_44_P226461	Sacm1l	NM_053798	116482	-0.04	-1.102	0.548357
A_44_P358258	Sfrs12	NM_020092	56763	-0.04	-1.102	0.336342
A_44_P1029892	Tmem14a_predicted	XM_343546		-0.04	-1.102	0.804639
A_43_P12695	Ncoa2	NM_031822	83724	-0.04	-1.102	0.376802
A_44_P1020748	RGD1309779_predicted	XM_343405		-0.04	-1.102	0.470272
A_44_P101960	BF408841	BF408841	300783	-0.04	-1.102	0.415347
A_44_P525267	XM_342322	XM_342322		-0.04	-1.102	0.579262
A_44_P456722	Pbx1_predicted	XM_222911	304947	-0.04	-1.102	0.476841
A_43_P19773	Wdr22	XM_234345	314273	-0.04	-1.102	0.526091
A_43_P13973	TC555433	TC555433		-0.04	-1.103	0.38013
A_43_P10386	CA511607	CA511607		-0.04	-1.103	0.582128
A_43_P15271	Ahcy	NM_017201	29443	-0.04	-1.103	0.4705
A_43_P16117	Pcdhga9	NM_001037158	116782	-0.04	-1.103	0.500128
A_44_P471848	XM_225257	XM_225257		-0.04	-1.103	0.551415

A_44_P1018252	Orc4l	NM_199092	295596	-0.04	-1.103	0.533557
A_44_P130226	RGD1305492_predicted	XM_215485		-0.04	-1.103	0.409346
A_44_P914747	RGD1308759_predicted	XM_001069582		-0.04	-1.103	0.323564
A_44_P255334	Six5_predicted	XM_218412		-0.04	-1.103	0.394309
A_44_P472954	Myh3	NM_012604	24583	-0.04	-1.103	0.405391
A_44_P522908	Fcho1_predicted	XM_214313		-0.04	-1.104	0.593879
A_44_P272081	Irxl1_predicted	XM_214497	291228	-0.04	-1.104	0.667697
A_44_P194663	XM_228559	XM_228559		-0.04	-1.104	0.317294
A_44_P311862	RGD1561445_predicted	XM_233277	298320	-0.04	-1.104	0.609555
A_44_P794770	Trak2	NM_133560	171086	-0.04	-1.104	0.440935
A_44_P524805	Olr1383_predicted	NM_214832	287232	-0.04	-1.104	0.354447
A_44_P797405	BQ782259	BQ782259		-0.04	-1.104	0.489409
A_44_P426369	L38931	L38931		-0.04	-1.104	0.424878
A_44_P504549	BF524978	BF524978		-0.04	-1.104	0.609846
A_44_P339818	Ralgds	NM_019250	29622	-0.04	-1.104	0.455044
A_44_P126276	Dolpp1_predicted	XM_231129		-0.04	-1.104	0.352119
A_44_P407402	Map3k2	XM_001059840		-0.04	-1.104	0.464147
A_44_P446780	AI230771	AI230771	117021	-0.04	-1.104	0.734593
A_44_P578654	LOC500088	NM_001024326	500088	-0.04	-1.104	0.563411
A_42_P702623	Zgpat	NM_001009656	296478	-0.04	-1.104	0.376962
A_43_P18315	RGD1564287_predicted	XM_342683	362360	-0.04	-1.104	0.405049
A_44_P777181	TC557926	TC557926		-0.04	-1.104	0.496692
A_43_P18815	Stag1_predicted	XM_236609		-0.04	-1.104	0.34505
A_42_P502768	LOC685841	XM_001065468	308765	-0.04	-1.104	0.3223
A_44_P148636	RGD1305050_predicted	XM_344860		-0.04	-1.104	0.611328
A_44_P1021361	Tlr3	NM_198791	364594	-0.04	-1.104	0.498003
A_43_P20305	Boc_predicted	XM_340986		-0.04	-1.104	0.585399
A_44_P152131	Fahd2a_predicted	XM_215851	296131	-0.04	-1.104	0.330689
A_44_P272393	Enpp4_predicted	XM_236955		-0.04	-1.104	0.637443
A_44_P697736	AI501106	AI501106		-0.04	-1.105	0.574428
A_42_P710324	Nkiras1_predicted	XM_223837		-0.04	-1.105	0.457188
A_44_P527089	ENSRNOT00000015089	ENSRNOT00000015089		-0.04	-1.105	0.716864
A_43_P15752	S100a1	S68809		-0.04	-1.105	0.518499
A_44_P311571	RGD1305344_predicted	XM_218634	308557	-0.04	-1.105	0.429202
A_44_P575055	LOC499094	NM_001017512	499094	-0.04	-1.105	0.312234
A_44_P494706	XM_236534	XM_236534		-0.04	-1.105	0.654496
A_44_P416685	Olr1192_predicted	NM_001000859	405152	-0.04	-1.105	0.527385
A_44_P538456	Olr36_predicted	NM_001000935	405257	-0.04	-1.105	0.39572
A_44_P789026	BF399371	BF399371		-0.04	-1.105	0.575815
A_44_P278938	Elf2	NM_001012181	361944	-0.04	-1.105	0.320478
A_44_P131654	BE102282	BE102282		-0.04	-1.105	0.586612
A_44_P406195	RGD1306108	NM_001013869	288908	-0.04	-1.105	0.447662
A_42_P703688	Plekhb1	NM_172033	64471	-0.04	-1.105	0.778239
A_44_P440633	Dlgh3	NM_031639	58948	-0.04	-1.105	0.599497
A_44_P302735	AW918794	AW918794	363445	-0.04	-1.105	0.489074
A_43_P13418	Kcnk2	NM_172041	170899	-0.04	-1.106	0.580281
A_44_P1014379	LOC315970	NM_001014097	315970	-0.04	-1.106	0.689288
A_44_P449947	RGD1564611_predicted	XM_341025	360753	-0.04	-1.106	0.413763
A_44_P1059264	Fbxl15_predicted	XM_347194		-0.04	-1.106	0.39222
A_44_P278923	Oxnad1_predicted	XM_224623		-0.04	-1.106	0.387143
A_43_P21574	Tdrd3	NM_001012043	306066	-0.04	-1.106	0.402565
A_43_P17564	Cryzl1	NM_001013044	288256	-0.04	-1.106	0.297578
A_44_P434118	Rhbdl4_predicted	XM_213408		-0.04	-1.106	0.510335
A_44_P500383	BF289376	BF289376	170944	-0.04	-1.106	0.546628
A_44_P994492	Ddah1	NM_022297	64157	-0.04	-1.106	0.550141
A_44_P489569	Ka11	NM_001008750	450226	-0.04	-1.106	0.753621
A_44_P487375	RGD1312014_predicted	XM_222897		-0.04	-1.106	0.390179
A_43_P22266	RGD1561161_predicted	XM_226771	294747	-0.04	-1.106	0.695074
A_42_P788428	XM_237316	XM_237316		-0.04	-1.106	0.562489
A_44_P149650	XM_224716	XM_224716		-0.04	-1.107	0.345285
A_42_P629531	AW916609	AW916609		-0.04	-1.107	0.408304
A_44_P227382	RGD1564004_predicted	XM_234144		-0.04	-1.107	0.460597
A_44_P264211	Ak3	NM_013218	26956	-0.04	-1.107	0.47388

A_44_P809836	TC550500	TC550500		-0.04	-1.107	0.627396
A_44_P349309	Grsf1	XM_223327	305256	-0.04	-1.107	0.48046
A_44_P445530	Ogdhl_predicted	XM_214261		-0.04	-1.107	0.420164
A_44_P243134	Mtap2	NM_013066	25595	-0.04	-1.107	0.636926
A_44_P170721	Cyb5r3	NM_138877	25035	-0.04	-1.107	0.505413
A_44_P1040941	Apeh	NM_012500	24206	-0.04	-1.107	0.354555
A_44_P235748	AA892795	AA892795	81816	-0.04	-1.107	0.495946
A_44_P124732	BF416433	BF416433	499418	-0.04	-1.107	0.671029
A_44_P1006954	AW914881	AW914881		-0.04	-1.107	0.480401
A_44_P185449	Cnnm3_predicted	XM_237093		-0.04	-1.107	0.578315
A_44_P928497	LOC499510	NM_001024297	499510	-0.04	-1.107	0.461053
A_44_P823606	DY471809	DY471809		-0.04	-1.108	0.652514
A_44_P722574	A_44_P722574	A_44_P722574		-0.04	-1.108	0.513626
A_42_P625147	Pglyrp1	NM_053373	84387	-0.04	-1.108	0.632115
A_44_P725248	DV725280	DV725280		-0.04	-1.108	0.653688
A_43_P13247	Psd	XM_001066749		-0.04	-1.108	0.51913
A_44_P696462	AW143313	AW143313		-0.04	-1.108	0.718218
A_44_P126164	RGD1561154_predicted	XR_007874	294046	-0.04	-1.108	0.556711
A_44_P776786	TC556118	TC556118		-0.04	-1.108	0.515116
A_44_P238878	RGD1305087_predicted	XM_345293	365946	-0.04	-1.108	0.680718
A_42_P675672	LOC292069	NM_001037094	292069	-0.04	-1.108	0.483865
A_43_P11068	LOC680039	XM_001054238		-0.04	-1.108	0.480531
A_44_P347325	Tspan3	NM_001005547	300733	-0.04	-1.108	0.595362
A_44_P602419	BE110143	BE110143		-0.04	-1.109	0.579262
A_44_P260433	RGD1306101_predicted	XM_214698	292051	-0.04	-1.109	0.306661
A_44_P544070	XM_576618	XM_576618		-0.04	-1.109	0.452341
A_44_P770440	CK469681	CK469681		-0.04	-1.109	0.354866
A_44_P453217	BF542832	BF542832		-0.04	-1.109	0.484671
A_44_P147396	RGD1310643_predicted	XM_230891		-0.04	-1.109	0.654205
A_44_P1044296	RGD1304881_predicted	XM_222457		-0.04	-1.109	0.510801
A_44_P241428	XM_228603	XM_228603		-0.04	-1.109	0.497466
A_44_P316867	A_44_P316867	A_44_P316867		-0.04	-1.109	0.481095
A_44_P1044878	Ap3d1	XM_234908	314633	-0.04	-1.109	0.31579
A_44_P683862	Blcap	NM_133582	171113	-0.04	-1.109	0.392532
A_44_P508079	Ndst1	NM_024361	29633	-0.04	-1.109	0.376755
A_44_P147786	BI275966	BI275966	361710	-0.04	-1.109	0.396243
A_44_P317529	AW528874	AW528874	361650	-0.05	-1.109	0.626786
A_42_P740209	Mpg	NM_012601	24561	-0.05	-1.109	0.339161
A_44_P297596	Ctnnd1_predicted	XM_242062		-0.05	-1.109	0.485562
A_44_P995887	TC555895	TC555895		-0.05	-1.109	0.730228
A_44_P281300	Txnl6_predicted	XM_224718		-0.05	-1.109	0.383753
A_42_P469248	Tnfsf12	NM_001001513	360548	-0.05	-1.109	0.713642
A_44_P536779	Kcng2	XM_225718		-0.05	-1.110	0.393051
A_44_P475940	Rassf8_predicted	XM_232528	312846	-0.05	-1.110	0.441746
A_44_P1049236	Immt	NM_001034928	312444	-0.05	-1.110	0.408571
A_44_P367513	Cyp2d13	NM_173093	24303	-0.05	-1.110	0.495806
A_44_P693659	A_44_P693659	A_44_P693659		-0.05	-1.110	0.45954
A_44_P451496	RGD1563429_predicted	XM_223154		-0.05	-1.110	0.414176
A_44_P471018	Tiparp_predicted	XM_227217		-0.05	-1.110	0.509679
A_44_P553047	Fndc3b_predicted	XM_226988	294925	-0.05	-1.110	0.590075
A_44_P747071	RGD1565941_predicted	XM_227600	295378	-0.05	-1.110	0.72178
A_44_P391275	S79271	S79271		-0.05	-1.110	0.557096
A_44_P852564	A_44_P852564	A_44_P852564		-0.05	-1.110	0.381187
A_43_P18370	TC560101	TC560101		-0.05	-1.110	0.400284
A_43_P19189	LOC680039	XM_001054175		-0.05	-1.110	0.46509
A_43_P14774	RGD1560600_predicted	XM_214099		-0.05	-1.110	0.361965
A_44_P207855	AABR03001498	AABR03001498		-0.05	-1.110	0.488056
A_44_P477116	BF548541	BF548541	140544	-0.05	-1.111	0.624512
A_44_P371166	AA955414	AA955414		-0.05	-1.111	0.611065
A_44_P417104	Sucla2_predicted	XM_341354		-0.05	-1.111	0.508349
A_44_P426806	RGD1564459_predicted	XM_342652	362331	-0.05	-1.111	0.557272
A_44_P174588	AA899215	AA899215		-0.05	-1.111	0.421509
A_44_P533778	Luzp1	NM_030830	79428	-0.05	-1.111	0.444555

A_44_P332694	Guk1	NM_001013115	303179	-0.05	-1.111	0.433422
A_44_P209840	AI716149	AI716149		-0.05	-1.111	0.443355
A_44_P281829	BF555590	BF555590	84582	-0.05	-1.111	0.284272
A_44_P654209	TC543677	TC543677		-0.05	-1.111	0.59043
A_43_P12851	Syngr2	NM_053553	89815	-0.05	-1.111	0.369841
A_44_P1026641	LOC691246	XM_001075996	691246	-0.05	-1.111	0.336566
A_44_P407740	RGD1562059_predicted	XM_342293		-0.05	-1.111	0.413669
A_44_P852679	LOC684978	XM_001061754		-0.05	-1.111	0.308779
A_42_P492082	Pex16	NM_001012088	311203	-0.05	-1.112	0.255081
A_44_P444801	Pon2	NM_001013082	296851	-0.05	-1.112	0.33728
A_42_P460609	Gabarapl2	NM_022706	64670	-0.05	-1.112	0.296316
A_44_P1050352	Dclre1a_predicted	XM_214730		-0.05	-1.112	0.302212
A_44_P885793	DY473143	DY473143		-0.05	-1.112	0.350344
A_44_P794304	TC532939	TC532939		-0.05	-1.112	0.704786
A_44_P234500	XM_235075	XM_235075		-0.05	-1.112	0.503774
A_44_P652161	ENSRNOT00000032094	ENSRNOT00000032094		-0.05	-1.112	0.325845
A_44_P596968	A_44_P596968	A_44_P596968		-0.05	-1.112	0.373576
A_42_P652419	AW920912	AW920912		-0.05	-1.112	0.463818
A_44_P539167	LOC317276	XR_009059	317276	-0.05	-1.112	0.377434
A_44_P996073	Anxa6	NM_024156	79125	-0.05	-1.112	0.718946
A_44_P214811	Tfrc	XM_001072774		-0.05	-1.112	0.489401
A_44_P356344	Ndufc2	NM_001009290	293130	-0.05	-1.112	0.466488
A_43_P19536	Gpt2_predicted	NM_001012057	307759	-0.05	-1.112	0.316336
A_44_P288306	Dusp23_predicted	XM_341156	360881	-0.05	-1.112	0.512279
A_44_P372627	A_44_P372627	A_44_P372627		-0.05	-1.112	0.350157
A_44_P862708	RGD1562181_predicted	XM_579875		-0.05	-1.112	0.345514
A_44_P382279	Socs6_predicted	XM_225667		-0.05	-1.112	0.429807
A_44_P530276	Rad51l3_predicted	XM_220773		-0.05	-1.113	0.341251
A_44_P241177	ENSRNOT00000039699	ENSRNOT00000039699		-0.05	-1.113	0.282815
A_44_P952690	A_44_P952690	A_44_P952690		-0.05	-1.113	0.365884
A_44_P885461	TC541875	TC541875		-0.05	-1.113	0.381594
A_44_P471541	Inhbe	NM_031815	83711	-0.05	-1.113	0.424603
A_42_P794130	Cdc20	NM_171993	64515	-0.05	-1.113	0.765078
A_44_P873549	TC550054	TC550054		-0.05	-1.113	0.480002
A_44_P696426	AW143061	AW143061		-0.05	-1.113	0.481447
A_44_P274505	Tgm6_predicted	XM_230601		-0.05	-1.113	0.355152
A_43_P12436	Hes5	NM_024383	79225	-0.05	-1.113	0.485315
A_44_P864286	AW915466	AW915466		-0.05	-1.113	0.51781
A_44_P217533	Capn1	NM_019152	29153	-0.05	-1.113	0.607709
A_44_P661516	CK598443	CK598443		-0.05	-1.113	0.320931
A_44_P174476	Fbln2	XM_232197	282583	-0.05	-1.113	0.728599
A_44_P699356	TC554523	TC554523		-0.05	-1.113	0.318912
A_44_P869955	TC559645	TC559645		-0.05	-1.113	0.576228
A_44_P441058	A_44_P441058	A_44_P441058		-0.05	-1.113	0.441722
A_42_P687767	RGD1308635_predicted	XM_216011	296587	-0.05	-1.113	0.344776
A_44_P363882	LOC360570	XM_001080824		-0.05	-1.113	0.402059
A_44_P944071	A_44_P944071	A_44_P944071		-0.05	-1.114	0.487457
A_44_P895660	AW916954	AW916954		-0.05	-1.114	0.505206
A_44_P102755	A_44_P102755	A_44_P102755		-0.05	-1.114	0.516001
A_44_P156513	Stx3	NM_031124	81802	-0.05	-1.114	0.39576
A_44_P252935	XM_223421	XM_223421		-0.05	-1.114	0.654929
A_44_P431112	XM_221812	XM_221812		-0.05	-1.114	0.310893
A_44_P404680	AA955550	AA955550	94194	-0.05	-1.114	0.313806
A_44_P644091	A_44_P644091	A_44_P644091		-0.05	-1.114	0.377059
A_44_P190188	A_44_P190188	A_44_P190188		-0.05	-1.114	0.630638
A_43_P13031	Barhl1	NM_057109	117232	-0.05	-1.114	0.41324
A_44_P299349	Prr3	NM_212544	361788	-0.05	-1.114	0.395279
A_44_P200968	Tlr6	NM_207604	305353	-0.05	-1.114	0.369179
A_44_P180802	Rnf151_predicted	XM_220225		-0.05	-1.114	0.434
A_44_P143806	BF420750	BF420750	288604	-0.05	-1.114	0.372911
A_44_P665738	DV728333	DV728333		-0.05	-1.114	0.583443
A_44_P337335	Ctrc	XM_001073208		-0.05	-1.114	0.578269
A_44_P370555	RGD1562833_predicted	XR_009098	365000	-0.05	-1.115	0.544902

A_43_P21370	Gprin1_predicted	XM_344571	364676	-0.05	-1.115	0.691676
A_44_P1029435	Ppm1k_predicted	XM_231833		-0.05	-1.115	0.441958
A_44_P225206	RGD1560291_predicted	XM_234957		-0.05	-1.115	0.42362
A_43_P18037	Ap3b1_predicted	XM_226666		-0.05	-1.115	0.542586
A_43_P11741	Il2ra	NM_013163	25704	-0.05	-1.115	0.662308
A_44_P215289	RGD1562436_predicted	XM_344868		-0.05	-1.115	0.677313
A_44_P367807	Mterf	NM_053499	85261	-0.05	-1.115	0.456184
A_44_P223854	LOC686892	XM_001074939		-0.05	-1.115	0.414145
A_43_P11190	Pum2	XM_216661		-0.05	-1.115	0.405049
A_44_P548676	A_44_P548676	A_44_P548676		-0.05	-1.115	0.386355
A_44_P300093	RGD1566054_predicted	XM_234029	314013	-0.05	-1.115	0.645031
A_44_P470808	Nab1	NM_022856	64824	-0.05	-1.115	0.513468
A_44_P447810	Doc2g	NM_001011937	293654	-0.05	-1.116	0.247627
A_44_P292487	Scpep1	NM_133383	114861	-0.05	-1.116	0.457059
A_44_P466866	Osbpl7_predicted	XM_220915		-0.05	-1.116	0.602727
A_44_P752355	A_44_P752355	A_44_P752355		-0.05	-1.116	0.630341
A_44_P357338	Ptp4a1	NM_031579	29463	-0.05	-1.116	0.399871
A_44_P643823	TC541851	TC541851		-0.05	-1.116	0.487318
A_44_P347641	AA956030	AA956030		-0.05	-1.116	0.453916
A_44_P409983	LOC691700	XM_001079317	691700	-0.05	-1.116	0.338957
A_44_P896312	AW144347	AW144347		-0.05	-1.116	0.60946
A_43_P21084	LOC297570	XM_232260		-0.05	-1.116	0.470388
A_44_P638741	TC558234	TC558234		-0.05	-1.116	0.593879
A_44_P306728	Gli2_predicted	XM_222557		-0.05	-1.116	0.709655
A_44_P898125	LOC363916	XR_009249	363916	-0.05	-1.116	0.624209
A_44_P196957	Plekha5	XM_342781	246237	-0.05	-1.117	0.624164
A_44_P539156	Dus3l	NM_001034923	301122	-0.05	-1.117	0.314399
A_42_P581001	S100a6	NM_053485	85247	-0.05	-1.117	0.446457
A_43_P22351	RGD1307594	NM_001024968	288454	-0.05	-1.117	0.676841
A_43_P13105	Serpinb7	NM_130404	117092	-0.05	-1.117	0.402059
A_43_P15172	Pacsin2	NM_130740	124461	-0.05	-1.117	0.34531
A_43_P13924	Emp2	NM_001007721	360468	-0.05	-1.117	0.563904
A_44_P466636	Cd72	NM_001015016	313498	-0.05	-1.117	0.419999
A_42_P533183	Slc1a3	NM_019225	29483	-0.05	-1.117	0.481136
A_43_P15073	RGD1562200_predicted	XM_343790	363471	-0.05	-1.117	0.378015
A_44_P339806	Mfn1	NM_138976	192647	-0.05	-1.117	0.359176
A_44_P250839	LOC316856	XM_229366		-0.05	-1.117	0.381594
A_44_P459452	A_44_P459452	A_44_P459452		-0.05	-1.117	0.309632
A_44_P874677	RGD1359349	NM_001007738	361744	-0.05	-1.118	0.603893
A_44_P1053578	B3gnt7	NM_001012134	316583	-0.05	-1.118	0.873286
A_44_P416672	Olr1104_predicted	NM_001000906	405216	-0.05	-1.118	0.570831
A_44_P304493	RGD1305572	NM_001024970	289315	-0.05	-1.118	0.454453
A_44_P367726	Hbld2	NM_181626	290985	-0.05	-1.118	0.278167
A_44_P1012690	Snrp70_predicted	XM_341857		-0.05	-1.118	0.347688
A_44_P255652	Akap9	XM_347223		-0.05	-1.118	0.612363
A_44_P204367	AI144744	AI144744	25526	-0.05	-1.118	0.504976
A_44_P166267	Abcb10	NM_001012166	361439	-0.05	-1.118	0.442791
A_42_P771158	Ssfa2_predicted	XM_001067501		-0.05	-1.119	0.489409
A_44_P276475	Garnl1	XM_578542	56785	-0.05	-1.119	0.469158
A_44_P876363	Vav2_predicted	XM_001075369		-0.05	-1.119	0.697622
A_44_P913700	Bxdc5	XM_575059	499725	-0.05	-1.119	0.409958
A_44_P505752	Olr1718_predicted	NM_214460	405205	-0.05	-1.119	0.496959
A_44_P452600	LOC691083	XM_001076777	691083	-0.05	-1.119	0.495517
A_44_P268381	AA964977	AA964977	25742	-0.05	-1.119	0.526796
A_44_P1021381	Taldo1	NM_031811	83688	-0.05	-1.119	0.415626
A_44_P716060	TC560359	TC560359		-0.05	-1.119	0.561628
A_44_P461912	Stag2_predicted	XM_233108	313304	-0.05	-1.119	0.527732
A_44_P250848	XM_212862	XM_212862		-0.05	-1.119	0.676719
A_44_P517820	LOC306792	XM_225216	306792	-0.05	-1.119	0.68296
A_44_P566000	TC551125	TC551125		-0.05	-1.119	0.494505
A_44_P713802	ENSRNOT00000047465	ENSRNOT00000047465		-0.05	-1.119	0.268106
A_43_P15292	Ctsb	NM_022597	64529	-0.05	-1.120	0.59545
A_44_P424289	Pabpc2_predicted	XM_225992		-0.05	-1.120	0.364508

A_43_P15989	Atp6v0a2_predicted	NM_053775	116455	-0.05	-1.120	0.418765
A_43_P15081	Hsd17b8	NM_212529	361802	-0.05	-1.120	0.315374
A_44_P199381	RGD1565679_predicted	XM_234016		-0.05	-1.120	0.338937
A_44_P1059827	AW143339	AW143339		-0.05	-1.120	0.339917
A_44_P899810	Prrg2_predicted	XM_341853		-0.05	-1.120	0.388959
A_44_P550313	Olr1332_predicted	NM_001000477	300632	-0.05	-1.120	0.647377
A_44_P557168	Dio2	NM_031720	65162	-0.05	-1.120	0.545715
A_44_P195853	BI303596	BI303596	298845	-0.05	-1.120	0.482327
A_44_P480433	Kazald1	NM_001033064	293997	-0.05	-1.120	0.355992
A_44_P437736	BF398077	BF398077		-0.05	-1.120	0.688637
A_44_P370460	Zfp697_predicted	XM_227502		-0.05	-1.120	0.302206
A_44_P328829	Nek8_predicted	XM_220639		-0.05	-1.120	0.421812
A_44_P503013	AA926148	AA926148	24310	-0.05	-1.121	0.408336
A_44_P485079	A_44_P485079	A_44_P485079		-0.05	-1.121	0.747275
A_44_P535473	BX883042	BX883042		-0.05	-1.121	0.503362
A_44_P638087	DY471385	DY471385		-0.05	-1.121	0.319834
A_44_P506481	RGD1560855_predicted	XM_228584		-0.05	-1.121	0.69338
A_44_P975513	TC536839	TC536839		-0.05	-1.121	0.382579
A_44_P276402	Gsk3a	NM_017344	50686	-0.05	-1.121	0.317854
A_44_P671098	TC561289	TC561289		-0.05	-1.121	0.306038
A_43_P15900	Ccl22	NM_057203	117551	-0.05	-1.121	0.306659
A_44_P237837	U64705	U64705		-0.05	-1.121	0.504557
A_43_P10608	BF548976	BF548976	363015	-0.05	-1.121	0.491019
A_44_P442437	BM986468	BM986468	25291	-0.05	-1.121	0.557037
A_43_P15302	Cdk5rap3	NM_024488	80278	-0.05	-1.121	0.44673
A_44_P967855	CO569441	CO569441		-0.05	-1.121	0.580544
A_44_P148523	AA925792	AA925792	24786	-0.05	-1.122	0.454006
A_44_P429520	CA506333	CA506333		-0.05	-1.122	0.529975
A_44_P408110	Sbf1_predicted	XM_001054780		-0.05	-1.122	0.376869
A_44_P461094	BM388298	BM388298		-0.05	-1.122	0.350907
A_44_P119060	RGD1559640_predicted	XM_577253	501840	-0.05	-1.122	0.295089
A_44_P1045469	DV727231	DV727231		-0.05	-1.122	0.755423
A_44_P938669	TC552370	TC552370		-0.05	-1.122	0.501859
A_44_P316540	RGD1560451_predicted	XM_345369		-0.05	-1.122	0.300551
A_44_P155109	RGD1308210	XM_341879	361601	-0.05	-1.122	0.307448
A_44_P322001	Morc3_predicted	XM_001054868		-0.05	-1.122	0.374416
A_44_P438300	LOC361487	NM_001014158	361487	-0.05	-1.122	0.311578
A_44_P560681	TC551983	TC551983		-0.05	-1.122	0.215915
A_44_P347174	Cecr5_predicted	XM_232249		-0.05	-1.122	0.354866
A_44_P306560	Hdac4	AF321132	363287	-0.05	-1.122	0.734979
A_44_P541171	RGD1311135_predicted	XM_344230	364136	-0.05	-1.122	0.252313
A_44_P794669	TC519684	TC519684		-0.05	-1.123	0.343694
A_44_P344642	Sorcs1_predicted	XM_220080	309533	-0.05	-1.123	0.505856
A_44_P324452	Rragc_predicted	XM_216515		-0.05	-1.123	0.451336
A_44_P212796	X76129	X76129		-0.05	-1.123	0.73062
A_44_P261012	LOC687022	XM_001078538		-0.05	-1.123	0.255722
A_44_P576564	XM_574922	XM_574922		-0.05	-1.123	0.553346
A_44_P523253	C1qtnf6	NM_001034932	315114	-0.05	-1.123	0.55
A_44_P517944	XM_217706	XM_217706		-0.05	-1.123	0.395116
A_43_P18296	XM_340859	XM_340859		-0.05	-1.123	0.242204
A_42_P499899	Prg2	NM_031619	58826	-0.05	-1.123	0.404472
A_44_P335609	Dscr1l2	NM_001012746	362627	-0.05	-1.123	0.371777
A_44_P1053032	RGD1304572_predicted	XM_001073394		-0.05	-1.123	0.355404
A_42_P685138	Dab1	NM_153621	266729	-0.05	-1.123	0.344857
A_44_P774690	AW917977	AW917977		-0.05	-1.123	0.253391
A_44_P285359	AW527958	AW527958	266729	-0.05	-1.123	0.558038
A_42_P786317	Snapc4_predicted	XM_342389		-0.05	-1.123	0.487581
A_44_P947632	TC549133	TC549133		-0.05	-1.123	0.289693
A_43_P12325	B4galnt1	NM_022860	64828	-0.05	-1.123	0.545983
A_44_P263042	LOC314600	XM_001081510		-0.05	-1.123	0.595436
A_44_P393696	LOC308990	NM_001025001	308990	-0.05	-1.123	0.403548
A_42_P543895	Pou3f4	NM_017252	29589	-0.05	-1.123	0.738519
A_44_P168083	Tegt	XM_576343		-0.05	-1.123	0.680469

A_44_P737624	A_44_P737624	A_44_P737624		-0.05	-1.123	0.808252
A_43_P12127	Ensa	NM_021842	60334	-0.05	-1.123	0.235648
A_44_P394550	Kiaa0415	NM_001037220	641386	-0.05	-1.123	0.540076
A_44_P229557	RGD1561554_predicted	XM_230513	311362	-0.05	-1.123	0.434091
A_43_P11005	Ppp1r13b_predicted	XM_234555		-0.05	-1.124	0.301709
A_43_P12798	Asah1	NM_053407	84431	-0.05	-1.124	0.438129
A_44_P570757	A_44_P570757	A_44_P570757		-0.05	-1.124	0.638991
A_44_P890117	TC565947	TC565947		-0.05	-1.124	0.488876
A_44_P176466	Wt1	NM_031534	24883	-0.05	-1.124	0.375738
A_44_P222810	Btbd7_predicted	XM_343098		-0.05	-1.124	0.25007
A_44_P218407	Pdcd7_predicted	XM_343413		-0.05	-1.124	0.438255
A_44_P928761	ENSRNOT00000032787	ENSRNOT00000032787		-0.05	-1.124	0.347402
A_44_P1031479	RGD1565591_predicted	XM_001077382		-0.05	-1.124	0.41177
A_44_P229842	XM_213245	XM_213245		-0.05	-1.124	0.530782
A_44_P683538	TC568208	TC568208		-0.05	-1.124	0.483926
A_44_P377733	A_44_P377733	A_44_P377733		-0.05	-1.124	0.453865
A_44_P382104	XM_223643	XM_223643		-0.05	-1.124	0.553744
A_44_P548502	Cep76	XM_214549	291540	-0.05	-1.124	0.443161
A_44_P286439	Trim42	NM_001013955	301106	-0.05	-1.124	0.52523
A_44_P140449	LOC680021	XM_001055358	680021	-0.05	-1.125	0.37344
A_44_P433651	XM_224197	XM_224197		-0.05	-1.125	0.629592
A_44_P482753	XM_218993	XM_218993		-0.05	-1.125	0.261268
A_44_P182085	AI237710	AI237710	500989	-0.05	-1.125	0.317706
A_44_P484228	BI278547	BI278547	300027	-0.05	-1.125	0.634494
A_44_P424440	LOC291480	XM_225800		-0.05	-1.125	0.372911
A_43_P18380	XM_215101	XM_215101		-0.05	-1.125	0.550088
A_44_P226315	Slc16a10	NM_138831	170566	-0.05	-1.125	0.689405
A_44_P859113	Ripk5	NM_199463	304791	-0.05	-1.125	0.50648
A_44_P345298	Cort	NM_012835	25305	-0.05	-1.125	0.349421
A_44_P396496	LOC682248	XM_001060664		-0.05	-1.125	0.366615
A_44_P192000	RGD1562305_predicted	XM_214902		-0.05	-1.125	0.465841
A_44_P409061	AA997598	AA997598	245961	-0.05	-1.125	0.500216
A_44_P915388	TC523810	TC523810		-0.05	-1.125	0.383191
A_44_P555123	Vamp2	AJ133104	24803	-0.05	-1.126	0.433753
A_44_P445701	ENSRNOT00000045507	ENSRNOT00000045507		-0.05	-1.126	0.390273
A_42_P709819	Tmed7	XM_001063185		-0.05	-1.126	0.458089
A_42_P716310	RGD1307615_predicted	XM_342385		-0.05	-1.126	0.400385
A_43_P22590	XM_215841	XM_215841		-0.05	-1.126	0.319995
A_44_P1042596	AI179987	AI179987	114637	-0.05	-1.126	0.383557
A_44_P342412	RGD1308317_predicted	XM_225044		-0.05	-1.126	0.390477
A_42_P547847	Fbp2	NM_053716	114508	-0.05	-1.126	0.335857
A_44_P282398	AA943558	AA943558		-0.05	-1.126	0.540235
A_44_P372958	Tsga13_predicted	XM_231582		-0.05	-1.126	0.314168
A_43_P20485	RGD1560373_predicted	XM_221655		-0.05	-1.126	0.45472
A_44_P243145	Nsf	NM_021748	60355	-0.05	-1.126	0.215188
A_44_P135832	RGD1310748_predicted	XM_234063		-0.05	-1.126	0.249943
A_44_P839357	TC543781	TC543781		-0.05	-1.126	0.358145
A_44_P741147	AW917588	AW917588		-0.05	-1.126	0.374743
A_44_P1034018	RGD1308356	NM_001033893	303112	-0.05	-1.126	0.353841
A_44_P212056	AA997830	AA997830	306490	-0.05	-1.126	0.491695
A_43_P15390	Madh7	NM_030858	81516	-0.05	-1.127	0.387143
A_44_P395950	ENSRNOT00000038784	ENSRNOT00000038784		-0.05	-1.127	0.610169
A_43_P19259	Igf2bp3	XM_231739		-0.05	-1.127	0.816181
A_43_P21541	Igsf3_predicted	XM_227520		-0.05	-1.127	0.405682
A_44_P915100	TC540416	TC540416		-0.05	-1.127	0.412115
A_44_P196198	Per3	NM_023978	78962	-0.05	-1.127	0.261941
A_44_P853624	RGD1308321_predicted	XM_001063334		-0.05	-1.127	0.446072
A_43_P21989	RGD1308380_predicted	XM_223529	305443	-0.05	-1.127	0.314984
A_44_P785821	RGD1560775_predicted	XM_576442	501031	-0.05	-1.127	0.288655
A_44_P222673	Tysnd1_predicted	XM_345106		-0.05	-1.127	0.369613
A_44_P761492	TC555931	TC555931		-0.05	-1.127	0.382579
A_42_P490296	Itgb4	NM_013180	25724	-0.05	-1.127	0.540018
A_44_P395538	Rabep2	NM_030585	80754	-0.05	-1.127	0.353183

A_44_P459710	LOC299220	XM_001059446		-0.05	-1.128	0.413029
A_44_P398855	XM_347119	XM_347119		-0.05	-1.128	0.240872
A_42_P556656	Pias4	XM_343155	362827	-0.05	-1.128	0.331446
A_44_P300910	CA505548	CA505548	289740	-0.05	-1.128	0.498534
A_44_P113933	Tyms	NM_019179	29261	-0.05	-1.128	0.51913
A_44_P636717	LOC685106	XM_001062312		-0.05	-1.128	0.425005
A_44_P222626	RGD1310645_predicted	XM_225679	307213	-0.05	-1.128	0.379837
A_44_P389717	BQ203711	BQ203711	360661	-0.05	-1.128	0.546568
A_44_P255359	Ndufs2	NM_001011907	289218	-0.05	-1.128	0.230961
A_44_P841829	TC564079	TC564079		-0.05	-1.128	0.434583
A_44_P538808	Wnt11	XM_238122	140584	-0.05	-1.128	0.524571
A_44_P231737	RGD1562755_predicted	XM_223453		-0.05	-1.128	0.242428
A_44_P365765	Slmap_predicted	XM_224579		-0.05	-1.128	0.412318
A_44_P126355	Rnpc1_predicted	XM_345477		-0.05	-1.128	0.405228
A_42_P538084	Crim1_predicted	XM_233798	298744	-0.05	-1.129	0.343407
A_44_P369083	AA996984	AA996984	287435	-0.05	-1.129	0.616535
A_44_P229051	Epim	NM_012748	25130	-0.05	-1.129	0.547513
A_43_P22256	Foxj3_predicted	XM_233463		-0.05	-1.129	0.292473
A_44_P497744	BQ204816	BQ204816	287522	-0.05	-1.129	0.642723
A_44_P314876	Six5_predicted	XM_001075407		-0.05	-1.129	0.377454
A_44_P412413	RGD1565366_predicted	XM_215056		-0.05	-1.129	0.491019
A_44_P377136	PRP-2	M86514	287750	-0.05	-1.129	0.547999
A_42_P688367	RGD1311625_predicted	XM_227694	310859	-0.05	-1.129	0.212336
A_43_P17298	Asahl_predicted	NM_001010967	497009	-0.05	-1.129	0.60014
A_43_P16517	Gpr107_predicted	XM_231134		-0.05	-1.129	0.334164
A_44_P433771	Reep5_predicted	XM_344661		-0.05	-1.129	0.4601
A_44_P214054	Tmed2	NM_031722	65165	-0.05	-1.129	0.389175
A_44_P533517	AA964687	AA964687	64154	-0.05	-1.129	0.465702
A_44_P420198	AW434978	AW434978		-0.05	-1.129	0.439154
A_44_P260624	Ppcdc_predicted	XM_343397		-0.05	-1.129	0.257711
A_42_P710659	Drd1ip	NM_138915	192349	-0.05	-1.129	0.320505
A_42_P524312	RGD1563531_predicted	XM_230003	311112	-0.05	-1.130	0.355162
A_44_P133453	XM_236776	XM_236776		-0.05	-1.130	0.484807
A_44_P389098	Atxn2_predicted	XM_213779		-0.05	-1.130	0.292808
A_44_P593368	BG673595	BG673595		-0.05	-1.130	0.601598
A_44_P297690	LOC680423	XM_001057104		-0.05	-1.130	0.387471
A_44_P321510	LOC691431	XM_001078245	691431	-0.05	-1.130	0.683214
A_44_P302301	RGD1563652_predicted	XM_001073081		-0.05	-1.130	0.589593
A_44_P121430	Peg12_predicted	XM_218737		-0.05	-1.130	0.514778
A_44_P196717	Stk32c_predicted	XM_344970		-0.05	-1.130	0.501621
A_44_P345319	Chrd	XM_221307	117275	-0.05	-1.130	0.578167
A_44_P262324	Fut2	NM_031635	58924	-0.05	-1.130	0.403251
A_44_P576733	LOC680014	XM_001055446	680014	-0.05	-1.130	0.340373
A_44_P660551	A_44_P660551	A_44_P660551		-0.05	-1.130	0.500998
A_44_P240800	Olr865_predicted	NM_001000410	298474	-0.05	-1.130	0.4413
A_44_P330806	Dsg2_predicted	XM_226112	307562	-0.05	-1.130	0.425103
A_44_P433273	ENSRNOT00000043973	ENSRNOT00000043973		-0.05	-1.130	0.423288
A_44_P272035	Senp2	NM_023989	78973	-0.05	-1.130	0.491695
A_44_P251692	Sfrs5	NM_019257	29667	-0.05	-1.130	0.409884
A_44_P276819	A_44_P276819	A_44_P276819		-0.05	-1.130	0.619063
A_44_P142850	RGD1304969_predicted	XM_220157		-0.05	-1.131	0.377806
A_44_P165962	RGD1560435_predicted	XM_218352		-0.05	-1.131	0.434603
A_44_P461201	Pcsk7	NM_019246	29606	-0.05	-1.131	0.555647
A_44_P281782	Osbpl11_predicted	XM_221396		-0.05	-1.131	0.467503
A_44_P623001	TC556078	TC556078		-0.05	-1.131	0.43895
A_44_P231983	Tead3	XM_215356		-0.05	-1.131	0.200448
A_44_P220351	LOC685687	XM_001064825	682507	-0.05	-1.131	0.53793
A_44_P128151	RT1-S3	XM_215303		-0.05	-1.131	0.400868
A_44_P243126	Cutl1	XM_347163	116639	-0.05	-1.131	0.534964
A_44_P406890	BC099158	BC099158		-0.05	-1.131	0.348569
A_43_P18969	Fkbp5	NM_001012174	361810	-0.05	-1.131	0.267433
A_44_P289862	Abhd6	NM_001007680	305795	-0.05	-1.131	0.675114
A_44_P629801	A_44_P629801	A_44_P629801		-0.05	-1.132	0.321149

A_44_P266892	Olr224_predicted	NM_001000553	365342	-0.05	-1.132	0.715429
A_44_P183142	XM_343286	XM_343286		-0.05	-1.132	0.312585
A_44_P511581	F13a1	NM_021698	60327	-0.05	-1.132	0.603252
A_43_P18736	XM_342224	XM_342224		-0.05	-1.132	0.464779
A_44_P193959	Rgs14	NM_053764	114705	-0.05	-1.132	0.499085
A_44_P287911	Af6	NM_013217	26955	-0.05	-1.132	0.262013
A_43_P15209	LOC311254	XM_001079389		-0.05	-1.132	0.464583
A_44_P747545	TC562450	TC562450		-0.05	-1.132	0.480308
A_44_P837935	TC521018	TC521018		-0.05	-1.132	0.46181
A_44_P241448	MGC109340	NM_001024267	317409	-0.05	-1.132	0.524156
A_44_P151609	Centg1	XM_001054911		-0.05	-1.132	0.466862
A_44_P888044	TC529772	TC529772		-0.05	-1.132	0.458194
A_44_P196401	RT1-Aw2	Y13890	24737	-0.05	-1.132	0.417918
A_44_P1045105	XM_218427	XM_218427		-0.05	-1.132	0.288148
A_44_P442359	AA926356	AA926356	89824	-0.05	-1.132	0.371177
A_44_P976336	TC517445	TC517445		-0.05	-1.133	0.299749
A_44_P1025349	Ube1l_predicted	XM_217252		-0.05	-1.133	0.346556
A_44_P219759	Yipf5	NM_001014150	361315	-0.05	-1.133	0.48676
A_44_P643560	A_44_P643560	A_44_P643560		-0.05	-1.133	0.360189
A_44_P357089	Fbxl20	NM_022272	64039	-0.05	-1.133	0.495313
A_44_P191021	Phyh	NM_053674	114209	-0.05	-1.133	0.562275
A_44_P499482	LOC686933	XM_001076375		-0.05	-1.133	0.499764
A_42_P549380	Ilkap	NM_022606	64538	-0.05	-1.133	0.428785
A_44_P501357	Copa	XM_222899		-0.05	-1.133	0.248259
A_44_P146123	AA892855	AA892855	309201	-0.05	-1.133	0.284122
A_44_P163421	Cacna1b	NM_147141	257648	-0.05	-1.133	0.423242
A_44_P1021682	Smarca2	NM_001004446	361745	-0.05	-1.133	0.328855
A_44_P777424	MGC125073	XM_001059161		-0.05	-1.133	0.311073
A_42_P571169	Ttc17	XM_230313		-0.05	-1.134	0.394492
A_44_P634946	Mdm2_predicted	XM_235169		-0.05	-1.134	0.391473
A_44_P432366	AI177871	AI177871	360804	-0.05	-1.134	0.436273
A_44_P974975	LOC684194	XM_001069349		-0.05	-1.134	0.200834
A_44_P156450	Ppm1b	NM_033096	24667	-0.05	-1.134	0.32357
A_44_P131209	XM_236489	XM_236489		-0.05	-1.134	0.276209
A_44_P971306	AI715122	AI715122		-0.05	-1.134	0.704393
A_44_P961856	TC560421	TC560421		-0.05	-1.134	0.363352
A_44_P899064	TC517011	TC517011		-0.05	-1.134	0.351355
A_43_P19260	RGD1559803_predicted	XM_001053368		-0.05	-1.134	0.299975
A_44_P290139	RGD735175	NM_199112	316530	-0.05	-1.135	0.262441
A_44_P1037523	Msto1_predicted	XM_215631		-0.05	-1.135	0.351586
A_44_P368123	Lims2	NM_001012163	361303	-0.05	-1.135	0.502738
A_43_P22076	RGD1564130_predicted	XM_347206	368042	-0.05	-1.135	0.494064
A_43_P12737	Hagh	NM_033349	24439	-0.05	-1.135	0.288416
A_42_P774878	Stxbp3	NM_053637	114095	-0.06	-1.135	0.345835
A_44_P426914	LOC683882	XM_001067474		-0.06	-1.135	0.4412
A_44_P227655	AI171249	AI171249		-0.06	-1.135	0.265638
A_44_P241126	LOC361646	XM_341925	361646	-0.06	-1.135	0.310263
A_43_P15600	Rab7l1	NM_133590	171122	-0.06	-1.135	0.342652
A_44_P419337	Kdelr2	NM_001013122	304290	-0.06	-1.135	0.417015
A_42_P462543	LOC368062	XM_347227	368062	-0.06	-1.135	0.366122
A_42_P589418	Bcar1	NM_012931	25414	-0.06	-1.136	0.243867
A_43_P15344	Atp5g3	NM_053756	114630	-0.06	-1.136	0.362712
A_44_P107785	Amfr_predicted	XM_341644	361367	-0.06	-1.136	0.432501
A_44_P175637	Gpr61_predicted	XM_227581		-0.06	-1.136	0.329174
A_44_P522606	LOC500594	XM_575967		-0.06	-1.136	0.550895
A_44_P869639	RGD1310433_predicted	XM_238368		-0.06	-1.136	0.270374
A_44_P623559	TC559924	TC559924		-0.06	-1.136	0.463462
A_44_P405583	Ndufa9	XM_001064382		-0.06	-1.136	0.404176
A_44_P447701	A_44_P447701	A_44_P447701		-0.06	-1.136	0.374195
A_44_P650529	CF111029	CF111029		-0.06	-1.137	0.471211
A_43_P21657	CB546974	CB546974		-0.06	-1.137	0.59374
A_44_P558784	AW918767	AW918767		-0.06	-1.137	0.46068
A_43_P11763	Add1	Z49081	24170	-0.06	-1.137	0.238055

A_44_P379903	XM_224509	XM_224509		-0.06	-1.137	0.430991
A_43_P18953	LOC680915	XM_001059437	680915	-0.06	-1.137	0.380739
A_44_P545314	Vps4a	NM_145678	246772	-0.06	-1.137	0.224556
A_42_P698874	RGD1309059_predicted	XM_234484		-0.06	-1.137	0.322057
A_42_P685368	Dapk2	NM_001013109	300799	-0.06	-1.137	0.436876
A_44_P776252	TC553461	TC553461		-0.06	-1.137	0.27871
A_44_P971500	Ivns1abp_predicted	XM_213898		-0.06	-1.137	0.307001
A_44_P248634	RGD1311907_predicted	XM_235559		-0.06	-1.137	0.349123
A_42_P538337	BF555840	BF555840	311857	-0.06	-1.137	0.191071
A_44_P945986	LOC681896	XM_001058858		-0.06	-1.137	0.247018
A_44_P287847	Ncr3	NM_181822	294251	-0.06	-1.137	0.59736
A_43_P15470	Adrb1	NM_012701	24925	-0.06	-1.137	0.319584
A_44_P227154	RGD1306441	XM_224432		-0.06	-1.137	0.453659
A_42_P667829	Clcnk1	NM_053327	79425	-0.06	-1.137	0.54823
A_44_P869930	TC559842	TC559842		-0.06	-1.137	0.24062
A_44_P400749	A_44_P400749	A_44_P400749		-0.06	-1.137	0.47445
A_43_P13333	Adam6	NM_138906	192271	-0.06	-1.138	0.35714
A_44_P144635	LOC682158	XM_001060178		-0.06	-1.138	0.167709
A_44_P591983	TC556497	TC556497		-0.06	-1.138	0.283969
A_43_P17397	RGD1310139_predicted	XM_226732	310040	-0.06	-1.138	0.311973
A_44_P235090	Btbd11_predicted	XM_001079428		-0.06	-1.138	0.74061
A_44_P476372	Ubqln4_predicted	XM_001074121		-0.06	-1.138	0.184404
A_44_P139749	Hrasls3	NM_017060	24913	-0.06	-1.138	0.459153
A_44_P989613	RGD1560046_predicted	XM_574335	499056	-0.06	-1.138	0.349631
A_44_P182879	Snx24	NM_001008364	361328	-0.06	-1.138	0.334039
A_44_P250488	Btbd1	NM_001011932	293060	-0.06	-1.138	0.359418
A_43_P21639	RGD1308089_predicted	XM_234320	314249	-0.06	-1.138	0.24729
A_44_P387025	ENSRNOT00000045717	ENSRNOT00000045717		-0.06	-1.138	0.578107
A_44_P576610	TC539948	TC539948		-0.06	-1.138	0.428785
A_44_P208157	Dnajc5	NM_024161	79130	-0.06	-1.138	0.199527
A_44_P173414	RGD1563178_predicted	XM_228582	302424	-0.06	-1.138	0.345694
A_44_P414353	Olr586_predicted	NM_001000660	404860	-0.06	-1.138	0.642272
A_44_P391979	Centg2_predicted	XM_237381		-0.06	-1.138	0.48103
A_43_P17591	RGD1309266_predicted	XM_237056	316335	-0.06	-1.138	0.259668
A_44_P867246	RT1-CE16	NM_001008839	24737	-0.06	-1.138	0.536496
A_43_P15602	LOC171412	NM_134391	171412	-0.06	-1.138	0.385616
A_44_P1020920	RGD1306714	XM_218479	308492	-0.06	-1.138	0.30593
A_44_P899209	Rit1_predicted	XM_574975		-0.06	-1.138	0.402643
A_44_P1023515	Ube2g2_predicted	XM_215371		-0.06	-1.138	0.353459
A_44_P291114	Azi2	NM_001025705	316051	-0.06	-1.138	0.40361
A_44_P312038	RGD1560019_predicted	XM_345959		-0.06	-1.139	0.606503
A_44_P461551	Rabl5	NM_001011902	288585	-0.06	-1.139	0.392773
A_44_P441966	H31108	H31108		-0.06	-1.139	0.287444
A_44_P367600	Olr889_predicted	NM_001000061	288818	-0.06	-1.139	0.263355
A_42_P744495	Cd63	NM_017125	29186	-0.06	-1.139	0.431378
A_43_P13742	AA817749	AA817749		-0.06	-1.139	0.278909
A_44_P174261	LOC680080	XM_001055588	680080	-0.06	-1.139	0.406949
A_44_P305064	A_44_P305064	A_44_P305064		-0.06	-1.139	0.478768
A_44_P238352	Olr203_predicted	NM_001000191	293350	-0.06	-1.139	0.498258
A_44_P775866	RGD1565917_predicted	XM_578572		-0.06	-1.139	0.330689
A_44_P976358	TC523929	TC523929		-0.06	-1.139	0.715845
A_42_P521940	XM_218198	XM_218198		-0.06	-1.139	0.440066
A_44_P395788	Wnt7a	XM_342723	114850	-0.06	-1.139	0.304144
A_44_P513677	XM_233515	XM_233515		-0.06	-1.139	0.560743
A_43_P17619	BF564613	BF564613	498333	-0.06	-1.139	0.439407
A_44_P839206	LOC499474	NM_001029925	499474	-0.06	-1.139	0.34591
A_44_P822464	Cap350	XM_001067472		-0.06	-1.139	0.342246
A_44_P1027846	Cited2	NM_053698	114490	-0.06	-1.140	0.307041
A_44_P285318	AI044237	AI044237	364534	-0.06	-1.140	0.522011
A_43_P19155	RGD1565362_predicted	XM_001055385		-0.06	-1.140	0.289611
A_44_P197128	XM_343277	XM_343277		-0.06	-1.140	0.308925
A_43_P20849	Slc35c2	XM_230856		-0.06	-1.140	0.585089
A_44_P117323	Ict1_predicted	XM_221110	303673	-0.06	-1.140	0.239842

A_44_P219516	AW252650	AW252650	292845	-0.06	-1.140	0.332299
A_44_P382570	LOC360479	NM_001014115	360479	-0.06	-1.140	0.43003
A_42_P759192	LOC498266	NM_001017494	498266	-0.06	-1.140	0.309331
A_44_P171098	Gsg1	NM_001013166	312793	-0.06	-1.140	0.608689
A_44_P105160	A_44_P105160	A_44_P105160		-0.06	-1.140	0.374743
A_44_P313890	XM_344873	XM_344873		-0.06	-1.140	0.554725
A_44_P637020	TC530451	TC530451		-0.06	-1.140	0.284272
A_44_P101493	XM_343028	XM_343028		-0.06	-1.140	0.252526
A_44_P370299	RGD1564463_predicted	XM_223005		-0.06	-1.140	0.628783
A_44_P170120	Vps36_predicted	XM_214382		-0.06	-1.140	0.296811
A_44_P412491	RGD1309487	NM_001013909	294783	-0.06	-1.140	0.560834
A_44_P264292	Maoa	XM_343764	29253	-0.06	-1.140	0.256309
A_44_P1031457	Rsb66	NM_181694	296610	-0.06	-1.141	0.467772
A_44_P494679	XM_346304	XM_346304		-0.06	-1.141	0.489716
A_44_P531111	AA893186	AA893186	300235	-0.06	-1.141	0.546568
A_44_P1048901	Fkbpl	NM_001002818	406168	-0.06	-1.141	0.204603
A_43_P14615	BF556399	BF556399		-0.06	-1.141	0.811356
A_44_P331003	Arid4a_predicted	XM_234281		-0.06	-1.141	0.226519
A_43_P10367	LOC300768	XM_001074062		-0.06	-1.141	0.552109
A_44_P283646	Plekhf1	NM_001013148	308543	-0.06	-1.141	0.445064
A_44_P232585	Adcy1_predicted	XM_223616		-0.06	-1.141	0.31928
A_44_P641302	RGD1564611_predicted	XM_341025	360753	-0.06	-1.141	0.341771
A_42_P659244	Cbx8	NM_001034078	303731	-0.06	-1.141	0.284122
A_43_P11370	Plp2_mapped	NM_207601	302562	-0.06	-1.141	0.429153
A_44_P656940	TC526990	TC526990		-0.06	-1.141	0.470484
A_44_P512214	AI010491	AI010491	259274	-0.06	-1.141	0.438304
A_44_P220661	A_44_P220661	A_44_P220661		-0.06	-1.141	0.229086
A_44_P991896	LOC691125	XM_001076905	691125	-0.06	-1.141	0.460129
A_44_P108017	RGD1563856_predicted	XM_343461	363126	-0.06	-1.141	0.166774
A_43_P13124	Pacsin2	NM_130740	124461	-0.06	-1.141	0.203884
A_44_P173072	Nosip_predicted	XM_214926		-0.06	-1.141	0.234393
A_44_P121867	LOC300332	XR_008746	300332	-0.06	-1.141	0.466581
A_43_P23342	Msh5	NM_212536	294252	-0.06	-1.141	0.648738
A_44_P481551	AA965220	AA965220	29739	-0.06	-1.141	0.434822
A_43_P14186	Arl5a	NM_053979	117050	-0.06	-1.141	0.304208
A_43_P17345	Ubp1_predicted	XM_238562	301038	-0.06	-1.141	0.299791
A_44_P793098	TC543459	TC543459		-0.06	-1.142	0.594871
A_44_P745393	ENSRNOT00000023274	ENSRNOT00000023274		-0.06	-1.142	0.516463
A_44_P407623	RGD1562278_predicted	XM_574558	499263	-0.06	-1.142	0.329398
A_44_P177950	Med28_predicted	XM_223463		-0.06	-1.142	0.498194
A_44_P335446	Dusp2	NM_001012089	311406	-0.06	-1.142	0.590363
A_44_P102845	Ube2v1_predicted	XM_215948		-0.06	-1.142	0.237507
A_42_P788087	Praf2_predicted	XM_346274		-0.06	-1.142	0.407875
A_44_P381822	Aldh5a1	XM_214478	291133	-0.06	-1.142	0.615364
A_44_P347384	RGD1305773_predicted	XM_213209	287044	-0.06	-1.142	0.407889
A_44_P929104	RGD1560964_predicted	XM_215220		-0.06	-1.142	0.227927
A_44_P187075	S63519	S63519		-0.06	-1.142	0.572517
A_44_P1071390	AY156086	AY156086		-0.06	-1.142	0.439407
A_44_P252093	Secisbp2	NM_024002	79049	-0.06	-1.142	0.21134
A_44_P347981	AW433572	AW433572	619581	-0.06	-1.142	0.214694
A_44_P414242	Grm6	NM_022920	24419	-0.06	-1.142	0.61118
A_42_P841987	Nrbf2	NM_022186	58839	-0.06	-1.142	0.344092
A_44_P638478	TC541382	TC541382		-0.06	-1.142	0.237175
A_42_P687133	Nrgn	NM_024140	64356	-0.06	-1.143	0.64053
A_44_P234490	XM_217166	XM_217166		-0.06	-1.143	0.626537
A_44_P206091	LOC682467	XM_001061628		-0.06	-1.143	0.193376
A_44_P300300	CA509607	CA509607	64465	-0.06	-1.143	0.227924
A_44_P129714	Ctsf	NM_001034110	361704	-0.06	-1.143	0.454452
A_44_P680949	AW143235	AW143235		-0.06	-1.143	0.582211
A_44_P456876	Lass4_predicted	XM_221796		-0.06	-1.143	0.590951
A_44_P355657	Pik4ca	NM_022301	64161	-0.06	-1.143	0.196853
A_44_P403227	Wasf1	NM_001025114	294568	-0.06	-1.143	0.366074
A_44_P580559	TC563465	TC563465		-0.06	-1.143	0.2726

A_44_P219915	Psme4	XM_001055468	498433	-0.06	-1.143	0.326267
A_44_P855500	TC532101	TC532101		-0.06	-1.143	0.208913
A_44_P144916	Ctdp1_predicted	XM_225717		-0.06	-1.143	0.243578
A_44_P342756	RGD1566272_predicted	XM_346024		-0.06	-1.143	0.244741
A_44_P883641	LOC500956	NM_001025054	500956	-0.06	-1.144	0.386298
A_42_P799702	Aard	NM_145093	246323	-0.06	-1.144	0.389925
A_44_P435494	LOC360760	XM_346914	360760	-0.06	-1.144	0.300148
A_44_P192612	A_44_P192612	A_44_P192612		-0.06	-1.144	0.392988
A_44_P715561	RGD1564491_predicted	XM_221305	303812	-0.06	-1.144	0.475263
A_44_P281661	RGD1311988_predicted	XM_236420		-0.06	-1.144	0.410926
A_44_P278519	Olr202_predicted	NM_001000550	365337	-0.06	-1.144	0.575986
A_44_P397995	AY325222	AY325222	503252	-0.06	-1.144	0.41271
A_44_P729931	LOC682404	XM_001061367		-0.06	-1.144	0.313658
A_44_P444343	AW142434	AW142434	310653	-0.06	-1.144	0.166902
A_44_P597706	AA875152	AA875152		-0.06	-1.144	0.496958
A_44_P213415	Vdac1	NM_031353	83529	-0.06	-1.144	0.414024
A_44_P330417	Twist1	NM_053530	85489	-0.06	-1.145	0.308705
A_44_P498937	Hspb7	XM_342966		-0.06	-1.145	0.237987
A_44_P461803	LOC362414	NM_001025734	362414	-0.06	-1.145	0.29216
A_44_P558133	XM_343295	XM_343295		-0.06	-1.145	0.343475
A_44_P267487	RGD1560401_predicted	XM_215383		-0.06	-1.145	0.275454
A_44_P869457	TC540667	TC540667		-0.06	-1.145	0.165606
A_44_P101233	AI175779	AI175779		-0.06	-1.145	0.42172
A_44_P177898	Clec11a	NM_001012459	29313	-0.06	-1.145	0.577907
A_43_P14874	Arf5	NM_024149	79117	-0.06	-1.145	0.393384
A_44_P899002	TC536420	TC536420		-0.06	-1.146	0.176176
A_44_P469492	LOC686495	XM_001074408		-0.06	-1.146	0.333173
A_44_P308447	AI029499	AI029499	361571	-0.06	-1.146	0.188905
A_44_P154766	Nudt12_predicted	XM_346080		-0.06	-1.146	0.456446
A_44_P326439	Tmem16f_predicted	XM_235640		-0.06	-1.146	0.499534
A_43_P18672	Sh3bp1	XM_235500	727679	-0.06	-1.146	0.251463
A_43_P13278	Atp2c2	NM_134462	171496	-0.06	-1.146	0.35075
A_44_P760832	TC551871	TC551871		-0.06	-1.146	0.34912
A_44_P1051042	C8g_predicted	XM_215990		-0.06	-1.146	0.47721
A_42_P634825	Stambp	NM_138531	171565	-0.06	-1.146	0.303304
A_44_P152307	TC522410	TC522410		-0.06	-1.146	0.231102
A_44_P575687	LOC686558	XM_001074722		-0.06	-1.147	0.343611
A_44_P496721	RGD1560277_predicted	XM_218822		-0.06	-1.147	0.366829
A_44_P452588	Nrg2	XM_344662	432361	-0.06	-1.147	0.34505
A_44_P135740	XM_346296	XM_346296		-0.06	-1.147	0.489773
A_44_P377198	LOC316052	XM_001061749	316052	-0.06	-1.147	0.622306
A_43_P23288	RGD1562515_predicted	XM_575556		-0.06	-1.147	0.553379
A_43_P18485	B4galt7	NM_001031661	364675	-0.06	-1.147	0.24952
A_44_P730758	TC541960	TC541960		-0.06	-1.147	0.206625
A_44_P309081	Hspa2	NM_021863	60460	-0.06	-1.147	0.377042
A_44_P479930	Kidins220	NM_053795	116478	-0.06	-1.147	0.231833
A_44_P362200	BC092191	BC092191		-0.06	-1.147	0.340718
A_44_P558173	Mrpl55_predicted	XM_213321		-0.06	-1.147	0.365467
A_43_P16968	RGD1562008_predicted	XM_236927	316228	-0.06	-1.148	0.340456
A_43_P23474	LOC363009	XM_343342		-0.06	-1.148	0.192413
A_44_P286484	RGD1306073_predicted	XM_001062053		-0.06	-1.148	0.342477
A_44_P1000400	XM_238571	XM_238571		-0.06	-1.148	0.284628
A_44_P839612	TC525768	TC525768		-0.06	-1.148	0.248321
A_44_P882084	AW919062	AW919062		-0.06	-1.148	0.282599
A_44_P300018	Map3k7_predicted	XM_232855		-0.06	-1.148	0.232675
A_44_P464083	LOC364558	NM_001014237	364558	-0.06	-1.148	0.223642
A_44_P875024	BE103036	BE103036		-0.06	-1.148	0.245542
A_44_P194531	Ubadc1	NM_001007742	362087	-0.06	-1.148	0.42935
A_44_P911187	AA858651	AA858651		-0.06	-1.148	0.38046
A_44_P265338	A_44_P265338	A_44_P265338		-0.06	-1.148	0.240831
A_44_P410272	MGC72560	NM_199102	308869	-0.06	-1.148	0.258494
A_44_P187388	LOC295077	XR_008019	295077	-0.06	-1.148	0.169234
A_44_P913381	RGD1565611_predicted	XM_573144		-0.06	-1.148	0.260238

A_44_P733598	LOC680730	XM_001058607		-0.06	-1.148	0.29394
A_43_P13808	LOC497941	NM_001017477	497941	-0.06	-1.148	0.281857
A_44_P334736	Edn1	NM_012548	24323	-0.06	-1.148	0.549651
A_44_P560961	Ell_predicted	XM_224727		-0.06	-1.148	0.274945
A_43_P17003	RGD727788	NM_182822	313453	-0.06	-1.149	0.486802
A_44_P350646	AI029858	AI029858		-0.06	-1.149	0.550661
A_44_P355758	Ralb	NM_053821	116546	-0.06	-1.149	0.355387
A_44_P138388	RGD1308106_predicted	XM_001076732		-0.06	-1.149	0.486734
A_44_P482512	Aplp2	XM_343513	64312	-0.06	-1.149	0.271164
A_44_P822989	RGD1562965_predicted	XM_001070627		-0.06	-1.149	0.223794
A_44_P536380	Gmeb2	NM_031803	83635	-0.06	-1.149	0.426531
A_44_P212922	Zfp329_predicted	XM_218291		-0.06	-1.149	0.450074
A_44_P338773	BF413197	BF413197	300207	-0.06	-1.149	0.348031
A_44_P266350	BQ196681	BQ196681	312358	-0.06	-1.149	0.376841
A_42_P600796	Ccdc53_predicted	XM_216873		-0.06	-1.149	0.398909
A_44_P235767	BG372727	BG372727	499619	-0.06	-1.149	0.473392
A_44_P235521	AA899102	AA899102	24377	-0.06	-1.149	0.370769
A_44_P520881	RGD1562673_predicted	XM_343866		-0.06	-1.149	0.50753
A_44_P807424	A_44_P807424	A_44_P807424		-0.06	-1.150	0.458194
A_44_P128101	Olr1111_predicted	NM_001000425	300205	-0.06	-1.150	0.308044
A_44_P371798	AI136559	AI136559	24686	-0.06	-1.150	0.254933
A_42_P540177	Dnajc17_predicted	XM_230468	311329	-0.06	-1.150	0.185131
A_42_P648652	Clca6	NM_201419	362053	-0.06	-1.150	0.417635
A_43_P13025	Gas6	NM_057100	58935	-0.06	-1.150	0.421543
A_43_P21661	Fign_predicted	XM_229979		-0.06	-1.150	0.301967
A_42_P538617	Slb	NM_053792	116475	-0.06	-1.150	0.335077
A_44_P311455	Nrg2	XM_344662	432361	-0.06	-1.150	0.19208
A_44_P1014700	LOC678914	XM_001053407		-0.06	-1.150	0.374735
A_44_P276120	Kiss1r	NM_023992	78976	-0.06	-1.150	0.39137
A_43_P17769	Stard3nl	NM_001008298	291182	-0.06	-1.150	0.248138
A_44_P242904	Grik3	NM_181373	298521	-0.06	-1.150	0.419611
A_44_P870848	BF544498	BF544498		-0.06	-1.151	0.412698
A_44_P920519	TC558142	TC558142		-0.06	-1.151	0.537868
A_44_P308973	XM_224147	XM_224147		-0.06	-1.151	0.31288
A_42_P576621	RGD1310905_predicted	XM_215797		-0.06	-1.151	0.210736
A_44_P203878	LOC691932	XM_001080162	691932	-0.06	-1.151	0.540869
A_44_P224436	Olr1350_predicted	NM_001000752	405022	-0.06	-1.151	0.409468
A_44_P895399	AW916109	AW916109		-0.06	-1.151	0.361365
A_44_P454838	XM_226100	XM_226100		-0.06	-1.151	0.267024
A_44_P513333	Lgals12_predicted	XM_219545		-0.06	-1.151	0.369579
A_44_P100660	ENSRNOT00000043103	ENSRNOT00000043103		-0.06	-1.151	0.304642
A_44_P512696	BM986545	BM986545	25365	-0.06	-1.151	0.238298
A_42_P677866	Csn2	NM_017120	29173	-0.06	-1.151	0.231648
A_44_P524765	Olr1491_predicted	NM_001000717	404969	-0.06	-1.151	0.450158
A_44_P586190	AA819112	AA819112		-0.06	-1.151	0.294981
A_44_P187352	LOC308398	XM_218397		-0.06	-1.152	0.299579
A_44_P540908	Htr3a	NM_024394	79246	-0.06	-1.152	0.41204
A_44_P206244	Tpst2	NM_001008508	288719	-0.06	-1.152	0.255892
A_44_P133182	ENSRNOT00000019955	ENSRNOT00000019955		-0.06	-1.152	0.581406
A_44_P316490	RGD1311612_predicted	XM_001076059		-0.06	-1.152	0.312286
A_44_P1057955	Cd164	NM_031812	83689	-0.06	-1.152	0.166165
A_44_P523401	AA956504	AA956504		-0.06	-1.152	0.516049
A_44_P119296	Ggps1	NM_001007626	291211	-0.06	-1.152	0.404032
A_43_P10559	BF550405	BF550405	501095	-0.06	-1.152	0.438129
A_44_P832655	LOC687118	XM_001077155		-0.06	-1.152	0.242823
A_43_P16962	LOC301128	BC098860	301128	-0.06	-1.152	0.404442
A_43_P16630	RGD1560708_predicted	XM_342341		-0.06	-1.152	0.473145
A_44_P148252	AI231547	AI231547	260321	-0.06	-1.152	0.46558
A_44_P304727	Gm672_predicted	XM_344701		-0.06	-1.153	0.170715
A_44_P324164	LOC684984	XM_001061779	684984	-0.06	-1.153	0.415645
A_44_P513866	XM_213656	XM_213656		-0.06	-1.153	0.405347
A_43_P20515	RGD1309519_predicted	XM_342213	361917	-0.06	-1.153	0.226336
A_44_P116471	Lgals8	NM_053862	116641	-0.06	-1.153	0.28305

A_44_P515754	XM_346943	XM_346943		-0.06	-1.153	0.469558
A_43_P20972	RGD1563429_predicted	XM_223154		-0.06	-1.153	0.16123
A_44_P821966	LOC500684	XM_001060736		-0.06	-1.153	0.242227
A_44_P156741	LOC361014	NM_001014136	361014	-0.06	-1.153	0.388427
A_44_P1037556	Txndc9	NM_172032	280671	-0.06	-1.153	0.360806
A_44_P442966	Vps37b_predicted	XM_213769		-0.06	-1.153	0.193043
A_43_P10923	Scamp2	NM_023955	65168	-0.06	-1.153	0.268694
A_44_P107266	AY387058	AY387058		-0.06	-1.153	0.211505
A_44_P533533	AI228230	AI228230	313668	-0.06	-1.153	0.258366
A_44_P451132	AI013900	AI013900	292762	-0.06	-1.153	0.372594
A_44_P484320	BE103471	BE103471		-0.06	-1.153	0.356417
A_44_P321488	XM_216537	XM_216537		-0.06	-1.153	0.219772
A_44_P246030	RGD1562686_predicted	XM_226533	307913	-0.06	-1.153	0.180151
A_43_P15220	Celsr2	XM_001070611		-0.06	-1.153	0.501498
A_44_P592973	TC560542	TC560542		-0.06	-1.153	0.532685
A_44_P282716	Nit1	NM_182668	289222	-0.06	-1.153	0.207137
A_44_P151179	Myl2	CO563307	24584	-0.06	-1.153	0.365662
A_44_P480490	Anxa11	NM_001011918	290527	-0.06	-1.153	0.306789
A_42_P652586	Stat5a	NM_017064	24918	-0.06	-1.154	0.304111
A_44_P259051	LOC652955	NM_001037658	652955	-0.06	-1.154	0.527353
A_44_P515768	RGD1305727_predicted	XM_225176		-0.06	-1.154	0.29216
A_44_P349094	L20988	L20988		-0.06	-1.154	0.653764
A_44_P342372	Hapln4_predicted	XM_341416		-0.06	-1.154	0.527456
A_44_P357703	BE109108	BE109108	305471	-0.06	-1.154	0.279603
A_44_P296570	BI286851	BI286851		-0.06	-1.154	0.356013
A_43_P18283	RGD1565757_predicted	XM_001065952		-0.06	-1.154	0.206402
A_44_P944912	TC519871	TC519871		-0.06	-1.154	0.224483
A_42_P460758	BF412134	BF412134	680045	-0.06	-1.154	0.498742
A_44_P435158	AW917664	AW917664		-0.06	-1.154	0.258334
A_44_P447907	Cgn_predicted	XM_227472	310655	-0.06	-1.155	0.412067
A_44_P214687	MGC109455	NM_001024271	360967	-0.06	-1.155	0.276034
A_44_P1032362	Numb	NM_133287	29419	-0.06	-1.155	0.263234
A_44_P195816	BM986566	BM986566	64302	-0.06	-1.155	0.537269
A_44_P314702	Slc13a1	NM_031651	58980	-0.06	-1.155	0.558699
A_43_P15943	Sos2	XM_001080400		-0.06	-1.155	0.392949
A_44_P295042	Fosb	XM_001057199	308411	-0.06	-1.155	0.229092
A_44_P208596	Car7_predicted	XM_001056926		-0.06	-1.155	0.393938
A_44_P257977	LOC296255	XR_007796	296255	-0.06	-1.155	0.607352
A_44_P181050	RGD1565775	XM_342277	361980	-0.06	-1.155	0.313806
A_44_P311762	Galnt3	NM_001015032	366061	-0.06	-1.155	0.430273
A_44_P675887	AW920694	AW920694		-0.06	-1.155	0.323483
A_42_P490305	AI013671	AI013671		-0.06	-1.155	0.47946
A_44_P539231	RGD1560794_predicted	XR_007933	299227	-0.06	-1.155	0.231274
A_44_P1042668	Hint3	XM_341742	246769	-0.06	-1.155	0.485729
A_44_P130665	RGD1311314	XM_221964		-0.06	-1.155	0.525088
A_44_P197026	LOC313535	XM_233435	313535	-0.06	-1.155	0.246267
A_44_P536841	A_44_P536841	A_44_P536841		-0.06	-1.156	0.41139
A_44_P355861	Cd2ap	NM_181475	316258	-0.06	-1.156	0.294302
A_44_P296473	AI013718	AI013718	25632	-0.06	-1.156	0.37543
A_44_P313661	RT1-CE12	NM_001008835	24737	-0.06	-1.156	0.426502
A_44_P329756	AI059074	AI059074		-0.06	-1.156	0.244319
A_44_P547592	BI289521	BI289521	116464	-0.06	-1.156	0.417261
A_43_P20288	CB548450	CB548450		-0.06	-1.156	0.273345
A_44_P253450	LOC497892	XM_573076		-0.06	-1.156	0.338599
A_43_P17837	Sesn1_predicted	XM_215423		-0.06	-1.156	0.250813
A_44_P316584	A_44_P316584	A_44_P316584		-0.06	-1.156	0.409522
A_44_P997146	Gapvd1_predicted	XM_231161	311880	-0.06	-1.156	0.434006
A_44_P980732	LOC684090	XM_001058703		-0.06	-1.157	0.30818
A_43_P18918	Acbd5	XM_001053289		-0.06	-1.157	0.29079
A_44_P889646	AW915800	AW915800		-0.06	-1.157	0.295721
A_44_P945050	TC520023	TC520023		-0.06	-1.157	0.227701
A_44_P212964	Plac8_predicted	XM_341188		-0.06	-1.157	0.37756
A_44_P482543	L20996	L20996		-0.06	-1.157	0.359018

A_44_P550765	Spfh2_predicted	XM_214372		-0.06	-1.157	0.13424
A_44_P1021844	XM_233139	XM_233139		-0.06	-1.157	0.472142
A_44_P434030	XM_345817	XM_345817		-0.06	-1.157	0.310123
A_44_P902742	Als2cr13_predicted	XM_343576		-0.06	-1.157	0.37656
A_44_P230697	DV717733	DV717733	363081	-0.06	-1.157	0.495584
A_44_P152517	CA510281	CA510281		-0.06	-1.157	0.454751
A_44_P368450	A_44_P368450	A_44_P368450		-0.06	-1.157	0.451855
A_44_P276504	A_44_P276504	A_44_P276504		-0.06	-1.157	0.607174
A_44_P133123	XM_342014	XM_342014		-0.06	-1.157	0.402981
A_44_P157721	Y00054	Y00054		-0.06	-1.157	0.480581
A_44_P1043943	Ankrd13d_predicted	XM_341982		-0.06	-1.158	0.355963
A_44_P294437	Tbce	NM_001012161	361255	-0.06	-1.158	0.491695
A_43_P10051	Saa4	NM_001009478	365245	-0.06	-1.158	0.226732
A_42_P580733	Dlst	NM_001006981	299201	-0.06	-1.158	0.1648
A_44_P245642	Sftpb	NM_138842	192155	-0.06	-1.158	0.315776
A_43_P13267	Rab3il1	NM_134411	171452	-0.06	-1.158	0.326016
A_44_P214288	Cabp7	NM_001007730	360970	-0.06	-1.158	0.254571
A_44_P255905	Tnk1_predicted	XM_220614		-0.06	-1.158	0.330998
A_44_P266568	RGD1307161	NM_001025669	305031	-0.06	-1.158	0.405228
A_44_P201139	AF196250	AF196250		-0.06	-1.158	0.371643
A_43_P19019	RGD1308795_predicted	XM_236392	315804	-0.06	-1.158	0.290023
A_44_P942715	Prlpc1	NM_020079	24658	-0.06	-1.158	0.334808
A_44_P618099	XM_221276	XM_221276		-0.06	-1.158	0.236715
A_44_P406732	AA899852	AA899852	29143	-0.06	-1.158	0.260743
A_44_P370426	Fbxo8	NM_001012050	306436	-0.06	-1.158	0.355387
A_44_P1054631	LOC681138	XM_001060452		-0.06	-1.158	0.272891
A_43_P10482	BF556670	BF556670		-0.06	-1.158	0.384056
A_44_P751956	AI599448	AI599448		-0.06	-1.158	0.622064
A_44_P533959	Scarb2	NM_054001	117106	-0.06	-1.158	0.346437
A_44_P438442	LOC684187	XM_001067748		-0.06	-1.159	0.195862
A_42_P712801	Entpd3	NM_178106	316077	-0.06	-1.159	0.39543
A_44_P493045	AA800604	AA800604	498256	-0.06	-1.159	0.361903
A_44_P374708	Cry1	NM_198750	299691	-0.06	-1.159	0.562919
A_44_P463805	Cd3z	NM_170789	25300	-0.06	-1.159	0.5315
A_44_P777990	TC543764	TC543764		-0.06	-1.159	0.564974
A_44_P825558	Ranbp10_predicted	XM_341676	361396	-0.06	-1.159	0.136423
A_44_P121092	Pde8a	NM_198767	308776	-0.06	-1.159	0.602764
A_44_P335377	Fbxw5	NM_001025730	362081	-0.06	-1.159	0.351363
A_44_P345553	BI301467	BI301467	363009	-0.06	-1.159	0.493659
A_43_P11702	Acadsb	NM_013084	25618	-0.06	-1.159	0.313353
A_44_P425150	Gp2	NM_134418	171459	-0.06	-1.159	0.212555
A_42_P603670	XM_218706	XM_218706		-0.06	-1.159	0.211412
A_44_P294475	BP485269	BP485269		-0.06	-1.159	0.236381
A_44_P256534	AW918699	AW918699		-0.06	-1.159	0.375273
A_43_P20098	Dock2	XM_001068649	360509	-0.06	-1.159	0.397053
A_44_P885652	RGD1565881_predicted	XM_573132		-0.06	-1.159	0.36583
A_44_P914911	Kiaa0415	NM_001037220	641386	-0.06	-1.160	0.215828
A_44_P105706	A_44_P105706	A_44_P105706		-0.06	-1.160	0.193602
A_44_P837070	ENSRNOT00000041792	ENSRNOT00000041792		-0.06	-1.160	0.268473
A_42_P621832	RGD1306636_predicted	XM_215083	293498	-0.06	-1.160	0.506978
A_44_P248352	RGD1564319_predicted	XM_345378		-0.06	-1.160	0.303394
A_44_P997962	RGD1303117	NM_001004219	292764	-0.06	-1.160	0.25632
A_43_P23051	LOC500419	NM_001031663	500419	-0.06	-1.160	0.466328
A_44_P220378	LOC685601	XM_001064490	685601	-0.06	-1.160	0.333266
A_42_P734649	RGD1309472	NM_001013969	303333	-0.06	-1.160	0.355585
A_44_P478711	Rffl	NM_001004068	282844	-0.06	-1.160	0.231731
A_44_P555638	LOC317380	NM_001014110	317380	-0.06	-1.161	0.142685
A_43_P10470	LOC299907	XM_216920	299907	-0.06	-1.161	0.362597
A_44_P763752	TC566471	TC566471		-0.06	-1.161	0.575169
A_42_P717308	Thap6_predicted	XM_223245		-0.06	-1.161	0.349207
A_44_P159259	Mypn_predicted	XM_228149		-0.06	-1.161	0.177677
A_44_P258017	Parp12_predicted	XM_342663		-0.06	-1.161	0.253606
A_43_P20007	Spnb4	XM_218364	308458	-0.06	-1.161	0.195075

A_44_P110918	Copb2	NM_021765	60384	-0.06	-1.161	0.358648
A_43_P23237	Sim2_predicted	XM_001055265		-0.06	-1.161	0.451439
A_44_P493924	Man2b1	NM_199404	361378	-0.06	-1.161	0.314963
A_44_P421391	Slc1a4	NM_198763	305540	-0.06	-1.161	0.251762
A_43_P20126	LOC684611	XM_001071235		-0.06	-1.161	0.362024
A_44_P176886	AI013050	AI013050	364712	-0.06	-1.161	0.242173
A_42_P655706	Ndufb4	NM_001037338	288088	-0.06	-1.161	0.260238
A_44_P655158	CO567276	CO567276	301056	-0.06	-1.161	0.412596
A_44_P284054	RGD1308907_predicted	XM_234425	314325	-0.06	-1.161	0.129725
A_44_P173935	Sdccag1	XM_216724		-0.07	-1.161	0.402507
A_44_P227174	Sh2d4a	NM_001012048	306376	-0.07	-1.162	0.157268
A_44_P319364	AI235590	AI235590	366262	-0.07	-1.162	0.159801
A_44_P605797	ENSRNOT00000029715	ENSRNOT00000029715		-0.07	-1.162	0.341683
A_44_P509944	AI012539	AI012539	295602	-0.07	-1.162	0.512745
A_44_P156904	RGD1308023_predicted	XM_001055483		-0.07	-1.162	0.354796
A_44_P947342	TC556001	TC556001		-0.07	-1.162	0.228958
A_44_P154850	BQ781947	BQ781947	287982	-0.07	-1.162	0.429195
A_43_P11343	AW142634	AW142634		-0.07	-1.162	0.484938
A_44_P301608	Ucp2	NM_019354	54315	-0.07	-1.162	0.398653
A_44_P255164	Centg1	XM_576238	65218	-0.07	-1.163	0.267688
A_44_P455283	BQ211607	BQ211607	315327	-0.07	-1.163	0.228724
A_44_P1040601	Mkln1	NM_031359	83536	-0.07	-1.163	0.19588
A_44_P415284	AA900501	AA900501	498030	-0.07	-1.163	0.424202
A_44_P370868	A_44_P370868	A_44_P370868		-0.07	-1.163	0.26219
A_44_P178475	Asb1_predicted	XM_237397		-0.07	-1.163	0.454813
A_44_P313898	A_44_P313898	A_44_P313898		-0.07	-1.163	0.262233
A_43_P12612	Kcnj3	NM_031610	50599	-0.07	-1.163	0.278872
A_43_P15794	Wbp4	NM_053766	114765	-0.07	-1.163	0.190266
A_43_P10157	Spire1_predicted	XM_001061383		-0.07	-1.163	0.496374
A_44_P647689	BG376442	BG376442		-0.07	-1.163	0.279705
A_43_P11261	RGD1309696_predicted	XM_001072924		-0.07	-1.163	0.55744
A_43_P13946	BM390804	BM390804	501515	-0.07	-1.163	0.214025
A_44_P212521	Gzmg	NM_153466	266704	-0.07	-1.163	0.223313
A_44_P390495	Gch	NM_024356	29244	-0.07	-1.163	0.491405
A_44_P197193	Gpr35	NM_001037359	367315	-0.07	-1.163	0.217587
A_43_P14432	TC520838	TC520838		-0.07	-1.164	0.180244
A_44_P307335	AA899122	AA899122		-0.07	-1.164	0.334541
A_44_P103457	CB548192	CB548192	316663	-0.07	-1.164	0.378812
A_44_P434109	Hes7_predicted	XM_220597		-0.07	-1.164	0.482327
A_43_P17937	RGD1306625_predicted	XM_340782		-0.07	-1.164	0.181743
A_44_P387290	Ptk9	NM_001008521	315265	-0.07	-1.164	0.224285
A_44_P443045	XM_344250	XM_344250		-0.07	-1.164	0.591992
A_43_P13127	Slc38a4	NM_130748	170573	-0.07	-1.164	0.77059
A_44_P314506	XM_221348	XM_221348		-0.07	-1.164	0.396442
A_43_P15003	TC519698	TC519698		-0.07	-1.164	0.352879
A_44_P199889	AA901214	AA901214		-0.07	-1.164	0.368803
A_44_P367926	XM_213990	XM_213990		-0.07	-1.164	0.357048
A_44_P356468	XM_344580	XM_344580		-0.07	-1.164	0.425898
A_43_P12096	Tmem33	NM_021671	59303	-0.07	-1.164	0.189454
A_44_P403766	CB545947	CB545947		-0.07	-1.164	0.359418
A_42_P799159	LOC681987	XM_001059213		-0.07	-1.164	0.322947
A_44_P927840	CX570298	CX570298	294324	-0.07	-1.165	0.246688
A_44_P441074	A_44_P441074	A_44_P441074		-0.07	-1.165	0.230914
A_44_P372544	Dab2ip	NM_138710	192126	-0.07	-1.165	0.263268
A_44_P354420	A_44_P354420	A_44_P354420		-0.07	-1.165	0.352303
A_44_P853145	TC535274	TC535274		-0.07	-1.165	0.281051
A_44_P110409	RGD1307151_predicted	XM_237212	316455	-0.07	-1.165	0.13739
A_44_P278688	Yme1l1	NM_053682	114217	-0.07	-1.165	0.257744
A_42_P726994	LOC498178	XM_573394	498178	-0.07	-1.165	0.201069
A_44_P640364	TC545427	TC545427		-0.07	-1.165	0.383384
A_44_P370715	LOC691715	XR_007182	691715	-0.07	-1.165	0.16599
A_44_P592248	LOC498185	NM_001017488	498185	-0.07	-1.165	0.335157
A_44_P241848	BF408129	BF408129		-0.07	-1.166	0.485357

A_44_P215646	LOC317274	NM_001025013	317274	-0.07	-1.166	0.316624
A_44_P823094	Lrrk1_predicted	XM_218760	308703	-0.07	-1.166	0.288401
A_42_P784931	Dopey2_predicted	XM_221640	304077	-0.07	-1.166	0.440301
A_43_P12665	Mmp24	NM_031757	83513	-0.07	-1.166	0.214594
A_44_P111575	Tank	NM_145788	252961	-0.07	-1.166	0.193811
A_44_P554486	AA850579	AA850579	362404	-0.07	-1.166	0.343946
A_44_P526713	AA945698	AA945698	113922	-0.07	-1.166	0.222897
A_44_P1011008	Atp6ap2	XM_217592	302526	-0.07	-1.166	0.358145
A_44_P506662	Trerf1_predicted	XM_236953		-0.07	-1.166	0.288667
A_44_P462285	Inpp5e	XM_342391	25695	-0.07	-1.166	0.147647
A_44_P121853	LOC313045	XM_232757	313045	-0.07	-1.166	0.293968
A_42_P610733	RGD1565884_predicted	XM_223941		-0.07	-1.166	0.322621
A_43_P11765	Casr	NM_016996	24247	-0.07	-1.166	0.393613
A_44_P132825	Cdh17	NM_053977	117048	-0.07	-1.167	0.59632
A_44_P137160	AA901137	AA901137	85492	-0.07	-1.167	0.402644
A_44_P747024	TC526537	TC526537		-0.07	-1.167	0.475531
A_42_P797910	LOC362414	NM_001025734	362414	-0.07	-1.167	0.115374
A_44_P476136	Alg1_predicted	XM_001076672		-0.07	-1.167	0.14063
A_44_P434083	Znf174_predicted	XM_220214		-0.07	-1.167	0.371383
A_44_P117955	BQ199946	BQ199946	499782	-0.07	-1.167	0.141616
A_44_P379878	Fbxo34_predicted	XM_223938		-0.07	-1.167	0.149295
A_43_P16662	Fndc3a_predicted	XM_224350		-0.07	-1.167	0.253996
A_44_P229196	Rap2ip	NM_198758	303569	-0.07	-1.167	0.274485
A_44_P354150	RGD1562262_predicted	XM_230784	311573	-0.07	-1.167	0.333187
A_44_P112098	ENSRNOT00000036364	ENSRNOT00000036364		-0.07	-1.167	0.283996
A_44_P228839	Pgrmc2	NM_001008374	361940	-0.07	-1.167	0.224381
A_44_P375613	Narfl	NM_001013183	360496	-0.07	-1.167	0.132322
A_44_P148321	AI233266	AI233266	361538	-0.07	-1.167	0.1873
A_44_P404198	Mapk3	NM_017347	50689	-0.07	-1.167	0.305474
A_44_P293283	XM_234433	XM_234433		-0.07	-1.168	0.188497
A_44_P535758	BF289287	BF289287		-0.07	-1.168	0.21821
A_43_P12795	Grb7	NM_053403	84427	-0.07	-1.168	0.23028
A_44_P792093	TC540214	TC540214		-0.07	-1.168	0.378356
A_44_P133409	RGD1311944_predicted	XM_342762		-0.07	-1.168	0.337795
A_43_P21913	Usp53_predicted	XM_215700		-0.07	-1.168	0.362392
A_44_P790155	AW142505	AW142505	301082	-0.07	-1.168	0.275156
A_44_P492165	XM_224155	XM_224155		-0.07	-1.168	0.524826
A_44_P196607	Arhgef18_predicted	XM_221775		-0.07	-1.168	0.238994
A_44_P121989	Fbxl17_predicted	XM_237458		-0.07	-1.168	0.123476
A_44_P880842	CV795401	CV795401		-0.07	-1.168	0.338702
A_44_P226919	Uqcrfs1	NM_001008888	291103	-0.07	-1.168	0.160184
A_43_P17344	Bcl2l13_predicted	XM_232252		-0.07	-1.168	0.173324
A_42_P699187	Ak2	NM_030986	24184	-0.07	-1.169	0.306573
A_44_P466267	XM_214882	XM_214882		-0.07	-1.169	0.447124
A_44_P201117	Fndc5	XM_001060505		-0.07	-1.169	0.471082
A_44_P301875	Wdr44	XM_343761	246152	-0.07	-1.169	0.364543
A_43_P11172	Fis1	XM_213746		-0.07	-1.169	0.291181
A_44_P168594	Fbxo18_predicted	XM_214505		-0.07	-1.169	0.367322
A_42_P728925	Gpi	NM_207592	292804	-0.07	-1.169	0.253606
A_43_P14919	Ppm2c	NM_019372	54705	-0.07	-1.169	0.670514
A_44_P375428	Tex10_predicted	XM_216393		-0.07	-1.169	0.46789
A_44_P140214	RGD1560069_predicted	XM_344446		-0.07	-1.170	0.175138
A_42_P825626	Opn4	NM_138860	192223	-0.07	-1.170	0.123211
A_44_P302353	Il27ra_predicted	XM_222455		-0.07	-1.170	0.312194
A_44_P132346	BE104341	BE104341	316131	-0.07	-1.170	0.289461
A_42_P552439	Cldn14	NM_001013429	304073	-0.07	-1.170	0.303298
A_44_P354573	Tax1bp3	NM_001025419	360564	-0.07	-1.170	0.194585
A_42_P559085	Sos2	XM_001080400		-0.07	-1.170	0.162602
A_43_P12090	Pold1	NM_021662	59294	-0.07	-1.170	0.453283
A_44_P312103	Cul3_predicted	XM_217454		-0.07	-1.170	0.174888
A_44_P183087	Moap1	NM_001013101	299261	-0.07	-1.170	0.334186
A_44_P805073	AI059932	AI059932		-0.07	-1.170	0.320505
A_44_P194198	AF037202	AF037202		-0.07	-1.170	0.350808

A_43_P15779	Hapln2	NM_022285	64057	-0.07	-1.170	0.413165
A_44_P398864	Cog1_predicted	XM_239373		-0.07	-1.170	0.183648
A_43_P13090	Bnip3l	NM_080888	140923	-0.07	-1.170	0.220043
A_44_P553072	A_44_P553072	A_44_P553072		-0.07	-1.170	0.443315
A_44_P140277	RGD1566247_predicted	XM_227823		-0.07	-1.171	0.209557
A_44_P716867	MGC114440	NM_001025772	500566	-0.07	-1.171	0.112839
A_44_P1031483	RGD1565591_predicted	XM_001077382		-0.07	-1.171	0.21055
A_44_P278639	Dmtf1	NM_053693	114485	-0.07	-1.171	0.330944
A_44_P257492	Adam4	NM_020305	57022	-0.07	-1.171	0.180709
A_44_P444375	BI295601	BI295601	688912	-0.07	-1.171	0.507777
A_44_P395977	Ndnl2	XM_219708	309259	-0.07	-1.171	0.16231
A_44_P835758	Srpr	NM_001034150	315548	-0.07	-1.171	0.24565
A_43_P19301	XM_235452	XM_235452		-0.07	-1.171	0.354447
A_43_P20615	RGD1564237_predicted	XM_216955	300027	-0.07	-1.171	0.197082
A_44_P700598	BP483652	BP483652		-0.07	-1.171	0.379543
A_44_P562727	TC527791	TC527791		-0.07	-1.171	0.351453
A_44_P234104	A_44_P234104	A_44_P234104		-0.07	-1.172	0.286946
A_42_P755887	Wbscr14	NM_133552	171078	-0.07	-1.172	0.351355
A_43_P17018	Zfp294	XM_001055727	288308	-0.07	-1.172	0.109178
A_43_P16999	BC060737_predicted	XM_233215		-0.07	-1.172	0.324615
A_44_P235085	BG373221	BG373221	313019	-0.07	-1.172	0.555346
A_44_P239758	AW524502	AW524502		-0.07	-1.172	0.194716
A_44_P821943	ENSRNOT00000046211	ENSRNOT00000046211		-0.07	-1.172	0.354176
A_44_P361603	AA819659	AA819659	314386	-0.07	-1.172	0.654777
A_44_P530637	Gmppa	NM_001025056	501167	-0.07	-1.172	0.236196
A_44_P328397	Rb1	XM_344434	24708	-0.07	-1.172	0.242823
A_44_P914478	A_44_P914478	A_44_P914478		-0.07	-1.172	0.303998
A_44_P429041	RGD1561178_predicted	XM_227654		-0.07	-1.172	0.258095
A_44_P309128	XM_222054	XM_222054		-0.07	-1.173	0.600397
A_44_P883770	TC534432	TC534432		-0.07	-1.173	0.143272
A_42_P719101	Mrpl40	NM_001024865	287962	-0.07	-1.173	0.288521
A_44_P173218	Rapgef2_predicted	XM_227301		-0.07	-1.173	0.290237
A_44_P291071	Rora_predicted	XM_217192		-0.07	-1.173	0.518817
A_44_P553498	XM_340972	XM_340972		-0.07	-1.173	0.217526
A_42_P558349	RGD1308489_predicted	XR_007342	313340	-0.07	-1.173	0.265549
A_44_P748832	Nuak2	NM_001007617	289419	-0.07	-1.173	0.487985
A_44_P387587	Azin1	NM_022585	58961	-0.07	-1.173	0.224274
A_43_P10019	RGD1303003	NM_001004225	294326	-0.07	-1.173	0.141856
A_44_P739689	TC564139	TC564139		-0.07	-1.173	0.167861
A_44_P448126	LOC367415	XM_346131	367415	-0.07	-1.173	0.286602
A_43_P18409	RGD1311593_predicted	XM_223362		-0.07	-1.173	0.306975
A_44_P504163	Ascl4_predicted	XM_235013		-0.07	-1.173	0.317733
A_44_P182601	RGD1561143_predicted	XM_222031	304382	-0.07	-1.173	0.379838
A_44_P857728	TC566732	TC566732		-0.07	-1.174	0.634226
A_44_P902587	RGD1562352_predicted	XM_574072		-0.07	-1.174	0.327193
A_44_P340030	XM_224023	XM_224023		-0.07	-1.174	0.362176
A_44_P122401	Rb1cc1_predicted	XM_232667		-0.07	-1.174	0.359468
A_44_P733106	TC560550	TC560550		-0.07	-1.174	0.559377
A_44_P490460	Tgds_predicted	XM_224518		-0.07	-1.174	0.378683
A_43_P23318	Tspyl5_predicted	XM_234776	314555	-0.07	-1.174	0.250147
A_43_P19672	Stard4_predicted	XM_214592		-0.07	-1.174	0.37326
A_44_P313091	AI008131	AI008131	81640	-0.07	-1.174	0.285563
A_44_P468968	Dicer1	XM_001068155	299284	-0.07	-1.174	0.209689
A_44_P555928	LOC681467	XM_001057746		-0.07	-1.174	0.256196
A_42_P553342	RGD1565055_predicted	XM_343266	362938	-0.07	-1.174	0.244854
A_44_P452907	RGD1566232_predicted	XM_343950		-0.07	-1.174	0.270344
A_44_P250229	LOC685766	XM_001081954	287750	-0.07	-1.174	0.406933
A_44_P348334	AA997362	AA997362	170916	-0.07	-1.174	0.190376
A_44_P832194	TC531872	TC531872		-0.07	-1.174	0.23237
A_44_P1025903	Vps33b	NM_022286	64060	-0.07	-1.174	0.242564
A_44_P999507	Pkp2	XM_213560	287925	-0.07	-1.174	0.182102
A_44_P284810	AA964050	AA964050	114089	-0.07	-1.174	0.356406
A_43_P19173	Sdccag1	XM_216724		-0.07	-1.175	0.204169

A_44_P513524	LOC682690	XM_001062696		-0.07	-1.175	0.324651
A_42_P540518	Gpr108	NM_199399	316136	-0.07	-1.175	0.20969
A_44_P335724	A_44_P335724	A_44_P335724		-0.07	-1.175	0.534704
A_44_P667206	RGD1563508_predicted	XM_001078729		-0.07	-1.175	0.268693
A_44_P180899	BE120759	BE120759	363273	-0.07	-1.175	0.216621
A_44_P480439	Kazald1	NM_001033064	293997	-0.07	-1.175	0.101305
A_44_P510974	Olr385_predicted	XM_220088		-0.07	-1.175	0.423919
A_44_P513298	LOC360910	NM_001014133	360997	-0.07	-1.175	0.363331
A_44_P240790	Olr371_predicted	NM_001000258	293803	-0.07	-1.175	0.174614
A_44_P412632	LOC361377	NM_001014152	361377	-0.07	-1.175	0.31812
A_44_P310048	BF548382	BF548382	682097	-0.07	-1.175	0.224576
A_44_P403075	A_44_P403075	A_44_P403075		-0.07	-1.175	0.17198
A_44_P558787	AW918767	AW918767		-0.07	-1.175	0.165494
A_44_P1028282	Mrpl51_predicted	XM_216269		-0.07	-1.175	0.107201
A_42_P770171	RGD1309062	NM_001008367	361501	-0.07	-1.175	0.322519
A_43_P13029	Acsl3	NM_057107	114024	-0.07	-1.175	0.277194
A_44_P994293	Csnk1d	NM_139060	64462	-0.07	-1.175	0.381527
A_44_P136388	BG665613	BG665613		-0.07	-1.175	0.127014
A_44_P452070	Olr142_predicted	NM_001000544	365330	-0.07	-1.176	0.504454
A_44_P474796	AW915356	AW915356	314961	-0.07	-1.176	0.740935
A_43_P20985	Ttll3_predicted	XM_342736		-0.07	-1.176	0.20977
A_44_P470699	Lhx3	XM_001059910	170671	-0.07	-1.176	0.21759
A_44_P917935	TC523076	TC523076		-0.07	-1.176	0.217374
A_44_P402468	Arrb1	NM_012910	25387	-0.07	-1.176	0.161188
A_44_P253393	Tm7sf4_predicted	XM_235262		-0.07	-1.176	0.348741
A_44_P101497	RGD1304572_predicted	XM_001073394		-0.07	-1.176	0.373631
A_44_P128195	Ppp1r1b	NM_138521	360616	-0.07	-1.176	0.443158
A_44_P426843	A_44_P426843	A_44_P426843		-0.07	-1.176	0.447399
A_43_P14795	MGC112844	XM_342152		-0.07	-1.176	0.308183
A_44_P439910	AW144490	AW144490	116639	-0.07	-1.176	0.342997
A_44_P552881	RGD1565800_predicted	XM_222215	304529	-0.07	-1.176	0.378276
A_44_P1000391	Atp8b1_predicted	XM_214553		-0.07	-1.176	0.239262
A_43_P22696	ENSRNOT00000022003	ENSRNOT00000022003		-0.07	-1.177	0.234749
A_44_P384558	LOC683554	XM_001066491		-0.07	-1.177	0.467183
A_44_P1020219	Rundc1_predicted	XM_220992		-0.07	-1.177	0.156282
A_43_P20412	Arsg	XM_221064		-0.07	-1.177	0.314399
A_44_P864520	BI296701	BI296701	500917	-0.07	-1.177	0.248682
A_44_P358460	Ly96	NM_001024279	448830	-0.07	-1.177	0.385783
A_44_P213346	Arl6ip2	XM_216629	298757	-0.07	-1.177	0.283215
A_44_P884766	TC523200	TC523200		-0.07	-1.177	0.20124
A_43_P23046	LOC298442	NM_001025656	298442	-0.07	-1.177	0.159155
A_44_P457421	Cradd_predicted	XM_235061		-0.07	-1.177	0.31314
A_43_P12890	Akap1	NM_053665	114124	-0.07	-1.177	0.321106
A_44_P502262	AA957701	AA957701		-0.07	-1.177	0.583514
A_44_P447894	B3gnt3_predicted	XM_224719		-0.07	-1.177	0.463818
A_43_P19759	Fscn2_predicted	XM_221196		-0.07	-1.178	0.58567
A_44_P724481	BI395560	BI395560		-0.07	-1.178	0.276251
A_43_P17359	Hmgb2l1_predicted	XM_226315		-0.07	-1.178	0.151377
A_44_P498231	BC108295	BC108295	300652	-0.07	-1.178	0.20969
A_44_P262500	Fkrp	NM_001025678	308390	-0.07	-1.178	0.37372
A_44_P293226	LOC301113	XM_217310		-0.07	-1.178	0.403666
A_42_P650386	Alg8	NM_001034127	293129	-0.07	-1.178	0.146295
A_44_P517990	RGD1308541_predicted	XM_218829	308768	-0.07	-1.178	0.313316
A_44_P370281	Herc2_predicted	XM_218720		-0.07	-1.178	0.109219
A_44_P992489	AABR03063179	AABR03063179		-0.07	-1.178	0.190106
A_42_P497153	Zc3h8	NM_001012090	311414	-0.07	-1.178	0.12419
A_44_P299671	Vps4b	NM_001025716	360834	-0.07	-1.178	0.227178
A_42_P819377	MGC105508	NM_001008302	292305	-0.07	-1.178	0.304502
A_44_P421855	RGD1561829_predicted	XM_001068914		-0.07	-1.178	0.366314
A_43_P16118	Celsr2	XM_001070611		-0.07	-1.178	0.555079
A_44_P147156	RGD1359713	NM_001005882	305340	-0.07	-1.179	0.220938
A_43_P14910	Ddc	NM_012545	24311	-0.07	-1.179	0.519339
A_44_P422944	BI285643	BI285643	299195	-0.07	-1.179	0.259668

A_44_P208324	Rab1	NM_031090	81754	-0.07	-1.179	0.208471
A_44_P499634	RGD1306028	XM_001066308		-0.07	-1.179	0.283421
A_43_P18163	RGD1565522_predicted	XM_237794	287564	-0.07	-1.179	0.505321
A_44_P123483	LOC681730	XM_001058187		-0.07	-1.179	0.129616
A_42_P518033	Hsd17b1	NM_012851	25322	-0.07	-1.179	0.14115
A_44_P524437	Pdha2	NM_053994	117098	-0.07	-1.179	0.352879
A_44_P508019	MGC116327	NM_001025761	498183	-0.07	-1.179	0.282019
A_44_P340104	LOC685374	XM_001062165	685374	-0.07	-1.179	0.282525
A_44_P150124	BI294724	BI294724	690585	-0.07	-1.179	0.406397
A_44_P522823	LOC681839	XM_001058665		-0.07	-1.179	0.466323
A_43_P20013	RGD1561371_predicted	XM_342138	361844	-0.07	-1.180	0.35876
A_44_P273449	Perp_predicted	XM_214953		-0.07	-1.180	0.34531
A_44_P196523	RGD1563422_predicted	XM_213922		-0.07	-1.180	0.097934
A_44_P217286	Cacna2d2	NM_175592	300992	-0.07	-1.180	0.252059
A_44_P1040746	Mpp6_predicted	XM_342682	362359	-0.07	-1.180	0.288855
A_44_P715863	Stau2	AY549448	171500	-0.07	-1.180	0.165145
A_44_P166133	XM_224889	XM_224889		-0.07	-1.180	0.210303
A_42_P502271	RGD1311958_predicted	XM_236376		-0.07	-1.180	0.317066
A_44_P1039856	Rock1	NM_031098	81762	-0.07	-1.180	0.191598
A_44_P272491	BF562148	BF562148		-0.07	-1.180	0.130936
A_44_P543879	A_44_P543879	A_44_P543879		-0.07	-1.180	0.113238
A_43_P16143	Per3	NM_023978	78962	-0.07	-1.180	0.152039
A_44_P110063	A_44_P110063	A_44_P110063		-0.07	-1.180	0.248922
A_44_P431236	RGD1310481_predicted	XM_341230		-0.07	-1.180	0.295694
A_44_P644809	TC551328	TC551328		-0.07	-1.180	0.298598
A_42_P568172	Abcc5	NM_053924	116721	-0.07	-1.180	0.263051
A_44_P372601	RGD1309759_predicted	XM_341726	361448	-0.07	-1.180	0.247941
A_44_P382464	RGD1308470	NM_001014198	362778	-0.07	-1.180	0.331447
A_43_P12841	Enpp1	NM_053535	85496	-0.07	-1.181	0.274691
A_44_P241845	AA900893	AA900893		-0.07	-1.181	0.490575
A_44_P446890	BI300764	BI300764	300813	-0.07	-1.181	0.189407
A_44_P500756	Prkcbp1	XM_001071994		-0.07	-1.181	0.355064
A_44_P296155	RT1-A2	NM_001008829	24737	-0.07	-1.181	0.404643
A_44_P172121	AI045568	AI045568		-0.07	-1.181	0.429834
A_44_P351412	Adcy7	AF184150	84420	-0.07	-1.181	0.334322
A_44_P395964	A_44_P395964	A_44_P395964		-0.07	-1.181	0.464423
A_44_P333509	BF544893	BF544893		-0.07	-1.181	0.432976
A_44_P398238	Iqce_predicted	XM_221965	304318	-0.07	-1.181	0.19134
A_44_P386748	Wsb2	NM_001007616	288692	-0.07	-1.181	0.24186
A_44_P126292	Bbs5_predicted	XM_342442		-0.07	-1.181	0.21753
A_44_P372484	U75399	U75399		-0.07	-1.181	0.180388
A_44_P468887	LOC685360	XM_001056772	685360	-0.07	-1.182	0.361365
A_42_P559874	BU759195	BU759195		-0.07	-1.182	0.137707
A_44_P507419	Trak2	NM_133560	171086	-0.07	-1.182	0.255087
A_44_P1057190	XM_216765	XM_216765		-0.07	-1.182	0.413115
A_44_P496994	Snai3_predicted	XM_226544		-0.07	-1.182	0.265931
A_44_P465990	Olr227_predicted	NM_001000203	293370	-0.07	-1.182	0.195083
A_44_P473389	RGD1563437_predicted	XM_214519	291356	-0.07	-1.182	0.271913
A_42_P785419	Cdk2	NM_199501	362817	-0.07	-1.182	0.539575
A_43_P17871	Bin3	NM_001013186	361065	-0.07	-1.182	0.355633
A_44_P139536	Hk1	NM_012734	25058	-0.07	-1.182	0.438422
A_44_P713783	A_44_P713783	A_44_P713783		-0.07	-1.182	0.100588
A_44_P385371	AW252115	AW252115	500246	-0.07	-1.182	0.225554
A_43_P18238	Arfip2	NM_001004222	293344	-0.07	-1.182	0.209673
A_43_P21814	Dcun1d1_predicted	XM_001067883		-0.07	-1.182	0.194188
A_44_P446539	Lsm8_predicted	XM_216102		-0.07	-1.182	0.346882
A_43_P12256	Adcy5	NM_022600	64532	-0.07	-1.182	0.387741
A_44_P327992	AY383708	AY383708	290408	-0.07	-1.182	0.321337
A_44_P449994	LOC365238	XM_001073383		-0.07	-1.182	0.202688
A_43_P12983	Pip5k2a	NM_053926	116723	-0.07	-1.182	0.334943
A_44_P419585	RGD1559615_predicted	XM_225620		-0.07	-1.182	0.547431
A_42_P558378	Unc5a	NM_022206	60629	-0.07	-1.183	0.253813
A_42_P545466	Chchd3_predicted	XM_238346		-0.07	-1.183	0.163704

A_44_P127933	AI715140	AI715140	690148	-0.07	-1.183	0.363054
A_43_P19167	Herc4	NM_001012074	309758	-0.07	-1.183	0.233292
A_44_P230370	Defa	NM_173329	286995	-0.07	-1.183	0.395379
A_44_P885583	TC524765	TC524765		-0.07	-1.183	0.337798
A_44_P461518	Mapk6	NM_031622	58840	-0.07	-1.183	0.216709
A_44_P485271	LOC680047	XM_001055473		-0.07	-1.183	0.461459
A_44_P820879	AI010235	AI010235	362833	-0.07	-1.183	0.297268
A_44_P638225	DV728620	DV728620		-0.07	-1.183	0.60944
A_43_P12976	Efna5	NM_053903	116683	-0.07	-1.183	0.500052
A_44_P715714	TC558300	TC558300		-0.07	-1.183	0.377512
A_44_P342526	RGD1561053_predicted	XM_575355		-0.07	-1.183	0.239944
A_44_P336520	AI235811	AI235811	64310	-0.07	-1.183	0.398044
A_44_P483376	BI286772	BI286772	299204	-0.07	-1.183	0.368595
A_44_P995218	TC555274	TC555274		-0.07	-1.183	0.130415
A_44_P226500	CK600897	CK600897		-0.07	-1.183	0.349813
A_44_P555082	Ddx26	XM_341337		-0.07	-1.184	0.171185
A_44_P852195	LOC682142	XM_001060088		-0.07	-1.184	0.265673
A_43_P12871	Rtn4r	NM_053613	113912	-0.07	-1.184	0.177793
A_44_P433157	Rsn	NM_031745	65201	-0.07	-1.184	0.32684
A_44_P359113	RGD1564060_predicted	XM_236596	315981	-0.07	-1.184	0.210586
A_44_P1036534	Paqr4	NM_001017377	302967	-0.07	-1.184	0.160822
A_44_P681569	AW142619	AW142619	24790	-0.07	-1.184	0.127474
A_42_P581135	RGD1307799	NM_001017454	307833	-0.07	-1.184	0.19209
A_42_P756393	AW920459	AW920459		-0.07	-1.184	0.210298
A_44_P112397	Ncoa6ip_predicted	XM_232648		-0.07	-1.184	0.274072
A_43_P17262	Tmod4_predicted	XM_215641		-0.07	-1.184	0.351983
A_44_P206083	A_44_P206083	A_44_P206083		-0.07	-1.184	0.268336
A_44_P865406	AW144075	AW144075		-0.07	-1.184	0.281933
A_44_P604154	BF416118	BF416118	314386	-0.07	-1.185	0.312701
A_44_P932288	TC566306	TC566306		-0.07	-1.185	0.321337
A_44_P280252	AA800318	AA800318	295703	-0.07	-1.185	0.600422
A_44_P451301	BI276735	BI276735	24571	-0.07	-1.185	0.304813
A_44_P683994	A_44_P683994	A_44_P683994		-0.07	-1.185	0.21505
A_44_P261326	Golt1b_predicted	XM_342782	362460	-0.07	-1.185	0.347852
A_44_P625348	TC518700	TC518700		-0.07	-1.185	0.135296
A_44_P1014420	RICS_predicted	XM_236020	315530	-0.07	-1.185	0.119783
A_44_P149635	RGD1565757_predicted	XM_215517	294862	-0.07	-1.185	0.129067
A_42_P457756	Pkib	NM_012627	24678	-0.07	-1.185	0.343455
A_44_P205783	Hoxb9_predicted	XM_220887		-0.07	-1.185	0.173914
A_44_P869808	LOC360975	NM_001017461	360975	-0.07	-1.185	0.241336
A_44_P140743	AI146186	AI146186		-0.07	-1.185	0.350531
A_44_P291081	Mon2	XM_001054316	314894	-0.07	-1.185	0.352943
A_44_P316122	Prkar2b	XM_343046		-0.07	-1.185	0.284628
A_42_P687186	Sdc4	NM_012649	24771	-0.07	-1.186	0.191511
A_44_P134970	LOC316632	NM_182671	314071	-0.07	-1.186	0.162726
A_44_P267638	RGD1559513_predicted	XM_234443	314337	-0.07	-1.186	0.294484
A_44_P517614	Trak2	NM_133560	171086	-0.07	-1.186	0.100286
A_44_P419987	AA925373	AA925373		-0.07	-1.186	0.32488
A_44_P147356	LOC291545	XM_001065665		-0.07	-1.186	0.152691
A_44_P669909	Dhx35_predicted	XM_342565		-0.07	-1.186	0.206417
A_42_P465408	Sfmbt1	NM_031647	58967	-0.07	-1.186	0.22789
A_44_P775797	ENSRNOT00000033768	ENSRNOT00000033768		-0.07	-1.186	0.191458
A_44_P286773	LOC690853	XM_001075880	690853	-0.07	-1.187	0.261268
A_44_P971455	AW918776	AW918776		-0.07	-1.187	0.534661
A_44_P822616	Ep300	XM_576312	170915	-0.07	-1.187	0.12364
A_44_P130175	BQ204899	BQ204899	287442	-0.07	-1.187	0.337753
A_44_P929840	TC554184	TC554184		-0.07	-1.187	0.261169
A_44_P587608	AA893596	AA893596	290270	-0.07	-1.187	0.080751
A_44_P659805	A_44_P659805	A_44_P659805		-0.07	-1.187	0.572459
A_44_P192583	RGD1311447_predicted	XM_343617	363276	-0.07	-1.187	0.268221
A_44_P527123	RGD1562142_predicted	XM_573182	497986	-0.07	-1.187	0.571792
A_44_P389156	RGD1311994_predicted	XM_223228		-0.07	-1.187	0.099158
A_44_P119215	Tbl1xr1_predicted	XM_345195		-0.07	-1.187	0.200792

A_44_P436628	AI176323	AI176323		-0.07	-1.187	0.567306
A_44_P367921	A_44_P367921	A_44_P367921		-0.07	-1.187	0.298887
A_44_P290852	LOC683071	XM_001064337		-0.07	-1.187	0.20715
A_44_P153774	Plce1	NM_053758	114633	-0.07	-1.187	0.177112
A_42_P638620	Lcn2	NM_130741	170496	-0.07	-1.188	0.54694
A_43_P15781	Jak1	XM_001061647		-0.07	-1.188	0.268693
A_44_P466230	Snrk	NM_138833	170837	-0.07	-1.188	0.194906
A_44_P360283	BC081903	BC081903	1E+08	-0.07	-1.188	0.142577
A_43_P21502	RGD1308321_predicted	XM_237855	288354	-0.07	-1.188	0.280757
A_43_P14433	Lims1_predicted	XM_001053446		-0.07	-1.188	0.406689
A_44_P931544	TC561596	TC561596		-0.07	-1.188	0.256494
A_44_P436556	RGD1311357_predicted	XM_001070093		-0.07	-1.188	0.469669
A_44_P423691	Lgals5	NM_012976	25475	-0.07	-1.188	0.367921
A_43_P23451	Carf_predicted	XM_217411		-0.07	-1.188	0.143414
A_44_P452512	A_44_P452512	A_44_P452512		-0.07	-1.188	0.349603
A_43_P16885	Mdm2_predicted	XM_235169		-0.07	-1.188	0.138114
A_44_P318674	Slc22a7	NM_053537	89776	-0.07	-1.188	0.196827
A_44_P867279	AF267750	AF267750	25599	-0.07	-1.188	0.20105
A_44_P870046	TC558796	TC558796		-0.07	-1.188	0.125123
A_44_P590364	ENSRNOT00000027163	ENSRNOT00000027163		-0.07	-1.188	0.239007
A_44_P557545	Rnf139_predicted	XM_235338	315000	-0.07	-1.188	0.280757
A_44_P543797	Phtf2_predicted	XM_238334		-0.08	-1.189	0.314025
A_44_P997994	Zfp96	NM_153475		-0.08	-1.189	0.195444
A_44_P288260	RGD1562376_predicted	XM_574333	361485	-0.08	-1.189	0.129153
A_44_P419565	RGD1310887_predicted	XM_231128		-0.08	-1.189	0.221283
A_43_P20937	Tmprss6_predicted	XM_235768	315388	-0.08	-1.189	0.134217
A_44_P218723	CA509996	CA509996	292148	-0.08	-1.189	0.299083
A_43_P22141	RGD1561201_predicted	XM_228556		-0.08	-1.189	0.48836
A_44_P195648	RGD1304890	XM_001057949		-0.08	-1.189	0.219901
A_44_P114298	Hiat1_predicted	XM_215693		-0.08	-1.189	0.220978
A_44_P165999	Cabc1	NM_001013185	360887	-0.08	-1.189	0.284266
A_44_P686192	TC564381	TC564381		-0.08	-1.189	0.696333
A_44_P178338	RGD1304675_predicted	XM_233728	313756	-0.08	-1.189	0.102971
A_44_P199624	Bad	NM_022698	64639	-0.08	-1.189	0.264449
A_44_P484876	M61004	M61004		-0.08	-1.190	0.210847
A_44_P998060	Prei3	NM_133528	171050	-0.08	-1.190	0.364971
A_44_P483358	BG666558	BG666558		-0.08	-1.190	0.181059
A_44_P440517	RT1-CE4	NM_001008842	414783	-0.08	-1.190	0.312359
A_44_P992011	AA850550	AA850550	301442	-0.08	-1.190	0.244012
A_43_P20745	Siae_predicted	XM_343373		-0.08	-1.190	0.13493
A_44_P363410	Ppfia1_predicted	XM_238162		-0.08	-1.190	0.141129
A_42_P678904	RGD1563547_predicted	XM_346881		-0.08	-1.190	0.385738
A_43_P17714	Usp20_predicted	XM_231148		-0.08	-1.190	0.372904
A_44_P415107	XM_233669	XM_233669		-0.08	-1.191	0.164471
A_44_P186702	AI237378	AI237378	685117	-0.08	-1.191	0.324893
A_44_P344181	Mtap6	NM_017204	29457	-0.08	-1.191	0.353804
A_44_P1003969	RGD1565589_predicted	XM_001062709		-0.08	-1.191	0.141998
A_44_P337592	A_44_P337592	A_44_P337592		-0.08	-1.191	0.243425
A_44_P536370	Msx3	XM_001058715	114504	-0.08	-1.191	0.283581
A_44_P282605	Ezh1_predicted	XM_001081456		-0.08	-1.191	0.225907
A_42_P557394	Isg20	NM_001008510	293052	-0.08	-1.191	0.336601
A_43_P23054	Nt5m_predicted	XM_213318		-0.08	-1.191	0.199938
A_44_P506852	Arhgef1	AF314539	60323	-0.08	-1.191	0.194698
A_43_P11943	Tnfrsf8	NM_019135	25069	-0.08	-1.191	0.260959
A_44_P1016674	Pde9a	NM_138543	191569	-0.08	-1.191	0.319591
A_44_P527530	A_44_P527530	A_44_P527530		-0.08	-1.191	0.302212
A_44_P222458	LOC498411	XM_573658		-0.08	-1.192	0.312346
A_44_P393653	XM_222561	XM_222561		-0.08	-1.192	0.214258
A_44_P515809	Zfp521_predicted	XM_226153		-0.08	-1.192	0.4412
A_44_P1012556	RGD1311800	NM_001013988	305234	-0.08	-1.192	0.171172
A_44_P635853	BG671506	BG671506		-0.08	-1.192	0.134016
A_44_P455145	Plxnb2	XM_243652		-0.08	-1.192	0.516674
A_44_P350843	RGD1306508_predicted	XM_214121		-0.08	-1.192	0.160025

A_44_P482785	Znf511_predicted	XM_215107		-0.08	-1.192	0.185548
A_44_P285874	Ppp1r12a	U50185	116670	-0.08	-1.192	0.25812
A_42_P583113	Adra2c	NM_138506	24175	-0.08	-1.192	0.382579
A_44_P837619	Ctnnd1_predicted	XM_242062		-0.08	-1.192	0.113401
A_44_P585615	Elf2	NM_001033909	361944	-0.08	-1.192	0.113527
A_43_P19977	Zswim1_predicted	XM_230846	311631	-0.08	-1.192	0.583433
A_44_P470794	Abcg8	NM_130414	155192	-0.08	-1.192	0.170254
A_44_P100457	A_44_P100457	A_44_P100457		-0.08	-1.193	0.33785
A_44_P375793	BF559475	BF559475		-0.08	-1.193	0.206381
A_44_P227267	RGD1310685_predicted	XM_342465	362164	-0.08	-1.193	0.366074
A_44_P358458	M21817	M21817		-0.08	-1.193	0.376802
A_44_P433359	Pde1b	NM_022710	29691	-0.08	-1.193	0.328379
A_44_P548440	RGD1309189_predicted	XM_227591		-0.08	-1.193	0.724619
A_42_P583487	LOC680172	XM_001055992	680172	-0.08	-1.193	0.104928
A_44_P336727	AI010476	AI010476	366957	-0.08	-1.193	0.258385
A_43_P11210	Srpr	NM_001034150	315548	-0.08	-1.193	0.179134
A_44_P740168	BG376818	BG376818		-0.08	-1.194	0.39211
A_44_P839624	TC544106	TC544106		-0.08	-1.194	0.349603
A_44_P386085	AABR03104805	AABR03104805		-0.08	-1.194	0.231961
A_44_P301512	G6pc3	NM_176077	303565	-0.08	-1.194	0.396701
A_44_P246054	LOC685397	XM_001063633		-0.08	-1.194	0.254458
A_44_P716112	TC559362	TC559362		-0.08	-1.194	0.472193
A_43_P11156	XM_216396	XM_216396		-0.08	-1.194	0.091007
A_44_P120740	AA800054	AA800054	81767	-0.08	-1.194	0.379254
A_44_P316619	A_44_P316619	A_44_P316619		-0.08	-1.194	0.209365
A_44_P755912	AW916093	AW916093		-0.08	-1.194	0.352776
A_44_P337647	XM_226613	XM_226613		-0.08	-1.195	0.355011
A_44_P450193	Fibcd1_predicted	XM_231138		-0.08	-1.195	0.515725
A_42_P630710	Mtif2	NM_001004254	305606	-0.08	-1.195	0.138208
A_42_P809694	BF563390	BF563390		-0.08	-1.195	0.365866
A_44_P791941	LOC501925	XM_577349	501925	-0.08	-1.195	0.170946
A_44_P155804	AI072266	AI072266	294734	-0.08	-1.195	0.412747
A_44_P140969	BE108264	BE108264	313262	-0.08	-1.195	0.287896
A_44_P814633	AI145762	AI145762		-0.08	-1.195	0.627088
A_44_P422463	Pcf11_predicted	XM_341883	361605	-0.08	-1.195	0.247708
A_44_P132743	Olr1486_predicted	NM_001000720	404972	-0.08	-1.195	0.501949
A_44_P728452	LOC361325	XM_001062437		-0.08	-1.195	0.20069
A_43_P19369	Prkg1_mapped	XM_219805		-0.08	-1.195	0.126183
A_44_P337675	Zic5_predicted	XM_341380		-0.08	-1.195	0.493647
A_44_P183137	LOC299907	XM_216920	299907	-0.08	-1.195	0.204679
A_43_P19048	LOC362414	NM_001025734	362414	-0.08	-1.195	0.138037
A_44_P459870	RGD1310348_predicted	XM_341010		-0.08	-1.195	0.273488
A_44_P883671	A_44_P883671	A_44_P883671		-0.08	-1.196	0.213392
A_44_P416087	AY053517	AY053517		-0.08	-1.196	0.30657
A_44_P282154	AA858911	AA858911		-0.08	-1.196	0.445324
A_44_P695277	RGD1563276_predicted	XM_001064622		-0.08	-1.196	0.183162
A_44_P185284	XM_345542	XM_345542		-0.08	-1.196	0.462814
A_44_P215917	RGD1563504_predicted	XM_344018	363781	-0.08	-1.196	0.448144
A_44_P742218	AI235676	AI235676	362341	-0.08	-1.196	0.176292
A_44_P248686	RGD1308143	XM_237468		-0.08	-1.196	0.245938
A_44_P645443	BF390882	BF390882	408244	-0.08	-1.196	0.14266
A_44_P264477	Pacsin1	NM_017294	29704	-0.08	-1.197	0.105669
A_42_P767684	Trex1	XM_217284		-0.08	-1.197	0.147568
A_44_P140041	XM_220096	XM_220096		-0.08	-1.197	0.286553
A_44_P559185	AI712679	AI712679	360915	-0.08	-1.197	0.323742
A_43_P15116	BF556336	BF556336		-0.08	-1.197	0.180244
A_43_P20363	Sf4	NM_001011920	290666	-0.08	-1.197	0.080807
A_44_P468117	RGD1564625_predicted	XM_220013	309475	-0.08	-1.197	0.203884
A_44_P382438	RGD1562206_predicted	XM_233884		-0.08	-1.197	0.092512
A_42_P715210	RGD1564709_predicted	XM_223174		-0.08	-1.197	0.19588
A_44_P300849	AA996757	AA996757	290485	-0.08	-1.197	0.309011
A_43_P15387	Guca2b	NM_022284	64055	-0.08	-1.197	0.412914
A_42_P841193	Ddit4l	NM_080399	140582	-0.08	-1.197	0.343936

A_44_P173112	XM_341935	XM_341935		-0.08	-1.197	0.246085
A_44_P323696	LOC361120	NM_001029921	361120	-0.08	-1.197	0.17452
A_43_P23266	Pcdhb21_predicted	XM_001056174		-0.08	-1.197	0.363038
A_44_P450159	Tuft1_predicted	XM_345244		-0.08	-1.197	0.346203
A_44_P332674	Myh13	XM_001078857		-0.08	-1.197	0.462726
A_44_P420927	RGD1307235_predicted	XM_219401	309053	-0.08	-1.197	0.255818
A_44_P353955	LOC679990	XM_574644	679990	-0.08	-1.198	0.166411
A_44_P286570	AI101363	AI101363	293118	-0.08	-1.198	0.183127
A_44_P288629	LOC363498	XM_001053773		-0.08	-1.198	0.595816
A_44_P116532	Cdh22	NM_019161	29182	-0.08	-1.198	0.277865
A_44_P1022637	Strn3	NM_001029897	114520	-0.08	-1.198	0.188122
A_44_P182414	Foxe3	XM_233428		-0.08	-1.198	0.133412
A_44_P325918	A_44_P325918	A_44_P325918		-0.08	-1.198	0.264713
A_44_P122644	LOC498601	AI044806	498601	-0.08	-1.198	0.281089
A_42_P792728	Hit39	NM_173330	286996	-0.08	-1.198	0.162527
A_44_P487942	AI104248	AI104248	294362	-0.08	-1.198	0.283841
A_44_P327988	LOC361230	AY310151	361230	-0.08	-1.198	0.434498
A_43_P18781	LOC362261	NM_001014184	362261	-0.08	-1.198	0.144592
A_44_P271975	RGD1562099_predicted	XM_237949	290325	-0.08	-1.198	0.277335
A_44_P659506	RGD1561105_predicted	XM_001067249		-0.08	-1.198	0.174978
A_44_P172787	LOC362774	NM_001008776	362774	-0.08	-1.198	0.319128
A_44_P670594	TC528756	TC528756		-0.08	-1.198	0.141962
A_43_P11292	XM_216330	XM_216330		-0.08	-1.198	0.117533
A_44_P470862	A_44_P470862	A_44_P470862		-0.08	-1.198	0.185242
A_43_P12312	Crhr2	NM_022714	64680	-0.08	-1.199	0.453991
A_43_P12649	Tmed2	NM_031722	65165	-0.08	-1.199	0.107618
A_43_P17072	Samd8	NM_001012040	305684	-0.08	-1.199	0.138649
A_44_P891653	A_44_P891653	A_44_P891653		-0.08	-1.199	0.111528
A_42_P623151	Dncli2	NM_031026	81655	-0.08	-1.199	0.149517
A_42_P502671	Znrf1_predicted	XM_342692		-0.08	-1.199	0.228057
A_42_P823706	Wdtc1_predicted	XM_001064989		-0.08	-1.199	0.09789
A_43_P15105	BF555945	BF555945		-0.08	-1.199	0.280471
A_44_P159155	RGD1311728_predicted	XM_227688	295449	-0.08	-1.199	0.174176
A_44_P152247	LOC316507	XM_237255		-0.08	-1.199	0.152803
A_44_P413920	AI008190	AI008190	80850	-0.08	-1.199	0.331613
A_44_P126085	Map4k1_predicted	XM_214883		-0.08	-1.199	0.3797
A_43_P16735	Nfe2l1_predicted	XM_340886		-0.08	-1.199	0.147956
A_44_P715742	BC098771	BC098771	315215	-0.08	-1.199	0.385451
A_44_P128629	Gcdh_predicted	XM_344744		-0.08	-1.199	0.234326
A_44_P208363	LOC682343	XM_001061098		-0.08	-1.199	0.216621
A_44_P360563	Grm5	NM_017012	24418	-0.08	-1.200	0.166416
A_43_P19780	RGD1562089_predicted	XM_235493	315145	-0.08	-1.200	0.10477
A_42_P692008	Hoxc6	XM_001069461		-0.08	-1.200	0.170037
A_42_P569832	Rab6b_predicted	XM_343459		-0.08	-1.200	0.277532
A_44_P961361	TC559237	TC559237		-0.08	-1.200	0.422637
A_44_P436284	Ankrd11_predicted	XM_344785		-0.08	-1.200	0.065418
A_44_P251051	Slc16a14_predicted	XM_237359		-0.08	-1.200	0.346982
A_44_P844819	BE106220	BE106220		-0.08	-1.200	0.243867
A_44_P694733	AW921385	AW921385		-0.08	-1.200	0.178454
A_44_P259595	AI169425	AI169425	681418	-0.08	-1.200	0.470068
A_44_P361064	XM_344378	XM_344378		-0.08	-1.200	0.459691
A_44_P1028918	AA996422	AA996422		-0.08	-1.200	0.118306
A_43_P20430	Lats2_predicted	XM_224169		-0.08	-1.201	0.100388
A_44_P132903	Cacna1c	NM_012517	24239	-0.08	-1.201	0.431907
A_44_P463831	Zfp423	NM_053583	94188	-0.08	-1.201	0.514205
A_44_P751504	TC560719	TC560719		-0.08	-1.201	0.155797
A_44_P685693	CA339354	CA339354		-0.08	-1.201	0.180522
A_44_P383676	AA964750	AA964750	171293	-0.08	-1.201	0.151233
A_44_P930160	Pdcd6ip	XM_001076624		-0.08	-1.201	0.319333
A_42_P553885	Synpo	NM_021695	60324	-0.08	-1.201	0.213325
A_44_P502518	AI233766	AI233766	287716	-0.08	-1.201	0.119065
A_44_P307303	ENSRNOT00000034884	ENSRNOT00000034884		-0.08	-1.201	0.434964
A_44_P185003	TC566974	TC566974		-0.08	-1.201	0.248531

A_44_P352252	Gpr26	NM_138841	192153	-0.08	-1.201	0.20559
A_44_P285794	XM_345753	XM_345753		-0.08	-1.201	0.249268
A_44_P191683	Olr192_predicted	NM_001000549	365336	-0.08	-1.201	0.399391
A_44_P188544	AW525656	AW525656	681031	-0.08	-1.201	0.34829
A_42_P587149	LOC679747	XM_001055910	679747	-0.08	-1.201	0.190376
A_42_P839036	RGD1311298_predicted	XM_213532	287840	-0.08	-1.201	0.217308
A_44_P378962	AA924509	AA924509	502374	-0.08	-1.202	0.34076
A_44_P304547	A_44_P304547	A_44_P304547		-0.08	-1.202	0.200489
A_44_P532401	Plek2_predicted	XM_234329		-0.08	-1.202	0.311928
A_42_P598679	Tmem16a_predicted	XM_219695		-0.08	-1.202	0.556181
A_44_P536797	Xkr7	NM_001012092	311549	-0.08	-1.202	0.157066
A_43_P12194	Aldh9a1	NM_022273	64040	-0.08	-1.202	0.292535
A_44_P221980	Cdc2l1	NM_145766	252879	-0.08	-1.202	0.231398
A_44_P549004	BG372060	BG372060		-0.08	-1.202	0.212701
A_44_P548314	RGD1309071	NM_001009970		-0.08	-1.202	0.14063
A_44_P271978	XM_344407	XM_344407		-0.08	-1.202	0.114586
A_44_P525402	RGD1306494_predicted	XM_342698		-0.08	-1.202	0.579094
A_44_P869844	TC525351	TC525351		-0.08	-1.202	0.226939
A_44_P372718	ENSRNOT00000036706	ENSRNOT00000036706		-0.08	-1.203	0.117569
A_44_P264915	RGD1310052_predicted	XM_219477		-0.08	-1.203	0.236702
A_42_P476785	BF556693	BF556693		-0.08	-1.203	0.137735
A_44_P252390	AW917391	AW917391		-0.08	-1.203	0.408621
A_44_P698293	RGD1564720_predicted	XM_001072481		-0.08	-1.203	0.577739
A_44_P604927	AW917657	AW917657		-0.08	-1.203	0.272415
A_44_P184567	Olr1384	NM_001002291	405398	-0.08	-1.203	0.138202
A_44_P491732	Olr20_predicted	NM_001000118	293083	-0.08	-1.203	0.356406
A_44_P370328	Ric8a	XM_215118	293614	-0.08	-1.203	0.177406
A_44_P553316	Col8a2_predicted	XM_233542	313592	-0.08	-1.203	0.406352
A_44_P370015	Pgrmc1	NM_021766	291948	-0.08	-1.203	0.15656
A_43_P15649	Dgat2	NM_001012345	252900	-0.08	-1.203	0.444929
A_44_P763434	RGD1560005_predicted	XM_576579	501153	-0.08	-1.203	0.368903
A_44_P781923	A_44_P781923	A_44_P781923		-0.08	-1.203	0.558568
A_43_P21536	RGD1305534_predicted	XM_236449	315864	-0.08	-1.204	0.165893
A_44_P806145	A_44_P806145	A_44_P806145		-0.08	-1.204	0.140362
A_44_P110582	AA818099	AA818099		-0.08	-1.204	0.142567
A_42_P643698	Cpeb2_predicted	XM_341227		-0.08	-1.204	0.17868
A_44_P200446	AI101475	AI101475	192252	-0.08	-1.204	0.376681
A_43_P21093	LOC681994	XM_001057753		-0.08	-1.204	0.379222
A_44_P429160	XM_342124	XM_342124		-0.08	-1.204	0.200939
A_44_P172140	Hfe	NM_053301	29199	-0.08	-1.204	0.267282
A_42_P600215	Abra	NM_175844	286965	-0.08	-1.204	0.183874
A_44_P842353	TC549357	TC549357		-0.08	-1.204	0.211976
A_43_P22225	Mdga1_predicted	XM_241623		-0.08	-1.204	0.130611
A_44_P278247	LOC309016	XM_219377	309016	-0.08	-1.204	0.159227
A_44_P487049	Olr87_predicted	NM_001000543	365329	-0.08	-1.204	0.417015
A_44_P466547	RGD1311132_predicted	XM_214688		-0.08	-1.205	0.264134
A_44_P606523	A_44_P606523	A_44_P606523		-0.08	-1.205	0.209217
A_44_P315727	AA900371	AA900371		-0.08	-1.205	0.387597
A_44_P545649	Parp16	NM_001014093	315760	-0.08	-1.205	0.109232
A_44_P477954	RGD1561843_predicted	XM_218061		-0.08	-1.205	0.180248
A_44_P233753	Tmem106b	NM_001004267	312132	-0.08	-1.205	0.391608
A_44_P623479	TC558413	TC558413		-0.08	-1.205	0.204097
A_44_P374369	AI600244	AI600244	316526	-0.08	-1.205	0.302066
A_44_P513789	Ccr1l1_predicted	XM_236742		-0.08	-1.205	0.246315
A_44_P129412	Dcx	NM_053379	84394	-0.08	-1.205	0.422415
A_44_P196975	A_44_P196975	A_44_P196975		-0.08	-1.205	0.183362
A_44_P822078	LOC501422	XM_576832		-0.08	-1.205	0.13757
A_44_P550728	Galntl2_predicted	XM_224621		-0.08	-1.206	0.526483
A_43_P20415	Tcfdp2_predicted	XM_217232		-0.08	-1.206	0.496058
A_43_P10650	RGD1566403_predicted	XM_577690	502228	-0.08	-1.206	0.163154
A_43_P11027	AA956634	AA956634		-0.08	-1.206	0.468215
A_44_P1070860	Rab38	AY425759	252916	-0.08	-1.206	0.356406
A_44_P421499	Igf1	NM_178866	24482	-0.08	-1.206	0.529539

A_44_P123877	A_44_P123877	A_44_P123877		-0.08	-1.206	0.25966
A_44_P114892	LOC287625	XR_009457	287625	-0.08	-1.207	0.098907
A_44_P202161	LOC684139	XM_001069100		-0.08	-1.207	0.280309
A_44_P728149	MGC108896	NM_001024304	499689	-0.08	-1.207	0.142126
A_44_P529516	Rtn3	NM_080909	140945	-0.08	-1.207	0.147076
A_44_P154843	BU760281	BU760281	361568	-0.08	-1.207	0.228005
A_44_P461989	Wdr78	NM_001024786	313417	-0.08	-1.207	0.098321
A_43_P18614	TC560425	TC560425		-0.08	-1.207	0.147175
A_44_P828591	Camp	CB577971	316010	-0.08	-1.207	0.255821
A_44_P499231	Nmi	NM_001034148	311021	-0.08	-1.207	0.310893
A_44_P191784	Calm1	NM_031969	24242	-0.08	-1.207	0.230064
A_43_P10705	RGD1309401	NM_001014163	361675	-0.08	-1.208	0.113985
A_44_P345032	Herc1_predicted	XM_236362	315771	-0.08	-1.208	0.185131
A_44_P975634	Tcfeb	NM_001025707	316214	-0.08	-1.208	0.304394
A_44_P248755	AA926192	AA926192		-0.08	-1.208	0.170443
A_44_P519746	BM986570	BM986570	64304	-0.08	-1.208	0.182656
A_44_P218967	AW917600	AW917600		-0.08	-1.208	0.282389
A_42_P716352	Gstm3	NM_031154	81869	-0.08	-1.208	0.565569
A_44_P335415	XM_228411	XM_228411		-0.08	-1.208	0.102357
A_44_P112531	A_44_P112531	A_44_P112531		-0.08	-1.208	0.152175
A_44_P297215	AY539908	AY539908		-0.08	-1.208	0.256393
A_44_P544134	BM392049	BM392049		-0.08	-1.208	0.109981
A_44_P562685	RGD1564163_predicted	XM_575917		-0.08	-1.208	0.141372
A_44_P357870	Cpt1a	NM_031559	25757	-0.08	-1.208	0.156108
A_44_P110400	Aloxe3_predicted	XM_213336		-0.08	-1.208	0.159618
A_44_P426443	Hcrtr1	NM_013064	25593	-0.08	-1.208	0.338595
A_44_P233808	Nf2	XM_341248		-0.08	-1.208	0.210879
A_44_P422948	AW915444	AW915444	500244	-0.08	-1.209	0.261169
A_44_P552838	Prtn3_predicted	NM_001024264	314615	-0.08	-1.209	0.357153
A_44_P146969	Zfp148	NM_031615	58820	-0.08	-1.209	0.177066
A_43_P11426	Pigk	NM_001011953	295543	-0.08	-1.209	0.16801
A_44_P443421	Spata21	NM_001004447	366491	-0.08	-1.209	0.099909
A_44_P401088	RGD1562627_predicted	XM_345846		-0.08	-1.209	0.177162
A_44_P322699	AI411693	AI411693	299357	-0.08	-1.209	0.233064
A_44_P401022	A_44_P401022	A_44_P401022		-0.08	-1.210	0.265476
A_44_P479130	AI145022	AI145022	54238	-0.08	-1.210	0.186431
A_44_P651304	AW142782	AW142782		-0.08	-1.210	0.347224
A_44_P180388	Klhl2_predicted	XM_001073589		-0.08	-1.210	0.425898
A_42_P828102	Ddah2	NM_212532	294239	-0.08	-1.210	0.281719
A_44_P414582	Kcne2	NM_133603	171138	-0.08	-1.210	0.173383
A_44_P559242	LOC688429	XM_001065808		-0.08	-1.210	0.138769
A_44_P364816	AA964725	AA964725	287375	-0.08	-1.210	0.15405
A_44_P363106	Lipe	NM_012859	25330	-0.08	-1.210	0.371643
A_44_P1043059	Spnb2	NM_001013130	305614	-0.08	-1.211	0.21002
A_44_P466517	RGD1562391_predicted	XM_345405		-0.08	-1.211	0.236693
A_44_P422836	Igfbp1	NM_013144	25685	-0.08	-1.211	0.220554
A_44_P998423	Tmed7	XM_001063185		-0.08	-1.211	0.185987
A_42_P568060	Ppp4r2_predicted	XM_216225		-0.08	-1.211	0.371134
A_43_P18075	CB606288	CB606288	308106	-0.08	-1.211	0.229261
A_43_P15597	Steap3	NM_133314	170824	-0.08	-1.211	0.409303
A_44_P435375	AA859970	AA859970	25473	-0.08	-1.211	0.355352
A_44_P320884	Birc4	NM_022231	63879	-0.08	-1.211	0.269805
A_44_P391294	Ighmbp2	NM_031586	29532	-0.08	-1.211	0.141592
A_44_P1006279	Sugt1	XM_214242	290408	-0.08	-1.211	0.110057
A_44_P496327	Prf1	NM_017330	50669	-0.08	-1.211	0.157517
A_44_P441117	Phf13_predicted	XM_233700		-0.08	-1.211	0.07948
A_44_P487189	AF433878	AF433878		-0.08	-1.212	0.272404
A_44_P185504	Rab5c_predicted	XM_213463		-0.08	-1.212	0.136544
A_43_P13546	CB546035	CB546035	85238	-0.08	-1.212	0.215295
A_44_P1007131	Ndufv3l	NM_022607	64539	-0.08	-1.212	0.096848
A_42_P808008	Myl7_predicted	XM_214074		-0.08	-1.212	0.370249
A_44_P438539	A_44_P438539	A_44_P438539		-0.08	-1.212	0.233065
A_42_P541857	Cab39_predicted	XM_217464		-0.08	-1.212	0.122558

A_44_P156343	Olr1645_predicted	NM_001000102	290056	-0.08	-1.212	0.454849
A_44_P754744	A_44_P754744	A_44_P754744		-0.08	-1.212	0.284716
A_44_P533760	Acbd3	NM_182843	289312	-0.08	-1.212	0.18496
A_44_P813453	CA503664	CA503664		-0.08	-1.213	0.236759
A_44_P1050218	Rcor1_predicted	XM_234546		-0.08	-1.213	0.22426
A_44_P638910	TC557610	TC557610		-0.08	-1.213	0.281003
A_43_P23182	RGD1310953	NM_001008366	361416	-0.08	-1.213	0.174162
A_43_P18054	Pigs	NM_001006602	303277	-0.08	-1.213	0.278922
A_44_P1029298	Ppfibp2	XM_001072553		-0.08	-1.213	0.152963
A_44_P153794	Ostf1	NM_148892	259275	-0.08	-1.213	0.226997
A_43_P19554	Zc3h5_predicted	XM_340939		-0.08	-1.213	0.191733
A_44_P441814	AI029437	AI029437		-0.08	-1.213	0.392326
A_44_P279301	Ltf_predicted	XM_236657		-0.08	-1.213	0.415233
A_44_P107573	XM_214046	XM_214046		-0.08	-1.213	0.147385
A_44_P175376	Aldh1b1	NM_001011975	298079	-0.08	-1.213	0.574986
A_43_P17384	RGD1303074	NM_001004230	296304	-0.08	-1.214	0.157241
A_44_P578637	TC526887	TC526887		-0.08	-1.214	0.207255
A_44_P219854	Ncoa3	XM_001072953		-0.08	-1.214	0.123787
A_44_P478125	LOC679180	XM_001055104		-0.08	-1.214	0.11761
A_44_P506597	Igsf9b_predicted	XM_235959		-0.08	-1.214	0.133482
A_42_P612449	Fdxr	NM_024153	79122	-0.08	-1.214	0.245422
A_44_P447066	BQ201045	BQ201045	294520	-0.08	-1.214	0.289461
A_44_P295334	RGD1565465_predicted	XM_575304		-0.08	-1.214	0.125316
A_44_P206166	A_44_P206166	A_44_P206166		-0.08	-1.214	0.147403
A_44_P554066	AI235452	AI235452	360572	-0.08	-1.214	0.177079
A_43_P12909	Ank	NM_053714	114506	-0.08	-1.214	0.132618
A_44_P161702	RGD1305820_predicted	XM_233671		-0.08	-1.214	0.226665
A_44_P321559	LOC300479	XR_007505	300479	-0.08	-1.214	0.331613
A_43_P20418	Zcchc14_predicted	XM_344780	365018	-0.08	-1.215	0.329294
A_44_P864440	AI549088	AI549088		-0.08	-1.215	0.444508
A_44_P251875	AI639532	AI639532	296369	-0.08	-1.215	0.387739
A_44_P548790	LOC299915	XM_235301		-0.08	-1.215	0.195396
A_44_P568805	CO556520	CO556520		-0.08	-1.215	0.135416
A_44_P1070327	A_44_P1070327	A_44_P1070327		-0.08	-1.215	0.108105
A_44_P260673	Tbn_predicted	XM_236948		-0.08	-1.215	0.055016
A_44_P494862	Btf3	NM_001008309	294680	-0.08	-1.215	0.123626
A_44_P241604	Pcaf	NM_001024252	301164	-0.08	-1.215	0.147721
A_44_P544113	RGD1307792_predicted	XM_001060982	303914	-0.08	-1.215	0.199938
A_44_P398299	RGD1310587	XM_341168	360894	-0.08	-1.215	0.354452
A_44_P517713	Olr1369_predicted	NM_001000494	302958	-0.08	-1.215	0.470345
A_44_P434893	LOC685284	XM_001060588	685284	-0.08	-1.215	0.147298
A_44_P557303	Olr575_predicted	NM_001000327	295813	-0.08	-1.215	0.275328
A_44_P128652	RGD1562785_predicted	XM_216085	296836	-0.08	-1.215	0.514205
A_43_P19077	RGD1311784_predicted	XM_213539	287871	-0.08	-1.216	0.179719
A_44_P781967	A_44_P781967	A_44_P781967		-0.08	-1.216	0.328262
A_44_P219664	Acat1	NM_017075	25014	-0.08	-1.216	0.133159
A_43_P21645	Nalp1_predicted	XM_340835	687766	-0.08	-1.216	0.072758
A_42_P730636	TC555648	TC555648		-0.08	-1.216	0.122452
A_43_P15414	Hcn3	NM_053685	114245	-0.08	-1.216	0.363094
A_44_P997737	AA942848	AA942848		-0.08	-1.216	0.149008
A_44_P844029	CB327904	CB327904		-0.08	-1.216	0.206614
A_44_P379301	BF283053	BF283053	362802	-0.08	-1.216	0.114653
A_43_P12069	Mos	NM_020102	24559	-0.08	-1.216	0.141491
A_44_P164117	A_44_P164117	A_44_P164117		-0.08	-1.216	0.249476
A_44_P699625	XM_343152	XM_343152		-0.08	-1.216	0.110439
A_43_P12960	Scg3	NM_053856	116635	-0.09	-1.216	0.415936
A_44_P812456	BC089106	BC089106	1E+08	-0.09	-1.216	0.113086
A_44_P219668	Acat1	NM_017075	25014	-0.09	-1.216	0.263191
A_44_P307241	Parp3	NM_001008328	300985	-0.09	-1.216	0.127294
A_44_P402641	Ugt1a2	NM_201423	113992	-0.09	-1.217	0.273618
A_43_P19673	Lrba_predicted	XM_342271		-0.09	-1.217	0.072467
A_44_P347210	XM_233031	XM_233031		-0.09	-1.217	0.178587
A_44_P359032	Pink1_predicted	XM_216565		-0.09	-1.217	0.33311

A_44_P566856	TC530801	TC530801		-0.09	-1.217	0.164223
A_42_P612592	Saps3_predicted	XM_001067905		-0.09	-1.217	0.262715
A_44_P349320	Mxd4_predicted	XM_341241		-0.09	-1.217	0.146608
A_44_P121429	Lmod1_predicted	XM_222660		-0.09	-1.217	0.244094
A_44_P1047924	Herpud1	NM_053523	85430	-0.09	-1.217	0.270413
A_44_P294857	X14183	X14183		-0.09	-1.217	0.286157
A_44_P100424	RGD1562875_predicted	XM_221920	288489	-0.09	-1.218	0.439945
A_44_P395425	AI070985	AI070985		-0.09	-1.218	0.259966
A_43_P21364	LOC363009	XM_343342		-0.09	-1.218	0.264834
A_44_P356962	Shoc2	NM_001013155	309548	-0.09	-1.218	0.10293
A_42_P619114	RGD1311098	NM_001014204	362998	-0.09	-1.218	0.096569
A_42_P561597	Dnajc10	XM_215751		-0.09	-1.218	0.139701
A_44_P215304	XM_222728	XM_222728		-0.09	-1.218	0.274776
A_44_P527060	Klk10	NM_001004100	292850	-0.09	-1.218	0.502089
A_44_P253782	Csnk1g1	NM_022288	64086	-0.09	-1.218	0.330348
A_44_P149925	RGD1311937_predicted	XM_233470		-0.09	-1.218	0.314399
A_44_P436244	Rttn_predicted	XM_214524	291377	-0.09	-1.219	0.236482
A_44_P129749	Crabp2	NM_017244	29563	-0.09	-1.219	0.440935
A_44_P370294	Za20d3	NM_001007630	293067	-0.09	-1.219	0.11884
A_44_P236167	L20997	L20997		-0.09	-1.219	0.465801
A_44_P280306	BF284850	BF284850	309415	-0.09	-1.219	0.212924
A_44_P519620	Litaf	XM_343856		-0.09	-1.219	0.109274
A_44_P148071	Gyk	NM_024381	79223	-0.09	-1.219	0.447037
A_44_P962319	TC563011	TC563011		-0.09	-1.219	0.144202
A_44_P757007	Bmp1	XM_573814	83470	-0.09	-1.219	0.380384
A_44_P272817	AA957028	AA957028		-0.09	-1.219	0.079339
A_43_P22785	RGD1305413	XM_342199		-0.09	-1.219	0.111819
A_43_P22068	RGD1562189_predicted	XM_221392	288054	-0.09	-1.219	0.20069
A_44_P552174	AA819525	AA819525	363169	-0.09	-1.219	0.431229
A_44_P426004	AW917246	AW917246	361940	-0.09	-1.220	0.087411
A_44_P148308	RGD1565099_predicted	XM_574467		-0.09	-1.220	0.116336
A_44_P324430	Mdh1	NM_033235	24551	-0.09	-1.220	0.333432
A_44_P444378	BI295601	BI295601	688912	-0.09	-1.220	0.083185
A_44_P333055	A_44_P333055	A_44_P333055		-0.09	-1.220	0.533955
A_42_P667914	Apg3l	NM_134394	171415	-0.09	-1.220	0.113427
A_44_P590748	RGD1560496_predicted	XM_575141		-0.09	-1.220	0.44867
A_44_P444363	AA893517	AA893517	304396	-0.09	-1.220	0.306573
A_44_P180519	Mon1b_predicted	XM_226493		-0.09	-1.221	0.167861
A_44_P1043208	Snx14_predicted	XM_236461		-0.09	-1.221	0.108333
A_44_P421547	Cast	NM_053295	25403	-0.09	-1.221	0.241055
A_44_P253654	CB545442	CB545442		-0.09	-1.221	0.122384
A_42_P671285	Ctdspl_predicted	XM_217293		-0.09	-1.221	0.123635
A_44_P1034417	Inpp5e	XM_342391	25695	-0.09	-1.221	0.179426
A_44_P855808	TC568136	TC568136		-0.09	-1.221	0.26963
A_44_P311178	Tph2	NM_173839	317675	-0.09	-1.221	0.462531
A_44_P255236	Acta1	NM_019212	29437	-0.09	-1.221	0.122615
A_44_P698284	LOC689962	XM_001072700		-0.09	-1.221	0.343187
A_44_P492025	Oasl1	NM_001009681	304545	-0.09	-1.221	0.548302
A_44_P1033684	Prpf4b	NM_001011923	291078	-0.09	-1.221	0.235205
A_44_P625180	TC561223	TC561223		-0.09	-1.221	0.405049
A_44_P230875	BF567478	BF567478	498910	-0.09	-1.222	0.094754
A_44_P197535	BF524125	BF524125		-0.09	-1.222	0.147756
A_44_P423813	Dfna5h	XM_231759	353316	-0.09	-1.222	0.484641
A_43_P11505	Spin2b	NM_012657	24794	-0.09	-1.222	0.378684
A_42_P598210	Mospd3	NM_001025629	288557	-0.09	-1.222	0.16378
A_43_P14467	Zcchc7_predicted	XM_216407		-0.09	-1.222	0.176982
A_44_P655666	TC567231	TC567231		-0.09	-1.222	0.091306
A_44_P699211	A_44_P699211	A_44_P699211		-0.09	-1.222	0.148789
A_44_P386619	L07398	L07398		-0.09	-1.222	0.264277
A_44_P304288	NP166219	NP166219		-0.09	-1.222	0.111955
A_44_P943654	BC085710	BC085710	54293	-0.09	-1.222	0.465862
A_44_P487181	M29996	M29996		-0.09	-1.222	0.410561
A_44_P452617	Otor_predicted	XM_345442		-0.09	-1.223	0.320771

A_44_P491831	Yc2	NM_001009920	494500	-0.09	-1.223	0.411256
A_44_P806854	A_44_P806854	A_44_P806854		-0.09	-1.223	0.164617
A_44_P111566	AW919037	AW919037		-0.09	-1.223	0.340456
A_44_P1006749	LOC685444	XM_216985	685444	-0.09	-1.223	0.346679
A_44_P1058085	LOC679326	XM_001055831		-0.09	-1.223	0.550333
A_44_P456197	Crhr1	AF039203	58959	-0.09	-1.223	0.309205
A_43_P14355	Gkap1	NM_001012160	361202	-0.09	-1.223	0.331432
A_43_P22809	A_43_P22809	A_43_P22809		-0.09	-1.223	0.312325
A_42_P838051	Oma1_predicted	XM_216446		-0.09	-1.223	0.083903
A_43_P13363	Tmem37	NM_139095	245953	-0.09	-1.223	0.276363
A_44_P1015839	RGD1563440_predicted	XM_216459		-0.09	-1.223	0.068752
A_44_P234338	RGD1561119_predicted	XM_231561	296952	-0.09	-1.224	0.09641
A_44_P154188	Gpr25_predicted	XM_344146		-0.09	-1.224	0.199527
A_44_P258751	Pdpk1	NM_031081	81745	-0.09	-1.224	0.08055
A_44_P128447	Abhd6	NM_001007680	305795	-0.09	-1.224	0.30046
A_44_P680689	AI235747	AI235747	24422	-0.09	-1.224	0.317146
A_44_P487856	Ecgf1	NM_001012122	315219	-0.09	-1.224	0.311973
A_44_P260628	XM_235080	XM_235080		-0.09	-1.224	0.176794
A_43_P19438	Zadh1	NM_001015009	299194	-0.09	-1.224	0.126179
A_44_P1045834	Gmppb_predicted	XM_343482		-0.09	-1.224	0.128612
A_44_P182536	Pmfbp1	NM_134393	171414	-0.09	-1.225	0.288451
A_44_P974107	AI103235	AI103235		-0.09	-1.225	0.089662
A_44_P294024	BI285791	BI285791	297694	-0.09	-1.225	0.208246
A_42_P499146	Osbpl6_predicted	XM_242057		-0.09	-1.225	0.425995
A_43_P22166	Guca1b_predicted	XM_236951		-0.09	-1.225	0.181743
A_44_P302726	AA819013	AA819013		-0.09	-1.225	0.094306
A_44_P975945	TC519035	TC519035		-0.09	-1.225	0.11588
A_44_P624972	TC567387	TC567387		-0.09	-1.225	0.119794
A_43_P11883	Tgfbr3	NM_017256	29610	-0.09	-1.225	0.637019
A_44_P130194	BM391810	BM391810		-0.09	-1.225	0.222326
A_44_P1049015	LOC679221	XM_001055029		-0.09	-1.225	0.158253
A_44_P717057	TC548741	TC548741		-0.09	-1.225	0.249778
A_44_P552655	Abcg3	NM_001004076	289453	-0.09	-1.225	0.135886
A_44_P838657	TC538538	TC538538		-0.09	-1.225	0.266492
A_44_P303455	AI176864	AI176864	79125	-0.09	-1.225	0.298299
A_43_P15355	Cs	NM_130755	170587	-0.09	-1.225	0.104457
A_42_P770556	Ppm1b	NM_033096	24667	-0.09	-1.225	0.187415
A_44_P142376	Ppp2r5a_predicted	XM_232413		-0.09	-1.226	0.237
A_42_P635860	Zfp111	NM_133323	170849	-0.09	-1.226	0.056432
A_44_P730454	Mapk8	XM_341399	116554	-0.09	-1.226	0.091361
A_44_P917581	LOC288165	XM_213636		-0.09	-1.226	0.140676
A_44_P203640	Klhl25	NM_001039006	293023	-0.09	-1.226	0.235438
A_44_P181202	BF555778	BF555778		-0.09	-1.226	0.273818
A_44_P792220	TC539913	TC539913		-0.09	-1.226	0.164209
A_44_P461917	RGD1562038_predicted	XM_232320	312668	-0.09	-1.226	0.359468
A_44_P410027	LOC361014	NM_001014136	361014	-0.09	-1.226	0.300945
A_43_P10863	RGD1564241_predicted	XM_343353	363022	-0.09	-1.226	0.210879
A_44_P409696	Raf1	NM_012639	24703	-0.09	-1.226	0.133683
A_44_P998720	Tjp3_predicted	XM_234924		-0.09	-1.226	0.065092
A_44_P366087	LOC680843	XR_005889	680843	-0.09	-1.226	0.303998
A_43_P15125	BF558690	BF558690	24256	-0.09	-1.226	0.094614
A_43_P15024	AW918099	AW918099	362018	-0.09	-1.226	0.192939
A_44_P551919	Ncor1	XM_577103	54299	-0.09	-1.226	0.229858
A_44_P264102	Bcl10	NM_031328	83477	-0.09	-1.226	0.126794
A_44_P322997	AW919338	AW919338		-0.09	-1.227	0.091177
A_44_P147470	A_44_P147470	A_44_P147470		-0.09	-1.227	0.117059
A_44_P140248	Hfe2	NM_001012080	310681	-0.09	-1.227	0.322882
A_42_P669388	Cnksr1	NM_001039011	298545	-0.09	-1.227	0.139575
A_43_P11783	Mpz	NM_017027	24564	-0.09	-1.227	0.173367
A_44_P579630	TC526418	TC526418		-0.09	-1.227	0.11352
A_44_P282794	Chd2_predicted	XM_218790		-0.09	-1.227	0.143459
A_44_P330918	RGD1559767_predicted	XM_346310		-0.09	-1.227	0.090804
A_44_P289548	BF401614	BF401614	294318	-0.09	-1.227	0.143057

A_42_P561930	BC104705	BC104705	297428	-0.09	-1.227	0.05927
A_44_P276607	XM_219480	XM_219480		-0.09	-1.227	0.198242
A_44_P285973	RGD1311361	XM_223187	305171	-0.09	-1.227	0.208516
A_43_P18742	Bxdc1_predicted	XM_215404		-0.09	-1.227	0.582641
A_44_P521520	BE120656	BE120656	315970	-0.09	-1.227	0.11205
A_44_P158709	Dom3z	NM_212497	361799	-0.09	-1.227	0.295962
A_44_P975235	RGD1309220	NM_001034835	316328	-0.09	-1.227	0.123032
A_44_P426512	Gpr39	XM_222578		-0.09	-1.227	0.104097
A_44_P336833	AI171268	AI171268	25585	-0.09	-1.227	0.078306
A_44_P518115	Sh2d4b_predicted	XM_237966	290612	-0.09	-1.228	0.301803
A_44_P659386	A_44_P659386	A_44_P659386		-0.09	-1.228	0.472826
A_44_P199321	RGD1561151_predicted	XM_229086		-0.09	-1.228	0.265521
A_44_P233887	Fgd4	NM_139263	246174	-0.09	-1.228	0.193793
A_43_P10073	MGC93920	NM_001007642	295663	-0.09	-1.229	0.081167
A_44_P143478	AI411618	AI411618	362634	-0.09	-1.229	0.305212
A_43_P15536	Ifnb1	NM_019127	24481	-0.09	-1.229	0.17207
A_44_P262209	Olr773_predicted	NM_001000373	296026	-0.09	-1.229	0.377806
A_43_P18529	Plcl2_predicted	XM_217326		-0.09	-1.229	0.413669
A_44_P354323	RGD1565705_predicted	XM_234709		-0.09	-1.229	0.189969
A_44_P899167	TC537343	TC537343		-0.09	-1.229	0.058223
A_43_P19889	Angptl6_predicted	XM_216613		-0.09	-1.229	0.34505
A_44_P977311	TC528645	TC528645		-0.09	-1.229	0.237099
A_44_P743576	AW917533	AW917533		-0.09	-1.229	0.374257
A_44_P378857	Gldc_predicted	XM_219785		-0.09	-1.229	0.187962
A_43_P17196	RGD1562114_predicted	XM_576184		-0.09	-1.229	0.14893
A_44_P575006	LOC365047	XR_005908	365047	-0.09	-1.229	0.227749
A_44_P852321	LOC680515	XM_001057508	680515	-0.09	-1.229	0.183759
A_44_P234224	A_44_P234224	A_44_P234224		-0.09	-1.229	0.334591
A_44_P296300	AI229298	AI229298	308890	-0.09	-1.229	0.208802
A_42_P498149	Lepre1	NM_053667	114200	-0.09	-1.229	0.072593
A_44_P170309	RGD1562407_predicted	XM_001064372		-0.09	-1.229	0.091394
A_44_P528235	CB547660	CB547660	679931	-0.09	-1.230	0.082532
A_44_P360767	Spink1	NM_012674	24833	-0.09	-1.230	0.502224
A_43_P13241	Ctsd	NM_134334	171293	-0.09	-1.230	0.29729
A_42_P502759	Aplp2	XM_001056031		-0.09	-1.230	0.144143
A_44_P506438	RGD1559971_predicted	XM_574786	499462	-0.09	-1.230	0.088096
A_43_P19227	RGD1561537_predicted	XM_341480	361197	-0.09	-1.230	0.181406
A_43_P12012	Crry	NM_001005330	54243	-0.09	-1.230	0.070325
A_44_P242649	AI549412	AI549412	296973	-0.09	-1.230	0.229039
A_44_P260701	RGD1565247_predicted	XM_220238	302987	-0.09	-1.230	0.111114
A_44_P212355	Lrp6_predicted	XM_232466		-0.09	-1.230	0.117508
A_44_P377875	RGD1309973	NM_001012350	301634	-0.09	-1.230	0.204187
A_43_P12604	Sv2c	NM_031593	29643	-0.09	-1.230	0.31611
A_43_P12014	Fgf2	NM_019305	54250	-0.09	-1.230	0.130411
A_44_P319618	CB606346	CB606346		-0.09	-1.230	0.195325
A_44_P339708	Camk2d	S69671		-0.09	-1.231	0.14289
A_44_P534844	CB547734	CB547734		-0.09	-1.231	0.344498
A_44_P757237	AW918657	AW918657		-0.09	-1.231	0.296933
A_44_P306639	Dap	NM_022526	64322	-0.09	-1.231	0.322061
A_44_P541426	Srms	NM_001011961	296472	-0.09	-1.231	0.331631
A_44_P899021	TC552850	TC552850		-0.09	-1.231	0.149788
A_44_P764689	A_44_P764689	A_44_P764689		-0.09	-1.231	0.135194
A_44_P811716	TC548986	TC548986		-0.09	-1.231	0.092655
A_42_P614692	Oplah	NM_053904	116684	-0.09	-1.231	0.186175
A_44_P246365	TC523264	TC523264		-0.09	-1.231	0.144852
A_43_P16725	RGD1311805	NM_001009638	291784	-0.09	-1.231	0.136364
A_44_P886652	TC532501	TC532501		-0.09	-1.231	0.108758
A_44_P440450	Capns1	NM_017118	29156	-0.09	-1.231	0.144043
A_43_P10536	RGD1309388_predicted	XM_214273		-0.09	-1.231	0.122662
A_44_P714633	Ccpg1_predicted	XM_343429		-0.09	-1.232	0.256391
A_42_P462015	Obfc1	NM_001011943	294025	-0.09	-1.232	0.130101
A_43_P19238	Sp110	NM_001034137	301570	-0.09	-1.232	0.066086
A_44_P459786	RGD1305680_predicted	XM_217342		-0.09	-1.232	0.191505

A_44_P368353	A_44_P368353	A_44_P368353		-0.09	-1.232	0.267462
A_43_P16617	Agtpbp1_predicted	XM_214420		-0.09	-1.232	0.099486
A_44_P354118	LOC682926	XM_001060811		-0.09	-1.232	0.213422
A_44_P482880	Iqgap3_predicted	XM_227396	310621	-0.09	-1.232	0.529193
A_43_P18824	Pank1_predicted	XM_215283		-0.09	-1.232	0.389284
A_44_P960035	Mllt6_predicted	XM_001081378		-0.09	-1.232	0.240563
A_43_P18932	Nmnat3	NM_001013224	363118	-0.09	-1.232	0.07019
A_44_P860743	AW915586	AW915586	305626	-0.09	-1.232	0.215495
A_44_P686987	Gpr160	NM_001025147	499588	-0.09	-1.232	0.526409
A_44_P350779	AI411388	AI411388	25502	-0.09	-1.233	0.146945
A_44_P518233	RGD1309979_predicted	XM_215848		-0.09	-1.233	0.084208
A_44_P221885	BF565038	BF565038		-0.09	-1.233	0.256045
A_42_P535555	Papd5_predicted	XM_226334		-0.09	-1.233	0.204571
A_44_P107352	Hcn3	NM_053685	114245	-0.09	-1.233	0.494064
A_42_P816740	Sh3yl1_predicted	XM_343045		-0.09	-1.233	0.177162
A_43_P16377	Olr1226	NM_001000442	300551	-0.09	-1.233	0.119438
A_43_P15865	Slc24a3	XM_342533	85267	-0.09	-1.233	0.280417
A_44_P643501	AI059089	AI059089		-0.09	-1.233	0.1873
A_42_P688809	Slc12a7	XM_001060536		-0.09	-1.233	0.083002
A_44_P103215	Sec23a_predicted	XM_347236		-0.09	-1.233	0.108491
A_44_P248484	LOC684134	XM_001069080		-0.09	-1.233	0.636716
A_43_P16926	RGD1309388_predicted	XM_214273		-0.09	-1.233	0.139324
A_44_P592432	TC540745	TC540745		-0.09	-1.233	0.115688
A_44_P445652	Nkx2-4_predicted	XM_345445	366213	-0.09	-1.234	0.113231
A_43_P20963	RGD1566180_predicted	XM_001060502		-0.09	-1.234	0.357772
A_43_P10469	BF550398	BF550398		-0.09	-1.234	0.160025
A_44_P229661	XM_342844	XM_342844		-0.09	-1.234	0.108661
A_43_P11535	Atxn1	NM_012726	25049	-0.09	-1.234	0.079383
A_42_P643349	LOC688228	XM_001081553		-0.09	-1.234	0.343852
A_43_P13381	Pomc	NM_139326	24664	-0.09	-1.234	0.054596
A_44_P545447	AW914894	AW914894	116638	-0.09	-1.234	0.22789
A_44_P425794	AI175528	AI175528	84509	-0.09	-1.235	0.114268
A_44_P414553	Zfx_predicted	XM_346318		-0.09	-1.235	0.135172
A_44_P978150	TC551607	TC551607		-0.09	-1.235	0.156317
A_44_P217810	Dzip1	XM_344460	364475	-0.09	-1.235	0.46671
A_44_P295308	RGD1309846_predicted	XM_001073409		-0.09	-1.235	0.150139
A_44_P485019	Auts2_predicted	XM_222103		-0.09	-1.235	0.146312
A_42_P712777	Cabin1	NM_053575	94165	-0.09	-1.235	0.094942
A_44_P397982	RGD1303130	NM_001004226	295231	-0.09	-1.235	0.06059
A_43_P22025	XM_230283	XM_230283		-0.09	-1.235	0.10293
A_44_P457284	A_44_P457284	A_44_P457284		-0.09	-1.235	0.36152
A_44_P319221	Dyrk2_predicted	XM_235179		-0.09	-1.235	0.159297
A_44_P105722	Ezh1_predicted	XM_220986		-0.09	-1.236	0.165787
A_44_P774707	RGD1311037	NM_001014258	366196	-0.09	-1.236	0.137615
A_44_P498903	RGD1564327_predicted	XM_344634	84381	-0.09	-1.236	0.213534
A_44_P254957	Sncb	NM_080777	113893	-0.09	-1.236	0.331613
A_43_P22719	Pla2g2e_predicted	XM_238421		-0.09	-1.236	0.350344
A_44_P332702	Inhbb	XM_344130	25196	-0.09	-1.237	0.22361
A_43_P17704	Slc17a5	NM_001009713	363103	-0.09	-1.237	0.068576
A_44_P301789	Rab10	NM_017359	50993	-0.09	-1.237	0.135903
A_43_P13545	CB547816	CB547816	116721	-0.09	-1.237	0.104672
A_44_P132801	Tsga10ip	NM_001004278	361707	-0.09	-1.237	0.177333
A_44_P161253	CB609118	CB609118		-0.09	-1.237	0.459439
A_44_P292692	AF000144	AF000144		-0.09	-1.237	0.105485
A_44_P140430	LOC296862	XR_008415		-0.09	-1.237	0.383081
A_44_P329389	BF557670	BF557670		-0.09	-1.237	0.412914
A_44_P160174	AI178629	AI178629	24968	-0.09	-1.237	0.346812
A_44_P1025790	Gsto1	NM_001007602	114846	-0.09	-1.238	0.104714
A_44_P1022265	Brwd1_predicted	XM_221627		-0.09	-1.238	0.080589
A_44_P548420	Pmvk	NM_001008352	310645	-0.09	-1.238	0.033434
A_44_P1051772	Ebag9	NM_001009665	299864	-0.09	-1.238	0.211376
A_43_P10651	BX883046	BX883046		-0.09	-1.238	0.298327
A_44_P606762	Aff4_predicted	XM_220420		-0.09	-1.238	0.149408

A_44_P412178	Htr7	NM_022938	65032	-0.09	-1.238	0.327538
A_44_P108304	Slc41a2_predicted	XM_343191		-0.09	-1.238	0.14826
A_44_P261599	AA997406	AA997406	499950	-0.09	-1.238	0.17963
A_44_P103349	RGD1311662_predicted	XM_221623		-0.09	-1.238	0.380772
A_44_P768489	AW921107	AW921107		-0.09	-1.239	0.262912
A_44_P302996	RGD1562968_predicted	XM_342171	361877	-0.09	-1.239	0.23211
A_44_P354534	AA817998	AA817998	309375	-0.09	-1.239	0.320213
A_44_P801735	AW918370	AW918370		-0.09	-1.239	0.277335
A_44_P608466	TC560603	TC560603		-0.09	-1.239	0.229934
A_44_P562272	TC525684	TC525684		-0.09	-1.239	0.22065
A_44_P423004	RGD1562209_predicted	XM_001064478		-0.09	-1.239	0.115605
A_44_P175939	AW142985	AW142985		-0.09	-1.239	0.122202
A_44_P484727	Olr197_predicted	NM_001000188	293339	-0.09	-1.239	0.171963
A_44_P292619	Ppfia3	XM_341856	140591	-0.09	-1.239	0.137716
A_44_P446756	AA999182	AA999182	24777	-0.09	-1.239	0.317025
A_44_P555664	XM_228987	XM_228987		-0.09	-1.239	0.154551
A_43_P14861	Tmem34	NM_178330	291946	-0.09	-1.239	0.278579
A_44_P295467	RGD1305350_predicted	XM_243032	313699	-0.09	-1.240	0.11103
A_42_P479064	BF551382	BF551382		-0.09	-1.240	0.074401
A_44_P221418	AW528833	AW528833	25050	-0.09	-1.240	0.095947
A_44_P1007008	Ube2l6	NM_001024755	295704	-0.09	-1.240	0.360252
A_44_P463915	XM_218225	XM_218225		-0.09	-1.240	0.154058
A_44_P299451	Cebpg	XM_001079636		-0.09	-1.240	0.055537
A_44_P302944	Pem	NM_022175	24631	-0.09	-1.240	0.341205
A_44_P175520	XM_219475	XM_219475		-0.09	-1.240	0.196406
A_44_P101223	Gcdh_predicted	XM_001066810		-0.09	-1.240	0.123235
A_44_P359035	XM_347073	XM_347073		-0.09	-1.240	0.097369
A_44_P401179	XM_212694	XM_212694		-0.09	-1.240	0.154497
A_44_P205572	Atpif1	NM_012915	25392	-0.09	-1.241	0.066601
A_44_P175785	XM_228759	XM_228759		-0.09	-1.241	0.060545
A_44_P521845	AI171692	AI171692	311844	-0.09	-1.241	0.153281
A_44_P753220	Sned1	XM_237415	316638	-0.09	-1.241	0.157451
A_44_P344273	Tloc1	NM_001034129	294912	-0.09	-1.241	0.103779
A_42_P534172	Cd52	NM_053983	117054	-0.09	-1.241	0.309409
A_44_P507021	AA925482	AA925482	317296	-0.09	-1.241	0.19344
A_44_P311518	XM_218058	XM_218058		-0.09	-1.241	0.20782
A_42_P617941	BF289725	BF289725	313867	-0.09	-1.241	0.119627
A_44_P129262	Clmn_predicted	XM_234500		-0.09	-1.241	0.227133
A_44_P545478	Pkig	NM_153469	266709	-0.09	-1.241	0.181743
A_42_P829031	Coasy	NM_001006954	287711	-0.09	-1.241	0.130282
A_44_P340357	Znf183	NM_001004445	314313	-0.09	-1.242	0.074334
A_43_P10656	Casc4_predicted	XM_001077018		-0.09	-1.242	0.290537
A_44_P166396	RGD1311622_predicted	XM_216348		-0.09	-1.242	0.101553
A_44_P667940	Centg2_predicted	XM_237381		-0.09	-1.242	0.112022
A_44_P425800	BU759048	BU759048	691431	-0.09	-1.242	0.324116
A_44_P178395	LOC680036	XM_001055430	680036	-0.09	-1.242	0.090183
A_44_P269843	Nagk	NM_001037768	297393	-0.09	-1.242	0.049847
A_44_P440672	Obox5_predicted	XM_218256		-0.09	-1.242	0.404475
A_44_P530953	AI169751	AI169751	361673	-0.09	-1.242	0.410825
A_44_P479631	Ocrl_mapped	XM_229106		-0.09	-1.242	0.332865
A_44_P156086	CV074085	CV074085	360697	-0.09	-1.242	0.350774
A_44_P109608	Gsta2	NM_017013	24422	-0.09	-1.243	0.147496
A_44_P328815	Smarcal1_predicted	XM_237241		-0.09	-1.243	0.09262
A_43_P19409	Nt5c1a_predicted	XM_233494		-0.09	-1.243	0.157306
A_44_P408751	AA996568	AA996568	287382	-0.09	-1.243	0.349207
A_44_P284561	AA859368	AA859368		-0.09	-1.243	0.101605
A_44_P743184	AW921086	AW921086	501061	-0.09	-1.243	0.165787
A_44_P104087	BF396482	BF396482		-0.09	-1.243	0.411475
A_44_P457159	RGD1565904_predicted	XM_226176		-0.09	-1.243	0.109761
A_44_P270109	Unc45b_predicted	XM_220771		-0.09	-1.243	0.122493
A_44_P450379	A_44_P450379	A_44_P450379		-0.09	-1.243	0.173904
A_43_P18744	Enpp5	NM_001012744	316249	-0.09	-1.243	0.171504
A_43_P18937	LOC687705	XM_001079735		-0.09	-1.243	0.066131

A_44_P859342	RGD1566151_predicted	XM_576456	501043	-0.09	-1.243	0.212555
A_44_P543329	Havcr2	XM_343881		-0.09	-1.243	0.094116
A_42_P572948	Prosapip1	NM_172022	280670	-0.09	-1.243	0.115374
A_44_P210120	Ptk9	NM_001008521	315265	-0.09	-1.243	0.138093
A_44_P822100	A_44_P822100	A_44_P822100		-0.09	-1.243	0.159054
A_44_P654096	TC525262	TC525262		-0.09	-1.243	0.286951
A_44_P260268	Tinf2	NM_001006962	290232	-0.09	-1.243	0.1591
A_42_P681251	Ica1	NM_030844	81024	-0.09	-1.244	0.080942
A_42_P665164	Slc9a3r2	NM_053811	116501	-0.09	-1.244	0.263214
A_43_P15557	Pter	NM_022224	63852	-0.09	-1.244	0.110634
A_44_P155549	XM_345136	XM_345136		-0.09	-1.244	0.361844
A_44_P825694	TC519316	TC519316		-0.09	-1.244	0.05978
A_42_P664501	Ganab_predicted	XM_215144		-0.09	-1.244	0.299649
A_44_P521779	AI058430	AI058430	114637	-0.09	-1.244	0.222377
A_44_P215447	Pbxip1	BC085776	310644	-0.09	-1.244	0.095706
A_44_P413164	CB548260	CB548260	294375	-0.09	-1.244	0.061092
A_44_P992523	Cops3	NM_001004200	287367	-0.09	-1.244	0.22554
A_44_P185661	BF561678	BF561678		-0.10	-1.245	0.215298
A_44_P517115	AA859373	AA859373	363925	-0.10	-1.245	0.181614
A_42_P509843	Tradd	XM_341671	246756	-0.10	-1.245	0.099461
A_44_P382219	XM_344567	XM_344567		-0.10	-1.245	0.23684
A_44_P946310	TC542159	TC542159		-0.10	-1.245	0.27911
A_44_P238125	AI711105	AI711105	293844	-0.10	-1.245	0.307752
A_44_P500478	AI101373	AI101373	361104	-0.10	-1.245	0.213825
A_44_P163271	Cyp2b13	NM_198733	292728	-0.10	-1.245	0.096549
A_44_P592726	TC558494	TC558494		-0.10	-1.245	0.127425
A_44_P354229	XM_228535	XM_228535		-0.10	-1.246	0.131893
A_44_P122265	BE106401	BE106401		-0.10	-1.246	0.086216
A_44_P998015	AW915543	AW915543	313838	-0.10	-1.246	0.318745
A_44_P354223	RGD1563213_predicted	XM_001057027		-0.10	-1.246	0.261153
A_42_P597638	Mte1	NM_138907	192272	-0.10	-1.246	0.155067
A_44_P243345	A_44_P243345	A_44_P243345		-0.10	-1.246	0.289212
A_44_P530041	Hdac8_predicted	XM_343804	363481	-0.10	-1.246	0.049543
A_42_P634674	Cox5a	NM_145783	252934	-0.10	-1.246	0.097193
A_44_P363216	AB097859	AB097859		-0.10	-1.246	0.210947
A_44_P345450	BF282241	BF282241		-0.10	-1.246	0.240199
A_44_P144015	AA998073	AA998073		-0.10	-1.246	0.265764
A_44_P439824	BF395095	BF395095	498587	-0.10	-1.246	0.054089
A_44_P243456	RGD1561955_predicted	XM_001072779		-0.10	-1.246	0.093162
A_44_P455254	BQ203347	BQ203347	289217	-0.10	-1.246	0.386155
A_44_P118549	BQ191778	BQ191778	365215	-0.10	-1.246	0.305878
A_44_P588583	BE110718	BE110718	316335	-0.10	-1.246	0.081446
A_44_P175626	ENSRNOT00000038497	ENSRNOT00000038497		-0.10	-1.247	0.064341
A_44_P220888	Calr3	NM_001012212	364529	-0.10	-1.247	0.120901
A_44_P116768	XM_215126	XM_215126		-0.10	-1.247	0.132478
A_44_P233583	Krt1-9	NM_153476	266717	-0.10	-1.247	0.382012
A_44_P689909	A_44_P689909	A_44_P689909		-0.10	-1.247	0.20665
A_42_P567568	DV727788	DV727788		-0.10	-1.247	0.152127
A_44_P279046	Ppp1cb	XM_229259		-0.10	-1.247	0.156361
A_42_P618345	Kpna1	NM_198726	288064	-0.10	-1.247	0.10598
A_44_P761534	LOC684112	XM_001068984		-0.10	-1.247	0.136955
A_44_P270119	RGD1307288_predicted	XM_213490	287761	-0.10	-1.247	0.132567
A_44_P1016222	Ncor1	XM_577103	54299	-0.10	-1.247	0.170802
A_44_P198408	BG666659	BG666659	294680	-0.10	-1.248	0.232914
A_44_P121547	LOC686547	XM_001074206		-0.10	-1.248	0.167316
A_44_P121401	RGD1306643_predicted	XM_237998	291019	-0.10	-1.248	0.087289
A_44_P1030594	Cebpg	XM_001079636		-0.10	-1.248	0.069706
A_44_P318188	AA892582	AA892582		-0.10	-1.248	0.250982
A_44_P1027780	Cap350	XM_341136	246304	-0.10	-1.248	0.076699
A_44_P332249	AI716801	AI716801	25238	-0.10	-1.248	0.31186
A_44_P532603	BF558585	BF558585		-0.10	-1.248	0.188953
A_44_P413151	BM389417	BM389417		-0.10	-1.248	0.472153
A_44_P472661	Klf2_predicted	NM_001007684	306330	-0.10	-1.248	0.094984

A_44_P361144	Btbd14a	XM_216005	296583	-0.10	-1.248	0.149188
A_44_P335241	Aldh3b1	NM_001006998	309147	-0.10	-1.249	0.285169
A_44_P993464	RGD1563202_predicted	XM_237782		-0.10	-1.249	0.056296
A_44_P207625	AA893219	AA893219	25134	-0.10	-1.249	0.262581
A_44_P973807	AA850706	AA850706	314280	-0.10	-1.249	0.116568
A_44_P306325	Serinc1	NM_182951	294421	-0.10	-1.249	0.070969
A_42_P499282	Msrb2	NM_001031660	361286	-0.10	-1.249	0.119246
A_44_P853038	RGD1311188_predicted	XM_001074596		-0.10	-1.249	0.220201
A_44_P351537	LOC308266	XM_218069		-0.10	-1.249	0.184844
A_44_P139270	AI044341	AI044341	116643	-0.10	-1.249	0.149948
A_43_P13862	RGD1563568_predicted	XM_001070192		-0.10	-1.249	0.522772
A_44_P245893	Snx25	XM_224863	306471	-0.10	-1.249	0.242352
A_44_P516144	Mpdu1	XM_220606		-0.10	-1.249	0.048418
A_44_P1039249	Dok3_predicted	XM_225170		-0.10	-1.249	0.517948
A_44_P762373	RGD1561416_predicted	XR_008803	500536	-0.10	-1.249	0.198754
A_44_P574253	Dapk2	XM_578739		-0.10	-1.250	0.315277
A_42_P529193	Sdhc	NM_001005534	289217	-0.10	-1.250	0.076459
A_44_P529962	A_44_P529962	A_44_P529962		-0.10	-1.250	0.074708
A_43_P13000	Slc25a20	NM_053965	117035	-0.10	-1.250	0.16192
A_44_P380416	AA819258	AA819258		-0.10	-1.250	0.19388
A_44_P355850	Coro6	NM_139115	245982	-0.10	-1.250	0.260016
A_44_P292828	XM_341060	XM_341060		-0.10	-1.250	0.057632
A_44_P247727	Dhfr	NM_130400	24312	-0.10	-1.250	0.36881
A_44_P624267	DV717924	DV717924		-0.10	-1.250	0.203163
A_44_P776578	TC555518	TC555518		-0.10	-1.250	0.14565
A_44_P152434	A_44_P152434	A_44_P152434		-0.10	-1.250	0.092646
A_44_P587335	BF389602	BF389602		-0.10	-1.251	0.171148
A_44_P214229	RGD1562529_predicted	XM_242139	311372	-0.10	-1.251	0.134356
A_44_P426833	RGD1566197_predicted	XM_228944		-0.10	-1.251	0.134994
A_43_P17027	RGD1305466	NM_001033689	291922	-0.10	-1.251	0.116713
A_44_P1037796	Arfgap1	NM_145090	246310	-0.10	-1.251	0.066518
A_43_P17851	Sema4b	XM_001065817	293042	-0.10	-1.251	0.079787
A_44_P231708	Lefty2	NM_001007556	289316	-0.10	-1.251	0.20432
A_44_P382618	Otop2_predicted	XM_221107		-0.10	-1.251	0.194238
A_44_P507673	AA891542	AA891542	313811	-0.10	-1.251	0.266867
A_44_P946089	TC558630	TC558630		-0.10	-1.251	0.127125
A_44_P187784	RGD1307032_predicted	XM_340759		-0.10	-1.251	0.144303
A_44_P1050889	Mlstd2	NM_001011933	293173	-0.10	-1.251	0.255161
A_44_P1044565	Cdadc1	NM_001012156	361052	-0.10	-1.251	0.115172
A_43_P16047	Gli1	XM_345832	140589	-0.10	-1.251	0.141248
A_44_P128423	Lrdd_predicted	XM_219485		-0.10	-1.252	0.212455
A_44_P649931	CK845504	CK845504	304193	-0.10	-1.252	0.090571
A_44_P947573	TC549243	TC549243		-0.10	-1.252	0.152902
A_44_P258052	Rb1cc1_predicted	XM_232667		-0.10	-1.252	0.121175
A_44_P525020	Dffa	NM_053679	114214	-0.10	-1.252	0.069431
A_43_P19265	RGD1562705_predicted	XM_342507	362205	-0.10	-1.252	0.355404
A_44_P459191	Sycp3	NM_013041	25561	-0.10	-1.252	0.404832
A_44_P313838	Lipc	NM_012597	24538	-0.10	-1.252	0.156533
A_44_P184753	RT1-A2	NM_001008829	24737	-0.10	-1.252	0.197577
A_44_P515707	A_44_P515707	A_44_P515707		-0.10	-1.252	0.235938
A_44_P132967	AABR03026346	AABR03026346		-0.10	-1.252	0.10131
A_44_P883921	TC536252	TC536252		-0.10	-1.252	0.162412
A_43_P20654	Rhpn2_predicted	XM_238787		-0.10	-1.252	0.11266
A_44_P354216	RGD1561642_predicted	XM_343785	363466	-0.10	-1.252	0.184091
A_44_P258361	AA900777	AA900777		-0.10	-1.252	0.047573
A_44_P234763	AW525613	AW525613	297333	-0.10	-1.253	0.261767
A_44_P523062	Atoh7_predicted	XM_345102		-0.10	-1.253	0.101728
A_44_P291728	Ttn	XM_001065955		-0.10	-1.253	0.234112
A_44_P547647	BM986620	BM986620	83427	-0.10	-1.253	0.181414
A_44_P207194	AI234860	AI234860	29526	-0.10	-1.253	0.18307
A_44_P622315	TC519223	TC519223		-0.10	-1.253	0.218531
A_43_P12829	Zp1	XM_001074922		-0.10	-1.253	0.189327
A_44_P929247	RGD1561655_predicted	XM_576190	500801	-0.10	-1.253	0.241821

A_44_P996338	Arid4a_predicted	XM_234281		-0.10	-1.253	0.319341
A_43_P22909	Pdzk6_predicted	XM_342237	361938	-0.10	-1.253	0.262441
A_44_P104952	Tert	NM_053423	301965	-0.10	-1.253	0.245894
A_44_P525101	ENSRNOT00000016719	ENSRNOT00000016719		-0.10	-1.253	0.059185
A_44_P229555	RGD1564528_predicted	XM_346956		-0.10	-1.254	0.265101
A_44_P311237	Olr1380_predicted	NM_001000857	405148	-0.10	-1.254	0.235125
A_44_P514577	AA955829	AA955829	29282	-0.10	-1.254	0.12857
A_44_P775907	A_44_P775907	A_44_P775907		-0.10	-1.254	0.081269
A_44_P206632	BF556609	BF556609		-0.10	-1.254	0.177152
A_44_P158498	Mina	NM_153309	266670	-0.10	-1.254	0.07948
A_44_P239985	Af6	NM_013217	26955	-0.10	-1.254	0.067174
A_44_P134287	Taf13_predicted	XM_227586		-0.10	-1.254	0.144784
A_43_P10712	BG665019	BG665019		-0.10	-1.254	0.198398
A_44_P129665	AI717054	AI717054		-0.10	-1.254	0.353841
A_44_P114153	M18840	M18840		-0.10	-1.254	0.122797
A_42_P455825	AW918123	AW918123		-0.10	-1.254	0.144454
A_44_P583151	A_44_P583151	A_44_P583151		-0.10	-1.255	0.223433
A_44_P502793	RGD1307700	NM_001009714	363219	-0.10	-1.255	0.156333
A_44_P1011244	MGC95210	NM_001005532	287798	-0.10	-1.255	0.231459
A_44_P156647	RGD1306212_predicted	XM_341114	360839	-0.10	-1.255	0.291286
A_44_P804214	BF554040	BF554040	306574	-0.10	-1.255	0.218233
A_44_P284222	BF556255	BF556255		-0.10	-1.255	0.145535
A_44_P100744	Sirt1_predicted	XM_228146		-0.10	-1.255	0.251412
A_44_P265188	Plekha5	XM_342781	246237	-0.10	-1.255	0.067464
A_42_P522171	RGD1307966_predicted	XM_001081512		-0.10	-1.255	0.348031
A_44_P1034068	BC105769	BC105769		-0.10	-1.255	0.090026
A_44_P808129	TC525252	TC525252		-0.10	-1.255	0.082674
A_43_P14387	Pdcd8	NM_031356	83533	-0.10	-1.256	0.044355
A_44_P410109	A_44_P410109	A_44_P410109		-0.10	-1.256	0.226825
A_44_P623506	TC543330	TC543330		-0.10	-1.256	0.185663
A_42_P637037	Clcn3	NM_053363	84360	-0.10	-1.256	0.089002
A_44_P236755	XM_234751	XM_234751		-0.10	-1.256	0.21225
A_44_P1050054	Pygl	NM_022268	64035	-0.10	-1.256	0.184575
A_44_P151264	AA900409	AA900409		-0.10	-1.256	0.187111
A_44_P977419	Gopc_predicted	XM_228173		-0.10	-1.256	0.145515
A_44_P365560	LOC297530	NM_199207	297530	-0.10	-1.256	0.105098
A_44_P524642	Obp1f	NM_138903	192267	-0.10	-1.256	0.127458
A_44_P547788	Clk3	NM_134340	171305	-0.10	-1.256	0.042443
A_44_P397927	Olr1734_predicted	NM_001001119	406018	-0.10	-1.256	0.120796
A_44_P318718	RGD1560125_predicted	XM_346920	360825	-0.10	-1.257	0.181743
A_44_P323785	XM_230974	XM_230974		-0.10	-1.257	0.107292
A_44_P282609	Ezh1_predicted	XM_001081456		-0.10	-1.257	0.073515
A_44_P474815	AI072966	AI072966		-0.10	-1.257	0.386384
A_44_P334181	AI230208	AI230208		-0.10	-1.257	0.241662
A_43_P10237	LOC498178	XM_573394	498178	-0.10	-1.257	0.133803
A_44_P255177	Cacna1s	L04684	116652	-0.10	-1.257	0.229542
A_44_P423938	Nog	XM_343954		-0.10	-1.258	0.128079
A_43_P17404	Rutbc1_predicted	XM_220699		-0.10	-1.258	0.042851
A_43_P14553	TC556391	TC556391		-0.10	-1.258	0.376716
A_44_P905727	TC526119	TC526119		-0.10	-1.258	0.280471
A_44_P701013	TC551966	TC551966		-0.10	-1.258	0.38877
A_43_P15874	Cap350	XM_341136	246304	-0.10	-1.258	0.126433
A_42_P566906	Peci	NM_001006966	291075	-0.10	-1.258	0.03314
A_43_P17566	XM_215061	XM_215061		-0.10	-1.258	0.136364
A_44_P335350	Myom2	XM_240481	306616	-0.10	-1.258	0.135259
A_44_P197041	Ypel5	NM_001035221	298792	-0.10	-1.259	0.204741
A_44_P551001	Ttll10	NM_001024758	298692	-0.10	-1.259	0.219772
A_44_P173497	XM_345604	XM_345604		-0.10	-1.259	0.354447
A_44_P467541	AA963075	AA963075	300803	-0.10	-1.259	0.26226
A_43_P12886	Nkx2-5	NM_053651	114109	-0.10	-1.259	0.172385
A_44_P125764	Olr499_predicted	NM_001000931	405251	-0.10	-1.259	0.170069
A_44_P702122	TC550238	TC550238		-0.10	-1.259	0.127183
A_44_P1034937	Smarcd3	NM_001011966	296732	-0.10	-1.259	0.117237

A_44_P283759	LOC364487	XM_344466		-0.10	-1.259	0.252591
A_44_P321447	Tceal8	NM_001014275	367909	-0.10	-1.259	0.069025
A_44_P277556	AA819312	AA819312		-0.10	-1.260	0.416887
A_44_P113403	RGD1561431_predicted	XM_001058923		-0.10	-1.260	0.392824
A_44_P376505	RGD1308143	BC083847	316685	-0.10	-1.260	0.095539
A_43_P13024	Tcea2	NM_057098	29575	-0.10	-1.260	0.174176
A_44_P1035856	BF558507	BF558507		-0.10	-1.260	0.080317
A_43_P14632	Acsl1	NM_012820	25288	-0.10	-1.260	0.115688
A_44_P417994	LOC497078	NM_001012227	497078	-0.10	-1.260	0.095947
A_44_P257563	Pctk3	XM_213886		-0.10	-1.260	0.187549
A_44_P447746	Kif21b_predicted	XM_223090		-0.10	-1.260	0.052691
A_42_P577482	BM387057	BM387057		-0.10	-1.260	0.273989
A_44_P286863	Nrip1_predicted	XM_221724		-0.10	-1.260	0.054808
A_43_P20056	Rnf135	NM_001012010	303350	-0.10	-1.260	0.177112
A_44_P506785	AA924414	AA924414		-0.10	-1.260	0.26963
A_44_P433223	Olr247_predicted	NM_001000217	293392	-0.10	-1.261	0.115982
A_44_P784405	BE102568	BE102568	367932	-0.10	-1.261	0.179611
A_44_P704817	A_44_P704817	A_44_P704817		-0.10	-1.261	0.124389
A_44_P1030943	Mpi_mapped	NM_001004081	300741	-0.10	-1.261	0.060053
A_44_P393605	Taf9l	NM_133615	171152	-0.10	-1.261	0.073506
A_44_P318794	RGD1562784_predicted	XM_219560	309197	-0.10	-1.261	0.032808
A_44_P527739	Pars2	NM_001014064	313429	-0.10	-1.261	0.136336
A_43_P12283	Mog	NM_022668	24558	-0.10	-1.261	0.132003
A_44_P187724	LOC680127	XM_001055833		-0.10	-1.261	0.123091
A_44_P394042	LOC313934	XM_233945	313934	-0.10	-1.261	0.160888
A_44_P187576	XM_217613	XM_217613		-0.10	-1.261	0.047494
A_44_P778533	TC528848	TC528848		-0.10	-1.262	0.306221
A_43_P21893	Cdh16	NM_001012055	307614	-0.10	-1.262	0.121046
A_44_P401083	Abhd14b	NM_001007664	300983	-0.10	-1.262	0.099095
A_44_P508264	Cdkn1b	NM_031762	83571	-0.10	-1.262	0.161285
A_44_P290627	Eftud1_predicted	XM_218845		-0.10	-1.262	0.177382
A_44_P510995	RGD1308117_predicted	XM_341349	361066	-0.10	-1.262	0.098506
A_43_P21022	Lhx6_predicted	XM_231175		-0.10	-1.262	0.255818
A_44_P142472	RGD1308064_predicted	XM_214293		-0.10	-1.262	0.052643
A_43_P23068	RGD1562243_predicted	XM_343495	363156	-0.10	-1.262	0.2529
A_44_P421539	Cast	NM_053295	25403	-0.10	-1.262	0.179382
A_44_P638915	TC543039	TC543039		-0.10	-1.262	0.346471
A_44_P431465	Sema3e_predicted	XM_231354	296789	-0.10	-1.262	0.247706
A_42_P570677	Usp8_predicted	XM_001078286		-0.10	-1.263	0.217676
A_43_P12732	Vps52	NM_033097	25218	-0.10	-1.263	0.06503
A_44_P295300	A_44_P295300	A_44_P295300		-0.10	-1.263	0.18416
A_42_P504240	RGD1306643_predicted	XM_237998	291019	-0.10	-1.263	0.053871
A_44_P414700	XM_216335	XM_216335		-0.10	-1.263	0.150706
A_43_P21660	Unc84a	NM_001007147	360773	-0.10	-1.263	0.109368
A_44_P499226	RGD1307214_predicted	XM_225444		-0.10	-1.263	0.076576
A_43_P11678	Tnr	NM_013045	25567	-0.10	-1.263	0.040751
A_44_P263095	RGD1562364_predicted	XM_214287		-0.10	-1.263	0.092345
A_44_P289758	AW535083	AW535083		-0.10	-1.263	0.072579
A_44_P293438	RGD1311925_predicted	XM_340949		-0.10	-1.263	0.056912
A_44_P330792	A_44_P330792	A_44_P330792		-0.10	-1.264	0.406535
A_44_P458332	AW141703	AW141703	24518	-0.10	-1.264	0.165816
A_44_P592381	RGD1563633_predicted	XM_238366	297504	-0.10	-1.264	0.050447
A_44_P372485	Nrg2	XM_344662	432361	-0.10	-1.264	0.237417
A_43_P10006	Map2k3	XM_239239	303200	-0.10	-1.264	0.092739
A_44_P897591	LOC302495	XM_001070285		-0.10	-1.264	0.308501
A_44_P450258	RGD1307696_predicted	XM_230827		-0.10	-1.264	0.049318
A_44_P250565	TC534083	TC534083		-0.10	-1.264	0.233748
A_44_P192193	RGD1564366_predicted	XM_344578		-0.10	-1.264	0.084149
A_44_P426780	Usp38_predicted	XM_226362		-0.10	-1.264	0.152763
A_44_P239634	AA925120	AA925120		-0.10	-1.264	0.082152
A_44_P156830	Ripk1_predicted	XM_225262		-0.10	-1.264	0.11044
A_43_P11104	TC561031	TC561031		-0.10	-1.264	0.209365
A_44_P134645	BI282339	BI282339	360596	-0.10	-1.265	0.387741

A_44_P265783	CB557839	CB557839		-0.10	-1.265	0.199561
A_42_P670371	RGD1311960_predicted	XM_223107	305095	-0.10	-1.265	0.172241
A_44_P698747	LOC685374	XM_001062165	685374	-0.10	-1.265	0.210204
A_44_P161770	RGD1564851_predicted	XM_001055712		-0.10	-1.265	0.159507
A_44_P792333	TC517425	TC517425		-0.10	-1.265	0.374068
A_44_P493214	Cyct	NM_012840	25310	-0.10	-1.265	0.197293
A_44_P508534	LOC308401	XR_009000	308401	-0.10	-1.266	0.070091
A_44_P553522	LOC303823	XM_221319	303823	-0.10	-1.266	0.18458
A_44_P365635	Ankrd27_predicted	XM_001079798		-0.10	-1.266	0.058389
A_43_P16132	AF241612	AF241612		-0.10	-1.266	0.15705
A_44_P547385	AA899649	AA899649	29246	-0.10	-1.266	0.138678
A_44_P412961	XM_221205	XM_221205		-0.10	-1.266	0.131083
A_44_P515876	RGD1560667_predicted	XM_226563		-0.10	-1.266	0.366615
A_44_P334543	BX883048	BX883048		-0.10	-1.266	0.094802
A_44_P151582	Il17re	NM_001004091	362417	-0.10	-1.266	0.186311
A_44_P421425	Olr309_predicted	NM_001000242	293607	-0.10	-1.266	0.455372
A_44_P494242	Anxa7	NM_130416	155423	-0.10	-1.266	0.098928
A_44_P403532	RGD1564019_predicted	XM_001078585		-0.10	-1.266	0.19899
A_44_P759863	AY623034	AY623034	300173	-0.10	-1.267	0.100755
A_44_P332530	Olr1488_predicted	NM_001000719	404971	-0.10	-1.267	0.358489
A_44_P482761	XM_219368	XM_219368		-0.10	-1.267	0.048611
A_42_P589355	XM_227946	XM_227946		-0.10	-1.267	0.118243
A_44_P552573	Foxa3	NM_017077	25100	-0.10	-1.267	0.249636
A_44_P302550	Cd320	NM_001014201	362851	-0.10	-1.267	0.0794
A_44_P928190	RGD1561961_predicted	XM_213926	289181	-0.10	-1.267	0.083382
A_42_P493567	Ctbs	NM_031023	81652	-0.10	-1.267	0.217947
A_44_P608694	TC562475	TC562475		-0.10	-1.267	0.311567
A_44_P114646	XM_228914	XM_228914		-0.10	-1.267	0.176737
A_42_P710870	AW917990	AW917990		-0.10	-1.267	0.384497
A_44_P244338	Doc2a	NM_022937	65031	-0.10	-1.267	0.222352
A_44_P372979	Leprel2_predicted	XM_216278		-0.10	-1.267	0.210889
A_44_P326001	Rfk	NM_001014106	499328	-0.10	-1.268	0.133879
A_44_P322968	BG666882	BG666882		-0.10	-1.268	0.338937
A_44_P899822	TC556841	TC556841		-0.10	-1.268	0.148029
A_44_P839602	TC553935	TC553935		-0.10	-1.268	0.327916
A_44_P863304	AW529994	AW529994		-0.10	-1.268	0.133019
A_44_P671842	TC560574	TC560574		-0.10	-1.268	0.047741
A_44_P189097	AI145607	AI145607	498600	-0.10	-1.268	0.560343
A_44_P390265	AA858718	AA858718	360678	-0.10	-1.269	0.102441
A_44_P880090	DV728750	DV728750		-0.10	-1.269	0.101898
A_44_P459052	Slc28a2	U67084	60423	-0.10	-1.269	0.425898
A_44_P181145	BX883045	BX883045		-0.10	-1.269	0.315456
A_43_P16066	Kif27	NM_198050	246209	-0.10	-1.269	0.519854
A_43_P11981	Aes	NM_019220	29466	-0.10	-1.269	0.113389
A_44_P474052	AI406544	AI406544	303792	-0.10	-1.269	0.12571
A_44_P730508	TC522946	TC522946		-0.10	-1.269	0.350332
A_44_P974709	LOC500077	NM_001024325	500077	-0.10	-1.269	0.026572
A_44_P852315	LOC499856	NM_001025042	499856	-0.10	-1.269	0.419964
A_44_P333078	RGD1305459_predicted	XM_231125	311827	-0.10	-1.269	0.069709
A_44_P1020018	Cnot7_predicted	XM_224894		-0.10	-1.270	0.190375
A_44_P816448	CK478957	CK478957		-0.10	-1.270	0.147174
A_44_P213001	Vwce_predicted	XM_219579	309209	-0.10	-1.270	0.109812
A_44_P351860	Fbxw9	XM_213838	288921	-0.10	-1.270	0.27456
A_44_P958894	LOC298795	NM_001013941	298795	-0.10	-1.270	0.316361
A_44_P142277	Kcnh5	NM_133610	171146	-0.10	-1.270	0.168527
A_44_P452846	RGD1564871_predicted	XM_236597	315947	-0.10	-1.270	0.452561
A_44_P932684	TC529062	TC529062		-0.10	-1.270	0.250305
A_42_P823679	Atp5o	NM_138883	192241	-0.10	-1.270	0.041233
A_44_P731007	CK479210	CK479210		-0.10	-1.270	0.056484
A_44_P1056949	LOC678834	XM_001053615		-0.10	-1.271	0.164881
A_44_P154622	LOC691426	XM_001078224	691426	-0.10	-1.271	0.076562
A_42_P773636	Tnfsf13	NM_001009623	287437	-0.10	-1.271	0.162607
A_44_P431338	LOC683066	XM_001064325		-0.10	-1.271	0.208017

A_44_P286430	RGD1559693_predicted	XM_235476	315117	-0.10	-1.271	0.182596
A_44_P637763	AY623033	AY623033	300173	-0.10	-1.271	0.079732
A_44_P403699	BF550442	BF550442		-0.10	-1.271	0.034276
A_44_P249259	BI303276	BI303276	303565	-0.10	-1.271	0.215498
A_44_P377958	AI112916	AI112916		-0.10	-1.271	0.08148
A_44_P275801	LOC681944	XM_001059058		-0.10	-1.271	0.058276
A_43_P15540	Vdp	NM_019379	56042	-0.10	-1.272	0.104625
A_43_P21908	Epha1_predicted	XM_231658		-0.10	-1.272	0.084984
A_44_P868984	TC556398	TC556398		-0.10	-1.272	0.177975
A_44_P978899	Tjp1_predicted	XM_218747		-0.10	-1.272	0.191511
A_44_P661747	A_44_P661747	A_44_P661747		-0.10	-1.272	0.114846
A_44_P130454	Dio2	NM_031720	65162	-0.10	-1.272	0.07665
A_42_P463675	Itga7	NM_030842	81008	-0.10	-1.272	0.343948
A_44_P124213	XM_238492	XM_238492		-0.10	-1.272	0.17158
A_43_P12843	Idi1	NM_053539	89784	-0.10	-1.272	0.132003
A_44_P498547	AA924864	AA924864	360650	-0.10	-1.272	0.283361
A_44_P291395	AI113042	AI113042	691075	-0.10	-1.272	0.261255
A_44_P473273	Vrk2_predicted	XM_341265		-0.10	-1.272	0.447555
A_44_P960687	TC556607	TC556607		-0.10	-1.272	0.107261
A_44_P395757	Grm4	NM_022666	24417	-0.10	-1.272	0.191044
A_44_P557934	RGD1309144	NM_001009658	297332	-0.10	-1.272	0.062206
A_44_P1023938	LOC298643	NM_001017450	298643	-0.10	-1.272	0.061305
A_44_P592086	TC556865	TC556865		-0.10	-1.273	0.073752
A_44_P388937	X65777	X65777		-0.10	-1.273	0.184846
A_44_P543965	A_44_P543965	A_44_P543965		-0.10	-1.273	0.146945
A_44_P528906	C1galt1c1	NM_001030033	302499	-0.10	-1.273	0.212305
A_44_P361329	A_44_P361329	A_44_P361329		-0.10	-1.273	0.215186
A_44_P527661	XM_232347	XM_232347		-0.10	-1.273	0.061128
A_44_P356551	Jph3_predicted	XM_226549		-0.10	-1.273	0.218429
A_44_P959044	LOC499390	NM_001024294	499390	-0.11	-1.274	0.053196
A_44_P438090	Plcb3	NM_033350	29322	-0.11	-1.274	0.124844
A_44_P438968	BF396589	BF396589	313139	-0.11	-1.274	0.150644
A_44_P271435	Rnase6	NM_206815	305842	-0.11	-1.274	0.198864
A_42_P523472	AW916914	AW916914		-0.11	-1.274	0.068674
A_43_P14633	DV720603	DV720603		-0.11	-1.274	0.256611
A_42_P685906	Atp5d	NM_139106	245965	-0.11	-1.274	0.043144
A_44_P515792	Elmo1_predicted	XM_341532		-0.11	-1.274	0.231164
A_44_P726568	Ptpn3	XM_001055793		-0.11	-1.274	0.033094
A_44_P452062	Olr48_predicted	NM_001000122	293188	-0.11	-1.274	0.140783
A_44_P881253	Pqlc1	NM_001013189	361352	-0.11	-1.274	0.138445
A_44_P1005536	L3mbtl3_predicted	XM_220113		-0.11	-1.274	0.101222
A_44_P195185	BQ205697	BQ205697	291927	-0.11	-1.274	0.181527
A_44_P216395	Gadd45a	NM_024127	25112	-0.11	-1.274	0.125004
A_44_P362954	Cat	NM_012520	24248	-0.11	-1.274	0.121282
A_44_P348778	AW917950	AW917950	315222	-0.11	-1.275	0.108726
A_44_P500907	Hmox2	NM_024387	79239	-0.11	-1.275	0.044144
A_42_P614711	BF548552	BF548552		-0.11	-1.275	0.044979
A_44_P355663	AI044782	AI044782	24232	-0.11	-1.275	0.122605
A_44_P1018662	RGD1565095_predicted	XM_001057297		-0.11	-1.275	0.075625
A_43_P21135	Wdr20	XM_234540	314453	-0.11	-1.275	0.076185
A_44_P557338	RGD1310132	XM_342238		-0.11	-1.275	0.378705
A_43_P20537	Slc35c1_predicted	XM_230292		-0.11	-1.275	0.077751
A_44_P437346	AW435442	AW435442	260321	-0.11	-1.275	0.103789
A_44_P996557	AW143102	AW143102		-0.11	-1.275	0.155625
A_44_P414484	Tph1	XM_341862		-0.11	-1.275	0.286344
A_44_P320812	Rec8L1	NM_001011916	290227	-0.11	-1.275	0.174736
A_44_P538012	BG672309	BG672309		-0.11	-1.275	0.224635
A_44_P1028132	BF564888	BF564888		-0.11	-1.275	0.253774
A_44_P395471	CK843267	CK843267		-0.11	-1.275	0.087043
A_44_P341319	BM986517	BM986517	94267	-0.11	-1.275	0.055342
A_43_P19216	RGD1306773	NM_001039610	315863	-0.11	-1.276	0.047301
A_44_P1003825	Irf3	NM_001006969	292892	-0.11	-1.276	0.054537
A_44_P161531	LOC679173	XM_001055055		-0.11	-1.276	0.111815

A_44_P583834	Cobl_predicted	XM_001053778		-0.11	-1.276	0.159959
A_43_P12384	Birc3	NM_023987	78971	-0.11	-1.276	0.324385
A_44_P243153	Ncoa6	XM_342552	116464	-0.11	-1.276	0.031239
A_44_P943136	AW917153	AW917153	367323	-0.11	-1.276	0.227849
A_43_P16842	Clpx	BC085867	300786	-0.11	-1.276	0.123989
A_44_P342187	Edg5	NM_017192	29415	-0.11	-1.276	0.113667
A_43_P13141	Rasgrp4	NM_130824	170668	-0.11	-1.276	0.169828
A_43_P17128	RGD1306772_predicted	XM_213787		-0.11	-1.276	0.212177
A_44_P347660	AA893226	AA893226	292401	-0.11	-1.276	0.261294
A_44_P318140	AA962998	AA962998		-0.11	-1.276	0.107196
A_44_P373039	XM_229903	XM_229903		-0.11	-1.276	0.090592
A_44_P281678	Ccdc12_predicted	XM_343490		-0.11	-1.277	0.150706
A_42_P483463	AI172502	AI172502		-0.11	-1.277	0.041221
A_44_P260423	A_44_P260423	A_44_P260423		-0.11	-1.277	0.206705
A_43_P15300	Pigm	NM_024144	79112	-0.11	-1.277	0.22392
A_44_P860396	RGD1566064_predicted	XM_341141	360865	-0.11	-1.277	0.297363
A_44_P269478	Pcp2	XM_221787		-0.11	-1.277	0.209617
A_44_P399097	BE113101	BE113101	316640	-0.11	-1.277	0.132271
A_44_P1021808	RGD1561796_predicted	XM_340757	360483	-0.11	-1.277	0.060516
A_44_P557199	Mlycd	NM_053477	85239	-0.11	-1.277	0.026095
A_44_P303619	AA859990	AA859990	293497	-0.11	-1.277	0.316065
A_44_P529581	Okl38	NM_138504	171493	-0.11	-1.278	0.056766
A_44_P1059369	Gdf11	XM_343148	29454	-0.11	-1.278	0.200685
A_43_P16440	Tmem30a	NM_001004248	300857	-0.11	-1.278	0.131232
A_44_P379815	RGD1311361	XM_223187	305171	-0.11	-1.278	0.183104
A_44_P118531	AW919008	AW919008	361692	-0.11	-1.278	0.134037
A_44_P330542	Birc2	NM_021752	60371	-0.11	-1.278	0.052154
A_44_P115841	AI013149	AI013149	113947	-0.11	-1.278	0.161294
A_44_P461130	Krt2-5	NM_183333	369017	-0.11	-1.278	0.10883
A_44_P1023847	RGD1307160_predicted	XM_219953	309451	-0.11	-1.278	0.048155
A_44_P438868	RGD1305846_predicted	XM_237403	316602	-0.11	-1.278	0.150846
A_43_P14623	Pcaf	NM_001024252	301164	-0.11	-1.278	0.067512
A_44_P589546	AA944569	AA944569	24666	-0.11	-1.278	0.228724
A_44_P667469	ENSRNOT00000021161	ENSRNOT00000021161		-0.11	-1.278	0.135024
A_44_P309596	XM_234755	XM_234755		-0.11	-1.278	0.392535
A_44_P668572	TC520632	TC520632		-0.11	-1.278	0.343606
A_44_P100904	XM_345792	XM_345792		-0.11	-1.278	0.068998
A_44_P340393	RGD1565753_predicted	XM_235086		-0.11	-1.278	0.107413
A_44_P1049252	Naprt1	NM_207609	315085	-0.11	-1.279	0.090218
A_44_P351181	Cml1	NM_133558	171084	-0.11	-1.279	0.239842
A_44_P239033	CB612540	CB612540		-0.11	-1.279	0.079151
A_44_P621633	Dhrs3	NM_001037199	313689	-0.11	-1.279	0.062556
A_44_P409518	Gmpr	NM_057188	117533	-0.11	-1.279	0.319624
A_44_P403866	Gata2	NM_033442	25159	-0.11	-1.279	0.437408
A_44_P393343	St3gal6	NM_207602	304023	-0.11	-1.279	0.280859
A_44_P392287	BF291148	BF291148	500294	-0.11	-1.279	0.120538
A_44_P117364	BQ208532	BQ208532	362110	-0.11	-1.280	0.084315
A_44_P441329	AA956212	AA956212		-0.11	-1.280	0.069796
A_43_P16774	Acat2	NM_001006995	308100	-0.11	-1.280	0.078255
A_44_P436312	LOC680117	XM_001054603	680117	-0.11	-1.280	0.132602
A_44_P576351	Fvt1_predicted	XM_341106		-0.11	-1.280	0.046332
A_44_P965229	TC533445	TC533445		-0.11	-1.280	0.042323
A_44_P595076	TC527483	TC527483		-0.11	-1.280	0.507885
A_42_P555131	RGD1306626	NM_001039612	362319	-0.11	-1.280	0.184132
A_44_P1057412	Ddx25	NM_031630	58856	-0.11	-1.280	0.240273
A_43_P10616	LOC688211	XM_001081514		-0.11	-1.280	0.066383
A_44_P214970	Asgr2	NM_017189	29403	-0.11	-1.281	0.157798
A_44_P899467	XM_574695	XM_574695		-0.11	-1.281	0.287391
A_44_P854745	TC543176	TC543176		-0.11	-1.281	0.08128
A_43_P10257	Vkorc1	NM_203335	309004	-0.11	-1.281	0.09655
A_44_P200630	AA851208	AA851208	501702	-0.11	-1.281	0.080364
A_44_P145511	AW142459	AW142459	309105	-0.11	-1.281	0.042899
A_44_P250030	Bmp6	NM_013107	25644	-0.11	-1.281	0.281369

A_43_P19852	RGD1561646_predicted	XM_573540		-0.11	-1.281	0.044694
A_44_P305092	AI113064	AI113064		-0.11	-1.281	0.14639
A_42_P813263	Bloc1s1_predicted	XM_213833		-0.11	-1.281	0.060055
A_44_P265030	A_44_P265030	A_44_P265030		-0.11	-1.281	0.055775
A_44_P256921	Elovl4_predicted	XM_236476	315851	-0.11	-1.282	0.204526
A_44_P456752	ENSRNOT00000014279	ENSRNOT00000014279		-0.11	-1.282	0.075078
A_44_P261761	BM392398	BM392398	290644	-0.11	-1.282	0.246267
A_43_P13041	Nr0b2	NM_057133	117274	-0.11	-1.282	0.10508
A_43_P17005	RGD1305387	NM_001025697	313200	-0.11	-1.282	0.042546
A_44_P137194	AA900990	AA900990	116501	-0.11	-1.282	0.29393
A_42_P516379	CF106882	CF106882		-0.11	-1.282	0.19449
A_44_P1054501	Eif2ak4_predicted	XM_230462		-0.11	-1.282	0.157693
A_44_P142184	L20993	L20993		-0.11	-1.282	0.24565
A_44_P563281	TC546410	TC546410		-0.11	-1.282	0.103844
A_44_P732262	TC532646	TC532646		-0.11	-1.282	0.088813
A_44_P496211	Hnrpu	NM_057139	117280	-0.11	-1.282	0.046474
A_44_P134496	AA997849	AA997849		-0.11	-1.282	0.449546
A_44_P372468	Pcdhga11	NM_001037153	116782	-0.11	-1.282	0.293941
A_44_P108028	XM_217271	XM_217271		-0.11	-1.282	0.130694
A_44_P200846	Sfxn5	NM_153298	261737	-0.11	-1.283	0.186889
A_44_P397163	Mlc2	NM_001035252	363925	-0.11	-1.283	0.10562
A_44_P974553	A_44_P974553	A_44_P974553		-0.11	-1.283	0.024443
A_44_P981019	TC541760	TC541760		-0.11	-1.283	0.266893
A_44_P254016	Avp	NM_016992	24221	-0.11	-1.283	0.236664
A_44_P819359	BG668084	BG668084	288762	-0.11	-1.283	0.128263
A_44_P517576	Sel1h	NM_177933	314352	-0.11	-1.283	0.042348
A_44_P1052361	Slc39a8	NM_001011952	295455	-0.11	-1.283	0.094232
A_44_P381770	Olr1130_predicted	NM_001000428	300400	-0.11	-1.283	0.218235
A_44_P473673	RGD1565180_predicted	XM_343497		-0.11	-1.283	0.132996
A_44_P274641	Itgb3bp	NM_001013213	362548	-0.11	-1.284	0.047802
A_44_P729436	TC534707	TC534707		-0.11	-1.284	0.495409
A_44_P293791	Atp6v0a1	NM_031604	29757	-0.11	-1.284	0.039336
A_43_P13809	AA955156	AA955156	304131	-0.11	-1.284	0.278601
A_44_P269280	Pou2f3	XM_343378		-0.11	-1.285	0.185848
A_44_P271405	Pxmp4	NM_172223	282634	-0.11	-1.285	0.068233
A_43_P16959	Zfp403	NM_001004273	360584	-0.11	-1.285	0.026367
A_43_P12855	Olfm1	NM_053573	93667	-0.11	-1.285	0.088563
A_44_P264145	Pdzk6_predicted	XM_342237	361938	-0.11	-1.285	0.25812
A_44_P158635	Bet1l	NM_019368	54400	-0.11	-1.285	0.047831
A_44_P397962	Olr62_predicted	NM_001000129	293201	-0.11	-1.285	0.379094
A_44_P1024567	Mxd4_predicted	XM_001065075		-0.11	-1.285	0.029438
A_42_P459431	Bphl	NM_001037206	361239	-0.11	-1.285	0.143195
A_44_P229746	RGD1307429_predicted	XM_343096	362770	-0.11	-1.285	0.205888
A_43_P10209	Dok4_predicted	XM_341642		-0.11	-1.285	0.054407
A_44_P290558	Fev	NM_144753	246271	-0.11	-1.285	0.077949
A_44_P558961	CA505525	CA505525	363836	-0.11	-1.285	0.245836
A_43_P19317	RGD1310166_predicted	XM_220804	303395	-0.11	-1.285	0.248757
A_44_P391134	Olr1365_predicted	NM_214824	287081	-0.11	-1.285	0.394603
A_44_P324574	AA817956	AA817956	289264	-0.11	-1.285	0.078539
A_43_P13744	CB547042	CB547042		-0.11	-1.285	0.255998
A_42_P482349	Rab3gap2	NM_001040154	289350	-0.11	-1.285	0.045526
A_42_P465144	Dennd2d_predicted	XM_227570		-0.11	-1.285	0.062311
A_44_P171064	RGD1308307_predicted	XM_226475		-0.11	-1.286	0.025544
A_43_P20057	CB545427	CB545427	362501	-0.11	-1.286	0.088381
A_44_P315437	BG669126	BG669126		-0.11	-1.286	0.081926
A_44_P292785	AF303035	AF303035		-0.11	-1.286	0.112737
A_43_P11435	Apoc3	NM_012501	24207	-0.11	-1.286	0.13248
A_44_P384871	LOC300024	XM_216959	300024	-0.11	-1.286	0.394015
A_44_P354934	AI713313	AI713313	689995	-0.11	-1.286	0.095175
A_43_P18423	Sf3b1	XM_343570	84486	-0.11	-1.286	0.055593
A_44_P374247	BF395647	BF395647	29557	-0.11	-1.286	0.105806
A_44_P1046097	RGD1306063_predicted	XM_214137		-0.11	-1.286	0.032048
A_42_P674200	Rnf25	NM_001012004	301515	-0.11	-1.286	0.124653

A_44_P798851	BI295850	BI295850		-0.11	-1.287	0.072776
A_44_P554377	RGD1306508_predicted	XM_214121		-0.11	-1.287	0.113842
A_44_P150857	BQ191500	BQ191500	691393	-0.11	-1.287	0.069691
A_44_P652265	RGD1561981_predicted	XM_575601		-0.11	-1.287	0.113689
A_44_P136911	Rnpc2	BC078917	362251	-0.11	-1.287	0.095227
A_42_P815499	BM392235	BM392235	302965	-0.11	-1.287	0.10131
A_44_P823568	TC542650	TC542650		-0.11	-1.287	0.405114
A_43_P11727	Scn5a	NM_013125	25665	-0.11	-1.287	0.11607
A_44_P415518	Dnajc3	NM_022232	63880	-0.11	-1.287	0.083769
A_44_P302551	Cd320	NM_001014201	362851	-0.11	-1.287	0.118952
A_42_P683614	Kcnj11	NM_031358	83535	-0.11	-1.287	0.612243
A_44_P213088	XM_227325	XM_227325		-0.11	-1.287	0.049195
A_44_P293463	H34946	H34946	360822	-0.11	-1.288	0.134591
A_42_P454824	Ap2a1_predicted	XM_218624		-0.11	-1.288	0.152426
A_42_P538135	Lhb	NM_012858	25329	-0.11	-1.288	0.122394
A_44_P203804	A_44_P203804	A_44_P203804		-0.11	-1.288	0.210706
A_44_P130504	Pth	NM_017044	24694	-0.11	-1.288	0.133367
A_44_P376043	BF390703	BF390703		-0.11	-1.288	0.082757
A_44_P727824	Ppp2r5a_predicted	XM_232413		-0.11	-1.288	0.093875
A_44_P103160	Inpp5b	XM_342909	362590	-0.11	-1.288	0.023333
A_43_P12845	Lrp3	NM_053541	89787	-0.11	-1.288	0.265673
A_43_P12703	Ndrg4	NM_031967	64457	-0.11	-1.288	0.264104
A_43_P16998	Foxo3a_predicted	XM_215421		-0.11	-1.288	0.023858
A_44_P720147	CB792426	CB792426	689019	-0.11	-1.288	0.191693
A_44_P269425	Efcbp2	NM_133415	170928	-0.11	-1.288	0.067239
A_44_P188901	BQ199946	BQ199946	499782	-0.11	-1.289	0.045326
A_44_P104687	Kcnc3	M84211	117101	-0.11	-1.289	0.266596
A_44_P110117	Ndufb7_predicted	XM_341664		-0.11	-1.289	0.020685
A_44_P299884	RGD1562874_predicted	XM_345327	366005	-0.11	-1.289	0.123634
A_43_P19101	RGD1562626_predicted	XM_220861		-0.11	-1.289	0.036927
A_44_P567248	A_44_P567248	A_44_P567248		-0.11	-1.290	0.067661
A_44_P1056599	XM_213637	XM_213637		-0.11	-1.290	0.116936
A_44_P482420	Olr1235_predicted	NM_001000447	300560	-0.11	-1.290	0.191776
A_44_P192641	AA819138	AA819138	361764	-0.11	-1.290	0.30364
A_44_P234023	XM_344131	XM_344131		-0.11	-1.290	0.066913
A_44_P250143	kif13B	NM_213626	305967	-0.11	-1.290	0.032084
A_44_P838888	Brip1_predicted	XM_340869	360588	-0.11	-1.290	0.282573
A_44_P146444	RGD1566077_predicted	XM_001076825		-0.11	-1.290	0.124095
A_44_P242591	AA900531	AA900531		-0.11	-1.291	0.043485
A_44_P477577	Rab1	NM_031090	81754	-0.11	-1.291	0.085576
A_44_P447394	Sox10	NM_019193	29361	-0.11	-1.291	0.224565
A_44_P475744	Tpd52_predicted	XM_215524		-0.11	-1.291	0.126799
A_44_P920712	LOC499337	XM_001078906		-0.11	-1.291	0.024108
A_44_P193244	AI009391	AI009391	687090	-0.11	-1.291	0.05825
A_44_P369808	Gstk1	NM_181371	297029	-0.11	-1.291	0.166902
A_44_P286816	BF556330	BF556330		-0.11	-1.291	0.223914
A_44_P275189	LOC682206	XM_001060419		-0.11	-1.291	0.023786
A_44_P436115	Sox7_predicted	XM_224283		-0.11	-1.291	0.515048
A_44_P223299	BF387487	BF387487	499967	-0.11	-1.291	0.135629
A_44_P638565	TC559341	TC559341		-0.11	-1.291	0.046767
A_44_P993051	Plunc	NM_172031	246238	-0.11	-1.292	0.147795
A_44_P177333	BG666117	BG666117		-0.11	-1.292	0.121004
A_44_P505435	AA819024	AA819024	29573	-0.11	-1.292	0.35616
A_44_P341120	AI103284	AI103284	299339	-0.11	-1.292	0.09676
A_44_P1048939	S100a5_predicted	XM_215608		-0.11	-1.292	0.115027
A_44_P840715	Galm	NM_001007704	313843	-0.11	-1.292	0.209373
A_44_P154802	AW524480	AW524480	502414	-0.11	-1.292	0.140736
A_43_P10851	Sh3glb1	NM_001011929	292156	-0.11	-1.292	0.049422
A_44_P208857	Mlc1_predicted	XM_235558		-0.11	-1.292	0.269713
A_44_P518547	Pitpnm3_predicted	XM_220629	287467	-0.11	-1.292	0.066842
A_43_P17693	Son	XM_221656	304092	-0.11	-1.292	0.022478
A_43_P14857	Tmbim4	NM_199116	362884	-0.11	-1.293	0.040343
A_44_P459092	BX883048	BX883048		-0.11	-1.293	0.053143

A_44_P459412	Spap1_predicted	XM_227483		-0.11	-1.293	0.028057
A_44_P714691	TC521231	TC521231		-0.11	-1.293	0.052411
A_44_P322960	Araf	NM_022532	64363	-0.11	-1.293	0.020981
A_44_P442838	AF216218	AF216218		-0.11	-1.293	0.138231
A_44_P486659	Mtmr2_predicted	XM_001068538		-0.11	-1.293	0.034447
A_44_P240953	Pcsk3	NM_019331	54281	-0.11	-1.293	0.298697
A_44_P188441	Myl3	NM_012606	24585	-0.11	-1.294	0.099461
A_44_P1011391	Usp40	XM_237371	316599	-0.11	-1.294	0.075375
A_44_P183260	AI232145	AI232145	292781	-0.11	-1.294	0.175798
A_44_P459987	CB546964	CB546964		-0.11	-1.294	0.282644
A_44_P552014	AI102717	AI102717		-0.11	-1.294	0.052461
A_43_P12927	Dusp1	NM_053769	114856	-0.11	-1.294	0.147353
A_44_P1054991	Gli1	XM_345832	140589	-0.11	-1.294	0.312948
A_42_P461726	Pwwp1	NM_133549	171074	-0.11	-1.294	0.379327
A_44_P371653	AI578866	AI578866		-0.11	-1.294	0.077098
A_44_P132111	BE100899	BE100899	360604	-0.11	-1.294	0.056165
A_44_P313230	LOC685888	XM_001062701	685888	-0.11	-1.294	0.335578
A_44_P758991	Rybp_predicted	XM_232220		-0.11	-1.295	0.068492
A_44_P274475	LOC291466	XR_007620	291466	-0.11	-1.295	0.274739
A_44_P683608	TC570367	TC570367		-0.11	-1.295	0.016978
A_44_P252178	BQ780816	BQ780816	691534	-0.11	-1.295	0.093795
A_44_P108901	AI170380	AI170380	192155	-0.11	-1.295	0.095898
A_44_P154182	Cyp2s1	XM_218347		-0.11	-1.295	0.195444
A_44_P314013	XM_224065	XM_224065		-0.11	-1.295	0.176621
A_43_P14809	Syde1_predicted	XM_001077806		-0.11	-1.295	0.183034
A_43_P23164	Slc22a13_predicted	XM_236685	316062	-0.11	-1.295	0.434498
A_44_P435067	BQ206852	BQ206852	312728	-0.11	-1.295	0.167848
A_44_P729525	RGD1562339_predicted	XM_579785	498107	-0.11	-1.296	0.093434
A_44_P264766	Itpr2	NM_031046	81678	-0.11	-1.296	0.359215
A_44_P605416	BM386674	BM386674	295265	-0.11	-1.296	0.186431
A_44_P474704	AW253642	AW253642	311802	-0.11	-1.296	0.075015
A_44_P1005988	XM_214619	XM_214619		-0.11	-1.296	0.111728
A_44_P370411	A_44_P370411	A_44_P370411		-0.11	-1.296	0.108947
A_44_P791812	Ncoa3	XM_215947	84584	-0.11	-1.296	0.184091
A_42_P621882	Copa	XM_222899		-0.11	-1.296	0.173314
A_44_P229938	AI178652	AI178652	308267	-0.11	-1.296	0.219808
A_44_P777051	TC523015	TC523015		-0.11	-1.296	0.068211
A_43_P20024	RGD1560123_predicted	XM_343498	363159	-0.11	-1.296	0.038354
A_43_P14265	TC523554	TC523554		-0.11	-1.296	0.165096
A_43_P15862	Gabrq	NM_031733	65187	-0.11	-1.296	0.274271
A_44_P274514	Dhx35_predicted	XM_342565		-0.11	-1.297	0.154914
A_44_P866788	LOC498222	XM_001058496		-0.11	-1.297	0.157548
A_44_P529299	Rtkn	NM_184046	297383	-0.11	-1.297	0.434392
A_44_P510972	RGD1311595	XM_219779	309307	-0.11	-1.297	0.066953
A_44_P382255	RGD1561985_predicted	XM_001054053		-0.11	-1.297	0.290908
A_42_P727584	Mapk6	NM_031622	58840	-0.11	-1.297	0.046834
A_44_P504053	LOC302782	XR_008348	302782	-0.11	-1.297	0.074871
A_43_P12577	Bcl2l1	NM_031535	24888	-0.11	-1.297	0.237459
A_44_P315183	AW916081	AW916081		-0.11	-1.297	0.03066
A_44_P522779	Mesp1_predicted	XM_218826		-0.11	-1.298	0.143414
A_44_P494470	RGD1560873_predicted	XM_001058121		-0.11	-1.298	0.338561
A_44_P106138	AA858748	AA858748		-0.11	-1.298	0.118098
A_43_P11055	TC551244	TC551244		-0.11	-1.298	0.060431
A_43_P12323	N5	NM_022857	64825	-0.11	-1.298	0.184575
A_43_P14157	Glud1	NM_012570	24399	-0.11	-1.298	0.034815
A_44_P431661	Vill_predicted	XM_217296	301057	-0.11	-1.298	0.10314
A_43_P19201	Dcun1d1_predicted	XM_227048		-0.11	-1.298	0.019968
A_44_P356078	Pgm1	NM_017033	24645	-0.11	-1.298	0.086789
A_44_P269582	Dusp8_predicted	XM_341963		-0.11	-1.298	0.05752
A_44_P962553	TC548118	TC548118		-0.11	-1.299	0.076447
A_44_P560113	A_44_P560113	A_44_P560113		-0.11	-1.299	0.138202
A_44_P299699	RGD1309948	NM_001013896	293098	-0.11	-1.299	0.015221
A_44_P466842	Hbq1_predicted	XM_001061675		-0.11	-1.299	0.049732

A_44_P314260	A_44_P314260	A_44_P314260		-0.11	-1.299	0.073057
A_44_P1042360	XM_342752	XM_342752		-0.11	-1.299	0.342064
A_44_P299953	XM_226409	XM_226409		-0.11	-1.299	0.186318
A_44_P307978	Cited2	NM_053698	114490	-0.11	-1.299	0.192354
A_43_P17903	RGD1311084_predicted	XM_231131		-0.11	-1.299	0.041567
A_44_P372892	RGD1305276	NM_001014149	361313	-0.11	-1.299	0.075826
A_42_P691339	ENSRNOT00000018076	ENSRNOT00000018076		-0.11	-1.299	0.152582
A_44_P438744	XM_233718	XM_233718		-0.11	-1.299	0.031931
A_44_P853540	TC522498	TC522498		-0.11	-1.299	0.031908
A_44_P842451	TC565812	TC565812		-0.11	-1.299	0.045031
A_43_P16099	LOC686059	XM_574411	114207	-0.11	-1.299	0.522865
A_44_P343037	CB546385	CB546385	680452	-0.11	-1.299	0.12863
A_44_P141649	AI171448	AI171448	691427	-0.11	-1.300	0.105999
A_43_P17060	RGD1562059_predicted	XM_001053262		-0.11	-1.300	0.145447
A_44_P548107	Nucb1	NM_053463	84595	-0.11	-1.300	0.082636
A_44_P100476	Mael_predicted	XM_344169		-0.11	-1.300	0.051114
A_44_P136526	AW918520	AW918520		-0.11	-1.300	0.029046
A_44_P429794	AI137938	AI137938	364388	-0.11	-1.300	0.055342
A_44_P677285	LOC360910	NM_001014133	360997	-0.11	-1.300	0.071752
A_44_P456479	Col23a1	NM_181636	353303	-0.11	-1.300	0.07838
A_44_P450500	LOC301231	XM_001059565		-0.11	-1.300	0.129173
A_44_P157289	BE117653	BE117653		-0.11	-1.301	0.130055
A_44_P742628	BI395631	BI395631		-0.11	-1.301	0.038271
A_44_P335452	Scand1_predicted	XM_342558		-0.11	-1.301	0.019624
A_44_P368011	XM_224048	XM_224048		-0.11	-1.301	0.350243
A_44_P409654	Amacr	NM_012816	25284	-0.11	-1.301	0.022083
A_44_P520290	RGD1309762_predicted	XM_222205	304503	-0.11	-1.301	0.027886
A_43_P21715	RGD1305062	XM_221694	304132	-0.11	-1.301	0.042661
A_44_P1040966	XM_343275	XM_343275		-0.11	-1.301	0.139237
A_44_P729882	TC555464	TC555464		-0.11	-1.301	0.263405
A_43_P13079	Cacng6	NM_080694	140727	-0.11	-1.301	0.049256
A_44_P214196	BF398408	BF398408		-0.11	-1.301	0.272774
A_42_P671800	Ca5a	NM_019293	54233	-0.11	-1.301	0.094726
A_44_P149789	XM_215921	XM_215921		-0.11	-1.301	0.122411
A_44_P387385	BM389833	BM389833	308589	-0.11	-1.301	0.09884
A_44_P106053	AI575648	AI575648	301570	-0.11	-1.302	0.174805
A_44_P316358	RGD1310470	XM_223229	305230	-0.11	-1.302	0.099421
A_44_P279095	Ascc1	NM_001007632	294512	-0.11	-1.302	0.132264
A_44_P459543	RGD1308461_predicted	XM_226525	307901	-0.11	-1.302	0.204571
A_44_P118540	Erbb2	NM_017003	24337	-0.11	-1.302	0.055882
A_44_P171506	Trim14_predicted	XM_001072308		-0.11	-1.302	0.116749
A_44_P641532	TC563752	TC563752		-0.11	-1.302	0.052597
A_43_P21635	LOC686059	XM_574411	114207	-0.11	-1.302	0.039449
A_44_P452182	Pcdhb13	XM_001055698	307489	-0.11	-1.302	0.193847
A_44_P276803	LOC683099	XM_001062864		-0.11	-1.302	0.095093
A_44_P544447	AI045333	AI045333	293497	-0.11	-1.303	0.043918
A_44_P231583	Csen	NM_032462	65199	-0.11	-1.303	0.076851
A_44_P101096	AA819838	AA819838		-0.11	-1.303	0.040934
A_44_P362158	Rbm25_predicted	XM_345703		-0.12	-1.303	0.129769
A_44_P232319	BE105426	BE105426	498109	-0.12	-1.303	0.296933
A_44_P515538	A_44_P515538	A_44_P515538		-0.12	-1.303	0.046358
A_44_P128951	LOC688210	XM_001081513		-0.12	-1.303	0.118143
A_44_P788049	AA859335	AA859335	29388	-0.12	-1.303	0.282119
A_44_P539215	A_44_P539215	A_44_P539215		-0.12	-1.304	0.275721
A_44_P133170	RGD1311539_predicted	XM_214216	290322	-0.12	-1.304	0.052058
A_43_P21873	RGD1311859_predicted	XM_220974		-0.12	-1.304	0.175064
A_44_P779971	BF523525	BF523525		-0.12	-1.304	0.067635
A_44_P103412	BE118909	BE118909		-0.12	-1.304	0.126834
A_44_P1003785	RGD1309310_predicted	XM_221119	303677	-0.12	-1.304	0.033394
A_44_P788091	CK839277	CK839277		-0.12	-1.304	0.244225
A_44_P555005	Olr1472_predicted	NM_001000944	405286	-0.12	-1.304	0.1211
A_44_P480614	RGD1565098_predicted	XM_574191	498902	-0.12	-1.304	0.064177
A_44_P1071060	LOC501173	AY387068	501173	-0.12	-1.305	0.080477

A_44_P840751	TC517707	TC517707		-0.12	-1.305	0.05724
A_44_P316604	RGD1560237_predicted	XM_577668		-0.12	-1.305	0.157185
A_44_P383965	Glg1	NM_017211	29476	-0.12	-1.305	0.019282
A_44_P853085	RGD1308260_predicted	XM_001078269		-0.12	-1.305	0.058819
A_43_P10329	Abhd2_predicted	XM_214979		-0.12	-1.305	0.095936
A_44_P268787	LOC683428	XM_001065904		-0.12	-1.305	0.031243
A_44_P465205	BE328941	BE328941	290651	-0.12	-1.305	0.06119
A_43_P16216	Mrc2_predicted	XM_215737	295631	-0.12	-1.305	0.193936
A_43_P21147	Otog_predicted	XM_218577		-0.12	-1.305	0.192558
A_44_P652323	RGD1565225_predicted	XM_575856		-0.12	-1.305	0.119108
A_44_P410534	RGD1306881_predicted	XM_340933	360656	-0.12	-1.305	0.015036
A_42_P795688	RGD1305703	NM_001014191	362521	-0.12	-1.306	0.03974
A_43_P21626	Plcd3_predicted	XM_221004		-0.12	-1.306	0.107469
A_44_P977195	TC527218	TC527218		-0.12	-1.306	0.125004
A_44_P241223	XM_224708	XM_224708		-0.12	-1.306	0.538675
A_43_P14252	Rab5c_predicted	XM_213463		-0.12	-1.306	0.098907
A_44_P822051	ENSRNOT00000031814	ENSRNOT00000031814		-0.12	-1.306	0.117402
A_44_P842321	CA509111	CA509111		-0.12	-1.306	0.062109
A_44_P436633	BI284261	BI284261	171555	-0.12	-1.306	0.354447
A_44_P482346	Ptk2b	NM_017318	50646	-0.12	-1.306	0.14667
A_43_P13091	As3mt	NM_080890	140925	-0.12	-1.306	0.086322
A_44_P590730	ENSRNOT00000032954	ENSRNOT00000032954		-0.12	-1.306	0.038326
A_44_P199524	LOC688144	XM_001081287		-0.12	-1.306	0.07999
A_43_P11897	Gabrd	NM_017289	29689	-0.12	-1.306	0.194719
A_44_P340830	XM_343644	XM_343644		-0.12	-1.306	0.085284
A_44_P538576	Hoxc6	XM_345882	252885	-0.12	-1.306	0.222878
A_44_P116449	Rala	NM_031093	81757	-0.12	-1.306	0.033017
A_44_P189859	Zfp297b	NM_001012094	311872	-0.12	-1.307	0.035601
A_44_P590922	A_44_P590922	A_44_P590922		-0.12	-1.307	0.443396
A_44_P987846	Dpp8_predicted	XM_001075075		-0.12	-1.307	0.037928
A_44_P424452	RGD1560171_predicted	XM_346366	367949	-0.12	-1.307	0.084472
A_44_P207369	BE113217	BE113217	295234	-0.12	-1.307	0.205485
A_43_P14849	Azi2	NM_001025705	316051	-0.12	-1.307	0.019352
A_44_P463480	Ppp2r5b	NM_181379	309179	-0.12	-1.307	0.026417
A_44_P304202	Slc6a2	NM_031343	83511	-0.12	-1.307	0.159866
A_44_P381092	RGD1564833_predicted	XM_001060344		-0.12	-1.307	0.109743
A_44_P241583	Kif9_predicted	XM_236648		-0.12	-1.307	0.090956
A_44_P698400	RGD1561302_predicted	XM_573803		-0.12	-1.308	0.167397
A_44_P414661	Hadh2	NM_031682	63864	-0.12	-1.308	0.031657
A_43_P12294	Rem2	NM_022685	64626	-0.12	-1.308	0.168574
A_43_P11882	Htr2a	NM_017254	29595	-0.12	-1.308	0.308735
A_44_P159430	Cln6_predicted	XM_236325	315746	-0.12	-1.308	0.14667
A_44_P207891	CR754229	CR754229	301085	-0.12	-1.308	0.164209
A_44_P272605	AI234637	AI234637	361728	-0.12	-1.308	0.08682
A_42_P660334	Ssu72	NM_001025657	298681	-0.12	-1.308	0.119438
A_44_P221168	AF387339	AF387339		-0.12	-1.308	0.066386
A_44_P198705	H1f0	NM_012578	24437	-0.12	-1.308	0.051938
A_44_P319256	Kdelr3_predicted	XM_235478	315131	-0.12	-1.308	0.166605
A_44_P996124	Tlr4	NM_019178	29260	-0.12	-1.308	0.095947
A_44_P730048	TC539285	TC539285		-0.12	-1.309	0.098759
A_44_P254283	Map4k3	XM_343006	170920	-0.12	-1.309	0.100347
A_44_P162576	BE103919	BE103919	290322	-0.12	-1.309	0.036774
A_42_P497393	AI008316	AI008316		-0.12	-1.309	0.075155
A_44_P182220	Amigo3	NM_178144	316003	-0.12	-1.309	0.024263
A_44_P491393	Nudt7_predicted	XM_341693		-0.12	-1.309	0.127895
A_43_P12406	Arf4	NM_024151	79120	-0.12	-1.309	0.088222
A_44_P355565	BQ207763	BQ207763	300732	-0.12	-1.309	0.108487
A_44_P1013667	Kif7_predicted	XM_001065361		-0.12	-1.309	0.233313
A_44_P929787	TC554783	TC554783		-0.12	-1.309	0.264449
A_43_P11650	Pax6	NM_013001	25509	-0.12	-1.309	0.105357
A_44_P288443	Thsd1_predicted	XM_344542		-0.12	-1.309	0.38709
A_44_P135134	Hook3	XM_224952	306548	-0.12	-1.310	0.017332
A_44_P195817	AA850940	AA850940	64302	-0.12	-1.310	0.053481

A_44_P170864	RGD1311920_predicted	XM_223651	305544	-0.12	-1.310	0.112275
A_44_P532249	Fkbp5	NM_001012174	361810	-0.12	-1.310	0.033568
A_44_P576521	TC555494	TC555494		-0.12	-1.310	0.129218
A_44_P366142	Zbtb16	NM_001013181	353227	-0.12	-1.310	0.206341
A_43_P11200	RGD1561500_predicted	XM_235711	315327	-0.12	-1.310	0.058335
A_44_P731043	A_44_P731043	A_44_P731043		-0.12	-1.310	0.134943
A_44_P210874	A_44_P210874	A_44_P210874		-0.12	-1.310	0.360879
A_44_P534518	Abtb1	NM_001005902	297432	-0.12	-1.310	0.030317
A_44_P918275	TC562320	TC562320		-0.12	-1.310	0.27454
A_44_P497807	Penk-rs	NM_017139	29237	-0.12	-1.310	0.124069
A_44_P150877	RGD1560454_predicted	XM_001080836		-0.12	-1.310	0.037859
A_44_P540963	Ptpn5	NM_019253	29644	-0.12	-1.310	0.038958
A_42_P462379	AW144299	AW144299		-0.12	-1.310	0.282074
A_44_P421672	Ager	NM_053336	81722	-0.12	-1.310	0.159801
A_44_P836799	ENSRNOT00000002546	ENSRNOT00000002546		-0.12	-1.311	0.05058
A_44_P541011	Nr2f1	NM_031130	81808	-0.12	-1.311	0.037198
A_44_P707095	TC527939	TC527939		-0.12	-1.311	0.114626
A_43_P20191	Pgm3_predicted	XM_343442		-0.12	-1.311	0.06244
A_43_P17528	Akr1e1	NM_001008342	307091	-0.12	-1.311	0.045073
A_44_P297722	Yipf6	NM_001025747	363476	-0.12	-1.311	0.026127
A_44_P523451	BF282236	BF282236	309684	-0.12	-1.311	0.082588
A_44_P500906	Dlgh1	NM_012788	25252	-0.12	-1.311	0.02725
A_44_P419933	XM_220611	XM_220611		-0.12	-1.311	0.109369
A_44_P779296	LOC502684	NM_001025060	502684	-0.12	-1.311	0.255387
A_44_P757372	AW918237	AW918237		-0.12	-1.311	0.235938
A_44_P124361	AA924327	AA924327		-0.12	-1.312	0.243448
A_42_P558206	DV723837	DV723837		-0.12	-1.312	0.108987
A_44_P351861	TC542260	TC542260		-0.12	-1.312	0.025558
A_43_P12049	LOC56081	NM_019384	56081	-0.12	-1.312	0.362954
A_44_P384979	LOC366012	NM_001014257	366012	-0.12	-1.312	0.441127
A_44_P437565	AF295545	AF295545		-0.12	-1.312	0.12629
A_43_P21315	Bai3_predicted	XM_217367		-0.12	-1.312	0.155646
A_44_P185719	RGD1308772_predicted	XM_214237	290381	-0.12	-1.313	0.023604
A_42_P579577	Sec14l3	XM_341244	64543	-0.12	-1.313	0.130865
A_44_P364884	BE107113	BE107113	304709	-0.12	-1.313	0.059297
A_44_P300897	BM389611	BM389611		-0.12	-1.313	0.077618
A_44_P231444	Slc39a11	NM_001013042	287796	-0.12	-1.313	0.012975
A_44_P314634	Tjp1_predicted	XM_218747		-0.12	-1.313	0.095512
A_44_P251005	C1ql4_predicted	XM_235645		-0.12	-1.313	0.266934
A_44_P125409	XM_216889	XM_216889		-0.12	-1.313	0.077902
A_44_P273863	Ndufs6	NM_019223	29478	-0.12	-1.313	0.027991
A_44_P577773	TC524514	TC524514		-0.12	-1.313	0.046828
A_44_P636387	LOC690744	XM_001075478		-0.12	-1.314	0.109911
A_44_P307064	LOC683136	XM_001064619		-0.12	-1.314	0.12455
A_44_P196356	Idh3B	NM_053581	94173	-0.12	-1.314	0.113822
A_44_P667951	A_44_P667951	A_44_P667951		-0.12	-1.314	0.238278
A_44_P199741	AW435424	AW435424	363599	-0.12	-1.314	0.040625
A_44_P307883	RGD1563224_predicted	XM_001068872		-0.12	-1.314	0.035683
A_44_P140896	BF390790	BF390790		-0.12	-1.314	0.176028
A_44_P478222	XM_345361	XM_345361		-0.12	-1.314	0.185932
A_44_P419948	RGD1559974_predicted	XM_220903		-0.12	-1.314	0.178993
A_43_P14347	DV727333	DV727333		-0.12	-1.314	0.126433
A_44_P339626	Olr1475_predicted	NM_001000027	287487	-0.12	-1.314	0.339115
A_44_P214837	Hand2	NM_022696	64637	-0.12	-1.314	0.176028
A_44_P861705	BQ208684	BQ208684	408240	-0.12	-1.315	0.146124
A_43_P10709	Smarca3_predicted	XM_001061239		-0.12	-1.315	0.046163
A_44_P533623	Ndufs1	NM_001005550	301458	-0.12	-1.315	0.079253
A_44_P940535	BG378253	BG378253	500434	-0.12	-1.315	0.316336
A_44_P179114	AA943131	AA943131	501702	-0.12	-1.315	0.034424
A_44_P643802	A_44_P643802	A_44_P643802		-0.12	-1.315	0.099985
A_44_P253756	AA955616	AA955616		-0.12	-1.315	0.407717
A_44_P271779	Frmd4b	XM_232212	252858	-0.12	-1.315	0.070247
A_42_P774448	Prkcc	NM_012628	24681	-0.12	-1.315	0.129142

A_44_P158659	Olr213_predicted	NM_001000735	404994	-0.12	-1.316	0.163857
A_44_P896698	AW914836	AW914836		-0.12	-1.316	0.131991
A_44_P100197	MGC72997	NM_001009537	494339	-0.12	-1.316	0.057301
A_44_P470868	Acpt_predicted	XM_218609		-0.12	-1.316	0.206233
A_44_P671854	TC541878	TC541878		-0.12	-1.316	0.019344
A_44_P344777	RGD1562744_predicted	XM_230014		-0.12	-1.316	0.036945
A_44_P502440	AW921285	AW921285		-0.12	-1.316	0.032972
A_44_P388095	LOC297530	NM_199207	297530	-0.12	-1.316	0.065431
A_44_P296859	AA946063	AA946063	363869	-0.12	-1.316	0.337795
A_44_P240512	AI104107	AI104107	64372	-0.12	-1.316	0.048446
A_44_P884800	RGD1566169_predicted	XM_001072960		-0.12	-1.316	0.101174
A_44_P343619	CK844847	CK844847		-0.12	-1.316	0.11176
A_43_P21317	Zfp612_predicted	XM_226455		-0.12	-1.317	0.025092
A_44_P223630	Camk2n1	CB580765	287005	-0.12	-1.317	0.026267
A_43_P18970	Abcd1_predicted	XM_343840		-0.12	-1.317	0.142952
A_43_P17131	LOC681027	XM_001059994	681027	-0.12	-1.317	0.088877
A_44_P543288	Stx5a	NM_031704	65134	-0.12	-1.317	0.077023
A_44_P823711	LOC682077	XM_001059737		-0.12	-1.317	0.034303
A_44_P947734	TC532979	TC532979		-0.12	-1.317	0.320362
A_43_P17213	Abhd1	NM_001008520	313917	-0.12	-1.317	0.044959
A_44_P871196	TC531473	TC531473		-0.12	-1.317	0.111385
A_43_P12813	Cox4i2	NM_053472	84683	-0.12	-1.318	0.2685
A_44_P115293	Copz2_predicted	XM_001081315		-0.12	-1.318	0.113171
A_44_P161266	ENSRNOT00000047908	ENSRNOT00000047908		-0.12	-1.318	0.049676
A_44_P290887	XM_346972	XM_346972		-0.12	-1.318	0.100858
A_44_P807669	TC539961	TC539961		-0.12	-1.318	0.312142
A_44_P250816	A_44_P250816	A_44_P250816		-0.12	-1.318	0.043222
A_42_P656898	AA957809	AA957809		-0.12	-1.318	0.023961
A_44_P258241	Dbx2	XM_001053826	541457	-0.12	-1.318	0.329519
A_44_P643552	AI112132	AI112132		-0.12	-1.318	0.033015
A_44_P1013330	LOC363153	XM_343492	363154	-0.12	-1.318	0.047904
A_43_P15235	Plagl1	NM_012760	25157	-0.12	-1.319	0.404176
A_43_P14727	RGD1305045_predicted	XM_215787		-0.12	-1.319	0.090436
A_43_P11875	Grik1	NM_017241	29559	-0.12	-1.319	0.065412
A_42_P509314	Tubb3	NM_139254	246118	-0.12	-1.319	0.205604
A_44_P487003	Olr807_predicted	NM_001000851	405141	-0.12	-1.319	0.212601
A_44_P1071010	ENSRNOT00000050740	ENSRNOT00000050740		-0.12	-1.320	0.062395
A_44_P448875	BF407194	BF407194	317258	-0.12	-1.320	0.140245
A_44_P835618	BF391375	BF391375		-0.12	-1.320	0.209496
A_44_P271668	Icmt	NM_133310	170818	-0.12	-1.320	0.205832
A_44_P119594	XM_346004	XM_346004		-0.12	-1.320	0.048065
A_44_P971970	AI639448	AI639448	246323	-0.12	-1.320	0.191886
A_44_P917564	LOC683630	XM_001066803		-0.12	-1.320	0.039283
A_43_P12191	Pdcd4	NM_022265	64031	-0.12	-1.320	0.141688
A_44_P521587	Arhgef3_predicted	XM_001055379		-0.12	-1.321	0.171221
A_44_P797239	A_44_P797239	A_44_P797239		-0.12	-1.321	0.372911
A_44_P238850	XM_227332	XM_227332		-0.12	-1.321	0.136336
A_44_P798772	TC527746	TC527746		-0.12	-1.321	0.029883
A_44_P204081	XM_345751	XM_345751		-0.12	-1.321	0.365467
A_44_P356407	Fcho1_predicted	XM_214313		-0.12	-1.321	0.115931
A_44_P295018	Cnksr3	NM_001012061	308113	-0.12	-1.321	0.017445
A_44_P281523	Cdx4_predicted	XM_228569		-0.12	-1.321	0.259446
A_43_P17580	Pck2_predicted	XM_341319		-0.12	-1.321	0.053153
A_44_P265278	XM_234023	XM_234023		-0.12	-1.321	0.090057
A_44_P319035	RGD1565367_predicted	XM_231601	312226	-0.12	-1.321	0.190351
A_42_P460785	Amacr	NM_012816	25284	-0.12	-1.321	0.131064
A_44_P138927	AA819517	AA819517		-0.12	-1.322	0.202825
A_44_P161875	ENSRNOT00000030654	ENSRNOT00000030654		-0.12	-1.322	0.195347
A_44_P1034107	Akap8l	NM_001013946	299569	-0.12	-1.322	0.040401
A_44_P115886	RGD1309534	NM_001014206	363016	-0.12	-1.322	0.089981
A_44_P244373	BF545451	BF545451	302934	-0.12	-1.322	0.158261
A_44_P199358	RGD1561327_predicted	XM_229162	317593	-0.12	-1.322	0.026551
A_44_P328090	Wnt5b	XM_342747	282582	-0.12	-1.322	0.051475

A_44_P466523	LOC296117	NM_001013919	296117	-0.12	-1.322	0.052855
A_44_P482405	Olr1608_predicted	NM_001000093	290006	-0.12	-1.322	0.370802
A_44_P407720	Slc10a5	NM_001025280	310230	-0.12	-1.322	0.274676
A_44_P775779	LOC500370	XR_009045	500370	-0.12	-1.322	0.037697
A_42_P838347	Cacng2	NM_053351	84347	-0.12	-1.322	0.049692
A_44_P884935	Plekhh1_predicted	XM_001080885		-0.12	-1.322	0.054363
A_44_P908788	CR463492	CR463492		-0.12	-1.323	0.248994
A_44_P930814	TC559381	TC559381		-0.12	-1.323	0.067759
A_44_P306156	RGD1560873_predicted	XM_001058121		-0.12	-1.323	0.115187
A_44_P321899	BI296771	BI296771		-0.12	-1.323	0.105282
A_44_P412452	Sh3pxd2a_predicted	XM_219971		-0.12	-1.323	0.146945
A_44_P341816	Kiss1	NM_181692	289023	-0.12	-1.323	0.106972
A_42_P690349	Smoc2_predicted	XM_214777		-0.12	-1.323	0.35491
A_44_P684022	TC555564	TC555564		-0.12	-1.323	0.059671
A_44_P620950	A_44_P620950	A_44_P620950		-0.12	-1.323	0.042893
A_44_P352129	Ankrd23_predicted	XM_237094		-0.12	-1.323	0.099701
A_44_P325962	XM_223190	XM_223190		-0.12	-1.323	0.047746
A_44_P555174	L20983	L20983		-0.12	-1.323	0.146462
A_44_P269980	Sp8_predicted	XM_234724	299499	-0.12	-1.323	0.099152
A_44_P356345	A_44_P356345	A_44_P356345		-0.12	-1.323	0.073676
A_44_P323900	LOC686184	XM_001071552		-0.12	-1.323	0.090804
A_44_P159234	RGD1311344_predicted	XM_230641		-0.12	-1.323	0.134052
A_44_P139633	AW917590	AW917590		-0.12	-1.324	0.0794
A_44_P285155	BG381287	BG381287	296649	-0.12	-1.324	0.11761
A_44_P269857	RGD1564868_predicted	XM_342788	362465	-0.12	-1.324	0.119752
A_44_P855773	DV723425	DV723425		-0.12	-1.324	0.042706
A_44_P281710	RGD1561796_predicted	XM_340757	360483	-0.12	-1.324	0.073033
A_44_P126681	BF558289	BF558289		-0.12	-1.324	0.089176
A_44_P206434	RGD1563803_predicted	XM_216850	299625	-0.12	-1.324	0.178334
A_44_P466108	Nfib	XM_342854		-0.12	-1.325	0.057371
A_44_P463061	BG373980	BG373980	299938	-0.12	-1.325	0.101097
A_44_P299550	Cldn4	NM_001012022	304407	-0.12	-1.325	0.099908
A_44_P554973	Cacna1g	NM_031601	29717	-0.12	-1.325	0.116402
A_44_P500975	Olr1639_predicted	NM_001000833	405122	-0.12	-1.325	0.080436
A_44_P596376	TC550080	TC550080		-0.12	-1.325	0.077905
A_44_P1032453	Ppa2_predicted	XM_227690	310856	-0.12	-1.325	0.018779
A_44_P1043555	XM_216326	XM_216326		-0.12	-1.325	0.250305
A_44_P211069	RGD1306119_predicted	XM_234404	314306	-0.12	-1.326	0.025544
A_44_P172587	AI145006	AI145006	291874	-0.12	-1.326	0.062791
A_44_P1046713	Cog4_predicted	XM_341688		-0.12	-1.326	0.017931
A_44_P396115	Glrp1_predicted	XM_227165		-0.12	-1.326	0.098542
A_44_P579569	Sos2	XM_001080400		-0.12	-1.326	0.037485
A_44_P309669	RGD1563984_predicted	XM_235695		-0.12	-1.326	0.051421
A_44_P516896	AI555906	AI555906	314374	-0.12	-1.326	0.143057
A_44_P267212	A_44_P267212	A_44_P267212		-0.12	-1.326	0.373507
A_44_P777928	TC542113	TC542113		-0.12	-1.326	0.037452
A_44_P385744	AI407974	AI407974	305310	-0.12	-1.326	0.146358
A_44_P444724	AI175764	AI175764	246074	-0.12	-1.326	0.117653
A_44_P286719	Snx25	XM_224863	306471	-0.12	-1.326	0.235806
A_43_P16096	Olr1370	NM_001000979	405334	-0.12	-1.327	0.064304
A_44_P868381	TC536217	TC536217		-0.12	-1.327	0.02943
A_44_P977792	RGD1562860_predicted	XM_341223	360945	-0.12	-1.327	0.027781
A_44_P206315	Wwp1	NM_001024757	297930	-0.12	-1.327	0.142888
A_44_P541587	Fbxo9	NM_001011998	300849	-0.12	-1.327	0.040882
A_44_P201428	Smarca3_predicted	XM_215728		-0.12	-1.327	0.055274
A_44_P436383	XM_346235	XM_346235		-0.12	-1.327	0.263011
A_42_P543670	Grik4	NM_012572	24406	-0.12	-1.327	0.224461
A_44_P947182	TC563438	TC563438		-0.12	-1.327	0.122612
A_44_P260655	A_44_P260655	A_44_P260655		-0.12	-1.327	0.022657
A_44_P864079	BF394271	BF394271		-0.12	-1.327	0.073545
A_44_P446774	AI030347	AI030347	309177	-0.12	-1.327	0.221976
A_43_P23245	Pnma2_predicted	XM_224322		-0.12	-1.327	0.043877
A_44_P168734	XM_342825	XM_342825		-0.12	-1.328	0.179255

A_44_P224714	RGD1566017_predicted	XM_001069000		-0.12	-1.328	0.022476
A_44_P771052	Slc41a3	NM_001037492	641603	-0.12	-1.328	0.253532
A_44_P475752	Ell_predicted	XM_224727		-0.12	-1.328	0.011723
A_42_P620448	Rybp_predicted	XM_232220		-0.12	-1.328	0.054906
A_42_P526293	LOC689397	XM_001073780		-0.12	-1.328	0.366667
A_44_P265287	Ttc6_predicted	XM_216713	299067	-0.12	-1.328	0.242859
A_44_P374741	Dnmt3a	NM_001003958	444984	-0.12	-1.328	0.179973
A_44_P400986	LOC302495	XM_001070285		-0.12	-1.328	0.025346
A_44_P776057	ENSRNOT00000031927	ENSRNOT00000031927		-0.12	-1.328	0.300334
A_43_P12827	Nme3	NM_053507	85269	-0.12	-1.328	0.036059
A_42_P756077	Phyhip	NM_001017376	290356	-0.12	-1.328	0.158048
A_44_P470717	A_44_P470717	A_44_P470717		-0.12	-1.328	0.023217
A_43_P17067	AW918080	AW918080	680076	-0.12	-1.328	0.147155
A_44_P466417	Irf2_predicted	XM_214350		-0.12	-1.329	0.039316
A_44_P320799	Gucy1a3	NM_017090	497757	-0.12	-1.329	0.116787
A_44_P245019	AW143927	AW143927	246331	-0.12	-1.329	0.132824
A_44_P501698	Galnt12_predicted	XM_232988	313233	-0.12	-1.329	0.015266
A_44_P378959	AA859348	AA859348		-0.12	-1.329	0.063442
A_44_P456134	AI072418	AI072418	84430	-0.12	-1.329	0.032665
A_44_P265366	Espl1_predicted	XM_235691	315330	-0.12	-1.329	0.144198
A_44_P209949	BQ211506	BQ211506	295235	-0.12	-1.329	0.036781
A_44_P850880	AA955605	AA955605		-0.12	-1.329	0.044822
A_44_P360845	Ghr	NM_017094	25235	-0.12	-1.329	0.043012
A_44_P117684	BX883050	BX883050		-0.12	-1.329	0.238175
A_44_P257518	Irs2	XM_573948	29376	-0.12	-1.329	0.014949
A_44_P461651	Bbs1_predicted	XM_219691		-0.12	-1.330	0.042576
A_42_P570756	Cox8a	NM_134345	171335	-0.12	-1.330	0.030819
A_42_P840999	Dlgap3	NM_173138	286923	-0.12	-1.330	0.0464
A_44_P325657	LOC306766	NM_001014007	306766	-0.12	-1.330	0.018677
A_44_P201558	Adam33_predicted	XM_230607		-0.12	-1.330	0.061659
A_44_P447288	AI412685	AI412685	288480	-0.12	-1.330	0.084091
A_44_P915624	Lig4_predicted	XM_001067682		-0.12	-1.330	0.052429
A_44_P854437	TC542832	TC542832		-0.12	-1.330	0.10889
A_44_P535899	ENSRNOT00000048767	ENSRNOT00000048767		-0.12	-1.330	0.218433
A_44_P111881	LOC498331	XM_001059696		-0.12	-1.330	0.023407
A_44_P107773	RGD1560815_predicted	XM_225924		-0.12	-1.330	0.111012
A_44_P1056682	LOC498368	NM_001017500	498368	-0.12	-1.330	0.233091
A_44_P228903	Ccbp2	NM_078621	140473	-0.12	-1.330	0.083119
A_44_P330143	Rhcg	NM_183053	293048	-0.12	-1.330	0.144926
A_44_P272309	A_44_P272309	A_44_P272309		-0.12	-1.330	0.0212
A_44_P418305	BQ207009	BQ207009	288516	-0.12	-1.331	0.011401
A_44_P701889	TC532159	TC532159		-0.12	-1.331	0.556628
A_42_P646241	Znf386	NM_019620	25165	-0.12	-1.331	0.031591
A_44_P440804	XM_224416	XM_224416		-0.12	-1.331	0.053345
A_44_P108166	BF407470	BF407470		-0.12	-1.331	0.104099
A_44_P549065	CB606318	CB606318	312371	-0.12	-1.331	0.031565
A_44_P412570	Il1f5_predicted	XM_231101		-0.12	-1.331	0.102935
A_44_P262382	Jdp1	NM_021865	60462	-0.12	-1.331	0.060654
A_42_P830214	RGD1305492_predicted	XM_215485		-0.12	-1.331	0.076765
A_44_P392004	TC542387	TC542387		-0.12	-1.331	0.213271
A_44_P273124	BQ192002	BQ192002	288907	-0.12	-1.331	0.058587
A_43_P16274	Chrna7	NM_012832	25302	-0.12	-1.331	0.335924
A_42_P511274	RT1-CE5	NM_001008843	24737	-0.12	-1.332	0.090303
A_44_P868714	Gata6	XM_001072268		-0.12	-1.332	0.071627
A_44_P316700	RGD1559982_predicted	XM_219714		-0.12	-1.332	0.098656
A_43_P11937	Chrm5	NM_017362	53949	-0.12	-1.332	0.215186
A_44_P495832	Pgm3_predicted	XM_343442		-0.12	-1.332	0.154705
A_44_P987257	AI555307	AI555307		-0.12	-1.332	0.129595
A_44_P450019	Trp53bp2_predicted	XM_223012	305025	-0.12	-1.332	0.171763
A_44_P368008	Lrp10	NM_001037777	305880	-0.12	-1.332	0.031089
A_43_P17372	Diras1_predicted	XM_345790		-0.12	-1.332	0.1523
A_44_P1007831	Zfp710_predicted	XM_214980	293044	-0.12	-1.332	0.070949
A_44_P178048	Tiparp_predicted	XM_227217		-0.12	-1.332	0.119936

A_44_P960935	TC556981	TC556981		-0.12	-1.332	0.020083
A_44_P193130	Sftpa1	NM_017329	24773	-0.12	-1.332	0.087421
A_42_P833332	Ins1	NM_019129	24505	-0.12	-1.332	0.151175
A_44_P760628	LOC503134	XR_009378	503134	-0.12	-1.332	0.107715
A_44_P1059789	Akap9	XM_001063778		-0.12	-1.332	0.091377
A_42_P728032	LOC683470	XM_001066058		-0.12	-1.333	0.252149
A_44_P319315	RGD1311564_predicted	XM_346895		-0.12	-1.333	0.125204
A_44_P637889	TC520832	TC520832		-0.12	-1.333	0.197062
A_44_P653596	TC523478	TC523478		-0.12	-1.333	0.025753
A_44_P278843	XM_217505	XM_217505		-0.12	-1.333	0.019505
A_43_P16362	X62329	X62329		-0.12	-1.333	0.494203
A_44_P179210	BP496210	BP496210	500547	-0.12	-1.333	0.069414
A_43_P21504	Kcnt2	NM_198762	304827	-0.13	-1.334	0.393617
A_44_P195356	Ece1	NM_053596	94204	-0.13	-1.334	0.009351
A_44_P840535	TC533968	TC533968		-0.13	-1.334	0.191556
A_44_P1000100	Trim45_predicted	XM_215666		-0.13	-1.334	0.059291
A_44_P258855	Qscn6	NM_053431	84491	-0.13	-1.334	0.034625
A_44_P554577	LOC361929	XM_342228	361929	-0.13	-1.334	0.032808
A_44_P414170	Gpi	NM_207592	292804	-0.13	-1.334	0.014811
A_44_P992458	TC531572	TC531572		-0.13	-1.334	0.157772
A_42_P492976	LOC691278	XM_001077518	691278	-0.13	-1.334	0.32131
A_44_P390963	Hps6	NM_181432	309446	-0.13	-1.334	0.035941
A_44_P575750	ENSRNOT00000000195	ENSRNOT00000000195		-0.13	-1.335	0.206952
A_44_P336033	BF410589	BF410589		-0.13	-1.335	0.295576
A_43_P19005	RGD1566426_predicted	XM_574285	498995	-0.13	-1.335	0.017046
A_44_P579102	TC568274	TC568274		-0.13	-1.335	0.070156
A_44_P126590	LOC363188	XM_001061775		-0.13	-1.335	0.053889
A_44_P111709	LOC286983	NM_173317	286983	-0.13	-1.335	0.236146
A_42_P623666	LOC301295	XM_217363	301295	-0.13	-1.335	0.056803
A_44_P859096	BQ205656	BQ205656	79223	-0.13	-1.335	0.323729
A_44_P245741	RGD1305013_predicted	XM_218573		-0.13	-1.335	0.139237
A_42_P554928	AW144225	AW144225		-0.13	-1.335	0.186065
A_44_P316126	Muc4	U06749		-0.13	-1.335	0.262599
A_44_P514992	BM392226	BM392226		-0.13	-1.335	0.176332
A_44_P353836	RGD1306962_predicted	XM_220112	309570	-0.13	-1.335	0.052157
A_44_P803822	AW918535	AW918535	29699	-0.13	-1.335	0.044266
A_42_P471335	Zfp523_predicted	XM_342102	361809	-0.13	-1.336	0.032976
A_44_P499166	Mtmr7_predicted	XM_240417		-0.13	-1.336	0.24952
A_44_P428228	AW914778	AW914778		-0.13	-1.336	0.090689
A_44_P391537	LOC681711	XM_001058069		-0.13	-1.336	0.067531
A_44_P400400	Hdac1_predicted	NM_001025409	297893	-0.13	-1.336	0.183078
A_44_P647531	AW918578	AW918578		-0.13	-1.336	0.030003
A_44_P317866	AI547958	AI547958	365466	-0.13	-1.336	0.121787
A_43_P13787	CB547683	CB547683		-0.13	-1.336	0.059279
A_44_P167524	AA997883	AA997883	303185	-0.13	-1.336	0.032561
A_44_P283111	AW530584	AW530584	303566	-0.13	-1.337	0.058126
A_44_P352009	Tmem54	NM_001034151	362605	-0.13	-1.337	0.203587
A_44_P330197	Stk23	NM_184045	293854	-0.13	-1.337	0.15252
A_44_P591963	DV728639	DV728639	306819	-0.13	-1.337	0.043948
A_44_P136020	BQ199246	BQ199246	296637	-0.13	-1.337	0.156511
A_44_P370714	Sh2d5_predicted	XM_001070433		-0.13	-1.338	0.018223
A_44_P146712	Mapk13	NM_019231	29513	-0.13	-1.338	0.123475
A_44_P310501	AA819143	AA819143	64185	-0.13	-1.338	0.228475
A_43_P21430	Smarca1_predicted	XM_229124	317575	-0.13	-1.338	0.058679
A_43_P19262	LOC687014	XM_001076731		-0.13	-1.338	0.203939
A_44_P685392	Gp9	NM_001031825	502858	-0.13	-1.338	0.122239
A_44_P696755	BM392202	BM392202	360842	-0.13	-1.339	0.086343
A_44_P1071190	BI296730	BI296730		-0.13	-1.339	0.1345
A_44_P382161	Myst1	NM_001017378	310194	-0.13	-1.339	0.022252
A_44_P243037	Olr1657_predicted	NM_001000536	364726	-0.13	-1.339	0.0546
A_44_P410622	AI013657	AI013657	311984	-0.13	-1.339	0.123634
A_44_P229948	BM391243	BM391243	287638	-0.13	-1.339	0.072579
A_44_P416871	Snip	NM_019378	56029	-0.13	-1.339	0.025415

A_43_P11924	Calca	NM_017338	24241	-0.13	-1.339	0.169949
A_43_P16831	XM_232055	XM_232055		-0.13	-1.339	0.031277
A_44_P653240	LOC682955	XM_001063844		-0.13	-1.339	0.020769
A_44_P980178	TC532579	TC532579		-0.13	-1.339	0.100432
A_44_P420614	AA900426	AA900426	81920	-0.13	-1.340	0.04302
A_44_P433621	RGD1309120	NM_001025016	360994	-0.13	-1.340	0.183641
A_44_P337816	LOC682152	XM_001060142		-0.13	-1.340	0.108491
A_44_P412322	XM_221928	XM_221928		-0.13	-1.340	0.049962
A_44_P535311	RGD1306001_predicted	XM_343305	362975	-0.13	-1.340	0.023685
A_44_P713311	Ncor1	XM_577103	54299	-0.13	-1.340	0.050836
A_44_P458992	Rapgef5	AY390379	362799	-0.13	-1.340	0.041544
A_43_P13042	Epn1	NM_057136	117277	-0.13	-1.340	0.021276
A_44_P790941	A_44_P790941	A_44_P790941		-0.13	-1.340	0.069821
A_44_P160294	Usp42_predicted	XM_001072044		-0.13	-1.340	0.020032
A_43_P13831	AA956484	AA956484		-0.13	-1.340	0.040663
A_44_P550670	Tmed4_predicted	XM_223611		-0.13	-1.340	0.034439
A_44_P123209	BI289004	BI289004	297387	-0.13	-1.340	0.279623
A_42_P629599	Ccdc5	NM_138864	192228	-0.13	-1.340	0.110419
A_44_P138289	Osgepl1	NM_001024787	314548	-0.13	-1.340	0.01099
A_44_P118590	AW914340	AW914340		-0.13	-1.341	0.085601
A_44_P536843	XM_228696	XM_228696		-0.13	-1.341	0.075826
A_42_P554722	Pigp_predicted	XM_213650		-0.13	-1.341	0.02184
A_44_P1047403	Zbtb7b_predicted	XM_215633		-0.13	-1.341	0.078452
A_44_P343768	BI282137	BI282137		-0.13	-1.341	0.030535
A_44_P342710	LOC691914	XM_001080079	691914	-0.13	-1.341	0.079164
A_44_P960652	TC522058	TC522058		-0.13	-1.341	0.032405
A_44_P822572	Dcun1d4_predicted	XM_001068939		-0.13	-1.341	0.139602
A_44_P170512	LOC365778	NM_001014251	365778	-0.13	-1.341	0.147708
A_44_P365351	Olr1346	NM_001000520	316633	-0.13	-1.341	0.346989
A_44_P853313	CB608963	CB608963		-0.13	-1.341	0.062808
A_44_P723923	A_44_P723923	A_44_P723923		-0.13	-1.341	0.310139
A_44_P492509	XM_234911	XM_234911		-0.13	-1.341	0.060524
A_44_P299380	Olr1416_predicted	NM_001000009	287314	-0.13	-1.341	0.199553
A_43_P15529	Ptgds	NM_013015	25526	-0.13	-1.341	0.411024
A_43_P10392	RGD1563580_predicted	XM_001070331		-0.13	-1.341	0.055629
A_44_P426483	RGD1307391_predicted	XM_001077322		-0.13	-1.342	0.348706
A_44_P340061	ENSRNOT00000050117	ENSRNOT00000050117		-0.13	-1.342	0.312134
A_44_P510886	RGD1305084_predicted	XM_218619	292884	-0.13	-1.342	0.051561
A_44_P389963	Slc39a5_predicted	XM_343140		-0.13	-1.342	0.060092
A_42_P767467	BF556870	BF556870		-0.13	-1.342	0.09188
A_44_P1035872	LOC361473	NM_001025723	361473	-0.13	-1.342	0.043618
A_44_P276242	Olr718_predicted	NM_001000361	295910	-0.13	-1.342	0.211976
A_44_P173726	RGD1561879_predicted	XM_001063903		-0.13	-1.343	0.202879
A_44_P478436	RGD1562299_predicted	XM_234348		-0.13	-1.343	0.020446
A_43_P16057	LOC286985	NM_173319	286985	-0.13	-1.343	0.060419
A_44_P511854	CB605807	CB605807		-0.13	-1.343	0.111035
A_44_P430982	Gng4	XM_001053747		-0.13	-1.343	0.089933
A_44_P440439	ENSRNOT00000025476	ENSRNOT00000025476		-0.13	-1.343	0.07032
A_44_P606150	LOC685945	XM_001065892		-0.13	-1.343	0.099347
A_44_P702222	TC533023	TC533023		-0.13	-1.343	0.02808
A_44_P757289	AW916375	AW916375		-0.13	-1.343	0.031406
A_44_P104054	Crhbp	NM_139183	29625	-0.13	-1.343	0.030582
A_44_P321960	AI599945	AI599945		-0.13	-1.344	0.060735
A_44_P532879	Gna11	NM_031033	81662	-0.13	-1.344	0.049987
A_44_P883997	TC518079	TC518079		-0.13	-1.344	0.099449
A_44_P1044371	Nudt19	NM_001004258	308518	-0.13	-1.344	0.104952
A_44_P781533	TC534057	TC534057		-0.13	-1.344	0.259716
A_44_P454227	Npb	NM_153293	259222	-0.13	-1.345	0.016288
A_44_P503768	ENSRNOT00000047149	ENSRNOT00000047149		-0.13	-1.345	0.058494
A_42_P800048	Ube4a	NM_207610	315608	-0.13	-1.345	0.028247
A_44_P354448	Hs3st3b1_predicted	XM_220557	303218	-0.13	-1.345	0.023978
A_43_P11405	Tmem63b_predicted	XM_001064906		-0.13	-1.345	0.015419
A_44_P475663	A_44_P475663	A_44_P475663		-0.13	-1.345	0.054834

A_44_P682817	RGD1561873_predicted	XM_577100	501699	-0.13	-1.345	0.163644
A_44_P121728	Il17rc_predicted	XM_216240	297520	-0.13	-1.345	0.028284
A_44_P997444	Tube1_predicted	XM_342151		-0.13	-1.345	0.039471
A_44_P1011953	RGD735140	NM_198779	362219	-0.13	-1.345	0.21558
A_44_P544705	AI102751	AI102751	289758	-0.13	-1.345	0.062481
A_44_P237455	AABR03068900	AABR03068900		-0.13	-1.345	0.224421
A_44_P1003567	TC537662	TC537662		-0.13	-1.346	0.03557
A_44_P156846	Lamc3_predicted	XM_231139		-0.13	-1.346	0.261213
A_44_P300751	Vegfa	NM_031836	83785	-0.13	-1.346	0.038715
A_42_P843424	Lipe	NM_012859	25330	-0.13	-1.346	0.066469
A_44_P323856	A_44_P323856	A_44_P323856		-0.13	-1.346	0.205616
A_44_P128840	MGC95155	NM_001005546	300676	-0.13	-1.346	0.207974
A_44_P104870	Crygd	NM_033095	24278	-0.13	-1.346	0.017466
A_44_P438758	RGD1565462_predicted	XM_234496		-0.13	-1.346	0.041449
A_44_P694719	AW921297	AW921297		-0.13	-1.346	0.237266
A_44_P201738	A_44_P201738	A_44_P201738		-0.13	-1.346	0.252951
A_44_P490127	XM_216761	XM_216761		-0.13	-1.346	0.234647
A_44_P426689	LOC295528	XM_001080231		-0.13	-1.346	0.062443
A_44_P178354	RGD1565168_predicted	XM_345725	366733	-0.13	-1.346	0.100825
A_43_P10223	Tmem9b_predicted	XM_001073254		-0.13	-1.346	0.032793
A_44_P520700	RGD1561742_predicted	XM_341377	361092	-0.13	-1.346	0.024935
A_42_P788080	Praf2_predicted	XM_001058767		-0.13	-1.347	0.099381
A_44_P383960	Kcna5	NM_012972	25470	-0.13	-1.347	0.106927
A_44_P669605	DV718686	DV718686		-0.13	-1.347	0.07813
A_44_P1057137	Phf1	NM_212538	294287	-0.13	-1.347	0.187044
A_44_P180084	Gpx5	XM_574039		-0.13	-1.347	0.206625
A_44_P338970	Pld1	NM_030992	25096	-0.13	-1.347	0.225704
A_43_P16887	Eraf_predicted	XM_215059		-0.13	-1.347	0.118638
A_44_P1033565	Nat9_predicted	XM_221103	303669	-0.13	-1.347	0.024326
A_44_P308827	Olr74_predicted	NM_001000134	293218	-0.13	-1.347	0.358489
A_42_P496649	RGD1306839_predicted	XM_215236		-0.13	-1.347	0.083798
A_44_P284169	A_44_P284169	A_44_P284169		-0.13	-1.347	0.135101
A_44_P754734	A_44_P754734	A_44_P754734		-0.13	-1.347	0.121791
A_44_P450856	Atp8b1_predicted	XM_001064261		-0.13	-1.347	0.084242
A_44_P850802	AA945994	AA945994	81712	-0.13	-1.348	0.057778
A_43_P12020	Kcnn3	NM_019315	54263	-0.13	-1.348	0.202282
A_44_P466200	Eif2ak3	NM_031599	29702	-0.13	-1.348	0.055119
A_44_P887540	LOC684758	XM_001071851		-0.13	-1.348	0.060984
A_44_P129303	CB606291	CB606291		-0.13	-1.348	0.124078
A_44_P429304	A_44_P429304	A_44_P429304		-0.13	-1.349	0.188905
A_44_P798023	RGD1565175_predicted	XM_576511	501096	-0.13	-1.349	0.089945
A_44_P708143	A_44_P708143	A_44_P708143		-0.13	-1.349	0.058697
A_42_P784063	Krt2-8	NM_199370	25626	-0.13	-1.349	0.224754
A_44_P317502	BG376626	BG376626	308758	-0.13	-1.349	0.144145
A_44_P600732	CK597461	CK597461		-0.13	-1.349	0.146945
A_44_P297044	Enth	NM_001002022	360515	-0.13	-1.349	0.215486
A_44_P745994	TC539069	TC539069		-0.13	-1.349	0.210298
A_44_P470992	Sox21_predicted	XM_224521	306168	-0.13	-1.349	0.061963
A_44_P1057331	Lyk4	NM_183056	360230	-0.13	-1.350	0.165412
A_44_P510924	LOC691015	XM_001069534		-0.13	-1.350	0.009996
A_44_P264017	LOC365511	XM_345065	365511	-0.13	-1.350	0.133695
A_44_P320213	AI059610	AI059610	360878	-0.13	-1.350	0.017397
A_44_P122118	BI296909	BI296909	292905	-0.13	-1.350	0.131792
A_43_P12821	Spa17	NM_053482	85244	-0.13	-1.350	0.041758
A_44_P200584	Pcaf	NM_001024252	301164	-0.13	-1.350	0.310139
A_44_P791139	A_44_P791139	A_44_P791139		-0.13	-1.350	0.024267
A_44_P555481	XM_345199	XM_345199		-0.13	-1.350	0.054843
A_44_P167407	AA875633	AA875633	287710	-0.13	-1.350	0.033683
A_44_P252210	Txnrd2	NM_022584	50551	-0.13	-1.350	0.012702
A_44_P463691	Gpr3	NM_153727	266769	-0.13	-1.350	0.077681
A_44_P264749	Rbl2	NM_031094	81758	-0.13	-1.350	0.033275
A_42_P476269	RGD1307879_predicted	XM_215387	294400	-0.13	-1.350	0.021531
A_44_P380566	BI296191	BI296191	362925	-0.13	-1.350	0.24106

A_44_P183359	Tmem24	NM_001011996	300666	-0.13	-1.350	0.280642
A_44_P1005530	L3mbtl3_predicted	XM_220113		-0.13	-1.350	0.029683
A_44_P955436	AI144904	AI144904		-0.13	-1.351	0.010007
A_43_P10036	TC537173	TC537173		-0.13	-1.351	0.00982
A_43_P16759	XM_214197	XM_214197		-0.13	-1.351	0.02109
A_44_P198990	XM_218979	XM_218979		-0.13	-1.351	0.075597
A_44_P451198	AA996430	AA996430	361378	-0.13	-1.351	0.137056
A_44_P203821	XM_345338	XM_345338		-0.13	-1.351	0.067183
A_44_P105608	XM_236241	XM_236241		-0.13	-1.351	0.119204
A_44_P494154	Dnah8	XM_228058	294854	-0.13	-1.351	0.327841
A_43_P19054	Dsc2	NM_001033688	291760	-0.13	-1.351	0.038462
A_44_P441227	XM_346882	XM_346882		-0.13	-1.351	0.059319
A_43_P22715	Man2a1	XM_001068826	25478	-0.13	-1.352	0.045397
A_43_P15659	XM_342062	XM_342062		-0.13	-1.352	0.086527
A_44_P299684	Snx26_predicted	XM_001079120		-0.13	-1.352	0.143272
A_44_P208601	Myh7b_predicted	XM_230774		-0.13	-1.352	0.049297
A_44_P576465	A_44_P576465	A_44_P576465		-0.13	-1.352	0.1927
A_44_P308416	Mt1a	DV714812	24567	-0.13	-1.352	0.558163
A_44_P671992	RGD1566201_predicted	XM_575387	500032	-0.13	-1.352	0.092396
A_44_P717216	TC566041	TC566041		-0.13	-1.352	0.161968
A_44_P536774	Kcng2	XM_225718		-0.13	-1.352	0.021659
A_44_P868611	XM_580054	XM_580054		-0.13	-1.352	0.109419
A_44_P368249	Spag8	XM_342826	362508	-0.13	-1.353	0.029786
A_44_P901182	TC528585	TC528585		-0.13	-1.353	0.028414
A_44_P440797	TCRVA8	XM_224098		-0.13	-1.353	0.276751
A_44_P266833	Syt3	NM_019122	25731	-0.13	-1.353	0.020414
A_44_P276378	U78137	U78137		-0.13	-1.353	0.144091
A_44_P493420	BG380309	BG380309	366474	-0.13	-1.353	0.047232
A_43_P10333	Efemp1	NM_001012039	305604	-0.13	-1.353	0.156638
A_44_P198804	Nrxn2	NM_053846	116595	-0.13	-1.353	0.117534
A_44_P247456	AW533027	AW533027		-0.13	-1.353	0.093813
A_44_P219055	BM986267	BM986267	25050	-0.13	-1.354	0.068277
A_44_P269499	XM_222163	XM_222163		-0.13	-1.354	0.072882
A_44_P297250	Fh1	NM_017005	24368	-0.13	-1.354	0.07839
A_44_P899896	DV729194	DV729194		-0.13	-1.354	0.356694
A_43_P10581	Mafk	NM_145673	246760	-0.13	-1.354	0.031721
A_44_P488690	Pltp_predicted	XM_215939	296371	-0.13	-1.354	0.031492
A_44_P316962	AI146082	AI146082		-0.13	-1.354	0.072521
A_44_P143349	Arfip1	NM_021763	60382	-0.13	-1.354	0.078402
A_44_P1031514	RGD1307778	NM_001014054	312135	-0.13	-1.354	0.151891
A_42_P725303	Cog7	NM_001033889	293456	-0.13	-1.354	0.024289
A_44_P506310	MGC94736	NM_001007685	306589	-0.13	-1.354	0.314941
A_44_P225286	Mgat4a	NM_001012225	367252	-0.13	-1.354	0.047883
A_44_P513723	A_44_P513723	A_44_P513723		-0.13	-1.355	0.0716
A_44_P200617	Ube2o_predicted	XM_221132	303689	-0.13	-1.355	0.291705
A_44_P966081	BF403327	BF403327	680590	-0.13	-1.355	0.374624
A_44_P140266	Slc22a15_predicted	XM_227523		-0.13	-1.355	0.173278
A_44_P261738	BX883043	BX883043		-0.13	-1.355	0.026559
A_44_P487732	LOC501417	XM_576828		-0.13	-1.355	0.2354
A_44_P1052976	RGD1563990_predicted	XM_217014		-0.13	-1.355	0.01083
A_44_P711001	AW533360	AW533360		-0.13	-1.355	0.051955
A_44_P158699	Olr1423_predicted	NM_001000006	287309	-0.13	-1.355	0.149759
A_44_P187481	XM_225870	XM_225870		-0.13	-1.355	0.023199
A_44_P1022868	AB033713	AB033713		-0.13	-1.355	0.128679
A_43_P19289	MGC94604	NM_001006960	289745	-0.13	-1.355	0.025314
A_43_P21717	Cbfa2t3_predicted	XM_341709		-0.13	-1.355	0.006396
A_42_P778313	Pde6b_predicted	XM_214126		-0.13	-1.355	0.042838
A_44_P449806	Bmp7	XM_342591	85272	-0.13	-1.355	0.057685
A_44_P852355	A_44_P852355	A_44_P852355		-0.13	-1.355	0.054017
A_44_P489200	Ssfa2_predicted	XM_001067501		-0.13	-1.356	0.100888
A_42_P664913	RGD1565715_predicted	XM_341434		-0.13	-1.356	0.150851
A_44_P1004376	Ins2	NM_019130	24506	-0.13	-1.356	0.139781
A_44_P397589	BE118414	BE118414	308875	-0.13	-1.356	0.05319

A_44_P268134	AA891917	AA891917	691149	-0.13	-1.356	0.024954
A_44_P231510	Tert	AF247818		-0.13	-1.356	0.030275
A_44_P972866	Plekha6_predicted	XM_341118	360842	-0.13	-1.356	0.020536
A_42_P719607	Cxcl7	NM_153721	246358	-0.13	-1.356	0.718946
A_44_P114927	AI599104	AI599104	312974	-0.13	-1.356	0.059022
A_44_P382859	BE120389	BE120389		-0.13	-1.357	0.20587
A_44_P294637	Nlgn3	NM_134336	171297	-0.13	-1.357	0.058775
A_44_P961443	TC558851	TC558851		-0.13	-1.357	0.018965
A_44_P628465	TC561197	TC561197		-0.13	-1.357	0.022295
A_44_P1039634	Odf3_predicted	XM_344975		-0.13	-1.357	0.048193
A_44_P483238	Hoxc5_predicted	XM_235702		-0.13	-1.357	0.035587
A_44_P175003	Ttr	NM_012681	24856	-0.13	-1.357	0.059368
A_44_P524630	P518	NM_198200	379044	-0.13	-1.357	0.027222
A_42_P802550	Pdlim1	NM_017365	54133	-0.13	-1.357	0.008659
A_44_P220193	Zfp364_predicted	XM_342300		-0.13	-1.357	0.015738
A_43_P22805	Unc5d_predicted	XM_240446		-0.13	-1.357	0.100351
A_44_P248222	Slc30a5_predicted	XM_226722		-0.13	-1.357	0.031158
A_44_P418639	CA507081	CA507081	294007	-0.13	-1.357	0.02106
A_44_P374837	Ppm1b	BC061986	24667	-0.13	-1.357	0.060937
A_44_P236433	Slitrk6_predicted	XM_224480		-0.13	-1.357	0.305309
A_44_P233168	BF389478	BF389478	288667	-0.13	-1.358	0.058223
A_44_P187999	AI229596	AI229596		-0.13	-1.358	0.226409
A_44_P361594	AA956228	AA956228		-0.13	-1.358	0.062181
A_44_P352825	AA892319	AA892319		-0.13	-1.358	0.139551
A_44_P482953	RGD1562529_predicted	XM_242139	311372	-0.13	-1.358	0.094926
A_43_P12453	Syt13	NM_030839	80977	-0.13	-1.358	0.024915
A_44_P870763	Dnali1	NM_001031647	298524	-0.13	-1.358	0.188068
A_44_P206985	Gnrh1	NM_012767	25194	-0.13	-1.358	0.049942
A_44_P339881	RGD1306410	NM_001014126	360768	-0.13	-1.358	0.028342
A_44_P158059	AI171503	AI171503	80897	-0.13	-1.358	0.145784
A_44_P100324	Fgf11	NM_130816	170632	-0.13	-1.358	0.168527
A_44_P303933	BG153272	BG153272	29236	-0.13	-1.358	0.211705
A_44_P112488	Galntl1	XM_343087	362760	-0.13	-1.358	0.145469
A_44_P210780	Trim2	XM_342268		-0.13	-1.358	0.169228
A_44_P454281	Ptger4	NM_032076	84023	-0.13	-1.359	0.123572
A_44_P563537	TC565684	TC565684		-0.13	-1.359	0.636068
A_44_P219046	AW142493	AW142493		-0.13	-1.359	0.041775
A_44_P804088	AA926260	AA926260	498416	-0.13	-1.359	0.123126
A_44_P763153	TC545616	TC545616		-0.13	-1.359	0.017537
A_44_P522009	BF282907	BF282907	313648	-0.13	-1.359	0.159801
A_44_P637281	TC523092	TC523092		-0.13	-1.359	0.099523
A_42_P455531	Chrm3	NM_012527	24260	-0.13	-1.359	0.063259
A_44_P502480	AI228238	AI228238	292098	-0.13	-1.359	0.11088
A_44_P351141	Ap3m1	NM_133593	171126	-0.13	-1.359	0.038286
A_43_P17678	Sbds	NM_001008289	288615	-0.13	-1.360	0.017308
A_44_P236744	LOC314393	XM_234490	314393	-0.13	-1.360	0.021147
A_43_P22634	ENSRNOT00000044918	ENSRNOT00000044918		-0.13	-1.360	0.022906
A_44_P775818	RGD1562911_predicted	XM_575895	500534	-0.13	-1.360	0.04378
A_44_P1055471	Ap3s1_predicted	XM_217560		-0.13	-1.360	0.157482
A_44_P960487	DV729077	DV729077		-0.13	-1.360	0.188538
A_43_P15475	Tpc1808	NM_022625	64560	-0.13	-1.360	0.619378
A_44_P225122	XM_346216	XM_346216		-0.13	-1.360	0.122071
A_44_P532165	Gfi1b_predicted	XM_231109		-0.13	-1.360	0.21027
A_44_P1056589	Fxyd7	NM_022008	63848	-0.13	-1.360	0.044457
A_44_P178080	XM_227648	XM_227648		-0.13	-1.361	0.046817
A_43_P15590	Fmod	NM_080698	64507	-0.13	-1.361	0.097111
A_44_P354266	Capza2	NM_001009180	493810	-0.13	-1.361	0.023445
A_44_P529226	Gabre	NM_023091	65191	-0.13	-1.361	0.266313
A_44_P762198	TC558103	TC558103		-0.13	-1.361	0.026974
A_44_P945862	LOC366012	NM_001014257	366012	-0.13	-1.361	0.014784
A_44_P430807	Olr828_predicted	NM_001000991	405351	-0.13	-1.361	0.276612
A_43_P17890	RGD1305157_predicted	XM_236724	316090	-0.13	-1.361	0.051932
A_44_P173244	Tcta	NM_001014005	306587	-0.13	-1.361	0.041886

A_44_P151513	Olr829_predicted	NM_001000901	405211	-0.13	-1.361	0.198591
A_44_P548241	Zfp54_predicted	XM_218037		-0.13	-1.361	0.108215
A_43_P14782	Tnk2	NM_001008336	303882	-0.13	-1.361	0.021696
A_44_P625773	LOC500726	XM_576105	500726	-0.13	-1.361	0.042348
A_42_P842513	Pctk1	NM_031077	81741	-0.13	-1.361	0.039137
A_44_P613902	TC567778	TC567778		-0.13	-1.361	0.006454
A_44_P329139	Arhgef3_predicted	XM_224588		-0.13	-1.361	0.10562
A_44_P716610	TC563006	TC563006		-0.13	-1.362	0.106999
A_43_P17990	Creb3l3	NM_001012115	314638	-0.13	-1.362	0.047626
A_44_P651072	CV104092	CV104092		-0.13	-1.362	0.132711
A_44_P913323	RGD1563285_predicted	XM_574571	499273	-0.13	-1.362	0.086664
A_44_P516745	Acaa1	NM_012489	24157	-0.13	-1.362	0.009648
A_44_P528965	AI236332	AI236332	302642	-0.13	-1.362	0.106967
A_44_P491878	Cdc34_predicted	NM_001013103	299602	-0.13	-1.362	0.082461
A_43_P13064	Klf9	NM_057211	117560	-0.13	-1.362	0.079923
A_44_P320053	AW525201	AW525201	498709	-0.13	-1.362	0.089367
A_44_P188547	AI028953	AI028953	681031	-0.13	-1.362	0.02237
A_44_P975286	CO403766	CO403766		-0.13	-1.362	0.030161
A_44_P240646	Cacna1e	NM_019294	54234	-0.13	-1.362	0.027069
A_44_P203173	Rtn4rl2	NM_181380	311169	-0.13	-1.363	0.040903
A_44_P560070	LOC364268	XM_001059018		-0.13	-1.363	0.044219
A_44_P407827	A_44_P407827	A_44_P407827		-0.13	-1.363	0.044229
A_43_P22811	Cd6	NM_175577	25752	-0.13	-1.363	0.083634
A_43_P11923	Pde3a	NM_017337	50678	-0.13	-1.363	0.036165
A_44_P499075	A_44_P499075	A_44_P499075		-0.13	-1.363	0.249429
A_44_P210763	RGD1308922_predicted	XM_224702		-0.13	-1.363	0.070052
A_44_P440756	TC548806	TC548806		-0.13	-1.364	0.13424
A_44_P547954	RT1-CE6	NR_002155		-0.13	-1.364	0.038909
A_44_P201708	RGD1562337_predicted	XM_233245	313418	-0.13	-1.364	0.031669
A_44_P966280	BF553083	BF553083		-0.13	-1.364	0.122398
A_44_P1057211	Fzr1_predicted	XM_243390		-0.13	-1.364	0.222326
A_43_P18944	Stard8_predicted	XM_231459		-0.13	-1.364	0.258313
A_44_P476948	AI072381	AI072381	294231	-0.13	-1.364	0.053371
A_44_P119107	LOC681211	XM_001060766		-0.13	-1.364	0.019673
A_44_P506717	Pcgf2_predicted	XM_213447		-0.13	-1.364	0.036774
A_44_P265209	RGD1560696_predicted	XM_345518		-0.13	-1.364	0.203258
A_44_P120458	AI045746	AI045746	290642	-0.13	-1.364	0.032129
A_44_P420788	Lypla1	NM_013006	25514	-0.13	-1.364	0.023685
A_44_P659155	BG378607	BG378607	24820	-0.14	-1.365	0.136378
A_44_P277079	XM_343887	XM_343887		-0.14	-1.365	0.173807
A_44_P421715	A_44_P421715	A_44_P421715		-0.14	-1.365	0.021385
A_44_P763063	RGD1563437_predicted	XM_214519	291356	-0.14	-1.365	0.036214
A_44_P159419	Tmprss9_predicted	XM_234914		-0.14	-1.365	0.041388
A_42_P806859	AI229721	AI229721		-0.14	-1.365	0.193589
A_44_P344421	Dock9	XM_224538		-0.14	-1.365	0.055068
A_42_P573296	Hdc	NM_017016	24443	-0.14	-1.365	0.505641
A_44_P618127	AW531732	AW531732		-0.14	-1.365	0.313844
A_42_P677623	Klf6	NM_031642	58954	-0.14	-1.365	0.031971
A_44_P637087	RGD1564591_predicted	XM_576542	501125	-0.14	-1.365	0.202879
A_44_P1023264	Slc37a4	NM_031589	29573	-0.14	-1.365	0.013076
A_44_P658658	BF390608	BF390608		-0.14	-1.366	0.078283
A_44_P285988	Chrdl2_predicted	XM_218964		-0.14	-1.366	0.206425
A_44_P313022	AA998476	AA998476	315215	-0.14	-1.366	0.08187
A_44_P983999	A_44_P983999	A_44_P983999		-0.14	-1.366	0.01857
A_44_P302068	Sox13_predicted	XM_222636		-0.14	-1.366	0.072604
A_44_P264381	Tdrd7	NM_138871	85425	-0.14	-1.366	0.021775
A_44_P538820	RGD1564153_predicted	XM_341942		-0.14	-1.366	0.066881
A_44_P190999	BF281299	BF281299	24609	-0.14	-1.366	0.139701
A_44_P123785	RGD1563498_predicted	XM_344957		-0.14	-1.366	0.010149
A_44_P1007729	Tpmt	NM_031329	83497	-0.14	-1.366	0.016414
A_44_P297788	XM_233855	XM_233855		-0.14	-1.366	0.146596
A_44_P396137	RGD1560642_predicted	XM_225006	306594	-0.14	-1.366	0.038715
A_44_P348999	Asb15	XM_216108	360206	-0.14	-1.366	0.062384

A_43_P12519	S100a10	NM_031114	81778	-0.14	-1.366	0.047512
A_44_P264276	LOC501052	XM_576468		-0.14	-1.367	0.015585
A_44_P475275	Olr1678_predicted	NM_001000893	405194	-0.14	-1.367	0.273382
A_44_P409840	Ccl17	NM_057151	117518	-0.14	-1.367	0.105429
A_44_P853879	TC523565	TC523565		-0.14	-1.367	0.018375
A_43_P19263	Myt1_predicted	XM_342605		-0.14	-1.367	0.318688
A_42_P802520	Pex14	NM_172063	64460	-0.14	-1.367	0.07044
A_44_P469746	AI178828	AI178828	116636	-0.14	-1.367	0.054286
A_44_P275967	BM384243	BM384243	288504	-0.14	-1.367	0.055904
A_42_P603500	LOC498358	XM_001057251		-0.14	-1.367	0.034843
A_44_P510675	Il12rb1	XM_344493	171333	-0.14	-1.367	0.022285
A_44_P203828	XM_345338	XM_345338		-0.14	-1.368	0.018139
A_44_P293067	RGD1561231_predicted	XM_225619		-0.14	-1.368	0.092553
A_42_P788030	Nckap1	XM_001067975		-0.14	-1.368	0.019641
A_44_P525024	Stk3	NM_031735	65189	-0.14	-1.368	0.016745
A_44_P252634	Yipf3	NM_001007801	301245	-0.14	-1.368	0.112509
A_44_P408520	Oprl1	NM_031569	29256	-0.14	-1.368	0.09735
A_44_P305135	Epb4.1l1	NM_021681	59317	-0.14	-1.368	0.055082
A_42_P492135	Pcbp4_predicted	XM_343468	363133	-0.14	-1.368	0.265474
A_44_P402705	Rab3b	NM_031091	81755	-0.14	-1.368	0.138208
A_44_P683643	TC518806	TC518806		-0.14	-1.368	0.041378
A_44_P468103	Alb	NM_134326	24186	-0.14	-1.368	0.140257
A_43_P19585	Gps2_predicted	XM_220615		-0.14	-1.369	0.133186
A_44_P417303	RGD1560784_predicted	XM_343770		-0.14	-1.369	0.152763
A_44_P555782	Jrkl_predicted	XM_235818		-0.14	-1.369	0.05571
A_44_P666425	RGD1560744_predicted	XM_225701	307251	-0.14	-1.369	0.050951
A_44_P848376	LOC687266	XM_001078256		-0.14	-1.369	0.022445
A_44_P123543	Lin7a	NM_053514	85327	-0.14	-1.369	0.285245
A_44_P987003	AI228153	AI228153	309622	-0.14	-1.369	0.144309
A_44_P289968	AA964693	AA964693	83688	-0.14	-1.369	0.030255
A_44_P196948	Rpgr	XM_346270		-0.14	-1.369	0.027975
A_44_P991239	Sh3glb1	NM_001011929	292156	-0.14	-1.369	0.064236
A_44_P152017	XM_227163	XM_227163		-0.14	-1.369	0.016989
A_44_P105554	Actrt2	NM_001013937	298671	-0.14	-1.369	0.057337
A_44_P116300	Ppargc1b	NM_176075	291567	-0.14	-1.369	0.091434
A_44_P636077	RGD1308377_predicted	XM_225093		-0.14	-1.370	0.077207
A_44_P382501	Foxr1_predicted	XM_243815	315601	-0.14	-1.370	0.030839
A_44_P449704	Olr690_predicted	NM_001000569	366111	-0.14	-1.370	0.12877
A_44_P461383	RGD1306812_predicted	XM_215580		-0.14	-1.370	0.137707
A_43_P11749	Hck	NM_013185	25734	-0.14	-1.370	0.068452
A_44_P989546	LOC303823	XM_001058114		-0.14	-1.370	0.096943
A_44_P981783	TC551251	TC551251		-0.14	-1.370	0.031759
A_44_P974229	LOC365186	XR_007164		-0.14	-1.370	0.189481
A_44_P402604	Olr710_predicted	NM_001000571	366113	-0.14	-1.370	0.257605
A_44_P961999	TC544379	TC544379		-0.14	-1.370	0.054875
A_43_P11167	BI294198	BI294198	298541	-0.14	-1.370	0.095175
A_44_P156582	Cpt1b	NM_013200	25756	-0.14	-1.370	0.077406
A_44_P339558	Myo5b	NM_017083	25132	-0.14	-1.370	0.057422
A_44_P982946	AABR03068037	AABR03068037		-0.14	-1.371	0.102124
A_44_P116332	Olr1338_predicted	NM_001000789	405073	-0.14	-1.371	0.297997
A_44_P160846	Rnasel	NM_182673	359726	-0.14	-1.371	0.148327
A_44_P901865	TC532372	TC532372		-0.14	-1.371	0.053561
A_44_P400958	Rab9b_predicted	XM_346351		-0.14	-1.371	0.079287
A_44_P536698	A_44_P536698	A_44_P536698		-0.14	-1.371	0.048125
A_44_P247745	Shank2	NM_201350	171093	-0.14	-1.371	0.060158
A_44_P742272	Hist1h4b	NM_022686	64627	-0.14	-1.371	0.11199
A_44_P381270	RGD1562136_predicted	XM_576623	501195	-0.14	-1.371	0.112725
A_44_P398785	RGD1306782_predicted	XM_235505		-0.14	-1.372	0.017314
A_44_P468360	Rgs3	NM_019340	54293	-0.14	-1.372	0.038995
A_44_P1021106	Lmo4	NM_001009708	362051	-0.14	-1.372	0.098453
A_44_P147618	Cohh1_predicted	XM_243588	315036	-0.14	-1.372	0.032863
A_44_P930249	A_44_P930249	A_44_P930249		-0.14	-1.372	0.15946
A_43_P13074	Rab3d	NM_080580	140665	-0.14	-1.372	0.031894

A_44_P999329	LOC500954	XM_576366		-0.14	-1.372	0.023721
A_42_P751152	Ndufb8_predicted	XM_215269		-0.14	-1.372	0.031902
A_44_P224734	XM_344817	XM_344817		-0.14	-1.372	0.115935
A_44_P516063	Lrrc35	NM_001014089	315591	-0.14	-1.372	0.046869
A_44_P210931	XM_342146	XM_342146		-0.14	-1.372	0.136378
A_44_P486071	Cul4b_predicted	XM_001058651		-0.14	-1.372	0.263452
A_44_P899737	TC522538	TC522538		-0.14	-1.372	0.043357
A_42_P629370	Klf15	NM_053536	85497	-0.14	-1.372	0.349587
A_44_P419777	A_44_P419777	A_44_P419777		-0.14	-1.372	0.06054
A_44_P368058	MGC93997	NM_001007697	310664	-0.14	-1.372	0.067368
A_44_P561040	TC520429	TC520429		-0.14	-1.372	0.021731
A_44_P186516	BQ211716	BQ211716	294292	-0.14	-1.373	0.146537
A_44_P229719	LOC298606	NM_001013936	298606	-0.14	-1.373	0.035936
A_44_P120792	Hook3	XM_224952	306548	-0.14	-1.373	0.056484
A_44_P396842	Ucn2	NM_133385	170896	-0.14	-1.373	0.260321
A_44_P452300	Pla2g5	NM_017174	29354	-0.14	-1.373	0.152933
A_44_P210703	Prkg1_mapped	XM_001080430		-0.14	-1.373	0.221917
A_44_P1058264	BG670702	BG670702		-0.14	-1.373	0.080906
A_43_P11084	AI231433	AI231433		-0.14	-1.373	0.204272
A_44_P492482	Plekhh1_predicted	XM_234332		-0.14	-1.373	0.112819
A_44_P824280	LOC682483	XM_001061691		-0.14	-1.374	0.021017
A_43_P18509	Etfa	NM_001009668	300726	-0.14	-1.374	0.113914
A_44_P105543	A_44_P105543	A_44_P105543		-0.14	-1.374	0.100019
A_44_P406445	Pigb_predicted	XM_001053705		-0.14	-1.374	0.045468
A_43_P16054	AF010436	AF010436		-0.14	-1.374	0.146772
A_44_P325599	G7c	NM_212499	309611	-0.14	-1.374	0.072261
A_42_P544436	Slc35a1_predicted	XM_232884		-0.14	-1.374	0.023146
A_42_P685050	Jam2	NM_001034004	619374	-0.14	-1.374	0.248531
A_43_P19145	RGD1309676	NM_001014140	361118	-0.14	-1.374	0.084441
A_44_P405120	Ctsg_predicted	XM_214205		-0.14	-1.374	0.21558
A_44_P147509	Cbara1	NM_199412	365567	-0.14	-1.374	0.07426
A_44_P263007	Rasl12_predicted	XM_236351		-0.14	-1.374	0.256581
A_44_P1023093	LOC362855	NM_207614	362855	-0.14	-1.374	0.031951
A_42_P827057	LOC298643	NM_001017450	298643	-0.14	-1.374	0.019793
A_44_P439942	AI599392	AI599392		-0.14	-1.374	0.079625
A_44_P498016	Dsm-1	NM_001037215	501103	-0.14	-1.374	0.046061
A_44_P526803	AW919096	AW919096		-0.14	-1.374	0.036652
A_44_P116213	LOC690987	XM_001076910		-0.14	-1.375	0.015334
A_43_P16784	Med25_predicted	XM_214950	292889	-0.14	-1.375	0.017813
A_44_P237988	BF387347	BF387347		-0.14	-1.375	0.088247
A_43_P12757	Admr	NM_053302	29307	-0.14	-1.375	0.088581
A_44_P242826	AI045489	AI045489	309456	-0.14	-1.375	0.079923
A_44_P476191	RGD1311429_predicted	XM_001081571		-0.14	-1.375	0.088055
A_44_P1024907	Icam5_predicted	XM_233737	313785	-0.14	-1.375	0.177416
A_44_P272758	AI172007	AI172007	498587	-0.14	-1.376	0.063502
A_44_P716085	TC523882	TC523882		-0.14	-1.376	0.213271
A_44_P276477	Garnl1	XM_578542	56785	-0.14	-1.376	0.021017
A_44_P532307	RGD1310773_predicted	XM_001057690		-0.14	-1.376	0.143528
A_44_P409022	AI178706	AI178706		-0.14	-1.376	0.079398
A_44_P377603	XM_344767	XM_344767		-0.14	-1.376	0.068697
A_44_P285575	Cox6c1	NM_173303	286962	-0.14	-1.377	0.034512
A_44_P316451	XM_347010	XM_347010		-0.14	-1.377	0.062556
A_44_P110491	BG375824	BG375824	361679	-0.14	-1.377	0.110881
A_42_P765830	Ndufb5_predicted	XM_215544		-0.14	-1.377	0.015069
A_44_P113879	Slco1a5	NM_030838	80900	-0.14	-1.377	0.043642
A_44_P438447	XM_224519	XM_224519		-0.14	-1.377	0.06272
A_44_P406474	Prkcz	NM_022507	25522	-0.14	-1.377	0.03656
A_44_P610294	TC525431	TC525431		-0.14	-1.377	0.033461
A_44_P558104	A_44_P558104	A_44_P558104		-0.14	-1.378	0.046611
A_44_P1059322	RGD1563506_predicted	XM_214982	293057	-0.14	-1.378	0.143544
A_44_P683133	ENSRNOT00000029909	ENSRNOT00000029909		-0.14	-1.378	0.039757
A_44_P473580	Rspo1_predicted	XM_233520		-0.14	-1.378	0.177844
A_44_P261755	BM392398	BM392398	290644	-0.14	-1.378	0.097533

A_44_P705063	AW251360	AW251360	315114	-0.14	-1.378	0.026549
A_43_P14529	Mpdz	NM_019196	29365	-0.14	-1.379	0.048832
A_44_P347417	ENSRNOT00000036270	ENSRNOT00000036270		-0.14	-1.379	0.038233
A_44_P868920	DV728362	DV728362		-0.14	-1.379	0.373263
A_44_P235869	AY136826	AY136826		-0.14	-1.379	0.014677
A_44_P638308	TC538845	TC538845		-0.14	-1.379	0.015368
A_44_P296883	BU759670	BU759670	307390	-0.14	-1.379	0.101962
A_44_P330318	Cdkl2	NM_001012035	305242	-0.14	-1.379	0.186029
A_44_P492094	RGD1561509_predicted	XM_218970	308868	-0.14	-1.379	0.140775
A_44_P791030	RGD1565302_predicted	XM_575656		-0.14	-1.379	0.015156
A_44_P357739	BE113965	BE113965	502632	-0.14	-1.379	0.103819
A_44_P505462	AA923923	AA923923	362361	-0.14	-1.379	0.042851
A_44_P110516	BE097218	BE097218	300447	-0.14	-1.380	0.148706
A_43_P20686	Gats	XM_222092	304410	-0.14	-1.380	0.059402
A_44_P245806	RGD1561986_predicted	XM_219665		-0.14	-1.380	0.039994
A_44_P433385	S58644	S58644		-0.14	-1.380	0.462726
A_42_P729496	Aps	NM_053669	114203	-0.14	-1.380	0.156722
A_44_P435675	Olr1596_predicted	NM_001000501	304991	-0.14	-1.380	0.158202
A_44_P1058916	LOC296318	NM_001013923	296318	-0.14	-1.380	0.075187
A_44_P306368	Lip1	NM_012732	25055	-0.14	-1.381	0.045908
A_42_P475340	Fbxw5	NM_001025730	362081	-0.14	-1.381	0.010123
A_44_P508355	Ache	X70141	83817	-0.14	-1.381	0.106559
A_44_P241398	Podxl2_predicted	XM_216209		-0.14	-1.381	0.184575
A_44_P314978	CB544341	CB544341	117548	-0.14	-1.381	0.038172
A_44_P1037997	BG670699	BG670699		-0.14	-1.381	0.027059
A_44_P426492	RGD1310292_predicted	XM_222240	304537	-0.14	-1.381	0.014712
A_44_P220882	Cpne5_predicted	XM_228044		-0.14	-1.381	0.079025
A_44_P380381	Lasp1	NM_032613	29278	-0.14	-1.382	0.010424
A_44_P963405	Samd14	NM_001024966	287637	-0.14	-1.382	0.035708
A_44_P281269	XM_344360	XM_344360		-0.14	-1.382	0.229086
A_44_P541378	Isoc1	NM_001014242	364879	-0.14	-1.382	0.031479
A_44_P377910	Iqcb1_predicted	XM_221420		-0.14	-1.382	0.064007
A_44_P405443	Manba	NM_001031655	310864	-0.14	-1.383	0.044576
A_43_P20782	CB544318	CB544318	363885	-0.14	-1.383	0.019042
A_43_P21489	Zmynd10	NM_001004284	363139	-0.14	-1.383	0.006892
A_44_P161159	Hcfc1r1	XM_573052	287097	-0.14	-1.383	0.008253
A_42_P822764	TC521012	TC521012		-0.14	-1.383	0.105114
A_44_P962473	RGD1564117_predicted	XM_001070369		-0.14	-1.383	0.199526
A_44_P622920	TC557339	TC557339		-0.14	-1.383	0.050655
A_44_P668185	RGD1310444_predicted	XR_006750	363015	-0.14	-1.383	0.072476
A_44_P476260	BF561025	BF561025		-0.14	-1.383	0.138978
A_44_P139770	Olr446_predicted	NM_001001050	405937	-0.14	-1.384	0.255056
A_44_P692795	A_44_P692795	A_44_P692795		-0.14	-1.384	0.084395
A_44_P296772	LOC686192	XM_001073285		-0.14	-1.384	0.143873
A_44_P156774	LOC681198	XM_001060719		-0.14	-1.384	0.29172
A_43_P20885	MGC72612	NM_001009538	494340	-0.14	-1.384	0.012199
A_44_P137356	Herpud1	NM_053523	85430	-0.14	-1.384	0.014045
A_44_P531758	Cryab	NM_012935	25420	-0.14	-1.384	0.141391
A_44_P324224	BF561003	BF561003	689651	-0.14	-1.384	0.168495
A_44_P117166	XM_234661	XM_234661		-0.14	-1.384	0.190716
A_42_P603716	Sec23a_predicted	XM_347236		-0.14	-1.385	0.035959
A_44_P529067	AA800971	AA800971	500554	-0.14	-1.385	0.027809
A_44_P319064	LOC683275	XM_001065204		-0.14	-1.385	0.104907
A_44_P288470	XM_342380	XM_342380		-0.14	-1.385	0.13972
A_44_P173299	RGD1309969	XM_342471		-0.14	-1.385	0.128649
A_44_P1040055	Azi2	NM_001025705	316051	-0.14	-1.385	0.035941
A_44_P292895	XM_223170	XM_223170		-0.14	-1.386	0.084607
A_44_P280840	Olr1509_predicted	NM_001000528	363642	-0.14	-1.386	0.262288
A_44_P557246	Erbb3	NM_017218	29496	-0.14	-1.386	0.112169
A_42_P794172	Rnf31_predicted	XM_344409		-0.14	-1.386	0.016039
A_44_P1020391	Fkbp2_predicted	XM_215196	293702	-0.14	-1.387	0.017035
A_44_P372569	Luzp1	NM_030830	79428	-0.14	-1.387	0.007431
A_42_P786109	AW144489	AW144489		-0.14	-1.387	0.077566

A_43_P18573	Lrba_predicted	XM_001066755		-0.14	-1.387	0.048442
A_44_P351461	Pcdhb11_predicted	XM_001064927		-0.14	-1.387	0.155594
A_44_P771831	BG665097	BG665097		-0.14	-1.387	0.179007
A_44_P425367	LOC500282	NM_001024332	500282	-0.14	-1.388	0.015632
A_43_P19350	RGD1306704_predicted	XM_215709	295483	-0.14	-1.388	0.184323
A_44_P702258	TC555467	TC555467		-0.14	-1.388	0.115898
A_42_P796938	Hdac11_predicted	XM_238362		-0.14	-1.388	0.114006
A_44_P182587	XM_576852	XM_576852		-0.14	-1.388	0.14639
A_44_P323787	XM_230974	XM_230974		-0.14	-1.388	0.025165
A_43_P12291	Alpi2	NM_022680	64621	-0.14	-1.388	0.033434
A_43_P12311	Srd5a2	NM_022711	64677	-0.14	-1.388	0.055622
A_43_P12799	Chst3	NM_053408	84468	-0.14	-1.388	0.029249
A_44_P340520	TC526514	TC526514		-0.14	-1.388	0.083643
A_44_P312999	BM390441	BM390441	311723	-0.14	-1.388	0.027558
A_44_P126381	RGD1560386_predicted	XM_215422	294521	-0.14	-1.388	0.287762
A_44_P555309	Piwil1_predicted	XM_344105		-0.14	-1.388	0.055861
A_44_P196306	Olr159_predicted	NM_001000170	293305	-0.14	-1.389	0.04953
A_42_P763424	Ua20	AY064511	246251	-0.14	-1.389	0.019419
A_44_P230263	AI408613	AI408613		-0.14	-1.389	0.010237
A_42_P579898	Gprk2l	NM_022928	59077	-0.14	-1.389	0.022878
A_44_P226759	XM_574978	XM_574978		-0.14	-1.389	0.006643
A_44_P547280	AI639231	AI639231	364510	-0.14	-1.389	0.063848
A_44_P395978	Ndnl2	XM_219708	309259	-0.14	-1.389	0.053561
A_44_P1003210	AF452728	AF452728	24674	-0.14	-1.389	0.018388
A_44_P142242	Cdh1	NM_031334	83502	-0.14	-1.390	0.024523
A_44_P341644	AI111346	AI111346	305066	-0.14	-1.390	0.075375
A_43_P17192	TC521454	TC521454		-0.14	-1.390	0.369073
A_44_P238237	Comt	NM_012531	24267	-0.14	-1.390	0.009538
A_44_P510436	Myr8	NM_138893	192253	-0.14	-1.390	0.137252
A_44_P463064	M27315	M27315		-0.14	-1.390	0.108535
A_43_P12073	Uts2r	NM_020537	57305	-0.14	-1.390	0.018357
A_42_P794149	AA848230	AA848230		-0.14	-1.390	0.031755
A_44_P359095	A_44_P359095	A_44_P359095		-0.14	-1.390	0.027023
A_44_P234970	Hrmt1l1	NM_001025144	499420	-0.14	-1.390	0.04512
A_43_P18479	XM_215818	XM_215818		-0.14	-1.391	0.124161
A_43_P16093	Olr837	NM_001000897	405207	-0.14	-1.391	0.10472
A_44_P264518	Hdmcp	NM_001001509	299316	-0.14	-1.391	0.058465
A_43_P15449	Slc10a2	NM_017222	29500	-0.14	-1.391	0.179396
A_44_P908874	A_44_P908874	A_44_P908874		-0.14	-1.391	0.114186
A_44_P900835	TC544267	TC544267		-0.14	-1.391	0.128476
A_44_P674035	AI104544	AI104544		-0.14	-1.391	0.096914
A_43_P14147	RGD1565289_predicted	XM_343793	363474	-0.14	-1.391	0.032747
A_44_P160113	AW918729	AW918729	362251	-0.14	-1.391	0.030852
A_44_P438292	Nkd2_predicted	XM_217743		-0.14	-1.391	0.052711
A_44_P394993	AI045171	AI045171		-0.14	-1.391	0.130098
A_44_P132293	BQ192168	BQ192168	298705	-0.14	-1.391	0.080751
A_44_P349817	LOC301455	XM_237215	301455	-0.14	-1.391	0.15699
A_44_P402375	Por	NM_031576	29441	-0.14	-1.391	0.008721
A_44_P293374	Kremen2_predicted	XM_220206		-0.14	-1.391	0.045085
A_44_P103411	BM385476	BM385476	378947	-0.14	-1.392	0.057012
A_42_P528779	Kif12	XM_575840	313254	-0.14	-1.392	0.125078
A_44_P187328	Gm963_predicted	XM_219524		-0.14	-1.392	0.182624
A_44_P809098	TC563058	TC563058		-0.14	-1.392	0.025509
A_44_P121347	Serpina6_mapped	NM_001009663	299270	-0.14	-1.392	0.10844
A_44_P431884	AA818187	AA818187		-0.14	-1.392	0.083584
A_44_P557117	Lgals2	CA507274	171134	-0.14	-1.392	0.166948
A_42_P777177	BF542992	BF542992	24674	-0.14	-1.392	0.018647
A_44_P320764	Olr1499_predicted	NM_001000031	287502	-0.14	-1.392	0.147212
A_44_P412349	A_44_P412349	A_44_P412349		-0.14	-1.392	0.131412
A_44_P239205	Rwdd2_predicted	XM_343443		-0.14	-1.392	0.200471
A_42_P680186	RGD1563286_predicted	XM_346287		-0.14	-1.392	0.021473
A_44_P273958	Evc	XM_223512	289712	-0.14	-1.392	0.091558
A_44_P627113	TC549538	TC549538		-0.14	-1.393	0.027443

A_44_P269609	Chuk_predicted	XM_219857		-0.14	-1.393	0.125605
A_44_P976552	TC559216	TC559216		-0.14	-1.393	0.077678
A_44_P401551	BF558506	BF558506		-0.14	-1.393	0.026314
A_44_P361161	RGD1562767_predicted	XM_342433	362133	-0.14	-1.393	0.113308
A_44_P496529	L20998	L20998		-0.14	-1.393	0.01407
A_44_P219774	Nostrin	NM_001024260	311111	-0.14	-1.393	0.043966
A_44_P870017	CO393619	CO393619		-0.14	-1.393	0.039615
A_44_P858422	BI292219	BI292219	56822	-0.14	-1.393	0.06498
A_44_P438255	Gpr27	NM_023099	65275	-0.14	-1.393	0.060495
A_44_P538970	Ptpdc1_predicted	XM_214440		-0.14	-1.393	0.056484
A_44_P379647	Uqcrc2	NM_001006970	293448	-0.14	-1.393	0.026982
A_44_P198064	AA849739	AA849739		-0.14	-1.393	0.257547
A_44_P791057	A_44_P791057	A_44_P791057		-0.14	-1.394	0.046117
A_44_P523326	XM_343940	XM_343940		-0.14	-1.394	0.018157
A_44_P105763	AI113065	AI113065		-0.14	-1.394	0.094982
A_44_P443064	Pex13_predicted	XM_223684		-0.14	-1.394	0.086235
A_44_P354315	Nkx2-9_predicted	XM_234179		-0.14	-1.394	0.107163
A_44_P494483	XM_215528	XM_215528		-0.14	-1.394	0.016157
A_44_P870394	TC545069	TC545069		-0.14	-1.394	0.059574
A_44_P104034	BQ190595	BQ190595	287429	-0.14	-1.394	0.10844
A_44_P876215	CF109454	CF109454		-0.14	-1.394	0.01281
A_44_P428965	RGD1562154_predicted	XM_223906		-0.14	-1.394	0.463113
A_43_P19760	Sdsl_predicted	XM_341089		-0.14	-1.394	0.068503
A_44_P361225	LOC292017	XM_226468	292017	-0.14	-1.394	0.031409
A_43_P15508	Rbl2	NM_031094	81758	-0.14	-1.394	0.047381
A_44_P540486	BF289433	BF289433		-0.14	-1.394	0.128457
A_44_P491883	NP516922	NP516922		-0.14	-1.394	0.100339
A_44_P319042	Kbtbd2_predicted	XM_231798		-0.14	-1.394	0.07942
A_43_P12872	Lenep	NM_053614	113917	-0.14	-1.394	0.021534
A_44_P653165	TC538863	TC538863		-0.14	-1.394	0.031002
A_44_P400393	Irs2	XM_573948	29376	-0.14	-1.395	0.027004
A_44_P526425	LOC365802	NM_001014253	365802	-0.14	-1.395	0.011391
A_44_P276724	RGD1563703_predicted	XM_341488	361206	-0.14	-1.395	0.385963
A_44_P504261	RGD1309930	NM_001014102	316426	-0.14	-1.395	0.152087
A_44_P333071	Lrp1	XM_243524	299858	-0.14	-1.395	0.073963
A_42_P818819	Chkb	NM_017177	29367	-0.14	-1.395	0.018396
A_43_P23475	X94514	X94514	304286	-0.14	-1.395	0.007453
A_44_P341633	AA945601	AA945601	1E+08	-0.14	-1.395	0.043365
A_44_P236047	Olr744_predicted	NM_001000577	366122	-0.14	-1.395	0.038982
A_44_P715059	TC539683	TC539683		-0.14	-1.395	0.022205
A_44_P291028	Arhgef19_predicted	XM_342965		-0.14	-1.395	0.097608
A_44_P509151	BE099218	BE099218		-0.14	-1.395	0.014071
A_44_P314541	AI227731	AI227731		-0.14	-1.395	0.323118
A_44_P839137	TC525484	TC525484		-0.14	-1.395	0.056824
A_44_P250476	Zdhhc13	NM_001039037	365252	-0.14	-1.395	0.048173
A_42_P739860	Dusp5	NM_133578	171109	-0.14	-1.395	0.029836
A_42_P458060	RGD1311499_predicted	XM_221199	303744	-0.14	-1.395	0.054874
A_44_P915826	TC542691	TC542691		-0.14	-1.396	0.196045
A_44_P259957	S100a1	NM_001007636	295214	-0.14	-1.396	0.046591
A_44_P480771	Hivep3_predicted	XM_233464		-0.14	-1.396	0.337468
A_44_P530389	Ecel1	NM_021776	60417	-0.14	-1.396	0.068042
A_44_P116221	Cdig2	AB086193	266732	-0.15	-1.396	0.016582
A_44_P668867	TC522661	TC522661		-0.15	-1.396	0.037006
A_44_P285498	Camk2n2	NM_021678	59314	-0.15	-1.396	0.0252
A_44_P686616	TC551601	TC551601		-0.15	-1.396	0.113874
A_44_P465554	Vdac2	NM_031354	83531	-0.15	-1.396	0.078565
A_44_P543012	Ptp4a2	NM_053475	85237	-0.15	-1.397	0.033372
A_44_P199541	Gjc1	XM_343965		-0.15	-1.397	0.167302
A_44_P222898	LOC688730	XM_001068081		-0.15	-1.397	0.05755
A_43_P15303	Arts1	NM_030836	80897	-0.15	-1.397	0.007862
A_43_P21122	Sytl2_predicted	XM_341882		-0.15	-1.397	0.065097
A_44_P262517	ENSRNOT00000035391	ENSRNOT00000035391		-0.15	-1.397	0.011238
A_44_P344703	RGD1562738_predicted	XM_574955		-0.15	-1.397	0.140814

A_44_P532754	Pbx3_predicted	XM_231158		-0.15	-1.397	0.023352
A_44_P239140	Morn1	NM_001005544	298676	-0.15	-1.397	0.073527
A_44_P946803	TC561358	TC561358		-0.15	-1.397	0.016809
A_44_P178014	RGD1560814_predicted	XM_224193		-0.15	-1.397	0.147174
A_43_P19791	XM_343063	XM_343063		-0.15	-1.397	0.01596
A_43_P16949	Sgca_predicted	XM_220884		-0.15	-1.397	0.037636
A_44_P363937	AA859053	AA859053		-0.15	-1.398	0.042786
A_44_P975327	B3gnt1_predicted	XM_001061345		-0.15	-1.398	0.087584
A_44_P812625	A_44_P812625	A_44_P812625		-0.15	-1.398	0.294302
A_44_P967122	CK843405	CK843405		-0.15	-1.398	0.057161
A_43_P17126	LOC683751	XM_001067331		-0.15	-1.398	0.06593
A_44_P276562	RGD1564063_predicted	XM_573447		-0.15	-1.398	0.150494
A_43_P20702	RGD1559463_predicted	XM_236297	300748	-0.15	-1.398	0.048143
A_43_P15281	Trp63	NM_019221	246334	-0.15	-1.398	0.176737
A_43_P16221	LOC311710	XM_230955	114588	-0.15	-1.398	0.06603
A_44_P947670	TC532601	TC532601		-0.15	-1.398	0.309113
A_44_P525222	Rln3r1	NM_001008310	294807	-0.15	-1.398	0.171494
A_44_P272631	AA818792	AA818792		-0.15	-1.398	0.053734
A_44_P540511	Pkib	NM_012627	24678	-0.15	-1.399	0.132377
A_44_P964783	TC566328	TC566328		-0.15	-1.399	0.236175
A_44_P809189	RGD1565064_predicted	XM_574308	499015	-0.15	-1.399	0.073909
A_44_P403172	XM_230501	XM_230501		-0.15	-1.399	0.047604
A_44_P519978	Olr544_predicted	NM_001001052	405940	-0.15	-1.399	0.136955
A_44_P719889	AA819660	AA819660		-0.15	-1.399	0.007963
A_42_P540711	Rasd2	NM_133568	171099	-0.15	-1.399	0.121541
A_44_P228748	BE112262	BE112262	619346	-0.15	-1.399	0.084364
A_44_P217339	Olr347_predicted	NM_001000758	405031	-0.15	-1.399	0.045588
A_44_P475932	Otud5	NM_001037496	363452	-0.15	-1.399	0.077007
A_42_P746109	Asmtl_predicted	XM_213725		-0.15	-1.399	0.047053
A_43_P21159	XM_219683	XM_219683		-0.15	-1.399	0.048056
A_43_P20982	Ptprm	XM_343640	29616	-0.15	-1.400	0.189066
A_44_P1015069	Btbd9	NM_001013073	294318	-0.15	-1.400	0.012686
A_44_P152847	RGD1559880_predicted	XM_342973		-0.15	-1.400	0.007329
A_43_P11032	BE113300	BE113300		-0.15	-1.400	0.062747
A_44_P476076	RGD1564752_predicted	XM_347101	363050	-0.15	-1.400	0.15619
A_44_P496911	A_44_P496911	A_44_P496911		-0.15	-1.400	0.046055
A_43_P16827	RGD1306332_predicted	XM_216815		-0.15	-1.400	0.016866
A_44_P719679	ENSRNOT00000046892	ENSRNOT00000046892		-0.15	-1.400	0.010944
A_44_P831218	CK596627	CK596627		-0.15	-1.401	0.174319
A_43_P10548	CB544265	CB544265		-0.15	-1.401	0.037132
A_44_P375173	XM_224033	XM_224033		-0.15	-1.401	0.106367
A_44_P141930	Slc27a5	NM_024143	79111	-0.15	-1.401	0.205416
A_44_P215799	XM_345949	XM_345949		-0.15	-1.401	0.038818
A_44_P667280	RGD1561684_predicted	XM_577125		-0.15	-1.401	0.241092
A_44_P373295	BF557098	BF557098	313507	-0.15	-1.401	0.296469
A_43_P11576	Adcy6	NM_012821	25289	-0.15	-1.401	0.018259
A_44_P417562	RGD1559575_predicted	XM_220362		-0.15	-1.401	0.055218
A_43_P11126	XM_214266	XM_214266		-0.15	-1.401	0.033262
A_44_P306554	Ihh	XM_001060415		-0.15	-1.402	0.056731
A_43_P13447	RGD1309823_predicted	XM_001067111		-0.15	-1.402	0.012333
A_44_P152237	RGD1308059	NM_001025022	362535	-0.15	-1.402	0.106972
A_44_P410318	LOC367619	XM_001071580		-0.15	-1.403	0.247598
A_44_P482885	Hormad1_predicted	XM_345248		-0.15	-1.403	0.074119
A_43_P20248	Pkp1_predicted	XM_222666		-0.15	-1.403	0.154106
A_44_P145137	A_44_P145137	A_44_P145137		-0.15	-1.403	0.020961
A_44_P507343	BE098803	BE098803		-0.15	-1.403	0.259395
A_44_P192151	A_44_P192151	A_44_P192151		-0.15	-1.403	0.047182
A_42_P604331	XM_579967	XM_579967		-0.15	-1.403	0.039607
A_44_P997608	LOC681867	XM_001056889		-0.15	-1.404	0.007977
A_44_P194637	RGD1564763_predicted	XM_346277	367747	-0.15	-1.404	0.032154
A_44_P124303	XM_237361	XM_237361		-0.15	-1.404	0.034474
A_44_P255499	Ube2q_predicted	XM_215612		-0.15	-1.404	0.011484
A_44_P289378	Cpt1b	NM_013200	25756	-0.15	-1.404	0.097511

A_44_P285820	Irs3	NM_032074	84021	-0.15	-1.404	0.065504
A_44_P128350	Lad1_predicted	XM_233122		-0.15	-1.404	0.024803
A_44_P516166	A_44_P516166	A_44_P516166		-0.15	-1.404	0.044413
A_44_P151558	Olr199_predicted	NM_001001034	405920	-0.15	-1.404	0.200448
A_44_P113775	BI289132	BI289132		-0.15	-1.405	0.083098
A_44_P226859	Fgb	M35602		-0.15	-1.405	0.082055
A_44_P219454	AI145654	AI145654	25725	-0.15	-1.405	0.02227
A_44_P138308	A_44_P138308	A_44_P138308		-0.15	-1.405	0.064954
A_43_P18687	Snx25	XM_224863	306471	-0.15	-1.405	0.016812
A_44_P106447	AI105223	AI105223	29253	-0.15	-1.405	0.109368
A_43_P17329	TC541702	TC541702		-0.15	-1.405	0.0509
A_44_P759344	AI104571	AI104571	295929	-0.15	-1.405	0.025422
A_44_P251104	AI576782	AI576782	315758	-0.15	-1.405	0.023444
A_44_P959369	A_44_P959369	A_44_P959369		-0.15	-1.405	0.036509
A_43_P12047	Sdfr1	NM_019380	56064	-0.15	-1.405	0.020246
A_44_P464065	Ushbp1	NM_001033687	290629	-0.15	-1.405	0.011495
A_44_P410607	AA955786	AA955786		-0.15	-1.405	0.021502
A_44_P163552	RGD1311660_predicted	XM_213726	288518	-0.15	-1.405	0.043849
A_44_P245998	Ldlrad3_predicted	XM_345406	366138	-0.15	-1.406	0.044884
A_43_P12083	Hand1	NM_021592	59112	-0.15	-1.406	0.032732
A_44_P316833	TC528432	TC528432		-0.15	-1.406	0.036356
A_43_P18931	RGD1304982_predicted	XM_213907	289138	-0.15	-1.406	0.044266
A_44_P554415	BI281796	BI281796	291964	-0.15	-1.406	0.43895
A_43_P13131	Fgf15	NM_130753	170582	-0.15	-1.406	0.018021
A_44_P516209	AA923830	AA923830		-0.15	-1.406	0.221992
A_44_P116831	RGD1308772_predicted	XM_214237	290381	-0.15	-1.406	0.088361
A_43_P17468	Surb7_predicted	XM_232531		-0.15	-1.406	0.141347
A_44_P803398	RGD1309744_predicted	XM_230574		-0.15	-1.406	0.022601
A_44_P316036	Olr724_predicted	NM_001000573	366115	-0.15	-1.406	0.118849
A_44_P206061	RGD1311595	XM_001079649		-0.15	-1.406	0.027006
A_44_P515645	RGD1305719_predicted	XM_223538	289727	-0.15	-1.406	0.01326
A_44_P157159	RGD1311914	NM_001030032	301067	-0.15	-1.406	0.053794
A_44_P1039417	Usp42_predicted	XM_237865		-0.15	-1.406	0.029032
A_44_P215112	Cbl27	XM_576311	192178	-0.15	-1.407	0.022174
A_44_P272475	RGD1560102_predicted	XM_344039		-0.15	-1.407	0.120654
A_44_P165524	Mrgprb1	NM_001002280	404640	-0.15	-1.407	0.302775
A_44_P464339	RGD1561484_predicted	XM_233593		-0.15	-1.407	0.031002
A_44_P289173	Grtp1	BC097985	361180	-0.15	-1.407	0.035823
A_44_P162232	Rcbtb1	XM_341330		-0.15	-1.407	0.044125
A_44_P643325	AW914948	AW914948	363009	-0.15	-1.407	0.038013
A_44_P836881	ENSRNOT00000047846	ENSRNOT00000047846		-0.15	-1.407	0.02759
A_44_P592652	TC524522	TC524522		-0.15	-1.407	0.022423
A_44_P414691	Wdr27_predicted	XM_001055617		-0.15	-1.407	0.064263
A_43_P20506	CB545830	CB545830	688800	-0.15	-1.407	0.067002
A_44_P322987	Crybb1	NM_012936	25421	-0.15	-1.408	0.076346
A_44_P165715	Ankrd9	NM_001012112	314457	-0.15	-1.408	0.007176
A_44_P161027	Zhx2	XM_235318	314988	-0.15	-1.408	0.025953
A_44_P513747	Eea1_predicted	XM_235064		-0.15	-1.408	0.005085
A_43_P22284	RGD1563878_predicted	XM_573201		-0.15	-1.408	0.096055
A_44_P255319	XM_218144	XM_218144		-0.15	-1.408	0.052574
A_43_P13404	Spag11	NM_145087	246305	-0.15	-1.408	0.046241
A_44_P551179	A_44_P551179	A_44_P551179		-0.15	-1.408	0.091792
A_44_P1042696	RGD1564450_predicted	XM_215355		-0.15	-1.408	0.015958
A_44_P554904	Prss2	NM_012729	25052	-0.15	-1.408	0.163708
A_44_P1004840	Tnfsf9	NM_181384	353218	-0.15	-1.408	0.052458
A_44_P447424	Olr808_predicted	NM_001000850	405140	-0.15	-1.408	0.014839
A_42_P703602	Mep1b	NM_013183	25727	-0.15	-1.408	0.134375
A_44_P447601	RGD1359156	NM_001005531	287747	-0.15	-1.409	0.031847
A_44_P415807	AA955172	AA955172	300149	-0.15	-1.409	0.145401
A_44_P538003	AI170530	AI170530	295231	-0.15	-1.409	0.137743
A_42_P542909	Stau2	NM_134466	171500	-0.15	-1.409	0.034556
A_44_P174207	Snx13_predicted	XM_343053		-0.15	-1.409	0.083643
A_44_P379012	Ctrl	NM_054009	117184	-0.15	-1.409	0.114692

A_44_P1056222	RGD1306148_predicted	XM_232937	313196	-0.15	-1.409	0.012992
A_44_P540570	AW140602	AW140602		-0.15	-1.409	0.012201
A_44_P179184	BE111756	BE111756	315648	-0.15	-1.410	0.018781
A_44_P410247	XM_345125	XM_345125		-0.15	-1.410	0.371643
A_44_P105844	AA818899	AA818899		-0.15	-1.410	0.012291
A_44_P776995	TC539667	TC539667		-0.15	-1.410	0.087507
A_44_P172210	DY471924	DY471924		-0.15	-1.410	0.014239
A_44_P424034	A_44_P424034	A_44_P424034		-0.15	-1.410	0.040824
A_44_P312202	AA866228	AA866228	306810	-0.15	-1.410	0.108194
A_44_P426219	Ngep	NM_001004071	367318	-0.15	-1.410	0.054724
A_44_P257965	RGD1305256_predicted	XM_230550	311443	-0.15	-1.410	0.052038
A_44_P546204	Acbd5	XM_001053289		-0.15	-1.410	0.031476
A_44_P516110	A_44_P516110	A_44_P516110		-0.15	-1.410	0.06603
A_44_P408430	BQ211181	BQ211181	24790	-0.15	-1.410	0.016301
A_44_P607953	TC542265	TC542265		-0.15	-1.410	0.008292
A_44_P396542	XM_343955	XM_343955		-0.15	-1.410	0.216339
A_44_P614997	BG381659	BG381659	308436	-0.15	-1.410	0.024741
A_44_P839411	TC544244	TC544244		-0.15	-1.411	0.018961
A_44_P294996	Mycs	NM_021837	24581	-0.15	-1.411	0.2832
A_44_P335986	Morp1	NM_022613	64548	-0.15	-1.411	0.180481
A_42_P626869	RGD1309585_predicted	XM_340803	360531	-0.15	-1.411	0.018079
A_44_P245443	Olr482_predicted	NM_001000561	366099	-0.15	-1.411	0.115893
A_42_P624945	Begain	NM_024163	79146	-0.15	-1.411	0.085763
A_44_P492954	BE101317	BE101317		-0.15	-1.411	0.008359
A_44_P461505	Dgka	NM_080787	140866	-0.15	-1.411	0.074857
A_44_P347970	AW142620	AW142620	301261	-0.15	-1.411	0.157241
A_44_P189953	Pdxk	NM_031769	83578	-0.15	-1.411	0.143582
A_44_P640931	TC543001	TC543001		-0.15	-1.411	0.010022
A_44_P530300	RGD1565996_predicted	XM_221491		-0.15	-1.411	0.009411
A_44_P352079	Tmprss4_predicted	XM_345932		-0.15	-1.411	0.006635
A_43_P10989	Cgm4	NM_012525	24257	-0.15	-1.411	0.008053
A_43_P12678	Pias3	NM_031784	83614	-0.15	-1.412	0.075246
A_42_P621952	AA899041	AA899041		-0.15	-1.412	0.014407
A_43_P12924	Abcg5	NM_053754	114628	-0.15	-1.412	0.024871
A_44_P180060	Hdac6	XM_228753	84581	-0.15	-1.412	0.339832
A_44_P506514	LOC301193	XM_236873	301193	-0.15	-1.412	0.435904
A_44_P467184	BQ191138	BQ191138		-0.15	-1.412	0.083582
A_44_P153070	AA943842	AA943842		-0.15	-1.412	0.01608
A_44_P400346	Stab2	AY007370	282580	-0.15	-1.412	0.005911
A_44_P258201	RGD1565866_predicted	XM_235047	687814	-0.15	-1.413	0.134503
A_44_P630159	TC551551	TC551551		-0.15	-1.413	0.131036
A_44_P518876	CA507185	CA507185	313254	-0.15	-1.413	0.231961
A_44_P482862	LOC498604	XM_573881	498604	-0.15	-1.413	0.012355
A_44_P265237	LOC305771	XR_006748	305771	-0.15	-1.413	0.06662
A_43_P23356	XM_232861	XM_232861		-0.15	-1.413	0.117864
A_44_P398142	Btg2	NM_017259	29619	-0.15	-1.413	0.01606
A_44_P365516	Tgfb1i4	BC059146	498545	-0.15	-1.413	0.043733
A_44_P314219	Rassf4	NM_001024275	362423	-0.15	-1.414	0.146095
A_44_P557302	Olr1744_predicted	NM_001001426	294198	-0.15	-1.414	0.094845
A_43_P19440	BQ206045	BQ206045	306306	-0.15	-1.414	0.060713
A_43_P14460	Prg4_predicted	XM_213903		-0.15	-1.414	0.012806
A_44_P100975	Litaf	XM_343856		-0.15	-1.414	0.008171
A_44_P301863	Gng12	XM_578287		-0.15	-1.414	0.010904
A_44_P100847	Ttc7	XM_343013	362696	-0.15	-1.414	0.064736
A_44_P229800	Ndrg1	NM_001011991	299923	-0.15	-1.414	0.127361
A_44_P262467	RGD1310945_predicted	XM_214760	292266	-0.15	-1.414	0.335157
A_44_P124099	LOC688677	XM_001067852		-0.15	-1.414	0.025056
A_44_P492234	Diras2_predicted	XM_225214		-0.15	-1.415	0.03546
A_44_P547609	AW141666	AW141666	25446	-0.15	-1.415	0.091652
A_44_P509014	A_44_P509014	A_44_P509014		-0.15	-1.415	0.06108
A_44_P349569	XM_345105	XM_345105		-0.15	-1.415	0.056541
A_44_P944786	TC534634	TC534634		-0.15	-1.415	0.008746
A_42_P465952	AI602520	AI602520	791259	-0.15	-1.415	0.05716

A_44_P794680	TC520152	TC520152		-0.15	-1.415	0.066953
A_44_P382194	Pbxip1	XM_227420		-0.15	-1.415	0.017636
A_44_P628190	TC560395	TC560395		-0.15	-1.415	0.093795
A_44_P991634	TC544790	TC544790		-0.15	-1.415	0.011141
A_43_P11616	Atf3	NM_012912	25389	-0.15	-1.415	0.135325
A_44_P734060	TC561398	TC561398		-0.15	-1.415	0.017228
A_44_P252728	RGD1564240_predicted	XM_001081630		-0.15	-1.415	0.019912
A_44_P276393	LOC498705	XM_573987		-0.15	-1.416	0.07903
A_44_P525059	LOC288741	NM_001010946	288741	-0.15	-1.416	0.043789
A_44_P187195	C1galt1	NM_022950	65044	-0.15	-1.416	0.023592
A_43_P15640	Pdha1	NM_001004072	29554	-0.15	-1.416	0.004076
A_44_P201307	LOC304923	XM_222823	304923	-0.15	-1.416	0.051676
A_44_P524334	BE107674	BE107674		-0.15	-1.416	0.096783
A_44_P242321	Acaa2	NM_130433	170465	-0.15	-1.416	0.093284
A_43_P13875	TC563595	TC563595		-0.15	-1.417	0.054022
A_44_P347368	Glb1_mapped	XM_236675		-0.15	-1.417	0.129273
A_44_P711020	Znrf1_predicted	XM_342692		-0.15	-1.417	0.016178
A_44_P504951	BF286096	BF286096		-0.15	-1.417	0.048064
A_44_P215731	RGD1559880_predicted	XM_342973		-0.15	-1.417	0.036446
A_44_P326366	Mpp5_predicted	XM_234328		-0.15	-1.417	0.046738
A_44_P276919	A_44_P276919	A_44_P276919		-0.15	-1.417	0.006168
A_44_P497215	Slc22a14_predicted	XM_236684		-0.15	-1.417	0.028693
A_44_P251064	Krt1-14	NM_001008751	287701	-0.15	-1.417	0.435581
A_44_P1031726	Dnajc10	XM_215751		-0.15	-1.417	0.042113
A_44_P225493	Usp15	NM_145184	171329	-0.15	-1.417	0.012892
A_44_P156521	LOC497995	NM_001025135	497995	-0.15	-1.418	0.192212
A_44_P290204	Dnm3	NM_138538	171574	-0.15	-1.418	0.142015
A_44_P353685	Stx12	NM_022939	65033	-0.15	-1.418	0.021428
A_44_P503835	RGD1563190_predicted	XM_344485	364513	-0.15	-1.418	0.090931
A_44_P141756	AI104388	AI104388	298687	-0.15	-1.418	0.113051
A_44_P109907	LOC498384	XR_006620	498384	-0.15	-1.418	0.034722
A_43_P10275	AW915934	AW915934		-0.15	-1.418	0.022722
A_44_P669786	RGD1564387_predicted	XM_001075923		-0.15	-1.418	0.069699
A_44_P139042	Znf579_predicted	XM_218200	308339	-0.15	-1.418	0.099824
A_43_P15984	Olr337	NM_001000507	309225	-0.15	-1.418	0.060723
A_44_P609899	Acpl2	NM_001007710	315939	-0.15	-1.418	0.031875
A_44_P591895	TC539703	TC539703		-0.15	-1.418	0.027528
A_44_P793901	TC529691	TC529691		-0.15	-1.418	0.028126
A_44_P469938	BQ209292	BQ209292	361396	-0.15	-1.419	0.109613
A_44_P1037364	LOC291411	BC084699	291411	-0.15	-1.419	0.018562
A_44_P203349	LOC500270	XM_575620		-0.15	-1.419	0.185211
A_44_P950583	CO396335	CO396335		-0.15	-1.419	0.072725
A_44_P103176	A_44_P103176	A_44_P103176		-0.15	-1.419	0.006033
A_43_P11865	Pts	NM_017220	29498	-0.15	-1.419	0.051535
A_44_P324888	CN544457	CN544457	362592	-0.15	-1.419	0.040286
A_44_P144871	Coq4	NM_001031662	366013	-0.15	-1.419	0.006937
A_44_P670043	TC544335	TC544335		-0.15	-1.419	0.028398
A_42_P806395	Col4a4	NM_001008332	301562	-0.15	-1.419	0.022186
A_44_P433773	Traf6_predicted	XM_230377		-0.15	-1.420	0.008902
A_44_P327758	AA924584	AA924584	362736	-0.15	-1.420	0.057199
A_44_P491897	Stx1b2	NM_012700	24923	-0.15	-1.420	0.059179
A_44_P333281	LOC365082	XR_008174	365082	-0.15	-1.420	0.027291
A_44_P337554	RGD1563497_predicted	XM_001080802		-0.15	-1.420	0.129655
A_44_P913890	A_44_P913890	A_44_P913890		-0.15	-1.420	0.023303
A_44_P730099	TC539235	TC539235		-0.15	-1.420	0.173546
A_44_P316908	RGD1559497_predicted	XM_221125	303684	-0.15	-1.420	0.233313
A_43_P14863	Sdhb_predicted	XM_216558	298596	-0.15	-1.420	0.00558
A_44_P851788	BC079001	BC079001	25497	-0.15	-1.420	0.015768
A_44_P303430	AW142654	AW142654		-0.15	-1.420	0.031993
A_44_P400147	Add3	NM_031552	25230	-0.15	-1.420	0.006673
A_44_P362969	Pde6h	NM_053688	114248	-0.15	-1.420	0.055063
A_44_P961330	TC524818	TC524818		-0.15	-1.421	0.018486
A_44_P781559	Ncoa1_predicted	XM_001070473		-0.15	-1.421	0.096783

A_44_P151981	Ddx26	XM_341337		-0.15	-1.421	0.029803
A_44_P153026	Garnl1	XM_578542	56785	-0.15	-1.421	0.004864
A_44_P222447	RGD1560394_predicted	XM_237926		-0.15	-1.421	0.129529
A_44_P125487	BM986254	BM986254	94201	-0.15	-1.421	0.099852
A_44_P956070	RGD1563595_predicted	XM_001077558		-0.15	-1.421	0.0148
A_44_P762089	TC558341	TC558341		-0.15	-1.421	0.023627
A_43_P14752	LOC690274	XM_001073940	690274	-0.15	-1.422	0.016105
A_44_P931582	TC542623	TC542623		-0.15	-1.422	0.039308
A_42_P756334	Myh6	NM_017239	29556	-0.15	-1.422	0.031843
A_44_P529859	Ing1	NM_001038591	306626	-0.15	-1.422	0.093166
A_44_P883945	Stx7	NM_021869	60466	-0.15	-1.422	0.019852
A_42_P781116	Paqr5	NM_001014092	315741	-0.15	-1.422	0.012
A_44_P240243	BG381726	BG381726	500443	-0.15	-1.422	0.14189
A_43_P14131	Rgs2	NM_053453	84583	-0.15	-1.422	0.276251
A_44_P478514	A_44_P478514	A_44_P478514		-0.15	-1.422	0.044576
A_44_P128820	A_44_P128820	A_44_P128820		-0.15	-1.422	0.039068
A_43_P14751	Aqp9	NM_022960	65054	-0.15	-1.422	0.015539
A_43_P10302	LOC679942	XM_001054429		-0.15	-1.422	0.135495
A_44_P671835	TC526343	TC526343		-0.15	-1.423	0.020655
A_44_P513635	A_44_P513635	A_44_P513635		-0.15	-1.423	0.035953
A_44_P587322	AW917250	AW917250		-0.15	-1.423	0.050541
A_44_P112481	XM_347072	XM_347072		-0.15	-1.423	0.037358
A_44_P330188	Acox2	NM_145770	252898	-0.15	-1.423	0.054385
A_44_P352912	AI575434	AI575434	303746	-0.15	-1.423	0.051986
A_42_P837568	L1cam	NM_017345	50687	-0.15	-1.423	0.106512
A_44_P501281	Pvrl1	XM_236210	192183	-0.15	-1.423	0.172647
A_42_P582777	RGD1565431_predicted	XM_574580		-0.15	-1.423	0.011712
A_44_P227510	Tmcc3_predicted	XM_235072		-0.15	-1.423	0.01246
A_43_P21874	Actl7a	NM_001011973	298017	-0.15	-1.423	0.03631
A_44_P950747	AI717736	AI717736	503164	-0.15	-1.424	0.017663
A_44_P437945	Bbc3	NM_173837	317673	-0.15	-1.424	0.014544
A_43_P15057	TC538830	TC538830		-0.15	-1.424	0.031158
A_43_P21136	Mapk8ip2	XM_235565	315220	-0.15	-1.424	0.037936
A_44_P155816	Eef2k	NM_012947	25435	-0.15	-1.424	0.016018
A_44_P192347	Asph_predicted	XM_232675		-0.15	-1.424	0.035026
A_44_P139889	Tert	NM_053423	301965	-0.15	-1.424	0.275292
A_44_P383169	BG381738	BG381738	315093	-0.15	-1.424	0.086323
A_44_P408763	AW523737	AW523737	690229	-0.15	-1.424	0.102538
A_44_P176726	BQ201963	BQ201963		-0.15	-1.424	0.01618
A_43_P22378	RGD1564164_predicted	XM_341562	361277	-0.15	-1.425	0.037452
A_44_P436447	XM_345755	XM_345755		-0.15	-1.425	0.215059
A_44_P636369	RGD1562941_predicted	XM_574390	499099	-0.15	-1.425	0.254797
A_44_P141614	AI236640	AI236640	363469	-0.15	-1.425	0.027832
A_44_P261677	Zfp276	NM_001012058	307924	-0.15	-1.425	0.044867
A_44_P105560	XM_229695	XM_229695		-0.15	-1.425	0.016268
A_44_P373962	AI070757	AI070757	246333	-0.15	-1.425	0.010432
A_44_P359869	BF398047	BF398047	304983	-0.15	-1.425	0.072501
A_44_P894467	AW915768	AW915768		-0.15	-1.425	0.152933
A_44_P532570	RGD1310335_predicted	XM_340982	360711	-0.15	-1.425	0.087142
A_44_P246381	Dgcr6_predicted	XM_221270		-0.15	-1.425	0.07311
A_44_P417942	AI169745	AI169745		-0.15	-1.426	0.082236
A_44_P284062	XM_345745	XM_345745		-0.15	-1.426	0.127914
A_43_P16011	Dnah7	XM_001065965		-0.15	-1.426	0.070115
A_44_P484512	AA945596	AA945596	361205	-0.15	-1.426	0.110122
A_44_P141253	AW918235	AW918235	29699	-0.15	-1.426	0.016061
A_44_P389019	RT1-CE10	NM_001008833	24737	-0.15	-1.426	0.025433
A_44_P190497	LOC317312	NM_001014107	317312	-0.15	-1.426	0.060801
A_43_P12244	Timm8b	NM_022541	64372	-0.15	-1.427	0.00915
A_42_P557293	RGD1308284_predicted	XM_342165		-0.15	-1.427	0.02536
A_43_P11729	Hmgcr	NM_013134	25675	-0.15	-1.427	0.066104
A_44_P252599	Olr464_predicted	NM_001000296	295734	-0.15	-1.427	0.035941
A_44_P269384	Cpn1	NM_053526	365466	-0.15	-1.427	0.014633
A_44_P348654	BM986472	BM986472	59073	-0.15	-1.427	0.067026

A_43_P12758	Msra	NM_053307	29447	-0.15	-1.427	0.030002
A_44_P1025812	LOC682941	XM_001063790		-0.15	-1.427	0.014281
A_44_P688963	TC567675	TC567675		-0.15	-1.428	0.055162
A_43_P17808	RGD1307603_predicted	XM_215189	293656	-0.15	-1.428	0.042211
A_44_P428575	LOC301444	XM_237195		-0.15	-1.428	0.026607
A_43_P11642	Ndufa5	NM_012985	25488	-0.15	-1.428	0.019949
A_44_P440827	Tssk6_predicted	XM_224740		-0.16	-1.429	0.198657
A_44_P173876	Pank1_predicted	XM_215283		-0.16	-1.429	0.030158
A_44_P1010902	Cldn7	NM_031702	65132	-0.16	-1.429	0.040349
A_44_P224864	XM_344333	XM_344333		-0.16	-1.429	0.263011
A_44_P444528	AI171288	AI171288	361834	-0.16	-1.429	0.075716
A_44_P161617	Irak2	NM_001025422	362418	-0.16	-1.429	0.019028
A_43_P10052	Rybp_predicted	XM_232220		-0.16	-1.429	0.01306
A_44_P475944	Enth	XM_001068335		-0.16	-1.430	0.051971
A_44_P1032898	Mgea6_predicted	XM_216709		-0.16	-1.430	0.016552
A_44_P808845	TC526841	TC526841		-0.16	-1.430	0.013368
A_44_P973707	RGD1309388_predicted	XM_214273		-0.16	-1.430	0.008562
A_44_P428914	Atp8a1_predicted	XM_223390	289615	-0.16	-1.430	0.009101
A_44_P368306	Extl1_predicted	XM_233547		-0.16	-1.430	0.044536
A_42_P511615	Ppcs	NM_001039010	298490	-0.16	-1.430	0.022858
A_42_P760101	Pold2	NM_001013050	289758	-0.16	-1.430	0.306408
A_44_P534949	AA818235	AA818235		-0.16	-1.430	0.052324
A_44_P497863	Neud4	XM_341822		-0.16	-1.430	0.033595
A_43_P12797	Kcnk9	NM_053405	84429	-0.16	-1.430	0.068576
A_44_P325324	BQ781530	BQ781530	297890	-0.16	-1.430	0.118055
A_43_P20419	Zfp276	NM_001012058	307924	-0.16	-1.430	0.004717
A_44_P514140	CB547618	CB547618		-0.16	-1.430	0.01596
A_44_P173087	XM_222750	XM_222750		-0.16	-1.430	0.061538
A_44_P146675	Fgf23	NM_130754	170583	-0.16	-1.430	0.044755
A_44_P807557	TC522047	TC522047		-0.16	-1.430	0.035487
A_44_P613130	AI548864	AI548864		-0.16	-1.431	0.059994
A_44_P258075	Zdhhc21	NM_001039009	298184	-0.16	-1.431	0.013082
A_44_P472106	Cacna1s	L04684	116652	-0.16	-1.431	0.034814
A_44_P414102	Cox6c	NM_019360	54322	-0.16	-1.431	0.006329
A_44_P319354	BM383925	BM383925		-0.16	-1.431	0.055658
A_44_P187815	XM_346084	XM_346084		-0.16	-1.431	0.055162
A_44_P362016	Adprtl1	XM_341326	361046	-0.16	-1.431	0.031734
A_44_P194182	ENSRNOT00000008752	ENSRNOT00000008752		-0.16	-1.431	0.092792
A_44_P274230	Hipk4	NM_001024776	308449	-0.16	-1.431	0.012975
A_44_P668752	TC539202	TC539202		-0.16	-1.432	0.007629
A_44_P859515	AW533375	AW533375		-0.16	-1.432	0.101998
A_44_P974365	ENSRNOT00000046703	ENSRNOT00000046703		-0.16	-1.432	0.339919
A_44_P251731	AA943056	AA943056	500116	-0.16	-1.432	0.137981
A_44_P533722	AW253367	AW253367	288710	-0.16	-1.432	0.030242
A_44_P399660	Kdelr3_predicted	XM_235478	315131	-0.16	-1.432	0.049935
A_44_P166261	A_44_P166261	A_44_P166261		-0.16	-1.432	0.046292
A_43_P11432	Acaa1	NM_012489	24157	-0.16	-1.432	0.011423
A_44_P235861	AI044949	AI044949	361285	-0.16	-1.432	0.219565
A_44_P959790	A_44_P959790	A_44_P959790		-0.16	-1.432	0.025467
A_44_P897620	CF110307	CF110307	360822	-0.16	-1.432	0.06836
A_44_P471285	Tnfrsf14	NM_001015034	366518	-0.16	-1.432	0.035178
A_44_P328782	RGD1563990_predicted	XM_001054912		-0.16	-1.433	0.02969
A_44_P505782	MGC72996	NM_198781	362301	-0.16	-1.433	0.021037
A_43_P17531	Muted_predicted	XM_225255		-0.16	-1.433	0.068241
A_44_P220942	CB547135	CB547135	499015	-0.16	-1.433	0.026517
A_44_P171791	AI105439	AI105439	299053	-0.16	-1.433	0.131695
A_44_P138280	Ptpn18	NM_001013111	301333	-0.16	-1.433	0.045826
A_44_P318444	Bcdo2	XM_236220	315644	-0.16	-1.433	0.071025
A_44_P104801	Olr1349_predicted	NM_001000484	301611	-0.16	-1.433	0.13757
A_44_P156930	Clecsf1_predicted	XM_344773		-0.16	-1.434	0.049076
A_44_P240103	BM385573	BM385573	311165	-0.16	-1.434	0.07058
A_44_P205599	Cyp2b3	NM_173294	286953	-0.16	-1.434	0.129564
A_44_P320987	Magi2	NM_053621	113970	-0.16	-1.434	0.051015

A_44_P252616	Olr1457_predicted	NM_001000526	363608	-0.16	-1.434	0.061954
A_43_P17789	RGD1306256_predicted	XM_341812		-0.16	-1.434	0.019559
A_44_P182835	XM_225280	XM_225280		-0.16	-1.434	0.041308
A_44_P858381	A_44_P858381	A_44_P858381		-0.16	-1.434	0.100188
A_44_P489076	AW915692	AW915692		-0.16	-1.434	0.040539
A_44_P614608	A_44_P614608	A_44_P614608		-0.16	-1.435	0.012157
A_43_P13021	Hsd17b3	NM_054007	117182	-0.16	-1.435	0.0902
A_44_P448320	RGD1560925_predicted	XR_008738	501196	-0.16	-1.435	0.130373
A_44_P178770	AW526333	AW526333	300837	-0.16	-1.435	0.030956
A_44_P986636	AW915589	AW915589		-0.16	-1.435	0.005578
A_44_P982636	A_44_P982636	A_44_P982636		-0.16	-1.435	0.115416
A_44_P898803	Sp3	NM_001029905		-0.16	-1.435	0.01361
A_44_P326198	Atp6v0d1	NM_001011927	291969	-0.16	-1.435	0.02943
A_43_P13043	Ebp	NM_057137	117278	-0.16	-1.435	0.019535
A_44_P611151	TC565292	TC565292		-0.16	-1.435	0.034232
A_44_P153206	AA944575	AA944575	24525	-0.16	-1.435	0.005383
A_44_P667489	RGD1565341_predicted	XM_574231		-0.16	-1.435	0.042496
A_44_P774197	BF289616	BF289616		-0.16	-1.435	0.023342
A_44_P200116	Pde10a	NM_022236	63885	-0.16	-1.436	0.071841
A_43_P11125	CB544867	CB544867		-0.16	-1.437	0.180522
A_43_P18909	Kif9_predicted	XM_236648		-0.16	-1.437	0.040154
A_43_P19663	Pax2_predicted	XM_239083		-0.16	-1.437	0.054086
A_44_P573701	AW918532	AW918532		-0.16	-1.437	0.069728
A_44_P1057197	Taf9l	NM_133615	171152	-0.16	-1.437	0.077762
A_44_P170209	BF397366	BF397366	313323	-0.16	-1.437	0.168754
A_44_P321984	BF409209	BF409209	689151	-0.16	-1.437	0.228814
A_44_P180696	RGD1565016_predicted	XM_234928	314647	-0.16	-1.437	0.189597
A_44_P501426	Pkd2l1_predicted	XM_219856		-0.16	-1.437	0.075587
A_44_P379331	LOC257642	NM_147136		-0.16	-1.438	0.016966
A_44_P340434	RGD1306041_predicted	XM_235549	300125	-0.16	-1.438	0.018849
A_44_P328111	S70690	S70690		-0.16	-1.438	0.076366
A_44_P221024	AA800191	AA800191	315345	-0.16	-1.438	0.020745
A_43_P12438	Hmox2	NM_024387	79239	-0.16	-1.438	0.015332
A_44_P238210	Mig12	NM_206950	404280	-0.16	-1.439	0.051476
A_44_P179996	LOC691644	XM_001077511	691644	-0.16	-1.439	0.03148
A_43_P16069	Qpct	AF039308		-0.16	-1.439	0.073423
A_42_P803726	Tcp11	NM_001007695	309641	-0.16	-1.439	0.01317
A_44_P404472	Rarb	XM_001059523		-0.16	-1.440	0.064263
A_44_P1017013	Ndufa7_predicted	XM_216859		-0.16	-1.440	0.040625
A_44_P606502	TC540977	TC540977		-0.16	-1.440	0.009641
A_44_P206030	LOC498369	XM_001078558		-0.16	-1.440	0.023833
A_44_P147443	Ptp4a1	NM_031579	29463	-0.16	-1.440	0.020643
A_43_P13417	Fzd2	NM_172035	64512	-0.16	-1.440	0.110299
A_44_P450516	XM_237116	XM_237116		-0.16	-1.440	0.039091
A_44_P655613	LOC684906	XM_001072408		-0.16	-1.440	0.077886
A_44_P622138	TC519466	TC519466		-0.16	-1.440	0.109454
A_44_P902836	LOC681908	XM_001058929		-0.16	-1.440	0.111591
A_43_P21439	Rps6ka5_predicted	XM_234468		-0.16	-1.440	0.004239
A_44_P320754	Olr37	NM_001000504	308853	-0.16	-1.441	0.070366
A_43_P20678	Phf19_predicted	XM_231165		-0.16	-1.441	0.157713
A_44_P148416	RGD1305809_predicted	XM_001059467		-0.16	-1.441	0.068548
A_44_P491242	BI282960	BI282960	191576	-0.16	-1.441	0.065699
A_44_P896682	LOC678766	XM_001053087		-0.16	-1.441	0.068454
A_44_P349399	RGD1561499_predicted	XM_226972		-0.16	-1.441	0.096679
A_44_P881750	AW144410	AW144410		-0.16	-1.441	0.143734
A_44_P105119	Klk15_predicted	XM_001080538		-0.16	-1.441	0.011437
A_44_P388673	Hp	NM_012582	24464	-0.16	-1.441	0.043594
A_43_P19006	RGD1563309_predicted	XM_347258	368088	-0.16	-1.441	0.009078
A_42_P743350	LOC683838	XM_001067698		-0.16	-1.441	0.007188
A_44_P926835	AI072072	AI072072	289350	-0.16	-1.441	0.162394
A_44_P140202	XM_344370	XM_344370		-0.16	-1.441	0.399763
A_43_P20588	Tbc1d2_predicted	XM_232990		-0.16	-1.441	0.008399
A_44_P135142	LOC498460	XM_573718		-0.16	-1.441	0.128788

A_44_P355594	AA957096	AA957096		-0.16	-1.441	0.07479
A_44_P440514	RT1-CE4	NM_001008842	414783	-0.16	-1.441	0.013274
A_42_P487852	Plec1	NM_022401	64204	-0.16	-1.442	0.020833
A_44_P422032	Jph2	NM_001037974	296345	-0.16	-1.442	0.222854
A_44_P197213	Zdhhc19	NM_001039259	288045	-0.16	-1.442	0.133525
A_44_P196172	Best5	NM_138881	65190	-0.16	-1.442	0.103042
A_44_P220616	Ccdc42_predicted	XM_220585		-0.16	-1.442	0.034365
A_44_P497218	LOC688807	XM_001068381	688807	-0.16	-1.442	0.045269
A_44_P418675	AA996732	AA996732	56083	-0.16	-1.442	0.012969
A_44_P305660	XM_342320	XM_342320		-0.16	-1.443	0.063092
A_44_P1047715	Urod	XM_342887		-0.16	-1.443	0.01661
A_44_P417714	BE119668	BE119668		-0.16	-1.443	0.203471
A_44_P340220	ENSRNOT00000028944	ENSRNOT00000028944		-0.16	-1.443	0.056484
A_44_P328758	Acpl2	NM_001007710	315939	-0.16	-1.443	0.009305
A_44_P729722	TC519155	TC519155		-0.16	-1.443	0.01726
A_44_P115826	CF976585	CF976585	362436	-0.16	-1.444	0.065412
A_42_P511265	RT1-CE7	NM_001008845	24737	-0.16	-1.444	0.052458
A_44_P179965	AY387076	AY387076		-0.16	-1.444	0.070628
A_44_P330960	A_44_P330960	A_44_P330960		-0.16	-1.444	0.045964
A_43_P21201	XM_233713	XM_233713		-0.16	-1.444	0.014294
A_44_P212813	RGD1309578	NM_001014162	361637	-0.16	-1.444	0.016927
A_44_P302164	LOC689095	XM_001069504	689095	-0.16	-1.444	0.034047
A_44_P975653	Edg2	NM_053936	116744	-0.16	-1.444	0.100367
A_44_P707189	BG666908	BG666908		-0.16	-1.444	0.02228
A_44_P457697	AI113328	AI113328	367901	-0.16	-1.445	0.026361
A_44_P131520	BG377183	BG377183	501747	-0.16	-1.445	0.060073
A_44_P131443	BM383860	BM383860	500300	-0.16	-1.445	0.150173
A_44_P120907	AA924697	AA924697	50681	-0.16	-1.445	0.075724
A_44_P160189	LOC679333	XM_001055867		-0.16	-1.445	0.009369
A_44_P104590	Cdc42bpb	NM_053620	113960	-0.16	-1.446	0.012519
A_44_P619367	RGD1561734_predicted	XM_576628	501201	-0.16	-1.446	0.093162
A_44_P156089	CV074085	CV074085	360697	-0.16	-1.446	0.008293
A_44_P895110	Samd14	NM_001024966	287637	-0.16	-1.446	0.154583
A_44_P152005	XM_341398	XM_341398		-0.16	-1.446	0.072758
A_44_P342657	Vps13d_predicted	XM_233792		-0.16	-1.446	0.012349
A_44_P928692	ENSRNOT00000002541	ENSRNOT00000002541		-0.16	-1.446	0.011847
A_43_P11449	Cpb1	NM_012533	24271	-0.16	-1.446	0.078373
A_44_P271526	Olr1352_predicted	NM_001000953	405301	-0.16	-1.446	0.149985
A_44_P107264	AY321322	AY321322		-0.16	-1.446	0.194275
A_44_P160967	Olr340_predicted	NM_001000253	293785	-0.16	-1.446	0.006805
A_44_P420524	AA894130	AA894130	292770	-0.16	-1.446	0.01735
A_44_P1012866	RGD1309735_predicted	XM_341044		-0.16	-1.446	0.023758
A_44_P180294	XM_344235	XM_344235		-0.16	-1.447	0.011266
A_44_P673292	AW525962	AW525962		-0.16	-1.447	0.017016
A_43_P15648	Foxo1a	XM_342244	84482	-0.16	-1.447	0.052103
A_44_P561166	TC552876	TC552876		-0.16	-1.447	0.360252
A_44_P192083	Gpr120_predicted	XM_215281		-0.16	-1.447	0.004703
A_44_P860166	TC543012	TC543012		-0.16	-1.447	0.010936
A_44_P787848	Jam2	NM_001034004	619374	-0.16	-1.447	0.181585
A_44_P402884	Pitpnm2_predicted	XM_222155		-0.16	-1.447	0.007902
A_44_P509138	XM_343983	XM_343983		-0.16	-1.448	0.011249
A_44_P316561	LOC502663	XM_001080969		-0.16	-1.448	0.017541
A_44_P472338	BQ195982	BQ195982	114632	-0.16	-1.448	0.037497
A_44_P135576	A_44_P135576	A_44_P135576		-0.16	-1.448	0.005098
A_44_P873456	TC532437	TC532437		-0.16	-1.448	0.056205
A_43_P17861	Tcfdp2_predicted	XM_217232		-0.16	-1.448	0.04496
A_43_P10560	Ncald	NM_001024371	553106	-0.16	-1.448	0.009644
A_44_P522760	LOC681814	XM_001058551		-0.16	-1.448	0.354239
A_44_P326031	LOC682925	XM_001064626		-0.16	-1.448	0.075309
A_44_P363691	Ankra2	NM_207595	294679	-0.16	-1.449	0.022723
A_44_P222122	Bcmo1	NM_053648	114106	-0.16	-1.449	0.057536
A_44_P538381	Ppp1r12a	NM_053890	116670	-0.16	-1.449	0.006066
A_42_P704363	AI599292	AI599292		-0.16	-1.449	0.058983

A_44_P198829	Rax	NM_053678	114213	-0.16	-1.449	0.01822
A_44_P884566	DV718743	DV718743		-0.16	-1.449	0.005434
A_43_P19333	RGD1563946_predicted	XM_224715		-0.16	-1.449	0.026203
A_44_P814740	LOC687399	XM_001078361		-0.16	-1.449	0.072774
A_44_P463888	Hdac4_predicted	XM_343629	363287	-0.16	-1.449	0.00247
A_43_P18080	RGD1563532_predicted	XM_001077488		-0.16	-1.449	0.028699
A_44_P996410	Hspb6	NM_138887	192245	-0.16	-1.449	0.011218
A_44_P301221	AI502668	AI502668		-0.16	-1.450	0.019376
A_44_P728865	RGD1565393_predicted	XM_574149	498864	-0.16	-1.450	0.067985
A_44_P845454	A_44_P845454	A_44_P845454		-0.16	-1.450	0.005061
A_44_P246328	A_44_P246328	A_44_P246328		-0.16	-1.450	0.022484
A_44_P486891	Mapk12	NM_021746	60352	-0.16	-1.450	0.032512
A_44_P1030841	RGD1309170_predicted	XM_342349	362047	-0.16	-1.451	0.018984
A_42_P672071	Ccl19_predicted	XM_342824		-0.16	-1.451	0.099852
A_43_P14713	RGD1310383_predicted	XM_001079551		-0.16	-1.451	0.016734
A_43_P10280	BC091324	BC091324		-0.16	-1.451	0.118461
A_44_P386890	XM_344376	XM_344376		-0.16	-1.451	0.153479
A_43_P15745	Mapk8	XM_341399	116554	-0.16	-1.451	0.090807
A_44_P297167	Cacna1e	AY029412	54234	-0.16	-1.451	0.06068
A_42_P805864	LOC684143	XM_001069125		-0.16	-1.451	0.08531
A_44_P330439	LOC500845	AY539882	500845	-0.16	-1.451	0.205601
A_44_P802466	BI288958	BI288958		-0.16	-1.451	0.10943
A_44_P233844	ENSRNOT00000040791	ENSRNOT00000040791		-0.16	-1.452	0.02086
A_44_P531611	Mcf2l	NM_053951		-0.16	-1.452	0.049422
A_44_P304730	MGC93707	NM_001005556	311430	-0.16	-1.452	0.005605
A_44_P853016	TC554359	TC554359		-0.16	-1.452	0.010364
A_44_P1038655	RGD1311859_predicted	XM_220974		-0.16	-1.453	0.014269
A_44_P133519	A_44_P133519	A_44_P133519		-0.16	-1.453	0.090554
A_44_P409662	B3gnt5	XM_344040		-0.16	-1.453	0.093224
A_43_P10924	Capza2	NM_001009180	493810	-0.16	-1.453	0.033743
A_43_P22263	Pcdhga5	NM_001037137	116782	-0.16	-1.453	0.042366
A_44_P262670	XM_227461	XM_227461		-0.16	-1.453	0.011038
A_44_P286369	LOC690755	XM_001075512		-0.16	-1.453	0.01712
A_44_P977056	TC561199	TC561199		-0.16	-1.453	0.020431
A_44_P283790	Mfhas1_predicted	XM_224916		-0.16	-1.453	0.0207
A_44_P396450	LOC686689	XM_001075273		-0.16	-1.453	0.006533
A_44_P767548	TC561984	TC561984		-0.16	-1.453	0.094517
A_44_P178381	RGD1561644_predicted	XM_578646		-0.16	-1.453	0.068499
A_44_P509922	Sgne1	NM_013175	25719	-0.16	-1.453	0.023936
A_44_P432388	Tdh_predicted	XM_214217		-0.16	-1.453	0.134754
A_44_P249161	Dab2ip	NM_138710	192126	-0.16	-1.454	0.037452
A_44_P664802	DV728182	DV728182		-0.16	-1.454	0.005761
A_44_P366079	XM_578513	XM_578513		-0.16	-1.454	0.010596
A_44_P980864	BG380414	BG380414	306748	-0.16	-1.454	0.068452
A_44_P555952	AI137755	AI137755		-0.16	-1.454	0.386155
A_44_P164337	AA892810	AA892810	116565	-0.16	-1.454	0.336389
A_44_P243718	XM_346373	XM_346373		-0.16	-1.454	0.03557
A_44_P440691	Map3k10	XM_218368	308463	-0.16	-1.454	0.008914
A_44_P656251	Spock1	XM_001067546		-0.16	-1.455	0.110057
A_44_P387100	Mical3	XM_001064549	362427	-0.16	-1.455	0.027313
A_43_P12718	Grip1	NM_032069	84016	-0.16	-1.455	0.365877
A_44_P819169	AA997848	AA997848	298836	-0.16	-1.455	0.023961
A_44_P199526	LOC688144	XM_001081287		-0.16	-1.455	0.053532
A_44_P1045734	Stac3_predicted	XM_343222	362895	-0.16	-1.455	0.099449
A_43_P20099	Mfi2_predicted	XM_237839		-0.16	-1.455	0.078743
A_44_P546000	RGD1565438_predicted	XM_344452		-0.16	-1.455	0.008361
A_44_P989658	LOC687913	XM_001080544		-0.16	-1.455	0.061494
A_44_P885558	TC560462	TC560462		-0.16	-1.455	0.223674
A_43_P23135	Jph1_predicted	XM_232578		-0.16	-1.456	0.068143
A_43_P16945	RGD1308124_predicted	XM_218663		-0.16	-1.456	0.006204
A_43_P18571	Tekt4	NM_001013965	302991	-0.16	-1.456	0.059077
A_44_P186165	RGD1562478_predicted	XM_578804		-0.16	-1.456	0.019695
A_42_P780882	XM_215174	XM_215174		-0.16	-1.456	0.007798

A_44_P326233	Fkbp14	NM_001013210	362366	-0.16	-1.456	0.010569
A_44_P167630	AI013756	AI013756	293476	-0.16	-1.456	0.1336
A_44_P520267	Gsh1_predicted	XM_221885		-0.16	-1.456	0.004933
A_44_P429063	A_44_P429063	A_44_P429063		-0.16	-1.456	0.131165
A_44_P138036	LOC502201	XM_577662	502201	-0.16	-1.456	0.035275
A_44_P187674	Insm2_predicted	XM_234255	314131	-0.16	-1.456	0.058895
A_44_P985640	AI555552	AI555552		-0.16	-1.456	0.033001
A_44_P234315	BF289587	BF289587		-0.16	-1.456	0.069457
A_44_P255409	Lrrc56	NM_001024902	365389	-0.16	-1.456	0.020084
A_44_P290391	Slc8a1	NM_019268	29715	-0.16	-1.456	0.045333
A_44_P137047	Rpl37	BQ191785	81770	-0.16	-1.457	0.070144
A_44_P487240	Hmgcs1	NM_017268	29637	-0.16	-1.457	0.120967
A_44_P126886	AA899661	AA899661		-0.16	-1.457	0.005136
A_43_P12126	Hist1h2aa	NM_021839	24828	-0.16	-1.457	0.122864
A_44_P262263	Eya2	NM_130427	156826	-0.16	-1.457	0.091305
A_44_P262173	Prg-2	NM_181634	314614	-0.16	-1.457	0.049312
A_44_P484677	Notch3	NM_020087	56761	-0.16	-1.457	0.216608
A_44_P398376	Nudt18	XM_341351	361068	-0.16	-1.458	0.013523
A_44_P365180	LOC286991	NM_173325	286991	-0.16	-1.458	0.19281
A_44_P504247	XM_340814	XM_340814		-0.16	-1.458	0.17796
A_44_P807408	BE097955	BE097955		-0.16	-1.458	0.006547
A_44_P507118	AA859001	AA859001	315953	-0.16	-1.458	0.073934
A_44_P859396	TC559587	TC559587		-0.16	-1.458	0.073738
A_43_P12892	Spata2	NM_053675	114210	-0.16	-1.458	0.003565
A_44_P123773	LOC361623	XM_341901	361623	-0.16	-1.458	0.248138
A_42_P753215	Synj2	NM_032071		-0.16	-1.458	0.006044
A_44_P213936	BF522788	BF522788		-0.16	-1.459	0.416772
A_44_P319180	LOC366669	XM_345689	366669	-0.16	-1.459	0.005081
A_44_P280376	AI009427	AI009427	291983	-0.16	-1.459	0.016904
A_44_P409043	BI301453	BI301453	29593	-0.16	-1.459	0.010528
A_44_P501408	LOC685765	XM_001065183	365390	-0.16	-1.459	0.099284
A_43_P15367	Lmbrd1	NM_139189	246046	-0.16	-1.459	0.028385
A_44_P559011	AW529628	AW529628	689588	-0.16	-1.459	0.044458
A_44_P103020	LOC499941	XM_575284		-0.16	-1.460	0.02263
A_44_P445074	Olr283_predicted	NM_001001010	405384	-0.16	-1.460	0.06182
A_44_P502711	AA925350	AA925350	79438	-0.16	-1.460	0.02644
A_44_P264024	CR756125	CR756125	313589	-0.16	-1.460	0.120606
A_44_P447465	Olr875_predicted	NM_001000054	288789	-0.16	-1.460	0.164405
A_44_P231439	Slc39a11	NM_001013042	287796	-0.16	-1.460	0.004727
A_44_P172426	AI011747	AI011747	84400	-0.16	-1.460	0.061997
A_44_P758912	BF522863	BF522863	287533	-0.16	-1.460	0.011172
A_44_P555042	Olr157_predicted	NM_001000169	293304	-0.16	-1.460	0.110956
A_44_P260589	Papln_predicted	XM_234393		-0.16	-1.461	0.065313
A_44_P939814	AI511420	AI511420		-0.16	-1.461	0.118306
A_44_P332675	Myh13	XM_001078857		-0.16	-1.461	0.105953
A_44_P546279	Galm	NM_001007704	313843	-0.16	-1.461	0.028704
A_43_P22541	RGD1308772_predicted	XM_214237	290381	-0.16	-1.461	0.114084
A_44_P387466	AA818877	AA818877	291964	-0.16	-1.461	0.023068
A_44_P147736	XM_344030	XM_344030		-0.16	-1.461	0.149995
A_44_P142117	Foxd3	XM_575873	29203	-0.16	-1.461	0.023561
A_44_P744605	Arsj	XM_227873		-0.16	-1.461	0.37995
A_44_P387531	AA900591	AA900591		-0.16	-1.462	0.149008
A_44_P131336	BE112615	BE112615		-0.16	-1.462	0.174043
A_42_P606348	AW143874	AW143874		-0.16	-1.462	0.123969
A_44_P767589	BE103187	BE103187	312707	-0.16	-1.462	0.020986
A_43_P18448	Apeg3	XM_218226		-0.16	-1.462	0.014093
A_44_P342239	Dstn	NM_001033666	502674	-0.16	-1.462	0.027254
A_44_P452818	A_44_P452818	A_44_P452818		-0.16	-1.462	0.039744
A_44_P145290	Pvrl3_predicted	XM_213626		-0.16	-1.462	0.009908
A_44_P466314	Tacc2	NM_001004415	309025	-0.16	-1.462	0.140734
A_44_P470134	BF545583	BF545583	288167	-0.17	-1.462	0.155519
A_44_P291120	A_44_P291120	A_44_P291120		-0.17	-1.462	0.043585
A_43_P16346	LOC207127	U89745	207127	-0.17	-1.462	0.007706

A_44_P469920	LOC294560	XM_215428		-0.17	-1.463	0.008974
A_44_P237731	AA964583	AA964583	361542	-0.17	-1.463	0.034241
A_44_P419833	XM_345778	XM_345778		-0.17	-1.463	0.087296
A_44_P383740	Cdc2l5	XM_225404	306998	-0.17	-1.463	0.073874
A_43_P21864	CB547155	CB547155		-0.17	-1.463	0.002815
A_44_P325221	AA955661	AA955661	307348	-0.17	-1.463	0.166507
A_44_P717914	TC560474	TC560474		-0.17	-1.463	0.277632
A_44_P808710	LOC684158	XM_001069205		-0.17	-1.463	0.086171
A_44_P319322	Krt1-12	NM_001008761	360625	-0.17	-1.463	0.043281
A_44_P238257	Trip10	NM_053920	116717	-0.17	-1.463	0.003429
A_44_P112030	RGD1560543_predicted	XM_574327		-0.17	-1.463	0.011298
A_44_P241133	XM_346927	XM_346927		-0.17	-1.463	0.024776
A_44_P524718	Serpina1	NM_022519	24648	-0.17	-1.464	0.086012
A_44_P1006056	LOC688211	XM_001081514		-0.17	-1.464	0.031951
A_44_P405083	Cacna1i	NM_020084	56827	-0.17	-1.464	0.028118
A_43_P19862	RGD1565716_predicted	XM_216733	299129	-0.17	-1.464	0.011059
A_44_P175125	Olr1567_predicted	NM_001000043	287970	-0.17	-1.464	0.011676
A_44_P658594	CO575464	CO575464		-0.17	-1.464	0.022629
A_44_P505125	AI171794	AI171794	116639	-0.17	-1.464	0.039198
A_44_P461970	LOC316820	XM_229242		-0.17	-1.464	0.242564
A_44_P246114	Tle1_predicted	XM_342851	362533	-0.17	-1.464	0.148677
A_44_P178519	Adamts7_predicted	XM_236471		-0.17	-1.464	0.017516
A_44_P380615	AI137249	AI137249	500377	-0.17	-1.465	0.109419
A_44_P399999	Suclg1	NM_053752	114597	-0.17	-1.465	0.006586
A_44_P120626	AA957419	AA957419		-0.17	-1.465	0.161042
A_42_P800916	Tdo2	NM_022403	64206	-0.17	-1.465	0.027829
A_43_P11460	Ela1	NM_012552	24331	-0.17	-1.465	0.005428
A_44_P499642	AI233728	AI233728	304528	-0.17	-1.465	0.006833
A_43_P18473	Mmp17_predicted	XM_239639		-0.17	-1.465	0.021261
A_44_P153437	AA800216	AA800216	498587	-0.17	-1.465	0.085018
A_43_P22037	Sall3_predicted	XM_344708		-0.17	-1.466	0.156912
A_44_P760599	Mtac2d1	NM_001025152	500707	-0.17	-1.466	0.060957
A_42_P481516	Atp5b	NM_134364	171374	-0.17	-1.466	0.009808
A_44_P220385	A_44_P220385	A_44_P220385		-0.17	-1.466	0.055366
A_44_P491489	AB033713	AB033713		-0.17	-1.466	0.025221
A_44_P443171	Hipk1_predicted	XM_345267		-0.17	-1.466	0.006604
A_43_P20926	Tbx19_predicted	XM_222834		-0.17	-1.466	0.022494
A_44_P147381	Tmc2_predicted	XM_230602		-0.17	-1.466	0.048064
A_44_P165698	Otp	AY169319	294640	-0.17	-1.467	0.023751
A_44_P498516	CB544878	CB544878	290561	-0.17	-1.467	0.014845
A_44_P113790	BF408957	BF408957	308759	-0.17	-1.467	0.096154
A_44_P714771	TC518464	TC518464		-0.17	-1.467	0.080135
A_44_P763205	TC562867	TC562867		-0.17	-1.467	0.10237
A_44_P154270	RGD1359349	NM_001007738	361744	-0.17	-1.467	0.02759
A_44_P236271	Hoxc8_mapped	XM_347335	368178	-0.17	-1.467	0.028562
A_44_P143223	CB606387	CB606387		-0.17	-1.467	0.148425
A_44_P372686	Rp1l1_predicted	XM_001066326		-0.17	-1.467	0.034004
A_44_P869863	TC526455	TC526455		-0.17	-1.467	0.03443
A_44_P625333	TC538192	TC538192		-0.17	-1.467	0.043965
A_43_P15885	Cttnbp2	XM_347230	282587	-0.17	-1.468	0.095576
A_44_P837846	DV723320	DV723320		-0.17	-1.468	0.016365
A_44_P977085	LOC680885	XM_001059309		-0.17	-1.468	0.14008
A_44_P373005	Wwp1	NM_001024757	297930	-0.17	-1.468	0.167967
A_44_P203549	Egln3	NM_019371	54702	-0.17	-1.468	0.036043
A_43_P11936	Rab10	M83677	50993	-0.17	-1.468	0.032591
A_44_P376862	Cldn3	NM_031700	65130	-0.17	-1.468	0.007886
A_44_P798344	BI291339	BI291339	500088	-0.17	-1.468	0.07058
A_44_P533146	BI285124	BI285124		-0.17	-1.469	0.016782
A_44_P537780	AA996504	AA996504	24471	-0.17	-1.469	0.006595
A_44_P726050	BF565756	BF565756		-0.17	-1.469	0.175667
A_44_P221260	AA955669	AA955669	79239	-0.17	-1.469	0.01039
A_44_P274976	BF550822	BF550822		-0.17	-1.469	0.137729
A_44_P300818	RGD1560454_predicted	XM_001080836		-0.17	-1.469	0.006916

A_44_P490708	AA924149	AA924149	362741	-0.17	-1.469	0.06244
A_44_P716145	Cxadr	NM_053570	89843	-0.17	-1.469	0.016306
A_42_P832637	Dirc2	NM_001012017	303902	-0.17	-1.470	0.008749
A_44_P710331	BX883042	BX883042		-0.17	-1.470	0.042222
A_43_P11966	Msmb	NM_019188	29311	-0.17	-1.470	0.035858
A_44_P309810	AA900268	AA900268		-0.17	-1.470	0.009494
A_44_P308141	BI292191	BI292191	360626	-0.17	-1.470	0.007216
A_44_P884739	Sez6l2_predicted	XM_219339		-0.17	-1.470	0.080555
A_44_P1046338	Tbc1d23_predicted	XM_221532		-0.17	-1.470	0.007768
A_44_P228485	AA899715	AA899715	155151	-0.17	-1.470	0.020628
A_42_P708614	Ndufa6_predicted	XM_235518	315167	-0.17	-1.470	0.004015
A_44_P353797	Neurog3	NM_021700	60329	-0.17	-1.471	0.011838
A_44_P477376	Nr3c1	NM_012576	24413	-0.17	-1.471	0.031252
A_44_P449663	Olr1602_predicted	NM_001000909	405219	-0.17	-1.471	0.272229
A_44_P508286	LOC679507	XM_001055381		-0.17	-1.471	0.045347
A_44_P165454	AI232793	AI232793		-0.17	-1.471	0.004727
A_44_P340163	Plcb4	NM_024353	25031	-0.17	-1.471	0.027511
A_44_P173465	A_44_P173465	A_44_P173465		-0.17	-1.471	0.034331
A_44_P255521	Efnb2_predicted	XM_225050		-0.17	-1.471	0.064144
A_44_P536958	Arhgap5	XM_216688		-0.17	-1.471	0.010182
A_44_P377492	Dctn6_predicted	XM_214362		-0.17	-1.471	0.03759
A_44_P181503	Suclg2	XM_001074551		-0.17	-1.471	0.008808
A_44_P229641	TC546134	TC546134		-0.17	-1.471	0.018629
A_44_P214873	Arntl	NM_024362	29657	-0.17	-1.471	0.047018
A_44_P477135	Vhl	NM_052801	24874	-0.17	-1.471	0.037322
A_44_P475670	A_44_P475670	A_44_P475670		-0.17	-1.471	0.035503
A_44_P243111	Rab3d	NM_080580	140665	-0.17	-1.472	0.010969
A_44_P406985	Vrk3	NM_001005561	361565	-0.17	-1.472	0.044785
A_43_P21956	RGD1563706_predicted	XM_342343		-0.17	-1.472	0.044712
A_44_P485190	RGD1565414_predicted	XM_227139	310416	-0.17	-1.472	0.006849
A_44_P951676	LOC497899	NM_001017472	497899	-0.17	-1.472	0.058504
A_44_P436352	Pdzd11_predicted	XM_217572		-0.17	-1.472	0.041093
A_44_P513499	Tspan18_predicted	XM_230297		-0.17	-1.472	0.110122
A_44_P174982	Pnpo	NM_022601	64533	-0.17	-1.472	0.107448
A_44_P527018	Olr386_predicted	NM_001000856	405147	-0.17	-1.472	0.164053
A_43_P16193	Kcna6	XM_575671	64358	-0.17	-1.472	0.267257
A_44_P505869	AY387092	AY387092		-0.17	-1.472	0.085357
A_44_P471294	A_44_P471294	A_44_P471294		-0.17	-1.472	0.030524
A_44_P291102	RGD1565316_predicted	XM_235445	315091	-0.17	-1.473	0.017268
A_44_P1046816	Stx8	NM_031656	59074	-0.17	-1.473	0.025956
A_44_P849314	AI136114	AI136114	304301	-0.17	-1.473	0.026105
A_44_P794875	TC559286	TC559286		-0.17	-1.473	0.009096
A_44_P231667	XM_218428	XM_218428		-0.17	-1.473	0.049036
A_44_P250403	Slc16a8	NM_031744	65200	-0.17	-1.473	0.044392
A_44_P1025476	Krt1-18	NM_053976	294853	-0.17	-1.474	0.025941
A_44_P760927	AW142524	AW142524	296279	-0.17	-1.474	0.010261
A_44_P367610	Olr104_predicted	NM_001000145	293243	-0.17	-1.474	0.215295
A_44_P899831	TC522894	TC522894		-0.17	-1.474	0.01893
A_44_P433383	S58644	S58644		-0.17	-1.474	0.258287
A_44_P153063	AA799575	AA799575		-0.17	-1.474	0.071854
A_44_P511079	RGD1309020	XM_214453		-0.17	-1.474	0.016391
A_44_P378691	AI713206	AI713206	317370	-0.17	-1.474	0.050247
A_44_P660703	AW144003	AW144003		-0.17	-1.474	0.017674
A_44_P577044	TC541432	TC541432		-0.17	-1.474	0.027254
A_44_P810009	TC534125	TC534125		-0.17	-1.475	0.02211
A_44_P470918	XM_219425	XM_219425		-0.17	-1.475	0.036661
A_44_P851614	AA859669	AA859669		-0.17	-1.475	0.064218
A_44_P210317	Olr651_predicted	NM_001000341	295858	-0.17	-1.475	0.036872
A_44_P337424	Ppfia4	XM_573454	140592	-0.17	-1.475	0.044389
A_43_P11951	Ucn	NM_019150	29151	-0.17	-1.475	0.016727
A_44_P502696	AA875523	AA875523	362816	-0.17	-1.475	0.011016
A_44_P1052607	Aox1	NM_019363	54349	-0.17	-1.475	0.148638
A_44_P684653	TC540835	TC540835		-0.17	-1.475	0.041871

A_44_P302613	Lkap	NM_133421	170946	-0.17	-1.475	0.006823
A_44_P162224	CB545165	CB545165		-0.17	-1.476	0.009806
A_44_P485835	AA900949	AA900949		-0.17	-1.476	0.124577
A_44_P663461	BF290287	BF290287	365410	-0.17	-1.476	0.008122
A_44_P428184	AI009411	AI009411	140654	-0.17	-1.476	0.063393
A_44_P333518	AA818565	AA818565		-0.17	-1.476	0.122411
A_44_P503932	ENSRNOT00000049357	ENSRNOT00000049357		-0.17	-1.476	0.030163
A_44_P206068	Pdzd7_predicted	XM_219940		-0.17	-1.476	0.053088
A_44_P253755	AA899663	AA899663		-0.17	-1.476	0.159866
A_44_P305169	AA900394	AA900394		-0.17	-1.476	0.207515
A_44_P274688	LOC685883	XM_001065619		-0.17	-1.477	0.034479
A_44_P455051	Ferd3l_predicted	XM_345658		-0.17	-1.477	0.016565
A_44_P283702	LOC688939	XM_001068901	688939	-0.17	-1.477	0.017815
A_44_P178503	RGD1566314_predicted	XM_221452	288106	-0.17	-1.477	0.078444
A_44_P247810	Olr327_predicted	NM_001000249	293765	-0.17	-1.477	0.132934
A_44_P201876	Ddx11_predicted	XM_237570	316767	-0.17	-1.477	0.197693
A_44_P168025	Olr796_predicted	NM_001000600	404788	-0.17	-1.477	0.095197
A_43_P13854	CB547744	CB547744		-0.17	-1.478	0.021302
A_44_P390475	AI030179	AI030179	436582	-0.17	-1.478	0.057059
A_44_P480757	Cachd1_predicted	XM_233227	298267	-0.17	-1.478	0.003925
A_44_P410348	Card12_predicted	XM_216640		-0.17	-1.478	0.022529
A_44_P634909	BI294052	BI294052	361504	-0.17	-1.478	0.007516
A_44_P279337	LOC303140	XM_220427	24915	-0.17	-1.478	0.052661
A_44_P474722	AI043958	AI043958		-0.17	-1.478	0.080319
A_44_P178348	ENSRNOT00000000387	ENSRNOT00000000387		-0.17	-1.478	0.02797
A_44_P443262	A_44_P443262	A_44_P443262		-0.17	-1.478	0.038164
A_44_P436873	Ogdhl_predicted	XM_214261		-0.17	-1.479	0.10634
A_43_P15145	TC532166	TC532166		-0.17	-1.479	0.068085
A_42_P479348	RGD1311698_predicted	XM_343107	362781	-0.17	-1.479	0.217377
A_43_P12697	Vamp8	NM_031827	83730	-0.17	-1.479	0.005352
A_44_P930637	Cpeb2_predicted	XM_341227		-0.17	-1.479	0.006057
A_44_P504244	RGD1562317_predicted	XM_237146		-0.17	-1.479	0.006067
A_44_P380070	RT1-M10-1	NM_001008851	414787	-0.17	-1.479	0.035708
A_44_P1036091	Zfp313	NM_001001517	362277	-0.17	-1.479	0.043574
A_44_P292669	Hoxa5	XM_001059031		-0.17	-1.479	0.013529
A_44_P925187	AW143490	AW143490		-0.17	-1.479	0.030603
A_44_P471082	LOC690132	XM_001073397		-0.17	-1.479	0.006345
A_44_P551169	B4galt4	NM_001012018	303923	-0.17	-1.479	0.382998
A_42_P728204	Gimap1	NM_001034849	312312	-0.17	-1.479	0.028704
A_44_P550712	XM_238225	XM_238225		-0.17	-1.479	0.029572
A_44_P251706	Slc1a1	NM_013032	25550	-0.17	-1.479	0.070451
A_44_P215562	LOC364964	XR_006098	364964	-0.17	-1.479	0.004638
A_44_P185219	Brpf3_predicted	XM_228039		-0.17	-1.479	0.005628
A_44_P400884	XM_345073	XM_345073		-0.17	-1.479	0.024929
A_42_P775217	Ly6g6c	NM_001001969	294241	-0.17	-1.480	0.08242
A_44_P704794	A_44_P704794	A_44_P704794		-0.17	-1.480	0.007329
A_44_P334875	Olr859_predicted	NM_001000585	366458	-0.17	-1.480	0.115997
A_44_P977969	TC550079	TC550079		-0.17	-1.480	0.069518
A_44_P247966	Nell1	NM_031069	81733	-0.17	-1.480	0.045763
A_44_P178616	BG378334	BG378334	361070	-0.17	-1.480	0.061563
A_44_P479267	AA996808	AA996808	301509	-0.17	-1.480	0.108411
A_44_P239849	BE095833	BE095833	360983	-0.17	-1.481	0.111203
A_44_P1024900	Lrp5_predicted	XM_215187		-0.17	-1.481	0.003919
A_44_P101644	Rock2	NM_013022	25537	-0.17	-1.481	0.010736
A_43_P12980	Ceacam9	NM_053919	116711	-0.17	-1.481	0.006396
A_44_P137641	A_44_P137641	A_44_P137641		-0.17	-1.481	0.057335
A_44_P255078	E230034O05Rik	NR_002154		-0.17	-1.481	0.011588
A_44_P231944	RGD1307465	NM_001009655	296200	-0.17	-1.481	0.038757
A_43_P18417	RGD1562629_predicted	XM_001059612		-0.17	-1.481	0.01659
A_43_P11159	CB544933	CB544933	305795	-0.17	-1.481	0.096329
A_44_P540034	AI012671	AI012671	291411	-0.17	-1.481	0.06591
A_44_P1045179	AI231349	AI231349	84510	-0.17	-1.481	0.024556
A_44_P766678	BF557813	BF557813		-0.17	-1.481	0.110917

A_44_P210977	A_44_P210977	A_44_P210977		-0.17	-1.482	0.003725
A_44_P429919	M54999	M54999		-0.17	-1.482	0.028679
A_44_P165328	AW914042	AW914042		-0.17	-1.482	0.054528
A_43_P15500	Adam7	NM_020301	29641	-0.17	-1.482	0.175732
A_44_P798113	A_44_P798113	A_44_P798113		-0.17	-1.482	0.099298
A_44_P853677	LOC365592	NM_001014249	365592	-0.17	-1.482	0.017393
A_44_P412533	Dcst1_predicted	XM_001074533		-0.17	-1.482	0.046966
A_44_P333146	LOC499903	XM_575249		-0.17	-1.482	0.022362
A_44_P478541	LOC679476	XM_001056553		-0.17	-1.482	0.124366
A_44_P469955	AI071299	AI071299	81813	-0.17	-1.482	0.026095
A_44_P286087	RGD1311161	NM_001014142	361151	-0.17	-1.482	0.012676
A_44_P489547	Nova2_predicted	XM_001075246		-0.17	-1.482	0.144716
A_44_P427570	AA924663	AA924663		-0.17	-1.482	0.048798
A_43_P17094	RGD1561916_predicted	XM_001068622		-0.17	-1.483	0.002674
A_44_P655212	TC529832	TC529832		-0.17	-1.483	0.021336
A_44_P297277	LOC365985	XM_001080050		-0.17	-1.483	0.030771
A_44_P424194	A_44_P424194	A_44_P424194		-0.17	-1.483	0.054536
A_44_P259867	Olr262_predicted	NM_001000221	293400	-0.17	-1.483	0.107169
A_44_P389123	Plekha6_predicted	XM_341118	360842	-0.17	-1.484	0.040528
A_44_P122632	B2m	CF111193	24223	-0.17	-1.484	0.011676
A_44_P762686	TC543725	TC543725		-0.17	-1.484	0.008115
A_44_P819950	AA924747	AA924747	29236	-0.17	-1.484	0.108735
A_44_P159877	AW252149	AW252149	307362	-0.17	-1.484	0.016694
A_44_P550095	AA799582	AA799582	117282	-0.17	-1.484	0.117727
A_44_P546632	BF559342	BF559342		-0.17	-1.484	0.054135
A_44_P479754	AW919892	AW919892		-0.17	-1.484	0.042496
A_44_P198020	BF405174	BF405174		-0.17	-1.484	0.027498
A_44_P113842	Max	NM_022210	60661	-0.17	-1.485	0.005557
A_44_P301399	BF289458	BF289458		-0.17	-1.485	0.216809
A_44_P222186	Pdzk1	NM_031712	65144	-0.17	-1.485	0.04838
A_44_P685740	TC544896	TC544896		-0.17	-1.485	0.088792
A_44_P266928	Surf1	NM_172068	64463	-0.17	-1.485	0.010215
A_44_P480916	RGD1310862_predicted	XM_220273	303016	-0.17	-1.485	0.081611
A_44_P514186	BI275705	BI275705	60666	-0.17	-1.485	0.039452
A_42_P511375	Abhd14a	NM_001009670	300982	-0.17	-1.485	0.091922
A_44_P448051	Vpreb3_predicted	XM_345093		-0.17	-1.485	0.07022
A_42_P780997	Atp5e	NM_139099	245958	-0.17	-1.485	0.031389
A_44_P294799	Adh4	AY310136	29646	-0.17	-1.485	0.0291
A_44_P261290	AA818860	AA818860	291023	-0.17	-1.485	0.005465
A_44_P939778	AA955391	AA955391	64474	-0.17	-1.486	0.165534
A_44_P503503	Irs2	XM_573948	29376	-0.17	-1.486	0.019955
A_44_P474424	BI297929	BI297929	500847	-0.17	-1.486	0.010333
A_42_P711464	Ccdc19	NM_001024882	304984	-0.17	-1.486	0.010587
A_43_P14440	Mtus1	BC072537	306487	-0.17	-1.486	0.054275
A_43_P23317	Rsnl2	NM_001013942	298801	-0.17	-1.486	0.357069
A_44_P107792	XM_229658	XM_229658		-0.17	-1.486	0.09141
A_44_P951522	BF564518	BF564518		-0.17	-1.486	0.091007
A_43_P19625	Rutbc2_predicted	XM_222270		-0.17	-1.487	0.002943
A_44_P133366	Tulp1_predicted	XM_228360		-0.17	-1.487	0.015531
A_44_P206409	Tmem30b_predicted	XM_234295		-0.17	-1.487	0.018271
A_44_P425143	Grin2d	NM_022797	24412	-0.17	-1.487	0.138093
A_43_P21192	XM_342850	XM_342850		-0.17	-1.487	0.116666
A_44_P432910	Cox6a1	NM_012814	25282	-0.17	-1.487	0.028219
A_44_P509035	XM_235328	XM_235328		-0.17	-1.487	0.026638
A_44_P556299	BF551363	BF551363		-0.17	-1.487	0.005221
A_43_P22280	Myom1	XM_237523	316740	-0.17	-1.487	0.027323
A_44_P344875	Reep1_predicted	XM_342708		-0.17	-1.487	0.096099
A_44_P443200	RGD1563247_predicted	XM_344630		-0.17	-1.487	0.099705
A_44_P459666	Ttc22_predicted	XM_233266		-0.17	-1.488	0.019502
A_44_P152500	AA956059	AA956059	500420	-0.17	-1.488	0.093226
A_43_P12602	Slc37a4	NM_031589	29573	-0.17	-1.488	0.006001
A_42_P755399	Dym_predicted	XM_214529		-0.17	-1.488	0.037347
A_44_P150126	AA924263	AA924263	300754	-0.17	-1.488	0.08733

A_44_P901064	TC526465	TC526465		-0.17	-1.488	0.012374
A_44_P640345	TC533553	TC533553		-0.17	-1.488	0.145824
A_44_P391952	RGD1310819_predicted	XM_237064	301351	-0.17	-1.488	0.008766
A_44_P243727	Yipf4	NM_001009712	362699	-0.17	-1.488	0.011458
A_44_P689395	Cox8h	AI103885	25250	-0.17	-1.489	0.015368
A_44_P671041	TC531904	TC531904		-0.17	-1.489	0.019376
A_43_P15815	Cntfr	NM_001003929	313173	-0.17	-1.489	0.053031
A_42_P475458	Pbx3_predicted	XM_231158		-0.17	-1.489	0.009843
A_44_P302155	B3gnt1_predicted	XM_223674		-0.17	-1.489	0.012892
A_44_P112322	RGD1308023_predicted	XM_001055483		-0.17	-1.489	0.060984
A_44_P542338	Epha1_predicted	XM_001072130		-0.17	-1.490	0.01626
A_42_P664699	Apbb1	NM_080478	29722	-0.17	-1.490	0.186882
A_44_P133611	Mrpl14_predicted	XM_217350		-0.17	-1.490	0.005263
A_44_P927936	LOC689046	XM_001060013	689046	-0.17	-1.490	0.003715
A_44_P272502	AA851305	AA851305		-0.17	-1.490	0.238228
A_44_P154498	RGD1559747_predicted	XM_231725		-0.17	-1.490	0.01242
A_44_P884777	TC523432	TC523432		-0.17	-1.490	0.018098
A_43_P15405	Hpca	NM_017122	29177	-0.17	-1.491	0.068493
A_44_P962846	Dock5_predicted	XM_001067769		-0.17	-1.491	0.016654
A_44_P137035	BI276726	BI276726	313323	-0.17	-1.491	0.008151
A_44_P993027	Hrasls3	NM_017060	24913	-0.17	-1.491	0.024053
A_44_P1015733	RGD1309400_predicted	XM_213403		-0.17	-1.491	0.004145
A_43_P12378	Golph3	NM_023977	78961	-0.17	-1.491	0.006944
A_44_P792341	TC557578	TC557578		-0.17	-1.491	0.021108
A_44_P1050755	Abp10	XM_001060944		-0.17	-1.491	0.06511
A_44_P150317	BF550562	BF550562		-0.17	-1.491	0.014481
A_44_P744700	ENSRNOT00000010246	ENSRNOT00000010246		-0.17	-1.491	0.198332
A_44_P982240	A_44_P982240	A_44_P982240		-0.17	-1.492	0.020476
A_44_P392524	CK600504	CK600504		-0.17	-1.492	0.104704
A_44_P293143	Kcng4_predicted	XM_226524		-0.17	-1.492	0.015748
A_42_P829349	Comtd1_predicted	XM_223785		-0.17	-1.492	0.004258
A_44_P382562	XM_227162	XM_227162		-0.17	-1.492	0.012423
A_44_P448388	AI112115	AI112115	296655	-0.17	-1.493	0.049571
A_44_P447635	Cplx2	NM_053878	116657	-0.17	-1.493	0.008708
A_44_P649412	AI011243	AI011243	84398	-0.17	-1.493	0.016151
A_44_P220054	RGD1561961_predicted	XM_213926	289181	-0.17	-1.493	0.003823
A_44_P257419	Olr132_predicted	NM_001001273	293271	-0.17	-1.493	0.150711
A_44_P536932	Uqcrh	NM_001009480	366448	-0.17	-1.493	0.005097
A_43_P19564	RGD1305574	XM_341388	361102	-0.17	-1.493	0.042749
A_44_P607752	TC541668	TC541668		-0.17	-1.493	0.146945
A_44_P896414	AI012782	AI012782		-0.17	-1.493	0.014678
A_44_P162683	AI014074	AI014074	58964	-0.17	-1.493	0.017055
A_43_P11671	Sstr4	NM_013036	25555	-0.17	-1.494	0.022631
A_44_P201349	Tparl	NM_001024802	364137	-0.17	-1.494	0.015003
A_44_P238710	Tjp1_predicted	XM_218747		-0.17	-1.494	0.014631
A_44_P384286	Gpr20	NM_022216	60667	-0.17	-1.494	0.032789
A_44_P384236	Cdx1_predicted	XM_344691		-0.17	-1.494	0.011393
A_44_P423458	AI100773	AI100773	691193	-0.17	-1.494	0.008989
A_44_P125575	AA859178	AA859178	171374	-0.17	-1.494	0.058056
A_44_P676250	TC529741	TC529741		-0.17	-1.494	0.1345
A_44_P366888	AW916827	AW916827	309809	-0.17	-1.494	0.024265
A_44_P375029	Dact2_predicted	XM_218002		-0.17	-1.495	0.007372
A_43_P19741	Crygn_predicted	XM_216055		-0.17	-1.495	0.031688
A_44_P316651	Klhl15_predicted	XM_234162		-0.17	-1.495	0.026267
A_42_P503419	Gprc5c	XM_213518	287805	-0.17	-1.495	0.027602
A_44_P107330	LOC246295	XM_575968	246295	-0.17	-1.495	0.039267
A_44_P253178	XM_345078	XM_345078		-0.17	-1.495	0.042183
A_44_P398128	Slc22a12	NM_001034943	365398	-0.17	-1.495	0.017217
A_42_P774468	MGC94339	NM_001007703	313771	-0.17	-1.495	0.024429
A_44_P999066	hshin7	NM_001008889	363982	-0.17	-1.495	0.026277
A_44_P1006931	XM_214536	XM_214536		-0.17	-1.496	0.124835
A_42_P755133	LOC686963	XM_001076515		-0.17	-1.496	0.017941
A_43_P13335	Foxe1	NM_138909	192274	-0.17	-1.496	0.03476

A_44_P525125	XM_219069	XM_219069		-0.17	-1.496	0.071975
A_44_P338059	RGD1562665_predicted	XM_343264	362936	-0.17	-1.496	0.003215
A_44_P825542	LOC312863	NM_001014061	312863	-0.17	-1.496	0.016211
A_44_P978901	Tjp1_predicted	XM_218747		-0.17	-1.496	0.015902
A_44_P240375	BI282031	BI282031	296571	-0.18	-1.496	0.03759
A_44_P136612	AI070005	AI070005	79115	-0.18	-1.497	0.013675
A_44_P288134	AB072242	AB072242		-0.18	-1.497	0.030439
A_44_P381420	AI169092	AI169092	25357	-0.18	-1.497	0.035395
A_44_P330652	Brsk2	XM_219498	293631	-0.18	-1.497	0.00473
A_43_P16380	Olr1356	NM_001000493	302954	-0.18	-1.497	0.026376
A_44_P355733	BF282908	BF282908	362851	-0.18	-1.498	0.02626
A_44_P134825	Tbce	NM_001012161	361255	-0.18	-1.498	0.008875
A_44_P328717	Mtm1	NM_001013047	288762	-0.18	-1.498	0.003012
A_44_P262274	Hoxa5	XM_001059031		-0.18	-1.498	0.015918
A_44_P148119	BQ200023	BQ200023	83805	-0.18	-1.498	0.019632
A_44_P105327	RGD1561546_predicted	XM_229383		-0.18	-1.498	0.167226
A_43_P16651	Cpeb4_predicted	XM_220269		-0.18	-1.498	0.012012
A_44_P351490	Cd82	NM_031797	83628	-0.18	-1.499	0.011993
A_42_P779933	Capn3	NM_017117	29155	-0.18	-1.499	0.088044
A_44_P304075	Taar7h	NM_175587	294130	-0.18	-1.499	0.149522
A_44_P225323	XM_346896	XM_346896		-0.18	-1.499	0.152989
A_44_P715158	TC520950	TC520950		-0.18	-1.499	0.034158
A_44_P321496	RGD1561426_predicted	XM_347067	362633	-0.18	-1.499	0.030157
A_42_P506453	AW914895	AW914895	296972	-0.18	-1.499	0.067384
A_43_P11228	AI235052	AI235052		-0.18	-1.500	0.037443
A_44_P447623	Ugdh	NM_031325	83472	-0.18	-1.500	0.01452
A_44_P278539	Olr1409_predicted	NM_001000004	287259	-0.18	-1.500	0.043449
A_44_P529743	RGD1562983_predicted	XM_574587		-0.18	-1.500	0.048528
A_44_P604354	AI145199	AI145199		-0.18	-1.500	0.055861
A_43_P12655	Slc27a2	NM_031736	65192	-0.18	-1.500	0.009168
A_44_P514987	Sdccag33_predicted	XM_225688	307217	-0.18	-1.500	0.101475
A_44_P405087	U95152	U95152		-0.18	-1.500	0.01111
A_44_P368294	Mpl_predicted	XM_345572	366455	-0.18	-1.500	0.110184
A_43_P15998	Ntn2l	XM_343867	114524	-0.18	-1.500	0.061
A_44_P353672	Kcnd2	NM_031730	65180	-0.18	-1.500	0.062443
A_44_P547197	AI029183	AI029183	24392	-0.18	-1.501	0.009785
A_44_P557444	NP798074	NP798074		-0.18	-1.501	0.013228
A_44_P856998	CO554029	CO554029	116666	-0.18	-1.501	0.02107
A_44_P345961	RGD1562793_predicted	XM_575489		-0.18	-1.501	0.055228
A_44_P998234	Lig4_predicted	XM_001067682		-0.18	-1.501	0.029836
A_44_P511145	RGD1562809_predicted	XM_230703		-0.18	-1.501	0.033412
A_44_P538602	Copb2	NM_021765	60384	-0.18	-1.501	0.031734
A_44_P997940	XM_214671	XM_214671		-0.18	-1.501	0.039991
A_44_P457488	BG668469	BG668469		-0.18	-1.501	0.011734
A_44_P248983	AA956558	AA956558	296293	-0.18	-1.501	0.019914
A_42_P634149	Cryba2	NM_173140	286925	-0.18	-1.501	0.09618
A_43_P11037	Nkx6-2_predicted	XM_001058301		-0.18	-1.501	0.034894
A_44_P123918	Slc35d2_predicted	XM_214408		-0.18	-1.501	0.097752
A_44_P349365	XM_344334	XM_344334		-0.18	-1.501	0.024169
A_44_P122620	BM389829	BM389829	315550	-0.18	-1.501	0.044918
A_44_P296622	AW144045	AW144045	59114	-0.18	-1.501	0.007573
A_44_P363442	LOC365107	XM_001063111		-0.18	-1.501	0.004326
A_44_P194909	Ripk4_predicted	XM_221619		-0.18	-1.501	0.02111
A_44_P320834	Man2a1	XM_001068826	25478	-0.18	-1.501	0.023195
A_44_P547056	Rab24	NM_001015023	361208	-0.18	-1.502	0.009348
A_44_P367971	RGD1565319_predicted	XM_219510		-0.18	-1.502	0.01306
A_44_P698487	ENSRNOT00000046457	ENSRNOT00000046457		-0.18	-1.502	0.004168
A_44_P978398	TC540103	TC540103		-0.18	-1.502	0.035515
A_44_P824294	TC544469	TC544469		-0.18	-1.502	0.085192
A_44_P295038	LOC286984	NM_173318	286984	-0.18	-1.502	0.056824
A_44_P783412	BF393107	BF393107		-0.18	-1.502	0.006082
A_44_P200341	BE117692	BE117692		-0.18	-1.502	0.017723
A_44_P503421	Tas1r2	XM_001074791	29408	-0.18	-1.502	0.02076

A_44_P138355	RGD1561232_predicted	XM_001070211		-0.18	-1.502	0.082269
A_44_P557344	Prkci	XM_342223	84006	-0.18	-1.502	0.015916
A_44_P235643	AI229638	AI229638		-0.18	-1.502	0.007224
A_42_P625419	RGD1565927_predicted	XM_221535	304020	-0.18	-1.502	0.007938
A_44_P248038	Atxn3	NM_021702	60331	-0.18	-1.503	0.005422
A_44_P724632	AI176008	AI176008		-0.18	-1.503	0.045315
A_43_P13253	UST4r	NM_134379	171397	-0.18	-1.503	0.239273
A_44_P134966	LOC316632	NM_182671	314071	-0.18	-1.503	0.005842
A_43_P18782	March8_predicted	XM_232293		-0.18	-1.503	0.009259
A_44_P355987	Olr1606	NM_001000502	305711	-0.18	-1.503	0.102124
A_43_P21037	Snta1	XM_342548	362242	-0.18	-1.503	0.016868
A_44_P334241	AI410703	AI410703	363061	-0.18	-1.503	0.018424
A_44_P327380	AA957498	AA957498		-0.18	-1.503	0.023356
A_43_P17807	LOC361914	NM_001017465	361914	-0.18	-1.503	0.092776
A_44_P262994	Myo5c_predicted	XM_236411		-0.18	-1.504	0.039321
A_44_P607486	Ches1_predicted	XM_234377		-0.18	-1.504	0.010216
A_44_P473314	RGD1309522	NM_001013997	306102	-0.18	-1.504	0.119951
A_42_P583245	RGD1561141_predicted	XM_230015	311122	-0.18	-1.504	0.048978
A_42_P809733	RGD1309410_predicted	XM_347094	363020	-0.18	-1.504	0.20309
A_42_P765066	Mb	NM_021588	59108	-0.18	-1.504	0.055
A_44_P222521	RGD1305557	XM_224729	306349	-0.18	-1.504	0.004336
A_44_P890247	BE098727	BE098727		-0.18	-1.504	0.045046
A_44_P176416	RGD1564300_predicted	XM_001053680		-0.18	-1.505	0.036365
A_44_P107204	Olr791_predicted	NM_001000580	366152	-0.18	-1.505	0.007355
A_44_P422602	AI232943	AI232943		-0.18	-1.505	0.038867
A_44_P114866	RGD1560678_predicted	XM_237287		-0.18	-1.505	0.0691
A_44_P686854	RGD1563224_predicted	XM_001068872		-0.18	-1.505	0.003624
A_44_P350074	LOC686892	XM_001075009		-0.18	-1.505	0.015277
A_44_P992673	RGD1309578	NM_001014162	361637	-0.18	-1.505	0.010633
A_44_P391986	Mlx	NM_001034112	360631	-0.18	-1.505	0.004568
A_42_P620006	Wfdc1	NM_133581	171112	-0.18	-1.505	0.166411
A_44_P182752	Fcho2_predicted	XM_219503	309129	-0.18	-1.506	0.030564
A_43_P10824	Cib2	NM_001015010	300719	-0.18	-1.506	0.048253
A_44_P723176	A_44_P723176	A_44_P723176		-0.18	-1.506	0.12869
A_44_P100272	Dnttip1	NM_134400	171437	-0.18	-1.506	0.002554
A_44_P537118	Mylk_predicted	XM_213611		-0.18	-1.506	0.009211
A_44_P361169	RGD1305072	XM_214589	291709	-0.18	-1.506	0.001448
A_44_P996482	Chmp5	NM_001025410	297995	-0.18	-1.506	0.011684
A_43_P11188	XM_215889	XM_215889		-0.18	-1.506	0.009347
A_44_P243670	ENSRNOT00000043690	ENSRNOT00000043690		-0.18	-1.506	0.013315
A_44_P816426	LOC498350	NM_001017498	498350	-0.18	-1.506	0.03711
A_44_P451026	Adra1b	NM_016991	24173	-0.18	-1.506	0.006524
A_44_P447999	ENSRNOT00000023661	ENSRNOT00000023661		-0.18	-1.506	0.008746
A_44_P467175	BE112236	BE112236	499689	-0.18	-1.506	0.025195
A_44_P221077	MGC108778	NM_001017479	497979	-0.18	-1.506	0.103484
A_44_P137942	A_44_P137942	A_44_P137942		-0.18	-1.506	0.034458
A_43_P12334	Ptprq	NM_022925	360417	-0.18	-1.506	0.047049
A_44_P548831	Slc23a3_predicted	XM_346061		-0.18	-1.507	0.072882
A_44_P168929	RGD1306656_predicted	XM_346892		-0.18	-1.507	0.12285
A_44_P281435	LOC296318	NM_001013923	296318	-0.18	-1.507	0.016783
A_44_P231876	RGD1310224	NM_001009275	291076	-0.18	-1.507	0.034959
A_44_P377835	Wnt1_mapped	XM_235639		-0.18	-1.507	0.032099
A_44_P1058570	Sigirr	NM_001024887	309106	-0.18	-1.507	0.00227
A_43_P18179	RGD1307160_predicted	XM_219953	309451	-0.18	-1.507	0.005049
A_44_P159080	XM_341332	XM_341332		-0.18	-1.507	0.021987
A_42_P803406	Nags_predicted	XM_220939		-0.18	-1.507	0.038453
A_43_P21282	RGD1560932_predicted	XM_001066988		-0.18	-1.507	0.05682
A_43_P18043	Tob2	NM_001007146	315159	-0.18	-1.507	0.005284
A_42_P809953	Vil2	NM_019357	54319	-0.18	-1.507	0.003909
A_44_P433003	AI180191	AI180191	58817	-0.18	-1.507	0.047362
A_44_P520108	Arhgef7	NM_053740	114559	-0.18	-1.507	0.03042
A_44_P378675	AI179100	AI179100	299207	-0.18	-1.508	0.054173
A_44_P461976	RGD1565687_predicted	XM_237402	301596	-0.18	-1.508	0.009742

A_44_P316172	Znf187	XM_577579	266792	-0.18	-1.508	0.022212
A_43_P18333	RGD1564117_predicted	XM_228547	317253	-0.18	-1.508	0.156511
A_44_P236051	Olr1222_predicted	NM_001000440	300548	-0.18	-1.508	0.025657
A_42_P641870	Tmem55a	NM_001024900	362490	-0.18	-1.508	0.10836
A_44_P745585	LOC499749	NM_001024309	499749	-0.18	-1.508	0.007905
A_44_P778980	TC532622	TC532622		-0.18	-1.509	0.065102
A_44_P222887	LOC686091	XM_001066510	686091	-0.18	-1.509	0.003876
A_42_P750340	RGD1563319_predicted	XM_215129	293632	-0.18	-1.509	0.059374
A_42_P507027	Tm6p1	NM_139107	245966	-0.18	-1.509	0.04294
A_44_P1000267	RGD1564625_predicted	XM_220013	309475	-0.18	-1.509	0.017915
A_43_P16022	Acvr1b	NM_199230	29381	-0.18	-1.509	0.008502
A_44_P430478	AI102926	AI102926		-0.18	-1.510	0.034853
A_44_P767294	TC544813	TC544813		-0.18	-1.510	0.003748
A_44_P273859	Il9r	NM_017021	24500	-0.18	-1.510	0.069887
A_43_P12088	Syt7	NM_021659	59267	-0.18	-1.510	0.075714
A_43_P14372	LOC684258	XM_001069610		-0.18	-1.510	0.004296
A_44_P1071170	M16349	M16349	24210	-0.18	-1.511	0.051545
A_44_P306852	XM_224115	XM_224115		-0.18	-1.511	0.11205
A_44_P780848	Chst9_predicted	XM_226142	291770	-0.18	-1.511	0.168507
A_44_P231976	RGD1559899_predicted	XM_226473		-0.18	-1.511	0.053135
A_43_P10085	TC537861	TC537861		-0.18	-1.511	0.012591
A_44_P1048727	Mppe1_predicted	XM_341627		-0.18	-1.511	0.10094
A_44_P179296	BF287585	BF287585		-0.18	-1.511	0.100858
A_43_P17469	BF559670	BF559670	298555	-0.18	-1.511	0.097608
A_43_P15751	Grm2	XM_343470		-0.18	-1.511	0.009618
A_44_P208640	RGD1559810_predicted	XM_345107		-0.18	-1.511	0.22326
A_44_P537410	Il3ra	NM_139260	246144	-0.18	-1.512	0.02626
A_44_P419406	A_44_P419406	A_44_P419406		-0.18	-1.512	0.010186
A_44_P963558	TC544207	TC544207		-0.18	-1.512	0.019723
A_44_P258158	A_44_P258158	A_44_P258158		-0.18	-1.512	0.081187
A_43_P22326	RGD1310623	NM_001034011	360868	-0.18	-1.512	0.083346
A_43_P19169	Dcbld1_predicted	XM_228172	309773	-0.18	-1.512	0.009768
A_44_P290436	AF189020	AF189020	81664	-0.18	-1.512	0.007674
A_44_P121330	Zfp37	NM_058209	115768	-0.18	-1.513	0.116796
A_44_P1060244	LOC690423	XM_001059707		-0.18	-1.513	0.005972
A_43_P12888	Pafah1b3	NM_053654	114113	-0.18	-1.513	0.011588
A_44_P433021	AI179984	AI179984	287526	-0.18	-1.513	0.029613
A_44_P637152	LOC680342	XM_001056750		-0.18	-1.513	0.036463
A_44_P142610	RGD1310800_predicted	XM_342577		-0.18	-1.513	0.001885
A_44_P414139	BE117892	BE117892		-0.18	-1.513	0.071467
A_44_P292951	A_44_P292951	A_44_P292951		-0.18	-1.513	0.138892
A_44_P227590	RGD1561262_predicted	XM_343911	363618	-0.18	-1.514	0.063506
A_44_P597956	A_44_P597956	A_44_P597956		-0.18	-1.514	0.019556
A_44_P932828	TC521066	TC521066		-0.18	-1.514	0.065271
A_44_P478607	AA925339	AA925339		-0.18	-1.514	0.06167
A_44_P210663	XM_341899	XM_341899		-0.18	-1.514	0.034167
A_44_P557253	Olr689_predicted	NM_001000997	405357	-0.18	-1.514	0.179086
A_44_P189452	U06751	U06751		-0.18	-1.515	0.141821
A_44_P700436	RGD1563580_predicted	XM_001070331		-0.18	-1.515	0.033361
A_42_P532072	Wfikkn1_predicted	XM_343870	363555	-0.18	-1.515	0.010782
A_43_P17059	Crlf1_predicted	XM_214312		-0.18	-1.515	0.004846
A_44_P100310	Z93350	Z93350	299357	-0.18	-1.515	0.038105
A_44_P188015	AA818078	AA818078	309172	-0.18	-1.515	0.063846
A_43_P18400	Pik3c2b_predicted	XM_213879		-0.18	-1.515	0.006869
A_44_P297645	Zswim3_predicted	XM_230845		-0.18	-1.516	0.030322
A_44_P656109	TC541191	TC541191		-0.18	-1.516	0.067618
A_44_P915922	TC539429	TC539429		-0.18	-1.516	0.042851
A_44_P311797	Cpxm1_predicted	XM_215840		-0.18	-1.516	0.344857
A_44_P220283	XM_230157	XM_230157		-0.18	-1.517	0.026279
A_44_P155775	Vamp4_predicted	XM_344168		-0.18	-1.517	0.03799
A_44_P398257	TC547634	TC547634		-0.18	-1.517	0.074266
A_44_P194301	Ubl3	NM_001015030	363869	-0.18	-1.517	0.013693
A_44_P491583	AF531422	AF531422		-0.18	-1.517	0.013567

A_44_P134077	BE121346	BE121346	691335	-0.18	-1.518	0.065504
A_44_P1055026	Rfxank	NM_001013136	306353	-0.18	-1.518	0.014226
A_44_P432525	AI070027	AI070027	83505	-0.18	-1.518	0.07616
A_43_P16372	Olr737	NM_001000616	404808	-0.18	-1.518	0.031497
A_44_P838130	TC556025	TC556025		-0.18	-1.518	0.157276
A_44_P621605	ENSRNOT00000032764	ENSRNOT00000032764		-0.18	-1.518	0.018564
A_44_P487218	Golgb1	NM_138885	192243	-0.18	-1.519	0.002536
A_44_P114004	Olr867_predicted	NM_001000412	298476	-0.18	-1.519	0.208963
A_44_P364850	AW915323	AW915323		-0.18	-1.519	0.002602
A_44_P500328	AI029729	AI029729	309312	-0.18	-1.519	0.010278
A_44_P1060096	Cideb_predicted	XM_344410		-0.18	-1.519	0.007816
A_44_P114282	RGD1559588_predicted	XM_221990		-0.18	-1.519	0.026484
A_44_P548903	BM392020	BM392020	289276	-0.18	-1.519	0.043383
A_44_P499667	BF289611	BF289611		-0.18	-1.519	0.008808
A_44_P834226	CK840287	CK840287	366909	-0.18	-1.519	0.06182
A_44_P138413	AA819769	AA819769		-0.18	-1.520	0.012229
A_44_P243426	Peli1	XM_001060915		-0.18	-1.520	0.006907
A_44_P297633	Irx3_predicted	XM_226322		-0.18	-1.520	0.040177
A_43_P20422	Cdc14a_predicted	XM_227618		-0.18	-1.520	0.015764
A_44_P309523	LOC501230	XM_001076269		-0.18	-1.520	0.012857
A_44_P309455	RGD1311257	NM_001008307	294333	-0.18	-1.520	0.071297
A_44_P518404	XM_233724	XM_233724		-0.18	-1.520	0.096009
A_44_P466989	CB545351	CB545351	287962	-0.18	-1.520	0.012044
A_44_P868963	Tnfrsf14	NM_001015034	366518	-0.18	-1.521	0.005175
A_44_P1048315	Tcta	NM_001014005	306587	-0.18	-1.521	0.003787
A_44_P497275	Zzef1_predicted	XM_237787	287476	-0.18	-1.521	0.005611
A_44_P134711	AI175566	AI175566	83462	-0.18	-1.521	0.11653
A_44_P139494	CN543488	CN543488	688966	-0.18	-1.521	0.026772
A_44_P235009	AI715793	AI715793		-0.18	-1.521	0.045829
A_44_P177994	Rhobtb3_predicted	XM_226604		-0.18	-1.521	0.017143
A_42_P620907	Ndufb3_predicted	XM_217400		-0.18	-1.521	0.014804
A_44_P1055271	LOC361041	NM_001014138		-0.18	-1.521	0.055228
A_44_P633489	DV723525	DV723525		-0.18	-1.521	0.043794
A_44_P727127	AI233885	AI233885	300802	-0.18	-1.521	0.057784
A_44_P400211	Sgpl1	NM_173116	286896	-0.18	-1.521	0.00721
A_44_P102197	Alox5ap	NM_017260	29624	-0.18	-1.521	0.02918
A_44_P209280	BM386775	BM386775	298543	-0.18	-1.521	0.047428
A_44_P676897	A_44_P676897	A_44_P676897		-0.18	-1.521	0.04564
A_44_P689110	TC533761	TC533761		-0.18	-1.522	0.003903
A_44_P117431	RGD1565373_predicted	XM_231622		-0.18	-1.522	0.18763
A_44_P243303	Zfp68_predicted	XM_221977		-0.18	-1.522	0.011841
A_44_P251677	BI289075	BI289075	362402	-0.18	-1.522	0.06764
A_44_P237642	BF413313	BF413313		-0.18	-1.523	0.032435
A_44_P1008888	Mt3	NM_053968	117038	-0.18	-1.523	0.040581
A_44_P767783	A_44_P767783	A_44_P767783		-0.18	-1.523	0.053174
A_43_P15048	AW914834	AW914834	362843	-0.18	-1.523	0.027014
A_44_P122422	AI706196	AI706196	292777	-0.18	-1.523	0.03618
A_44_P515170	Nadsyn1	NM_181480	353255	-0.18	-1.523	0.008632
A_44_P402425	Bwk1	NM_198743	296466	-0.18	-1.524	0.003171
A_44_P432747	AA957610	AA957610		-0.18	-1.524	0.062975
A_44_P1001762	BI275770	BI275770		-0.18	-1.524	0.225644
A_44_P1070483	AW920918	AW920918	310625	-0.18	-1.524	0.019993
A_44_P925145	AW921160	AW921160		-0.18	-1.524	0.075114
A_44_P428197	Uqcrb_predicted	XM_343225	362897	-0.18	-1.524	0.001946
A_44_P419991	BF389753	BF389753		-0.18	-1.524	0.045748
A_44_P129330	Rhpn2_predicted	XM_001079717		-0.18	-1.525	0.030683
A_44_P529660	A_44_P529660	A_44_P529660		-0.18	-1.525	0.021009
A_44_P325605	G7c	NM_212499	309611	-0.18	-1.525	0.015575
A_44_P466159	Centa1	NM_133567	171097	-0.18	-1.525	0.005021
A_44_P166113	RGD1565017_predicted	XM_224631		-0.18	-1.525	0.07305
A_44_P496341	Olr1543_predicted	NM_001001104	406000	-0.18	-1.525	0.331446
A_44_P105249	A_44_P105249	A_44_P105249		-0.18	-1.525	0.322776
A_44_P898543	LOC683749	XM_001067319		-0.18	-1.525	0.009369

A_44_P407695	XM_344358	XM_344358		-0.18	-1.525	0.158773
A_44_P301074	Tmem23	NM_181386	353229	-0.18	-1.525	0.01108
A_44_P609144	TC531140	TC531140		-0.18	-1.525	0.093649
A_42_P470966	RGD1560397_predicted	XM_001081205		-0.18	-1.525	0.004746
A_44_P450386	XM_233850	XM_233850		-0.18	-1.526	0.047959
A_44_P217063	LOC500282	NM_001024332	500282	-0.18	-1.526	0.007086
A_44_P213198	Pak7_predicted	XM_230625		-0.18	-1.526	0.056735
A_44_P433713	Proz_predicted	XM_225018		-0.18	-1.526	0.067512
A_43_P12034	Syt5	NM_019350	54309	-0.18	-1.526	0.040569
A_43_P21706	LOC684630	XM_001071321		-0.18	-1.526	0.011055
A_44_P323232	Olr1641_predicted	NM_001000100	290050	-0.18	-1.526	0.06763
A_43_P10631	AW142959	AW142959		-0.18	-1.526	0.03862
A_44_P389261	Klhl26_predicted	XM_224731		-0.18	-1.526	0.003587
A_44_P357436	CF109359	CF109359		-0.18	-1.526	0.017137
A_44_P198644	Olr619_predicted	NM_001000338	295843	-0.18	-1.526	0.063027
A_43_P13426	Ceacam10	NM_173339	287009	-0.18	-1.527	0.372362
A_44_P423899	AF053092	AF053092		-0.18	-1.527	0.055494
A_44_P235926	Gng7	NM_024138	58979	-0.18	-1.527	0.134048
A_44_P479791	AI071381	AI071381	291962	-0.18	-1.527	0.033662
A_44_P460160	Cacng5	NM_080693	140726	-0.18	-1.527	0.068687
A_44_P359777	AW918701	AW918701	117099	-0.18	-1.528	0.127239
A_44_P243520	RGD1308290_predicted	XM_341121		-0.18	-1.528	0.003375
A_44_P124095	LOC681862	XM_001058737		-0.18	-1.528	0.032972
A_44_P227206	XM_229843	XM_229843		-0.18	-1.528	0.014302
A_43_P21800	JIK	NM_001024254	304530	-0.18	-1.528	0.006983
A_44_P905187	Dnajc6_predicted	XM_233231		-0.18	-1.528	0.084829
A_42_P545536	AA964544	AA964544		-0.18	-1.528	0.012836
A_44_P484994	XM_218103	XM_218103		-0.18	-1.528	0.009441
A_42_P718331	Sstr3	NM_133522	171044	-0.18	-1.529	0.071335
A_42_P696406	XM_213226	XM_213226		-0.18	-1.529	0.058126
A_43_P15665	Bckdha	NM_012782	25244	-0.18	-1.529	0.01729
A_43_P16519	Mpp2	XM_340912	85275	-0.18	-1.529	0.053917
A_44_P704483	LOC303140	DQ119106	24915	-0.18	-1.529	0.054933
A_44_P634032	AI179709	AI179709	305149	-0.18	-1.529	0.070883
A_44_P379805	Ankrd42_predicted	XM_215000	293117	-0.18	-1.529	0.007797
A_43_P18653	Dock5_predicted	XM_224311		-0.18	-1.529	0.026729
A_44_P1050554	RGD1565545_predicted	XM_218197	308337	-0.18	-1.530	0.004768
A_44_P481380	Pax4	NM_031799	83630	-0.18	-1.530	0.067013
A_42_P563553	RGD1562272_predicted	XM_001081219		-0.18	-1.530	0.031614
A_44_P770931	CR468303	CR468303		-0.18	-1.530	0.02459
A_44_P419210	LOC290098	NM_001039205		-0.18	-1.530	0.010667
A_44_P745648	RGD1309660_predicted	XM_226652	309953	-0.18	-1.530	0.075625
A_44_P433888	A_44_P433888	A_44_P433888		-0.18	-1.530	0.107893
A_44_P288185	Itpr3	NM_013138	25679	-0.18	-1.530	0.039583
A_44_P164132	RGD1309576_predicted	XM_213341	287405	-0.18	-1.530	0.015665
A_43_P15881	Olr1482_predicted	NM_001000026	287484	-0.18	-1.530	0.063931
A_44_P519871	Srd5a1	NM_017070	24950	-0.18	-1.530	0.117073
A_44_P869736	TC558604	TC558604		-0.18	-1.531	0.004612
A_44_P377645	XM_342774	XM_342774		-0.18	-1.531	0.005854
A_44_P635379	BF403180	BF403180		-0.18	-1.531	0.29062
A_44_P471217	A_44_P471217	A_44_P471217		-0.18	-1.531	0.013406
A_44_P529418	Akap9	XM_001063778		-0.18	-1.531	0.018804
A_44_P183160	RGD1305450_predicted	XM_345881		-0.18	-1.531	0.028103
A_44_P288691	A_44_P288691	A_44_P288691		-0.19	-1.531	0.016198
A_44_P312774	AA955780	AA955780	64665	-0.19	-1.531	0.009714
A_44_P531647	Plcg2	NM_017168	29337	-0.19	-1.531	0.017487
A_44_P501593	Acss2_predicted	XM_230773		-0.19	-1.531	0.013082
A_43_P15633	Pkn2	XM_215718		-0.19	-1.531	0.013853
A_44_P501868	XM_235557	XM_235557		-0.19	-1.532	0.020202
A_44_P838681	LOC365344	XR_006886	365344	-0.19	-1.532	0.024885
A_44_P210268	Olr1108_predicted	NM_001000993	405353	-0.19	-1.532	0.082818
A_44_P252657	Pou6f1	XM_343334		-0.19	-1.532	0.023337
A_44_P418585	AW915208	AW915208		-0.19	-1.532	0.01712

A_43_P13801	BQ783112	BQ783112		-0.19	-1.532	0.024968
A_43_P13696	Ptpn3	XM_001055793		-0.19	-1.532	0.030127
A_44_P856610	TC545251	TC545251		-0.19	-1.532	0.008808
A_44_P289284	Shank1	NM_031751	78957	-0.19	-1.532	0.145559
A_44_P191632	Odz2	NM_020088	117242	-0.19	-1.532	0.080964
A_44_P444569	BE113640	BE113640	24779	-0.19	-1.532	0.020324
A_42_P695770	RGD1565407_predicted	XM_214288		-0.19	-1.533	0.00312
A_44_P289125	Spsb4_predicted	XM_001069568		-0.19	-1.533	0.180866
A_44_P265544	Crybb3	NM_031690	64349	-0.19	-1.533	0.053302
A_44_P539256	Larp6_predicted	XM_236338		-0.19	-1.533	0.048129
A_44_P700166	TC557848	TC557848		-0.19	-1.533	0.140449
A_44_P918103	TC539107	TC539107		-0.19	-1.533	0.05328
A_44_P459288	RGD1561523_predicted	XM_223172		-0.19	-1.533	0.015796
A_44_P548864	Otop3_predicted	XM_221108		-0.19	-1.533	0.03731
A_44_P121703	Aire_predicted	XM_228067		-0.19	-1.533	0.100024
A_44_P491374	BF553979	BF553979	360811	-0.19	-1.534	0.089763
A_44_P222367	RGD1310656_predicted	XM_222183		-0.19	-1.534	0.010604
A_43_P21269	CB546606	CB546606	287876	-0.19	-1.534	0.028038
A_44_P312373	AA955577	AA955577	25236	-0.19	-1.534	0.059173
A_44_P311275	Olr1375_predicted	NM_214829	287088	-0.19	-1.534	0.217904
A_44_P416005	AI501179	AI501179	25151	-0.19	-1.534	0.026543
A_44_P850979	BF549558	BF549558	314171	-0.19	-1.534	0.058465
A_44_P714007	LOC500292	NM_001024333	500292	-0.19	-1.534	0.029989
A_44_P408887	Trak2	NM_133560	171086	-0.19	-1.534	0.009411
A_44_P714524	CX570178	CX570178		-0.19	-1.535	0.009437
A_44_P433992	RGD1565917_predicted	XM_578572		-0.19	-1.535	0.014356
A_43_P11525	Svs2	NM_012710	25013	-0.19	-1.535	0.051467
A_44_P558992	AI137488	AI137488	361940	-0.19	-1.535	0.00868
A_44_P508643	Oit1_predicted	XM_214143		-0.19	-1.535	0.039568
A_44_P786554	AI103367	AI103367	362484	-0.19	-1.535	0.043895
A_44_P515521	Sfrs5	NM_019257	29667	-0.19	-1.535	0.003668
A_44_P429006	Gdf1_predicted	XM_224733		-0.19	-1.535	0.029349
A_44_P134094	AW529441	AW529441	686841	-0.19	-1.535	0.020748
A_44_P376454	AA858545	AA858545	65164	-0.19	-1.535	0.061907
A_44_P194083	Sec61g	AY318964	367253	-0.19	-1.536	0.005833
A_43_P11301	TC535339	TC535339		-0.19	-1.536	0.006946
A_44_P240828	Giyd2	NM_001009292	293489	-0.19	-1.536	0.018063
A_43_P21903	Atp6v1b1_predicted	XM_232119		-0.19	-1.536	0.065463
A_44_P119933	BI281874	BI281874	311114	-0.19	-1.536	0.06279
A_44_P180350	RGD1308113	NM_001008296	290224	-0.19	-1.536	0.007477
A_44_P710478	AI145372	AI145372		-0.19	-1.537	0.276531
A_44_P371016	BQ199937	BQ199937	289753	-0.19	-1.537	0.03688
A_43_P15117	BF556558	BF556558		-0.19	-1.537	0.067137
A_44_P527763	XM_345610	XM_345610		-0.19	-1.537	0.017616
A_42_P562635	Tmem115_predicted	XM_343473		-0.19	-1.537	0.174162
A_44_P1011822	AW915804	AW915804		-0.19	-1.537	0.008118
A_44_P210868	Sptbn5_predicted	XM_238278	296090	-0.19	-1.537	0.02798
A_44_P241479	Cbara1	NM_199412	365567	-0.19	-1.537	0.01629
A_44_P182881	Rnf36	NM_001013160	311373	-0.19	-1.537	0.00447
A_44_P556427	Pgc	NM_133284	24864	-0.19	-1.538	0.048336
A_42_P466321	Matk	NM_021859	60450	-0.19	-1.538	0.054685
A_44_P471505	AA893724	AA893724	290533	-0.19	-1.538	0.077105
A_44_P484607	Gipr	NM_012714	25024	-0.19	-1.538	0.145751
A_44_P116634	Prkar2b	XM_343046		-0.19	-1.538	0.031754
A_44_P926241	AA924847	AA924847	298298	-0.19	-1.538	0.006556
A_44_P247630	BF388415	BF388415		-0.19	-1.538	0.035249
A_44_P349831	RGD1311234_predicted	XM_343945		-0.19	-1.538	0.025872
A_44_P455715	AABR03063000	AABR03063000		-0.19	-1.538	0.070345
A_44_P142391	LOC360919	XM_341193		-0.19	-1.538	0.012335
A_44_P552958	Kctd8_predicted	XM_344237		-0.19	-1.538	0.011524
A_44_P136845	Spag9_predicted	XM_340879		-0.19	-1.539	0.002056
A_42_P635881	Slc32a1	NM_031782	83612	-0.19	-1.539	0.088756
A_44_P151675	Gfra3	NM_053398	84422	-0.19	-1.539	0.008979

A_44_P123125	AI170850	AI170850	246120	-0.19	-1.539	0.037844
A_44_P119264	Cyp2u1	NM_001024779	310848	-0.19	-1.539	0.004198
A_43_P13346	Prokr2	NM_138978	192649	-0.19	-1.539	0.016301
A_44_P285930	Znf541_predicted	XM_217807		-0.19	-1.539	0.014212
A_44_P526676	Tyrobp	AI102519	361537	-0.19	-1.539	0.017698
A_44_P702192	TC549309	TC549309		-0.19	-1.540	0.072564
A_43_P19626	Sncaip_predicted	XM_225768		-0.19	-1.540	0.084491
A_44_P442705	Olr1240_predicted	NM_001000448	300565	-0.19	-1.540	0.091861
A_44_P309097	Sctr	NM_031115	81779	-0.19	-1.540	0.148297
A_44_P1043979	Ndufs5b	NM_001030052	362588	-0.19	-1.541	0.006386
A_44_P107499	RGD1565538_predicted	XM_222038		-0.19	-1.541	0.191989
A_44_P128359	RGD1563721_predicted	XM_219703	309252	-0.19	-1.541	0.05278
A_44_P211005	A_44_P211005	A_44_P211005		-0.19	-1.542	0.041601
A_44_P522827	Htra3_predicted	XM_341237	360959	-0.19	-1.542	0.053335
A_44_P374988	Calm1	NM_031969	24242	-0.19	-1.542	0.015898
A_44_P266033	BM391824	BM391824	361309	-0.19	-1.542	0.024583
A_43_P10865	Ube2e2	XM_341288		-0.19	-1.542	0.02212
A_44_P136055	CB544791	CB544791		-0.19	-1.542	0.045099
A_44_P205528	Iiig9	NM_145786	252958	-0.19	-1.542	0.059844
A_44_P290679	XM_214085	XM_214085		-0.19	-1.542	0.005146
A_44_P448864	Apc	NM_012499	24205	-0.19	-1.542	0.019705
A_44_P534310	XM_224103	XM_224103		-0.19	-1.543	0.034004
A_44_P359426	Jwa	NM_022682	64623	-0.19	-1.543	0.047232
A_44_P668980	TC540138	TC540138		-0.19	-1.543	0.096177
A_44_P448693	Cryzl1	NM_001013044	288256	-0.19	-1.543	0.013287
A_44_P129848	AW525988	AW525988	300062	-0.19	-1.543	0.022272
A_43_P12022	Tpsab1	NM_019322	54271	-0.19	-1.543	0.073331
A_44_P579214	TC535018	TC535018		-0.19	-1.543	0.208207
A_44_P543855	Diap2_predicted	XM_228441		-0.19	-1.544	0.03301
A_44_P1048543	Ghrh	NM_031577	29446	-0.19	-1.544	0.01464
A_44_P358358	H2-T18	NM_001002821	406194	-0.19	-1.544	0.010988
A_42_P688604	Ces1	NM_031565	29225	-0.19	-1.544	0.054933
A_44_P205691	Olr477_predicted	NM_001000304	295747	-0.19	-1.545	0.076154
A_44_P778283	TC526024	TC526024		-0.19	-1.545	0.013454
A_44_P238067	Snurf	NM_130738	81781	-0.19	-1.545	0.067512
A_43_P11809	Umod	NM_017082	25128	-0.19	-1.545	0.101242
A_44_P803922	BF545749	BF545749		-0.19	-1.545	0.007388
A_44_P144001	Pam	NM_013000	25508	-0.19	-1.545	0.021302
A_42_P760354	Pla2g2c	NM_019202	29387	-0.19	-1.545	0.012452
A_44_P711197	AI136300	AI136300		-0.19	-1.546	0.019703
A_44_P411743	Gpx2	BQ196649	29326	-0.19	-1.546	0.052513
A_43_P21332	LOC366889	NM_001039340	366889	-0.19	-1.546	0.027605
A_44_P989250	CV120833	CV120833		-0.19	-1.546	0.012264
A_44_P511416	Naga	NM_001012120	315165	-0.19	-1.547	0.007966
A_44_P112852	LOC363091	NM_001014211	363091	-0.19	-1.547	0.0176
A_44_P373188	XM_343852	XM_343852		-0.19	-1.547	0.015212
A_44_P286551	AA956791	AA956791	362339	-0.19	-1.547	0.177416
A_44_P465619	AA875532	AA875532	361695	-0.19	-1.547	0.040503
A_43_P11603	Sstr5	NM_012882	25354	-0.19	-1.547	0.019236
A_43_P20190	RGD1311340_predicted	XM_219672		-0.19	-1.547	0.057404
A_44_P227277	LOC683645	XM_001066873		-0.19	-1.547	0.028575
A_44_P313926	RGD1304688_predicted	XM_223730		-0.19	-1.548	0.030051
A_44_P509329	BF547360	BF547360		-0.19	-1.548	0.007295
A_44_P425660	AI233162	AI233162	361443	-0.19	-1.548	0.013883
A_44_P121325	Rab22a_predicted	XM_345479		-0.19	-1.548	0.005413
A_44_P492041	Snx26_predicted	XM_001079120		-0.19	-1.548	0.004941
A_44_P552915	Cpt1c	XM_001075728	308579	-0.19	-1.548	0.008365
A_44_P727067	AW143701	AW143701		-0.19	-1.548	0.004356
A_43_P16139	Ralgps2	XM_222773		-0.19	-1.549	0.027703
A_44_P536951	XM_345649	XM_345649		-0.19	-1.549	0.060746
A_43_P17400	Pmm2_predicted	XM_220141		-0.19	-1.549	0.007249
A_44_P269635	XM_344389	XM_344389		-0.19	-1.549	0.025686
A_42_P680338	Fut1	NM_031236	81919	-0.19	-1.549	0.015742

A_44_P1008245	Casc1_predicted	XM_216310		-0.19	-1.549	0.023495
A_44_P261726	BQ780678	BQ780678	294350	-0.19	-1.550	0.12612
A_44_P212676	Crat	NM_001004085	311849	-0.19	-1.550	0.011915
A_43_P19012	RGD1310143	NM_001010951	298771	-0.19	-1.550	0.003854
A_44_P188729	AF115770	AF115770		-0.19	-1.550	0.011025
A_44_P1028460	Syap1	NM_001004253	302678	-0.19	-1.550	0.015036
A_44_P133464	XM_347133	XM_347133		-0.19	-1.550	0.030361
A_43_P11993	Ptprf	NM_019249	360406	-0.19	-1.550	0.005958
A_44_P509917	Isl1	NM_017339	64444	-0.19	-1.551	0.044269
A_44_P870065	TC560508	TC560508		-0.19	-1.551	0.029081
A_44_P368134	Sncaip_predicted	XM_225768		-0.19	-1.551	0.036118
A_44_P219585	Kras	NM_031515	24525	-0.19	-1.551	0.005477
A_44_P129029	Hoxa7_mapped	XM_575478		-0.19	-1.551	0.014875
A_44_P486793	BE108409	BE108409		-0.19	-1.551	0.056524
A_44_P359257	AA926091	AA926091		-0.19	-1.551	0.125047
A_44_P505500	LOC683288	XM_001065258		-0.19	-1.551	0.004596
A_44_P198957	RGD1565197_predicted	XM_218653	308587	-0.19	-1.551	0.008866
A_44_P952377	A_44_P952377	A_44_P952377		-0.19	-1.551	0.036401
A_44_P349050	Minpp1	XM_342044		-0.19	-1.551	0.0158
A_44_P483728	Rps6ka1	NM_031107	81771	-0.19	-1.551	0.007957
A_44_P217723	Art1_predicted	XM_218975		-0.19	-1.551	0.00532
A_44_P461262	Olr424_predicted	NM_001000392	296686	-0.19	-1.552	0.161629
A_44_P1017206	RGD1307753_predicted	XM_213845	288907	-0.19	-1.552	0.060987
A_43_P17969	Nrcam	AY574273	497815	-0.19	-1.552	0.029286
A_44_P454426	Akap9	XM_001063778		-0.19	-1.552	0.002333
A_44_P385824	AI231472	AI231472	29393	-0.19	-1.553	0.043699
A_44_P291952	Scarf1_predicted	XM_001080505		-0.19	-1.553	0.031143
A_43_P11516	Vipr1	NM_012685	24875	-0.19	-1.553	0.00363
A_44_P105205	XM_344305	XM_344305		-0.19	-1.553	0.114846
A_44_P298751	AA892545	AA892545	309131	-0.19	-1.553	0.015832
A_44_P293460	AI103155	AI103155		-0.19	-1.553	0.032289
A_44_P553552	AABR03000487	AABR03000487		-0.19	-1.553	0.053371
A_44_P502252	Hoxa9_predicted	XM_001057018		-0.19	-1.553	0.016253
A_44_P284804	AA964050	AA964050	114089	-0.19	-1.554	0.009841
A_44_P133728	AA818176	AA818176		-0.19	-1.554	0.004387
A_44_P194572	ENSRNOT00000036227	ENSRNOT00000036227		-0.19	-1.554	0.033756
A_44_P1018289	BF551187	BF551187		-0.19	-1.554	0.003429
A_42_P522857	Mgst3_predicted	XM_213943	289197	-0.19	-1.554	0.017046
A_44_P301902	AB097857	AB097857		-0.19	-1.554	0.033001
A_44_P900688	TC559564	TC559564		-0.19	-1.554	0.10956
A_44_P326880	AI059663	AI059663		-0.19	-1.554	0.026376
A_44_P973931	BF412064	BF412064		-0.19	-1.555	0.028909
A_44_P366067	A_44_P366067	A_44_P366067		-0.19	-1.555	0.099931
A_44_P817108	BE119393	BE119393		-0.19	-1.555	0.007691
A_44_P1045526	Piga_mapped	XM_343783		-0.19	-1.555	0.009551
A_44_P291488	CB548117	CB548117	309187	-0.19	-1.555	0.042099
A_44_P321419	XM_228597	XM_228597		-0.19	-1.555	0.144365
A_43_P17983	Cul1_predicted	XM_342679		-0.19	-1.555	0.010182
A_44_P126498	Klhdc1_predicted	XM_234272		-0.19	-1.555	0.003037
A_44_P295275	LOC291761	XR_007320	291761	-0.19	-1.555	0.03911
A_44_P369322	AI030250	AI030250	293754	-0.19	-1.556	0.115026
A_44_P128110	AA926063	NR_002156		-0.19	-1.556	0.020509
A_44_P551860	BF420770	BF420770	316123	-0.19	-1.556	0.028258
A_44_P524208	BF406671	BF406671		-0.19	-1.556	0.006037
A_44_P435867	RGD1559626_predicted	XM_224879		-0.19	-1.556	0.001519
A_44_P288359	RGD1563739_predicted	XM_223826		-0.19	-1.556	0.005397
A_44_P380060	Lrrc50	NM_001014154	361419	-0.19	-1.556	0.009265
A_44_P245433	Olr221_predicted	NM_001000200	293367	-0.19	-1.556	0.065499
A_44_P518131	Adam34_predicted	XM_344523		-0.19	-1.556	0.079068
A_44_P281565	Hook1_predicted	XM_233196		-0.19	-1.556	0.00393
A_44_P101280	BQ210058	BQ210058	689055	-0.19	-1.556	0.047838
A_44_P917844	Cdc14a_predicted	XM_227618		-0.19	-1.557	0.012374
A_44_P341915	Olr360_predicted	NM_001000755	405026	-0.19	-1.557	0.014636

A_44_P363585	Arhgap26_predicted	XM_225989		-0.19	-1.557	0.073013
A_44_P791631	RGD1565414_predicted	XM_001054658		-0.19	-1.557	0.003098
A_43_P11288	RGD1562991_predicted	XM_215225		-0.19	-1.557	0.011568
A_44_P202527	AW916440	AW916440		-0.19	-1.557	0.00284
A_44_P806069	ENSRNOT00000037631	ENSRNOT00000037631		-0.19	-1.557	0.001792
A_44_P113643	AA998203	AA998203	503035	-0.19	-1.558	0.016105
A_44_P159176	XM_345325	XM_345325		-0.19	-1.558	0.038781
A_43_P11476	Inha	NM_012590	24504	-0.19	-1.558	0.01503
A_44_P480472	XM_224421	XM_224421		-0.19	-1.558	0.014403
A_44_P348726	AI232526	AI232526	288924	-0.19	-1.558	0.004556
A_43_P23061	BF544435	BF544435		-0.19	-1.558	0.028158
A_42_P668858	Tcf2	NM_013103	25640	-0.19	-1.558	0.009094
A_44_P473074	Slc22a5	NM_019269	29726	-0.19	-1.558	0.018587
A_43_P11221	Afg3l2	XM_225866	307350	-0.19	-1.559	0.001361
A_44_P431049	Rrad	NM_053338	83521	-0.19	-1.559	0.003715
A_44_P867427	A_44_P867427	A_44_P867427		-0.19	-1.559	0.03876
A_44_P463488	Amh	NM_012902	25378	-0.19	-1.559	0.082677
A_44_P332021	S100a4	BF396146	24615	-0.19	-1.559	0.167397
A_44_P982706	A_44_P982706	A_44_P982706		-0.19	-1.559	0.003594
A_44_P999132	Masp2	NM_172043	64459	-0.19	-1.559	0.008293
A_43_P12878	St14	NM_053635	114093	-0.19	-1.559	0.027014
A_42_P597340	RGD1309529_predicted	XM_215378		-0.19	-1.560	0.00435
A_44_P418415	AI060197	AI060197	360698	-0.19	-1.560	0.008211
A_44_P377781	XM_234990	XM_234990		-0.19	-1.560	0.046967
A_44_P461331	Slc25a16	XM_342129	361836	-0.19	-1.560	0.001073
A_43_P17945	F12	NM_001014006	306761	-0.19	-1.560	0.007044
A_44_P1014659	Plek2_predicted	XM_234329		-0.19	-1.560	0.008879
A_44_P527614	XM_230900	XM_230900		-0.19	-1.560	0.038715
A_44_P169972	BI294262	BI294262	366734	-0.19	-1.560	0.052472
A_43_P22252	Abcg4	XM_236186		-0.19	-1.560	0.00473
A_44_P266817	Klra22	NM_173291	24933	-0.19	-1.560	0.035536
A_42_P463998	Abcc6	NM_031013	81642	-0.19	-1.561	0.01819
A_44_P322805	BE114153	BE114153	293488	-0.19	-1.561	0.111348
A_44_P376940	Otop1	NM_181433	305427	-0.19	-1.561	0.169643
A_44_P234168	XM_226966	XM_226966		-0.19	-1.561	0.016458
A_44_P130654	RGD1562454_predicted	XM_218668		-0.19	-1.561	0.009143
A_44_P475893	XM_226554	XM_226554		-0.19	-1.561	0.028731
A_44_P370345	RGD1311920_predicted	XM_223651	305544	-0.19	-1.561	0.012629
A_44_P170368	Fmo4	NM_144562	246247	-0.19	-1.562	0.022276
A_44_P1047966	RGD1564440_predicted	XM_342386	362085	-0.19	-1.562	0.018014
A_44_P376677	AW143323	AW143323		-0.19	-1.562	0.208683
A_43_P13081	Cacng8	NM_080696	140729	-0.19	-1.562	0.004578
A_44_P302145	LOC501925	XM_577349	501925	-0.19	-1.562	0.109906
A_43_P12065	Park2	NM_020093	56816	-0.19	-1.562	0.064177
A_44_P255039	Olr307_predicted	NM_001000241	293605	-0.19	-1.562	0.103779
A_44_P256457	Usp52	NM_001008862	408200	-0.19	-1.563	0.005097
A_44_P945931	TC524217	TC524217		-0.19	-1.563	0.035961
A_44_P437214	CK843054	CK843054	313774	-0.19	-1.563	0.039401
A_43_P21978	Lamb3	XM_223087	305078	-0.19	-1.563	0.029599
A_43_P10944	LOC686539	XM_001074652		-0.19	-1.563	0.194382
A_44_P433367	Elac2	AF546755	282826	-0.19	-1.563	0.023831
A_44_P114052	Htr6	NM_024365	64354	-0.19	-1.563	0.00375
A_44_P279240	Rpl29	NM_017150	29283	-0.19	-1.563	0.025051
A_44_P312695	M71245	M71245		-0.19	-1.564	0.031886
A_44_P353102	AA819532	AA819532	117103	-0.19	-1.564	0.143775
A_43_P13772	CB547736	CB547736		-0.19	-1.564	0.051376
A_44_P554867	AW917688	AW917688		-0.19	-1.564	0.085601
A_44_P1049532	RGD1311863	NM_001024974	293871	-0.19	-1.564	0.02839
A_44_P334810	Olr862_predicted	NM_001001071	405961	-0.19	-1.564	0.204518
A_44_P344612	Ms4a1_predicted	XM_219590		-0.19	-1.564	0.034925
A_44_P330998	XM_343048	XM_343048		-0.19	-1.564	0.002073
A_42_P676553	Tnni3	NM_017144	29248	-0.19	-1.564	0.058119
A_44_P334928	Ubtf	XM_340913		-0.19	-1.564	0.038733

A_44_P253525	AA817877	AA817877		-0.19	-1.565	0.035901
A_44_P638239	TC523444	TC523444		-0.19	-1.565	0.00526
A_44_P382803	BF559195	BF559195		-0.19	-1.565	0.057335
A_44_P574106	AA851392	AA851392	293502	-0.19	-1.565	0.004389
A_43_P20285	CB548449	CB548449	289362	-0.19	-1.565	0.009459
A_44_P244506	AA946181	AA946181	293343	-0.19	-1.565	0.024765
A_44_P621621	LOC684409	XM_001070273		-0.19	-1.565	0.06376
A_42_P573412	RGD1307150_predicted	XM_217268		-0.19	-1.566	0.392936
A_44_P492707	BQ208687	BQ208687	309377	-0.19	-1.566	0.034134
A_44_P276530	RGD1310594_predicted	XM_213776		-0.19	-1.566	0.003218
A_44_P746944	TC560163	TC560163		-0.19	-1.566	0.006059
A_44_P325971	A_44_P325971	A_44_P325971		-0.19	-1.566	0.02143
A_44_P158112	CA945919	CA945919	317335	-0.19	-1.566	0.115768
A_44_P480593	ENSRNOT00000048078	ENSRNOT00000048078		-0.19	-1.566	0.21769
A_44_P469166	AA946389	AA946389	362581	-0.19	-1.566	0.029561
A_44_P361041	ENSRNOT00000038839	ENSRNOT00000038839		-0.19	-1.567	0.002444
A_44_P477038	AI501306	AI501306	25625	-0.20	-1.567	0.159188
A_44_P928969	A_44_P928969	A_44_P928969		-0.20	-1.567	0.310244
A_44_P854011	Ppfia4	XM_001061955		-0.20	-1.567	0.038982
A_44_P343064	BF285323	BF285323	290880	-0.20	-1.567	0.064518
A_44_P353822	Mip	XM_343137		-0.20	-1.567	0.051891
A_44_P118625	Calb1	NM_031984	83839	-0.20	-1.567	0.005484
A_44_P752186	TC530797	TC530797		-0.20	-1.567	0.041205
A_42_P514501	Grm3	XM_001062001		-0.20	-1.568	0.105638
A_44_P307416	AI556132	AI556132		-0.20	-1.568	0.038026
A_44_P372847	LOC365969	XM_345306		-0.20	-1.568	0.014677
A_44_P537250	CB545242	CB545242	287622	-0.20	-1.568	0.032655
A_44_P334491	AW525042	AW525042	313689	-0.20	-1.568	0.04223
A_43_P19170	Cnih2	NM_001025132	361705	-0.20	-1.568	0.006278
A_44_P255280	Gata1	NM_012764	25172	-0.20	-1.568	0.040528
A_42_P830209	Frmpd1_predicted	XM_233002		-0.20	-1.568	0.01387
A_44_P197251	AA866259	AA866259		-0.20	-1.568	0.024594
A_44_P381069	Glul	BC072694	24957	-0.20	-1.569	0.014505
A_44_P236561	A_44_P236561	A_44_P236561		-0.20	-1.569	0.100219
A_44_P109342	Hspa1a	NM_031971	24472	-0.20	-1.569	0.030705
A_44_P419426	Tlr10_predicted	XM_223422		-0.20	-1.569	0.044666
A_44_P145334	BI302561	BI302561	303601	-0.20	-1.569	0.058111
A_44_P328450	LOC290921	XM_225059		-0.20	-1.569	0.032538
A_44_P210087	AI030133	AI030133	64636	-0.20	-1.569	0.030168
A_44_P363181	Cpd	NM_012836	25306	-0.20	-1.570	0.024502
A_44_P1024981	Alas1	NM_024484	65155	-0.20	-1.570	0.0129
A_44_P606516	TC517867	TC517867		-0.20	-1.570	0.002899
A_44_P321075	Rasal2_predicted	XM_222780		-0.20	-1.570	0.016894
A_44_P364155	BQ192072	BQ192072		-0.20	-1.571	0.011264
A_43_P18339	LOC688705	XM_001067750	688705	-0.20	-1.571	0.043162
A_43_P14888	Sgne1	NM_013175	25719	-0.20	-1.571	0.110558
A_44_P286264	Gba2	XM_216490	298399	-0.20	-1.571	0.009811
A_44_P567252	AI229633	AI229633	25348	-0.20	-1.571	0.055966
A_44_P114533	A_44_P114533	A_44_P114533		-0.20	-1.571	0.036902
A_44_P103205	A_44_P103205	A_44_P103205		-0.20	-1.571	0.122112
A_43_P23371	XM_343598	XM_343598		-0.20	-1.571	0.033998
A_44_P854908	CB583330	CB583330		-0.20	-1.572	0.005396
A_42_P472509	LOC683547	XM_001066473		-0.20	-1.572	0.002689
A_43_P11753	Acot7	NM_013214	26759	-0.20	-1.572	0.006889
A_44_P199326	A_44_P199326	A_44_P199326		-0.20	-1.572	0.004426
A_44_P929812	LOC691853	XM_001079869	691853	-0.20	-1.572	0.010136
A_44_P327997	Zhx3	XM_230811		-0.20	-1.573	0.141405
A_43_P19268	Gsh2_predicted	XM_344233	364140	-0.20	-1.573	0.058505
A_44_P128986	LOC619574	NM_001034959	619574	-0.20	-1.573	0.037206
A_44_P398077	LOC682950	XM_001060921		-0.20	-1.573	0.072678
A_44_P111313	BF403405	BF403405	317431	-0.20	-1.573	0.080364
A_44_P262854	ENSRNOT00000041931	ENSRNOT00000041931		-0.20	-1.573	0.015191
A_42_P708480	Slco1a4	NM_131906	170698	-0.20	-1.573	0.105089

A_44_P1024721	Slc28a2	NM_031664	60423	-0.20	-1.573	0.074495
A_42_P646290	Gnaz	NM_013189	25740	-0.20	-1.573	0.077365
A_44_P261720	BQ780678	BQ780678	294350	-0.20	-1.573	0.147214
A_44_P980785	A_44_P980785	A_44_P980785		-0.20	-1.573	0.112325
A_44_P426478	XM_239603	XM_239603		-0.20	-1.573	0.005696
A_44_P557345	Prkci	XM_342223	84006	-0.20	-1.574	0.027738
A_44_P360486	Cyp4b1	NM_016999	24307	-0.20	-1.574	0.032072
A_44_P120068	AA800803	AA800803	360639	-0.20	-1.574	0.013892
A_44_P1003212	Igf2r	NM_012756	25151	-0.20	-1.574	0.015136
A_44_P510588	Olr1334_predicted	NM_001000478	300634	-0.20	-1.574	0.076299
A_44_P376994	Lsamp	NM_017242	29561	-0.20	-1.574	0.139672
A_44_P1039034	AW918967	AW918967		-0.20	-1.574	0.013331
A_44_P900178	TC558449	TC558449		-0.20	-1.574	0.036048
A_43_P12714	C3ar1	NM_032060	84007	-0.20	-1.574	0.140104
A_44_P421897	XM_227056	XM_227056		-0.20	-1.574	0.036884
A_44_P277058	RGD1304775_predicted	XM_237151	316399	-0.20	-1.575	0.011552
A_44_P455501	BF410227	BF410227	301113	-0.20	-1.575	0.013638
A_42_P495498	LOC679252	XM_001054661		-0.20	-1.575	0.01147
A_44_P385196	AA894292	AA894292		-0.20	-1.575	0.018155
A_44_P256188	LOC497860	BC103490	497860	-0.20	-1.575	0.006261
A_44_P619324	AB014883	AB014883		-0.20	-1.575	0.032338
A_44_P639627	Chd1_predicted	XM_001056703		-0.20	-1.575	0.012793
A_44_P118337	AI029007	AI029007	298688	-0.20	-1.575	0.200871
A_44_P442569	Esr2	NM_012754	25149	-0.20	-1.575	0.063442
A_44_P114774	RGD1563028_predicted	XM_343190		-0.20	-1.576	0.024197
A_44_P291196	RGD1563331_predicted	XM_221083	303660	-0.20	-1.576	0.00332
A_44_P130217	BG670404	BG670404	294734	-0.20	-1.576	0.021272
A_44_P732939	Cpeb4_predicted	XM_220269		-0.20	-1.576	0.022349
A_43_P22700	TC532619	TC532619		-0.20	-1.576	0.00803
A_44_P1036685	Mrpl12	NM_001029900	303746	-0.20	-1.576	0.008607
A_44_P127328	AA859869	AA859869	83806	-0.20	-1.576	0.010706
A_44_P396273	A_44_P396273	A_44_P396273		-0.20	-1.576	0.066061
A_44_P123813	Cobl_predicted	XM_223597		-0.20	-1.576	0.023284
A_42_P685404	Gpc2	NM_138511	171517	-0.20	-1.576	0.226812
A_44_P534361	RGD1565043_predicted	XM_001074859		-0.20	-1.576	0.018772
A_44_P480682	RGD1308850_predicted	XM_342744		-0.20	-1.577	0.081876
A_44_P898999	TC553312	TC553312		-0.20	-1.577	0.010486
A_44_P395173	AI104361	AI104361	362437	-0.20	-1.577	0.040824
A_44_P340172	Hao1_mapped	XM_230613		-0.20	-1.577	0.023342
A_44_P183290	AA900739	AA900739		-0.20	-1.578	0.049422
A_44_P112683	BF550202	BF550202	313742	-0.20	-1.578	0.019631
A_44_P318957	LOC680020	XM_001055350		-0.20	-1.578	0.095539
A_44_P492674	BQ209315	BQ209315		-0.20	-1.578	0.003377
A_44_P276227	Usp19	NM_001001516	361190	-0.20	-1.578	0.027323
A_43_P13711	LOC502414	NM_001025059	502414	-0.20	-1.579	0.012807
A_44_P548257	Tmem58_predicted	XM_341125		-0.20	-1.579	0.135407
A_44_P358361	H2-T18	NM_001002821	406194	-0.20	-1.579	0.029134
A_44_P545553	Dgkb	NM_019304	54248	-0.20	-1.579	0.022568
A_42_P585995	RGD1308116_predicted	XM_227081	310376	-0.20	-1.579	0.039207
A_44_P302458	Trim14_predicted	XM_232992		-0.20	-1.579	0.015921
A_44_P431795	AA924907	AA924907		-0.20	-1.579	0.026551
A_44_P101713	AW434670	AW434670	316071	-0.20	-1.579	0.068576
A_44_P208304	Pak3	NM_019210	29433	-0.20	-1.579	0.052461
A_44_P461470	Ank	NM_053714	114506	-0.20	-1.579	0.011266
A_44_P421385	Olr1399_predicted	NM_001001095	405990	-0.20	-1.580	0.033414
A_43_P22649	RGD1564161_predicted	XM_342881		-0.20	-1.580	0.030051
A_44_P837792	TC537731	TC537731		-0.20	-1.580	0.013308
A_44_P241321	AW915986	AW915986		-0.20	-1.580	0.043199
A_44_P591147	LOC685774	XM_001065216		-0.20	-1.580	0.10264
A_44_P103254	XM_235289	XM_235289		-0.20	-1.581	0.061869
A_44_P107869	RGD1565951_predicted	XM_234130		-0.20	-1.581	0.047626
A_43_P15556	Gulo	NM_022220	60671	-0.20	-1.581	0.113389
A_44_P138410	AA858873	AA858873		-0.20	-1.581	0.133108

A_43_P11720	Apoa2	NM_013112	25649	-0.20	-1.581	0.041498
A_43_P12744	Chrnb4	NM_052806	25103	-0.20	-1.582	0.025225
A_44_P419004	Agpat1	NM_212458	406165	-0.20	-1.582	0.003021
A_44_P196316	Olr6_predicted	NM_001000539	365175	-0.20	-1.582	0.046826
A_43_P18361	Cd7_predicted	XM_221216		-0.20	-1.582	0.003021
A_44_P419451	Ms4a12_predicted	XM_219588		-0.20	-1.582	0.020171
A_44_P1018147	Isoc1	NM_001014242	364879	-0.20	-1.582	0.011372
A_44_P677215	LOC682010	XM_001058281		-0.20	-1.582	0.016425
A_44_P328205	Bdkrb1	NM_030851	81509	-0.20	-1.582	0.013737
A_42_P604382	RGD1561064_predicted	XM_573691		-0.20	-1.582	0.022984
A_43_P18460	LOC678709	XM_001053080	678709	-0.20	-1.582	0.007921
A_44_P467402	AI103873	AI103873	362245	-0.20	-1.582	0.003543
A_44_P269095	Sardh	NM_053664	114123	-0.20	-1.583	0.233901
A_44_P208317	RGD1560695_predicted	XM_344801		-0.20	-1.583	0.038494
A_44_P348229	Fuca	NM_012562	24375	-0.20	-1.583	0.010473
A_44_P211127	Paqr9_predicted	XM_236503	315904	-0.20	-1.583	0.002947
A_44_P142016	Psen1	NM_019163	29192	-0.20	-1.584	0.006608
A_44_P389212	RGD1307336	NM_001024975	293950	-0.20	-1.584	0.010785
A_44_P202940	AI171966	AI171966	294273	-0.20	-1.584	0.003623
A_44_P271541	MGC72942	NM_212516	300677	-0.20	-1.584	0.002377
A_44_P777439	TC524058	TC524058		-0.20	-1.584	0.006211
A_44_P328701	ENSRNOT00000043091	ENSRNOT00000043091		-0.20	-1.584	0.185663
A_44_P782781	BF398551	BF398551		-0.20	-1.584	0.050528
A_44_P521564	AI045861	AI045861	117263	-0.20	-1.585	0.009305
A_44_P1028551	Pmm1	NM_001008323	300089	-0.20	-1.585	0.001959
A_44_P318539	Camk2b	NM_001042354	24245	-0.20	-1.586	0.00715
A_44_P527270	Pthr1	NM_020073	56813	-0.20	-1.586	0.006168
A_44_P518445	XM_234865	XM_234865		-0.20	-1.586	0.013189
A_44_P184205	BF406830	BF406830	689134	-0.20	-1.586	0.041138
A_44_P515776	RGD1564676_predicted	XM_344589		-0.20	-1.586	0.046597
A_44_P491253	Zmynd10	NM_001004284	363139	-0.20	-1.586	0.001902
A_44_P185921	BF545106	BF545106		-0.20	-1.586	0.004817
A_43_P19563	XM_341697	XM_341697		-0.20	-1.586	0.044273
A_44_P165732	Plp	NM_030990	24943	-0.20	-1.587	0.008675
A_44_P1056293	Ppp2r5d	XM_001062510	363193	-0.20	-1.587	0.001805
A_44_P178357	RGD1565168_predicted	XM_345725	366733	-0.20	-1.587	0.342566
A_44_P290987	LOC301132	XM_217320		-0.20	-1.587	0.021724
A_44_P284247	BE112719	BE112719	685612	-0.20	-1.587	0.03567
A_44_P260227	RGD1560591_predicted	XM_341993	361708	-0.20	-1.587	0.026362
A_44_P896283	BF404842	BF404842		-0.20	-1.587	0.038243
A_44_P366832	Cdc42bpa	NM_053657	114116	-0.20	-1.588	0.001367
A_44_P175041	Nr1d1	NM_145775	252917	-0.20	-1.588	0.088018
A_44_P406819	AA998221	AA998221		-0.20	-1.588	0.003903
A_44_P102628	Kcnk1	NM_021688	59324	-0.20	-1.588	0.00431
A_42_P675031	Kcnb1	NM_013186	25736	-0.20	-1.588	0.002844
A_44_P443315	RGD1564768_predicted	XM_228743		-0.20	-1.588	0.002809
A_44_P468737	Rnf125_predicted	XM_341581		-0.20	-1.588	0.005332
A_44_P993934	Rnf8	NM_001025727	361815	-0.20	-1.589	0.073083
A_44_P144988	RGD1309807	XM_342702	362378	-0.20	-1.589	0.016491
A_44_P392207	BE112995	BE112995		-0.20	-1.589	0.04499
A_43_P23118	Igfbpl1_predicted	XM_345529		-0.20	-1.589	0.007953
A_44_P845111	AW140998	AW140998		-0.20	-1.589	0.020667
A_43_P17277	Gja12_predicted	XM_573100	497913	-0.20	-1.589	0.057305
A_44_P575442	ENSRNOT00000041572	ENSRNOT00000041572		-0.20	-1.589	0.13424
A_44_P1022834	Lrp11_predicted	XM_238072		-0.20	-1.589	0.05924
A_44_P160504	AI010416	AI010416	360548	-0.20	-1.589	0.014427
A_42_P486144	Gata4	NM_144730	54254	-0.20	-1.589	0.125953
A_44_P144277	Pkn1	NM_017175	29355	-0.20	-1.589	0.001618
A_44_P535496	BI273936	BI273936	679869	-0.20	-1.590	0.041797
A_44_P561324	Akap1	BC081753	114124	-0.20	-1.590	0.005023
A_44_P346750	Impa1	NM_032057	83523	-0.20	-1.590	0.005064
A_44_P491872	Insrr	XM_574972	60663	-0.20	-1.590	0.005998
A_44_P273837	Fgf22	NM_130751	170579	-0.20	-1.590	0.007565

A_44_P414565	Gabra5	NM_017295	29707	-0.20	-1.590	0.027541
A_44_P508475	Dnm2	NM_013199	25751	-0.20	-1.590	0.081187
A_44_P404768	AF468681	AF468681		-0.20	-1.591	0.011256
A_44_P382523	RGD1311958_predicted	XM_236376		-0.20	-1.591	0.022329
A_44_P148542	AI059837	AI059837	304055	-0.20	-1.591	0.00645
A_44_P441881	AI178495	AI178495		-0.20	-1.591	0.074119
A_44_P450307	Eps8_predicted	XM_001072957		-0.20	-1.591	0.003094
A_44_P466896	AI113011	AI113011		-0.20	-1.591	0.004482
A_44_P379951	LOC364654	XM_344558	364654	-0.20	-1.591	0.032179
A_44_P139836	Pgea1	NM_145676	246768	-0.20	-1.592	0.021454
A_44_P237938	Vps36_predicted	XM_214382		-0.20	-1.592	0.008472
A_44_P107000	AI409747	AI409747	295105	-0.20	-1.592	0.010493
A_44_P708829	TC536840	TC536840		-0.20	-1.592	0.039719
A_43_P23458	Klk12_predicted	XM_218644		-0.20	-1.592	0.002697
A_44_P424092	RGD1307161	NM_001025669	305031	-0.20	-1.592	0.048647
A_44_P700601	DV717996	DV717996	688864	-0.20	-1.592	0.004454
A_44_P154008	Mtnr1b	XM_345899	192646	-0.20	-1.592	0.010768
A_44_P109998	RGD1562174_predicted	XM_227287	310538	-0.20	-1.592	0.001712
A_43_P12431	Gja3	NM_024376	79217	-0.20	-1.593	0.015075
A_42_P536393	Mrps36_predicted	XM_215468	294696	-0.20	-1.593	0.001424
A_44_P573268	AW143290	AW143290		-0.20	-1.593	0.004105
A_43_P15062	Rpl3l_predicted	XM_213231	287122	-0.20	-1.594	0.016387
A_44_P263146	AA851427	AA851427	652928	-0.20	-1.594	0.036823
A_44_P730251	TC522656	TC522656		-0.20	-1.594	0.011129
A_44_P271923	Rom1	NM_001009690	309201	-0.20	-1.594	0.003963
A_44_P1056401	RGD1303232	NM_001004261	309381	-0.20	-1.594	0.01402
A_42_P740161	Wdr79	NM_001007610	287432	-0.20	-1.594	0.105718
A_44_P470772	Casq2	NM_017131	29209	-0.20	-1.595	0.009479
A_44_P628127	RGD1561030_predicted	XM_001066889		-0.20	-1.595	0.020217
A_43_P13345	Prokr1	NM_138977	192648	-0.20	-1.595	0.075692
A_44_P115321	AA944251	AA944251		-0.20	-1.595	0.033113
A_44_P280493	AA964882	AA964882	680229	-0.20	-1.595	0.136059
A_42_P780613	Hs3st2	NM_181370	293451	-0.20	-1.595	0.05986
A_44_P294461	AA925221	AA925221	689079	-0.20	-1.595	0.01321
A_43_P13016	Zbtb7a	NM_054002	117107	-0.20	-1.596	0.003258
A_43_P12474	Ap2a2	NM_031008	81637	-0.20	-1.596	0.032972
A_42_P487031	B4galt3	NM_001009539	494342	-0.20	-1.596	0.081362
A_44_P204789	Pex11b	NM_001025684	310682	-0.20	-1.596	0.011554
A_44_P263812	AA851658	AA851658	64472	-0.20	-1.596	0.086555
A_44_P577723	TC560802	TC560802		-0.20	-1.596	0.058593
A_44_P162918	AI045503	AI045503	25680	-0.20	-1.596	0.109274
A_44_P316611	Pknox1	NM_001013074	294322	-0.20	-1.596	0.138941
A_44_P244063	LOC363458	XM_001056684		-0.20	-1.597	0.020868
A_44_P1043073	LOC686503	XM_001074441		-0.20	-1.597	0.11501
A_44_P433568	Sytl2_predicted	XM_341882		-0.20	-1.597	0.016005
A_44_P540944	Plekha5	XM_342781	246237	-0.20	-1.597	0.001397
A_44_P548976	BF282495	BF282495	306460	-0.20	-1.597	0.059974
A_42_P763157	Cyp2t1	NM_134369	171380	-0.20	-1.597	0.038317
A_44_P107558	XM_344200	XM_344200		-0.20	-1.597	0.212414
A_44_P1056429	RGD1307844_predicted	XM_236715	316085	-0.20	-1.597	0.001894
A_44_P853554	TC556784	TC556784		-0.20	-1.597	0.036781
A_44_P696611	BI301428	BI301428	367100	-0.20	-1.598	0.012728
A_44_P241732	AW253701	AW253701	290669	-0.20	-1.598	0.017306
A_44_P242694	AI071874	AI071874	24924	-0.20	-1.599	0.046873
A_44_P102725	LOC680440	XM_001057808		-0.20	-1.599	0.044485
A_43_P11961	Galr2	NM_019172	29234	-0.20	-1.599	0.003759
A_44_P436089	RGD1563485_predicted	XM_217657	307992	-0.20	-1.599	0.029167
A_44_P159382	Trp73_predicted	XM_342992		-0.20	-1.599	0.022083
A_44_P251241	AA923942	AA923942		-0.20	-1.599	0.026212
A_42_P638474	TC524131	TC524131		-0.20	-1.599	0.006237
A_44_P949064	TC530154	TC530154		-0.20	-1.600	0.137459
A_44_P330330	Slc5a2	NM_022590	64522	-0.20	-1.600	0.059974
A_44_P369189	AI012814	AI012814	58966	-0.20	-1.600	0.02509

A_44_P552670	LOC499715	XM_575044		-0.20	-1.600	0.043373
A_43_P21209	RGD1307598	NM_001037644	304656	-0.20	-1.600	0.005541
A_43_P13900	AA899996	AA899996		-0.20	-1.600	0.019164
A_44_P690506	BE107707	BE107707		-0.20	-1.600	0.022188
A_43_P12995	Kcns1	NM_053954	117023	-0.20	-1.600	0.027033
A_44_P919895	TC544448	TC544448		-0.20	-1.600	0.070969
A_44_P383088	AF366899	AF366899		-0.20	-1.600	0.018265
A_44_P823383	TC558225	TC558225		-0.20	-1.600	0.050242
A_44_P636423	LOC685110	XM_001063661	685110	-0.20	-1.600	0.186292
A_44_P395673	Olr857_predicted	NM_001000583	366456	-0.20	-1.601	0.035399
A_44_P409709	Phldb1	XM_001067414		-0.20	-1.601	0.00865
A_43_P19895	Rnf29	NM_001012218	365751	-0.20	-1.601	0.031937
A_43_P21148	Gabra1	NM_183326	29705	-0.20	-1.601	0.036098
A_44_P198349	Aco2	NM_024398	79250	-0.20	-1.601	0.012445
A_43_P16272	Cftr	M89906	24255	-0.20	-1.601	0.027775
A_44_P135241	M18848	M18848		-0.20	-1.601	0.017017
A_43_P11268	Cdkn1b	NM_031762	83571	-0.20	-1.601	0.016671
A_44_P106932	CK597683	CK597683	293481	-0.20	-1.601	0.012869
A_44_P788211	RGD1310623	NM_001034011	360868	-0.20	-1.601	0.043167
A_44_P217107	AA818576	AA818576		-0.20	-1.601	0.01966
A_43_P16562	RGD1310686	NM_001008360	360480	-0.20	-1.602	0.117504
A_44_P133260	A_44_P133260	A_44_P133260		-0.20	-1.602	0.07275
A_44_P457578	AA858732	AA858732	25211	-0.20	-1.602	0.043326
A_44_P683244	ENSRNOT00000049532	ENSRNOT00000049532		-0.20	-1.602	0.019699
A_44_P563512	TC527750	TC527750		-0.20	-1.602	0.028664
A_43_P21419	Alg12_predicted	XM_235555		-0.20	-1.602	0.042786
A_44_P379832	XM_344241	XM_344241		-0.20	-1.602	0.005963
A_44_P1021866	TC561781	TC561781		-0.20	-1.602	0.017006
A_44_P128888	Tbc1d22a_predicted	XM_001078492		-0.20	-1.602	0.001189
A_42_P672622	Epb4.1l1	NM_172090	59317	-0.20	-1.602	0.007351
A_43_P10533	AW918416	AW918416	360618	-0.20	-1.603	0.015652
A_43_P14928	Gak	NM_031030	81659	-0.20	-1.603	0.001802
A_43_P21329	Als2cr13_predicted	XM_343576		-0.20	-1.603	0.002793
A_44_P543559	RGD1311340_predicted	XM_219672		-0.20	-1.603	0.00622
A_44_P295961	AA956484	AA956484		-0.20	-1.603	0.008611
A_44_P520339	RGD1562464_predicted	XM_222908		-0.20	-1.603	0.015238
A_44_P557626	Klk9_predicted	XM_218647		-0.20	-1.603	0.028212
A_44_P408577	AI228129	AI228129	679949	-0.20	-1.603	0.108871
A_44_P428227	AW920832	AW920832		-0.21	-1.603	0.109479
A_44_P914875	TC556312	TC556312		-0.21	-1.603	0.007794
A_44_P590627	A_44_P590627	A_44_P590627		-0.21	-1.603	0.25529
A_44_P201448	LOC682257	XM_001059001		-0.21	-1.603	0.051142
A_44_P653693	TC523586	TC523586		-0.21	-1.604	0.005059
A_44_P223876	AI171535	AI171535	288398	-0.21	-1.604	0.079862
A_43_P18643	Gstm6_predicted	XM_215682		-0.21	-1.604	0.065952
A_44_P446882	Abcb6	NM_080582	140669	-0.21	-1.604	0.002011
A_44_P428988	LOC686420	XM_001074024		-0.21	-1.604	0.003641
A_44_P391126	Olr56_predicted	NM_001000130	293205	-0.21	-1.604	0.029481
A_44_P281540	LOC294560	XM_215428		-0.21	-1.604	0.009575
A_44_P493571	AA999013	AA999013	305506	-0.21	-1.604	0.032528
A_44_P1051631	Ilvbl_predicted	XM_343174		-0.21	-1.605	0.003121
A_44_P929395	LOC680068	XM_001055549	680068	-0.21	-1.605	0.040785
A_44_P246010	Kif16b_predicted	XM_230634		-0.21	-1.605	0.0017
A_44_P159218	RGD1563001_predicted	XM_230567		-0.21	-1.605	0.030342
A_43_P17491	Mmp15_predicted	XM_238034		-0.21	-1.606	0.000576
A_44_P306204	Tph1	X53501		-0.21	-1.606	0.06549
A_44_P370219	XM_221810	XM_221810		-0.21	-1.606	0.023561
A_43_P18164	RGD1562672_predicted	XM_228590		-0.21	-1.606	0.01485
A_44_P347434	Hoxb2_predicted	XM_220894		-0.21	-1.606	0.027198
A_44_P447823	XM_219913	XM_219913		-0.21	-1.606	0.021098
A_44_P177793	A_44_P177793	A_44_P177793		-0.21	-1.606	0.0359
A_44_P492153	Mtmr6_predicted	XM_224271		-0.21	-1.606	0.005163
A_44_P636857	ENSRNOT00000038550	ENSRNOT00000038550		-0.21	-1.606	0.047555

A_44_P296180	Arg2	NM_019168	29215	-0.21	-1.606	0.003286
A_44_P457558	AI144777	AI144777		-0.21	-1.607	0.026399
A_44_P421312	P2ry1	NM_012800	25265	-0.21	-1.607	0.011999
A_44_P809962	TC533709	TC533709		-0.21	-1.607	0.033275
A_44_P388435	AI059275	AI059275	64154	-0.21	-1.607	0.040931
A_44_P466645	XM_216425	XM_216425		-0.21	-1.607	0.149503
A_44_P490337	BQ210985	BQ210985	296624	-0.21	-1.607	0.007446
A_44_P353872	RGD1305932_predicted	XM_218387	292755	-0.21	-1.607	0.003429
A_42_P786933	Myo1d	NM_012983	25485	-0.21	-1.607	0.00138
A_44_P188275	Spag4	NM_031792	83623	-0.21	-1.607	0.007662
A_43_P15129	BF558849	BF558849		-0.21	-1.608	0.004717
A_44_P346637	Pcp4	NM_013002	25510	-0.21	-1.608	0.028165
A_42_P562711	Ndufb9_predicted	XM_216929	299954	-0.21	-1.608	0.000867
A_44_P220575	Irak4_predicted	XM_217026		-0.21	-1.608	0.006122
A_44_P829715	TC541000	TC541000		-0.21	-1.608	0.006766
A_44_P1002531	AI237606	AI237606	303836	-0.21	-1.608	0.027282
A_44_P372377	Olr1439_predicted	NM_001000016	287328	-0.21	-1.608	0.102894
A_42_P734868	Kcnab3	NM_031652	58981	-0.21	-1.609	0.003226
A_44_P1035678	RGD1309605_predicted	XM_214513		-0.21	-1.609	0.003492
A_44_P761309	LOC362261	NM_001014184	362261	-0.21	-1.609	0.001334
A_44_P793279	TC544290	TC544290		-0.21	-1.609	0.001839
A_44_P370355	LOC305639	XR_005817	305639	-0.21	-1.609	0.013094
A_43_P13147	Fgf6	NM_131908	170700	-0.21	-1.609	0.004693
A_44_P902375	TC523917	TC523917		-0.21	-1.609	0.105638
A_44_P268859	AI044306	AI044306	361351	-0.21	-1.609	0.018224
A_44_P220519	Lrrn6a	XM_236268	315691	-0.21	-1.609	0.003433
A_44_P550397	Hlf	XM_001081149		-0.21	-1.610	0.010718
A_44_P384046	Olr1330_predicted	NM_001000476	300630	-0.21	-1.610	0.065076
A_44_P730282	DV724088	DV724088		-0.21	-1.610	0.014959
A_44_P340019	A_44_P340019	A_44_P340019		-0.21	-1.610	0.110579
A_44_P444614	BF284803	BF284803	293624	-0.21	-1.610	0.039422
A_44_P179806	Prm2	NM_012873	25345	-0.21	-1.610	0.007888
A_44_P149380	Aoc3	NM_031582	29473	-0.21	-1.610	0.006681
A_44_P167873	AA819899	AA819899	64365	-0.21	-1.610	0.05025
A_44_P541489	XM_343659	XM_343659		-0.21	-1.610	0.267933
A_44_P242329	Acaa2	NM_130433	170465	-0.21	-1.611	0.016786
A_43_P14882	Errfi1	NM_001014071	313729	-0.21	-1.611	0.003167
A_44_P491238	CB720720	CB720720	499927	-0.21	-1.611	0.069329
A_43_P17164	XM_217321	XM_217321		-0.21	-1.611	0.013971
A_44_P377418	Prkg1_mapped	XM_219805		-0.21	-1.611	0.060516
A_44_P450284	XM_227986	XM_227986		-0.21	-1.611	0.002431
A_42_P584710	Robo2	XM_213677	84409	-0.21	-1.611	0.118456
A_44_P901471	TC564172	TC564172		-0.21	-1.611	0.10562
A_43_P21830	XM_233269	XM_233269		-0.21	-1.611	0.010005
A_44_P416824	Otx2	XM_224009	305858	-0.21	-1.611	0.010973
A_42_P593381	RGD1307343	NM_001024988	300463	-0.21	-1.612	0.009458
A_44_P510809	Ptk2	NM_013081	25614	-0.21	-1.612	0.003903
A_44_P139561	BF408990	BF408990	302969	-0.21	-1.612	0.065504
A_43_P16158	XM_225491	XM_225491		-0.21	-1.612	0.021349
A_44_P348892	Olr1318_predicted	NM_001001087	405980	-0.21	-1.612	0.033364
A_44_P267828	AA957185	AA957185		-0.21	-1.613	0.111217
A_44_P380792	BF418815	BF418815	24366	-0.21	-1.613	0.03732
A_44_P338081	Zfp641_predicted	XM_235625		-0.21	-1.613	0.005062
A_44_P272148	Spire2_predicted	XM_001078003		-0.21	-1.613	0.002025
A_44_P478142	A_44_P478142	A_44_P478142		-0.21	-1.614	0.028593
A_44_P212083	RGD1566064_predicted	XM_341141	360865	-0.21	-1.614	0.056603
A_43_P13257	Acsbg1	NM_134389	171410	-0.21	-1.615	0.016263
A_43_P16259	Hoxd3_mapped	XM_213633	288152	-0.21	-1.615	0.009229
A_43_P19361	LOC316122	NM_212524	316122	-0.21	-1.615	0.012819
A_44_P446946	AI231792	AI231792	293524	-0.21	-1.615	0.07138
A_43_P17127	Abca8a_predicted	XM_221100	303638	-0.21	-1.615	0.015368
A_44_P225576	Armcx6	NM_001007757	363496	-0.21	-1.615	0.013334
A_42_P655644	Hadhb	NM_133618	171155	-0.21	-1.615	0.008731

A_44_P554925	RT1-Cl	NM_206848	24737	-0.21	-1.615	0.006805
A_44_P470186	AA849987	AA849987	360471	-0.21	-1.615	0.026564
A_44_P533731	AW251931	AW251931		-0.21	-1.615	0.106051
A_44_P520603	LOC690743	XM_001075467	690743	-0.21	-1.615	0.059731
A_44_P559149	CB568740	CB568740		-0.21	-1.616	0.009429
A_44_P575319	A_44_P575319	A_44_P575319		-0.21	-1.616	0.003588
A_44_P813707	BF548111	BF548111		-0.21	-1.616	0.073794
A_44_P456342	Dyrk2_predicted	XM_235179		-0.21	-1.616	0.027356
A_44_P122416	AI706196	AI706196	292777	-0.21	-1.616	0.039952
A_44_P456433	Prkcz	NM_022507	25522	-0.21	-1.616	0.002759
A_42_P502363	Znf575_predicted	XM_218446		-0.21	-1.616	0.062409
A_42_P663744	RGD1306208_predicted	XM_215978		-0.21	-1.616	0.018351
A_44_P473715	XM_237297	XM_237297		-0.21	-1.616	0.006439
A_42_P642743	Fh1	NM_017005	24368	-0.21	-1.616	0.002801
A_44_P368461	RGD1305170_predicted	XM_221358		-0.21	-1.616	0.013313
A_44_P547561	Znf213_predicted	XM_237754		-0.21	-1.616	0.001958
A_44_P1007613	Abca3	XM_220219	302973	-0.21	-1.616	0.008869
A_43_P21564	Trp53inp1	NM_181084	297822	-0.21	-1.616	0.013467
A_44_P448328	ENSRNOT00000042413	ENSRNOT00000042413		-0.21	-1.616	0.007049
A_44_P286127	LOC681858	XM_001058730		-0.21	-1.617	0.050666
A_44_P405800	TC529126	TC529126		-0.21	-1.617	0.005719
A_44_P182099	AI030774	AI030774	292949	-0.21	-1.617	0.004707
A_44_P748242	TC527496	TC527496		-0.21	-1.617	0.043322
A_44_P700917	TC559380	TC559380		-0.21	-1.617	0.002428
A_44_P791108	LOC313672	NM_001014070	313672	-0.21	-1.618	0.002279
A_44_P323045	Arl1	NM_022385	64187	-0.21	-1.618	0.004594
A_44_P187221	Rab27a	NM_017317	50645	-0.21	-1.618	0.034274
A_44_P898579	XM_232947	XM_232947		-0.21	-1.618	0.023155
A_42_P773675	Edg8	NM_021775	60399	-0.21	-1.618	0.057161
A_44_P539597	BM986638	BM986638		-0.21	-1.618	0.050315
A_44_P510844	LOC684385	XM_001070151		-0.21	-1.618	0.010804
A_44_P556319	Fgf18	NM_019199	29369	-0.21	-1.618	0.037863
A_44_P271812	RGD1563859_predicted	XM_218050		-0.21	-1.618	0.042084
A_44_P1047992	Proc	NM_012803	25268	-0.21	-1.618	0.017308
A_44_P120986	Ghsr	NM_032075	84022	-0.21	-1.618	0.010001
A_42_P707727	Slc22a4	NM_022270	64037	-0.21	-1.618	0.139638
A_44_P454300	Hatl3	NM_001003979	408213	-0.21	-1.619	0.048551
A_44_P410121	A_44_P410121	A_44_P410121		-0.21	-1.619	0.002459
A_44_P184363	Cand2	NM_181362	192226	-0.21	-1.619	0.007157
A_43_P18145	XM_233139	XM_233139		-0.21	-1.619	0.001261
A_44_P260555	Eif2c3_predicted	XM_233543	313593	-0.21	-1.619	0.037833
A_44_P575780	LOC680486	XM_001057401		-0.21	-1.619	0.003739
A_44_P271442	Myh3	NM_012604	24583	-0.21	-1.619	0.03551
A_44_P745129	TC534772	TC534772		-0.21	-1.619	0.143954
A_43_P16836	Cblc	NM_001034920	292699	-0.21	-1.619	0.049572
A_44_P321089	ENSRNOT00000005748	ENSRNOT00000005748		-0.21	-1.620	0.002258
A_44_P372853	Psx1_predicted	XM_231064		-0.21	-1.620	0.012381
A_44_P382585	XM_220453	XM_220453		-0.21	-1.620	0.029481
A_44_P149842	XM_346273	XM_346273		-0.21	-1.620	0.030504
A_44_P367431	BC086414	BC086414		-0.21	-1.620	0.004043
A_44_P917020	TC566361	TC566361		-0.21	-1.620	0.004156
A_44_P706560	CA509728	CA509728		-0.21	-1.620	0.039704
A_44_P533740	Nphs1	NM_022628	64563	-0.21	-1.621	0.088043
A_44_P239162	Dot1l_predicted	XM_343159		-0.21	-1.621	0.146687
A_44_P1005524	Il2rg	AY325195	140924	-0.21	-1.621	0.013162
A_44_P354124	LOC682926	XM_001060811		-0.21	-1.621	0.003728
A_44_P178408	RGD1308195_predicted	XM_235419	315074	-0.21	-1.621	0.072087
A_44_P321622	RGD1311484	NM_001009701	360502	-0.21	-1.621	0.033001
A_42_P745958	Atrn	NM_031351	83526	-0.21	-1.621	0.003833
A_44_P946164	RGD1561517_predicted	XM_575271	499927	-0.21	-1.621	0.03905
A_44_P288187	Itpr3	NM_013138	25679	-0.21	-1.621	0.025459
A_44_P228995	Akap7	NM_001001801	361458	-0.21	-1.621	0.032576
A_44_P363143	Grm6	AJ245718	24419	-0.21	-1.622	0.019608

A_44_P852146	A_44_P852146	A_44_P852146		-0.21	-1.622	0.007292
A_44_P276435	Nat1	NM_053853	116631	-0.21	-1.622	0.018079
A_44_P250094	Olr831_predicted	NM_001000899	405209	-0.21	-1.622	0.019169
A_44_P468540	Mill1	NM_001011931	292671	-0.21	-1.622	0.285348
A_42_P780039	Ntrk1	NM_021589	59109	-0.21	-1.622	0.046856
A_44_P147055	Gjb1	NM_017251	29584	-0.21	-1.623	0.026055
A_44_P447850	LOC688997	XM_001069111		-0.21	-1.623	0.280058
A_44_P178400	XM_236459	XM_236459		-0.21	-1.623	0.008897
A_44_P699561	TC555342	TC555342		-0.21	-1.624	0.004781
A_43_P19525	Arhgap12_predicted	XM_225439		-0.21	-1.624	0.018357
A_43_P18011	Stam2	NM_001012085	311030	-0.21	-1.624	0.001434
A_43_P12915	Bcl2l10	NM_053733	114552	-0.21	-1.624	0.040938
A_44_P194274	Dio1	NM_021653	25430	-0.21	-1.624	0.083951
A_44_P291088	Mon2	XM_001054316	314894	-0.21	-1.624	0.007354
A_44_P293124	Tasp1_predicted	XM_230631		-0.21	-1.624	0.00368
A_44_P158899	Gpd2	NM_012736	25062	-0.21	-1.625	0.005163
A_44_P426041	Stk22s1	NM_199088	292890	-0.21	-1.625	0.008156
A_42_P783025	AW915739	AW915739	500034	-0.21	-1.625	0.016577
A_42_P779282	Scn3b	NM_139097	245956	-0.21	-1.625	0.073117
A_44_P164001	A_44_P164001	A_44_P164001		-0.21	-1.625	0.026432
A_44_P441001	LOC363437	NM_001024901	363437	-0.21	-1.625	0.019164
A_44_P960020	TC527735	TC527735		-0.21	-1.626	0.021349
A_44_P946867	TC527418	TC527418		-0.21	-1.626	0.020344
A_44_P130880	Usp53_predicted	XM_215700		-0.21	-1.626	0.015773
A_42_P720964	LOC303515	XM_239336	303515	-0.21	-1.626	0.013983
A_44_P601939	AI549199	AI549199		-0.21	-1.626	0.055482
A_43_P12570	Il3	NM_031513	24495	-0.21	-1.626	0.017952
A_44_P419206	LOC680104	XM_001055744		-0.21	-1.627	0.005999
A_44_P177590	Olr1337_predicted	NM_001000480	300637	-0.21	-1.627	0.074843
A_44_P342788	Wnt10a_predicted	XM_237296		-0.21	-1.627	0.018292
A_44_P349689	Spg3a	NM_001009831	362750	-0.21	-1.627	0.024885
A_44_P681872	AW141628	AW141628		-0.21	-1.627	0.018108
A_44_P100283	XM_346203	XM_346203		-0.21	-1.627	0.058126
A_44_P261606	AI180339	AI180339	297893	-0.21	-1.627	0.071579
A_44_P1034640	DV727166	DV727166	363171	-0.21	-1.627	0.002594
A_44_P348286	S74324	S74324		-0.21	-1.627	0.173133
A_44_P1025411	LOC498451	XM_001068016		-0.21	-1.627	0.004715
A_44_P602689	AW917134	AW917134		-0.21	-1.627	0.04029
A_42_P560984	RGD1565641_predicted	XM_574891		-0.21	-1.628	0.002428
A_44_P377011	Olr481_predicted	NM_001000684	404889	-0.21	-1.628	0.008461
A_44_P369965	Olr1295_predicted	NM_001000598	367065	-0.21	-1.629	0.029896
A_44_P972936	AI013918	AI013918	245966	-0.21	-1.629	0.006938
A_42_P504926	BX883044	BX883044		-0.21	-1.629	0.001455
A_44_P242664	AA859484	AA859484	29676	-0.21	-1.629	0.018608
A_44_P775461	LOC688553	XM_001067393	688553	-0.21	-1.629	0.0182
A_44_P383349	AW920888	AW920888		-0.21	-1.629	0.011111
A_44_P695589	DV724285	DV724285		-0.21	-1.629	0.04548
A_44_P349113	A_44_P349113	A_44_P349113		-0.21	-1.629	0.060314
A_44_P157030	LOC316493	XM_001078812		-0.21	-1.629	0.18613
A_44_P830181	TC545512	TC545512		-0.21	-1.629	0.003155
A_44_P883793	TC552432	TC552432		-0.21	-1.629	0.073411
A_44_P105366	Slc25a5	NM_057102	25176	-0.21	-1.629	0.006888
A_44_P136059	BE104359	BE104359		-0.21	-1.630	0.104995
A_43_P13237	Gpha2	NM_133619	171158	-0.21	-1.630	0.040751
A_44_P312922	BF285225	BF285225	690109	-0.21	-1.630	0.012003
A_44_P551065	Ly6a_predicted	XM_343263	362935	-0.21	-1.630	0.094626
A_44_P193188	Il4	NM_201270	287287	-0.21	-1.630	0.008399
A_44_P165817	NP798075	NP798075		-0.21	-1.630	0.051962
A_44_P463996	Inpp5a_predicted	XM_344971		-0.21	-1.630	0.007578
A_44_P698144	RGD1566222_predicted	XM_574352	499071	-0.21	-1.630	0.014007
A_44_P165643	LOC314251	NM_212522	314251	-0.21	-1.630	0.015208
A_44_P517762	Pttg1ip	NM_001013238	365548	-0.21	-1.631	0.00668
A_44_P363525	A_44_P363525	A_44_P363525		-0.21	-1.631	0.085371

A_44_P755377	TC551035	TC551035		-0.21	-1.631	0.065049
A_44_P321212	LOC306346	XM_224724		-0.21	-1.631	0.018319
A_44_P205980	Sult2b1_predicted	NM_001039665	292915	-0.21	-1.631	0.026684
A_44_P512916	Ns5atp4	NM_207607	311934	-0.21	-1.631	0.002274
A_44_P142944	AA925384	AA925384		-0.21	-1.631	0.013758
A_44_P128079	Olr1557_predicted	NM_001000050	288190	-0.21	-1.632	0.007691
A_44_P377050	Olr1688_predicted	NM_001000275	294164	-0.21	-1.632	0.085469
A_43_P20375	RGD1306739_predicted	XM_342127	361834	-0.21	-1.632	0.045924
A_44_P336584	AA875598	AA875598	361258	-0.21	-1.633	0.002051
A_44_P165820	Sdha	NM_130428	157074	-0.21	-1.633	0.000741
A_44_P428146	Pde4dip	NM_022382	64183	-0.21	-1.633	0.006952
A_42_P608780	Gdf15	NM_019216	29455	-0.21	-1.633	0.006653
A_44_P309559	RGD1563049_predicted	XM_238409	298493	-0.21	-1.633	0.086936
A_44_P138098	RGD1564981_predicted	XR_008599	366303	-0.21	-1.633	0.001318
A_44_P464314	RGD1562030_predicted	XM_219729		-0.21	-1.633	0.022137
A_44_P124134	LOC681488	XM_001057065		-0.21	-1.633	0.073024
A_44_P974313	Ncor1	XM_577103	54299	-0.21	-1.633	0.006147
A_44_P213214	Npepl1_predicted	XM_230892		-0.21	-1.633	0.002736
A_42_P504523	Wrnip1	NM_172332	282835	-0.21	-1.634	0.078621
A_44_P772908	BF558676	BF558676		-0.21	-1.634	0.002678
A_44_P674198	LOC680216	XM_001057422		-0.21	-1.634	0.178159
A_44_P671768	RGD1309655	NM_001013862	287766	-0.21	-1.634	0.052477
A_44_P1003623	AI113137	AI113137		-0.21	-1.634	0.008438
A_42_P490612	Zc3h7a_predicted	XM_340742		-0.21	-1.634	0.002744
A_44_P115030	BQ206691	BQ206691	362831	-0.21	-1.634	0.012379
A_44_P233091	AA874832	AA874832	288671	-0.21	-1.634	0.002193
A_44_P387086	Hoxa9_predicted	XM_001057018		-0.21	-1.634	0.011587
A_43_P12207	Ddah1	NM_022297	64157	-0.21	-1.634	0.055082
A_44_P473058	Hbe1_predicted	NM_001008890	293267	-0.21	-1.635	0.021335
A_44_P121280	Kcne1	NM_012973	25471	-0.21	-1.635	0.009866
A_44_P563522	TC548593	TC548593		-0.21	-1.635	0.021272
A_44_P513444	A_44_P513444	A_44_P513444		-0.21	-1.635	0.03451
A_44_P148522	AA925792	AA925792	24786	-0.21	-1.635	0.019344
A_44_P278418	Cyp11b2	NM_012538	24294	-0.21	-1.635	0.048238
A_44_P1042940	TC537424	TC537424		-0.21	-1.636	0.044194
A_44_P1055082	Dyrk2_predicted	XM_235179		-0.21	-1.636	0.003364
A_44_P162661	BG378875	BG378875	310704	-0.21	-1.636	0.073013
A_44_P229589	RGD1564830_predicted	XM_226359		-0.21	-1.636	0.105365
A_44_P479372	BG372885	BG372885	292688	-0.21	-1.636	0.001567
A_44_P933398	TC528713	TC528713		-0.21	-1.636	0.04378
A_44_P396772	Lrrn1	NM_001037363	500280	-0.21	-1.636	0.130018
A_44_P390172	RT1-Aw2	AJ004888	24737	-0.21	-1.636	0.001647
A_44_P477633	Ncstn	NM_174864	289231	-0.21	-1.636	0.011129
A_44_P351263	Ly6g6f	NM_001003691	309609	-0.21	-1.637	0.007782
A_42_P500675	Hcrt	NM_013179	25723	-0.21	-1.637	0.022556
A_44_P243477	LOC681047	XM_001060083		-0.21	-1.637	0.008827
A_44_P1059229	TC539775	TC539775		-0.21	-1.637	0.005093
A_42_P532458	Papss1_predicted	XM_215701		-0.21	-1.637	0.003019
A_44_P144336	Olr176_predicted	NM_001000178	293321	-0.21	-1.638	0.064584
A_44_P196569	LOC316632	NM_182671	314071	-0.21	-1.638	0.005958
A_44_P537095	A_44_P537095	A_44_P537095		-0.21	-1.638	0.004027
A_44_P1048404	Rab8a	BC105863	117103	-0.21	-1.638	0.004197
A_44_P784011	TC530727	TC530727		-0.21	-1.638	0.166768
A_44_P400850	A_44_P400850	A_44_P400850		-0.21	-1.638	0.021067
A_44_P208733	RGD1565911_predicted	XM_233160		-0.21	-1.638	0.094126
A_44_P327204	BI285141	BI285141	25145	-0.21	-1.638	0.080851
A_44_P149743	Sema6a_predicted	XM_341612		-0.21	-1.638	0.043861
A_44_P268192	AI045896	AI045896		-0.21	-1.639	0.004544
A_44_P913633	LOC686013	XM_001066168	686013	-0.21	-1.639	0.00605
A_44_P411912	Ceacam10	NM_173339	287009	-0.21	-1.639	0.057404
A_44_P532038	Pycs_predicted	XM_342048		-0.21	-1.639	0.014484
A_44_P431600	XM_234646	XM_234646		-0.21	-1.639	0.113914
A_44_P204405	BE106971	BE106971	315117	-0.21	-1.640	0.028532

A_44_P985259	A_44_P985259	A_44_P985259		-0.21	-1.640	0.014728
A_44_P506956	BQ208586	BQ208586	500545	-0.21	-1.640	0.004741
A_44_P759788	LOC498796	NM_001025034	498796	-0.21	-1.640	0.007353
A_44_P757948	LOC309362	XM_219860	309362	-0.22	-1.641	0.019593
A_44_P448740	Omp	NM_012616	24612	-0.22	-1.641	0.007598
A_44_P498959	XM_341732	XM_341732		-0.22	-1.641	0.107469
A_44_P156895	Sall3_predicted	XM_344708		-0.22	-1.641	0.002474
A_44_P748252	TC563773	TC563773		-0.22	-1.641	0.04417
A_43_P11689	Ppp1cb	NM_013065	25594	-0.22	-1.641	0.017582
A_43_P16650	XM_214107	XM_214107		-0.22	-1.641	0.033275
A_43_P13256	Dcxr	NM_134387	171408	-0.22	-1.642	0.005024
A_44_P869241	TC540417	TC540417		-0.22	-1.642	0.014504
A_44_P999838	RGD1565079_predicted	XM_236191		-0.22	-1.642	0.023137
A_44_P532133	XM_342323	XM_342323		-0.22	-1.642	0.040472
A_44_P874982	A_44_P874982	A_44_P874982		-0.22	-1.642	0.024997
A_44_P523114	LOC690280	XM_001073950		-0.22	-1.643	0.295499
A_44_P702404	TC520424	TC520424		-0.22	-1.643	0.003032
A_44_P479008	AW523545	AW523545	311462	-0.22	-1.643	0.015782
A_44_P398132	Slc22a12	NM_001034943	365398	-0.22	-1.643	0.0448
A_44_P192595	XM_220947	XM_220947		-0.22	-1.643	0.006229
A_44_P1047259	Ppp1r12a	NM_053890	116670	-0.22	-1.643	0.002913
A_44_P335125	Grpr	NM_012706	24938	-0.22	-1.644	0.101494
A_44_P186919	Olr546_predicted	NM_001001054	405942	-0.22	-1.644	0.024394
A_44_P863933	LOC687813	XM_001080233		-0.22	-1.644	0.001019
A_44_P506221	Neurl_predicted	XM_219970		-0.22	-1.644	0.002131
A_44_P156873	Zfp533_predicted	XM_230026		-0.22	-1.644	0.078381
A_44_P443611	AA859455	AA859455	293615	-0.22	-1.644	0.036545
A_44_P837602	TC560388	TC560388		-0.22	-1.644	0.03572
A_42_P500847	Dhrs7c_predicted	XM_213343	287411	-0.22	-1.644	0.080155
A_44_P154554	XM_232950	XM_232950		-0.22	-1.644	0.070284
A_43_P13226	Slc31a1	NM_133600	171135	-0.22	-1.645	0.00486
A_44_P192406	Dusp9	NM_001037973	293847	-0.22	-1.645	0.043326
A_44_P152183	XM_231797	XM_231797		-0.22	-1.645	0.003362
A_44_P338813	AI103956	AI103956	79250	-0.22	-1.645	0.005709
A_44_P788899	RGD1305755	NM_001039336	305923	-0.22	-1.645	0.00342
A_44_P389508	XM_342902	XM_342902		-0.22	-1.645	0.045899
A_44_P820495	AA859324	AA859324		-0.22	-1.646	0.079584
A_44_P973511	Lrp11_predicted	XM_238072		-0.22	-1.646	0.026212
A_44_P328016	Rxrg	NM_031765	83574	-0.22	-1.646	0.009728
A_44_P440627	Myo1e	NM_173101	25484	-0.22	-1.646	0.001979
A_44_P157690	Snrpn	NM_031117	81781	-0.22	-1.646	0.007141
A_44_P582377	A_44_P582377	A_44_P582377		-0.22	-1.647	0.059158
A_44_P482859	Sema5a_predicted	XM_241275		-0.22	-1.647	0.033839
A_44_P332668	Lnpep	NM_133574	171105	-0.22	-1.647	0.003871
A_44_P494196	S79307	S79307		-0.22	-1.647	0.12857
A_44_P592538	DN934259	DN934259		-0.22	-1.647	0.054401
A_44_P439531	AI045937	AI045937	65204	-0.22	-1.647	0.031506
A_44_P182488	XM_345745	XM_345745		-0.22	-1.647	0.210736
A_44_P666872	DY311851	DY311851	303812	-0.22	-1.647	0.173313
A_44_P1000560	RGD1305781	XM_235378	315037	-0.22	-1.647	0.07474
A_44_P841672	TC544349	TC544349		-0.22	-1.648	0.012702
A_44_P536867	LOC678820	XM_001053303		-0.22	-1.648	0.046468
A_42_P665509	Mapk8ip	NM_053777	116457	-0.22	-1.648	0.009765
A_44_P959259	LOC689285	XM_001070258		-0.22	-1.648	0.039298
A_44_P446771	AI229455	AI229455	309177	-0.22	-1.648	0.034723
A_44_P1009001	Adcy9_predicted	XM_220178		-0.22	-1.648	0.001413
A_44_P959710	LOC300506	XR_005718		-0.22	-1.648	0.06716
A_44_P974137	AI716115	AI716115	309158	-0.22	-1.648	0.00616
A_44_P165648	Olr1749_predicted	NM_001001429	294203	-0.22	-1.648	0.023623
A_44_P423195	BQ210837	BQ210837	364974	-0.22	-1.648	0.015558
A_44_P187906	BQ202988	BQ202988		-0.22	-1.648	0.014353
A_44_P451071	Prkcd	NM_133307	170538	-0.22	-1.649	0.006626
A_44_P321157	LOC688634	XM_001067699	688634	-0.22	-1.649	0.017245

A_44_P151037	AI044707	AI044707	24494	-0.22	-1.650	0.002009
A_43_P19928	BF565121	BF565121	309209	-0.22	-1.650	0.027955
A_43_P13259	Efha1	NM_134396	171433	-0.22	-1.650	0.022733
A_43_P12262	Inhbc	NM_022614	64549	-0.22	-1.650	0.018759
A_44_P161312	RGD1565384_predicted	XM_222667	304823	-0.22	-1.650	0.027451
A_44_P496331	Olr1254_predicted	NM_001001085	405976	-0.22	-1.650	0.013229
A_44_P331832	AI176595	AI176595	25697	-0.22	-1.650	0.151512
A_44_P334847	G6b	NM_001003976	406865	-0.22	-1.650	0.011217
A_44_P721930	BF405335	BF405335		-0.22	-1.651	0.063641
A_44_P503745	Gpr139	NM_001024241	293545	-0.22	-1.651	0.023891
A_44_P459760	RGD1560778_predicted	XM_236630		-0.22	-1.651	0.007472
A_44_P522889	A_44_P522889	A_44_P522889		-0.22	-1.651	0.002334
A_44_P327882	Ppp1r14d	NM_172011	259226	-0.22	-1.651	0.019302
A_43_P23459	XM_235679	XM_235679		-0.22	-1.651	0.173339
A_42_P572123	Fxyd1	NM_031648	58971	-0.22	-1.652	0.208703
A_44_P200728	AW919056	AW919056	299201	-0.22	-1.652	0.013314
A_44_P654145	Zfp365	NM_001025145	499425	-0.22	-1.652	0.259032
A_43_P18398	Nmnat2_predicted	XM_001071224		-0.22	-1.652	0.039817
A_42_P651991	Atp2a1	NM_058213	116601	-0.22	-1.652	0.020968
A_43_P19094	Elovl7_predicted	XM_342192	361895	-0.22	-1.652	0.018446
A_43_P16715	Sertad2	NM_001024903	498423	-0.22	-1.653	0.00131
A_44_P560687	TC551845	TC551845		-0.22	-1.653	0.001053
A_44_P234473	LOC679253	XM_001057693		-0.22	-1.653	0.025604
A_44_P837067	LOC686985	XM_001076617		-0.22	-1.653	0.085193
A_44_P110271	XM_345652	XM_345652		-0.22	-1.653	0.030071
A_44_P960350	DV716763	DV716763		-0.22	-1.653	0.008936
A_44_P146147	BI274245	BI274245	304653	-0.22	-1.653	0.010448
A_44_P432035	AW531000	AW531000	307859	-0.22	-1.654	0.009069
A_42_P774912	C4bpb	NM_016995	24236	-0.22	-1.654	0.101667
A_44_P137605	ENSRNOT00000019378	ENSRNOT00000019378		-0.22	-1.654	0.006152
A_44_P344749	XM_225257	XM_225257		-0.22	-1.654	0.004387
A_44_P1070020	A_44_P1070020	A_44_P1070020		-0.22	-1.654	0.033841
A_44_P657006	TC560495	TC560495		-0.22	-1.654	0.023367
A_44_P881169	BE329329	BE329329	298836	-0.22	-1.655	0.018229
A_44_P355306	AI639488	AI639488	314856	-0.22	-1.655	0.019909
A_44_P342739	Ribc2	NM_001013949	300122	-0.22	-1.655	0.026601
A_42_P546708	Khdrbs3	NM_022249	64015	-0.22	-1.655	0.147549
A_44_P114650	XM_228601	XM_228601		-0.22	-1.655	0.066493
A_44_P523198	Akap8l	NM_001013946	299569	-0.22	-1.655	0.00369
A_44_P506413	XM_227949	XM_227949		-0.22	-1.655	0.045899
A_44_P328386	XM_224105	XM_224105		-0.22	-1.655	0.046365
A_44_P396454	LOC501039	XM_576451	501039	-0.22	-1.655	0.011515
A_44_P480222	Mapk9	NM_017322	50658	-0.22	-1.655	0.009179
A_44_P745811	CX569439	CX569439		-0.22	-1.655	0.012287
A_44_P614028	AI406967	AI406967		-0.22	-1.656	0.03631
A_44_P169574	AI045802	AI045802	291132	-0.22	-1.656	0.004457
A_44_P274303	A_44_P274303	A_44_P274303		-0.22	-1.656	0.067512
A_44_P151759	Cdig2	AB086193	266732	-0.22	-1.656	0.000655
A_44_P311786	A_44_P311786	A_44_P311786		-0.22	-1.656	0.03759
A_44_P901521	TC547124	TC547124		-0.22	-1.656	0.024659
A_44_P540785	Olr326_predicted	NM_001000248	293764	-0.22	-1.656	0.052055
A_44_P458999	Hey2	XM_344806	155430	-0.22	-1.656	0.046334
A_44_P942286	BM386384	BM386384	89825	-0.22	-1.656	0.021668
A_44_P480463	XM_344365	XM_344365		-0.22	-1.657	0.00724
A_44_P522891	LOC498480	XM_573738		-0.22	-1.657	0.216964
A_44_P639923	TC564397	TC564397		-0.22	-1.657	0.03328
A_44_P115774	CK481403	CK481403	362246	-0.22	-1.657	0.017023
A_44_P285709	Coro1b	NM_019222	29474	-0.22	-1.657	0.001942
A_44_P231418	Atrn	NM_031351	83526	-0.22	-1.657	0.004572
A_43_P22629	LOC686680	XM_001075247		-0.22	-1.657	0.014161
A_43_P15478	Nr5a1	XM_001054966		-0.22	-1.657	0.089276
A_44_P684783	TC553545	TC553545		-0.22	-1.657	0.077979
A_44_P324845	AW142990	AW142990		-0.22	-1.657	0.011423

A_44_P638119	TC556752	TC556752		-0.22	-1.658	0.005429
A_44_P499608	RGD1561080_predicted	XM_340895	360620	-0.22	-1.658	0.042681
A_44_P276714	Ankrd10	XM_341466	361183	-0.22	-1.658	0.00951
A_44_P201633	A_44_P201633	A_44_P201633		-0.22	-1.658	0.02948
A_44_P469713	BQ189960	BQ189960	309140	-0.22	-1.658	0.013665
A_44_P183249	AA955357	AA955357		-0.22	-1.659	0.010566
A_44_P299479	Gpr176	XM_342493	117257	-0.22	-1.659	0.286599
A_44_P902511	CA339611	CA339611		-0.22	-1.659	0.013416
A_43_P16137	Sema3d	XM_342625		-0.22	-1.659	0.089927
A_44_P555843	LOC300274	XR_006492	300274	-0.22	-1.659	0.038004
A_43_P16071	Tnr	AF049655	25567	-0.22	-1.659	0.044203
A_44_P1016085	Zcsl3_predicted	XM_342483	362184	-0.22	-1.659	0.106029
A_44_P182082	Eif5b	XM_001057637		-0.22	-1.659	0.003833
A_44_P505968	ENSRNOT00000022060	ENSRNOT00000022060		-0.22	-1.659	0.020588
A_44_P365642	LOC690603	XM_001074943		-0.22	-1.659	0.010937
A_44_P760330	A_44_P760330	A_44_P760330		-0.22	-1.660	0.005698
A_42_P591475	CO393605	CO393605		-0.22	-1.660	0.001477
A_43_P21987	Sp2_mapped	XM_220916		-0.22	-1.660	0.027282
A_44_P315886	Magi3	NM_139084	245903	-0.22	-1.660	0.019611
A_44_P273885	MGC72974	NM_198772	316976	-0.22	-1.660	0.122734
A_44_P381969	Esrrb	NM_001008516	299210	-0.22	-1.661	0.035445
A_42_P834147	MGC112899	NM_001037795	500087	-0.22	-1.661	0.00597
A_44_P421163	BM986434	BM986434	24191	-0.22	-1.661	0.013502
A_44_P268441	Alg5	NM_001025407	295051	-0.22	-1.661	0.004042
A_44_P155773	Vamp4_predicted	XM_344168		-0.22	-1.661	0.001821
A_44_P974035	BI278180	BI278180		-0.22	-1.661	0.010955
A_44_P292587	Olr1454_predicted	NM_001000020	287343	-0.22	-1.662	0.037073
A_44_P576858	TC557397	TC557397		-0.22	-1.662	0.134754
A_44_P297208	L21001	L21001		-0.22	-1.662	0.046873
A_44_P623694	LOC679058	XM_001054509		-0.22	-1.662	0.011519
A_44_P473377	RGD1311667	NM_001029913	291165	-0.22	-1.663	0.035858
A_43_P16225	J01879	J01879		-0.22	-1.663	0.041584
A_44_P731679	TC562426	TC562426		-0.22	-1.663	0.045511
A_42_P718022	Tnni2	NM_017185	29389	-0.22	-1.664	0.013076
A_43_P21731	Grlf1_predicted	XM_001053554	306400	-0.22	-1.664	0.003649
A_44_P717825	TC542591	TC542591		-0.22	-1.664	0.049312
A_44_P114500	LOC365960	NM_001037210	365960	-0.22	-1.664	0.018151
A_44_P217295	Kcna2	NM_012970	25468	-0.22	-1.664	0.019734
A_44_P518003	RGD1564833_predicted	XM_001060344		-0.22	-1.664	0.000537
A_42_P769921	AW917548	AW917548		-0.22	-1.665	0.017096
A_44_P113141	RGD1561742_predicted	XM_341377	361092	-0.22	-1.665	0.007056
A_44_P118816	Lnpep	NM_133574	171105	-0.22	-1.665	0.000592
A_44_P522274	AA799471	AA799471		-0.22	-1.665	0.017541
A_44_P1001914	LOC304743	NM_001024997	304743	-0.22	-1.665	0.001771
A_44_P805357	Ncln	NM_001014082	314648	-0.22	-1.666	0.045965
A_44_P806532	LOC500442	NM_001024342	500442	-0.22	-1.666	0.006154
A_44_P475602	A_44_P475602	A_44_P475602		-0.22	-1.666	0.055228
A_44_P638654	Ihh	XM_001060415		-0.22	-1.668	0.004142
A_44_P525181	LOC679695	XM_001054075		-0.22	-1.668	0.177677
A_44_P560176	A_44_P560176	A_44_P560176		-0.22	-1.668	0.069407
A_44_P471353	A_44_P471353	A_44_P471353		-0.22	-1.668	0.002378
A_44_P448234	LOC367113	NM_001014268	367113	-0.22	-1.668	0.016634
A_44_P606049	ENSRNOT00000020846	ENSRNOT00000020846		-0.22	-1.668	0.006277
A_44_P746808	TC558906	TC558906		-0.22	-1.668	0.002818
A_44_P195080	CB546795	CB546795	499800	-0.22	-1.668	0.001481
A_44_P336798	AI236754	AI236754	171493	-0.22	-1.668	0.010498
A_44_P578452	TC544981	TC544981		-0.22	-1.669	0.00468
A_44_P481915	Id4	NM_175582	291023	-0.22	-1.669	0.020036
A_44_P533028	RGD1310262_predicted	XM_222453		-0.22	-1.669	0.022529
A_44_P880302	AW916124	AW916124		-0.22	-1.669	0.01819
A_42_P830311	Mrap_predicted	XM_213659	288271	-0.22	-1.669	0.028191
A_44_P686638	TC568507	TC568507		-0.22	-1.669	0.091354
A_44_P269544	Mgst3_predicted	XM_213943	289197	-0.22	-1.669	0.004752

A_44_P268999	BE108148	BE108148	25578	-0.22	-1.670	0.049451
A_44_P338174	BI289351	BI289351	315994	-0.22	-1.670	0.022519
A_44_P117015	Ndufb2_predicted	XM_342664		-0.22	-1.670	0.000762
A_44_P408347	BE109513	BE109513	680717	-0.22	-1.670	0.021426
A_44_P147199	LOC498464	XM_573721	498464	-0.22	-1.670	0.201932
A_44_P121215	Dnm1l	AF107048	114114	-0.22	-1.670	0.010044
A_44_P168917	Zfp206_predicted	XM_220198		-0.22	-1.670	0.010541
A_44_P682891	Nuak2	NM_001007617	289419	-0.22	-1.670	0.000811
A_43_P18922	Sema5a_predicted	XM_241275		-0.22	-1.670	0.072035
A_44_P486477	AA818699	AA818699	306628	-0.22	-1.671	0.045826
A_43_P12844	Cox17	NM_053540	89786	-0.22	-1.671	0.000852
A_44_P128186	Tas1r1	XM_342986		-0.22	-1.671	0.009233
A_42_P653827	Map2k2	NM_133283	58960	-0.22	-1.671	0.001201
A_42_P817228	Slc22a18	NM_001004260	309131	-0.22	-1.671	0.006083
A_42_P622913	Psenen	NM_001008764	292788	-0.22	-1.671	0.002873
A_43_P20900	RGD1562952_predicted	XM_001072232		-0.22	-1.671	0.007379
A_44_P230799	BI282204	BI282204	360664	-0.22	-1.672	0.011107
A_44_P510102	BQ783165	BQ783165	679944	-0.22	-1.672	0.01406
A_44_P370385	Dnajc15_predicted	XM_214238		-0.22	-1.672	0.001931
A_44_P169327	LOC684538	XM_001070886		-0.22	-1.672	0.004378
A_44_P598999	A_44_P598999	A_44_P598999		-0.22	-1.672	0.022244
A_44_P349089	Ifna1	NM_001014786	298210	-0.22	-1.672	0.014907
A_42_P797805	Slc22a6	NM_017224	29509	-0.22	-1.673	0.020315
A_44_P136902	BF416970	BF416970	171339	-0.22	-1.673	0.022721
A_44_P149056	Prm3	NM_001002855	442921	-0.22	-1.673	0.055596
A_44_P206352	A_44_P206352	A_44_P206352		-0.22	-1.674	0.076054
A_44_P315895	Slc14a2	NM_177962	54302	-0.22	-1.674	0.028872
A_44_P163369	Artn	NM_053397	362572	-0.22	-1.674	0.025114
A_42_P839121	Ece1	NM_053596	94204	-0.22	-1.674	0.007737
A_43_P15113	Myom1	XM_237523	316740	-0.22	-1.674	0.03688
A_44_P266454	MGC94464	NM_001007647	297337	-0.22	-1.675	0.000732
A_44_P474867	Sftpc	BU946660	50683	-0.22	-1.675	0.02739
A_44_P268837	LOC296318	NM_001013923	296318	-0.22	-1.675	0.033319
A_44_P194068	Olr880_predicted	NM_001000055	288790	-0.22	-1.675	0.069927
A_44_P970110	AW253396	AW253396		-0.22	-1.675	0.00343
A_44_P370694	Dmbx1_predicted	XM_233404		-0.22	-1.675	0.008314
A_44_P487950	AA924736	AA924736		-0.22	-1.675	0.006621
A_43_P14971	Pxmp2	NM_031587	29533	-0.22	-1.676	0.006374
A_44_P960581	TC522415	TC522415		-0.22	-1.676	0.030995
A_43_P19594	Ror2_predicted	XM_225181		-0.22	-1.676	0.013058
A_44_P540389	AI009605	AI009605	26954	-0.22	-1.676	0.084504
A_44_P863419	CK839576	CK839576		-0.22	-1.676	0.00103
A_44_P130713	LOC691041	XM_001076631		-0.22	-1.676	0.14691
A_44_P699502	TC538378	TC538378		-0.22	-1.676	0.001505
A_44_P463420	BQ210920	BQ210920	360854	-0.22	-1.676	0.022273
A_44_P549272	BF396818	BF396818	83571	-0.22	-1.676	0.008004
A_44_P358492	Fdft1	NM_019238	29580	-0.22	-1.676	0.00872
A_43_P19633	Nkx2-2_predicted	XM_345446	366214	-0.22	-1.676	0.003809
A_44_P103337	RGD1307851	NM_001013861	287741	-0.22	-1.676	0.094096
A_43_P14931	Nfe2l2	NM_031789	83619	-0.22	-1.676	0.001174
A_44_P535568	Gpc3	NM_012774	25236	-0.22	-1.677	0.024152
A_42_P594699	Zdhhc7	NM_133394	170906	-0.22	-1.677	0.004807
A_44_P260396	Alkbh3	NM_001014180	362169	-0.22	-1.677	0.001544
A_44_P245474	Hal	BC089809	29301	-0.22	-1.677	0.000743
A_44_P451568	Myo1e	NM_173101	25484	-0.22	-1.677	0.003021
A_43_P19505	Hrbl_predicted	XM_222017		-0.22	-1.678	0.007582
A_44_P858453	BP477365	BP477365		-0.22	-1.678	0.011643
A_44_P245636	Strbp	NM_053416	84476	-0.22	-1.678	0.017314
A_44_P575605	A_44_P575605	A_44_P575605		-0.22	-1.678	0.00269
A_44_P748217	RGD1559545_predicted	XM_578549		-0.22	-1.678	0.001833
A_43_P11574	St8sia1	NM_012813	25280	-0.22	-1.678	0.007263
A_44_P289941	AA925160	AA925160	363136	-0.22	-1.679	0.001477
A_43_P22058	Lin28_predicted	XM_001067034		-0.23	-1.679	0.002593

A_44_P661776	A_44_P661776	A_44_P661776		-0.23	-1.679	0.026196
A_44_P285072	AA955151	AA955151	246298	-0.23	-1.679	0.00098
A_43_P14175	Cycs	NM_012839	25309	-0.23	-1.679	0.001704
A_44_P537421	AA925378	AA925378		-0.23	-1.679	0.015022
A_44_P391382	A_44_P391382	A_44_P391382		-0.23	-1.679	0.099431
A_44_P902085	TC562616	TC562616		-0.23	-1.679	0.003225
A_44_P268864	Mgll	NM_138502	29254	-0.23	-1.679	0.047623
A_42_P558144	Prodh2	XM_341825	361538	-0.23	-1.679	0.042213
A_44_P1034201	Lamb3	XM_001069930		-0.23	-1.680	0.018928
A_44_P508823	RGD1563901_predicted	XM_230973	311716	-0.23	-1.680	0.018339
A_44_P124289	RGD1305664	XM_220588		-0.23	-1.680	0.034328
A_44_P960453	TC521512	TC521512		-0.23	-1.680	0.003232
A_44_P286252	RGD1304952	NM_001014059	312711	-0.23	-1.680	0.027616
A_44_P494391	XM_215087	XM_215087		-0.23	-1.680	0.045269
A_44_P403415	Mpped1_predicted	XM_343301	362971	-0.23	-1.680	0.027472
A_42_P779097	LOC680427	XM_001056898	680427	-0.23	-1.681	0.020938
A_44_P208617	XM_212972	XM_212972		-0.23	-1.681	0.044251
A_44_P715320	TC522881	TC522881		-0.23	-1.681	0.001996
A_44_P169222	AA956183	AA956183		-0.23	-1.681	0.002679
A_42_P525170	Abcd2	NM_033352	84356	-0.23	-1.682	0.075817
A_44_P496315	Otx1	NM_013109	25646	-0.23	-1.682	0.001696
A_44_P876244	AI229404	AI229404		-0.23	-1.682	0.041388
A_44_P484877	AF475093	AF475093	363061	-0.23	-1.682	0.00101
A_44_P352163	A_44_P352163	A_44_P352163		-0.23	-1.682	0.003411
A_44_P957259	CR754047	CR754047	680889	-0.23	-1.682	0.244955
A_44_P578301	TC527892	TC527892		-0.23	-1.682	0.076649
A_44_P457625	CA513011	CA513011	316258	-0.23	-1.683	0.001995
A_43_P18278	LOC500591	XM_001076183		-0.23	-1.683	0.007154
A_44_P401828	AI229655	AI229655		-0.23	-1.683	0.030793
A_44_P469079	XM_347116	XM_347116		-0.23	-1.684	0.021241
A_44_P194447	LOC685929	XM_001065801		-0.23	-1.684	0.24308
A_44_P250520	RGD1305412_predicted	XM_219390		-0.23	-1.684	0.00181
A_43_P16310	Gpr50	XM_346374	117097	-0.23	-1.684	0.010526
A_44_P606828	CF108700	CF108700		-0.23	-1.684	0.002181
A_44_P354310	LOC691589	XM_001078944	691589	-0.23	-1.684	0.002314
A_44_P149958	Ches1_predicted	XM_234377		-0.23	-1.685	0.000735
A_44_P392878	ENSRNOT00000040993	ENSRNOT00000040993		-0.23	-1.685	0.007684
A_44_P1070970	AY325228	AY325228		-0.23	-1.685	0.020986
A_44_P269797	LOC685105	XM_001062311		-0.23	-1.685	0.098167
A_44_P300137	Atp8b3_predicted	XM_234937	299616	-0.23	-1.685	0.006734
A_44_P136894	BI295768	BI295768	246233	-0.23	-1.685	0.052425
A_44_P396817	Prlh	NM_022222	63850	-0.23	-1.685	0.00369
A_44_P255201	Cant1	NM_144754	246272	-0.23	-1.685	0.003481
A_44_P705134	AI176136	AI176136	315037	-0.23	-1.685	0.001404
A_44_P913854	ENSRNOT00000046568	ENSRNOT00000046568		-0.23	-1.685	0.001402
A_44_P433721	Amt	NM_001014004	306586	-0.23	-1.686	0.037708
A_42_P629457	AA957193	AA957193		-0.23	-1.686	0.024759
A_44_P243901	Lrrc30_predicted	XM_237538		-0.23	-1.686	0.00806
A_43_P12541	Cib1	NM_031145	81823	-0.23	-1.686	0.002068
A_44_P289608	AI235842	AI235842	24787	-0.23	-1.686	0.041961
A_44_P205913	Gorasp1	NM_019385	56082	-0.23	-1.687	0.001873
A_44_P540460	AA942995	AA942995	298594	-0.23	-1.687	0.007226
A_44_P726025	AA964300	AA964300		-0.23	-1.687	0.019422
A_43_P20819	RGD1561038_predicted	XM_225450		-0.23	-1.687	0.030899
A_44_P555752	XM_233616	XM_233616		-0.23	-1.687	0.006023
A_43_P13896	CB606443	CB606443		-0.23	-1.687	0.009827
A_44_P501639	Man1a_predicted	NM_001033656	294410	-0.23	-1.687	0.006698
A_42_P547027	Fuca2	NM_001004218	292485	-0.23	-1.687	0.02024
A_44_P352521	BI288535	BI288535	301062	-0.23	-1.687	0.017603
A_44_P377164	S81002	S81002		-0.23	-1.687	0.004821
A_44_P137187	AB033713	AB033713		-0.23	-1.688	0.023615
A_44_P668786	A_44_P668786	A_44_P668786		-0.23	-1.688	0.00257
A_44_P199371	XM_237488	XM_237488		-0.23	-1.688	0.024691

A_44_P456913	RGD1565772_predicted	XM_218203	308341	-0.23	-1.688	0.027731
A_44_P563496	TC548420	TC548420		-0.23	-1.689	0.002287
A_43_P23188	LOC678766	XM_001053087		-0.23	-1.689	0.010055
A_42_P546008	Sftpd	NM_012878	25350	-0.23	-1.689	0.051114
A_44_P951025	A_44_P951025	A_44_P951025		-0.23	-1.689	0.003481
A_44_P282108	BI288131	BI288131		-0.23	-1.689	0.033795
A_44_P351837	RGD1561276_predicted	XM_575198		-0.23	-1.689	0.036822
A_42_P765648	Prpg2	NM_172065	257651	-0.23	-1.689	0.021423
A_43_P13334	Slc22a9	NM_138908	192273	-0.23	-1.689	0.015626
A_44_P311870	LOC500331	NM_001024337	500331	-0.23	-1.690	0.003359
A_43_P12986	Edg2	NM_053936	116744	-0.23	-1.690	0.001764
A_43_P18408	Slc35a3	NM_001012082	310808	-0.23	-1.690	0.004725
A_44_P100087	Olr876_predicted	NM_001000708	404957	-0.23	-1.690	0.024677
A_44_P468293	Olr115_predicted	NM_001000152	293253	-0.23	-1.691	0.02091
A_44_P308962	Scgb3a1	NM_001013180	338418	-0.23	-1.691	0.032833
A_44_P184865	Ptp4a1	NM_031579	29463	-0.23	-1.691	0.006224
A_44_P343615	Agtr2	NM_012494	24182	-0.23	-1.691	0.046241
A_44_P886305	TC563523	TC563523		-0.23	-1.691	0.11607
A_43_P13126	Acot12	NM_130747	170570	-0.23	-1.691	0.027243
A_43_P19867	RGD1562792_predicted	XM_214910	292813	-0.23	-1.692	0.005251
A_44_P380315	LOC363188	XM_001061775		-0.23	-1.692	0.002647
A_44_P440709	RGD1304620_predicted	XM_222799		-0.23	-1.692	0.013368
A_44_P426624	XM_344380	XM_344380		-0.23	-1.692	0.035636
A_44_P283071	AA859795	AA859795	303158	-0.23	-1.692	0.034601
A_44_P164068	LOC299907	XM_216920	299907	-0.23	-1.692	0.004796
A_44_P865695	AW525937	AW525937	301512	-0.23	-1.692	0.012259
A_44_P624643	TC547485	TC547485		-0.23	-1.692	0.007921
A_43_P21008	RGD1565016_predicted	XM_234928	314647	-0.23	-1.692	0.016896
A_44_P428922	XM_341979	XM_341979		-0.23	-1.692	0.002683
A_44_P580799	TC561516	TC561516		-0.23	-1.693	0.013701
A_44_P310581	Dok4_predicted	XM_341642		-0.23	-1.693	0.001159
A_44_P461705	XM_224118	XM_224118		-0.23	-1.693	0.056958
A_44_P400634	XM_344206	XM_344206		-0.23	-1.693	0.004763
A_44_P432112	BQ204663	BQ204663	685174	-0.23	-1.693	0.019304
A_44_P477047	Spna2	NM_171983	64159	-0.23	-1.693	0.001316
A_43_P13086	P2rx5	NM_080780	113995	-0.23	-1.693	0.021238
A_44_P398917	AA875457	AA875457		-0.23	-1.694	0.153242
A_44_P696363	LOC259224	BQ194351	259224	-0.23	-1.695	0.005573
A_44_P141621	AI030759	AI030759	363469	-0.23	-1.695	0.020125
A_44_P1004340	Sar1b	NM_001009622	287276	-0.23	-1.695	0.001595
A_44_P856400	LOC691909	XM_001080060	691909	-0.23	-1.695	0.008597
A_44_P306586	Hbe2_predicted	NM_001024805	502359	-0.23	-1.695	0.005437
A_44_P778186	TC561517	TC561517		-0.23	-1.695	0.16281
A_44_P956213	BI285090	BI285090	311545	-0.23	-1.695	0.003825
A_44_P1032538	Gng3	XM_001073457		-0.23	-1.695	0.02086
A_44_P537616	BE099608	BE099608		-0.23	-1.695	0.03811
A_44_P381523	Tax1bp1	NM_001004199	246244	-0.23	-1.695	0.00518
A_44_P393007	AA946313	AA946313	24791	-0.23	-1.695	0.011047
A_44_P211256	RGD1561343_predicted	XM_578186	502689	-0.23	-1.695	0.003682
A_44_P414860	XM_345276	XM_345276		-0.23	-1.696	0.008648
A_44_P275729	AA848404	AA848404	192351	-0.23	-1.696	0.028735
A_43_P13792	AA925776	AA925776		-0.23	-1.696	0.116918
A_44_P233712	Olr246_predicted	NM_001000216	293391	-0.23	-1.696	0.034625
A_43_P17925	Abca8b_predicted	XM_221074	303637	-0.23	-1.696	0.029886
A_44_P456599	Zdhhc23	NM_213627	363783	-0.23	-1.696	0.004787
A_42_P683840	Ccl27_predicted	XM_342823		-0.23	-1.697	0.026943
A_44_P289132	Spsb4_predicted	XM_236555		-0.23	-1.697	0.093759
A_44_P445429	A_44_P445429	A_44_P445429		-0.23	-1.697	0.003047
A_44_P149916	Zfp275_predicted	XM_219713		-0.23	-1.697	0.035487
A_44_P311988	RGD1304563_predicted	XM_001069093		-0.23	-1.698	0.000828
A_44_P250561	RGD1307158_predicted	XM_001072759		-0.23	-1.698	0.01032
A_43_P11879	Htr2b	NM_017250	29581	-0.23	-1.698	0.375273
A_44_P836794	RGD1559779_predicted	XM_573274	498073	-0.23	-1.698	0.009236

A_44_P534467	RGD1311678	AY539878	296311	-0.23	-1.698	0.024487
A_43_P18024	CB544885	CB544885	314730	-0.23	-1.699	0.020835
A_44_P486246	AW916189	AW916189		-0.23	-1.699	0.008012
A_44_P272504	BQ781691	BQ781691		-0.23	-1.699	0.050947
A_44_P760531	RGD1559864_predicted	XM_575808	500446	-0.23	-1.699	0.009373
A_44_P605678	RGD1559538_predicted	XM_573117	497930	-0.23	-1.699	0.00577
A_44_P388273	BQ194242	BQ194242	288689	-0.23	-1.699	0.002068
A_44_P241556	Adamts15_predicted	XM_235976		-0.23	-1.699	0.005399
A_44_P603427	BC104707	BC104707		-0.23	-1.700	0.014306
A_42_P828983	Mamdc1	NM_199269	314180	-0.23	-1.700	0.008088
A_44_P720233	TC556941	TC556941		-0.23	-1.700	0.008019
A_44_P659530	BM986371	BM986371		-0.23	-1.700	0.004339
A_44_P818459	XM_575152	XM_575152		-0.23	-1.700	0.019186
A_44_P269796	RGD1563510_predicted	XM_342547	362240	-0.23	-1.700	0.002711
A_44_P453676	AA944315	AA944315	297337	-0.23	-1.700	0.030688
A_44_P1004731	Ctss	NM_017320	50654	-0.23	-1.700	0.008093
A_44_P589185	AI145447	AI145447		-0.23	-1.700	0.001155
A_43_P11466	Fuca	NM_012562	24375	-0.23	-1.701	0.004542
A_44_P117285	XM_578795	XM_578795		-0.23	-1.701	0.025005
A_44_P290756	Mfap3l	NM_001012049	306424	-0.23	-1.701	0.006166
A_44_P202726	BF406960	BF406960	315160	-0.23	-1.701	0.003602
A_44_P154638	Zfyve21_predicted	XM_343115		-0.23	-1.701	0.018339
A_43_P15094	BF550406	BF550406		-0.23	-1.701	0.006392
A_42_P727231	Metrnl	NM_001014104	316842	-0.23	-1.702	0.001039
A_44_P174749	BF396509	BF396509	302969	-0.23	-1.702	0.017773
A_44_P206614	CA333998	CA333998		-0.23	-1.703	0.006066
A_44_P172866	LOC499062	XM_001075446	1E+08	-0.23	-1.703	0.007983
A_44_P444497	BI279854	BI279854	288785	-0.23	-1.703	0.019914
A_43_P15507	Indo	NM_023973	66029	-0.23	-1.704	0.048052
A_44_P134342	BF525012	BF525012	362279	-0.23	-1.704	0.023363
A_44_P316644	LOC683430	XM_001065910		-0.23	-1.704	0.023858
A_44_P491885	Z93351	Z93351		-0.23	-1.704	0.035451
A_44_P142640	A_44_P142640	A_44_P142640		-0.23	-1.704	0.026683
A_44_P553980	RGD1566064_predicted	XM_341141	360865	-0.23	-1.704	0.033983
A_44_P426989	RGD1307461_predicted	XM_236620		-0.23	-1.705	0.006723
A_44_P216235	Atp5i	NM_080481	140608	-0.23	-1.705	0.003629
A_44_P231556	Rgs16	XM_341134		-0.23	-1.705	0.052203
A_44_P340977	AABR03116353	AABR03116353		-0.23	-1.705	0.042541
A_42_P536702	Sftpc	NM_017342	50683	-0.23	-1.705	0.011717
A_42_P770330	LOC684934	XM_001062632		-0.23	-1.705	0.001053
A_42_P462469	Lrrc41	NM_001009710	362566	-0.23	-1.706	0.052458
A_43_P14817	Afp	NM_012493	24177	-0.23	-1.706	0.045572
A_44_P178417	RGD1563996_predicted	XM_235511	315163	-0.23	-1.706	0.015778
A_43_P15593	Chrm1	NM_080773	25229	-0.23	-1.706	0.001438
A_44_P427076	RGD1309655	NM_001013862	287766	-0.23	-1.706	0.017046
A_44_P836723	Rccd1_predicted	XM_218819	308760	-0.23	-1.707	0.027285
A_44_P421582	Tagln3	NM_031676	63837	-0.23	-1.707	0.026199
A_43_P15198	TC557452	TC557452		-0.23	-1.707	0.008869
A_44_P245711	RGD1560195_predicted	XM_222209		-0.23	-1.707	0.026331
A_44_P490315	AW144124	AW144124		-0.23	-1.707	0.032596
A_43_P15928	DLP2	XM_220603	303242	-0.23	-1.707	0.019129
A_44_P529269	Cdc42bpa	NM_053657	114116	-0.23	-1.708	0.000457
A_43_P10935	RGD1562339_predicted	XM_579785	498107	-0.23	-1.708	0.011266
A_43_P13894	Eea1_predicted	XM_235064		-0.23	-1.708	0.005331
A_44_P239864	AI231358	AI231358	362837	-0.23	-1.708	0.003763
A_44_P184008	BI279526	BI279526	294270	-0.23	-1.708	0.002336
A_44_P252559	Olr1682_predicted	NM_001000890	405191	-0.23	-1.708	0.030003
A_44_P1057475	LOC500419	NM_001031663	500419	-0.23	-1.709	0.015423
A_44_P959220	RGD1565212_predicted	XM_573805		-0.23	-1.709	0.005634
A_44_P369493	LOC500528	NM_001014067	500528	-0.23	-1.709	0.020619
A_44_P482281	Mapk10	NM_012806	25272	-0.23	-1.709	0.008122
A_44_P382603	ENSRNOT00000030885	ENSRNOT00000030885		-0.23	-1.709	0.000873
A_44_P213139	A_44_P213139	A_44_P213139		-0.23	-1.709	0.001551

A_44_P993121	Rab9	NM_053458	84589	-0.23	-1.709	0.004168
A_43_P11733	Ppard	NM_013141	25682	-0.23	-1.710	0.000735
A_44_P475374	Foxd4	XM_001078871		-0.23	-1.710	0.005117
A_44_P156462	Ren1	NM_012642	24715	-0.23	-1.710	0.165671
A_44_P200475	AI411527	AI411527	286888	-0.23	-1.710	0.006531
A_44_P227994	Itpka	NM_031045	81677	-0.23	-1.710	0.016629
A_44_P408574	AI228129	AI228129	679949	-0.23	-1.710	0.010149
A_44_P345738	BQ208844	BQ208844	362952	-0.23	-1.711	0.002585
A_44_P499992	AA858703	AA858703		-0.23	-1.711	0.040544
A_44_P178918	AA923937	AA923937	360985	-0.23	-1.712	0.025909
A_44_P414153	BF397707	BF397707		-0.23	-1.712	0.00604
A_44_P335296	XM_344357	XM_344357		-0.23	-1.712	0.006328
A_43_P10978	AA875244	AA875244	316064	-0.23	-1.712	0.035621
A_44_P1018622	RGD1359691	NM_001007611	287534	-0.23	-1.712	0.002515
A_42_P718390	MGC124825	NM_001033887	291444	-0.23	-1.713	0.003543
A_43_P15477	Csn10	NM_031562	29188	-0.23	-1.713	0.033044
A_43_P14378	Wdr13_predicted	XM_228783		-0.23	-1.713	0.003539
A_43_P14403	TC536843	TC536843		-0.23	-1.713	0.00177
A_44_P226109	Csf1r	X61479		-0.23	-1.714	0.246205
A_44_P914219	TC534822	TC534822		-0.23	-1.715	0.090787
A_44_P124146	XM_342929	XM_342929		-0.23	-1.715	0.00538
A_44_P161432	LOC678842	XM_001053443		-0.23	-1.715	0.108588
A_44_P532341	A_44_P532341	A_44_P532341		-0.23	-1.715	0.014294
A_44_P464132	A_44_P464132	A_44_P464132		-0.23	-1.715	0.038015
A_44_P735332	A_44_P735332	A_44_P735332		-0.23	-1.715	0.003543
A_44_P988227	AI101256	AI101256	305178	-0.23	-1.716	0.007629
A_44_P296478	AI013718	AI013718	25632	-0.23	-1.716	0.002174
A_44_P459547	A_44_P459547	A_44_P459547		-0.23	-1.716	0.018493
A_44_P411290	RGD1359593	NM_001014266	366872	-0.23	-1.716	0.00306
A_44_P421565	Kcnk6	NM_053806	116491	-0.23	-1.716	0.001336
A_44_P1000245	RGD1561817_predicted	XM_215530		-0.23	-1.716	0.000808
A_44_P214408	RGD1565768_predicted	XM_001073062		-0.23	-1.716	0.008293
A_42_P705083	Il17b	XM_341621	116472	-0.23	-1.716	0.217723
A_44_P314400	LOC363153	XM_343492	363154	-0.23	-1.716	0.021393
A_44_P166472	A_44_P166472	A_44_P166472		-0.23	-1.716	0.107125
A_44_P533844	Olr760_predicted	NM_001001069	405957	-0.23	-1.716	0.058333
A_44_P354388	A_44_P354388	A_44_P354388		-0.23	-1.716	0.035084
A_44_P452797	RGD1309534	NM_001014206	363016	-0.23	-1.716	0.013824
A_44_P137701	Cryge	NM_173289	24279	-0.23	-1.716	0.006793
A_43_P20161	RGD1308481_predicted	XM_226509	307897	-0.23	-1.716	0.004809
A_44_P1004392	LOC288109	XM_221455	288109	-0.23	-1.717	0.007693
A_44_P916237	TC544189	TC544189		-0.23	-1.717	0.004098
A_44_P165236	AI072694	AI072694	690899	-0.23	-1.717	0.014021
A_44_P456888	Cyp2w1_predicted	XM_221971	288517	-0.23	-1.717	0.031753
A_44_P313323	AA818217	AA818217	252928	-0.23	-1.717	0.00093
A_44_P292730	Rnase1_predicted	NM_001013232	364303	-0.23	-1.717	0.018639
A_44_P194730	Zbtb40_predicted	XM_342953	362635	-0.23	-1.718	0.001022
A_44_P996798	A_44_P996798	A_44_P996798		-0.23	-1.718	0.017143
A_44_P452393	LOC681316	XM_001061215		-0.24	-1.718	0.010824
A_43_P23184	LOC290704	XM_001053156		-0.24	-1.718	0.000542
A_44_P123296	Lhfpl4	NM_181387	353230	-0.24	-1.718	0.020939
A_44_P314159	Emilin3_predicted	XM_342567		-0.24	-1.719	0.007196
A_44_P414345	Olr559_predicted	NM_001000668	404870	-0.24	-1.719	0.038172
A_44_P369238	BG380409	BG380409	287847	-0.24	-1.719	0.005065
A_44_P324220	AI600195	AI600195		-0.24	-1.719	0.014792
A_44_P524041	BQ196053	BQ196053	363139	-0.24	-1.719	0.060306
A_44_P489280	Vegp2	NM_053574	94106	-0.24	-1.719	0.020678
A_43_P23310	Pitpnm2_predicted	XM_222155		-0.24	-1.719	0.001566
A_44_P796993	TC534010	TC534010		-0.24	-1.719	0.012407
A_44_P222936	Slc36a3	NM_001024764	303148	-0.24	-1.720	0.115503
A_44_P637299	Gdf11	XM_001071574		-0.24	-1.720	0.355011
A_44_P174487	AA875107	AA875107	691001	-0.24	-1.720	0.01713
A_44_P347250	Dhcr24	XM_216452		-0.24	-1.720	0.004767

A_44_P447477	Olr10_predicted	NM_001000115	293072	-0.24	-1.721	0.011568
A_44_P969565	LOC501403	XM_001081757		-0.24	-1.721	0.000948
A_44_P187428	RGD1561671_predicted	XM_341496		-0.24	-1.721	0.123919
A_42_P500080	Nkg7	NM_133540	171062	-0.24	-1.721	0.076758
A_44_P143337	AI102280	AI102280	301334	-0.24	-1.722	0.001142
A_44_P210753	LOC687008	XM_001075496		-0.24	-1.722	0.000357
A_44_P121887	Sidt2_predicted	XM_001068825		-0.24	-1.722	0.04207
A_44_P982738	AA997617	AA997617	25604	-0.24	-1.722	0.019977
A_44_P558028	LOC500528	NM_001014067	500528	-0.24	-1.722	0.008384
A_44_P959463	LOC681522	XM_001057230		-0.24	-1.722	0.003898
A_44_P682992	LOC680521	XM_001057547		-0.24	-1.722	0.084125
A_44_P389603	XM_235306	XM_235306		-0.24	-1.723	0.008012
A_44_P667264	LOC687654	XM_001079574		-0.24	-1.723	0.011717
A_42_P832832	Ckm	NM_012530	24265	-0.24	-1.723	0.031872
A_44_P284164	Clk1	XM_217405		-0.24	-1.723	0.025398
A_44_P241799	AI170252	AI170252		-0.24	-1.723	0.005314
A_44_P286123	RGD1305778	NM_001014043	310849	-0.24	-1.723	0.01671
A_44_P445608	LOC498781	XM_001063340	498781	-0.24	-1.723	0.08187
A_42_P681326	Dll3	NM_053666	114125	-0.24	-1.724	0.052711
A_44_P543576	A_44_P543576	A_44_P543576		-0.24	-1.724	0.069781
A_44_P632296	AW142609	AW142609	407765	-0.24	-1.724	0.027592
A_44_P410203	RGD1305202_predicted	XM_001062898		-0.24	-1.724	0.001273
A_42_P640019	TC523754	TC523754		-0.24	-1.724	0.006396
A_44_P438338	A_44_P438338	A_44_P438338		-0.24	-1.724	0.005791
A_44_P553535	BX883046	BX883046		-0.24	-1.725	0.005598
A_44_P424410	A_44_P424410	A_44_P424410		-0.24	-1.725	0.000997
A_44_P349348	RGD1564797_predicted	XM_574698		-0.24	-1.725	0.011215
A_44_P358883	ENSRNOT00000033863	ENSRNOT00000033863		-0.24	-1.725	0.048923
A_44_P431685	MGC116197	NM_001025755	367620	-0.24	-1.725	0.402358
A_44_P412215	Spn	XM_344964	24796	-0.24	-1.725	0.07828
A_44_P942903	Mpp5_predicted	XM_234328		-0.24	-1.725	0.007493
A_44_P163290	Olr691_predicted	NM_001000630	404822	-0.24	-1.725	0.023246
A_44_P250213	Rab4a	NM_013019	25532	-0.24	-1.725	0.000811
A_42_P757500	Itpr2	NM_031046	81678	-0.24	-1.726	0.003111
A_44_P145671	BE109570	BE109570	315707	-0.24	-1.726	0.002913
A_44_P530075	A_44_P530075	A_44_P530075		-0.24	-1.726	0.087229
A_44_P873329	TC568157	TC568157		-0.24	-1.726	0.005399
A_43_P19816	Smyd1_predicted	XM_216172		-0.24	-1.727	0.007282
A_44_P1004665	RGD1309139_predicted	XM_342321	362020	-0.24	-1.727	0.018121
A_44_P598944	AW143747	AW143747		-0.24	-1.727	0.002754
A_44_P330643	Polr2b_predicted	XM_214021		-0.24	-1.727	0.013164
A_44_P260453	RGD1564459_predicted	XM_342652	362331	-0.24	-1.727	0.092337
A_44_P550680	A_44_P550680	A_44_P550680		-0.24	-1.727	0.02267
A_44_P868863	TC539103	TC539103		-0.24	-1.728	0.011142
A_44_P106456	Pdlim2	NM_001007622	290354	-0.24	-1.728	0.005444
A_44_P311326	Cyp2b2	XM_341808	361523	-0.24	-1.729	0.011717
A_44_P166338	LOC301752	XR_008043	301752	-0.24	-1.729	0.162334
A_44_P349289	A_44_P349289	A_44_P349289		-0.24	-1.729	0.009913
A_44_P207363	BE113217	BE113217	295234	-0.24	-1.729	0.052855
A_44_P729018	BC087158	BC087158	1E+08	-0.24	-1.730	0.011162
A_43_P18637	XM_216838	XM_216838		-0.24	-1.730	0.013529
A_44_P792143	AW920896	AW920896		-0.24	-1.730	0.013076
A_44_P577287	TC558907	TC558907		-0.24	-1.730	0.058617
A_44_P363909	Caskin2_predicted	XM_221120		-0.24	-1.730	0.002109
A_44_P824441	TC527870	TC527870		-0.24	-1.730	0.024928
A_44_P463054	RGD1565217_predicted	XM_574851		-0.24	-1.730	0.003134
A_44_P391517	Nkx3-1	NM_001034144	305999	-0.24	-1.730	0.038504
A_44_P288896	BF284120	BF284120	94268	-0.24	-1.731	0.001221
A_44_P493566	AA999013	AA999013	305506	-0.24	-1.731	0.004248
A_44_P337455	Tyro3	NM_017092	25232	-0.24	-1.732	0.001112
A_44_P248111	Igsf4c_predicted	XM_001059474		-0.24	-1.732	0.005361
A_44_P384273	Kcnk12	NM_022292	64119	-0.24	-1.732	0.015307
A_44_P137805	XM_219385	XM_219385		-0.24	-1.732	0.022648

A_44_P928410	Dtnbp1	AY623025	641528	-0.24	-1.732	0.00679
A_44_P590268	LOC497940	NM_001017476	497940	-0.24	-1.732	0.000951
A_43_P14852	AW144408	AW144408		-0.24	-1.732	0.003491
A_44_P530108	A_44_P530108	A_44_P530108		-0.24	-1.732	0.119622
A_44_P312199	AA925676	AA925676		-0.24	-1.733	0.000963
A_44_P532599	BE099478	BE099478		-0.24	-1.734	0.012846
A_43_P13176	Agpat4	NM_133406	170919	-0.24	-1.734	0.014195
A_44_P669960	TC560395	TC560395		-0.24	-1.734	0.003006
A_44_P253601	Josd1	NM_001025009	315134	-0.24	-1.735	0.006763
A_44_P940327	AI008579	AI008579		-0.24	-1.735	0.008732
A_44_P914760	Elovl7_predicted	XM_342192	361895	-0.24	-1.735	0.024112
A_44_P442823	Tnfrsf5	NM_134360	171369	-0.24	-1.735	0.01786
A_44_P392012	A_44_P392012	A_44_P392012		-0.24	-1.735	0.040824
A_44_P393580	Mbc2	NM_017249	29579	-0.24	-1.735	0.001708
A_44_P205376	Fjx1_predicted	XM_345408		-0.24	-1.735	0.183176
A_42_P646738	Fbxo4_predicted	XM_227090		-0.24	-1.736	0.025372
A_44_P509010	XM_343379	XM_343379		-0.24	-1.736	0.009998
A_44_P809996	TC550946	TC550946		-0.24	-1.736	0.005439
A_43_P11817	Ghr	NM_017094	25235	-0.24	-1.736	0.020028
A_43_P11507	Svp4	NM_012662	24802	-0.24	-1.736	0.007123
A_43_P15654	Hcn2	NM_053684	114244	-0.24	-1.736	0.001405
A_44_P311735	Zdhhc12	NM_001013239	366014	-0.24	-1.736	0.122386
A_44_P726036	BI291916	BI291916		-0.24	-1.736	0.002295
A_44_P213454	TC553078	TC553078		-0.24	-1.737	0.000966
A_44_P391732	RGD1566387_predicted	XM_233287	298326	-0.24	-1.737	0.014972
A_44_P130961	RGD1306349_predicted	XM_231006	296512	-0.24	-1.737	0.00027
A_43_P11818	Crp	NM_017096	25419	-0.24	-1.738	0.009285
A_43_P17575	LOC361929	XM_342228	361929	-0.24	-1.738	0.010392
A_44_P1043157	Cxcl9	NM_145672	246759	-0.24	-1.738	0.07895
A_44_P373012	RGD1310376_predicted	XM_233381		-0.24	-1.738	0.008033
A_44_P147879	AA900787	AA900787	317423	-0.24	-1.739	0.010802
A_44_P623602	TC559402	TC559402		-0.24	-1.739	0.005387
A_44_P508254	LOC687625	XM_001074320		-0.24	-1.739	0.007528
A_42_P635377	AA942767	AA942767		-0.24	-1.739	0.003212
A_44_P227132	RGD1564324_predicted	XM_214176		-0.24	-1.739	0.013755
A_44_P522788	A_44_P522788	A_44_P522788		-0.24	-1.739	0.012399
A_44_P126642	Ttc3_predicted	XM_340973		-0.24	-1.739	0.00139
A_44_P503552	Lrp16	NM_139337	246233	-0.24	-1.739	0.003669
A_44_P261847	AA800005	AA800005	64315	-0.24	-1.739	0.027757
A_44_P395147	BQ782568	BQ782568	497902	-0.24	-1.740	0.095697
A_42_P635228	BF549025	BF549025	310074	-0.24	-1.740	0.010183
A_44_P370502	Slc25a12_predicted	XM_342445	362145	-0.24	-1.740	0.009536
A_44_P142401	RGD1564950_predicted	XM_223466		-0.24	-1.740	0.007075
A_44_P260102	Rasa2	XM_343450		-0.24	-1.740	0.000398
A_44_P403279	LOC685817	XM_001065390	685817	-0.24	-1.741	0.007014
A_44_P257953	RGD1560744_predicted	XM_225701	307251	-0.24	-1.741	0.010122
A_42_P564690	RGD1309316_predicted	XM_218559	292913	-0.24	-1.741	0.003024
A_43_P20953	Arhgap20	NM_213629	367085	-0.24	-1.741	0.098913
A_44_P431713	Als2cr7_predicted	XM_001069526		-0.24	-1.741	0.012546
A_44_P428535	Olr384_predicted	NM_001000263	293817	-0.24	-1.741	0.042207
A_44_P138898	AABR03076009	AABR03076009		-0.24	-1.741	0.051413
A_42_P780715	XM_223541	XM_223541		-0.24	-1.742	0.002198
A_42_P683328	Ratsg2	NM_173120	286900	-0.24	-1.742	0.00032
A_44_P879017	BF407382	BF407382		-0.24	-1.742	0.033412
A_44_P337560	XM_346991	XM_346991		-0.24	-1.742	0.024659
A_42_P541851	BF561434	BF561434	293690	-0.24	-1.742	0.003492
A_44_P885692	TC527071	TC527071		-0.24	-1.743	0.004472
A_43_P14557	A_43_P14557	A_43_P14557		-0.24	-1.743	0.030795
A_44_P493246	AW523375	AW523375	300763	-0.24	-1.743	0.005698
A_44_P119972	AI412064	AI412064	54226	-0.24	-1.743	0.029584
A_42_P574345	Hand1	BC097327	24256	-0.24	-1.743	0.014559
A_44_P293724	Usmg5	NM_133544	171069	-0.24	-1.743	0.001041
A_43_P10382	Slit1	AB073215	65047	-0.24	-1.743	0.012618

A_44_P114833	LOC302192	XM_217539	302192	-0.24	-1.743	0.035087
A_44_P597179	CO403017	CO403017		-0.24	-1.744	0.024831
A_44_P546356	XM_235358	XM_235358		-0.24	-1.744	0.011686
A_44_P189393	Olr1230_predicted	NM_001000595	367060	-0.24	-1.744	0.141161
A_44_P173556	XM_229666	XM_229666		-0.24	-1.744	0.013998
A_43_P21921	Cd226_predicted	XM_225664		-0.24	-1.744	0.089356
A_44_P222616	RGD1565629_predicted	XM_225862	307358	-0.24	-1.744	0.039902
A_43_P11667	St8sia3	NM_013029	25547	-0.24	-1.744	0.00806
A_44_P700840	TC560371	TC560371		-0.24	-1.745	0.018157
A_43_P11919	Pgam2	NM_017328	24959	-0.24	-1.745	0.00159
A_44_P1051106	XM_343269	XM_343269		-0.24	-1.745	0.005709
A_44_P137377	Taar8c	NM_175600	319105	-0.24	-1.745	0.05181
A_44_P387429	Hrh3	NM_053506	85268	-0.24	-1.745	0.035849
A_43_P13861	CB547746	CB547746	301056	-0.24	-1.745	0.003275
A_44_P833595	AA892485	AA892485	81654	-0.24	-1.746	0.025675
A_44_P302491	Ifna11_predicted	XM_233150		-0.24	-1.746	0.00998
A_44_P241458	XM_236872	XM_236872		-0.24	-1.746	0.10738
A_44_P385579	AW918085	AW918085		-0.24	-1.746	0.001677
A_44_P1071200	AJ517195	AJ517195		-0.24	-1.746	0.020431
A_44_P189734	RGD1561241_predicted	XM_001058603		-0.24	-1.746	0.000711
A_43_P12270	Bbox1	NM_022629	64564	-0.24	-1.747	0.034424
A_44_P538165	Fbxo23	NM_001013138	306771	-0.24	-1.747	0.00415
A_43_P15397	Capn5	NM_134461	171495	-0.24	-1.748	0.01136
A_44_P201934	BE107091	BE107091	117535	-0.24	-1.748	0.034281
A_44_P219926	Zbtb10	NM_024489	80338	-0.24	-1.748	0.003053
A_44_P257522	Agrp	XM_574228	25582	-0.24	-1.748	0.013941
A_44_P154344	XM_227151	XM_227151		-0.24	-1.748	0.043618
A_44_P189763	RGD1560636_predicted	XM_223587	305467	-0.24	-1.749	0.007702
A_44_P311096	Abcb4	NM_012690	24891	-0.24	-1.749	0.301649
A_44_P496496	Sh3gl2	XM_342857	116743	-0.24	-1.749	0.052073
A_44_P728793	RGD1566185_predicted	XM_573760		-0.24	-1.749	0.003254
A_44_P609265	TC566080	TC566080		-0.24	-1.749	0.008272
A_43_P20504	Rbm35b_predicted	XM_226421		-0.24	-1.749	0.002449
A_44_P554339	AABR03055433	AABR03055433		-0.24	-1.750	0.03516
A_44_P205869	Gpr149	NM_138891	192251	-0.24	-1.750	0.017046
A_44_P632287	BE110241	BE110241		-0.24	-1.750	0.187111
A_44_P103918	AA944422	AA944422	54321	-0.24	-1.750	0.053561
A_44_P959836	Ids	XM_579042	363513	-0.24	-1.750	0.00257
A_44_P558838	BF417038	BF417038		-0.24	-1.750	0.014369
A_44_P400999	RGD1562337_predicted	XM_233245	313418	-0.24	-1.750	0.005001
A_42_P702550	Sts	NM_012661	24800	-0.24	-1.751	0.003326
A_44_P976524	TC559191	TC559191		-0.24	-1.751	0.03889
A_44_P543303	Frmd4b	XM_232212	252858	-0.24	-1.751	0.004722
A_44_P201601	XM_216096	XM_216096		-0.24	-1.751	0.004037
A_44_P323120	Ggt6	NM_001002820	408206	-0.24	-1.751	0.001725
A_43_P19527	LOC360997	NM_001037205	360997	-0.24	-1.752	0.002962
A_44_P323624	A_44_P323624	A_44_P323624		-0.24	-1.752	0.017447
A_44_P827767	TC550365	TC550365		-0.24	-1.752	0.005384
A_42_P538400	Ntrk2	NM_012731	25054	-0.24	-1.752	0.03938
A_44_P186973	Olr417_predicted	NM_001000388	296679	-0.24	-1.752	0.010788
A_44_P715637	TC524696	TC524696		-0.24	-1.752	0.012168
A_44_P1014079	RGD1309108_predicted	XM_236102	315578	-0.24	-1.752	0.013972
A_44_P344981	RGD1560726_predicted	XM_233976		-0.24	-1.752	0.01629
A_44_P339594	Olr60_predicted	NM_001000748	405017	-0.24	-1.753	0.033888
A_44_P539244	RGD1308556	NM_001014200	362828	-0.24	-1.753	0.107181
A_44_P393235	Has1	NM_172323	282821	-0.24	-1.753	0.016853
A_44_P123542	Dlx1	XM_230987	296500	-0.24	-1.753	0.051939
A_44_P333384	RGD1310036	NM_001024788	316000	-0.24	-1.753	0.011475
A_44_P506258	Bhlhb5_predicted	XM_345190		-0.24	-1.754	0.087421
A_44_P446389	AI230968	AI230968	289230	-0.24	-1.754	0.004679
A_43_P20691	Dyx1c1	NM_001007010	363096	-0.24	-1.754	0.014727
A_44_P194454	XM_214167	XM_214167		-0.24	-1.754	0.047876
A_44_P313585	Ankra2	NM_207595	294679	-0.24	-1.754	0.01772

A_44_P268941	Tex264	NM_001007665	300988	-0.24	-1.754	0.000835
A_44_P713598	A_44_P713598	A_44_P713598		-0.24	-1.755	0.021205
A_44_P332586	Zswim6	XM_226779		-0.24	-1.755	0.000803
A_44_P373030	LOC301121	XM_236777		-0.24	-1.755	0.017983
A_43_P19030	Ptcd2_predicted	XM_226716		-0.24	-1.755	0.009343
A_44_P338196	AA858874	AA858874		-0.24	-1.755	0.018431
A_44_P344077	Flcn	NM_199390	303185	-0.24	-1.756	0.000735
A_44_P361857	Cpeb4_predicted	XM_220269		-0.24	-1.756	0.008367
A_44_P372701	XM_344243	XM_344243		-0.24	-1.757	0.002986
A_43_P16908	AW917070	AW917070	304650	-0.24	-1.757	0.00082
A_44_P423222	AI234161	AI234161	84475	-0.24	-1.757	0.004361
A_44_P308696	Grin2b	NM_012574	24410	-0.24	-1.757	0.025903
A_44_P209260	BE104224	BE104224	25478	-0.24	-1.758	0.060321
A_44_P1044030	RGD1561025_predicted	XM_341155		-0.24	-1.758	0.005564
A_43_P13165	Kcnq2	NM_133322	170848	-0.24	-1.758	0.012086
A_44_P809720	TC566592	TC566592		-0.25	-1.758	0.009868
A_44_P374823	LOC292872	XM_214931		-0.25	-1.759	0.001456
A_44_P163519	Rock2	NM_013022	25537	-0.25	-1.760	0.001009
A_44_P402578	Il7	NM_013110	25647	-0.25	-1.760	0.00622
A_44_P169882	AI071640	AI071640	313323	-0.25	-1.760	0.002068
A_44_P435037	AI232788	AI232788	79129	-0.25	-1.761	0.007056
A_44_P131107	XM_573907	XM_573907		-0.25	-1.761	0.001485
A_42_P795294	Rab27b	NM_053459	84590	-0.25	-1.761	0.003576
A_44_P229359	Mesp2_predicted	XM_218825		-0.25	-1.761	0.014608
A_43_P11606	Adk	NM_012895	25368	-0.25	-1.761	0.020217
A_44_P111723	Hap1	NM_024133	29430	-0.25	-1.761	0.028914
A_44_P228228	AW916309	AW916309	367113	-0.25	-1.761	0.013589
A_44_P1059122	RGD1311946	NM_001025683	309145	-0.25	-1.761	0.002553
A_43_P15451	Mcpt6	NM_019180	29268	-0.25	-1.762	0.042851
A_44_P974320	LOC360570	XM_001080824		-0.25	-1.762	0.004454
A_44_P149822	Mgam_predicted	XM_231714		-0.25	-1.762	0.001231
A_44_P889695	AI059487	AI059487	307916	-0.25	-1.762	0.006699
A_44_P726979	BF285140	BF285140	293678	-0.25	-1.762	0.036487
A_44_P134465	AA963226	AA963226	192275	-0.25	-1.762	0.024565
A_44_P543652	A_44_P543652	A_44_P543652		-0.25	-1.762	0.052513
A_44_P112841	AI232365	AI232365		-0.25	-1.762	0.001353
A_44_P505199	Gltp_predicted	XM_213793	288707	-0.25	-1.762	0.00475
A_44_P642834	TC550813	TC550813		-0.25	-1.762	0.01052
A_44_P307151	RGD1562679_predicted	XM_233522	298528	-0.25	-1.763	0.002256
A_44_P112469	Cdc42ep3_predicted	XM_233815	313838	-0.25	-1.763	0.002218
A_44_P1020780	Tmc4	NM_001034104	308310	-0.25	-1.763	0.003252
A_44_P350713	AI030022	AI030022	300519	-0.25	-1.763	0.005163
A_42_P675688	LOC286911	NM_173127	286911	-0.25	-1.763	0.014928
A_44_P251124	Ptpn18	NM_001013111	301333	-0.25	-1.764	0.006311
A_44_P224466	Olr567_predicted	NM_001000326	295806	-0.25	-1.764	0.027787
A_44_P175833	LOC367391	XM_346122		-0.25	-1.764	0.022912
A_44_P436641	AA955229	AA955229		-0.25	-1.764	0.003429
A_44_P457869	BF414018	BF414018	303330	-0.25	-1.764	0.00247
A_42_P567267	LOC682651	XM_001062488		-0.25	-1.764	0.091792
A_44_P525859	AA817940	AA817940		-0.25	-1.764	0.003652
A_44_P337271	Mtus1	XM_573915		-0.25	-1.764	0.002426
A_44_P295621	LOC360570	XM_340847		-0.25	-1.765	0.000792
A_43_P21681	Slc12a7	XM_001060536		-0.25	-1.765	0.000446
A_44_P533672	BG670971	BG670971		-0.25	-1.765	0.093695
A_44_P651814	LOC687262	XM_001077750		-0.25	-1.765	0.025376
A_44_P988207	BM392264	BM392264		-0.25	-1.765	0.01199
A_43_P15805	Trhr2	NM_181364	252859	-0.25	-1.765	0.0225
A_44_P387119	RGD1560203_predicted	XM_228941		-0.25	-1.765	0.008438
A_44_P449282	BI294916	BI294916	306330	-0.25	-1.765	0.006595
A_42_P550396	Galnt10	NM_130742	170501	-0.25	-1.766	0.000198
A_44_P323349	Gria4	NM_017263	29629	-0.25	-1.766	0.007192
A_44_P376768	AA925615	AA925615	299706	-0.25	-1.766	0.016577
A_44_P243708	MGC116197	NM_001025755	367620	-0.25	-1.766	0.37361

A_44_P195839	LOC682926	XM_001060811		-0.25	-1.766	0.016443
A_44_P392626	BQ191871	BQ191871	361680	-0.25	-1.766	0.034351
A_43_P20443	MGC114427	NM_001024893	317297	-0.25	-1.767	0.061944
A_43_P12048	Atp5h	NM_019383	641434	-0.25	-1.768	0.000478
A_44_P319582	LOC684800	XM_001071989		-0.25	-1.768	0.006882
A_44_P490484	AI044674	AI044674		-0.25	-1.768	0.002538
A_44_P1038392	Cox7a2	NM_022503	29507	-0.25	-1.768	0.000905
A_44_P898195	RGD1561846_predicted	XM_573630	498392	-0.25	-1.768	0.024098
A_44_P883883	LOC679861	XM_001054768	679861	-0.25	-1.768	0.005668
A_44_P122052	AA925174	AA925174	366515	-0.25	-1.768	0.003605
A_42_P548800	mrpl9	NM_001007696	310653	-0.25	-1.769	0.000355
A_44_P468949	Vps13d_predicted	XM_233792		-0.25	-1.769	0.002757
A_44_P829145	TC560772	TC560772		-0.25	-1.769	0.009873
A_44_P771864	Cdc14a_predicted	XM_001073065		-0.25	-1.769	0.014353
A_43_P14968	Faah	NM_024132	29347	-0.25	-1.769	0.00177
A_44_P326289	Jmjd2c_predicted	XM_216426		-0.25	-1.769	0.001343
A_44_P501451	XM_224068	XM_224068		-0.25	-1.769	0.036365
A_43_P22274	Mg29_predicted	XM_342316		-0.25	-1.769	0.011965
A_44_P227044	XM_341140	XM_341140		-0.25	-1.769	0.021607
A_42_P723936	Atp2b3	XM_343839		-0.25	-1.770	0.002269
A_44_P293213	RGD1563084_predicted	XR_007329	315912	-0.25	-1.770	0.000783
A_44_P392890	BM392112	BM392112	292588	-0.25	-1.770	0.026426
A_44_P628530	TC565616	TC565616		-0.25	-1.770	0.003933
A_44_P370953	AI044685	AI044685		-0.25	-1.770	0.029577
A_43_P10925	RGD1561090_predicted	XM_001067936		-0.25	-1.770	0.025712
A_44_P589021	AI045508	AI045508	314621	-0.25	-1.770	0.021319
A_44_P195291	RGD1560252_predicted	XM_233382		-0.25	-1.770	0.001399
A_44_P208765	Mthfr_predicted	XM_342975	362657	-0.25	-1.770	0.003277
A_43_P12059	Rnase4	NM_020082	56759	-0.25	-1.770	0.003861
A_44_P292061	RGD1563309_predicted	XM_001065445		-0.25	-1.771	0.00067
A_43_P23287	RGD1562107_predicted	XM_343545	363205	-0.25	-1.771	0.054591
A_44_P339348	AW914933	AW914933	292098	-0.25	-1.771	0.112613
A_44_P171305	XM_345875	XM_345875		-0.25	-1.771	0.059504
A_42_P828242	Lgr4	NM_173328	286994	-0.25	-1.772	0.022518
A_44_P901290	TC562886	TC562886		-0.25	-1.772	0.026723
A_44_P183191	Tnip1_predicted	XM_343894		-0.25	-1.772	0.001255
A_44_P309573	Cbll1_predicted	XM_234039		-0.25	-1.772	0.003016
A_44_P525349	RGD1310131_predicted	XM_215832	296145	-0.25	-1.772	0.001891
A_44_P837888	Acpl2	NM_001007710	315939	-0.25	-1.773	0.024261
A_44_P262102	Pafah1b3	NM_053654	114113	-0.25	-1.773	0.000778
A_43_P14987	Atp5j	NM_053602	94271	-0.25	-1.773	0.000589
A_44_P259796	Accn3	NM_173135	286920	-0.25	-1.773	0.002927
A_44_P440321	Olr788_predicted	NM_001000602	404790	-0.25	-1.773	0.018709
A_44_P341535	BI282584	BI282584	686092	-0.25	-1.773	0.001105
A_44_P243691	LOC301881	XM_229363		-0.25	-1.773	0.131635
A_44_P165494	Itgae	NM_031768	83577	-0.25	-1.773	0.024648
A_44_P608437	LOC498029	BC088247	498029	-0.25	-1.773	0.003522
A_44_P478637	BI274236	BI274236		-0.25	-1.773	0.055058
A_44_P354532	BE095847	BE095847		-0.25	-1.774	0.016
A_44_P462169	XM_344035	XM_344035		-0.25	-1.774	0.038403
A_44_P529682	Chit1_predicted	XM_213878		-0.25	-1.774	0.071062
A_44_P1045820	RGD1565763_predicted	XM_214631	291847	-0.25	-1.774	0.001891
A_44_P519723	LOC684567	XM_001071017		-0.25	-1.774	0.004281
A_44_P184599	Olr137_predicted	NM_001001283	405003	-0.25	-1.774	0.018357
A_44_P240523	AI169232	AI169232	116478	-0.25	-1.774	0.023337
A_44_P623610	TC525804	TC525804		-0.25	-1.774	0.003284
A_44_P752777	BF404531	BF404531		-0.25	-1.775	0.000859
A_44_P902971	AA849718	AA849718	114709	-0.25	-1.775	0.013957
A_44_P203355	Hapln2	NM_022285	64057	-0.25	-1.775	0.004478
A_44_P333521	BE114108	BE114108	83518	-0.25	-1.776	0.026212
A_44_P511516	Zbtb20_predicted	XM_213621		-0.25	-1.776	0.00098
A_44_P827919	TC520730	TC520730		-0.25	-1.776	0.083112
A_44_P561859	CK843557	CK843557		-0.25	-1.776	0.016664

A_44_P450471	Ppp1r16a_predicted	XM_343274	362944	-0.25	-1.777	0.000548
A_44_P172164	AW435110	AW435110		-0.25	-1.777	0.022122
A_43_P11824	Clcn5	NM_017106	25749	-0.25	-1.777	0.002109
A_44_P336326	BQ201831	BQ201831		-0.25	-1.777	0.005706
A_44_P255095	LOC292449	NM_001013892		-0.25	-1.778	0.026105
A_44_P178213	RGD1560479_predicted	XM_228309		-0.25	-1.778	0.023742
A_44_P402897	A_44_P402897	A_44_P402897		-0.25	-1.778	0.005497
A_44_P1013007	Elmo3	NM_001030028	291962	-0.25	-1.778	0.001113
A_44_P182739	Pdzk8_predicted	XM_217651		-0.25	-1.778	0.001319
A_44_P501314	XM_218158	XM_218158		-0.25	-1.778	0.013143
A_44_P276885	LOC690807	XM_001075698		-0.25	-1.778	0.00841
A_44_P207282	CA509361	CA509361	301005	-0.25	-1.779	0.0155
A_44_P563049	TC540682	TC540682		-0.25	-1.779	0.01677
A_44_P186317	Eps8_predicted	XM_001072957		-0.25	-1.779	0.000619
A_44_P305200	Cstb	NM_012838	25308	-0.25	-1.779	0.005841
A_43_P13556	CB547822	CB547822		-0.25	-1.779	0.010587
A_44_P578847	TC549847	TC549847		-0.25	-1.779	0.011786
A_44_P133087	Odz4_predicted	XM_218927	308831	-0.25	-1.779	0.004899
A_44_P339936	Xpr1_predicted	XM_223117		-0.25	-1.779	0.000647
A_44_P148121	BQ200023	BQ200023	83805	-0.25	-1.780	0.028578
A_43_P14036	isg12(b)	XM_238467		-0.25	-1.780	0.021521
A_44_P311046	AA946377	AA946377	79433	-0.25	-1.780	0.01729
A_44_P1013163	Clybl	XM_240311	306198	-0.25	-1.780	0.000775
A_44_P307167	Lrrc38_predicted	XM_233624		-0.25	-1.780	0.014633
A_44_P212242	BQ202056	BQ202056	360611	-0.25	-1.780	0.023875
A_44_P314270	RGD1562590_predicted	XM_231787	312365	-0.25	-1.780	0.029461
A_43_P13272	Agt	NM_134432	24179	-0.25	-1.781	0.039878
A_42_P810313	Mtf1_predicted	XM_342910		-0.25	-1.781	0.000542
A_44_P422321	Vps53_predicted	XM_213395		-0.25	-1.781	0.001894
A_44_P137681	Khk	NM_031855	25659	-0.25	-1.782	0.004474
A_43_P11538	Hk1	NM_012734	25058	-0.25	-1.782	0.002358
A_44_P509144	XM_344017	XM_344017		-0.25	-1.782	0.003735
A_43_P21221	LOC680434	XM_001057161		-0.25	-1.782	0.000745
A_44_P1056503	Ppm1j	NM_001005540	295341	-0.25	-1.782	0.020512
A_44_P388545	BM986478	BM986478	79238	-0.25	-1.782	0.005377
A_44_P809032	RGD1566001_predicted	XM_575888		-0.25	-1.783	0.012684
A_44_P309499	RGD1560439_predicted	XM_228962	302703	-0.25	-1.783	0.035471
A_44_P112050	RGD1560523_predicted	XM_573428	171347	-0.25	-1.783	0.002009
A_44_P1054280	Pck2_predicted	XM_001055522		-0.25	-1.783	0.003175
A_44_P337706	RGD1563455_predicted	XM_227852		-0.25	-1.783	0.132618
A_44_P620807	Ndufa9	XM_001064382		-0.25	-1.784	0.003542
A_44_P498620	Cga	NM_053918	116700	-0.25	-1.784	0.003339
A_44_P665909	AW141926	AW141926		-0.25	-1.784	0.014089
A_44_P331319	AA900438	AA900438		-0.25	-1.784	0.005846
A_44_P196909	Wfdc5_predicted	XM_215934		-0.25	-1.784	0.024848
A_44_P412159	Cast	NM_053295	25403	-0.25	-1.784	0.004899
A_44_P219042	XM_215101	XM_215101		-0.25	-1.784	0.000714
A_44_P837093	RGD1308023_predicted	XM_001055483		-0.25	-1.784	0.003624
A_44_P1004081	Mrs2l	AF288290	79032	-0.25	-1.785	0.086128
A_44_P824812	TC530091	TC530091		-0.25	-1.785	0.006918
A_44_P133226	Stox2	XM_224852	306459	-0.25	-1.785	0.002975
A_44_P527205	RGD1565230_predicted	NM_001025116	305844	-0.25	-1.785	0.00343
A_44_P487876	Rnf167	NM_001008361	360554	-0.25	-1.785	0.000521
A_44_P591109	ENSRNOT00000035767	ENSRNOT00000035767		-0.25	-1.785	0.0059
A_44_P880338	AI169278	AI169278	24917	-0.25	-1.785	0.031041
A_44_P537273	BE119515	BE119515	309646	-0.25	-1.785	0.000936
A_44_P604438	AW142635	AW142635	312656	-0.25	-1.785	0.003619
A_44_P1018359	Rutbc3	NM_198787	362963	-0.25	-1.785	0.007457
A_44_P886332	TC529406	TC529406		-0.25	-1.786	0.008087
A_44_P328849	RGD1560562_predicted	XM_221017		-0.25	-1.787	0.014015
A_44_P206563	Tbx21_predicted	XM_220914		-0.25	-1.787	0.01631
A_44_P1005583	Phox2a	NM_053869	116648	-0.25	-1.787	0.007258
A_42_P592205	Gltp_predicted	XM_213793	288707	-0.25	-1.788	0.000624

A_44_P377951	BE105479	BE105479		-0.25	-1.788	0.002059
A_44_P469889	AI408677	AI408677	360678	-0.25	-1.788	0.023298
A_44_P396581	CB547296	CB547296		-0.25	-1.788	0.027809
A_44_P730248	Evi1_predicted	XM_226987		-0.25	-1.788	0.018178
A_44_P315218	AABR03025850	AABR03025850		-0.25	-1.788	0.03631
A_44_P402888	Eps8l1_predicted	XM_341782		-0.25	-1.788	0.03049
A_44_P326273	RGD1310364_predicted	XM_232840	313111	-0.25	-1.788	0.024454
A_44_P448364	BF561110	BF561110		-0.25	-1.789	0.008632
A_42_P758200	Zfp179	NM_138613	24916	-0.25	-1.789	0.014918
A_44_P270658	AW918933	AW918933		-0.25	-1.789	0.02583
A_44_P122333	AA926021	AA926021		-0.25	-1.790	0.008708
A_44_P468283	Olr789_predicted	NM_001000377	296042	-0.25	-1.790	0.045744
A_44_P736397	TC540649	TC540649		-0.25	-1.790	0.002271
A_44_P499577	Foxi1_predicted	XM_220287		-0.25	-1.790	0.0038
A_44_P507961	BF414048	BF414048	362530	-0.25	-1.790	0.122292
A_44_P112564	XM_235474	XM_235474		-0.25	-1.791	0.001003
A_44_P248770	AA818994	AA818994		-0.25	-1.791	0.001965
A_44_P273839	Reg3g	NM_173097	24620	-0.25	-1.791	0.358815
A_42_P505393	Serpinb6a	NM_199085	291085	-0.25	-1.791	0.002952
A_44_P204097	RGD1307278_predicted	XM_343172		-0.25	-1.791	0.035309
A_44_P929498	AW143774	AW143774		-0.25	-1.792	0.016208
A_44_P380031	Tom1	NM_001008365	361370	-0.25	-1.792	0.008241
A_44_P207907	Ppp1cc	NM_022498	24669	-0.25	-1.792	0.002305
A_44_P123333	Pparg	NM_013124	25664	-0.25	-1.792	0.021073
A_44_P410576	BF408265	BF408265	363915	-0.25	-1.792	0.019905
A_42_P602822	AW913892	AW913892	24716	-0.25	-1.793	0.010055
A_44_P148982	AI045720	AI045720	312451	-0.25	-1.793	0.018454
A_44_P165800	Dspp	NM_012790	25254	-0.25	-1.794	0.036631
A_44_P240330	RGD1565768_predicted	XM_217645		-0.25	-1.794	0.002304
A_42_P822638	Tsc22d3	NM_031345	83514	-0.25	-1.794	0.004274
A_44_P178419	XM_235663	XM_235663		-0.25	-1.794	0.000533
A_44_P977136	TC527193	TC527193		-0.25	-1.794	0.00142
A_44_P961111	TC557854	TC557854		-0.25	-1.794	0.00532
A_44_P394782	AA891821	AA891821	316530	-0.25	-1.795	0.045326
A_44_P472726	AI145286	AI145286		-0.25	-1.795	0.004733
A_44_P340477	RGD1559567_predicted	XM_343918		-0.25	-1.795	0.000789
A_44_P582787	AW535616	AW535616		-0.25	-1.795	0.014712
A_44_P605454	AY325217	AY325217		-0.25	-1.795	0.000828
A_42_P512387	Lim2	NM_053771	114903	-0.25	-1.796	0.019548
A_42_P688815	Slc12a7	XM_001060536		-0.25	-1.796	0.000957
A_44_P578950	TC527514	TC527514		-0.25	-1.796	0.006771
A_44_P506491	XM_216406	XM_216406		-0.25	-1.796	0.008879
A_43_P18171	Ldhd	NM_001008893	307858	-0.25	-1.796	0.002284
A_43_P14367	TC560879	TC560879		-0.25	-1.796	0.001283
A_44_P126114	LOC683045	XM_001064254		-0.25	-1.797	0.007354
A_44_P256806	BF406540	BF406540	308857	-0.25	-1.797	0.001399
A_42_P686916	Tex9_predicted	XM_001054229		-0.25	-1.797	0.001132
A_44_P304499	Centd2	XM_341895	361617	-0.25	-1.797	0.000493
A_42_P631615	LOC292780	XM_214896	292780	-0.25	-1.797	0.001685
A_44_P148548	AI059837	AI059837	304055	-0.25	-1.798	0.004397
A_44_P428229	AW914778	AW914778		-0.25	-1.798	0.10058
A_44_P368017	Pcdh17_predicted	XM_224389		-0.25	-1.798	0.034276
A_44_P1040434	RGD1311310_predicted	XM_235517	315166	-0.25	-1.799	0.000558
A_44_P358444	Wnt2	XM_575397	114487	-0.25	-1.799	0.026374
A_44_P167408	AW144014	AW144014	287710	-0.25	-1.799	0.001978
A_42_P504358	RGD1305347_predicted	XM_342895		-0.26	-1.799	0.000509
A_44_P323304	Pygb	XM_342542		-0.26	-1.800	0.005588
A_44_P872646	TC546950	TC546950		-0.26	-1.800	0.010539
A_44_P440663	Prdm7_predicted	XM_344847		-0.26	-1.800	0.01084
A_44_P294932	Cds1	NM_031242	81925	-0.26	-1.800	0.014363
A_44_P829888	LOC498796	NM_001025034	498796	-0.26	-1.800	0.007237
A_44_P172850	RT1-CE5	NM_001008843	24737	-0.26	-1.800	0.002953
A_43_P19047	Golph3l	NM_001007698	310669	-0.26	-1.801	0.001035

A_44_P450496	LOC685672	XM_001064779	685672	-0.26	-1.801	0.023651
A_44_P119273	Kif13a_predicted	XM_217893		-0.26	-1.801	0.000692
A_44_P305626	AA955883	AA955883	29510	-0.26	-1.801	0.018944
A_44_P821444	RGD1562102_predicted	XM_573237		-0.26	-1.801	0.010966
A_43_P13004	Cdh17	NM_053977	117048	-0.26	-1.802	0.001903
A_43_P12921	Cntn5	NM_053746	114589	-0.26	-1.802	0.021108
A_44_P761041	Trim2	XM_001065860		-0.26	-1.802	0.004715
A_44_P958139	CO387496	CO387496	315926	-0.26	-1.802	0.01897
A_44_P313954	ENSRNOT00000049488	ENSRNOT00000049488		-0.26	-1.802	0.004864
A_44_P800492	A_44_P800492	A_44_P800492		-0.26	-1.802	0.025177
A_44_P347340	Slc37a2_predicted	XM_345918	367056	-0.26	-1.802	0.057211
A_44_P113907	Cmbp	NM_170787	259269	-0.26	-1.802	0.000444
A_43_P12141	Stx7	NM_021869	60466	-0.26	-1.803	0.022531
A_44_P868418	AI043758	AI043758	295325	-0.26	-1.803	0.002202
A_44_P775232	ENSRNOT00000022577	ENSRNOT00000022577		-0.26	-1.803	0.024871
A_44_P555539	Dsc2	NM_001033688	291760	-0.26	-1.803	0.003738
A_44_P274362	TC523690	TC523690		-0.26	-1.804	0.003686
A_44_P169677	BQ779899	BQ779899	681389	-0.26	-1.804	0.032712
A_44_P320073	Cds1	NM_031242	81925	-0.26	-1.804	0.05194
A_44_P727504	BF289421	BF289421		-0.26	-1.804	0.003714
A_44_P470627	LOC501232	NM_001024365	29225	-0.26	-1.804	0.003719
A_44_P1012463	TC525449	TC525449		-0.26	-1.804	0.078381
A_44_P303929	Rit2	NM_001013060	291713	-0.26	-1.804	0.077128
A_43_P11955	Syn2	NM_019159	29179	-0.26	-1.804	0.04483
A_44_P137040	AA964058	AA964058	81770	-0.26	-1.804	0.014363
A_44_P362343	AA859680	AA859680	304649	-0.26	-1.805	0.00715
A_44_P872195	LOC499337	XM_001078906		-0.26	-1.805	0.002731
A_44_P778740	TC529780	TC529780		-0.26	-1.805	0.001401
A_42_P696282	RGD1311186_predicted	XM_215110		-0.26	-1.805	0.001091
A_44_P1000935	Sord	NM_017052	24788	-0.26	-1.805	0.013745
A_44_P544513	BM385726	BM385726		-0.26	-1.805	0.004331
A_44_P533337	BM386267	BM386267	1E+08	-0.26	-1.806	0.015076
A_44_P943883	A_44_P943883	A_44_P943883		-0.26	-1.806	0.012768
A_44_P883893	Acss1_predicted	XM_215897		-0.26	-1.806	0.00089
A_43_P15033	TC555136	TC555136		-0.26	-1.806	0.001873
A_44_P323223	Mrgprd	NM_001001506	293648	-0.26	-1.806	0.06413
A_44_P978862	TC565420	TC565420		-0.26	-1.806	0.051928
A_44_P871130	LOC500908	XM_001077427		-0.26	-1.806	0.002203
A_44_P197140	TC533929	TC533929		-0.26	-1.806	0.046178
A_44_P107622	LOC289968	XR_008797	289968	-0.26	-1.807	0.01303
A_44_P510003	AI145586	AI145586	688540	-0.26	-1.807	0.002235
A_44_P119244	XM_227418	XM_227418		-0.26	-1.807	0.007612
A_44_P190144	Ddef1_predicted	XM_235296		-0.26	-1.807	0.021876
A_44_P452209	NP516904	NP516904		-0.26	-1.808	0.01523
A_44_P379772	Fbxo17	NM_001013064	292757	-0.26	-1.808	0.008423
A_44_P332515	Olr1523	NM_001000713	404962	-0.26	-1.809	0.012012
A_44_P219596	Prop1	NM_153627	266738	-0.26	-1.809	0.002661
A_44_P478850	Htr3b	NM_022189	58963	-0.26	-1.809	0.005805
A_44_P199343	A_44_P199343	A_44_P199343		-0.26	-1.809	0.021301
A_44_P472034	AI411542	AI411542	312052	-0.26	-1.809	0.014199
A_44_P240973	Kit	NM_022264	64030	-0.26	-1.809	0.014695
A_43_P19921	Mtap7_predicted	XM_214968		-0.26	-1.809	0.00069
A_44_P415247	A_44_P415247	A_44_P415247		-0.26	-1.810	0.052691
A_44_P416066	AI070998	AI070998	85472	-0.26	-1.810	0.018494
A_44_P142952	BE111679	BE111679	29502	-0.26	-1.810	0.024205
A_44_P311351	Pou2f3	X67303	116544	-0.26	-1.810	0.00228
A_44_P556944	AI409880	AI409880		-0.26	-1.810	0.007528
A_44_P262550	XM_341913	XM_341913		-0.26	-1.810	0.002435
A_44_P951579	A_44_P951579	A_44_P951579		-0.26	-1.810	0.031919
A_44_P562314	TC524618	TC524618		-0.26	-1.810	0.001967
A_44_P901264	TC545358	TC545358		-0.26	-1.810	0.000542
A_44_P137003	Ocil	NM_130402	113937	-0.26	-1.810	0.029346
A_44_P904150	TC566444	TC566444		-0.26	-1.811	0.00029

A_44_P135559	LOC685479	XM_001063950		-0.26	-1.811	0.029217
A_44_P292568	Olr233_predicted	NM_001000207	293377	-0.26	-1.811	0.291035
A_44_P267778	RGD1310708_predicted	XM_220774		-0.26	-1.812	0.000662
A_44_P195653	AA818262	AA818262	362850	-0.26	-1.812	0.029034
A_44_P534409	Nebl_predicted	XM_225644	307189	-0.26	-1.812	0.022553
A_44_P247906	Kcnj3	U42423	50599	-0.26	-1.812	0.009302
A_43_P17569	RGD1562860_predicted	XM_001055920		-0.26	-1.812	0.000403
A_44_P130476	Rnase1	NM_001029904	364304	-0.26	-1.813	0.005096
A_44_P444247	Tmf1	NM_053671	114206	-0.26	-1.813	0.000539
A_44_P346807	Tsc22d3	NM_031345	83514	-0.26	-1.813	0.00037
A_44_P187005	Abcb9	NM_022238	63886	-0.26	-1.813	0.003264
A_44_P168543	XM_342294	XM_342294		-0.26	-1.813	0.00438
A_44_P529904	RGD1305898_predicted	XM_341560	361275	-0.26	-1.813	0.253774
A_44_P443161	Ank1	XM_240464		-0.26	-1.814	0.024139
A_44_P234048	Gpa33_predicted	XM_341149	360873	-0.26	-1.814	0.000513
A_42_P472274	Slurp1_predicted	XM_216966	300016	-0.26	-1.814	0.016523
A_44_P420343	BF523202	BF523202		-0.26	-1.814	0.017072
A_44_P126714	BE098732	BE098732	316129	-0.26	-1.814	0.009866
A_44_P330023	AA998363	AA998363	299923	-0.26	-1.814	0.040562
A_44_P761302	TC521008	TC521008		-0.26	-1.815	0.001455
A_44_P473586	MGC94915	NM_001007009	362712	-0.26	-1.815	0.005148
A_44_P864029	AI454557	AI454557		-0.26	-1.815	0.013788
A_44_P538521	Star	NM_031558	25557	-0.26	-1.815	0.028673
A_44_P146773	Olr8_predicted	NM_001000113	292563	-0.26	-1.816	0.012146
A_43_P19900	Zfyve1_predicted	XM_216762		-0.26	-1.816	0.000542
A_44_P688801	TC550574	TC550574		-0.26	-1.816	0.003704
A_44_P445244	Artn	NM_053397	362572	-0.26	-1.816	0.003868
A_44_P712441	RGD1559449_predicted	XM_576572		-0.26	-1.817	0.014504
A_43_P12390	Tas2r16	NM_023994	78980	-0.26	-1.817	0.007674
A_44_P185079	LOC290763	XM_224880	290763	-0.26	-1.817	0.076058
A_44_P735286	A_44_P735286	A_44_P735286		-0.26	-1.817	0.0769
A_44_P178159	Rnf24_predicted	XM_342522		-0.26	-1.817	0.002028
A_42_P544167	Tep1	NM_022591	64523	-0.26	-1.818	0.00031
A_44_P971738	BF281977	BF281977		-0.26	-1.818	0.016562
A_44_P459918	Dnaja4	NM_001025411	300721	-0.26	-1.818	0.042786
A_44_P135424	XM_223116	XM_223116		-0.26	-1.818	0.002271
A_44_P228177	AA818520	AA818520	498406	-0.26	-1.818	0.003501
A_44_P114421	LOC684725	XM_001071746		-0.26	-1.818	0.194979
A_43_P13162	Slc40a1	NM_133315	170840	-0.26	-1.818	0.020512
A_43_P18239	RGD1565768_predicted	XM_217645		-0.26	-1.819	0.000933
A_44_P270395	Pvrl3_predicted	XM_001059227		-0.26	-1.819	0.001746
A_44_P250028	Ppp1r3b	NM_138912	192280	-0.26	-1.820	0.006143
A_42_P677354	Scn8a	NM_019266	29710	-0.26	-1.820	0.024498
A_44_P213320	LOC691626	XM_001079069		-0.26	-1.820	0.322077
A_44_P391068	Gstm4	NM_020540	57298	-0.26	-1.820	0.013289
A_44_P525712	AI412054	AI412054	494529	-0.26	-1.821	0.005102
A_44_P175568	XM_344339	XM_344339		-0.26	-1.821	0.005726
A_44_P560841	TC536478	TC536478		-0.26	-1.821	0.001448
A_44_P821544	LOC681091	XM_001062522		-0.26	-1.821	0.002099
A_43_P22111	Cnnm4_predicted	XM_343554	363216	-0.26	-1.821	0.00395
A_44_P609199	TC566027	TC566027		-0.26	-1.821	0.013043
A_44_P539039	XM_230709	XM_230709		-0.26	-1.821	0.040313
A_43_P14184	Acadl	NM_012819	25287	-0.26	-1.822	0.001565
A_42_P692476	Slc39a4_predicted	XM_216970		-0.26	-1.823	0.022885
A_44_P377091	LOC500761	NM_001024351	500761	-0.26	-1.823	0.001062
A_44_P487632	Ifna4_predicted	XM_001053305		-0.26	-1.823	0.073069
A_44_P314793	AI639319	AI639319	361419	-0.26	-1.824	0.055663
A_44_P315286	AA963823	AA963823	29338	-0.26	-1.824	0.051646
A_44_P394436	BM384260	BM384260	361467	-0.26	-1.824	0.020706
A_44_P484793	Cbl27	XM_001077084		-0.26	-1.824	0.001309
A_44_P161723	RGD1305298_predicted	XM_238476	299330	-0.26	-1.824	0.001469
A_44_P119251	RGD1306520	XM_227573	310775	-0.26	-1.824	0.004235
A_44_P344950	LOC498470	XM_573727		-0.26	-1.824	0.169586

A_44_P357635	AA860061	AA860061	25056	-0.26	-1.824	0.003482
A_44_P400767	Neurog2_predicted	XM_227716		-0.26	-1.825	0.002059
A_44_P344959	LOC689103	XM_001066317	689103	-0.26	-1.825	0.000458
A_43_P22202	Aox3	NM_001008527	493909	-0.26	-1.826	0.003249
A_44_P306414	Olr1679_predicted	NM_001000892	405193	-0.26	-1.826	0.021131
A_44_P432355	Ugt1a2	NM_201423	113992	-0.26	-1.826	0.013058
A_44_P235241	AI029054	AI029054	83514	-0.26	-1.826	0.018414
A_44_P838992	LOC687609	XM_001079372		-0.26	-1.826	0.001338
A_44_P250928	XM_345733	XM_345733		-0.26	-1.826	0.014473
A_44_P148560	AW143690	AW143690		-0.26	-1.827	0.020958
A_44_P321359	Zfp612_predicted	XM_226455		-0.26	-1.827	0.003494
A_44_P511034	Veph1	NM_001014171	361954	-0.26	-1.827	0.010432
A_44_P560021	LOC686081	XM_001064019		-0.26	-1.827	0.050795
A_44_P1029032	Slco1c1	NM_053441	84511	-0.26	-1.827	0.044378
A_44_P125913	Syt1	NM_001033680	25716	-0.26	-1.827	0.014015
A_44_P373230	Alox12e_predicted	XM_220567		-0.26	-1.827	0.00698
A_44_P398455	Sec24d_predicted	XM_227663		-0.26	-1.828	0.024304
A_44_P525207	A_44_P525207	A_44_P525207		-0.26	-1.828	0.006537
A_44_P386633	Hdgfrp3	NM_145785	252941	-0.26	-1.828	0.007111
A_44_P101708	AW434670	AW434670	316071	-0.26	-1.829	0.019934
A_44_P395258	AI236635	AI236635	287280	-0.26	-1.829	0.039762
A_43_P21849	RGD1565709_predicted	XM_342764	362442	-0.26	-1.829	0.295795
A_44_P407146	Npm2	NM_203340	290359	-0.26	-1.829	0.080588
A_44_P992056	Tmem106a	NM_001024967	287722	-0.26	-1.830	0.018175
A_43_P22368	RGD1565895_predicted	XM_218953	308850	-0.26	-1.830	0.001434
A_44_P728923	A_44_P728923	A_44_P728923		-0.26	-1.830	0.037358
A_43_P14062	Btg1	NM_017258	29618	-0.26	-1.831	0.000348
A_44_P229118	Muc1	XM_342281	24571	-0.26	-1.831	0.131083
A_44_P508653	S100z_predicted	XM_226710		-0.26	-1.831	0.007791
A_44_P281788	BQ196033	BQ196033	500292	-0.26	-1.831	0.021385
A_44_P700056	TC556993	TC556993		-0.26	-1.832	0.047213
A_42_P620285	Stk25	NM_184049	373542	-0.26	-1.832	0.002362
A_44_P537090	Asb18_predicted	XM_001066456		-0.26	-1.832	0.002209
A_44_P543808	XM_228157	XM_228157		-0.26	-1.832	0.005482
A_44_P354030	A_44_P354030	A_44_P354030		-0.26	-1.832	0.043326
A_44_P607632	LOC691678	XM_001079243	691678	-0.26	-1.832	0.025308
A_42_P498868	LOC301506	XM_238588		-0.26	-1.832	0.037373
A_44_P375490	Pik3cg_predicted	XM_234053		-0.26	-1.832	0.0073
A_44_P276673	LOC688622	XM_226759		-0.26	-1.833	0.042535
A_44_P579264	CF109910	CF109910		-0.26	-1.833	0.104069
A_44_P831689	A_44_P831689	A_44_P831689		-0.26	-1.833	0.007237
A_44_P242374	AA996913	AA996913	360668	-0.26	-1.833	0.012591
A_44_P140710	AA818118	AA818118	361545	-0.26	-1.834	0.005625
A_44_P435189	AI104536	AI104536	501529	-0.26	-1.834	0.048548
A_44_P417749	AA923941	AA923941		-0.26	-1.834	0.008792
A_44_P514522	BI285007	BI285007	292019	-0.26	-1.834	0.016767
A_44_P172168	AI502605	AI502605		-0.26	-1.834	0.12112
A_44_P255458	XM_344344	XM_344344		-0.26	-1.835	0.01428
A_44_P270015	XM_236355	XM_236355		-0.26	-1.835	0.023819
A_43_P16279	Col9a1	XM_223124	305104	-0.26	-1.835	0.014695
A_44_P271900	RGD1310926_predicted	XM_217903		-0.26	-1.835	0.051468
A_44_P388763	Olr1535_predicted	NM_001000945	405287	-0.26	-1.835	0.009019
A_43_P18464	RGD1562533_predicted	XM_001060522		-0.26	-1.835	0.016322
A_44_P476960	AA900854	AA900854	406864	-0.26	-1.836	0.001991
A_44_P366554	BG380473	BG380473	287299	-0.26	-1.836	0.00415
A_44_P975437	Cldn4	NM_001012022	304407	-0.26	-1.836	0.001741
A_44_P701913	TC545016	TC545016		-0.26	-1.836	0.023294
A_44_P333526	AA819652	AA819652		-0.26	-1.836	0.009009
A_44_P924208	A_44_P924208	A_44_P924208		-0.26	-1.837	0.010266
A_44_P527144	St6gal2	XM_236826	301155	-0.26	-1.837	0.026638
A_44_P437001	BF407099	BF407099		-0.26	-1.837	0.045826
A_44_P955563	BG377332	BG377332		-0.26	-1.837	0.011319
A_44_P227316	A_44_P227316	A_44_P227316		-0.26	-1.837	0.005178

A_44_P831755	LOC500825	NM_001025052	500825	-0.26	-1.838	0.029164
A_43_P11123	AW529623	AW529623		-0.26	-1.839	0.080143
A_44_P963682	TC527400	TC527400		-0.26	-1.839	0.003488
A_44_P468560	Lmx1a_predicted	XM_222902		-0.26	-1.839	0.003789
A_44_P161736	A_44_P161736	A_44_P161736		-0.26	-1.839	0.028578
A_44_P199659	RGD1308795_predicted	XM_236392	315804	-0.26	-1.839	0.003187
A_44_P368065	Adprhl1	NM_001013054	290880	-0.26	-1.839	0.001091
A_43_P20743	Elmo1_predicted	XM_341532		-0.26	-1.839	0.000775
A_44_P297680	RGD1562230_predicted	XM_342133	361839	-0.26	-1.840	0.001233
A_44_P555625	RGD1565470_predicted	XM_001068791		-0.26	-1.840	0.050189
A_44_P179622	BM986233	BM986233	64202	-0.26	-1.840	0.019726
A_44_P915917	DY314586	DY314586		-0.26	-1.840	0.002169
A_43_P12427	Gabrg3	NM_024370	79211	-0.26	-1.840	0.009676
A_42_P840836	Hnt	NM_017354	50864	-0.26	-1.840	0.008132
A_44_P1030081	LOC315712	NM_001014091	315712	-0.26	-1.840	0.013451
A_44_P396507	Pelp1	NM_001024270	360552	-0.27	-1.841	0.047546
A_44_P914261	TC535495	TC535495		-0.27	-1.841	0.000611
A_44_P359825	AI178993	AI178993	116698	-0.27	-1.841	0.006792
A_44_P776488	TC554985	TC554985		-0.27	-1.841	0.021857
A_44_P229870	Zzef1_predicted	XM_237787	287476	-0.27	-1.841	0.0009
A_44_P115842	AA965028	AA965028	113947	-0.27	-1.842	0.004075
A_44_P151533	Olr150_predicted	NM_001000739	405001	-0.27	-1.842	0.036091
A_44_P850143	AW530281	AW530281	680117	-0.27	-1.842	0.000927
A_43_P22452	XM_218295	XM_218295		-0.27	-1.842	0.007271
A_44_P388121	BG375463	BG375463	360480	-0.27	-1.842	0.011401
A_44_P791571	AABR03006190	AABR03006190		-0.27	-1.842	0.029874
A_44_P753355	AI502658	AI502658	117557	-0.27	-1.842	0.018287
A_44_P140748	BF411461	BF411461		-0.27	-1.842	0.0466
A_44_P656575	TC545665	TC545665		-0.27	-1.843	0.069934
A_44_P426792	RT1-M6-2	NM_001008853	365527	-0.27	-1.843	0.001634
A_44_P628484	TC551486	TC551486		-0.27	-1.843	0.001682
A_44_P788147	LOC679267	XM_001055528		-0.27	-1.844	0.001475
A_44_P387677	MGC94464	NM_001007647	297337	-0.27	-1.844	0.000777
A_44_P341977	E030032D13Rik	NR_002149		-0.27	-1.844	0.004384
A_44_P136027	BQ199246	BQ199246	296637	-0.27	-1.844	0.026601
A_44_P166206	Nostrin	NM_001024260	311111	-0.27	-1.844	0.002786
A_44_P431792	BE097492	BE097492	366951	-0.27	-1.845	0.042611
A_42_P623358	Ren1	NM_012642	24715	-0.27	-1.845	0.013228
A_44_P720560	CK597214	CK597214		-0.27	-1.845	0.002288
A_44_P123450	Olr663_predicted	NM_001000346	295869	-0.27	-1.845	0.003843
A_44_P820981	AW920863	AW920863		-0.27	-1.845	0.006244
A_44_P229161	XM_344378	XM_344378		-0.27	-1.846	0.028552
A_44_P546108	Ehf_predicted	XM_215790		-0.27	-1.846	0.000277
A_44_P441729	X13119	X13119		-0.27	-1.846	0.006122
A_43_P15528	Hrh2	NM_012965	25461	-0.27	-1.846	0.022148
A_44_P980148	TC549743	TC549743		-0.27	-1.846	0.01398
A_42_P758492	Pitx3	NM_019247	29609	-0.27	-1.847	0.004146
A_44_P393362	Olr423_predicted	NM_001000391	296685	-0.27	-1.847	0.014764
A_44_P815210	BF387773	BF387773		-0.27	-1.847	0.004851
A_44_P321059	A_44_P321059	A_44_P321059		-0.27	-1.848	0.044735
A_44_P438627	RGD1559928_predicted	XM_001062576		-0.27	-1.848	0.013095
A_42_P555365	Slc17a3	NM_153622	266730	-0.27	-1.848	0.003501
A_43_P15238	Apoc4	X56190	83418	-0.27	-1.849	0.004659
A_44_P391964	TC542405	TC542405		-0.27	-1.849	0.013731
A_44_P523372	Nedd9	NM_001011922	291044	-0.27	-1.849	0.000408
A_44_P577108	Rasgef1c_predicted	XM_340792		-0.27	-1.849	0.030979
A_44_P305922	CA507578	CA507578	29541	-0.27	-1.850	0.032295
A_44_P486055	Rab27b	NM_053459	84590	-0.27	-1.850	0.003941
A_44_P484909	P2rx4	NM_031594	29659	-0.27	-1.850	0.00458
A_44_P810617	TC527012	TC527012		-0.27	-1.851	0.000946
A_44_P112568	LOC680590	XM_001057907	680590	-0.27	-1.851	0.027738
A_44_P415945	BF407551	BF407551		-0.27	-1.851	0.019673
A_44_P119956	BF417776	BF417776		-0.27	-1.851	0.000266

A_44_P790705	LOC498396	XM_001065133		-0.27	-1.851	0.013654
A_44_P688603	LOC680068	XM_001055549	680068	-0.27	-1.851	0.010768
A_44_P368901	RGD1566408_predicted	XM_575928		-0.27	-1.852	0.036509
A_44_P939143	LOC501248	XM_576667		-0.27	-1.852	0.255294
A_44_P337207	Olr168_predicted	NM_001000174	293315	-0.27	-1.852	0.012827
A_42_P669907	Slc16a7	NM_017302	29735	-0.27	-1.852	0.000864
A_44_P396422	RGD1563325_predicted	XM_343187		-0.27	-1.852	0.001939
A_44_P307033	LOC686477	XR_008948	680033	-0.27	-1.852	0.000564
A_44_P557428	Cacna1d	AF370009	29716	-0.27	-1.852	0.007766
A_44_P632635	BE103109	BE103109		-0.27	-1.853	0.02032
A_43_P20779	LOC500528	NM_001014067	500528	-0.27	-1.853	0.00032
A_44_P327665	AI071717	AI071717		-0.27	-1.853	0.004194
A_44_P393688	XM_344204	XM_344204		-0.27	-1.854	0.008423
A_44_P524039	BQ196053	BQ196053	363139	-0.27	-1.854	0.003664
A_44_P369822	C1rl	NM_001002804	408246	-0.27	-1.854	0.007378
A_44_P292518	Mcpt4	NM_019321	54270	-0.27	-1.854	0.010439
A_44_P198628	Olr1702_predicted	NM_001001374	294157	-0.27	-1.854	0.01618
A_44_P233425	Nedd9	NM_001011922	291044	-0.27	-1.855	0.001218
A_44_P153901	Olr1061_predicted	NM_001000065	288829	-0.27	-1.855	0.009954
A_42_P748948	Gck	NM_012565	24385	-0.27	-1.855	0.014947
A_44_P590410	A_44_P590410	A_44_P590410		-0.27	-1.855	0.207546
A_44_P173156	Taf5_predicted	XM_219965		-0.27	-1.856	0.005114
A_44_P144809	XM_342248	XM_342248		-0.27	-1.856	0.01362
A_44_P323053	AI556317	AI556317	295425	-0.27	-1.856	0.001273
A_44_P132937	Rph3a	NM_133518	171039	-0.27	-1.856	0.116877
A_44_P507773	RGD1565037_predicted	BG663014	498398	-0.27	-1.856	0.007966
A_42_P582044	Mrps30_predicted	XM_215487		-0.27	-1.856	0.000291
A_44_P933805	TC563973	TC563973		-0.27	-1.857	0.001873
A_44_P287842	Cyp2c70	NM_138512	171518	-0.27	-1.857	0.005324
A_44_P667790	ENSRNOT00000020215	ENSRNOT00000020215		-0.27	-1.857	0.002502
A_44_P269194	Olr1569_predicted	NM_001000042	287969	-0.27	-1.857	0.00111
A_44_P1013405	Acy3	NM_001009603	293653	-0.27	-1.857	0.00198
A_42_P759993	Mefv	NM_031634	58923	-0.27	-1.858	0.127722
A_44_P313325	AA819534	AA819534	252928	-0.27	-1.858	0.015914
A_44_P296631	RGD1306495	NM_001009413	312437	-0.27	-1.859	0.008227
A_44_P482563	LOC502310	XM_577774	502310	-0.27	-1.859	0.034197
A_44_P778808	TC530561	TC530561		-0.27	-1.859	0.010209
A_44_P307220	XM_235060	XM_235060		-0.27	-1.859	0.012959
A_44_P405480	A_44_P405480	A_44_P405480		-0.27	-1.859	0.012606
A_42_P791436	Gfra2	NM_012750	25136	-0.27	-1.859	0.038252
A_44_P729156	A_44_P729156	A_44_P729156		-0.27	-1.859	0.073199
A_44_P271586	Acat2	AY325187	308100	-0.27	-1.859	0.003981
A_44_P1016217	Ncor1	XM_577103	54299	-0.27	-1.860	0.002007
A_44_P910962	DV727545	DV727545		-0.27	-1.860	0.07515
A_43_P22401	RGD1565856_predicted	XM_215012		-0.27	-1.860	0.004659
A_44_P534334	tGap1	NM_001007635	294892	-0.27	-1.860	0.003267
A_44_P276106	Apc	NM_012499	24205	-0.27	-1.861	0.004603
A_44_P160180	AI501707	AI501707	24968	-0.27	-1.861	0.025903
A_44_P449256	CA509611	CA509611	297444	-0.27	-1.861	0.015832
A_42_P606073	Acadvl	NM_012891	25363	-0.27	-1.861	0.001442
A_44_P555201	Apoh	NM_001009626	287774	-0.27	-1.862	0.045872
A_44_P991864	Ece1	NM_053596	94204	-0.27	-1.862	0.000274
A_44_P179858	Insr	NM_017071	24954	-0.27	-1.863	0.000732
A_44_P438426	Scara5_predicted	XM_224299	305974	-0.27	-1.863	0.000283
A_44_P682087	AI101197	AI101197		-0.27	-1.863	0.000629
A_44_P525088	Gpr43	NM_001005877	292794	-0.27	-1.863	0.005745
A_44_P372011	BE115624	BE115624	288591	-0.27	-1.863	0.018551
A_44_P134059	AA943011	AA943011	315600	-0.27	-1.863	0.018533
A_44_P1040503	Pctp	NM_017225	29510	-0.27	-1.863	0.00265
A_44_P425322	AI102750	AI102750	297406	-0.27	-1.864	0.013224
A_44_P194166	Dhh	XM_343327	84380	-0.27	-1.864	0.055327
A_44_P501410	RGD1563743_predicted	XM_344272		-0.27	-1.864	0.004811
A_44_P114433	XM_344359	XM_344359		-0.27	-1.864	0.035909

A_44_P135238	Wnt7a	XM_342723	114850	-0.27	-1.864	0.011168
A_43_P20758	CB546077	CB546077	364405	-0.27	-1.865	0.006378
A_43_P13230	Ehhadh	NM_133606	171142	-0.27	-1.865	0.016519
A_44_P530813	Casp3	NM_012922	25402	-0.27	-1.865	0.012242
A_44_P1044760	Rapgef4	XM_215985	252857	-0.27	-1.866	0.022729
A_42_P496739	Stap2	NM_001025026	363334	-0.27	-1.866	0.001337
A_44_P358943	Asph_predicted	XM_232675		-0.27	-1.866	0.005899
A_44_P152048	LOC501463	XM_576873	501463	-0.27	-1.866	0.007702
A_44_P186951	Abca7	NM_207598	299609	-0.27	-1.866	0.008486
A_44_P639330	TC527103	TC527103		-0.27	-1.866	0.002312
A_44_P230106	AA899244	AA899244		-0.27	-1.867	0.035352
A_44_P850353	BF563221	BF563221		-0.27	-1.867	0.004142
A_44_P385905	AA817805	AA817805	367562	-0.27	-1.867	0.012267
A_44_P376112	X72758	X72758		-0.27	-1.867	0.008483
A_44_P936139	CK358538	CK358538		-0.27	-1.867	0.002757
A_44_P283650	Fam31b_predicted	XM_222678		-0.27	-1.868	0.001746
A_44_P121003	Aspa	NM_024399	79251	-0.27	-1.868	0.001377
A_44_P1044244	RGD1306583	NM_001013908	294709	-0.27	-1.868	0.005709
A_44_P460604	AI145395	AI145395		-0.27	-1.869	0.000902
A_44_P1047158	Tnip2	NM_001024771	305451	-0.27	-1.869	0.060016
A_44_P1049865	Crot	NM_031987	83842	-0.27	-1.869	0.003014
A_44_P137633	Adora1	NM_017155	29290	-0.27	-1.869	0.025052
A_42_P641915	Adck2_predicted	XM_231655		-0.27	-1.869	0.000877
A_44_P538740	A_44_P538740	A_44_P538740		-0.27	-1.870	0.000523
A_44_P536567	A_44_P536567	A_44_P536567		-0.27	-1.870	0.016345
A_44_P693754	CR471794	CR471794		-0.27	-1.870	0.01507
A_44_P196389	Glra1	NM_013133	25674	-0.27	-1.870	0.005241
A_44_P728872	CV075306	CV075306	364953	-0.27	-1.870	0.001245
A_44_P609842	TC540415	TC540415		-0.27	-1.870	0.142108
A_44_P158050	RGD1564887_predicted	XM_574677	499363	-0.27	-1.871	0.037695
A_44_P207319	AA801136	AA801136	499866	-0.27	-1.871	0.06318
A_44_P387160	A_44_P387160	A_44_P387160		-0.27	-1.871	0.071854
A_44_P1026123	XM_341806	XM_341806		-0.27	-1.871	0.006696
A_44_P769654	A_44_P769654	A_44_P769654		-0.27	-1.872	0.012591
A_44_P268521	AI237056	AI237056	307234	-0.27	-1.872	0.01146
A_44_P264977	XM_215482	XM_215482		-0.27	-1.872	0.001225
A_44_P552280	AA963232	AA963232	361991	-0.27	-1.872	0.007292
A_43_P17609	Pdlim2	NM_001007622	290354	-0.27	-1.873	0.004244
A_44_P107308	Gfra1	NM_012959	25454	-0.27	-1.873	0.002472
A_44_P556075	AA900435	AA900435		-0.27	-1.873	0.090275
A_44_P639634	TC545544	TC545544		-0.27	-1.873	0.018693
A_43_P10563	BF391881	BF391881		-0.27	-1.873	0.005752
A_44_P475026	BM384018	BM384018	292710	-0.27	-1.873	0.012806
A_43_P19690	Zbtb5_predicted	XM_233005		-0.27	-1.873	0.038992
A_44_P428886	LOC689060	XM_001068051		-0.27	-1.874	0.009957
A_44_P929574	TC552799	TC552799		-0.27	-1.874	0.021419
A_42_P633019	Grifin	NM_057187	117130	-0.27	-1.874	0.115884
A_44_P259961	Runx2	XM_346016	367218	-0.27	-1.875	0.001722
A_44_P396370	RGD1359529	NM_001014193	362626	-0.27	-1.876	0.001705
A_42_P489478	Cox7b	NM_182819	303393	-0.27	-1.876	0.000696
A_43_P20496	Grid2ip_predicted	XM_239604		-0.27	-1.876	0.046745
A_44_P544748	BE100832	BE100832	308986	-0.27	-1.876	0.006708
A_43_P21870	Oact2	XM_234011		-0.27	-1.877	0.055323
A_44_P529411	AY383682	AY383682		-0.27	-1.878	0.016519
A_44_P542339	Epha1_predicted	XM_001072130		-0.27	-1.878	0.014921
A_44_P490231	RGD1561662_predicted	XM_236949	301232	-0.27	-1.878	0.008909
A_44_P468618	Spfh1_predicted	XM_219858		-0.27	-1.878	0.001504
A_44_P135857	XM_239589	XM_239589		-0.27	-1.878	0.004361
A_44_P236482	RGD1562196_predicted	XM_345252		-0.27	-1.879	0.025314
A_44_P167632	AI013756	AI013756	293476	-0.27	-1.880	0.006176
A_44_P104784	Olr578_predicted	NM_001000568	366109	-0.27	-1.880	0.013501
A_44_P863259	RGD1566399_predicted	XR_008228	688634	-0.27	-1.880	0.006379
A_44_P293264	RGD1559481_predicted	XM_234034		-0.27	-1.880	0.000244

A_44_P340302	RGD1561558_predicted	XM_228669	317314	-0.27	-1.880	0.02652
A_44_P229451	A_44_P229451	A_44_P229451		-0.27	-1.880	0.003324
A_44_P812382	LOC679252	XM_001054661		-0.27	-1.880	0.001002
A_44_P300084	TC567561	TC567561		-0.27	-1.881	0.0007
A_44_P388745	Olr1343_predicted	NM_001001088	405982	-0.27	-1.881	0.001599
A_44_P429027	RGD1359334	NM_001007638	295265	-0.27	-1.881	0.005775
A_44_P506756	CA505255	CA505255	192235	-0.27	-1.881	0.016043
A_44_P595141	TC562467	TC562467		-0.27	-1.881	0.038844
A_44_P112082	RGD1305335_predicted	XM_218905	293107	-0.27	-1.882	0.01089
A_44_P469176	AI535415	AI535415	361967	-0.27	-1.882	0.021005
A_44_P1043302	Crb3	NM_001025661	301112	-0.27	-1.882	0.000685
A_42_P684538	Slc2a1	NM_138827	24778	-0.27	-1.882	0.000301
A_44_P694928	BM392039	BM392039		-0.27	-1.882	0.02138
A_44_P271886	XM_341873	XM_341873		-0.27	-1.882	0.128171
A_44_P216589	AI548221	AI548221	60372	-0.27	-1.883	0.008107
A_44_P223252	Psmc3	NM_031595	29677	-0.27	-1.883	0.012128
A_44_P515421	Ache	X70140	83817	-0.27	-1.883	0.01406
A_44_P318308	Kcnh6	NM_053937	116745	-0.27	-1.884	0.001603
A_44_P650867	AW917197	AW917197		-0.28	-1.884	0.011884
A_44_P255470	A_44_P255470	A_44_P255470		-0.28	-1.884	0.071076
A_44_P318202	LOC684874	XM_001072279		-0.28	-1.884	0.000565
A_44_P215157	Lin7b	NM_021758	60377	-0.28	-1.884	0.005875
A_44_P124982	BI285307	BI285307	408223	-0.28	-1.884	0.010822
A_44_P165196	BI303655	BI303655	25390	-0.28	-1.884	0.017015
A_43_P13172	Stk17b	NM_133392	170904	-0.28	-1.884	0.000635
A_44_P482213	AI030790	AI030790	303563	-0.28	-1.884	0.030487
A_44_P281178	XM_341159	XM_341159		-0.28	-1.884	0.00152
A_43_P23319	Usp13_predicted	XM_001066621		-0.28	-1.884	0.004299
A_44_P124219	Sln	NM_001013247	367086	-0.28	-1.885	0.026129
A_44_P718826	A_44_P718826	A_44_P718826		-0.28	-1.885	0.049215
A_44_P750262	TC531560	TC531560		-0.28	-1.885	0.001878
A_44_P698805	RGD1563444_predicted	XM_575945		-0.28	-1.886	0.016204
A_42_P689873	Cmtm8	NM_198754	301045	-0.28	-1.886	0.003709
A_44_P290327	MGC94881	NM_001004234	297708	-0.28	-1.886	0.002304
A_44_P448138	LOC363434	XM_343756		-0.28	-1.886	0.326729
A_44_P754597	TC534293	TC534293		-0.28	-1.886	0.003746
A_44_P407787	Edf1_predicted	XM_215993		-0.28	-1.886	0.000222
A_44_P593721	TC564197	TC564197		-0.28	-1.886	0.012852
A_44_P335402	Mocos_predicted	XM_341584		-0.28	-1.887	0.004596
A_44_P791953	TC521717	TC521717		-0.28	-1.887	0.000531
A_44_P528621	Hadhsc	NM_057186	113965	-0.28	-1.887	0.002685
A_44_P476583	AA858942	AA858942		-0.28	-1.887	0.000433
A_44_P342606	A_44_P342606	A_44_P342606		-0.28	-1.888	0.003217
A_44_P227221	RGD1564206_predicted	XM_217680	308021	-0.28	-1.889	0.002512
A_44_P331065	XM_236353	XM_236353		-0.28	-1.889	0.052832
A_44_P1058735	TC520163	TC520163		-0.28	-1.889	0.006034
A_44_P252698	LOC365909	AF058787	365909	-0.28	-1.890	0.001902
A_44_P147142	XM_223739	XM_223739		-0.28	-1.890	0.002088
A_44_P430027	AI233208	AI233208	260416	-0.28	-1.890	0.043057
A_44_P304966	Ankk1_predicted	XM_345937		-0.28	-1.891	0.001016
A_42_P528580	Hddc3_predicted	XM_218817		-0.28	-1.891	0.001384
A_44_P428854	LOC685222	XM_001062874		-0.28	-1.891	0.033529
A_44_P286788	LOC500904	XM_576306	500904	-0.28	-1.891	0.003254
A_44_P504285	Pitpnm3_predicted	XM_001080102		-0.28	-1.891	0.001997
A_44_P469809	Adcyap1r1	NM_133511	24167	-0.28	-1.891	0.019894
A_43_P19437	Rhbdl2_predicted	XM_216520		-0.28	-1.892	0.001053
A_44_P462621	AF074962	AF074962		-0.28	-1.892	0.004137
A_44_P869559	TC524444	TC524444		-0.28	-1.893	0.007399
A_43_P15978	Fgf4	NM_053809	116499	-0.28	-1.893	0.003515
A_44_P215689	XM_343744	XM_343744		-0.28	-1.894	0.323596
A_44_P126360	A_44_P126360	A_44_P126360		-0.28	-1.894	0.00881
A_44_P293633	BF405109	BF405109	353251	-0.28	-1.894	0.013552
A_44_P116738	XM_344182	XM_344182		-0.28	-1.895	0.002389

A_44_P569163	A_44_P569163	A_44_P569163		-0.28	-1.895	0.001612
A_44_P930723	RGD1560818_predicted	XM_574407		-0.28	-1.895	0.014114
A_44_P194404	Pitpnm1	NM_001008369	361694	-0.28	-1.895	0.000215
A_44_P824666	TC526534	TC526534		-0.28	-1.895	0.001446
A_42_P513859	BF550844	BF550844		-0.28	-1.895	0.011137
A_44_P930192	TC556333	TC556333		-0.28	-1.896	0.012863
A_44_P918747	TC530342	TC530342		-0.28	-1.896	0.001302
A_43_P16771	LOC682869	XM_001061265		-0.28	-1.896	0.001851
A_43_P19284	Bcar3_predicted	XM_227657		-0.28	-1.896	0.000429
A_44_P187649	XM_233465	XM_233465		-0.28	-1.896	0.007921
A_44_P534717	Sbf1_predicted	XM_217021	300147	-0.28	-1.897	0.00454
A_43_P15610	Svs1	NM_199095	297081	-0.28	-1.897	0.009666
A_44_P992780	Ugp2	NM_001024743	289827	-0.28	-1.897	0.009721
A_42_P534068	Adam15	NM_020308	57025	-0.28	-1.897	0.005353
A_44_P869511	TC524157	TC524157		-0.28	-1.897	0.090421
A_44_P328696	RGD1310748_predicted	XM_234063		-0.28	-1.898	0.015495
A_44_P494221	Gja12_predicted	XM_573100	497913	-0.28	-1.898	0.017187
A_44_P537417	AA926196	AA926196		-0.28	-1.898	0.001971
A_44_P285047	Tex10_predicted	XM_001054184		-0.28	-1.898	0.007179
A_44_P717190	TC550157	TC550157		-0.28	-1.900	0.001758
A_44_P456324	AA799453	AA799453	25573	-0.28	-1.901	0.004442
A_44_P196222	Asmt	NM_144759		-0.28	-1.901	0.018096
A_44_P172187	BI303604	BI303604	360704	-0.28	-1.901	0.031368
A_44_P175720	A_44_P175720	A_44_P175720		-0.28	-1.901	0.016447
A_44_P413614	JIK	NM_001024254	304530	-0.28	-1.901	0.001194
A_44_P346383	BF417211	BF417211		-0.28	-1.901	0.07138
A_44_P210507	A_44_P210507	A_44_P210507		-0.28	-1.902	0.036658
A_43_P13851	CB547207	CB547207		-0.28	-1.902	0.005615
A_44_P491470	AA997994	AA997994	24638	-0.28	-1.902	0.007283
A_44_P725407	BG666391	BG666391		-0.28	-1.902	0.011086
A_44_P332890	LOC308670	AB191343	308670	-0.28	-1.902	0.004158
A_44_P174316	BI395801	BI395801	25655	-0.28	-1.902	0.009182
A_44_P278881	RGD1562558_predicted	XM_223985		-0.28	-1.902	0.001508
A_44_P432183	BG153306	BG153306		-0.28	-1.902	0.00908
A_44_P325020	BI296792	BI296792		-0.28	-1.903	0.005697
A_44_P287136	BE120346	BE120346	309595	-0.28	-1.903	0.001847
A_44_P1044227	RGD1561903_predicted	XM_213758	288604	-0.28	-1.903	0.021545
A_43_P19923	RGD1562645_predicted	XM_001073775		-0.28	-1.903	0.010466
A_44_P500309	BQ203469	BQ203469	294513	-0.28	-1.903	0.001334
A_44_P403438	XM_237142	XM_237142		-0.28	-1.903	0.046241
A_44_P144018	BE099798	BE099798	500700	-0.28	-1.903	0.002785
A_44_P638464	TC558364	TC558364		-0.28	-1.904	0.013883
A_44_P636580	RGD1561458_predicted	XM_001062361		-0.28	-1.904	0.005239
A_44_P422346	Gbe1	XM_221747	288333	-0.28	-1.904	0.009765
A_44_P360629	Olr1498_predicted	NM_001000030	287501	-0.28	-1.904	0.000585
A_44_P105474	RGD1561666_predicted	XM_229093		-0.28	-1.904	0.004503
A_44_P324340	BG665267	BG665267	301388	-0.28	-1.904	0.000295
A_44_P532557	LOC304131	NM_001013979	304131	-0.28	-1.904	0.012675
A_44_P959785	ENSRNOT00000046703	ENSRNOT00000046703		-0.28	-1.904	0.006328
A_44_P644032	A_44_P644032	A_44_P644032		-0.28	-1.904	0.005661
A_44_P436336	Mageh1	NM_001013250	367767	-0.28	-1.904	0.056603
A_44_P321053	XM_218228	XM_218228		-0.28	-1.904	0.031879
A_44_P855259	TC563644	TC563644		-0.28	-1.904	0.003787
A_44_P368326	A_44_P368326	A_44_P368326		-0.28	-1.905	0.013529
A_44_P105486	A_44_P105486	A_44_P105486		-0.28	-1.905	0.049554
A_44_P427078	Slc26a11_predicted	XM_340944	360670	-0.28	-1.905	0.002287
A_44_P203914	LOC690350	XM_001074288	690350	-0.28	-1.905	0.000562
A_44_P562830	Dusp15_predicted	XM_001060408		-0.28	-1.906	0.006682
A_44_P370673	LOC301137	XM_236799		-0.28	-1.906	0.00549
A_44_P138689	AA957167	AA957167	293684	-0.28	-1.906	0.000889
A_44_P450929	BQ204539	BQ204539	363982	-0.28	-1.906	0.036027
A_44_P319138	A_44_P319138	A_44_P319138		-0.28	-1.906	0.002339
A_43_P23463	XM_343611	XM_343611		-0.28	-1.907	0.012244

A_44_P500586	BQ190447	BQ190447	304809	-0.28	-1.907	0.011717
A_44_P491450	Slco2b1	NM_080786	140860	-0.28	-1.907	0.018759
A_44_P727972	BI295606	BI295606		-0.28	-1.907	0.013467
A_44_P425619	Pgm3_predicted	XM_343442		-0.28	-1.907	0.003321
A_44_P503303	Taar9	NM_175602	319107	-0.28	-1.907	0.021312
A_44_P511440	Gpr45_predicted	XM_237112		-0.28	-1.908	0.005401
A_44_P727192	BF398845	BF398845		-0.28	-1.908	0.004121
A_44_P207818	AW915168	AW915168	83472	-0.28	-1.909	0.013351
A_43_P11519	Ceacam3	NM_012702	24926	-0.28	-1.909	0.039601
A_42_P468909	Wnk4	NM_175579	287715	-0.28	-1.909	0.000363
A_43_P13836	CB547163	CB547163	54299	-0.28	-1.909	0.002191
A_42_P808426	Ifitm2	NM_030833	114709	-0.28	-1.909	0.016801
A_44_P332041	Arl1	NM_022385	64187	-0.28	-1.909	0.00078
A_44_P151295	AA925555	AA925555	312709	-0.28	-1.910	0.014074
A_44_P745177	TC536840	TC536840		-0.28	-1.910	0.001063
A_44_P170738	LOC683220	XM_001064971		-0.28	-1.911	0.013529
A_44_P471173	RGD1309766_predicted	XM_232051	312439	-0.28	-1.911	0.00028
A_44_P336772	Josd1	NM_001025009	315134	-0.28	-1.911	0.001401
A_44_P260072	Tbxas1	NM_012687	24886	-0.28	-1.912	0.005419
A_44_P184243	AI235576	AI235576	360348	-0.28	-1.912	0.005602
A_43_P20944	RGD1305560_predicted	XM_237329	301561	-0.28	-1.912	0.014189
A_44_P752665	BF401600	BF401600		-0.28	-1.912	0.007921
A_44_P381113	AW434173	AW434173	304666	-0.28	-1.912	0.043044
A_44_P847378	BF415581	BF415581		-0.28	-1.913	0.045138
A_44_P227983	AW253723	AW253723	680308	-0.28	-1.913	0.021261
A_44_P351252	Olr1353_predicted	NM_001000750	405020	-0.28	-1.913	0.012333
A_43_P12265	Slc7a2	NM_022619	64554	-0.28	-1.913	0.039162
A_44_P741030	AA818718	AA818718		-0.28	-1.914	0.009848
A_44_P461640	Klf3_mapped	XM_223423		-0.28	-1.914	0.000488
A_44_P197694	Lhb	U25802	25329	-0.28	-1.914	0.046942
A_44_P673994	A_44_P673994	A_44_P673994		-0.28	-1.914	0.013949
A_44_P366202	Wnt9a_predicted	XM_220556		-0.28	-1.914	0.004205
A_44_P542475	AI556107	AI556107	27064	-0.28	-1.915	0.019955
A_43_P12146	Hnf4a	NM_022180	25735	-0.28	-1.915	0.000488
A_44_P125553	M27315	M27315		-0.28	-1.915	0.004865
A_44_P755803	AI145976	AI145976		-0.28	-1.915	0.031951
A_44_P771330	AW536039	AW536039		-0.28	-1.916	0.00248
A_44_P522047	LOC684871	XM_001072266		-0.28	-1.916	0.002135
A_44_P238286	Olr287	NM_173334	287001	-0.28	-1.916	0.020277
A_43_P12160	Unc5b	NM_022207	60630	-0.28	-1.916	0.00572
A_44_P325589	Olr164_predicted	NM_001000737	404998	-0.28	-1.916	0.010322
A_44_P713833	RGD1560967_predicted	XM_575015	362021	-0.28	-1.917	0.006883
A_44_P111843	RGD1308398	NM_001014053	311803	-0.28	-1.917	0.0006
A_44_P549129	RGD1561983_predicted	XM_001063609		-0.28	-1.917	0.001552
A_44_P174374	Ephx1	NM_012844	25315	-0.28	-1.917	0.001049
A_44_P721879	A_44_P721879	A_44_P721879		-0.28	-1.918	0.022729
A_44_P914452	TC537606	TC537606		-0.28	-1.918	0.000326
A_44_P175719	Bpil1_predicted	XM_215892		-0.28	-1.918	0.009676
A_44_P530060	XM_346120	XM_346120		-0.28	-1.918	0.00103
A_44_P307274	LOC301334	XM_237049	301334	-0.28	-1.919	0.031676
A_42_P640679	Odf1	NM_024126	24610	-0.28	-1.919	0.006617
A_44_P288726	Mbd3l2_predicted	XM_235852		-0.28	-1.919	0.026868
A_44_P306507	Hig1	NM_080902	140937	-0.28	-1.920	0.090661
A_44_P107177	Hrh1	NM_017018	24448	-0.28	-1.920	0.000186
A_44_P611866	RGD1561734_predicted	XM_576628	501201	-0.28	-1.920	0.007733
A_44_P172990	Oprd1	NM_012617	24613	-0.28	-1.921	0.006413
A_44_P654498	TC559522	TC559522		-0.28	-1.921	0.00604
A_44_P537149	AA866246	AA866246	79223	-0.28	-1.921	0.033309
A_44_P109455	Gstm2	NM_177426	24424	-0.28	-1.921	0.000685
A_42_P677628	Klf6	NM_031642	58954	-0.28	-1.922	0.002124
A_44_P229581	Snph_predicted	XM_230716		-0.28	-1.922	0.008395
A_43_P16353	U95154	U95154		-0.28	-1.922	0.047305
A_44_P341244	AA963324	AA963324	690263	-0.28	-1.922	0.067193

A_44_P635831	AA848951	AA848951		-0.28	-1.922	0.004284
A_44_P760182	ENSRNOT00000040046	ENSRNOT00000040046		-0.28	-1.923	0.018573
A_44_P208711	RGD1565423_predicted	XM_343720		-0.28	-1.923	0.126859
A_44_P481026	BM392107	BM392107	113960	-0.28	-1.924	0.004131
A_44_P549753	AA848758	AA848758	113965	-0.28	-1.924	0.021147
A_44_P204423	AI137314	AI137314	353252	-0.28	-1.925	0.001391
A_44_P290693	A_44_P290693	A_44_P290693		-0.28	-1.925	0.000661
A_44_P483564	BQ192627	BQ192627	298084	-0.28	-1.925	0.000444
A_44_P293001	Arse	XM_227066		-0.28	-1.925	0.000806
A_44_P483082	XM_346113	XM_346113		-0.28	-1.925	0.052067
A_44_P541672	XM_346067	XM_346067		-0.28	-1.925	0.0176
A_44_P391620	RGD1562989_predicted	XM_229637	302075	-0.28	-1.926	0.000898
A_44_P358654	RGD1562061_predicted	XM_219062		-0.28	-1.926	0.022658
A_44_P326430	XM_345988	XM_345988		-0.28	-1.926	0.001493
A_44_P1010950	Leng1_predicted	XM_214797		-0.28	-1.926	0.000406
A_44_P588098	AI555277	AI555277		-0.28	-1.927	0.115931
A_44_P1017458	Spata20	NM_199402	360604	-0.28	-1.927	0.00667
A_44_P905354	TC526788	TC526788		-0.28	-1.927	0.010718
A_44_P432358	Ugt1a6	NM_057105	113992	-0.29	-1.928	0.008438
A_44_P220183	Evi1_predicted	XM_226987		-0.29	-1.929	0.002183
A_44_P111507	Tcte1_predicted	XM_001067461		-0.29	-1.929	0.004339
A_44_P102359	Pde7b	NM_080894	140929	-0.29	-1.929	0.002134
A_44_P837006	A_44_P837006	A_44_P837006		-0.29	-1.930	0.021883
A_44_P112311	RGD1565472_predicted	XM_573266		-0.29	-1.930	0.000164
A_44_P808150	TC541992	TC541992		-0.29	-1.931	0.008071
A_44_P436399	RGD1306880_predicted	XM_001073980		-0.29	-1.931	0.001575
A_44_P888530	TC531468	TC531468		-0.29	-1.931	0.021261
A_44_P531956	XM_341765	XM_341765		-0.29	-1.931	0.020899
A_44_P314482	Samd14	NM_001024966	287637	-0.29	-1.931	0.011698
A_44_P944342	LOC499418	NM_001024296	499418	-0.29	-1.931	0.019159
A_44_P450421	XM_234680	XM_234680		-0.29	-1.931	0.003012
A_44_P249578	AI008930	AI008930	360646	-0.29	-1.931	0.001953
A_44_P372061	Cyp2d22	NM_138515	171522	-0.29	-1.932	0.005598
A_44_P496305	Edg2	NM_053936	116744	-0.29	-1.932	0.013082
A_44_P396090	XM_345181	XM_345181		-0.29	-1.932	0.024054
A_44_P395512	Slc25a3	NM_139100	245959	-0.29	-1.933	0.000389
A_43_P15213	XM_341160	XM_341160		-0.29	-1.933	0.001213
A_44_P329239	AA858471	AA858471		-0.29	-1.933	0.013282
A_44_P797611	A_44_P797611	A_44_P797611		-0.29	-1.933	0.030002
A_43_P22519	Spaca3_predicted	XM_213416		-0.29	-1.933	0.092264
A_44_P986848	Sytl2_predicted	XM_001061661		-0.29	-1.934	0.00296
A_44_P214379	Kif16b_predicted	XM_230634		-0.29	-1.934	0.003534
A_44_P140118	XM_344207	XM_344207		-0.29	-1.935	0.056049
A_44_P473475	LOC298346	NM_001024873	298346	-0.29	-1.935	0.059788
A_44_P824534	TC525942	TC525942		-0.29	-1.935	0.0099
A_44_P377337	Cox6b_predicted	XM_344879		-0.29	-1.935	0.064003
A_44_P337501	RGD1309903_predicted	XM_218665	292947	-0.29	-1.936	0.003542
A_44_P197581	BQ200095	BQ200095		-0.29	-1.936	0.01055
A_44_P905751	BF564703	BF564703		-0.29	-1.936	0.032828
A_44_P1003602	LOC300024	XM_216959	300024	-0.29	-1.936	0.008697
A_44_P458945	Olr229_predicted	NM_001000204	293373	-0.29	-1.936	0.025558
A_43_P17026	Riok3_predicted	XM_341578		-0.29	-1.936	0.00307
A_44_P540755	Olr1455_predicted	NM_001000771	405046	-0.29	-1.937	0.007209
A_44_P336304	BQ205045	BQ205045	303666	-0.29	-1.937	0.043281
A_44_P960610	TC556310	TC556310		-0.29	-1.937	0.006784
A_44_P762268	TC525039	TC525039		-0.29	-1.937	0.000539
A_44_P418173	S79794	S79794		-0.29	-1.937	0.000855
A_44_P963064	TC528863	TC528863		-0.29	-1.938	0.001053
A_44_P145191	A_44_P145191	A_44_P145191		-0.29	-1.938	0.040286
A_43_P17374	LOC679462	XM_001056482		-0.29	-1.938	0.00046
A_44_P394737	Kb35	NM_001008809	406227	-0.29	-1.938	0.004734
A_44_P606003	ENSRNOT00000049838	ENSRNOT00000049838		-0.29	-1.939	0.076758
A_44_P715868	TC542913	TC542913		-0.29	-1.939	0.019632

A_44_P1044835	Fgb	NM_020071	24366	-0.29	-1.940	0.006225
A_44_P175734	XM_230869	XM_230869		-0.29	-1.941	0.026784
A_44_P617847	AA946455	AA946455		-0.29	-1.941	0.02903
A_44_P480296	Hadha	NM_130826	170670	-0.29	-1.941	0.000135
A_44_P578505	TC544660	TC544660		-0.29	-1.942	0.014559
A_44_P367779	Cd38	NM_013127	25668	-0.29	-1.942	0.007357
A_44_P271269	AI104443	AI104443	25123	-0.29	-1.943	0.008032
A_44_P360900	Maged1	NM_053409	84469	-0.29	-1.943	0.000407
A_44_P214111	Dmrt2_predicted	XM_219927		-0.29	-1.943	0.012322
A_44_P481927	AI169631	AI169631	25344	-0.29	-1.943	0.042512
A_44_P407600	RGD1309105_predicted	XM_213949		-0.29	-1.944	0.000669
A_44_P301729	Th	NM_012740	25085	-0.29	-1.944	0.00376
A_44_P1019407	Rnf103	NM_053438	84508	-0.29	-1.944	0.000241
A_42_P671635	BF556088	BF556088		-0.29	-1.944	0.000185
A_44_P390241	AI045279	AI045279	114638	-0.29	-1.944	0.004139
A_44_P131296	RGD1563281_predicted	XM_220923		-0.29	-1.944	0.001946
A_44_P314771	BQ211804	BQ211804		-0.29	-1.944	0.00803
A_44_P876303	TC549002	TC549002		-0.29	-1.944	0.048795
A_43_P10856	LOC682750	XM_001062917		-0.29	-1.944	0.004979
A_42_P477222	Crygd	NM_033095	24278	-0.29	-1.945	0.019774
A_44_P605461	LOC257642	NM_147136		-0.29	-1.945	0.00109
A_44_P478096	LOC687046	XM_001076853		-0.29	-1.945	0.067971
A_44_P497985	RGD1561652_predicted	XM_243980	316039	-0.29	-1.945	0.000297
A_44_P334166	AA818858	AA818858		-0.29	-1.946	0.025368
A_44_P128029	Olr1462_predicted	NM_001001097	405993	-0.29	-1.946	0.070228
A_44_P144564	Chrm4	XM_345403		-0.29	-1.946	0.001685
A_44_P977035	TC527000	TC527000		-0.29	-1.947	0.013883
A_44_P553031	XM_344362	XM_344362		-0.29	-1.947	0.054403
A_44_P729113	RGD1560971_predicted	XM_578420		-0.29	-1.947	0.007565
A_44_P561102	Camk2b	NM_001042354	24245	-0.29	-1.947	0.001927
A_44_P222238	LOC500350	XM_575703		-0.29	-1.947	0.006944
A_44_P527521	RGD1306058_predicted	XM_341506	361224	-0.29	-1.948	0.000887
A_44_P443556	A_44_P443556	A_44_P443556		-0.29	-1.948	0.072795
A_43_P18389	BF566406	BF566406	366244	-0.29	-1.948	0.002502
A_44_P601953	BF283353	BF283353		-0.29	-1.948	0.005501
A_44_P730837	TC541817	TC541817		-0.29	-1.949	0.00382
A_44_P575424	MGC108776	NM_001017466	361916	-0.29	-1.949	0.001314
A_44_P478534	XM_238578	XM_238578		-0.29	-1.949	0.003142
A_44_P482158	BE099470	BE099470	500795	-0.29	-1.949	0.003861
A_44_P423710	Olr1622_predicted	NM_001000838	405127	-0.29	-1.949	0.007259
A_44_P295576	A_44_P295576	A_44_P295576		-0.29	-1.950	0.015741
A_44_P449910	DY309915	DY309915	497798	-0.29	-1.950	0.010249
A_44_P376423	AI599243	AI599243	291103	-0.29	-1.951	0.00961
A_44_P498735	Olr1418_predicted	NM_001000008	287313	-0.29	-1.951	0.006556
A_44_P453002	BQ206830	BQ206830		-0.29	-1.951	0.027524
A_44_P498890	Acadvl	NM_012891	25363	-0.29	-1.951	0.001576
A_44_P438135	Pacs1	AF076184	171444	-0.29	-1.951	0.103277
A_44_P113954	Olr608_predicted	NM_001000655	404853	-0.29	-1.952	0.081972
A_44_P381384	AW143018	AW143018		-0.29	-1.952	0.002337
A_44_P541144	Igsf9_predicted	XM_222905		-0.29	-1.952	0.018693
A_44_P374062	Pdlim7	NM_173125	286908	-0.29	-1.952	0.007806
A_44_P770432	A_44_P770432	A_44_P770432		-0.29	-1.952	0.048349
A_44_P338703	AW918458	AW918458		-0.29	-1.952	0.000429
A_44_P557826	RGD1566040_predicted	XM_344619		-0.29	-1.953	0.013946
A_43_P16374	Tpcr12	XM_001070532		-0.29	-1.953	0.002941
A_44_P655651	TC533179	TC533179		-0.29	-1.953	0.063722
A_44_P932876	LOC364637	NM_001014239	364637	-0.29	-1.953	0.005647
A_44_P201201	Col10a1	XM_001053056	25681	-0.29	-1.953	0.03358
A_44_P731487	RGD1562463_predicted	XM_001074239		-0.29	-1.954	0.006348
A_44_P167205	AI229849	AI229849	360950	-0.29	-1.955	0.020185
A_44_P167909	Ffar1	NM_153304	266607	-0.29	-1.955	0.0109
A_44_P362271	BQ192967	BQ192967	689574	-0.29	-1.955	0.015469
A_44_P184273	Atp1a1	NM_012504	24211	-0.29	-1.955	0.002979

A_44_P355229	AW433846	AW433846	679038	-0.29	-1.955	0.008945
A_44_P204145	RGD1565726_predicted	XM_343506	363167	-0.29	-1.955	0.017154
A_44_P281426	A_44_P281426	A_44_P281426		-0.29	-1.955	0.082659
A_44_P321745	BE113943	BE113943	502894	-0.29	-1.956	0.002538
A_44_P365676	A_44_P365676	A_44_P365676		-0.29	-1.956	0.009259
A_42_P624493	Adamts13_predicted	XM_342395	362091	-0.29	-1.956	0.018212
A_44_P489340	Olr1191	NM_001000981	405336	-0.29	-1.956	0.005379
A_44_P899425	BF403483	BF403483		-0.29	-1.957	0.024597
A_44_P523135	A_44_P523135	A_44_P523135		-0.29	-1.957	0.005826
A_44_P555604	XM_226573	XM_226573		-0.29	-1.957	0.00685
A_44_P508583	LOC684642	XM_001071381		-0.29	-1.957	0.010988
A_42_P624921	XM_219523	XM_219523		-0.29	-1.958	0.000523
A_44_P417729	AA900767	AA900767		-0.29	-1.958	0.000975
A_44_P485260	LOC295618	XR_008470	295618	-0.29	-1.958	0.010344
A_44_P366979	AA926042	AA926042	287470	-0.29	-1.958	0.001714
A_44_P156811	XM_227544	XM_227544		-0.29	-1.958	0.078777
A_44_P640884	TC542560	TC542560		-0.29	-1.958	0.009211
A_44_P431092	Cyp2d22	NM_138515	171522	-0.29	-1.959	0.000669
A_44_P450314	RGD1563860_predicted	XM_576965	501560	-0.29	-1.959	0.000466
A_44_P287753	AA817965	AA817965	338401	-0.29	-1.959	0.001565
A_43_P17902	LOC684135	XM_001069081		-0.29	-1.960	0.019548
A_44_P103966	AI714262	AI714262	292155	-0.29	-1.960	0.003492
A_44_P119160	Papss2_predicted	XM_215288		-0.29	-1.960	0.01874
A_43_P12941	Calcr	NM_053816	116506	-0.29	-1.960	0.048021
A_44_P241650	RGD1565729_predicted	XM_220559	287358	-0.29	-1.961	0.01406
A_44_P859795	BF403973	BF403973		-0.29	-1.961	0.02138
A_44_P494137	Ambn	NM_012900	25376	-0.29	-1.962	0.00336
A_44_P962150	RGD1561983_predicted	XM_001063609		-0.29	-1.962	0.004536
A_44_P684379	TC538942	TC538942		-0.29	-1.962	0.001356
A_44_P296397	AI012613	AI012613		-0.29	-1.963	0.023306
A_44_P365269	Jph4	NM_001003711	445271	-0.29	-1.963	0.002352
A_44_P313734	Trpc3	AF313482	60395	-0.29	-1.964	0.001653
A_44_P154313	XM_224054	XM_224054		-0.29	-1.964	0.023164
A_44_P176372	BE100151	BE100151		-0.29	-1.964	0.007938
A_44_P118291	AW915010	AW915010		-0.29	-1.964	0.00163
A_44_P425324	AI030625	AI030625	297406	-0.29	-1.964	0.002336
A_44_P823790	TC542005	TC542005		-0.29	-1.964	0.005289
A_44_P333544	AA924146	AA924146		-0.29	-1.965	0.004384
A_44_P424496	TC561396	TC561396		-0.29	-1.965	0.005163
A_44_P229540	RGD1561337_predicted	XM_574397	499105	-0.29	-1.965	0.000636
A_44_P520123	Flt1	NM_019306	54251	-0.29	-1.966	0.084794
A_44_P459772	RGD1562257_predicted	XM_235563		-0.29	-1.966	0.006053
A_44_P237475	AABR03057674	AABR03057674		-0.29	-1.966	0.032666
A_43_P15627	Cryba1	XM_340846	25583	-0.29	-1.966	0.009262
A_44_P372810	LOC306464	NM_001025673	306464	-0.29	-1.966	0.003504
A_44_P575763	A_44_P575763	A_44_P575763		-0.29	-1.966	0.002732
A_44_P804447	AA964300	AA964300		-0.29	-1.967	0.002003
A_44_P334900	Gdf6	NM_001013038	252834	-0.29	-1.967	0.006948
A_44_P342841	BG380737	BG380737	361699	-0.29	-1.967	0.002227
A_44_P334904	Mxi1	NM_013160	25701	-0.29	-1.968	0.003659
A_44_P380436	AA818386	AA818386		-0.29	-1.968	0.014959
A_44_P727229	AI230826	AI230826		-0.29	-1.968	0.012416
A_44_P337077	Lhfp	NM_181085	300286	-0.29	-1.969	0.003865
A_44_P229568	Zadh2_predicted	XM_214526		-0.29	-1.969	0.001151
A_42_P563156	AW141285	AW141285	363243	-0.29	-1.969	0.000815
A_42_P632813	Epn3	NM_001024791	360605	-0.29	-1.969	9.16E-05
A_44_P313272	Slc6a13	NM_133623	171163	-0.29	-1.969	0.005267
A_44_P1015804	RGD1310693_predicted	XM_236938		-0.29	-1.970	0.111398
A_44_P898505	RGD1562720_predicted	XM_575549		-0.29	-1.970	0.009793
A_44_P672649	TC530294	TC530294		-0.29	-1.971	0.031719
A_44_P318819	ENSRNOT00000043649	ENSRNOT00000043649		-0.29	-1.971	0.000109
A_42_P739079	Armcx2	NM_001014274	367903	-0.29	-1.972	0.077908
A_44_P103667	AW434086	AW434086		-0.29	-1.972	0.014922

A_44_P1042216	TC555328	TC555328		-0.29	-1.972	0.001174
A_44_P1002320	BF289377	BF289377		-0.29	-1.972	0.001124
A_44_P136679	Isoc1	NM_001014242	364879	-0.29	-1.972	0.000388
A_44_P511665	LOC501283	XM_576698	501283	-0.29	-1.972	0.003189
A_44_P921994	AA963560	AA963560		-0.30	-1.972	0.025846
A_44_P487826	RGD1564306_predicted	XM_229957		-0.30	-1.973	0.016007
A_44_P365146	BI290548	BI290548	303747	-0.30	-1.973	0.001291
A_44_P431914	AI599255	AI599255		-0.30	-1.973	0.004222
A_44_P498795	Ces1	XM_001064425		-0.30	-1.973	0.002368
A_44_P486624	Plekha6_predicted	XM_341118	360842	-0.30	-1.974	0.001021
A_44_P555886	A_44_P555886	A_44_P555886		-0.30	-1.974	0.008072
A_43_P19118	LOC691933	XM_001080175	681849	-0.30	-1.974	0.060545
A_42_P695669	AW915538	AW915538		-0.30	-1.974	0.002119
A_44_P886035	TC546137	TC546137		-0.30	-1.974	0.002045
A_44_P393070	AI044508	AI044508	25247	-0.30	-1.974	0.006143
A_44_P296884	AW917678	AW917678	500419	-0.30	-1.975	0.003128
A_44_P264536	Olr703_predicted	NM_001000359	295902	-0.30	-1.975	0.069212
A_43_P15579	Blr1	NM_053303	29363	-0.30	-1.975	0.027474
A_42_P472375	Pdk2	NM_030872	81530	-0.30	-1.975	0.001054
A_44_P895217	BF543471	BF543471		-0.30	-1.975	0.001264
A_44_P458704	Glul	BC072694	24957	-0.30	-1.975	0.000523
A_44_P182158	Hspa12a_predicted	XM_001065021		-0.30	-1.976	0.000523
A_44_P327526	BE107069	BE107069		-0.30	-1.976	0.010828
A_44_P464539	AI176713	AI176713	58834	-0.30	-1.976	0.042431
A_44_P254503	AA800034	AA800034	81784	-0.30	-1.976	0.045382
A_44_P304323	Ccl5	NM_031116	81780	-0.30	-1.976	0.004227
A_43_P11983	Rgs9	NM_019224	29481	-0.30	-1.976	0.021261
A_44_P365470	Gdnf	NM_019139	25453	-0.30	-1.977	0.006187
A_44_P167638	AW534176	AW534176	80843	-0.30	-1.977	0.10263
A_44_P478162	RGD1562629_predicted	XM_001059612		-0.30	-1.978	0.004712
A_42_P560288	Sdhd	NM_198788	363061	-0.30	-1.978	0.000115
A_43_P10967	Rhot2	NM_181823	287156	-0.30	-1.978	0.000214
A_42_P693191	Spas1	NM_134384	171403	-0.30	-1.978	0.003624
A_44_P1039444	Lasp1	NM_032613	29278	-0.30	-1.979	0.000883
A_44_P420980	AI072953	AI072953	499010	-0.30	-1.979	0.024494
A_44_P1056161	Prkab1	NM_031976	83803	-0.30	-1.979	0.002871
A_44_P549840	RGD1306781_predicted	XM_343995	363720	-0.30	-1.979	0.00458
A_43_P15981	Treh	XM_343380	60576	-0.30	-1.980	0.000309
A_44_P834837	BI286389	BI286389	287702	-0.30	-1.980	0.001053
A_44_P449764	Gria3	NM_032990	29628	-0.30	-1.980	0.009538
A_44_P112362	MGC109491	NM_001009709	362334	-0.30	-1.980	0.002522
A_44_P350189	XM_218392	XM_218392		-0.30	-1.980	0.023023
A_44_P342830	ENSRNOT00000045042	ENSRNOT00000045042		-0.30	-1.980	0.003122
A_44_P499388	A_44_P499388	A_44_P499388		-0.30	-1.981	0.007463
A_44_P383220	BI302848	BI302848	313108	-0.30	-1.981	0.007002
A_44_P185517	XM_221674	XM_221674		-0.30	-1.981	0.049334
A_44_P158451	AW919001	AW919001		-0.30	-1.981	0.020779
A_44_P342212	A_44_P342212	A_44_P342212		-0.30	-1.981	0.003909
A_44_P777483	LOC683626	XM_001066790		-0.30	-1.981	0.002931
A_44_P187881	AA819036	AA819036		-0.30	-1.982	0.004474
A_44_P290074	AI030162	AI030162	365652	-0.30	-1.983	0.001094
A_43_P15154	Ihh	XM_001060415		-0.30	-1.983	0.000328
A_44_P870442	TC561220	TC561220		-0.30	-1.983	0.013721
A_44_P530975	BE097240	BE097240	689025	-0.30	-1.984	0.023298
A_44_P464502	Gfap	NM_017009	24387	-0.30	-1.984	0.005999
A_44_P442648	Olr1501_predicted	NM_001000715	404967	-0.30	-1.984	0.025518
A_44_P146173	AI058658	AI058658	299314	-0.30	-1.985	0.043281
A_44_P400912	LOC298175	XR_008257	298175	-0.30	-1.985	0.001852
A_44_P535612	B2m	CF111193	24223	-0.30	-1.985	0.00257
A_44_P473318	A_44_P473318	A_44_P473318		-0.30	-1.985	0.009121
A_44_P361047	ENSRNOT00000052235	ENSRNOT00000052235		-0.30	-1.985	0.000309
A_44_P565315	TC562080	TC562080		-0.30	-1.985	0.004976
A_44_P527622	A_44_P527622	A_44_P527622		-0.30	-1.986	0.048874

A_44_P223279	BI279017	BI279017	287422	-0.30	-1.986	0.052099
A_44_P356474	A_44_P356474	A_44_P356474		-0.30	-1.986	0.000631
A_44_P1043506	LOC686547	XM_001074155		-0.30	-1.986	0.000692
A_44_P165421	BF401555	BF401555		-0.30	-1.986	0.007149
A_44_P136603	Slc9a3r1	NM_021594	59114	-0.30	-1.987	0.000464
A_43_P22784	BF389338	BF389338		-0.30	-1.987	0.005031
A_44_P398330	RGD1564894_predicted	XM_219626	309227	-0.30	-1.988	0.009593
A_44_P361066	XM_344442	XM_344442		-0.30	-1.988	0.016978
A_44_P401232	BM392175	BM392175	25675	-0.30	-1.988	0.012612
A_44_P405302	Cpxm2_predicted	XM_215106		-0.30	-1.988	0.219562
A_44_P247631	BF388415	BF388415		-0.30	-1.989	0.008141
A_44_P289630	Gria2	NM_017261	29627	-0.30	-1.989	0.040932
A_44_P342598	A_44_P342598	A_44_P342598		-0.30	-1.989	0.002353
A_44_P524755	Olr322_predicted	NM_001000762	405036	-0.30	-1.989	0.001449
A_44_P372874	Klhl23_predicted	XM_230006	311114	-0.30	-1.989	0.028543
A_44_P244257	Fgd4	NM_139263	246174	-0.30	-1.989	0.015128
A_42_P472520	RGD1561113_predicted	XM_575113	499780	-0.30	-1.990	0.001483
A_44_P377902	Sdk2_predicted	XM_340930		-0.30	-1.990	0.000179
A_44_P328727	LOC686694	XM_001075283		-0.30	-1.991	0.013529
A_44_P517929	Cox5b	NM_053586	94194	-0.30	-1.991	0.001008
A_44_P507307	BQ191158	BQ191158	306809	-0.30	-1.991	0.030149
A_44_P363237	Pcyt2	NM_053568	89841	-0.30	-1.992	0.001493
A_44_P252491	Kcnj4	NM_053870	116649	-0.30	-1.992	0.014114
A_44_P115814	AI044097	AI044097		-0.30	-1.993	0.013721
A_44_P391085	Olr1126_predicted	NM_001000880	405179	-0.30	-1.993	0.029027
A_44_P505596	AI232101	AI232101	298584	-0.30	-1.994	0.015361
A_44_P502880	AI060016	AI060016		-0.30	-1.994	0.005098
A_44_P532686	Efnb1	NM_017089	25186	-0.30	-1.994	0.000697
A_44_P398388	RGD1561819_predicted	XM_001058905		-0.30	-1.994	0.00082
A_43_P16369	Olr1413	NM_214821	252887	-0.30	-1.994	0.001434
A_44_P116904	Vmp_predicted	XM_225314		-0.30	-1.994	0.037697
A_43_P12729	Mir16	NM_032615	60418	-0.30	-1.995	0.001356
A_44_P1033367	RGD1305773_predicted	XM_213209	287044	-0.30	-1.995	0.000204
A_44_P1002470	BC087666	BC087666		-0.30	-1.995	0.000469
A_44_P445966	RGD1563955_predicted	XM_343615	363274	-0.30	-1.995	0.106278
A_44_P315905	Acr	NM_012490	24163	-0.30	-1.996	0.029839
A_44_P413229	AA899418	AA899418		-0.30	-1.996	0.003596
A_44_P180099	Cnga4	NM_053496	85258	-0.30	-1.996	0.00048
A_44_P536813	LOC687303	XM_001077913		-0.30	-1.996	0.01774
A_44_P497356	AA893659	AA893659	362778	-0.30	-1.996	0.098494
A_44_P243069	AY325249	AY325249		-0.30	-1.996	0.006649
A_44_P717136	TC533874	TC533874		-0.30	-1.997	0.016192
A_44_P311945	RGD1310351_predicted	XM_216499		-0.30	-1.997	0.000166
A_44_P290403	Dlx2	XM_230986	296499	-0.30	-1.998	0.00365
A_43_P12196	Nmur2	NM_022275	64042	-0.30	-1.998	0.009108
A_43_P12036	Tpo	NM_019353	54314	-0.30	-1.998	0.031491
A_44_P435381	Agxt	NM_030656	24792	-0.30	-1.998	0.022648
A_44_P644356	LOC684767	XM_001071870		-0.30	-1.998	0.001254
A_44_P298064	RGD1563167_predicted	XM_574454	499156	-0.30	-1.998	0.003021
A_44_P923543	TC551587	TC551587		-0.30	-1.998	0.035395
A_44_P791604	TC519958	TC519958		-0.30	-1.999	0.003682
A_44_P491187	BM386513	BM386513	54241	-0.30	-1.999	0.038233
A_44_P647196	A_44_P647196	A_44_P647196		-0.30	-1.999	0.005058
A_44_P1050433	LOC686858	XM_001076046		-0.30	-1.999	0.126799
A_44_P265389	A_44_P265389	A_44_P265389		-0.30	-1.999	0.000844
A_44_P141247	BF562621	BF562621	367153	-0.30	-2.000	0.024819
A_44_P168430	Atp8a1_predicted	XM_223390	289615	-0.30	-2.000	0.000376
A_44_P541692	Hexim2_predicted	XM_221008		-0.30	-2.000	0.000942
A_42_P485243	RGD1563940_predicted	XM_575484		-0.30	-2.000	0.0005
A_44_P473544	XM_343713	XM_343713		-0.30	-2.000	0.028122
A_44_P725497	AI704799	AI704799	286919	-0.30	-2.000	0.004586
A_44_P536724	Rabepk	NM_001024871	296649	-0.30	-2.001	0.00095
A_44_P118993	Mat2a	NM_134351	171347	-0.30	-2.001	0.001154

A_44_P192091	LOC679631	XM_001053800		-0.30	-2.001	0.152403
A_44_P501707	XM_343657	XM_343657		-0.30	-2.001	0.086647
A_43_P13297	Mt1a	NM_138826	24567	-0.30	-2.002	0.02487
A_44_P522396	Etfdh	NM_198742	295143	-0.30	-2.002	0.000255
A_44_P175751	LOC690211	XM_001070526	690211	-0.30	-2.002	0.002493
A_44_P118054	AI176647	AI176647	83537	-0.30	-2.002	0.000843
A_44_P516227	AI177637	AI177637	361182	-0.30	-2.003	0.00939
A_44_P1071270	AF010441	AF010441		-0.30	-2.003	0.002174
A_44_P536247	Olr128_predicted	NM_001000159	293263	-0.30	-2.004	0.08943
A_43_P12005	Resp18	NM_019278	50561	-0.30	-2.004	0.012921
A_44_P448245	Ppp2r3a	XM_576459	363122	-0.30	-2.004	0.001258
A_44_P328338	RGD1311946	NM_001025683	309145	-0.30	-2.005	0.000312
A_43_P20949	Cdr2	NM_001025682	308958	-0.30	-2.005	0.02032
A_44_P113053	AJ225642	AJ225642	288562	-0.30	-2.006	0.002527
A_44_P1043457	Lgals8	NM_053862	116641	-0.30	-2.006	0.000315
A_44_P371259	AA901353	AA901353		-0.30	-2.006	0.002558
A_44_P910410	BG380728	BG380728	304885	-0.30	-2.006	0.014608
A_44_P214017	Pde11a	AB059361	140928	-0.30	-2.007	0.007404
A_44_P183488	Casp7	NM_022260	64026	-0.30	-2.007	0.000936
A_43_P15601	Mat2a	NM_134351	171347	-0.30	-2.007	0.00138
A_44_P550263	Olr1720_predicted	NM_001001116	406015	-0.30	-2.007	0.002787
A_44_P414469	Hadha	NM_130826	170670	-0.30	-2.007	0.00315
A_44_P362706	AI454550	AI454550	312616	-0.30	-2.007	0.007074
A_44_P1026651	Itm2b	NM_001006963	290364	-0.30	-2.009	0.00082
A_44_P503516	AF010444	AF010444		-0.30	-2.009	0.003217
A_44_P854045	TC541129	TC541129		-0.30	-2.009	0.000465
A_42_P476224	Tox_predicted	XM_001063218		-0.30	-2.009	0.001845
A_44_P180067	Cx39	XM_001065136		-0.30	-2.009	0.022349
A_44_P180965	BF399890	BF399890	360508	-0.30	-2.010	0.007654
A_44_P426350	M84361	M84361		-0.30	-2.010	0.001053
A_44_P352016	A_44_P352016	A_44_P352016		-0.30	-2.010	0.040528
A_44_P463345	Vkorc1	AW253787	309004	-0.30	-2.011	0.003944
A_42_P738277	Kcnk6	NM_053806	116491	-0.30	-2.011	0.000141
A_44_P360958	LOC304903	XM_001073420		-0.30	-2.011	0.003279
A_44_P158828	XM_344353	XM_344353		-0.30	-2.011	0.007256
A_44_P487379	XM_223134	XM_223134		-0.30	-2.011	0.007961
A_44_P122826	AA925323	AA925323		-0.30	-2.012	0.001578
A_44_P729081	A_44_P729081	A_44_P729081		-0.30	-2.012	0.005336
A_44_P299362	Olr206_predicted	NM_001000193	293353	-0.30	-2.012	0.023869
A_44_P602114	AI044225	AI044225		-0.30	-2.012	0.010254
A_44_P224459	Olr1750_predicted	NM_212493	294204	-0.30	-2.012	0.001792
A_44_P269184	Olr295_predicted	NM_001000233	293595	-0.30	-2.012	0.069579
A_44_P557716	LOC498467	XM_573724		-0.30	-2.013	0.093592
A_44_P577612	TC557676	TC557676		-0.30	-2.014	0.010114
A_44_P349004	Foxd2_predicted	XM_233422		-0.30	-2.014	0.001153
A_44_P287652	BF553180	BF553180		-0.30	-2.014	0.000548
A_43_P16196	Xylt1	XM_341912	64133	-0.30	-2.015	0.005578
A_43_P14526	CF111111	CF111111		-0.30	-2.015	0.000901
A_44_P440781	RGD1564747_predicted	XM_573736		-0.30	-2.016	0.151826
A_44_P664438	AW918170	AW918170		-0.30	-2.016	0.009594
A_44_P190455	BI274176	BI274176	290284	-0.30	-2.017	0.005094
A_44_P629728	TC561493	TC561493		-0.30	-2.017	0.02485
A_44_P485515	RGD1308422	XM_235886	315451	-0.30	-2.017	0.010926
A_44_P823303	TC558351	TC558351		-0.30	-2.018	0.001124
A_42_P602511	Kcnn4	NM_023021	65206	-0.30	-2.018	0.005498
A_43_P14825	Gabarap	NM_172036	58974	-0.31	-2.018	0.000278
A_44_P792384	TC519727	TC519727		-0.31	-2.018	0.000153
A_44_P363923	AA924474	AA924474		-0.31	-2.019	0.07813
A_44_P321605	LOC501089	XM_576504		-0.31	-2.019	0.222553
A_44_P746189	TC557460	TC557460		-0.31	-2.019	0.003332
A_44_P208477	XM_224121	XM_224121		-0.31	-2.019	0.006213
A_44_P650616	AW914985	AW914985		-0.31	-2.020	0.000663
A_44_P280954	Klk1c10	XM_214947	292858	-0.31	-2.021	0.023388

A_44_P308718	Maged2	NM_080479	113947	-0.31	-2.021	0.063745
A_44_P328094	M57705	M57705	54314	-0.31	-2.021	0.013573
A_44_P554332	Cycs	NM_012839	25309	-0.31	-2.021	0.000247
A_44_P355928	Ctss	NM_017320	50654	-0.31	-2.022	0.006362
A_43_P11451	Ctrb	NM_012536	24291	-0.31	-2.022	0.000782
A_44_P211535	CB547307	CB547307		-0.31	-2.023	0.014719
A_44_P449834	ENSRNOT00000044458	ENSRNOT00000044458		-0.31	-2.023	0.025998
A_43_P10694	Tmem24	NM_001011996	300666	-0.31	-2.023	0.000363
A_44_P712912	AI230632	AI230632		-0.31	-2.024	0.013194
A_44_P179814	Gas6	NM_057100	58935	-0.31	-2.024	0.001314
A_44_P904775	A_44_P904775	A_44_P904775		-0.31	-2.025	0.000736
A_44_P358582	XM_222276	XM_222276		-0.31	-2.027	0.000466
A_42_P662543	Atp6v0d2	NM_001011972	297932	-0.31	-2.027	0.004285
A_44_P520890	XM_343910	XM_343910		-0.31	-2.027	0.001065
A_44_P864127	AW917705	AW917705		-0.31	-2.027	0.053309
A_43_P16157	RGD1559914_predicted	XM_226743	294691	-0.31	-2.027	0.015572
A_44_P379377	Fetub	NM_053348	83928	-0.31	-2.028	0.009882
A_44_P157474	Cbp	NM_022253	64019	-0.31	-2.028	0.002919
A_44_P851639	AI101331	AI101331		-0.31	-2.028	0.007042
A_44_P753601	A_44_P753601	A_44_P753601		-0.31	-2.028	0.053576
A_44_P239379	AI102505	AI102505	171335	-0.31	-2.028	0.000643
A_44_P497754	Glis1_predicted	XM_233779		-0.31	-2.028	0.02649
A_44_P1051650	Sema3b_predicted	XM_001074382		-0.31	-2.028	0.002164
A_44_P527341	LOC680069	XM_001054790	680069	-0.31	-2.028	0.001493
A_44_P845543	TC561882	TC561882		-0.31	-2.029	0.045932
A_44_P526219	AI058977	AI058977	25696	-0.31	-2.029	0.028397
A_42_P592385	Abp1	NM_022935	65029	-0.31	-2.029	0.181406
A_44_P765013	LOC684585	XM_001071132		-0.31	-2.029	0.00025
A_44_P760430	LOC688956	XM_001068968	688956	-0.31	-2.030	0.097347
A_44_P281076	Slc4a2	NM_017048	24780	-0.31	-2.031	0.005202
A_44_P418141	AW520745	AW520745	308576	-0.31	-2.032	0.015921
A_44_P225390	BF282898	BF282898	289197	-0.31	-2.032	0.003114
A_44_P471669	AA818690	AA818690		-0.31	-2.032	0.001964
A_44_P494687	RGD1559633_predicted	XM_231456		-0.31	-2.032	0.009261
A_43_P12085	Dbil5	NM_021596	59116	-0.31	-2.032	0.004002
A_44_P900443	DV719652	DV719652		-0.31	-2.033	0.015065
A_44_P578999	ENSRNOT00000045593	ENSRNOT00000045593		-0.31	-2.033	0.046468
A_44_P312122	RGD1308212	NM_001024234	287466	-0.31	-2.033	0.009855
A_44_P200946	Olr16_predicted	NM_001000694	404899	-0.31	-2.033	0.030209
A_44_P383992	Olr821_predicted	NM_001000842	405131	-0.31	-2.033	0.006229
A_44_P220481	LOC500573	NM_001034949	500573	-0.31	-2.034	0.00064
A_43_P19334	Msh5	NM_212536	294252	-0.31	-2.034	0.052489
A_44_P235462	AI029293	AI029293	24552	-0.31	-2.034	0.034334
A_44_P468153	Thrb	NM_012672	24831	-0.31	-2.035	0.002007
A_44_P1035007	Chrne	NM_017194	29422	-0.31	-2.035	0.022594
A_44_P511275	LOC685904	XM_001065695		-0.31	-2.035	0.159144
A_44_P439956	Itpr2	NM_031046	81678	-0.31	-2.035	0.00304
A_44_P515305	Olr120_predicted	NM_001000154	293256	-0.31	-2.035	0.017121
A_44_P1033294	RGD1563144_predicted	XM_573746	498486	-0.31	-2.036	0.001196
A_44_P255756	RGD1308101_predicted	XM_001070679		-0.31	-2.036	0.011502
A_44_P454532	Ambp	NM_012901	25377	-0.31	-2.036	0.003124
A_43_P11857	Avpr1b	NM_017205	29462	-0.31	-2.037	0.015966
A_44_P519174	Crebl2	NM_001015027	362453	-0.31	-2.037	0.000376
A_44_P415835	BI304009	BI304009	24914	-0.31	-2.037	0.056726
A_44_P517671	Olr1122_predicted	NM_001000882	405181	-0.31	-2.037	0.011504
A_44_P102087	AI712700	AI712700	362708	-0.31	-2.038	0.015799
A_44_P518673	Epb4.9_predicted	XM_001071215		-0.31	-2.039	0.000694
A_44_P272528	BE100578	BE100578		-0.31	-2.039	0.001235
A_44_P189893	RGD1306423_predicted	XM_341614		-0.31	-2.039	0.0028
A_43_P22932	RGD1311600_predicted	XM_225770		-0.31	-2.039	0.00077
A_44_P913570	ENSRNOT00000029763	ENSRNOT00000029763		-0.31	-2.041	0.001985
A_44_P951080	AI137522	AI137522	361671	-0.31	-2.041	0.003245
A_43_P10301	Esd_mapped	XM_214241		-0.31	-2.041	0.000912

A_44_P546782	BF406035	BF406035		-0.31	-2.041	0.02824
A_44_P509426	BQ192067	BQ192067	364706	-0.31	-2.042	0.004594
A_44_P730740	ENSRNOT00000004392	ENSRNOT00000004392		-0.31	-2.042	0.001426
A_44_P558430	XM_224117	XM_224117		-0.31	-2.042	0.013316
A_43_P23041	RGD1304953_predicted	XM_342276		-0.31	-2.042	0.036799
A_44_P535841	BQ204201	BQ204201	298545	-0.31	-2.043	0.004459
A_44_P899239	LOC500015	XM_001069386		-0.31	-2.043	0.011585
A_44_P386891	XM_344376	XM_344376		-0.31	-2.043	0.079131
A_42_P778184	Tnnc2	NM_001037351	296369	-0.31	-2.043	0.01216
A_44_P725232	BF402703	BF402703		-0.31	-2.044	0.009326
A_44_P444039	Acss1_predicted	XM_215897		-0.31	-2.045	0.000243
A_44_P807585	LOC688274	XM_001081727		-0.31	-2.045	0.025524
A_43_P15709	Sra1	NM_183329	252891	-0.31	-2.045	0.000431
A_44_P244546	BM389499	BM389499	680815	-0.31	-2.045	0.004579
A_44_P250291	Sorl1	AB026993	300652	-0.31	-2.046	0.008438
A_42_P667763	Fut2	NM_031635	58924	-0.31	-2.046	0.000633
A_44_P163888	A_44_P163888	A_44_P163888		-0.31	-2.046	0.03688
A_44_P213426	Ncald	NM_001024371	553106	-0.31	-2.046	0.000868
A_43_P15262	Ahr	NM_013149	25690	-0.31	-2.046	0.002514
A_44_P605767	Golga3_predicted	XM_001078840		-0.31	-2.046	0.000662
A_43_P21180	Elmod1_predicted	XR_008801	315670	-0.31	-2.047	0.012591
A_44_P192141	RGD1309403_predicted	XM_227140		-0.31	-2.047	0.004392
A_44_P142709	LOC688490	XM_001067146		-0.31	-2.048	0.011097
A_42_P464489	Sema6b	NM_053471	84609	-0.31	-2.048	0.006598
A_43_P16375	Olr1366	NM_001000980	405335	-0.31	-2.049	0.029574
A_44_P337441	Lipg	NM_001012741	291437	-0.31	-2.050	0.001985
A_44_P504345	AA963109	AA963109		-0.31	-2.051	0.017136
A_44_P493652	AI408425	AI408425	317468	-0.31	-2.051	0.030984
A_44_P438473	XM_342247	XM_342247		-0.31	-2.051	0.000658
A_44_P193545	RGD1305677	NM_001011557	293113	-0.31	-2.052	0.001999
A_44_P988400	CF111186	CF111186		-0.31	-2.052	0.000337
A_44_P210307	Olr1687	NM_001000508	309574	-0.31	-2.053	0.010618
A_44_P702052	TC533989	TC533989		-0.31	-2.053	0.000365
A_42_P495969	Apoc1	NM_012824	25292	-0.31	-2.053	0.001405
A_44_P354244	Epb4.1l4b_predicted	XM_345535	366376	-0.31	-2.054	0.005769
A_44_P133393	XM_233316	XM_233316		-0.31	-2.054	0.003618
A_43_P16370	Olr728	NM_001000363	295917	-0.31	-2.054	0.016619
A_44_P492214	Eps8l3_predicted	XM_215677		-0.31	-2.055	0.000221
A_44_P791286	A_44_P791286	A_44_P791286		-0.31	-2.055	0.001392
A_44_P304297	Taar5	NM_001009650	294123	-0.31	-2.055	0.026975
A_44_P600286	RGD1565539_predicted	XM_575056	499723	-0.31	-2.055	0.257353
A_44_P375412	RGD1565441_predicted	XM_228462		-0.31	-2.055	0.130295
A_44_P104652	Slc38a2	NM_181090	29642	-0.31	-2.056	0.000253
A_42_P532117	Retn	NM_144741	246250	-0.31	-2.056	0.006812
A_44_P777710	DY472288	DY472288		-0.31	-2.056	0.000307
A_44_P806809	Sh3bgrl_predicted	XM_001060518		-0.31	-2.057	0.000398
A_44_P277151	BQ200021	BQ200021		-0.31	-2.058	0.011001
A_42_P758052	Acp6	NM_001031645	295305	-0.31	-2.058	0.000947
A_44_P744892	LOC300308	NM_001013952	300308	-0.31	-2.058	0.014816
A_44_P246318	RGD1564984_predicted	XM_220274		-0.31	-2.059	0.002952
A_44_P255784	Six6_predicted	XM_234288		-0.31	-2.059	0.0178
A_43_P10807	CB556383	CB556383		-0.31	-2.059	0.007598
A_44_P513511	RGD1565601_predicted	XM_226242		-0.31	-2.059	0.005831
A_43_P13137	Tas1r3	NM_130818	170634	-0.31	-2.060	0.004168
A_43_P13155	Xtrp3	NM_133296	113918	-0.31	-2.060	0.006812
A_44_P753662	AA997458	AA997458	362224	-0.31	-2.060	0.005117
A_44_P557354	Gsc	XM_343101	317715	-0.31	-2.061	0.010497
A_44_P769875	RGD1561153_predicted	XM_574117	498834	-0.31	-2.061	0.00023
A_43_P18730	LOC304000	NM_001006990	304000	-0.31	-2.061	0.000399
A_44_P480865	Nudt16_predicted	XM_343464	363129	-0.31	-2.062	0.001946
A_44_P485025	RGD1306556_predicted	XM_222271	288744	-0.31	-2.062	0.000471
A_44_P189909	RGD1310753	NM_001025020	362235	-0.31	-2.062	0.008988
A_42_P694679	Hdac4_predicted	XM_343629	363287	-0.31	-2.062	0.000601

A_44_P156922	Serinc3	NM_001008312	296350	-0.31	-2.062	0.000109
A_44_P391751	XM_232601	XM_232601		-0.31	-2.063	0.025225
A_43_P20014	RGD1560492_predicted	XM_341316	361039	-0.31	-2.063	0.001111
A_44_P937195	AA901022	AA901022		-0.31	-2.063	0.022885
A_44_P607121	CO401314	CO401314	294643	-0.31	-2.064	0.003295
A_44_P926728	AA850212	AA850212	83826	-0.31	-2.065	0.015649
A_44_P980353	Trim2	XM_001065860		-0.31	-2.065	0.001042
A_44_P414950	XM_222474	XM_222474		-0.31	-2.065	0.001002
A_44_P470892	A_44_P470892	A_44_P470892		-0.32	-2.066	0.00037
A_44_P1036037	Ppil2	NM_001017383	360746	-0.32	-2.066	0.001565
A_44_P345023	RGD1562467_predicted	XM_234966		-0.32	-2.066	0.006678
A_44_P265127	RGD1564090_predicted	XM_215936		-0.32	-2.067	0.014021
A_44_P291381	BF524975	BF524975	299811	-0.32	-2.067	0.031967
A_44_P183620	CB544800	CB544800	302987	-0.32	-2.068	0.071993
A_44_P373153	RGD1560646_predicted	XM_236444	315840	-0.32	-2.068	0.00395
A_44_P374638	Hsd17b2	NM_024391	79243	-0.32	-2.068	0.003053
A_43_P22816	Gpc5_predicted	XM_224489		-0.32	-2.068	0.033146
A_44_P658768	AI070598	AI070598	171500	-0.32	-2.069	0.007491
A_44_P107958	XM_345672	XM_345672		-0.32	-2.069	0.010741
A_44_P381937	Cyp21a1	NM_057101	24298	-0.32	-2.070	0.008678
A_44_P467524	AI407001	AI407001	25482	-0.32	-2.071	0.026006
A_44_P452445	RGD1307961_predicted	XM_219988	309467	-0.32	-2.072	0.00054
A_44_P1028502	LOC681066	XM_001060160	681066	-0.32	-2.072	0.002122
A_44_P191285	RGD1564171_predicted	XM_001074953		-0.32	-2.072	0.011547
A_43_P12024	Cntn3	NM_019329	54279	-0.32	-2.073	0.031143
A_44_P345110	Tom1l2_predicted	XM_340810		-0.32	-2.073	5.29E-05
A_44_P498771	Bsg	NM_012783	25246	-0.32	-2.074	0.000295
A_44_P482823	Lhfpl2_predicted	XM_226668		-0.32	-2.074	0.002412
A_44_P712627	AI105305	AI105305	192645	-0.32	-2.074	0.006057
A_44_P741723	Il13ra1	NM_145789	252963	-0.32	-2.074	0.000362
A_44_P349752	A_44_P349752	A_44_P349752		-0.32	-2.074	0.042576
A_44_P209747	AA892264	AA892264	81916	-0.32	-2.075	0.116679
A_44_P390367	Ahsg	NM_012898	25373	-0.32	-2.075	0.010319
A_44_P101058	AA900938	AA900938		-0.32	-2.076	0.009848
A_44_P157563	AA963043	AA963043		-0.32	-2.077	0.003795
A_42_P631685	Insl6	NM_022583	50546	-0.32	-2.077	0.016559
A_44_P501761	Aim1l_predicted	XM_216539	298543	-0.32	-2.077	0.000187
A_44_P925486	BI284779	BI284779	501736	-0.32	-2.077	0.030072
A_44_P435627	Olr527_predicted	NM_001000675	404878	-0.32	-2.077	0.042204
A_44_P506423	RGD1564895_predicted	XM_231564	312192	-0.32	-2.077	0.001764
A_44_P536485	RGD1559710_predicted	XM_221789	288386	-0.32	-2.077	0.011751
A_44_P133572	RGD1565120_predicted	XM_576393		-0.32	-2.077	0.029161
A_44_P723917	A_44_P723917	A_44_P723917		-0.32	-2.078	0.014282
A_44_P380503	BF290959	BF290959	361195	-0.32	-2.078	0.012341
A_44_P729564	DY570167	DY570167		-0.32	-2.078	0.004053
A_44_P144538	Ebf2_predicted	XM_341343		-0.32	-2.079	0.026607
A_44_P506148	A_44_P506148	A_44_P506148		-0.32	-2.079	0.006892
A_44_P194842	MGC116197	NM_001025755	367620	-0.32	-2.079	0.135325
A_44_P482335	Gys2	NM_013089	25623	-0.32	-2.080	0.004818
A_44_P550931	Gem_predicted	XM_232788		-0.32	-2.080	0.000711
A_44_P398399	Tm9sf2	NM_001005554	306197	-0.32	-2.080	0.000187
A_44_P337991	RGD1564089_predicted	XM_234399		-0.32	-2.080	0.00054
A_44_P333452	RGD1563309_predicted	XM_347258	368088	-0.32	-2.081	0.000147
A_44_P218263	Sned1	XM_237415	316638	-0.32	-2.081	0.001288
A_44_P999115	Tmc5	NM_001012216	365360	-0.32	-2.082	0.000348
A_44_P101338	BF567649	BF567649		-0.32	-2.082	0.001935
A_44_P143541	Lrrfip2	NM_001024761	301035	-0.32	-2.082	0.000125
A_44_P335687	RGD1561304_predicted	XM_343454	363120	-0.32	-2.083	0.012431
A_44_P470565	Bat5	NM_212531	361796	-0.32	-2.083	0.000342
A_44_P1026969	RGD1310870	NM_001012358	361626	-0.32	-2.083	0.001017
A_44_P638170	TC522882	TC522882		-0.32	-2.083	0.000137
A_44_P624624	TC546683	TC546683		-0.32	-2.083	0.008449
A_44_P881100	AW144073	AW144073		-0.32	-2.084	0.01579

A_44_P574134	Slc35f5_predicted	XM_222576		-0.32	-2.084	0.002131
A_44_P273691	BF284294	BF284294	362460	-0.32	-2.084	0.026314
A_44_P398805	Ptpn18	BC098788	301333	-0.32	-2.085	0.002314
A_44_P295639	Gbe1	XM_221747	288333	-0.32	-2.085	0.000379
A_44_P262452	Syn1	NM_019133	24949	-0.32	-2.085	0.003634
A_44_P523289	RGD1306143_predicted	XM_220245		-0.32	-2.086	0.001881
A_44_P484766	LOC498084	XM_573287		-0.32	-2.086	0.024504
A_44_P412955	LOC679923	XM_001054981		-0.32	-2.086	0.003063
A_43_P16416	MAST1	NM_181089	353118	-0.32	-2.087	0.077012
A_44_P297763	XM_229258	XM_229258		-0.32	-2.087	0.040472
A_42_P840729	AW917747	AW917747		-0.32	-2.087	0.000493
A_44_P221974	Abcc3	NM_080581	140668	-0.32	-2.088	0.001853
A_44_P760046	A_44_P760046	A_44_P760046		-0.32	-2.088	0.02868
A_43_P22155	Optc_predicted	XM_222641		-0.32	-2.089	0.001205
A_44_P729699	TC520552	TC520552		-0.32	-2.089	0.004817
A_44_P398275	XM_238783	XM_238783		-0.32	-2.089	0.003576
A_44_P819401	AA957643	AA957643		-0.32	-2.089	0.004071
A_44_P766752	CV102938	CV102938		-0.32	-2.089	0.000157
A_44_P481017	Setbp1_predicted	XM_225744	291423	-0.32	-2.090	0.000198
A_43_P19494	LOC688972	XM_001069014	688972	-0.32	-2.091	0.018161
A_44_P511346	XM_234553	XM_234553		-0.32	-2.091	0.000921
A_44_P685354	TC559451	TC559451		-0.32	-2.092	0.016068
A_44_P626161	TC565451	TC565451		-0.32	-2.092	0.000321
A_44_P545948	LOC293740	XR_008997	293740	-0.32	-2.092	0.000426
A_44_P336075	RGD1306100_predicted	XM_344259		-0.32	-2.092	0.000351
A_42_P763224	Scgb1c1_predicted	XM_219490		-0.32	-2.093	0.000204
A_44_P440772	Sema4g_predicted	XM_342055	361764	-0.32	-2.093	0.000219
A_44_P349883	AI228437	AI228437		-0.32	-2.093	0.010718
A_44_P271347	RGD1305797_predicted	XM_238399		-0.32	-2.093	0.000156
A_42_P751562	Mettl7a	NM_001037355	315306	-0.32	-2.094	0.000176
A_44_P674922	AI549155	AI549155		-0.32	-2.094	0.000237
A_44_P449215	AW251678	AW251678	298700	-0.32	-2.094	0.015143
A_44_P177807	Epas1	NM_023090	29452	-0.32	-2.094	0.000908
A_44_P742499	AW913988	AW913988		-0.32	-2.094	0.000974
A_43_P18806	LOC691099	XM_001076828		-0.32	-2.094	0.088572
A_44_P279398	LOC360684	XM_340956	360684	-0.32	-2.094	0.0021
A_44_P520792	Rdh5_predicted	XM_345770	366791	-0.32	-2.095	0.002567
A_44_P214650	AA945636	AA945636	140661	-0.32	-2.096	0.00549
A_43_P10714	RGD1559803_predicted	XM_001053368		-0.32	-2.096	0.002368
A_44_P650305	BE104538	BE104538	316317	-0.32	-2.097	0.04523
A_44_P913290	TC526229	TC526229		-0.32	-2.098	0.007973
A_44_P222465	LOC498438	XM_344284		-0.32	-2.099	0.001543
A_44_P328806	RGD1561785_predicted	XM_213271	287157	-0.32	-2.099	0.024681
A_44_P423036	CR754963	CR754963	298762	-0.32	-2.100	0.007361
A_44_P158316	BF284692	BF284692		-0.32	-2.100	0.001227
A_43_P17282	Cnnm1_predicted	XM_219879		-0.32	-2.101	0.001919
A_44_P415459	CB547990	CB547990	503165	-0.32	-2.101	0.001066
A_44_P277176	AI045810	AI045810	79214	-0.32	-2.101	0.019285
A_44_P408798	AI548269	AI548269	58852	-0.32	-2.101	0.017257
A_44_P328784	RGD1565158_predicted	XM_222529		-0.32	-2.102	0.007413
A_44_P220610	Fahd1	NM_001024991	302980	-0.32	-2.103	0.000268
A_44_P259892	Olr952_predicted	NM_001000074	288880	-0.32	-2.103	0.005328
A_43_P12100	Nxph4	NM_021680	59316	-0.32	-2.105	0.056905
A_43_P11567	Gamt	NM_012793	25257	-0.32	-2.105	0.002366
A_42_P458876	Muc5ac	U83139	65188	-0.32	-2.105	0.004722
A_44_P803088	AI145252	AI145252		-0.32	-2.105	0.02227
A_44_P575881	RGD1564173_predicted	XM_579040	367947	-0.32	-2.106	0.002809
A_44_P215626	Foxp3_predicted	XM_228771		-0.32	-2.106	0.002512
A_43_P14111	Kitl	AB105879	60427	-0.32	-2.106	0.006771
A_44_P840849	Golga3_predicted	XM_001078840		-0.32	-2.106	8.68E-05
A_44_P210898	RGD1562561_predicted	XM_226405		-0.32	-2.107	0.00806
A_44_P377784	Ndufa12_predicted	XM_216880		-0.32	-2.107	0.000696
A_44_P328962	AI317833	AI317833	361390	-0.32	-2.108	0.001152

A_44_P204894	Tspan4	NM_001013070	293627	-0.32	-2.108	0.000313
A_44_P507139	CB547852	CB547852		-0.32	-2.108	0.000362
A_44_P187156	Fgfr3	NM_053429	84489	-0.32	-2.108	0.000476
A_44_P622992	LOC500377	NM_001024339	500377	-0.32	-2.108	0.004348
A_44_P396481	RGD1305537_predicted	XM_343849		-0.32	-2.109	0.013379
A_44_P458368	AA997825	AA997825	266686	-0.32	-2.109	0.000653
A_44_P289799	BF408425	BF408425	316327	-0.32	-2.109	0.023137
A_43_P15252	Col2a1	NM_012929	25412	-0.32	-2.109	0.010254
A_44_P400202	Hadha	NM_130826	170670	-0.32	-2.109	7.28E-05
A_44_P884109	Trim2	XM_001065860		-0.32	-2.110	0.01093
A_43_P22556	Cidea_predicted	XM_214551	291541	-0.32	-2.110	0.002995
A_42_P789902	Epb4.2_predicted	XM_342503		-0.32	-2.110	0.001816
A_44_P302208	XM_224125	XM_224125		-0.32	-2.111	0.014745
A_42_P614299	XM_215835	XM_215835		-0.32	-2.111	0.003902
A_44_P736228	BG380366	BG380366	303901	-0.32	-2.112	0.000184
A_43_P15447	Bglap2	NM_013414	25295	-0.32	-2.113	0.008733
A_44_P192020	A_44_P192020	A_44_P192020		-0.32	-2.113	0.00212
A_44_P539340	LOC363256	XM_343596		-0.32	-2.113	0.001904
A_44_P976816	TC526159	TC526159		-0.33	-2.113	0.001787
A_44_P494031	Olr733_predicted	NM_001000618	404810	-0.33	-2.114	0.073686
A_43_P14778	AA956338	AA956338	287177	-0.33	-2.114	0.001377
A_44_P187874	BQ190539	BQ190539	25233	-0.33	-2.114	0.00269
A_44_P383360	Trim2	XM_001065860		-0.33	-2.115	0.00127
A_44_P426640	LOC294901	XR_007578	294901	-0.33	-2.116	0.058872
A_44_P578608	TC547498	TC547498		-0.33	-2.116	0.015298
A_43_P12936	Sec14l2	NM_053801	116486	-0.33	-2.116	0.193871
A_44_P443524	RGD1562074_predicted	XM_236940	301253	-0.33	-2.116	0.001853
A_42_P763077	Aqp2	NM_012909	25386	-0.33	-2.116	0.068985
A_44_P466331	Snx15	NM_001024752	293691	-0.33	-2.117	0.000311
A_44_P151812	XM_218114	XM_218114		-0.33	-2.117	0.026212
A_44_P260891	BU759568	BU759568	1E+08	-0.33	-2.118	0.001985
A_44_P555153	Cryba1	XM_340846	25583	-0.33	-2.118	0.002581
A_44_P172936	Zfp179	NM_138613	24916	-0.33	-2.118	0.008271
A_44_P484966	V1rb5_predicted	U36896	497787	-0.33	-2.118	0.001947
A_44_P369206	Rnase4	NM_020082	56759	-0.33	-2.118	0.002132
A_44_P287782	BE108923	BE108923	362092	-0.33	-2.119	0.007584
A_44_P553408	Dock3_predicted	XM_236616		-0.33	-2.119	0.010407
A_44_P316926	BM389600	BM389600		-0.33	-2.119	0.034424
A_44_P464330	RGD1304963_predicted	XM_233868	313891	-0.33	-2.119	0.00072
A_44_P222292	Vac14	NM_177930	307842	-0.33	-2.119	0.009706
A_44_P370441	RGD1559837_predicted	XM_227432		-0.33	-2.119	0.017887
A_44_P162657	AW141855	AW141855	25426	-0.33	-2.119	0.035909
A_44_P181428	BM387272	BM387272	362790	-0.33	-2.119	0.008284
A_44_P181718	BI285476	BI285476	296313	-0.33	-2.120	0.017843
A_44_P260709	XM_220591	XM_220591		-0.33	-2.120	0.00242
A_44_P398856	Krtap3-3_predicted	XM_343966		-0.33	-2.121	0.025467
A_44_P407980	A_44_P407980	A_44_P407980		-0.33	-2.121	0.009929
A_44_P297949	XM_340838	XM_340838		-0.33	-2.121	0.000258
A_44_P439796	BM986522	BM986522	29186	-0.33	-2.121	0.00081
A_42_P596630	Ppapdc2	NM_001034854	619549	-0.33	-2.121	0.000838
A_44_P468147	Adam28	NM_181693	290344	-0.33	-2.121	0.004891
A_44_P557217	Cst6	NM_133566	171096	-0.33	-2.121	0.003837
A_43_P16568	Wdr45	NM_001013958	302559	-0.33	-2.122	0.000086
A_44_P423385	AA957487	AA957487	287346	-0.33	-2.122	0.000171
A_42_P684264	RGD1561817_predicted	XM_215530		-0.33	-2.122	0.002874
A_43_P15126	BF558717	BF558717		-0.33	-2.123	0.000838
A_44_P1027971	LOC688730	XM_001068081		-0.33	-2.123	0.030372
A_44_P375269	CB546586	CB546586		-0.33	-2.123	0.010899
A_43_P18329	Stim1_predicted	XM_341896		-0.33	-2.123	0.000234
A_42_P572725	Abcc3	NM_080581	140668	-0.33	-2.124	0.000948
A_44_P945709	TC536965	TC536965		-0.33	-2.124	0.116343
A_44_P545122	CA945782	CA945782	290868	-0.33	-2.124	0.006126
A_44_P501536	Armc4_predicted	XM_225474	307036	-0.33	-2.124	0.028296

A_43_P11890	Hmgcs1	NM_017268	29637	-0.33	-2.124	0.000377
A_44_P382423	Bnc2_predicted	XM_233123		-0.33	-2.125	0.02493
A_44_P280123	AA874884	AA874884	24451	-0.33	-2.125	0.00679
A_43_P12635	P2ry4	NM_031680	63843	-0.33	-2.125	0.005558
A_44_P204671	AI576557	AI576557	311911	-0.33	-2.125	0.0091
A_44_P150042	Tcte1_predicted	XM_236941		-0.33	-2.126	0.040882
A_44_P618374	AW918245	AW918245		-0.33	-2.126	0.001148
A_42_P841704	Sh3tc1_predicted	XM_223527		-0.33	-2.126	0.000855
A_44_P684795	Irs2	XM_001076309		-0.33	-2.126	0.002353
A_44_P428955	XM_223777	XM_223777		-0.33	-2.127	0.001677
A_44_P817603	AI059671	AI059671		-0.33	-2.127	0.065807
A_44_P835883	AW917211	AW917211	293504	-0.33	-2.127	0.01156
A_43_P14969	Fbp1	NM_012558	24362	-0.33	-2.127	0.001034
A_44_P452145	Camk2b	NM_021739	24245	-0.33	-2.128	0.001279
A_44_P137218	BI395685	BI395685		-0.33	-2.128	0.02516
A_44_P306932	LOC365960	NM_001037210	365960	-0.33	-2.128	0.000564
A_44_P353405	Hrc	NM_181369	292905	-0.33	-2.128	0.001903
A_44_P225060	XM_342706	XM_342706		-0.33	-2.129	0.026478
A_44_P368993	AABR03102812	AABR03102812		-0.33	-2.129	0.050671
A_44_P273459	AA819702	AA819702	361824	-0.33	-2.129	0.013978
A_44_P314525	BQ190588	BQ190588		-0.33	-2.130	0.004139
A_44_P243738	RGD1560959_predicted	XM_233583		-0.33	-2.130	0.017558
A_44_P1037285	Slc44a4	NM_212541	294255	-0.33	-2.130	0.000399
A_42_P556666	LOC500709	XM_576090	500709	-0.33	-2.130	0.000195
A_44_P517734	RGD1306952	NM_001008770	310201	-0.33	-2.130	0.009926
A_42_P800771	RGD1563825_predicted	XM_240367	306366	-0.33	-2.130	0.078226
A_44_P109283	AA997322	AA997322	24530	-0.33	-2.132	0.000544
A_44_P184279	Atp1a1	NM_012504	24211	-0.33	-2.132	0.000386
A_44_P388949	LOC500035	XM_575391		-0.33	-2.132	0.015632
A_44_P574311	AW921380	AW921380	494529	-0.33	-2.132	0.000225
A_44_P435604	Atp1b1	NM_013113	25650	-0.33	-2.132	0.000181
A_44_P337547	XM_218486	XM_218486		-0.33	-2.133	0.031591
A_44_P123473	Aurkaip1	NM_001004237	298687	-0.33	-2.133	0.000187
A_44_P338501	Dpys	NM_031705	65135	-0.33	-2.133	0.012379
A_43_P10290	Tspan1	NM_001004236	298436	-0.33	-2.133	0.001897
A_44_P332657	Mybpc1	XM_343196	362867	-0.33	-2.133	0.038094
A_44_P966370	LOC688390	XM_001066733		-0.33	-2.135	0.002068
A_44_P192820	CA511634	CA511634		-0.33	-2.136	0.003903
A_44_P312240	BQ207094	BQ207094	501099	-0.33	-2.136	0.005823
A_44_P341283	BF281945	BF281945		-0.33	-2.136	0.011479
A_44_P523787	BF400026	BF400026	317161	-0.33	-2.136	0.008809
A_43_P23392	Il1f8_predicted	XM_342377		-0.33	-2.136	0.009926
A_44_P510751	LOC680794	XM_001058904		-0.33	-2.137	0.001755
A_44_P147285	A_44_P147285	A_44_P147285		-0.33	-2.137	0.028909
A_44_P194130	Myh14	XM_218617	308572	-0.33	-2.137	0.001505
A_44_P763705	Pof1b_predicted	XM_228452		-0.33	-2.137	0.006264
A_43_P10721	AW918560	AW918560		-0.33	-2.137	0.000489
A_44_P449544	Slc35e4	NM_153316	266687	-0.33	-2.138	0.000344
A_44_P948250	Il13ra1	NM_145789	252963	-0.33	-2.138	0.001564
A_44_P644261	TC528224	TC528224		-0.33	-2.138	0.003796
A_43_P15812	Hspb7	XM_342966		-0.33	-2.139	0.010473
A_44_P205621	Kcnh3	NM_017108	27150	-0.33	-2.139	0.023758
A_44_P1049097	Krtdap	AB011028	65186	-0.33	-2.139	0.041901
A_44_P169333	LOC684538	XM_001070886		-0.33	-2.141	0.001547
A_44_P277404	AA956764	AA956764	303008	-0.33	-2.142	0.002387
A_44_P837560	TC534940	TC534940		-0.33	-2.143	0.000472
A_44_P443631	BF549985	BF549985		-0.33	-2.143	0.000504
A_43_P22775	LOC681840	XM_001058666		-0.33	-2.143	0.00163
A_44_P244388	AABR03057977	AABR03057977		-0.33	-2.144	0.005601
A_44_P121535	XM_344353	XM_344353		-0.33	-2.144	0.007782
A_44_P527450	XM_344393	XM_344393		-0.33	-2.144	0.035822
A_44_P119039	LOC690653	XM_001075107		-0.33	-2.144	0.028725
A_44_P882860	A_44_P882860	A_44_P882860		-0.33	-2.144	0.081926

A_44_P236770	TC543610	TC543610		-0.33	-2.144	0.027275
A_44_P962711	TC566391	TC566391		-0.33	-2.144	0.001975
A_44_P787860	AI137363	AI137363	245978	-0.33	-2.145	0.013437
A_44_P131084	RGD1562693_predicted	XM_346355	367930	-0.33	-2.145	0.002163
A_44_P527024	Col11a2_mapped	NM_212528	294279	-0.33	-2.145	0.000896
A_44_P390734	AW143618	AW143618		-0.33	-2.146	0.031239
A_43_P16646	Tex264	NM_001007665	300988	-0.33	-2.146	0.000191
A_44_P475765	Samd7_predicted	XM_226973	310257	-0.33	-2.147	0.005598
A_42_P802334	Cel	NM_016997	24254	-0.33	-2.147	0.005341
A_44_P217633	A_44_P217633	A_44_P217633		-0.33	-2.147	0.0017
A_44_P213776	Rims1	NM_052829	84556	-0.33	-2.148	0.074065
A_44_P648053	AI044208	AI044208		-0.33	-2.148	0.010571
A_44_P280755	Msi1h	NM_148890	259272	-0.33	-2.148	0.015869
A_44_P625012	A_44_P625012	A_44_P625012		-0.33	-2.148	0.004009
A_44_P407567	RGD1308782	NM_001025677	308318	-0.33	-2.148	0.004542
A_44_P423765	Olr610_predicted	NM_001000336	295836	-0.33	-2.148	0.022431
A_43_P20478	Slc37a1	NM_001011944	294321	-0.33	-2.148	7.54E-05
A_44_P750974	TC562373	TC562373		-0.33	-2.148	0.003549
A_44_P934688	TC550085	TC550085		-0.33	-2.148	0.004738
A_44_P285786	NP516897	NP516897		-0.33	-2.150	0.012622
A_42_P698240	Ltb4dh	NM_138863	192227	-0.33	-2.150	0.005031
A_44_P401480	BG375795	BG375795	315962	-0.33	-2.150	0.006008
A_44_P940828	AW520848	AW520848		-0.33	-2.150	0.054399
A_42_P733814	Tox_predicted	XM_342800		-0.33	-2.151	0.000626
A_44_P340286	XM_343719	XM_343719		-0.33	-2.151	0.025861
A_44_P365303	Olr336_predicted	NM_001000759	405032	-0.33	-2.151	0.024519
A_44_P449281	BI294916	BI294916	306330	-0.33	-2.153	0.00503
A_42_P676630	Gpld1	XM_214480	291132	-0.33	-2.153	0.004861
A_42_P672420	Rasd1	XM_001077321		-0.33	-2.153	0.005772
A_44_P974994	LOC367694	XR_008238	367694	-0.33	-2.153	0.000648
A_44_P488167	AA900165	AA900165	60355	-0.33	-2.154	0.000711
A_44_P705136	AI176136	AI176136	315037	-0.33	-2.154	0.000222
A_44_P920308	AW252891	AW252891		-0.33	-2.154	0.008376
A_44_P217846	XM_227356	XM_227356		-0.33	-2.154	0.026457
A_44_P137322	Lhx9	NM_181367	289048	-0.33	-2.154	0.009137
A_44_P760016	ENSRNOT00000044353	ENSRNOT00000044353		-0.33	-2.154	0.031937
A_44_P1015754	Slc22a3	NM_019230	29504	-0.33	-2.154	0.010634
A_44_P543844	LOC500419	NM_001031663	500419	-0.33	-2.155	0.000185
A_44_P248591	RGD1561243_predicted	XM_343383	363055	-0.33	-2.155	0.002078
A_43_P22562	AW920054	AW920054	361492	-0.33	-2.155	0.000457
A_44_P543861	LOC301879	XM_229361		-0.33	-2.155	0.056484
A_44_P128488	Edg4_predicted	XM_341418		-0.33	-2.155	0.001106
A_44_P208133	Klk1c10	XM_214947	292858	-0.33	-2.156	0.000278
A_44_P541131	Ndn	NM_001008558	308690	-0.33	-2.156	0.01269
A_44_P408114	XM_343516	XM_343516		-0.33	-2.156	0.002183
A_42_P663871	Gzma	NM_153468	266708	-0.33	-2.156	0.000324
A_44_P823938	TC543636	TC543636		-0.33	-2.157	0.055074
A_44_P914151	TC557658	TC557658		-0.33	-2.157	0.000697
A_44_P250186	LOC304000	NM_001006990	304000	-0.33	-2.157	7.81E-05
A_44_P555703	XM_347122	XM_347122		-0.33	-2.157	0.017149
A_44_P316046	Olr1234_predicted	NM_001000446	300558	-0.33	-2.157	0.005971
A_44_P484474	AI237388	AI237388	300994	-0.33	-2.157	0.000686
A_44_P281822	BQ206176	BQ206176	367172	-0.33	-2.158	0.00915
A_44_P789130	BF418059	BF418059		-0.33	-2.158	0.001552
A_44_P462422	CB547704	CB547704		-0.33	-2.158	0.002098
A_44_P222845	Myo5c_predicted	XM_236411		-0.33	-2.158	0.000812
A_44_P1000561	RGD1305781	XM_235378	315037	-0.33	-2.159	0.0009
A_44_P371050	AA851185	AA851185		-0.33	-2.159	0.004971
A_44_P231792	RGD1564936_predicted	XM_342169	361875	-0.33	-2.160	0.001421
A_44_P271953	LOC679879	XM_001054812		-0.33	-2.160	0.057487
A_44_P1024756	Mybpc1	XM_343196	362867	-0.33	-2.160	0.01796
A_44_P817760	LOC679462	XM_001056482		-0.33	-2.161	0.002319
A_44_P128757	A_44_P128757	A_44_P128757		-0.33	-2.161	0.008379

A_44_P1007321	Plcl2_predicted	XM_001056257		-0.33	-2.161	0.000318
A_44_P287560	H33491	H33491	117278	-0.33	-2.162	0.003597
A_44_P668249	TC519642	TC519642		-0.33	-2.162	0.011838
A_44_P1039435	Lasp1	NM_032613	29278	-0.33	-2.162	0.000605
A_44_P1053772	Galt	NM_001013089	298003	-0.34	-2.163	0.000973
A_44_P472828	Chm	NM_017067	24942	-0.34	-2.163	0.035073
A_44_P199469	Gsdmdc1_predicted	XM_235434	315084	-0.34	-2.163	0.000758
A_44_P105156	Stard10	NM_001013069	293150	-0.34	-2.164	0.000146
A_44_P425380	Ndrg1	NM_001011991	299923	-0.34	-2.164	0.005129
A_44_P259947	Kcnma1	NM_031828	83731	-0.34	-2.165	0.006841
A_44_P274218	XM_218253	XM_218253		-0.34	-2.165	0.003506
A_44_P328380	XM_224105	XM_224105		-0.34	-2.165	0.014447
A_44_P414630	LOC298116	NM_001003409	259246	-0.34	-2.165	0.006925
A_44_P496105	AW522725	AW522725	293144	-0.34	-2.166	0.001201
A_44_P449551	Slc35e4	NM_153316	266687	-0.34	-2.166	0.005578
A_44_P405177	Ela2	NM_012553	24332	-0.34	-2.166	0.08094
A_43_P11585	Gast	NM_012849	25320	-0.34	-2.168	0.000938
A_44_P616694	BG668519	BG668519		-0.34	-2.168	0.005021
A_44_P671497	AW921183	AW921183		-0.34	-2.168	0.000384
A_43_P14933	Ggh	NM_012960	25455	-0.34	-2.170	8.52E-05
A_44_P394074	Oxct2a	NM_001012221	366463	-0.34	-2.170	0.002219
A_44_P591196	Sfrp5_predicted	XM_001055342		-0.34	-2.171	0.011322
A_44_P265807	Aldh3a2	NM_031731	65183	-0.34	-2.171	0.000379
A_43_P16211	Dnahc3	XM_001065872	117249	-0.34	-2.172	0.004626
A_43_P12510	Rab3b	NM_031091	81755	-0.34	-2.172	0.112932
A_42_P548840	Slc9a1	NM_012652	24782	-0.34	-2.173	0.004254
A_42_P800008	Slc4a3	NM_017049	24781	-0.34	-2.173	0.006409
A_44_P329823	AI179651	AI179651	499417	-0.34	-2.173	0.000595
A_44_P445743	Ccin	NM_001005904	298392	-0.34	-2.173	0.000735
A_43_P21004	CB544375	CB544375		-0.34	-2.174	0.001853
A_44_P367012	BG377024	BG377024		-0.34	-2.174	0.0509
A_43_P14402	Clk1	XM_217405		-0.34	-2.174	0.000108
A_42_P833045	Gap43	NM_017195	29423	-0.34	-2.174	0.001653
A_43_P15483	Slc28a1	NM_053863	116642	-0.34	-2.175	0.007724
A_44_P613427	TC545768	TC545768		-0.34	-2.175	0.000343
A_44_P643423	A_44_P643423	A_44_P643423		-0.34	-2.176	0.005221
A_44_P605884	Golph2_predicted	XM_225147	683591	-0.34	-2.176	0.001608
A_43_P15737	Vil2	NM_019357	54319	-0.34	-2.176	0.000254
A_44_P515560	RGD1310788_predicted	XM_001079575		-0.34	-2.176	0.053336
A_44_P621029	BI301310	BI301310		-0.34	-2.176	0.014355
A_44_P487960	BM390495	BM390495	300030	-0.34	-2.179	0.005823
A_44_P1020477	RGD1565884_predicted	XM_223941		-0.34	-2.179	0.013376
A_44_P470535	Olr1616_predicted	NM_001000840	405129	-0.34	-2.180	0.009304
A_43_P15396	Nalp6	XM_577848	171390	-0.34	-2.180	0.026815
A_44_P414963	Slc2a10_predicted	XM_345471		-0.34	-2.181	0.001333
A_42_P473360	Vps13d_predicted	XM_233792		-0.34	-2.181	0.00037
A_44_P389072	Il13ra1	NM_145789	252963	-0.34	-2.182	0.000354
A_42_P486847	Tmco3_predicted	XM_225016	306607	-0.34	-2.182	0.003163
A_44_P466845	Hbq1_predicted	XM_001061675		-0.34	-2.182	0.01294
A_44_P129685	AW142659	AW142659	316003	-0.34	-2.183	0.000423
A_44_P241698	XM_221389	XM_221389		-0.34	-2.183	0.084824
A_44_P362889	Ndufa1_predicted	XM_343760		-0.34	-2.184	0.000565
A_44_P319260	LOC362994	NM_001014203	362994	-0.34	-2.185	0.013663
A_44_P706666	A_44_P706666	A_44_P706666		-0.34	-2.186	0.000408
A_44_P515265	Olr1201_predicted	NM_001000818	405106	-0.34	-2.186	0.004843
A_44_P538882	LOC309362	XM_219860	309362	-0.34	-2.186	6.07E-05
A_44_P1041460	Rragd_predicted	XM_216363		-0.34	-2.186	0.011575
A_44_P101427	AI071510	AI071510	499112	-0.34	-2.186	0.002703
A_43_P19484	Doxl1	NM_199233	312316	-0.34	-2.186	0.003293
A_44_P496460	LOC683758	XM_001067366		-0.34	-2.186	0.015006
A_44_P374586	Cyb5	NM_022245	64001	-0.34	-2.187	0.000107
A_44_P545193	Hsd11b2	NM_017081	25117	-0.34	-2.187	0.000374
A_44_P637998	CB582188	CB582188		-0.34	-2.187	0.0529

A_44_P542249	BQ190629	BQ190629	294963	-0.34	-2.187	0.0404
A_44_P721783	AW917081	AW917081		-0.34	-2.188	0.01324
A_44_P306784	Lnx1_predicted	XM_341204		-0.34	-2.188	0.004971
A_43_P19433	Farp2_predicted	XM_237444		-0.34	-2.188	0.0009
A_42_P554066	Tasp1_predicted	XM_230631		-0.34	-2.189	0.001102
A_44_P597467	CO386096	CO386096		-0.34	-2.189	0.000505
A_44_P102746	Trpm1	NM_001037734	361586	-0.34	-2.191	0.000738
A_44_P436844	BQ206577	BQ206577	85251	-0.34	-2.191	0.009303
A_44_P683382	LOC686624	XM_001075025		-0.34	-2.191	0.030673
A_44_P250660	A_44_P250660	A_44_P250660		-0.34	-2.191	0.017564
A_44_P309423	RGD1562546_predicted	XM_344729	364953	-0.34	-2.192	0.001018
A_44_P522706	XM_221806	XM_221806		-0.34	-2.192	0.007806
A_44_P1001820	Slc22a5	NM_019269	29726	-0.34	-2.194	0.009817
A_44_P290879	Rrbp1_predicted	XM_230637	311483	-0.34	-2.195	0.000233
A_44_P257735	RGD1564043_predicted	XM_223014		-0.34	-2.195	0.034458
A_43_P11622	Cd59	NM_012925	25407	-0.34	-2.196	0.003615
A_44_P551181	CB547684	CB547684		-0.34	-2.196	0.005848
A_44_P445484	RGD1561956_predicted	XM_344287		-0.34	-2.197	5.56E-05
A_44_P492084	RGD1562220_predicted	XM_001068657		-0.34	-2.197	0.01656
A_44_P215880	Rilp_predicted	XM_213380		-0.34	-2.197	0.005448
A_42_P686680	Ubqln2_predicted	XM_228806		-0.34	-2.197	0.003592
A_44_P862865	BE102978	BE102978		-0.34	-2.197	0.021857
A_44_P356003	Olr1232_predicted	NM_001000445	300557	-0.34	-2.198	0.004385
A_44_P161411	RGD1562952_predicted	XM_001072232		-0.34	-2.198	0.000417
A_44_P945969	TC541717	TC541717		-0.34	-2.199	0.00117
A_43_P15995	Birc1b	AJ271303	191568	-0.34	-2.199	0.006213
A_43_P14045	Sema4g_predicted	XM_342055	361764	-0.34	-2.199	0.000266
A_44_P534582	LOC367515	NM_001014271	367515	-0.34	-2.199	0.013659
A_44_P178179	Pgbd5_predicted	XM_226561		-0.34	-2.199	0.001764
A_44_P400936	ENSRNOT00000044402	ENSRNOT00000044402		-0.34	-2.200	0.013764
A_44_P555261	Aldh6a1	NM_031057	81708	-0.34	-2.201	0.002874
A_42_P603700	LOC246266	NM_144750	246266	-0.34	-2.201	0.030655
A_44_P501220	Mcf2l	NM_053951		-0.34	-2.202	0.000129
A_44_P434428	BF395191	BF395191		-0.34	-2.202	0.002077
A_44_P238921	Pcdha2	NM_199504	116741	-0.34	-2.202	0.000236
A_44_P485499	LOC299152	NM_001025658	299152	-0.34	-2.203	0.000618
A_44_P177726	AF028784	AF028784		-0.34	-2.203	0.008449
A_44_P183659	AI179324	AI179324	293410	-0.34	-2.203	0.009664
A_44_P181115	AA925467	AA925467		-0.34	-2.205	0.000698
A_44_P296819	BE106940	BE106940	64356	-0.34	-2.205	0.000879
A_44_P203457	Tcrav	XM_344471		-0.34	-2.205	0.007691
A_43_P18201	Zfp339_predicted	XM_230639		-0.34	-2.205	0.005465
A_43_P10693	BG671006	BG671006		-0.34	-2.206	0.000277
A_44_P532517	LOC691221	XM_001077260	691221	-0.34	-2.207	0.00395
A_44_P428782	Scp2	NM_138508	25541	-0.34	-2.207	6.47E-05
A_44_P531076	AI010962	AI010962		-0.34	-2.208	0.00245
A_44_P501637	Man1a_predicted	NM_001033656	294410	-0.34	-2.209	4.85E-05
A_44_P208319	RGD1560695_predicted	XM_344801		-0.34	-2.210	0.00262
A_44_P462998	BI279090	BI279090	690966	-0.34	-2.210	0.019376
A_44_P102814	A_44_P102814	A_44_P102814		-0.34	-2.210	0.010208
A_44_P637675	TC538578	TC538578		-0.34	-2.210	0.001553
A_44_P350142	AA925681	AA925681		-0.34	-2.211	0.002118
A_44_P817685	AI136887	AI136887		-0.34	-2.212	0.002327
A_44_P540099	AA944007	AA944007	84595	-0.34	-2.212	0.010934
A_42_P690569	Ndufv2	NM_031064	81728	-0.34	-2.212	4.78E-05
A_44_P459696	A_44_P459696	A_44_P459696		-0.34	-2.212	0.003631
A_44_P790431	XM_344391	XM_344391		-0.34	-2.213	0.003947
A_44_P389493	XM_343740	XM_343740		-0.35	-2.213	4.75E-05
A_44_P410591	AI176554	AI176554		-0.35	-2.213	0.019386
A_44_P135650	Smtnl1_predicted	XM_230278	311167	-0.35	-2.214	0.003746
A_44_P478305	Atp6v0a4_predicted	XM_231615		-0.35	-2.215	0.018617
A_44_P124167	A_44_P124167	A_44_P124167		-0.35	-2.215	0.013379
A_44_P458570	Fut4	NM_022219	60670	-0.35	-2.216	0.000735

A_42_P586939	Adra1b	NM_016991	24173	-0.35	-2.216	0.020686
A_44_P745478	TC538112	TC538112		-0.35	-2.216	0.000146
A_44_P928299	AI111983	AI111983	497860	-0.35	-2.217	0.005877
A_43_P22470	Trfr2_predicted	XM_222022		-0.35	-2.217	0.041031
A_44_P163313	Arhgap20	NM_213629	367085	-0.35	-2.217	0.028722
A_44_P211120	A_44_P211120	A_44_P211120		-0.35	-2.217	0.001712
A_44_P1032623	LOC367113	NM_001014268	367113	-0.35	-2.217	0.000184
A_44_P221461	BI298356	BI298356	25177	-0.35	-2.217	0.057822
A_44_P122042	AA926035	AA926035	364208	-0.35	-2.218	0.001066
A_44_P791333	A_44_P791333	A_44_P791333		-0.35	-2.218	8.14E-05
A_43_P10160	AA945948	AA945948	310689	-0.35	-2.218	0.020933
A_44_P250714	A_44_P250714	A_44_P250714		-0.35	-2.219	0.01906
A_44_P317611	BF389173	BF389173		-0.35	-2.219	0.030548
A_44_P863291	AI639382	AI639382		-0.35	-2.220	0.01508
A_44_P293497	BI276762	BI276762	287466	-0.35	-2.221	0.019133
A_44_P509771	AI009594	AI009594	306322	-0.35	-2.222	0.03982
A_44_P255625	A_44_P255625	A_44_P255625		-0.35	-2.223	0.002299
A_44_P548798	Pkhd1_predicted	XM_236979		-0.35	-2.223	0.015652
A_44_P543752	A_44_P543752	A_44_P543752		-0.35	-2.223	0.000765
A_44_P249990	Mal2	NM_198786	362911	-0.35	-2.224	0.000935
A_44_P470146	AA891422	AA891422	140937	-0.35	-2.224	0.004428
A_44_P568283	BM389610	BM389610		-0.35	-2.225	0.004209
A_44_P486724	AA800669	AA800669	117281	-0.35	-2.225	0.003475
A_44_P717436	TC534231	TC534231		-0.35	-2.226	0.018562
A_44_P442931	Titf1	XM_216720	25628	-0.35	-2.226	0.011807
A_44_P405618	RGD1561090_predicted	XM_233065	313278	-0.35	-2.226	0.003404
A_44_P541244	XM_224032	XM_224032		-0.35	-2.226	0.008554
A_42_P803137	Spint2	NM_199087	292770	-0.35	-2.227	0.000184
A_44_P436942	RGD1561961_predicted	XM_213926	289181	-0.35	-2.228	0.017794
A_44_P501937	Lpo_predicted	XM_220831		-0.35	-2.228	0.029178
A_44_P234071	LOC305166	NM_001025670	305166	-0.35	-2.228	0.000465
A_43_P15466	Pde5a	NM_133584	171115	-0.35	-2.228	0.000862
A_44_P324013	RGD1309492_predicted	XM_234430		-0.35	-2.228	0.00044
A_44_P183027	LOC367646	XM_346238		-0.35	-2.228	0.033184
A_44_P546234	A_44_P546234	A_44_P546234		-0.35	-2.228	0.003848
A_44_P136342	Ugp2	NM_001024743	289827	-0.35	-2.228	0.000743
A_44_P700820	TC524593	TC524593		-0.35	-2.229	0.008547
A_44_P356291	Uqcrb_predicted	XM_343225	362897	-0.35	-2.229	8.81E-05
A_44_P302334	Cdh16	NM_001012055	307614	-0.35	-2.229	0.00615
A_44_P548095	AY168780	AY168780		-0.35	-2.230	0.004916
A_44_P574783	Golph4	XM_001062524		-0.35	-2.230	0.000614
A_44_P974255	LOC690114	XM_001073318		-0.35	-2.230	0.021616
A_44_P724928	AI763958	AI763958		-0.35	-2.230	0.026507
A_44_P1060167	Vps33a	NM_022961	65081	-0.35	-2.230	0.001043
A_44_P307463	BI276352	BI276352	311187	-0.35	-2.231	0.001033
A_44_P133544	XM_345798	XM_345798		-0.35	-2.231	0.003898
A_44_P264694	LOC680551	XM_001057696		-0.35	-2.232	0.027853
A_44_P242938	Ces6	NM_144743	246252	-0.35	-2.233	0.013642
A_44_P760452	RGD1561171_predicted	XM_575469		-0.35	-2.233	0.000419
A_44_P463036	CA508371	CA508371	361207	-0.35	-2.234	0.001676
A_44_P555496	A_44_P555496	A_44_P555496		-0.35	-2.234	0.045496
A_44_P140429	Asb9_predicted	XM_346301	367785	-0.35	-2.234	0.010856
A_44_P728688	LOC685378	XM_001063552		-0.35	-2.234	0.006487
A_44_P837263	ENSRNOT00000038196	ENSRNOT00000038196		-0.35	-2.234	0.001176
A_44_P916037	TC525953	TC525953		-0.35	-2.234	0.003406
A_44_P515798	RGD1308977_predicted	XM_214510		-0.35	-2.234	0.00027
A_44_P116652	Chrnd	NM_019298	54240	-0.35	-2.236	0.001953
A_44_P227251	RGD1310484_predicted	XM_214594		-0.35	-2.236	0.000295
A_44_P852754	TC544658	TC544658		-0.35	-2.236	0.000149
A_43_P19717	RGD1311867_predicted	XM_344745	364976	-0.35	-2.236	0.001008
A_44_P447560	Acvr2a	NM_031571	29263	-0.35	-2.237	0.000836
A_44_P383679	AA998008	AA998008	171293	-0.35	-2.237	0.014926
A_44_P374390	AW143132	AW143132		-0.35	-2.237	0.000852

A_44_P414803	XM_344351	XM_344351		-0.35	-2.237	0.006045
A_44_P541566	LOC691708	XM_001079338	691708	-0.35	-2.238	0.010655
A_42_P600870	LOC500413	XM_575775	500413	-0.35	-2.238	0.023251
A_44_P245665	Atp2b1	NM_053311	29598	-0.35	-2.238	0.000117
A_44_P924738	AW916360	AW916360		-0.35	-2.241	9.55E-05
A_44_P402536	Lep	NM_013076	25608	-0.35	-2.241	0.002997
A_44_P146753	Olr39_predicted	NM_001000128	293198	-0.35	-2.241	0.00064
A_44_P234323	XM_345077	XM_345077		-0.35	-2.242	0.000735
A_44_P464367	XM_345750	XM_345750		-0.35	-2.242	0.014452
A_44_P1029357	RGD1563485_predicted	XM_217657	307992	-0.35	-2.242	0.004378
A_44_P262593	Ankrd2_predicted	XM_219881		-0.35	-2.244	0.023466
A_44_P815267	A_44_P815267	A_44_P815267		-0.35	-2.244	0.000855
A_44_P316849	RGD1562369_predicted	XM_576570		-0.35	-2.245	0.000451
A_44_P547236	RGD1560915_predicted	XM_575419		-0.35	-2.246	0.001053
A_43_P11900	Kcnj2	NM_017296	29712	-0.35	-2.246	0.020779
A_44_P493256	AA818120	AA818120	367086	-0.35	-2.246	0.04104
A_44_P117005	XM_216078	XM_216078		-0.35	-2.247	0.002392
A_44_P459660	RGD1559581_predicted	XM_228661		-0.35	-2.247	0.036162
A_44_P173019	Slc6a8	NM_017348	50690	-0.35	-2.247	0.012452
A_44_P660246	Cald1	NM_013146	25687	-0.35	-2.247	0.008425
A_44_P974618	RGD1566351_predicted	XM_575110	499776	-0.35	-2.247	0.000149
A_44_P179925	Olr43_predicted	NM_001000541	365322	-0.35	-2.248	0.029113
A_43_P12761	Atp2b1	NM_053311	29598	-0.35	-2.248	8.81E-05
A_44_P832271	A_44_P832271	A_44_P832271		-0.35	-2.249	0.004113
A_44_P280224	AA875449	AA875449	282819	-0.35	-2.249	0.010282
A_43_P10303	BG670819	BG670819	499818	-0.35	-2.249	0.007938
A_44_P310557	BF409560	BF409560	300983	-0.35	-2.249	0.023412
A_44_P260644	RGD1564335_predicted	XM_576264	500865	-0.35	-2.250	0.00047
A_44_P531925	A_44_P531925	A_44_P531925		-0.35	-2.250	0.016352
A_44_P464119	A_44_P464119	A_44_P464119		-0.35	-2.250	0.01087
A_44_P292040	AI029946	AI029946	289380	-0.35	-2.250	0.057022
A_44_P234602	A_44_P234602	A_44_P234602		-0.35	-2.250	0.00122
A_42_P819052	XM_219895	XM_219895		-0.35	-2.252	0.000628
A_44_P262091	Fabp3	NM_024162	79131	-0.35	-2.252	0.00631
A_44_P553280	XM_228626	XM_228626		-0.35	-2.252	0.026054
A_44_P325133	RGD1305459_predicted	XM_001078833		-0.35	-2.252	0.006947
A_44_P334442	DV728037	DV728037		-0.35	-2.253	0.002052
A_44_P398837	BF560064	BF560064	497891	-0.35	-2.253	0.000711
A_44_P252623	Try10	NM_001004097	408247	-0.35	-2.254	0.082274
A_44_P406248	BM392313	BM392313	114766	-0.35	-2.254	0.020129
A_43_P12346	Mertk	NM_022943	65037	-0.35	-2.254	0.00128
A_44_P1014472	RGD1310121_predicted	XM_341746		-0.35	-2.254	0.000348
A_44_P421172	BI275923	BI275923	680945	-0.35	-2.254	0.017595
A_44_P496653	RGD1566381_predicted	XM_221799		-0.35	-2.255	0.002569
A_44_P772793	AW916452	AW916452		-0.35	-2.256	0.001282
A_43_P19866	TC522685	TC522685		-0.35	-2.256	0.000408
A_44_P545498	Sox11	NM_053349	84046	-0.35	-2.256	0.003848
A_44_P391192	Cyp2g1	XM_341809		-0.35	-2.257	0.004033
A_44_P801407	A_44_P801407	A_44_P801407		-0.35	-2.257	0.087921
A_44_P215259	XM_344081	XM_344081		-0.35	-2.257	0.035706
A_44_P837782	TC520328	TC520328		-0.35	-2.257	3.39E-05
A_43_P20593	Slc25a29	NM_001010958	314441	-0.35	-2.258	0.000326
A_44_P163454	V01257	V01257		-0.35	-2.260	0.001633
A_44_P900001	Arhgef15_predicted	XM_001079166		-0.35	-2.260	0.001002
A_44_P557929	TC545506	TC545506		-0.35	-2.261	0.01096
A_43_P12932	Stx1a	NM_053788	116470	-0.35	-2.261	0.000576
A_44_P178498	B3galt5_predicted	XM_221525		-0.35	-2.261	0.000129
A_42_P511118	Slc17a6	NM_053427	84487	-0.35	-2.262	0.028483
A_43_P12351	Loc65050	NM_022956	65050	-0.35	-2.263	0.004623
A_44_P531145	AI113265	AI113265	155012	-0.35	-2.263	0.00418
A_42_P821549	BI395616	BI395616		-0.35	-2.263	0.001572
A_44_P620106	Tgfbi	XM_573983	116487	-0.35	-2.264	0.056083
A_44_P547934	Olr833_predicted	NM_001000408	298397	-0.35	-2.264	0.009863

A_43_P13805	AA955077	AA955077		-0.36	-2.265	0.001308
A_44_P274414	ENSRNOT00000040365	ENSRNOT00000040365		-0.36	-2.265	0.000922
A_44_P291189	Hspb9_predicted	XM_343967		-0.36	-2.265	0.002156
A_44_P140026	Tle2	NM_001039013	299636	-0.36	-2.266	0.010799
A_44_P411286	BM385494	BM385494	362597	-0.36	-2.266	0.004694
A_44_P282872	AA996805	AA996805	619574	-0.36	-2.266	0.003002
A_43_P16697	RGD1564876_predicted	XM_001080983		-0.36	-2.266	0.005962
A_44_P305950	AW532187	AW532187	170944	-0.36	-2.267	0.0037
A_44_P545594	Olr239_predicted	NM_001000211	293383	-0.36	-2.268	0.027038
A_44_P983155	LOC292209	XM_238066		-0.36	-2.268	0.000116
A_44_P703394	TC563202	TC563202		-0.36	-2.268	0.075119
A_44_P898051	RGD1560459_predicted	XM_573131	497943	-0.36	-2.268	0.02028
A_44_P167403	AA875633	AA875633	287710	-0.36	-2.268	0.004612
A_44_P752274	CB582130	CB582130		-0.36	-2.268	0.000411
A_44_P374848	Chp	NM_024139	64152	-0.36	-2.269	0.000275
A_44_P369389	BF556192	BF556192		-0.36	-2.269	0.000113
A_44_P415776	AA819759	AA819759	681031	-0.36	-2.269	0.004911
A_44_P508691	LOC689029	XM_001068287	689029	-0.36	-2.270	0.000168
A_44_P282567	AI060229	AI060229		-0.36	-2.270	0.013282
A_44_P791696	DV725758	DV725758		-0.36	-2.270	0.016257
A_44_P626204	TC564181	TC564181		-0.36	-2.270	0.002529
A_44_P834317	AW916822	AW916822		-0.36	-2.271	0.07058
A_43_P12766	Syt8	NM_053325	60566	-0.36	-2.271	0.030002
A_43_P16515	XM_215130	XM_215130		-0.36	-2.272	0.000474
A_44_P525085	XM_214905	XM_214905		-0.36	-2.273	0.000379
A_43_P14977	LOC498545	XM_573819		-0.36	-2.273	0.000114
A_44_P208102	RGD1306094	NM_001008763	289587	-0.36	-2.273	0.00057
A_44_P225227	XM_236202	XM_236202		-0.36	-2.274	0.028097
A_44_P391109	Ush1c	AY588414	308596	-0.36	-2.274	4.57E-05
A_44_P429755	Lin7c	NM_021851	60442	-0.36	-2.274	0.001427
A_44_P480858	XM_235375	XM_235375		-0.36	-2.274	0.078631
A_44_P960804	TC556987	TC556987		-0.36	-2.275	0.021564
A_44_P388063	AA943963	AA943963	59076	-0.36	-2.275	0.00088
A_44_P525300	AY539886	AY539886		-0.36	-2.275	0.007621
A_44_P433614	Vmd2	NM_001011940	293735	-0.36	-2.276	0.003828
A_42_P650906	Mcee_predicted	XM_215213		-0.36	-2.276	0.000659
A_44_P173421	Zcchc5_predicted	XM_228502	317221	-0.36	-2.276	0.024507
A_44_P330382	Dnah6	XM_342709	117250	-0.36	-2.276	0.005225
A_44_P288668	Zfp352_predicted	XM_233185		-0.36	-2.277	0.011825
A_44_P834081	AI059920	AI059920		-0.36	-2.277	0.007278
A_44_P290953	RGD1564200_predicted	XM_001063324		-0.36	-2.278	0.0002
A_44_P145438	BF401593	BF401593		-0.36	-2.278	0.024484
A_44_P267228	Hsd17b13	NM_001009684	305150	-0.36	-2.278	0.002142
A_44_P692981	RGD1560652_predicted	XM_575705		-0.36	-2.278	0.00045
A_44_P142837	Cmya1_predicted	XM_236702	316071	-0.36	-2.278	0.00414
A_44_P363428	RGD1564522_predicted	XM_223671		-0.36	-2.278	0.001542
A_44_P550602	RGD1561338_predicted	XM_218631		-0.36	-2.279	0.002347
A_44_P108950	AI547409	AI547409	25216	-0.36	-2.279	0.01163
A_44_P172856	Gh1	NM_001034848	24391	-0.36	-2.279	0.030792
A_44_P497302	St6galnac2	NM_001031652	303692	-0.36	-2.279	0.003629
A_44_P452959	CB547141	CB547141		-0.36	-2.281	0.011956
A_44_P662992	AI169727	AI169727		-0.36	-2.282	0.005387
A_44_P220861	Npff	NM_022586	60337	-0.36	-2.282	0.018108
A_42_P716553	Rhod_predicted	XM_215193		-0.36	-2.283	0.000917
A_43_P17337	XM_343348	XM_343348		-0.36	-2.284	0.000267
A_43_P21462	XM_342961	XM_342961		-0.36	-2.284	0.016785
A_44_P291130	RGD1564844_predicted	XM_236851		-0.36	-2.284	0.005198
A_44_P375676	BG374711	BG374711	361399	-0.36	-2.284	0.000748
A_44_P449929	LOC679007	XM_001054187		-0.36	-2.285	0.023298
A_44_P224249	CA507885	CA507885		-0.36	-2.285	0.000708
A_44_P514824	BQ202476	BQ202476	311849	-0.36	-2.286	0.000876
A_44_P405139	Arhgef11	NM_023982	78966	-0.36	-2.286	7.96E-05
A_43_P20416	Sync_predicted	XM_342925		-0.36	-2.286	0.000155

A_44_P978012	TC526598	TC526598		-0.36	-2.286	0.002785
A_44_P574959	LOC681694	XM_001057999		-0.36	-2.286	0.0003
A_44_P531593	Smad3	NM_013095	25631	-0.36	-2.287	0.009991
A_44_P201178	Pib5pa	NM_133562	171088	-0.36	-2.287	0.001472
A_44_P386724	Xpnpep2	NM_057155	117522	-0.36	-2.287	0.000801
A_44_P213433	Sema3b_predicted	XM_343479		-0.36	-2.288	0.00138
A_44_P424335	RGD1308358_predicted	XM_226397		-0.36	-2.288	0.001622
A_44_P397657	AA998783	AA998783	83418	-0.36	-2.288	0.028693
A_44_P545624	Klk4	NM_001004101	408210	-0.36	-2.288	0.043939
A_44_P236407	Lnx1_predicted	XM_341204		-0.36	-2.288	0.000278
A_44_P401615	Esr1	NM_012689	24890	-0.36	-2.288	0.056484
A_44_P208688	Pof1b_predicted	XM_228452		-0.36	-2.289	0.000549
A_42_P764466	Arhgap18_predicted	XM_215247		-0.36	-2.289	0.000276
A_44_P335479	XM_228074	XM_228074		-0.36	-2.290	0.001149
A_44_P563970	TC551408	TC551408		-0.36	-2.290	0.002059
A_44_P333266	Znrf4	NM_001024878	301127	-0.36	-2.292	0.005364
A_44_P242924	Trpc1	NM_053558	89821	-0.36	-2.293	0.022352
A_44_P175932	Tmem24	NM_001011996	300666	-0.36	-2.294	0.000066
A_44_P108185	AA819655	AA819655	691066	-0.36	-2.294	0.010329
A_44_P433923	LOC691522	XM_001078640		-0.36	-2.294	0.143057
A_44_P1024656	XM_228738	XM_228738		-0.36	-2.294	0.000557
A_44_P505671	Cipar1	NM_173114	286894	-0.36	-2.295	0.000111
A_44_P901483	TC547190	TC547190		-0.36	-2.295	0.043808
A_44_P430208	BG376471	BG376471	304361	-0.36	-2.295	0.022956
A_44_P279274	A_44_P279274	A_44_P279274		-0.36	-2.297	0.036652
A_44_P362486	BQ780215	BQ780215		-0.36	-2.297	0.011172
A_44_P386437	Olr1697_predicted	NM_001001111	406010	-0.36	-2.298	0.009693
A_44_P292634	Fth1	NM_012848	25319	-0.36	-2.298	0.007921
A_44_P402050	BE109691	BE109691		-0.36	-2.299	0.020208
A_44_P470207	BQ211747	BQ211747		-0.36	-2.299	0.001159
A_44_P488306	AI230784	AI230784		-0.36	-2.299	0.013402
A_44_P1051981	Evpl_predicted	XM_221129		-0.36	-2.300	0.000925
A_44_P512679	AI229025	AI229025		-0.36	-2.300	0.004808
A_44_P685023	TC559262	TC559262		-0.36	-2.301	0.000976
A_42_P485015	Lrrc4c_predicted	XM_230311		-0.36	-2.301	0.000456
A_44_P424962	AA875357	AA875357	24244	-0.36	-2.301	0.000651
A_44_P506828	AI112095	AI112095	303468	-0.36	-2.301	0.000244
A_44_P311636	RGD1564372_predicted	XM_344301		-0.36	-2.301	6.73E-05
A_42_P504653	F10	NM_017143	29243	-0.36	-2.303	0.011028
A_44_P147181	Ovol1_predicted	XM_219513		-0.36	-2.303	0.000647
A_44_P335564	LOC501251	XM_576670	687781	-0.36	-2.305	0.176415
A_44_P112622	XM_220924	XM_220924		-0.36	-2.305	0.003543
A_42_P624309	Ltc4s	NM_053639	114097	-0.36	-2.306	0.000846
A_44_P639137	TC543577	TC543577		-0.36	-2.307	0.000954
A_44_P372630	XM_218301	XM_218301		-0.36	-2.307	0.028747
A_44_P301367	AA800240	AA800240	170670	-0.36	-2.307	0.003861
A_43_P12698	Lgals3	NM_031832	83781	-0.36	-2.308	0.001157
A_44_P250763	XM_228010	XM_228010		-0.36	-2.309	0.007858
A_44_P555839	XM_217006	XM_217006		-0.36	-2.310	0.03553
A_44_P129840	AW525988	AW525988	300062	-0.36	-2.311	0.005178
A_44_P262662	LOC295241	XM_227413	295241	-0.36	-2.311	0.002744
A_44_P231457	Kcna1	NM_173095	24520	-0.36	-2.311	0.011807
A_44_P113551	BX883048	BX883048		-0.36	-2.311	0.001602
A_44_P503096	AI233877	AI233877		-0.36	-2.312	0.00355
A_44_P839849	TC545623	TC545623		-0.36	-2.313	0.000187
A_44_P201481	XM_227829	XM_227829		-0.36	-2.313	0.005999
A_42_P689905	Apoc2_predicted	XM_214872		-0.36	-2.315	0.000244
A_44_P876604	BE102568	BE102568	367932	-0.36	-2.315	0.018742
A_44_P378999	BI282349	BI282349	29740	-0.36	-2.315	0.025938
A_44_P1051724	Heph	NM_133304	117240	-0.36	-2.316	0.000445
A_44_P340418	A_44_P340418	A_44_P340418		-0.36	-2.317	0.000533
A_44_P132610	Slc20a1	NM_031148	81826	-0.37	-2.317	0.000341
A_42_P525886	Crabp1_mapped	XM_236253		-0.37	-2.317	0.003434

A_44_P975355	AI178492	AI178492		-0.37	-2.318	0.000773
A_44_P508549	Ppp1r12b_predicted	XM_222656		-0.37	-2.318	0.001046
A_43_P19450	Prkca	XM_343975		-0.37	-2.318	0.000121
A_44_P425058	Cspg3	NM_031653	58982	-0.37	-2.319	0.006826
A_44_P461612	Fmn2_predicted	XM_001058601		-0.37	-2.320	0.014198
A_44_P961053	TC558266	TC558266		-0.37	-2.320	0.017813
A_44_P316185	LOC307727	XM_001060574		-0.37	-2.321	0.003575
A_44_P281868	BI278309	BI278309	360887	-0.37	-2.321	0.00257
A_44_P378896	AI547815	AI547815	293526	-0.37	-2.321	0.00237
A_44_P424259	A_44_P424259	A_44_P424259		-0.37	-2.321	0.016627
A_44_P137517	MGC72615	NM_001009534	494321	-0.37	-2.323	0.002687
A_44_P303197	Svs5	NM_133516	171027	-0.37	-2.324	0.000413
A_44_P231498	Pax3	XM_343601	114502	-0.37	-2.325	0.020095
A_44_P340472	Sox8_predicted	XM_220283		-0.37	-2.325	0.001752
A_44_P217648	Mmd2_predicted	NM_001037217	304301	-0.37	-2.326	0.035042
A_44_P182757	XM_224063	XM_224063		-0.37	-2.326	0.034005
A_44_P307952	AI104089	AI104089	287383	-0.37	-2.328	0.000887
A_42_P576549	Mybpc3_predicted	XM_215771		-0.37	-2.328	0.011173
A_44_P222266	NM_172048	NM_172048		-0.37	-2.328	0.013501
A_44_P442589	Efna1	NM_053599	94268	-0.37	-2.329	0.001937
A_44_P353157	Tmem30b_predicted	XM_001080722		-0.37	-2.329	4.14E-05
A_44_P759686	Rnase1_predicted	CV795496	364303	-0.37	-2.331	0.000948
A_42_P480082	Golga2	NM_022596	64528	-0.37	-2.331	0.004801
A_44_P553249	RGD1561818_predicted	XM_228942	317494	-0.37	-2.331	0.008044
A_43_P12749	Nr1i2	NM_052980	84385	-0.37	-2.331	8.57E-05
A_43_P22744	AW920815	AW920815	302950	-0.37	-2.332	0.000153
A_44_P367767	LOC360721	NM_001029903	360721	-0.37	-2.333	0.000135
A_44_P443328	RGD1560898_predicted	XM_228803	317395	-0.37	-2.333	0.023604
A_44_P246136	LOC304551	XM_239684	304551	-0.37	-2.334	0.005281
A_44_P546104	XM_238028	XM_238028		-0.37	-2.335	0.000803
A_42_P601398	XM_238275	XM_238275		-0.37	-2.335	0.005487
A_44_P714729	TC551845	TC551845		-0.37	-2.335	0.000134
A_43_P22157	Pcdhga2	NM_001037139	116782	-0.37	-2.335	0.001757
A_44_P294914	RGD1562499_predicted	XM_218652	308586	-0.37	-2.336	0.001114
A_43_P14979	TC521314	TC521314		-0.37	-2.336	5.54E-05
A_44_P536333	Grin1	NM_017010	24408	-0.37	-2.336	0.004605
A_44_P748917	TC519640	TC519640		-0.37	-2.337	0.000149
A_44_P229007	Olr736_predicted	NM_001001384	366118	-0.37	-2.338	0.06363
A_43_P15909	BM493233	BM493233		-0.37	-2.338	0.01503
A_44_P368363	A_44_P368363	A_44_P368363		-0.37	-2.338	0.016
A_43_P19512	Ank3	NM_031805	361833	-0.37	-2.339	0.000266
A_44_P899264	TC537077	TC537077		-0.37	-2.339	0.000651
A_42_P526030	Decr1	NM_057197	117543	-0.37	-2.340	5.57E-05
A_44_P304024	Gmfg	NM_181091	113940	-0.37	-2.340	0.000531
A_44_P427885	AW141591	AW141591	24583	-0.37	-2.341	0.00264
A_44_P1001451	Pga5	NM_021753	60372	-0.37	-2.342	0.002387
A_43_P13491	Elmo1_predicted	XM_341532		-0.37	-2.343	4.58E-05
A_43_P22555	RGD1307524_predicted	XM_219909	309415	-0.37	-2.343	0.002267
A_44_P522524	Nr4a2	NM_019328	54278	-0.37	-2.343	0.029741
A_44_P375500	RGD1305248_predicted	XM_216738	299151	-0.37	-2.345	0.000341
A_44_P527985	BQ779714	BQ779714		-0.37	-2.345	0.01544
A_44_P378022	BI292178	BI292178		-0.37	-2.345	0.001651
A_44_P1035733	Dkc1	NM_133419	170944	-0.37	-2.345	0.004299
A_44_P304880	LOC366473	NM_001014261	366473	-0.37	-2.346	5.11E-05
A_44_P215420	XM_343737	XM_343737		-0.37	-2.346	0.009841
A_44_P337959	Slc1a7_predicted	XM_345559		-0.37	-2.346	0.004331
A_44_P821805	ENSRNOT00000029319	ENSRNOT00000029319		-0.37	-2.346	0.002096
A_44_P555417	RGD1310475_predicted	XM_215248		-0.37	-2.347	0.005091
A_44_P178366	Ndufs7	NM_001008525	362837	-0.37	-2.347	0.000475
A_44_P508632	Nkx2-3_predicted	XM_219880		-0.37	-2.347	0.000911
A_44_P837276	LOC684332	XM_001069899		-0.37	-2.348	0.038238
A_44_P410506	XM_237388	XM_237388		-0.37	-2.348	6.94E-05
A_44_P454351	Abo_predicted	XM_215986		-0.37	-2.349	0.000211

A_44_P313188	AW921026	AW921026		-0.37	-2.350	0.000622
A_44_P274154	Foxd2_predicted	XM_233422		-0.37	-2.350	0.001942
A_44_P898229	A_44_P898229	A_44_P898229		-0.37	-2.350	0.004091
A_44_P444116	AABR03025850	AABR03025850		-0.37	-2.350	0.001718
A_44_P515287	Olr1588_predicted	NM_001000082	289250	-0.37	-2.350	0.062761
A_44_P453196	AI716055	AI716055	364140	-0.37	-2.351	0.000998
A_44_P748575	LOC498796	NM_001025034	498796	-0.37	-2.352	2.74E-05
A_44_P439894	AI231572	AI231572	290985	-0.37	-2.352	0.018188
A_44_P489590	Gnao	NM_017327	50664	-0.37	-2.352	0.000133
A_44_P613805	A_44_P613805	A_44_P613805		-0.37	-2.352	0.000341
A_44_P1025667	Ccdc37_predicted	XM_216219	297444	-0.37	-2.353	0.003943
A_44_P265416	RGD1562727_predicted	XM_346076		-0.37	-2.353	0.000905
A_44_P730345	TC539999	TC539999		-0.37	-2.354	0.003567
A_44_P260358	Slc27a4	XM_231115	311839	-0.37	-2.355	0.000453
A_43_P10605	BM385672	BM385672	297784	-0.37	-2.355	0.00366
A_44_P570176	A_44_P570176	A_44_P570176		-0.37	-2.355	0.000259
A_43_P23192	RGD1565610_predicted	XM_340983	360712	-0.37	-2.355	0.002347
A_44_P273734	Man1a_predicted	BC090336	294410	-0.37	-2.356	0.007419
A_44_P513438	LOC364637	NM_001014239	364637	-0.37	-2.357	0.001512
A_44_P444122	AABR03025850	AABR03025850		-0.37	-2.357	0.004775
A_44_P125974	Gpx6	NM_147165	259233	-0.37	-2.358	0.003816
A_44_P243433	A_44_P243433	A_44_P243433		-0.37	-2.358	0.015665
A_44_P371984	Dpp4	J02997	25253	-0.37	-2.359	0.053371
A_44_P429286	XM_345589	XM_345589		-0.37	-2.359	0.004799
A_44_P356312	XM_215022	XM_215022		-0.37	-2.360	0.00043
A_43_P15533	Cebpe	NM_017095	25410	-0.37	-2.361	0.00552
A_44_P547191	BG378227	BG378227	24392	-0.37	-2.362	0.006271
A_44_P182306	Olr563_predicted	NM_001000666	404868	-0.37	-2.362	0.001897
A_44_P210961	A_44_P210961	A_44_P210961		-0.37	-2.362	0.000963
A_43_P22815	RGD1559578_predicted	XM_213524	287783	-0.37	-2.362	0.012408
A_44_P222161	Col10a1	XM_001053056	25681	-0.37	-2.362	0.008918
A_44_P322929	BQ190679	BQ190679	500006	-0.37	-2.363	0.001361
A_44_P523361	AI137731	AI137731		-0.37	-2.364	0.010086
A_44_P1049844	LOC500797	XM_001077148		-0.37	-2.364	0.000368
A_44_P1048795	Dst_predicted	XM_237042		-0.37	-2.364	0.005979
A_43_P18271	RGD1564335_predicted	XM_576264	500865	-0.37	-2.365	0.003202
A_44_P313507	Cdkn2b	NM_130812	25164	-0.37	-2.366	0.002888
A_44_P610959	TC564657	TC564657		-0.37	-2.366	0.0005
A_44_P412064	Olr218_predicted	NM_001000551	365340	-0.37	-2.366	0.000611
A_44_P508924	LOC691594	XM_001078957	691594	-0.37	-2.367	0.098173
A_44_P525482	CB546704	CB546704		-0.37	-2.367	0.003306
A_44_P478488	A_44_P478488	A_44_P478488		-0.37	-2.367	0.019405
A_42_P612109	Crygc	XM_343582		-0.37	-2.367	0.075823
A_44_P514112	BQ210707	BQ210707	24183	-0.37	-2.368	0.027115
A_44_P590739	RGD1565512_predicted	XM_575095	499759	-0.37	-2.368	0.01125
A_43_P11965	Arl4a	NM_019186	29308	-0.37	-2.369	7.45E-05
A_44_P638446	TC558628	TC558628		-0.37	-2.369	0.000283
A_43_P12956	Tff2	NM_053844	116592	-0.37	-2.369	0.000653
A_44_P342314	RGD1308373	NM_001014232	364208	-0.37	-2.370	0.00212
A_44_P1071120	ENSRNOT00000046889	ENSRNOT00000046889		-0.37	-2.371	0.000484
A_44_P367013	BG377024	BG377024		-0.37	-2.371	0.012333
A_44_P439554	BI294349	BI294349	297685	-0.38	-2.371	0.000484
A_44_P306339	Atp7b	NM_012511	24218	-0.38	-2.371	4.29E-05
A_44_P195803	AW917161	AW917161	293949	-0.38	-2.372	0.000372
A_44_P218256	LOC682510	XM_001061801		-0.38	-2.372	0.016862
A_44_P493894	M27315	M27315		-0.38	-2.372	0.000259
A_44_P792063	TC556542	TC556542		-0.38	-2.372	0.000314
A_44_P326495	XM_573167	XM_573167		-0.38	-2.372	0.000584
A_44_P214595	AI059015	AI059015		-0.38	-2.374	0.031721
A_44_P353850	XM_217851	XM_217851		-0.38	-2.374	0.002173
A_44_P226658	Scnn1g	NM_017046	24768	-0.38	-2.374	0.038087
A_44_P359216	A_44_P359216	A_44_P359216		-0.38	-2.374	0.004168
A_44_P534415	XM_214608	XM_214608		-0.38	-2.374	0.006264

A_42_P733671	Tnfsf10	NM_145681	246775	-0.38	-2.375	0.004217
A_44_P809794	TC566405	TC566405		-0.38	-2.375	0.008332
A_44_P262989	Mfrp_predicted	XM_236179		-0.38	-2.376	0.004536
A_44_P542716	AI112577	AI112577	114117	-0.38	-2.377	0.000431
A_44_P349375	A_44_P349375	A_44_P349375		-0.38	-2.377	0.001029
A_44_P163176	Tor1aip2	NM_199100	304881	-0.38	-2.378	0.000496
A_44_P116858	A_44_P116858	A_44_P116858		-0.38	-2.378	0.056801
A_44_P693334	CK600448	CK600448		-0.38	-2.378	0.009836
A_44_P248644	RGD1562597_predicted	XM_222536	304721	-0.38	-2.378	2.34E-05
A_44_P533406	AA999049	AA999049	29421	-0.38	-2.378	0.014018
A_44_P424441	XM_347136	XM_347136		-0.38	-2.379	0.000122
A_44_P465233	Fgfbp1	NM_022603	64535	-0.38	-2.379	0.00117
A_44_P293243	A_44_P293243	A_44_P293243		-0.38	-2.380	0.001912
A_44_P117142	RGD1308559_predicted	XM_233587		-0.38	-2.380	0.00549
A_44_P283153	Cdig1l	NM_153623		-0.38	-2.380	0.004767
A_44_P261444	Nfkbia	XM_001075778		-0.38	-2.381	0.007237
A_44_P397153	AA875004	AA875004	300687	-0.38	-2.381	0.002484
A_44_P592757	DV718099	DV718099	310207	-0.38	-2.382	0.020292
A_44_P282210	AA925463	AA925463	29372	-0.38	-2.382	0.00054
A_44_P739855	BI292154	BI292154	500366	-0.38	-2.382	0.000465
A_44_P450089	XM_223808	XM_223808		-0.38	-2.383	0.012259
A_44_P220353	XM_228242	XM_228242		-0.38	-2.383	0.034161
A_44_P809642	TC549439	TC549439		-0.38	-2.384	0.00054
A_42_P502582	Ela3b_predicted	XM_216566		-0.38	-2.384	0.000228
A_44_P898115	MGC125213	NM_001034153	498146	-0.38	-2.385	0.000603
A_44_P236486	RGD1564583_predicted	XM_227557		-0.38	-2.385	0.0008
A_44_P378322	AA858768	AA858768		-0.38	-2.385	0.04491
A_43_P17833	RGD1565470_predicted	XM_001068791		-0.38	-2.385	0.006823
A_44_P289925	AW913991	AW913991	680066	-0.38	-2.387	0.001675
A_44_P412032	Olr1700_predicted	NM_001001113	406012	-0.38	-2.388	0.004075
A_44_P454736	RGD1560896_predicted	XM_574173	498885	-0.38	-2.388	9.78E-05
A_43_P13160	Grin3b	NM_133308	170796	-0.38	-2.388	0.00096
A_44_P520005	Chst7	NM_207600	302302	-0.38	-2.388	0.001957
A_44_P154522	RGD1566031_predicted	XM_228906		-0.38	-2.388	0.011086
A_44_P1007870	Wfdc3_predicted	XM_215938		-0.38	-2.388	0.001444
A_44_P159070	A_44_P159070	A_44_P159070		-0.38	-2.389	0.000243
A_44_P562556	TC542712	TC542712		-0.38	-2.390	0.001682
A_44_P858108	TC551639	TC551639		-0.38	-2.390	0.007894
A_43_P13083	Tacr2	NM_080768	25007	-0.38	-2.391	0.00475
A_44_P123841	XM_215441	XM_215441		-0.38	-2.392	0.003941
A_44_P243469	LOC682925	XM_001064626		-0.38	-2.392	0.001261
A_44_P885531	TC543896	TC543896		-0.38	-2.392	0.002364
A_44_P180576	XM_226009	XM_226009		-0.38	-2.393	0.007355
A_43_P14989	RGD1307907_predicted	XM_001081122		-0.38	-2.393	0.000629
A_44_P955260	AW921008	AW921008		-0.38	-2.393	0.005698
A_42_P707132	Prap1	NM_031669	60574	-0.38	-2.393	0.002373
A_43_P16395	Rhbdl1	XM_340773	117025	-0.38	-2.394	0.00194
A_44_P262617	XM_214166	XM_214166		-0.38	-2.394	0.040794
A_44_P492543	LOC315953	NM_001014096	315953	-0.38	-2.395	0.000514
A_44_P161589	RGD1305697_predicted	XM_231426		-0.38	-2.395	0.000564
A_44_P119371	RGD1562488_predicted	XM_215321	294268	-0.38	-2.396	0.003823
A_44_P401498	Sema6a_predicted	XM_341612		-0.38	-2.396	0.004039
A_44_P157037	Prrg3_predicted	XM_219845	293935	-0.38	-2.397	0.020476
A_44_P1043814	Dhrs8	NM_001004209	289456	-0.38	-2.397	0.000105
A_44_P402750	Gda	NM_031776	83585	-0.38	-2.397	0.000368
A_43_P11230	RGD1563633_predicted	XM_238366	297504	-0.38	-2.398	0.001384
A_43_P21769	Glt25d2_predicted	XM_222718	289081	-0.38	-2.398	0.016322
A_44_P222404	RGD1310371	XM_218852	308794	-0.38	-2.400	0.046061
A_44_P461251	Olr30_predicted	NM_001000120	293091	-0.38	-2.401	0.010983
A_44_P127869	Id4	NM_175582	291023	-0.38	-2.401	0.001142
A_44_P402737	LOC501618	XM_577017		-0.38	-2.401	0.002331
A_44_P702082	TC546713	TC546713		-0.38	-2.402	0.000873
A_43_P14461	LOC498749	NM_001034947	498749	-0.38	-2.403	0.000118

A_44_P241781	BF558420	BF558420	314751	-0.38	-2.404	0.000405
A_44_P668388	RGD1561494_predicted	XM_575577		-0.38	-2.404	0.000485
A_44_P957585	AI227704	AI227704	50664	-0.38	-2.405	0.00058
A_44_P555334	Etnk2_predicted	XM_341119		-0.38	-2.405	0.008423
A_44_P149466	XM_344060	XM_344060		-0.38	-2.406	0.000406
A_44_P994368	Nrap_predicted	XM_217637		-0.38	-2.407	0.009802
A_44_P112181	RGD1311241_predicted	XM_226757	310086	-0.38	-2.407	0.007438
A_44_P333345	Abhd9_predicted	XM_345799		-0.38	-2.407	0.001931
A_44_P1017315	Vhl	NM_052801	24874	-0.38	-2.408	0.000133
A_44_P531821	Hoxc4_mapped	XM_235703		-0.38	-2.408	0.002134
A_44_P440988	Nt5dc1_predicted	XM_215405		-0.38	-2.409	0.000399
A_44_P527902	XM_220257	XM_220257		-0.38	-2.409	0.015836
A_43_P12864	Pgf	NM_053595	94203	-0.38	-2.409	0.007379
A_44_P101066	AI575373	AI575373	293950	-0.38	-2.410	0.015024
A_44_P373222	XM_343871	XM_343871		-0.38	-2.410	0.002018
A_44_P983188	AA818818	AA818818	500705	-0.38	-2.411	0.000246
A_44_P332821	XM_218048	XM_218048		-0.38	-2.411	0.018654
A_44_P322910	Pdgfa	NM_012801	25266	-0.38	-2.412	0.000833
A_44_P152177	Spock2_predicted	XM_342134		-0.38	-2.412	0.001023
A_44_P548286	A_44_P548286	A_44_P548286		-0.38	-2.413	0.000745
A_44_P154392	XM_347024	XM_347024		-0.38	-2.414	0.010961
A_44_P233956	Cyp4f5	NM_173124	286905	-0.38	-2.415	0.000685
A_44_P389178	RGD1560911_predicted	XM_219486	309112	-0.38	-2.415	3.09E-05
A_44_P762552	TC559607	TC559607		-0.38	-2.416	0.007224
A_44_P131240	Dst_predicted	XM_237042		-0.38	-2.416	0.00062
A_44_P114692	LOC363433	XM_343755		-0.38	-2.417	0.01343
A_44_P340498	RGD1559720_predicted	XM_213445		-0.38	-2.417	0.000415
A_44_P775910	RGD1562739_predicted	XM_576274	500875	-0.38	-2.418	0.000476
A_43_P15910	Tcra-v22.1	XM_224084		-0.38	-2.418	0.002506
A_44_P144482	Sert1	AF077195	246151	-0.38	-2.419	0.000482
A_44_P628544	A_44_P628544	A_44_P628544		-0.38	-2.419	0.014816
A_44_P637856	TC534459	TC534459		-0.38	-2.420	4.97E-05
A_44_P326425	RGD1310265_predicted	XM_001070727		-0.38	-2.420	0.002071
A_42_P540609	LOC680711	XM_001056892	680711	-0.38	-2.420	0.001247
A_44_P325785	TC558014	TC558014		-0.38	-2.421	0.000143
A_44_P744256	LOC691120	XM_001077920		-0.38	-2.421	0.000423
A_44_P698456	A_44_P698456	A_44_P698456		-0.38	-2.421	0.031239
A_44_P229670	LOC687464	XM_001078688		-0.38	-2.422	0.126964
A_44_P113035	RGD1564638_predicted	XM_220173	287060	-0.38	-2.422	0.016433
A_43_P12240	Hif3a	NM_022528	64345	-0.38	-2.423	0.000149
A_44_P240588	AA817929	AA817929	64561	-0.38	-2.424	0.011838
A_44_P494869	Guca1a_predicted	XM_217341		-0.38	-2.424	0.004143
A_43_P19071	RGD1564947_predicted	XM_228781	317368	-0.38	-2.425	0.006823
A_44_P144257	Fzd5	NM_173838	317674	-0.38	-2.425	0.001266
A_44_P918516	TC563612	TC563612		-0.38	-2.425	0.002193
A_44_P509763	AA955473	AA955473	294337	-0.39	-2.427	0.015237
A_44_P238738	LOC501091	XM_576506	501091	-0.39	-2.428	0.126659
A_44_P403133	XM_344628	XM_344628		-0.39	-2.428	0.012298
A_44_P456256	AA925161	AA925161	25062	-0.39	-2.428	0.01994
A_44_P482995	XM_226523	XM_226523		-0.39	-2.430	0.005884
A_44_P204499	BF546233	BF546233	292263	-0.39	-2.431	0.003518
A_44_P486872	BF281782	BF281782		-0.39	-2.431	0.023068
A_44_P213289	Manea	NM_080785	140808	-0.39	-2.432	0.004866
A_44_P330101	BF389646	BF389646	499210	-0.39	-2.432	0.001426
A_44_P606329	Ppp2r3a	XM_576459	363122	-0.39	-2.432	0.000557
A_44_P823597	Akap7	NM_001001801	361458	-0.39	-2.432	0.00026
A_44_P552604	Olr734_predicted	NM_001000617	404809	-0.39	-2.432	0.020747
A_43_P11998	Scn11a	NM_019265	29701	-0.39	-2.433	0.012875
A_44_P187215	Dio3	NM_017210	29475	-0.39	-2.433	0.002785
A_44_P1055506	LOC683733	XM_001072472		-0.39	-2.434	0.000302
A_44_P124324	RGD1563442_predicted	XM_220977	303537	-0.39	-2.434	0.003252
A_44_P760751	ENSRNOT00000050216	ENSRNOT00000050216		-0.39	-2.434	0.002461
A_44_P215680	LOC690042	XM_001060929	690042	-0.39	-2.435	0.017677

A_44_P347230	LOC691491	XM_001078524		-0.39	-2.435	0.025203
A_44_P307567	AA955917	AA955917		-0.39	-2.436	0.002302
A_44_P308768	Olr1256_predicted	NM_001001086	405977	-0.39	-2.436	0.047342
A_44_P335468	XM_226040	XM_226040		-0.39	-2.437	0.008151
A_44_P506245	XM_344382	XM_344382		-0.39	-2.439	0.004397
A_44_P226832	Rasgrf1	XM_001063727		-0.39	-2.439	0.000464
A_44_P398717	XM_234629	XM_234629		-0.39	-2.440	0.00625
A_44_P404853	Acacb	NM_053922	116719	-0.39	-2.440	0.005643
A_44_P906402	BG374064	BG374064		-0.39	-2.440	0.021696
A_42_P509766	Ppara	NM_013196	25747	-0.39	-2.443	0.000358
A_43_P13175	Cry2	NM_133405	170917	-0.39	-2.443	0.000224
A_44_P851810	Cycs	NM_012839	25309	-0.39	-2.443	0.000106
A_44_P175797	RGD1563378_predicted	XM_228994		-0.39	-2.444	0.007712
A_44_P958659	AI136123	AI136123		-0.39	-2.444	0.010129
A_44_P180529	AABR03116794	AABR03116794		-0.39	-2.444	0.000678
A_44_P529645	RGD1565374_predicted	XM_573339	498128	-0.39	-2.444	0.008617
A_44_P516948	AW920944	AW920944		-0.39	-2.446	0.000588
A_43_P19939	TC563650	TC563650		-0.39	-2.446	0.000962
A_44_P394033	XM_346139	XM_346139		-0.39	-2.446	0.015047
A_44_P335797	A_44_P335797	A_44_P335797		-0.39	-2.447	0.000422
A_44_P172197	XM_216802	XM_216802		-0.39	-2.447	0.000373
A_44_P916173	TC544155	TC544155		-0.39	-2.448	0.005085
A_44_P1004218	Gne	NM_053765	114711	-0.39	-2.448	0.00027
A_44_P236187	Lgals7	NM_022582	29518	-0.39	-2.448	0.000201
A_42_P633797	Dpep2	NM_001011928	291984	-0.39	-2.448	0.000348
A_44_P166449	Znf494_predicted	XM_234948		-0.39	-2.448	0.000782
A_43_P22739	RGD1565785_predicted	XM_001061435		-0.39	-2.448	0.027637
A_44_P355059	AA819118	AA819118	25331	-0.39	-2.449	0.000767
A_44_P527972	AA900269	AA900269		-0.39	-2.449	0.022243
A_44_P808244	TC539843	TC539843		-0.39	-2.449	0.004291
A_42_P470649	Dio1	NM_021653	25430	-0.39	-2.451	0.020999
A_44_P173255	XM_345305	XM_345305		-0.39	-2.452	0.010835
A_43_P12500	Nell2	NM_031070	81734	-0.39	-2.452	0.03276
A_44_P357835	BF282088	BF282088		-0.39	-2.452	0.001881
A_44_P403672	AA818090	AA818090		-0.39	-2.453	0.000565
A_44_P421738	ENSRNOT00000037335	ENSRNOT00000037335		-0.39	-2.454	0.000536
A_44_P167452	Y17323	Y17323		-0.39	-2.454	0.005117
A_44_P109135	BF393807	BF393807	303614	-0.39	-2.455	0.003505
A_43_P17632	Camta2_predicted	XM_213362		-0.39	-2.455	0.000138
A_42_P461587	Ngfb_mapped	XM_227525	310738	-0.39	-2.456	0.00053
A_44_P532625	BG374259	BG374259	25521	-0.39	-2.456	0.020084
A_44_P419275	Dcamkl1	NM_053343	83825	-0.39	-2.456	0.000348
A_44_P224477	Olr1358_predicted	NM_214822	287077	-0.39	-2.457	0.00084
A_44_P490353	AA851395	AA851395		-0.39	-2.458	0.039878
A_44_P308877	Dmd	NM_001005244	24907	-0.39	-2.458	0.000705
A_44_P344228	RT1-DOa	NM_183051	24984	-0.39	-2.459	0.004429
A_44_P365794	Pdgfrl	NM_001011921	290771	-0.39	-2.460	0.000309
A_44_P1026191	LOC362156	NM_001039338	362156	-0.39	-2.460	0.002596
A_44_P347431	A_44_P347431	A_44_P347431		-0.39	-2.460	0.003321
A_44_P185246	A_44_P185246	A_44_P185246		-0.39	-2.460	0.030322
A_44_P213687	BF399537	BF399537	498311	-0.39	-2.460	0.019459
A_44_P410005	Ugp2	NM_001024743	289827	-0.39	-2.461	0.000074
A_44_P279249	A_44_P279249	A_44_P279249		-0.39	-2.462	0.013199
A_44_P182494	XM_224104	XM_224104		-0.39	-2.463	0.014949
A_44_P980898	CV795283	CV795283		-0.39	-2.463	0.006544
A_44_P729029	RGD1559502_predicted	XM_575420		-0.39	-2.466	0.00374
A_42_P598412	Apobec1	NM_012907	25383	-0.39	-2.466	0.00014
A_44_P115764	AA996522	AA996522	362103	-0.39	-2.466	0.002629
A_44_P197263	BF282730	BF282730		-0.39	-2.469	0.000417
A_44_P379940	XM_345228	XM_345228		-0.39	-2.469	0.000765
A_44_P180614	LOC691653	XM_001079159	691653	-0.39	-2.471	0.001074
A_44_P531098	AI716248	AI716248	361749	-0.39	-2.471	0.005806
A_44_P454646	RGD1308581_predicted	XM_341181	360906	-0.39	-2.471	0.004695

A_44_P858575	BF523622	BF523622		-0.39	-2.471	0.000149
A_42_P725318	Cdk5r1	NM_053891	116671	-0.39	-2.471	0.000115
A_44_P558054	A_44_P558054	A_44_P558054		-0.39	-2.473	0.01259
A_44_P172901	Cftr	NM_031506	24255	-0.39	-2.474	0.000281
A_44_P306551	Cacna1a	NM_012918	25398	-0.39	-2.475	0.000187
A_44_P184110	AI101158	AI101158		-0.39	-2.475	0.000505
A_44_P633413	RGD1306809_predicted	XM_345167		-0.39	-2.475	0.053904
A_43_P13816	CB547130	CB547130		-0.39	-2.475	0.001235
A_44_P318235	AA891949	AA891949	29223	-0.39	-2.476	0.009551
A_44_P171118	RGD1564843_predicted	XM_229068		-0.39	-2.476	0.002497
A_44_P425837	AI011756	AI011756	289231	-0.39	-2.476	0.000427
A_44_P123566	AF203698	AF203698		-0.39	-2.477	0.000178
A_44_P506113	RGD1561380_predicted	XM_218285		-0.39	-2.478	0.002527
A_44_P190329	LOC682604	XM_001062257		-0.39	-2.478	0.013488
A_44_P155956	AW918633	AW918633	300721	-0.39	-2.479	0.001366
A_44_P424405	Nbpwr1	NM_001014784	297795	-0.39	-2.479	0.016198
A_44_P808189	TC540063	TC540063		-0.39	-2.479	0.000825
A_44_P609306	TC550189	TC550189		-0.39	-2.480	0.026252
A_44_P369637	AA945533	AA945533	58978	-0.39	-2.480	0.000408
A_44_P485440	XM_229422	XM_229422		-0.39	-2.480	0.039802
A_44_P489841	Slitrk3_predicted	XM_227282		-0.39	-2.481	0.004379
A_44_P171427	RGD1310376_predicted	XM_001063105		-0.39	-2.481	0.000586
A_44_P167930	Mobp	NM_012720	25037	-0.39	-2.481	0.005074
A_42_P756696	Cpeb4_predicted	XM_220269		-0.39	-2.482	0.002269
A_44_P310306	M27315	M27315		-0.39	-2.482	0.001582
A_44_P316066	LOC287992	AY383659	287992	-0.40	-2.483	0.002975
A_44_P1040926	RGD1562954_predicted	XM_225544		-0.40	-2.483	0.001882
A_42_P467277	Gabbr2	NM_031802	83633	-0.40	-2.483	0.014689
A_44_P239887	BE120516	BE120516		-0.40	-2.484	0.000708
A_44_P744401	Tbx5	NM_001009964	304514	-0.40	-2.484	0.000598
A_44_P355405	BF403853	BF403853		-0.40	-2.484	0.013813
A_44_P485029	LOC301971	XR_007977	301971	-0.40	-2.485	0.00679
A_44_P190260	A_44_P190260	A_44_P190260		-0.40	-2.485	0.003767
A_43_P12136	Crx	NM_021855	60446	-0.40	-2.486	0.013272
A_44_P448002	RGD1308684_predicted	XM_001058226		-0.40	-2.486	0.001243
A_43_P19175	Dscaml1_predicted	XM_236203		-0.40	-2.486	0.06064
A_44_P299749	XM_345006	XM_345006		-0.40	-2.486	0.000121
A_44_P417206	RGD1561620_predicted	XM_225507		-0.40	-2.486	0.027157
A_44_P288849	XM_220757	XM_220757		-0.40	-2.487	0.015095
A_44_P116875	LOC368158	XM_347322	499668	-0.40	-2.487	0.000222
A_44_P488078	Centg2_predicted	XM_237381		-0.40	-2.488	0.0017
A_44_P153789	Ache	NM_172009	83817	-0.40	-2.489	0.02524
A_44_P485684	AI408856	AI408856		-0.40	-2.490	0.009851
A_43_P12247	Fthfd	NM_022547	64392	-0.40	-2.490	0.000704
A_43_P16184	AJ007467	AJ007467		-0.40	-2.490	0.006034
A_44_P395384	AI229116	AI229116	498922	-0.40	-2.491	0.006807
A_42_P748540	Slc6a17	NM_001033079	613226	-0.40	-2.492	0.005139
A_44_P534762	Wnt3a_predicted	XM_220546		-0.40	-2.492	0.011387
A_42_P455982	Pgm1_predicted	XM_214047		-0.40	-2.493	0.00032
A_44_P407914	LOC302576	NM_001013959	302576	-0.40	-2.493	0.023893
A_44_P103956	CB546220	CB546220	360577	-0.40	-2.494	0.005055
A_44_P232285	A_44_P232285	A_44_P232285		-0.40	-2.495	0.000123
A_44_P387299	TC528577	TC528577		-0.40	-2.495	0.002223
A_44_P225307	XM_343878	XM_343878		-0.40	-2.496	0.007372
A_44_P868262	TC555772	TC555772		-0.40	-2.496	0.000153
A_43_P15576	Chn1	NM_032083	84030	-0.40	-2.496	0.002009
A_44_P745827	TC539254	TC539254		-0.40	-2.497	0.000121
A_44_P948592	TC527204	TC527204		-0.40	-2.498	0.000589
A_44_P248282	LOC685102	XM_001062306		-0.40	-2.499	0.004325
A_44_P103309	RGD1565739_predicted	XM_237343	301543	-0.40	-2.499	0.002258
A_44_P260679	Golph3l	NM_001007698	310669	-0.40	-2.500	7.61E-05
A_42_P774527	Gzmc	NM_134332	171290	-0.40	-2.500	0.005557
A_44_P265225	XM_347122	XM_347122		-0.40	-2.500	0.132934

A_44_P257788	Trpd52l3_predicted	XM_001079669		-0.40	-2.500	0.024544
A_43_P18228	RGD1560938_predicted	XM_343031	362713	-0.40	-2.500	0.002874
A_44_P165203	AA996426	AA996426	25390	-0.40	-2.502	0.001011
A_44_P102881	A_44_P102881	A_44_P102881		-0.40	-2.503	0.020842
A_43_P16643	Abhd4_predicted	XM_344403		-0.40	-2.504	0.08854
A_44_P148196	Klk5	XM_574439		-0.40	-2.504	0.007206
A_44_P206195	RGD1565321_predicted	XM_344642		-0.40	-2.504	0.005602
A_42_P491486	Pllp	NM_022533	64364	-0.40	-2.504	0.000845
A_43_P20833	Bai1_predicted	XM_343260	362931	-0.40	-2.505	0.000621
A_44_P492298	ENSRNOT00000032928	ENSRNOT00000032928		-0.40	-2.505	0.008527
A_44_P155838	AI136420	AI136420	294810	-0.40	-2.506	0.000741
A_44_P384583	Trim36_predicted	XM_225947		-0.40	-2.506	0.000528
A_44_P978641	A_44_P978641	A_44_P978641		-0.40	-2.510	0.001227
A_44_P454882	A_44_P454882	A_44_P454882		-0.40	-2.511	0.009472
A_44_P729266	LOC300506	XR_005718		-0.40	-2.511	0.001688
A_44_P215760	A_44_P215760	A_44_P215760		-0.40	-2.511	0.000444
A_44_P532320	Wdr32_predicted	XM_232999		-0.40	-2.511	0.058119
A_44_P231337	Olr637_predicted	NM_001001126	406053	-0.40	-2.512	0.042598
A_44_P496798	A_44_P496798	A_44_P496798		-0.40	-2.512	0.002312
A_44_P278113	M27315	M27315		-0.40	-2.513	0.000414
A_44_P346011	AW251927	AW251927	300024	-0.40	-2.514	0.000413
A_44_P156600	Mcoln1_predicted	XM_213684		-0.40	-2.515	0.000211
A_44_P852373	ENSRNOT00000034929	ENSRNOT00000034929		-0.40	-2.517	0.005963
A_42_P598531	Cds1	NM_031242	81925	-0.40	-2.517	0.001989
A_44_P860207	BQ196586	BQ196586	362935	-0.40	-2.517	0.003156
A_44_P175954	Slc25a5	NM_057102	25176	-0.40	-2.518	0.000106
A_44_P100365	Scn2a1	NM_012647	24766	-0.40	-2.518	0.01212
A_43_P11935	Cdon	NM_017358	50938	-0.40	-2.519	0.007551
A_44_P520737	Oxct2a	NM_001012221	366463	-0.40	-2.520	0.006366
A_44_P844543	AI179520	AI179520		-0.40	-2.521	0.000453
A_44_P456760	RGD1565364_predicted	XM_575779	500418	-0.40	-2.522	0.001567
A_44_P626893	TC566859	TC566859		-0.40	-2.523	0.000372
A_44_P676132	BF289533	BF289533		-0.40	-2.523	0.003366
A_44_P443500	RGD1309808_predicted	XM_343282	686810	-0.40	-2.523	1.95E-05
A_44_P593161	Gna14	NM_001013151	309242	-0.40	-2.524	0.001931
A_44_P412200	NP516857	NP516857		-0.40	-2.524	0.000278
A_44_P468728	RGD1308114	NM_001014146	361253	-0.40	-2.526	0.001734
A_44_P925737	BF281791	BF281791	29556	-0.40	-2.526	0.014995
A_44_P194793	BM387908	BM387908		-0.40	-2.526	0.011071
A_44_P944006	LOC499337	XM_001078906		-0.40	-2.526	3.69E-05
A_44_P714816	CA509943	CA509943		-0.40	-2.527	7.52E-05
A_44_P412147	M26926	M26926		-0.40	-2.527	0.000764
A_44_P347199	Fbxo10_predicted	XM_342829	362511	-0.40	-2.527	0.000283
A_44_P141996	Olr1450_predicted	NM_001000774	405049	-0.40	-2.528	0.015519
A_44_P793873	TC563317	TC563317		-0.40	-2.528	0.005861
A_44_P460743	AI008386	AI008386	303158	-0.40	-2.529	0.006993
A_44_P355149	BX883042	BX883042		-0.40	-2.530	0.000184
A_44_P721599	A_44_P721599	A_44_P721599		-0.40	-2.533	0.003333
A_44_P298473	Siat7F	NM_001015036	407765	-0.40	-2.535	0.001426
A_44_P490308	Muc20_predicted	XM_221383		-0.40	-2.535	0.001725
A_44_P128201	U25282	U25282		-0.40	-2.535	0.000461
A_44_P348154	Snca	NM_019169	29219	-0.40	-2.535	0.058261
A_43_P19032	LOC681994	XM_001057753		-0.40	-2.536	0.012458
A_43_P12511	Grk1	NM_031096	81760	-0.40	-2.536	0.003567
A_44_P100659	RGD1564535_predicted	XM_345326		-0.40	-2.538	0.001089
A_44_P698168	LOC689048	XM_001069328	689048	-0.40	-2.538	0.006052
A_44_P554985	Olr1391_predicted	NM_001001089	405983	-0.40	-2.538	0.000111
A_44_P169472	AB033702	AB033702		-0.40	-2.538	0.000358
A_44_P470595	Olr1344_predicted	NM_001000483	301610	-0.40	-2.539	0.007849
A_44_P332088	BF284283	BF284283	298567	-0.40	-2.539	0.001233
A_44_P130270	Nudt6	NM_181363	207120	-0.40	-2.540	0.001853
A_43_P12860	Cox5b	NM_053586	94194	-0.41	-2.542	0.000251
A_44_P1020923	Arr3	XM_001053467	171107	-0.41	-2.542	0.001271

A_44_P1048380	RGD1311381_predicted	XM_217218		-0.41	-2.542	0.000173
A_44_P448041	Abcb11	XM_579531	83569	-0.41	-2.542	0.004767
A_44_P299733	LOC501251	XM_576670	687781	-0.41	-2.543	0.011358
A_44_P433593	Phox2b_predicted	XM_344239	364152	-0.41	-2.544	0.001701
A_44_P181411	AI102920	AI102920	296368	-0.41	-2.544	0.00018
A_44_P453311	BF388434	BF388434	299909	-0.41	-2.544	0.00045
A_44_P148422	AI535219	AI535219	306817	-0.41	-2.544	0.000847
A_44_P356829	RGD1306484_predicted	XM_343890		-0.41	-2.544	0.001776
A_42_P754888	Dlk1	NM_053744	114587	-0.41	-2.545	0.011503
A_44_P126692	AI231808	AI231808	310811	-0.41	-2.545	0.003429
A_44_P442765	Krt14	D63774	303530	-0.41	-2.546	0.001097
A_44_P363927	BG377273	BG377273	289419	-0.41	-2.547	0.001283
A_44_P731232	TC560546	TC560546		-0.41	-2.547	0.000271
A_43_P15111	Tmem9b_predicted	XM_001073254		-0.41	-2.548	2.22E-05
A_44_P251045	Aox2	NM_001008522	316421	-0.41	-2.550	0.00027
A_44_P409896	A_44_P409896	A_44_P409896		-0.41	-2.550	0.014749
A_44_P431169	Chst8_predicted	XM_218506		-0.41	-2.550	0.009229
A_44_P233557	RSB-11-77	NM_182669	293180	-0.41	-2.551	0.000184
A_44_P259745	Dsg4	NM_199490	291754	-0.41	-2.552	0.023208
A_44_P760170	ENSRNOT00000008154	ENSRNOT00000008154		-0.41	-2.552	0.002572
A_44_P143886	Insig1	NM_022392	64194	-0.41	-2.553	8.92E-05
A_43_P13410	Serpina4	NM_145097	246328	-0.41	-2.553	0.011559
A_44_P316194	Cyp2b15	NM_017156	29295	-0.41	-2.554	0.02873
A_43_P22936	Akap3	NM_001005557	312720	-0.41	-2.557	0.001068
A_44_P332568	Olr1381_predicted	NM_001000523	363549	-0.41	-2.558	0.000708
A_44_P193564	CA503849	CA503849	310324	-0.41	-2.558	0.008498
A_44_P475144	BF282856	BF282856	298091	-0.41	-2.560	0.002384
A_44_P400272	Olr771_predicted	NM_001000610	404799	-0.41	-2.561	0.009128
A_44_P409927	RGD1563034_predicted	XM_218458	292720	-0.41	-2.561	0.001153
A_44_P102901	A_44_P102901	A_44_P102901		-0.41	-2.561	0.000745
A_44_P207677	AA900863	AA900863	287436	-0.41	-2.563	0.01163
A_44_P424665	A_44_P424665	A_44_P424665		-0.41	-2.563	0.00037
A_44_P100185	Ctnnd2	XM_001065606		-0.41	-2.563	0.011517
A_44_P698307	LOC679750	XM_001054323	679750	-0.41	-2.563	0.005176
A_44_P271339	BF562149	BF562149	114845	-0.41	-2.564	0.000128
A_44_P474646	BF283351	BF283351	362900	-0.41	-2.564	0.004162
A_42_P586943	Adra1b	NM_016991	24173	-0.41	-2.565	0.001196
A_44_P545717	Cacna1e	NM_019294	54234	-0.41	-2.565	0.000137
A_44_P196835	RGD1305821	NM_001012349	296635	-0.41	-2.566	0.003618
A_44_P406665	AI171088	AI171088	84596	-0.41	-2.566	0.000298
A_44_P389034	Trp53inp2	XM_342551	362246	-0.41	-2.566	4.43E-05
A_44_P321253	LOC686543	XM_001074671		-0.41	-2.567	0.012954
A_42_P540774	Acadm	NM_016986	24158	-0.41	-2.567	4.68E-05
A_44_P531414	Il18	AY077842	29197	-0.41	-2.568	0.001891
A_44_P407259	Usp2	NM_053774	115771	-0.41	-2.568	0.002373
A_43_P10503	TC536486	TC536486		-0.41	-2.569	0.006018
A_44_P523190	RGD1311756_predicted	XM_343095	362769	-0.41	-2.570	9.64E-05
A_44_P313815	Tesk2	NM_133396	170908	-0.41	-2.570	0.001113
A_44_P496447	Ntf3	NM_031073	81737	-0.41	-2.570	0.001026
A_43_P19602	Slc35f5_predicted	XM_222576		-0.41	-2.570	0.000688
A_44_P545677	Pou3f2	XM_345510		-0.41	-2.571	0.000168
A_44_P279650	Ivl	NM_022195	60583	-0.41	-2.572	6.83E-05
A_44_P278659	AJ517196	AJ517196	83731	-0.41	-2.573	0.000732
A_44_P262602	A_44_P262602	A_44_P262602		-0.41	-2.574	0.00027
A_44_P735575	AI009820	AI009820	288993	-0.41	-2.574	0.005063
A_44_P131001	XM_341721	XM_341721		-0.41	-2.574	0.018971
A_44_P250988	RGD1563386_predicted	XM_001077714		-0.41	-2.574	0.000071
A_44_P361465	RGD1564322_predicted	XM_576327	500917	-0.41	-2.574	0.002403
A_44_P448381	AA819079	AA819079		-0.41	-2.576	0.000131
A_44_P246105	Car9_predicted	XM_233380		-0.41	-2.576	0.000519
A_44_P156136	AA817829	AA817829	362045	-0.41	-2.577	0.010254
A_44_P794163	TC548816	TC548816		-0.41	-2.579	0.000465
A_44_P155781	Vamp4_predicted	XM_344168		-0.41	-2.579	0.000367

A_44_P465303	AA964361	AA964361		-0.41	-2.580	0.000232
A_44_P380228	RGD1307122	NM_001014079	314321	-0.41	-2.580	0.005262
A_44_P1013248	LOC680434	XM_001057161		-0.41	-2.581	1.29E-05
A_44_P1025386	BF564217	BF564217	305843	-0.41	-2.583	0.000131
A_44_P382138	ENSRNOT00000035017	ENSRNOT00000035017		-0.41	-2.584	7.25E-05
A_44_P900249	TC525120	TC525120		-0.41	-2.584	0.006619
A_44_P660244	Cald1	NM_013146	25687	-0.41	-2.585	0.018693
A_44_P241045	XM_344809	XM_344809		-0.41	-2.586	0.004168
A_44_P262686	Kcna10_predicted	XM_227577	295360	-0.41	-2.586	0.000304
A_44_P439142	AI104683	AI104683		-0.41	-2.587	0.001204
A_44_P730489	TC523356	TC523356		-0.41	-2.588	0.000209
A_44_P243439	XM_344307	XM_344307		-0.41	-2.588	0.000866
A_44_P130829	XM_224226	XM_224226		-0.41	-2.589	0.005628
A_44_P330890	Nodal_predicted	XM_228285		-0.41	-2.590	0.000142
A_44_P855630	TC532845	TC532845		-0.41	-2.590	0.000332
A_43_P12950	Kcnj9	NM_053834		-0.41	-2.590	0.00252
A_44_P299897	A_44_P299897	A_44_P299897		-0.41	-2.591	0.064566
A_44_P179787	Panx2	NM_199409	362979	-0.41	-2.593	0.000161
A_43_P16064	Olr1696	NM_206850	294158	-0.41	-2.593	0.009523
A_44_P520273	A_44_P520273	A_44_P520273		-0.41	-2.594	0.003429
A_44_P841532	TC545363	TC545363		-0.41	-2.594	0.006493
A_42_P486203	Agtr1a	NM_030985	24180	-0.41	-2.595	0.000506
A_44_P145684	Gnrhr	NM_031038	81668	-0.41	-2.595	0.003543
A_43_P16095	Olr1280	NM_001000517	315571	-0.41	-2.596	0.007274
A_44_P273195	AI228042	AI228042	298103	-0.41	-2.597	0.020418
A_44_P316819	RGD1306763_predicted	XM_235468	315112	-0.41	-2.598	0.002506
A_44_P518076	XM_223996	XM_223996		-0.41	-2.598	0.002553
A_44_P940589	AA998488	AA998488		-0.41	-2.598	0.021399
A_44_P528997	RGD1564616_predicted	XM_573089		-0.41	-2.598	0.000121
A_44_P824386	TC525865	TC525865		-0.41	-2.599	0.004481
A_44_P871833	TC558621	TC558621		-0.41	-2.599	0.00026
A_44_P330778	RGD1564786_predicted	XM_225265	306889	-0.41	-2.600	0.037901
A_43_P17660	LOC289786	XM_214092	289786	-0.42	-2.600	0.001317
A_44_P558379	BG671006	BG671006		-0.42	-2.601	0.000389
A_44_P934325	TC566260	TC566260		-0.42	-2.602	0.005291
A_44_P477871	Slc6a4	NM_013034	25553	-0.42	-2.602	0.001283
A_44_P902033	TC551203	TC551203		-0.42	-2.603	0.007921
A_44_P122884	BI295882	BI295882	681059	-0.42	-2.603	0.017625
A_44_P194458	XM_341387	XM_341387		-0.42	-2.604	0.002585
A_44_P338051	A_44_P338051	A_44_P338051		-0.42	-2.605	0.009673
A_44_P204048	A_44_P204048	A_44_P204048		-0.42	-2.605	0.002886
A_43_P11520	Thrsp	NM_012703	25357	-0.42	-2.605	0.000445
A_44_P1037609	RGD1560523_predicted	XM_573428	171347	-0.42	-2.605	8.92E-05
A_44_P298886	CB328278	CB328278		-0.42	-2.607	0.00481
A_44_P428232	AW914778	AW914778		-0.42	-2.607	0.003369
A_44_P112260	Lcn8_predicted	XM_345329		-0.42	-2.608	0.001822
A_44_P103326	Tex14_predicted	XM_220825	287603	-0.42	-2.608	0.017421
A_44_P267142	RGD1563053_predicted	XM_341725		-0.42	-2.608	0.011712
A_44_P147496	XM_346135	XM_346135		-0.42	-2.608	0.078225
A_44_P137497	AY325224	AY325224		-0.42	-2.609	0.008141
A_44_P978840	DV720702	DV720702		-0.42	-2.609	0.003582
A_44_P304667	Slc35b3_predicted	XM_225253	306866	-0.42	-2.611	0.00042
A_44_P775185	RGD1562080_predicted	XM_574108	498827	-0.42	-2.611	0.000926
A_43_P12176	Accn4	NM_022234	63882	-0.42	-2.613	0.000154
A_43_P20704	Dst_predicted	XM_237042		-0.42	-2.613	0.000301
A_44_P883687	ENSRNOT00000046494	ENSRNOT00000046494		-0.42	-2.613	0.014783
A_44_P292711	Slc5a11	XM_574554	252854	-0.42	-2.614	0.000913
A_44_P252644	Efna3	XM_574979	170901	-0.42	-2.615	0.003792
A_44_P206008	A_44_P206008	A_44_P206008		-0.42	-2.615	0.019466
A_44_P185264	RGD1560013_predicted	XM_001063907		-0.42	-2.616	0.001957
A_44_P917653	LOC500377	NM_001024339	500377	-0.42	-2.617	0.00031
A_44_P498648	Olr1361	NM_173333	287000	-0.42	-2.618	0.001484
A_43_P17408	CB547962	CB547962	313278	-0.42	-2.619	0.000743

A_44_P691255	CO396119	CO396119		-0.42	-2.619	0.000941
A_44_P454920	RGD1563108_predicted	XM_345089		-0.42	-2.620	0.002218
A_43_P13054	Hadhsc	NM_057186	113965	-0.42	-2.622	0.000187
A_43_P11904	Edg1	NM_017301	29733	-0.42	-2.623	0.000398
A_44_P279986	BF418734	BF418734	298443	-0.42	-2.624	0.003543
A_44_P651719	ENSRNOT00000030490	ENSRNOT00000030490		-0.42	-2.625	0.015298
A_44_P520045	Olr1002_predicted	NM_001001371	288881	-0.42	-2.626	0.002152
A_42_P636761	Gzmm	XM_001076783		-0.42	-2.628	0.00026
A_44_P404180	BI285576	BI285576	296654	-0.42	-2.629	0.006091
A_44_P550845	RGD1306349_predicted	XM_001080558		-0.42	-2.629	0.030657
A_44_P483274	A_44_P483274	A_44_P483274		-0.42	-2.629	0.000308
A_44_P335830	BM385325	BM385325		-0.42	-2.629	0.020246
A_44_P446578	Ugt1a1	NM_012683	113992	-0.42	-2.630	0.005413
A_44_P670369	TC545643	TC545643		-0.42	-2.630	0.000735
A_44_P1029714	Cma1	NM_013092	25627	-0.42	-2.632	0.003961
A_42_P655689	Cdx2	NM_023963	66019	-0.42	-2.633	0.001721
A_42_P818323	Gfra3	NM_053398	84422	-0.42	-2.635	0.007582
A_44_P630833	A_44_P630833	A_44_P630833		-0.42	-2.635	0.001084
A_44_P746384	TC523460	TC523460		-0.42	-2.636	0.000119
A_44_P258084	XM_225798	XM_225798		-0.42	-2.636	0.009671
A_44_P554965	Mamdc4	NM_145768	252882	-0.42	-2.637	0.004722
A_44_P534689	A_44_P534689	A_44_P534689		-0.42	-2.637	0.011105
A_43_P22944	Tepp	NM_201655	291850	-0.42	-2.638	0.014335
A_44_P298934	BE116552	BE116552	299858	-0.42	-2.638	0.00062
A_44_P332896	Kctd14_predicted	XM_218935	308836	-0.42	-2.639	0.001363
A_44_P184057	BE102354	BE102354	290447	-0.42	-2.639	0.022931
A_43_P12232	Acads	NM_022512	64304	-0.42	-2.639	0.000403
A_44_P457235	Scube3_predicted	XM_228030	294297	-0.42	-2.642	0.000922
A_44_P136841	AI014169	AI014169	117514	-0.42	-2.644	0.000864
A_44_P301717	Olr584_predicted	NM_001000661	404861	-0.42	-2.644	0.023052
A_44_P480979	Dnahc11	XM_213534		-0.42	-2.644	0.00124
A_44_P732648	TC562707	TC562707		-0.42	-2.644	0.00647
A_44_P278549	Scn10a	NM_017247	29571	-0.42	-2.646	0.004071
A_44_P541094	A_44_P541094	A_44_P541094		-0.42	-2.647	0.003047
A_42_P537501	Dpp4	NM_012789	25253	-0.42	-2.647	0.045131
A_44_P321043	RGD1561759_predicted	XM_217910		-0.42	-2.647	0.021017
A_44_P869404	TC523910	TC523910		-0.42	-2.648	0.000864
A_44_P782256	TC568410	TC568410		-0.42	-2.649	0.000554
A_44_P391043	MGC114246	NM_001017509	498688	-0.42	-2.649	0.010254
A_44_P208081	Olr1092_predicted	NM_001001380	299570	-0.42	-2.649	0.008434
A_44_P380788	AW252746	AW252746	679990	-0.42	-2.649	0.010822
A_44_P1027758	AW915041	AW915041		-0.42	-2.649	0.000189
A_44_P465798	BF397977	BF397977	290361	-0.42	-2.650	0.001604
A_44_P236087	Qpc	NM_001025134	497902	-0.42	-2.650	0.004046
A_44_P163918	A_44_P163918	A_44_P163918		-0.42	-2.657	0.007477
A_44_P592790	Pank1_predicted	XM_215283		-0.42	-2.658	0.000377
A_44_P405581	Upf3b_predicted	XM_233312	313449	-0.42	-2.658	0.002295
A_44_P212602	Olr232_predicted	NM_001001035	405921	-0.42	-2.658	0.059064
A_44_P170496	Sqrdl	XM_342606		-0.42	-2.659	0.002798
A_44_P183948	M27315	M27315		-0.42	-2.660	0.000555
A_43_P10334	Scn4b	NM_001008880	315611	-0.42	-2.660	0.001049
A_43_P10572	BU758451	BU758451	365355	-0.43	-2.663	0.018849
A_43_P10331	CB605803	CB605803		-0.43	-2.664	0.001419
A_43_P17922	Myrip	NM_182844	360034	-0.43	-2.666	4.16E-05
A_44_P290822	BE098382	BE098382		-0.43	-2.669	0.002003
A_44_P1008753	Fhl1	NM_145669	25177	-0.43	-2.670	0.000235
A_44_P607877	TC541863	TC541863		-0.43	-2.670	0.001429
A_44_P311323	Cyp2b2	XM_341808	361523	-0.43	-2.671	0.0043
A_43_P16128	Lhx8_predicted	NM_001012219	365963	-0.43	-2.671	0.000191
A_42_P483557	XM_227391	XM_227391		-0.43	-2.671	9.48E-05
A_42_P459574	MGC105647	NM_001007008	361824	-0.43	-2.671	0.000074
A_44_P197018	XM_219724	XM_219724		-0.43	-2.672	0.000324
A_42_P623839	RGD1305797_predicted	XM_001060174		-0.43	-2.674	7.52E-05

A_44_P794416	TC544695	TC544695		-0.43	-2.674	0.000895
A_44_P119019	Cck	NM_012829	25298	-0.43	-2.675	9.24E-06
A_44_P468224	Olr1394_predicted	NM_001001091	405985	-0.43	-2.675	0.015648
A_44_P729942	TC538764	TC538764		-0.43	-2.676	3.87E-05
A_44_P562652	AA964077	AA964077		-0.43	-2.676	0.02711
A_43_P19057	Carkl	NM_001033682	287479	-0.43	-2.677	0.002294
A_43_P20995	Rai14	NM_001011947	294804	-0.43	-2.678	0.000402
A_44_P858898	BG372823	BG372823		-0.43	-2.678	0.00081
A_44_P379619	RGD1560260_predicted	XM_574715	365526	-0.43	-2.678	0.003139
A_44_P184564	Uncx4.1	NM_017179	29375	-0.43	-2.681	0.000589
A_44_P222004	Il15	NM_013129	25670	-0.43	-2.682	0.001275
A_43_P12903	Ccl28	NM_053700	114492	-0.43	-2.682	0.000377
A_44_P886593	TC548855	TC548855		-0.43	-2.683	0.027074
A_44_P412933	D1bwg1363e	NM_001005906	316533	-0.43	-2.684	0.000304
A_44_P241055	XM_344830	XM_344830		-0.43	-2.686	0.016398
A_44_P915194	TC557698	TC557698		-0.43	-2.687	0.000226
A_44_P208590	XM_345377	XM_345377		-0.43	-2.687	0.002166
A_44_P178278	LOC301839	XM_001061753		-0.43	-2.689	0.000408
A_44_P482721	RGD1561496_predicted	XM_222721	289085	-0.43	-2.691	9.24E-06
A_44_P192393	LOC301118	XM_236790		-0.43	-2.695	0.004847
A_44_P302172	A_44_P302172	A_44_P302172		-0.43	-2.699	0.000351
A_44_P168684	XM_345095	XM_345095		-0.43	-2.699	0.000293
A_44_P324654	AABR03005186	AABR03005186		-0.43	-2.699	0.002518
A_44_P168959	A_44_P168959	A_44_P168959		-0.43	-2.701	0.00147
A_44_P375561	Bsn	NM_019146	29138	-0.43	-2.703	0.001092
A_44_P962865	TC550274	TC550274		-0.43	-2.704	0.025056
A_44_P697233	AW141305	AW141305		-0.43	-2.704	0.020579
A_44_P261538	RGD1359529	NM_001014193	362626	-0.43	-2.704	0.000214
A_42_P718858	Rtbnd_predicted	XM_222476		-0.43	-2.704	0.001257
A_44_P136010	Sema5b_predicted	XM_239438		-0.43	-2.704	0.00027
A_44_P112527	RGD1563717_predicted	XM_001076145		-0.43	-2.706	0.031731
A_44_P886240	TC562597	TC562597		-0.43	-2.708	0.000456
A_44_P274061	RT1-CE3	NM_001008841	24737	-0.43	-2.710	2.65E-05
A_44_P560363	LOC502825	XR_006950	502825	-0.43	-2.712	0.00355
A_44_P101652	AI228830	AI228830	83792	-0.43	-2.712	0.000106
A_43_P12385	Zfp354c	NM_023988	78972	-0.43	-2.714	0.052067
A_43_P14965	AC096777	AC096777		-0.43	-2.715	0.000351
A_44_P372930	Flrt3_predicted	XM_345441	366205	-0.43	-2.715	0.012412
A_44_P913396	RGD1562370_predicted	XM_573202		-0.43	-2.717	0.025833
A_44_P355782	BE099063	BE099063	246143	-0.43	-2.717	0.00039
A_44_P175654	A_44_P175654	A_44_P175654		-0.43	-2.717	7.07E-05
A_44_P314568	AI176079	AI176079	81738	-0.43	-2.717	0.04102
A_44_P473286	ENSRNOT00000041446	ENSRNOT00000041446		-0.43	-2.718	0.012693
A_44_P759216	AI009709	AI009709		-0.43	-2.718	0.000801
A_44_P177146	AI599699	AI599699	252855	-0.43	-2.719	0.06549
A_44_P170836	Ern2_predicted	XM_344959		-0.43	-2.719	0.000454
A_44_P290350	Gpr44	NM_001012070	309212	-0.43	-2.720	0.035645
A_44_P351546	Fth1	NM_012848	25319	-0.43	-2.721	0.001019
A_44_P138030	Lyl1	NM_001007677	304663	-0.43	-2.721	0.000816
A_44_P374212	BF285164	BF285164	360718	-0.43	-2.721	0.002764
A_44_P313723	XM_575540	XM_575540		-0.43	-2.722	0.001902
A_44_P457272	Irs4_predicted	XM_235721	315350	-0.44	-2.725	0.000944
A_44_P490212	XM_217064	XM_217064		-0.44	-2.725	0.001
A_44_P740369	CR754347	CR754347		-0.44	-2.725	0.000675
A_42_P503281	Clic5	NM_053603	94272	-0.44	-2.725	0.000192
A_43_P11570	Abcd3	NM_012804	25270	-0.44	-2.727	3.34E-05
A_44_P501035	Olr1096_predicted	NM_001000485	302027	-0.44	-2.727	0.011146
A_44_P647673	AI555463	AI555463		-0.44	-2.727	0.012827
A_44_P324707	RT1-Aw2	NM_012645	24737	-0.44	-2.728	0.000539
A_43_P15261	Nr3c2	NM_013131	25672	-0.44	-2.728	5.36E-05
A_44_P357529	BG379780	BG379780	293829	-0.44	-2.729	0.003963
A_44_P407954	XM_228823	XM_228823		-0.44	-2.729	0.001883
A_44_P575132	A_44_P575132	A_44_P575132		-0.44	-2.729	0.00481

A_44_P375021	LOC680273	XM_001056413		-0.44	-2.730	0.000259
A_44_P889581	TC551296	TC551296		-0.44	-2.730	0.000224
A_44_P365142	BI290548	BI290548	303747	-0.44	-2.731	0.015069
A_43_P13051	St8sia2	NM_057156	117523	-0.44	-2.734	8.64E-05
A_44_P869212	LOC288979	XM_222564		-0.44	-2.735	0.000202
A_44_P418563	BQ194244	BQ194244	298006	-0.44	-2.735	0.009146
A_44_P362915	Il13ra1	NM_145789	252963	-0.44	-2.735	0.000329
A_44_P466033	Arrdc4	XM_218783		-0.44	-2.735	0.000146
A_44_P212552	Tob1	NM_133317	170842	-0.44	-2.737	0.000444
A_44_P884971	TC524290	TC524290		-0.44	-2.737	0.000618
A_44_P375453	RGD1559932_predicted	XM_233161		-0.44	-2.738	0.007448
A_44_P503471	Itga7	NM_030842	81008	-0.44	-2.743	0.010724
A_44_P504289	XM_220964	XM_220964		-0.44	-2.743	0.0087
A_44_P236182	LOC499544	XM_574867		-0.44	-2.744	0.059351
A_44_P513193	Gamt	NM_012793	25257	-0.44	-2.745	0.005213
A_44_P217726	XM_218995	XM_218995		-0.44	-2.745	0.003314
A_44_P512874	Cth	NM_017074	24962	-0.44	-2.748	0.000407
A_44_P621708	RGD1562562_predicted	XM_576330	500921	-0.44	-2.748	0.00025
A_44_P506299	RGD1562344_predicted	XM_215660		-0.44	-2.748	2.55E-05
A_44_P511562	AI231033	AI231033	85238	-0.44	-2.750	0.00037
A_44_P193446	Cd38	NM_013127	25668	-0.44	-2.750	0.001466
A_44_P382152	XM_345161	XM_345161		-0.44	-2.752	0.011216
A_44_P344205	Olr868_predicted	NM_001000821	405110	-0.44	-2.752	0.007391
A_44_P421985	XM_215767	XM_215767		-0.44	-2.753	0.04953
A_44_P738643	A_44_P738643	A_44_P738643		-0.44	-2.755	7.25E-05
A_44_P138247	A_44_P138247	A_44_P138247		-0.44	-2.756	0.005769
A_43_P15854	LOC310395	XM_227134	171555	-0.44	-2.758	0.000011
A_44_P623782	TC560770	TC560770		-0.44	-2.759	0.002578
A_43_P14838	Cart1	NM_012921	25401	-0.44	-2.760	0.000293
A_44_P1025613	Pon3	NM_001004086	312086	-0.44	-2.760	0.004049
A_44_P653647	RGD1562562_predicted	XM_576330	500921	-0.44	-2.761	3.77E-05
A_44_P367433	BC086414	BC086414		-0.44	-2.763	0.003704
A_44_P537854	AI013847	AI013847	363082	-0.44	-2.763	0.001125
A_44_P155135	BF414152	BF414152	81637	-0.44	-2.765	0.000518
A_44_P1071248	D90049	D90049		-0.44	-2.767	0.004076
A_44_P233569	Gnb5	NM_031770	83579	-0.44	-2.770	0.001052
A_44_P196050	BM384214	BM384214	297894	-0.44	-2.770	0.000587
A_44_P180772	RGD1563979_predicted	XM_236920	316212	-0.44	-2.770	0.000223
A_43_P11848	Bmp2	NM_017178	29373	-0.44	-2.771	0.002961
A_44_P489661	XM_213731	XM_213731		-0.44	-2.771	0.000375
A_44_P475772	XM_227406	XM_227406		-0.44	-2.773	5.07E-05
A_44_P234531	RGD1308613	NM_001014086		-0.44	-2.773	0.000187
A_44_P987572	AA943160	AA943160	654441	-0.44	-2.777	0.045892
A_44_P772513	CK844318	CK844318	500568	-0.44	-2.777	0.003688
A_43_P18155	Zic2_predicted	XM_341381		-0.44	-2.778	0.021257
A_44_P463065	BF290512	BF290512		-0.44	-2.778	0.00042
A_44_P506282	A_44_P506282	A_44_P506282		-0.44	-2.779	0.000329
A_44_P511552	BI294871	BI294871		-0.44	-2.779	0.010364
A_43_P11437	Asgr1	NM_012503	24210	-0.44	-2.779	0.000388
A_44_P301560	BF284944	BF284944	297998	-0.44	-2.780	0.015265
A_44_P928636	ENSRNOT00000039453	ENSRNOT00000039453		-0.44	-2.781	0.002099
A_44_P464458	ENSRNOT00000043218	ENSRNOT00000043218		-0.44	-2.781	0.052941
A_44_P385319	AA925708	AA925708	360872	-0.44	-2.782	0.006511
A_44_P156642	RGD1304717_predicted	XM_218339		-0.44	-2.784	0.004248
A_44_P311324	Cyp2b2	XM_341808	361523	-0.44	-2.784	0.002474
A_44_P1016730	AI169598	AI169598		-0.44	-2.784	0.00388
A_44_P494723	XM_343716	XM_343716		-0.44	-2.785	0.053153
A_44_P402507	Retsat	NM_145084	246298	-0.44	-2.785	0.000221
A_44_P744687	BC087158	BC087158	1E+08	-0.45	-2.786	0.03391
A_44_P515461	Bpnt1	NM_171990	64473	-0.45	-2.786	2.96E-05
A_44_P384735	XM_222123	XM_222123		-0.45	-2.786	0.026651
A_44_P547791	Gpr119	NM_181770	302813	-0.45	-2.787	0.000458
A_44_P342456	AW921958	AW921958		-0.45	-2.788	0.001764

A_44_P497290	Map3k14_predicted	XM_340918		-0.45	-2.789	0.003911
A_44_P438701	LOC288979	XM_222564		-0.45	-2.790	0.005093
A_44_P828123	CO393245	CO393245		-0.45	-2.790	0.001668
A_44_P865962	Mapre3	NM_001007656	298848	-0.45	-2.792	4.96E-05
A_44_P324981	BX883042	BX883042		-0.45	-2.796	0.042879
A_44_P105034	Alox5	NM_012822	25290	-0.45	-2.799	0.000653
A_44_P171008	LOC678918	XM_001053805		-0.45	-2.799	4.81E-05
A_44_P760485	A_44_P760485	A_44_P760485		-0.45	-2.800	0.001258
A_44_P244516	BI283060	BI283060	293860	-0.45	-2.801	0.00239
A_43_P12344	Nr1i3	NM_022941	65035	-0.45	-2.801	0.000698
A_44_P763837	TC533434	TC533434		-0.45	-2.801	0.0005
A_44_P678317	TC530121	TC530121		-0.45	-2.801	0.000278
A_44_P114604	A_44_P114604	A_44_P114604		-0.45	-2.802	0.002529
A_44_P384389	LOC691211	XM_001077221		-0.45	-2.802	0.000101
A_43_P18385	LOC691966	XM_001080417	691966	-0.45	-2.807	0.002211
A_44_P541615	XM_235484	XM_235484		-0.45	-2.807	0.000458
A_44_P111231	BM385414	BM385414	361501	-0.45	-2.807	0.002902
A_44_P264947	RGD1564670_predicted	XM_220043	309510	-0.45	-2.808	0.004355
A_44_P102643	Slc12a5	NM_134363	171373	-0.45	-2.812	0.033866
A_43_P16609	MGC94941	NM_001004233	296988	-0.45	-2.813	1.52E-05
A_44_P397481	AI234003	AI234003		-0.45	-2.814	0.001401
A_44_P422218	A_44_P422218	A_44_P422218		-0.45	-2.814	0.018935
A_44_P308824	Olr1593_predicted	NM_001000085	289255	-0.45	-2.817	0.004503
A_44_P632193	RGD1566112_predicted	XM_576067	500685	-0.45	-2.818	0.000249
A_44_P1002360	Cryl1	NM_175757	290277	-0.45	-2.818	0.000175
A_43_P13017	B3gat1	NM_054003	117108	-0.45	-2.818	0.006166
A_43_P11901	Kcnj5	NM_017297	29713	-0.45	-2.820	0.003041
A_44_P245453	Olr1436_predicted	NM_001000014	287324	-0.45	-2.820	0.000381
A_44_P100139	Olr4_predicted	NM_001000111	292560	-0.45	-2.822	0.02583
A_44_P538640	Azgp1	NM_012826	25294	-0.45	-2.824	0.00053
A_44_P761708	Slc27a4	XM_231115	311839	-0.45	-2.824	3.91E-05
A_44_P468905	XM_229376	XM_229376		-0.45	-2.826	0.006743
A_44_P289637	Slc4a1	NM_012651	24779	-0.45	-2.828	0.002869
A_44_P1052006	Prss21	NM_181477	353251	-0.45	-2.830	0.007252
A_44_P976960	RGD1559807_predicted	XM_001066511		-0.45	-2.830	8.81E-05
A_43_P15933	Chrm4	XM_345403		-0.45	-2.830	0.014047
A_43_P21816	Myom1	XM_237523	316740	-0.45	-2.830	0.023113
A_44_P249694	AI170621	AI170621	307524	-0.45	-2.831	0.005412
A_44_P141840	BG670847	BG670847		-0.45	-2.831	8.95E-05
A_44_P168761	XM_229333	XM_229333		-0.45	-2.832	0.021319
A_44_P122308	AI177116	AI177116	361517	-0.45	-2.832	0.002299
A_44_P183016	XM_343695	XM_343695		-0.45	-2.833	0.007848
A_44_P187449	XM_344595	XM_344595		-0.45	-2.835	0.007982
A_44_P224924	RGD1563451_predicted	XM_227350		-0.45	-2.835	0.011039
A_44_P609350	TC532812	TC532812		-0.45	-2.836	0.031552
A_44_P137699	Mpst	NM_138843	192172	-0.45	-2.836	0.000399
A_44_P141897	Abcb1	NM_012623	24646	-0.45	-2.837	0.000938
A_44_P559427	AA956740	AA956740	64476	-0.45	-2.837	0.008988
A_44_P188222	P2rx7	NM_019256	29665	-0.45	-2.837	5.01E-05
A_44_P280539	Slc25a5	NM_057102	25176	-0.45	-2.837	9.88E-06
A_43_P15811	Kitl	NM_021843	60427	-0.45	-2.838	2.28E-05
A_44_P543279	Rbp3	XM_341400	24711	-0.45	-2.838	4.15E-05
A_44_P746247	TC557386	TC557386		-0.45	-2.838	0.000201
A_44_P365462	Cacna1d	NM_017298	29716	-0.45	-2.839	0.000458
A_44_P364859	CB547501	CB547501	300017	-0.45	-2.839	0.020845
A_44_P918566	TC563191	TC563191		-0.45	-2.840	0.011358
A_44_P232060	XM_229347	XM_229347		-0.45	-2.842	0.010701
A_44_P722291	AI229537	AI229537	299343	-0.45	-2.844	0.020344
A_44_P102721	Stard6	NM_001007627	291527	-0.45	-2.844	0.000249
A_44_P671931	TC528821	TC528821		-0.45	-2.844	0.000482
A_44_P393706	RGD1309701_predicted	XM_237514		-0.45	-2.847	0.000364
A_44_P432950	AA900681	AA900681	25514	-0.45	-2.850	0.000539
A_44_P148634	AA925527	AA925527	297029	-0.45	-2.850	0.000378

A_43_P13304	Tgoln2	NM_138840	192152	-0.45	-2.850	1.43E-05
A_42_P695648	Lhx5	NM_139036	124451	-0.46	-2.851	0.009131
A_43_P22663	Il17rb_predicted	XM_224604		-0.46	-2.852	0.007241
A_44_P900646	TC525909	TC525909		-0.46	-2.852	0.005501
A_44_P1038716	RGD1565166_predicted	XM_213215		-0.46	-2.853	0.001096
A_44_P203665	Ccng2_predicted	XM_223270		-0.46	-2.856	0.000143
A_44_P260804	BM390913	BM390913		-0.46	-2.856	0.007075
A_44_P531999	LOC685366	XM_001063504		-0.46	-2.857	0.001132
A_44_P130491	Tnp2	NM_017057	24840	-0.46	-2.860	0.008549
A_44_P408197	A_44_P408197	A_44_P408197		-0.46	-2.861	0.003983
A_43_P17175	Serpinb1a	NM_001031642	291091	-0.46	-2.861	0.00022
A_44_P199813	AA955436	AA955436		-0.46	-2.861	0.008325
A_44_P946595	TC526191	TC526191		-0.46	-2.862	0.001724
A_44_P356526	Acss1_predicted	XM_215897		-0.46	-2.863	0.000113
A_44_P476896	AI178206	AI178206	500226	-0.46	-2.863	0.002744
A_44_P192431	LOC688600	XM_001067558		-0.46	-2.864	0.001287
A_42_P596050	Pcdh21	NM_053572	93662	-0.46	-2.864	0.00012
A_44_P306296	BI303604	BI303604	360704	-0.46	-2.865	0.002061
A_44_P614539	CK475991	CK475991		-0.46	-2.865	0.010038
A_44_P1019480	Cdc42ep2	NM_001009689	309175	-0.46	-2.865	0.000865
A_44_P241206	XM_577449	XM_577449		-0.46	-2.866	0.000128
A_42_P768563	LOC679610	XM_001053203	679610	-0.46	-2.867	0.001521
A_44_P698846	A_44_P698846	A_44_P698846		-0.46	-2.867	0.00017
A_44_P291996	BQ190716	BQ190716	317322	-0.46	-2.867	0.005525
A_44_P145283	Krtap14_predicted	XM_344003		-0.46	-2.869	0.000227
A_44_P116261	Gapdhs	NM_023964	66020	-0.46	-2.870	0.03114
A_44_P241690	Gpr128_predicted	XM_221527		-0.46	-2.870	0.000193
A_44_P156361	Olr694_predicted	NM_001000357	295896	-0.46	-2.871	0.043179
A_42_P722989	Purg_predicted	XM_341448	361162	-0.46	-2.871	0.001592
A_44_P368780	AA899887	AA899887		-0.46	-2.873	0.000446
A_44_P166524	Lonrf2_predicted	XM_237078	301361	-0.46	-2.874	0.009775
A_44_P761949	RGD1309655	NM_001013862	287766	-0.46	-2.874	0.000074
A_44_P133449	Elf4_predicted	XM_229125	302811	-0.46	-2.875	0.018123
A_44_P356851	RGD1562534_predicted	XM_001080900		-0.46	-2.876	7.38E-05
A_44_P353003	BI301474	BI301474	306587	-0.46	-2.876	0.00488
A_44_P535411	AW251950	AW251950	305685	-0.46	-2.878	0.00095
A_44_P867355	ENSRNOT00000027998	ENSRNOT00000027998		-0.46	-2.878	0.000385
A_44_P507835	AI176590	AI176590	299982	-0.46	-2.879	0.003856
A_44_P457153	RGD1564999_predicted	XM_225498		-0.46	-2.879	0.011702
A_44_P499209	LOC679020	XM_001054275		-0.46	-2.879	0.005615
A_44_P277340	Egfl5_predicted	XM_001066182		-0.46	-2.883	0.000685
A_43_P12947	Il13	NM_053828	116553	-0.46	-2.884	0.003545
A_44_P351605	A_44_P351605	A_44_P351605		-0.46	-2.885	0.013693
A_44_P271569	Olr1376_predicted	NM_214827	287084	-0.46	-2.885	0.002512
A_44_P426578	A_44_P426578	A_44_P426578		-0.46	-2.886	0.001361
A_44_P292934	LOC293746	XM_219594		-0.46	-2.887	0.001816
A_44_P472674	AW143016	AW143016		-0.46	-2.887	0.005178
A_42_P755115	bsnd	NM_138979	192675	-0.46	-2.887	0.027082
A_44_P116697	Golga3_predicted	XM_231418		-0.46	-2.887	0.000114
A_44_P316822	RGD1304827	NM_001009437	316091	-0.46	-2.888	0.005838
A_44_P128573	Chst9_predicted	XM_226142	291770	-0.46	-2.888	0.036203
A_44_P645452	BF550250	BF550250		-0.46	-2.889	0.00062
A_43_P22276	XM_342995	XM_342995		-0.46	-2.890	0.000269
A_44_P311322	Cyp2b2	XM_341808	361523	-0.46	-2.891	0.00038
A_44_P710285	Stim1_predicted	XM_341896		-0.46	-2.892	1.96E-05
A_44_P366265	BQ190001	BQ190001		-0.46	-2.892	0.013656
A_44_P622560	TC539049	TC539049		-0.46	-2.894	3.92E-05
A_44_P560710	TC563170	TC563170		-0.46	-2.894	0.00017
A_44_P855180	TC528480	TC528480		-0.46	-2.895	0.010062
A_44_P456444	Nid2	XM_573694	302248	-0.46	-2.895	0.000291
A_44_P454719	RGD1562952_predicted	XM_001072232		-0.46	-2.895	2.85E-05
A_44_P435320	AA851161	AA851161	299850	-0.46	-2.896	0.005096
A_42_P571388	Gpx6	NM_147165	259233	-0.46	-2.897	0.000385

A_44_P655182	TC550712	TC550712		-0.46	-2.898	0.001349
A_44_P194967	AW253040	AW253040	288762	-0.46	-2.899	0.000313
A_43_P16600	Atcay_predicted	NM_001040190	362826	-0.46	-2.900	0.000864
A_44_P281492	RGD1564931_predicted	XM_228797		-0.46	-2.903	0.031197
A_44_P1039205	M23721	M23721		-0.46	-2.905	0.001831
A_44_P155835	AA893860	AA893860	294810	-0.46	-2.905	0.004091
A_44_P185015	Spetex-2B	NM_001011697	364261	-0.46	-2.908	0.041365
A_44_P535156	AW253668	AW253668	29583	-0.46	-2.909	0.040938
A_44_P412938	LOC687719	XM_001079896		-0.46	-2.909	0.002203
A_44_P1070856	Scnn1a	NM_031548	25122	-0.46	-2.911	7.66E-05
A_44_P730016	TC538999	TC538999		-0.46	-2.911	0.000289
A_44_P699644	LOC313672	NM_001014070	313672	-0.46	-2.913	0.000653
A_44_P155971	RGD1563325_predicted	XM_343187		-0.46	-2.915	0.003963
A_44_P329457	AI044436	AI044436	294258	-0.46	-2.915	0.001302
A_44_P428691	Elovl6	NM_134383	171402	-0.46	-2.916	0.000381
A_44_P414996	Gfpt1	NM_001005879	297417	-0.46	-2.917	5.44E-05
A_44_P832287	A_44_P832287	A_44_P832287		-0.46	-2.917	0.014051
A_44_P419137	Col1a1	M12199		-0.47	-2.919	0.017753
A_44_P342821	Cd300lf	NM_001025111	287818	-0.47	-2.919	0.000113
A_44_P503904	RGD1564347_predicted	XM_344622		-0.47	-2.919	0.007312
A_44_P613057	AI548588	AI548588		-0.47	-2.921	0.000143
A_44_P133470	CB546704	CB546704		-0.47	-2.921	0.000608
A_44_P361309	A_44_P361309	A_44_P361309		-0.47	-2.921	0.014353
A_44_P430365	BQ202091	BQ202091	502674	-0.47	-2.921	0.001248
A_44_P974524	LOC498933	NM_001025764	498933	-0.47	-2.922	0.026558
A_44_P890548	BE099973	BE099973		-0.47	-2.922	0.041252
A_44_P156714	XM_341232	XM_341232		-0.47	-2.923	0.001187
A_44_P126448	RGD1564845_predicted	XM_343831	363507	-0.47	-2.924	0.001365
A_44_P261779	LOC683733	XM_001072472		-0.47	-2.924	0.00018
A_42_P800656	Hes6	NM_001013179	316626	-0.47	-2.925	0.009107
A_44_P108680	Ppp2r2c	NM_057116	117256	-0.47	-2.926	0.000219
A_44_P474669	AA818788	AA818788	302499	-0.47	-2.927	0.00041
A_44_P267684	RGD1307051_predicted	XM_235174		-0.47	-2.930	0.004627
A_44_P373483	Slco2a1	NM_022667	24546	-0.47	-2.930	0.000233
A_42_P641082	Sil1	NM_199376	291673	-0.47	-2.932	0.002825
A_44_P713220	AI409065	AI409065	498309	-0.47	-2.933	0.001868
A_44_P189406	Spint1	NM_001004265	311331	-0.47	-2.933	5.07E-05
A_42_P619056	Gcnt2	NM_001001511	306860	-0.47	-2.933	0.00039
A_44_P243048	Olr1293_predicted	NM_001000460	300599	-0.47	-2.934	0.002257
A_44_P153996	AF145794	AF145794		-0.47	-2.936	0.010114
A_44_P319112	A_44_P319112	A_44_P319112		-0.47	-2.937	0.000436
A_44_P230126	AI044448	AI044448		-0.47	-2.939	0.000227
A_42_P460340	Mal2	NM_198786	362911	-0.47	-2.940	0.000189
A_44_P187300	XM_344202	XM_344202		-0.47	-2.940	0.012433
A_44_P208839	ENSRNOT00000010996	ENSRNOT00000010996		-0.47	-2.941	0.000359
A_43_P12321	Fabp9	NM_022854	64822	-0.47	-2.941	0.000153
A_44_P183944	AW920940	AW920940		-0.47	-2.944	0.00024
A_44_P290457	Ptprh	XM_574357	171125	-0.47	-2.944	3.13E-05
A_44_P985331	CK469557	CK469557		-0.47	-2.945	0.021023
A_44_P696040	AW916567	AW916567		-0.47	-2.946	0.000195
A_44_P422880	AI045512	AI045512	65190	-0.47	-2.948	0.021682
A_44_P609586	TC533122	TC533122		-0.47	-2.949	0.000102
A_44_P103312	Pipox	NM_001012009	303272	-0.47	-2.949	0.000173
A_44_P252891	Ppfia3	XM_001080725		-0.47	-2.950	0.008728
A_44_P338109	LOC497892	XM_573076		-0.47	-2.951	0.000476
A_43_P15934	Olr1468	NM_001000724	404977	-0.47	-2.952	0.011604
A_42_P532505	Ccl11	NM_019205	29397	-0.47	-2.952	0.002128
A_44_P594033	TC528454	TC528454		-0.47	-2.953	6.75E-06
A_44_P1012940	RGD1306072_predicted	XM_222458	304654	-0.47	-2.954	0.024419
A_44_P944882	XM_343658	XM_343658		-0.47	-2.955	0.001437
A_44_P224722	XM_217707	XM_217707		-0.47	-2.955	0.023482
A_44_P557233	Mag	NM_017190	29409	-0.47	-2.955	0.000124
A_44_P374756	Olr1540_predicted	NM_001001383	363762	-0.47	-2.955	0.023651

A_44_P447957	Akr1c12_predicted	XM_341549	364773	-0.47	-2.955	0.000268
A_44_P240881	Art3	NM_001012034	305235	-0.47	-2.957	9.32E-05
A_44_P412373	RGD1308141_predicted	XM_218574	308594	-0.47	-2.958	0.022235
A_44_P353864	LOC684840	XM_001072148		-0.47	-2.961	0.007379
A_44_P837538	M23953	M23953		-0.47	-2.961	0.000261
A_44_P299855	AI029290	AI029290	362015	-0.47	-2.963	0.001452
A_44_P659248	AI548214	AI548214		-0.47	-2.963	0.022003
A_44_P268069	BE095703	BE095703	690442	-0.47	-2.964	0.007783
A_44_P287366	AI171778	AI171778	360488	-0.47	-2.967	0.008495
A_44_P406690	Cmpk	NM_001025655	298410	-0.47	-2.967	0.000196
A_44_P406169	Galnt7	NM_022926	29750	-0.47	-2.967	0.000143
A_44_P957865	RGD1563346_predicted	XM_577480		-0.47	-2.967	0.007586
A_44_P549083	BQ210233	BQ210233	60562	-0.47	-2.972	0.011933
A_44_P778258	TC527116	TC527116		-0.47	-2.972	0.009044
A_44_P382593	Scarf1_predicted	XM_220712		-0.47	-2.973	0.000397
A_44_P114384	Cabp4_predicted	XM_344981		-0.47	-2.973	0.000587
A_44_P386519	LOC683341	XM_001065519		-0.47	-2.977	0.006802
A_44_P295739	BE116973	BE116973	309156	-0.47	-2.977	0.007815
A_44_P226597	Pycard	NM_172322	282817	-0.47	-2.978	7.32E-05
A_44_P494734	A_44_P494734	A_44_P494734		-0.47	-2.980	0.001336
A_44_P333302	Gpr113_predicted	XM_233935		-0.47	-2.980	0.001899
A_42_P754122	Hoxa4	L03557	79241	-0.47	-2.980	0.000467
A_44_P236943	AA925922	AA925922		-0.47	-2.981	0.006095
A_44_P133799	LOC678925	XM_001054722		-0.47	-2.985	0.003713
A_43_P18681	Col14a1_predicted	XM_235308	314981	-0.48	-2.987	0.001513
A_44_P180731	Zfpm2_predicted	XM_235253	314930	-0.48	-2.988	0.00032
A_42_P727191	Csrp2	NM_177425	29317	-0.48	-2.988	0.004712
A_44_P546244	XM_236868	XM_236868		-0.48	-2.988	0.034853
A_43_P14813	TC536615	TC536615		-0.48	-2.989	0.000527
A_44_P131372	BU759576	BU759576	315664	-0.48	-2.990	0.006451
A_44_P284145	RGD1311406_predicted	XM_346015		-0.48	-2.991	9.49E-05
A_42_P694208	RGD1311126_predicted	XM_216483	298366	-0.48	-2.992	2.84E-05
A_44_P147336	LOC691045	XM_229338		-0.48	-2.997	0.022417
A_44_P610403	TC546195	TC546195		-0.48	-3.000	0.000192
A_44_P269567	RGD1311575_predicted	XM_214022	289568	-0.48	-3.002	0.003402
A_44_P237621	Txnip	NM_001008767	117514	-0.48	-3.003	0.000179
A_44_P299598	XM_342715	XM_342715		-0.48	-3.004	0.012541
A_42_P492542	Jak3	NM_012855	25326	-0.48	-3.004	0.001966
A_44_P329154	BG374002	BG374002		-0.48	-3.004	0.002742
A_44_P105351	XM_341604	XM_341604		-0.48	-3.007	0.000508
A_44_P141872	BE100006	BE100006		-0.48	-3.009	0.016226
A_43_P17984	RGD1308373	NM_001014232	364208	-0.48	-3.009	0.002249
A_44_P131870	AI233614	AI233614	361600	-0.48	-3.010	0.009131
A_44_P189629	RGD1560561_predicted	XM_577202		-0.48	-3.010	0.001099
A_44_P541262	LOC687768	XM_001080056		-0.48	-3.010	0.000607
A_44_P579741	A_44_P579741	A_44_P579741		-0.48	-3.011	0.000301
A_44_P466776	RGD1562754_predicted	XM_345837	366925	-0.48	-3.011	0.016204
A_44_P119453	LOC683719	XM_001067187		-0.48	-3.013	0.000417
A_44_P141767	Tnrc9_predicted	XM_238039		-0.48	-3.013	0.000136
A_44_P460845	XM_345603	XM_345603		-0.48	-3.013	0.000225
A_44_P220403	LOC259245	NM_147213	259245	-0.48	-3.016	0.003734
A_44_P543978	LOC501015	XM_001056441		-0.48	-3.016	0.000267
A_44_P267570	A_44_P267570	A_44_P267570		-0.48	-3.018	0.009795
A_44_P114557	LOC682470	XM_001061637		-0.48	-3.019	0.011618
A_44_P689228	TC550975	TC550975		-0.48	-3.019	0.000358
A_44_P986018	BF420742	BF420742		-0.48	-3.020	0.00042
A_44_P553098	LOC361187	XM_341469		-0.48	-3.020	0.007285
A_44_P522278	AA799471	AA799471		-0.48	-3.021	0.000986
A_42_P840867	RGD1561455_predicted	XR_008037	364885	-0.48	-3.021	5.22E-05
A_44_P899971	TC540895	TC540895		-0.48	-3.021	0.002694
A_44_P187616	A_44_P187616	A_44_P187616		-0.48	-3.024	0.017264
A_44_P122108	AI410261	AI410261		-0.48	-3.025	0.013529
A_42_P591516	AA998612	AA998612		-0.48	-3.026	0.000818

A_44_P286848	AI228397	AI228397		-0.48	-3.027	0.000195
A_44_P453465	AI172261	AI172261	29463	-0.48	-3.027	0.003834
A_44_P441195	Acad11_predicted	XM_236582		-0.48	-3.032	0.000478
A_44_P180701	A_44_P180701	A_44_P180701		-0.48	-3.033	0.018231
A_44_P335079	Tff3	NM_013042	25563	-0.48	-3.034	0.012788
A_44_P570801	A_44_P570801	A_44_P570801		-0.48	-3.035	0.000238
A_44_P255991	AA818751	AA818751		-0.48	-3.037	0.002269
A_44_P255305	Rgs17_predicted	XM_217837		-0.48	-3.038	0.001776
A_44_P190898	AI228657	AI228657	117542	-0.48	-3.038	0.00013
A_43_P12115	Prom1	NM_021751	60357	-0.48	-3.041	0.00033
A_44_P438346	Eiih	NM_001009965	308843	-0.48	-3.042	0.000222
A_44_P443717	BQ194049	BQ194049	311349	-0.48	-3.042	0.001577
A_44_P298033	BG380347	BG380347		-0.48	-3.042	0.000989
A_44_P153345	AI059874	AI059874		-0.48	-3.044	0.022427
A_44_P885189	Lrrc4	NM_001037336	641521	-0.48	-3.044	0.000303
A_44_P682021	DV728383	DV728383	291694	-0.48	-3.045	0.000204
A_43_P13096	Hig1	NM_080902	140937	-0.48	-3.046	7.38E-05
A_44_P454804	XM_575055	XM_575055		-0.48	-3.048	0.003053
A_44_P1037342	Cyp3a13	NM_147206	171352	-0.48	-3.049	0.000317
A_44_P452410	A_44_P452410	A_44_P452410		-0.48	-3.049	0.002919
A_43_P15918	Rimbp2	XM_341071	266780	-0.48	-3.049	0.000351
A_44_P244915	AI144956	AI144956	360496	-0.48	-3.050	0.001037
A_44_P192336	Tspan9_predicted	XM_232372		-0.48	-3.051	0.050541
A_44_P900333	TC558323	TC558323		-0.48	-3.052	0.000264
A_44_P132614	Slc20a1	NM_031148	81826	-0.48	-3.054	0.000212
A_44_P690078	CB567832	CB567832		-0.49	-3.055	0.000151
A_44_P335320	A_44_P335320	A_44_P335320		-0.49	-3.055	0.012747
A_42_P761436	LOC259224	NM_153294	259224	-0.49	-3.055	0.001046
A_44_P868155	LOC681167	XM_001060573	681167	-0.49	-3.059	0.015971
A_44_P192577	A_44_P192577	A_44_P192577		-0.49	-3.059	0.002007
A_44_P536097	Mug2	NM_001002826	408236	-0.49	-3.059	0.012312
A_44_P612314	A_44_P612314	A_44_P612314		-0.49	-3.063	0.002211
A_43_P12865	Nudt4	NM_053598	94267	-0.49	-3.065	4.18E-05
A_44_P331157	A_44_P331157	A_44_P331157		-0.49	-3.065	0.02952
A_44_P860485	TC566895	TC566895		-0.49	-3.072	0.012983
A_44_P790417	LOC498572	NM_001017506	498572	-0.49	-3.074	0.000453
A_44_P684845	TC542489	TC542489		-0.49	-3.074	0.031002
A_44_P263117	Etv5_predicted	XM_221387		-0.49	-3.076	0.000619
A_44_P377396	Gna14	NM_001013151	309242	-0.49	-3.076	0.002313
A_44_P496262	Casp3	NM_012922	25402	-0.49	-3.078	4.14E-05
A_44_P260463	XM_345112	XM_345112		-0.49	-3.078	0.142388
A_44_P480424	Tigd3_predicted	XM_219522		-0.49	-3.078	0.000785
A_43_P11706	Gys2	NM_013089	25623	-0.49	-3.079	0.004145
A_44_P227243	RGD1309610_predicted	XM_342436		-0.49	-3.084	0.000187
A_43_P11082	TC539442	TC539442		-0.49	-3.086	8.33E-05
A_44_P898673	Dd5	XM_576252	117060	-0.49	-3.090	3.29E-05
A_43_P11315	Etfb	NM_001004220	292845	-0.49	-3.094	4.99E-05
A_44_P234663	LOC683626	XM_001066790		-0.49	-3.096	0.001711
A_44_P157134	Anxa13_predicted	XM_343245	362915	-0.49	-3.096	0.008879
A_44_P510518	Olr1631_predicted	NM_001000837	405126	-0.49	-3.097	0.009047
A_44_P304169	Olr1341_predicted	NM_001000482	300643	-0.49	-3.098	0.001899
A_44_P450335	RGD1307218	NM_001014190	362518	-0.49	-3.098	0.00037
A_44_P620398	DY472906	DY472906		-0.49	-3.100	0.000132
A_44_P205649	Olr306_predicted	NM_001000766	405040	-0.49	-3.101	0.006453
A_42_P805179	CF110294	CF110294		-0.49	-3.104	0.000254
A_44_P380157	LOC690298	XM_001074009		-0.49	-3.104	0.093899
A_44_P634789	AW143623	AW143623		-0.49	-3.105	8.35E-05
A_44_P511698	XM_345692	XM_345692		-0.49	-3.105	0.000937
A_44_P328062	Edn3	XM_001072605		-0.49	-3.106	0.001335
A_44_P277719	BF392952	BF392952	305854	-0.49	-3.108	0.010114
A_44_P163944	Coro2a	NM_001012101	313235	-0.49	-3.108	0.000258
A_42_P800852	RGD1565257_predicted	XM_230007		-0.49	-3.108	0.002006
A_44_P288224	Serpina12	NM_138825	191570	-0.49	-3.109	0.008792

A_42_P679814	Cyp3a18	NM_145782	252931	-0.49	-3.110	0.000071
A_44_P428230	AW914778	AW914778		-0.49	-3.116	0.000873
A_44_P172645	Dcamkl1	NM_053343	83825	-0.49	-3.117	0.002267
A_44_P852703	LOC685819	XM_001065393		-0.49	-3.118	0.010266
A_43_P12996	Crym	NM_053955	117024	-0.49	-3.119	2.52E-05
A_44_P508804	A_44_P508804	A_44_P508804		-0.49	-3.120	0.000287
A_44_P806474	RGD1564754_predicted	XM_575589		-0.49	-3.122	0.000289
A_43_P11502	Slc4a1	NM_012651	24779	-0.49	-3.125	0.000413
A_44_P363403	A_44_P363403	A_44_P363403		-0.49	-3.125	0.000183
A_44_P588914	AW920717	AW920717		-0.49	-3.125	0.000481
A_44_P257670	RGD1565233_predicted	XM_217877	292316	-0.49	-3.126	0.003364
A_44_P874109	AI013853	AI013853	124323	-0.50	-3.127	0.002048
A_44_P699928	TC556644	TC556644		-0.50	-3.128	4.42E-05
A_44_P504235	LOC301334	XM_237049	301334	-0.50	-3.130	0.002045
A_44_P209854	BM389675	BM389675		-0.50	-3.133	0.004236
A_44_P145081	RGD1562476_predicted	XM_215227		-0.50	-3.134	7.22E-06
A_44_P211286	AA866223	AA866223		-0.50	-3.135	0.009369
A_44_P164523	BE111202	BE111202	29674	-0.50	-3.136	0.000192
A_44_P961982	TC543622	TC543622		-0.50	-3.136	0.00771
A_44_P457890	Hspa12a_predicted	XM_217648		-0.50	-3.138	0.074446
A_44_P283869	A_44_P283869	A_44_P283869		-0.50	-3.139	0.002313
A_43_P10814	LOC310395	XM_227134	171555	-0.50	-3.140	0.000325
A_44_P642722	TC551395	TC551395		-0.50	-3.141	0.001993
A_44_P274083	Aqp11	NM_173105	286758	-0.50	-3.148	0.000199
A_42_P490703	Slc25a11	NM_022398	64201	-0.50	-3.148	2.05E-05
A_42_P460737	RGD1559968_predicted	XM_224759	306380	-0.50	-3.150	0.000443
A_44_P200636	RGD1565681_predicted	XM_577104	501702	-0.50	-3.150	0.001511
A_44_P283424	Pon1	NM_032077	84024	-0.50	-3.151	0.003163
A_44_P422202	XM_347076	XM_347076		-0.50	-3.152	0.000704
A_44_P480728	LOC317070	XM_229775		-0.50	-3.152	0.000106
A_42_P809869	Tst	NM_012808	25274	-0.50	-3.153	0.000361
A_44_P278653	Klk12	NM_001005382	292855	-0.50	-3.153	0.00173
A_44_P102426	Gcx1	NM_199392	311615	-0.50	-3.156	0.008066
A_44_P523323	XM_220714	XM_220714		-0.50	-3.157	0.000995
A_44_P367798	Ipmk	NM_134417	171458	-0.50	-3.159	1.31E-05
A_44_P732571	TC566135	TC566135		-0.50	-3.160	0.000122
A_44_P410461	Kb36	NM_001008808	300248	-0.50	-3.160	0.034462
A_44_P109426	Six3	NM_023990	78974	-0.50	-3.162	0.001346
A_44_P216720	BQ207749	BQ207749	304979	-0.50	-3.163	0.021499
A_44_P102465	Olr1107_predicted	NM_001000424	300202	-0.50	-3.164	0.00371
A_43_P15448	Chrna5	NM_017078	25102	-0.50	-3.164	0.003557
A_44_P417133	A_44_P417133	A_44_P417133		-0.50	-3.164	0.005356
A_42_P736390	AW143333	AW143333	361050	-0.50	-3.165	3.23E-05
A_44_P463069	M27315	M27315		-0.50	-3.169	0.000159
A_44_P111934	Scamper	AY163813		-0.50	-3.171	0.019488
A_44_P764308	TC554403	TC554403		-0.50	-3.174	0.00274
A_44_P250628	LOC502549	XM_001053637		-0.50	-3.176	0.00731
A_44_P467108	BF393108	BF393108		-0.50	-3.176	0.000358
A_44_P792087	TC555391	TC555391		-0.50	-3.177	0.001277
A_44_P141501	BE107890	BE107890	246246	-0.50	-3.177	0.008578
A_44_P417164	RGD1560232_predicted	XM_574986		-0.50	-3.177	0.004655
A_44_P168190	Scn8a	M27223	29710	-0.50	-3.179	0.003658
A_44_P545705	Chrm1	AJ006522	25229	-0.50	-3.181	0.000386
A_44_P545605	Olr462_predicted	NM_001000295	295732	-0.50	-3.182	0.002004
A_44_P318546	Nritp	XM_341519	64559	-0.50	-3.183	0.000063
A_44_P510738	Pigr	NM_012723	25046	-0.50	-3.185	0.000454
A_44_P797077	CB581857	CB581857		-0.50	-3.188	0.000129
A_43_P12487	Grm7	NM_031040	81672	-0.50	-3.190	0.000254
A_44_P417353	XM_229713	XM_229713		-0.50	-3.194	0.018851
A_44_P454927	XM_228184	XM_228184		-0.50	-3.195	0.032898
A_44_P397423	AI044267	AI044267	314751	-0.51	-3.199	0.000438
A_44_P477866	Slc6a4	NM_013034	25553	-0.51	-3.200	0.003275
A_44_P506328	XM_342423	XM_342423		-0.51	-3.201	0.001518

A_44_P974940	RGD1562392_predicted	XM_346007		-0.51	-3.204	0.000208
A_44_P394246	XM_237571	XM_237571		-0.51	-3.204	0.003748
A_44_P519112	BE099435	BE099435	310306	-0.51	-3.205	0.000113
A_44_P140465	LOC501437	XM_576846	501437	-0.51	-3.209	0.000177
A_44_P181911	AI453862	AI453862	288925	-0.51	-3.209	0.000935
A_43_P10445	Rad23a	NM_001013190	361381	-0.51	-3.210	2.04E-05
A_44_P355271	AI058940	AI058940		-0.51	-3.210	0.0003
A_44_P461105	Prlpd	NM_022537	64368	-0.51	-3.211	0.01147
A_44_P345344	AI112941	AI112941		-0.51	-3.212	0.000279
A_44_P393610	A_44_P393610	A_44_P393610		-0.51	-3.213	0.005179
A_44_P960558	TC539257	TC539257		-0.51	-3.214	0.000249
A_42_P780389	Actl7b	NM_001025417	313183	-0.51	-3.214	0.000716
A_44_P552759	AF159099	AF159099		-0.51	-3.215	0.003392
A_44_P962701	RGD1563510_predicted	XM_001063400		-0.51	-3.215	0.000153
A_42_P574859	Hamp	NM_053469	84604	-0.51	-3.216	0.000204
A_44_P397472	BQ205131	BQ205131		-0.51	-3.216	0.00081
A_44_P474483	AW533447	AW533447	303902	-0.51	-3.216	0.021121
A_44_P775640	Dppa3	XM_216263		-0.51	-3.219	0.000553
A_44_P623560	TC540430	TC540430		-0.51	-3.219	0.000207
A_43_P17677	Apc2_predicted	XM_234910		-0.51	-3.219	0.009676
A_44_P975723	CB784117	CB784117		-0.51	-3.221	0.008449
A_44_P492474	Mell1_predicted	XM_233712		-0.51	-3.224	7.23E-05
A_44_P335232	XM_214027	XM_214027		-0.51	-3.225	0.001046
A_44_P147538	Myom3_predicted	XM_233562		-0.51	-3.230	0.000651
A_44_P412397	A_44_P412397	A_44_P412397		-0.51	-3.230	0.014212
A_44_P1023801	Kif23_predicted	XM_236313		-0.51	-3.233	0.002693
A_44_P475968	XM_223867	XM_223867		-0.51	-3.237	0.003453
A_44_P370005	Spna1	NM_001011908	289257	-0.51	-3.240	0.003756
A_44_P1034720	RGD1305090_predicted	XM_342916	362598	-0.51	-3.240	0.000503
A_44_P491144	X52757	X52757		-0.51	-3.240	7.72E-05
A_42_P681441	Eps8l2_predicted	XM_341958		-0.51	-3.242	3.23E-05
A_44_P240088	AI175366	AI175366	302913	-0.51	-3.243	0.000379
A_44_P241351	XM_230578	XM_230578		-0.51	-3.243	0.000278
A_44_P150384	CA509558	CA509558		-0.51	-3.245	0.00637
A_44_P147020	LOC690021	XM_001072959		-0.51	-3.246	0.01321
A_44_P122545	AI317872	AI317872	305031	-0.51	-3.249	0.002069
A_44_P393886	RGD1310484_predicted	XM_214594		-0.51	-3.250	4.51E-05
A_43_P19135	RGD1559518_predicted	XM_244043	316229	-0.51	-3.250	0.019009
A_44_P384318	A_44_P384318	A_44_P384318		-0.51	-3.251	0.007508
A_44_P243534	RGD1311307	NM_001025719	361238	-0.51	-3.253	0.003801
A_44_P487723	LOC294557	XM_228347		-0.51	-3.255	0.000107
A_44_P306080	AI102825	AI102825		-0.51	-3.255	0.000663
A_44_P227680	CA508481	CA508481	287436	-0.51	-3.257	0.022667
A_44_P297872	St3gal1	NM_001013219	362924	-0.51	-3.258	4.81E-05
A_44_P999125	Masp2	Y18569	64459	-0.51	-3.260	0.000416
A_44_P856419	TC561708	TC561708		-0.51	-3.261	0.000181
A_44_P424819	BE117489	BE117489	294568	-0.51	-3.263	0.001065
A_44_P111489	BG671456	BG671456		-0.51	-3.266	5.33E-05
A_44_P699567	TC538681	TC538681		-0.51	-3.267	4.94E-05
A_42_P470864	Them2_predicted	XM_214475		-0.51	-3.267	2.72E-05
A_44_P381781	Bat4	NM_001034157	415064	-0.51	-3.269	0.004297
A_44_P109503	Gzmb	NM_138517	171528	-0.51	-3.269	0.001431
A_44_P463063	M27315	M27315		-0.51	-3.270	3.34E-05
A_44_P201871	LOC691911	XM_001080066	691911	-0.51	-3.273	0.00096
A_44_P445854	RGD1564722_predicted	XM_343160	362832	-0.52	-3.274	0.034479
A_44_P906452	BI293639	BI293639		-0.52	-3.278	0.001373
A_43_P11713	Fkbp1a	NM_013102	25639	-0.52	-3.279	1.73E-05
A_44_P318628	Chrnb2	NM_019297	54239	-0.52	-3.282	0.000135
A_44_P154935	BF550875	BF550875		-0.52	-3.283	0.005202
A_44_P114656	ENSRNOT00000004016	ENSRNOT00000004016		-0.52	-3.283	6.85E-05
A_44_P241057	RGD1565723_predicted	XM_577714		-0.52	-3.283	0.00453
A_43_P12364	Nmur1	NM_023100	65276	-0.52	-3.284	0.000151
A_44_P961483	TC560708	TC560708		-0.52	-3.285	0.006386

A_44_P500321	AI102064	AI102064	309312	-0.52	-3.285	0.013364
A_42_P685356	BG670434	BG670434		-0.52	-3.288	0.000386
A_44_P384910	RGD1560806_predicted	XR_007881	301376	-0.52	-3.289	2.63E-05
A_43_P17868	Gdpd2_predicted	XM_228566		-0.52	-3.293	0.000417
A_44_P834269	AW529005	AW529005		-0.52	-3.294	1.52E-05
A_44_P401019	Mapre3	NM_001007656	298848	-0.52	-3.296	2.73E-05
A_44_P356636	LOC363306	XM_001068086	363306	-0.52	-3.297	0.045259
A_44_P555862	LOC681802	XM_001058492		-0.52	-3.300	8.25E-05
A_44_P240526	AI071379	AI071379	116478	-0.52	-3.302	0.021822
A_44_P214121	AW920871	AW920871		-0.52	-3.302	7.12E-05
A_44_P637195	TC534119	TC534119		-0.52	-3.302	0.000661
A_44_P101033	XM_343999	XM_343999		-0.52	-3.303	0.013634
A_44_P189428	Zbtb16	NM_001013181	353227	-0.52	-3.305	0.000148
A_44_P711313	AI234162	AI234162	303903	-0.52	-3.308	0.002294
A_44_P794508	TC567927	TC567927		-0.52	-3.308	0.009841
A_44_P330835	XM_344710	XM_344710		-0.52	-3.310	0.01554
A_44_P848868	BI290076	BI290076		-0.52	-3.311	0.012836
A_44_P419218	AJ224441	AJ224441		-0.52	-3.311	5.95E-05
A_42_P645142	40059	NM_019375	56003	-0.52	-3.315	0.00057
A_44_P125123	AI071763	AI071763		-0.52	-3.316	0.001341
A_44_P126027	Pfkfb4	NM_019333	54283	-0.52	-3.318	1.82E-05
A_44_P477286	DV719188	DV719188		-0.52	-3.320	0.000088
A_44_P232210	AI412781	AI412781		-0.52	-3.320	0.027738
A_44_P841357	A_44_P841357	A_44_P841357		-0.52	-3.322	0.000418
A_43_P10648	TC538971	TC538971		-0.52	-3.324	0.000522
A_44_P306162	Ugt8	NM_019276	50555	-0.52	-3.327	0.000475
A_42_P476474	Fhl1	NM_001033926	25177	-0.52	-3.327	8.16E-05
A_44_P123500	Scospondin	NM_001007016	474348	-0.52	-3.328	8.74E-05
A_44_P135373	A_44_P135373	A_44_P135373		-0.52	-3.328	0.019175
A_44_P388959	Lrp3	NM_053541	89787	-0.52	-3.330	0.003998
A_44_P532585	LOC679979	XM_221297		-0.52	-3.331	0.012157
A_44_P403545	BQ191078	BQ191078	124461	-0.52	-3.334	0.010111
A_43_P10785	RGD1306952	NM_001008770	310201	-0.52	-3.339	0.000493
A_44_P182557	Manea	NM_080785	140808	-0.52	-3.339	0.000377
A_43_P10069	DV717672	DV717672		-0.52	-3.342	2.28E-05
A_44_P124294	Orc2l	NM_001012003	301430	-0.52	-3.343	0.004627
A_44_P797293	CO396111	CO396111		-0.52	-3.347	0.009559
A_44_P848100	AI058281	AI058281		-0.52	-3.348	0.008272
A_44_P536757	Osbpl6_predicted	XM_242057		-0.52	-3.349	0.026222
A_44_P438904	Cldn8	NM_001037774	304124	-0.53	-3.350	0.000394
A_44_P156850	RGD1559604_predicted	XM_574075	498790	-0.53	-3.351	4.01E-05
A_44_P368436	LOC311710	XM_230955	114588	-0.53	-3.351	0.015986
A_44_P444791	AI502079	AI502079	291733	-0.53	-3.354	0.009748
A_44_P324155	RGD1309701_predicted	XM_237514		-0.53	-3.354	0.000215
A_44_P305131	AA819208	AA819208		-0.53	-3.354	0.007644
A_44_P728387	AW915320	AW915320		-0.53	-3.354	0.007932
A_44_P222435	Apex2_mapped	XM_223499		-0.53	-3.356	5.78E-05
A_44_P152199	Zcchc12	NM_001014065	313436	-0.53	-3.359	0.001794
A_44_P251787	AW142969	AW142969		-0.53	-3.360	0.000175
A_44_P758422	AW143472	AW143472		-0.53	-3.361	0.000294
A_43_P12580	Scnn1a	NM_031548	25122	-0.53	-3.363	6.13E-06
A_44_P354070	A_44_P354070	A_44_P354070		-0.53	-3.364	0.000745
A_44_P461952	XM_233901	XM_233901		-0.53	-3.366	0.005396
A_43_P21171	Tecta_predicted	XM_236124		-0.53	-3.368	0.000911
A_44_P159271	RGD1311589_predicted	XM_231844		-0.53	-3.369	0.001831
A_43_P14506	LOC499506	XM_574831		-0.53	-3.370	0.001026
A_44_P304449	LOC308394	NM_001025679	308394	-0.53	-3.372	0.001333
A_43_P22561	Sox6	NM_001024751	293165	-0.53	-3.372	0.000503
A_44_P386187	AABR03004099	AABR03004099		-0.53	-3.373	0.001101
A_44_P515839	A_44_P515839	A_44_P515839		-0.53	-3.373	0.002155
A_44_P324254	AW527241	AW527241		-0.53	-3.374	0.000249
A_43_P11926	Acox1	NM_017340	50681	-0.53	-3.377	4.32E-06
A_44_P164201	AA955045	AA955045		-0.53	-3.377	0.000935

A_44_P654106	BM422817	BM422817		-0.53	-3.378	0.000241
A_44_P365848	XM_225618	XM_225618		-0.53	-3.378	0.017629
A_44_P298036	AA818571	AA818571		-0.53	-3.382	0.016559
A_44_P262814	XM_228282	XM_228282		-0.53	-3.384	0.002158
A_44_P228403	AA875092	AA875092	81008	-0.53	-3.385	0.000144
A_43_P10098	Gnb5	NM_031770	83579	-0.53	-3.389	0.000041
A_44_P528306	AI177505	AI177505	688972	-0.53	-3.390	0.000346
A_44_P630082	LOC683188	XM_001064839		-0.53	-3.390	0.002757
A_44_P149182	Chp	NM_024139	64152	-0.53	-3.396	1.13E-05
A_44_P433147	Gnat3	NM_173139	286924	-0.53	-3.399	0.003639
A_44_P433752	XM_344604	XM_344604		-0.53	-3.399	0.011665
A_44_P321095	LOC681856	XM_001058723		-0.53	-3.400	0.007598
A_44_P962886	TC533990	TC533990		-0.53	-3.400	0.001445
A_44_P248069	ENSRNOT00000049215	ENSRNOT00000049215		-0.53	-3.403	0.000434
A_44_P123704	A_44_P123704	A_44_P123704		-0.53	-3.404	0.006359
A_44_P334799	Mbp	NM_001025293	24547	-0.53	-3.404	0.011676
A_44_P883191	LOC685924	XM_001065777		-0.53	-3.406	0.000262
A_44_P239084	LOC367632	XM_346234		-0.53	-3.407	0.033203
A_44_P547168	Insig1	NM_022392	64194	-0.53	-3.407	8.73E-05
A_44_P157215	RGD1309644_predicted	XM_237394	316624	-0.53	-3.410	0.010833
A_44_P359548	BF542749	BF542749		-0.53	-3.412	7.97E-06
A_44_P161009	LOC362901	NM_001008877	362901	-0.53	-3.414	0.004406
A_44_P121351	Drd2	NM_012547	24318	-0.53	-3.418	0.000912
A_44_P189237	Lmo3	NM_139112	245979	-0.53	-3.423	0.008767
A_44_P335344	LOC499658	XM_574982	499658	-0.53	-3.427	0.003663
A_44_P163572	RGD1563342_predicted	XM_222254	304572	-0.53	-3.427	0.002449
A_44_P387780	Cyp8b1	NM_031241	81924	-0.54	-3.428	0.000963
A_44_P110352	LOC503164	XM_235459		-0.54	-3.428	9.55E-05
A_44_P480567	LOC684568	XM_001071021		-0.54	-3.428	0.000244
A_44_P1071143	M63970	M63970		-0.54	-3.430	0.000358
A_44_P267591	XM_233268	XM_233268		-0.54	-3.430	0.002119
A_44_P741373	BM390141	BM390141	316055	-0.54	-3.432	0.007498
A_44_P1056519	Lrfn2	XM_236914		-0.54	-3.432	0.000195
A_44_P533343	BM386267	BM386267	1E+08	-0.54	-3.433	0.000222
A_44_P292765	Pde1c	NM_031078	81742	-0.54	-3.433	0.000302
A_44_P943696	BF567763	BF567763		-0.54	-3.437	0.027946
A_44_P180431	RGD1560014_predicted	XM_001072319		-0.54	-3.439	0.022484
A_43_P13038	Slc26a2	NM_057127	117267	-0.54	-3.442	0.000068
A_44_P288043	Oxtr	NM_012871	25342	-0.54	-3.442	0.003029
A_44_P130936	A_44_P130936	A_44_P130936		-0.54	-3.443	0.000223
A_44_P777829	TC525249	TC525249		-0.54	-3.447	0.000902
A_44_P408557	AA955985	AA955985		-0.54	-3.447	0.000462
A_42_P605711	RGD1311381_predicted	XM_001063356		-0.54	-3.447	2.28E-05
A_44_P779316	TC551515	TC551515		-0.54	-3.452	0.010111
A_44_P297405	RGD1311379_predicted	XM_218982		-0.54	-3.453	0.001107
A_42_P606156	Kcnh4	NM_053630	114032	-0.54	-3.454	0.008697
A_44_P215383	LOC368128	XM_001055916		-0.54	-3.456	0.002529
A_44_P552728	Six1	XM_343079	114634	-0.54	-3.457	0.002801
A_44_P509000	Piwil4	XM_001074148		-0.54	-3.457	0.001618
A_43_P15464	Capn8	NM_133309	170808	-0.54	-3.458	8.92E-05
A_44_P121592	XM_344552	XM_344552		-0.54	-3.458	0.026943
A_42_P502621	Avil	NM_024401	79253	-0.54	-3.459	0.000219
A_44_P513698	RGD1308878_predicted	XM_233638		-0.54	-3.459	0.007591
A_43_P21277	RGD1559968_predicted	XM_001070777		-0.54	-3.463	0.001125
A_44_P475337	Olr1214_predicted	NM_001000438	300538	-0.54	-3.464	0.020636
A_44_P1056603	XM_214221	XM_214221		-0.54	-3.465	4.43E-05
A_44_P499394	XM_229294	XM_229294		-0.54	-3.469	0.019517
A_44_P270861	BI275994	BI275994	56083	-0.54	-3.471	0.009582
A_43_P21358	Nova1	XM_234098	298992	-0.54	-3.472	0.011166
A_44_P424529	Car12	XM_343416		-0.54	-3.472	0.000459
A_44_P276957	RGD1560424_predicted	XM_233601		-0.54	-3.472	0.000422
A_44_P202954	BQ201365	BQ201365	364878	-0.54	-3.475	0.002725
A_44_P142179	AF435963	AF435963		-0.54	-3.477	0.000679

A_44_P337682	Zfp488_predicted	XM_224697		-0.54	-3.482	0.000778
A_44_P960490	RGD1561967_predicted	XM_573497	498272	-0.54	-3.484	2.15E-05
A_42_P689013	Txn1	NM_053800	116484	-0.54	-3.486	0.000127
A_43_P10594	RGD1305679	NM_001014144	361228	-0.54	-3.487	0.000926
A_44_P960491	RGD1561967_predicted	XM_573497	498272	-0.54	-3.487	0.000017
A_44_P180422	RGD1563576_predicted	XM_240482		-0.54	-3.487	0.000764
A_42_P522194	Atp2a3	NM_012914	25391	-0.54	-3.490	1.54E-05
A_44_P306930	RGD1560014_predicted	XM_001072319		-0.54	-3.492	0.010021
A_43_P11754	Akr7a3	NM_013215	26760	-0.54	-3.493	3.53E-05
A_44_P135525	XM_344356	XM_344356		-0.54	-3.494	0.000421
A_44_P642946	LOC365985	XM_001080050		-0.54	-3.495	0.002575
A_44_P454937	LOC686606	XM_001074955		-0.54	-3.495	4.06E-05
A_44_P623578	TC523914	TC523914		-0.54	-3.496	5.69E-05
A_44_P410167	RGD1309038	XM_225887	307393	-0.54	-3.497	0.003592
A_44_P539148	Egfl5_predicted	XM_233047		-0.54	-3.499	0.000574
A_44_P430892	LOC298111	NM_001024248	298111	-0.54	-3.501	0.014478
A_44_P993914	Pdia5	NM_001014125	360722	-0.54	-3.503	6.45E-05
A_44_P443277	ENSRNOT00000007513	ENSRNOT00000007513		-0.54	-3.507	0.00682
A_44_P431533	LOC501315	XM_576728	501315	-0.55	-3.508	0.024053
A_44_P143896	BQ211430	BQ211430	361527	-0.55	-3.509	0.010114
A_44_P134993	Agxt	NM_030656	24792	-0.55	-3.509	0.0012
A_44_P1057763	Prdx6	NM_053576	94167	-0.55	-3.511	0.000662
A_44_P337311	Thbs4	XM_342172	29220	-0.55	-3.511	0.037587
A_44_P379416	Btnl8	NM_212489	406160	-0.55	-3.512	0.000136
A_44_P663181	AI763722	AI763722		-0.55	-3.518	0.00792
A_44_P1041365	Cipar1	NM_173114	286894	-0.55	-3.522	2.12E-05
A_44_P915992	TC560519	TC560519		-0.55	-3.524	0.000127
A_44_P994707	XM_216649	XM_216649		-0.55	-3.524	0.011482
A_44_P807363	TC555972	TC555972		-0.55	-3.528	0.024502
A_44_P470450	Mrgprb2	NM_001002285	404645	-0.55	-3.529	0.000204
A_43_P15622	Camk1g	NM_182842	171358	-0.55	-3.530	0.021883
A_44_P236847	Efnb3_predicted	XM_340824		-0.55	-3.532	0.00025
A_44_P436123	MGC108776	NM_001017466	361916	-0.55	-3.539	0.000171
A_44_P169012	AA901000	AA901000		-0.55	-3.541	0.013903
A_44_P260740	Cbr3_predicted	XM_221641		-0.55	-3.542	0.00034
A_44_P118752	Olr241_predicted	NM_001000734	404992	-0.55	-3.543	0.00902
A_44_P365760	Cdh9_predicted	XM_226861		-0.55	-3.544	0.028693
A_44_P530165	RGD1305351	NM_001013953	300663	-0.55	-3.546	0.000242
A_44_P276899	XM_346110	XM_346110		-0.55	-3.548	0.003668
A_44_P593282	TC543197	TC543197		-0.55	-3.549	0.000124
A_42_P793598	LOC378467	NM_194354	378467	-0.55	-3.553	2.12E-05
A_42_P817086	Scnn1b	NM_012648	24767	-0.55	-3.554	0.00023
A_44_P1043668	RGD1307172	NM_001014003	306540	-0.55	-3.555	0.014473
A_44_P478981	Klrg1	NM_031649	58975	-0.55	-3.555	0.000801
A_44_P1020655	Oxt	NM_012996	25504	-0.55	-3.557	4.14E-05
A_44_P250439	LOC688480	XM_001067114		-0.55	-3.567	0.037734
A_44_P517880	Fgf1	NM_012846	25317	-0.55	-3.570	0.000427
A_44_P878941	BE099838	BE099838		-0.55	-3.571	0.007921
A_44_P181910	AI599071	AI599071	288925	-0.55	-3.577	7.06E-05
A_44_P161052	RGD1562373_predicted	NM_001040019	501072	-0.55	-3.579	4.42E-05
A_42_P642557	Arrdc1	XM_345323	366001	-0.55	-3.581	7.77E-06
A_44_P105425	RGD1309236_predicted	XM_342661	362341	-0.55	-3.582	0.016719
A_43_P11553	Htr2c	NM_012765	25187	-0.55	-3.583	0.000259
A_44_P182195	Ton	NM_012677	24841	-0.55	-3.587	0.00113
A_44_P151734	Atp2a3	NM_012914	25391	-0.55	-3.587	6.27E-06
A_44_P428233	AW914778	AW914778		-0.55	-3.587	0.000484
A_44_P539444	AA997385	AA997385	303233	-0.55	-3.589	0.005306
A_44_P392886	M27315	M27315		-0.56	-3.593	5.49E-05
A_44_P269818	RGD1311940_predicted	XM_342601		-0.56	-3.599	7.53E-06
A_44_P290168	Fah	NM_017181	29383	-0.56	-3.599	0.000763
A_44_P943890	ENSRNOT00000033161	ENSRNOT00000033161		-0.56	-3.599	0.015069
A_44_P494439	Cnnm2	NM_001011942	294014	-0.56	-3.600	2.06E-05
A_42_P737278	RGD1311381_predicted	XM_001063356		-0.56	-3.602	4.8E-06

A_44_P300328	Chga	NM_021655	24258	-0.56	-3.603	0.00017
A_42_P543004	Socs2	NM_058208	84607	-0.56	-3.603	0.000222
A_44_P333899	X14435	X14435		-0.56	-3.609	0.004642
A_44_P377684	XM_346156	XM_346156		-0.56	-3.613	0.013318
A_44_P290656	Dnah3_predicted	XM_219388	293539	-0.56	-3.614	0.017137
A_44_P366026	XM_225810	XM_225810		-0.56	-3.614	0.04493
A_42_P831499	Egf	NM_012842	25313	-0.56	-3.615	0.000222
A_44_P400965	XM_222132	XM_222132		-0.56	-3.615	0.012208
A_44_P274624	LOC367493	XM_001075365		-0.56	-3.616	0.017805
A_44_P580120	TC563372	TC563372		-0.56	-3.617	0.031547
A_44_P389744	BI303389	BI303389		-0.56	-3.618	0.00363
A_44_P197663	Mcpt1l2	XM_573783		-0.56	-3.618	0.000397
A_43_P12433	Grid2	NM_024379	79220	-0.56	-3.619	0.000858
A_44_P316002	Nr1i3	NM_022941	65035	-0.56	-3.621	0.004298
A_43_P14961	AA850752	AA850752	368002	-0.56	-3.622	9.85E-05
A_44_P277669	RGD1559961_predicted	XM_573168	497974	-0.56	-3.624	2.03E-05
A_44_P358548	Kcns2	NM_023966	66022	-0.56	-3.628	0.012374
A_44_P525705	BE111870	BE111870	361652	-0.56	-3.632	0.001243
A_44_P129869	AA997129	AA997129	117036	-0.56	-3.633	0.000352
A_44_P464297	XM_229326	XM_229326		-0.56	-3.634	0.010344
A_43_P11776	Gstm1	NM_017014	24423	-0.56	-3.642	2.41E-05
A_43_P18537	RGD1561023_predicted	XM_573680	498429	-0.56	-3.646	0.016621
A_44_P604656	AI237884	AI237884	24177	-0.56	-3.647	0.011573
A_44_P506614	LOC681204	XM_001060741		-0.56	-3.649	0.008483
A_44_P502353	LOC259246	NM_147214	259246	-0.56	-3.652	0.008452
A_44_P400784	RGD1561654_predicted	XM_227826		-0.56	-3.652	0.000127
A_44_P269903	XM_346173	XM_346173		-0.56	-3.653	0.002587
A_44_P287956	Spergen2	NM_198684	305491	-0.56	-3.653	0.001409
A_44_P314146	LOC685617	XM_001064543		-0.56	-3.654	0.028212
A_44_P1011855	RGD1309744_predicted	XM_230574		-0.56	-3.655	3.55E-06
A_44_P508212	Olr79	NM_001002270	293222	-0.56	-3.656	0.000587
A_44_P776182	TC552147	TC552147		-0.56	-3.658	0.007677
A_44_P109778	Hdac7a	XM_345868	84582	-0.56	-3.661	0.004245
A_44_P167305	CA505012	CA505012	64831	-0.56	-3.661	0.013529
A_43_P23036	RGD1305248_predicted	XM_216738	299151	-0.56	-3.666	0.000415
A_44_P154754	RGD1311987_predicted	XM_220517	287366	-0.56	-3.666	0.000281
A_44_P107882	XM_232928	XM_232928		-0.56	-3.668	0.000353
A_44_P138167	Uap1_predicted	XM_222863	304954	-0.56	-3.670	4.52E-05
A_44_P563679	TC532519	TC532519		-0.56	-3.671	2.89E-05
A_44_P422152	LOC681405	XM_001061602		-0.56	-3.671	0.016734
A_44_P135020	LOC259244	NM_147212	259244	-0.56	-3.671	0.021351
A_44_P709366	BF286186	BF286186		-0.56	-3.671	0.008879
A_44_P775952	ENSRNOT00000043791	ENSRNOT00000043791		-0.56	-3.672	0.026517
A_44_P790362	AA900750	AA900750		-0.57	-3.673	0.01237
A_43_P19789	XM_216963	XM_216963		-0.57	-3.675	7.39E-06
A_44_P367586	Olr390_predicted	NM_001000558	365885	-0.57	-3.677	0.002662
A_43_P15424	Rapgef3	NM_021690	59326	-0.57	-3.680	9.49E-05
A_44_P241885	Ech1	NM_022594	64526	-0.57	-3.684	3.95E-06
A_44_P518460	Tmprss13_predicted	XM_236201	300682	-0.57	-3.690	0.098863
A_44_P255619	Vmd2l1_predicted	XM_344742		-0.57	-3.691	0.000709
A_44_P160697	RGD1311381_predicted	XM_001063356		-0.57	-3.694	0.000063
A_44_P988121	RGD1560201_predicted	XM_001078310		-0.57	-3.700	0.000494
A_44_P182431	Srd5a1	NM_017070	24950	-0.57	-3.701	9.72E-05
A_44_P624421	TC528313	TC528313		-0.57	-3.701	0.004741
A_44_P550490	Grem1	NM_019282	50566	-0.57	-3.701	7.63E-05
A_44_P165612	Olr1415_predicted	NM_001000781	405059	-0.57	-3.705	0.017011
A_44_P220424	MGC116197	NM_001025755	367620	-0.57	-3.707	0.006961
A_43_P13138	Dhrs9	NM_130819	170635	-0.57	-3.709	0.00012
A_44_P636568	ENSRNOT00000006196	ENSRNOT00000006196		-0.57	-3.710	0.012679
A_44_P252946	Dpf2_predicted	XM_341998		-0.57	-3.713	1.52E-05
A_44_P151574	Olr1401_predicted	NM_001000003	287247	-0.57	-3.716	0.001139
A_44_P284001	MGC116197	NM_001025755	367620	-0.57	-3.716	0.03713
A_44_P288916	CB544899	CB544899		-0.57	-3.722	0.00103

A_44_P780781	TC564995	TC564995		-0.57	-3.724	0.00027
A_44_P762342	TC542488	TC542488		-0.57	-3.726	0.001259
A_43_P16359	Epha8	XM_342952	60589	-0.57	-3.727	0.004332
A_44_P398438	RGD1564313_predicted	XM_578051		-0.57	-3.729	0.006873
A_44_P212575	Btnl7	NM_212488	406159	-0.57	-3.729	8.59E-05
A_44_P496948	Casc4_predicted	XM_342506	362204	-0.57	-3.730	0.001326
A_44_P471631	Gpatc2	NM_001011909	289362	-0.57	-3.731	2.52E-05
A_44_P187418	LOC306365	XM_224750		-0.57	-3.731	0.028201
A_44_P363051	Olr154_predicted	NM_001000547	365333	-0.57	-3.733	0.003275
A_44_P371144	AW917537	AW917537		-0.57	-3.733	0.000843
A_44_P404367	AA859192	AA859192	361973	-0.57	-3.738	0.020372
A_44_P111825	RGD1305679	NM_001014144	361228	-0.57	-3.742	0.00027
A_44_P656430	TC526914	TC526914		-0.57	-3.744	3.09E-05
A_44_P121296	Ctsr	NM_175581	290975	-0.57	-3.745	0.006428
A_44_P106351	AW528637	AW528637		-0.57	-3.746	0.000735
A_44_P194806	Baalc	NM_144762	140720	-0.57	-3.748	0.02598
A_44_P473243	RGD1310958_predicted	XM_223359	305307	-0.57	-3.749	1.93E-05
A_42_P636400	LOC191574	NM_138547	191574	-0.57	-3.750	0.003163
A_44_P218152	RGD1566251_predicted	XM_234969		-0.57	-3.751	0.015069
A_44_P806582	ENSRNOT00000042438	ENSRNOT00000042438		-0.57	-3.754	0.014352
A_43_P20548	RGD1560989_predicted	XM_232779	313060	-0.57	-3.756	0.000753
A_44_P310499	AA850040	AA850040	64185	-0.58	-3.758	0.008093
A_44_P489683	Ptprh	XM_574357	171125	-0.58	-3.759	3.91E-05
A_44_P125069	Trp53inp2	XM_001063973		-0.58	-3.766	3.02E-05
A_43_P10259	Coro2a	XM_575819		-0.58	-3.766	0.000018
A_44_P776390	TC520405	TC520405		-0.58	-3.771	3.41E-05
A_42_P485961	Chgb	NM_012526	24259	-0.58	-3.777	0.005618
A_44_P360972	RGD1562647_predicted	XM_344197		-0.58	-3.778	0.000659
A_43_P15669	LOC687008	XM_001075496		-0.58	-3.778	4.36E-05
A_44_P1037706	RGD1564596_predicted	XM_217593		-0.58	-3.779	1.61E-05
A_44_P372950	RGD1304884_predicted	XM_226529		-0.58	-3.780	3.87E-05
A_44_P144828	RGD1564513_predicted	XM_345237		-0.58	-3.787	0.006244
A_43_P12110	Nr5a2	NM_021742	60349	-0.58	-3.789	7.61E-06
A_44_P323012	LOC363266	BC099202	363266	-0.58	-3.791	0.000377
A_44_P325894	Zfp316_predicted	XM_221916		-0.58	-3.792	0.002368
A_43_P13148	Hrh4	NM_131909	170704	-0.58	-3.797	0.007101
A_42_P631122	Cd302	NM_001013916	295629	-0.58	-3.800	0.000248
A_44_P167875	AA819899	AA819899	64365	-0.58	-3.803	0.000294
A_44_P229686	LOC690466	XM_229431		-0.58	-3.803	0.017678
A_44_P175621	Rag1ap1_predicted	XM_215626		-0.58	-3.808	4.32E-06
A_44_P581964	A_44_P581964	A_44_P581964		-0.58	-3.812	0.000898
A_44_P474577	AI008416	AI008416	364154	-0.58	-3.815	0.000407
A_44_P410265	RGD1562126_predicted	XM_228699		-0.58	-3.821	0.007565
A_44_P241504	RGD1559655_predicted	XM_342947	362629	-0.58	-3.824	2.44E-05
A_44_P406074	Insig1	NM_022392	64194	-0.58	-3.825	0.000162
A_44_P278794	Etsrp71_predicted	XM_341830	361544	-0.58	-3.826	4.77E-05
A_44_P203812	Gmds	NM_001039606	291095	-0.58	-3.826	8.18E-06
A_44_P116251	Senp17	NM_001002833	408216	-0.58	-3.829	0.014728
A_44_P548224	A_44_P548224	A_44_P548224		-0.58	-3.836	0.000318
A_44_P370267	Ethe1_predicted	XM_214838		-0.58	-3.838	0.000309
A_44_P406069	Insig1	NM_022392	64194	-0.58	-3.844	8.78E-06
A_44_P113662	AA925959	AA925959	313554	-0.59	-3.849	0.000455
A_42_P806264	Zfand2b	NM_001025745	363253	-0.59	-3.850	0.00124
A_43_P22148	LOC690865	XM_001075929	690865	-0.59	-3.855	0.014583
A_44_P1009594	Cyp3a62	NM_001024232	170509	-0.59	-3.856	0.000406
A_44_P744974	Mlph	NM_001012135	316620	-0.59	-3.858	1.55E-05
A_44_P494274	Trpv6	NM_053686	114246	-0.59	-3.862	0.006877
A_44_P110221	LOC689083	XM_001069448		-0.59	-3.863	0.001481
A_44_P476160	RGD1565062_predicted	XM_576582	501158	-0.59	-3.864	0.005372
A_44_P283508	Fmo1	NM_012792	25256	-0.59	-3.867	0.000309
A_44_P838490	Rgr_predicted	XM_224673		-0.59	-3.867	0.000478
A_44_P683495	RGD1565058_predicted	XM_576989	501587	-0.59	-3.867	0.00013
A_44_P490092	N22.1	XM_233427	313527	-0.59	-3.868	2.71E-05

A_44_P325629	Olr1425_predicted	NM_001000010	287316	-0.59	-3.868	9.47E-05
A_44_P246081	Gpr162_predicted	XM_342757		-0.59	-3.872	0.000187
A_42_P754715	Slc13a2	NM_031746	65202	-0.59	-3.873	5.33E-05
A_44_P635089	Etv5_predicted	XM_001057770		-0.59	-3.874	0.000483
A_44_P182795	A_44_P182795	A_44_P182795		-0.59	-3.877	0.003816
A_44_P171995	Fbp1	NM_012558	24362	-0.59	-3.884	0.002146
A_44_P400299	Olr428_predicted	NM_001000394	296689	-0.59	-3.888	0.000465
A_44_P545426	Slc16a1	NM_012716	25027	-0.59	-3.889	0.000241
A_44_P168159	Daf1	NM_022269	64036	-0.59	-3.890	0.000188
A_44_P395315	Gpt1	NM_031039	81670	-0.59	-3.895	5.73E-06
A_44_P1037972	Ckmt1	NM_001012738	29593	-0.59	-3.896	2.3E-06
A_43_P16946	Ifnar1_predicted	XM_213649		-0.59	-3.900	0.001316
A_44_P834352	AA998814	AA998814		-0.59	-3.902	0.012309
A_43_P21764	RGD1308319_predicted	XM_237548	316764	-0.59	-3.907	0.004087
A_44_P405793	Npas2_predicted	XM_244089		-0.59	-3.907	4.83E-06
A_43_P19318	Cdk9	NM_001007743	362110	-0.59	-3.911	0.001513
A_44_P962921	TC567098	TC567098		-0.59	-3.919	0.002662
A_44_P775631	RGD1564854_predicted	XM_578108		-0.59	-3.922	2.07E-05
A_44_P720404	RGD1564895_predicted	XM_231564	312192	-0.59	-3.923	5.62E-05
A_44_P284011	XM_233420	XM_233420		-0.59	-3.924	0.000484
A_44_P286273	LOC683719	XM_001067187		-0.59	-3.926	0.000021
A_44_P450348	A_44_P450348	A_44_P450348		-0.59	-3.929	0.008791
A_44_P391426	Klk11_predicted	XM_218645		-0.59	-3.931	1.93E-05
A_42_P646567	LOC691209	XM_001066648		-0.59	-3.932	0.000405
A_44_P350968	AA945826	AA945826		-0.60	-3.936	0.001966
A_43_P11116	Tmprss8	NM_199371	287106	-0.60	-3.937	7.75E-05
A_43_P14444	BC091425	BC091425		-0.60	-3.937	7.74E-05
A_44_P436513	ttc21a_predicted	XM_001078305		-0.60	-3.938	0.00129
A_44_P519345	CF110294	CF110294		-0.60	-3.945	4.76E-05
A_44_P541744	AA819014	AA819014	83840	-0.60	-3.946	1.86E-05
A_44_P154579	XM_229609	XM_229609		-0.60	-3.947	0.053238
A_44_P763823	TC551463	TC551463		-0.60	-3.950	0.017794
A_42_P685320	RGD1566130_predicted	XM_001073977		-0.60	-3.955	3.46E-05
A_43_P22136	RGD1306809_predicted	XM_345167		-0.60	-3.958	0.000249
A_44_P504580	AI029143	AI029143	310693	-0.60	-3.963	0.000967
A_42_P812008	Nupr1	NM_053611	113900	-0.60	-3.967	0.000179
A_44_P604882	AI548777	AI548777		-0.60	-3.970	0.002663
A_44_P349277	RGD1562653_predicted	XM_218843		-0.60	-3.973	9.58E-06
A_44_P163968	MGC116197	NM_001025755	367620	-0.60	-3.977	0.009331
A_44_P161233	Il18	NM_019165	29197	-0.60	-3.980	0.000426
A_43_P12586	Ptgis	NM_031557	25527	-0.60	-3.981	0.001813
A_44_P541120	RGD1311107	NM_001014016	308428	-0.60	-3.983	0.002326
A_44_P153755	Fshr	NM_199237	25449	-0.60	-3.988	0.00096
A_44_P494995	BF549345	BF549345	313339	-0.60	-3.990	0.000438
A_44_P274321	XM_344318	XM_344318		-0.60	-3.990	0.002497
A_44_P276492	Vdr	NM_017058	24873	-0.60	-3.996	0.000259
A_44_P171004	LOC307582	XM_226159		-0.60	-3.999	4.78E-05
A_44_P259681	AI454895	AI454895	59114	-0.60	-4.001	1.88E-05
A_44_P683099	RGD1564313_predicted	XM_578051		-0.60	-4.001	0.012003
A_44_P448717	Prom1	NM_021751	60357	-0.60	-4.008	6.89E-05
A_43_P15916	Ryr3	XM_342491	170546	-0.60	-4.010	0.00011
A_44_P244888	CN542383	CN542383	363674	-0.60	-4.018	0.003659
A_44_P224516	Scg2	NM_022669	24765	-0.60	-4.018	0.001132
A_44_P191762	Cyp2b15	XM_001070774		-0.60	-4.018	0.000735
A_44_P332943	Pla2g3_predicted	XM_223553		-0.60	-4.019	0.000117
A_44_P515417	Pnsp1	NM_001024283	498854	-0.60	-4.020	0.022572
A_44_P253674	BF290953	BF290953		-0.60	-4.020	0.000109
A_44_P217761	LOC680443	XM_001057208	680443	-0.61	-4.029	0.006407
A_44_P820923	AI233279	AI233279	312516	-0.61	-4.039	0.001175
A_44_P450685	LOC687561	XM_001079149		-0.61	-4.043	8.47E-05
A_44_P105078	LOC682030	XM_001059475		-0.61	-4.046	0.013723
A_44_P187264	Lrfn1_predicted	XM_344874		-0.61	-4.054	0.000312
A_44_P553791	Apob	NM_019287	54225	-0.61	-4.056	2.49E-05

A_44_P297337	RGD1562031_predicted	XR_008157	363391	-0.61	-4.057	0.000398
A_44_P358703	Spetex-2D	NM_001011701	364261	-0.61	-4.057	0.013528
A_44_P374909	AB097854	AB097854		-0.61	-4.058	0.00222
A_44_P967547	A_44_P967547	A_44_P967547		-0.61	-4.058	0.004008
A_44_P283670	A_44_P283670	A_44_P283670		-0.61	-4.059	0.000116
A_42_P504077	Pdxp	AF318578	727679	-0.61	-4.059	0.000286
A_43_P11958	Chad	NM_019164	29195	-0.61	-4.061	0.003738
A_44_P438015	Olr183_predicted	NM_001000181	293328	-0.61	-4.069	0.006522
A_44_P123965	XM_344678	XM_344678		-0.61	-4.073	0.012759
A_42_P533312	Pc	NM_012744	25104	-0.61	-4.074	7.33E-06
A_44_P677914	A_44_P677914	A_44_P677914		-0.61	-4.075	0.003505
A_42_P496291	CB547706	CB547706		-0.61	-4.079	0.001374
A_44_P785903	BQ782222	BQ782222		-0.61	-4.080	0.001111
A_44_P1002317	RGD1561665_predicted	XM_001055117		-0.61	-4.081	6.49E-05
A_44_P162012	AI012402	AI012402	307382	-0.61	-4.082	0.002625
A_44_P455846	BQ207336	BQ207336		-0.61	-4.086	0.015678
A_44_P413701	Mor1	NM_031151	81829	-0.61	-4.087	7.17E-05
A_44_P433701	ENSRNOT00000041834	ENSRNOT00000041834		-0.61	-4.088	0.019327
A_44_P121385	XM_344807	XM_344807		-0.61	-4.088	0.000201
A_44_P128007	Slco2b1	NM_080786	140860	-0.61	-4.089	0.000456
A_44_P348812	Hist1h1t	NM_012579	24438	-0.61	-4.090	0.000166
A_44_P463749	Rasd1	XM_340809	64455	-0.61	-4.090	5.59E-05
A_43_P11833	Clcn2	NM_017137	29232	-0.61	-4.091	3.08E-05
A_44_P185115	XM_233898	XM_233898		-0.61	-4.099	0.005991
A_42_P784541	Mpv17l_predicted	XM_340739		-0.61	-4.100	0.002844
A_44_P315575	AI180367	AI180367	64464	-0.61	-4.102	0.000352
A_44_P206455	A_44_P206455	A_44_P206455		-0.61	-4.109	0.002543
A_44_P446312	CB547642	CB547642	315634	-0.61	-4.111	0.004231
A_44_P166544	ENSRNOT00000020184	ENSRNOT00000020184		-0.61	-4.113	0.000221
A_42_P836392	Elovl6	NM_134383	171402	-0.61	-4.114	2.52E-05
A_44_P496712	Smg7_predicted	XM_341132	360855	-0.61	-4.117	0.010639
A_44_P550453	Naaladl1	NM_031759	83568	-0.61	-4.117	0.000176
A_44_P152317	A_44_P152317	A_44_P152317		-0.62	-4.121	0.003137
A_44_P111306	BG373817	BG373817	116675	-0.62	-4.126	0.000498
A_44_P676009	A_44_P676009	A_44_P676009		-0.62	-4.126	0.000214
A_44_P551965	Y17323	Y17323		-0.62	-4.130	0.00011
A_42_P498463	Pla2g10	NM_017176	29359	-0.62	-4.134	9.62E-05
A_44_P124723	BF416433	BF416433	499418	-0.62	-4.134	0.012168
A_44_P501724	LOC501308	XM_576721		-0.62	-4.138	0.019657
A_42_P586141	Plcd1	NM_017035	24655	-0.62	-4.141	0.000129
A_44_P313997	LOC365507	XM_345062		-0.62	-4.141	0.00037
A_44_P141963	Sult1c2a	NM_001013177	316153	-0.62	-4.145	9.16E-05
A_44_P668293	TC519976	TC519976		-0.62	-4.148	0.000392
A_42_P576525	Sstr2	NM_019348	54305	-0.62	-4.153	0.000935
A_42_P707992	Tspan18_predicted	XM_230297		-0.62	-4.153	0.00053
A_44_P448674	AA858650	AA858650		-0.62	-4.156	0.000352
A_44_P1031063	RGD1305326_predicted	XM_215707	295462	-0.62	-4.156	0.000773
A_44_P1013851	Bmp2	NM_017178	29373	-0.62	-4.159	3.64E-05
A_43_P18263	Cugbp1	NM_001025421	362160	-0.62	-4.161	0.000621
A_44_P549258	BE104457	BE104457		-0.62	-4.167	0.0148
A_44_P438034	Olr1378_predicted	NM_214828	287086	-0.62	-4.167	0.000155
A_44_P606411	RGD1564406_predicted	XM_576988	501586	-0.62	-4.170	0.000309
A_44_P482325	Fhl1	NM_145669	25177	-0.62	-4.173	4.75E-06
A_44_P142523	RGD1560576_predicted	XM_001070261		-0.62	-4.175	6.03E-05
A_44_P283449	Kalrn	AF232668	84009	-0.62	-4.176	3.03E-05
A_44_P452349	ENSRNOT00000035902	ENSRNOT00000035902		-0.62	-4.178	0.019165
A_44_P590971	ENSRNOT00000038482	ENSRNOT00000038482		-0.62	-4.179	0.001493
A_44_P213477	LOC682159	XM_001060179		-0.62	-4.183	0.000399
A_44_P212894	X83264	X83264		-0.62	-4.191	0.000318
A_44_P468258	Casp1	NM_012762	25166	-0.62	-4.192	0.000178
A_44_P533811	LOC259246	NM_147214	259246	-0.62	-4.194	0.008461
A_44_P265409	A_44_P265409	A_44_P265409		-0.62	-4.196	0.000147
A_44_P348719	AA899157	AA899157	362277	-0.62	-4.197	0.029659

A_44_P130856	LOC684383	XM_001070139		-0.62	-4.198	0.002155
A_43_P12301	Ntsr2	NM_022695	64636	-0.62	-4.198	0.000304
A_44_P323360	Ank3	NM_001033984	361833	-0.62	-4.199	0.000271
A_44_P438460	A_44_P438460	A_44_P438460		-0.62	-4.200	0.015526
A_44_P445031	Fbxo32	NM_133521	171043	-0.62	-4.201	0.000445
A_44_P654754	TC559712	TC559712		-0.62	-4.202	0.005317
A_44_P430033	AI231180	AI231180	260416	-0.62	-4.202	0.000588
A_44_P252707	Cdh3	XM_226426	116777	-0.62	-4.202	0.00198
A_44_P548024	Sult1c2	NM_133547	171072	-0.62	-4.205	5.22E-05
A_44_P258095	A_44_P258095	A_44_P258095		-0.62	-4.212	0.015295
A_44_P196256	Olr1203_predicted	NM_001001082	405972	-0.62	-4.213	0.017407
A_44_P265176	XM_228733	XM_228733		-0.63	-4.219	0.002064
A_42_P681606	LOC688173	XM_001081394		-0.63	-4.220	0.000732
A_44_P178202	Luc7l2_predicted	XM_231620		-0.63	-4.239	5.45E-06
A_44_P257749	RGD1565301_predicted	XM_344954		-0.63	-4.244	0.000317
A_44_P255728	LOC363337	NM_001014221	363337	-0.63	-4.244	0.013196
A_44_P492173	Capsl_predicted	XM_215500		-0.63	-4.247	0.002426
A_43_P23060	Hsfy1	NM_001012132	316409	-0.63	-4.254	0.017445
A_44_P245708	Gm52_predicted	NM_001014771	288602	-0.63	-4.265	0.002298
A_44_P276990	RGD1307401	NM_001014088	315500	-0.63	-4.266	5.63E-06
A_44_P852201	A_44_P852201	A_44_P852201		-0.63	-4.267	0.000136
A_44_P367872	XM_344844	XM_344844		-0.63	-4.268	0.012041
A_44_P213903	BG380118	BG380118	311670	-0.63	-4.272	3.89E-05
A_44_P440698	RGD1564982_predicted	XM_218633	308556	-0.63	-4.276	0.000101
A_44_P247880	Csf2	XM_340799	116630	-0.63	-4.277	4.51E-05
A_44_P471237	XM_229439	XM_229439		-0.63	-4.280	0.013529
A_44_P211168	RGD1559960_predicted	XM_576496	501084	-0.63	-4.281	0.000074
A_44_P226752	Olr1006_predicted	NM_001000075	288886	-0.63	-4.281	0.025969
A_44_P291328	AA818363	AA818363	364676	-0.63	-4.284	0.001617
A_44_P306581	Gdf5	XM_001066344		-0.63	-4.286	0.000331
A_44_P459679	A_44_P459679	A_44_P459679		-0.63	-4.289	2.67E-06
A_44_P887721	XM_576679	XM_576679		-0.63	-4.292	0.017223
A_44_P322590	Klf9	NM_057211	117560	-0.63	-4.295	0.00042
A_44_P781160	TC528119	TC528119		-0.63	-4.299	0.02
A_44_P330109	Hps1	NM_040669	114638	-0.63	-4.304	6.29E-05
A_44_P154733	Rgs11	XM_001062856		-0.63	-4.305	7.24E-06
A_44_P460433	AI576488	AI576488	296959	-0.63	-4.307	2.38E-05
A_43_P17112	LOC361041	NM_001014138		-0.63	-4.307	0.000315
A_44_P329873	AA859361	AA859361	362454	-0.63	-4.310	0.000137
A_44_P745633	TC538833	TC538833		-0.63	-4.313	0.009083
A_44_P442728	ENSRNOT00000043992	ENSRNOT00000043992		-0.63	-4.314	0.00053
A_44_P170257	AW252095	AW252095	287159	-0.64	-4.315	0.00016
A_44_P293804	BI294806	BI294806		-0.64	-4.317	0.002032
A_44_P302476	XM_343707	XM_343707		-0.64	-4.325	0.004902
A_44_P448892	AA899841	AA899841	362934	-0.64	-4.332	0.003747
A_44_P454790	LOC306312	XM_224680	306312	-0.64	-4.333	0.001261
A_44_P988749	BI274156	BI274156	296360	-0.64	-4.341	0.001936
A_44_P219699	Olr749_predicted	NM_001000615	404804	-0.64	-4.348	0.00059
A_44_P512017	P2rx2	NM_053656	114115	-0.64	-4.351	2.15E-06
A_42_P719350	RGD1562974_predicted	XM_001081721		-0.64	-4.351	5.03E-06
A_44_P293405	RGD1559450_predicted	XM_573124	497937	-0.64	-4.353	0.002065
A_44_P548857	Igsf4d_predicted	XM_340958		-0.64	-4.354	4.21E-05
A_44_P700971	TC524582	TC524582		-0.64	-4.357	0.004377
A_44_P123706	RGD1565723_predicted	XM_577714		-0.64	-4.358	0.0005
A_43_P22318	Prss32_predicted	XM_220209		-0.64	-4.362	0.000124
A_44_P330424	Acf	NM_133400	170912	-0.64	-4.364	0.000215
A_44_P305482	Ppargc1a	NM_031347	83516	-0.64	-4.365	3.87E-06
A_44_P192772	RGD1564833_predicted	XM_573525	498297	-0.64	-4.366	2.03E-06
A_44_P128342	Gpr4	NM_001025680	308408	-0.64	-4.369	0.000442
A_44_P1021367	RGD1310484_predicted	XM_214594		-0.64	-4.376	5.39E-06
A_44_P478172	RGD1566337_predicted	XM_227344		-0.64	-4.381	0.006916
A_44_P208963	AI009123	AI009123	307642	-0.64	-4.385	0.013468
A_42_P774181	Gnat1_predicted	XM_343480		-0.64	-4.387	0.002747

A_44_P443976	St3gal3	NM_031697	64445	-0.64	-4.390	9.68E-06
A_44_P166068	Spetex-2D	NM_001011701	364261	-0.64	-4.394	0.027892
A_44_P128396	Ugt2b10_predicted	XM_223299	305264	-0.64	-4.407	3.15E-05
A_44_P131470	Hexb	NM_001011946	294673	-0.64	-4.412	7.21E-06
A_43_P17914	Dpf3_predicted	XM_238462	299186	-0.65	-4.416	2.96E-05
A_44_P908591	LOC501126	XM_001055017	501126	-0.65	-4.417	4.79E-05
A_43_P11499	Rbp2	NM_012640	24710	-0.65	-4.420	6.84E-05
A_44_P152350	LOC685987	XM_001066074		-0.65	-4.421	0.006275
A_43_P15808	Myo1a	XM_238479	299509	-0.65	-4.422	8.49E-05
A_44_P886003	TC528135	TC528135		-0.65	-4.423	0.004882
A_44_P469394	Amph1	NM_022217	60668	-0.65	-4.424	0.001516
A_42_P601665	Cxadrl1	NM_199406	361873	-0.65	-4.425	4.61E-06
A_44_P288428	LOC365791	NM_001014252	365791	-0.65	-4.425	0.012632
A_43_P17415	Slc16a6	NM_198760	303772	-0.65	-4.431	1.46E-05
A_43_P17103	Ms4a8b_predicted	XM_342026		-0.65	-4.431	5.39E-06
A_44_P223320	BE115491	BE115491		-0.65	-4.431	0.007578
A_44_P655056	TC545913	TC545913		-0.65	-4.432	0.011401
A_44_P305870	AI171466	AI171466	291966	-0.65	-4.433	0.008699
A_44_P443377	XM_343688	XM_343688		-0.65	-4.440	0.031059
A_44_P526978	Bmp3	NM_017105	25667	-0.65	-4.448	0.000429
A_44_P917688	TC523731	TC523731		-0.65	-4.455	0.000927
A_44_P274900	BF412752	BF412752	302953	-0.65	-4.455	0.001324
A_44_P913193	LOC684788	XM_001071941		-0.65	-4.458	0.000389
A_44_P436040	MGC114410	NM_001025671	305251	-0.65	-4.459	2.48E-05
A_44_P108682	Ppp2r2c	NM_057116	117256	-0.65	-4.467	0.000406
A_44_P199514	Thap4	NM_001005564	363291	-0.65	-4.467	4.8E-06
A_44_P407204	Pgap1	NM_201990	316400	-0.65	-4.468	0.00018
A_44_P883087	RGD1562103_predicted	XM_573469		-0.65	-4.472	5.31E-05
A_44_P270868	AI502665	AI502665	56083	-0.65	-4.473	0.000393
A_44_P288156	Cbr1	NM_019170	29224	-0.65	-4.489	0.000128
A_44_P496408	RGD1303144	NM_001004266	311833	-0.65	-4.494	0.009959
A_43_P12974	F2rl1	NM_053897	116677	-0.65	-4.494	0.000233
A_44_P129053	AA848383	AA848383		-0.65	-4.505	0.000855
A_44_P534478	A_44_P534478	A_44_P534478		-0.65	-4.506	0.000244
A_44_P325808	Optn	NM_145081	246294	-0.65	-4.509	5.41E-06
A_44_P928726	LOC683745	XM_001067304		-0.65	-4.510	0.000626
A_44_P463530	Optn	NM_145081	246294	-0.66	-4.519	7.65E-06
A_44_P806703	LOC503316	XR_009187	503316	-0.66	-4.526	0.004748
A_44_P293893	Kalrn	NM_032062	84009	-0.66	-4.526	0.000131
A_44_P619218	CO402545	CO402545	314352	-0.66	-4.529	0.000157
A_44_P369997	Trpm4	XM_574447	171143	-0.66	-4.532	6.56E-06
A_44_P426919	XM_345601	XM_345601		-0.66	-4.541	0.000298
A_44_P456836	ENSRNOT00000049443	ENSRNOT00000049443		-0.66	-4.543	0.000021
A_44_P775538	LOC679060	XM_001054517		-0.66	-4.543	3.75E-05
A_43_P15545	Tcam1	NM_021673	59305	-0.66	-4.548	0.007399
A_42_P741034	RGD1566282_predicted	XM_236533	315911	-0.66	-4.549	0.000836
A_44_P347167	RGD1563150_predicted	XM_233323		-0.66	-4.561	0.000928
A_44_P400693	LOC686177	XM_001072778		-0.66	-4.570	0.000014
A_44_P515895	RGD1565470_predicted	XM_216130	297025	-0.66	-4.570	0.009974
A_44_P761679	BF550875	BF550875		-0.66	-4.572	0.019309
A_42_P524009	AW143180	AW143180		-0.66	-4.573	0.000296
A_44_P467696	Rln3	NM_170667	266997	-0.66	-4.576	0.015012
A_44_P223971	AI059491	AI059491	116593	-0.66	-4.576	0.000244
A_44_P201675	XM_229476	XM_229476		-0.66	-4.585	0.021261
A_42_P527738	RGD1311293	NM_001025010	315599	-0.66	-4.585	0.000201
A_42_P610788	Ampd3	NM_031544	25095	-0.66	-4.592	4.57E-06
A_44_P353587	Olr1012_predicted	NM_001000071	288861	-0.66	-4.593	0.000287
A_44_P102523	Slc13a2	NM_031746	65202	-0.67	-4.633	3.39E-05
A_44_P262888	LOC363366	XM_343700		-0.67	-4.634	0.003825
A_44_P121635	A_44_P121635	A_44_P121635		-0.67	-4.634	0.007586
A_44_P883196	ENSRNOT00000048619	ENSRNOT00000048619		-0.67	-4.634	0.019934
A_44_P821368	RGD1560958_predicted	XM_574575		-0.67	-4.635	0.00425
A_44_P312484	Mtmr4_predicted	XM_213426		-0.67	-4.637	2.76E-05

A_44_P541505	LOC688519	XM_001067241		-0.67	-4.641	0.00034
A_44_P242949	Cyp2j9	NM_175766	313375	-0.67	-4.645	5.66E-05
A_44_P958682	Sult1c2a	NM_001013177	316153	-0.67	-4.648	7.16E-05
A_44_P725554	BF288456	BF288456	360928	-0.67	-4.651	0.000454
A_44_P306687	Sytl4	NM_080410	140594	-0.67	-4.656	4.21E-05
A_44_P341029	AABR03128894	AABR03128894		-0.67	-4.658	0.002405
A_44_P290734	LOC680220	XM_001056169		-0.67	-4.659	0.000153
A_44_P433676	Hnf4g_predicted	XM_345188		-0.67	-4.667	0.000106
A_42_P781006	Slc6a8	NM_017348	50690	-0.67	-4.668	3.13E-05
A_44_P163750	RGD1564015_predicted	XM_578062		-0.67	-4.669	0.005868
A_44_P504363	AA924881	AA924881	361126	-0.67	-4.681	0.000477
A_44_P676327	LOC498372	XM_001061548		-0.67	-4.682	0.004738
A_44_P110801	CB544812	CB544812	308106	-0.67	-4.688	8.23E-06
A_44_P388500	BF283661	BF283661		-0.67	-4.692	0.012659
A_42_P614984	Ucp1	NM_012682	24860	-0.67	-4.695	0.015138
A_44_P380268	RGD1560570_predicted	XM_229665		-0.67	-4.699	0.008586
A_44_P194944	BI286131	BI286131	289357	-0.67	-4.700	0.00023
A_42_P544328	Mic2l1	NM_134459	171485	-0.67	-4.702	8.54E-05
A_44_P330760	A_44_P330760	A_44_P330760		-0.67	-4.708	0.012585
A_44_P548507	Chn1	NM_032083	84030	-0.67	-4.719	8.82E-06
A_44_P478144	LOC290595	XM_237967		-0.67	-4.722	0.00032
A_44_P392359	Myrip	NM_182844	360034	-0.67	-4.726	3.88E-05
A_44_P180627	LOC691487	XM_001078495		-0.67	-4.730	0.001543
A_44_P243355	Atp10a	XM_344901	365266	-0.68	-4.735	0.000678
A_44_P233677	Olr1699_predicted	NM_001001112	406011	-0.68	-4.737	0.004749
A_44_P111927	RGD1562035_predicted	XM_224209		-0.68	-4.738	6.69E-05
A_44_P498854	Muc10	NM_133513	65182	-0.68	-4.740	6.88E-05
A_44_P296468	AI071174	AI071174		-0.68	-4.741	4.52E-05
A_44_P356738	LOC501080	XM_576492		-0.68	-4.745	0.000367
A_42_P489232	AW916024	AW916024	294375	-0.68	-4.747	5.69E-05
A_44_P503125	AW529067	AW529067		-0.68	-4.753	0.012201
A_44_P410299	XM_229360	XM_229360		-0.68	-4.754	0.01527
A_44_P222733	XM_229373	XM_229373		-0.68	-4.756	0.007361
A_44_P220533	Vsig2_predicted	XM_236059		-0.68	-4.757	0.000189
A_44_P297703	RGD1564437_predicted	XM_228808		-0.68	-4.758	0.001508
A_44_P126564	Mon1a_predicted	XM_236627	315999	-0.68	-4.760	0.000419
A_44_P194351	A_44_P194351	A_44_P194351		-0.68	-4.760	0.016695
A_44_P207107	AW532911	AW532911	308690	-0.68	-4.762	0.002101
A_44_P109443	Mylk2	NM_057209	117558	-0.68	-4.765	0.000511
A_43_P22377	AI058259	AI058259	298051	-0.68	-4.771	0.000646
A_44_P512369	AI716212	AI716212		-0.68	-4.772	0.000233
A_43_P11040	RGD1305061	NM_001009639	291966	-0.68	-4.775	2.16E-05
A_44_P217452	Tgoln2	NM_138840	192152	-0.68	-4.775	1.09E-05
A_44_P254437	BQ783109	BQ783109	298934	-0.68	-4.777	0.003015
A_44_P395374	Serpini1	NM_053779	116459	-0.68	-4.778	0.003539
A_44_P328535	XM_342556	XM_342556		-0.68	-4.785	7.52E-05
A_44_P168358	RGD1560808_predicted	XM_577734		-0.68	-4.785	0.016469
A_44_P991472	Rnf39	NM_134374	171387	-0.68	-4.786	0.000031
A_44_P959291	A_44_P959291	A_44_P959291		-0.68	-4.786	0.002606
A_44_P342008	Mxd1	XM_342716	362391	-0.68	-4.789	4.57E-06
A_43_P13294	Gpsn2	NM_138549	191576	-0.68	-4.791	0.000359
A_44_P454645	Igsf4b_predicted	XM_341157		-0.68	-4.793	0.00063
A_44_P179999	Cngb1	AJ224680	83686	-0.68	-4.799	1.12E-05
A_44_P355907	Abo	NM_023094	65270	-0.68	-4.810	2.27E-05
A_44_P704889	AI230259	AI230259	305482	-0.68	-4.819	0.000122
A_44_P118688	Slc25a10	NM_133418	170943	-0.68	-4.821	1.76E-05
A_44_P274675	Npas3_predicted	XM_234124		-0.68	-4.825	4.89E-05
A_44_P149906	LOC367632	XM_346234		-0.68	-4.831	0.018056
A_44_P320743	Olr804_predicted	NM_001000852	405142	-0.68	-4.840	7.81E-05
A_44_P1045950	Pcsk1n	NM_019279	246333	-0.68	-4.840	5.63E-06
A_44_P284198	XM_346096	XM_346096		-0.69	-4.842	0.00404
A_44_P400977	XM_229530	XM_229530		-0.69	-4.848	0.006963
A_42_P547616	Ndrg2	NM_133583	171114	-0.69	-4.850	0.000105

A_44_P417055	RGD1566292_predicted	XM_001054022		-0.69	-4.852	0.000284
A_44_P363071	Olr1106_predicted	NM_001000423	300199	-0.69	-4.858	0.00334
A_44_P336207	CB325472	CB325472		-0.69	-4.866	0.001451
A_44_P121781	LOC690619	XM_001074985		-0.69	-4.875	0.005577
A_44_P356890	AI406707	AI406707		-0.69	-4.877	5.39E-05
A_44_P229289	RGD1306565_predicted	XM_001073260		-0.69	-4.885	5.41E-05
A_44_P324018	XM_345747	XM_345747		-0.69	-4.887	0.000531
A_44_P340040	A_44_P340040	A_44_P340040		-0.69	-4.890	0.004992
A_44_P204016	LOC689958	XM_001072685		-0.69	-4.891	0.007766
A_44_P102588	Ces2	NM_133586	171118	-0.69	-4.894	0.000109
A_44_P118708	Olr1730_predicted	NM_213732	294187	-0.69	-4.906	0.00138
A_44_P715164	TC522684	TC522684		-0.69	-4.909	0.005095
A_43_P15319	Pla2g1b	NM_031585	29526	-0.69	-4.915	2.67E-06
A_44_P163957	XM_343681	XM_343681		-0.69	-4.917	0.00163
A_44_P821475	RGD1563425_predicted	XM_573340		-0.69	-4.933	0.004785
A_44_P398424	RGD1560014_predicted	XM_224795	306409	-0.69	-4.937	0.005339
A_44_P402464	Abcg2	NM_181381	312382	-0.69	-4.940	0.003412
A_44_P418944	Entpd5	NM_199394	314312	-0.69	-4.940	2.18E-06
A_44_P889765	A_44_P889765	A_44_P889765		-0.69	-4.948	0.001272
A_44_P107891	A_44_P107891	A_44_P107891		-0.69	-4.953	0.012545
A_44_P339646	AY325136	AY325136		-0.69	-4.954	0.000663
A_44_P384066	G6pc	NM_013098	25634	-0.70	-4.957	0.019972
A_44_P1071434	X89818	X89818	25122	-0.70	-4.959	9.83E-05
A_44_P633381	AI715326	AI715326		-0.70	-4.961	0.018167
A_44_P585834	A_44_P585834	A_44_P585834		-0.70	-4.969	0.000767
A_44_P1026848	Cryba4	NM_031689	64348	-0.70	-4.971	1.54E-05
A_44_P388375	BI282062	BI282062	366995	-0.70	-4.976	6.13E-06
A_44_P271398	BF281861	BF281861	362220	-0.70	-4.977	0.009929
A_44_P187326	RGD1563323_predicted	XM_341985	361702	-0.70	-4.989	4.19E-05
A_44_P1041880	Bcas1	BC103480	246755	-0.70	-4.989	5.82E-06
A_43_P11263	BF559333	BF559333		-0.70	-4.989	9.06E-06
A_44_P455509	AA925534	AA925534		-0.70	-4.990	0.00027
A_44_P184931	XM_218639	XM_218639		-0.70	-4.995	5.94E-06
A_44_P552692	Apob	NM_019287	54225	-0.70	-4.999	6.93E-05
A_44_P100496	Prdm8_predicted	XM_223205		-0.70	-5.000	1.99E-06
A_43_P12380	Npffr2	NM_023980	78964	-0.70	-5.003	0.002378
A_44_P203842	ENSRNOT00000024343	ENSRNOT00000024343		-0.70	-5.005	1.99E-06
A_42_P527070	Gchfr	NM_133595	171128	-0.70	-5.014	0.002406
A_44_P190037	XM_225822	XM_225822		-0.70	-5.014	0.002402
A_44_P139203	BF544403	BF544403		-0.70	-5.016	0.000212
A_44_P938161	A_44_P938161	A_44_P938161		-0.70	-5.018	0.034511
A_44_P123381	Prkg2	NM_013012	25523	-0.70	-5.026	0.003624
A_44_P252517	Havcr1	NM_173149	286934	-0.70	-5.026	0.001071
A_44_P561635	Rimbp2	XM_001073406		-0.70	-5.035	0.000759
A_44_P151482	Il22ra2	NM_001003404	444986	-0.70	-5.037	0.005732
A_44_P290662	XM_223443	XM_223443		-0.70	-5.040	0.014275
A_44_P280965	Mcl1	NM_021846	60430	-0.70	-5.044	6.45E-05
A_44_P548079	Atp2a3	AF458230	25391	-0.70	-5.051	0.001045
A_44_P527875	LOC679472	XM_001056525		-0.70	-5.055	0.000194
A_44_P382398	A_44_P382398	A_44_P382398		-0.70	-5.057	0.031239
A_44_P441221	Daam2_predicted	XM_236909	316201	-0.70	-5.060	5.89E-05
A_44_P363382	LOC689435	XM_001070788		-0.70	-5.061	0.006115
A_44_P420046	Ghrl	NM_021669	59301	-0.70	-5.062	0.000597
A_44_P867296	Ggn	NM_001013065	292765	-0.70	-5.063	4.57E-06
A_44_P744396	Tbx5	NM_001009964	304514	-0.70	-5.063	0.001415
A_44_P729866	CA511309	CA511309		-0.70	-5.068	4.8E-06
A_44_P398506	ENSRNOT00000020530	ENSRNOT00000020530		-0.71	-5.075	5.68E-05
A_44_P381764	Olr82_predicted	NM_001001282	365328	-0.71	-5.078	0.000705
A_44_P508294	Arhgef1	NM_021694	60323	-0.71	-5.085	5.44E-05
A_44_P543769	Sdcbp2	NM_001025692	311532	-0.71	-5.093	3.16E-05
A_44_P222416	RGD1564814_predicted	XM_577315		-0.71	-5.096	0.009776
A_44_P504133	RGD1312038_predicted	XM_343054	362732	-0.71	-5.109	0.000296
A_44_P135757	LOC679999	XM_001055256		-0.71	-5.110	0.000645

A_44_P227399	LOC367436	XM_346138		-0.71	-5.112	0.007604
A_44_P510481	Cuzd1	NM_054005	117179	-0.71	-5.117	0.026665
A_44_P124104	XM_241937	XM_241937		-0.71	-5.119	0.004627
A_44_P198474	Bcas1	NM_145670	246755	-0.71	-5.123	3.93E-05
A_44_P344937	XM_346150	XM_346150		-0.71	-5.140	0.000233
A_44_P800685	A_44_P800685	A_44_P800685		-0.71	-5.149	0.001091
A_44_P837219	Gucy2c	NM_013170	25711	-0.71	-5.149	2.62E-05
A_44_P157024	LOC691800	XM_001079679		-0.71	-5.150	0.010558
A_44_P683445	A_44_P683445	A_44_P683445		-0.71	-5.164	0.005084
A_42_P792348	BF556841	BF556841		-0.71	-5.166	7.52E-05
A_44_P945456	CO386194	CO386194	316351	-0.71	-5.170	6.54E-06
A_44_P445267	D84486	D84486		-0.71	-5.178	0.00685
A_42_P722862	TC537913	TC537913		-0.71	-5.184	0.00014
A_44_P937380	A_44_P937380	A_44_P937380		-0.71	-5.186	0.010241
A_44_P396347	LOC687062	XM_001076927		-0.71	-5.187	0.001362
A_44_P232076	LOC688758	XM_001068185		-0.71	-5.187	0.00911
A_44_P385633	AI230395	AI230395	117152	-0.72	-5.194	0.013983
A_44_P158441	Nr3c2	NM_013131	25672	-0.72	-5.198	4.35E-06
A_44_P516940	BF281337	BF281337	25626	-0.72	-5.200	0.00117
A_44_P518332	Dach2_predicted	XM_228458	302333	-0.72	-5.202	0.004409
A_44_P130380	Olr1250_predicted	NM_001000806	405092	-0.72	-5.202	0.002458
A_44_P445774	XM_346214	XM_346214		-0.72	-5.206	0.013412
A_44_P959775	XM_346212	XM_346212		-0.72	-5.214	0.002466
A_44_P931240	LOC684055	XM_001068703		-0.72	-5.214	0.027738
A_44_P253380	RGD1563738_predicted	XM_345920		-0.72	-5.216	0.000369
A_44_P337522	A_44_P337522	A_44_P337522		-0.72	-5.217	0.004433
A_44_P551963	Y17323	Y17323		-0.72	-5.227	5.68E-05
A_44_P429855	AABR03057448	AABR03057448		-0.72	-5.229	0.00343
A_44_P860287	CB726043	CB726043		-0.72	-5.234	0.015264
A_44_P375447	LOC363424	XM_343747		-0.72	-5.242	0.020195
A_44_P553476	RGD1310573_predicted	XM_343916		-0.72	-5.244	0.000444
A_44_P485305	LOC685424	XM_001063747		-0.72	-5.255	0.004712
A_44_P419034	Olr181_predicted	NM_001001030	405916	-0.72	-5.264	0.007858
A_44_P921786	AI072054	AI072054		-0.72	-5.265	0.006315
A_44_P133335	LOC685424	XM_001063747		-0.72	-5.269	0.014796
A_44_P393897	39875	NM_001007759	364878	-0.72	-5.276	9.37E-05
A_44_P532047	Spetex-2B	NM_001011697	364261	-0.72	-5.277	0.018911
A_44_P912291	AW523955	AW523955		-0.72	-5.284	0.004009
A_44_P382201	RGD1564690_predicted	XM_344533		-0.72	-5.294	7.37E-05
A_42_P645923	Cbs	NM_012522	24250	-0.72	-5.300	0.001454
A_44_P1046615	LOC687030	XM_001076797		-0.72	-5.303	2.37E-05
A_44_P131094	XM_229548	XM_229548		-0.73	-5.309	0.006812
A_44_P461371	S66250	S66250		-0.73	-5.310	2.98E-06
A_43_P11660	Pspn	NM_013014	25525	-0.73	-5.316	5.03E-06
A_44_P250603	RGD1564225_predicted	XM_001064096		-0.73	-5.325	0.004133
A_44_P419757	XM_346210	XM_346210		-0.73	-5.334	0.005083
A_44_P534479	A_44_P534479	A_44_P534479		-0.73	-5.335	0.005221
A_44_P520810	XM_235992	XM_235992		-0.73	-5.348	3.87E-05
A_44_P641108	TC542295	TC542295		-0.73	-5.355	5.97E-06
A_44_P285777	XM_346280	XM_346280		-0.73	-5.370	4.47E-05
A_44_P328028	Lct	XM_341115	116569	-0.73	-5.373	0.005257
A_44_P892359	AABR03128054	AABR03128054		-0.73	-5.380	0.007339
A_44_P961496	TC525536	TC525536		-0.73	-5.386	0.000193
A_44_P229171	ENSRNOT00000042191	ENSRNOT00000042191		-0.73	-5.401	0.005098
A_44_P886974	CB328023	CB328023		-0.73	-5.402	0.004332
A_44_P538496	Olr1434_predicted	NM_001000013	287322	-0.73	-5.406	0.000128
A_44_P112442	LOC301816	XM_229292		-0.73	-5.410	0.005269
A_44_P1021630	Akr1cl1_predicted	XM_341550		-0.73	-5.418	0.005715
A_44_P278767	A_44_P278767	A_44_P278767		-0.73	-5.425	0.004266
A_44_P210985	XM_229368	XM_229368		-0.74	-5.447	0.004173
A_43_P17957	RGD1561459_predicted	XM_341884		-0.74	-5.448	7.95E-05
A_44_P215837	A_44_P215837	A_44_P215837		-0.74	-5.449	2.27E-06
A_44_P483089	XM_343690	XM_343690		-0.74	-5.449	0.003599

A_44_P670762	TC556462	TC556462		-0.74	-5.453	6.47E-05
A_44_P194679	LOC291521	XM_225791		-0.74	-5.459	0.006407
A_43_P11444	S100g	NM_012521	24249	-0.74	-5.475	0.000943
A_44_P325829	Galnt5	NM_031796	83627	-0.74	-5.485	0.000248
A_44_P157060	ENSRNOT00000040314	ENSRNOT00000040314		-0.74	-5.486	0.00006
A_44_P523028	LOC689312	XM_001070367		-0.74	-5.487	0.011552
A_44_P913644	LOC365659	XR_008752	365659	-0.74	-5.500	0.000119
A_44_P563610	TC544530	TC544530		-0.74	-5.519	9.82E-05
A_44_P319128	XM_225832	XM_225832		-0.74	-5.523	0.00594
A_44_P159049	RGD1564018_predicted	XM_345002		-0.74	-5.534	5.13E-06
A_44_P504069	LOC687858	XM_346154		-0.74	-5.550	0.012992
A_44_P706154	BF289057	BF289057		-0.74	-5.557	7.14E-05
A_44_P124121	LOC363337	NM_001014221	363337	-0.74	-5.557	0.017004
A_44_P330828	MGC105649	NM_001008518	302884	-0.75	-5.561	1.32E-05
A_44_P199107	RGD1562545_predicted	XM_345241		-0.75	-5.564	0.011451
A_44_P145095	Six2_predicted	XM_345630	366542	-0.75	-5.577	0.000593
A_44_P931389	CA509267	CA509267		-0.75	-5.578	0.000405
A_44_P440395	LOC691810	XM_001079728		-0.75	-5.578	0.009714
A_44_P577579	TC542766	TC542766		-0.75	-5.591	0.029922
A_44_P650648	BF561546	BF561546		-0.75	-5.599	2.73E-05
A_44_P232550	BM385941	BM385941		-0.75	-5.604	0.020868
A_44_P744708	LOC678714	XM_001053098		-0.75	-5.615	9.24E-06
A_44_P238756	Trpm6_predicted	XM_219747	293874	-0.75	-5.621	7.03E-05
A_44_P367591	Olr1481	NM_001000527	363639	-0.75	-5.625	0.002388
A_44_P129407	BF281577	BF281577	362182	-0.75	-5.633	0.022519
A_44_P998008	Bace2	NM_001002802	288227	-0.75	-5.635	1.62E-06
A_43_P15538	Gja6	NM_019308	54256	-0.75	-5.643	0.000352
A_44_P426870	XM_229422	XM_229422		-0.75	-5.644	0.007226
A_44_P218079	LOC367471	XM_346152		-0.75	-5.653	0.0051
A_43_P23225	Scarf2_predicted	XM_237825		-0.75	-5.658	0.000388
A_44_P423641	Lgals9	NM_012977	25476	-0.75	-5.662	4.48E-05
A_44_P407606	LOC690395	XM_001074324		-0.75	-5.673	0.007797
A_44_P485125	LOC498444	XM_573699		-0.75	-5.684	0.004707
A_44_P323984	XM_343019	XM_343019		-0.76	-5.698	2.39E-05
A_44_P914086	LOC367582	XR_009491		-0.76	-5.701	0.013613
A_44_P932272	TC566831	TC566831		-0.76	-5.705	0.017108
A_44_P638840	TC525756	TC525756		-0.76	-5.713	2.44E-05
A_44_P1040494	XM_216848	XM_216848		-0.76	-5.721	0.000812
A_42_P792497	Cyp2j4	NM_023025	65210	-0.76	-5.725	1.04E-05
A_44_P543439	Slc35d3_predicted	XM_218777		-0.76	-5.731	2.47E-05
A_44_P550952	LOC685429	XM_001063769		-0.76	-5.737	0.004812
A_44_P327392	BI275469	BI275469		-0.76	-5.738	0.000175
A_44_P325863	Ptprr	NM_053594	94202	-0.76	-5.741	0.009713
A_44_P485301	Mall	NM_001014182	362211	-0.76	-5.743	1.79E-05
A_44_P290976	LOC367357	XM_346104		-0.76	-5.744	0.018894
A_44_P404754	AW917108	AW917108	316067	-0.76	-5.748	6.63E-05
A_44_P667961	LOC691117	XM_001076891		-0.76	-5.754	0.003651
A_43_P11676	Tff3	NM_013042	25563	-0.76	-5.754	0.000225
A_44_P586393	BF413452	BF413452		-0.76	-5.755	0.00432
A_44_P296703	AW520784	AW520784	366568	-0.76	-5.759	0.00092
A_44_P117100	MGC116197	NM_001025755	367620	-0.76	-5.765	0.012638
A_44_P140079	Tcfcp2l1_predicted	XM_222570		-0.76	-5.783	6.12E-06
A_44_P560100	A_44_P560100	A_44_P560100		-0.76	-5.784	0.016868
A_44_P575807	XM_578879	XM_578879		-0.76	-5.796	0.005709
A_44_P1002280	RGD1302996	NM_213610	294231	-0.76	-5.799	0.000125
A_44_P404275	AW141608	AW141608	113937	-0.76	-5.804	2.8E-06
A_44_P409677	Nucb1	BC062084	84595	-0.76	-5.814	6.07E-06
A_44_P1011512	Ank3	NM_031805	361833	-0.76	-5.818	3.3E-06
A_44_P342367	XM_577999	XM_577999		-0.77	-5.825	0.002609
A_44_P159110	Sema3g	XM_224613	290562	-0.77	-5.826	0.000805
A_44_P1032752	Tcea3	NM_001015008	298559	-0.77	-5.843	9.24E-06
A_44_P302179	RGD1559914_predicted	XM_226743	294691	-0.77	-5.845	5.79E-05
A_44_P171261	RGD1309791_predicted	XM_236252		-0.77	-5.845	0.000037

A_44_P808142	CB716026	CB716026		-0.77	-5.845	0.000458
A_44_P189343	KIFC2	NM_198752	300053	-0.77	-5.848	1.26E-05
A_44_P398244	RGD1561667_predicted	XM_217999		-0.77	-5.848	0.004695
A_44_P281402	RGD1310242	XM_225899		-0.77	-5.850	4.21E-06
A_44_P572450	Srprb	BC098951	300965	-0.77	-5.852	0.000316
A_44_P405074	NP072018	NP072018		-0.77	-5.858	1.02E-05
A_43_P16045	AB072252	AB072252		-0.77	-5.863	0.000256
A_44_P976992	TC560869	TC560869		-0.77	-5.872	0.003931
A_44_P963185	TC552071	TC552071		-0.77	-5.884	0.000984
A_44_P508906	XM_343652	XM_343652		-0.77	-5.888	0.004542
A_44_P255734	XM_229800	XM_229800		-0.77	-5.894	0.016727
A_44_P101370	AW531675	AW531675	406163	-0.77	-5.897	0.00025
A_44_P540316	AW914885	AW914885		-0.77	-5.902	3.95E-06
A_44_P853184	TC538420	TC538420		-0.77	-5.907	0.000095
A_44_P442645	Olr1449_predicted	NM_001000775	405050	-0.77	-5.909	0.017204
A_44_P295414	LOC679726	XM_001054210		-0.77	-5.912	0.023043
A_44_P1014241	Ramp1	NM_031645	58965	-0.77	-5.916	5.88E-05
A_44_P548630	XM_229624	XM_229624		-0.77	-5.917	0.004075
A_43_P11344	Np_mapped	XM_214155		-0.77	-5.934	0.000221
A_44_P684315	RGD1566076_predicted	XM_573820		-0.77	-5.935	0.000327
A_44_P550684	Spetex-2G	NM_001009971	361099	-0.77	-5.939	0.004412
A_44_P123492	Epb4.1l3	NM_053927	116724	-0.77	-5.945	2.78E-06
A_44_P456901	RGD1561706_predicted	XM_344825		-0.77	-5.955	0.020084
A_44_P383847	BI289548	BI289548	295293	-0.78	-5.968	0.000293
A_44_P288153	Cbr1	NM_019170	29224	-0.78	-5.972	6.29E-05
A_42_P816479	Glrx1	NM_022278	64045	-0.78	-5.979	1.23E-06
A_44_P511262	LOC301816	XM_229292		-0.78	-5.984	0.00533
A_44_P145065	LOC681336	XM_001061313		-0.78	-5.987	0.003624
A_44_P848972	RGD1564895_predicted	XM_231564	312192	-0.78	-5.988	9.93E-06
A_44_P140669	Abi3	NM_001013118	303476	-0.78	-5.996	0.000379
A_44_P226881	Pde2a	NM_031079	81743	-0.78	-6.003	4.83E-06
A_44_P536890	LOC302140	XM_229717		-0.78	-6.005	0.0059
A_44_P234798	BF390686	BF390686	303373	-0.78	-6.007	0.010315
A_44_P974207	LOC367461	XR_006543	367461	-0.78	-6.013	0.004593
A_44_P575866	ENSRNOT00000048313	ENSRNOT00000048313		-0.78	-6.032	0.000185
A_44_P295430	LOC691605	XM_001078992		-0.78	-6.033	0.014202
A_44_P149896	LOC363337	NM_001014221	363337	-0.78	-6.044	0.002719
A_43_P16988	Wdr59	XM_001077628		-0.78	-6.069	0.000208
A_44_P358558	RGD1562697_predicted	XM_218121		-0.78	-6.077	0.016259
A_44_P213875	Alx3	NM_001007012	365900	-0.78	-6.078	8.44E-05
A_43_P19941	Lrrc48	NM_001013857	287371	-0.78	-6.083	1.75E-05
A_44_P379412	Btnl8	NM_212489	406160	-0.78	-6.087	6.34E-05
A_44_P152029	LOC295184	XR_007460	295184	-0.78	-6.088	0.000063
A_44_P454708	XM_345057	XM_345057		-0.78	-6.088	0.003482
A_44_P220088	Npal1_predicted	XM_223366		-0.78	-6.094	2.84E-06
A_44_P436373	XM_229650	XM_229650		-0.79	-6.097	0.004628
A_44_P465468	AI059853	AI059853		-0.79	-6.097	5.23E-05
A_43_P15850	Cyp2j9	NM_175766	313375	-0.79	-6.101	7.38E-06
A_44_P130670	LOC365107	XM_001063111		-0.79	-6.106	0.018486
A_44_P481013	AI230230	AI230230	291974	-0.79	-6.107	0.000448
A_44_P316394	A_44_P316394	A_44_P316394		-0.79	-6.111	0.0036
A_44_P189906	XM_345426	XM_345426		-0.79	-6.111	7.57E-05
A_44_P445004	Slc4a4	NM_053424	84484	-0.79	-6.136	3.29E-05
A_44_P529994	Stox1_predicted	XM_228160	294388	-0.79	-6.148	0.000143
A_44_P523355	AA955360	AA955360		-0.79	-6.162	2.37E-05
A_44_P337930	XM_221162	XM_221162		-0.79	-6.164	0.005117
A_44_P150017	LOC503164	XM_235459		-0.79	-6.177	4.29E-06
A_44_P553218	RGD1563918_predicted	XM_577934		-0.79	-6.189	0.034939
A_44_P434055	RGD1561652_predicted	XM_243980	316039	-0.79	-6.203	0.005443
A_44_P534430	RGD1562019_predicted	XM_577621		-0.79	-6.213	0.012789
A_43_P10691	AI008694	AI008694	309031	-0.79	-6.219	0.031146
A_44_P227956	BF405897	BF405897	301114	-0.79	-6.231	0.011041
A_42_P600372	Creb3l1	NM_001005562	362165	-0.80	-6.250	0.000197

A_44_P621454	RGD1562922_predicted	XM_575325		-0.80	-6.254	4.14E-06
A_44_P224977	LOC688970	XM_001069010		-0.80	-6.265	0.008721
A_44_P375252	Ehmt1_predicted	XM_342379		-0.80	-6.268	0.000003
A_44_P235226	AI009774	AI009774	300838	-0.80	-6.277	0.060314
A_44_P356657	XM_229439	XM_229439		-0.80	-6.286	0.005348
A_44_P174245	LOC364558	NM_001014237	364558	-0.80	-6.295	0.000138
A_44_P460874	CA506147	CA506147	308738	-0.80	-6.298	0.002029
A_44_P323955	A_44_P323955	A_44_P323955		-0.80	-6.298	0.00105
A_44_P687053	LOC690012	XM_001072919	690012	-0.80	-6.306	0.00048
A_42_P631958	Zic4_predicted	XM_236504		-0.80	-6.318	7.57E-05
A_44_P395944	XM_217933	XM_217933		-0.80	-6.332	0.006315
A_44_P433921	XM_347129	XM_347129		-0.81	-6.389	0.000142
A_43_P18403	Mthfs	NM_001009349	300886	-0.81	-6.393	4.39E-05
A_44_P438798	XM_345957	XM_345957		-0.81	-6.416	0.001501
A_44_P447781	LOC680682	XM_001056700	680682	-0.81	-6.420	0.012168
A_44_P468522	RGD1560580_predicted	XM_222167	288646	-0.81	-6.435	0.005431
A_44_P396720	AA957618	AA957618		-0.81	-6.448	0.000274
A_44_P1048149	RGD1311830_predicted	XM_343414		-0.81	-6.453	0.000581
A_44_P361327	LOC501473	XM_001070503		-0.81	-6.458	0.031591
A_44_P254613	Gosr1	NM_053584	94189	-0.81	-6.463	0.006059
A_44_P217314	Chdh	NM_198731	290551	-0.81	-6.475	0.000062
A_44_P247843	Olr1091_predicted	NM_001000593	366843	-0.81	-6.483	0.003116
A_44_P944304	ENSRNOT00000016430	ENSRNOT00000016430		-0.81	-6.492	0.001624
A_43_P15021	Slc25a29	NM_001010958	314441	-0.81	-6.494	9.35E-05
A_44_P215808	RGD1564703_predicted	XM_235335		-0.81	-6.512	0.000087
A_44_P149519	A_44_P149519	A_44_P149519		-0.81	-6.513	0.001679
A_44_P799807	BF398472	BF398472		-0.82	-6.531	6.84E-06
A_44_P198746	P2rx3	NM_031075	81739	-0.82	-6.531	0.000357
A_44_P353654	Dmd	NM_001005246	24907	-0.82	-6.543	3.23E-05
A_44_P476713	AABR03130795	AABR03130795		-0.82	-6.548	0.00033
A_43_P14558	Ngfg	NM_031523	24594	-0.82	-6.554	2.12E-05
A_44_P408928	AA926202	AA926202	300033	-0.82	-6.561	0.004065
A_44_P399414	Klk7	NM_012593	24523	-0.82	-6.561	8.15E-06
A_44_P168468	XM_223882	XM_223882		-0.82	-6.561	0.019304
A_44_P146448	M16406	M16406		-0.82	-6.564	0.000423
A_44_P468902	XM_226288	XM_226288		-0.82	-6.571	0.001616
A_44_P121796	XM_343751	XM_343751		-0.82	-6.578	0.004891
A_44_P729100	A_44_P729100	A_44_P729100		-0.82	-6.594	3.09E-06
A_44_P398641	XM_217515	XM_217515		-0.82	-6.598	0.013282
A_42_P472699	Amn_predicted	XM_234547		-0.82	-6.605	2.29E-06
A_44_P328087	Snai1	NM_053805	116490	-0.82	-6.620	0.000733
A_42_P641517	Mcpt1	U67915	29265	-0.82	-6.621	0.000164
A_44_P137217	BM986231	BM986231		-0.82	-6.624	0.000348
A_44_P168898	XM_343522	XM_343522		-0.82	-6.629	0.004499
A_44_P399864	AI060126	AI060126	84487	-0.82	-6.637	0.02616
A_44_P289526	AI168950	AI168950	307526	-0.82	-6.643	5.24E-05
A_44_P975554	TC555358	TC555358		-0.82	-6.645	2.75E-06
A_44_P236681	XM_229427	XM_229427		-0.82	-6.650	0.011249
A_43_P19848	RGD1309870_predicted	XM_214086		-0.82	-6.666	3.34E-05
A_44_P419188	Hnf4a	NM_022180	25735	-0.82	-6.668	0.000021
A_44_P337914	XM_229254	XM_229254		-0.82	-6.671	0.00528
A_44_P596890	AA891475	AA891475	501828	-0.82	-6.672	0.000174
A_44_P314045	RGD1564205_predicted	XM_573954		-0.82	-6.680	0.022773
A_44_P551197	BE109586	BE109586	287873	-0.83	-6.691	0.000543
A_44_P278397	Ss18l1	NM_138918	192352	-0.83	-6.699	2.37E-05
A_44_P442774	U06747	U06747		-0.83	-6.720	0.000626
A_44_P570604	DN932947	DN932947		-0.83	-6.727	0.000187
A_44_P311726	A_44_P311726	A_44_P311726		-0.83	-6.730	9.58E-06
A_44_P386163	Hsd3b1_predicted	NM_001007719	360348	-0.83	-6.732	6.08E-05
A_44_P313542	Sgk	NM_019232	29517	-0.83	-6.745	1.16E-05
A_44_P342225	RGD1565464_predicted	XM_218017		-0.83	-6.759	2.67E-06
A_44_P121308	Rasgrf2	NM_053721	114513	-0.83	-6.768	0.001082
A_44_P548151	Dlgap2	NM_053901	116681	-0.83	-6.770	0.007055

A_44_P199430	XM_235761	XM_235761		-0.83	-6.773	0.006048
A_44_P270952	AA892987	AA892987	361420	-0.83	-6.776	0.000358
A_44_P318921	Zfp169_predicted	XM_225207		-0.83	-6.793	0.00041
A_44_P869337	TC558017	TC558017		-0.83	-6.804	0.000171
A_44_P529950	RGD1565104_predicted	XM_574212		-0.83	-6.805	0.000181
A_43_P11967	Mcp	NM_019190	29333	-0.83	-6.808	0.001552
A_44_P400677	Kcnv2_predicted	XM_220024		-0.83	-6.808	0.000178
A_44_P217266	Xpo1	NM_053490	85252	-0.83	-6.812	3.87E-05
A_44_P958227	BF286911	BF286911		-0.83	-6.813	0.000925
A_43_P12908	Klf4	NM_053713	114505	-0.83	-6.814	1.62E-06
A_44_P156864	RGD1562268_predicted	XM_226158		-0.83	-6.821	0.00054
A_44_P685317	XM_579901	XM_579901		-0.83	-6.835	0.023088
A_44_P309420	RGD1304644	NM_001037198	311536	-0.83	-6.837	0.000446
A_44_P710192	Tmem45b	NM_001033067	315524	-0.84	-6.839	1.53E-05
A_44_P515211	Tnfsf15	NM_145765	252878	-0.84	-6.839	0.000539
A_44_P477827	Sgk2	XM_342570	171497	-0.84	-6.839	0.000277
A_44_P334779	LOC259245	NM_147213	259245	-0.84	-6.853	0.010934
A_44_P302221	LOC689753	XM_001071886		-0.84	-6.885	0.012829
A_44_P468733	Armetl1_predicted	NM_001037543	361276	-0.84	-6.885	0.016734
A_44_P141644	AI599296	AI599296		-0.84	-6.895	0.003219
A_44_P455011	LOC686041	XM_001066301	686041	-0.84	-6.906	0.000155
A_44_P279172	LOC291516	XM_225786		-0.84	-6.919	0.003452
A_44_P152258	XM_225836	XM_225836		-0.84	-6.921	0.010541
A_44_P403531	BM386224	BM386224	295305	-0.84	-6.922	0.000231
A_44_P201981	Inpp5a_predicted	XM_344971		-0.84	-6.938	2.73E-05
A_44_P384517	RGD1562014_predicted	XM_227869		-0.84	-6.938	0.021811
A_44_P405640	LOC367632	XM_346234		-0.84	-6.953	0.0059
A_44_P379471	Olr237_predicted	NM_001000210	293382	-0.84	-6.967	0.011838
A_44_P154445	RGD1560471_predicted	XM_225892		-0.84	-6.969	0.001549
A_44_P107910	XM_229478	XM_229478		-0.84	-6.971	0.003319
A_44_P172655	Nat2	NM_053854	116631	-0.84	-6.979	3.35E-05
A_44_P994385	Mucdhl	NM_138525	171554	-0.84	-6.986	1.58E-06
A_44_P987448	BF414619	BF414619		-0.84	-6.994	0.01125
A_44_P416617	Tff1	NM_057129	117270	-0.85	-7.027	0.000302
A_44_P328654	LOC501474	XM_576882	501474	-0.85	-7.035	0.045622
A_44_P260537	A_44_P260537	A_44_P260537		-0.85	-7.058	0.005235
A_43_P19706	Actn2_predicted	XM_214499	291245	-0.85	-7.084	0.000213
A_44_P151953	XM_344288	XM_344288		-0.85	-7.094	0.003972
[truncated: 59,453 more chars]
