# Supplementary figures and images for: Gene expression profile and genomic alterations in colonic tumours induced by 1,2-dimethylhydrazine (DMH) in rats
Source: BMC Cancer. 2010 May 11;10:194. doi: 10.1186/1471-2407-10-194 (PMC2877689; doi:10.1186/1471-2407-10-194)

Additional file 3

Title: Hierarchical cluster analysis of gene expression data

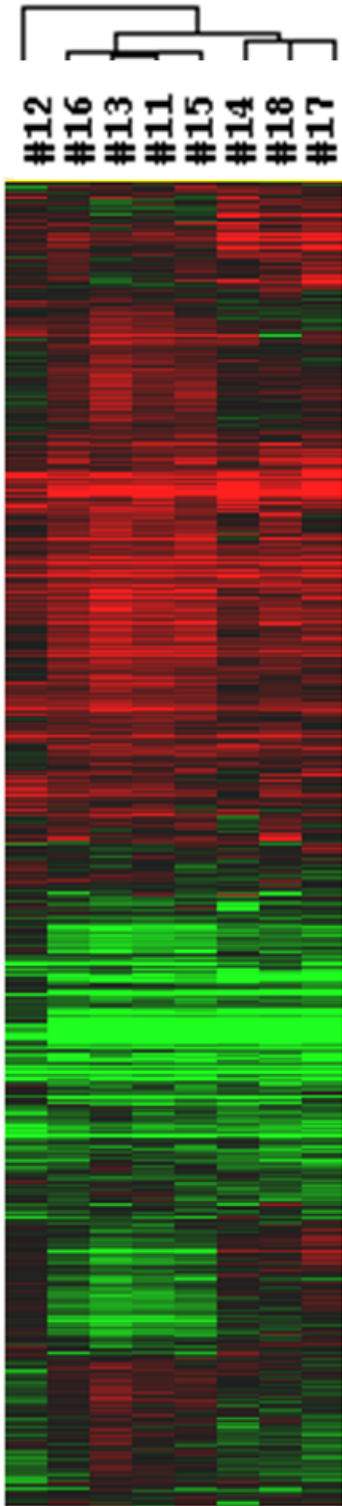

Supplement: Additional file 3 — Hierarchical cluster analysis of gene expression data. Hierarchical cluster analysis of gene expression data from the 8 DMH-induced tumours (samples # 11-18) compared to the corresponding normal colon mucosa. The analysis was performed on all genes which passed the quality control step present in 100% of the experiments. [file 1471-2407-10-194-S3.PDF]
