# Supplementary material for: Gene expression profile and genomic alterations in colonic tumours induced by 1,2-dimethylhydrazine (DMH) in rats
Source: BMC Cancer. 2010 May 11;10:194. doi: 10.1186/1471-2407-10-194 (PMC2877689; doi:10.1186/1471-2407-10-194)
Supplement: Additional file 4 — List of the genes belonging to the significantly up-regulated pathways. List of the genes (EntrezGene ID: http://www.ncbi.nlm.nih.gov/gene) belonging to the significantly up-regulated pathways shown in Table 2. [file 1471-2407-10-194-S4.PDF]

Additional File 4:

Title: List of the genes belonging to the significantly up-regulated pathways.

**MAPP Name**	**Genes changed (Entrez Gene ID)**	**Probes linked to the GO term or MAPP measured**
Rn_DNA_replication	116468,246327,25737,288414,288532,291927,293144,301430,307126,313479,316273,361698,362817,50522,59102,85242,89809	116468,246327,25737,288414,288532,289758,291927,293144,295596,301430,304528,307126,313138,313479,316273,361698,362817,50522,59102,59294,85242,89809
Rn_Matrix_Metalloproteinases	116510,117033,117061,171045,24835,25335,25358,29543,366964,65205,81687,81707	116510,117033,117061,171045,24835,25246,25335,25358,29543,366964,65205,81687,81707
Rn_TNF-alpha-NF-kB	114562,116596,117263,117279,140934,156767,24185,24471,246240,246756,24835,25124,25233,25404,25573,25576,25577,25578,25581,25586,25614,25622,25625,26962,287670,287772,288532,292747,293860,29542,29568,29753,299331,301246,301252,301515,303518,305240,306007,309165,309295,309452,312641,313867,315713,316241,353233,353256,361055,361825,361925,362788,56010,58934,64044,641452,64302,64314,64640,81525,81650,81736,81774,83427,83581,83582,83805,83806,84577.	114562,115771,116549,116596,116633,117263,117279,140657,140934,156767,24185,24471,246240,24672,246756,24835,25069,25124,25233,252961,25402,25404,25522,25573,25576,25577,25578,25581,25586,25614,25622,25625,266610,26962,287280,287367,287670,287772,288532,289924,29155,292747,29355,293860,29476,29542,29568,29677,29753,297893,299331,301246,301252,301442,301515,303518,303602,303604,305240,305451,305606,306007,308267,309165,309295,309452,312641,313867,315713,316051,316241,317335,353233,353256,361055,361167,361765,361810,361825,361925,362491,362607,362788,363599,408217,50659,54244,56010,56011,58934,60328,60371,64026,64044,641452,64302,64314,64322,64640,78971,81525,81650,81736,81774,81920,83427,83581,83582,83614,83805,83806,83840,84020,84027,84351,84577,84581
Rn_G1_to_S_cell_cycle	114483,114851,116651,246060,246327,24842,25163,25737,266713,288532,291927,301323,301430,308937,313479,313978,316273,362817,399489,406	114483,114851,116651,171102,171150,24577,246060,246327,24708,24842,25112,25163,25164,25737,266713,288532,291927,295596,301323,301430,308937,313138,313479,313978,314638,316273,362165,362817,399489,406169,54237,54238,58919,591

	169,54237,54238,58919,59102,64033,81646,84389,85242,94201	02,64033,81646,83571,84389,85242,94201
Rn_mRNA_processing	116650,116655,116697,117280,117282,140931,171365,192246,252855,259229,287113,287530,287643,289693,290666,291295,292688,292729,29428,29497,295270,29578,298075,298916,299620,308335,311166,313035,313131,313583,317259,319110,361814,362160,362630,363633,494445,499420,60421,64301,80846,83498,84473,84474	116650,116655,116697,117259,117280,117282,140931,171305,171365,192246,252855,259229,287113,287269,287530,287643,288701,289693,290666,291078,291295,291534,291877,292688,292729,29428,29497,295270,29578,29667,298075,298916,299284,299620,300996,308335,311166,311670,313035,313131,313583,317259,317385,319110,361035,361126,361814,362152,362160,362251,362361,362630,363633,494445,499420,59087,60421,63996,64301,79256,80846,81781,83498,84473,84474,84486
Rn_Translation_Factors	116636,117019,117045,171145,171350,171361,287444,288516,293484,29565,298700,299872,299899,300253,302697,303831,307503,311371,361845,362952,54318,56783,64514,84005	116636,117019,117045,171145,171350,171361,192234,25435,27137,287444,288516,292148,293484,29565,29702,298700,299872,299899,300253,302697,303831,307503,311371,361845,362952,54318,56783,64514,84005
Rn_Ribosomal_Proteins	117042,117053,122772,124323,124440,140654,140655,140661,140662,161477,245981,25347,26962,27139,28298,290641,291434,29236,29257,29258,29282,29283,29284,29285,29286,29287,29288,29304,29426,294282,296709,29752,297755,300079,317646,362631,57809,58927,64156,64205,64298,64302,64306,64307,64638,64640,65043,65136,65139,79449,81729,81763,81764,81765,81766,81767,81768,81770,81772,81773,81774,81775,81776,81777,83789	117042,117053,122772,122799,124323,124440,140654,140655,140661,140662,161477,24159,245981,25347,25348,26962,27139,28298,290641,291434,29236,29257,29258,29282,29283,29284,29285,29286,29287,29288,29304,29426,294282,296709,29752,297755,300079,317646,360572,362631,499057,499782,500547,57809,58927,64156,64205,64298,64302,64306,64307,64638,64640,65043,65136,65139,79449,81729,81763,81764,81765,81766,81767,81768,81769,81770,81772,81773,81774,81775,81776,81777,83789,83840,94266
Rn_Cell_cycle	114212,114483,114494,114851,116651,140583,171103,171576,24842,25163,25203,25737,288532,291927,301430,308937,311860,313479,313978,316	114212,114483,114494,114851,116651,140583,170915,171102,171103,171576,24708,24842,25112,25163,25203,25631,25737,288532,291927,295596,297176,301430,308937,311860,313138,313479,313978,315330,316273,361195,363088,399489,50554,5

	273,399489,50554,54237,56010,59086,64033,64193,84389,84577,84578,84582,94201	4237,56010,59086,63996,64033,64193,64515,83502,83571,84027,84389,84577,84578,84581,84582,94201
Rn_RNA_transcription	117017,170922,266713,294236,303918,306012,361365,362858,363633,366277,373541,81674,83503,83828,84389	117017,117526,170922,171150,266713,291703,294236,303918,306012,361365,362858,363633,366277,373541,81673,81674,83503,83828,84389
Rn_TGF_Beta_Signaling_Pathway	156726,161452,24373,24516,24617,24835,25124,25125,25296,25639,29200,29357,29591,311061,316742,367218,50554,59086,59107,59328,60584,81736,81810,84353,84404,84598,85435	156726,161452,24373,24516,24617,24835,25124,25125,25296,25313,25631,25639,25671,29200,29357,293621,29591,29610,311061,314322,316742,367100,367218,50554,50658,50689,54244,59086,59107,59328,60584,81516,81736,81810,83837,84353,84404,84598,85435
Rn_Inflammatory_Response_Pathway	117036,140433,156767,171369,192362,24493,24494,24835,25408,25625,25661,295279,313050,56822,84032,84352	117036,117539,140433,140924,156767,171369,192362,24493,24494,24835,25084,25408,25625,25661,287287,29393,295279,301348,313050,56822,84032,84352
Rn_Proteasome_Degradation	116725,117262,117263,161475,24770,24968,25581,287670,287716,287772,287984,289990,292766,29630,29666,29670,29671,29672,29673,29674,29675,50522,58854,83499,83806,85492,94198	116725,117262,117263,161475,24770,24828,24967,24968,25489,25581,287670,287716,287772,287984,289924,289990,291983,292766,29614,29630,29666,29668,29670,29671,29672,29673,29674,29675,29676,29677,50522,58854,58940,64631,64701,79435,81816,81827,83499,83806,85492,94198
Rn_Alpha6-Beta4-Integrin	114517,114851,116636,117036,117045,140433,192362,24185,24553,25150,25335,25467,25576,25577,25578,25614,29236,29357,29741,29753,309368,311860,363875,56718,64315,81504,81818,83805,85385	114517,114851,116636,117036,117045,117273,140433,170538,192362,24185,24329,24337,24553,24884,25150,25335,25402,25467,25576,25577,25578,25614,25631,25724,29236,29357,29376,29431,297383,29741,29753,305078,306871,309368,311860,363875,56011,56718,60664,64204,64315,64639,81504,81818,83805,85243,85385
Rn_B_Cell_Receptor	114483,114494,116554,116590,117282,155918,171136,24185,24516,24674,24675,25124,25125,25150,25155,25262,25614,25621,25622,25676,25738,286974,287942,288077,289211,29304,294048,29600,29741,29748,305269,3	114203,114483,114486,114494,116554,116590,116663,116689,117273,117282,140607,155918,170538,170851,171055,171136,171150,24185,24224,24516,24674,24675,24703,24708,24887,25124,25125,25150,25155,25262,25309,25414,25614,25621,25622,25676,25734,25738,286974,287942,288077,288669,289014,289211,289820,291044,29304,29337,29340,294048,29600,2974

	05450,309165,309295,312477,313050,313874,315707,317676,353256,360854,361400,367901,499356,54227,54259,58918,60587,63881,64033,64547,81504,81515,81646,81649,84353,84582,85385,85421,89812,94201	1,29748,301333,301348,303836,305269,305450,309165,309295,309419,312477,313050,313498,313845,313874,315707,317676,353256,360854,361400,364403,367901,499356,50646,50686,50689,54227,54236,54245,54259,54268,58918,58960,60587,63881,64026,64033,64547,81504,81515,81530,81646,81647,81649,81678,81732,81745,83477,83840,84027,84351,84353,84477,84482,84492,84582,85385,85420,85421,89812,94201
Rn_Signaling_of_Hepatocyte_Growth_Factor_Receptor	116554,116590,171337,24446,24511,24516,24553,25118,25125,25614,25622,25676,287942,295347,360820,63881,81504,83805	116554,116590,170851,171337,24446,24511,24516,24553,24703,25118,25125,25614,25622,25676,287942,293621,29431,295347,360820,50557,50646,50689,54245,58960,63881,81504,83805
Rn_Nucleotide_Metabolism	24465,24689,25502,29240,29562,315150,83782,84596,85241,85472	24312,24465,24689,25502,29240,29562,302642,307498,315150,59294,83782,84596,85241,85472
Rn_Complement_Activation_Classical	192262,24233,29687,298566,312705,362634,64023	192262,24233,24237,29687,298566,312705,362634,64023,64036
