# Supplementary material for: Gene expression profile and genomic alterations in colonic tumours induced by 1,2-dimethylhydrazine (DMH) in rats
Source: BMC Cancer. 2010 May 11;10:194. doi: 10.1186/1471-2407-10-194 (PMC2877689; doi:10.1186/1471-2407-10-194)
Supplement: Additional file 5 — List of the genes belonging to the significantly down-regulated pathways. List of the genes (EntrezGene ID: http://www.ncbi.nlm.nih.gov/gene) belonging to the significantly down-regulated pathways shown in Table 2. [file 1471-2407-10-194-S5.PDF]

Additional File 5:

Title: List of the genes belonging to the significantly down-regulated pathways.

**MAPP Name**	**Genes changed (EntrezGene ID)**	**Probes linked to the GO term or MAPP measured**
Rn_Nuclear_Receptors	24413,24831,24873,25664,25682,25735,25747,299210,54278,58852,60349,65035,81808,81812,83574,83826	113984,24413,245980,24705,24831,24873,24890,25149,25271,25664,25682,25735,25747,299210,361801,50659,54278,58851,58852,60349,65035,79240,81808,81812,83574,83826
Rn_Unsaturated_Fatty_Acid_Beta_Oxidation	117543,170670,171155,24158,25287,29740	117543,170670,171155,24158,25287,29740
Rn_Electron_Transport_Chain	140608,157074,171335,171374,192241,245958,245965,24860,25176,25250,25282,25309,25488,291103,293448,29478,29507,298596,303393,363061,54322,64463,81728,89786,94194,94271	113907,114630,116550,140608,157074,171082,171335,171374,171375,192241,245958,245965,24860,25176,25250,25278,25282,252934,25309,25310,25488,289217,291103,293448,29445,29478,29507,29754,298596,301011,303393,363061,54315,54322,58927,64463,65262,81511,81728,85333,89786,94194,94271
Rn_Nuclear_receptors_in_lipid_metabolism_and_toxicity	114628,171352,24307,24646,24873,25270,25664,25682,25747,58852,60351,65035,81924	114628,154985,171352,24307,24646,24705,24873,24891,25270,25664,25682,25747,313210,58852,60351,65035,81924,84356,85264
Rn_Fatty_Acid_Beta_Oxidation	113965,170670,171155,24158,64304	113965,140547,170670,171155,24158,64304
Rn_Krebs-TCA_Cycle	114597,117098,157074,24368,25104,29554,298596,363061,79250,81530,81654,81829	114096,114597,117098,157074,170587,24368,24551,25104,25179,26955,289217,289950,29554,298596,298942,362404,363061,79250,81530,81654,81829,89813,94173
Rn_Irinotecan_pathway	113992,171118,29225,312382,65036	113992,171118,24565,29225,312382,65036
Rn_Peptide_GPCRs	24180,24613,24807,25342,25354,25449,282832,29234,29310,29462,29471,54305,81509,81668,84022	117029,171044,171056,24180,24326,24613,24807,24938,25033,25108,25245,25342,25354,25449,25601,282832,287673,29234,29256,29310,29358,29462,29471,50672,54305,60463,60628,81509,8

		1668,84007,84022
Rn_Calcium_regulation_in_cardiac_cells	114117,114207,170538,24242,24244,24245,24392,25229,25289,25390,25391,25398,25521,25522,25578,25639,25645,25650,25655,25679,29209,29481,29584,29598,29699,29713,29715,29716,302950,50599,50664,54234,54293,59075,59076,59077,64202,79217,81662,81664,81678.	114117,114120,114207,114705,117055,170538,24176,24239,24242,24244,24245,24260,24392,24400,245986,24678,24681,24925,25050,25229,25262,25289,25297,25387,25390,25391,25398,25521,25522,25576,25577,25578,25639,25643,25645,25650,25655,25679,25686,25740,257648,266709,29209,29241,29322,29340,29413,29480,29481,294962,29584,29585,29586,29598,29639,29693,29699,29713,29715,29716,29753,302950,394266,50564,50599,50663,50664,53949,54223,54234,54289,54291,54293,54294,56010,56011,58979,59075,59076,59077,59293,60449,64199,64202,64508,64532,64672,79125,79217,79218,81636,81645,81662,81664,81666,81667,81678,81749,84420,84583,85420,85421
Rn_Glucocorticoid_Mineralocorticoid_Metabolism	191574,24298,25117,25146,29632	191574,24298,25116,25117,25146,29632,29680
